# Chemo- and regioselective oxygenation of C(sp^3^)–H bonds in aliphatic alcohols using a covalently bound directing activator and atmospheric oxygen†
†Electronic supplementary information (ESI) available: Experimental details, including procedures, syntheses and characterization of new products; ^1^H, ^13^C, and ^19^F NMR spectra. CCDC 1415615. For ESI and crystallographic data in CIF or other electronic format, see DOI: 10.1039/c5sc04476f


**DOI:** 10.1039/c5sc04476f

**Published:** 2015-11-27

**Authors:** Jun Ozawa, Masayuki Tashiro, Jizhi Ni, Kounosuke Oisaki, Motomu Kanai

**Affiliations:** a Graduate School of Pharmaceutical Sciences , The University of Tokyo , 7-3-1 Bunkyo-ku , Tokyo 113-0033 , Japan . Email: oisaki@mol.f.u-tokyo.ac.jp ; Email: kanai@mol.f.u-tokyo.ac.jp; b Japan Science Technology Agency (JST) , ERATO Kanai Life Science Catalysis Project , 7-3-1 Bunkyo-ku , Tokyo 113-0033 , Japan

## Abstract

Chemically reactive directing groups (directing activators) represent a promising strategy for mild and regioselective C(sp^3^)–H functionalization. The use of a radical *N*-oxyl directing activator promoted the aerobic oxygenation of benzylic, propargylic, tertiary, and unactivated acyclic methylene C(sp^3^)–H bonds in aliphatic alcohols with γ- (or δ-) selectivity under mild conditions (room temperature to 50 °C). The reaction was unaffected by the presence of various oxidation-sensitive functional groups, which proved to be problematic in previously reported studies on the oxidation of C(sp^3^)–H bonds. Structural modifications on the directing activator altered the regioselectivity, and thus provided an ultra-remote aerobic C(sp^3^)–H oxygenation. The observed reactivity and regioselectivity could be rationalized in terms of the intramolecular conformational accessibility of the *N*-oxyl radical and the electronic characteristics of C(sp^3^)–H bonds.

## Abstract 2

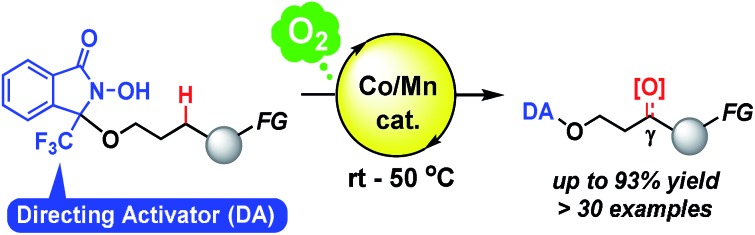
Aerobic, site-selective C(sp^3^)–H oxygenation using a novel *N*-oxyl radical directing activator (chemically reactive directing group) is described.

## Introduction

The functionalization of unactivated C–H bonds is a research area that is currently being investigated intensively. Especially for the streamlined synthesis of complex drug lead molecules, containing a multitude of C(sp^3^)–H bonds, a controlled functionalization of these bonds can be a powerful synthetic tool.^1^ In order to apply C(sp^3^)–H functionalizations to drug lead syntheses, two key features are necessary: (1) mild and clean reaction conditions to assure functional group tolerance,^2^ and (2) site-selectivity due to the commonly encountered presence of multiple C–H bonds.^3^

We were thus interested in a site-selective oxygenation of alcohols that is able to target C(sp^3^)–H bonds located remotely from the hydroxy group,^4^ using clean and abundant aerobic oxygen (O_2_) as the oxidant.^5^ This unprecedented reaction pattern should provide conceptually improved synthetic routes to various biologically active drug leads, containing multiple functional groups based on oxygen. Ideally, these routes should exhibit a high redox economy,^6^ and generate a minimum of potentially toxic waste. The three main obstacles to overcome in the development of such reactions are: (1) the applicability to ubiquitous, but unreactive acyclic methylene C(sp^3^)–H bonds;^7^ (2) the conversion of such unreactive C(sp^3^)–H bonds at a specific position, while simultaneously overriding the innately higher reactivity of the C–H bonds at α-position with respect to the oxygen atom;^8^ and (3) the low efficiency of triplet O_2_ in initiation of the oxygenation reaction.

**Scheme 1:**
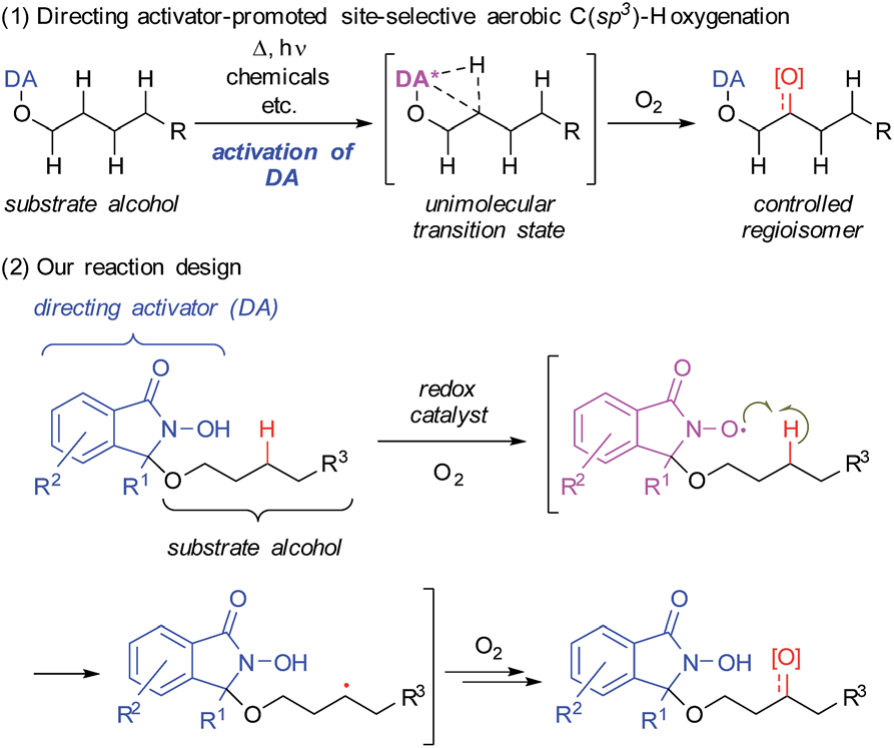
Chemo- and regioselective intramolecular oxidation of C(sp^3^)–H bonds in aliphatic alcohols using a novel directing activator.

Herein, we describe the use of a chemically reactive directing group or “directing activator” (DA)^9^,^10^ in order to circumvent the aforementioned obstacles, thus expecting to produce the entropically preferred unimolecular transition states leading to a mild and chemoselective cleavage of specific C–H bonds, including very challenging methylene C(sp^3^)–H bonds (Scheme 1-1).^11^

## Results and discussion

### Optimization of reaction conditions

**Scheme 2:**
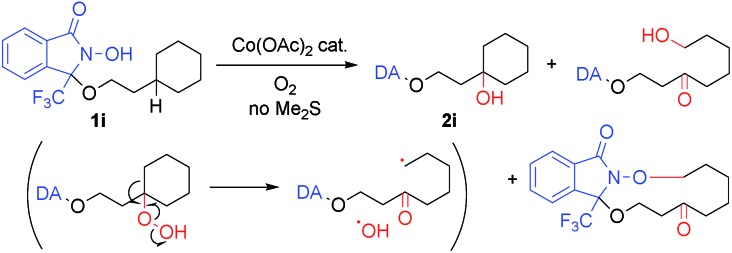
Undesired C–C bond cleavage of **1i***via* decomposition of hydroperoxide intermediate.

We devised a novel DA inspired by Ishii's *N*-hydroxyphthalimide (NHPI) chemistry (Scheme 1-2),^12^ which is based on an *N*-oxyl radical, generated from the *N*-hydroxyamide moiety of the DA in the presence of O_2_. This moiety is able to cleave C(sp^3^)–H bonds homolytically to produce a carbon radical. By covalently attaching the DA to the hydroxy group of substrate alcohols, this C(sp^3^)–H activation step should become an intramolecular process. Thus, it should be possible to selectively cleave specific C(sp^3^)–H bonds that can engage in suitable spatial contact with the *in situ*-generated *N*-oxyl radical of the DA.^13^ Subsequently, the thus generated carbon radical would be trapped by O_2_ to produce the corresponding oxygenated alcohols or ketones.^14^

Based on this reaction design, we began our investigation of aerobic C–H oxygenation by modifying the DA structure and performing a screening of oxygenation conditions using DA-bound alcohol substrates (Table 1). In order to attach covalently the substrate alcohols to the DA, one of the two imide carbonyl groups in NHPI was transformed into an aminal group, leading to an *N*-hydroxyisoindolinone structure. As the reactivity of *N*-oxyl radicals towards C–H cleavage follows their electron-deficiency,^15^ we tried to introduce electron-withdrawing groups and found that especially the introduction of a trifluoromethyl group at the 3-position of the DA (R^1^ = CF_3_) was effective. Then we screened suitable metal catalysts that could promote *N*-oxyl radical formation from the *N*-hydroxy group (see Scheme 5) using 1-butanol-derivative **1a** as the substrate and 2,2,2-trifluoroethanol (TFE) as the solvent (Table 1, entries 1–11). We found that Co(OAc)_2_ (entry 6) and Mn(OAc)_3_·2H_2_O (entry 8) were effective. Although the α-C(sp^3^)–H bond adjacent to the ether oxygen atom is the innately more reactive site,^8^ it was the less reactive γ-C(sp^3^) atom that was predominantly oxygenated. Metal salts bearing counterions other than acetate (*i.e.*, acac, NO_3_, halide, and OTf) showed very low reactivity.^16^ The reaction was cleaner with the combinational use of Co(OAc)_2_ and Mn(OAc)_3_·2H_2_O, and the desired C–H oxygenation product **2a** was obtained in 67% NMR yield (entry 10). The catalyst loading could be reduced to 5 mol% of each metal without loss of efficiency (entry 11). A DA with a simple alkyl substitution (R^1^ = Et), instead of a CF_3_ group, did not show satisfactory performance (entry 12). Introduction of another CF_3_ moiety on the aromatic ring of the DA did not improve the result (entry 13). Other types of DA modification resulted in production of complex reaction mixtures.^16^

**Table 1:** Investigation of a metal catalyst and DA

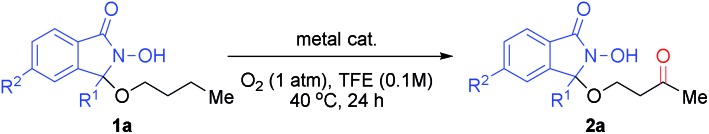				
Entry	Metal (mol%)	R^1^	R^2^	Yield^*a*^
1	None	CF_3_	H	0
2	CuOAc (20)	CF_3_	H	0
3	Cu(OAc)_2_ (20)	CF_3_	H	0
4	Fe(OAc)_2_ (20)	CF_3_	H	0
5	Fe(OH)(OAc)_2_ (20)	CF_3_	H	0
6	Co(OAc)_2_ (20)	CF_3_	H	40
7	Mn(OAc)_2_ (20)	CF_3_	H	3
8	Mn(OAc)_3_·2H_2_O (20)	CF_3_	H	36
9	Co(OAc)_2_ (10) + Mn(OAc)_2_ (10)	CF_3_	H	16
10	Co(OAc)_2_ (10) + Mn(OAc)_3_·2H_2_O (10)	CF_3_	H	67
11	Co(OAc)_2_ (5) + Mn(OAc)_3_·2H_2_O (5)	CF_3_	H	67^*b*^(62)
12	Co(OAc)_2_ (5) + Mn(OAc)_3_·2H_2_O (5)	Et	H	0
13	Co(OAc)_2_ (5) + Mn(OAc)_3_·2H_2_O (5)	CF_3_	CF_3_	62

^*a*^Yields were calculated from the ^1^H NMR spectra of crude reaction mixtures using 1,1,2,2-tetrachloroethane as an internal standard. Isolated yields are given in parentheses.

^*b*^The reaction time was 18 h.

On the basis of this study, we established that a DA containing an *N*-hydroxy-3-trifluoromethylisoindolinone moiety and reaction conditions using Co(OAc)_2_ (5 mol%), Mn(OAc)_3_·2H_2_O (5 mol%), and O_2_ (1 atm) in TFE (0.1 M) at 40 °C represent optimal conditions (condition A). The use of a fluoroalcohol solvent was crucial for high reactivity, as fluoroalcohols are able to stabilize radicals, dissolve molecular oxygen, and are resistant to oxidation.^17^

Applying the cobalt-catalyzed conditions to the oxygenation of tertiary C(sp^3^)–H bonds, however, resulted in the formation of complex product mixtures. For example, exposing **1i** to condition A afforded **2i** and C–C bond-cleaved products (Scheme 2).^18^ As the presence of these decomposition products may be explained by the formation of hydroperoxide intermediates, we carried out a screening of the reductants in order to ensure optimal *in situ* reduction conditions for such problematic species. We found that Me_2_S provided the best results, as this additive was not susceptible to oxidation using the Co/O_2_ catalytic system in the absence of substrates **1**.^19^ Further optimization finally allowed us to identify the use of Co(OAc)_2_ (1 mol%), Me_2_S (1.2 equiv.), and O_2_ (1 atm) in TFE (0.1 M) at 40 °C (condition B) as the best set of conditions for the oxygenation of tertiary C(sp^3^)–H bonds.

### Substrate scope and limitations

Using these conditions allowed the oxygenation of a broad variety of DA-bound alcohols as shown in Table 2. Applying condition A converted simple methylene C(sp^3^)–H bonds of aliphatic alcohols (**1a–1g**) regioselectively into the corresponding C=O bonds (**2a–2g**). Using condition B, the oxygenation of tertiary C(sp^3^)–H bonds proceeded generally in higher yield (**2h–2r**). The condition B was applicable to a gram-scale reaction of **1h** without significant loss of efficiency.^16^ Using either condition A or B allowed the oxygenation of benzylic and propargylic C(sp^3^)–H bonds, which proceeded rapidly, even at lower temperature (**2s–2ae**).^20^

**Table 2:** Reaction scope with respect to alcohol substrates^*a*^

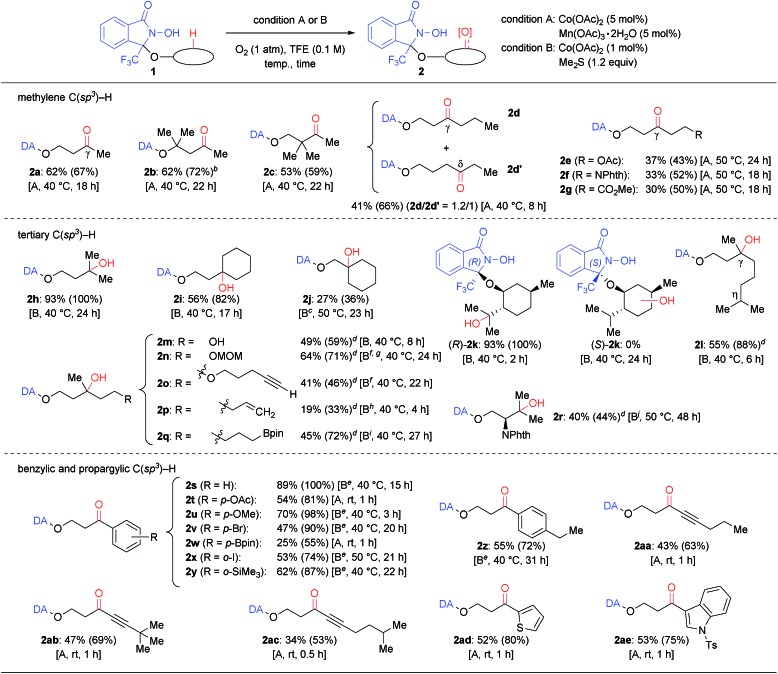

^*a*^Isolated yields are described and yields in parentheses were calculated from the ^1^H NMR spectra of crude reaction mixtures using 1,1,2,2-tetrachloroethane as an internal standard.

^*b*^The product was obtained as a cyclic hemiacetal.

^*c*^2 mol% of Co(OAc)_2_ were used.

^*d*^Starting materials **1** and products **2** were obtained as diastereoisomer mixtures.

^*e*^2.2 equiv. of Me_2_S were used.

^*f*^2 equiv. of Me_2_S were used.

^*g*^0.05 M.

^*h*^0.3 mol% Co(OAc)_2_ were used.

^*i*^3 equiv. of Me_2_S were used.

^*j*^0.5 mol% Co(OAc)_2_ were used and Me_2_S was added after **1** was consumed.

Our approach, based on using a radical DA and molecular oxygen, thus provided access to previously unattained C–H oxidation protocols. Especially the following three points should be worth noting: firstly, the C–H oxygenation proceeded only at specific and predictable positions depending on the accessibility of the *N*-oxyl radical moiety in DA. For example, the very challenging substrate **1d** possesses a flexible alkyl chain, but was converted into a 1.2 : 1 mixture of γ-oxo (**2d**) and δ-oxo (**2d′**) products. Conversely, the corresponding α-, β-, and ε-oxo products were not detected or detected only in trace amounts. Oxygenation of substrates **1e–1g**, containing an ester or a phthalimide moiety, occurred exclusively at the γ-position.^21^ Moreover, β-tertiary C–H bonds were observed to be significantly less reactive than γ-tertiary C–H bonds (**2i***vs.***2j**; **1j** was unreactive in 24 h at 40 °C). Benzylic C–H oxygenations also showed a similar reactivity tendency.^16^ Substrates containing two or more tertiary, benzylic, and propargylic positions such as (*R*)-**1k**, **1l**, **1z**, **1aa**, and **1ac** afforded products that were selectively oxygenated at the γ-position. For DA-bound (+)-menthol, *i.e.* a diastereoisomer mixture of (*R*)-**1k** and (*S*)-**1k**, containing three tertiary C(sp^3^)–H bonds, only one specific C–H bond of (*R*)-**1k** was converted into a C–OH bond. The corresponding product (*R*)-**2k** exhibited a partial isopulegol hydrate structure and was obtained within 2 h in almost quantitative yield. However, diastereomer (*S*)-**1k** was completely unreactive. The contrasting reactivity between these two diastereomers is probably due to the accessibility of the C–H bond to the intramolecular *N*-oxyl radical moiety, as suggested by the X-ray structure of *O*-(4-nitrobenzyl)-(*R*)-**1k** and molecular modeling.^16^ This notion is supported particularly by the observation that the chemoselectivity can be switched, depending on the position of a specific C–H bond to the *N*-oxyl radical: in case of **1o**, oxygenation was selective towards a γ-tertiary C–H bond rather than towards a propargyl C–H bond, whereas oxygenation of **1ac** was selective towards a propargyl C–H bond rather than towards a tertiary C–H bond.

Secondly, various oxidation-sensitive functional groups were tolerated, due to the mild reaction conditions employed that avoid the use of reactive oxidants. Thus, C–H oxygenation could be conducted in the presence of electron-rich (hetero)aromatic rings (**2u**, **2ad** and **2ae**), haloarenes (**2v** and **2x**), a silyl arene (**2y**), aryl and alkyl boronates (**2q** and **2w**), a terminal hydroxy group (**2m**), an acetal (**2n**), ethers (**2n**, **2p**, and **2u**), a C=C double bond (**2p**), and a C≡C triple bond (**2o** and **2aa–2ac**).

Thirdly, the DA approach was able to override the innate reactivity difference between C–H bonds. The observed regioselectivity in the reaction of (*R*)-**1k** differed from that in the previously reported Fe- or Ru-catalyzed C(sp^3^)–H hydroxylation of *O*-acylmenthol.^22^ In addition, the γ-tertiary C–H bond was selectivity oxygenated in the reactions of **1m**, **1n**, **1o**, and **1p**, even though those compounds contain more reactive α-hydroxy (**1m**), acetal methylene (**1n**), propargylic (**1o**), and allylic (**1p**) C–H bonds.

### An extension to ultra-remote aerobic C–H oxygenation

**Scheme 3:**
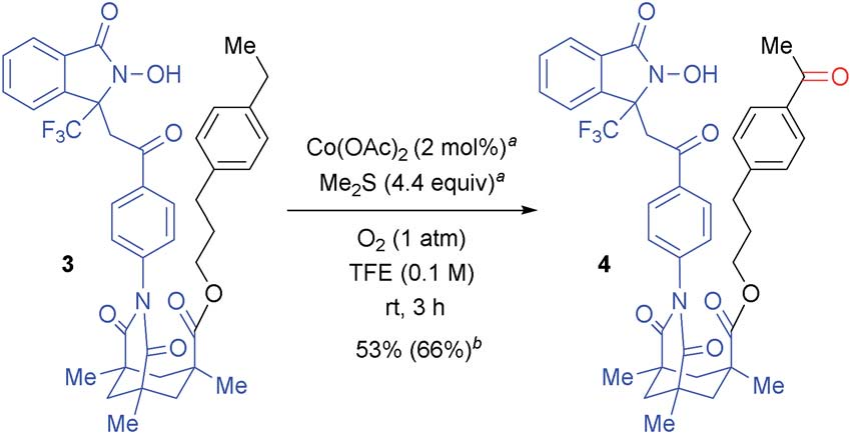
Selective ultra-remote aerobic C–H oxygenation. ^*a*^ 1 mol% Co(OAc)_2_ and 2.2 equiv. of Me_2_S were added at 0 h and 1.5 h. ^*b*^ Isolated yield is described and yield in parenthesis was calculated from the ^1^H NMR spectra of the crude mixture using 1,1,2,2-tetrachloroethane as an internal standard.

The regioselectivity can be changed *via* the linker structure. As shown in Scheme 3, Kemp's triacid^23^-based “long-arm” DA exclusively oxygenated the remote benzylic position of 3-(4-ethylphenyl)propan-1-ol (**3**), thus demonstrating complementary regioselectivity to the oxygenation of **1z** (Table 2). The DA-promoted remote C–H oxidation was pioneered by Breslow,^24^ but selective and remote C–H oxygenation *with use of molecular oxygen* has not yet been reported in the literature. This result thus provides an opportunity for a controlled switching of the oxygenation site by designing a suitable linker between the target C(sp^3^)–H bond and the reactive *N*-oxyl radical moiety.^20^

### Confirmation of intramolecularity

**Scheme 4:**
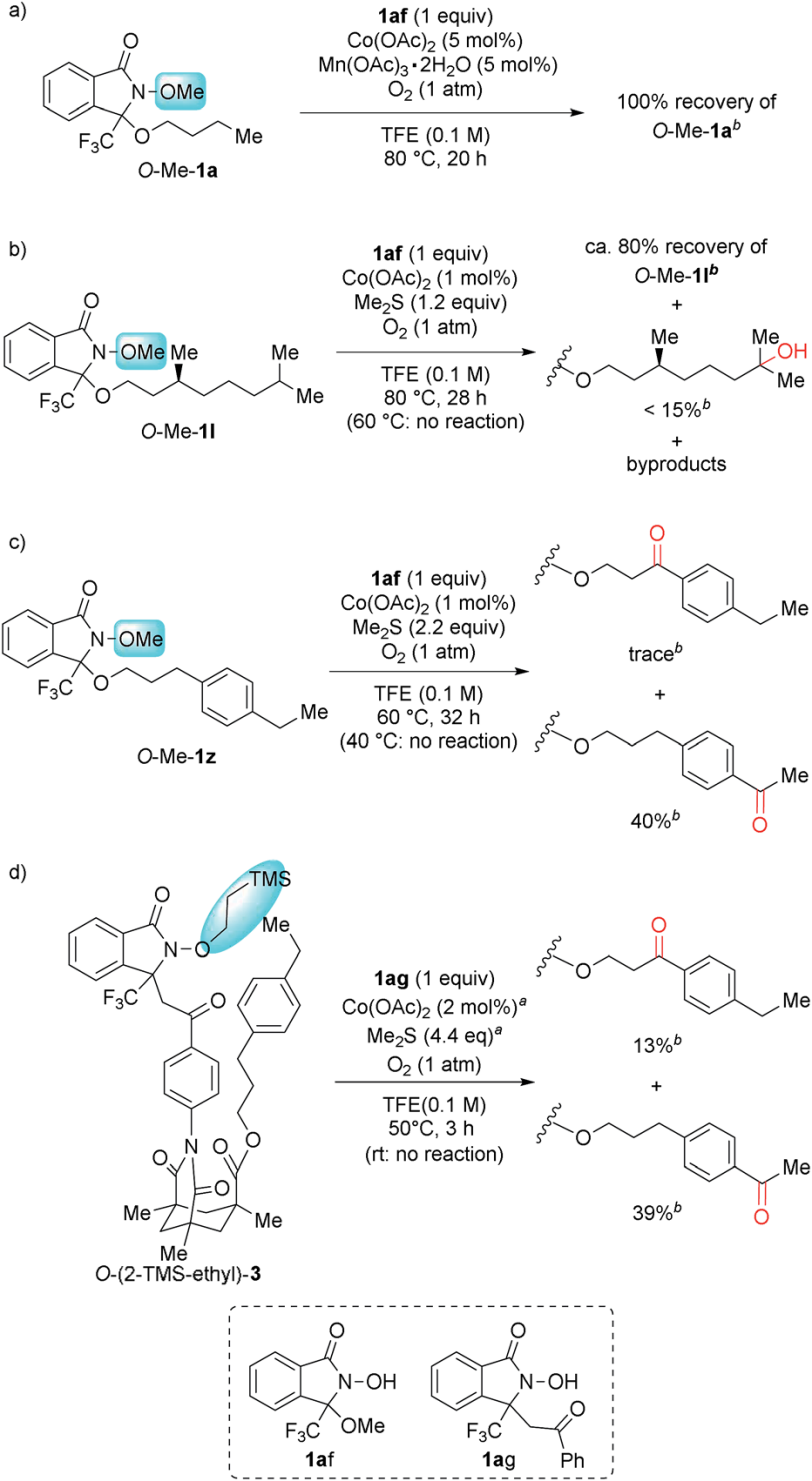
Confirmation of an intramolecular reaction mechanism. *^a^* 1 mol% Co(OAc)_2_ and 2.2 equiv. of Me_2_S were added at 0 h and 1.5 h. *^b^* The yield was calculated from the ^1^H NMR spectra of the crude mixture using 1,1,2,2-tetrachloroethane as an internal standard.

To verify the intramolecular nature of the current C(sp^3^)–H oxygenation, we conducted several control experiments (Scheme 4). Intermolecular C–H oxygenation of *O*-protected **1** or **3** (*O*-Me-**1a**, *O*-Me-**1l**, *O*-Me-**1z**, *O*-(2-TMS-ethyl)-**3**) promoted by an equimolar amount of DA-bound methanol (**1af**) or acetophenone (**1ag**) produced a significantly different profile compared to oxygenation, shown in Table 2 and Scheme 3. Thus, intermolecular methylene oxygenation of *O*-Me-**1a** in the presence of **1af** resulted in complete recovery of unchanged *O*-Me-**1a** after 20 h at 80 °C (Scheme 4-a), while **1a** was converted to the corresponding ketone **2a** in 67% yield after 18 h at 40 °C (Table 2). As for tertiary C–H oxygenation, while **1l** was converted to the corresponding γ-alcohol **2l** in 88% yield for 6 h at 40 °C (Table 2), *O*-Me-**1l** remained unaffected in the intermolecular oxygenation with **1af** for 24 h at 60 °C. The oxygenation started to proceed at 80 °C, but the η-alcohol was obtained as the major product in low yield (<15% yield, Scheme 4-b). In the case of benzylic oxygenation, **1z** was converted to ketone **2z** in 72% yield in 31 h at 40 °C with perfect γ-selectivity (Table 2), while the intermolecular oxygenation of *O*-Me-**1z** with **1af** proceeded only at 60 °C, and the sterically less-hindered remote benzylic position was predominantly oxygenated in moderate yield (40%, Scheme 4-c). Compound **3** was converted to **4** in 3 h at room temperature in 66% yield with perfect remote selectivity (Scheme 3), while *O*-(2-TMS-ethyl)-**3** with **1ag** required a raised temperature (50 °C), and the position selectivity was moderate (Scheme 4-d).

These contrasting results in four types of substrates strongly suggest that an intramolecular DA-promoted C–H activation is crucial for the success of the method described herein.

### Plausible reaction mechanism

**Scheme 5:**
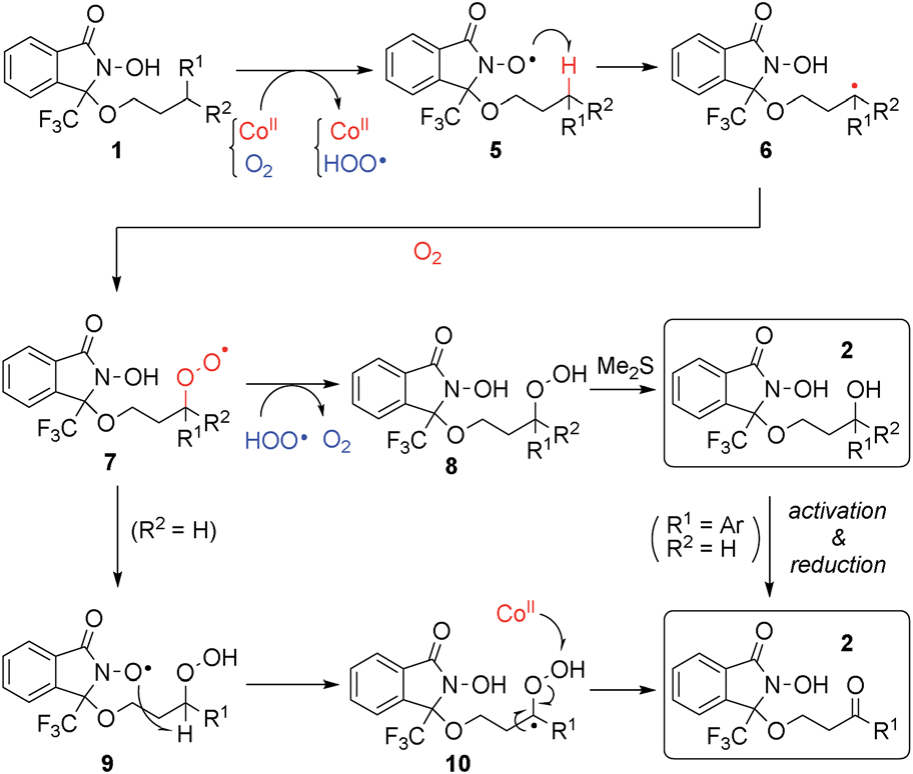
Plausible reaction mechanism.

We propose a plausible reaction mechanism in Scheme 5. The initiation step must be the one-electron oxidation of the *N*-hydroxy group of **1** by Co(iii) species, which are generated through the reaction between Co(ii) and O_2_.^25^ The thus-generated *N*-oxyl radical **5** abstracts a hydrogen atom of a C–H bond at a proximate position, generating carbon radical species **6**. Trapping **6** with molecular oxygen, the resulting alkyl peroxy radical **7** is quenched by a hydroperoxy radical generated in the initiation step to produce alkyl peroxide **8**, or through intramolecular hydrogen abstraction from DA, generating **9**. In the case of tertiary C–H oxygenation, undesired decomposition pathway from **8** (see Scheme 2)^18^ is suppressed by *in situ* reduction with Me_2_S to produce the corresponding tertiary alcohols. In the case of methylene oxygenation, the corresponding ketone is produced either through β-elimination from alkylperoxy species **8** or **9**, or oxidation of the secondary alcohol generated from **8** with Me_2_S.

## Conclusions

In conclusion, we have developed a method for the regioselective C(sp^3^)–H oxygenation of aliphatic alcohols, using an *N*-oxyl radical group as a directing activator. Benzylic, propargylic, tertiary, and even the very challenging acyclic methylene C(sp^3^)–H bonds were thus converted to C=O or C–OH bonds under mild conditions (room temperature to 50 °C), while high functional group tolerance was maintained. Molecular oxygen was used as the stoichiometric oxidant, and the reactions proceeded regioselectively at the γ (and δ) position(s), whereas the α, β, and other positions beyond the δ position remained intact. This regioselectivity can be explained in terms of the intramolecular accessibility of the reactive *N*-oxyl radical site, despite the low regioselectivity between γ and δ positions in electronically non-biased substrates is a current limitation that must be solved in future works. Preliminary structural tuning of the DA led to an alteration of the regioselectivity, providing a selective ultra-remote aerobic C–H oxygenation. Although laborious synthesis of DA-bound substrates has remained problematic at this stage, devising catalytic applications of DAs will overcome this limitation. Efforts in such a direction are currently ongoing in our laboratory.

## Supplementary Material

Supplementary information

Crystal structure data
