# Supplementary material for: Chemo- and regioselective oxygenation of C(sp3)–H bonds in aliphatic alcohols using a covalently bound directing activator and atmospheric oxygen
Source: Chem Sci. 2015 Nov 27;7(3):1904–9. doi: 10.1039/c5sc04476f (PMC5965247; doi:10.1039/c5sc04476f)

Electronic Supplementary Material (ESI) for Chemical Science.
This journal is © The Royal Society of Chemistry 2015

Supporting Information

Chemo- and Regioselective Oxygenation of C(sp^3)–H Bonds in Aliphatic Alcohols Using a Covalently Bound Directing Activator and Atmospheric Oxygen

Jun Ozawa, Masayuki Tashiro, Jizhi Ni, Kounosuke Oisaki,* and Motomu Kanai*

Graduate School of Pharmaceutical Sciences, The University of Tokyo

Kanai Life Science Catalysis Project, ERATO, Japan Science Technology Agency,

7-3-1, Hongo, Bunkyo-ku, Tokyo 113-0033, Japan.

Phone: +81-3-5841-4835 (Oisaki); +81-3-5841-4830 (Kanai)

Fax: +81-3-5684-5206

Email: oisaki@mol.f.u-tokyo.ac.jp (Oisaki); kanai@mol.f.u-tokyo.ac.jp (Kanai)

Contents

1. **General method** S2
2. **Typical experimental procedure** S3
3. **Typical synthetic procedure for directing-activator-anchored alcohols** S5
4. **The procedure for gram-scale oxidation and the removal of the directing activator** S11
5. **Experimental details** S15
6. **Selective ultra-remote C-H oxygenation** S19
7. **Analytical data** S24

1. General method

^{1}H NMR spectra were recorded on JEOL ECX-500 (500.16 MHz for ^{1}H NMR and 125.77 MHz for ^{13}C NMR), and JEOL ECS-400 (391.78 MHz for ^{1}H NMR, 98.52 MHz for ^{13}C NMR, 125.70 MHz for ^{11}B NMR, and 368.64 MHz for ^{19}F NMR) spectrometers. Chemical shifts were reported downfield from tetramethylsilane (δ = 0 ppm) for ^{1}H NMR. For ^{1}H and ^{13}C NMR, chemical shifts were reported in the scale relative to the solvent used as an internal reference (δ = 7.26 and 77.00 ppm ($CDCl_3$), 2.04 and 29.80 ppm (acetone-d_6); ^{1}H and ^{13}C NMR respectively). Chemical shifts for ^{11}B NMR and ^{19}F NMR were reported in the scale relative to $PhB(OH)_2$ (δ = 31.00 ppm) and C_6F_6 (δ = −164.90 ppm) respectively as an external standard. Infrared (IR) spectra were recorded on a JASCO FT/IR 410 Fourier transform infrared spectrophotometer. ESI-MS spectra were measured on a Waters ZQ4000 spectrometer (for LRMS), and a JEOL JMS-T100LC AccuTOF spectrometer (for HRMS). Column chromatography was performed with silica gel Merck 60 (230-400 mesh ASTM). Gel permeation chromatography (GPC) purification was conducted on a Japan Analytical Industry Co., Ltd. LC9210NEXT equipped with JAIGEL-1H and JAIGEL-2H, and $CHCl_3$ was used as an eluent. HPLC was conducted by JASCO HPLC systems (pump: PU-2080; detector: UV-2075, measured at 254 nm). All reactions other than substrates synthesis were carried out in normal solvents without any purification (purchased from Aldrich or Wako Pure Chemical Industries, Ltd.) unless otherwise noted. Reagents whose preparations are not described in this manuscript were purchased from Aldrich, Tokyo Chemical Industry Co., Ltd. (TCI), Kanto Chemical Co., Inc., and Wako Pure Chemical Industries, Ltd., and used without further purification except for triethylamine, which was distilled from CaH_2. NMR yields were calculated by ^{1}H NMR of crude products using 1,1,2,2-tetrachloroethane as an internal standard. The oxygenated compounds **2a**, **2d** + **2d'**, **2h**, **2i**, **2k**, **2m**, and **2s** were isolated by silica gel column chromatography (eluent: hexane/EtOAc); other products were isolated by preparative TLC (eluent: hexane/EtOAc).

2. Typical experimental procedure

(A) The oxygenation of secondary C(*sp*^{3})-H bonds:

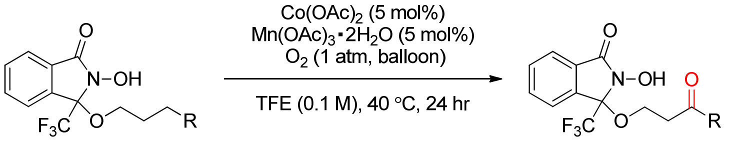

1 **2**

Oxygenation of **1a**: To a test tube were added $Co(OAc)_2$ (0.89 mg, 0.005 mmol), $Mn(OAc)_3{\cdot}2H_2O$ (1.34 mg, 0.005 mmol), **1a** (R = Me, 28.9 mg, 0.1 mmol), and 2,2,2-trifluoroethanol (TFE,1 mL, 0.1 M). The mixture was stirred at 40 °C after replacing the air inside the tube with O_2 (1 atm, balloon). After **1a** was consumed, which was checked on TLC, TFE was removed by evaporation and then EtOAc was added. The EtOAc solution was passed through silica gel to remove the metals. EtOAc was removed from the filtrate by evaporation and the residue was dried under reduced pressure to afford the crude mixture. NMR yield was calculated as 67% from the ^{1}H NMR spectra of the crude mixture using 1,1,2,2-tetrachloroethane as an internal standard. Purification by silica gel column chromatography (EtOAc/hexane = 1/1) afforded **2a** as a colorless sticky liquid (18.6 mg, 62%).

2a: ^{1}H NMR (500 MHz, $CDCl_3$) δ: 8.83 (brs, 1H), 7.87 (d, 1H, *J* = 7.5 Hz), 7.58-7.68 (m, 3H), 3.57 (dt, 1H, *J* = 3.5 Hz, 9.8 Hz), 3.21-3.26 (m, 1H), 2.93 (ddd, 1H, *J* = 3.5 Hz, 9.2 Hz, 18.9 Hz), 2.63 (dt, 1H, *J* = 18.9 Hz, 3.5 Hz), 2.23 (s, 3H); ^{13}C NMR (126 MHz, $CDCl_3$) δ: 208.3, 163.8, 133.9, 133.0, 131.7, 130.5, 124.2, 124.1, 121.7 (q, *J* = 286.7 Hz), 90.5 (q, *J* = 32.4 Hz), 57.7, 42.1, 30.4; ^{19}F NMR (369 MHz, $CDCl_3$) δ: –78.2; IR (neat, cm^{-1}) ν: 3213, 2952, 1719, 1469, 1363, 1304, 1197; LRMS (ESI): *m/z* 326 $[M+Na]^+$; HRMS (ESI): *m/z* calcd for $C_{13}H_{12}F_3NO_4Na$ $[M+Na]^+$ 326.0611, Found 326.0620.

(B) The oxygenation of tertiary or benzylic C(*sp*^{3})-H bonds:

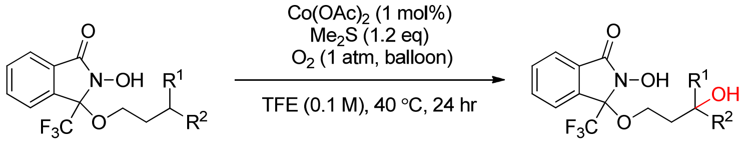

1 **2**

Oxygenation of **1h**: To a test tube were added **1h** ($R^1 = R^2$ = Me, 30.3 mg, 0.1 mmol) and TFE (1 mL, 0.1 M). Me_2S (9 μL, 0.12 mmol) was added after replacing the air inside the tube with O_2 (1 atm, balloon) and finally $Co(OAc)_2$ (0.5 M DMSO solution, 2 μL, 0.001 mmol) was added. The mixture was stirred at 40 °C and after **1h** was consumed, which was checked on TLC, TFE was removed by evaporation and then EtOAc was added. The EtOAc solution was passed through silica gel to remove the metal and DMSO. EtOAc was removed from the filtrate by evaporation and the residue was dried under reduced pressure to afford the crude mixture. NMR yield was calculated as 100% from the ^{1}H NMR spectra of the crude mixture using 1,1,2,2-tetrachloroethane as an internal standard. Purification by silica gel column chromatography (EtOAc/hexane = 1/1) afforded **2h** as a colorless sticky liquid (29.6 mg, 93%).

2h: ^{1}H NMR (500 MHz, $CDCl_3$) δ: 9.88 (s, 1H), 7.84 (d, 1H, *J* = 7.5 Hz), 7.64 (dd, 1H, *J* = 7.5 Hz, 7.5 Hz), 7.59-7.64 (m, 2H), 3.80 (brs, 1H), 3.55 (dt, 1H, *J* = 4.0 Hz, 9.8 Hz), 3.25-3.32 (m, 1H), 1.93-2.02 (m, 1H),

1.69 (dt, 1H, *J* = 14.9 Hz, 4.1 Hz), 1.37 (s, 3H), 1.25 (s, 3H); ^{13}C NMR (126 MHz, $CDCl_3$) δ: 164.9, 134.4, 133.0, 131.6, 130.3, 124.1, 124.0, 121.8 (q, *J* = 286.7 Hz), 90.9 (q, *J* = 32.4 Hz), 70.7, 60.8, 40.9, 30.0; ^{19}F NMR (369 MHz, $CDCl_3$) δ: –78.2; IR (neat, cm^{-1}) ν: 3395, 3172, 2972, 1722, 1470, 1381, 1305, 1199; LRMS (ESI): *m/z* 342 $[M+Na]^+$; HRMS (ESI): *m/z* calcd for $C_{14}H_{16}F_3NO_4Na$ $[M+Na]^+$ 342.0924, Found 342.0921.

3. Typical synthetic procedure for directing-activator-anchored alcohols

Procedure A: for all the substrates except for ones listed bellow

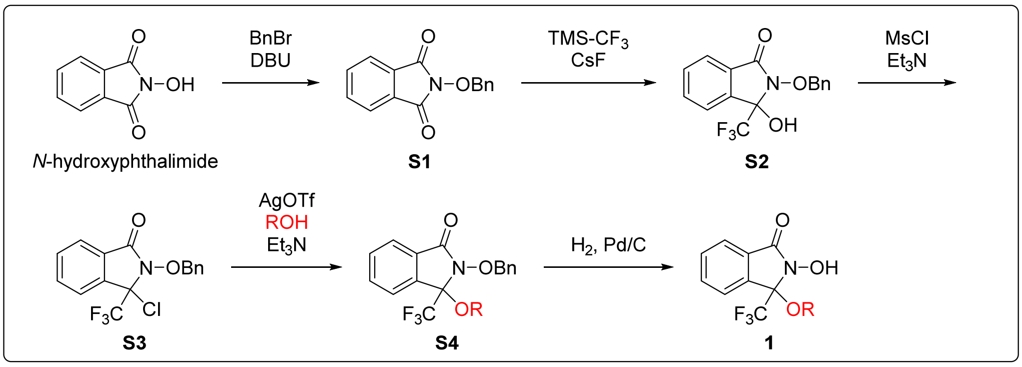

Procedure B: for **1o**, **1v**, **1x**, **1aa**, **1ab**, **1ac** and **1ad**

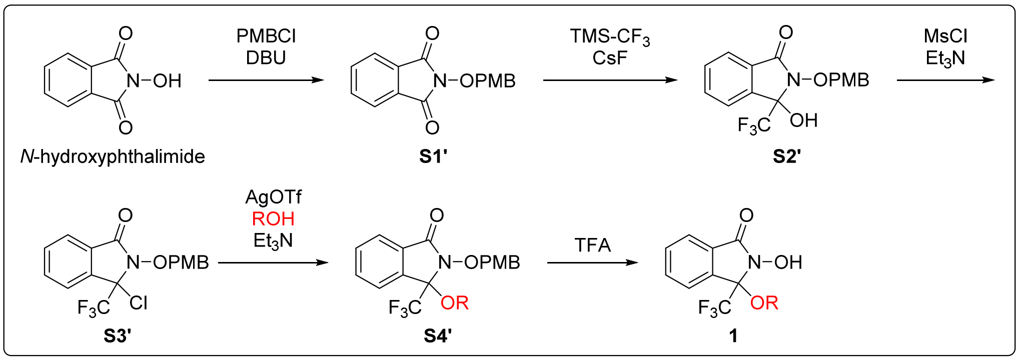

Procedure C: for **1p**

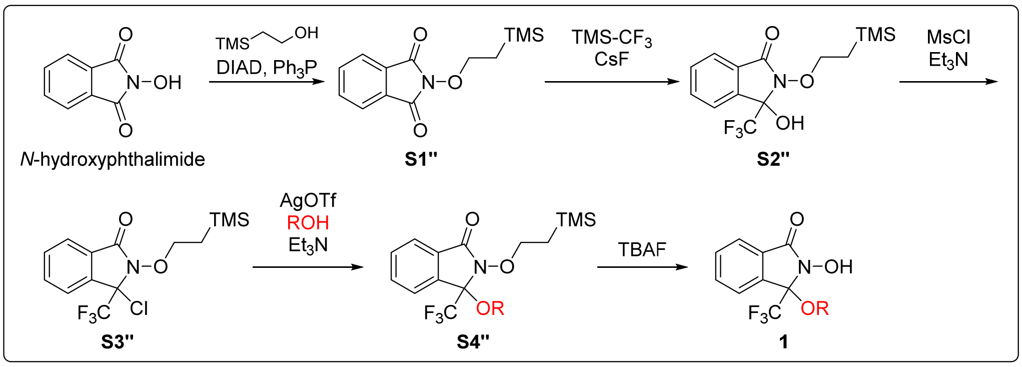

Typical experimental procedure for Procedure A: *O*-benzyl-*N*-hydroxyphthalimide (S1)

To a stirred mixture of *N*-hydroxyphthalimide (97.8 g, 600 mmol, 1 eq) and benzyl bromide (79 mL, 660 mmol, 1.1 eq) in DMF (1.2 L, 0.5 M) was added DBU (108 mL, 720 mmol, 1.2 eq) over 30 minutes at ambient temperature. After completion of the addition, 2.4 L of HCl (1 N in water) was added, and the white precipitate was filtered off and washed with water to afford wet **S1**. Azeotropic removal of remained water with toluene (twice) afforded **S1** as white solid (147.6 g, 97% yield).

^{1}H NMR (500 MHz, $CDCl_3$) δ: 7.80 (dd, 2H, *J* = 2.9 Hz, 5.2 Hz), 7.72 (dd, 2H, *J* = 2.9 Hz, 5.2 Hz), 7.53 (dd, 2H, *J* = 4.0 Hz, 7.5 Hz), 7.36-7.40 (m, 3H), 5.21 (s, 2H); ^{13}C NMR (126 MHz, $CDCl_3$) δ: 163.4, 134.4, 133.6, 129.8, 129.3, 128.8, 128.5, 123.4, 79.8; IR (KBr, cm^{-1}) ν: 3076, 3034, 2954, 2887, 1789, 1731, 1464; LRMS (ESI): *m/z* 276 $[M+Na]^+$; HRMS (ESI): *m/z* calcd for $C_{15}H_{11}NO_3Na$ $[M+Na]^+$ 276.0631,

Found 276.0630.

***N*-benzyloxy-3-hydroxy-3-trifluoromethyl-isoindolinone (S2)**

To a stirred mixture of **S1** (139 g, 550 mmol, 1 eq) and $TMSCF_3$ (98mL mL, 660 mmol, 1.2 eq) in dry DMF (550 mL, 1 M) was added CsF (100 g, 660 mmol, 1.2 eq) at 0 °C, and the reaction mixture was stirred for one hour. 1 N HCl aq. (500 mL) was added and the mixture was extracted with ethyl acetate/hexane (1/1). The organic layer was washed with water and saturated NaCl aq. solution, and dried over Na_2SO_4. The solution was passed through short silica gel pad and evaporation of the solvent afforded **S2** as light yellow solid (169.3 g, 95% yield). **S2** was used in the next step without further purification.

^{1}H NMR (500 MHz, acetone-d_6) δ: 7.78-7.85 (m, 3H), 7.72-7.76 (m, 1H), 7.59 (d, 2H, J = 7.5 Hz), 7.37-7.45 (m, 3H), 5.34 (d, 1H, J = 9.8 Hz), 5.22 (d, 1H, J = 9.8 Hz); ^{13}C NMR (126 MHz, acetone-d_6) δ: 164.9, 139.1, 136.0, 134.5, 132.5, 130.2, 130.0, 129.5, 129.1, 125.0, 124.2, 123.9 (q, J = 286.7 Hz), 88.0 (q, J = 32.4 Hz), 80.5; ^{19}F NMR (369 MHz, acetone-d_6) δ: –79.5; IR (KBr, cm^{-1}) ν: 3223, 3031, 1719, 1618, 1470, 1378, 1261, 1195; LRMS (ESI): *m/z* 346 $[M+Na]^+$; HRMS (ESI): *m/z* calcd for $C_{16}H_{12}F_3NO_3Na$ $[M+Na]^+$ 346.0662, Found 346.0674.

***N*-benzyloxy-3-chloro-3-trifluoromethyl-isoindolinone (S3)**

To a stirred mixture of **S2** (169 g, 523 mmol, 1 eq) and triethylamine (117 mL, 836 mmol, 1.6 eq) in CH_2Cl_2 (45 mL, 1 M) was added methanesulfonyl chloride (61 mL, 784 mmol, 1.5 eq) at 0 °C. The mixture was stirred for three hours before water was added. The separated organic layer was washed with water and saturated NaCl aq. solution, and dried over Na_2SO_4. The solution was passed through short silica gel pad, and evaporation of the solvent afforded **S3** as light yellow liquid (179 g, 100% yield). For practical use, **S3** is stored as 1 M solution in heptane, or **S3** is solidified by seeding.

^{1}H NMR (400 MHz, $CDCl_3$) δ: 7.90 (d, 1H, J = 7.2 Hz), 7.71-7.76 (m, 2H), 7.63-770 (m, 1H), 7.55-7.61 (m, 2H), 7.37-7.46 (m, 3H), 5.41 (d, 1H, J = 9.4 Hz), 5.25 (d, 1H, J = 9.4 Hz); ^{13}C NMR (126 MHz, $CDCl_3$) δ: 164.5, 137.9, 134.2, 133.9, 131.8, 129.6, 129.1, 128.5, 127.3, 124.4, 124.1, 121.7 (q, J = 284.3 Hz), 79.7, 79.5 (q, J = 36.4 Hz); ^{19}F NMR (369 MHz, $CDCl_3$) δ: –75.8; IR (neat, cm^{-1}) ν: 1755, 1469, 1258, 1197; LRMS (ESI): *m/z* 364 $[M+Na]^+$; HRMS (ESI): *m/z* calcd for $C_{16}H_{11}ClF_3NO_2Na$ $[M+Na]^+$ 364.0323, Found 364.0325.

***N*-benzyloxy-3-butoxy-3-trifluoromethyl-isoindolinone (S4a)**

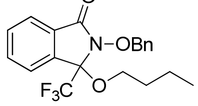

To a light-protected mixture of AgOTf (6.9 g, 26.7 mmol, 1.5 eq), 1-butanol (2.0 mL, 21.4 mmol, 1.2 eq), and triethylamine (3.2 mL, 23.1 mmol, 1.3 eq) in toluene (17.8 mL) was added **S3** (1 M solution in toluene, 17.8 mL, 17.8 mmol, 1 eq) at ambient temperature. The reaction mixture was stirred overnight and saturated NaCl aq. solution was added. After filtration over Celite pad to remove the silver salts, the organic layer was separated, washed with water and saturated NaCl aq. solution, and dried over Na_2SO_4.

Evaporating the solvent afforded the crude oily liquid. Purification by silica gel column chromatography (hexane/EtOAc = 10/1) afforded **S4a** (R = Bu) as slightly yellow liquid (5.7 g, 84%).
^{1}H NMR (500 MHz, $CDCl_3$) δ: 7.90 (d, 1H, *J* = 7.7 Hz), 7.69 (dd, 1H, *J* = 6.9 Hz, 7.8 Hz), 7.60-7.66 (m, 2 H), 7.57 (d, 2H, *J* = 6.9 Hz), 7.35-7.43 (m, 3H), 5.32 (d, 1H, *J* = 9.8 Hz), 5.16 (d, 1H, *J* = 9.8 Hz), 3.28 (dt, 1H, *J* = 8.6 Hz, 6.3 Hz), 2.95 (dt, 1H, *J* = 8.6 Hz, 6.3 Hz), 1.44-1.59 (m, 2H), 1.23-1.39 (m, 2H), 0.83 (t, 3H, *J* = 7.2 Hz); ^{13}C NMR (126 MHz, $CDCl_3$) δ: 165.6, 135.3, 134.6, 133.4, 131.6, 130.0, 129.3, 128.8, 128.4, 124.2, 124.1, 122.2 (q, *J* = 286.7 Hz), 91.0 (q, *J* = 32.4 Hz), 79.1, 63.8, 31.0, 18.9, 13.6; ^{19}F NMR (369 MHz, $CDCl_3$) δ: –78.1; IR (neat, cm^{-1}) ν: 2960, 1746, 1468, 1294, 1195; LRMS (ESI): *m/z* 402 $[M+Na]^+$; HRMS (ESI): *m/z* calcd for $C_{20}H_{20}F_3NO_3Na$ $[M+Na]^+$ 402.1288, Found 402.1284.

3-butoxy-*N*-hydroxy-3-trifluoromethyl-isoindolinone (1a)

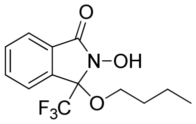

A stirred mixture of **S4a** (2.6 g, 6.8 mmol, 1 eq) and Pd/C (10 wt%, 362 mg, 0.34 mmol, 0.05 eq) in ethanol (14 mL, 0.5 M) was exposed to H_2 (balloon pressure) at ambient temperature. The reaction mixture was stirred for two hours and then H_2 was removed. Filtration over Celite pad and evaporation of the filtrate afforded a crude oily liquid. Purification by silica gel column chromatography (hexane/EtOAc = 4/1) afforded **1a** as slightly orange liquid. The liquid was gradually solidified into white solid (1.8 g, 92%).
^{1}H NMR (500 MHz, $CDCl_3$) δ: 9.88 (s, 1H), 7.79 (d, 1H, *J* = 7.8 Hz), 7.66 (dd, 1H, *J* = 7.4 Hz, 6.9 Hz), 7.61 (d, 1H, *J* = 7.4 Hz), 7.60 (dd, 1H, *J* = 6.9 Hz, 7.8 Hz), 3.43 (dt, 1H, *J* = 8.6 Hz, 6.6 Hz), 2.98 (dt, 1H, *J* = 8.6 Hz, 6.6 Hz), 1.54-1.68 (m, 2H), 1.29-1.46 (m, 2H), 0.88 (t, 3H, *J* = 7.2 Hz); ^{13}C NMR (126 MHz, $CDCl_3$) δ: 165.9, 135.3, 133.2, 131.5, 130.0, 124.0, 123.8, 121.9 (q, *J* = 286.7 Hz), 91.0 (q, *J* = 32.4 Hz), 64.1, 31.1, 19.0, 13.7; ^{19}F NMR (369 MHz, $CDCl_3$) δ: –78.5; IR (KBr, cm^{-1}) ν: 3435, 3136, 2964, 1878, 1719, 1472, 1305, 1201; LRMS (ESI): *m/z* 312 $[M+Na]^+$; HRMS (ESI): *m/z* calcd for $C_{13}H_{14}F_3NO_3Na$ $[M+Na]^+$ 312.0818, Found 312.0831.

Typical experimental procedure for Procedure B

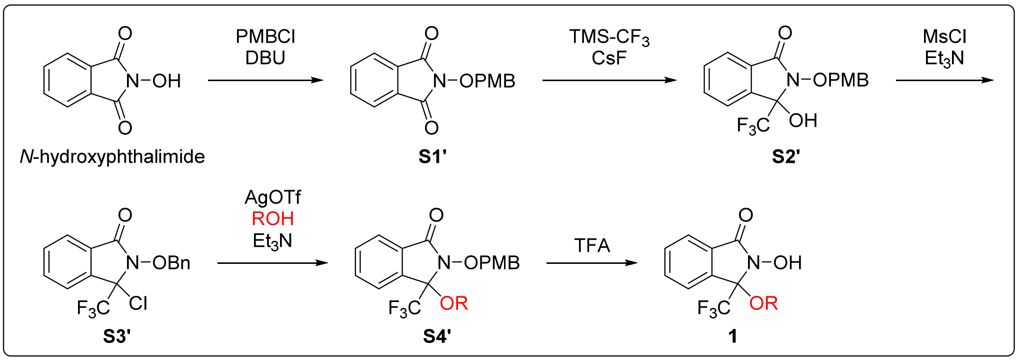

***O*-(4-methoxybenzyl)-*N*-hydroxyphthalimide (S1')**

To a stirred mixture of *N*-hydroxyphthalimide (8.16 g, 50 mmol, 1 eq) and DBU (9.7 mL, 65 mmol, 1.3 eq) in DMF (50 mL, 1 M) was added 4-methoxybenzyl chloride (6.6 mL, 55 mmol, 1.1 eq) at 0 °C. After two hours, 100 mL of HCl (1 N in water) was added. White precipitate was filtered off, washed with cold water, and then dissolved in CH_2Cl_2. Drying the solution over Na_2SO_4 and evaporating the solvent afforded **S1'** as white solid (11.0 g, 78% yield).

^{1}H NMR (500 MHz, $CDCl_3$) δ: 7.79 (dd, 2H, *J* = 5.4 Hz, 3.2 Hz), 7.72 (dd, 2H, *J* = 5.4 Hz, 3.2 Hz), 7.44 (d, 2H, *J* = 8.6 Hz), 6.88 (d, 2H, *J* = 8.6 Hz), 5.14 (s, 2H), 3.79 (s, 3H); ^{13}C NMR (126 MHz, $CDCl_3$) δ: 163.5, 160.4, 134.3, 131.6, 128.9, 125.8, 123.4, 113.9, 79.4, 55.2; IR (KBr, cm^{-1}) ν: 2945, 1725, 1611, 1516, 1386, 1259; LRMS (ESI): *m/z* 306 $[M+Na]^+$; HRMS (ESI): *m/z* calcd for $C_{16}H_{13}NO_4Na$ $[M+Na]^+$ 306.0737, Found 306.0745.

3-hydroxy-*N*-((4-methoxybenzyl)oxy)-3-(trifluoromethyl)isoindolinone (S2')

The same procedure as the conversion of **S1** to **S2** afforded **S2'** as light yellow solid (13 g, 97%) from 11 g of **S1'**. **S2'** was also used in the next step without further purification.

^{1}H NMR (500 MHz, acetone-d_6) δ: 7.76-7.84 (m, 3H), 7.70-7.75 (m, 1H), 7.51 (d, 2H, *J* = 8.0 H), 6.97 (d, 2H, *J* = 8.0 H), 5.26 (d, 1H, *J* = 9.2 Hz), 5.14 (d, 1H, *J* = 9.2 Hz), 3.82 (s, 3H); ^{13}C NMR (126 MHz, acetone-d_6) δ: 164.8, 161.1, 139.2, 134.4, 132.4, 132.0, 130.2, 128.1, 124.9, 124.2, 123.9 (q, *J* = 286.7 Hz), 114.5, 88.0 (q, *J* = 32.8 Hz), 80.2, 55.5; ^{19}F NMR (369 MHz, acetone-d_6) δ: –79.5; IR (KBr, cm^{-1}) ν: 3233, 1703, 1616, 1517, 1254, 1200; LRMS (ESI): *m/z* 376 $[M+Na]^+$; HRMS (ESI): *m/z* calcd for $C_{17}H_{14}F_3NO_4Na$ $[M+Na]^+$ 376.0767, Found 376.0773.

3-chloro-*N*-((4-methoxybenzyl)oxy)-3-(trifluoromethyl)isoindolinone (S3')

The same procedure as the conversion of **S2** to **S3** afforded **S3'** as yellow solid (11 g, 79%) from 13 g of **S2'**.

^{1}H NMR (400 MHz, $CDCl_3$) δ: 7.89 (d, 1H, *J* = 7.6 Hz), 7.62-7.77 (m, 3H), 7.50 (d, 2H, *J* = 8.5 Hz), 6.94 (d, 2H, *J* = 8.5 Hz), 5.33 (d, 1H, *J* = 9.0 Hz), 5.17 (d, 1H, *J* = 9.0 Hz), 3.82 (s, 3H); ^{13}C NMR (100 MHz, $CDCl_3$) δ: 164.5, 160.3, 137.9, 134.1, 131.8, 131.5, 127.4, 126.2, 124.4, 124.1, 121.7 (q, *J* = 284.4 Hz), 113.9, 79.6 (q, *J* = 36.3 Hz), 79.4, 55.2; ^{19}F NMR (369 MHz, $CDCl_3$) δ: –75.7; IR (KBr, cm^{-1}) ν: 1755, 1612, 1517, 1199; LRMS (ESI): *m/z* 394 $[M+Na]^+$; HRMS (ESI): *m/z* calcd for $C_{17}H_{13}ClF_3NO_3Na$ $[M+Na]^+$ 394.0428, Found 394.0425.

***N*-((4-methoxybenzyl)oxy)-3-(oct-4-yn-1-yloxy)-3-trifluoromethyl-isoindolinone (S4'aa)**

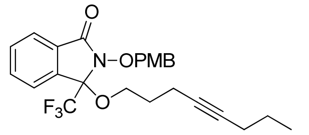

The same procedure as the conversion of **S3** to **S4a** afforded **S4'aa** as light yellow liquid (256.6 mg, 60%) from 116.1 mg of **S3'**.

^{1}H NMR (400 MHz, $CDCl_3$) δ: 7.87-7.92 (m, 1H), 7.60-7.71 (m, 3H), 7.45-7.51 (m, 2H), 6.90-6.95 (m,

2H), 5.25 (d, 1H, J = 9.4 Hz), 5.08 (d, 1H, J = 9.4 Hz), 3.82 (s, 3H), 3.38 (dt, 1H, J = 8.5 Hz, 5.7 Hz), 3.04-3.13 (m, 1H), 2.17-2.29 (m, 2H), 1.95-2.01 (m, 2H), 1.61-1.80 (m, 2H), 1.32-1.42 (m, 2H), 0.86 (t, 3H, J = 7.4 Hz); ^{13}C NMR (100 MHz, $CDCl_3$) δ: 165.3, 160.0, 134.9, 133.2, 131.5, 131.0, 130.0, 126.7, 124.2, 123.9, 122.0 (q, J = 286.4 Hz), 113.6, 90.9 (q, J = 32.9 Hz), 80.7, 78.7, 78.5, 62.3, 55.0, 28.3, 22.1, 20.4, 15.0, 13.2; ^{19}F NMR (369 MHz, $CDCl_3$) δ: –78.0; IR (neat, cm^{-1}) ν: 2960, 1744, 1613, 1516, 1468, 1252, 1186; LRMS (ESI): *m/z* 484 [M+Na]$^+$; HRMS (ESI): *m/z* calcd for $C_{25}H_{26}F_3NO_4Na$ [M+Na]$^+$ 484.1706, Found 484.1707.

N-hydroxy-3-(oct-4-yn-1-yloxy)-3-trifluoromethyl-isoindolinone (1aa)

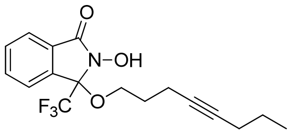

To a stirred mixture of **S4'aa** (250 mg, 0.54 mmol, 1 eq), pentamethylbenzene (326 mg, 2.2 mmol, 4 eq; a cation scavenger[1]) in CH_2Cl_2 (3 mL, 0.2 M) was added trifluoroacetic acid (267 μL, 10 wt% to the solvent). After half a day, saturated $NaHCO_3$ aq. solution was added. The organic layer was washed with water and saturated NaCl aq. solution, and dried over Na_2SO_4. Evaporating the solvent afforded a crude liquid. Purification by silica gel column chromatography (hexane/EtOAc = 3/1) afforded **1aa** (R = oct-4-yn-1-yl) as yellow liquid (151 mg, 82%).

^{1}H NMR (500 MHz, $CDCl_3$) δ: 9.28 (brs, 1H), 7.82 (d, 1H, J = 6.9 Hz), 7.57-7.68 (m, 3H), 3.44-3.50 (m, 1H), 3.13-3.18 (m, 1H), 2.29-2.34 (m, 2H), 1.72-1.85 (m, 2H), 1.37-1.45 (m, 2H), 0.88 (t, 3H, J = 7.4 Hz); ^{13}C NMR (126 MHz, $CDCl_3$) δ: 165.4, 135.0, 133.1, 131.5, 130.1, 124.2, 123.9, 121.9 (q, J = 286.7 Hz), 90.9 (q, J = 33.2 Hz), 81.3, 79.0, 62.5, 28.2, 22.3, 20.6, 15.1, 13.4; ^{19}F NMR (369 MHz, $CDCl_3$) δ: –78.4; IR (neat, cm^{-1}) ν: 3419, 2962, 1718, 1469, 1192; LRMS (ESI): *m/z* 364 [M+Na]$^+$; HRMS (ESI): *m/z* calcd for $C_{17}H_{18}F_3NO_3Na$ [M+Na]$^+$ 364.1131, Found 364.1138.

Typical experimental procedure for Procedure C

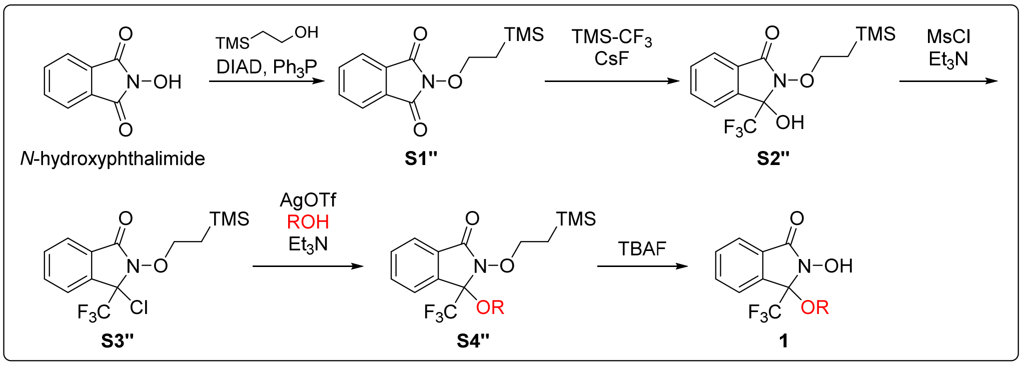

2-(2-(trimethylsilyl)ethoxy)isoindoline-1,3-dione (S1")

[1] K. Okano, K.-i. Okuyama, T. Fukuyama, H. Tokuyama *Synlett* **2008**, 1977-1980.

To a stirred mixture of *N*-hydroxyphthalimide (24.5 g, 150 mmol, 1 eq), 2-trimethylsilyl ethanol (21.4 mL, 150 mmol, 1 eq), and Ph_3P (43.3 g, 165 mmol, 1.1 eq) in anhydrous $CHCl_3$ (300 mL, 0.5 M) was added dropwise diethyl azodicarboxylate (2.2 M in toluene, 72 mL, 157 mmol, 1.05 eq) at 0 °C. After completion of the addition, the cold bath was removed and the mixture was allowed to stir at room temperature for about 12 hours. The reaction mixture was diluted with hexane/Et_2O (5/1) and was passed through SiO_2. Evaporation of the solvent afforded a crude mixture. Purification by silica gel column chromatography (hexane/ Et_2O = 5/1) afforded **S1''** as gray solid (24.6 g, 62% yield).

^{1}H NMR (500 MHz, $CDCl_3$) δ: 7.81 (dd, 2H, *J* = 5.4 Hz, 3.2 Hz), 7.72 (dd, 2H, *J* = 5.4 Hz, 3.2 Hz), 4.23-4.28 (m, 2H), 1.16-1.22 (m, 2H), 0.04 (s, 9H); ^{13}C NMR (126 MHz, $CDCl_3$) δ: 163.8, 134.3, 129.0, 123.4, 76.4, 17.0, –1.51; IR (neat, cm^{-1}) ν: 2953, 1790, 1723, 1467, 1383; LRMS (ESI): *m/z* 286 $[M+Na]^+$; HRMS (ESI): *m/z* calcd for $C_{13}H_{17}NO_3SiNa$ $[M+Na]^+$ 286.0870, Found 286.0869.

3-hydroxy-3-(trifluoromethyl)-2-(2-(trimethylsilyl)ethoxy)isoindolin-1-one (S2'')

The same procedure as the conversion of **S1** to **S2** afforded **S2''** as yellow solid (22.2 g, 71%) from 24.6 g of **S1''**.

^{1}H NMR (500 MHz, $CDCl_3$) δ: 7.75 (d, 1H, *J* = 7.4 Hz), 7.68 (d, 1H, *J* = 7.4 Hz), 7.63 (td, 1H, *J* = 7.4 Hz, 1.1 Hz), 7.57 (td, 1H, *J* = 7.4 Hz, 1.1 Hz), 4.88 (brs, 1H), 4.33-4.40 (m, 1H), 4.13-4.20 (m, 1H), 1.04-1.17 (m, 2H), 0.03 (s, 9H); ^{13}C NMR (126 MHz, $CDCl_3$) δ: 166.5, 137.8, 133.6, 131.5, 128.9, 124.2, 123.9, 122.6 (q, *J* = 286.7 Hz), 86.3 (q, *J* = 33.6 Hz), 76.1, 17.1, –1.56; ^{19}F NMR (369 MHz, $CDCl_3$) δ: –79.6; IR (KBr, cm^{-1}) ν: 3125, 2957, 1698, 1473, 1380, 1254, 1203, 1174; LRMS (ESI): *m/z* 356 $[M+Na]^+$; HRMS (ESI): *m/z* calcd for $C_{14}H_{18}F_3NO_3SiNa$ $[M+Na]^+$ 356.0900, Found 356.0903.

3-chloro-3-(trifluoromethyl)-2-(2-(trimethylsilyl)ethoxy)isoindolin-1-one (S3'')

The same procedure as the conversion of **S2** to **S3** afforded **S3''** as yellow liquid (23.4 g, 100%) from 22.2 g of **S2''**.

^{1}H NMR (500 MHz, $CDCl_3$) δ: 7.85 (d, 1H, *J* = 7.4 Hz), 7.67-7.74 (m, 2H), 7.62-7.66 (m, 1H), 4.42-4.50 (m, 1H), 4.28-4.35 (m, 1H), 1.13-1.23 (m, 2H), 0.06 (s, 9H); ^{13}C NMR (126 MHz, $CDCl_3$) δ: 164.5, 138.0, 134.0, 131.8, 127.6, 124.3, 124.1, 121.7 (q, *J* = 284.3 Hz), 79.6 (q, *J* = 36.4 Hz), 76.0, 17.1, –1.54; ^{19}F NMR (369 MHz, $CDCl_3$) δ: –75.9; IR (neat, cm^{-1}) ν: 1757, 1469, 1252, 1196; LRMS (ESI): *m/z* 374 $[M+Na]^+$; HRMS (ESI): *m/z* calcd for $C_{14}H_{17}ClF_3NO_2SiNa$ $[M+Na]^+$ 374.0561, Found 374.0567.

3-((3-methyloct-7-en-1-yl)oxy)-3-trifluoromethyl-*N*-(2-(trimethylsilyl)ethoxy)-isoindolinone (S4''p, diastereomixture)

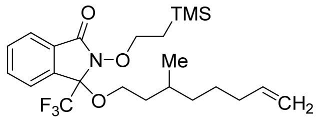

The same procedure as the conversion of **S3** to **S4a** afforded **S4''p** as colorless liquid (670.6 mg, 83%) from 619.1 mg of **S3''**.

^{1}H NMR (500 MHz, $CDCl_3$) δ: 7.87 (d, 1H + 1H, *J* = 6.9 Hz), 7.60-7.69 (m, 2H + 2H), 7.58 (d, 1H + 1H, *J* = 7.4 Hz), 5.72-5.82 (m, 1H + 1H), 4.90-5.00 (m, 2H + 2H), 4.33-4.40 (m, 1H + 1H), 4.15-4.22 (m, 1H + 1H), 3.30-3.38 (m, 1H + 1H), 2.93-3.01 (m, 1H + 1H), 1.95-2.05 (m, 2H + 2H), 1.49-1.68 (m, 2H + 2H), 1.00-1.44 (m, 7H + 7H), 0.81 (d, 3H, *J* = 6.9 Hz), 0.77 (d, 3H, *J* = 6.3 Hz), 0.06 (s, 9H + 9H); ^{13}C NMR (100 MHz, $CDCl_3$) δ: 165.52, 165.56, 138.93, 138.91, 135.3, 135.2, 133.2, 131.5, 130.3, 124.12, 124.07, 122.1 (q, *J* = 286.1 Hz), 114.31, 114.28, 90.8 (q, *J* = 32.7 Hz), 75.2, 62.4, 36.4, 36.1, 36.0, 35.9, 33.9, 29.5, 29.4, 26.1, 19.6, 19.3, 17.08, 17.05, –1.5; ^{19}F NMR (369 MHz, $CDCl_3$) δ: –78.3; IR (neat, cm^{-1}) ν: 3078, 2953, 1747, 1641, 1615, 1468, 1187; LRMS (ESI): *m/z* 480 $[M+Na]^+$; HRMS (ESI): *m/z* calcd for $C_{23}H_{34}F_3NO_3SiNa$ $[M+Na]^+$ 480.2152, Found 480.2153.

***N*-hydroxy-3-((3-methyloct-7-en-1-yl)oxy)-3-trifluoromethyl-isoindolinone (1p, diastereomixture)**

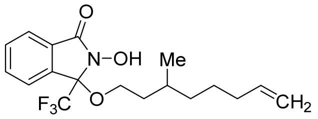

To a stirred mixture of **S4"p** (642 mg, 1.4 mmol, 1 eq) in THF (3 mL, 0.5 M) was added tetrabutylammonium fluoride trihydrate (1.3 g, 4.2 mmol, 3 eq). After three hours, saturated NH_4Cl aq. solution was added. The organic layer was washed with water and saturated NaCl aq. solution, and dried over Na_2SO_4. Evaporating the solvent afforded the crude liquid. Purification by silica gel column chromatography (hexane/EtOAc = 3/1) afforded **1p** (R = 3-methyloct-7-en-1-yl) as colorless liquid (470 mg, 93%).

^{1}H NMR (400 MHz, $CDCl_3$) δ: 9.69 (brs, 1H), 9.68 (brs, 1H), 7.77-7.82 (m, 1H + 1H), 7.57-7.69 (m, 3H + 3H), 5.72-5.85 (m, 1H + 1H), 4.90-5.02 (m, 2H + 2H), 3.42-3.51 (m, 1H + 1H), 2.95-3.05 (m, 1H + 1H), 1.95-2.05 (m, 2H + 2H), 1.51-1.75 (m, 2H + 2H), 1.18-1.49 (m, 4H + 4H), 1.02-1.18 (m, 1H + 1H), 0.85 (d, 3H, *J* = 6.3 Hz), 0.80 (d, 3H, *J* = 6.7 Hz); ^{13}C NMR (100 MHz, $CDCl_3$) δ: 165.8, 139.02, 138.99, 135.31, 135.30, 133.2, 131.5, 130.0, 124.0, 123.9, 121.9 (q, *J* = 286.1 Hz), 91.0 (q, *J* = 32.9 Hz), 62.68, 62.66, 36.4, 36.2, 36.0, 35.9, 33.95, 33.91, 29.5, 29.4, 26.14, 26.13, 19.5, 19.3; ^{19}F NMR (369 MHz, $CDCl_3$) δ: –78.6; IR (neat, cm^{-1}) ν: 3148, 2930, 1718, 1470, 1306, 1200; LRMS (ESI): *m/z* 380 $[M+Na]^+$; HRMS (ESI): *m/z* calcd for $C_{18}H_{22}F_3NO_3Na$ $[M+Na]^+$ 380.1444, Found 380.1457.

4. The procedure for gram-scale oxidation and the removal of the directing activator

There is difference from the typical procedure in the equivalent of the reagents.

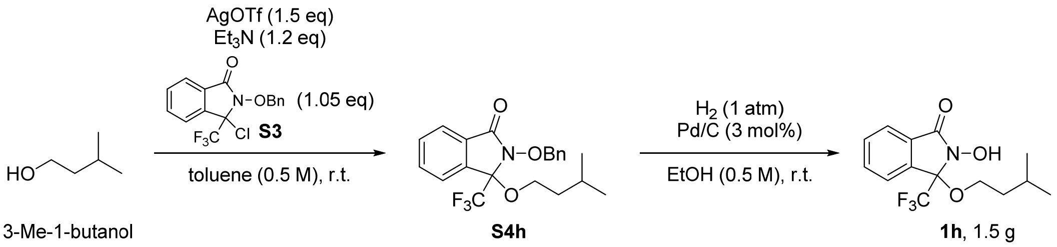

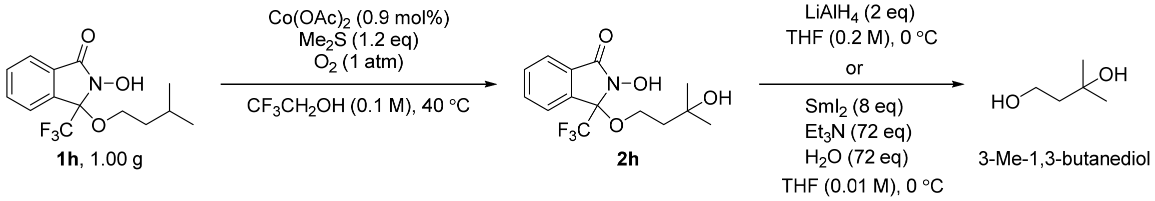

***N*-benzyloxy-3-isopentyloxy-3-trifluoromethyl-isoindolinone (S4h)**

To a light-protected round-bottom flask (dried by heat gun under reduced pressure) were added AgOTf (2.16 g, 8.39 mmol, 1.5 eq), dry toluene (11 mL, 0.5 M), 3-methyl-1-butanol (609 μL, 5.59 mmol, 1 eq), triethylamine (930 μL, 6.71 mmol, 1.2 eq), and finally dry toluene solution of **S3** (about 3 mL, 5.87 mmol, 1.05 eq) at ambient temperature. The reaction mixture was stirred overnight and saturated NaCl aq. solution was added. After filtration over Celite pad to remove the silver salts, the organic layer was washed with water and saturated NaCl aq. solution, and dried over Na_2SO_4. Evaporating the solvent afforded the crude oily liquid. Purification by silica gel column chromatography (hexane/EtOAc = 10/1) afforded **S4h** (R = isopentyl) as slightly yellow liquid (2.17 g, 97%).

^{1}H NMR (500 MHz, $CDCl_3$) δ: 7.91 (d, 1H, J = 7.5 Hz), 7.69 (dd, 1H, J = 7.2 Hz, 8.1 Hz), 7.61-7.67 (m, 2H), 7.57 (d, 2H, J = 6.3 Hz), 7.35-7.43 (m, 3H), 5.33 (d, 1H, J = 9.7 Hz), 5.16 (d, 1H, J = 9.7 Hz), 3.31 (dt, 1H, J = 9.2 Hz, 6.3 Hz), 2.98 (dt, 1H, J = 9.2 Hz, 6.3 Hz), 1.62-1.72 (m, 1H), 1.35-1.50 (m, 2H), 0.81 (d, 3H, J = 6.6 Hz), 0.79 (d, 3H, J = 6.6 Hz); ^{13}C NMR (126 MHz, $CDCl_3$) δ: 165.6, 135.3, 134.6, 133.4, 131.6, 130.0, 129.3, 128.8, 128.4, 124.1, 122.2 (q, J = 286.7 Hz), 90.9 (q, J = 32.4 Hz), 79.1, 62.5, 37.7, 24.7, 22.4, 22.2; ^{19}F NMR (369 MHz, $CDCl_3$) δ: –78.1; IR (neat, cm^{-1}) ν: 2957, 1745, 1643, 1468, 1190; LRMS (ESI): *m/z* 416 $[M+Na]^+$; HRMS (ESI): *m/z* calcd for $C_{21}H_{22}F_3NO_3Na$ $[M+Na]^+$ 416.1444, Found 416.1456.

***N*-hydroxy-3-isopentyloxy-3-trifluoromethyl-isoindolinone (1h)**

A stirred mixture of **S4h** (2.17 g, 5.39 mmol, 1 eq) and Pd/C (10 wt%, 170 mg, 0.16 mmol, 0.03 eq) in ethanol (11 mL, 0.5 M) was exposed to H_2 (balloon pressure) at ambient temperature. The reaction mixture was stirred for two hours and then H_2 was removed. Filtration over Celite pad and evaporation of the filtrate afforded the crude oily liquid. Purification by silica gel column chromatography (hexane/EtOAc = 4/1) afforded **1h** as slightly orange liquid. The liquid was gradually solidified into white solid (1.56 g, 95%).

^{1}H NMR (500 MHz, $CDCl_3$) δ: 9.93 (brs, 1H), 7.79 (d, 1H, J = 6.9 Hz), 7.65-7.69 (m, 1H), 7.58-7.62 (m, 2H), 3.47 (dt, 1H, J = 9,2 Hz, 6.9 Hz), 3.00 (dt, 1H, J = 9,2 Hz, 6.9 Hz), 1.69-1.78 (m, 1H), 1.45-1.59 (m, 2H), 0.88 (d, 3H, J = 6.9 Hz) , 0.83 (d, 3H, J = 6.9 Hz); ^{13}C NMR (126 MHz, $CDCl_3$) δ: 165.9, 135.3, 133.2, 131.5, 130.0, 124.0, 123.9, 121.9 (q, J = 286.7 Hz), 91.0 (q, J = 33.6 Hz), 62.8, 37.8, 24.8, 22.6,

22.3; ^{19}F NMR (369 MHz, $CDCl_3$) δ: –78.5; IR (KBr, cm^{-1}) ν: 3137, 2959, 1718, 1507, 1472, 1194; LRMS (ESI): *m/z* 326 $[M+Na]^+$; HRMS (ESI): *m/z* calcd for $C_{14}H_{16}F_3NO_3Na$ $[M+Na]^+$ 326.0975, Found 326.0966.

***N*-hydroxy-3-(3-hydroxy-3-methylbutoxy)-3-trifluoromethyl-isoindolinone (2h)**

To a round-bottom flask were added **1h** (1.00 g, 3.30 mmol, 1 eq) and TFE (33 mL, 0.1 M). Me_2S (289 μL, 3.96 mmol, 1.2 eq) was added after replacing the air inside the tube with O_2 (1 atm, balloon) and finally $Co(OAc)_2$ (0.5 M solution in DMSO, 59.4 μL, 0.0297 mmol, 0.9 mol%) was added. The mixture was stirred at 40 °C and after **1h** was consumed, which was checked on TLC, TFE was removed by evaporation and then EtOAc was added. The EtOAc solution was passed through silica gel to remove the metal and DMSO, and EtOAc was removed from the filtrate by evaporation. The residue was dried under reduced pressure to afford the crude mixture. Purification by silica gel column chromatography (EtOAc/hexane = 1/1) afforded **2h** as a colorless sticky liquid (840 mg, 80%).

Removal of directing activator

$LiAlH_4$ conditions

To a round-bottom flask (dried by heat gun under reduced pressure) were added **2h** (840 mg, 2.3 mmol, 1 eq) and dry THF (13 mL, 0.2 M), and the mixture was cooled at –78 °C. $LiAlH_4$ (200 mg, 2 eq) was added to the reaction mixture in three portions (65 mg; 70 mg; 65 mg), and 15 minutes later the reaction temperature was raised to 0 °C. Two hours later water (200 μL), 4 M NaOH aq. (200 μL), and water (600 μL) were added successively to the reaction mixture, with vigorous stirring. Insoluble materials were removed by filtration over Celite pad and the filtrate was dried under reduced pressure to obtain the crude mixture. Purification by silica gel column chromatography (hexane/EtOAc = 1/2) and Kugelrohr distillation afforded 3-methyl-1,3-butanediol as colorless liquid (221 mg, 81%).

^{1}H NMR (500 MHz, $CDCl_3$) δ: 3.90 (t, 2H, J = 5.7 Hz), 2.22 (bs, 2H), 1.75 (t, 2H, J = 5.7 Hz), 1.30 (s, 6H); LRMS (ESI): *m/z* 127 $[M+Na]^+$: identical to a commercial material.

SmI_2 conditions[2]

To a flask containing **2i** (18.0 mg, 0.05 mmol, 1 eq) were added SmI_2 in THF (freshly prepared just before use as reported,[3] 4 mL, ca. 0.4 mmol, 8 eq), Et_3N (500 μL, 3.6 mmol, 72 eq), and water (65 μL, 36 mmol, 72 eq) at room temperature under argon atmosphere. After three hours, excess SmI_2 was oxidized by bubbling air through the reaction mixture. Filtration over Celite pad and evaporation of the filtrate afforded the crude mixture (69.4 mg, 84% NMR yield).

^{1}H NMR (500 MHz, $CDCl_3$) δ: 3.82 (t, 2H, J = 5.7 Hz), 3.71 (s,12H), 3.23 (s, 1H), 1.68 (t, 2H, J = 5.7 Hz), 1.52-1.62 (m, 4H), 1.35-1.50 (m, 5H), 1.23-1.32 (m, 1H); LRMS (ESI): m/z 167 $[M+Na]^+$: identical to a

---

2 M. Szostak, M. Spain, A. J. Eberhart, D. J. Procter *J. Am. Chem. Soc.* **2014**, *136*, 2268-2271.
3 M. Szostak, M. Spain, D. J. Procter *J. Org. Chem.* **2012**, *77*, 3049-3059.

reported data[4].

Removal of directing activator from **2aa** and **2ae** is also successfully demonstrated.

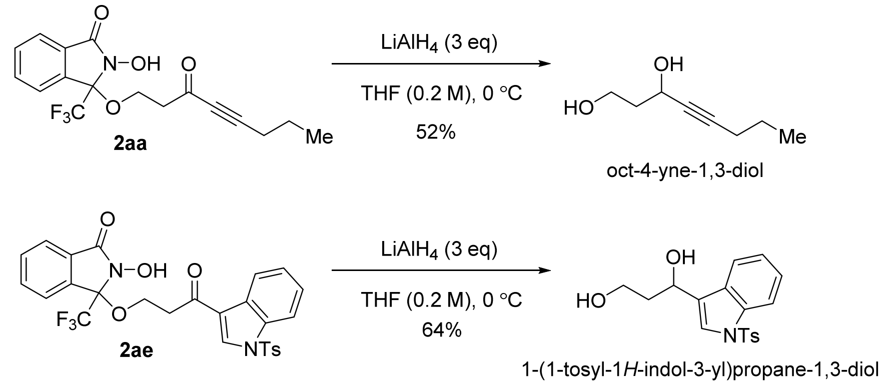

oct-4-yne-1,3-diol: ^{1}H NMR (500 MHz, $CDCl_3$) δ: 4.64 (s, 1H), 3.94–4.02 (m, 1H), 3.81–3.89 (m, 1H), 2.51 (s, 1H), 2.19 (td, 2H, J = 7.2 Hz, 1.9 Hz), 2.17 (s, 1H), 1.88–2.02 (m, 2H), 1.49–1.57 (m, 2H), 0.98 (t, 3H, J = 7.4 Hz); ^{13}C NMR (126 MHz, acetone-d_6) δ: 84.2, 83.3, 60.3, 59.5, 42.0, 22.9, 21.0, 13.6; IR (neat, cm^{-1}) ν: 3347, 2961, 1717, 1430, 1050; LRMS (ESI): *m/z* 165 $[M+Na]^+$; HRMS (ESI): *m/z* calcd for $C_8H_{14}O_2Na$ $[M+Na]^+$ 165.0886, Found 165.0886.

1-(1-tosyl-1*H*-indol-3-yl)propane-1,3-diol: ^{1}H NMR (500 MHz, $CDCl_3$) δ: 7.98 (d, 1H, J = 8.6 Hz), 7.77 (d, 2H, J = 8.0 Hz), 7.60 (d, 1H, J = 8.0 Hz), 7.55 (s, 1H), 7.30–7.34 (m, 1H), 7.20–7.25 (m, 3H), 5.20–5.25 (m, 1H), 3.85–3.95 (m, 2H), 2.99 (s, 1H), 2.34 (s, 3H), 2.27 (s, 1H), 2.06–2.20 (m, 2H); ^{13}C NMR (126 MHz, $CDCl_3$) δ: 145.0, 135.5, 135.2, 129.9, 128.6, 126.8, 125.6, 124.9, 123.2, 122.7, 120.2, 113.8, 68.0, 61.4, 38.4, 21.6; IR (neat, cm^{-1}) ν: 3375, 2925, 1597, 1447, 1367, 1276, 1173; LRMS (ESI): *m/z* 368 $[M+Na]^+$; HRMS (ESI): *m/z* calcd for $C_{18}H_{19}NO_4SNa$ $[M+Na]^+$ 368.0927, Found 368.0908.

[4] T. He, W.-C. Gao, W.-K. Wang, C. Zhang *Adv. Synth. Catal.* **2014**, *356*, 1113-1118.

5. Experimental details

5.1. Optimization of the oxygenation conditions

5.1.1. Investigation of Directing Activator (DA)

We synthesized several DA (**A-G**) bound-alcohols and compared their reactivity under condition A [$Co(OAc)_2$ (5 mol%), $Mn(OAc)_3$•$2H_2O$ (5 mol%) in 2,2,2-trifluoroethanol (TFE, 0.1 M) under O_2 (1 atm) at 40 °C] (Table S1).

Table S1

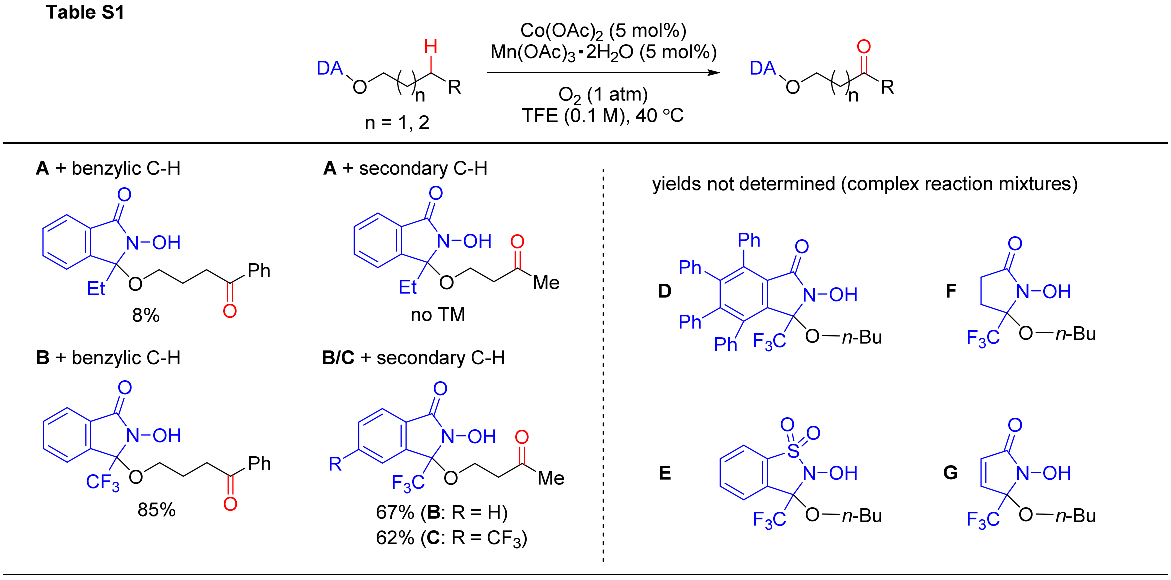

With **A**, benzylic C–H oxygenation produced the corresponding phenyl ketone product in only 8% yield. A more challenging methylene C(sp^3)–H oxygenation did not proceed at all. In both cases, the efficiency of the C–H oxygenation was much lower than that of *N*-hydroxyphthalimide itself. Because the reactivity of *N*-oxyl radicals is in accord with their electron-deficiency, we next designed **B** bearing a CF_3 group at the α-carbon of the nitrogen atom to enhance the reactivity of the DA. As a result, **B** promoted simple methylene C(sp^3)–H oxygenation to produce γ-oxo product in 67% yield. Although the α-C(sp^3)–H bond adjacent to the ether oxygen atom is the innate reactive site, the less reactive γ-C(sp^3)–H was predominantly oxygenated. Under the same conditions, benzylic C(sp^3)–H oxygenation also proceeded well (85% yield). Introducing an additional CF_3 substituent at the benzene ring (**C**), however, did not further improve the yield. Additional structural modifications (**D**, **E**, **F**, or **G**) were not successful, resulting in complex mixtures and/or decomposition of the DA during the reaction. Thus, **B** proved to be the best DA.

5.1.2. Secondary C(sp^3)-H oxygenation

Solvents, metal salts, and additives were screened for higher yields of secondary C(sp^3)-H oxygenation. Tested additives such as some acids, bases, molecular sieves, and reductants all brought no improvements.

The screening of the solvents for 1-pentanol oxygenation is shown below. As shown in Table S2, TFE (2,2,2-trifluoroethanol) and HFIP (1,1,1,3,3,3-hexafluoroisopropanol) accelerated the reaction to almost the same degree and produced almost the same yields, so the cheaper TFE was employed as the best solvent.

Table S2

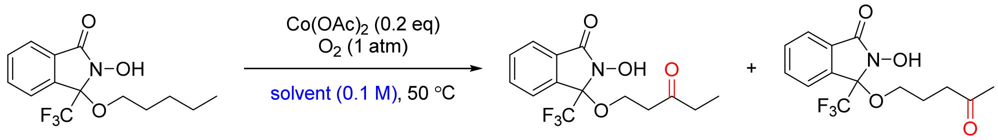

S1a **S2a**: γ-oxo **S2a'**: δ-oxo

solvent	**B2ba** [%]	**B2bb** [%]	rxn time [hr]
MeCN	7	4	19
MeOH	0	0	19
EtOH	4	2	19
TFE	12	12	2.5
HFIP	12	16	2.5
CF_3Ph	12.	5	19
EtOAc	4	1	19
MeO^tBu	3	3	19

(NMR yield)

Then, metal salts were screened using **B**-anchored 1-butanol instead of **B**-anchored 1-pentanol because **B**-anchored 1-butanol gave only one regioisomer and made the analysis easier.

Table S3

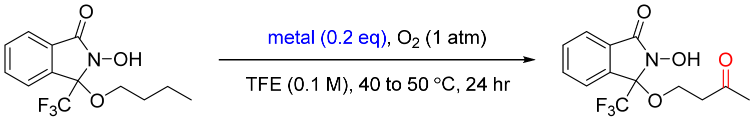

1a **2a**

metal	NMR yield	metal	NMR yield
$Co(OAc)_2$	40	$Cu(OTf)_2$	0
$Co(acac)_2$	0	$FeCl_2$	0
$Co(NO_3)_2 \cdot 6H_2O$	7	$FeCl_3$	0
$Mn(OAc)_2$	3	$Fe(OTf)_2$	0
$Mn(OAc)_3 \cdot 2H_2O$	36	$Fe(OTf)_3$	0
$MnBr_2$	0	$Fe(OAc)_2$	0
$Mn(acac)_2$	0	$Fe(OH)(OAc)_2$	0
$Mn(OTf)_2$	5	no metal	0
CuOAc	0	$Co(OAc)_2$ + $Mn(OAc)_2$ (1:1)	16
$Cu(OAc)_2$	0	$Co(OAc)_2$ + $Mn(OAc)_3 \cdot 2H_2O$ (1:1)	67
CuBr	0	$Co(acac)_2$ + $Mn(OAc)_3 \cdot 2H_2O$ (1:1)	50
$CuOTf \cdot 0.5PhH$	0	$Co(NO_3)_2 \cdot 6H_2O$ + $Mn(OAc)_3 \cdot 2H_2O$ (1:1)	41

As shown in Table S3, the combination of $Co(OAc)_2$ and $Mn(OAc)_3 \cdot 2H_2O$ produced the best result. The role of $Mn(OAc)_3 \cdot 2H_2O$ is not clear, but we suppose that the main role of $Mn(OAc)_3 \cdot 2H_2O$ is to trap reversibly reactive intermediates such as peroxy radicals and alkoxy radicals to suppress unfavorable side reactions, considering the fact that the reaction rate became lower by adding $Mn(OAc)_3 \cdot 2H_2O$ than when $Co(OAc)_2$ alone was used as a metal source even though $Mn(OAc)_3 \cdot 2H_2O$ itself was able to catalyze the reaction.

The 1-butanol oxygenation was conducted at 30, 40, or 50 °C. The reaction didn't complete at 30 °C, and became slightly complicated at 50 °C compared to that at 40 °C. Therefore, 40 °C was revealed to be the best reaction temperature.

Then, the optimal conditions for secondary C(sp^3)-H oxygenation were these:
$Co(OAc)_2$ (5 mol%), $Mn(OAc)_3 \cdot 2H_2O$ (5 mol%), O_2 (1 atm), TFE (0.1 M), 40 °C.

5.1.3. Tertiary C(sp^3)-H oxygenation

Although 67% NMR yield was obtained for 1-butanol oxygenation under condition A, the reaction became complicated and only 20% NMR yield was obtained for 3-methyl-1-butanol oxygenation (**1h**) under the same conditions. It was probably because condition A was too harsh for tertiary C(sp^3)-H oxygenation, so the reaction conditions had to be refined for higher yields. The goal was accomplished by reducing the amount of the metal salts. Lower reaction temperatures and different solvents were not effective. The best reaction conditions were these:

$Co(OAc)_2$ (1 mol%), Me_2S (1.2 eq), O_2 (1 atm), TFE (0.1 M), 40 °C.

5.2. Absolute configuration of DA-anchored (+)-menthol (1k)

X-ray analysis of less polar diastereomer of *O*-(4-nitrobenzyl)-**1k** was conducted to determine the absolute configuration of DA-anchored (+)-menthol. The configuration of the hemiaminal center was revealed to be (*R*), which accords with that of reactive isomer in aerobic C-H oxygenation.

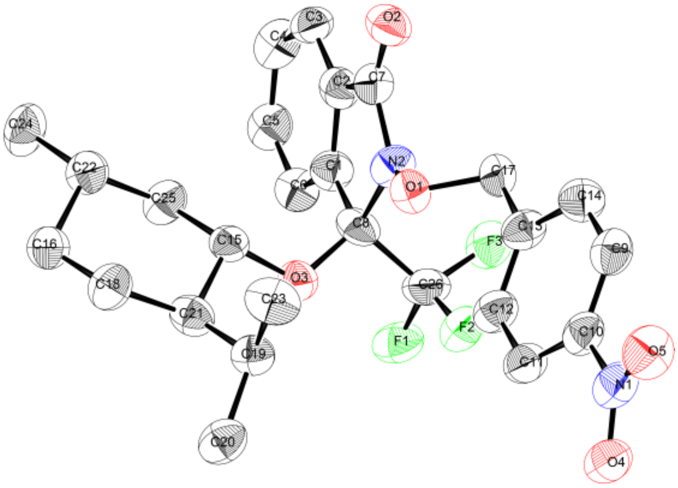

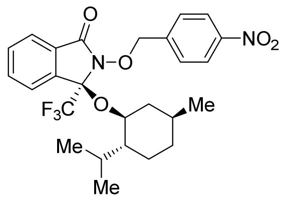

(*R*)-S4k'

Crystallization procedure: less polar diastereomer of *O*-(4-nitrobenzyl)-**1k** was dissolved in MeCN and the solvent was gradually evaporated under air at room temperature. After a few days a needle crystal was obtained.

The supposed reason for the difference in the reactivity is depicted in the figure below.

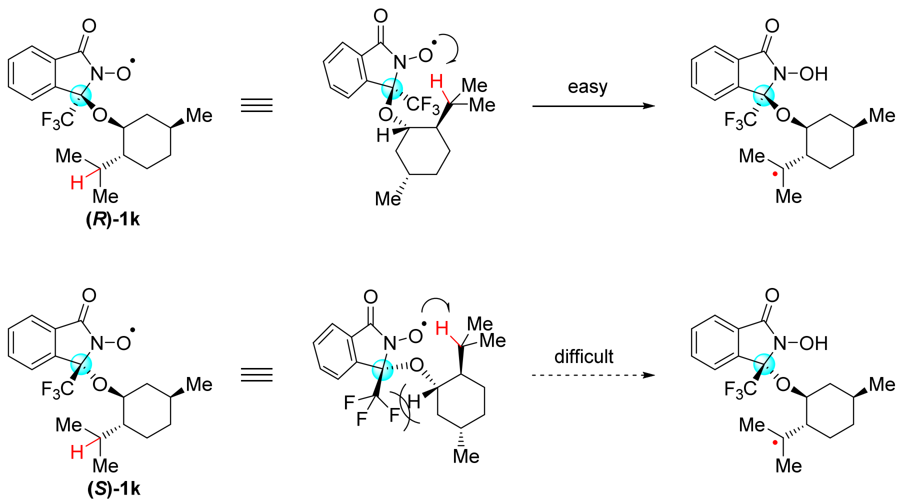

According to molecular modeling, there is no steric repulsion during the abstraction of the red hydrogen in (*R*)-**1k** and it proceeds smoothly; whereas in the case of (*S*)-**1k** there is a steric repulsion between CF_3 and the axial α-hydrogen of the oxygen atom in menthol that makes it difficult for the *N*-oxyl radical to access the red hydrogen.

5.3. Comparison of reaction speed in benzylic C–H oxygenation

The chemical yield after 30 min was compared for β-, γ-, and δ-C–H oxygenation. Reaction speed of β-C-H oxygenation is significantly slower than γ- and δ-C-H oxygenation. The difference between γ- and δ-C-H oxygenation is, however, not large. This low γ/δ selectivity is a current limitation of our method.

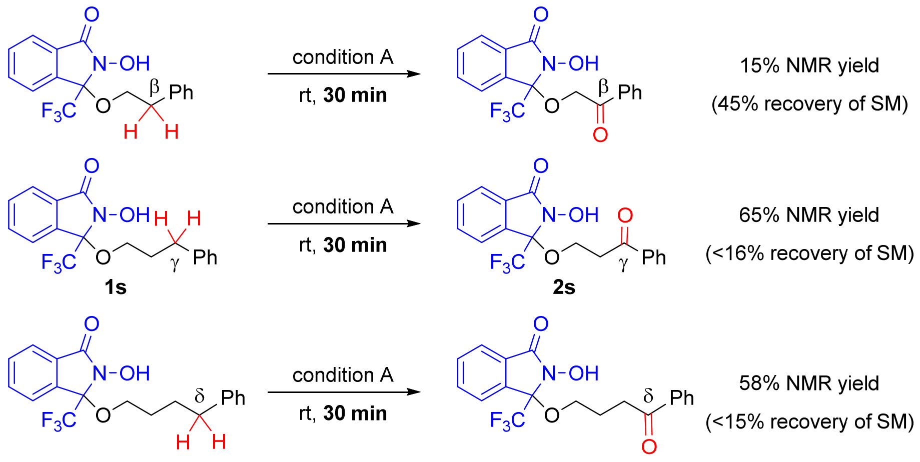

condition A: $Co(OAc)_2$ (5 mol%), $Mn(OAc)_3{\cdot}2H_2O$ (5 mol%), O_2 (1 atm), TFE (0.1 M)

6. Selective ultra-remote C-H oxygenation

6.1. Synthesis of substrate 3

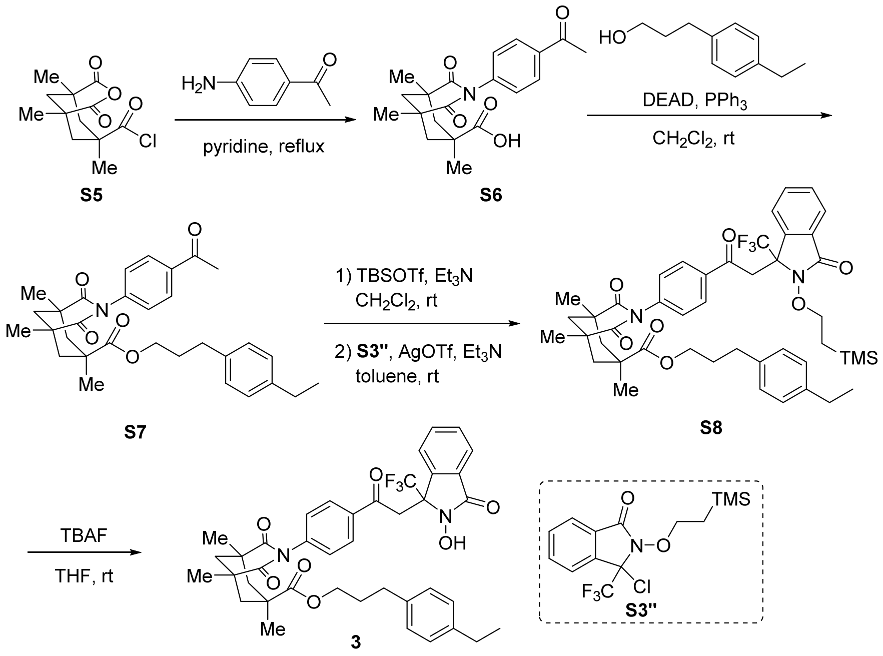

Synthetic procedure from S5 to S6

To a round bottom flask were added **S5**[5] (2.15 g, 8.3 mmol), *p*-acetylaniline (1.07 g, 7.9 mmol) and pyridine (42 mL, 0.2 M). The mixture was stirred at reflux for 18 h. Pyridine was removed by evaporation. The obtained crude mixture was purified by silica gel column chromatography (EtOAc/hexane = 2/1) to afford **S6** with inseparable byproducts as a colorless sticky liquid (2.98 g). This mixture was used for next step.

Synthetic procedure from S6 to S7

To a round bottom flask were added acid impure **S6** (566 mg, ca. 1.6 mmol), 3-(4-ethylphenyl)propan-1-ol (286 mg, 1.74 mmol), diethyl azodicarboxylate (2.5 M solution in toluene, 0.759 mL, 1.90 mmol), PPh_3 (456 mg, 1.90 mmol) and CH_2Cl_2 (8.7 mL, 0.2 M). The mixture was stirred at room temperature for 2.5 h, followed by all the volatiles were removed by evaporation. The obtained crude mixture was purified by silica gel column chromatography (EtOAc/hexane = 1/2) to afford **S7** as a colorless sticky liquid (532 mg, 69%, 2 steps).

^{1}H NMR (392 MHz, CD_3OD) δ: 7.99 (d, 2H, J = 8.1 Hz), 7.27 (d, 2H, J = 8.1 Hz), 7.01 (d, 2H, J = 7.6 Hz), 6.96 (d, 2H, J = 7.6 Hz), 3.99 (t, 2H, J = 6.1 Hz), 2.71 (d, 2H, J = 13.9 Hz), 2.60 (s, 3H), 2.51-2.60 (m, 2H+2H), 2.24 (d, 1H, J = 13.5 Hz), 1.70-1.80 (m, 2H), 1.58 (d, 1H, J = 13.5 Hz), 1.42 (d, 2H, J = 13.9 Hz), 1.30 (s, 3H), 1.27 (s, 3H), 1.18 (t, 3H, J = 7.6 Hz); ^{13}C NMR (126 MHz, $CDCl_3$) δ: 197.6, 176.1, 176.1,

[5] (a) Q. Ye, I. V. Komarov, A. J. Kirby, M. Jones, Jr. *J. Org. Chem.* **2002**, *67*, 9288-9294. (b) T. J. Dale, J. Rebek, Jr. *J. Am. Chem. Soc.* **2006**, *128*, 4500-4501.

142.0, 140.2, 138.4, 136.3, 128.9, 128.8, 128.4, 128.0, 64.9, 44.3, 44.0, 31.7, 31.1, 30.1, 28.5, 26.8, 26.1, 15.8; IR (neat, cm^{-1}) ν: 2964, 1721, 1689, 1602, 1460, 1357, 1321, 1265, 1174; LRMS (ESI): *m/z* 526 $[M+Na]^+$; HRMS (ESI): *m/z* calcd for $C_{31}H_{37}NO_5Na$ $[M+Na]^+$ 526.2564, Found 526.2541.

Synthetic procedure from S7 to S8

To solution of ketone **S7** (288 mg, 0.588 mmol) in CH_2Cl_2 (2.94 mL, 0.2 M) were added triethylamine (0.590 mL, 4.23 mmol) and TBSOTf (0.494 mL, 2.82 mmol). The mixture was stirred at room temperature for 40 min. After ketone was consumed, which was checked on TLC, pentane and H_2O were added. The organic layer was separated and washed with 10% aqueous $CuSO_4$ solution and 5%$NaHCO_3$ aq. solution, and then dried over Na_2SO_4. Filtration followed by evaporating the solvent at room temperature afforded the crude mixture containing the corresponding enol silyl ether.

To a round bottom flask were added the all amount of the crude mixture, **S3”** (0.394 mL, 1.18 mmol), Et_3N (0.246 mL, 1.76 mmol) and toluene (5.88 mL, 0.1 M), followed by AgOTf (376 mg, 1.47 mmol). The mixture was stirred at room temperature for 18 h, and saturated NaCl aq. solution was added. After filtration over Celite pad to remove the silver salts, the organic layer was separated and washed with water and saturated NaCl aq. solution, and dried over Na_2SO_4. After filtration, all the volatiles were removed by evaporation. The obtained crude mixture was purified by silica gel column chromatography (EtOAc/hexane = 2/5) to afford **S8** as colorless sticky liquid (421 mg, 87% yield).

^{1}H NMR (500 MHz, CD_3OD) δ: 7.85-7.90 (m, 1H), 7.84 (d, 2H, *J* = 8.6 Hz), 7.60-7.66 (m, 3H), 7.22 (d, 2H, *J* = 8.6 Hz), 7.00 (d, 2H, *J* = 8.0 Hz), 6.96 (d, 2H, *J* = 8.0 Hz), 4.60 (brs, 1H), 4.37-4.43 (m, 1H), 4.09-4.20 (m, 3H), 3.91-3.97 (m, 2H), 2.69 (d, 2H, *J* = 13.7 Hz), 2.54 (q, 2H, *J* = 7.4 Hz), 2.49 (t, 2H, *J* = 7.4 Hz), 2.21 (d, 1H, *J* = 13.2 Hz), 1.67-1.73 (m, 2H), 1.56 (d, 1H, *J* = 13.2 Hz), 1.39 (d, 2H, *J* = 13.7 Hz), 1.28 (s, 6H), 1.25 (s, 3H), 1.16 (t, 3H, *J* = 7.4 Hz), 0.90-1.10 (m, 2H), –0.01 (s, 9H); ^{13}C NMR (126 MHz, CD_3OD) δ:194.4, 178.3, 178.3, 178.0, 170.9, 143.1, 142.4, 140.00, 139.8, 137.2, 134.7, 131.9, 131.5, 130.4, 129.6, 129.3, 129.0, 126.1 (q, *J* = 283.1 Hz), 124.9, 124.3, 76.4, 69.0 (q, *J* = 30.1 Hz), 66.1, 44.9, 43.9, 43.6, 42.05, 42.03, 32.8, 31.40, 31.36, 29.6, 26.3 17.9, 16.5, –1.25; ^{19}F NMR (369 MHz, $CDCl_3$) δ: –76.1; IR (neat, cm^{-1}) ν: 2963, 1733, 1693, 1602, 1463, 1356, 1321, 1273, 1178, 1092; LRMS (ESI): *m/z* 841 $[M+Na]^+$; HRMS (ESI): *m/z* calcd for $C_{43}H_{53}F_3N_2O_7SiNa$ $[M+Na]^+$ 841.3466, Found 841.3436.

Synthetic procedure from S8 to 3

To a solution of **S8** (421 mg, 0.514 mmol) in THF (0.590 mL, 4.23 mmol) was added tetrabutylammonium fluoride (1.0 M solution in THF, 1.54 mL, 1.54 mmol). The mixture was stirred at room temperature. After **S8** was consumed, which was checked on TLC, water was added. The organic layer was separated and the water layer was extracted by EtOAc two times. The combined organic layer was dried over Na_2SO_4. After filtration, all the volatiles were removed by evaporation. The obtained crude mixture was purified by silica gel column chromatography (EtOAc/hexane = 2/3) to afford **3** as colorless sticky liquid (287 mg, 78% yield)

^{1}H NMR (500 MHz, CD_3OD) δ: 7.85-7.90 (m, 3H), 7.57-7.65 (m, 3H), 7.22 (d, 2H, *J* = 8.0 Hz), 7.01 (d,

2H, J = 8.0 Hz), 6.96 (d, 2H, J = 8.0 Hz), 4.17-4.22 (m, 2H), 3.96 (t, 2H, J = 6.3 Hz), 2.69 (d, 2H, J = 13.7 Hz), 2.55 (q, 2H, J = 7.7 Hz), 2.21 (d, 1H, J = 13.2 Hz), 1.68-1.75 (m, 2H), 1.56 (d, 1H, J = 13.2 Hz), 1.40 (d, 2H, J = 13.7 Hz), 1.28 (s, 6H), 1.16 (t, 3H, J = 7.7 Hz); ^{13}C NMR (126 MHz, $CDCl_3$) δ:193.4, 176.30, 176.27, 168.1, 147.25, 142.21, 141.1, 138.6, 137.7, 135.8, 133.1, 130.7, 129.3, 129.1, 128.9, 128.6, 128.2, 128.0, 125.7, 124.5 (q, J = 289.5 Hz), 123.4, 121.1, 68.2 (q, J = 27.2 Hz), 65.1, 44.6, 44.5, 44.1, 42.5, 41.2, 34.5, 32.0, 31.2, 30.3, 30.1, 28.7, 26.3, 16.0; ^{19}F NMR (369 MHz, $CDCl_3$) δ: –75.8; IR (neat, cm^{-1}) ν: 3420, 2930, 1725, 1692, 1600, 1321, 1273, 1176, 1091; LRMS (ESI): *m/z* 741 [M+Na]$^+$; HRMS (ESI): *m/z* calcd for $C_{40}H_{41}F_3N_2O_7Na$ [M+Na]$^+$ 741.2758, Found 741.2794.

6.2. Oxygenation of 3 (ultra-remote position-selective aerobic C-H oxygenation)

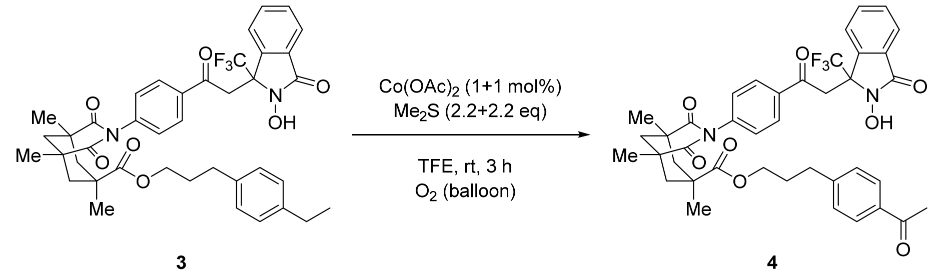

3 **4**

To a test tube were added **3** (30.0 mg, 41.7 μmol) and TFE (0.42 mL, 0.1 M). Me_2S (6.71 μL, 91.8 μmol) was added after replacing the air inside the tube with O_2 (1 atm, balloon), and finally $Co(OAc)_2$ (0.2 M solution in DMSO, 2.1 μL, 0.42 μmol) was added. The mixture was stirred at room temperature. After 1.5 h, $Co(OAc)_2$ (0.2 M solution in DMSO, 2.1 μL, 0.42 μmol) and Me_2S (6.71 μL, 91.8 μmol) were added. After **3** was consumed, which was checked on TLC, TFE was removed by evaporation and water was added. The aqueous layer was extracted by EtOAc three times. The combined organic layer was dried over Na_2SO_4. After filtration, all the volatiles were removed by evaporation. The yield was determined as 66% by ^{1}H NMR of the crude mixture with 1,1,2,2-tetrachloroethane as the internal standard. Purification by neutral silica gel column chromatography (EtOAc/hexane = 2/3) afforded **4** as a white solid (16.3 mg, 53%).

^{1}H NMR (500 MHz, CD_3OD) δ: 7.80-7.88 (m, 3H), 7.78 (d, 2H, J = 8.0 Hz), 7.55-7.65 (m, 3H), 7.23 (d, 2H, J = 8.0 Hz), 7.18 (d, 2H, J = 8.0 Hz), 4.10-4.16 (m, 2H), 3.92-4.00 (m, 2H), 2.68 (d, 1H, J = 14.3 Hz), 2.61 (t, 2H, J = 7.4 Hz), 2.51 (s, 3H), 2.15-2.23 (m, 1H), 1.70-1.79 (m, 2H), 1.57 (d, 1H, J = 13.2 Hz), 1.40 (d, 1H, J = 14.3 Hz), 1.28 (s, 6H), 1.25 (s, 3H); ^{13}C NMR (126 MHz, CD_3OD) δ: 200.3, 178.0, 177.6, 169.2, 148.7, 142.0, 137.1, 136.0, 133.7, 132.2, 131.0, 130.0, 129.7, 129.5, 129.0, 125.7 (d, J = 278.3 Hz), 124.3, 123.9, 65.6, 44.5, 43.5, 43.2, 41.7, 34.9, 32.8, 31.0, 30.6, 30.5, 29.3, 26.5, 25.9; ^{19}F NMR (369 MHz, $CDCl_3$) δ: –75.5; IR (neat, cm^{-1}) ν: 3432, 2929, 1690, 1605, 1463, 1358, 1321, 1273, 1178, 1093; LRMS (ESI): *m/z* 755 [M+Na]$^+$; HRMS (ESI): *m/z* calcd for $C_{40}H_{39}F_3N_2O_8Na$ [M+Na]$^+$ 755.2551, Found 755.2537.

6.3. Removal of directing activator

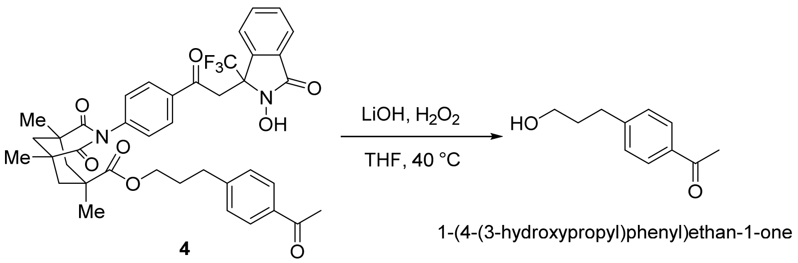

To a test tube were added **4** (2.8 mg, 3.8 µmol) and THF/H_2O (3/1, 80 µL, 0.05 M). To the mixture were added LiOH (1 M solution in H_2O, 20 µL, 20 µmol) and H_2O_2 (10 M in H_2O, 3.2 µL, 32 µmol). After stirring for 21 h, LiOH (1 M solution in H_2O, 20 µL, 20 µmol) and H_2O_2 (10 M in H_2O, 3.2 µL, 32 µmol) were further added. The mixture was stirred at 40 °C for 24 h. After **4** was consumed, which was checked on TLC, water was added. The aqueous layer was extracted by EtOAc three times. The combined organic layer was dried over Na_2SO_4. After filtration, all the volatiles were removed by evaporation to afford the crude mixture. The yield was determined to be 79% by ^1H NMR of the obtained crude mixture with 1,1,2,2-tetrachloroethane as the internal standard.

^1H NMR (500 MHz, $CDCl_3$) δ: 7.89 (d, 2H, J = 8.0 Hz), 7.30 (d, 2H, J = 8.0 Hz), 3.68 (t, 2H, J = 6.3 Hz), 2.78 (t, 2H, J = 2.78 Hz), 2.59 (s, 3H), 1.85-1.95 (m, 2H), LRMS (ESI): m/z 201 $[M+Na]^+$; identical to a reported data.[6]

6.4. Structural anaylsis of oxygenated products

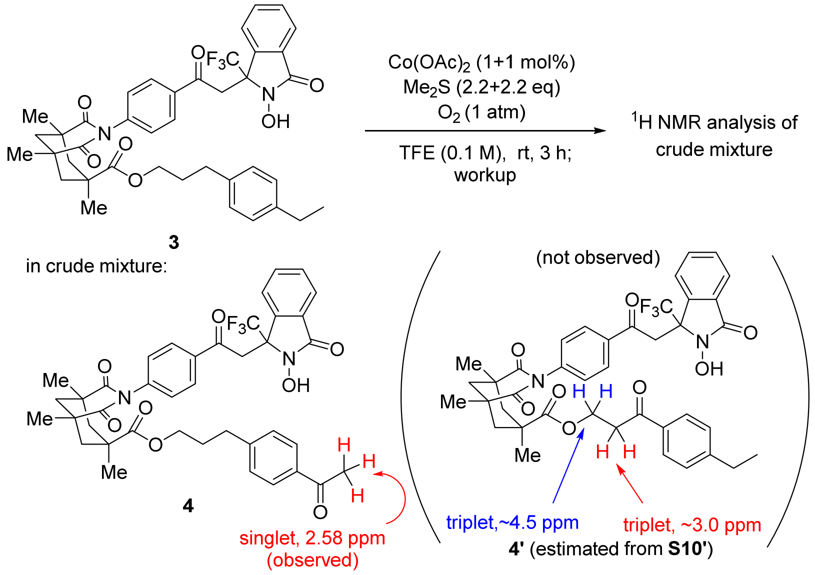

After aerobic oxygenation of **3**, ^1H NMR chart of the crude mixture was carefully analyzed. No triplet peak was observed around 3.00-3.50 ppm, which corresponds to the α-methylene protons of phenyl ketone **4'** (marked in red in **4'** of the above scheme), while a singlet peak at 2.58 ppm was observed, which corresponds to the α-methyl protons of phenyl ketone of **4** (marked in red in **4**). For comparison, oxidized

[6] Z.-L. Shen, K. K. K. Goh, Y.-S. Yang, Y.-C. Lai, C. H. A. Wong, H.-L. Cheong, T.-P. Loh, *Angew. Chem. Int. Ed.* **2011**, *50*, 511.

products of model substrate **S11** afforded α-methylene protons of phenyl ketone **S12'** observed at 3.35 ppm as triplet (marked in red in **S12'** of the scheme below), and α-methyl protons of ketone **S12** observed at 2.58 ppm as singlet (marked in red in **S12**). This fact indicates that regioisomer **4'** did not generate at all. Thus, we concluded that the oxygenation of **3** occurred exclusively at the remote benzylic position.

The intermolecular oxygenation of model compound **S11** produced a 2 : 1 regiomixture of **S12** and **S12'** at 60 °C. This result, combined with the contrasting and exclusive remote-regioselectivity in oxygenation of **3**, further supported the notion that oxygenation of **3** proceeded in an intramolecular manner controlled by the "long-arm linker" directing activator.

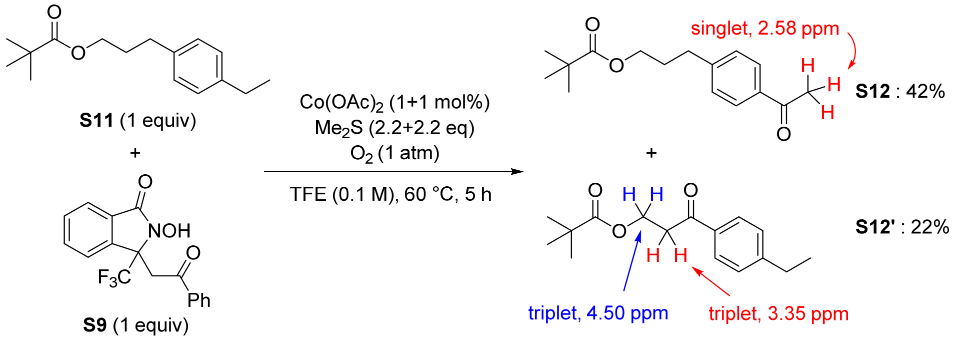

7. Analytical data

Full spectroscopic data were described for **S4**, **1**, and **2** related to Table 1.

***N*-benzyloxy-3- ((2-methylpentan-2-yl)oxy)-3-trifluoromethyl-isoindolinone (S4b)**

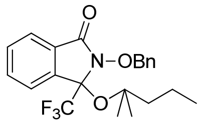

colorless liquid; ^{1}H NMR (500 MHz, $CDCl_3$) δ: 7.88 (dd, 1H, J = 6.0 Hz, 2.0 Hz), 7.61-7.73 (m, 3H), 7.56 (d, 2H, J = 6.9 Hz), 5.45 (d, 1H, J = 9.2 Hz), 5.06 (d, 1H, J = 9.2 Hz), 1.32-1.50 (m, 4H), 1.03 (s, 3H), 0.93 (s, 3H), 0.79-0.84 (m, 3H); ^{13}C NMR (126 MHz, $CDCl_3$) δ: 165.1, 137.3, 134.8, 132.6, 131.4, 130.3, 129.2, 128.6, 128.4, 125.4, 123.9, 122.7 (q, J = 286.0 Hz), 89.1 (q, J = 32.3 Hz), 82.3, 78.5, 46.1, 27.3, 26.0, 16.9, 14.3; ^{19}F NMR (369 MHz, $CDCl_3$) δ: –79.7; IR (neat, cm^{-1}) ν: 2961, 1739, 1646, 1190; LRMS (ESI): *m/z* 430 [M+Na]$^+$; HRMS (ESI): *m/z* calcd for $C_{22}H_{24}F_3NO_3Na$ [M+Na]$^+$ 430.1601, Found 430.1620.

***N*-hydroxy-3-((2-methylpentan-2-yl)oxy)-3-trifluoromethyl-isoindolinone (1b)**

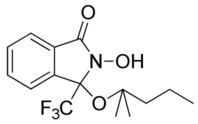

reddish solid; ^{1}H NMR (500 MHz, $CDCl_3$) δ: 9.95 (brs, 1H), 7.78 (d, 1H, J = 7.3 Hz), 7.66 (d, 1H, J = 7.3 Hz), 7.61 (dd, 1H, J = 7.3 Hz, 7.3 Hz), 7.57 (dd, 1H, J = 7.3 Hz, 7.3 Hz), 1.40-1.55 (m, 4H), 1.15 (s, 3H), 0.96 (s, 3H), 0.90 (t, 3H, J = 6.6 Hz); ^{13}C NMR (126 MHz, $CDCl_3$) δ: 165.4, 137.6, 132.5, 131.2, 129.8, 125.2, 123.7, 122.1 (q, J = 288.2 Hz), 89.9 (q, J = 32.3 Hz), 82.6, 46.2, 27.2, 26.2, 17.1, 14.4; ^{19}F NMR (369 MHz, $CDCl_3$) δ: –79.5, –79.7 (rotamers); IR (KBr, cm^{-1}) ν: 3159, 2962, 1718, 1473, 1391, 1373, 1192; LRMS (ESI): *m/z* 340 [M+Na]$^+$; HRMS (ESI): *m/z* calcd for $C_{15}H_{18}F_3NO_3Na$ [M+Na]$^+$ 340.1131, Found 340.1117.

the mixture of
***N*-hydroxy-3- ((2-methyl-4-oxopentan-2-yl)oxy)-3-trifluoromethyl-isoindolinone (2b)**
and
4-hydroxy-2,2,4-trimethyl-11b-(trifluoromethyl)-3,4-dihydro-2*H*-[1,4,2]dioxazepino[3,2-*a*]isoindol-7(11b*H*)-one (2b'; diastereomixture)

Starting material **1b** (31.7 mg, 0.1 mmol) was oxygenated under condition A of section 2, and **2b'** (20.7 mg, 0.062 mmol) was isolated as described in the General method (62% yield).

2b easily cyclizes to form **2b'** and the equilibrium position lies far to the right.

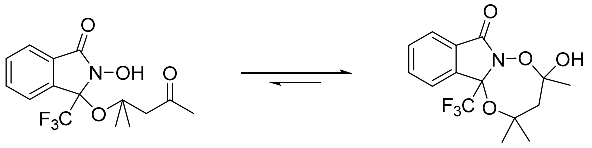

2b **2b'**

No peaks of **2b** were detected in NMR spectra when the solvent was $CDCl_3$; the peaks of **2b** could be slightly detected when the solvent was acetone-d_6 as shown below ($CDCl_3$ should be avoided to take a ^{13}C NMR spectrum because **2b'** doesn't dissolve in $CDCl_3$ very much).

white solid; ^1H NMR (500 MHz, acetone-d_6; underlined chemical shifts for **2b**) δ: 9.47 (s, 1H), 7.68-7.86 (m, 4H + 4H), 5.68-5.71 (m, 1H), 2.81 (d, 1H, J = 14.9 Hz), 2.72 (d, 1H, J = 14.9 Hz), 2.23 (d, 1H, J = 14.9 Hz), 2.21 (s, 3H), 2.10 (d, 1H, J = 14.9 Hz), 1.60 (s, 3H), 1.31 (s, 3H), 1.18 (s, 3H), 1.12 (s, 3H), 1.11 (s, 3H); ^{13}C NMR (126 MHz, acetone-d_6; underlined chemical shifts for **2b**; wavylined shifts for **2b** or **2b'**; dashed-lined shifts for both **2b** and **2b'**; two shifts in brackets derived from "the same" carbon of the diastereomers) δ: 207.4, 164.0, 163.5, 139.8, 137.8, 134.1, 133.6, 132.6, 132.0, 131.5, 130.4, 126.6, 124.7, 124.2, 124.1, 123.6 (q, J = 288.5 Hz), [107.8, 107.7], 90.3 (q, J = 31.7 Hz), 56.1, 52.8, 33.9, 32.1, 28.2, [27.6, 27.5], 26.1, 25.6; ^{19}F NMR (369 MHz, $CDCl_3$; underlined chemical shifts for **2b**) δ: –79.1, –79.7; IR (KBr, cm^{-1}) ν: 3460, 2985, 1730, 1469, 1250, 1191; LRMS (ESI): *m/z* 354 $[M+Na]^+$; HRMS (ESI): *m/z* calcd for $C_{15}H_{16}F_3NO_4Na$ $[M+Na]^+$ 354.0924, Found 354.0924.

N-benzyloxy-3- (2,2-dimethylbutoxy)-3-trifluoromethyl-isoindolinone (S4c)

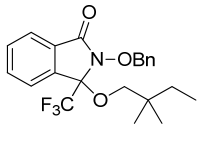

colorless liquid; ^1H NMR (500 MHz, $CDCl_3$) δ: 7.91 (d, 1H, J = 7.4 Hz), 7.70 (dd, 1H, J = 7.4 Hz, 7.4 Hz), 7.65 (dd, 1H, J = 7.4 Hz, 7.4 Hz), 7.60 (d, 1H, J = 7.4 Hz), 7.55 (d, 2H, J = 7.4 Hz), 7.35-7.43 (m, 3H), 5.34 (d, 1H, J = 9.2 Hz), 5.14 (d, 1H, J = 9.2 Hz), 3.05 (d, 1H, J = 8.3 Hz), 2.58 (d, 1H, J = 8.3 Hz), 1.29 (q, 2H, J = 7.5 Hz), 0.84 (s, 3H), 0.82 (s, 3H), 0.73 (t, 3H, J = 7.5 Hz); ^{13}C NMR (126 MHz, $CDCl_3$) δ: 165.7, 135.4, 134.6, 133.4, 131.6, 130.1, 129.2, 128.7, 128.4, 124.15, 124.13, 122.3 (q, J = 286.3 Hz), 90.8 (q, J = 32.3 Hz), 79.0, 71.7, 34.1, 31.1, 23.8, 23.7, 8.0; ^{19}F NMR (369 MHz, $CDCl_3$) δ: –78.3; IR (neat, cm^{-1}) ν: 2965, 1747, 1468, 1294, 1190; LRMS (ESI): *m/z* 430 $[M+Na]^+$; HRMS (ESI): *m/z* calcd for $C_{22}H_{24}F_3NO_3Na$ $[M+Na]^+$ 430.1601, Found 430.1614.

3-(2,2-dimethylbutoxy)-*N*-hydroxy-3-trifluoromethyl-isoindolinone (1c)

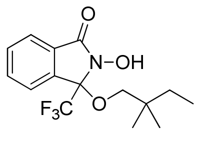

white solid; ^1H NMR (500 MHz, $CDCl_3$) δ: 9.89 (brs, 1H), 7.79 (d, 1H, J = 7.4 Hz), 7.66 (dd, 1H, J = 7.4 Hz, 7.4 Hz), 7.56-7.62 (m, 2H), 3.16 (d, 1H, J = 8.0 Hz), 2.61 (d, 1H, J = 8.0 Hz), 1.29-1.40 (m, 2H), 0.91 (s, 3H), 0.88 (s, 3H), 0.81 (t, 3H, J = 7.4 Hz); ^{13}C NMR (126 MHz, $CDCl_3$) δ: 165.8, 135.3, 133.2, 131.4, 130.0, 124.0, 123.8, 122.0 (q, J = 287.1 Hz), 90.9 (q, J = 32.8 Hz), 71.8, 34.1, 31.2, 23.8, 23.7, 8.1; ^{19}F NMR (369 MHz, $CDCl_3$) δ: –78.6; IR (KBr, cm^{-1}) ν: 3168, 2964, 2883, 1721, 1525, 1473, 1382, 1311, 1203; LRMS (ESI): *m/z* 340 $[M+Na]^+$; HRMS (ESI): *m/z* calcd for $C_{15}H_{18}F_3NO_3Na$ $[M+Na]^+$ 340.1131, Found 340.1117.

3-(2,2-dimethyl-3-oxobutoxy)-*N*-hydrooxy-3-trifluoromethyl-isoindolinone (2c)

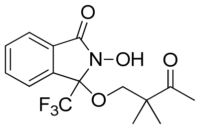

Starting material **1c** (31.7 mg, 0.1 mmol) was oxygenated under condition A of section 2, and **2c** (17.7 mg, 0.053 mmol) was isolated as described in the General method (53% yield).

white solid; ^{1}H NMR (400 MHz, $CDCl_3$) δ: 9.17 (brs, 1H), 7.84 (d, 1H, J = 7.2 Hz), 7.54-7.70 (m, 3H), 3.49 (d, 1H, J = 8.7 Hz), 2.87 (d, 1H, J = 8.7 Hz), 2.23 (s, 3H), 1.23 (s, 3H), 1.11 (s, 3H); ^{13}C NMR (126 MHz, $CDCl_3$) δ: 213.4, 164.6, 134.2, 133.2, 131.7, 130.2, 124.1, 121.7 (q, J = 286.1 Hz), 90.5 (q, J = 34.1 Hz), 69.0, 47.9, 25.8, 22.0, 21.7; ^{19}F NMR (369 MHz, $CDCl_3$) δ: –78.4; IR (KBr, cm^{-1}) ν: 3138, 2941, 1709, 1471, 1304, 1199; LRMS (ESI): *m/z* 354 $[M+Na]^+$; HRMS (ESI): *m/z* calcd for $C_{15}H_{16}F_3NO_4Na$ $[M+Na]^+$ 354.0924, Found 354.0924.

***N*-benzyloxy-3-hexoxy-3-trifluoromethyl-isoindolinone (S4d)**

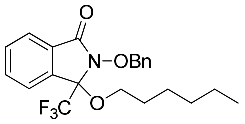

light yellow liquid; ^{1}H NMR (400 MHz, $CDCl_3$) δ: 7.90 (d, 1H, J = 6.7 Hz), 7.59-7.72 (m, 3H), 7.53-7.58 (m, 2H), 7.35-7.43 (m, 3H), 5.31 (d, 1H, J = 9.9 Hz), 5.14 (d, 1H, J = 9.9 Hz), 3.25 (dt, 1H, J = 8.7 Hz, 6.5 Hz), 2.93 (dt, 1H, J = 8.7 Hz, 6.5 Hz), 1.47-1.57 (m, 2H), 1.13-1.34 (m, 6H), 0.82 (t, 3H, J = 7.0 Hz); ^{13}C NMR (126 MHz, $CDCl_3$) δ: 165.6, 135.3, 134.6, 133.4, 131.6, 130.0, 129.3, 128.8, 128.4, 124.2, 124.1, 122.2 (q, J = 285.9 Hz), 91.0 (q, J = 32.8 Hz), 79.1, 64.2, 31.3, 28.9, 25.4, 22.4, 13.9; ^{19}F NMR (369 MHz, $CDCl_3$) δ: –78.1; IR (neat, cm^{-1}) ν: 2934, 1746, 1468, 1294, 1188; LRMS (ESI): *m/z* 430 $[M+Na]^+$; HRMS (ESI): *m/z* calcd for $C_{22}H_{24}F_3NO_3Na$ $[M+Na]^+$ 430.1601, Found 430.1604.

3-hexoxy-*N*-hydroxy-3-trifluoromethyl-isoindolinone (1d)

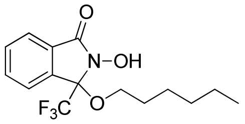

light yellow liquid; ^{1}H NMR (500 MHz, $CDCl_3$) δ: 9.88 (brs, 1H), 7.79 (d, 1H, J = 6.9), 7.66 (dd, 1H, J = 6.9 Hz, 7.5 Hz), 7.60 (d, 1H, J = 6.9 Hz), 7.59 (dd, 1H, J = 6.9 Hz, 7.5 Hz), 3.42 (dt, 1H, J = 8.6 Hz, 6.3 Hz), 2.97 (dt, 1H, J = 8.6 Hz, 6.3 Hz), 1.56-1.67 (m, 2H), 1.19-1.41 (m, 6H), 0.86 (t, 3H, J = 6.9 Hz); ^{13}C NMR (126 MHz, $CDCl_3$) δ: 165.9, 135.3, 133.2, 131.4, 130.0, 124.0, 123.8, 121.9 (q, J = 286.7 Hz), 91.0 (q, J = 32.4 Hz), 64.4, 31.4, 29.0, 25.4, 22.5, 13.9; ^{19}F NMR (369 MHz, $CDCl_3$) δ: –78.5; IR (neat, cm^{-1}) ν: 3141, 2935, 1718, 1470, 1200; LRMS (ESI): *m/z* 340 $[M+Na]^+$; HRMS (ESI): *m/z* calcd for $C_{15}H_{18}F_3NO_3Na$ $[M+Na]^+$ 340.1131, Found 340.1117.

***N*-hydroxy-3-((3-oxohexyl)oxy)-3-trifluoromethyl-isoindolinone (2d)**

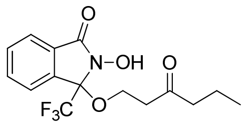

Starting material **1d** (95.2 mg, 0.3 mmol) was oxygenated under condition A of section 2, and regiomixture of **2d** + **2d'** (41.0 mg, 0.12 mmol) was obtained according to the procedure described in the General method(41% combined yield).

HPLC purification was required for the separation of the oxygenation products of 1-hexanol, γ-oxo- (**2d**) and δ-oxo-1-hexanol (**2d'**), which ware not separable by normal silica gel column chromatography.

column:	Inertsil® Diol φ 20 mm x 250 mm
eluent:	hexane/$CHCl_3$ = 2/1
flow rate:	9.5 mL/min
RT:	46 min (**2d**), 83 min (**2d'**)

light yellow liquid; ^{1}H NMR (400 MHz, $CDCl_3$) δ: 8.99 (brs, 1H), 7.85 (d, 1H, *J* = 7.2 Hz), 7.56-7.67 (m, 3H), 3.52-3.60 (m, 1H), 3.21-3.28 (m, 1H), 2.81-2.91 (m, 1H), 2.61-2.69 (m, 1H), 2.44 (t, 2H, *J* = 7.2 Hz), 1.54-1.65 (m, 2H), 0.89 (t, 3H, *J* = 7.4 Hz); ^{13}C NMR (100 MHz, $CDCl_3$) δ: 210.7, 163.9, 133.9, 133.0, 131.6, 130.4, 124.1, 121.7 (q, *J* = 286.3 Hz), 90.5 (q, *J* = 34.1 Hz), 57.9, 45.1, 41.1, 17.0, 13.4; ^{19}F NMR (369 MHz, $CDCl_3$) δ: –78.2; IR (neat, cm^{-1}) ν: 3219, 2964, 1717, 1469, 1302, 1198; LRMS (ESI): *m/z* 354 $[M+Na]^+$; HRMS (ESI): *m/z* calcd for $C_{15}H_{16}F_3NO_4Na$ $[M+Na]^+$ 354.0924, Found 354.0929.

***N*-hydroxy-3-((4-oxohexyl)oxy)-3-trifluoromethyl-isoindolinone (2d')**

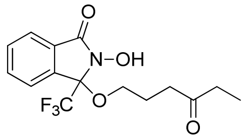

light yellow liquid; ^{1}H NMR (400 MHz, $CDCl_3$) δ: 9.50 (brs, 1H), 7.83 (d, 1H, *J* = 7.6 Hz), 7.55-7.68 (m, 3H), 3,25-3.32 (m, 1H), 3.07-3.14 (m, 1H), 2.54-2.69 (m, 2H), 2.48 (q, 2H, *J* = 7.3 Hz), 1.80-2.00 (m, 2H), 1.04 (t, 3H, *J* = 7.3 Hz); ^{13}C NMR (126 MHz, $CDCl_3$) δ: 212.5, 165.2, 134.8, 133.2, 131.6, 130.1, 124.1, 124.0, 121.9 (q, *J* = 286.7 Hz), 90.8 (q, *J* = 33.2 Hz), 62.8, 38.3, 35.9, 23.4, 7.7; ^{19}F NMR (369 MHz, $CDCl_3$) δ: –78.3; IR (neat, cm^{-1}) ν: 3218, 2925, 1716, 1469, 1190; LRMS (ESI): *m/z* 354 $[M+Na]^+$; HRMS (ESI): *m/z* calcd for $C_{15}H_{16}F_3NO_4Na$ $[M+Na]^+$ 354.0924, Found 354.0929.

5-((*N*-benzyloxy-3-oxo-1-(trifluoromethyl)isoindolin-1-yl)oxy)pentyl acetate (S4e)

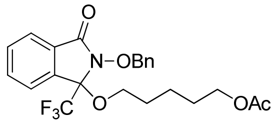

light yellow liquid; ^{1}H NMR (500 MHz, $CDCl_3$) δ: 7.89 (d, 1H, *J* = 7.5 Hz), 7.69 (ddd, 1H, 1.2 Hz, 7.5 Hz, 7.5 Hz), 7.63 (ddd, 1H, *J* = 1.2 Hz, 7.5 Hz, 7.5 Hz), 7.60 (d, 2H, *J* = 7.5 Hz), 7.52-7.56 (m, 2H), 7.34-7.41

(m, 3H), 5.31 (d, 1H, *J* = 9.8 Hz), 5.12 (d, 1H, *J* = 9.8 Hz), 3.96 (t, 2H, *J* = 6.3 Hz), 3.23 (dt, 1H, *J* = 8.6 Hz, 6.3 Hz), 2.93 (dt, 1H, *J* = 8.6 Hz, 6.3 Hz), 1.50-1.57 (m, 4H), 1.30-1.38 (m, 2H); ^{13}C NMR (126 MHz, $CDCl_3$) δ: 171.0, 165.6, 135.2, 134.5, 133.5, 131.6, 130.0, 129.3, 128.8, 128.4, 124.1, 122.2 (q, *J* = 285.5 Hz), 90.8 (q, *J* = 33.6 Hz), 79.1, 64.1, 63.7, 28.5, 28.0, 22.2, 20.8; ^{19}F NMR (369 MHz, $CDCl_3$) δ: –78.1; IR (neat, cm^{-1}) ν: 2951, 1741, 1469, 1367, 1244, 1189; LRMS (ESI): *m/z* 474 $[M+Na]^+$; HRMS (ESI): *m/z* calcd for $C_{23}H_{24}F_3NO_5Na$ $[M+Na]^+$ 474.1499, Found 474.1496.

5-((*N*-hydroxy-3-oxo-1-(trifluoromethyl)isoindolin-1-yl)oxy)pentyl acetate (1e)

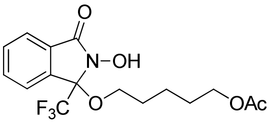

light yellow liquid; ^{1}H NMR (500 MHz, $CDCl_3$) δ: 9.76 (brs, 1H), 7.80 (d, 1H, *J* = 7.5 Hz), 7.66 (ddd, 1H, *J* = 1.2 Hz, 7.5 Hz, 7.5 Hz), 7.60 (dd, 2H, *J* = 7.5 Hz, 7.5 Hz), 4.06 (t, 2H, *J* = 6.9 Hz), 3.40 (dt, 1H, *J* = 9.2 Hz, 6.3 Hz), 2.98 (dt, 1H, *J* = 9.2 Hz, 6.3 Hz), 2.02 (s, 3H), 1.57-1.67 (m, 4H), 1.34-1.50 (m, 2H); ^{13}C NMR (126 MHz, $CDCl_3$) δ: 171.5, 165.7, 135.1, 133.2, 131.5, 130.0, 124.0, 123.9, 121.9 (q, *J* = 285.5 Hz), 90.9 (q, *J* = 33.6 Hz), 64.3, 63.9, 28.5, 28.1, 22.2, 20.9; ^{19}F NMR (369 MHz, $CDCl_3$) δ: –78.5; IR (neat, cm^{-1}) ν: 3175, 2955, 1736, 1470, 1241, 1198; LRMS (ESI): *m/z* 384 $[M+Na]^+$; HRMS (ESI): *m/z* calcd for $C_{16}H_{18}F_3NO_5Na$ $[M+Na]^+$ 384.1029, Found 384.1039.

5-((*N*-hydroxy-3-oxo-1-(trifluoromethyl)isoindolin-1-yl)oxy)-3-oxopentyl acetate (2e)

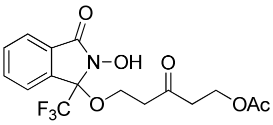

Starting material **1e** (36.1 mg, 0.1 mmol) was oxygenated under condition A of section 2, and **2e** (13.8 mg, 0.037 mmol) was isolated as described in the General method (37% yield).

light yellow liquid; ^{1}H NMR (500 MHz, $CDCl_3$) δ: 8.58 (brs, 1H), 7.88 (d, 1H, *J* = 7.4 Hz), 7.58-7.69 (m, 3H), 4.34 (t, 2H, *J* = 6.0 Hz), 3.59 (dt, 1H, *J* = 3.4 Hz, 9.5 Hz), 3.26-3.30 (m, 1H), 2.93 (ddd, 1H, *J* = 18.3 Hz, 3.4 Hz, 1.8 Hz), 2.83 (t, 2H, *J* = 6.0 Hz), 2.70 (ddd, 1H, *J* = 18.3 Hz, 3.4 Hz, 1.8 Hz), 2.02 (s, 3H); ^{13}C NMR (126 MHz, $CDCl_3$) δ: 207.4, 170.9, 163.9, 133.9, 133.0, 131.8, 130.4, 124.2, 124.1, 121.7 (q, *J* = 286.7 Hz), 90.5 (q, *J* = 33.6 Hz), 58.8, 57.7, 41.9, 41.6, 20.7; ^{19}F NMR (369 MHz, $CDCl_3$) δ: –78.2; IR (neat, cm^{-1}) ν: 3302, 2955, 1738, 1469, 1370, 1191; LRMS (ESI): *m/z* 398 $[M+Na]^+$; HRMS (ESI): *m/z* calcd for $C_{16}H_{16}F_3NO_6Na$ $[M+Na]^+$ 398.0822, Found 398.0808.

2-(5-((2-(benzyloxy)-3-oxo-1-(trifluoromethyl)isoindolin-1-yl)oxy)pentyl)isoindoline-1,3-dione (S4f)

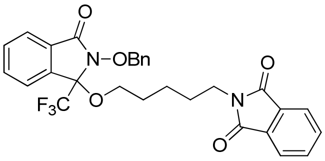

light yellow solid; ^{1}H NMR (500 MHz, $CDCl_3$) δ: 7.88 (d, 1H, J = 7.4 Hz), 7.78-7.83 (m, 2H), 7.66-7.72 (m, 3H), 7.58-7.65 (m, 2H), 7.54 (d, 2H, J = 6.9 Hz), 7.31-7.40 (m, 3H), 5.29 (d, 1H, J = 9.7 Hz), 5.12 (d, 1H, J = 9.7 Hz), 3.60 (t, 2H, J = 7.2 Hz), 3.20-3.26 (m, 1H), 2.88-2.94 (m, 1H), 1.48-1.64 (m, 4H), 1.23-1.39 (m, 2H); ^{13}C NMR (126 MHz, $CDCl_3$) δ: 168.3, 165.6, 135.2, 134.6, 133.8, 133.5, 132.0, 131.6, 129.9, 129.3, 128.8, 128.4, 124.2, 124.1, 123.1, 122.1 (q, J = 286.0 Hz), 90.9 (q, J = 32.9 Hz), 79.1, 63.7, 37.6, 28.4, 28.1, 23.1; ^{19}F NMR (369 MHz, $CDCl_3$) δ: –78.0; IR (KBr, cm^{-1}) ν: 3463, 3038, 2948, 2893, 1742, 1707, 1607, 1194; LRMS (ESI): m/z 561 $[M+Na]^+$; HRMS (ESI): m/z calcd for $C_{29}H_{25}F_3N_2O_5Na$ $[M+Na]^+$ 561.1608, Found 561.1587.

2-(5-((2-hydroxy-3-oxo-1-(trifluoromethyl)isoindolin-1-yl)oxy)pentyl)isoindoline-1,3-dione (1f)

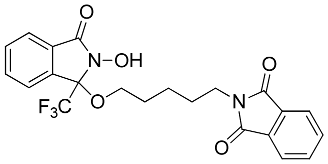

white solid; ^{1}H NMR (400 MHz, acetone-d_6) δ: 9.55 (brs, 1H), 7.66-7.84 (m, 8H), 3.61 (t, 2H, J = 7.2 Hz), 3.36-3.44 (m, 1H), 2.95-3.04 (m, 1H), 1.55-1.71 (m, 4H), 1.28-1.48 (m, 2H); ^{13}C NMR (100 MHz, acetone-d_6) δ: 168.7, 164.6, 135.7, 134.9, 134.1, 132.9, 132.6, 131.5, 125.1, 124.2, 123.6, 123.3 (q, J = 286.6 Hz), 91.4 (q, J = 31.9 Hz), 64.2, 38.1, 29.2, 28.7, 23.8; ^{19}F NMR (369 MHz, acetone-d_6) δ: –78.7; IR (KBr, cm^{-1}) ν: 3524, 3368, 2940, 2889, 2712, 1731, 1699, 1200; LRMS (ESI): m/z 471 $[M+Na]^+$; HRMS (ESI): m/z calcd for $C_{22}H_{19}F_3N_2O_5Na$ $[M+Na]^+$ 471.1138, Found 471.1119.

2-(5-((2-hydroxy-3-oxo-1-(trifluoromethyl)isoindolin-1-yl)oxy)-3-oxopentyl)isoindoline-1,3-dione (2f)

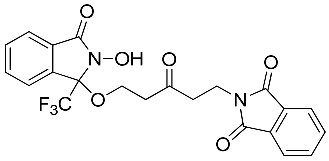

Starting material **1f** (89.7 mg, 0.2 mmol) was oxygenated under condition A of section 2, and **2f** (30.9 mg, 0.067 mmol) was isolated as described in the General method (33% yield).

light yellow liquid; ^{1}H NMR (500 MHz, $CDCl_3$) δ: 8.32 (brs, 1H), 7.89 (dd, 1H, J = 6.3 Hz, 1.1 Hz), 7.84 (dd, 2H, J = 3.1 Hz, 5.6 Hz), 7.72 (dd, 2H, J = 3.1 Hz, 5.6 Hz), 7.58-7.69 (m, 3H), 3.98 (t, 2H, J = 7.2 Hz), 3.57 (dt, 1H, J = 2.9 Hz, 9.7 Hz), 3.23-3.28 (m, 1H), 2.91-3.02 (m, 3H), 2.67-2.75 (m, 1H); ^{13}C NMR (126 MHz, $CDCl_3$) δ: 207.7, 168.1, 163.8, 134.1, 133.9, 133.0, 131.8, 131.7, 130.4, 124.2, 124.1, 123.4, 121.7 (q, J = 286.6 Hz), 90.5 (q, J = 33.6 Hz), 57.7, 41.3, 41.2, 32.7; ^{19}F NMR (369 MHz, $CDCl_3$) δ: –78.1; IR (neat, cm^{-1}) ν: 3222, 2950, 1714, 1469, 1397, 1374, 1191; LRMS (ESI): m/z 485 $[M+Na]^+$; HRMS (ESI): m/z calcd for $C_{22}H_{17}F_3N_2O_6Na$ $[M+Na]^+$ 485.0931, Found 485.0941.

methyl 6-((2-(benzyloxy)-3-oxo-1-(trifluoromethyl)isoindolin-1-yl)oxy)hexanoate (S4g)

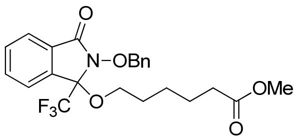

colorless liquid; ^{1}H NMR (500 MHz, $CDCl_3$) δ: 7.89 (d, 1H, *J* = 7.4 Hz), 7.58-7.71 (m, 3H), 7.52-7.57 (m, 2H), 7.34-7.42 (m, 3H), 5.31 (d, 1H, *J* = 9.5 Hz), 5.13 (d, 1H, *J* = 9.5 Hz), 3.63 (s, 3H), 3.20-3.26 (m, 1H), 2.90-2.96 (m, 1H), 2.22 (t, 2H, *J* = 7.4 Hz), 1.47-1.58 (m, 4H), 1.24-1.36 (m, 2H); ^{13}C NMR (126 MHz, $CDCl_3$) δ: 173.9, 165.6, 135.2, 134.6, 133.5, 131.6, 130.0, 129.3, 128.8, 128.4, 124.2, 124.1, 122.1 (q, *J* = 286.0 Hz), 90.1 (q, *J* = 32.9 Hz), 79.1, 63.8, 51.4, 33.7, 28.6, 25.3, 24.4; ^{19}F NMR (369 MHz, $CDCl_3$) δ: –78.1; IR (neat, cm^{-1}) ν: 2950, 1741, 1468, 1194; LRMS (ESI): *m/z* 474 $[M+Na]^+$; HRMS (ESI): *m/z* calcd for $C_{23}H_{24}F_3NO_5Na$ $[M+Na]^+$ 474.1499, Found 474.1515.

methyl 6-((2-hydroxy-3-oxo-1-(trifluoromethyl)isoindolin-1-yl)oxy)hexanoate (1g)

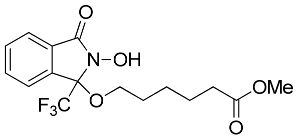

red liquid; ^{1}H NMR (500 MHz, $CDCl_3$) δ: 9.71 (brs, 1H), 7.81 (d, 1H, *J* = 7.4 Hz), 7.63-7.68 (m, 1H), 7.56-7.62 (m, 2H), 3.64 (s, 3H), 3.36-3.42 (m, 1H), 2.95-3.01 (m, 1H), 2.29 (t, 2H, *J* = 7.4 Hz), 1.55-1.66 (m, 4H), 1.30-1.46 (m, 2H); ^{13}C NMR (126 MHz, $CDCl_3$) δ: 174.4, 165.6, 135.0, 133.2, 131.5, 130.0, 124.0, 123.9, 121.9 (q, *J* = 286.7 Hz), 90.9 (q, *J* = 32.8 Hz), 63.8, 51.6, 33.8, 28.5, 25.2, 24.3; ^{19}F NMR (369 MHz, $CDCl_3$) δ: –78.4; IR (neat, cm^{-1}) ν: 3177, 2951, 1719, 1469, 1303, 1199; LRMS (ESI): *m/z* 384 $[M+Na]^+$; HRMS (ESI): *m/z* calcd for $C_{16}H_{18}F_3NO_5Na$ $[M+Na]^+$ 384.1029, Found 384.1029.

methyl 6-((2-hydroxy-3-oxo-1-(trifluoromethyl)isoindolin-1-yl)oxy)-4-oxohexanoate (2g)

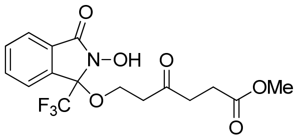

Starting material **1g** (72.3mg, 0.2 mmol) was oxygenated under condition A of section 2, and **2g** (22.2 mg, 0.059 mmol) was isolated as described in the General method (30% yield).

light yellow liquid; ^{1}H NMR (500 MHz, $CDCl_3$) δ: 8.60 (brs, 1H), 7.88 (d, 1H, *J* = 8.0 Hz), 7.57-7.68 (m, 3H), 3.67 (s, 3H), 3.57 (dt, 1H, *J* = 2.5 Hz, 9.6 Hz), 3.24-3.29 (m, 1H), 2.97 (ddd, 1H, *J* = 18.3 Hz, 10.0 Hz, 3.7 Hz), 2.62-2.87 (m, 4H), 2.55-2.62 (m, 1H); ^{13}C NMR (126 MHz, $CDCl_3$) δ: 208.8, 173.1, 163.7, 133.8, 132.9, 131.7, 130.5, 124.2, 124.1, 121.2 (q, *J* = 286.6 Hz), 90.5 (q, *J* = 33.6 Hz), 57.8, 52.0, 41.4, 37.5, 27.6; ^{19}F NMR (369 MHz, $CDCl_3$) δ: –78.1; IR (neat, cm^{-1}) ν: 3222, 2954, 1718, 1197; LRMS (ESI): *m/z* 398 $[M+Na]^+$; HRMS (ESI): *m/z* calcd for $C_{16}H_{16}F_3NO_6Na$ $[M+Na]^+$ 398.0822, Found 398.0804.

***N*-benzyloxy-3-(2-cyclohexylethoxy)-3-trifluoromethyl-isoindolinone (S4i)**

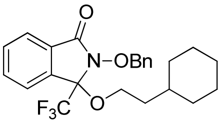

light yellow liquid; ^{1}H NMR (500 MHz, $CDCl_3$) δ: 7.90 (d, 1H, 7.5 Hz), 7.67-7.72 (m, 1H), 7.60-7.66 (m, 2H), 7.57 (dd, J = 1.2 Hz 8.1 Hz), 7.35-7.43 (m, 3H), 5.33 (d, 1H, J = 9.7 Hz), 5.15 (d, 1H, J = 9.7 Hz), 3.31 (dd, 1H, J = 8.6 Hz, 6.9 Hz), 2.99 (dd, 1H, J = 8.6 Hz, 6.9 Hz), 1.50-1.65 (m, 5H), 1.40-1.45 (m, 2H), 1.26-1.38 (m, 1H), 1.02-1.20 (m, 3H), 0.74-0.83 (m, 2H); ^{13}C NMR (126 MHz, $CDCl_3$) δ: 165.6, 135.3, 134.6, 133.4, 131.6, 130.0, 129.3, 128.8, 128.4, 124.13, 124.10, 122.2 (q, J = 287.6 Hz), 90.9 (q, J = 33.6 Hz), 79.1, 62.1, 36.4, 34.1, 33.2, 33.05, 33.02, 26.3, 26.05, 25.96; ^{19}F NMR (369 MHz, $CDCl_3$) δ: –78.1; IR (neat, cm^{-1}) ν: 2924, 1746, 1664, 1468, 1295, 1189; LRMS (ESI): *m/z* 456 $[M+Na]^+$; HRMS (ESI): *m/z* calcd for $C_{24}H_{26}F_3NO_3Na$ $[M+Na]^+$ 456.1757, Found 456.1754.

3-(2-cyclohexylethoxy)-*N*-hydroxy-3-trifluoromethyl-isoindolinone (1i)

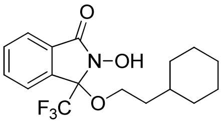

white solid; ^{1}H NMR (500 MHz, $CDCl_3$) δ: 9.87 (s, 1H), 7.79 (d, 1H, J = 8.0 Hz), 7.66 (dd, 1H, J = 8.0 Hz, 7.4 Hz), 7.57-7.62 (m, 2H), 3.47 (dt, 1H, J = 8.0 Hz, 6.9 Hz), 3.00 (dt, 1H, J = 8.0 Hz, 6.9 Hz), 1.47-1.69 (m, 6H), 1.32-1.43 (m, 1H), 1.05-1.28 (m, 4H), 0.88-0.90 (m, 2H); ^{13}C NMR (126 MHz, $CDCl_3$) δ: 165.9, 135.3, 133.2, 131.4, 130.0, 124.0, 123.9, 121.9 (q, J = 286.7 Hz), 91.0 (q, J = 32.4 Hz), 62.4, 36.4, 34.2, 33.2, 33.0, 26.4, 26.2, 26.1; ^{19}F NMR (369 MHz, $CDCl_3$) δ: –78.5; IR (KBr, cm^{-1}) ν: 3176, 3114, 2933, 2854, 1721, 1452, 1309, 1185; LRMS (ESI): *m/z* 366 $[M+Na]^+$; HRMS (ESI): *m/z* calcd for $C_{17}H_{20}F_3NO_3Na$ $[M+Na]^+$ 366.1288, Found 366.1277.

***N*-hydroxy-3-(2-(1-hydroxycyclohexyl)ethoxy)-3-trifluoromethyl-isoindolinone (2i)**

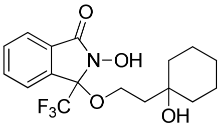

Starting material **1i** (1.02 g, 2.97 mmol) was oxygenated under condition B of section 2, and **2i** (597 mg, 1.66 mmol) was isolated as described in the General method (56% yield).
white solid; ^{1}H NMR (500 MHz, $CDCl_3$) δ: 9.42 (s, 1H), 7.88 (d, 1H, J = 7.5 H), 7.58-7.68 (m, 3H), 3.53 (dt, 1H, J = 3.4 Hz, 9.8 Hz), 3.27-3.33 (m, 1H), 1.96 (ddd, 1H, J = 4.6 Hz, 5.7 Hz, 14.9 Hz), 1.84-1.91 (m, 1H), 1.22-1.66 (m, 10H); ^{13}C NMR (126 MHz, acetone-d_6) δ: 164.2, 135.6, 133.9, 132.7, 131.8, 125.2, 124.3, 123.3 (q, J = 285.5 Hz), 91.4 (q, J = 32.4 Hz), 70.7, 61.0, 41.4, 38.6, 38.5, 26.4, 22.7; ^{19}F NMR (369 MHz, acetone-d_6) δ: –78.8; IR (KBr, cm^{-1}) ν: 3321, 2933, 2857, 2771, 1720, 1473, 1193; LRMS (ESI): *m/z* 382 $[M+Na]^+$; HRMS (ESI): *m/z* calcd for $C_{17}H_{20}F_3NO_4Na$ $[M+Na]^+$ 382.1237, Found 382.1249.

***N*-benzyloxy-3-cyclohexylmethoxy-3-trifluoromethyl-isoindolinone (S4j)**

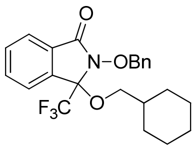

light yellow liquid; ^{1}H NMR (400 MHz, $CDCl_3$) δ: 7.90 (d, 1H, *J* = 7.6 Hz), 7.69 (ddd, 1H, *J* = 1.3 Hz, 7.6 Hz, 7.6 Hz), 7.64 (dd, 1H, *J* = 7.6 Hz, 7.6 Hz), 7.54-7.62 (m, 3H), 7.35-7.44 (m, 3H), 5.32 (d, 1H, *J* = 9.9 Hz), 5.26 (d, 1H, *J* = 9.9 Hz), 3.11 (dd, 1 H, *J* = 6.3 Hz, 8.5 Hz), 2.71 (dd, 1H, *J* = 6.7 Hz, 8.5 Hz), 1.71-1.82 (m, 1H), 1.50-1.71 (m, 5H), 1.02-1.28 (m, 3H), 0.76-0.89 (m, 2H); ^{13}C NMR (100 MHz, $CDCl_3$) δ: 165.6, 135.3, 134.6, 133.4, 131.6, 130.0, 129.3, 128.7, 128.4, 124.2, 124.1, 122.2 (q, *J* = 285.6 Hz), 90.9 (q, *J* = 32.0 Hz), 79.0, 69.2, 37.3, 29.7, 29.4, 26.3, 25.6, 25.5; ^{19}F NMR (369 MHz, $CDCl_3$) δ: –78.0; IR (neat, cm^{-1}) ν: 2927, 1746, 1648, 1291, 1187; LRMS (ESI): *m/z* 442 $[M+Na]^+$; HRMS (ESI): *m/z* calcd for $C_{23}H_{24}F_3NO_3Na$ $[M+Na]^+$ 442.1601, Found 442.1620.

3-cyclohexylmethoxy-*N*-hydroxy-3-trifluoromethyl-isoindolinone (1j)

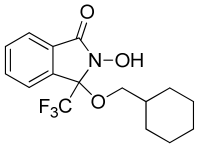

white solid; ^{1}H NMR (500 MHz, $CDCl_3$) δ: 9.86 (brs, 1H), 7.89 (d, 1H, *J* = 7.5 Hz), 7.66 (dd, 1H, *J* = 7.5 Hz, 7.5 Hz), 7.60 (dd, 2H, *J* = 7.5 Hz, 7.5 Hz), 3.24 (dd, 1H, *J* = 6.3 Hz, 8.1 Hz), 2.73 (dd, 1H, *J* = 7.5 Hz, 7.5 Hz), 1.62-1.82 (m, 6H), 1.18-1.30 (m, 2H), 1.07-1.18 (m, 1H), 0.81-0.98 (m, 2H); ^{13}C NMR (126 MHz, $CDCl_3$) δ: 165.8, 135.3, 133.2, 131.4, 130.0, 124.1, 123.8, 121.9 (q, *J* = 285.5 Hz), 91.0 (q, *J* = 33.6 Hz), 69.5, 37.3, 29.7, 29.4, 26.4, 25.7, 25.6; ^{19}F NMR (369 MHz, $CDCl_3$) δ: –78.5; IR (KBr, cm^{-1}) ν: 3117, 2925, 2856, 1708, 1473, 13990, 1309, 1184; LRMS (ESI): *m/z* 352 $[M+Na]^+$; HRMS (ESI): *m/z* calcd for $C_{16}H_{18}F_3NO_3Na$ $[M+Na]^+$ 352.1131, Found 352.1118.

***N*-hydroxy-3-((1-hydroxycyclohexyl)methoxy)-3-trifluoromethyl-isoindolinone (2j)**

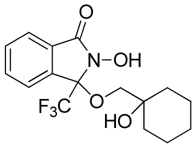

Starting material **1j** (32.9 mg, 0.1 mmol) was oxygenated under condition B of section 2, and **2j** (9.2 mg, 0.027 mmol) was isolated as described in the General method (27% yield).

white solid; ^{1}H NMR (500 MHz, $CDCl_3$) δ: 8.97 (brs, 1H), 7.84 (d, 1H, *J* = 6.9 Hz), 7.61-7.70 (m, 3H), 3.33 (d, 1H, *J* = 11.5 Hz), 2.99 (d, 1H, *J* = 11.5 Hz), 1.89-1.97 (m, 1H), 1.48-1.70 (m, 5H), 1.39-1.48 (m, 2H), 1.24-1.34 (m, 2H); ^{13}C NMR (100 MHz, $CDCl_3$) δ: 163.9, 134.7, 133.0, 131.6, 130.8, 124.0, 123.8, 121.9 (q, *J* = 286.6 Hz), 91.0 (q, *J* = 33.2 Hz), 73.4, 71.6, 34.1, 32.7, 25.6, 21.5; ^{19}F NMR (369 MHz, $CDCl_3$) δ: –78.0; IR (neat, cm^{-1}) ν: 3318, 2936, 2861, 1723, 1469, 1298, 1191; LRMS (ESI): *m/z* 368 $[M+Na]^+$; HRMS (ESI): *m/z* calcd for $C_{16}H_{18}F_3NO_4Na$ $[M+Na]^+$ 368.1080, Found 368.1092.

(*R*)-*N*-benzyloxy-3-(((1*S*,2*R*,5*S*)-2-isopropyl-5-methylcyclohexyl)oxy)-3-trifluoromethyl-isoindolinone ((*R*)-S4k)

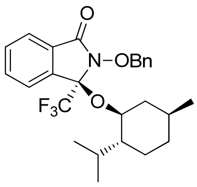

white solid; ^{1}H NMR (500 MHz, $CDCl_3$) δ: 7.88 (dd, 1H, *J* = 1.7 Hz, 6.3 Hz), 7.67-7.71 (m, 1H), 7.59-7.67 (m, 2H), 7.55 (dd, 2H, *J* = 1.7 Hz, 7.4 Hz), 7.36-7.42 (m, 3H), 5.46 (d, 1H, *J* = 8.6 Hz), 5.01 (d, 1H, *J* = 8.6 Hz), 3.40 (dt, 1H, *J* = 4.6 Hz 10.4 Hz), 2.31-2.41 (m, 1H), 1.43-1.55 (m, 2H), 1.22-1.29 (m, 1H), 0.90-1.01 (m, 1H), 0.75-0.84 (m, 2H), 0.79 (d, 3H, *J* = 6.9 Hz), 0.58-0.71 (m, 2H), 0.54 (d, 1H, *J* = 6.9 Hz), 0.33 (d, 3H, *J* = 6.9 Hz); ^{13}C NMR (126 MHz, $CDCl_3$) δ: 164.7, 138.1, 134.0, 132.9, 131.3, 130.0, 129.0, 128.9, 128.3, 124.7, 123.8, 122.7 (q, *J* = 286.7 Hz), 89.1 (q, *J* = 33.6 Hz), 78.6, 76.0, 48.6, 42.5, 33.7, 31.1, 24.4, 22.8, 22.0, 21.4, 16.0; ^{19}F NMR (369 MHz, $CDCl_3$) δ: –78.3; IR (KBr, cm^{-1}) ν: 3037, 2954, 2931, 2863, 1740, 1616, 1187; LRMS (ESI): *m/z* 484 $[M+Na]^+$; HRMS (ESI): *m/z* calcd for $C_{26}H_{30}F_3NO_3Na$ $[M+Na]^+$ 484.2070, Found 484.2088.

(*R*)-*N*-hydroxy-3-(((1*S*,2*R*,5*S*)-2-isopropyl-5-methylcyclohexyl)oxy)-3-trifluoromethyl-isoindolinone ((*R*)-1k)

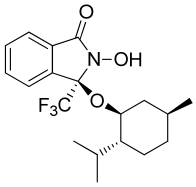

reddish solid; ^{1}H NMR (400 MHz, $CDCl_3$) δ: 9.74 (brs, 1H), 7.76 (d, 1H, *J* = 6.7 Hz), 7.53-7.67 (m, 3H), 3.64 (dt, 1H, *J* = 4.3 Hz, 10.4 Hz), 2.45-2.57 (m, 1H), 1.58-1.68 (m, 1H), 1.49-1.58 (m, 1H), 1.28-1.43 (m, 1H), 0.87-1.15 (m, 3H), 0.95 (d, 3H, *J* = 7.2 Hz), 0.83 (d, 3H, *J* = 6.7 Hz), 0.59 (d, 3H, *J* = 6.7 Hz); ^{13}C NMR (100 MHz, $CDCl_3$) δ: 165.7, 138.0, 132.8, 131.2, 128.9, 124.7, 123.6, 122.2 (q, *J* = 287.5 Hz), 90.1 (q, *J* = 32.6 Hz), 76.7, 48.6, 42.8, 33.9, 31.2, 24.9, 23.0, 22.0, 21.4, 15.9; ^{19}F NMR (369 MHz, $CDCl_3$) δ: –78.5; IR (KBr, cm^{-1}) ν: 3198, 2959, 2871, 1719, 1472, 1375, 1290, 1192; LRMS (ESI): *m/z* 394 $[M+Na]^+$; HRMS (ESI): *m/z* calcd for $C_{19}H_{24}F_3NO_3Na$ $[M+Na]^+$ 394.1601, Found 394.1611.

(*R*)-*N*-hydroxy-3-(((1*S*,2*S*,5*S*)-2-(2-hydroxypropan-2-yl)-5-methylcyclohexyl)oxy)-3-trifluoromethyl-isoindolinone ((*R*)-2k)

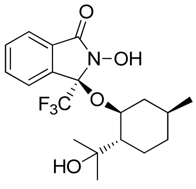

Starting material (*R*)-**1k** (37.1 mg, 0.1 mmol) was oxygenated under condition B of section 2, and (*R*)-**2k** (36.2 mg, 0.093 mmol) was isolated as described in the General method (93% yield).

white solid; ^{1}H NMR (400 MHz, $CDCl_3$) δ: 10.4 (brs, 1H), 7.80-7.87 (m, 1H), 7.66-7.73 (m, 1H), 7.57-7.64 (m, 2H), 4.26 (brs, 1H), 3.75-3.83 (m, 1H), 1.57-1.67 (m, 1H), 1.51-1.60 (m, 1H), 1.44 (s, 3H), 1.32-1.39 (m, 1H), 1.27 (s, 3H), 0.93-1.13 (m, 2H), 0.72-0.87 (m, 1H), 0.66 (d, 3H, J = 6.3 Hz); ^{13}C NMR (100 MHz, $CDCl_3$) δ: 163.6, 135.9, 132.0, 131.5, 130.6, 125.6, 123.8, 122.1 (q, J = 285.6 Hz), 90.6 (q, J = 32.0 Hz), 74.5, 52.0, 43.1, 33.7, 31.0, 30.9, 27.2, 25.7, 21.9; ^{19}F NMR (369 MHz, $CDCl_3$) δ: –77.3; IR (KBr, cm^{-1}) ν: 3366, 2974, 2928, 2866, 1726, 1191; LRMS (ESI): *m/z* 410 [M+Na]$^+$; HRMS (ESI): *m/z* calcd for $C_{19}H_{24}F_3NO_4Na$ [M+Na]$^+$ 410.1550, Found 410.1551.

_N_-benzyloxy-3-(((_S_)-3,7-dimethyloct-6-en-1-yl)oxy)-3-trifluoromethyl-isoindolinone (S4l)

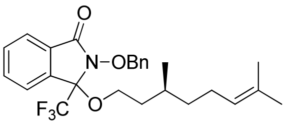

light yellow liquid (diastereomixture); ^{1}H NMR (500 MHz, $CDCl_3$) δ: 7.91 (d, 1H + 1H, J = 7.4 Hz), 7.60-7.72 (m, 3H + 3H), 7.55-7.59 (m, 2H + 2H), 7.35-7.43 (m, 3H + 3H), 5.31-5.35 (m, 1H + 1H), 5.14-5.18 (m, 1H + 1H), 5.00-5.07 (m, 1H + 1H), 3.27-3.36 (m, 1H + 1H), 2.96-3.03 (m, 1H + 1H), 1.83-2.00 (m, 1H + 1H), 1.68 (s, 3H), 1.67 (s, 3H), 1.48-1.68 (m, 2H + 2H), 1.59 (s, 3H), 1.58 (s, 3H), 1.19-1.40 (m, 2H + 2H), 1.01-1.14 (m, 1H + 1H), 0.75-0.79 (m, 3H + 3H); ^{13}C NMR (126 MHz, $CDCl_3$) δ: 165.6, 165.5, 135.31, 135.26, 134.6, 133.4, 131.6, 131.2, 131.1, 130.04, 130.02, 129.30, 129.28, 128.8, 128.39, 128.37, 124.5, 124.1, 122.2 (q, J = 285.1), 90.93 (q, J = 32.8 Hz), 90.90 (q, J = 32.8 Hz), 79.1, 77.2, 62.43, 62.40, 36.9, 36.7, 35.9, 35.8, 29.1, 29.0, 25.6, 25.2, 19.4, 19.2, 17.5; ^{19}F NMR (369 MHz, $CDCl_3$) δ: –78.0, –78.1; IR (neat, cm^{-1}) ν: 2925, 1746, 1468, 1294, 1188; LRMS (ESI): *m/z* 484 [M+Na]$^+$; HRMS (ESI): *m/z* calcd for $C_{22}H_{26}N_2O_2Na$ [M+Na]$^+$ 484.2070, Found 484.2062.

3-(((_S_)-3,7-dimethyloctyl)oxy)-_N_-hydroxy-3-trifluoromethyl-isoindolinone (1l)

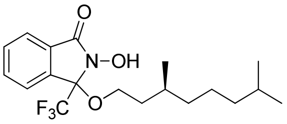

light yellow liquid (diastereomixture); ^{1}H NMR (500 MHz, $CDCl_3$) δ: 10.1 (brs, 1H + 1H), 7.80 (d, 1H + 1H, J = 7.5 Hz), 7.65-7.70 (m, 1H + 1H), 7.58-7.63 (m, 2H + 2H), 3.47-3.55 (m, 1H + 1H), 2.98-3.06 (m, 1H + 1H), 1.61-1.75 (m, 1H + 1H), 1.53-1.61 (m, 1H + 1H), 1.37-1.53 (m, 2H + 2H), 1.15-1.33 (m, 3H + 3H), 1.00-1.15 (m, 3H + 3H), 0.78-0.86 (m, 9H + 9H); ^{13}C NMR (126 MHz, $CDCl_3$) δ: 165.93, 165.92, 135.3, 133.2, 131.5, 130.0, 124.0, 123.9, 121.9 (q, J = 286.3 Hz), 91.1 (q, J = 33.2 Hz), 62.8, 62.7, 39.2, 39.1, 37.3, 37.0, 36.1, 36.0, 29.61, 29.58, 27.9, 24.6, 24.5, 22.63, 22.61, 22.5¸ 19.5, 19.3; ^{19}F NMR (369 MHz, $CDCl_3$) δ: –78.5; IR (neat, cm^{-1}) ν: 3154, 2954, 1718, 1469, 1306, 1201; LRMS (ESI): *m/z* 396 [M+Na]$^+$; HRMS (ESI): *m/z* calcd for $C_{19}H_{26}F_3NO_3Na$ [M+Na]$^+$ 396.1757, Found 396.1768.

_N_-hydroxy-3-((3-hydroxy-3,7-dimethyloctyl)oxy)-3-trifluoromethyl-isoindolinone (2l)

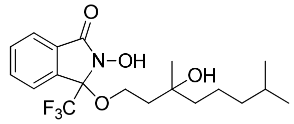

Starting material **1l** (37.3 mg, 0.1 mmol) was oxygenated under condition B of section 2, and **2l** (21.5 mg, 0.055 mmol) was obtained as a diastereomixture according to the procedure as described in the General method (55% yield).

light yellow liquid (diastereomixture); ^{1}H NMR (500 MHz, $CDCl_3$) δ: 9.65-9.86 (brs, 1H + 1H), 7.83-7.88 (m, 1H + 1H), 7.58-7.68 (m, 3H + 3H), 3.52-3.60 (m, 1H + 1H), 3.42 (brs, 1H + 1H), 3.24-3.32 (m, 1H + 1H), 1.95-2.03 (m, 1H), 1.87-1.95 (m, 1H), 1.57-1.74 (m, 2H + 2H), 1.40-1.57 (m, 2H + 2H), 1.36 (s, 3H), 1.23-1.38 (m, 2H), 1.18 (s, 3H), 1.07-1.20 (m, 2H + 2H), 0.82-0.88 (m, 6H + 6H); ^{13}C NMR (126 MHz, $CDCl_3$) δ: 164.8, 134.44, 134.41, 133.0, 131.6, 130.4, 124.15, 124.13, 124.0, 121.8 (q, J = 284.9 Hz), 90.9 (q, J = 34.0 Hz), 72.7, 72.6, 60.7, 60.6, 43.4, 43.0, 39.28, 39.26, 39.1, 27.8, 27.2, 26.8, 22.54, 22.52, 22.5, 21.8, 21.6; ^{19}F NMR (369 MHz, $CDCl_3$) δ: –78.2; IR (neat, cm^{-1}) ν: 3406, 3161, 2954, 1721, 1469, 1383, 1306, 1200; LRMS (ESI): *m/z* 412 [M+Na]$^+$; HRMS (ESI): *m/z* calcd for $C_{19}H_{26}F_3NO_4Na$ [M+Na]$^+$ 412.1706, Found 412.1701.

***N*-benzyloxy-3-((5-hydroxy-3-methylpentyl)oxy)-3-trifluoromethyl-isoindolinone (S4m)**

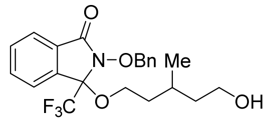

colorless liquid (diastereomixture); ^{1}H NMR (500 MHz, $CDCl_3$) δ: 7.90 (d, 1H + 1H, J = 6.9 Hz), 7.59-7.72 (m, 3H + 3H), 7.53-7.58 (m, 2H + 2H), 7.35-7.43 (m, 3H + 3H), 5.30-5.35 (m, 1H), 5.10-5.14 (m, 1H), 3.49-3.65 (m, 2H), 3.25-3.33 (m, 1H + 1H), 2.94-3.03 (m, 1H + 1H), 1.63-1.73 (m, 1H + 1H), 1.45-1.60 (m, 2H + 2H), 1.24-1.43 (m, 2H + 2H), 0.76-0.80 (m, 3H + 3H); ^{13}C NMR (100 MHz, $CDCl_3$) δ: 165.7, 165.6, 135.25, 135.21, 134.535, 134.525, 133.52, 133.50, 131.7, 130.0, 129.5, 129.45, 129.37, 128.9, 128.8, 128.5, 128.4, 124.2, 124.1, 122.2 (q, J = 286.1 Hz), 90.80 (q, J = 32.9 Hz), 90.78 (q, J = 32.9 Hz), 79.1, 62.3, 62.1, 60.714, 60.705, 39.41, 39.40, 26.5, 26.2, 19.42, 19.35; ^{19}F NMR (369 MHz, $CDCl_3$) δ: –78.2; IR (neat, cm^{-1}) ν: 3417, 2929, 1742, 1468, 1295, 1189; LRMS (ESI): *m/z* 446 [M+Na]$^+$; HRMS (ESI): *m/z* calcd for $C_{22}H_{24}F_3NO_4Na$ [M+Na]$^+$ 446.1550, Found 446.1536.

***N*-hydroxy-3-((5-hydroxy-3-methylpentyl)oxy)-3-trifluoromethyl-isoindolinone (1m)**

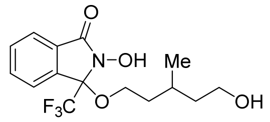

white solid (diastereomixture); ^{1}H NMR (400 MHz, acetone-d_6) δ: 7.70-7.88 (m, 4H + 4H), 3.51-3.66 (m, 2H + 2H), 3.37-3.47 (m, 1H + 1H), 3.05-3.19 (m, 1H + 1H), 1.80-1.91 (m, 1H), 1.69-1.80 (m, 1H), 1.40-1.68 (m, 3H + 3H), 1.18-1.31 (m, 1H + 1H), 0.82-0.87 (m, 3H + 3H); ^{13}C NMR (100 MHz, acetone-d_6) δ: 164.7, 135.8, 135.7, 134.1, 134.0, 132.6, 131.7, 131.6, 125.21, 125.19, 125.16, 125.14,

124.30, 124.25, 123.4 (q, J = 286.1 Hz), 123.3 (q, J = 286.1 Hz), 91.6 (q, J = 31.9 Hz), 91.5 (q, J = 32.4 Hz), 63.0, 62.6, 60.25, 60.19, 40.3, 39.9, 37.0, 36.9, 27.4, 26.5, 19.99, 19.98; ^{19}F NMR (369 MHz, acetone-d_6) δ: –78.6, –78.8; IR (KBr, cm^{-1}) ν: 3374, 2955, 2775, 1724, 1470, 1304, 1200; LRMS (ESI): *m/z* 356 [M+Na]$^+$; HRMS (ESI): *m/z* calcd for $C_{15}H_{18}F_3NO_4Na$ [M+Na]$^+$ 356.1080, Found 356.1075.

3-((3,5-dihydroxy-3-methylpentyl)oxy)-*N*-hydroxy-3-trifluoromethyl-isoindolinone (2m)

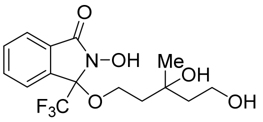

Starting material **1m** (100 mg, 0.3 mmol) was oxygenated under condition B of section 2, and **2m** (51.6 mg, 0.15 mmol) was obtained as a diastereomixture according to the procedure described in the General method (49% yield).

colorless liquid (diastereomixture); ^{1}H NMR (500 MHz, $CDCl_3$) δ: 9.73 (brs, 1H), 9.67 (brs, 1H), 7.88 (d, 1H, J = 7.4 Hz), 7.58-7.69 (m, 3H + 3H), 3.84-4.03 (m, 2H + 2H), 3.50-3.60 (m, 1H + 1H), 3.25-3.33 (m, 1H + 1H), 2.02-2.10 (m, 1H), 1.88-2.01 (m, 1H + 1H), 1.76-1.83 (m, 1H), 1.64-1.76 (m, 2H + 2H), 1.44 (s, 3H), 1.61 (s, 3H); ^{13}C NMR (126 MHz, $CDCl_3$) δ: 164.9, 164.7, 134.4, 134.3, 133.1, 133.0, 131.67, 131.65, 130.4, 130.3, 124.1, 124.0, 121.9 (q, J = 286.7 Hz), 90.9 (q, J = 32.4 Hz), 90.8 (q, J = 32.4 Hz), 73.09, 73.06, 59.34, 59.28, 42.3, 40.3, 39.7, 29.1, 27.3, 26.4; ^{19}F NMR (369 MHz, $CDCl_3$) δ: –78.2; IR (neat, cm^{-1}) ν: 3391, 2930, 1720, 1469, 1191; LRMS (ESI): *m/z* 372 [M+Na]$^+$; HRMS (ESI): *m/z* calcd for $C_{15}H_{18}F_3NO_5Na$ [M+Na]$^+$ 372.1029, Found 372.1021.

***N*-benzyloxy-3-((5-(methoxymethoxy)-3-methylpentyl)oxy)-3-trifluoromethyl-isoindolinone (S4n)**

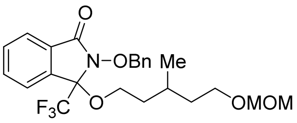

colorless liquid (diastereomixture); ^{1}H NMR (500 MHz, $CDCl_3$) δ: 7.89 (d, 1H + 1H, J = 6.9 Hz), 7.59-7.71 (m, 3H + 3H), 7.53-7.57 (m, 2H + 2H), 7.33-7.43 (m, 3H + 3H), 5.29-5.33 (m, 1H + 1H), 5.11-5.15 (m, 1H + 1H), 4.56 (s, 2H), 4.55 (s, 2H), 3.42-3.53 (m, 2H + 2H), 3.32 (s, 3H), 3.31 (s, 3H), 3.27-3.35 (m, 1H + 1H), 2.94-3.02 (m, 1H + 1H), 1.47-1.76 (m, 3H + 3H), 1.28-1.42 (m, 2H + 2H), 0.77-0.80 (m, 3H + 3H); ^{13}C NMR (126 MHz, $CDCl_3$) δ: 165.6, 165.5, 135.3, 135.2, 134.58, 134.56, 133.44, 133.42, 131.6, 130.03, 130.01, 129.33, 129.30, 128.79, 128.77, 128.40, 128.38, 124.2, 124.1, 122.2 (q, J = 285.1 Hz), 122.1 (q, J = 286.7 Hz), 96.3, 90.9 (q, J = 32.8 Hz), 90.8 (q, J = 33.2 Hz), 79.1, 65.6, 65.5, 62.2, 55.0, 36.6, 36.4, 35.9, 35.8, 26.71, 26.68, 19.3, 19.2; ^{19}F NMR (369 MHz, $CDCl_3$) δ: –78.08, –78.14; IR (neat, cm^{-1}) ν: 2931, 1745, 1468, 1294, 1189; LRMS (ESI): *m/z* 490 [M+Na]$^+$; HRMS (ESI): *m/z* calcd for $C_{24}H_{28}F_3NO_5Na$ [M+Na]$^+$ 490.1812, Found 490.1799.

***N*-hydroxy-3-((5-(methoxymethoxy)-3-methylpentyl)oxy)-3-trifluoromethyl-isoindolinone (1n)**

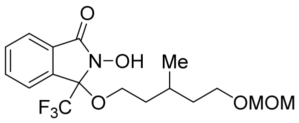

colorless liquid (diastereomixture); ^{1}H NMR (400 MHz, $CDCl_3$) δ: 9.61 (brs, 1H), 9.36 (brs, 1H), 7.80-7.86 (m, 1H + 1H), 7.56-7.68 (m 3H + 3H), 4.63 (s, 2H), 4.59 (s, 2H), 3.48-3.60 (m, 2H + 2H), 3.35-3.47 (m, 1H + 1H), 3.37 (s, 3H), 3.34 (s, 3H), 3.01-3.15 (m, 1H + 1H), 1.22-1.92 (m, 5H + 5H), 0.84-0.88 (m, 3H + 3H); ^{13}C NMR (126 MHz, $CDCl_3$) δ: 165.5, 165.4, 135.1, 135.0, 133.13, 133.06, 131.5, 130.2, 130.1, 124.1, 124.0, 123.9, 121.92 (q, J = 286.7 Hz), 121.89 (q, J = 286.7 Hz), 96.3, 96.2, 91.0 (q, J = 32.8 Hz), 90.9 (q, J = 33.2 Hz), 65.8, 65.5, 62.6, 61.9, 55.3, 55.1, 36.3, 36.0, 35.9, 35.8, 27.0, 25.9, 19.6, 19.5; ^{19}F NMR (369 MHz, $CDCl_3$) δ: –78.2, –78.5; IR (neat, cm^{-1}) ν: 3172, 2934, 1720, 1469, 1305, 1198; LRMS (ESI): *m/z* 400 [M+Na]$^+$; HRMS (ESI): *m/z* calcd for $C_{17}H_{22}F_3NO_5Na$ [M+Na]$^+$ 400.1342, Found 400.1331.

***N*-hydroxy-3-((3-hydroxy-5-(methoxymethoxy)-3-methylpentyl)oxy)-3-trifluoromethyl-isoindolinone (2n)**

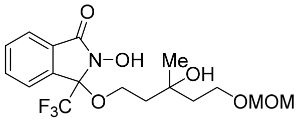

Starting material **1n** (37.7 mg, 0.1 mmol) was oxygenated under condition B of section 2, and **2n** (25.1 mg, 0.064 mmol) was obtained as a diastereomixture according to the purification procedure described in the General method (64% yield).

colorless liquid (diastereomixture); ^{1}H NMR (400 MHz, $CDCl_3$) δ: 9.43 (brs, 1H + 1H), 7.85-7.90 (m, 1H + 1H), 7.56-7.67 (m, 3H + 3H), 4.61 (s, 2H), 4.58-4.60 (m, 2H), 4.44 (brs, 1H), 4.06 (brs, 1H), 3.66-3.87 (m, 2H + 2H), 3.43-3.60 (m, 1H + 1H), 3.38 (s, 3H), 3.34 (s, 3H), 3.24-3.40 (m, 1H + 1H), 1.56-2.13 (m, 4H + 4H), 1.46 (s, 3H), 1.19 (s, 3H); ^{13}C NMR (100 MHz, $CDCl_3$) δ: 164.0, 163.8, 134.4, 134.2, 132.8, 132.7, 131.6, 131.5, 130.8, 130.7, 124.12, 124.08, 124.0, 122.0 (q, J = 286.6 Hz), 121.9 (q, J = 286.6 Hz), 96.49, 96.47, 90.8 (q, J = 32.9 Hz), 90.6 (q, J = 32.9 Hz), 72.5, 72.4, 64.4, 60.2, 60.1, 55.7, 55.6, 41.1, 40.5, 39.8, 39.4, 27.4, 26.5; ^{19}F NMR (369 MHz, $CDCl_3$) δ: –78.1, –78.2; IR (neat, cm^{-1}) ν: 3423, 2937, 1721, 1469, 1191; LRMS (ESI): *m/z* 416 [M+Na]$^+$; HRMS (ESI): *m/z* calcd for $C_{17}H_{22}F_3NO_6Na$ [M+Na]$^+$ 416.1291, Found 416.1301.

***N*-(4-methoxybenzyl)oxy-3-((3-methyl-5-(pent-4-yn-1-yloxy)pentyl)oxy)-3-trifluoromethyl-isoindolinone (S4'o)**

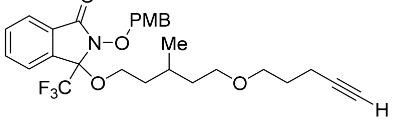

colorless liquid (diastereomixture); ^{1}H NMR (500 MHz, $CDCl_3$) δ: 7.88 (d, 1H + 1H, J = 7.4 Hz), 7.58-7.70 (m, 3H + 3H), 7.45-7.50 (m, 2H + 2H), 6.91 (d, 2H + 2H, J = 8.6 Hz), 5.24 (d, 1H + 1H, J = 9.7 Hz), 5.03-5.08 (m, 1H + 1H), 3.81 (s, 3H + 3H), 3.26-3.46 (m, 5H + 5H), 2.93-3.01 (m, 1H + 1H),

2.21-2.26 (m, 2H + 2H), 1.93 (t, 1H + 1H, J = 2.6 Hz), 1.69-1.77 (m, 2H + 2H), 1.44-1.69 (m, 3H + 3H), 1.26-1.42 (m, 2H + 2H), 0.78 (d, 3H, J = 6.9 Hz), 0.76 (d, 3H, J = 6.9 Hz); ^{13}C NMR (126 MHz, $CDCl_3$) δ: 165.5, 165.4, 160.1, 135.30, 135.25, 133.37, 133.35, 131.6, 131.12, 131.10, 130.1, 126.8, 124.2, 124.11, 124.07, 122.2 (q, J = 286.3 Hz), 113.8, 90.9 (q, J = 32.8 Hz), 90.8 (q, J = 32.8 Hz), 83.92, 83.91, 78.8, 69.0, 68.9, 68.8, 68.7, 68.35, 68.33, 62.29, 62.27, 55.2, 36.5, 36.3, 36.0, 35.9, 28.5, 26.83, 26.80, 19.4, 19.3, 15.1; ^{19}F NMR (369 MHz, $CDCl_3$) δ: –78.07, –78.13; IR (neat, cm^{-1}) ν: 3301, 2953, 2870, 1743, 1613, 1516, 1468, 1253, 1188, 1113; LRMS (ESI): *m/z* 542 $[M+Na]^+$; HRMS (ESI): *m/z* calcd for $C_{28}H_{32}F_3NO_5Na$ $[M+Na]^+$ 542.2125, Found 542.2121.

***N*-hydroxy-3-((3-methyl-5-(pent-4-yn-1-yloxy)pentyl)oxy)-3-trifluoromethyl-isoindolinone (1o)**

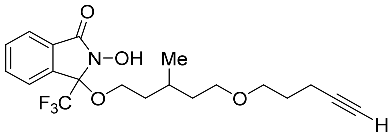

yellow liquid (diastereomixture); ^{1}H NMR (500 MHz, $CDCl_3$) δ: 9.46 (brs, 1H + 1H), 7.80-7.86 (m, 1H + 1H), 7.63-7.67 (m, 1H + 1H), 7.57-7.63 (m, 2H + 2H), 3.31-3.63 (m, 5H + 5H), 3.11-3.18 (m, 1H), 3.04-3.11 (m, 1H), 2.23-2.32 (m, 2H + 2H), 1.91-1.95 (m, 1H + 1H), 1.56-1.95 (m, 5H + 5H), 1.37-1.49 (m, 1H + 1H), 1.27-1.36 (m, 1H), 1.14-1.24 (m, 1H), 0.87 (d, 3H + 3H, J = 6.3 Hz); ^{13}C NMR (126 MHz, $CDCl_3$) δ: 165.6, 165.4, 135.0, 134.9, 133.1, 133.0, 131.47, 131.45, 130.3, 130.1, 124.2, 124.1, 124.0, 123.9, 121.88 (q, J = 286.7 Hz), 121.85 (q, J = 286.7 Hz), 91.1 (q, J = 32.8 Hz), 91.0 (q, J = 33.6 Hz), 83.82, 83.75, 69.5, 69.2, 68.9, 68.6, 68.52, 68.46, 63.0, 61.7, 36.2, 36.1, 36.0, 35.4, 28.3, 28.0, 27.4, 25.4, 19.8, 19.7, 15.1, 15.0; ^{19}F NMR (369 MHz, $CDCl_3$) δ: –77.6, –78.0; IR (neat, cm^{-1}) ν: 3302, 2932, 2874, 1718, 1469, 1303, 1198; LRMS (ESI): *m/z* 422 $[M+Na]^+$; HRMS (ESI): *m/z* calcd for $C_{20}H_{24}F_3NO_4Na$ $[M+Na]^+$ 422.1550, Found 422.1564.

***N*-hydroxy-3-((3-hydroxy-3-methyl-5-(pent-4-yn-1-yloxy)pentyl)oxy-3-trifluoromethyl-isoindolinon e (2o)**

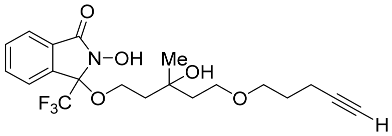

Starting material **1o** (32.0 mg, 0.08 mmol) was oxygenated under condition B of section 2, and **2o** (13.7 mg, 0.033 mmol) was obtained as a diastereomixture according to the purification procedure described in the General method (41% yield).

colorless liquid (diastereomixture); ^{1}H NMR (500 MHz, $CDCl_3$) δ: 9.46 (s, 1H), 9.39 (s, 1H), 7.86-7.92 (m, 1H + 1H), 7.57-7.67 (m, 3H + 3H), 4.65 (s, 1H), 4.31 (s, 1H), 3.25-3.76 (m, 6H + 6H), 2.22-2.29 (m, 2H + 2H), 2.02-2.13 (m, 1H), 1.96 (t, 1H, J = 2.3 Hz), 1.86-1.96 (m, 1H + 1H), 1.67-1.82 (m, 3H + 4H), 1.55-1.65 (m, 1H + 1H), 1.47 (s, 3H), 1.17 (s, 3H); ^{13}C NMR (126 MHz, $CDCl_3$) δ: 163.7, 163.6, 134.3, 134.2, 132.7, 132.6, 131.54, 131.47, 130.9, 130.8, 124.11, 124.07, 124.0, 123.9, 122.0 (q, J = 286.6 Hz), 121.9 (q, J = 286.6 Hz), 90.5 (q, J = 32.9 Hz), 83.4, 83.2, 72.8, 72.5, 70.0, 69.9, 69.0, 68.8, 67.9, 67.8, 60.1,

60.0, 41.2, 40.4, 39.8, 39.2, 28.1, 28.0, 27.6, 26.2, 15.21, 15.18; ^{19}F NMR (369 MHz, $CDCl_3$) δ: –78.1; IR (neat, cm^{-1}) ν: 3404, 3304, 2936, 2118, 1723, 1469, 1377, 1303, 1198; LRMS (ESI): *m/z* 438 $[M+Na]^+$; HRMS (ESI): *m/z* calcd for $C_{20}H_{24}F_3NO_5Na$ $[M+Na]^+$ 438.1499, Found 438.1499.

***N*-hydroxy-3-((3-hydroxy-3-methyloct-7-en-1-yl)oxy)-3-trifluoromethyl-isoindolinone (2p)**

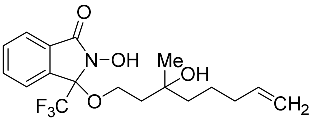

Starting material **1p** (14.3 mg, 0.04 mmol) was oxygenated under condition B of section 2, and **2p** (2.8 mg, 0.0075 mmol) was obtained as a diastereomixture according to the purification procedure described in the General method (19% yield).

colorless liquid (diastereomixture); ^{1}H NMR (500 MHz, $CDCl_3$) δ: 9.49 (brs, 1H), 9.43 (brs, 1H), 7.83-7.92 (m, 1H + 1H), 7.58-7.70 (m, 3H + 3H), 5.70-5.87 (m, 1H + 1H), 4.90-5.04 (m, 2H + 2H), 3.50-3.58 (m, 1H + 1H), 3.25-3.33 (m, 1H + 1H), 3.17 (brs, 1H + 1H), 1.86-2.10 (m, 3H + 3H), 1.58-1.72 (m, 2H + 2H), 1.35-1.55 (m, 3H + 3H), 1.36 (s, 3H), 1.19 (s, 3H); ^{13}C NMR (100 MHz, $CDCl_3$) δ: 164.6, 138.6, 138.4, 134.4, 134.3, 133.0, 131.64, 131.63, 130.4, 124.20, 124.18, 124.1, 121.9 (q, J = 286.1 Hz), 121.8 (q, J = 286.1 Hz), 114.8, 114.7, 90.89 (q, J = 33.4 Hz), 90.86 (q, J = 33.4 Hz), 72.8, 72.7, 60.6, 60.5, 42.6, 42.3, 39.3, 39.1, 34.0, 33.9, 27.3, 26.8, 23.4, 23.2; ^{19}F NMR (369 MHz, $CDCl_3$) δ: –78.2; IR (neat, cm^{-1}) ν: 3377, 2937, 1719, 1469, 1303, 1190; LRMS (ESI): *m/z* 396 $[M+Na]^+$; HRMS (ESI): *m/z* calcd for $C_{18}H_{22}F_3NO_4Na$ $[M+Na]^+$ 396.1393, Found 396.1388.

***N*-benzyloxy-3-((3-methyl-8-(4,4,5,5-tetramethyl-1,3,2-dioxaborolan-2-yl)octyl)oxy)-3-trifluoromethyl-isoindolinone (S4q)**

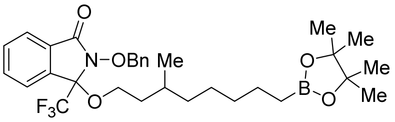

colorless liquid (diastereomixture); ^{1}H NMR (400 MHz, $CDCl_3$) δ: 7.89 (d, 1H + 1H, J = 7.2 Hz), 7.57-7.72 (m, 2H + 2H), 7.55 (d, 1H + 1H, J = 6.7 Hz), 5.25-5.33 (m, 1H + 1H), 5.13 (d, 1H, J = 9.4 Hz), 3.23-3.35 (m, 1H + 1H), 2.91-3.01 (m, 1H + 1H), 1.42-1.65 (m, 2H + 2H), 1.22-1.41 (m, 4H + 4H), 1.23 (s, 12H + 12H), 1.10-1.22 (m, 4H + 4H), 0.91-1.08 (m, 1H + 1H), 0.69-0.78 (m, 5H + 5H); ^{13}C NMR (100 MHz, $CDCl_3$) δ: 165.6, 165.5, 135.32, 135.27, 134.6, 133.4, 131.6, 130.05, 130.03, 129.30, 129.27, 128.77, 128.76, 128.40, 128.38, 124.2, 124.1, 122.2 (q, J = 285.6 Hz), 91.0 (q, J = 32.9 Hz), 90.9 (q, J = 32.9 Hz), 82.8, 79.10, 79.07, 62.48, 62.46, 53.4, 36.8, 36.5, 36.0, 35.9, 32.56, 32.55, 29.47, 29.46, 26.53, 26.51, 24.7, 23.9, 19.4, 19.2; ^{11}B NMR (126 MHz, $CDCl_3$) δ: 36.1; ^{19}F NMR (369 MHz, $CDCl_3$) δ: –78.0, –78.1; IR (neat, cm^{-1}) ν: 2927, 1747, 1468, 1378, 1195; LRMS (ESI): *m/z* 598 $[M+Na]^+$; HRMS (ESI): *m/z* calcd for $C_{31}H_{41}BF_3NO_5Na$ $[M+Na]^+$ 598.2922, Found 598.2937.

***N*-hydroxy-3-((3-methyl-8-(4,4,5,5-tetramethyl-1,3,2-dioxaborolan-2-yl)octyl)oxy)-3-trifluoromethyl-isoindolinone (1q)**

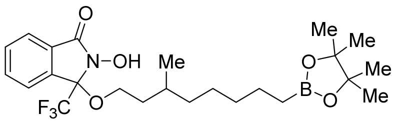

colorless liquid (diastereomixture); ^{1}H NMR (400 MHz, $CDCl_3$) δ: 9.70 (s, 1H), 9.69 (s, 1H), 7.75-7.82 (m, 1H + 1H), 7.55-7.68 (m, 3H + 3H), 3.39-3.50 (m, 1H + 1H), 2.91-3.03 (m, 1H + 1H), 1.47-1.69 (m, 2H + 2H), 1.33-1.47 (m, 3H + 3H), 1.17-1.30 (m, 5H + 5H), 1.23 (s. 12H + 12H), 1.05-1.10 (m, 1H + 1H), 0.70-0.83 (m, 5H + 5H); ^{13}C NMR (100 MHz, $CDCl_3$) δ: 165.8, 135.33, 135.32, 133.2, 131.4, 130.04, 130.01, 123.94, 123.85, 121.9 (q, J = 285.6 Hz), 90.92 (q, J = 32.6 Hz), 90.91 (q, J = 32.6 Hz), 82.93, 82.90, 62.7, 62.6, 36.51, 36.47, 36.1, 36.0, 32.5, 32.3, 29.6, 29.3, 26.5, 26.4, 24.72, 24.69, 23.9, 23.7, 19.5, 19.3; ^{11}B NMR (126 MHz, $CDCl_3$) δ: 36.3; ^{19}F NMR (369 MHz, $CDCl_3$) δ: –78.5; IR (neat, cm^{-1}) ν: 3423, 2927, 1720, 1469, 1378, 1315, 1200; LRMS (ESI): *m/z* 508 $[M+Na]^+$; HRMS (ESI): *m/z* calcd for $C_{24}H_{35}BF_3NO_5Na$ $[M+Na]^+$ 508.2453, Found 508.2443.

***N*-hydroxy-3-((3-hydroxy-3-methyl-8-(4,4,5,5-tetramethyl-1,3,2-dioxaborolan-2-yl)octyl)oxy)-3-trifluoromethyl-isoindolinone (2q)**

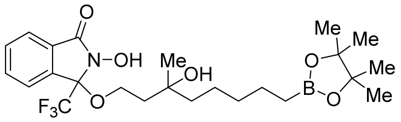

Starting material **1q** (38.8 mg, 0.08 mmol) was oxygenated under condition B of section 2, and **2q** (18.2 mg, 0.036 mmol) was obtained as a diastereomixture according to the purification procedure described in the General method (45% yield).

colorless liquid (diastereomixture); ^{1}H NMR (400 MHz, $CDCl_3$) δ: 9.45 (brs, 1H), 9.40 (brs, 1H), 7.84-7.90 (m, 1H + 1H), 7.58-7.69 (m, 3H + 3H), 3.46-3.57 (m, 1H + 1H), 3.23-3.33 (m, 1H + 1H), 3.03 (brs, 1H), 2.94 (brs, 1H), 1.84-2.02 (m, 2H + 2H), 1.56-1.70 (m, 2H + 2H), 1.22-1.52 (m, 6H + 6H), 1.33 (s, 3H), 1.24 (s, 12H), 1.23 (s, 12H), 1.16 (s, 3H), 0.71-0.80 (m, 2H + 2H); ^{13}C NMR (126 MHz, $CDCl_3$) δ: 164.53, 164.48, 134.43, 134.41, 132.9, 131.6, 130.5, 124.2, 124.1, 124.0, 121.90 (q, J = 286.7 Hz), 121.86 (q, J = 286.7 Hz), 90.9 (q, J = 32.4 Hz), 90.8 (q, J = 33.6 Hz), 82.92, 82.90, 72.9, 72.7, 60.62, 60.59, 43.0, 42.8, 39.3, 39.2, 32.64, 32.59, 29.2, 27.3, 26.7, 24.8, 24.7, 23.8, 23.72, 23.70, 23.5; ^{11}B NMR (126 MHz, $CDCl_3$) δ: 35.8; ^{19}F NMR (369 MHz, $CDCl_3$) δ: –78.1; IR (neat, cm^{-1}) ν: 3423, 2933, 1723, 1469, 1374, 1315, 1199; LRMS (ESI): *m/z* 524 $[M+Na]^+$; HRMS (ESI): *m/z* calcd for $C_{24}H_{35}BF_3NO_6Na$ $[M+Na]^+$ 524.2402, Found 524.2407.

2-((2*R*)-1-((*N*-(benzyloxy)-3-oxo-1-(trifluoromethyl)isoindolin-1-yl)oxy)-3-methylbutan-2-yl)isoindoline-1,3-dione (S4r)

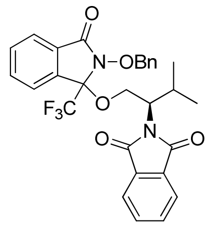

colorless sticky liquid (diastereomixture); ^{1}H NMR (400 MHz, $CDCl_3$) δ: 7.85-7.93 (m, 2H + 2H), 7.62-7.80 (m, 5H + 5H), 7.53-7.62 (m, 2H + 2H), 7.27-7.46 (m, 4H + 4H), 5.30 (d, 1H, *J* = 10.1 Hz), 5.14 (d, 1H, *J* = 10.1 Hz), 5.10 (d, 1H, *J* = 9.9 Hz), 4.96 (d, 1H, *J* = 9.9 Hz), 4.00-4.15 (m, 1H + 1H), 3.90-3.96 (m, 1H), 3.60-3.66 (m, 1H), 3.52-3.57 (m, 1H), 3.33-3.38 (m, 1H), 2.22-2.34 (m, 1H), 2.12-2.22 (m, 1H), 0.75-0.86 (m, 6H); ^{13}C NMR (126 MHz, $CDCl_3$) δ: 168.39, 168.37, 165.5, 165.0, 134.51, 134.49, 134.3, 134.2, 134.0, 133.8, 133.52, 133.50, 131.9, 131.8, 131.5, 131.4, 129.9, 129.7, 129.2, 129.1, 128.8, 128.5, 128.4, 128.2, 124.4, 124.3, 124.2, 124.1, 123.1, 123.0, 121.8 (q, *J* = 286.3 Hz), 90.8 (q, *J* = 33.2 Hz), 90.7 (q, *J* = 33.2 Hz), 79.1, 79.0, 62.5, 61.8, 57.2, 56.7, 27.8, 27.4, 20.0, 19.9, 19.7, 19.6; ^{19}F NMR (369 MHz, $CDCl_3$) δ: –78.0, –78.3; IR (neat, cm^{-1}) ν: 1747, 1711, 1647, 1468, 1389, 1189; LRMS (ESI): *m/z* 561 $[M+Na]^+$; HRMS (ESI): *m/z* calcd for $C_{29}H_{25}F_3N_2O_5Na$ $[M+Na]^+$ 561.1608, Found 561.1583.

2-((2*R*)-1-((2-hydroxy-3-oxo-1-(trifluoromethyl)isoindolin-1-yl)oxy)-3-methylbutan-2-yl)isoindoline-1,3-dione (1r)

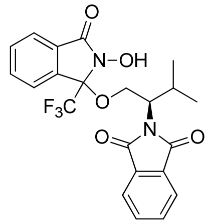

white solid (diastereomixture); ^{1}H NMR (500 MHz, $CDCl_3$) δ: 9.04 (brs, 1H), 8.09 (brs, 1H), 7.82-7.89 (m, 3H + 3H), 7.72-7.79 (m, 2H + 2H), 7.57-7.72 (m, 2H + 2H), 7.52-7.57 (m, 1H), 7.30 (d, 1H, *J* = 7.5 Hz), 4.14-4.25 (m, 1H + 1H), 4.08 (dd, 1H, *J* = 9.4 Hz, 9.4 Hz), 3.73-3.77 (m, 1H + 1H), 3.35-3.39 (m, 1H), 2.16-2.33 (m, 1H + 1H), 0.80-0.95 (m, 6H + 6H); ^{13}C NMR (126 MHz, $CDCl_3$) δ: 169.2, 168.7, 165.4, 165.0, 134.4, 134.3, 134.14, 134.07, 133.3, 133.2, 131.8, 131.7, 131.5, 130.0, 129.9, 124.2, 124.1, 124.0, 123.29, 123.26, 121.5 (q, *J* = 286.3 Hz), 90.8 (q, *J* = 33.0 Hz), 90.7 (q, *J* = 33.6 Hz), 62.3, 61.9, 57.1, 57.0, 28.0, 27.7, 20.0, 19.9, 19.7; ^{19}F NMR (369 MHz, $CDCl_3$) δ: –78.3, –78.4; IR (KBr, cm^{-1}) ν: 3219, 2968, 1713, 1469, 1390, 1200; LRMS (ESI): *m/z* 471 $[M+Na]^+$; HRMS (ESI): *m/z* calcd for $C_{22}H_{19}F_3N_2O_5Na$ $[M+Na]^+$ 471.1138, Found 471.1120.

2-((2*S*)-3-hydroxy-1-((*N*-hydroxy-3-oxo-1-(trifluoromethyl)isoindolin-1-yl)oxy)-3-methylbutan-2-yl)isoindoline-1,3-dione (2r)

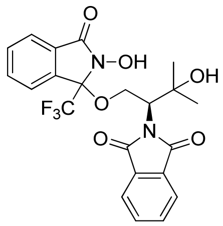

Starting material **1r** (89.7 mg, 0.2 mmol) was oxygenated under condition B of section 2, and **2r** (37.5 mg, 0.081 mmol) was obtained as a diastereomixture according to the purification procedure described in the General method (40% yield).

white solid (diastereomixture); ^{1}H NMR (500 MHz, $CDCl_3$; underlined chemical shifts for one isomer) δ: 9.05 (brs, 1H), 9.05 (brs, 1H), 7.90-7.95 (m, 2H), 7.83-7.88 (m, 1H), 7.83-7.88 (m, 3H), 7.76-7.80 (m, 2H), 7.76-7.80 (m, 2H), 7.61-7.69 (m, 3H), 7.52-7.61 (m, 2H), 7.37 (d, 1H, *J* = 7.5 Hz), 4.66 (brs, 1H), 4.59 (dd, 1H, *J* = 7.5 Hz, 7.5 Hz), 4.40 (dd, 1H, *J* = 3.7 Hz, 8.5 Hz), 4.28 (brs, 1H), 3.94 (dd, 1H, *J* = 7.5 Hz, 9.5 Hz), 3.91 (dd, 1H, *J* = 8.5 Hz, 10.7 Hz), 3.53 (dd, 1H, *J* = 3.7 Hz, 10.7 Hz), 3.45 (dd, 1H, *J* = 7.5 Hz, 9.5 Hz), 1.55 (s, 3H), 1.25 (s, 3H), 1.18 (s, 3H), 1.16 (s, 3H); ^{13}C NMR (126 MHz, $CDCl_3$) δ: 164.6, 164.0, 134.8, 134.5, 134.2, 133.7, 133.2, 133.0, 131.9, 131.8, 131.5, 131.2, 130.5, 130.1, 124.4, 124.1, 123.9, 123.8, 121.7 (q, *J* = 287.1 Hz), 121.5 (q, *J* = 286.7 Hz), 90.8 (q, *J* = 33.6 Hz), 90.7 (q, *J* = 33.2 Hz), 72.0, 71.5, 60.8, 60.4, 59.6, 58.1, 28.4, 27.98, 27.96, 27.6; ^{19}F NMR (369 MHz, $CDCl_3$) δ: –77.8, –78.3; IR (KBr, cm^{-1}) ν: 3449, 2979, 1704, 1396, 1199; LRMS (ESI): *m/z* 487 [M+Na]$^+$; HRMS (ESI): *m/z* calcd for $C_{22}H_{19}F_3N_2O_6Na$ [M+Na]$^+$ 487.1087, Found 487.1083.

***N*-benzyloxy-3-(3-phenylpropoxy)-3-trifluoromethyl-isoindolinone (S4s)**

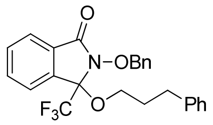

light yellow liquid; ^{1}H NMR (500 MHz, $CDCl_3$) δ: 7.89 (dd, 1H, *J* = 6.9 Hz, 1.2 Hz), 7.62-7.68 (m, 2H), 7.56-7.59 (m, 3H), 7.36-7.44 (m, 3H), 7.19-7.25 (m, 2H), 7.13-7.18 (m, 1H), 7.09-7.12 (m, 2H), 5.31 (d, *J* = 9.8 Hz), 5.14 (d, *J* = 9.8 Hz), 3.29 (dt, *J* = 9.2 Hz, 5.8 Hz), 2.96 (dt, *J* = 9.2 Hz, 5.8 Hz), 2.60-2.71 (m, 2H), 1.78-1.92 (m, 2H); ^{13}C NMR (126 MHz, $CDCl_3$) δ: 165.6, 141.1, 135.2, 134.6, 133.4, 131.6, 130.0, 129.4, 128.9, 128.5, 128.4, 128.3, 125.9, 124.2, 124.1, 122.2 (q, *J* = 285.5 Hz), 90.9 (q, *J* = 33.6 Hz), 79.1, 63.1, 31.8, 30.5; ^{19}F NMR (369 MHz, $CDCl_3$) δ: –78.0; IR (neat, cm^{-1}) ν: 2950, 1744, 1468, 1190; LRMS (ESI): *m/z* 464 [M+Na]$^+$; HRMS (ESI): *m/z* calcd for $C_{25}H_{22}F_3NO_3Na$ [M+Na]$^+$ 464.1444, Found 464.1450.

***N*-hydroxy-3-(3-phenylpropoxy)-3-trifluoromethyl-isoindolinone (1s)**

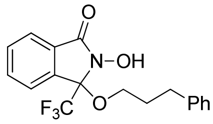

light yellow liquid; ^{1}H NMR (500 MHz, $CDCl_3$) δ: 10.12 (brs, 1H), 7.80 (d, 1H, *J* = 7.5 Hz), 7.67 (dd, 1H, *J* = 7.5 Hz, 7.5 Hz), 7.59-7.63 (m, 2H), 7.30 (dd, 2H, *J* = 7.5 Hz, 7.5 Hz), 7.19-7.23 (m, 3H), 3.35 (dt, 1H, *J*

= 8.6 Hz, 5.8 Hz), 2.95-3.05 (m, 1H), 2.67-2.77 (m, 2H), 1.90-1.98 (m, 2H); ^{13}C NMR (126 MHz, $CDCl_3$) δ:165.9, 141.3, 135.0, 133.2, 131.5, 129.8, 128.4, 128.3, 125.8, 124.0, 123.8, 121.9 (q, J = 285.5 Hz), 91.0 (q, J = 33.6 Hz), 63.4, 31.3, 30.5; ^{19}F NMR (369 MHz, $CDCl_3$) δ: –78.3; IR (neat, cm^{-1}) ν: 3144, 2948, 1717, 1469, 1305, 1200; LRMS (ESI): *m/z* 374 $[M+Na]^+$; HRMS (ESI): *m/z* calcd for $C_{18}H_{16}F_3NO_3Na$ $[M+Na]^+$ 374.0975, Found 374.0990.

***N*-hydroxy-3-(3-oxo-3-phenylpropoxy)-3-trifluoromethyl-isoindolinone (2s)**

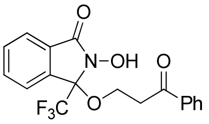

Starting material **1s** (35.1 mg, 0.1 mmol) was oxygenated under condition B of section 2, and **2s** (32.4 mg, 0.089 mmol) was isolated according to the purification procedure described in the General method (89% yield).

light yellow liquid; ^{1}H NMR (500 MHz, $CDCl_3$) δ: 8.91 (brs, 1H), 7.98 (d, 2H, J = 7.5 Hz), 7.89 (d, 1H, J = 8.0 Hz), 7.66-7.70 (m, 1H), 7.59-7.66 (m, 3H), 7.49 (dd, 2H, J = 7.5 Hz), 3.77 (dt, 1H, J = 3.1 Hz, 9.6 Hz), 3.54 (ddd, 1H, J = 18.3 Hz, 9.6 Hz, 3.1 Hz), 3.43 (dt, 1H, J = 9.6 Hz, 3.1 Hz), 3.16 (dt, 1H, J = 18.3 Hz, 3.1 Hz); ^{13}C NMR (100 MHz, $CDCl_3$) δ: 198.8, 163.6, 135.9, 134.1, 133.9, 132.9, 131.7, 130.6, 128.8, 128.3, 124.2, 124.1, 121.8 (q, J = 286.6 Hz), 90.5 (q, J = 32.9 Hz), 57.9, 37.2, 29.2; ^{19}F NMR (369 MHz, $CDCl_3$) δ: –78.1; IR (neat, cm^{-1}) ν: 3220, 2952, 1718, 1197; LRMS (ESI): *m/z* 388 $[M+Na]^+$; HRMS (ESI): *m/z* calcd for $C_{18}H_{14}F_3NO_4Na$ $[M+Na]^+$ 388.0767, Found 388.0759.

4-(3-((2-benzyloxy-3-oxo-1-(trifluoromethyl)isoindolin-1-yl)oxy)propyl)phenyl acetate (S4t)

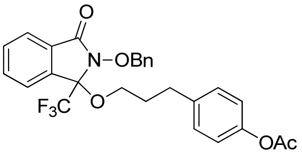

light yellow liquid; ^{1}H NMR (500 MHz, $CDCl_3$) δ: 7.88 (d, 1H, J = 7.4 Hz), 7.60-7.70 (m, 2H), 7.54-7.58 (m, 3H), 7.34-7.44 (m, 3H), 7.09 (d, 2H, J = 8.6 Hz), 6.92 (d, 2H, J = 8.6 Hz), 5.32 (d, 1H, J = 9.7 Hz), 5.14 (d, 1H, J = 9.7 Hz), 3.27 (dt, 1H, J = 8.6 Hz, 6.3 Hz), 2.92-2.98 (m, 1H), 2.64 (t, 2H, J = 7.7 Hz), 2.28 (s, 3H), 1.76-1.89 (m, 2H); ^{13}C NMR (126 MHz, $CDCl_3$) δ: 169.5, 165.6, 148.8, 138.6, 135.1, 134.6, 133.5, 131.7, 129.9, 129.4, 129.3, 128.8, 128.4, 124.2, 124.1, 122.2 (q, J = 285.5 Hz), 121.3, 90.9 (q, J = 33.2 Hz), 79.1, 63.0, 31.1, 30.3, 21.0; ^{19}F NMR (369 MHz, $CDCl_3$) δ: –78.0; IR (neat, cm^{-1}) ν: 2949, 1747, 1194; LRMS (ESI): *m/z* 522 $[M+Na]^+$; HRMS (ESI): *m/z* calcd for $C_{27}H_{24}F_3NO_5Na$ $[M+Na]^+$ 522.1499, Found 522.1492.

4-(3-((2-hydroxy-3-oxo-1-(trifluoromethyl)isoindolin-1-yl)oxy)propyl)phenyl acetate (1t)

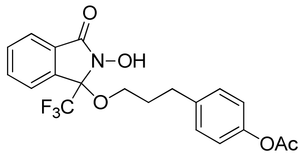

reddish sticky liquid; ^{1}H NMR (500 MHz, $CDCl_3$) δ: 9.78 (brs, 1H), 7.77 (d, 1H, J = 7.4 Hz), 7.62-7.66 (m, 1H), 7.53-7.60 (m, 2H), 7.17 (d, 2H, J = 8.6 Hz), 6.98 (d, 2H, J = 8.6 Hz), 3.40-3.46 (m, 1H), 2.97-3.05 (m, 1H), 2.68-2.78 (m, 2H), 2.28 (s, 3H), 1.85-2.01 (m, 2H); ^{13}C NMR (126 MHz, $CDCl_3$) δ: 169.7, 165.8, 148.8, 138.9, 135.0, 133.3, 131.5, 129.9, 129.3, 124.0, 123.9, 121.9 (q, J = 286.7 Hz), 121.3, 91.0 (q, J = 32.8 Hz), 63.2, 31.2, 30.4, 21.0; ^{19}F NMR (369 MHz, $CDCl_3$) δ: –78.4; IR (neat, cm^{-1}) ν: 3425, 1719, 1645, 1196; LRMS (ESI): *m/z* 432 $[M+Na]^+$; HRMS (ESI): *m/z* calcd for $C_{20}H_{18}F_3NO_5Na$ $[M+Na]^+$ 432.1029, Found 432.1011.

4-(3-((2-hydroxy-3-oxo-1-(trifluoromethyl)isoindolin-1-yl)oxy)propanoyl)phenyl acetate (2t)

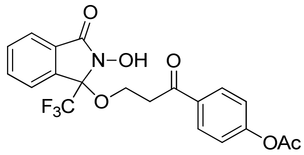

Starting material **1t** (53.8 mg, 0.13 mmol) was oxygenated under condition A of section 2, and **2t** (30.0 mg, 0.071 mmol) was isolated according to the purification procedure described in the General method (54% yield).

white solid; ^{1}H NMR (500 MHz, $CDCl_3$) δ: 8.97 (brs, 1H), 8.00 (dd, 2H, J = 8.6 Hz, 1.1 Hz), 7.86 (d, 1H, J = 8.0 Hz), 7.60-7.69 (m, 3H), 7.21 (dd, 2H, J = 8.6 Hz, 1.1 Hz), 3.72-3.78 (m, 1H), 3.42-3.51 (m, 2H), 3.13-3.22 (m, 1H), 2.32 (s, 3H); ^{13}C NMR (100 MHz, $CDCl_3$) δ: 197.5, 168.7, 163.8, 155.0, 134.0, 133.6, 133.0, 131.7, 130.5, 130.0, 124.2, 124.1, 122.0, 121.7 (q, J = 286.6 Hz), 90.5 (q, J = 33.2 Hz), 58.0, 37.3, 21.1; ^{19}F NMR (369 MHz, $CDCl_3$) δ: –78.1; IR (neat, cm^{-1}) ν: 3243, 1719, 1195; LRMS (ESI): *m/z* 446 $[M+Na]^+$; HRMS (ESI): *m/z* calcd for $C_{20}H_{16}F_3NO_6Na$ $[M+Na]^+$ 446.0822, Found 446.0825.

***N*-benzyloxy-3-(3-(4-methoxyphenyl)propoxy)- 3-trifluoromethyl-isoindolinone (S4u)**

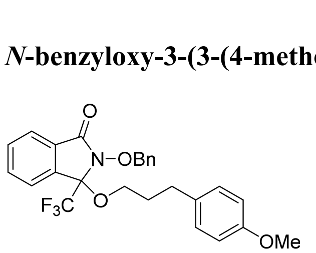

colorless liquid; ^{1}H NMR (500 MHz, $CDCl_3$) δ: 7.89 (dd, 1H, J = 6.3 Hz, 1.1 Hz), 7.60-7.69 (m, 2H), 7.55-7.60 (m, 3H), 7.35-7.44 (m, 3H), 7.00-7.04 (m, 2H), 6.72-6.67 (m, 2H), 5.31 (d, 1H, J = 9.7 Hz), 5.13 (d, 1H, J = 9.7 Hz), 3.75 (s, 3H), 3.25-3.32 (m, 1H), 2.92-2.99 (m, 1H), 2.53-2.65 (m, 2H), 1.76-1.89 (m, 2H); ^{13}C NMR (126 MHz, $CDCl_3$) δ: 165.5, 157.8, 135.2, 134.6, 133.4, 133.1, 131.6, 130.0, 129.4, 129.2, 128.8, 128.4, 124.2, 124.1, 122.2 (q, J = 285.9 Hz), 113.7, 90.9 (q, J = 32.8 Hz), 79.1, 63.0, 55.1, 30.8, 30.7; ^{19}F NMR (369 MHz, $CDCl_3$) δ: –78.0; IR (neat, cm^{-1}) ν: 2952, 1745, 1613, 1513, 1468, 1188; LRMS

(ESI): *m/z* 494 $[M+Na]^+$; HRMS (ESI): *m/z* calcd for $C_{26}H_{24}F_3NO_4Na$ $[M+Na]^+$ 494.1550, Found 494.1547.

***N*-hydroxy-3-(3-(4-methoxyphenyl)propoxy)-3-trifluoromethyl-isoindolinone (1u)**

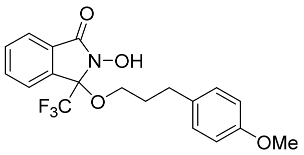

colorless liquid; ^{1}H NMR (400 MHz, $CDCl_3$) δ: 9.70 (brs, 1H), 7.74-7.79 (m, 1H), 7.55-7.67 (m, 3H), 7.05-7.12 (m, 2H), 6.78-6.85 (m, 2H), 3.78 (s, 3H), 3.40-3.47 (m, 1H), 2.97-3.05(m, 1H), 2.60-2.74 (m, 2H), 1.83-2.01 (m, 2H); ^{13}C NMR (126 MHz, $CDCl_3$) δ: 165.8, 157.8, 135.1, 133.4, 133.2, 131.5, 129.9, 129.3, 124.1, 123.9, 121.9 (q, J = 286.7 Hz), 113.8, 91.0 (q, J = 32.8 Hz), 63.4, 55.2, 30.9, 30.8; ^{19}F NMR (369 MHz, $CDCl_3$) δ: –78.4; IR (neat, cm^{-1}) ν: 3422, 3173, 2951, 1717, 1613, 1513, 1301, 1191; LRMS (ESI): *m/z* 404 $[M+Na]^+$; HRMS (ESI): *m/z* calcd for $C_{19}H_{18}F_3NO_4Na$ $[M+Na]^+$ 404.1080, Found 404.1074.

***N*-hydroxy-3-(3-(4-methoxyphenyl)-3-oxopropoxy)-3-trifluoromethyl-isoindolinone (2u)**

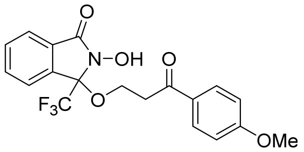

Starting material **1u** (38.1 mg, 0.1 mmol) was oxygenated under condition B of section 2, and **2u** (27.7 mg, 0.070 mmol) was isolated according to the purification procedure described in the General method (70% yield).

light yellow solid; ^{1}H NMR (500 MHz, $CDCl_3$) δ: 9.03 (brs, 1H), 7.95 (d, 2H, J = 8.8 Hz), 7.90 (d, 1H, J = 6.9 Hz), 7.61-7.69 (m, 3H), 6.94 (d, 2H, J = 8.8 Hz), 3.87 (s, 3H), 3.74 (td, 1H, J = 9.7 Hz, 2.3 Hz), 3.43-3.52 (m, 1H), 3.37-3.43 (m, 1H), 3.04-3.11 (m, 1H); ^{13}C NMR (126 MHz, $CDCl_3$) δ: 197.3, 164.4, 163.8, 134.0, 132.8, 131.7, 130.8, 129.0, 128.4, 124.2, 124.0, 121.8 (q, J = 287.1 Hz), 114.0, 90.5 (q, J = 33.2 Hz), 57.8, 55.5, 36.7; ^{19}F NMR (369 MHz, $CDCl_3$) δ: –78.1; IR (KBr, cm^{-1}) ν: 3152, 2967, 1739, 1659, 1600, 1184; LRMS (ESI): *m/z* 418 $[M+Na]^+$; HRMS (ESI): *m/z* calcd for $C_{19}H_{16}F_3NO_5Na$ $[M+Na]^+$ 418.0873, Found 418.0874.

3-(3-(4-bromophenyl)propoxy)-*N*-((4-methoxybenzyl)oxy)-3-trifluoromethyl-isoindolinone (S4'v)

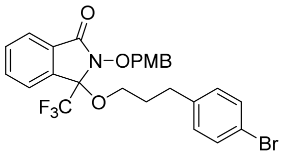

colorless liquid; ^{1}H NMR (400 MHz, $CDCl_3$) δ: 7.85-7.90 (m, 1H), 7.60-7.70 (m, 2H), 7.56 (d, 1H, J = 7.2

Hz), 7.47-7.52 (m, 2H), 7.25-7.30 (m, 2H), 6.92-6.97 (m, 4H), 5.26 (d, 1H, *J* = 9.2 Hz), 5.04 (d, 1H, *J* = 9.2 Hz), 3.82 (s, 3H), 3.20-3.28 (m, 1H), 2.87-2.95 (m, 1H), 2.52-2.66 (m, 2H), 1.75-1.84 (m, 2H); ^{13}C NMR (100 MHz, $CDCl_3$) δ: 165.5, 160.1, 140.0, 135.1, 133.4, 131.7, 131.28, 131.25, 130.2, 130.0, 126.7, 124.11, 124.05, 122.2 (q, *J* = 285.6 Hz), 119.6, 113.8, 90.7 (q, *J* = 32.9 Hz), 78.8, 62.7, 55.2, 31.1, 30.2; ^{19}F NMR (369 MHz, $CDCl_3$) δ: –78.1; IR (neat, cm^{-1}) ν: 2953, 1742, 1613, 1545, 1253, 1189; LRMS (ESI): *m/z* 572 $[M+Na]^+$; HRMS (ESI): *m/z* calcd for $C_{26}H_{23}BrF_3NO_4Na$ $[M+Na]^+$ 572.0655, Found 572.0671.

3-(3-(4-bromophenyl)propoxy)-*N*-hydroxy-3-trifluoromethyl-isoindolinone (1v)

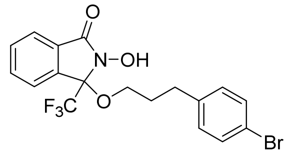

light orange gum; ^{1}H NMR (400 MHz, $CDCl_3$) δ: 9.80 (s, 1H), 7.75 (d, 1H, *J* = 7.2 Hz), 7.53-7.68 (m, 3H), 7.37 (d, 2H, *J* = 8.1 Hz), 7.05 (d, 2H, *J* = 8.1 Hz), 3.37-3.46 (m, 1H), 2.94-3.04 (m, 1H), 2.63-2.76 (m, 2H), 1.83-2.00 (m, 2H); ^{13}C NMR (100 MHz, $CDCl_3$) δ: 165.8, 140.3, 135.0, 133.3, 131.6, 131.4, 130.2, 129.8, 124.0, 123.9, 121.9 (q, *J* = 286.6 Hz), 119.6, 90.9 (q, *J* = 33.2 Hz), 63.2, 31.3, 30.4; ^{19}F NMR (369 MHz, $CDCl_3$) δ: –78.4; IR (neat, cm^{-1}) ν: 3144, 2950, 1717, 1488, 1305, 1200; LRMS (ESI): *m/z* 452 $[M+Na]^+$; HRMS (ESI): *m/z* calcd for $C_{18}H_{15}BrF_3NO_3Na$ $[M+Na]^+$ 452.0080, Found 452.0098.

3-(3-(4-bromophenyl)-3-oxopropoxy)-*N*-hydroxy-3-trifluoromethyl-isoindolinone (2v)

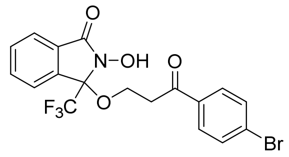

Starting material **1v** (17.2 mg, 0.04 mmol) was oxygenated under condition B of section 2, and **2v** (8.3 mg, 0.019 mmol) was isolated according to the purification procedure described in the General method (47% yield).

light yellow solid; ^{1}H NMR (500 MHz, $CDCl_3$) δ: 8.48 (brs, 1H), 7.93 (dd, 1H, *J* = 1.1 Hz, 6.3 Hz), 7.85 (d, 2H, *J* = 8.0 Hz), 7.61-7.70 (m, 5H), 3.72-3.79 (m, 1H), 3.47-3.56 (m, 1H), 3.38-3.43 (m, 1H), 3.04-3.11 (m, 1H); ^{13}C NMR (126 MHz, $CDCl_3$) δ: 197.7, 164.0, 134.8, 134.0, 133.0, 132.1, 131.7, 130.4, 129.8, 129.4, 124.2, 124.1, 121.7 (q, *J* = 285.9 Hz), 90.6 (q, *J* = 32.8 Hz), 58.1, 37.3; ^{19}F NMR (369 MHz, $CDCl_3$) δ: –78.1; IR (KBr, cm^{-1}) ν: 3220, 2950, 1720, 1678, 1586, 1469, 1398, 1278, 1120; LRMS (ESI): *m/z* 466 $[M+Na]^+$; HRMS (ESI): *m/z* calcd for $C_{18}H_{13}BrF_3NO_4Na$ $[M+Na]^+$ 465.9872, Found 465.9855.

***N*-benzyloxy-3-(3-(4-(4,4,5,5-tetramethyl-1,3,2-dioxaborolan-2-yl)phenyl)propoxy)-3-trifluoromethyl-isoindolinone (S4w)**

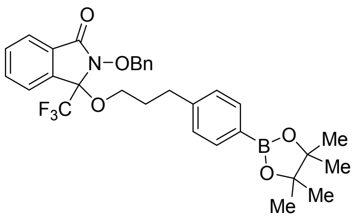

colorless liquid; ^{1}H NMR (400 MHz, $CDCl_3$) δ: 7.89 (dd, 1H, J = 6.5 Hz, 1.6 Hz), 7.60-7.71 (m, 4H), 7.54-7.60 (m, 3H), 7.35-7.46 (m, 3H), 7.12 (d, 2H, J = 8.1 Hz), 5.31 (d, 1H, J = 9.9 Hz), 5.14 (d, 1H, J = 9.9 Hz), 3.24-3.33 (m, 1H), 2.92-3.00 (m, 1H), 2.58-2.73 (m, 2H), 1.74-1.95 (m, 2H), 1.35 (s, 12H); ^{13}C NMR (100 MHz, $CDCl_3$) δ: 165.6, 144.5, 135.1, 134.9, 134.6, 133.4, 131.6, 130.0, 129.3, 128.8, 128.4, 127.8, 124.2, 124.1, 122.2 (q, J = 286.1 Hz), 90.9 (q, J = 33.0 Hz), 83.6, 79.1, 63.1, 32.0, 30.3, 24.8; ^{11}B NMR (126 MHz, $CDCl_3$) δ: 32.8; ^{19}F NMR (369 MHz, $CDCl_3$) δ: –78.0; IR (neat, cm^{-1}) ν: 2978, 1746, 1612, 1469, 1362, 1195; LRMS (ESI): *m/z* 590 $[M+Na]^+$; HRMS (ESI): *m/z* calcd for $C_{31}H_{33}BF_3NO_5Na$ $[M+Na]^+$ 590.2296, Found 590.2293.

***N*-hydroxy-3-(3-(4-(4,4,5,5-tetramethyl-1,3,2-dioxaborolan-2-yl)phenyl)propoxy)-3-trifluoromethyl -isoindolinone (1w)**

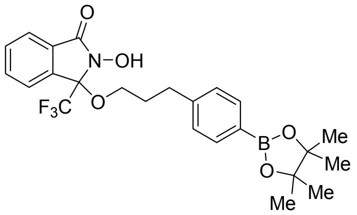

white solid; ^{1}H NMR (400 MHz, $CDCl_3$) δ: 9.79 (s, 1H), 7.76 (d, 1H, J = 6.7 Hz), 7.73 (d, 2H, J = 8.1 Hz), 7.54-7.67 (m, 3H), 7.18 (d, 2H, J = 8.1 Hz), 3.40-3.49 (m, 1H), 2.96-3.06 (m, 1H), 2.67-2.82 (m, 2H), 1.85-2.05 (m, 2H), 1.35 (s, 12H); ^{13}C NMR (100 MHz, $CDCl_3$) δ: 165.8, 144.8, 135.0, 134.9, 133.2, 131.5, 129.9, 127.9, 124.0, 123.9, 121.9 (q, J = 286.6 Hz), 91.0 (q, J = 33.2 Hz), 83.7, 63.4, 32.1, 30.4, 24.8; ^{11}B NMR (126 MHz, $CDCl_3$) δ: 33.3; ^{19}F NMR (369 MHz, $CDCl_3$) δ: –78.4; IR (KBr, cm^{-1}) ν: 3177, 2979, 1719, 1613, 1470, 1362, 1200; LRMS (ESI): *m/z* 500 $[M+Na]^+$; HRMS (ESI): *m/z* calcd for $C_{24}H_{27}BF_3NO_5Na$ $[M+Na]^+$ 500.1827, Found 500.1802.

***N*-hydroxy-3-(3-oxo-3-(4-(4,4,5,5-tetramethyl-1,3,2-dioxaborolan-2-yl)phenyl)propoxy)-3-trifluoro methyl-isoindolinone (2w)**

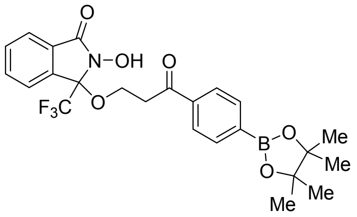

Starting material **1w** (19.1 mg, 0.04 mmol) was oxygenated under condition A of section 2, and **2w** (4.9 mg, 0.010 mmol) was isolated according to the purification procedure described in the General method

(25% yield).

light yellow solid; ^{1}H NMR (500 MHz, $CDCl_3$) δ: 8.60 (s, 1H), 7.89-7.97 (m, 5H), 7.61-7.70 (m, 3H), 3.74-3.80 (m, 1H), 3.53-3.61 (m, 1H), 3.38-3.43 (m, 1H), 3.08-3.16 (m, 1H), 1.36 (s, 12H); ^{13}C NMR (100 MHz, $CDCl_3$) δ: 199.1, 163.6, 137.8, 135.0, 133.9, 132.9, 131.7, 130.7, 127.3, 124.3, 124.1, 121.8 (q, J = 285.6 Hz), 90.5 (q, J = 33.5 Hz), 84.3, 57.8, 37.4, 24.8; ^{11}B NMR (126 MHz, $CDCl_3$) δ: 32.9; ^{19}F NMR (369 MHz, $CDCl_3$) δ: –78.1; IR (neat, cm^{-1}) ν: 3426, 2979, 1722, 1508, 1469, 1360, 1199; LRMS (ESI): *m/z* 514 $[M+Na]^+$; HRMS (ESI): *m/z* calcd for $C_{24}H_{25}BF_3NO_6Na$ $[M+Na]^+$ 514.1619, Found 514.1621.

3-(3-(2-iodophenyl)propoxy)-*N*-((4-methoxybenzyl)oxy)-3-trifluoromethyl-isoindolinone (S4'x)

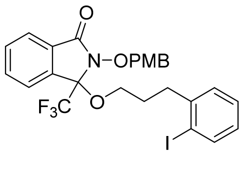

yellow liquid; ^{1}H NMR (500 MHz, $CDCl_3$) δ: 7.89-7.76 (d, 1H, J = 7.4 Hz), 7.61-7.71 (m, 3H), 7.48-7.53 (m, 2H), 7.11-7.18 (m, 2H), 6.90-6.94 (m, 2H), 6.82-6.87 (m, 1H), 5.26 (d, 1H), J = 9.2 Hz), 5.06 (d, 1H), J = 9.2 Hz), 3.81 (s, 3H), 3.29-3.36 (m, 1H), 2.96-3.02 (m, 1H), 2.67-2.78 (m, 2H), 1.78-1.87 (m, 2H); ^{13}C NMR (126 MHz, $CDCl_3$) δ: 165.5, 160.1, 143.7, 139.5, 135.2, 133.4, 131.7, 131.2, 130.1, 129.4, 128.2, 127.8, 126.8, 124.24, 124.16, 122.2 (q, J = 286.7 Hz), 113.9, 100.4, 90.9 (q, J = 32.8 Hz), 78.9, 63.1, 55.3, 36.9, 29.3; ^{19}F NMR (369 MHz, $CDCl_3$) δ: –78.0; IR (neat, cm^{-1}) ν: 1742, 1613, 1515, 1467, 1252, 1187; LRMS (ESI): *m/z* 620 $[M+Na]^+$; HRMS (ESI): *m/z* calcd for $C_{26}H_{23}F_3INO_4Na$ $[M+Na]^+$ 620.0516, Found 620.0514.

***N*-hydroxy-3-(3-(2-iodophenyl)propoxy)-3-trifluoromethyl-isoindolinone (1x)**

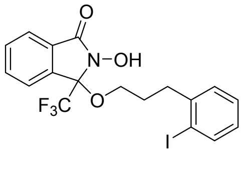

light yellow liquid; ^{1}H NMR (400 MHz, $CDCl_3$) δ: 9.81 (brs, 1H), 7.79 (d, 2H, J = 7.6 Hz), 7.56-7.69 (m, 3H), 7.23-7.29 (m, 2H), 6.83-6.91 (m, 1H), 3.45-3.54 (m, 1H), 3.02-3.10 (m, 1H), 2.76-2.90 (m, 2H), 1.87-2.02 (m, 2H); ^{13}C NMR (100 MHz, $CDCl_3$) δ: 165.7, 143.8, 139.5, 135.1, 133.3, 131.6, 129.9, 129.6, 128.4, 127.9, 124.1, 124.0, 121.9 (q, J = 286.6 Hz), 100.4, 91.0 (q, J = 33.2 Hz), 63.4, 37.0, 29.3; ^{19}F NMR (369 MHz, $CDCl_3$) δ: –78.4; IR (neat, cm^{-1}) ν: 3143, 2943, 1716, 1468, 1304, 1192; LRMS (ESI): *m/z* 500 $[M+Na]^+$; HRMS (ESI): *m/z* calcd for $C_{18}H_{15}F_3INO_3Na$ $[M+Na]^+$ 499.9941, Found 499.9943.

***N*-hydroxy-3-(3-(2-iodophenyl)-3-oxopropoxy)-3-trifluoromethyl-isoindolinone (2x)**

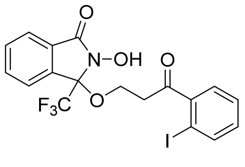

Starting material **1x** (23.9 mg, 0.05 mmol) was oxygenated under condition B of section 2, and **2x** (13.0 mg, 0.026 mmol) was isolated according to the purification procedure described in the General method (53% yield).

light yellow liquid; ^{1}H NMR (500 MHz, $CDCl_3$) δ: 8.77 (brs, 1H), 7.96 (d, 1H, J = 7.7 Hz), 7.88 (d, 1H, J = 6.9 Hz), 7.60-7.70 (m, 3H), 7.50 (dd, 1H, J = 7.7 Hz, 1.5 Hz), 7.44 (t, 1H, J = 7.7 Hz), 7.16 (td, 1H, J = 7.7 Hz, 1.5 Hz), 3.79 (td, 1H, J = 8.9 Hz, 4.0 Hz), 3.42-3.51 (m, 1H), 3.31-3.42 (m, 1H), 3.13-3.24 (m, 1H); ^{13}C NMR (126 MHz, $CDCl_3$) δ: 202.7, 164.3, 142.8, 141.1, 134.1, 133.1, 132.4, 131.8, 130.4, 128.4, 128.2, 124.2, 121.8 (q, J = 286.7 Hz), 91.1, 90.7 (q, J = 33.6 Hz), 58.4, 40.6; ^{19}F NMR (369 MHz, $CDCl_3$) δ: –78.0; IR (neat, cm^{-1}) ν: 3166, 2926, 1715, 1468, 1192; LRMS (ESI): *m/z* 514 $[M+Na]^+$; HRMS (ESI): *m/z* calcd for $C_{18}H_{13}F_3INO_4Na$ $[M+Na]^+$ 513.9734, Found 513.9727.

***N*-benzyloxy-3-trifluoromethyl-3-(3-(2-(trimethylsilyl)phenyl)propoxy)-isoindolinone (S4y)**

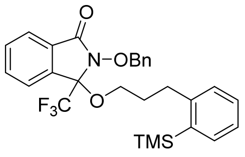

colorless liquid; ^{1}H NMR (500 MHz, $CDCl_3$) δ: 7.93 (d, 1H, J = 7.4 Hz), 7.63-7.74 (m, 3H), 7.57 (d, 2H, J = 6.9 Hz), 7.47 (dd, 1H, J = 7.4 Hz, 1.4 Hz), 7.35-7.43 (m, 3H), 7.26 (td, 1H, J = 7.4 Hz, 1.4 Hz), 7.17 (t, 1H, J = 7.4 Hz), 7.13 (d, 1H, J = 7.4 Hz), 5.35 (d, 1H, J = 9.2 Hz), 5.17 (d, 1H, J = 9.2 Hz), 3.37-3.43 (m, 1H), 3.03-3.10 (m, 1H), 2.72-2.85 (m, 2H), 1.77-1.90 (m, 2H), 0.31 (s, 9H); ^{13}C NMR (126 MHz, $CDCl_3$) δ: 165.6, 147.1, 138.1, 135.2, 134.57, 134.56, 133.5, 131.7, 130.1, 129.4, 129.2, 128.8, 128.4, 125.2, 124.2, 122.2 (q, J = 285.9 Hz), 90.9 (q, J = 33.2 Hz), 79.2, 63.8, 32.5, 31.7, 0.24; ^{19}F NMR (369 MHz, $CDCl_3$) δ: –78.0; IR (neat, cm^{-1}) ν: 1746, 1465, 1188; LRMS (ESI): *m/z* 536 $[M+Na]^+$; HRMS (ESI): *m/z* calcd for $C_{28}H_{30}F_3NO_3SiNa$ $[M+Na]^+$ 536.1839, Found 536.1816.

***N*-hydroxy-3-trifluoromethyl-3-(3-(2-(trimethylsilyl)phenyl)propoxy)-isoindolinone (1y)**

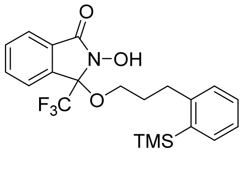

colorless liquid; ^{1}H NMR (500 MHz, $CDCl_3$) δ: 10.00 (brs, 1H), 7.84 (d, 1H, J = 7.4 Hz), 7.60-7.74 (m, 3H), 7.53 (d, 1H, J = 7.4 Hz), 7.34 (t, 1H, J = 7.4 Hz), 7.19-7.25 (m, 2H), 3.60-3.66 (m, 1H), 3.14-3.21 (m, 1H), 2.86-3.00 (m, 2H), 1.90-2.07 (m, 2H), 0.39 (s, 9H); ^{13}C NMR (126 MHz, $CDCl_3$) δ: 165.9, 147.3, 138.3, 135.2, 134.6, 133.3, 131.5, 130.0, 129.2, 128.5, 125.2, 124.0, 123.9, 121.9 (q, J = 286.7 Hz), 91.1 (q,

J = 32.8 Hz), 64.0, 32.6, 31.6, 0.27; ^{19}F NMR (369 MHz, $CDCl_3$) δ: –78.4; IR (neat, cm^{-1}) ν: 3152, 2953, 1718, 1469, 1200; LRMS (ESI): *m/z* 446 $[M+Na]^+$; HRMS (ESI): *m/z* calcd for $C_{21}H_{24}F_3NO_3SiNa$ $[M+Na]^+$ 446.1370, Found 446.1351.

N-hydroxy-3-(3-oxo-3-(2-(trimethylsilyl)phenyl)propoxy)-3-trifluoromethyl-isoindolinone (2y)

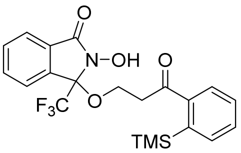

Starting material **1y** (98.0 mg, 0.23 mmol) was oxygenated under condition B of section 2, and **2y** (62.6 mg, 0.14 mmol) was isolated according to the purification procedure described in the General method (62% yield).

light yellow solid; ^{1}H NMR (400 MHz, $CDCl_3$) δ: 8.83 (brs, 1H), 7.88-7.93 (m, 2H), 7.77 (dd, 1H, J = 7.4 Hz, 1.1 Hz), 7.60-7.71 (m, 3H), 7.56 (td, 1H, J = 7.5 Hz, 1.3 Hz), 7.48 (td, 1H, J = 7.5 Hz, 1.3 Hz), 3.79 (td, 1H, J = 9.4 Hz, 2.7 Hz), 3.47-3.58 (m, 1H), 3.39-3.47 (m, 1H), 3.16-3.26 (m, 1H), 0.29 (s, 9H); ^{13}C NMR (100 MHz, $CDCl_3$) δ: 201.3, 163.4, 142.9, 141.5, 136.3, 133.9, 132.9, 132.5, 131.7, 130.7, 129.5, 128.9, 124.3, 124.1, 121.8 (q, J = 286.6 Hz), 90.5 (q, J = 33.5 Hz), 57.7, 37.9, 0.23; ^{19}F NMR (369 MHz, $CDCl_3$) δ: –78.0; IR (KBr, cm^{-1}) ν: 3228, 2953, 1719, 1469, 1200; LRMS (ESI): *m/z* 460 $[M+Na]^+$; HRMS (ESI): *m/z* calcd for $C_{21}H_{22}F_3NO_4SiNa$ $[M+Na]^+$ 460.1162, Found 460.1180.

3-(3-(4-ethylphenyl)propoxy)-N-benzyloxy-3-trifluoromethyl-isoindolinone (S4z)

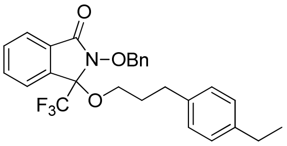

light yellow liquid; ^{1}H NMR (500 MHz, $CDCl_3$) δ: 7.89 (d, 1H, J = 6.9 Hz), 7.61-7.68 (m, 2H), 7.55-7.60 (m, 3H), 7.36-7.44 (m, 3H), 7.00-7.06 (m, 4H), 5.30 (d, 1H, J = 9.5 Hz), 5.13 (d, 1H, J = 9.5 Hz), 3.26-3.32 (m, 1H), 2.94-2.99 (m, 1H), 2.56-2.68 (m, 4H), 1.77-1.92 (m, 2H), 1.21 (t, 3H, J = 7.7 Hz); ^{13}C NMR (126 MHz, $CDCl_3$) δ: 165.6, 141.8, 138.3, 135.2, 134.6, 133.4, 131.6, 130.1, 129.4, 128.8, 128.5, 128.3, 127.8, 124.3, 124.1, 122.2 (q, J = 285.5 Hz), 91.0 (q, J = 32.8 Hz), 79.1, 63.2, 31.3, 30.5, 28.4, 15.6; ^{19}F NMR (369 MHz, $CDCl_3$) δ: –78.0; IR (neat, cm^{-1}) ν: 2960, 1745, 1468, 1188; LRMS (ESI): *m/z* 492 $[M+Na]^+$; HRMS (ESI): *m/z* calcd for $C_{27}H_{26}F_3NO_3Na$ $[M+Na]^+$ 492.1757, Found 492.1767.

3-(3-(4-ethylphenyl)propoxy)-N-hydroxy-3-trifluoromethyl-isoindolinone (1z)

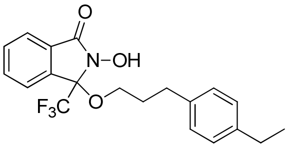

light yellow liquid; ^{1}H NMR (500 MHz, $CDCl_3$) δ: 9.80 (brs, 1H), 7.75 (d, 1H, J = 6.9 Hz), 7.59-7.64 (m, 1H), 7.53-7.58 (m, 2H), 7.05-7.12 (m, 4H), 3.41-3.47 (m, 1H), 2.96-3.04 (m, 1H), 2.63-2.75 (m, 2H), 2.60

(q, 2H, J = 7.7 Hz), 1.85-2.01 (m, 2H), 1.21 (t, 3H, J = 7.7 Hz); ^{13}C NMR (126 MHz, $CDCl_3$) δ: 165.8, 141.7, 138.5, 135.1, 133.2, 131.5, 129.9, 128.3, 127.8, 124.1, 123.9, 121.9 (q, J = 286.7 Hz), 91.0 (q, J = 32.8 Hz), 63.5, 31.4, 30.6, 28.4, 15.6; ^{19}F NMR (369 MHz, $CDCl_3$) δ: –78.4; IR (neat, cm^{-1}) ν: 3421, 2962, 1716, 1643, 1200; LRMS (ESI): *m/z* 402 $[M+Na]^+$; HRMS (ESI): *m/z* calcd for $C_{20}H_{20}F_3NO_3Na$ $[M+Na]^+$ 402.1288, Found 402.1273.

3-(3-(4-ethylphenyl)-3-oxopropoxy)-*N*-hydroxy-3-trifluoromethyl-isoindolinone (2z)

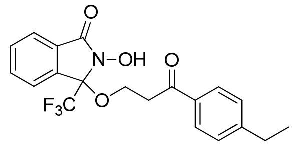

Starting material **1z** (15.2 mg, 0.04 mmol) was oxygenated under condition B of section 2, and **2z** (8.6 mg, 0.022 mmol) was isolated according to the purification procedure described in the General method (55% yield).

light yellow liquid; ^{1}H NMR (500 MHz, $CDCl_3$) δ: 8.82 (brs, 1H), 7.89-7.94 (m, 3H), 7.61-7.70 (m, 3H), 7.31 (d, 2H, J = 8.0 Hz), 3.73-3.80 (m, 1H), 3.50-3.58 (m, 1H), 3.37-3.43 (m, 1H), 3.05-3.12 (m, 1H), 2.72 (q, 2H, J = 7.5 Hz), 1.26 (t, 3H, J = 7.5 Hz); ^{13}C NMR (126 MHz, $CDCl_3$) δ: 198.6, 163.2, 151.5, 133.9, 133.7, 132.8, 131.7, 130.9, 128.7, 128.3, 124.3, 124.0, 121.8 (q, J = 286.7 Hz), 90.4 (q, J = 33.4 Hz), 57.6, 37.0, 29.0, 15.1; ^{19}F NMR (369 MHz, $CDCl_3$) δ: –78.1; IR (neat, cm^{-1}) ν: 3433, 1644, 1190; LRMS (ESI): *m/z* 416 $[M+Na]^+$; HRMS (ESI): *m/z* calcd for $C_{20}H_{18}F_3NO_4Na$ $[M+Na]^+$ 416.1080, Found 416.1088.

***N*-hydroxy-3-((3-oxooct-4-yn-1-yl)oxy)-3-trifluoromethyl-isoindolinone (2aa)**

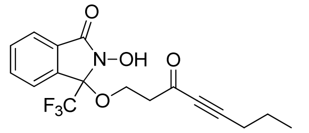

Starting material **1aa** (13.7 mg, 0.04 mmol) was oxygenated under condition A of section 2, and **2aa** (6.1 mg, 0.017 mmol) was isolated according to the purification procedure described in the General method (43% yield).
light yellow liquid (unstable); ^{1}H NMR (400 MHz, $CDCl_3$) δ: 7.87 (d, 1H, J = 6.7 Hz), 7.56-7.71 (m, 3H), 3.62 (dt, 1H, J = 3.6 Hz, 9.2 Hz), 3.26-3.34 (m, 1H), 2.97-3.08 (m, 1H), 2.79-2.88 (m, 1H), 2.36 (t, 2H, J = 7.0 Hz), 1.55-1.67 (m, 2H), 1.01 (t, 3H, J = 7.4 Hz); ^{13}C NMR (100 MHz, $CDCl_3$) δ: 185.7, 164.1, 134.0, 133.1, 131.7, 130.4, 124.2, 121.7 (q, J = 286.7 Hz), 97.2, 90.6 (q, J = 33.2 Hz), 80.8, 57.7, 44.2, 21.1, 20.9, 13.4; ^{19}F NMR (369 MHz, $CDCl_3$) δ: –78.2; IR (neat, cm^{-1}) ν: 3215, 2967, 2213, 1720, 1674, 1470, 1191; LRMS (ESI): *m/z* 378 $[M+Na]^+$; HRMS (ESI): *m/z* calcd for $C_{17}H_{16}F_3NO_4Na$ $[M+Na]^+$ 378.0924, Found 378.0929.

3-((6,6-dimethylhept-4-yn-1-yl)oxy)-*N*-((4-methoxybenzyl)oxy)-3-trifluoromethyl-isoindolinone (S4'ab)

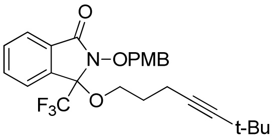

light yellow liquid; ^{1}H NMR (500 MHz, $CDCl_3$) δ: 7.89 (d, 1H, *J* = 6.9 Hz), 7.59-7.71 (m, 3H), 7.49 (d, 2H, *J* = 8.6 Hz), 6.92 (d, 2H, *J* = 8.6 Hz), 5.25 (d, 1H, *J* = 9.2 Hz), 5.08 (d, 1H, *J* = 9.2 Hz), 3.81 (s, 3H), 3.33-3.39 (m, 1H), 3.05-3.12 (m, 1H), 2.15-2.26 (m, 2H), 1.62-1.78 (m, 2H), 1.07 (s, 9H); ^{13}C NMR (126 MHz, $CDCl_3$) δ: 165.4, 160.1, 135.2, 133.4, 131.6, 131.1, 130.2, 126.8, 124.3, 124.1, 122.1 (q, *J* = 286.3 Hz), 113.8, 91.0 (q, *J* = 33.2 Hz), 89.6, 78.9, 76.9, 62.6, 55.2, 31.2, 28.6, 27.1, 15.1; ^{19}F NMR (369 MHz, $CDCl_3$) δ: –78.0; IR (neat, cm^{-1}) ν: 2966, 1746, 1613, 1516, 1468, 1253, 1187; LRMS (ESI): *m/z* 498 $[M+Na]^+$; HRMS (ESI): *m/z* calcd for $C_{26}H_{28}F_3NO_4Na$ $[M+Na]^+$ 498.1863, Found 498.1855.

3-((6,6-dimethylhept-4-yn-1-yl)oxy)-*N*-hydroxy-3-trifluoromethyl-isoindolinone (1ab)

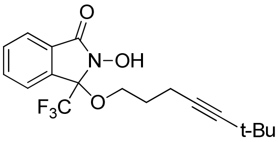

yellow liquid; ^{1}H NMR (500 MHz, $CDCl_3$) δ: 9.41 (brs, 1H), 7.82 (d, 1H, *J* = 7.4 Hz), 7.57-7.68 (m, 3H), 3.43-3.49 (m, 1H), 3.12-3.17 (m, 1H), 2.29 (t, 2H, *J* = 6.9 Hz), 1.71-1.84 (m, 2H), 1.10 (s, 9H); ^{13}C NMR (126 MHz, $CDCl_3$) δ: 165.4, 135.0, 133.2, 131.5, 130.0, 124.1, 123.9, 121.9 (q, *J* = 285.5 Hz), 90.9 (q, *J* = 33.2 Hz), 90.0, 77.1, 62.5, 31.2, 28.4, 27.2, 15.1; ^{19}F NMR (369 MHz, $CDCl_3$) δ: –78.4; IR (neat, cm^{-1}) ν: 3423, 2968, 1719, 1470, 1200; LRMS (ESI): *m/z* 378 $[M+Na]^+$; HRMS (ESI): *m/z* calcd for $C_{18}H_{20}F_3NO_3Na$ $[M+Na]^+$ 378.1288, Found 378.1280.

3-((6,6-dimethyl-3-oxohept-4-yn-1-yl)oxy)-*N*-hydroxy-3-trifluoromethyl-isoindolinone (2ab)

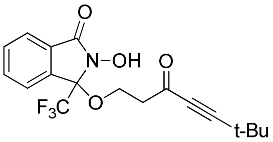

Starting material **1ab** (18.2 mg, 0.04 mmol) was oxygenated under condition A of section 2, and **2ab** (7.6 mg, 0.021 mmol) was isolated according to the purification procedure described in the General method (47% yield).

light yellow liquid (unstable); ^{1}H NMR (500 MHz, $CDCl_3$) δ: 8.69 (brs, 1H), 7.85-7.88 (m, 1H), 7.59-7.69 (m, 3H), 3.62 (dt, 1H, *J* = 4.0 Hz, 9.2 Hz), 3.27-3.32 (m, 1H), 2.97-3.06 (m, 1H), 2.80-2.87 (m, 1H), 1.27 (s, 9H); ^{13}C NMR (126 MHz, $CDCl_3$) δ: 185.9, 164.2, 134.1, 133.1, 131.7, 130.4, 124.2, 123.9, 121.7 (q, *J* = 286.3 Hz), 104.3, 90.6 (q, *J* = 33.6 Hz), 79.2, 57.7, 44.2, 29.9, 27.8; ^{19}F NMR (369 MHz, $CDCl_3$) δ: –78.2; IR (neat, cm^{-1}) ν: 3196, 2974, 2211, 1719, 1675, 1470, 1199; LRMS (ESI): *m/z* 392 $[M+Na]^+$; HRMS (ESI): *m/z* calcd for $C_{18}H_{18}F_3NO_4Na$ $[M+Na]^+$ 392.1080, Found 392.1070.

***N*-((4-methoxybenzyl)oxy)-3-((8-methylnon-4-yn-1-yl)oxy)-3-(trifluoromethyl)-isoindolinone (S4'ac)**

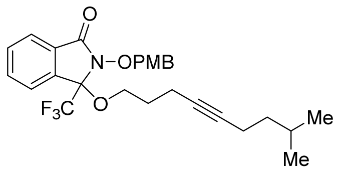

colorless liquid; ^{1}H NMR (400 MHz, $CDCl_3$) δ: 7.89 (dd, 1H, J = 6.5 Hz, 1.1 Hz), 7.60-7.71 (m, 3H), 7.46-7.52 (m, 2H), 6.90-6.95 (m, 2H), 5.25 (d, 1H, J = 9.2 Hz), 5.08 (d, 1H, J = 9.2 Hz), 3.82 (s, 3H), 3.34-3.41 (m, 1H), 3.04-3.12 (m, 1H), 2.19-2.26 (m, 2H), 1.96-2.04 (m, 2H), 1.61-1.80 (m, 2H), 1.50-1.60 (m, 1H), 1.24 (q, 2H, J = 7.2 Hz), 0.80-0.84 (m, 6H); ^{13}C NMR (100 MHz, $CDCl_3$) δ: 165.4, 160.1, 135.1, 133.3, 131.6, 131.1, 130.2, 126.8, 124.3, 124.1, 122.1 (q, J = 285.9 Hz), 113.8, 91.0 (q, J = 32.9 Hz), 81.0, 78.9, 78.4, 62.5, 55.2, 37.9, 28.5, 27.1, 22.10, 22.08, 16.6, 15.2; ^{19}F NMR (369 MHz, $CDCl_3$) δ: –78.0; IR (neat, cm^{-1}) ν: 2956, 1744, 1613, 1515, 1468, 1253, 1187; LRMS (ESI): *m/z* 512 $[M+Na]^+$; HRMS (ESI): *m/z* calcd for $C_{27}H_{30}F_3NO_4Na$ $[M+Na]^+$ 512.2019, Found 512.2003.

N-hydroxy-3-((8-methylnon-4-yn-1-yl)oxy)-3-(trifluoromethyl)-isoindolinone (1ac)

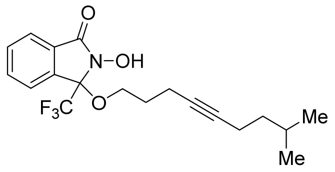

light yellow liquid; ^{1}H NMR (500 MHz, $CDCl_3$) δ: 9.30 (brs, 1H), 7.82 (d, 1H, J = 7.4 Hz), 7.58-7.68 (m, 3H), 3.44-3.50 (m, 1H), 3.12-3.18 (m, 1H), 2.29-2.35 (m, 2H), 2.04-2.10 (m, 2H), 1.71-1.85 (m, 2H), 1.53-1.63 (m, 1H), 1.29 (q, 2H, J = 7.5 Hz), 0.82-0.86 (m, 6H); ^{13}C NMR (126 MHz, $CDCl_3$) δ: 165.4, 135.0, 133.1, 131.5, 130.1, 124.2, 123.9, 121.9 (q, J = 286.7 Hz), 90.9 (q, J = 33.2 Hz), 81.5, 78.7, 62.5, 37.9, 28.2, 27.2, 22.1, 16.6, 15.2; ^{19}F NMR (369 MHz, $CDCl_3$) δ: –78.2; IR (neat, cm^{-1}) ν: 3159, 2956, 1718, 1469, 1305, 1191; LRMS (ESI): *m/z* 392 $[M+Na]^+$; HRMS (ESI): *m/z* calcd for $C_{19}H_{22}F_3NO_3Na$ $[M+Na]^+$ 392.1444, Found 392.1442.

N-hydroxy-3-((8-methyl-3-oxonon-4-yn-1-yl)oxy)-3-(trifluoromethyl)-isoindolinone (2ac)

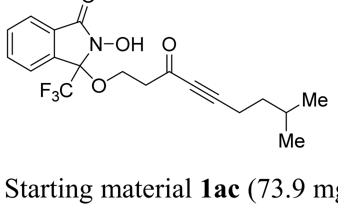

Starting material **1ac** (73.9 mg, 0.2 mmol) was oxygenated under condition A of section 2, and **2ac** (26.0 mg, 0.068 mmol) was isolated according to the purification procedure described in the General method (34% yield).

yellow liquid; ^{1}H NMR (400 MHz, $CDCl_3$) δ: 8.77 (brs, 1H), 7.86 (d, 1H, J = 6.7 Hz), 7.58-7.69 (m, 3H), 3.62 (td, 1H, J = 9.0 Hz, 4.0 Hz), 3.27-3.34 (m, 1H), 2.95-3.05 (m, 1H), 2.84 (td, 1H, J = 4.7 Hz, 18.5 Hz), 2.37 (t, 2H, J = 7.3 Hz), 1.60-1.73 (m, 1H), 1.47 (q, 2H, J = 7.3 Hz), 0.90 (d, 6H, J = 6.7 Hz); ^{13}C NMR (100 MHz, $CDCl_3$) δ: 185.6, 164.3, 134.1, 133.1, 131.7, 130.3, 124.19, 124.16, 121.7 (q, J = 286.6 Hz),

97.3, 90.6 (q, J = 33.5 Hz), 80.6, 57.8, 44.2, 36.3, 27.3, 21.9, 17.0; ^{19}F NMR (369 MHz, $CDCl_3$) δ: –78.2; IR (neat, cm^{-1}) ν: 3182, 2958, 2212, 1721, 1675, 1469, 1190; LRMS (ESI): *m/z* 406 $[M+Na]^+$; HRMS (ESI): *m/z* calcd for $C_{19}H_{20}F_3NO_4Na$ $[M+Na]^+$ 406.1237, Found 406.1234.

***N*-((4-methoxybenzyl)oxy)-3-(3-(thiophen-2-yl)propoxy)-3-trifluoromethyl-isoindolinone (S4'ad)**

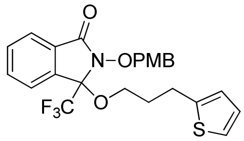

light yellow liquid; ^{1}H NMR (500 MHz, $CDCl_3$) δ: 7.89 (d, 1H, J = 6.9 Hz), 7.61-7.69 (m, 2H), 7.58 (d, 1H, J = 7.4 Hz), 7.50 (d, 2H, J = 8.6 Hz), 7.07 (dd, 1H, J = 5.2 Hz, 1.1 Hz), 6.92-6.96 (m, 2H), 6.84 (dd, 1H, J = 5.2 Hz, 3.4 Hz), 6.69-6.71 (m, 1H), 5.25 (d, 1H, J = 9.5 Hz), 5.06 (d, 1H, J = 9.5 Hz), 3.82 (s, 3H), 3.28-3.35 (m, 1H), 2.96-3.03 (m, 1H), 2.87 (t, 2H, J = 7.4 Hz), 1.83-1.97 (m, 2H); ^{13}C NMR (126 MHz, $CDCl_3$) δ: 165.5, 160.1, 143.8, 135.1, 133.4, 131.6, 131.2, 130.1, 126.8, 126.7, 124.4, 124.2, 124.1, 123.1, 122.2 (q, J = 285.5 Hz), 113.8, 90.8 (q, J = 32.8 Hz), 78.8, 62.8, 55.2, 30.8, 26.0; ^{19}F NMR (369 MHz, $CDCl_3$) δ: –78.0; IR (neat, cm^{-1}) ν: 3437, 2953, 1742, 1613, 1515, 1253, 1188; LRMS (ESI): *m/z* 500 $[M+Na]^+$; HRMS (ESI): *m/z* calcd for $C_{24}H_{22}F_3NO_4SNa$ $[M+Na]^+$ 500.1114, Found 500.1136.

***N*-hydroxy-3-(3-(thiophen-2-yl)propoxy)-3-trifluoromethyl-isoindolinone (1ad)**

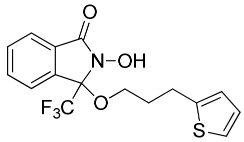

light yellow liquid; ^{1}H NMR (500 MHz, $CDCl_3$) δ: 9.66 (brs, 1H), 7.76-7.80 (m, 1H), 7.62-7.67 (m, 1H), 7.56-7.61 (m, 2H), 7.08-7.10 (m, 1H), 6.90 (dd, 1H, J = 5.2 Hz, 3.4 Hz), 3.42-3.49 (m, 1H), 3.03-3.09 (m, 1H), 2.97 (t, 2H, J = 7.4 Hz), 1.93-2.09 (m, 2H); ^{13}C NMR (126 MHz, $CDCl_3$) δ: 165.8, 144.1, 135.0, 133.3, 131.5, 129.9, 126.7, 124.5, 124.1, 123.9, 123.1, 121.9 (q, J = 286.3 Hz), 91.0 (q, J = 33.2 Hz), 63.1, 30.9, 26.1; ^{19}F NMR (369 MHz, $CDCl_3$) δ: –78.4; IR (neat, cm^{-1}) ν: 3158, 2950, 1718, 1469, 1200; LRMS (ESI): *m/z* 380 $[M+Na]^+$; HRMS (ESI): *m/z* calcd for $C_{16}H_{14}F_3NO_3SNa$ $[M+Na]^+$ 380.0539, Found 380.0547.

***N*-hydroxy-3-(3-oxo-3-(thiophen-2-yl)propoxy)-3-trifluoromethyl-isoindolinone (2ad)**

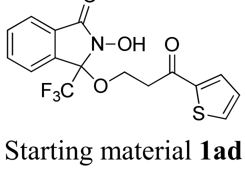

Starting material **1ad** (35.7 mg, 0.1 mmol) was oxygenated under condition A of section 2, and **2ad** (19.4 mg, 0.052 mmol) was isolated according to the purification procedure described in the General method (52% yield).

light yellow solid; ^{1}H NMR (500 MHz, $CDCl_3$) δ: 8.79 (brs, 1H), 7.88 (d, 1H, J = 7.4 Hz), 7.79 (d, 1H, J = 4.0 Hz), 7.72 (d, 1H, J = 5.2 Hz), 7.60-7.69 (m, 3H), 7.14-7.17 (m, 1H), 3.75 (dt, 1H, J= 3.1 Hz, 9.9 Hz), 3.39-3.49 (m, 2H), 3.10-3.37 (m, 1H); ^{13}C NMR (126 MHz, $CDCl_3$) δ: 191.3, 163.9, 143.1, 135.0, 134.0,

133.2, 133.0, 131.7, 130.5, 128.4, 124.2, 124.1, 121.8 (q, J = 286.7 Hz), 90.6 (q, J = 33.6 Hz), 58.0, 38.0; ^{19}F NMR (369 MHz, $CDCl_3$) δ: –78.1; IR (KBr, cm^{-1}) ν: 3178, 2959, 1741, 1637, 1418, 1197; LRMS (ESI): *m/z* 394 $[M+Na]^+$; HRMS (ESI): *m/z* calcd for $C_{16}H_{12}F_3NO_4SNa$ $[M+Na]^+$ 394.0331, Found 394.0337.

***N*-benzyloxy-3-(3-(1-tosyl-1*H*-indol-3-yl)propoxy)-3-trifluoromethyl-isoindolinone (S4ae)**

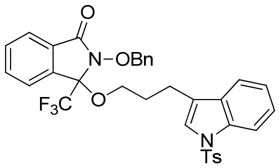

white solid; ^{1}H NMR (400 MHz, $CDCl_3$) δ: 7.97 (d, 1H, J = 8.5 Hz), 7.88-7.93 (m, 1H), 7.71 (d, 2H, J = 8.5 Hz), 7.53-7.70 (m, 5H), 7.34-7.43 (m, 4H), 7.27-7.33 (m, 1H), 7.25 (s, 1H), 7.15-7.22 (m, 3H), 5.35 (d, 1H, J = 9.7 Hz), 5.14 (d, 1H, J = 9.7 Hz), 3.26-3.35 (m, 1H), 2.96-3.04 (m, 1H), 2.63-2.79 (m, 2H), 2.31 (s, 3H), 1.81-2.00 (m, 2H); ^{13}C NMR (100 MHz, $CDCl_3$) δ: 165.6, 144.7, 135.2, 135.0, 134.5, 133.6, 131.7, 130.7, 129.8, 129.7, 129.3, 128.8, 128.4, 126.6, 124.6, 124.2, 124.1, 122.9, 122.6, 122.2 (q, J = 285.2 Hz), 121.9, 119.3, 113.6, 90.8 (q, J = 32.9 Hz), 79.1, 63.0, 28.1, 21.4, 20.9; ^{19}F NMR (369 MHz, $CDCl_3$) δ: –78.0; IR (KBr, cm^{-1}) ν: 3064, 2950, 1744, 1448, 1370, 1174; LRMS (ESI): *m/z* 657 $[M+Na]^+$; HRMS (ESI): *m/z* calcd for $C_{34}H_{29}F_3N_2O_5SNa$ $[M+Na]^+$ 657.1642, Found 657.1631.

***N*-hydroxy-3-(3-(1-tosyl-1*H*-indol-3-yl)propoxy)-3-trifluoromethyl-isoindolinone (1ae)**

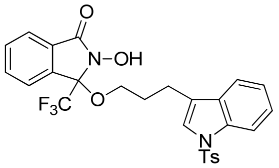

white solid; ^{1}H NMR (500 MHz, $CDCl_3$) δ: 9.84 (brs,1H), 7.97 (d, 1H, J = 8.0 Hz), 7.73 (d, 3H, J = 8.0 Hz), 7.50-7.65 (m, 3H), 7.47 (d, 1H, J = 8.0 Hz), 7.28-7.34 (m, 2H), 7.16-7.23 (m, 3H), 3.44-3.50 (m, 1H), 3.02-3.09 (m, 1H), 2.80 (t, 2H, J = 7.4 Hz), 2.30 (s, 3H), 1.93-2.10 (m, 2H); ^{13}C NMR (126 MHz, $CDCl_3$) δ: 165.8, 144.7, 135.3, 135.2, 135.0, 133.4, 131.6, 130.8, 129.8, 126.7, 124.6, 124.0, 123.9, 122.9, 122.8, 122.0, 121.9 (q, J = 286.7 Hz), 119.4, 113.6, 91.0 (q, J = 33.6 Hz), 63.2, 28.2, 21.4, 21.0; ^{19}F NMR (369 MHz, $CDCl_3$) δ: –78.3; IR (KBr, cm^{-1}) ν: 3117, 2943, 1718, 1448, 1372, 1174; LRMS (ESI): *m/z* 567 $[M+Na]^+$; HRMS (ESI): *m/z* calcd for $C_{27}H_{23}F_3N_2O_5SNa$ $[M+Na]^+$ 567.1172, Found 567.1165.

***N*-hydroxy-3-(3-oxo-3-(1-tosyl-1*H*-indol-3-yl)propoxy)-3-trifluoromethyl-isoindolinone (2ae)**

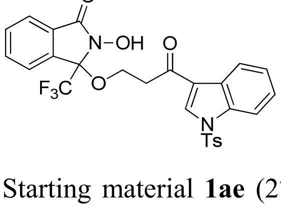

Starting material **1ae** (27.2 mg, 0.05 mmol) was oxygenated under condition A of section 2, and **2ae** (14.8 mg, 0.026 mmol) was isolated according to the purification procedure described in the General

method (53% yield).

light yellow solid; ^{1}H NMR (500 MHz, $CDCl_3$) δ: 9.04 (brs, 1H), 8.32 (s, 1H), 8.24 (d, 1H, J = 8.0 Hz), 7.86-7.92 (m, 2H), 7.83 (d, 2H, J = 8.6 Hz), 7.58-7.69 (m, 3H), 7.31-7.39 (m, 2H), 7.28 (d, 2H, J = 8.6 Hz), 3.76-3.83 (m, 1H), 3.41-3.49 (m, 2H), 3.11-3.20 (m ,1H), 2.35 (s, 3H); ^{13}C NMR (126 MHz, $CDCl_3$) δ: 194.5, 163.7, 146.2, 134.7, 134.2, 133.9, 132.91, 132.89, 131.7, 130.6, 130.3, 127.2, 127.1, 126.0, 125.1, 124.2, 124.1, 123.0, 121.8 (q, J = 286.7 Hz), 120.6, 113.0, 90.5 (q, J = 32.4 Hz), 57.8, 38.3, 21.6; ^{19}F NMR (369 MHz, $CDCl_3$) δ: –78.0; IR (KBr, cm^{-1}) ν: 3135, 2952, 1736, 1656, 1536, 1382, 1178; LRMS (ESI): *m/z* 581 $[M+Na]^+$; HRMS (ESI): *m/z* calcd for $C_{27}H_{21}F_3N_2O_6SNa$ $[M+Na]^+$ 581.0965, Found 581.0978.

O-Bn-NHPI

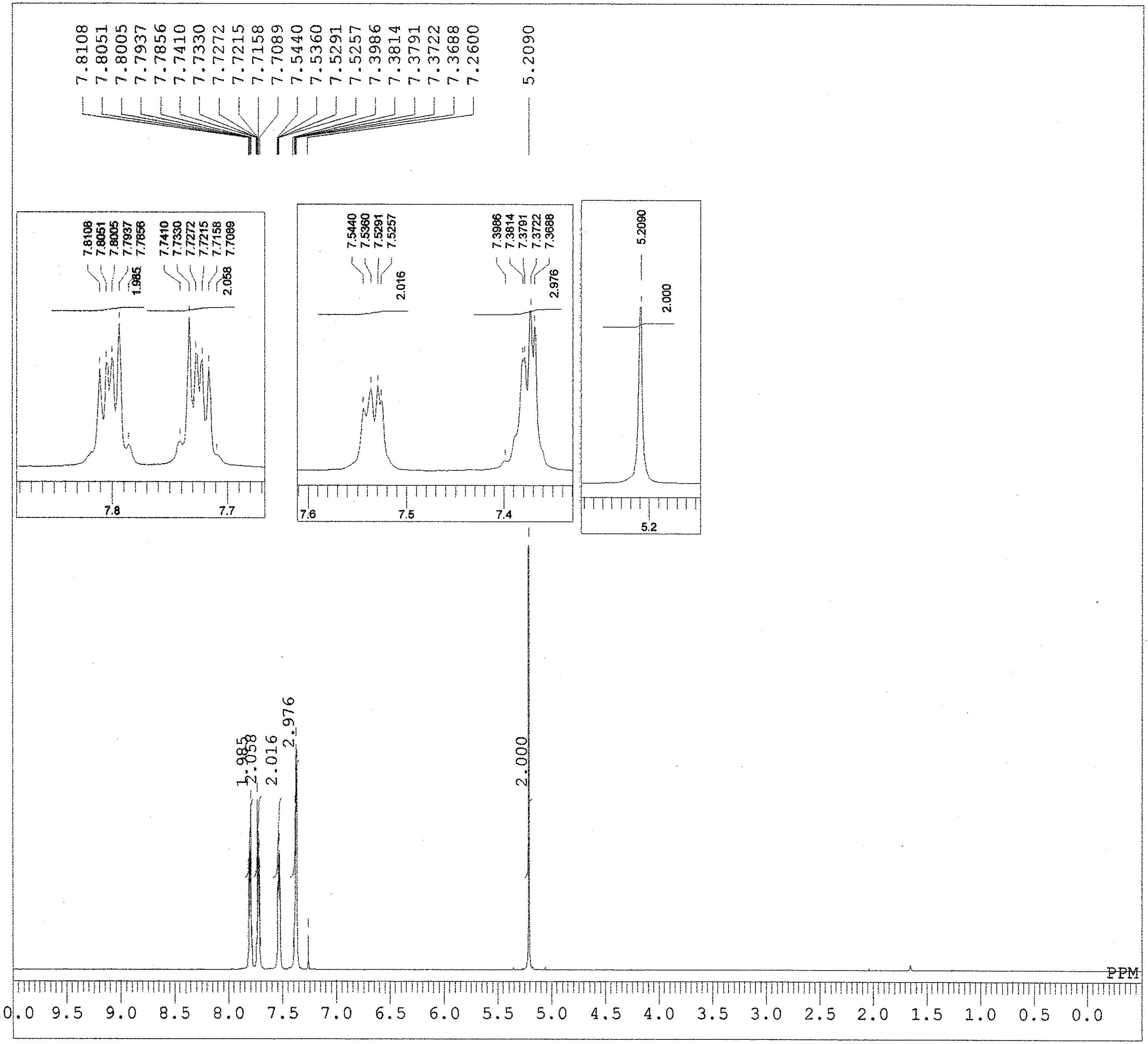

DFILE	ozawa03-087_1H.jdf
COMNT	O-Bn-NHPI
DATIM	2014-01-31 16:45:47
OBNUC	1H
EXMOD	proton.jxp
OBFRQ	500.16 MHz
OBSET	2.41 KHz
OBFIN	6.01 Hz
POINT	16384
FREQU	9384.38 Hz
SCANS	4
ACQTM	1.7459 sec
PD	5.0000 sec
PW1	5.55 usec
IRNUC	1H
CTEMP	21.7 c
SLVNT	CDCL3
EXREF	7.26 ppm
BF	1.00 Hz
RGAIN	34

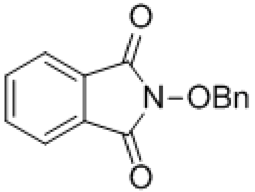

S1

O-Bn-NHPI

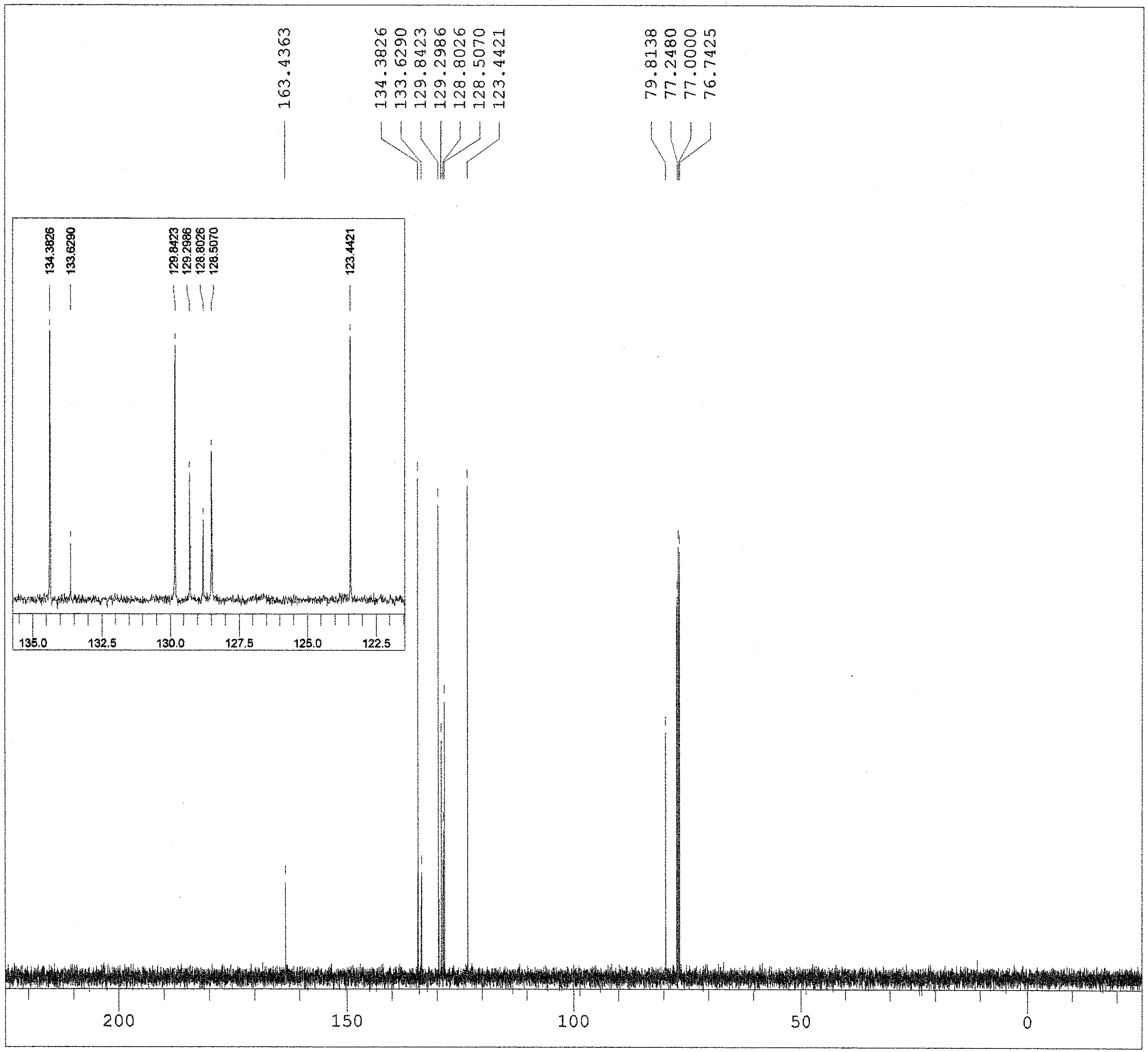

DFILE	ozawa03-087_13C.jdf
COMNT	O-Bn-NHPI
DATIM	2014-01-31 16:46:51
OBNUC	13C
EXMOD	carbon.jxp
OBFRQ	125.77 MHz
OBSET	7.87 KHz
OBFIN	4.21 Hz
POINT	32767
FREQU	39308.18 Hz
SCANS	20
ACQTM	0.8336 sec
PD	3.0000 sec
PW1	3.40 usec
IRNUC	1H
CTEMP	21.7 c
SLVNT	CDCL3
EXREF	77.00 ppm
BF	1.00 Hz
RGAIN	60

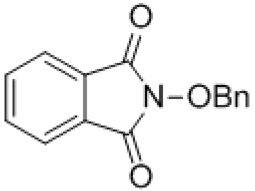

S1

S2, CF3-OH

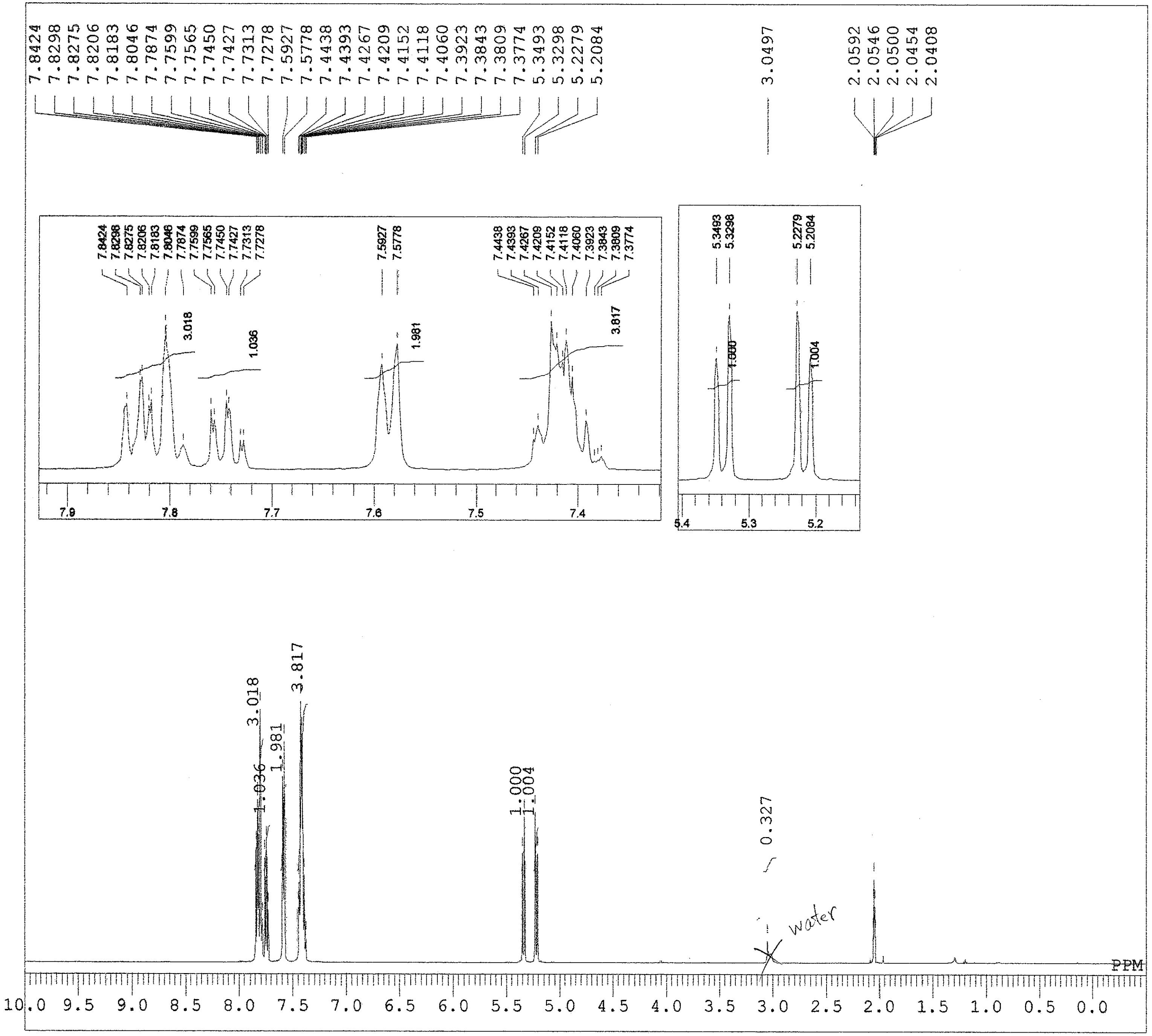

DFILE	ozawa04-026_1H.jdf
COMNT	S2, CF3-OH
DATIM	2014-01-31 21:15:49
OBNUC	1H
EXMOD	proton.jxp
OBFRQ	500.16 MHz
OBSET	2.41 KHz
OBFIN	6.01 Hz
POINT	16384
FREQU	9384.38 Hz
SCANS	4
ACQTM	1.7459 sec
PD	5.0000 sec
PW1	5.55 usec
IRNUC	1H
CTEMP	21.7 c
SLVNT	ACETN
EXREF	2.05 ppm
BF	0.12 Hz
RGAIN	32

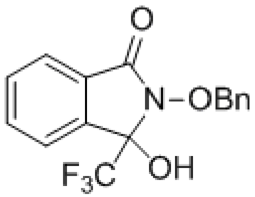

S2

S2, CF3-OH

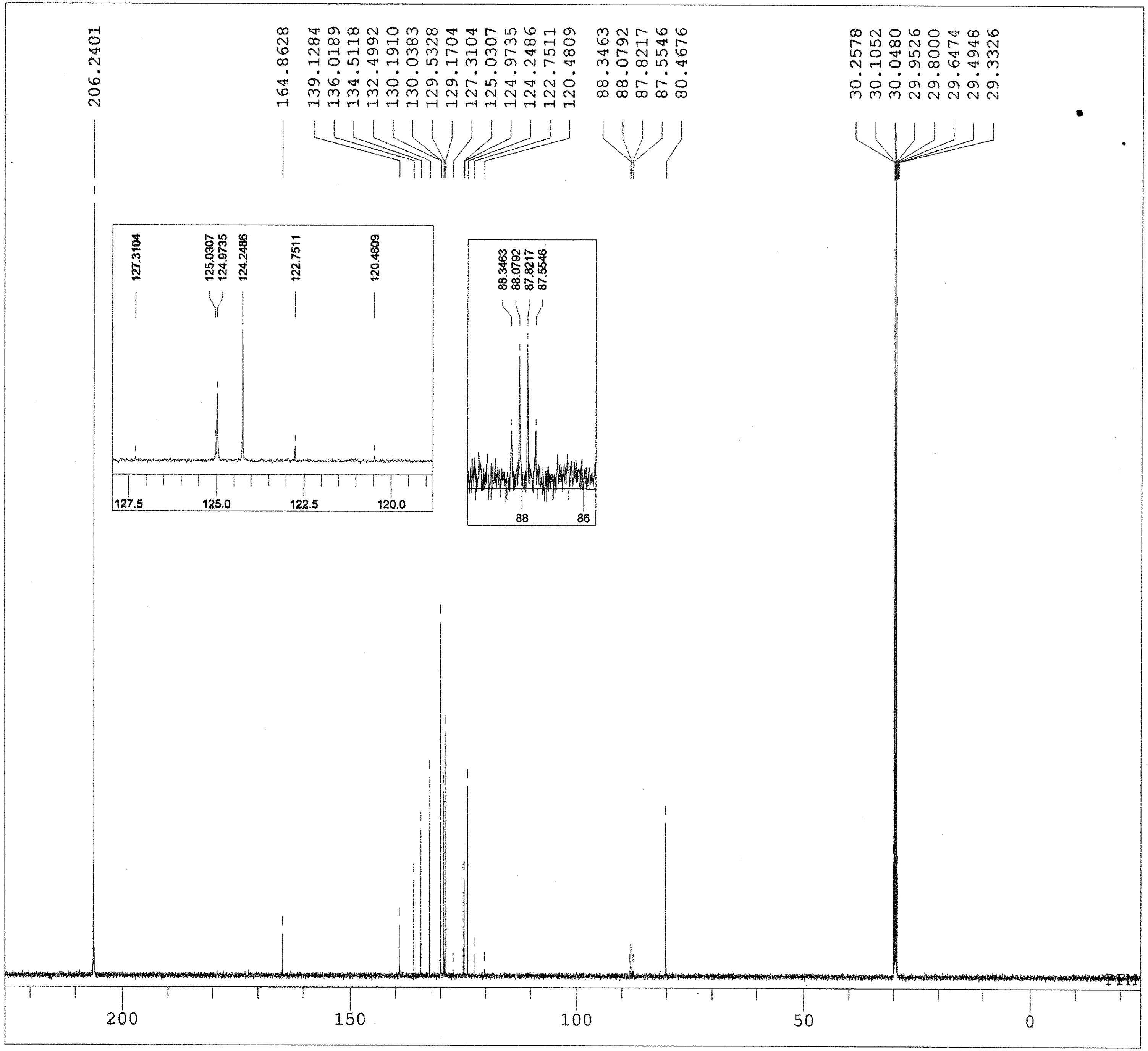

DFILE	ozawa04-026_13C.jdf
COMNT	S2, CF3-OH
DATIM	2014-01-31 21:16:52
OBNUC	13C
EXMOD	carbon.jxp
OBFRQ	125.77 MHz
OBSET	7.87 KHz
OBFIN	4.21 Hz
POINT	32767
FREQU	39308.18 Hz
SCANS	176
ACQTM	0.8336 sec
PD	3.0000 sec
PW1	3.40 usec
IRNUC	1H
CTEMP	22.1 c
SLVNT	ACETN
EXREF	29.80 ppm
BF	1.00 Hz
RGAIN	60

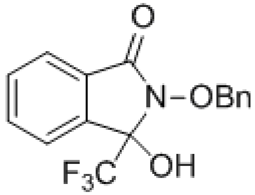

S2

S2, CF3-OH

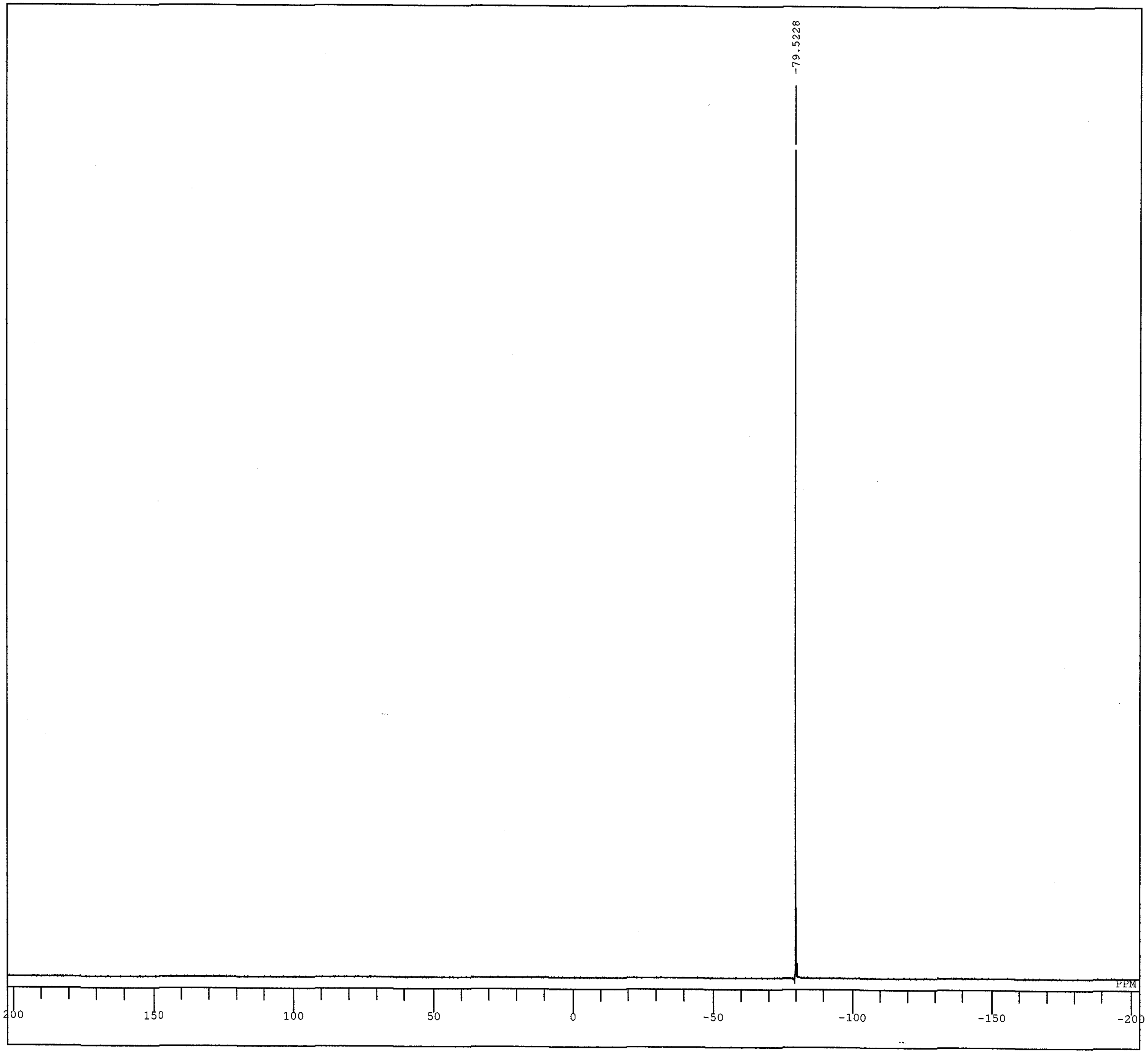

DFILE ozawa04-026_19F.jdf
COMNT S2, CF3-OH
DATIM 31-01-2014 20:52:12
OBNUC 19F
EXMOD proton.jxp
OBFRQ 368.64 MHz
OBSET 7.63 KHz
OBFIN 2.85 Hz
POINT 16384
FREQU 186567.17 Hz
SCANS 4
ACQTM 0.0878 sec
PD 5.0000 sec
PW1 3.90 usec
IRNUC 19F
CTEMP 21.3 c
SLVNT ACETN
EXREF -164.90 ppm
BF 0.12 Hz
RGAIN 46

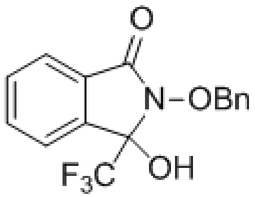

S2

S3, CF3-Cl

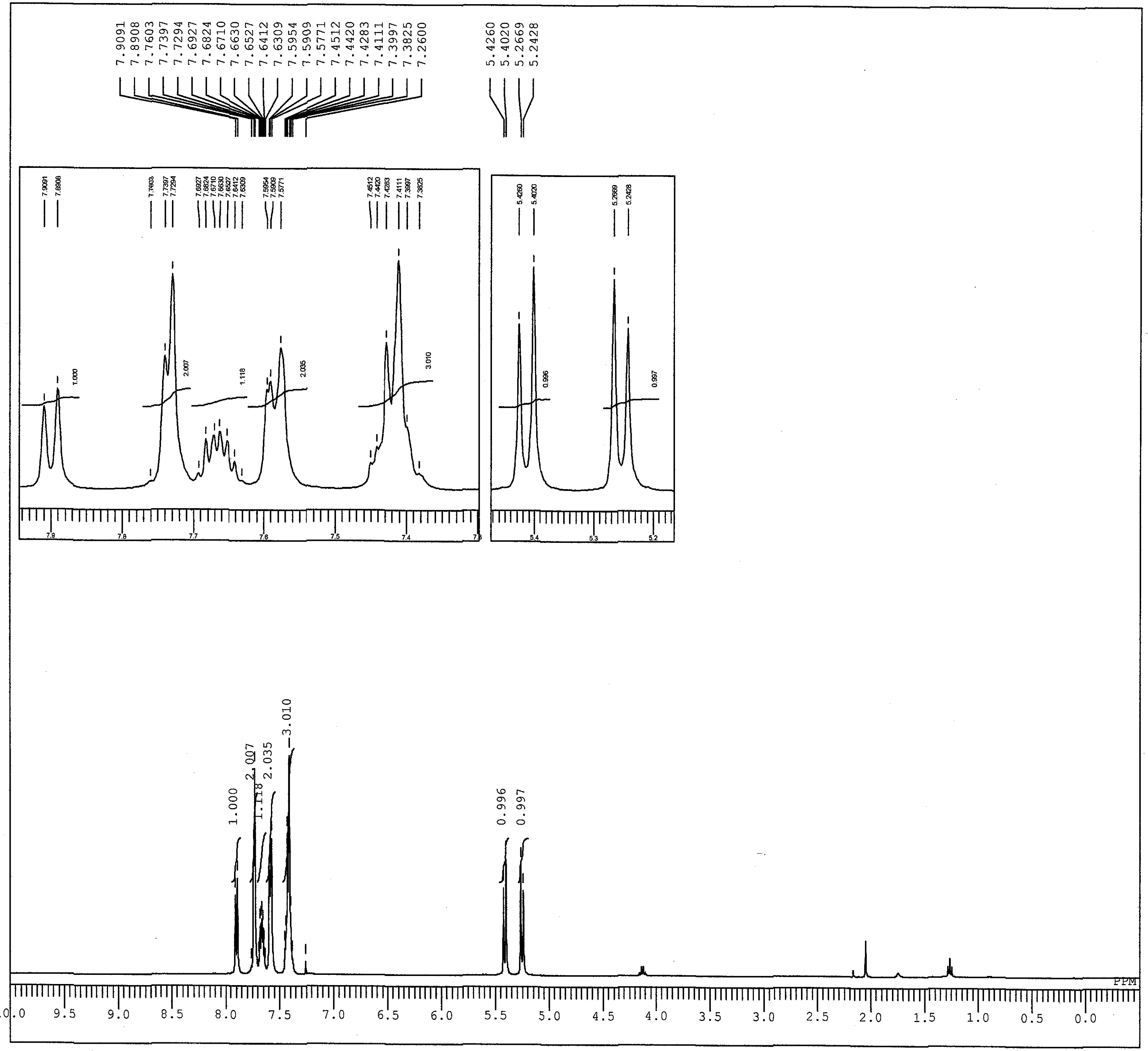

DFILE ozawa04-131_1H.jdf
COMNT S3, CF3-Cl
DATIM 11-02-2014 20:18:48
OBNUC 1H
EXMOD proton.jxp
OBFRQ 391.78 MHz
OBSET 8.51 KHz
OBFIN 3.34 Hz
POINT 16384
FREQU 7348.62 Hz
SCANS 4
ACQTM 2.2295 sec
PD 5.0000 sec
PW1 5.25 usec
IRNUC 1H
CTEMP 21.1 c
SLVNT CDCL3
EXREF 7.26 ppm
BF 1.00 Hz
RGAIN 28

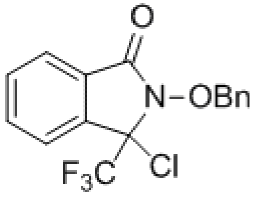

S3

S3, CF3-Cl

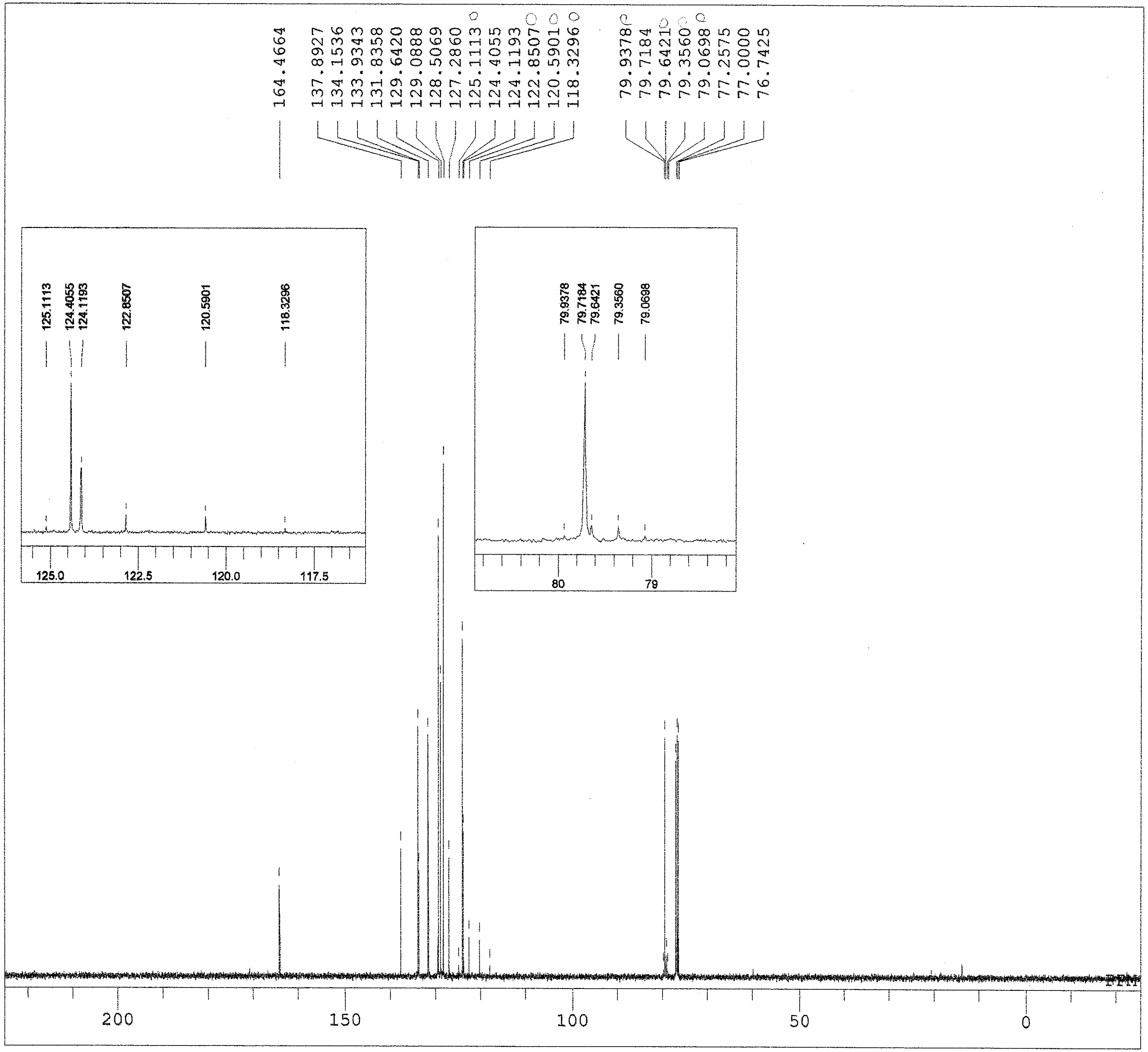

DFILE	ozawa04-131_13C.jdf
COMNT	S3, CF3-Cl
DATIM	2014-02-11 20:50:43
OBNUC	13C
EXMOD	carbon.jxp
OBFRQ	125.77 MHz
OBSET	7.87 KHz
OBFIN	4.21 Hz
POINT	32767
FREQU	39308.18 Hz
SCANS	84
ACQTM	0.8336 sec
PD	3.0000 sec
PW1	3.40 usec
IRNUC	1H
CTEMP	21.6 c
SLVNT	CDCL3
EXREF	77.00 ppm
BF	1.00 Hz
RGAIN	60

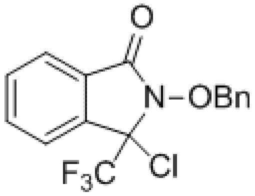

S3

S3, CF3-Cl

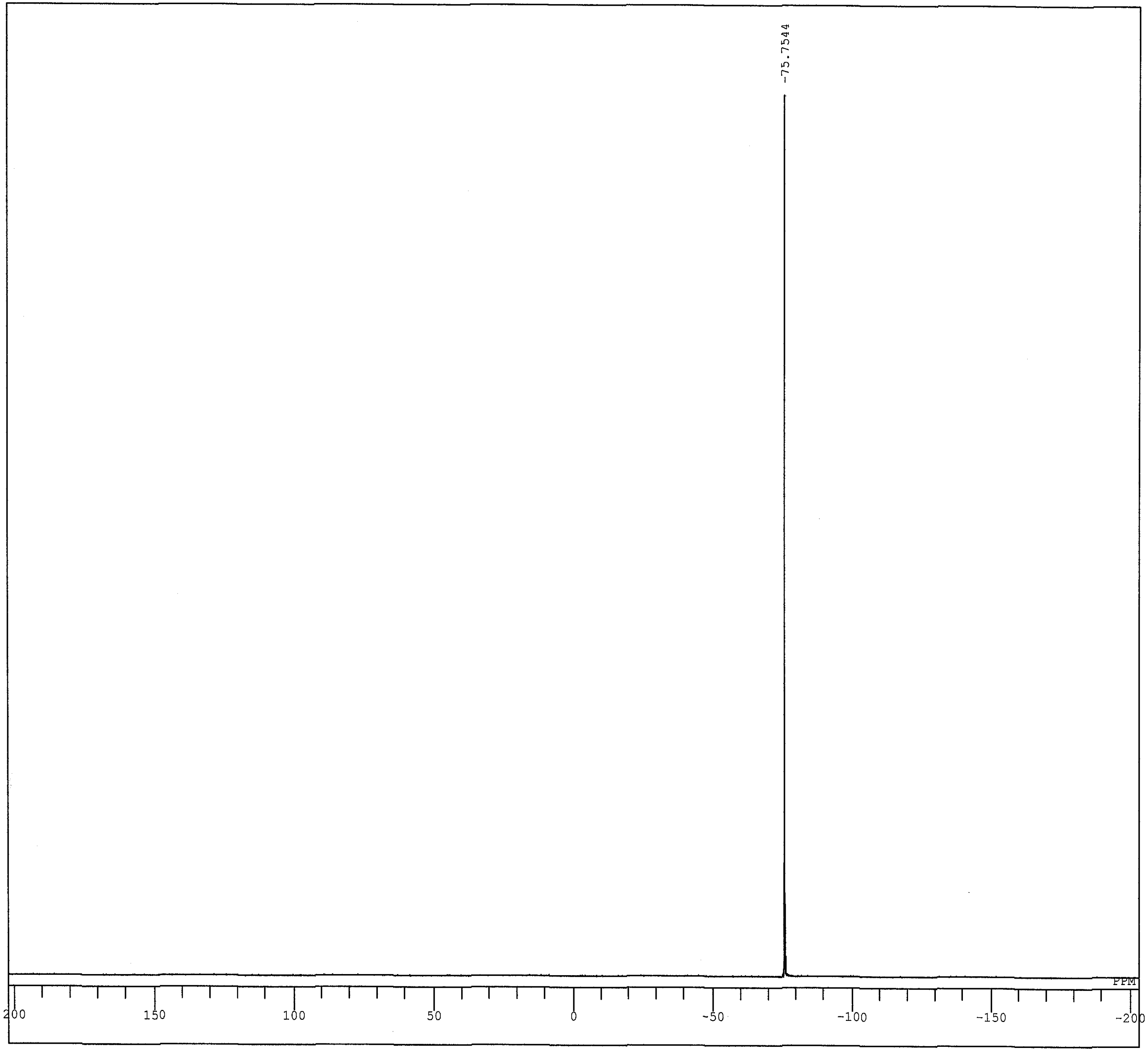

DFILE	ozawa04-131_19F.jdf
COMNT	S3, CF3-Cl
DATIM	11-02-2014 20:16:32
OBNUC	19F
EXMOD	proton.jxp
OBFRQ	368.64 MHz
OBSET	7.63 KHz
OBFIN	2.85 Hz
POINT	16384
FREQU	186567.17 Hz
SCANS	4
ACQTM	0.0878 sec
PD	5.0000 sec
PW1	3.90 usec
IRNUC	19F
CTEMP	20.9 c
SLVNT	CDCL3
EXREF	-164.90 ppm
BF	1.00 Hz
RGAIN	44

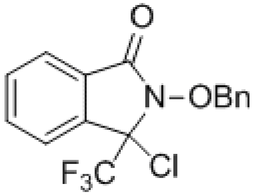

S3

PMB-NHPI

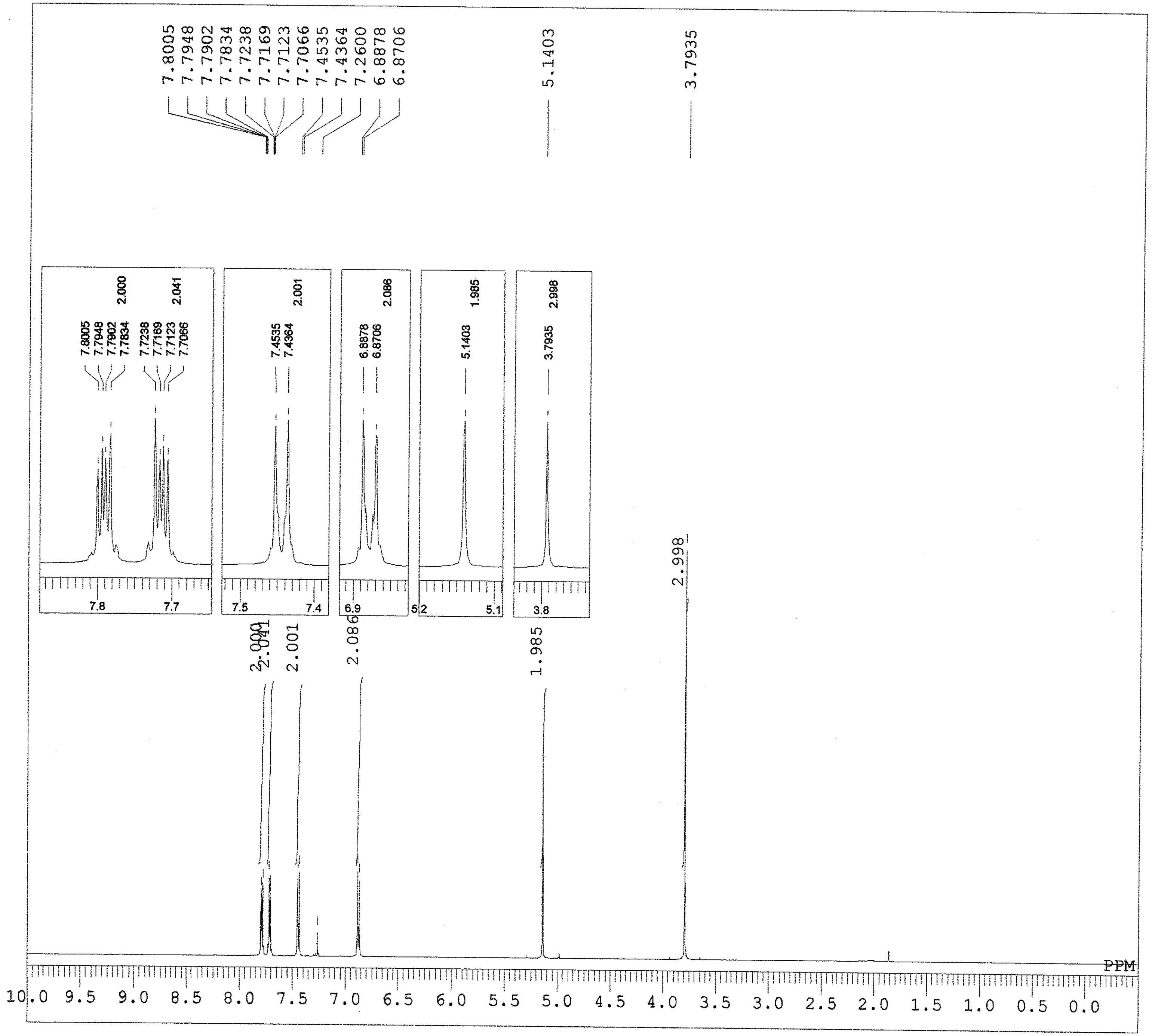

DFILE	Ta02-134_1H.jdf
COMNT	PMB-NHPI
DATIM	2014-08-16 15:28:57
OBNUC	1H
EXMOD	proton.jxp
OBFRQ	500.16 MHz
OBSET	2.41 KHz
OBFIN	6.01 Hz
POINT	16384
FREQU	9384.38 Hz
SCANS	4
ACQTM	1.7459 sec
PD	5.0000 sec
PW1	5.55 usec
IRNUC	1H
CTEMP	26.3 c
SLVNT	CDCL3
EXREF	7.26 ppm
BF	0.12 Hz
RGAIN	36

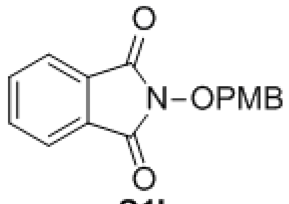

S1'

PMB-NHPI

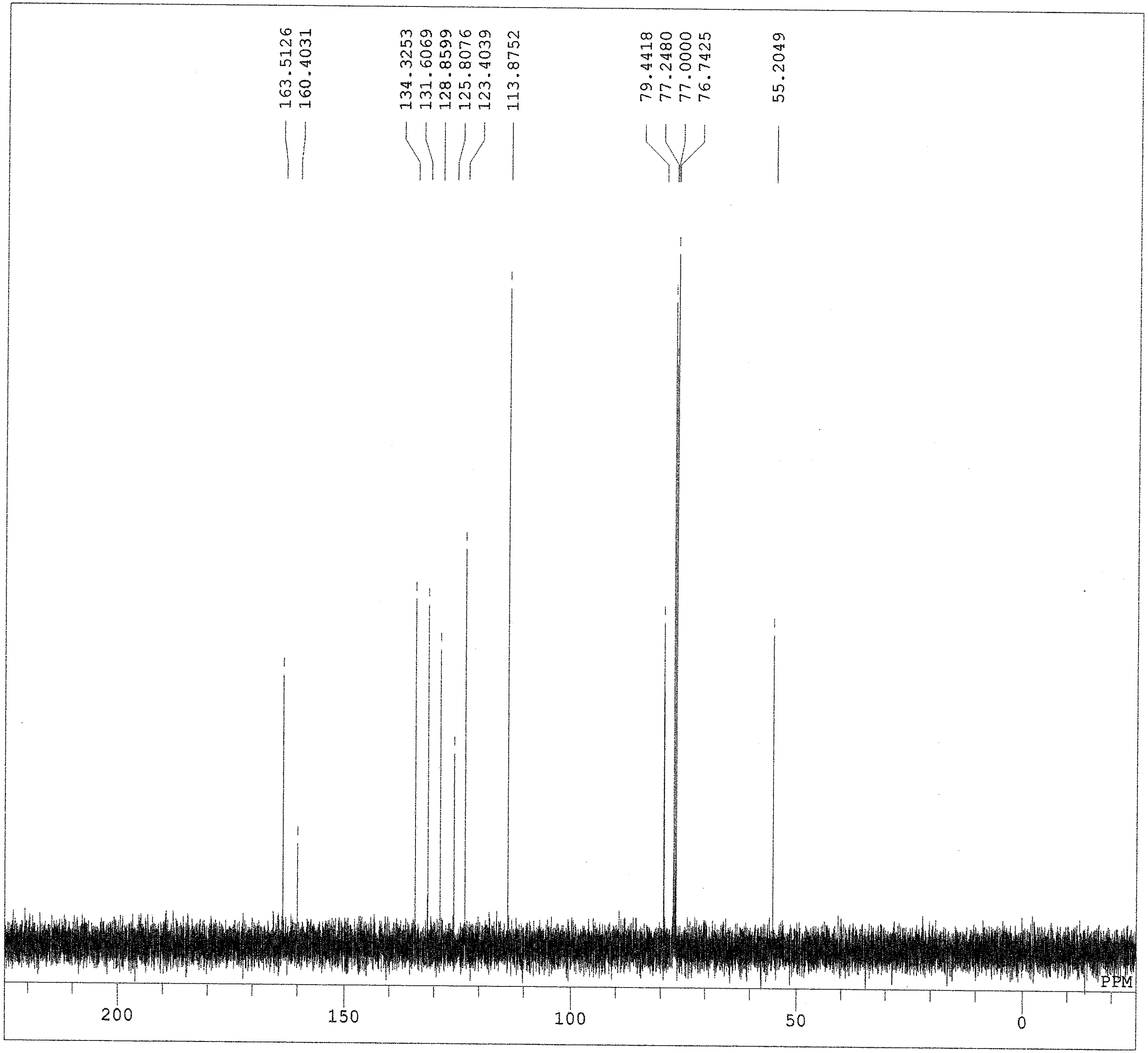

DFILE	Ta02-134_13C-.jdf
COMNT	PMB-NHPI
DATIM	2014-08-16 15:30:00
OBNUC	13C
EXMOD	carbon.jxp
OBFRQ	125.77 MHz
OBSET	7.87 KHz
OBFIN	4.21 Hz
POINT	32767
FREQU	39308.18 Hz
SCANS	20
ACQTM	0.8336 sec
PD	3.0000 sec
PW1	3.40 usec
IRNUC	1H
CTEMP	26.5 c
SLVNT	CDCL3
EXREF	77.00 ppm
BF	0.12 Hz
RGAIN	60

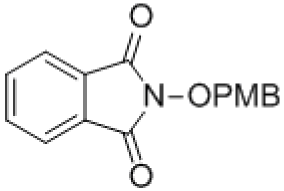

S1'

PMB, CF3

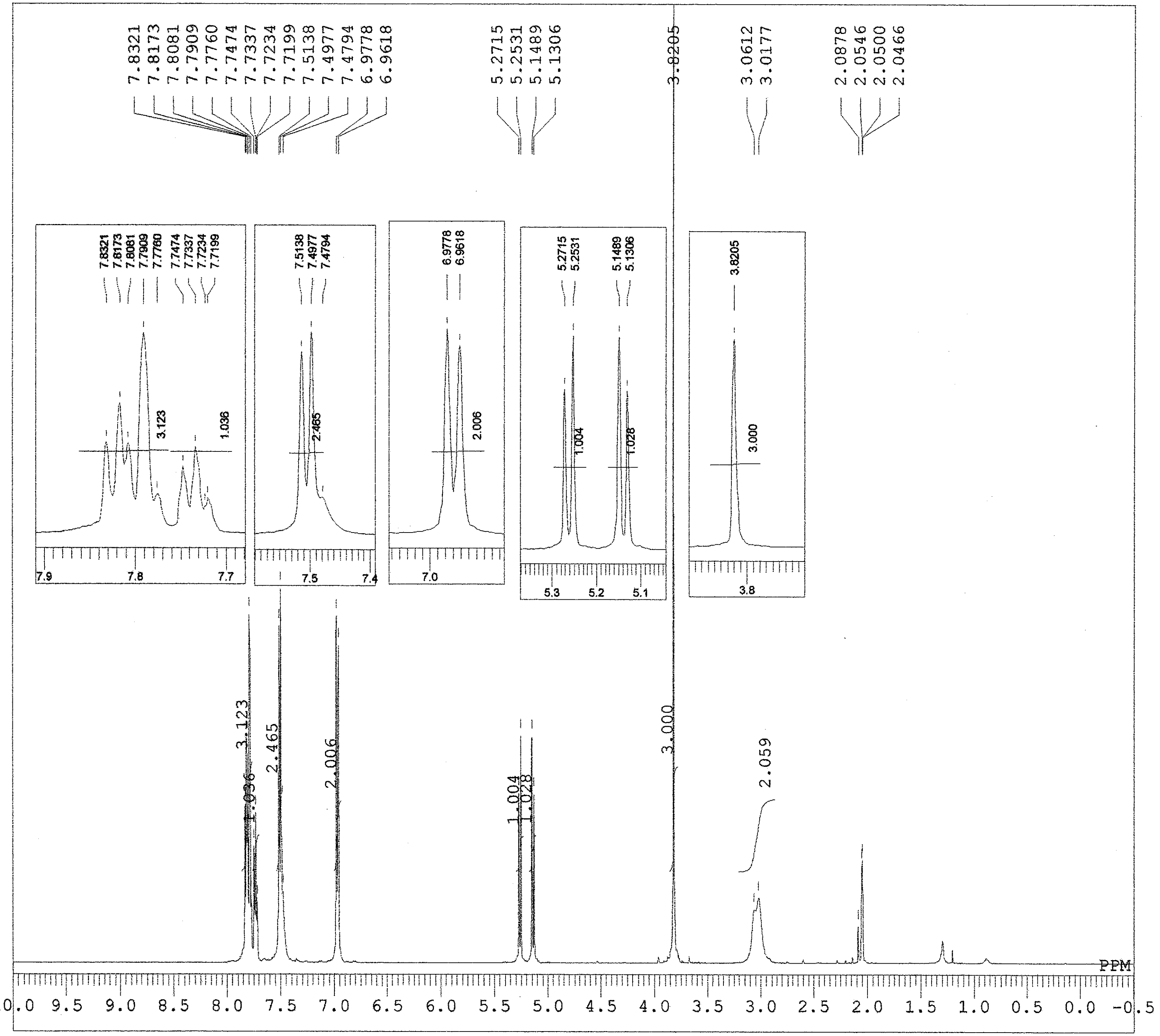

DFILE	ozawa05-028_1H.als
COMNT	PMB, CF3
DATIM	2014-06-04 19:09:38
OBNUC	1H
EXMOD	proton.jxp
OBFRQ	500.16 MHz
OBSET	2.41 KHz
OBFIN	6.01 Hz
POINT	13107
FREQU	7507.51 Hz
SCANS	4
ACQTM	1.7459 sec
PD	5.0000 sec
PW1	5.55 usec
IRNUC	1H
CTEMP	25.2 c
SLVNT	ACETN
EXREF	2.05 ppm
BF	0.12 Hz
RGAIN	30

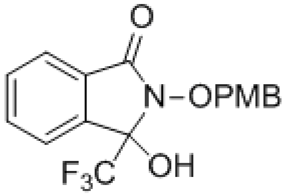

S2'

PMB, CF3

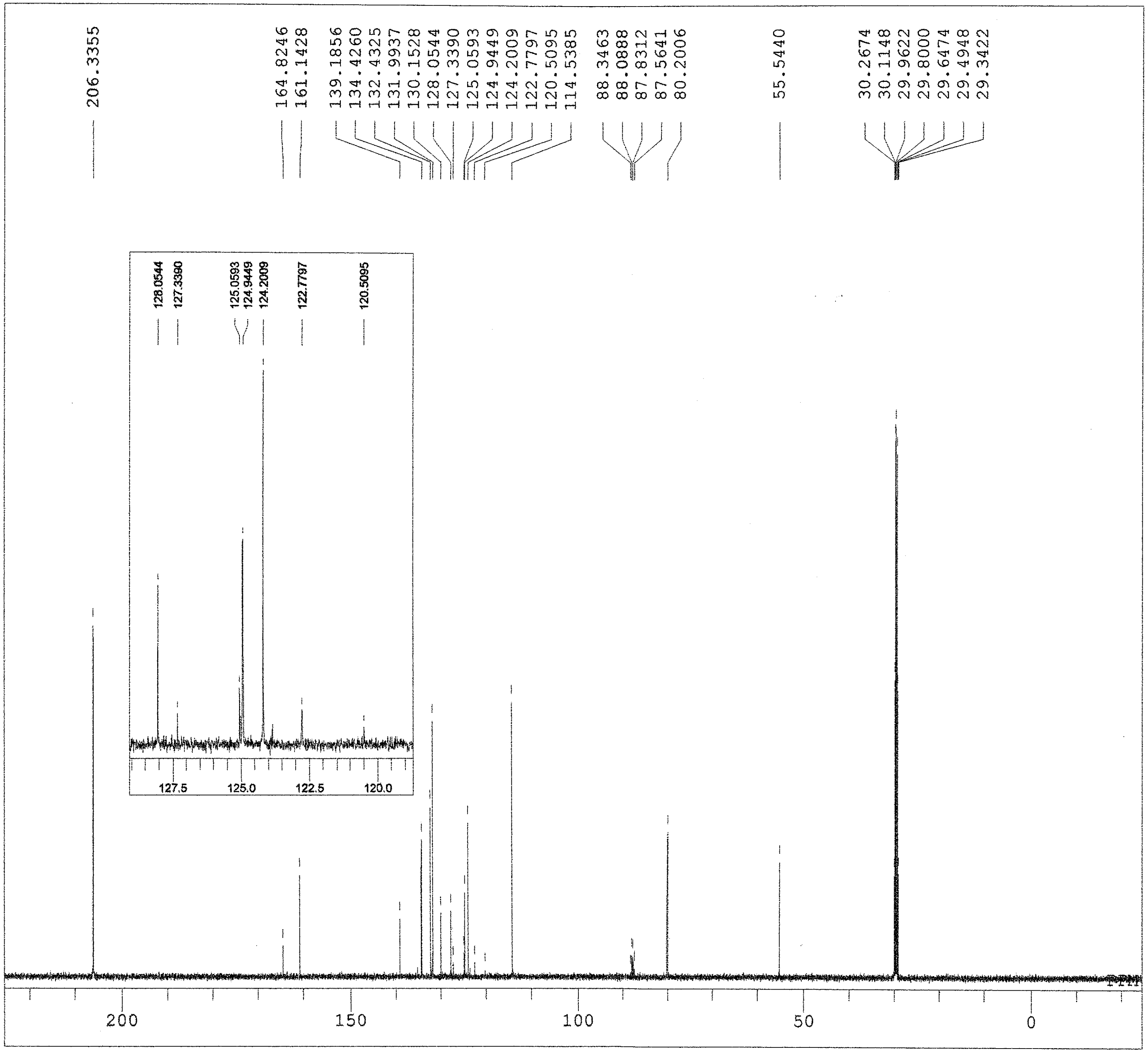

DFILE	ozawa05-028_13C.jdf
COMNT	PMB, CF3
DATIM	2014-06-04 19:10:42
OBNUC	13C
EXMOD	carbon.jxp
OBFRQ	125.77 MHz
OBSET	7.87 KHz
OBFIN	4.21 Hz
POINT	32767
FREQU	39308.18 Hz
SCANS	292
ACQTM	0.8336 sec
PD	3.0000 sec
PW1	3.40 usec
IRNUC	1H
CTEMP	25.9 c
SLVNT	ACETN
EXREF	29.80 ppm
BF	0.12 Hz
RGAIN	60

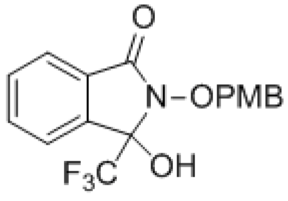

S2'

PMB, CF3-OH

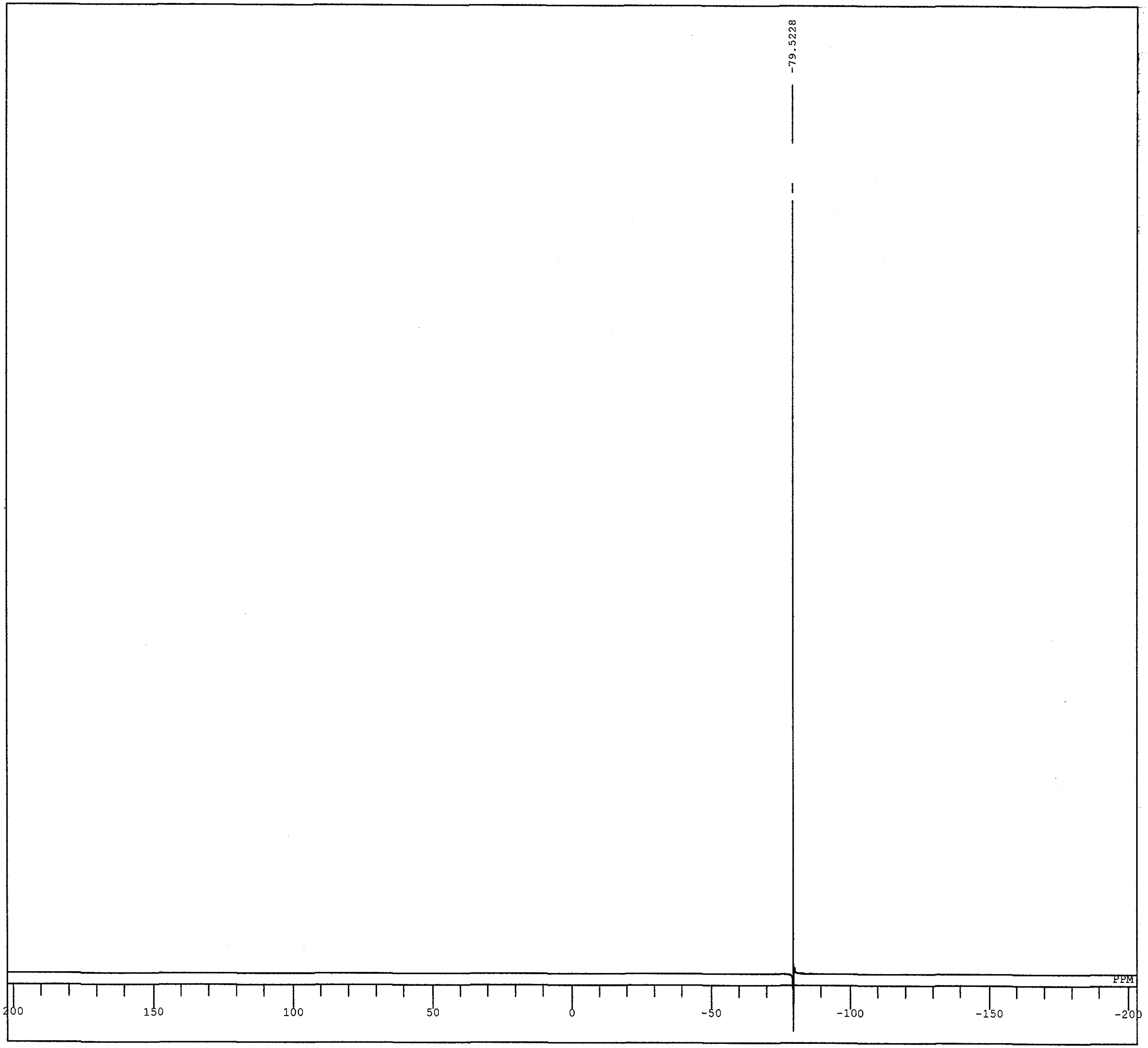

DFILE ozawa05-028_19F.jdf
COMNT PMB, CF3-OH
DATIM 06-06-2014 16:03:34
OBNUC 19F
EXMOD proton.jxp
OBFRQ 368.64 MHz
OBSET 7.63 KHz
OBFIN 2.85 Hz
POINT 16384
FREQU 186567.17 Hz
SCANS 4
ACQTM 0.0878 sec
PD 5.0000 sec
PW1 3.90 usec
IRNUC 19F
CTEMP 24.0 c
SLVNT ACETN
EXREF -164.90 ppm
BF 0.12 Hz
RGAIN 48

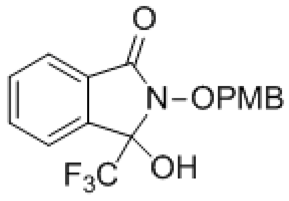

S2'

CF3-Cl, PMB

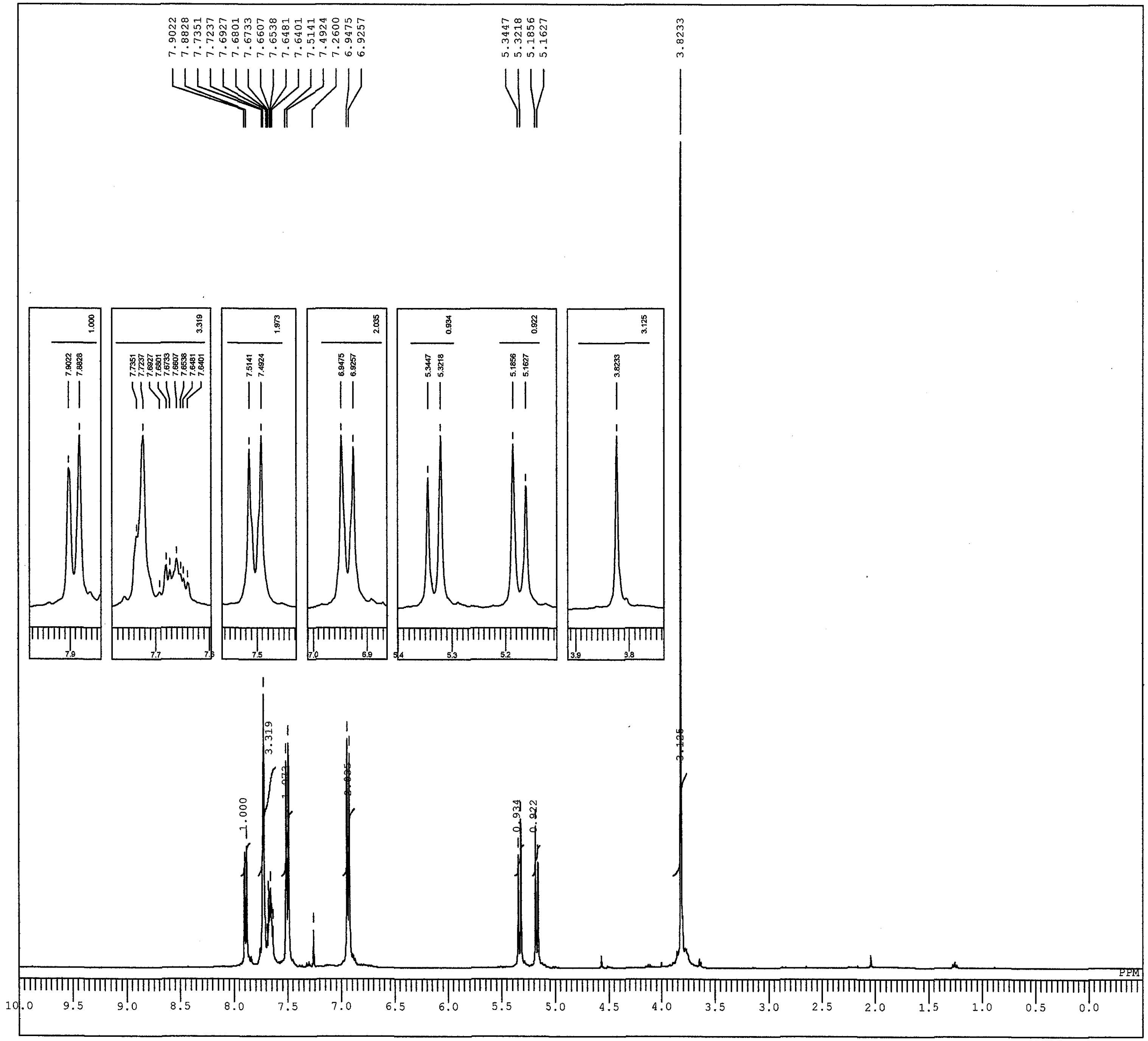


```
DFILE  ozawa05-030_1H.jdf
COMNT  CF3-Cl, PMB
DATIM  16-08-2014 12:34:12
OBNUC  1H
EXMOD  proton.jxp
OBFRQ        391.78 MHz
OBSET          8.51 KHz
OBFIN          3.34 Hz
POINT         16384
FREQU       7348.62 Hz
SCANS             4
ACQTM        2.2295 sec
PD           5.0000 sec
PW1            5.25 usec
IRNUC  1H
CTEMP          24.2 c
SLVNT  CDCL3
EXREF          7.26 ppm
BF             1.20 Hz
RGAIN            30
```

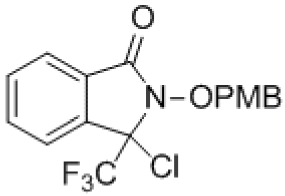

S3'

CF3-Cl, PMB

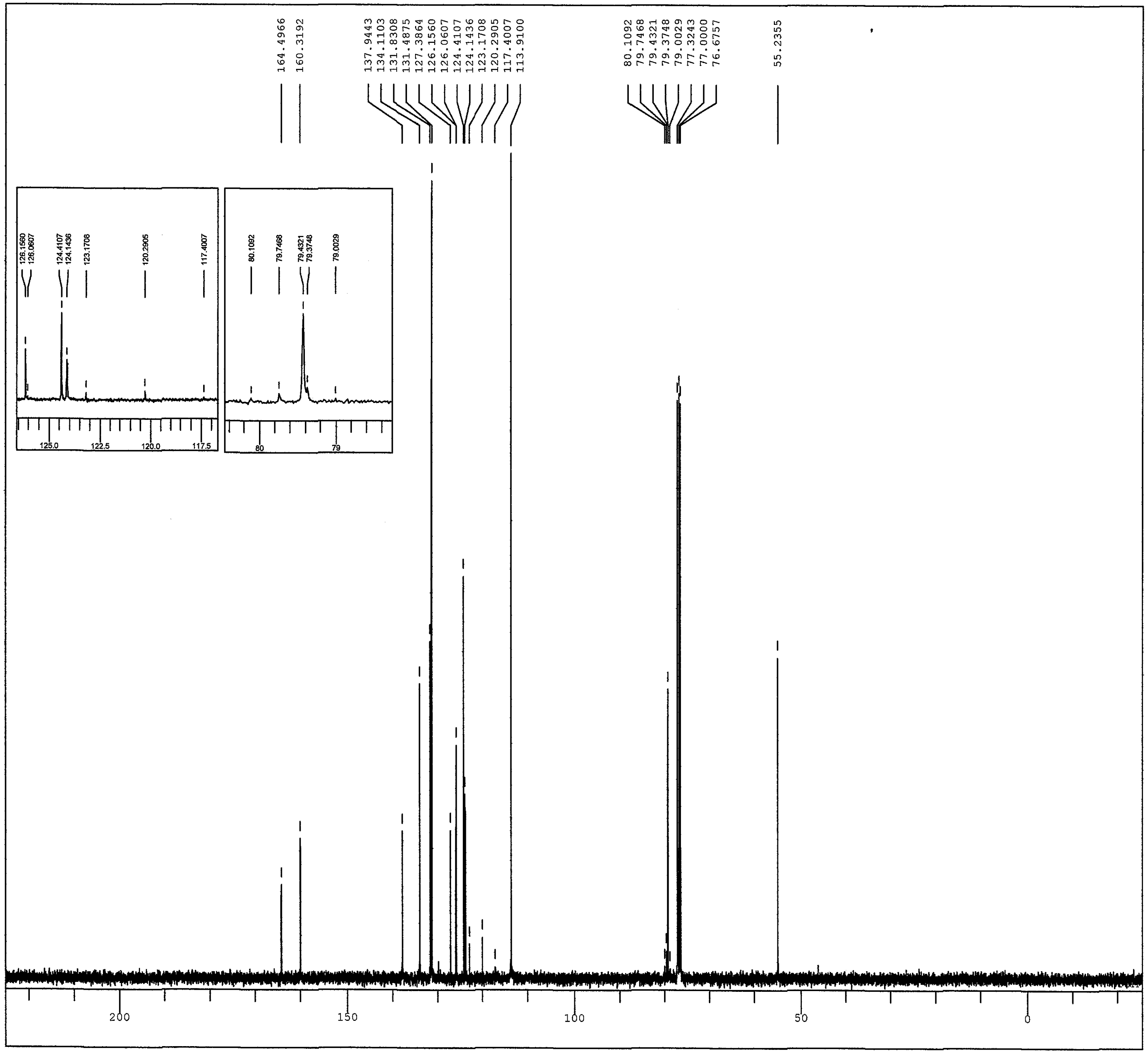

DFILE ozawa05-030_13C.jdf
COMNT CF3-Cl, PMB
DATIM 16-08-2014 12:35:19
OBNUC 13C
EXMOD carbon.jxp
OBFRQ 98.52 MHz
OBSET 4.64 KHz
OBFIN 8.74 Hz
POINT 32767
FREQU 30788.18 Hz
SCANS 200
ACQTM 1.0643 sec
PD 3.0000 sec
PW1 3.00 usec
IRNUC 1H
CTEMP 24.4 c
SLVNT CDCL3
EXREF 77.00 ppm
BF 1.00 Hz
RGAIN 60

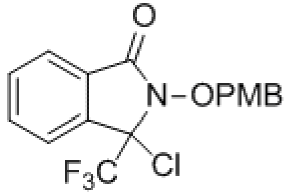

S3'

CF3-Cl, PMB

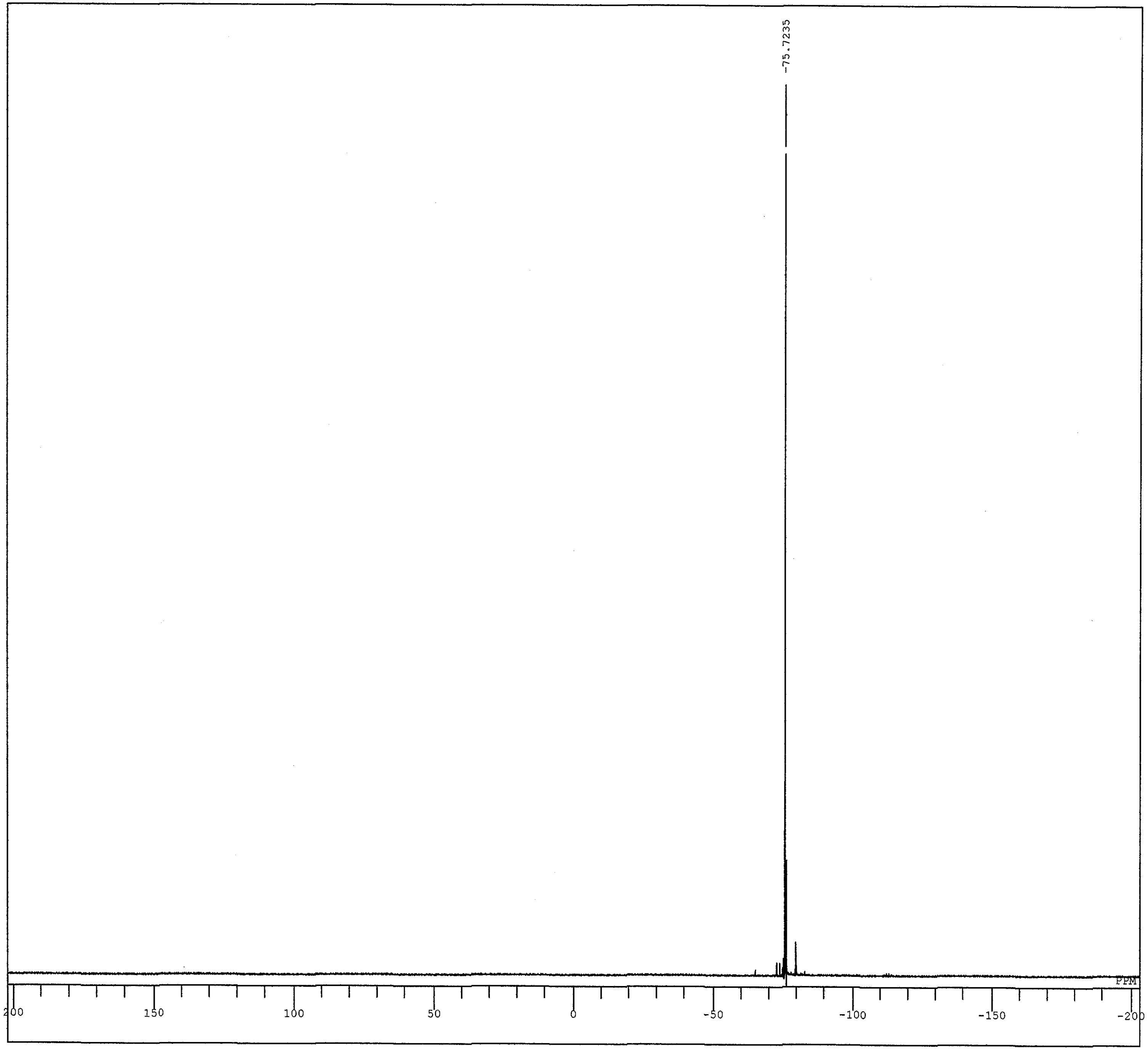

DFILE ozawa05-030_19F.jdf
COMNT CF3-Cl, PMB
DATIM 16-08-2014 12:32:11
OBNUC 19F
EXMOD proton.jxp
OBFRQ 368.64 MHz
OBSET 7.63 KHz
OBFIN 2.85 Hz
POINT 16384
FREQU 186567.17 Hz
SCANS 4
ACQTM 0.0878 sec
PD 5.0000 sec
PW1 3.90 usec
IRNUC 19F
CTEMP 24.1 c
SLVNT CDCL3
EXREF -164.90 ppm
BF 1.20 Hz
RGAIN 46

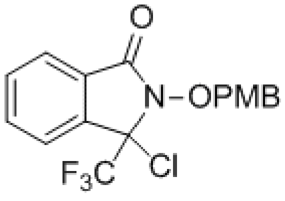

S3'

TMS-Et-NHPI

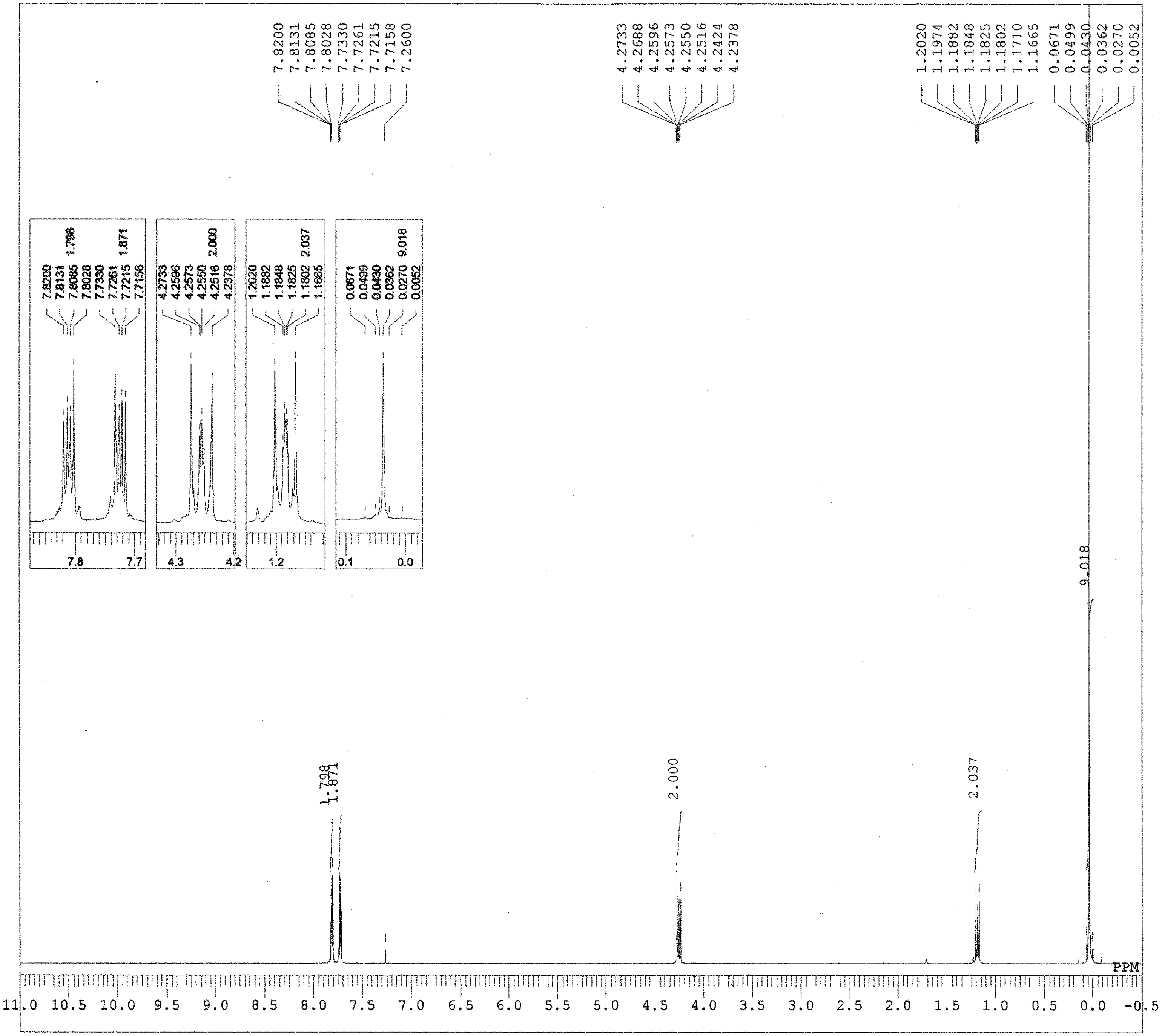

DFILE	ozawa07-066_1H.jdf
COMNT	TMS-Et-NHPI
DATIM	2015-04-01 10:10:32
OBNUC	1H
EXMOD	proton.jxp
OBFRQ	500.16 MHz
OBSET	2.41 KHz
OBFIN	6.01 Hz
POINT	16384
FREQU	9384.38 Hz
SCANS	4
ACQTM	1.7459 sec
PD	5.0000 sec
PW1	5.55 usec
IRNUC	1H
CTEMP	27.8 c
SLVNT	CDCL3
EXREF	7.26 ppm
BF	0.12 Hz
RGAIN	26

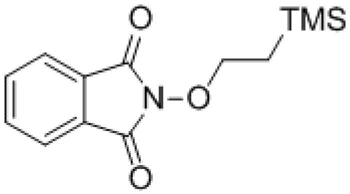

S1''

TMS-Et-NHPI

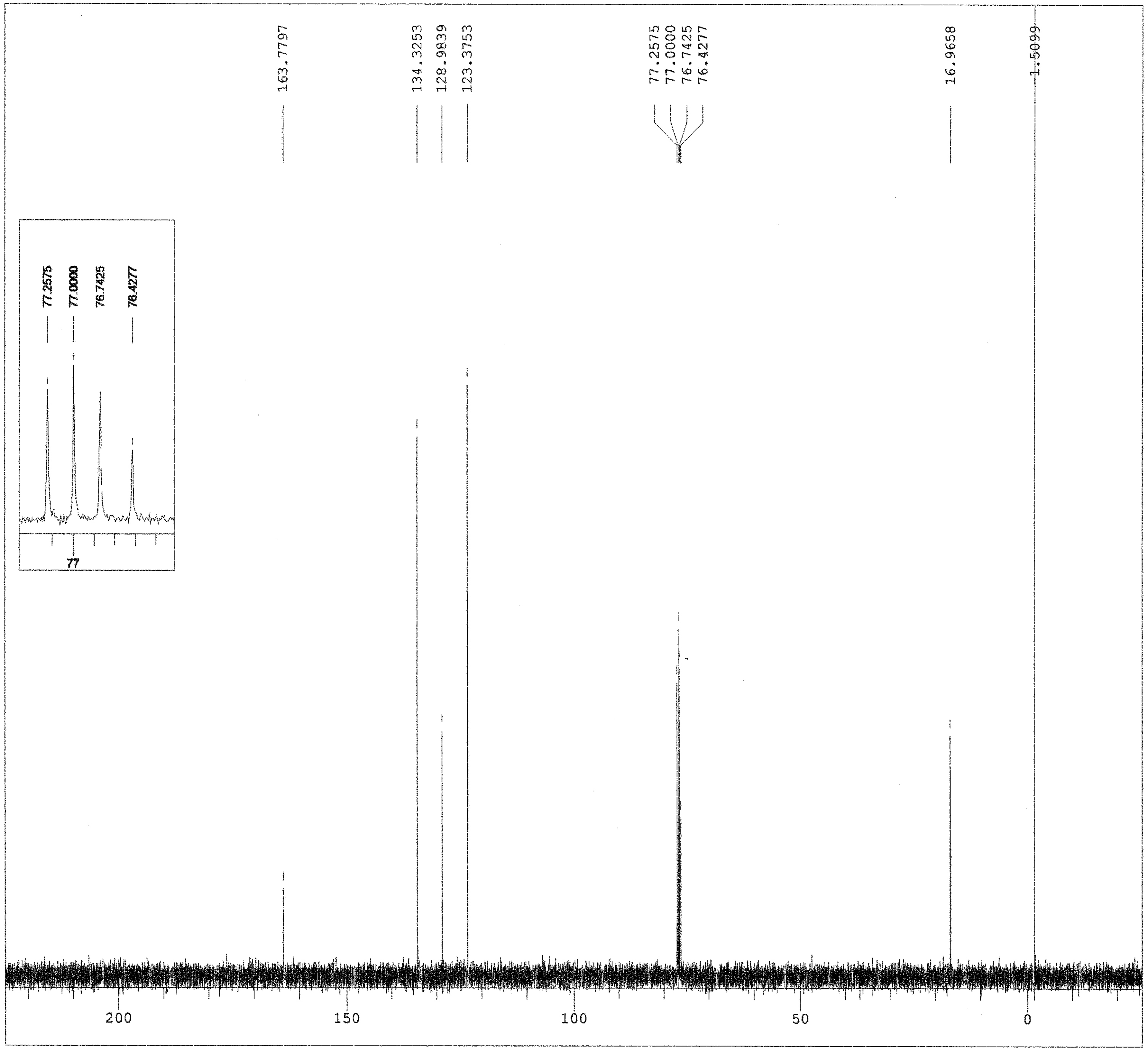

DFILE	ozawa07-066_13C.jdf
COMNT	TMS-Et-NHPI
DATIM	2015-04-01 10:11:34
OBNUC	13C
EXMOD	carbon.jxp
OBFRQ	125.77 MHz
OBSET	7.87 KHz
OBFIN	4.21 Hz
POINT	32767
FREQU	39308.18 Hz
SCANS	16
ACQTM	0.8336 sec
PD	2.0000 sec
PW1	3.40 usec
IRNUC	1H
CTEMP	27.9 c
SLVNT	CDCL3
EXREF	77.00 ppm
BF	0.12 Hz
RGAIN	60

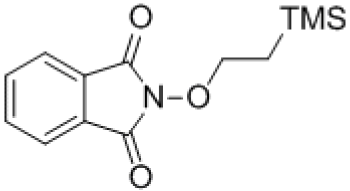

S1''

TMS-Et, CF3-OH

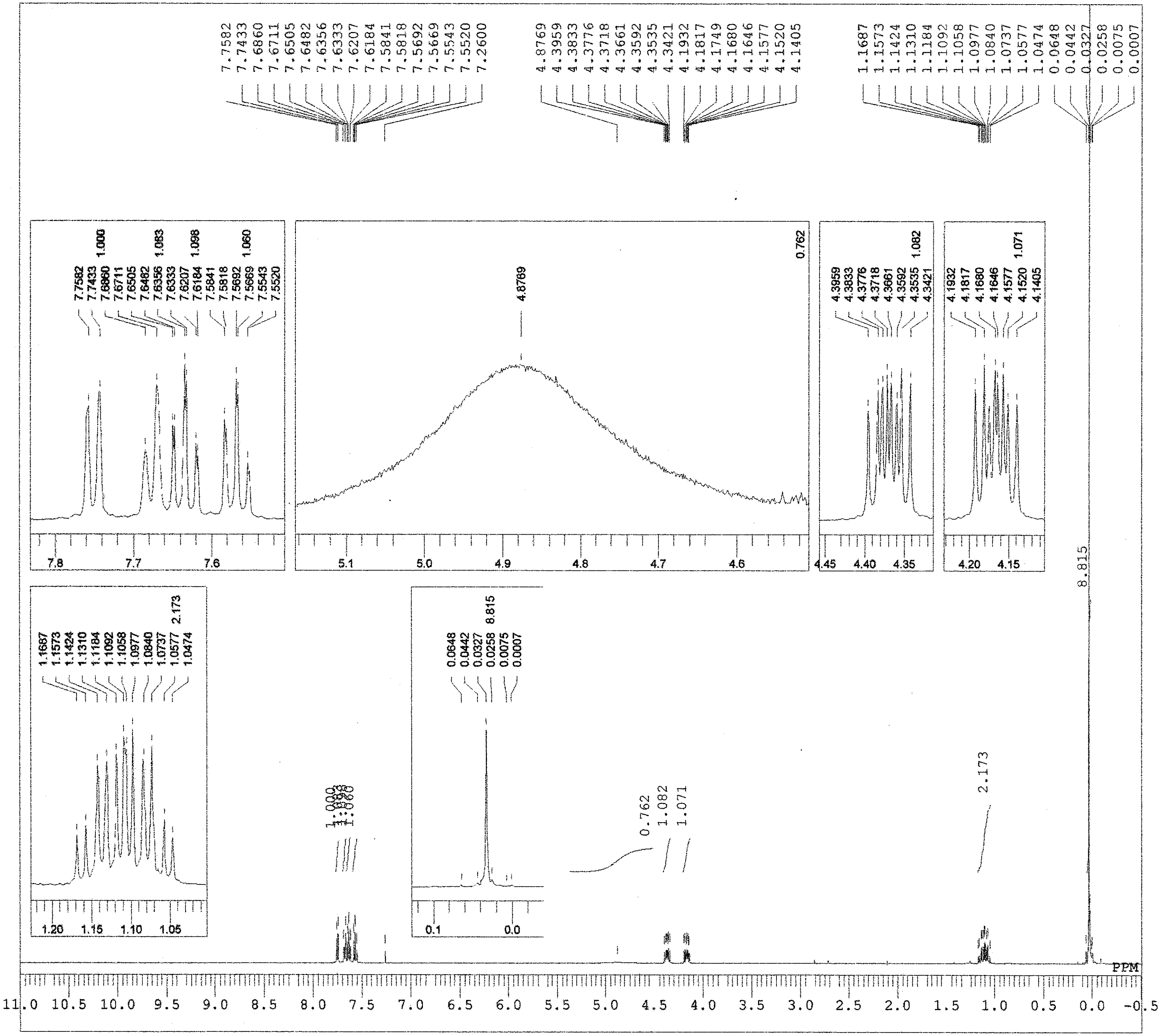

DFILE	ozawa06-56_1H.jdf
COMNT	TMS-Et, CF3-OH
DATIM	2015-04-01 15:15:38
OBNUC	1H
EXMOD	proton.jxp
OBFRQ	500.16 MHz
OBSET	2.41 KHz
OBFIN	6.01 Hz
POINT	16384
FREQU	9384.38 Hz
SCANS	4
ACQTM	1.7459 sec
PD	5.0000 sec
PW1	5.55 usec
IRNUC	1H
CTEMP	28.0 c
SLVNT	CDCL3
EXREF	7.26 ppm
BF	0.12 Hz
RGAIN	28

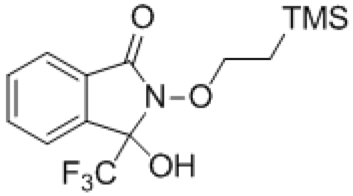

S2''

TMS-Et, CF3-OH

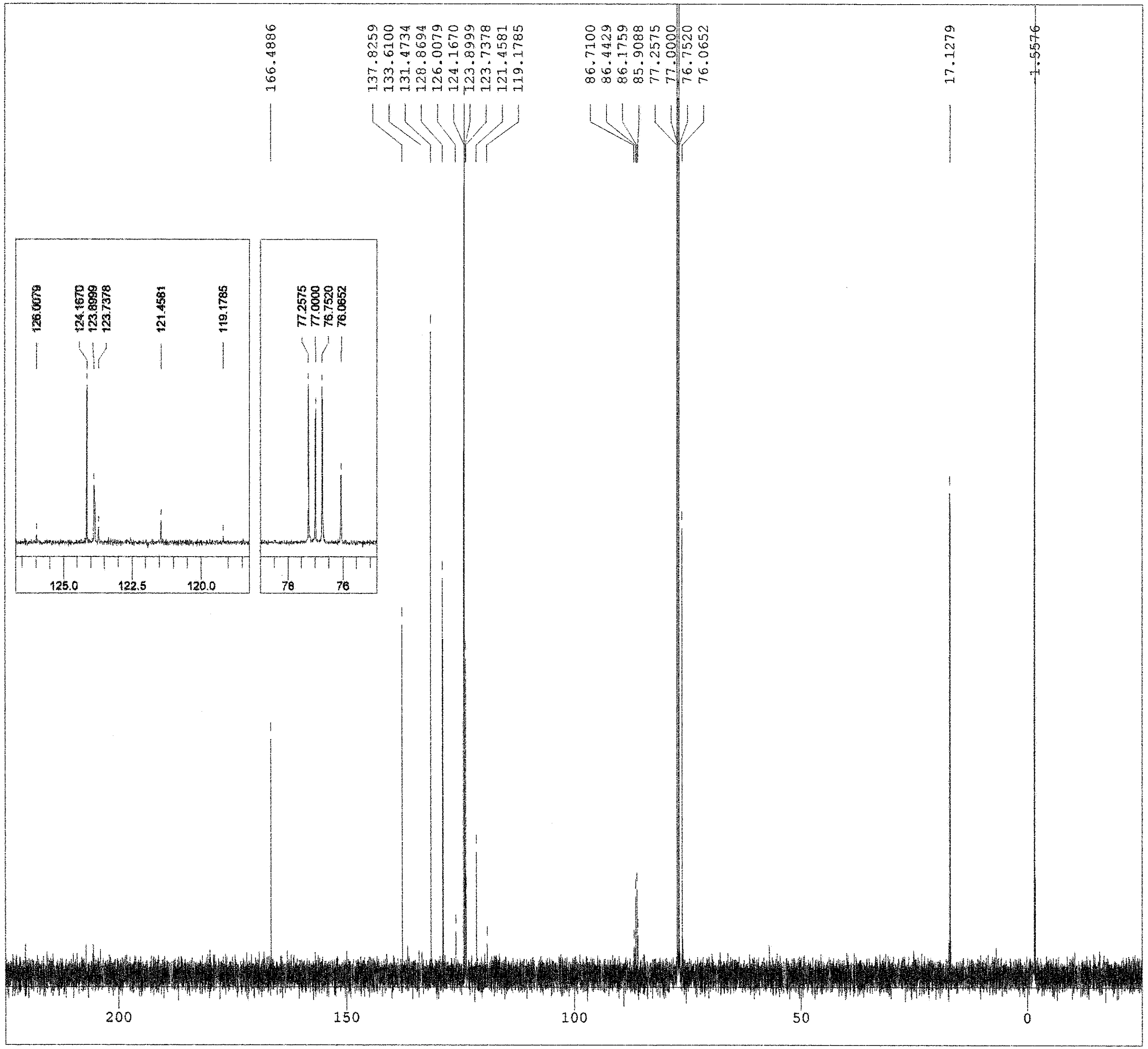

DFILE	ozawa06-56_13C.jdf
COMNT	TMS-Et, CF3-OH
DATIM	2015-04-01 15:16:41
OBNUC	13C
EXMOD	carbon.jxp
OBFRQ	125.77 MHz
OBSET	7.87 KHz
OBFIN	4.21 Hz
POINT	32767
FREQU	39308.18 Hz
SCANS	180
ACQTM	0.8336 sec
PD	2.0000 sec
PW1	3.40 usec
IRNUC	1H
CTEMP	28.4 c
SLVNT	CDCL3
EXREF	77.00 ppm
BF	0.12 Hz
RGAIN	60

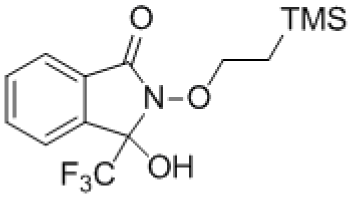

S2''

TMS-Et, CF3-OH

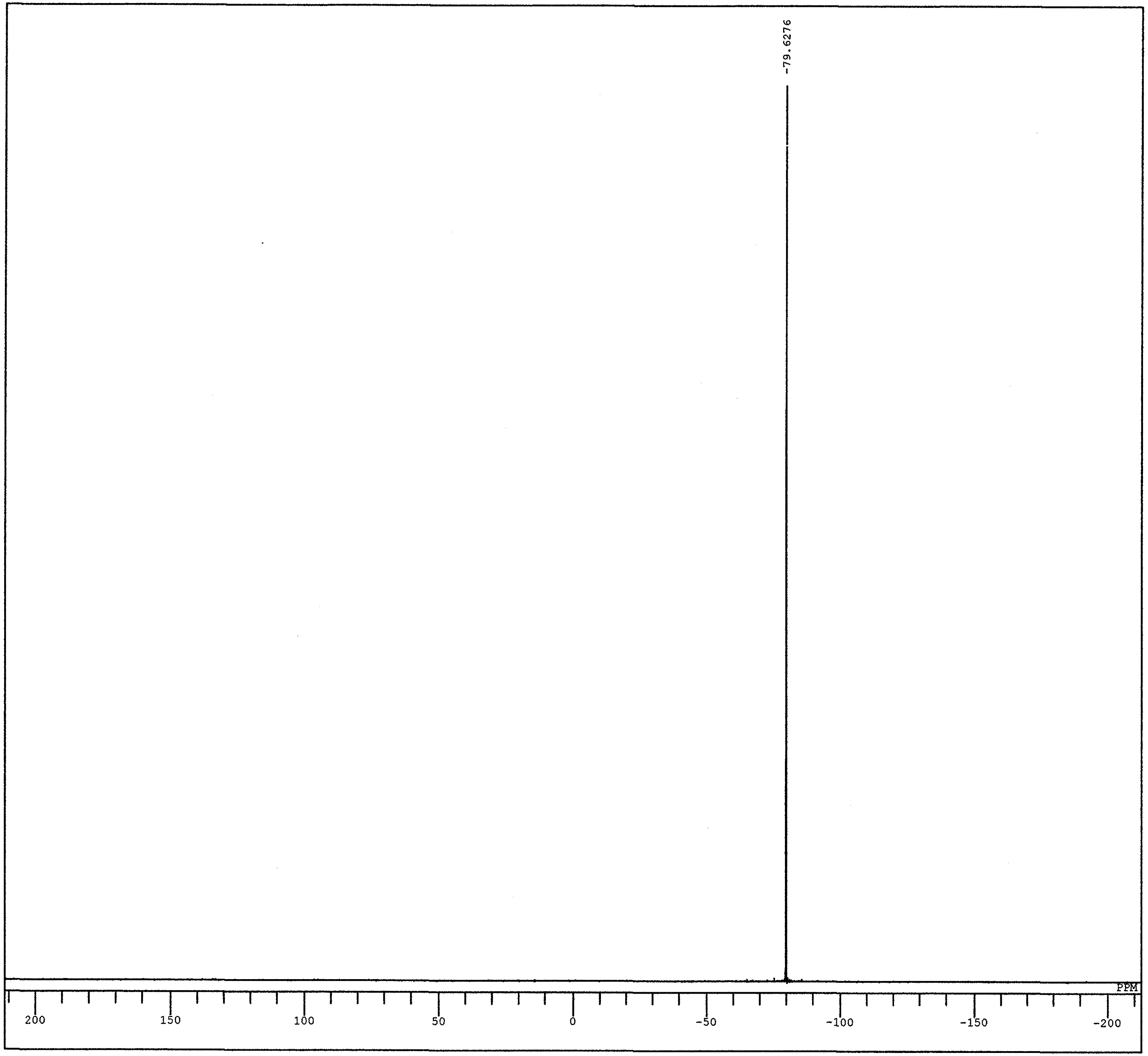


```
DFILE  ozawa07-056_19F.jdf
COMNT  TMS-Et, CF3-OH
DATIM  01-04-2015 16:39:46
OBNUC  19F
EXMOD  proton.jxp
OBFRQ        368.64 MHz
OBSET          7.63 KHz
OBFIN          2.85 Hz
POINT        16384
FREQU     195312.50 Hz
SCANS            4
ACQTM        0.0839 sec
PD           5.0000 sec
PW1            3.90 usec
IRNUC  19F
CTEMP          26.7 c
SLVNT  CDCL3
EXREF       -164.90 ppm
BF             0.12 Hz
RGAIN            48
```

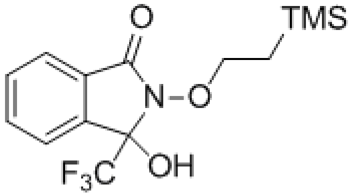

S2''

TMS-Et, CF3-Cl

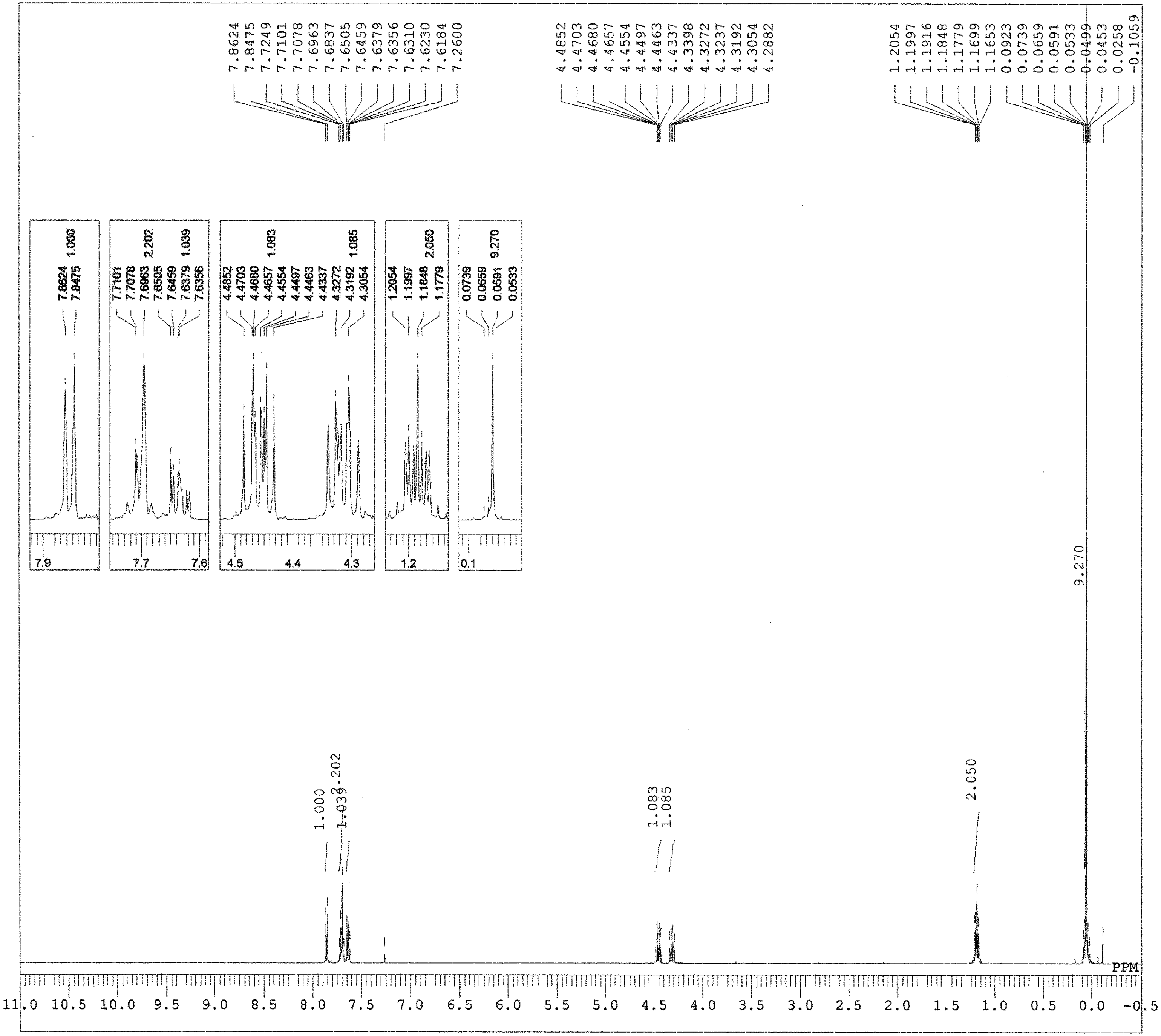

DFILE	KO08-129_1H.jdf
COMNT	TMS-Et, CF3-Cl
DATIM	2015-04-01 15:06:01
OBNUC	1H
EXMOD	proton.jxp
OBFRQ	500.16 MHz
OBSET	2.41 KHz
OBFIN	6.01 Hz
POINT	16384
FREQU	9384.38 Hz
SCANS	4
ACQTM	1.7459 sec
PD	5.0000 sec
PW1	5.55 usec
IRNUC	1H
CTEMP	27.9 c
SLVNT	CDCL3
EXREF	7.26 ppm
BF	0.12 Hz
RGAIN	26

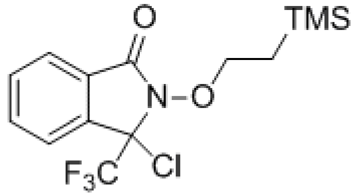

S3''

TMS-Et, CF3-Cl

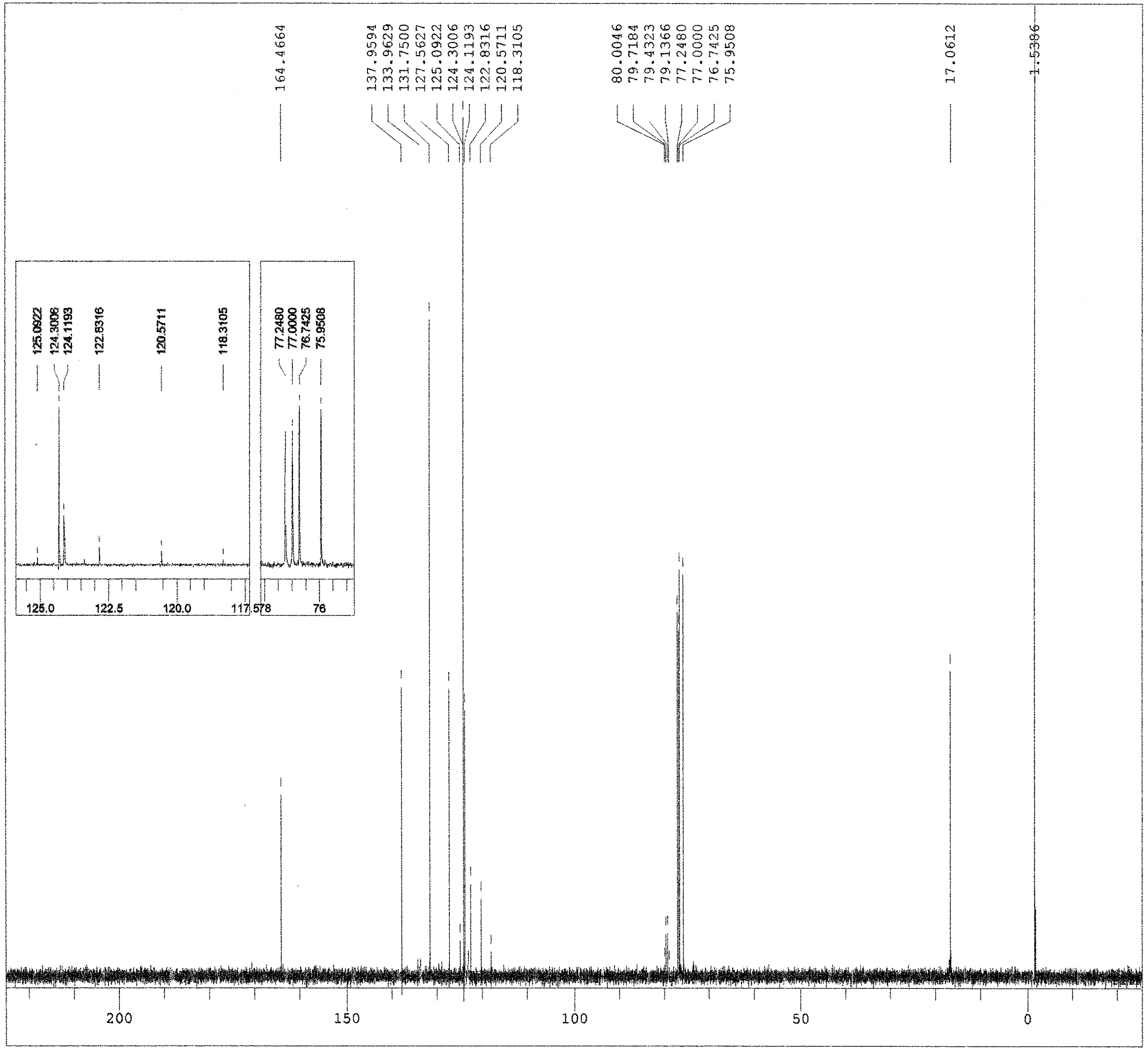

DFILE	KO08-129_13C.jdf
COMNT	TMS-Et, CF3-Cl
DATIM	2015-04-01 15:07:04
OBNUC	13C
EXMOD	carbon.jxp
OBFRQ	125.77 MHz
OBSET	7.87 KHz
OBFIN	4.21 Hz
POINT	32767
FREQU	39308.18 Hz
SCANS	112
ACQTM	0.8336 sec
PD	2.0000 sec
PW1	3.40 usec
IRNUC	1H
CTEMP	28.2 c
SLVNT	CDCL3
EXREF	77.00 ppm
BF	0.12 Hz
RGAIN	60

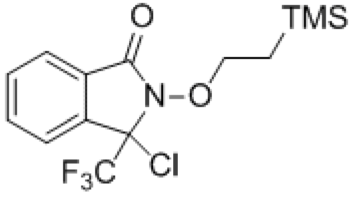

S3"

TMS-Et, CF3-Cl

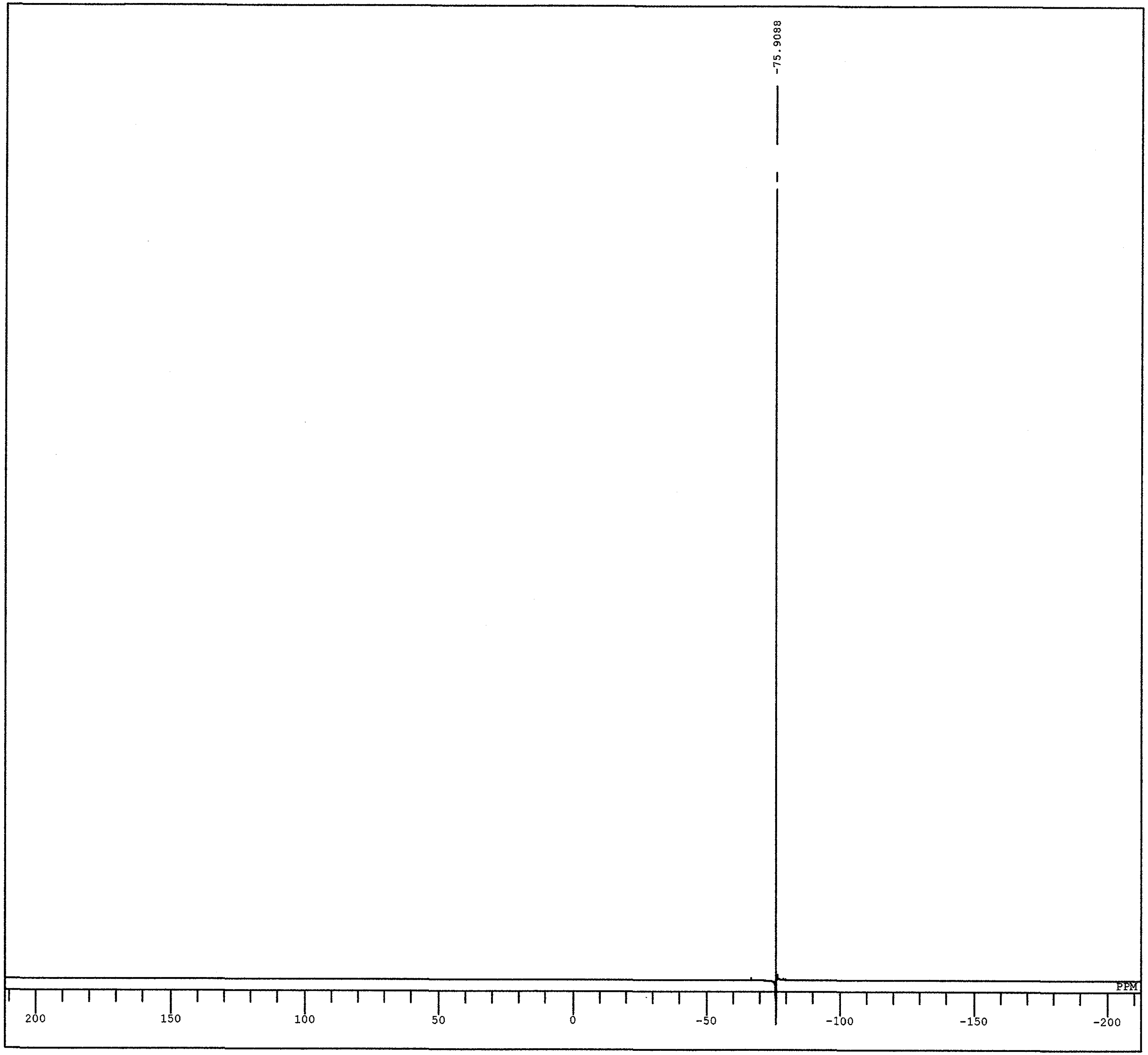


```
DFILE  KO08-129_19F.jdf
COMNT  TMS-Et, CF3-Cl
DATIM  01-04-2015 16:36:52
OBNUC  19F
EXMOD  proton.jxp
OBFRQ        368.64 MHz
OBSET          7.63 KHz
OBFIN          2.85 Hz
POINT         16384
FREQU     195312.50 Hz
SCANS             4
ACQTM        0.0839 sec
PD           5.0000 sec
PW1            3.90 usec
IRNUC  19F
CTEMP          26.8 c
SLVNT  CDCL3
EXREF       -164.90 ppm
BF             0.12 Hz
RGAIN            46
```

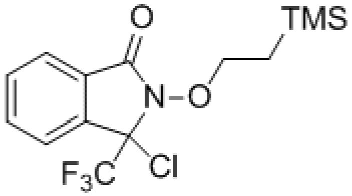

S3"

BuOH, Bn

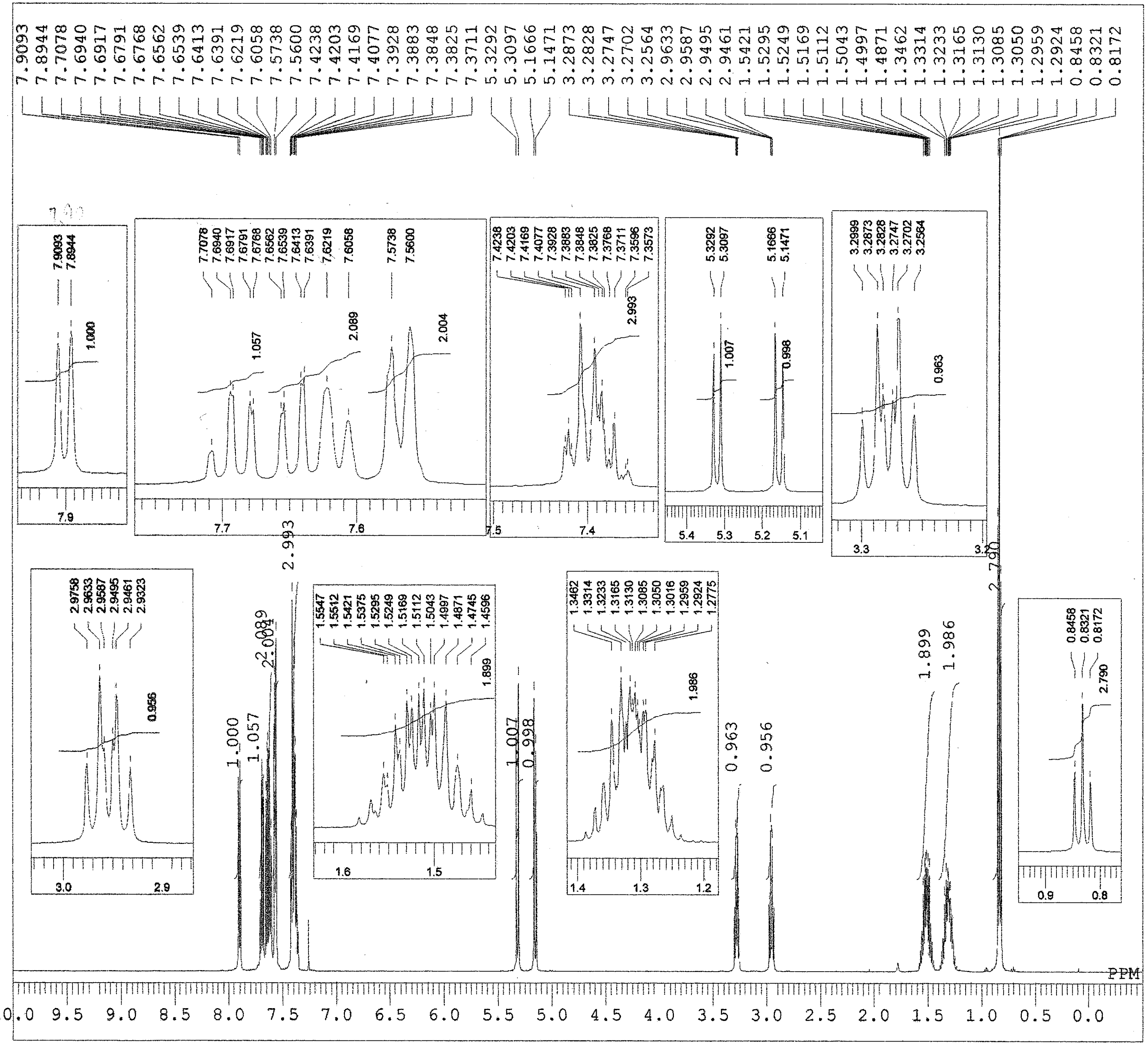

DFILE	ozawa04-104_1H.jdf
COMNT	BuOH, Bn
DATIM	2014-01-16 15:05:58
OBNUC	1H
EXMOD	proton.jxp
OBFRQ	500.16 MHz
OBSET	2.41 KHz
OBFIN	6.01 Hz
POINT	16384
FREQU	9384.38 Hz
SCANS	4
ACQTM	1.7459 sec
PD	5.0000 sec
PW1	5.55 usec
IRNUC	1H
CTEMP	21.2 c
SLVNT	CDCL3
EXREF	7.26 ppm
BF	0.12 Hz
RGAIN	28

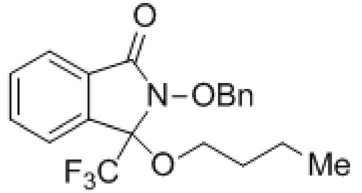

S4a

BuOH, Bn

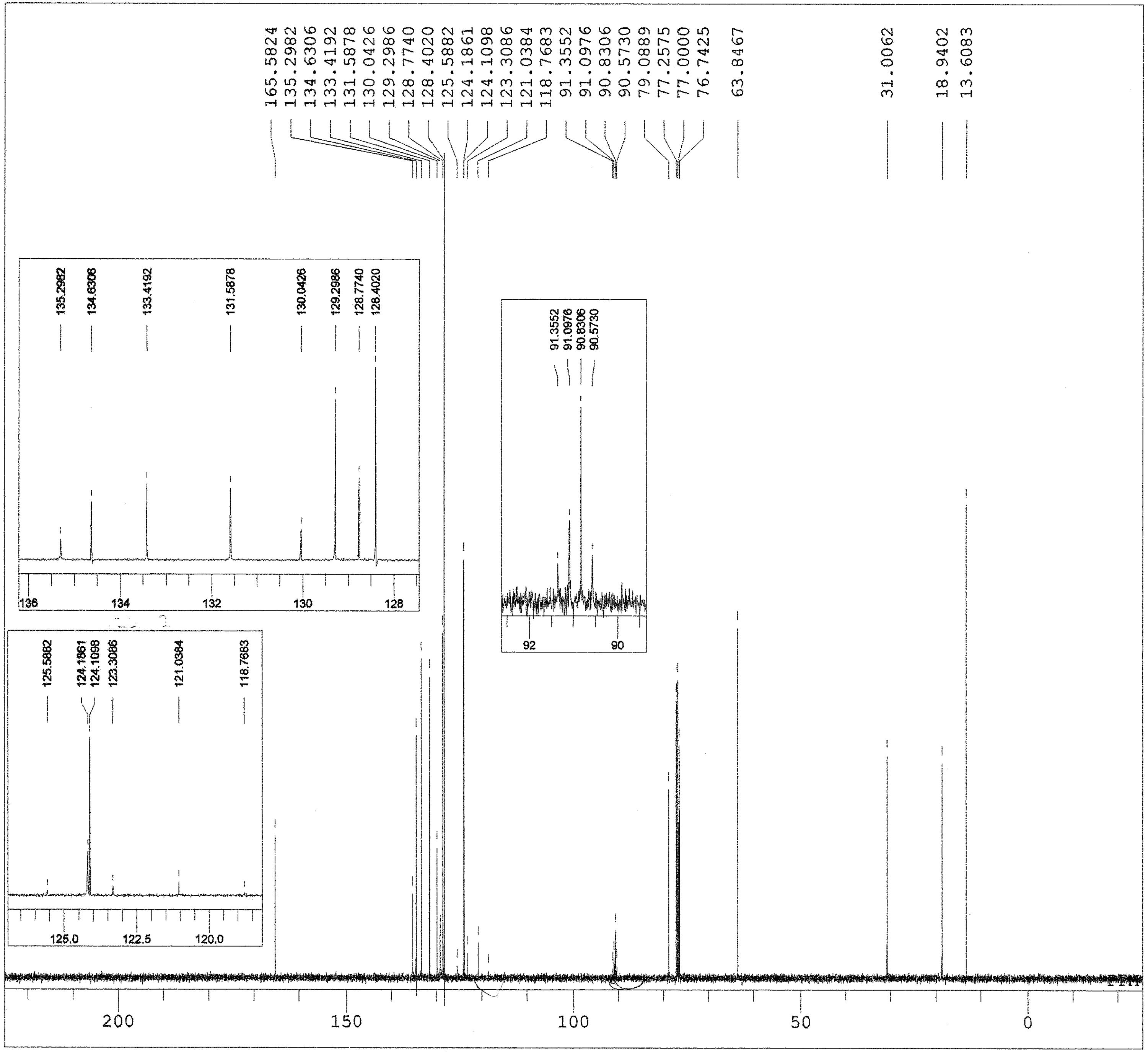

DFILE ozawa04-104_13C.jdf
COMNT BuOH, Bn
DATIM 2014-01-16 15:07:01
OBNUC 13C
EXMOD carbon.jxp
OBFRQ 125.77 MHz
OBSET 7.87 KHz
OBFIN 4.21 Hz
POINT 32767
FREQU 39308.18 Hz
SCANS 152
ACQTM 0.8336 sec
PD 3.0000 sec
PW1 3.40 usec
IRNUC 1H
CTEMP 21.7 c
SLVNT CDCL3
EXREF 77.00 ppm
BF 0.12 Hz
RGAIN 60

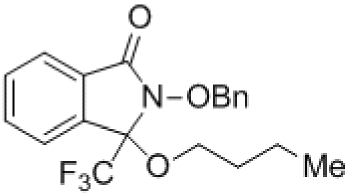

S4a

BuOH, Bn

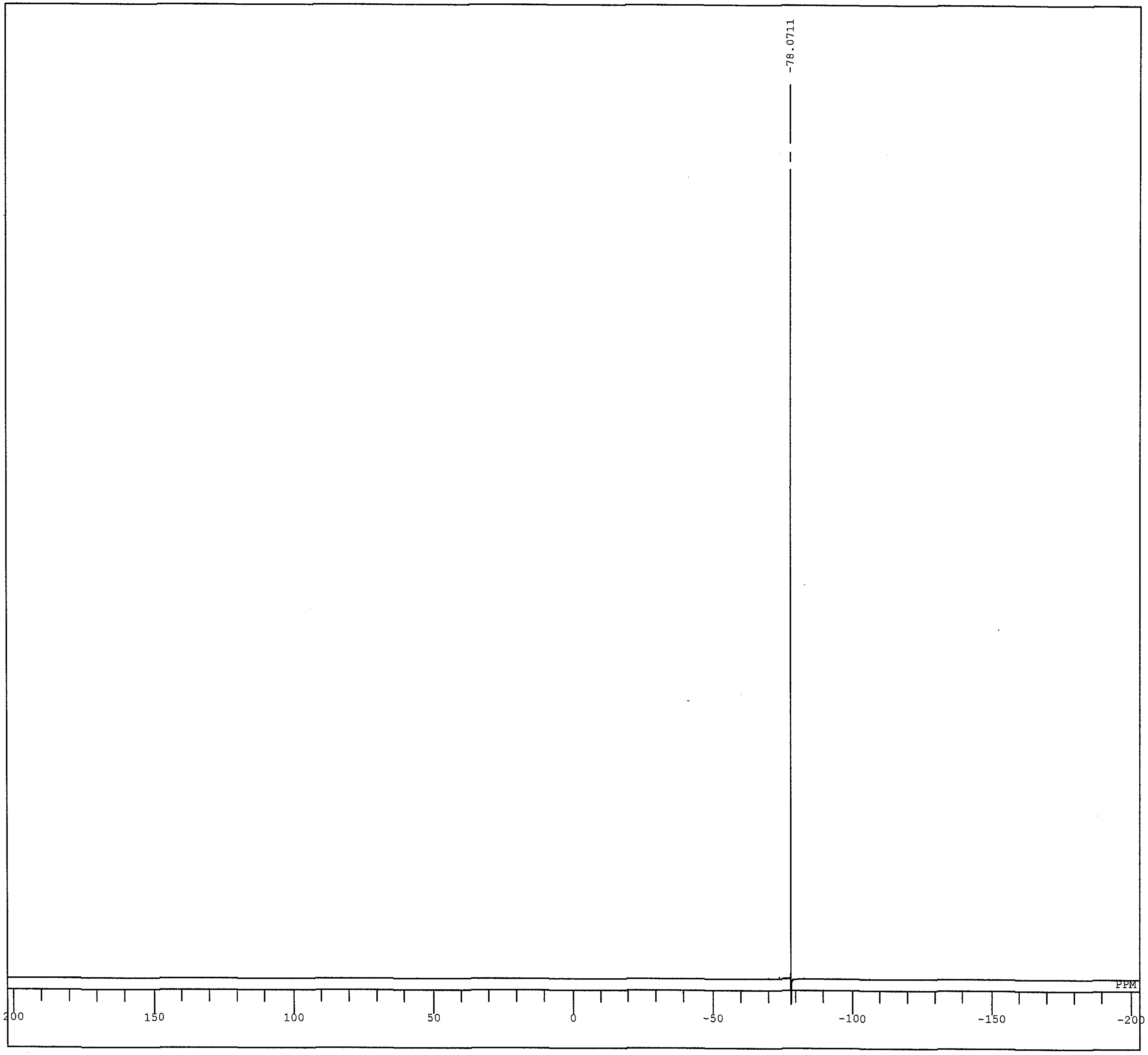

DFILE ozawa04-104_19F.jdf
COMNT BuOH, Bn
DATIM 16-01-2014 15:46:24
OBNUC 19F
EXMOD proton.jxp
OBFRQ 368.64 MHz
OBSET 7.63 KHz
OBFIN 2.85 Hz
POINT 16384
FREQU 186567.17 Hz
SCANS 4
ACQTM 0.0878 sec
PD 5.0000 sec
PW1 3.90 usec
IRNUC 19F
CTEMP 20.8 c
SLVNT CDCL3
EXREF -164.90 ppm
BF 0.12 Hz
RGAIN 44

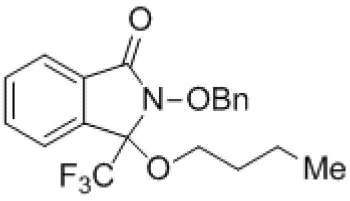

S4a

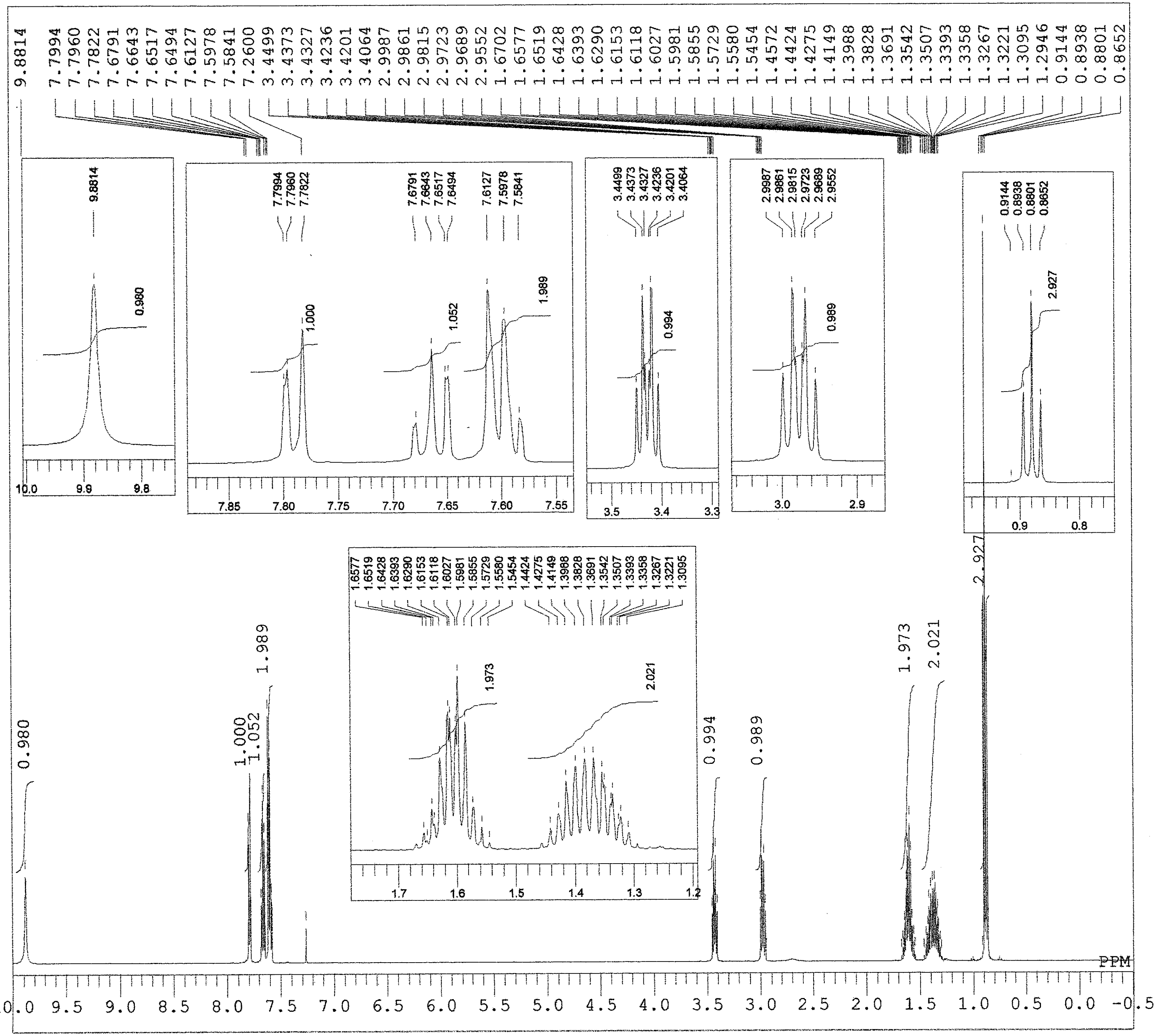

DFILE	ozawa03-092_1H.als
COMNT	
DATIM	2013-11-14 19:52:17
OBNUC	1H
EXMOD	proton.jxp
OBFRQ	500.16 MHz
OBSET	2.41 KHz
OBFIN	6.01 Hz
POINT	13107
FREQU	7507.51 Hz
SCANS	4
ACQTM	1.7459 sec
PD	5.0000 sec
PW1	5.55 usec
IRNUC	1H
CTEMP	22.9 c
SLVNT	CDCL3
EXREF	7.26 ppm
BF	0.12 Hz
RGAIN	26

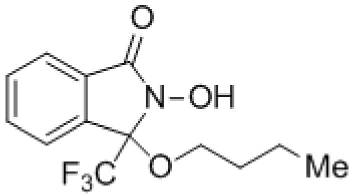

1a

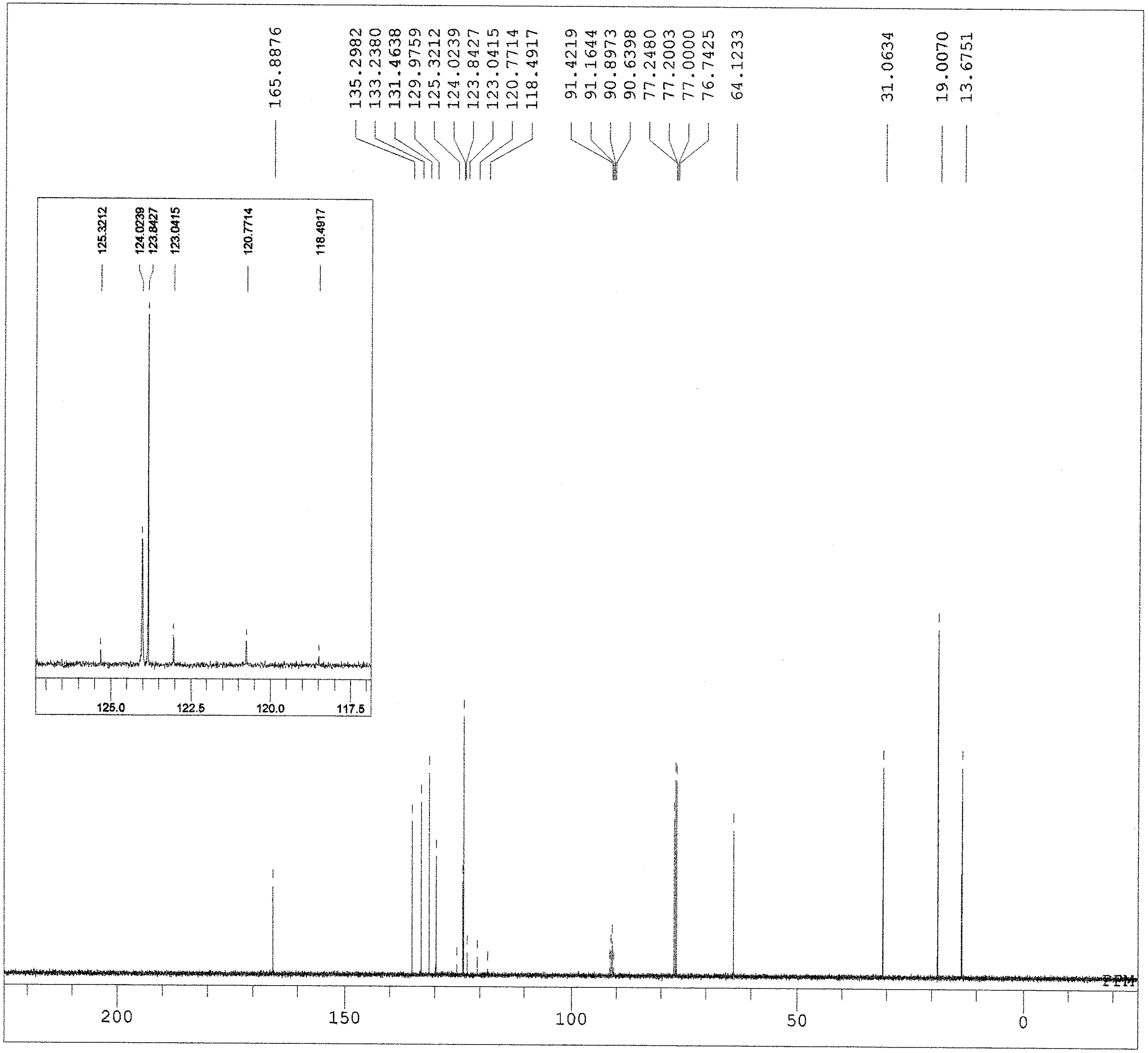

DFILE	ozawa03-092_13C.jdf
COMNT	
DATIM	2013-11-14 19:53:44
OBNUC	13C
EXMOD	carbon.jxp
OBFRQ	125.77 MHz
OBSET	7.87 KHz
OBFIN	4.21 Hz
POINT	32767
FREQU	39308.18 Hz
SCANS	160
ACQTM	0.8336 sec
PD	3.0000 sec
PW1	3.40 usec
IRNUC	1H
CTEMP	23.4 c
SLVNT	CDCL3
EXREF	77.00 ppm
BF	0.12 Hz
RGAIN	60

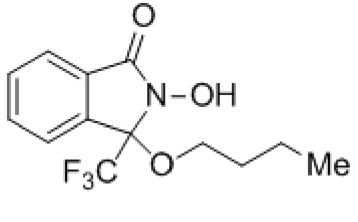

1a

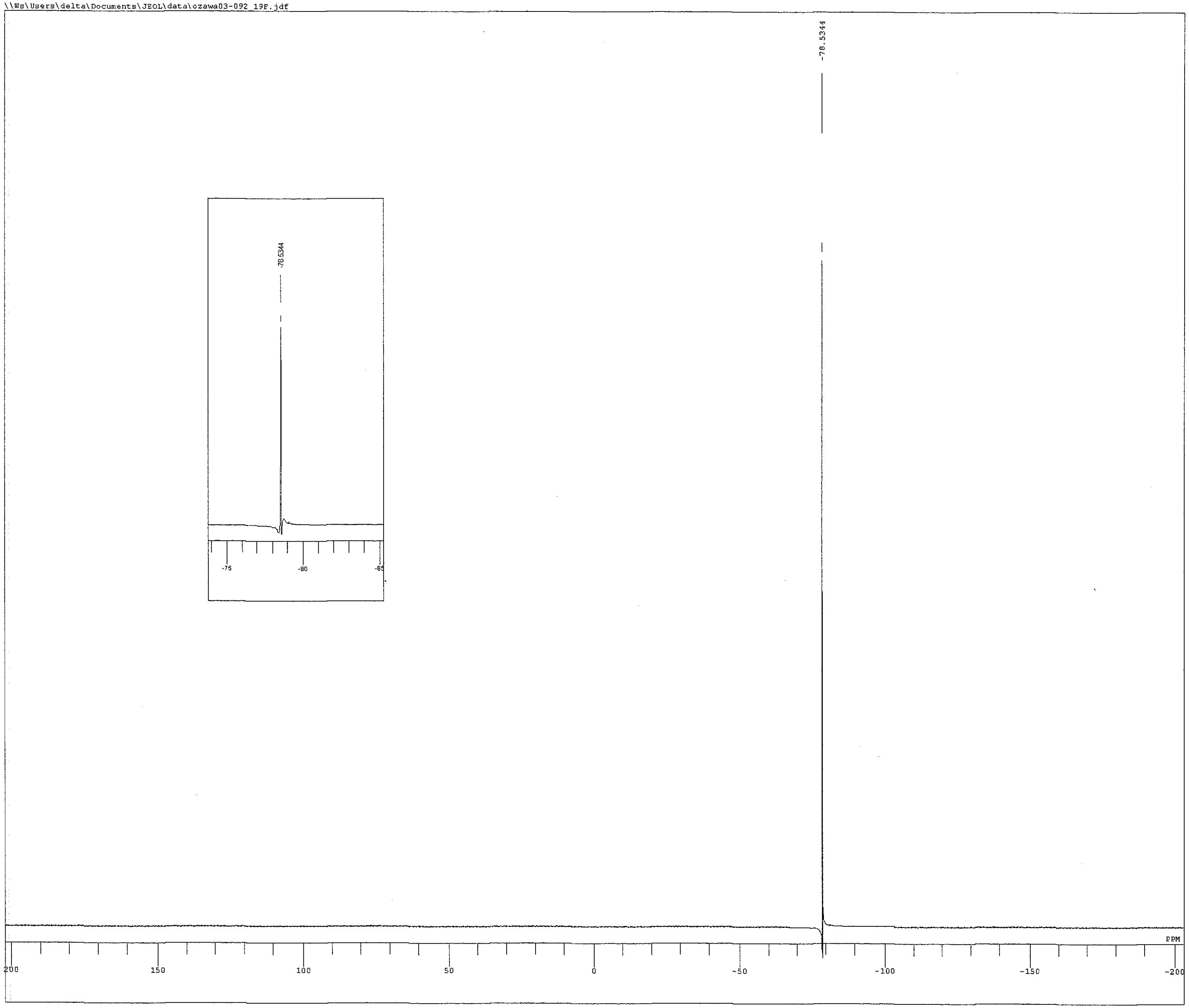


```
DFILE  ozawa03-092_19F.jdf
COMNT
DATIM  14-11-2013 20:23:21
OBNUC  19F
EXMOD  proton.jxp
OBFRQ        368.64 MHz
OBSET          7.63 KHz
OBFIN          2.85 Hz
POINT         16384
FREQU     186567.17 Hz
SCANS             8
ACQTM        0.0878 sec
PD           5.0000 sec
PW1            3.90 usec
IRNUC  19F
CTEMP          23.0 c
SLVNT  CDCL3
EXREF       -164.90 ppm
BF             0.12 Hz
RGAIN            44
```

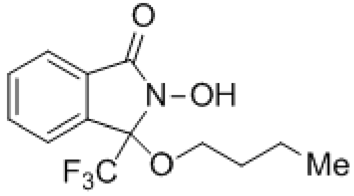

1a

BuOH, [O]

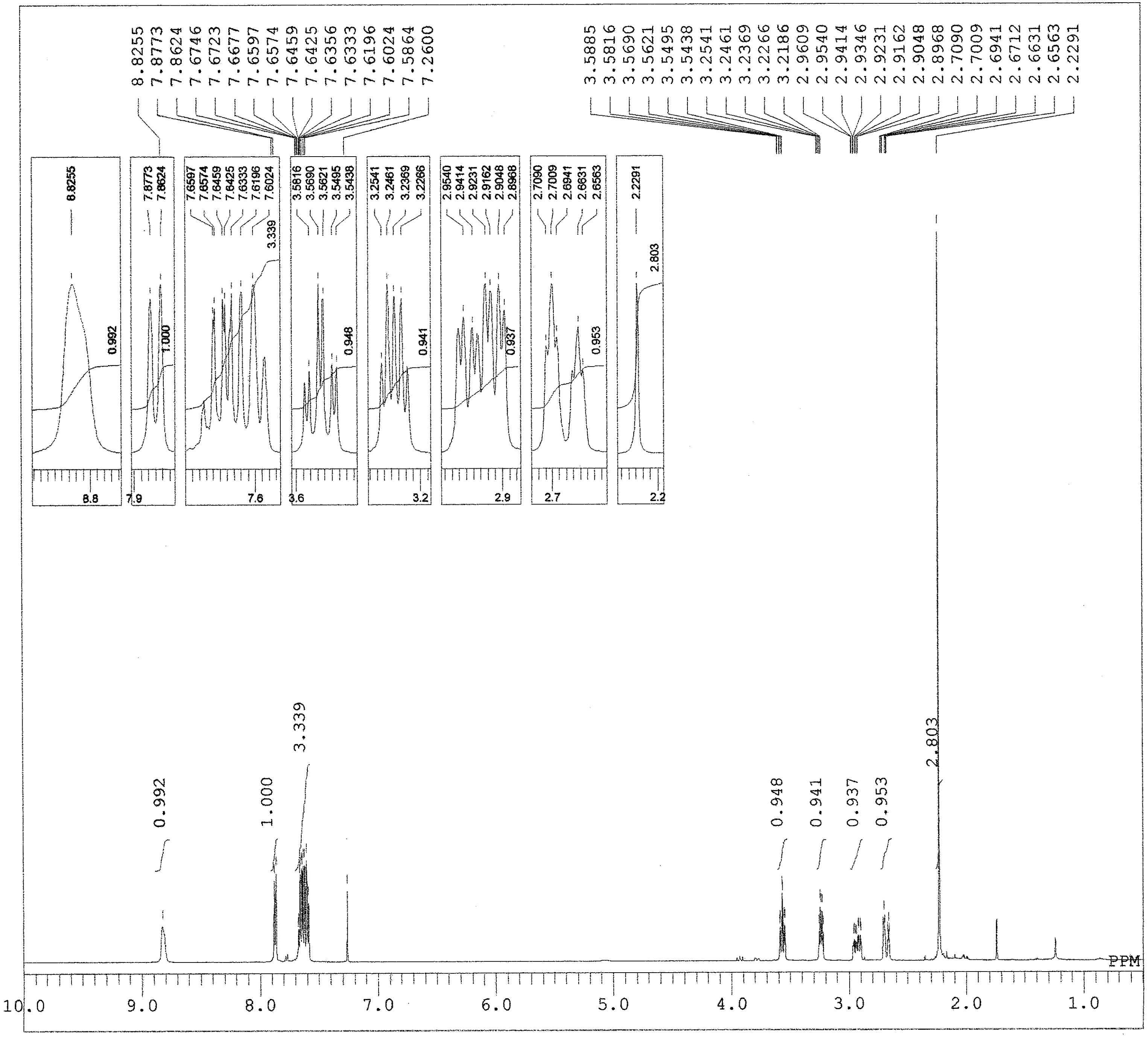

DFILE	ozawa04-106_1H.als
COMNT	BuOH, [O]
DATIM	2014-01-21 11:59:18
OBNUC	1H
EXMOD	proton.jxp
OBFRQ	500.16 MHz
OBSET	2.41 KHz
OBFIN	6.01 Hz
POINT	13107
FREQU	7507.51 Hz
SCANS	4
ACQTM	1.7459 sec
PD	5.0000 sec
PW1	5.55 usec
IRNUC	1H
CTEMP	21.3 c
SLVNT	CDCL3
EXREF	7.26 ppm
BF	0.12 Hz
RGAIN	36

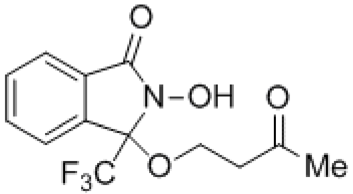

2a

BuOH, O2

\\172.20.1.201\users\delta\Documents\JEOL\data\ozawa04-106_13C.jdf

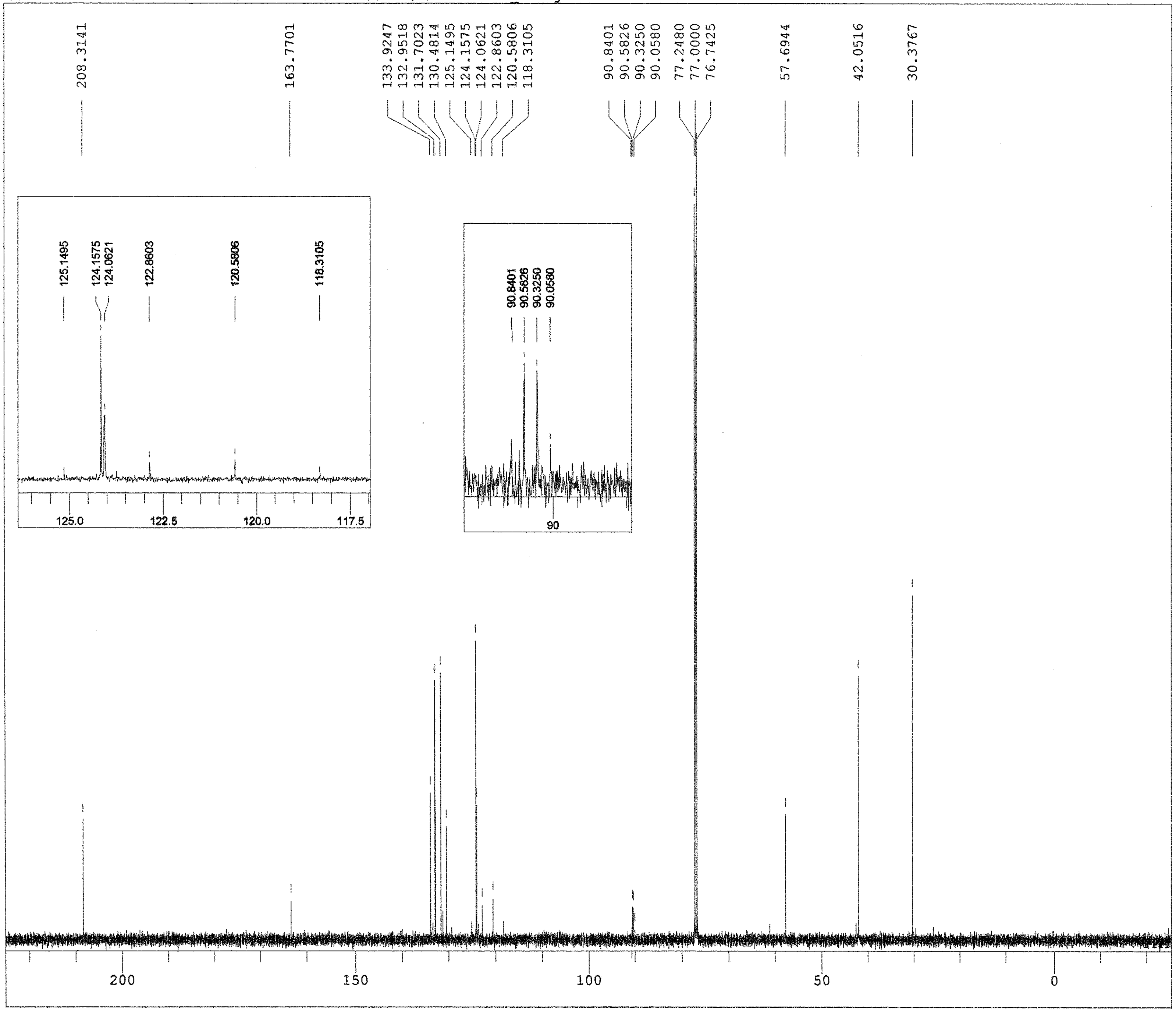

DFILE ozawa04-106_13C.jdf
COMNT BuOH, O2
DATIM 2014-01-21 12:00:35
OBNUC 13C
EXMOD carbon.jxp
OBFRQ 125.77 MHz
OBSET 7.87 KHz
OBFIN 4.21 Hz
POINT 32767
FREQU 39308.18 Hz
SCANS 400
ACQTM 0.8336 sec
PD 3.0000 sec
PW1 3.40 usec
IRNUC 1H
CTEMP 21.9 c
SLVNT CDCL3
EXREF 77.00 ppm
BF 0.12 Hz
RGAIN 60

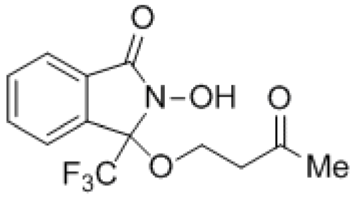

2a

BuOH, [O]

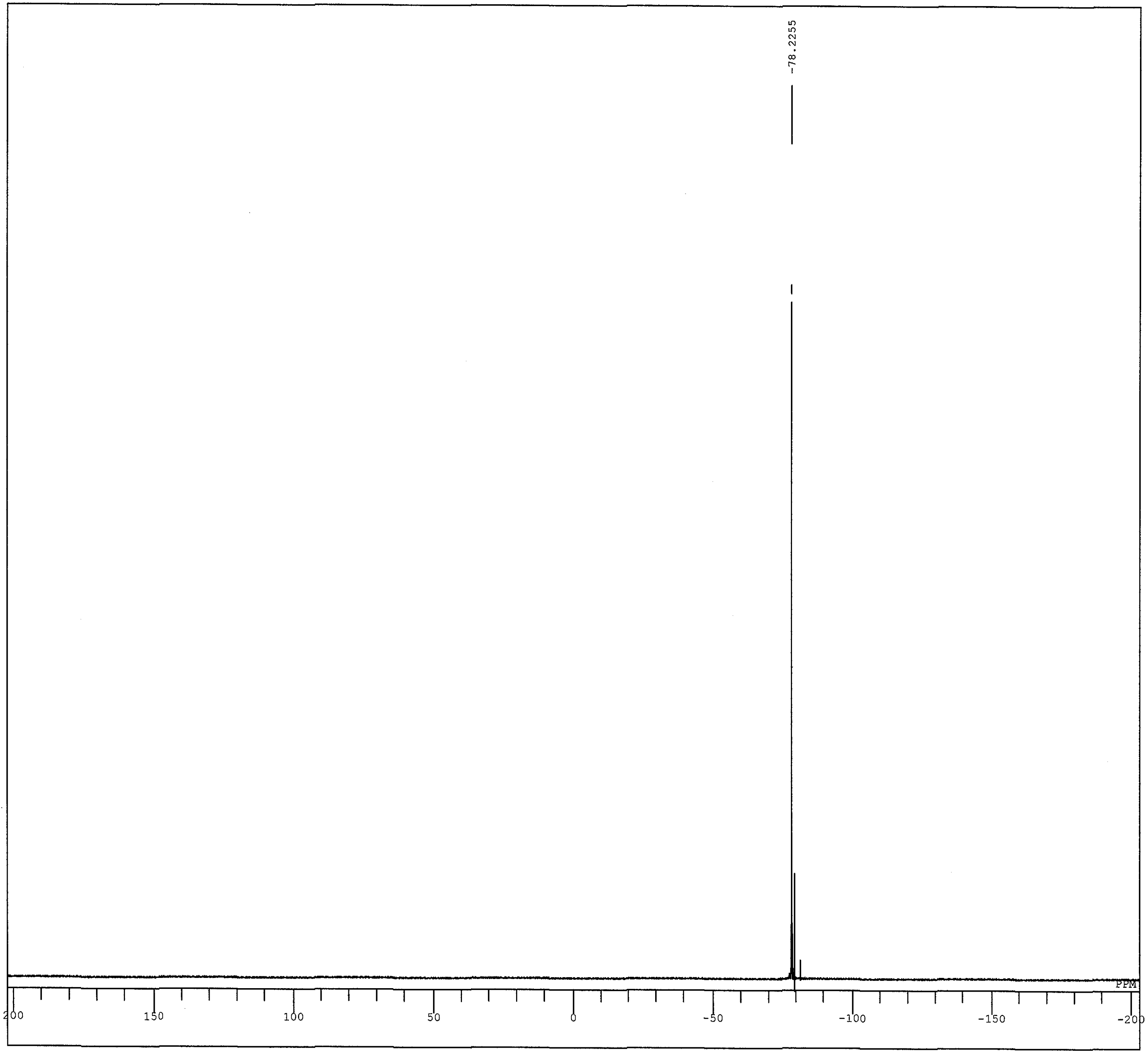


```
DFILE  2a_ozawa04-106_19F.jdf
COMNT  BuOH, [O]
DATIM  21-01-2014 13:09:20
OBNUC  19F
EXMOD  proton.jxp
OBFRQ        368.64 MHz
OBSET          7.63 KHz
OBFIN          2.85 Hz
POINT         16384
FREQU     186567.17 Hz
SCANS             4
ACQTM        0.0878 sec
PD           5.0000 sec
PW1            3.90 usec
IRNUC  19F
CTEMP          20.9 c
SLVNT  CDCL3
EXREF       -164.90 ppm
BF             0.12 Hz
RGAIN            46
```

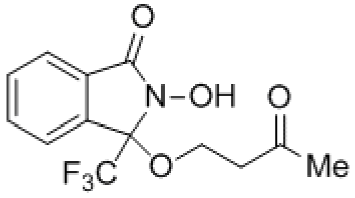

2a

1,1-dMe-BuOH, Bn

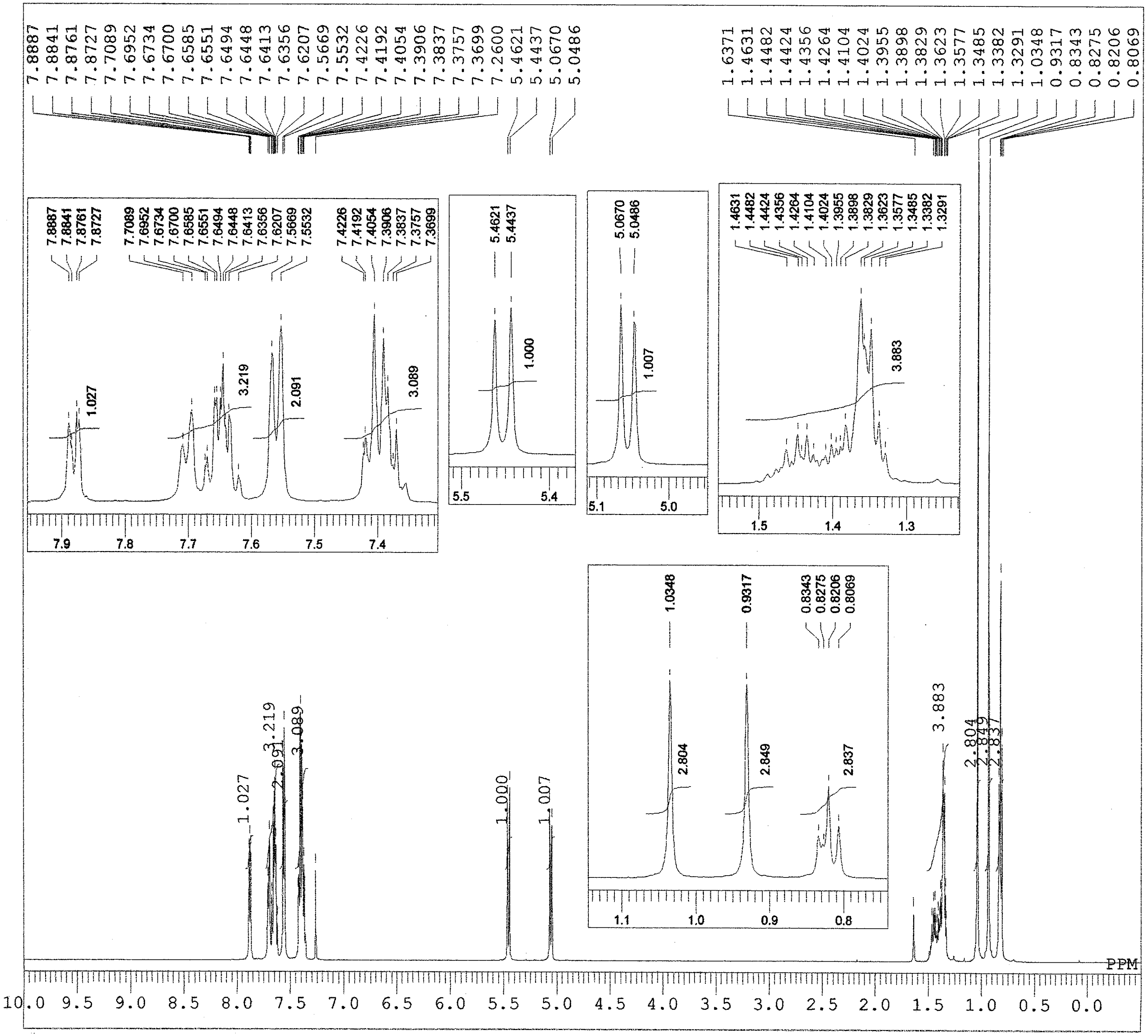

DFILE	ozawa04-140_1H.jdf
COMNT	1,1-dMe-BuOH, Bn
DATIM	2014-03-06 17:19:07
OBNUC	1H
EXMOD	proton.jxp
OBFRQ	500.16 MHz
OBSET	2.41 KHz
OBFIN	6.01 Hz
POINT	16384
FREQU	9384.38 Hz
SCANS	4
ACQTM	1.7459 sec
PD	5.0000 sec
PW1	5.55 usec
IRNUC	1H
CTEMP	21.2 c
SLVNT	CDCL3
EXREF	7.26 ppm
BF	1.00 Hz
RGAIN	34

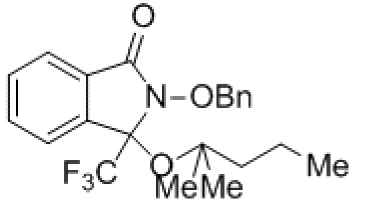

S4b

1, 1-dMe-BuOH, Bn

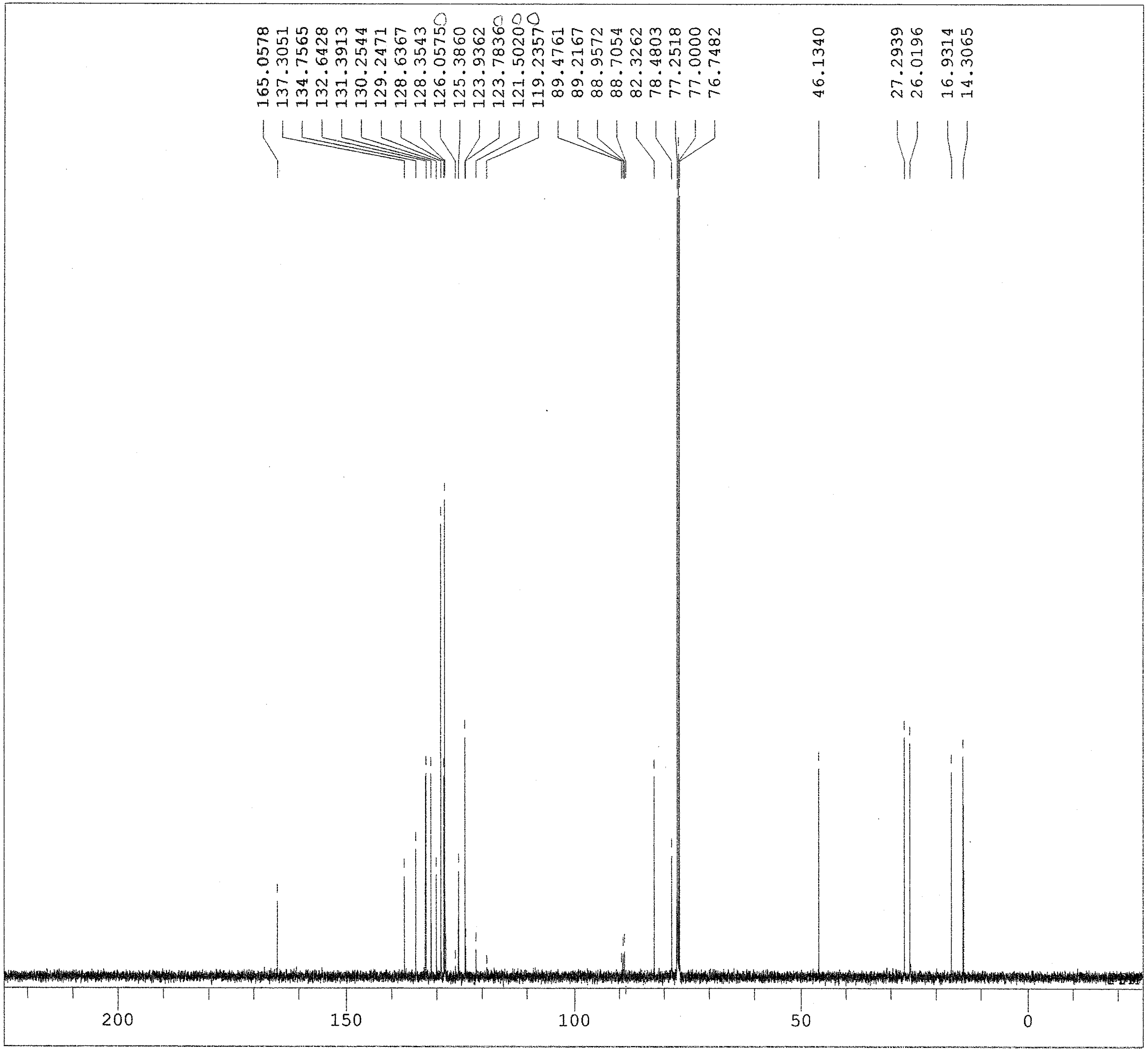

DFILE	ozawa04-140_13C.jdf
COMNT	1,1-dMe-BuOH, Bn
DATIM	2014-03-06 17:20:28
OBNUC	13C
EXMOD	carbon.jxp
OBFRQ	125.77 MHz
OBSET	7.87 KHz
OBFIN	4.21 Hz
POINT	32768
FREQU	31446.54 Hz
SCANS	224
ACQTM	1.0420 sec
PD	3.0000 sec
PW1	3.40 usec
IRNUC	1H
CTEMP	21.6 c
SLVNT	CDCL3
EXREF	77.00 ppm
BF	1.00 Hz
RGAIN	72

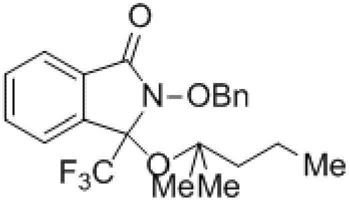

S4b

1, 1-dMe-BuOH, Bn

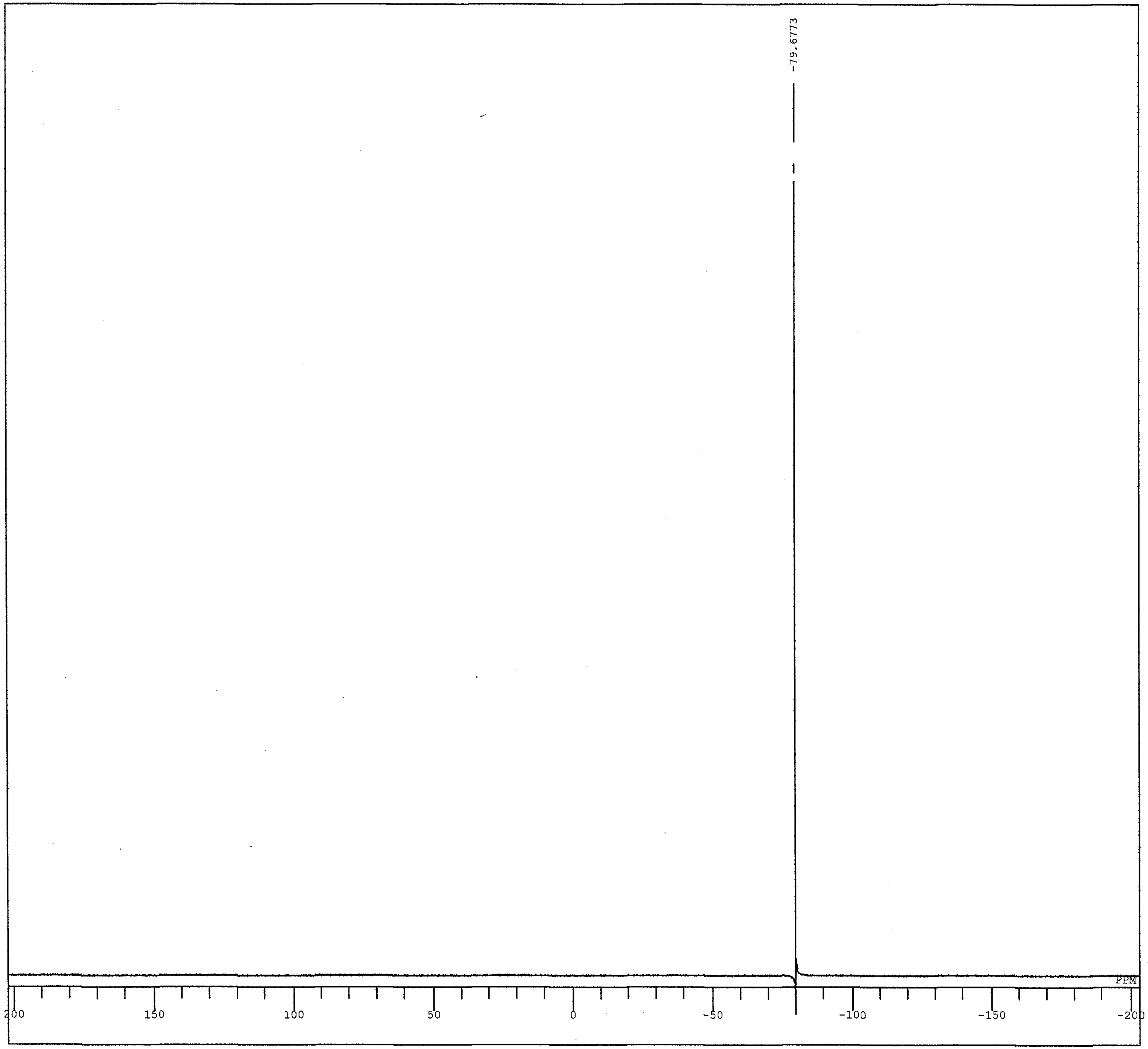

DFILE ozawa04-140_19F.jdf
COMNT 1,1-dMe-BuOH, Bn
DATIM 06-03-2014 20:36:23
OBNUC 19F
EXMOD proton.jxp
OBFRQ 368.64 MHz
OBSET 7.63 KHz
OBFIN 2.85 Hz
POINT 16384
FREQU 186567.17 Hz
SCANS 4
ACQTM 0.0878 sec
PD 5.0000 sec
PW1 3.90 usec
IRNUC 19F
CTEMP 21.4 c
SLVNT CDCL3
EXREF -164.90 ppm
BF 0.12 Hz
RGAIN 48

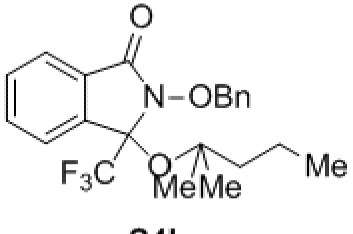

S4b

1,1-dMe-BuOH, OH

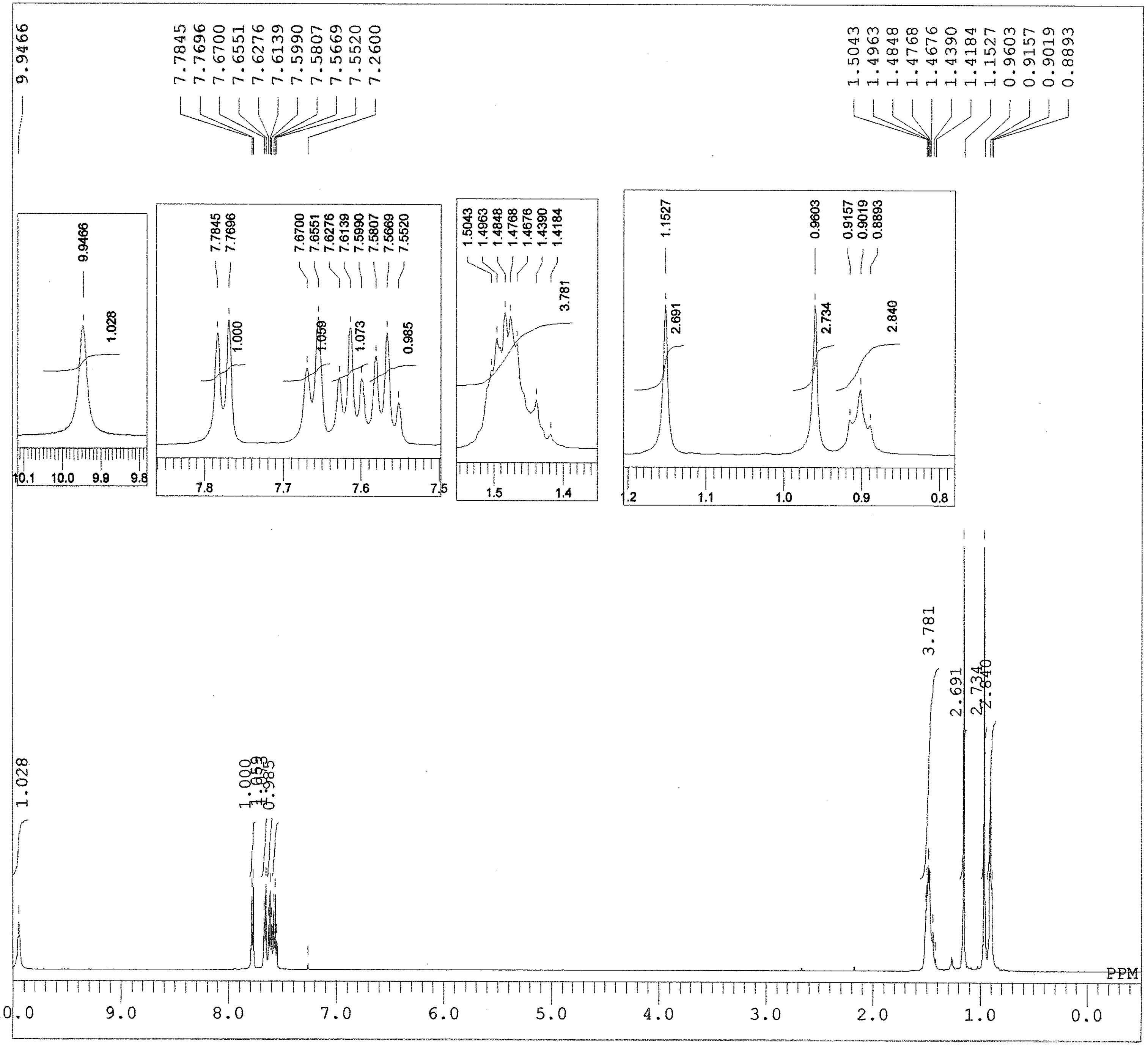

DFILE ozawa04-143_1H.jdf
COMNT 1,1-dMe-BuOH, OH
DATIM 2014-03-06 17:10:23
OBNUC 1H
EXMOD proton.jxp
OBFRQ 500.16 MHz
OBSET 2.41 KHz
OBFIN 6.01 Hz
POINT 16384
FREQU 9384.38 Hz
SCANS 4
ACQTM 1.7459 sec
PD 5.0000 sec
PW1 5.55 usec
IRNUC 1H
CTEMP 21.5 c
SLVNT CDCL3
EXREF 7.26 ppm
BF 1.00 Hz
RGAIN 24

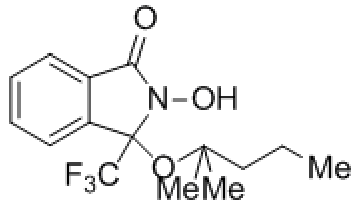

1b

1, 1-dMe-BuOH, OH

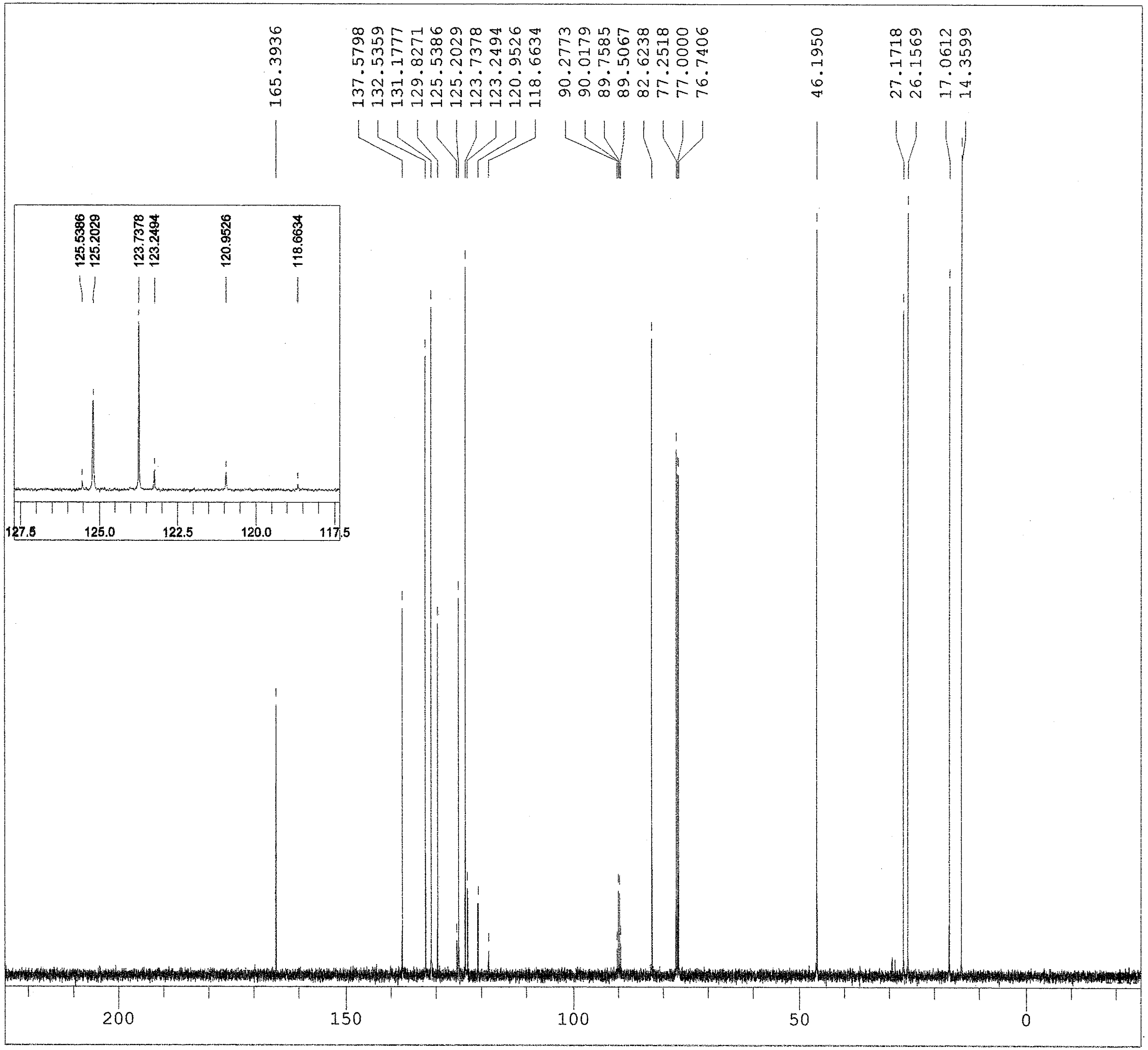

DFILE ozawa04-143_13C.jdf
COMNT 1,1-dMe-BuOH, OH
DATIM 2014-03-06 17:11:34
OBNUC 13C
EXMOD carbon.jxp
OBFRQ 125.77 MHz
OBSET 7.87 KHz
OBFIN 4.21 Hz
POINT 32768
FREQU 31446.54 Hz
SCANS 64
ACQTM 1.0420 sec
PD 3.0000 sec
PW1 3.40 usec
IRNUC 1H
CTEMP 21.3 c
SLVNT CDCL3
EXREF 77.00 ppm
BF 1.00 Hz
RGAIN 72

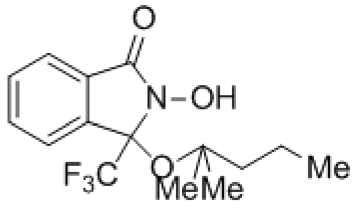

1b

1,1-dMe-BuOH, OH

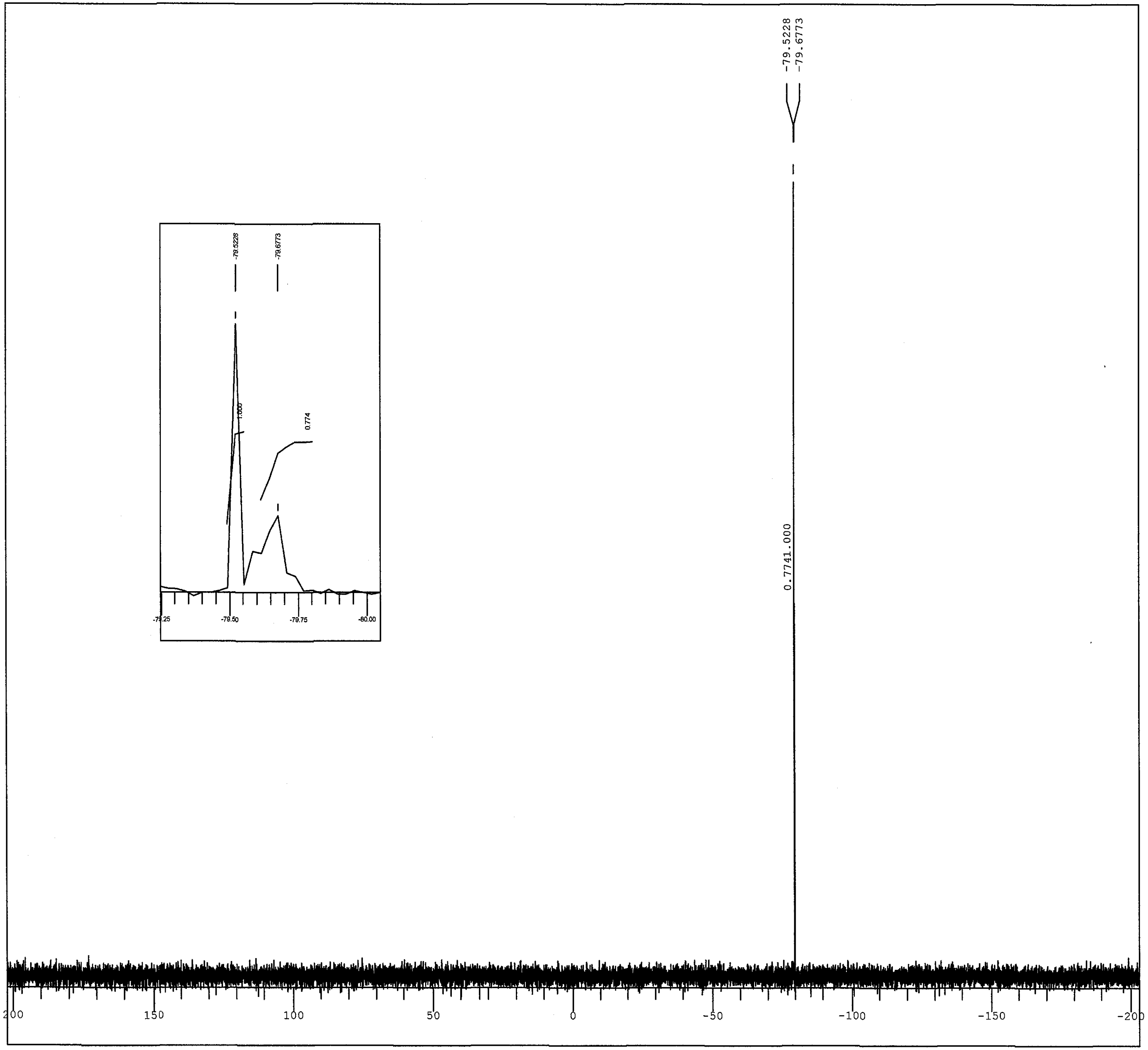

DFILE ozawa04-143_19F.jdf
COMNT 1,1-dMe-BuOH, OH
DATIM 07-03-2014 17:33:56
OBNUC 19F
EXMOD proton.jxp
OBFRQ 368.64 MHz
OBSET 7.63 KHz
OBFIN 2.85 Hz
POINT 16384
FREQU 186567.17 Hz
SCANS 4
ACQTM 0.0878 sec
PD 5.0000 sec
PW1 3.90 usec
IRNUC 19F
CTEMP 21.1 c
SLVNT CDCL3
EXREF -164.90 ppm
BF 0.12 Hz
RGAIN 50

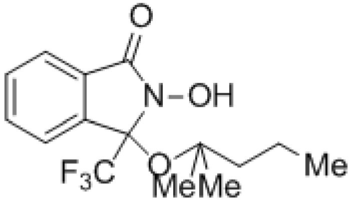

1b

1,1-dMe-BuOH, [O]

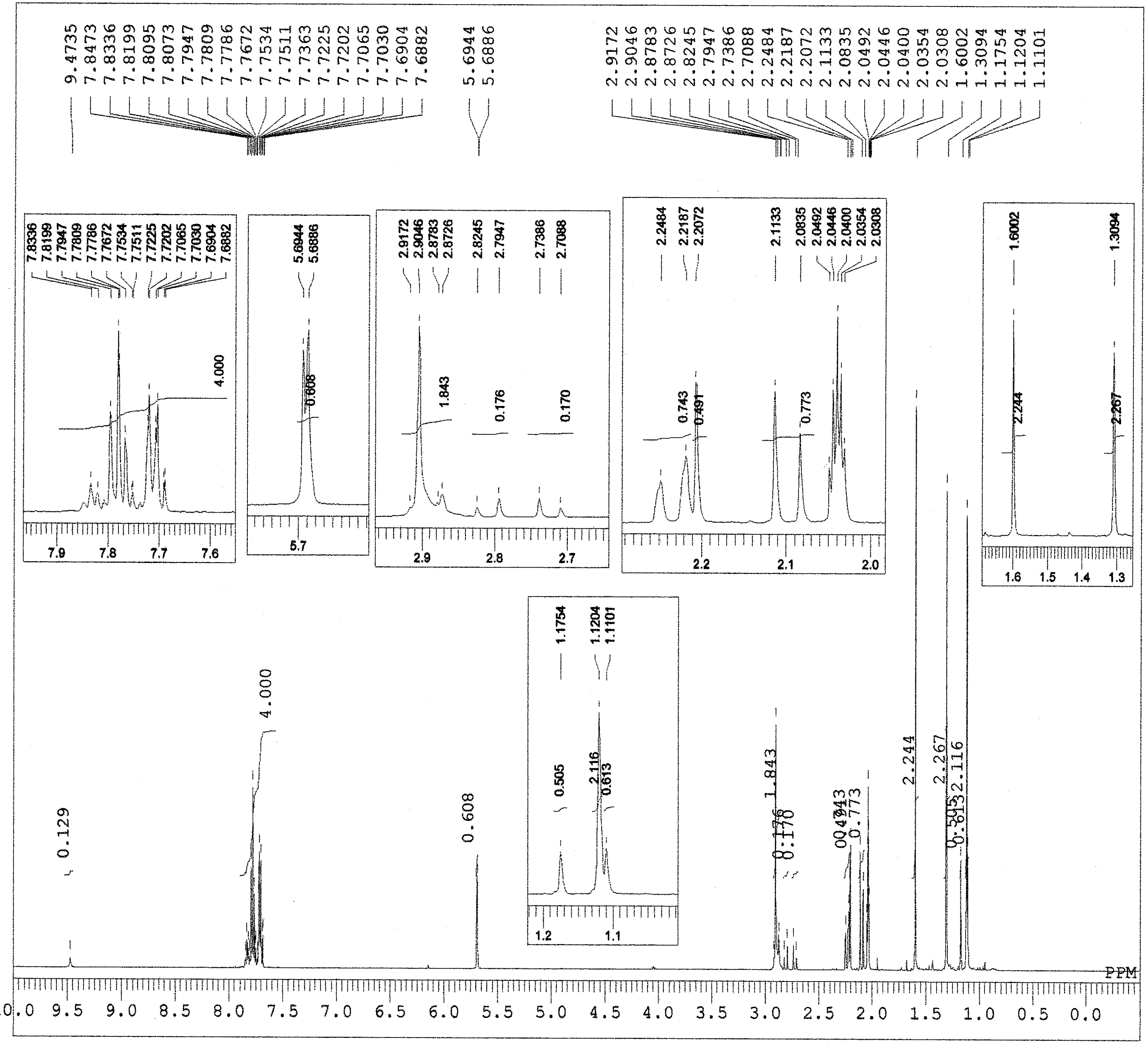

DFILE	ozawa04-144_1.als
COMNT	1,1-dMe-BuOH, [O]
DATIM	2014-03-09 19:10:36
OBNUC	1H
EXMOD	proton.jxp
OBFRQ	500.16 MHz
OBSET	2.41 KHz
OBFIN	6.01 Hz
POINT	13107
FREQU	7507.51 Hz
SCANS	4
ACQTM	1.7459 sec
PD	5.0000 sec
PW1	5.55 usec
IRNUC	1H
CTEMP	20.8 c
SLVNT	ACETN
EXREF	2.04 ppm
BF	0.12 Hz
RGAIN	36

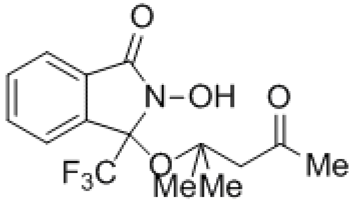

2b (minor)

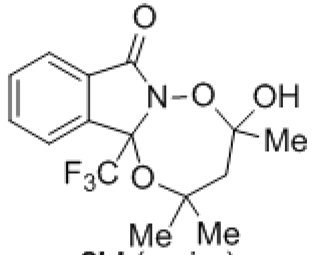

2b' (major)

1,1-dMe-BuOH, [O]

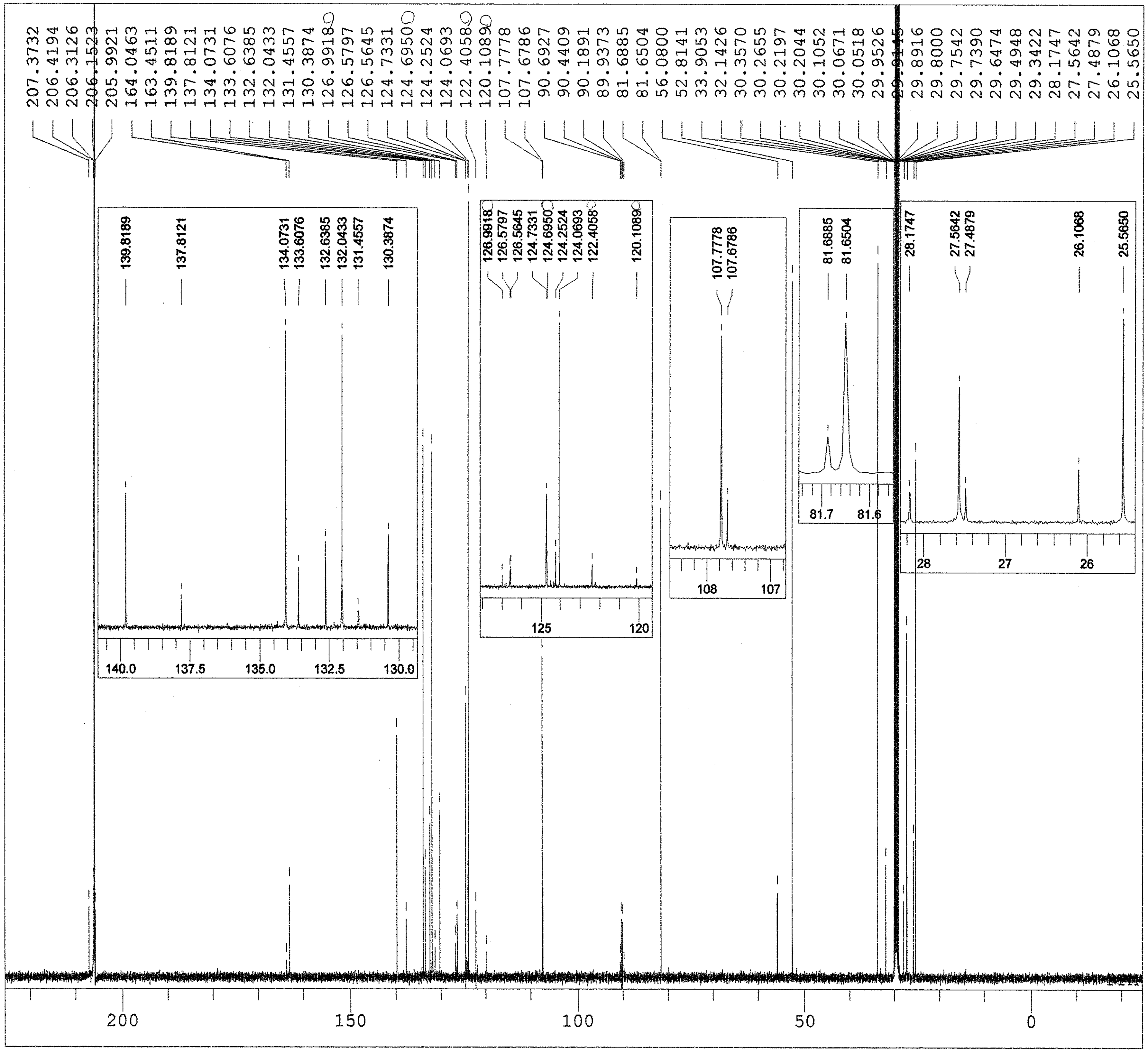

DFILE	ozawa04-144_1_13C.jdf
COMNT	1,1-dMe-BuOH, [O]
DATIM	2014-03-09 20:54:37
OBNUC	13C
EXMOD	carbon.jxp
OBFRQ	125.77 MHz
OBSET	7.87 KHz
OBFIN	4.21 Hz
POINT	32768
FREQU	31446.54 Hz
SCANS	12093
ACQTM	1.0420 sec
PD	3.0000 sec
PW1	3.40 usec
IRNUC	1H
CTEMP	21.6 c
SLVNT	ACETN
EXREF	29.80 ppm
BF	0.12 Hz
RGAIN	72

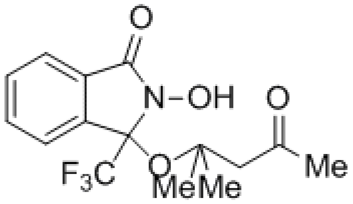

2b (minor)

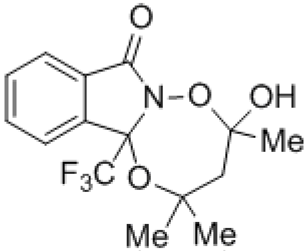

2b' (major)

1, 1-dMe-BuOH, [O]

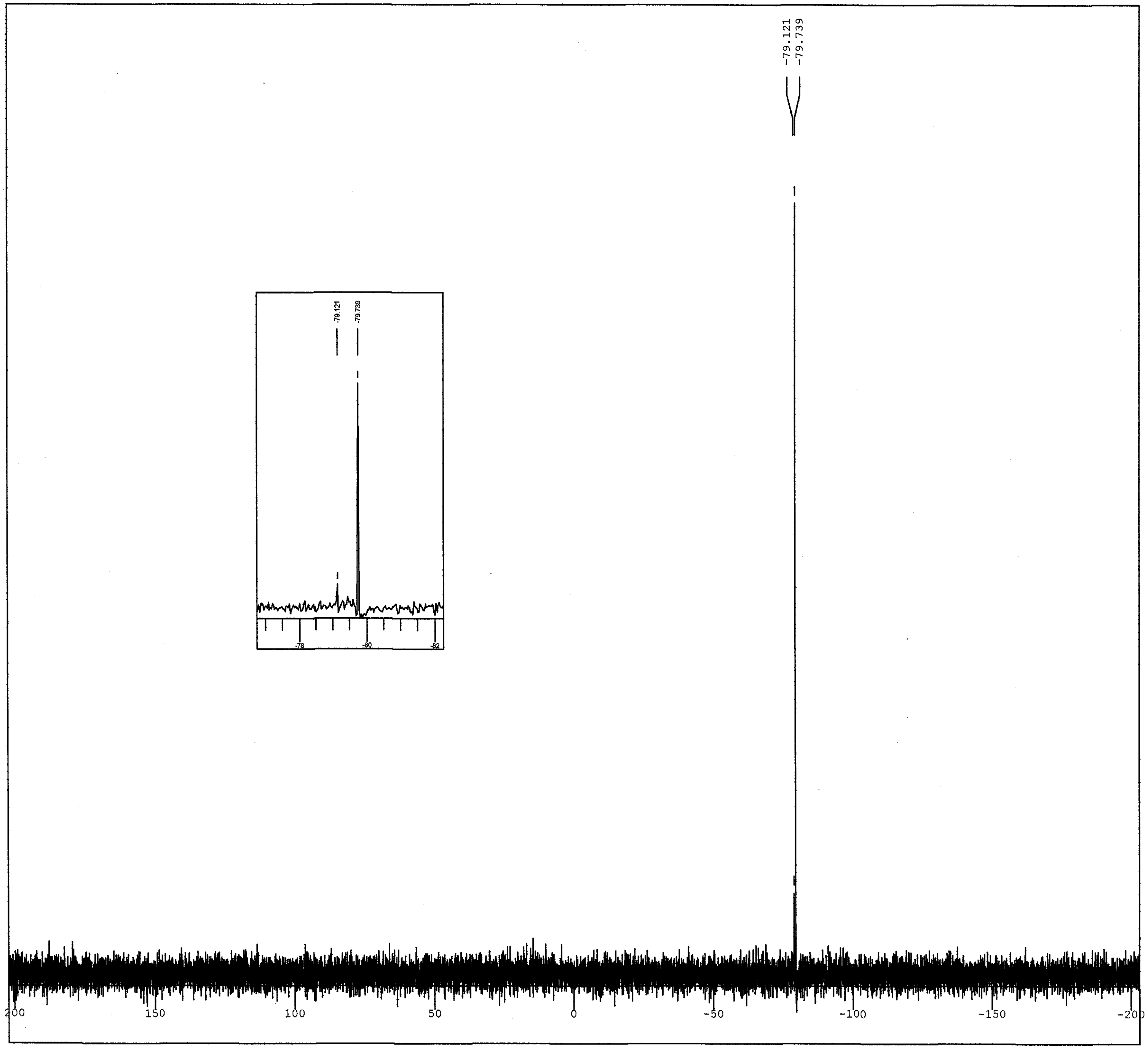

DFILE ozawa04-144_1_19F.jdf
COMNT 1,1-dMe-BuOH, [O]
DATIM 11-03-2014 16:28:22
OBNUC 19F
EXMOD proton.jxp
OBFRQ 368.64 MHz
OBSET 7.63 KHz
OBFIN 2.85 Hz
POINT 16384
FREQU 186567.17 Hz
SCANS 4
ACQTM 0.0878 sec
PD 5.0000 sec
PW1 3.90 usec
IRNUC 19F
CTEMP 21.0 c
SLVNT CDCL3
EXREF -164.90 ppm
BF 0.12 Hz
RGAIN 50

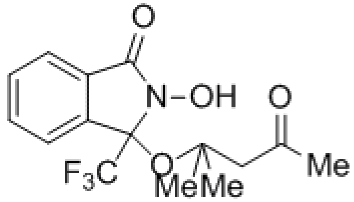

2b (minor)

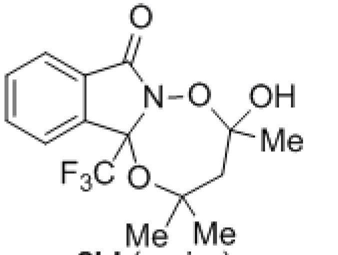

2b' (major)

\\172.20.1.201\users\delta\Documents\JEOL\data\ozawa04-144_1_HMQC.jdf

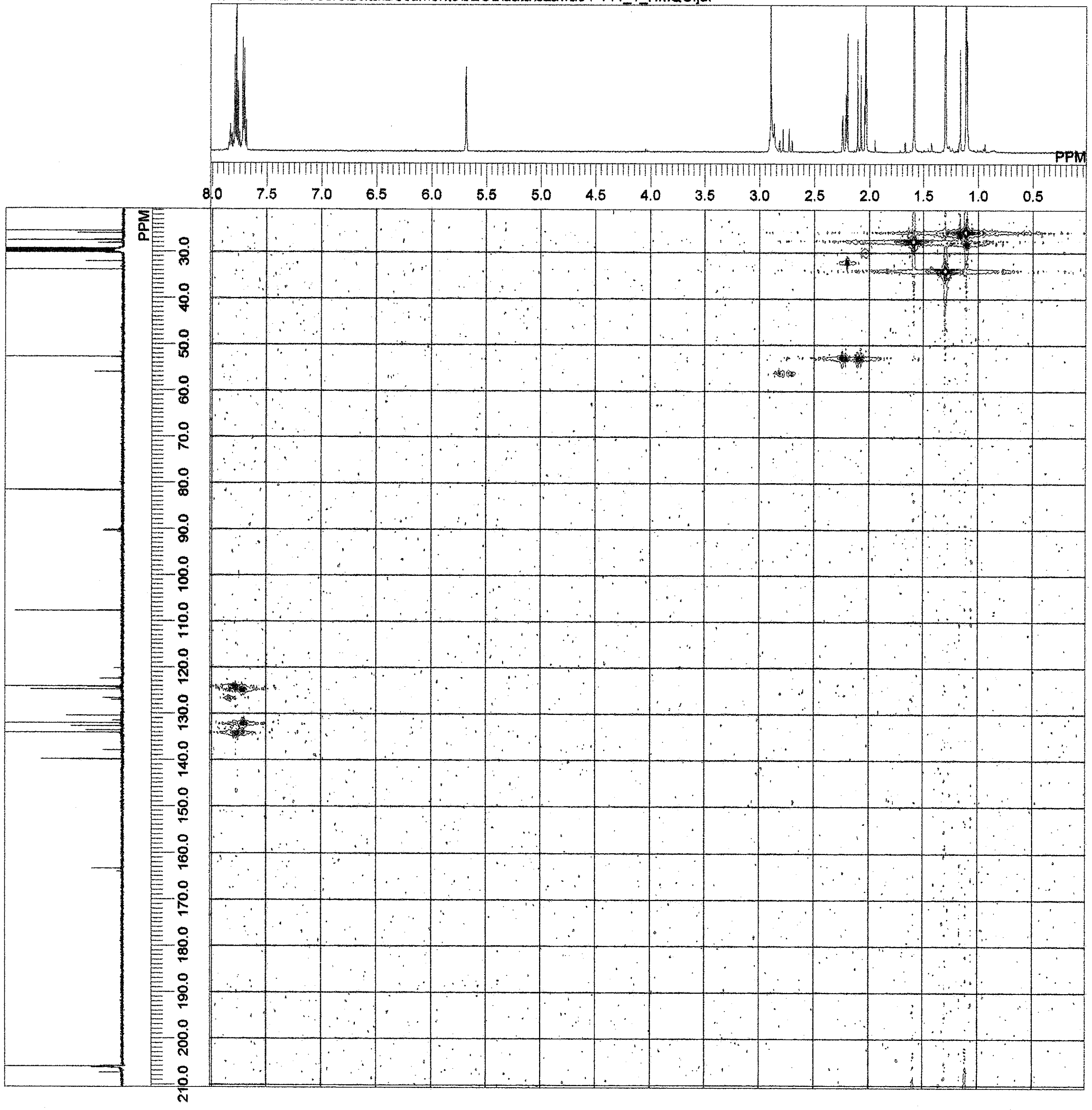

DFILE	ozawa04-144_1_HMQC.jdf
COMNT	1,1-dMe-BuOH, [O]
DATIM	10-03-2014 22:56:31
EXMOD	hmqc.jxp
OBNUC	1H
OBFRQ	500.16 MHz
OBSET	1.91 KHz
OBFIN	5.85 Hz
POINT	1024
FREQU	5004.00 Hz
CLPNT	512
TODAT	512
CLFRQ	23900.57 Hz
SCANS	4
ACQTM	0.2046 sec
PD	1.5000 sec
PW1	11.10 usec
PW2	0.00 usec
PW3	0.00 usec
PI1	0.0000 msec
PI2	0.0000 msec
PI3	0.0000 msec
IRNUC	13C
CTEMP	21.5 c
SLVNT	ACETN
EXREF	2.04 ppm
CLEXR	29.80
RGAIN	90

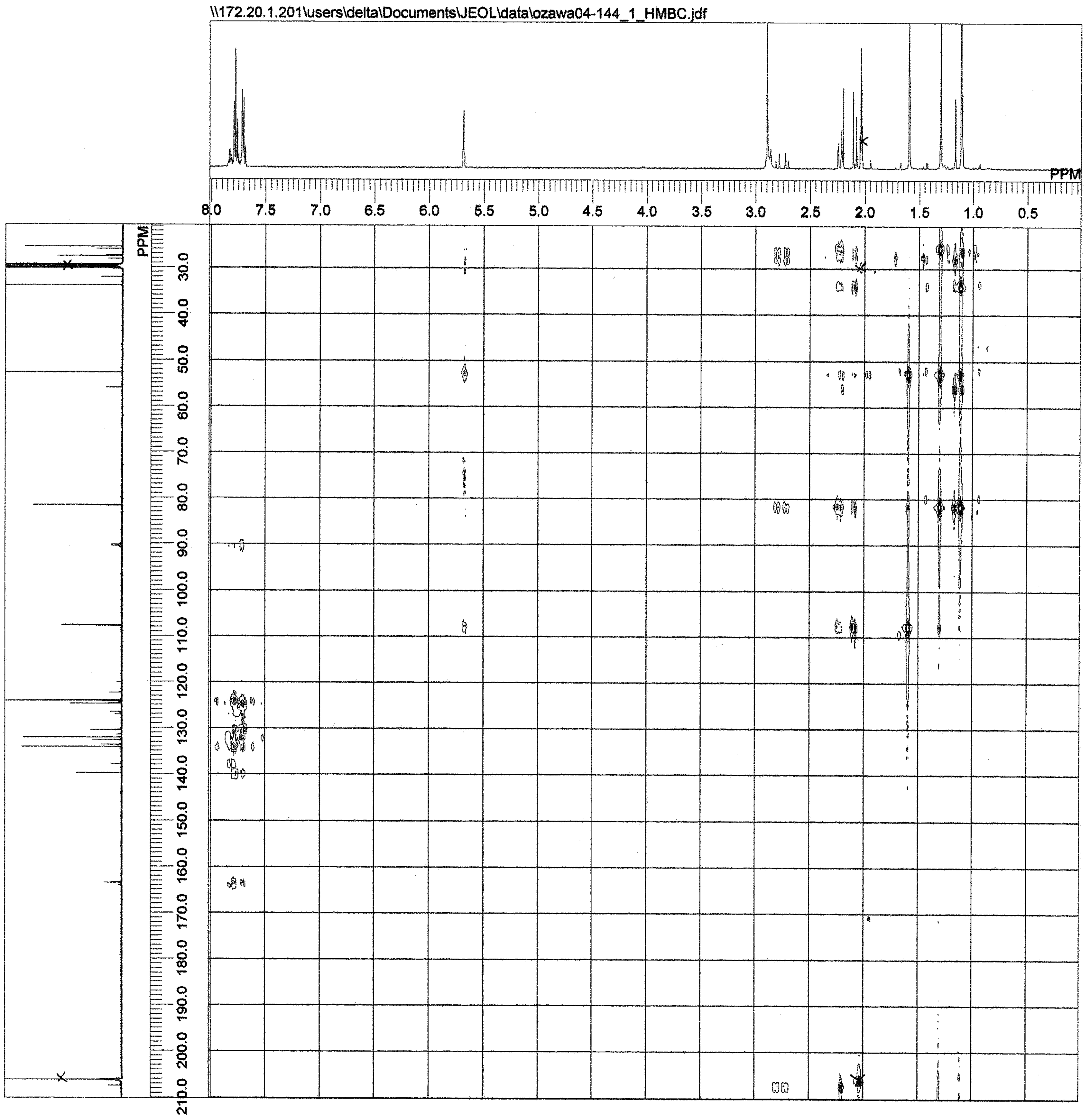

DFILE	ozawa04-144_1_HMBC.jdf
COMNT	1,1-dMe-BuOH, [O]
DATIM	10-03-2014 23:56:07
EXMOD	hmbc.jxp
OBNUC	1H
OBFRQ	500.16 MHz
OBSET	1.91 KHz
OBFIN	5.85 Hz
POINT	2047
FREQU	5004.00 Hz
CLPNT	256
TODAT	256
CLFRQ	23900.57 Hz
SCANS	60
ACQTM	0.4093 sec
PD	1.5000 sec
PW1	11.10 usec
PW2	0.00 usec
PW3	0.00 usec
PI1	0.0000 msec
PI2	0.0000 msec
PI3	0.0000 msec
IRNUC	13C
CTEMP	21.3 c
SLVNT	ACETN
EXREF	2.04 ppm
CLEXR	29.80
RGAIN	90

2,2-dMe-BuOH, Bn

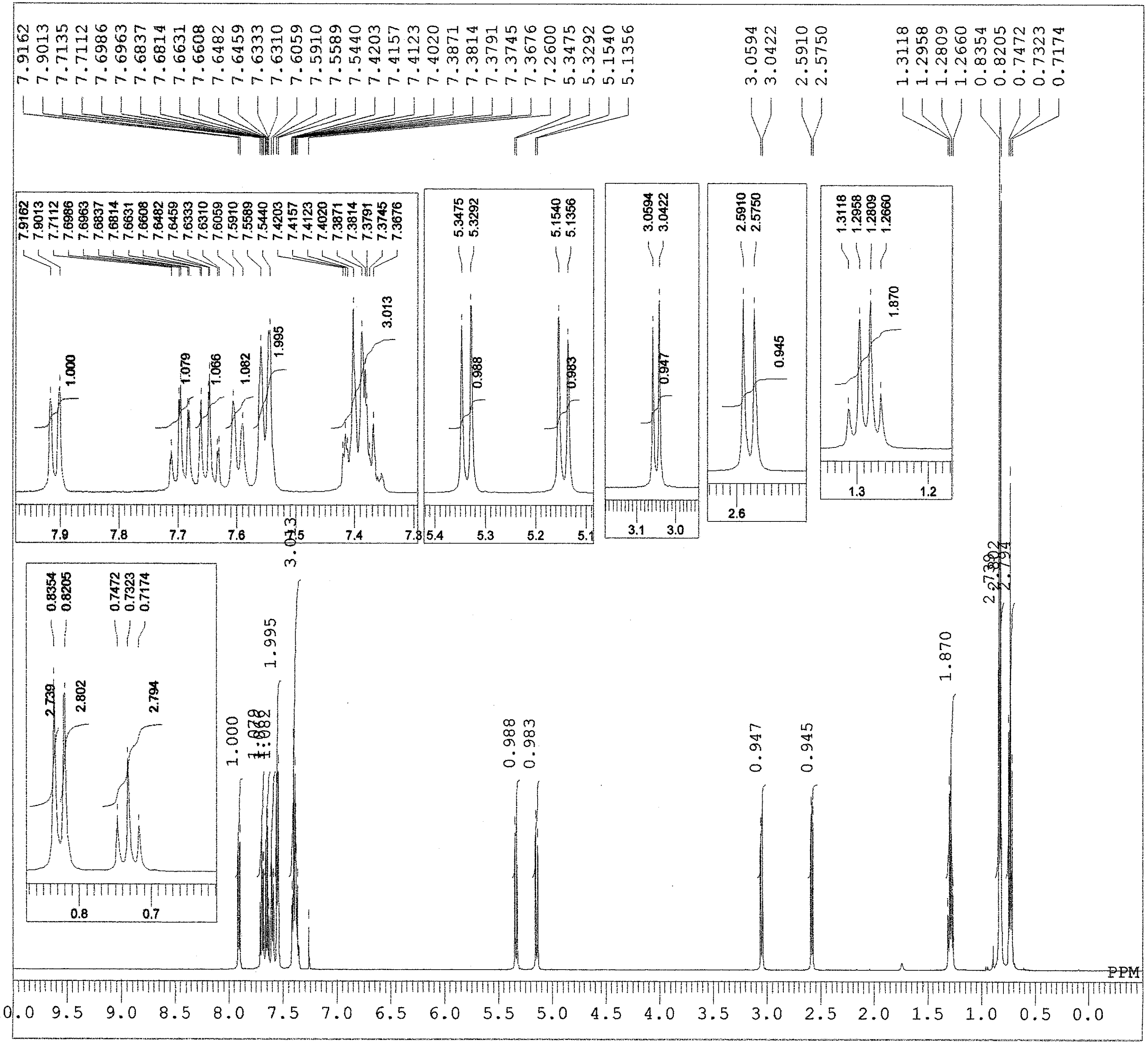

DFILE	ozawa04-142_1_1H.als
COMNT	2,2-dMe-BuOH, Bn
DATIM	2014-03-06 19:21:02
OBNUC	1H
EXMOD	proton.jxp
OBFRQ	500.16 MHz
OBSET	2.41 KHz
OBFIN	6.01 Hz
POINT	13107
FREQU	7507.51 Hz
SCANS	4
ACQTM	1.7459 sec
PD	5.0000 sec
PW1	5.55 usec
IRNUC	1H
CTEMP	21.6 c
SLVNT	CDCL3
EXREF	7.26 ppm
BF	0.12 Hz
RGAIN	28

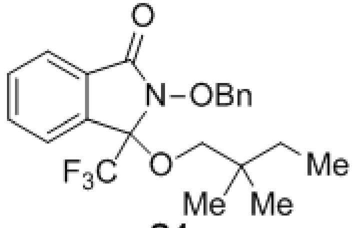

S4c

2,2-dMe-BuOH, Bn

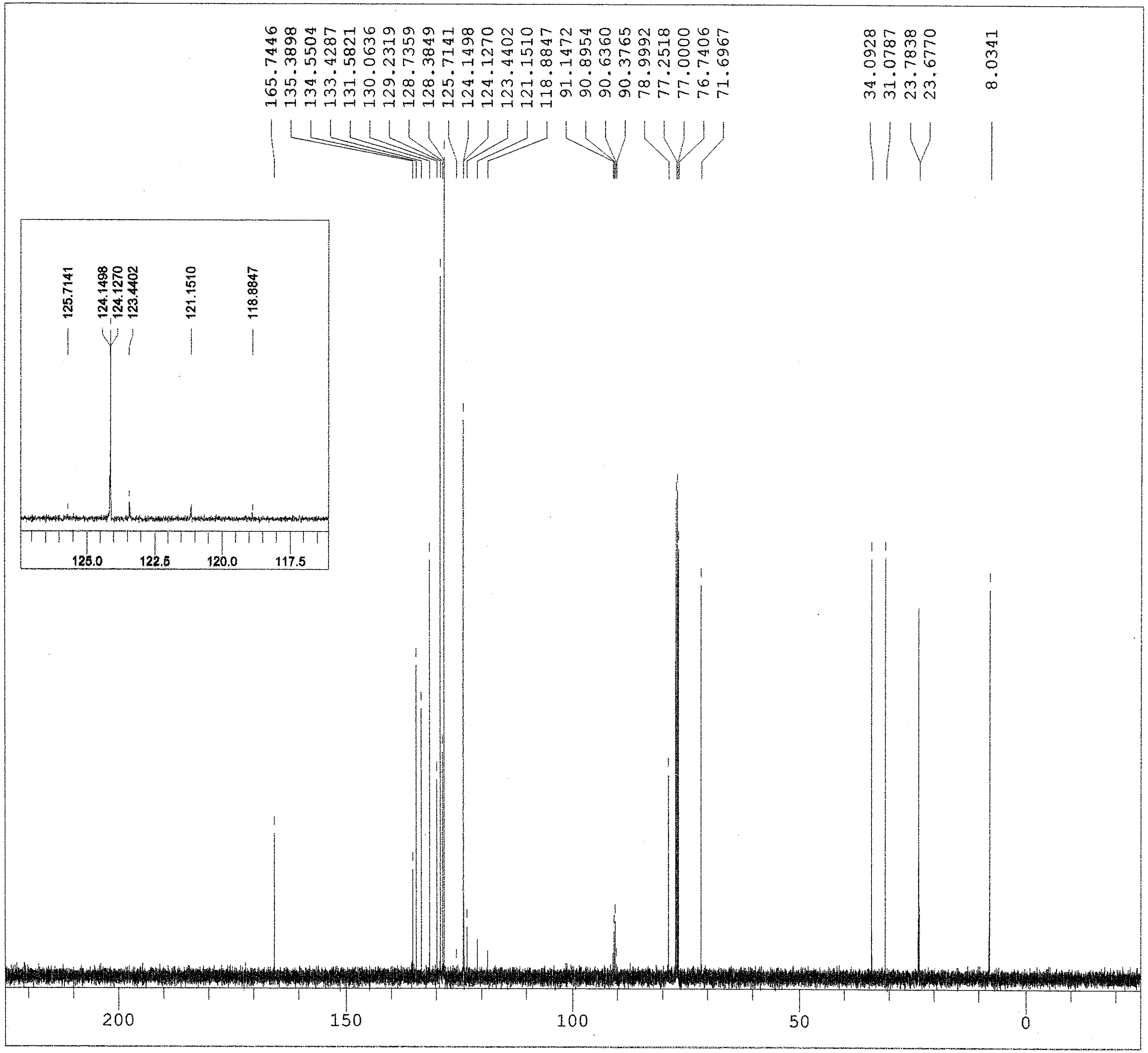

DFILE	ozawa04-142_1_13C.jdf
COMNT	2,2-dMe-BuOH, Bn
DATIM	2014-03-06 19:22:14
OBNUC	13C
EXMOD	carbon.jxp
OBFRQ	125.77 MHz
OBSET	7.87 KHz
OBFIN	4.21 Hz
POINT	32768
FREQU	31446.54 Hz
SCANS	78
ACQTM	1.0420 sec
PD	3.0000 sec
PW1	3.40 usec
IRNUC	1H
CTEMP	21.7 c
SLVNT	CDCL3
EXREF	77.00 ppm
BF	0.12 Hz
RGAIN	72

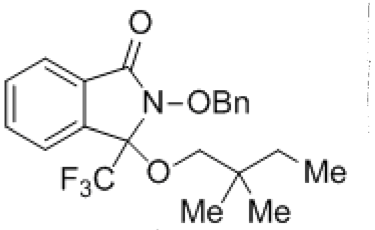

S4c

2, 2-dMe-BuOH, Bn

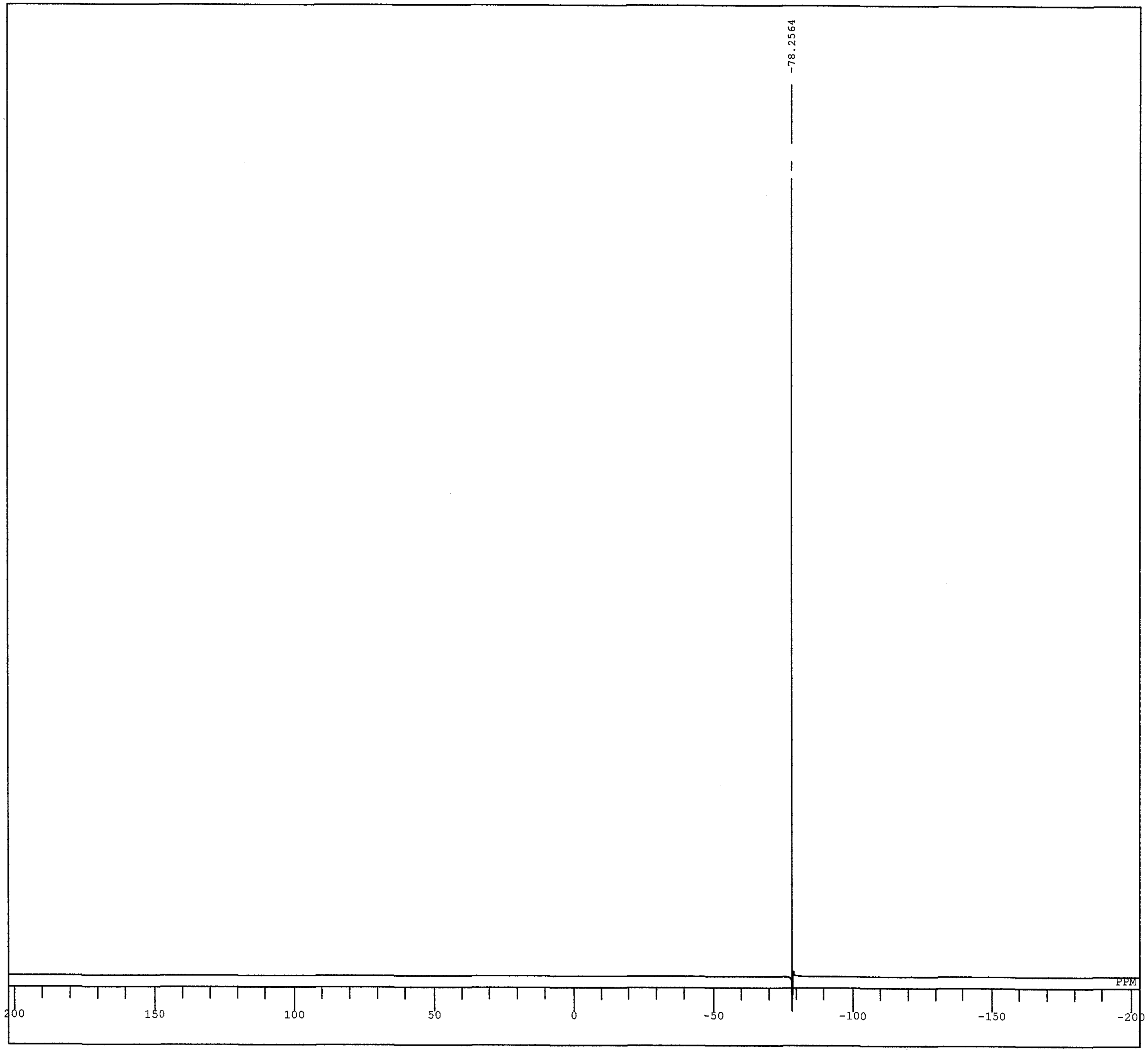

DFILE ozawa04-142_1_19F.jdf
COMNT 2,2-dMe-BuOH, Bn
DATIM 06-03-2014 20:39:07
OBNUC 19F
EXMOD proton.jxp
OBFRQ 368.64 MHz
OBSET 7.63 KHz
OBFIN 2.85 Hz
POINT 16384
FREQU 186567.17 Hz
SCANS 4
ACQTM 0.0878 sec
PD 5.0000 sec
PW1 3.90 usec
IRNUC 19F
CTEMP 21.4 c
SLVNT CDCL3
EXREF -164.90 ppm
BF 0.12 Hz
RGAIN 46

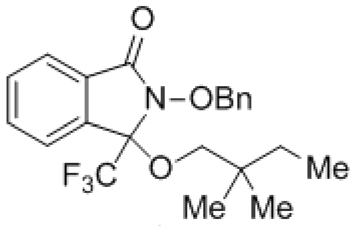

S4c

OH

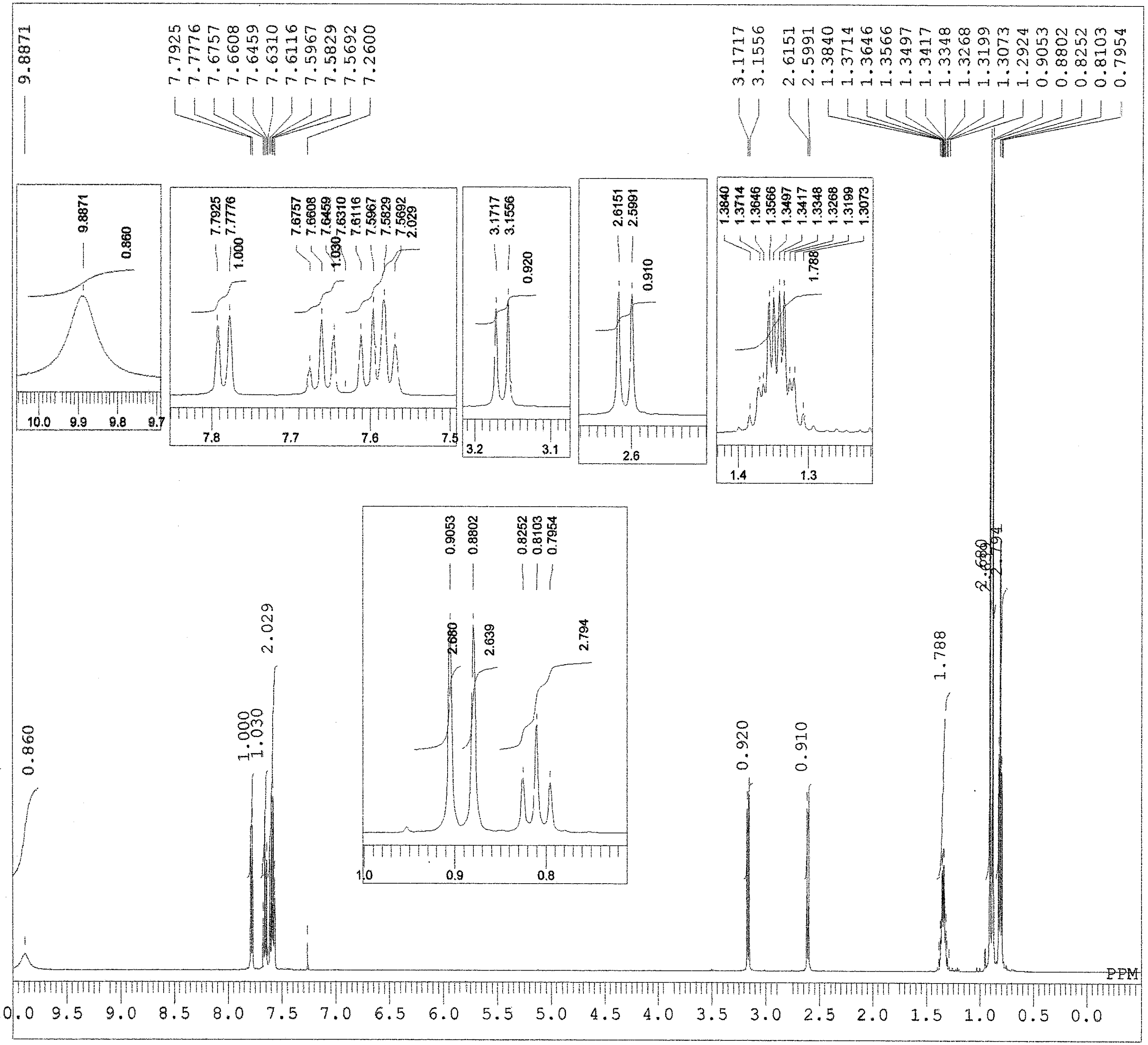

DFILE Ni_2,2-dMe-BuOH_1H.jdf
COMNT OH
DATIM 2014-03-03 20:17:29
OBNUC 1H
EXMOD proton.jxp
OBFRQ 500.16 MHz
OBSET 2.41 KHz
OBFIN 6.01 Hz
POINT 16384
FREQU 9384.38 Hz
SCANS 4
ACQTM 1.7459 sec
PD 5.0000 sec
PW1 5.55 usec
IRNUC 1H
CTEMP 21.1 c
SLVNT CDCL3
EXREF 7.26 ppm
BF 0.12 Hz
RGAIN 28

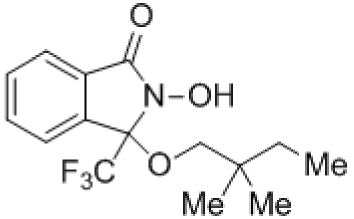

1c

OH

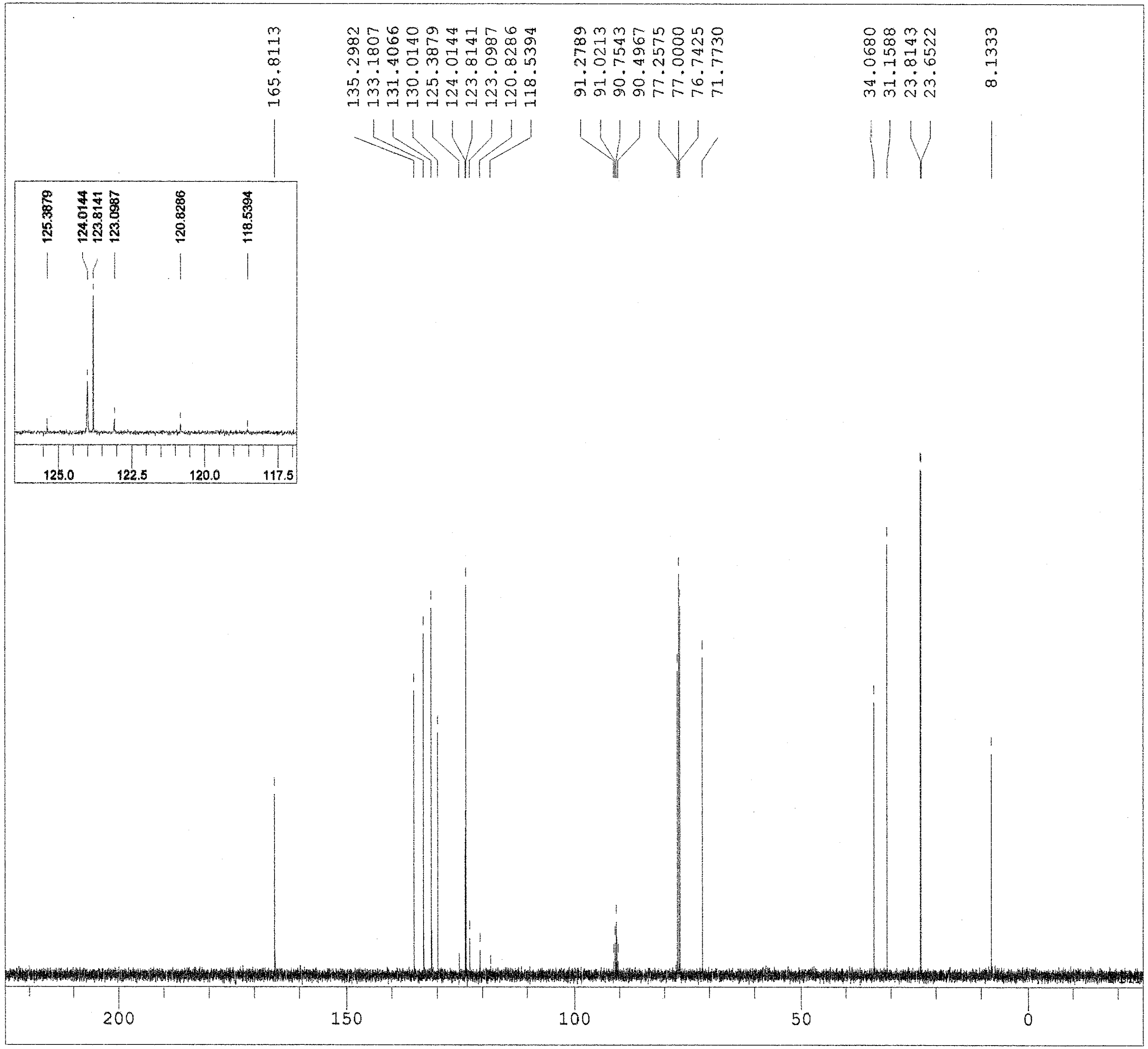

DFILE Ni_2,2-dMe-BuOH_13C.jdf
COMNT OH
DATIM 2014-03-03 20:18:32
OBNUC 13C
EXMOD carbon.jxp
OBFRQ 125.77 MHz
OBSET 7.87 KHz
OBFIN 4.21 Hz
POINT 32767
FREQU 39308.18 Hz
SCANS 80
ACQTM 0.8336 sec
PD 3.0000 sec
PW1 3.40 usec
IRNUC 1H
CTEMP 21.3 c
SLVNT CDCL3
EXREF 77.00 ppm
BF 0.12 Hz
RGAIN 60

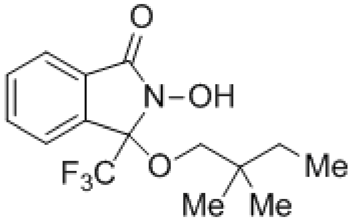

1c

OH

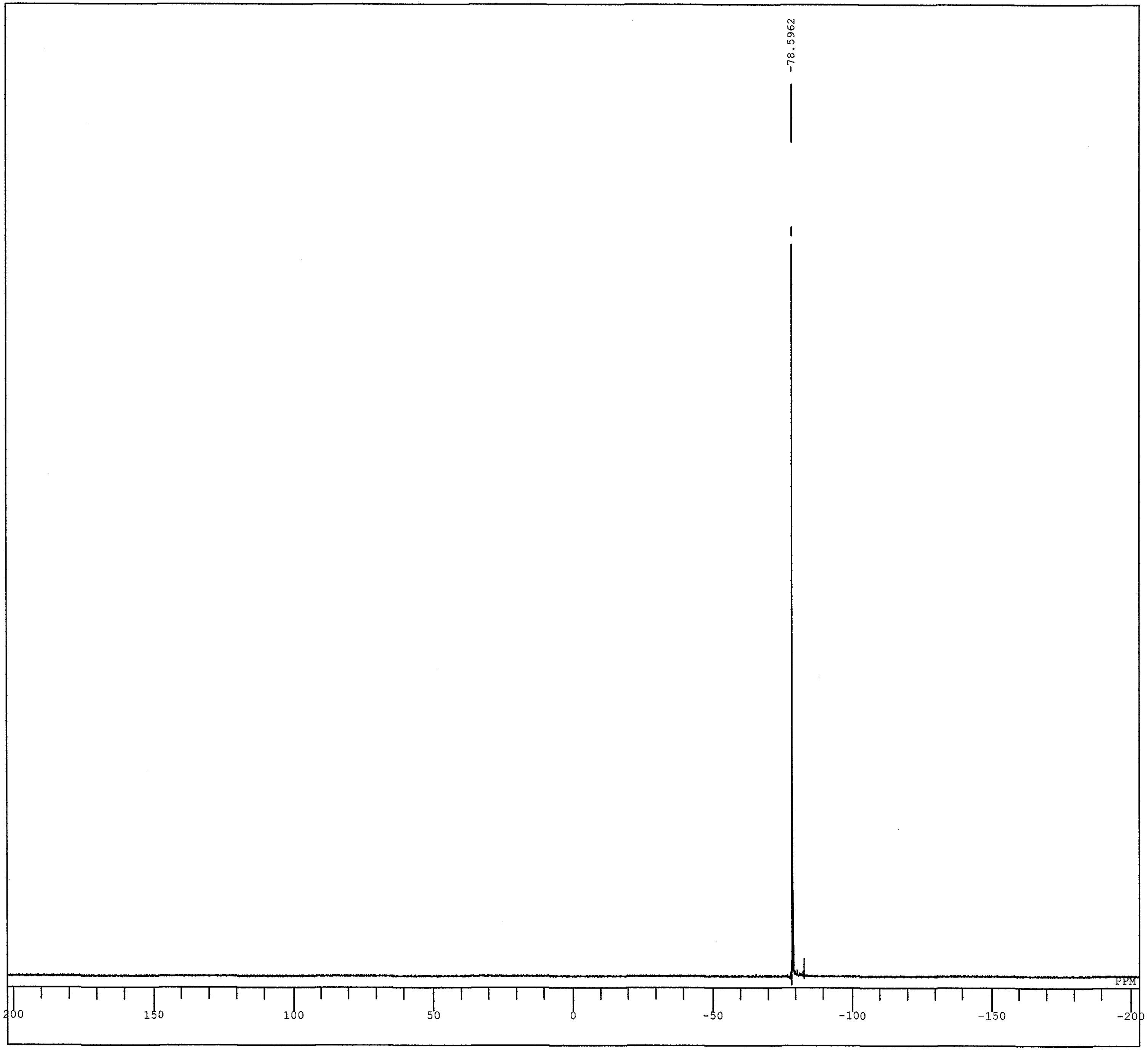

DFILE	Ni_2,2-dMe-BuOH_19F.jdf
COMNT	OH
DATIM	03-03-2014 20:57:45
OBNUC	19F
EXMOD	proton.jxp
OBFRQ	368.64 MHz
OBSET	7.63 KHz
OBFIN	2.85 Hz
POINT	16384
FREQU	186567.17 Hz
SCANS	4
ACQTM	0.0878 sec
PD	5.0000 sec
PW1	3.90 usec
IRNUC	19F
CTEMP	21.0 c
SLVNT	CDCL3
EXREF	-164.90 ppm
BF	0.12 Hz
RGAIN	44

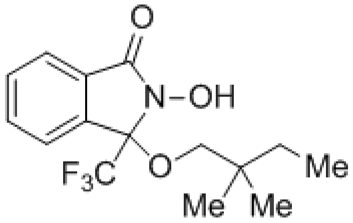

1c

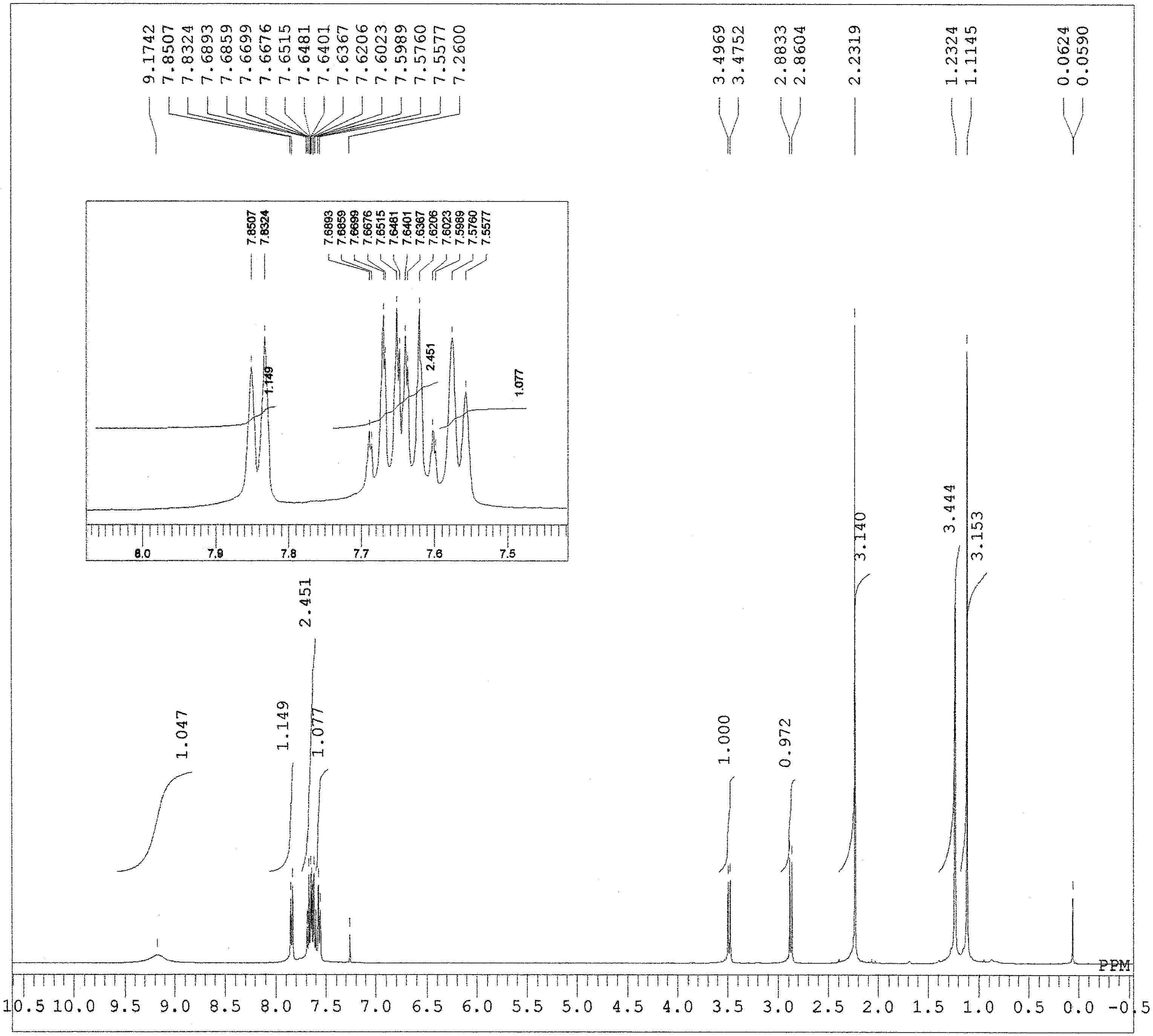

DFILE ni140206-C-K-peak1-H1-1-1.als
COMNT
DATIM 06-02-2014 16:27:48
OBNUC 1H
EXMOD proton.jxp
OBFRQ 391.78 MHz
OBSET 8.51 KHz
OBFIN 3.34 Hz
POINT 13107
FREQU 5878.90 Hz
SCANS 18
ACQTM 2.2295 sec
PD 5.0000 sec
PW1 5.25 usec
IRNUC 1H
CTEMP 21.2 c
SLVNT CDCL3
EXREF 7.26 ppm
BF 0.12 Hz
RGAIN 30

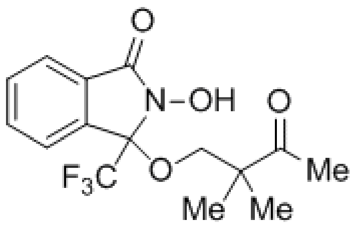

2c

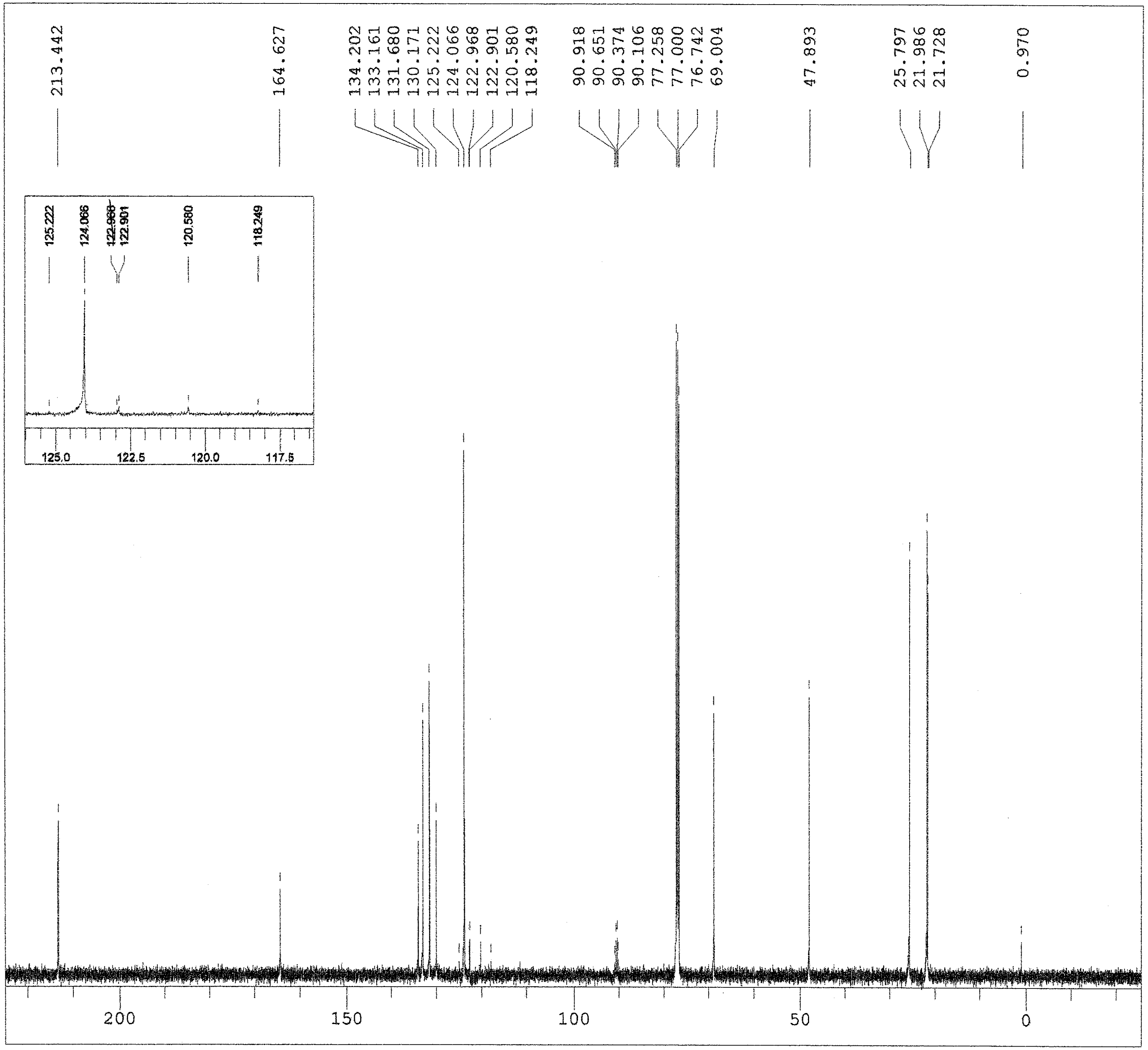

DFILE	ni140206-C-K-C13.als
COMNT	
DATIM	2014-02-03 01:46:38
OBNUC	13C
EXMOD	single_pulse_dec
OBFRQ	123.26 MHz
OBSET	2.31 KHz
OBFIN	6.71 Hz
POINT	26214
FREQU	30863.73 Hz
SCANS	2236
ACQTM	0.8493 sec
PD	2.0000 sec
PW1	3.40 usec
IRNUC	1H
CTEMP	22.7 c
SLVNT	CDCL3
EXREF	77.00 ppm
BF	0.05 Hz
RGAIN	50

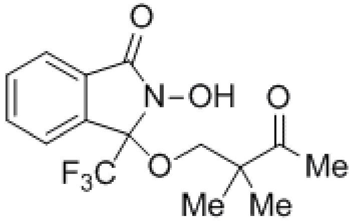

2c

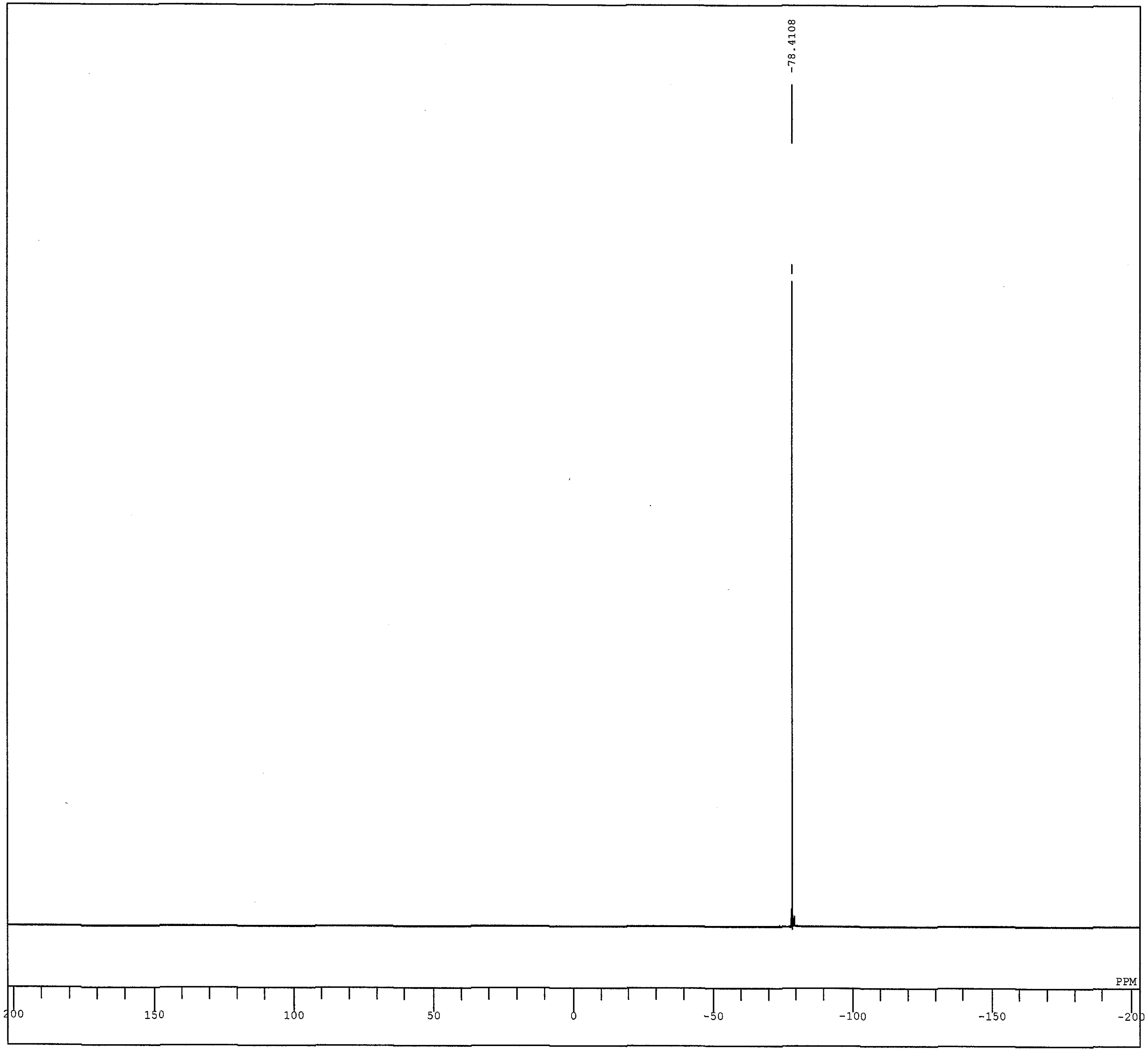

DFILE	ni140206-C-K-peak1-F19-1-1.jdf
COMNT	
DATIM	06-02-2014 16:32:06
OBNUC	19F
EXMOD	proton.jxp
OBFRQ	368.64 MHz
OBSET	7.63 KHz
OBFIN	2.85 Hz
POINT	16384
FREQU	186567.17 Hz
SCANS	4
ACQTM	0.0878 sec
PD	5.0000 sec
PW1	3.90 usec
IRNUC	19F
CTEMP	21.1 c
SLVNT	CDCL3
EXREF	-164.90 ppm
BF	0.12 Hz
RGAIN	46

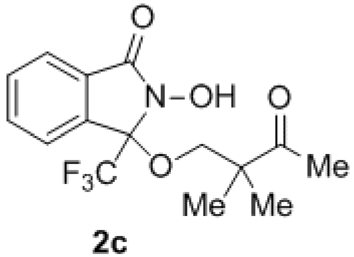

2c

hexOH, Bn

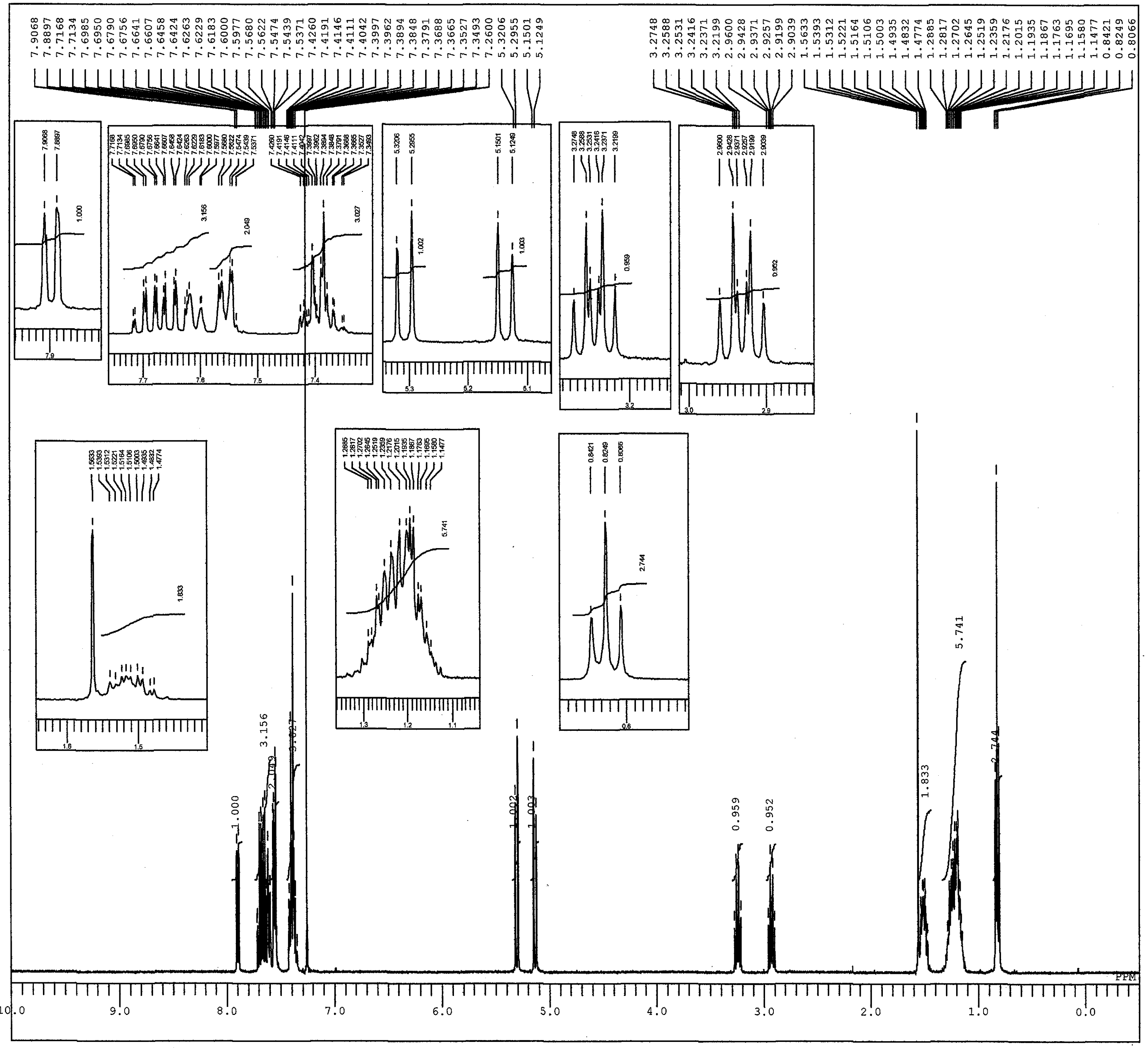

DFILE	ozawa04-007_1H.als
COMNT	hexOH, Bn
DATIM	15-03-2014 15:16:19
OBNUC	1H
EXMOD	proton.jxp
OBFRQ	391.78 MHz
OBSET	8.51 KHz
OBFIN	3.34 Hz
POINT	13107
FREQU	5878.90 Hz
SCANS	4
ACQTM	2.2295 sec
PD	5.0000 sec
PW1	5.25 usec
IRNUC	1H
CTEMP	21.0 c
SLVNT	CDCL3
EXREF	7.26 ppm
BF	0.12 Hz
RGAIN	46

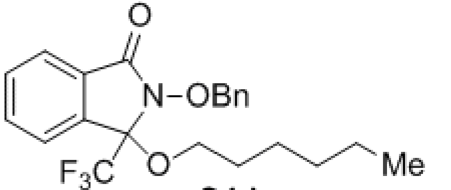

S4d

hexOH, Bn

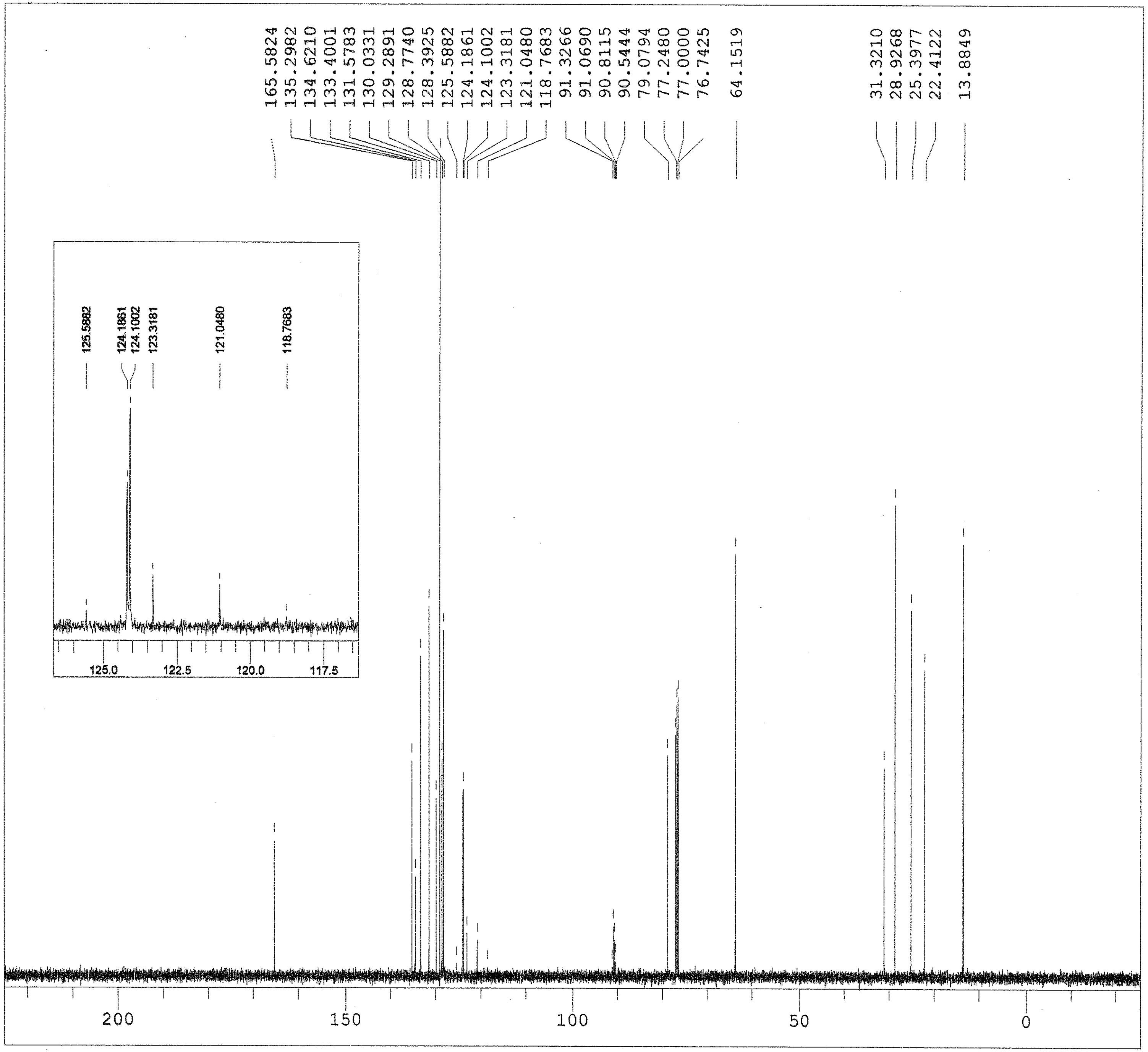

DFILE	ozawa04-151_13C.jdf
COMNT	hexOH, Bn
DATIM	2014-03-22 19:03:22
OBNUC	13C
EXMOD	carbon.jxp
OBFRQ	125.77 MHz
OBSET	7.87 KHz
OBFIN	4.21 Hz
POINT	32767
FREQU	39308.18 Hz
SCANS	68
ACQTM	0.8336 sec
PD	3.0000 sec
PW1	3.40 usec
IRNUC	1H
CTEMP	21.6 c
SLVNT	CDCL3
EXREF	77.00 ppm
BF	0.12 Hz
RGAIN	60

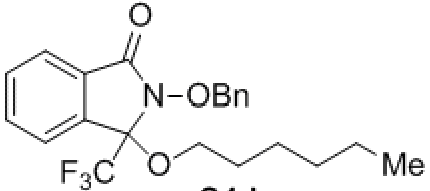

S4d

hexOH, Bn

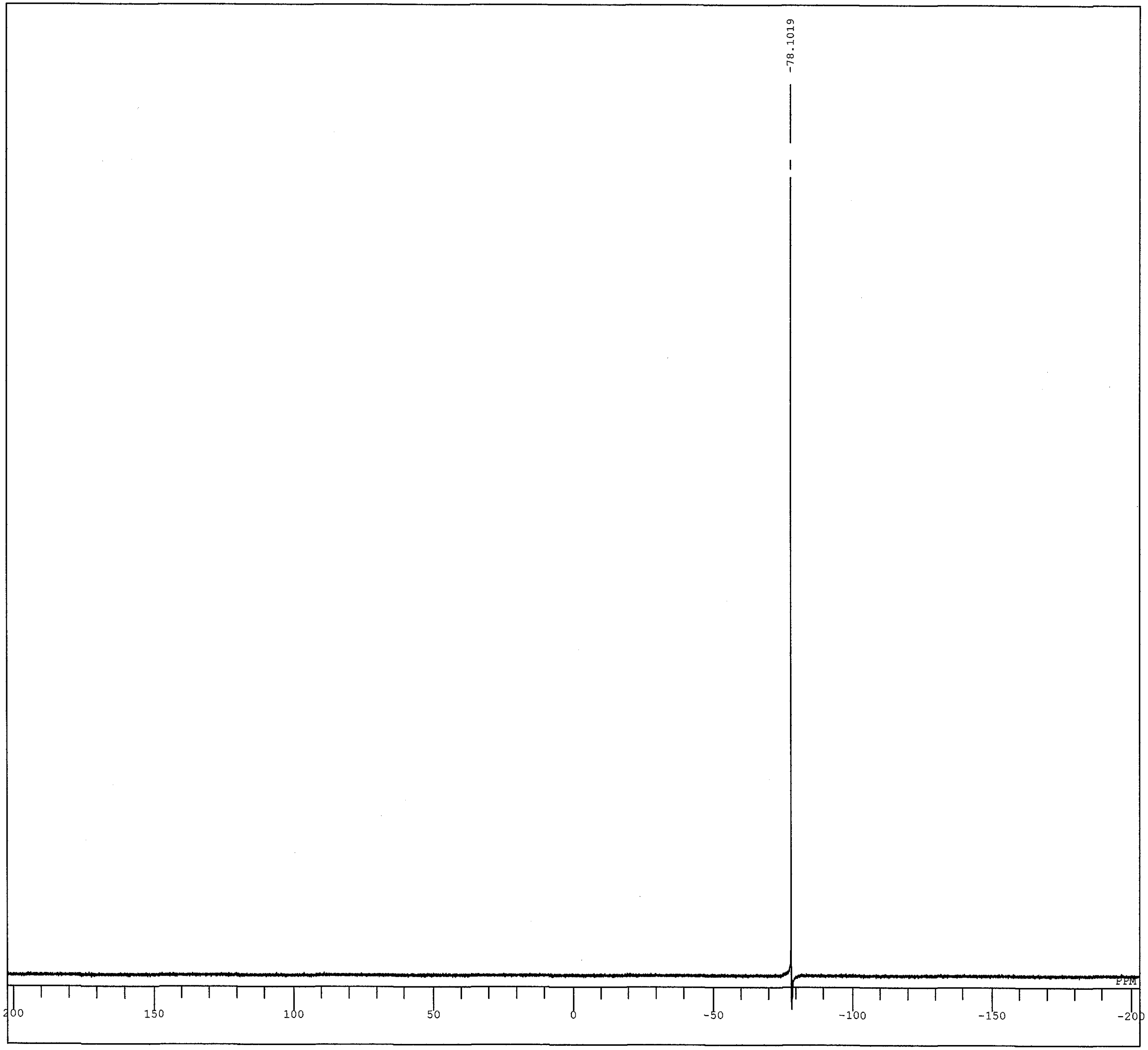

DFILE ozawa04-007_19F.jdf
COMNT hexOH, Bn
DATIM 15-03-2014 15:14:02
OBNUC 19F
EXMOD proton.jxp
OBFRQ 368.64 MHz
OBSET 7.63 KHz
OBFIN 2.85 Hz
POINT 16384
FREQU 186567.17 Hz
SCANS 8
ACQTM 0.0878 sec
PD 5.0000 sec
PW1 3.90 usec
IRNUC 19F
CTEMP 20.9 c
SLVNT CDCL3
EXREF -164.90 ppm
BF 0.12 Hz
RGAIN 46

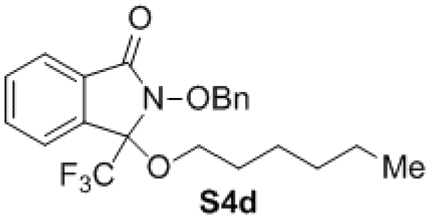

S4d

hexOH, OH

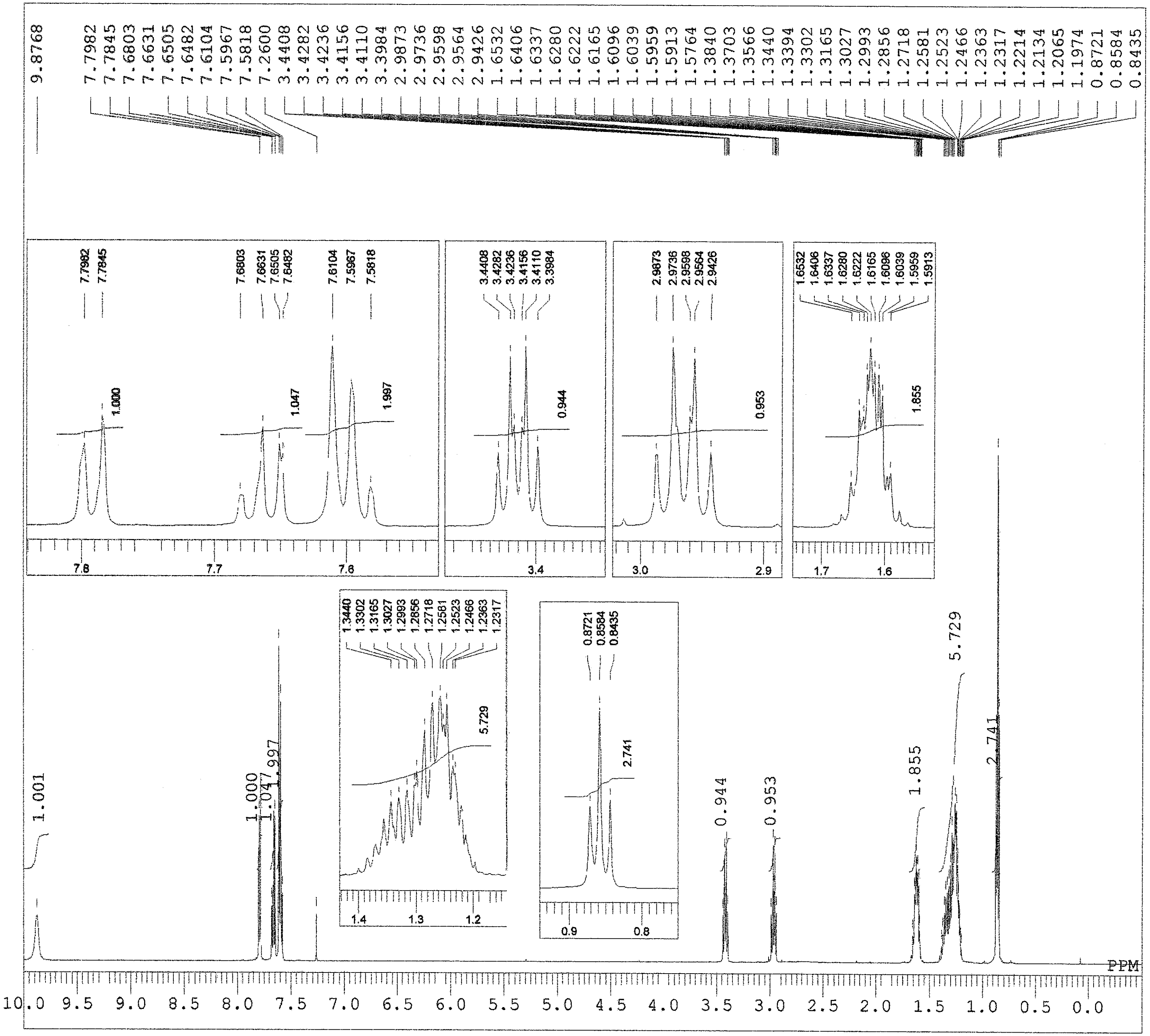

DFILE	ozawa04-010_1H.jdf
COMNT	hexOH, OH
DATIM	2014-01-17 19:39:58
OBNUC	1H
EXMOD	proton.jxp
OBFRQ	500.16 MHz
OBSET	2.41 KHz
OBFIN	6.01 Hz
POINT	16384
FREQU	9384.38 Hz
SCANS	4
ACQTM	1.7459 sec
PD	5.0000 sec
PW1	5.55 usec
IRNUC	1H
CTEMP	21.5 c
SLVNT	CDCL3
EXREF	7.26 ppm
BF	0.12 Hz
RGAIN	28

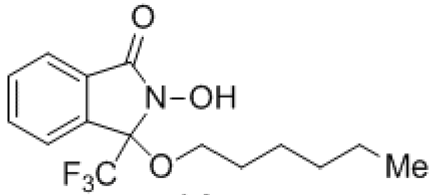

1d

hexOH, OH

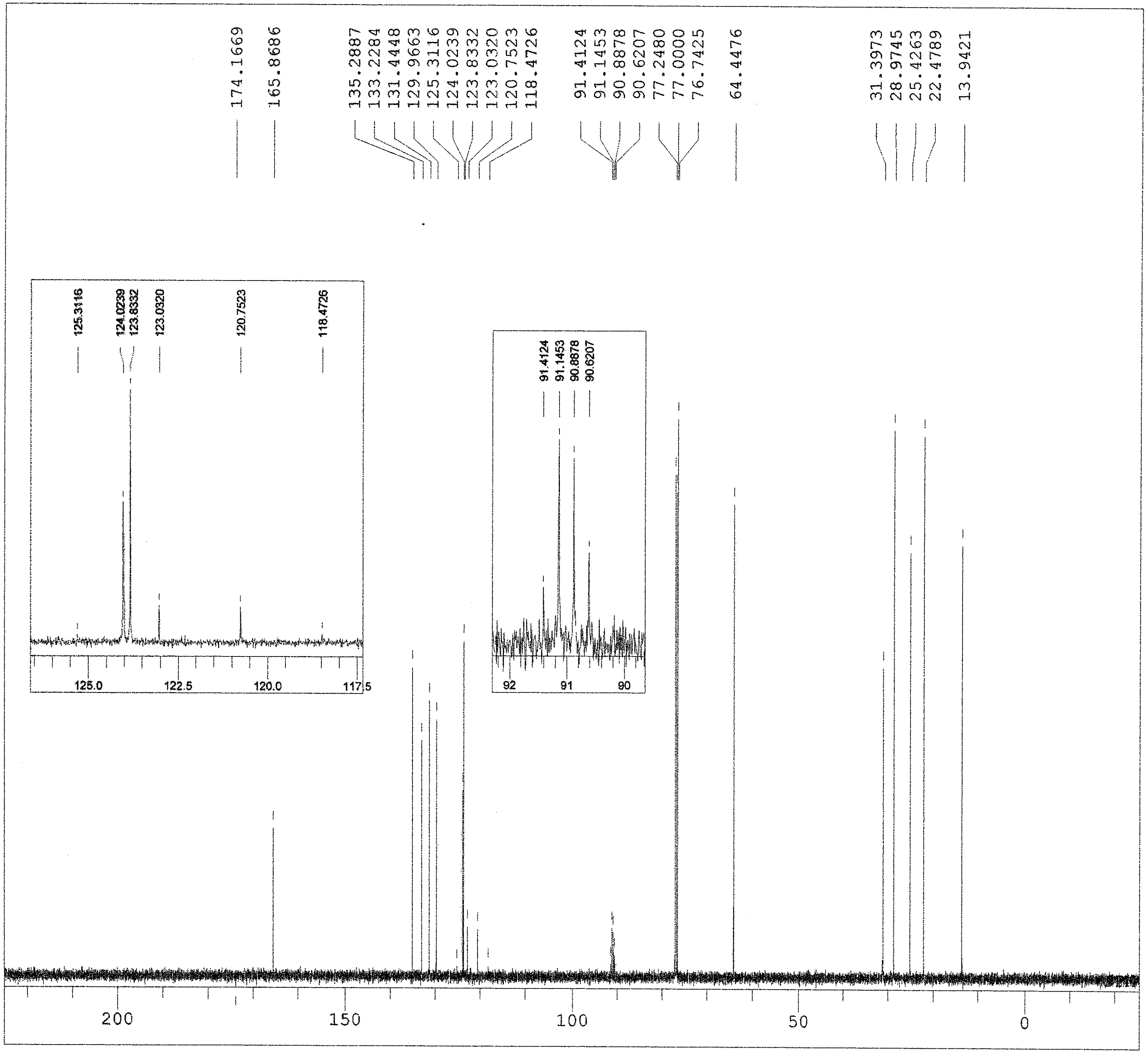

DFILE ozawa04-010_13C.jdf
COMNT hexOH, OH
DATIM 2014-01-17 19:41:00
OBNUC 13C
EXMOD carbon.jxp
OBFRQ 125.77 MHz
OBSET 7.87 KHz
OBFIN 4.21 Hz
POINT 32767
FREQU 39308.18 Hz
SCANS 164
ACQTM 0.8336 sec
PD 3.0000 sec
PW1 3.40 usec
IRNUC 1H
CTEMP 21.7 c
SLVNT CDCL3
EXREF 77.00 ppm
BF 0.12 Hz
RGAIN 60

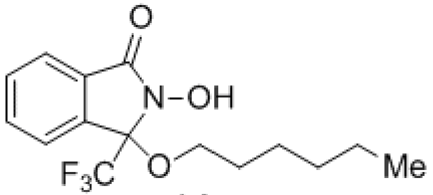

1d

hexanol, OH

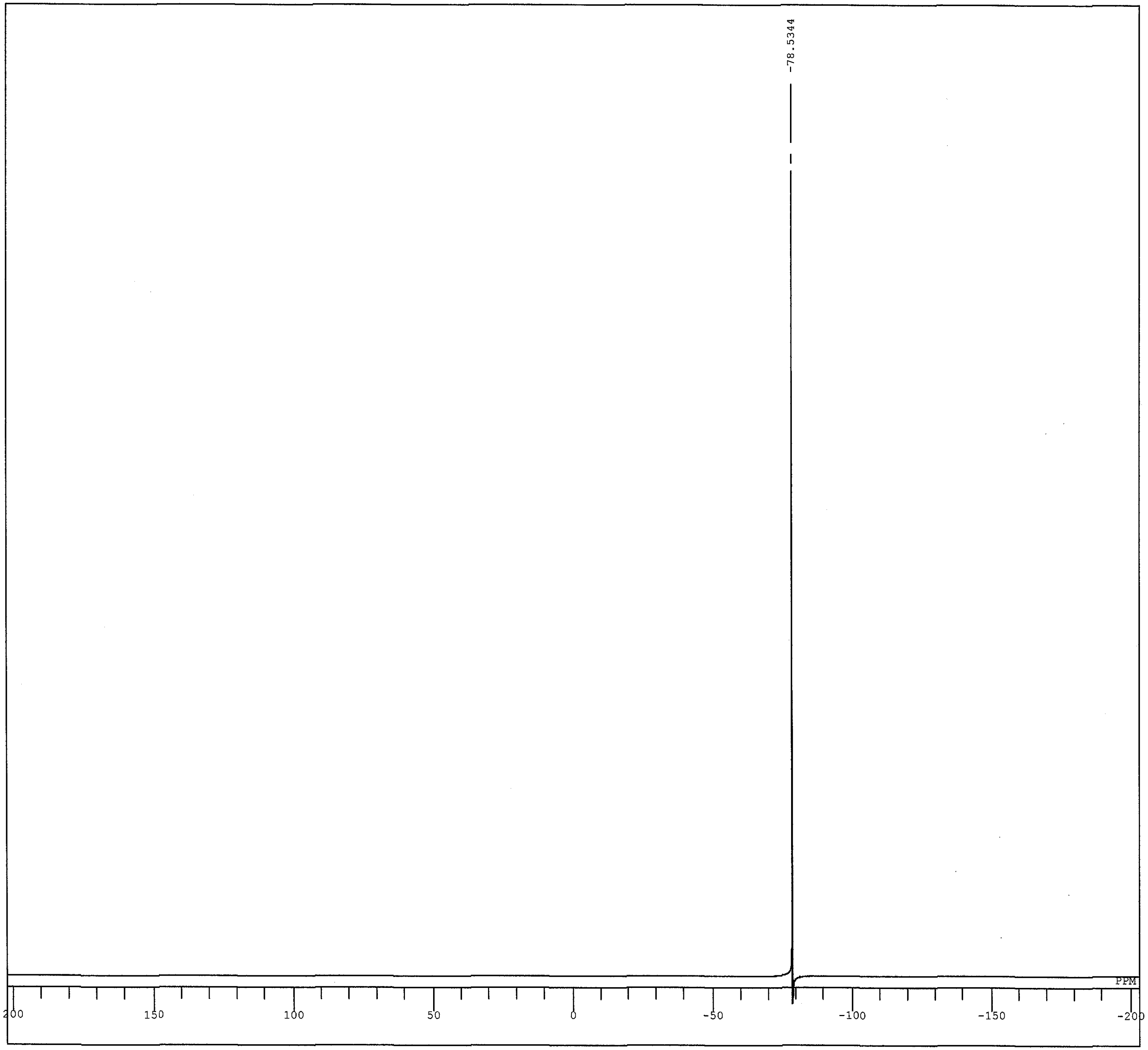

DFILE	ozawa04-010_19F.jdf
COMNT	hexanol, OH
DATIM	17-01-2014 13:04:22
OBNUC	19F
EXMOD	proton.jxp
OBFRQ	368.64 MHz
OBSET	7.63 KHz
OBFIN	2.85 Hz
POINT	16384
FREQU	186567.17 Hz
SCANS	4
ACQTM	0.0878 sec
PD	5.0000 sec
PW1	3.90 usec
IRNUC	19F
CTEMP	20.9 c
SLVNT	CDCL3
EXREF	-164.90 ppm
BF	0.12 Hz
RGAIN	44

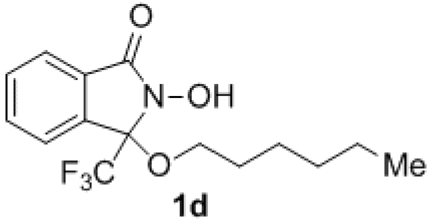

1d

hex, [O], LP

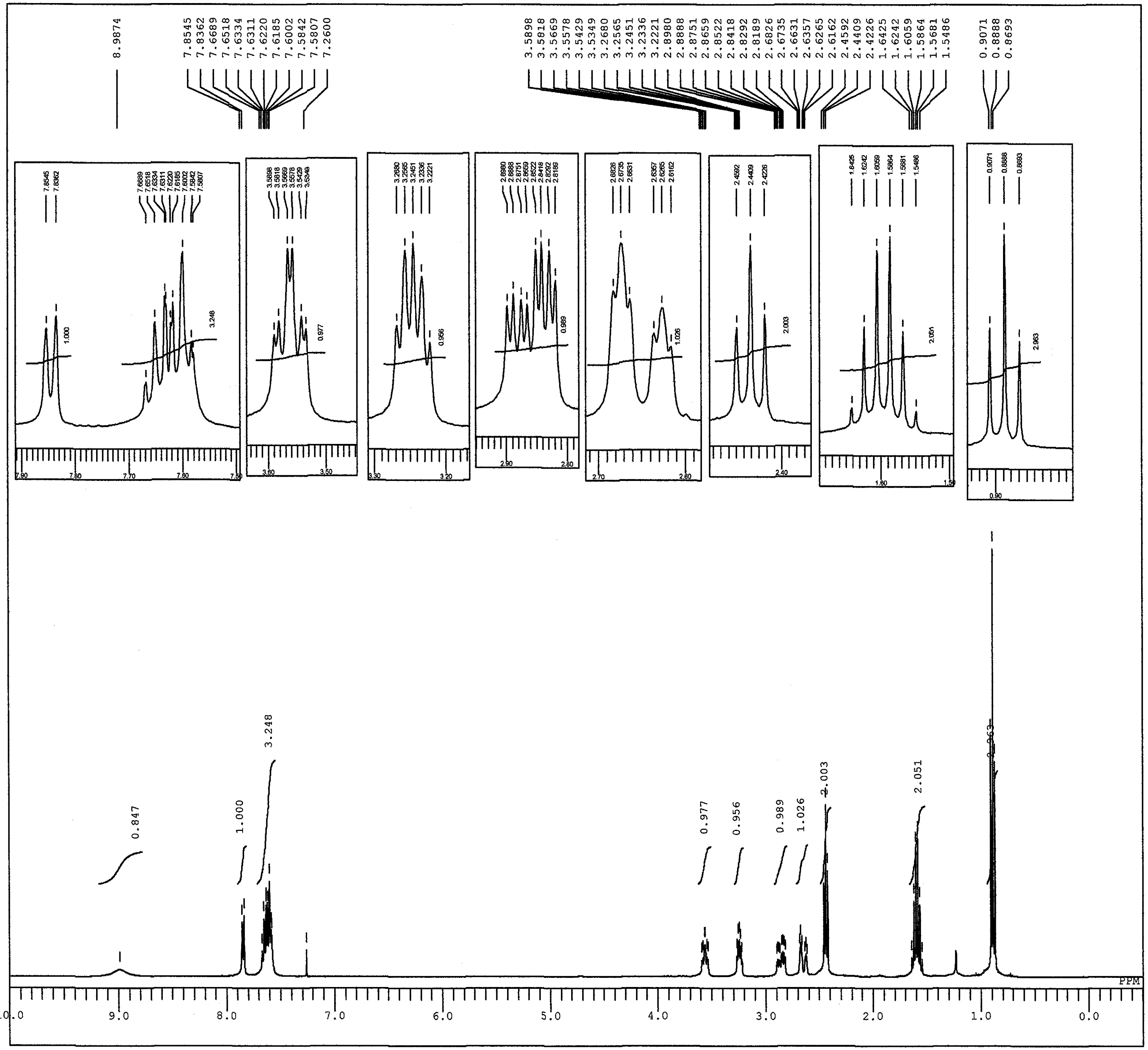

DFILE ozawa05-017_1_1H.jdf
COMNT hex, [O], LP
DATIM 28-05-2014 20:50:45
OBNUC 1H
EXMOD proton.jxp
OBFRQ 391.78 MHz
OBSET 8.51 KHz
OBFIN 3.34 Hz
POINT 16384
FREQU 7352.94 Hz
SCANS 4
ACQTM 2.2282 sec
PD 5.0000 sec
PW1 5.25 usec
IRNUC 1H
CTEMP 23.6 c
SLVNT CDCL3
EXREF 7.26 ppm
BF 0.12 Hz
RGAIN 26

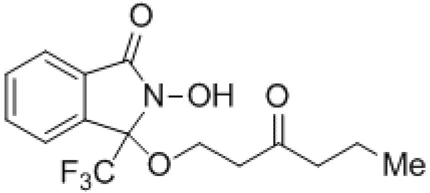

2d

hex, [O], LP

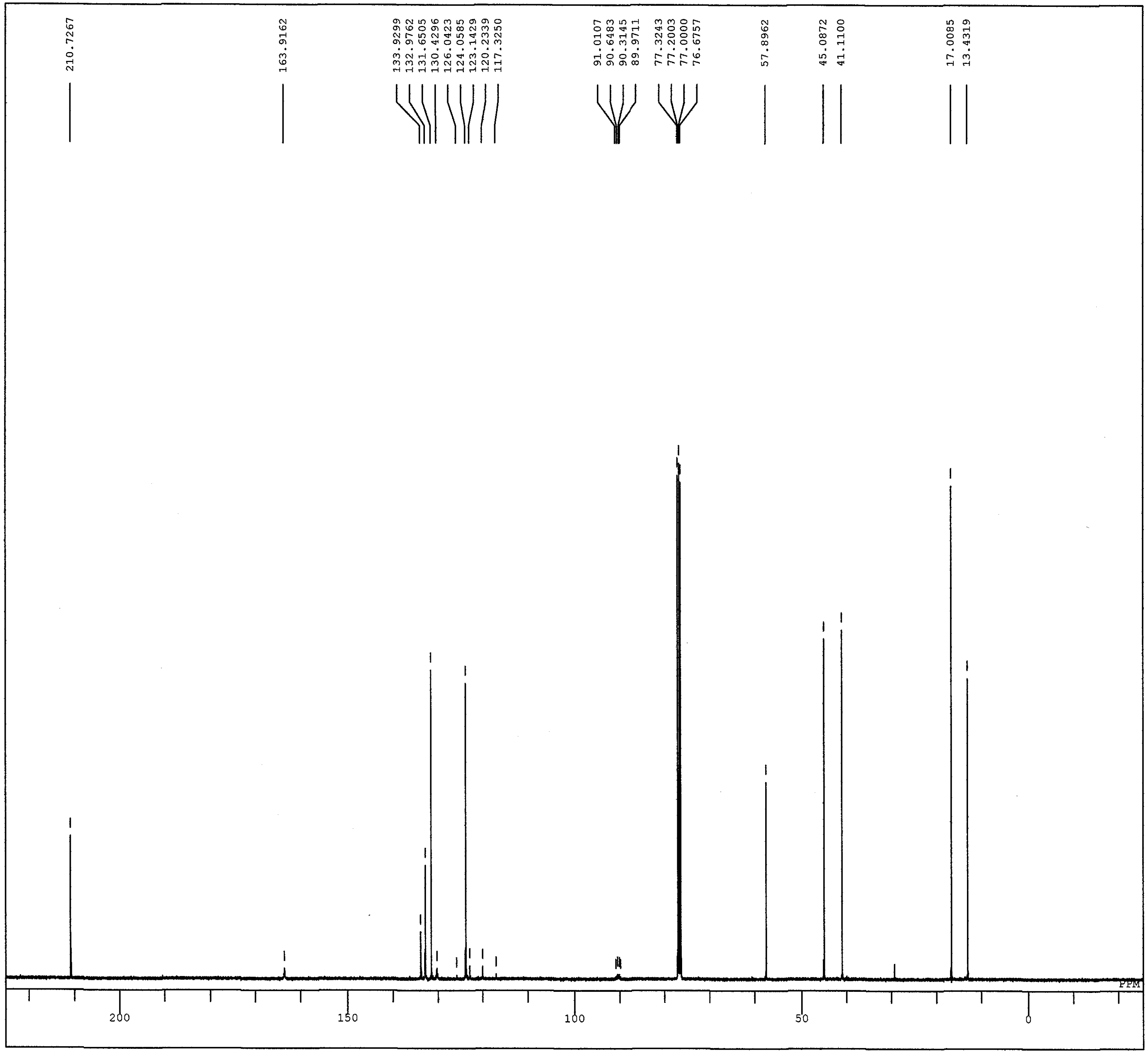

DFILE ozawa05-017_1_13C.als
COMNT hex, [O], LP
DATIM 28-05-2014 23:01:37
OBNUC 13C
EXMOD carbon.jxp
OBFRQ 98.52 MHz
OBSET 4.64 KHz
OBFIN 8.74 Hz
POINT 26214
FREQU 24630.54 Hz
SCANS 7000
ACQTM 1.0643 sec
PD 3.0000 sec
PW1 3.00 usec
IRNUC 1H
CTEMP 23.7 c
SLVNT CDCL3
EXREF 77.00 ppm
BF 0.12 Hz
RGAIN 60

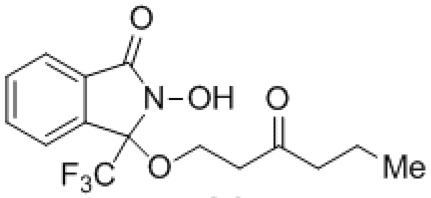

2d

hex, [O], LP

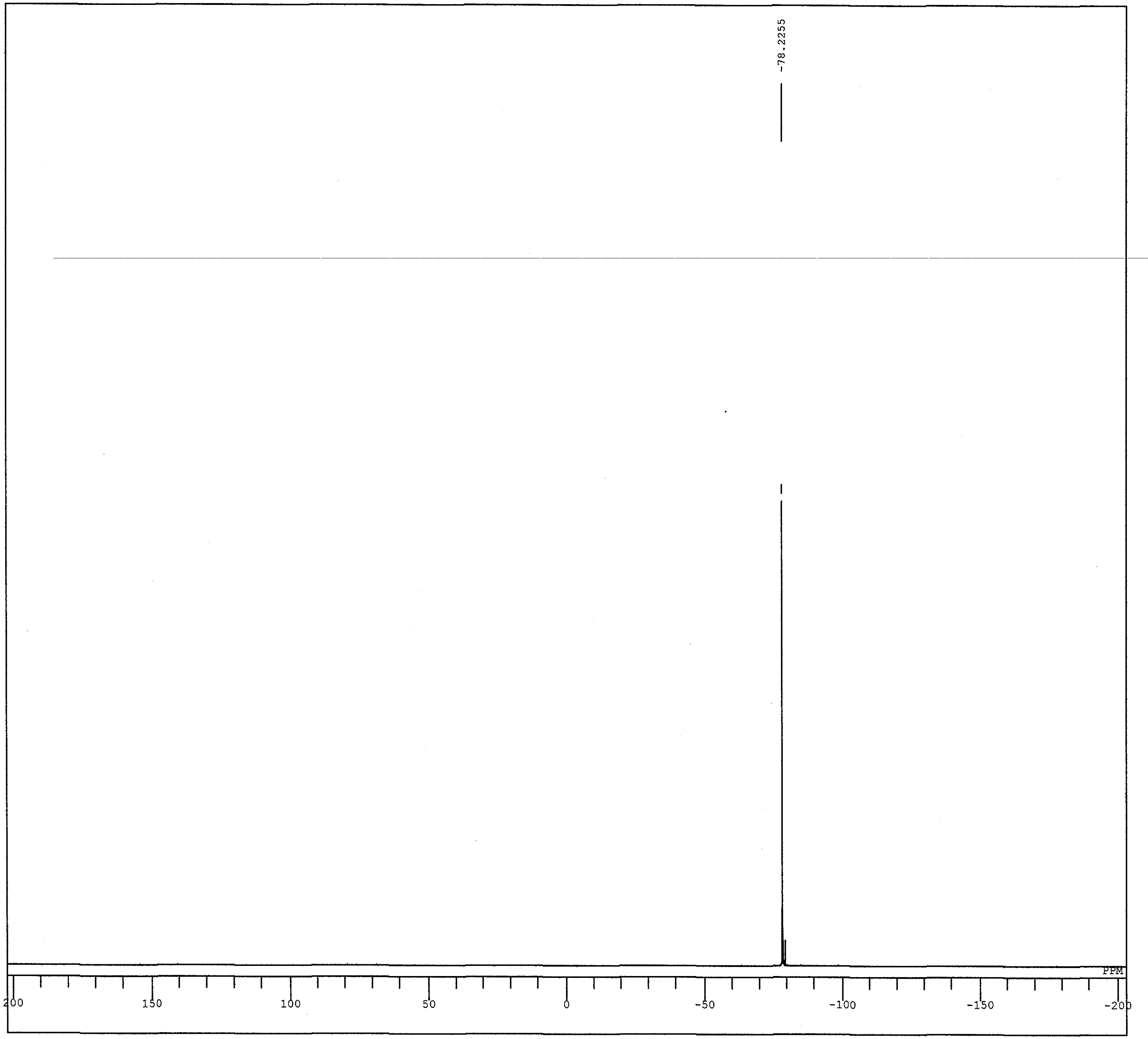

DFILE ozawa05-017_1_19F.jdf
COMNT hex, [O], LP
DATIM 28-05-2014 20:48:29
OBNUC 19F
EXMOD proton.jxp
OBFRQ 368.64 MHz
OBSET 7.63 KHz
OBFIN 2.85 Hz
POINT 16384
FREQU 186567.17 Hz
SCANS 4
ACQTM 0.0878 sec
PD 5.0000 sec
PW1 3.90 usec
IRNUC 19F
CTEMP 23.6 c
SLVNT CDCL3
EXREF -164.90 ppm
BF 0.12 Hz
RGAIN 44

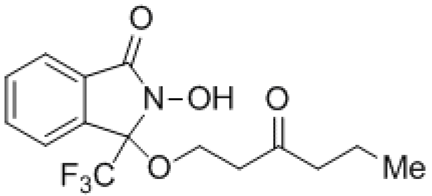

2d

hex, [O], MP

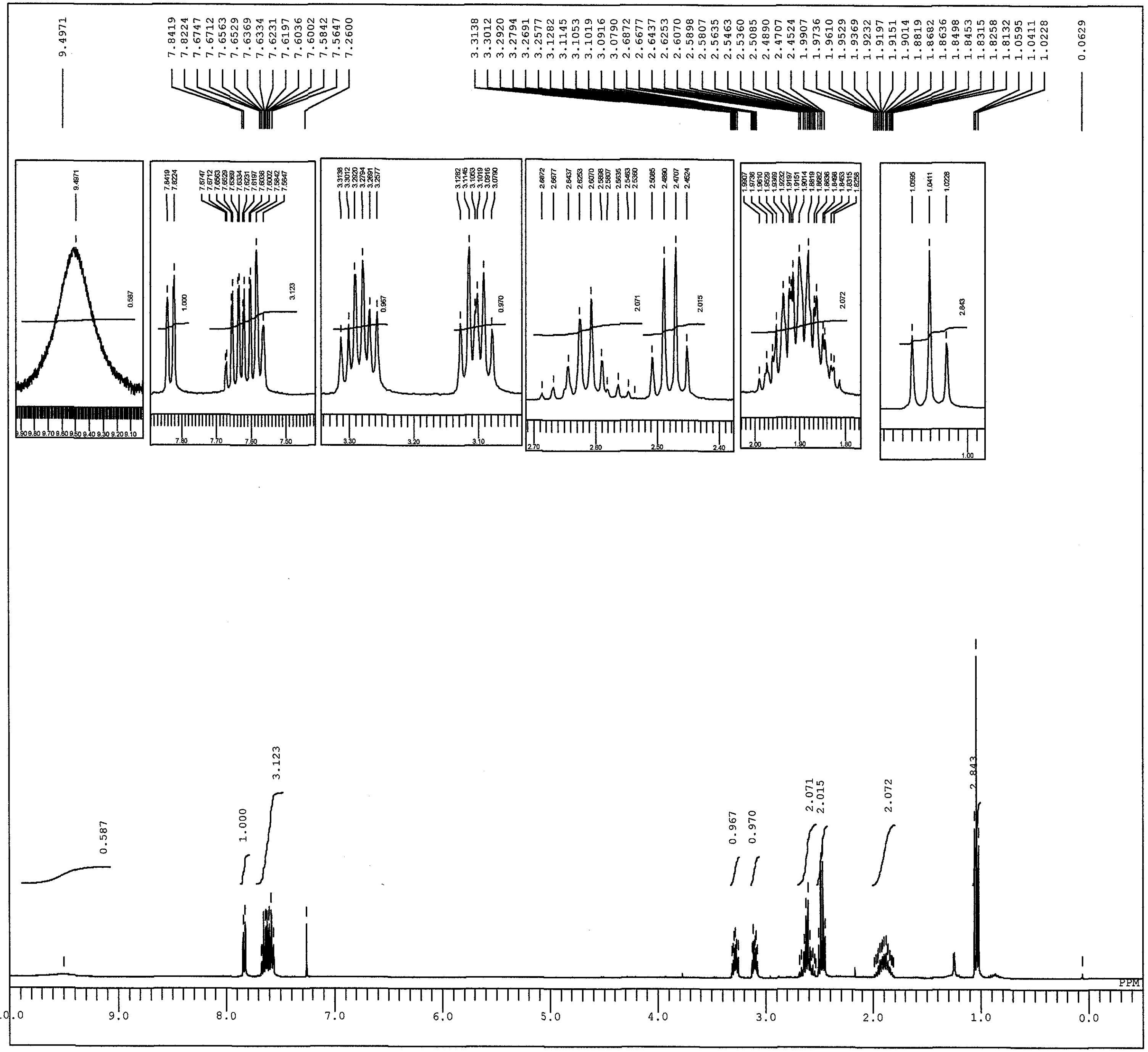

DFILE ozawa05-017_2_1H.jdf
COMNT hex, [O], MP
DATIM 28-05-2014 20:41:38
OBNUC 1H
EXMOD proton.jxp
OBFRQ 391.78 MHz
OBSET 8.51 KHz
OBFIN 3.34 Hz
POINT 16384
FREQU 7352.94 Hz
SCANS 4
ACQTM 2.2282 sec
PD 5.0000 sec
PW1 5.25 usec
IRNUC 1H
CTEMP 23.6 c
SLVNT CDCL3
EXREF 7.26 ppm
BF 0.12 Hz
RGAIN 30

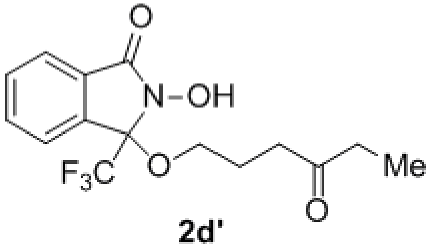

2d'

hex, [O], MP

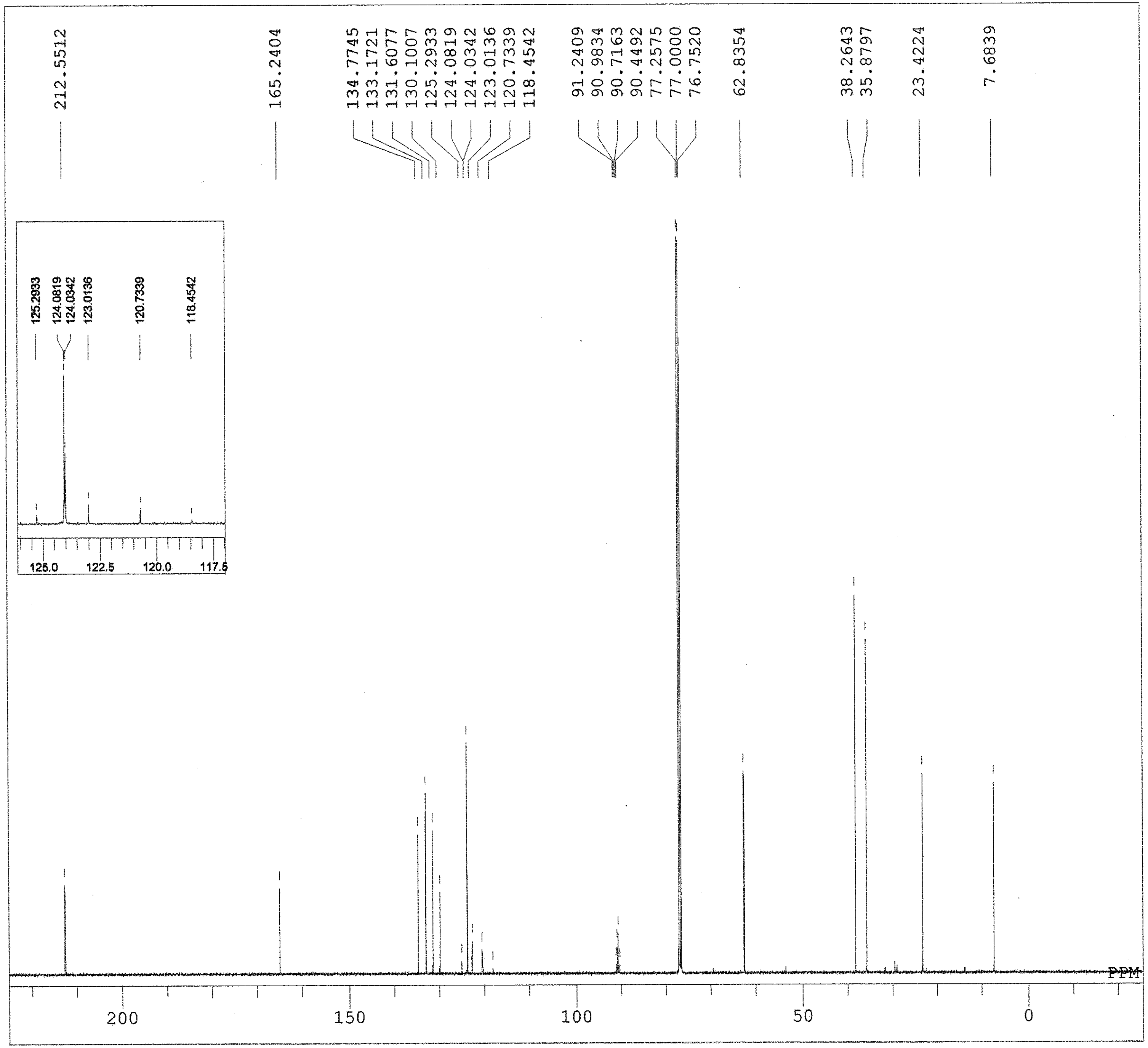

DFILE	ozawa05-017_2_13C.als	
COMNT	hex, [O], MP	
DATIM	2014-05-28 22:52:51	
OBNUC	13C	
EXMOD	carbon.jxp	
OBFRQ	125.77	MHz
OBSET	7.87	KHz
OBFIN	4.21	Hz
POINT	26214	
FREQU	31446.54	Hz
SCANS	10000	
ACQTM	0.8336	sec
PD	3.0000	sec
PW1	3.40	usec
IRNUC	1H	
CTEMP	26.4	c
SLVNT	CDCL3	
EXREF	77.00	ppm
BF	0.12	Hz
RGAIN	60	

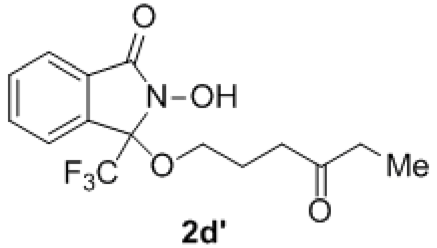

2d'

hex, [O], MP

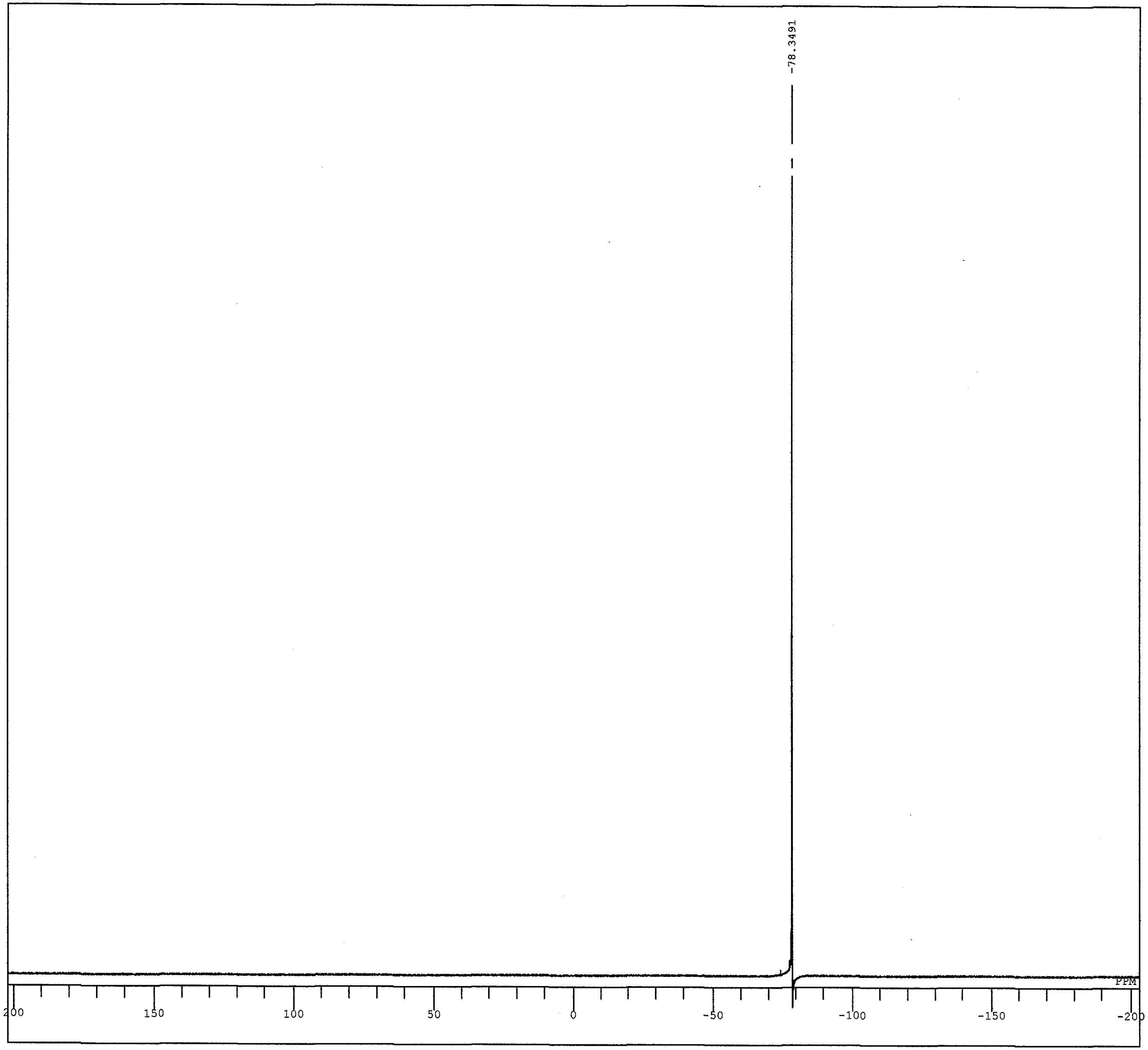

DFILE	ozawa05-017_2_19F.jdf
COMNT	hex, [O], MP
DATIM	28-05-2014 20:43:43
OBNUC	19F
EXMOD	proton.jxp
OBFRQ	368.64 MHz
OBSET	7.63 KHz
OBFIN	2.85 Hz
POINT	16384
FREQU	186567.17 Hz
SCANS	4
ACQTM	0.0878 sec
PD	5.0000 sec
PW1	3.90 usec
IRNUC	19F
CTEMP	23.6 c
SLVNT	CDCL3
EXREF	-164.90 ppm
BF	0.12 Hz
RGAIN	46

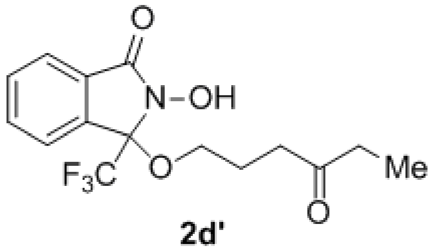

2d'

OAc, Bn

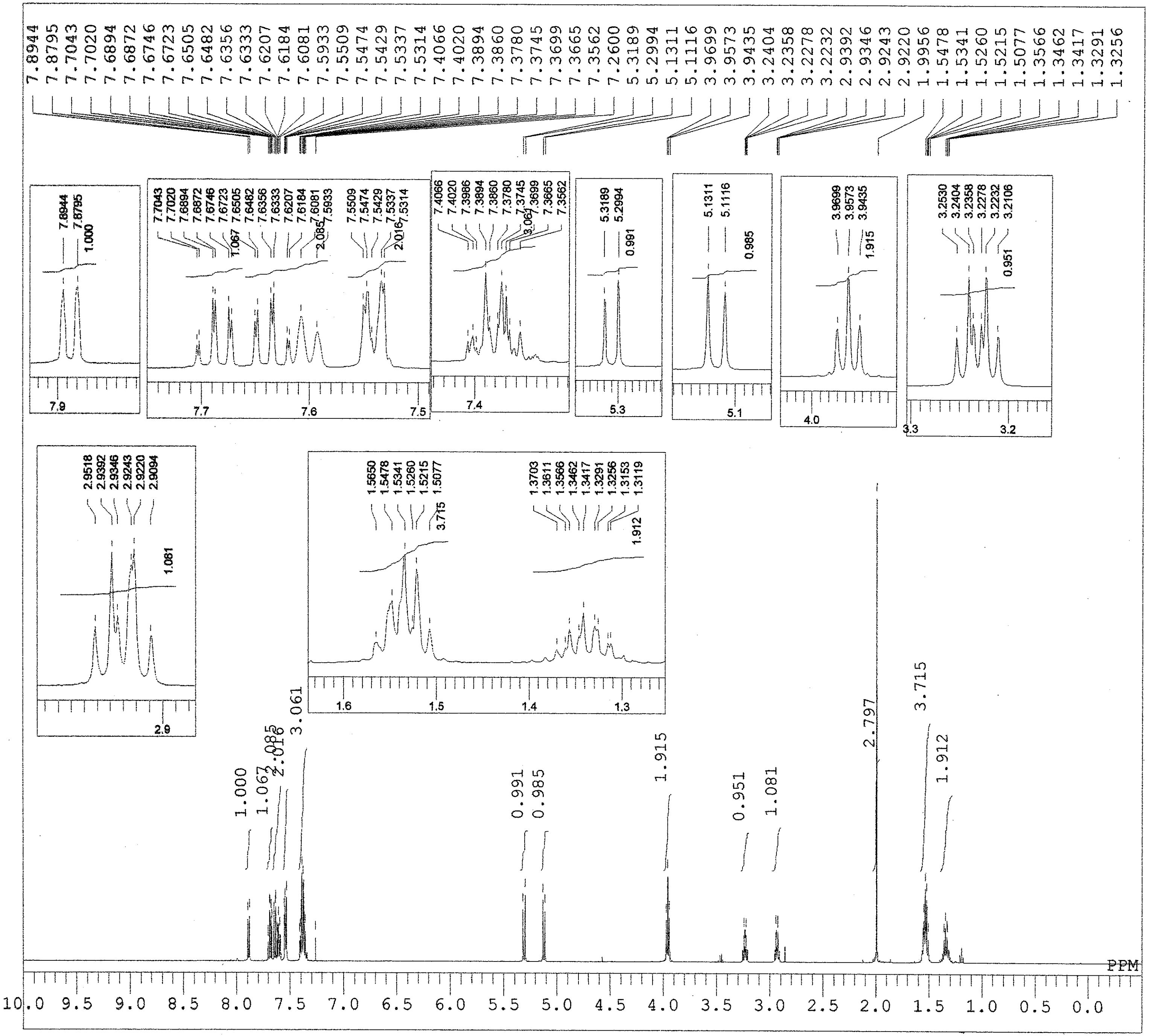

DFILE	ozawa04-122_1_C_1H.jdf
COMNT	OAc, Bn
DATIM	2014-02-02 13:56:54
OBNUC	1H
EXMOD	proton.jxp
OBFRQ	500.16 MHz
OBSET	2.41 KHz
OBFIN	6.01 Hz
POINT	16384
FREQU	9384.38 Hz
SCANS	4
ACQTM	1.7459 sec
PD	5.0000 sec
PW1	5.55 usec
IRNUC	1H
CTEMP	21.2 c
SLVNT	CDCL3
EXREF	7.26 ppm
BF	0.12 Hz
RGAIN	28

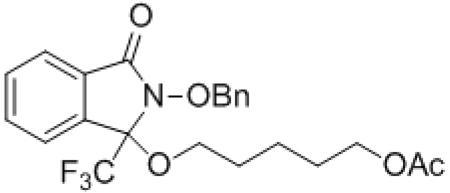

S4e

OAc, Bn

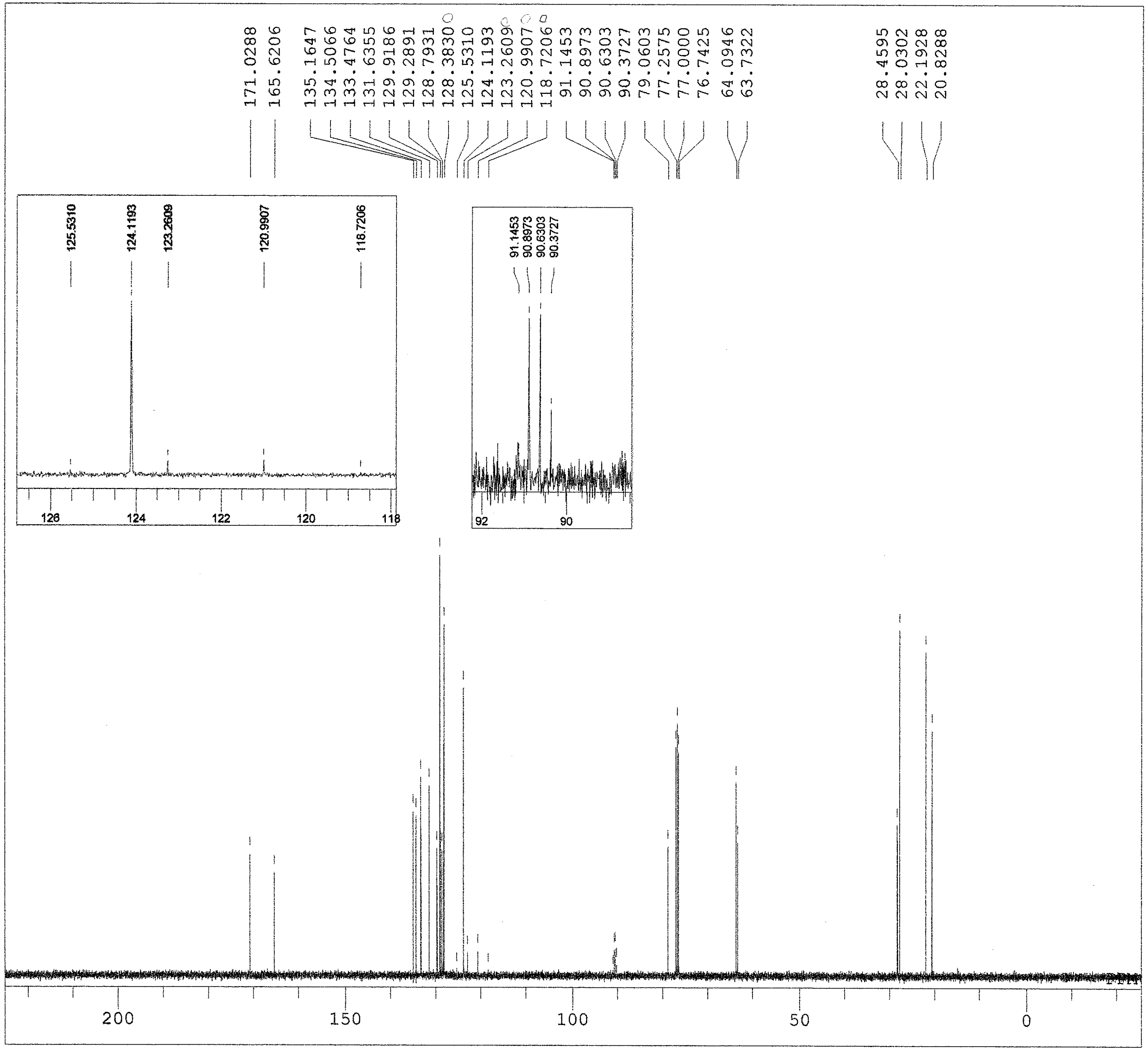

DFILE	ozawa04-122_1_C_13C.jdf
COMNT	OAc, Bn
DATIM	2014-02-02 13:57:58
OBNUC	13C
EXMOD	carbon.jxp
OBFRQ	125.77 MHz
OBSET	7.87 KHz
OBFIN	4.21 Hz
POINT	32767
FREQU	39308.18 Hz
SCANS	88
ACQTM	0.8336 sec
PD	3.0000 sec
PW1	3.40 usec
IRNUC	1H
CTEMP	22.1 c
SLVNT	CDCL3
EXREF	77.00 ppm
BF	0.12 Hz
RGAIN	60

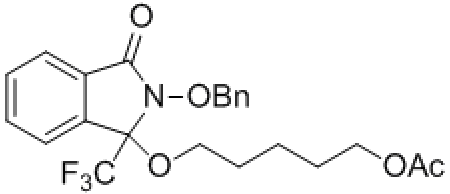

S4e

OAc, Bn

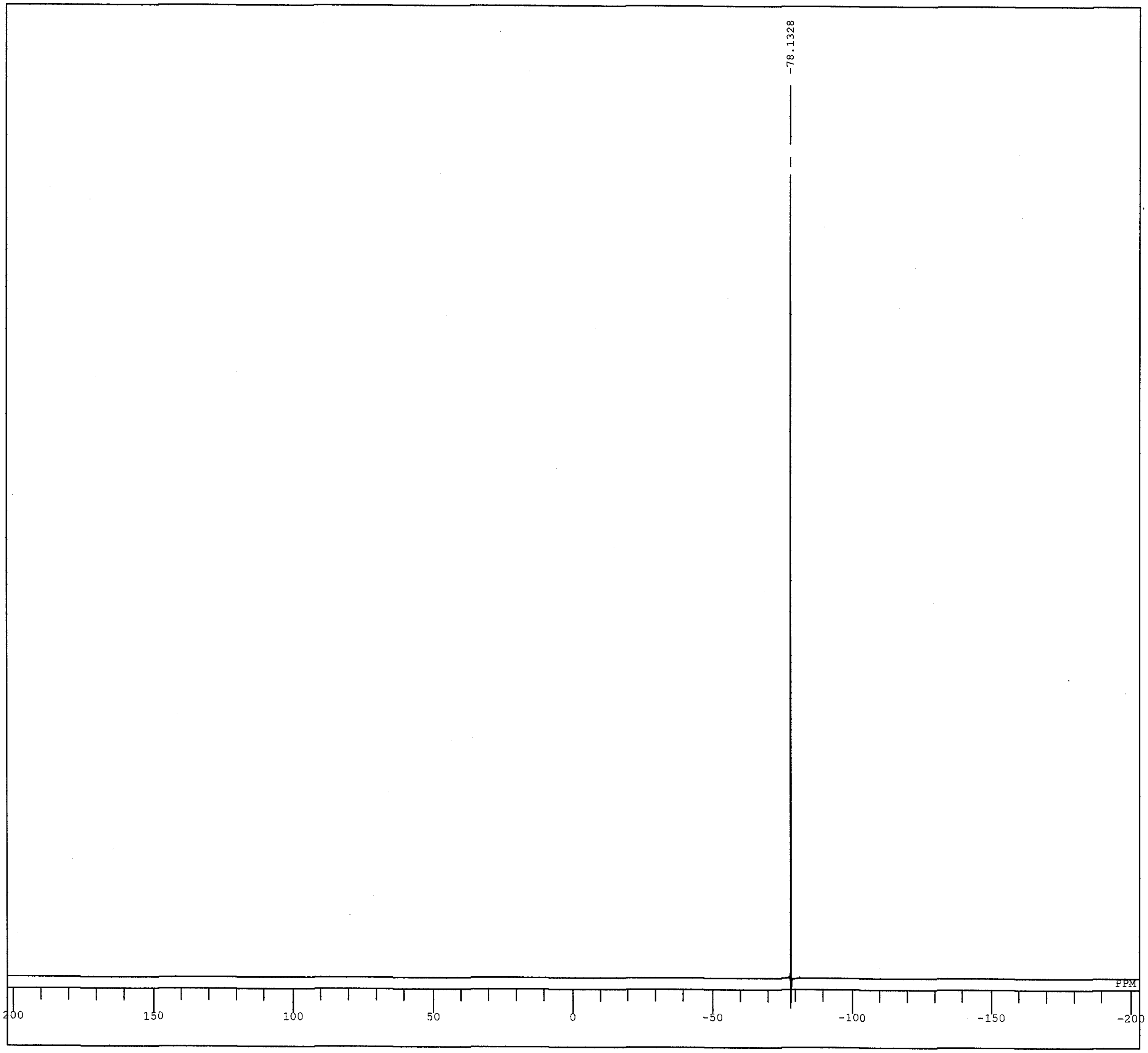

DFILE ozawa04-122_1_C_19F.jdf
COMNT OAc, Bn
DATIM 02-02-2014 14:37:26
OBNUC 19F
EXMOD proton.jxp
OBFRQ 368.64 MHz
OBSET 7.63 KHz
OBFIN 2.85 Hz
POINT 16384
FREQU 186567.17 Hz
SCANS 4
ACQTM 0.0878 sec
PD 5.0000 sec
PW1 3.90 usec
IRNUC 19F
CTEMP 21.0 c
SLVNT CDCL3
EXREF -164.90 ppm
BF 0.12 Hz
RGAIN 46

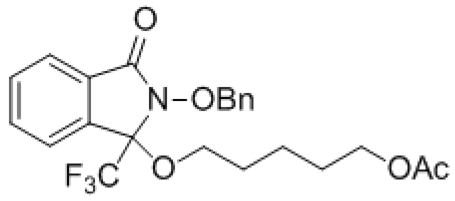

S4e

OAc, OH

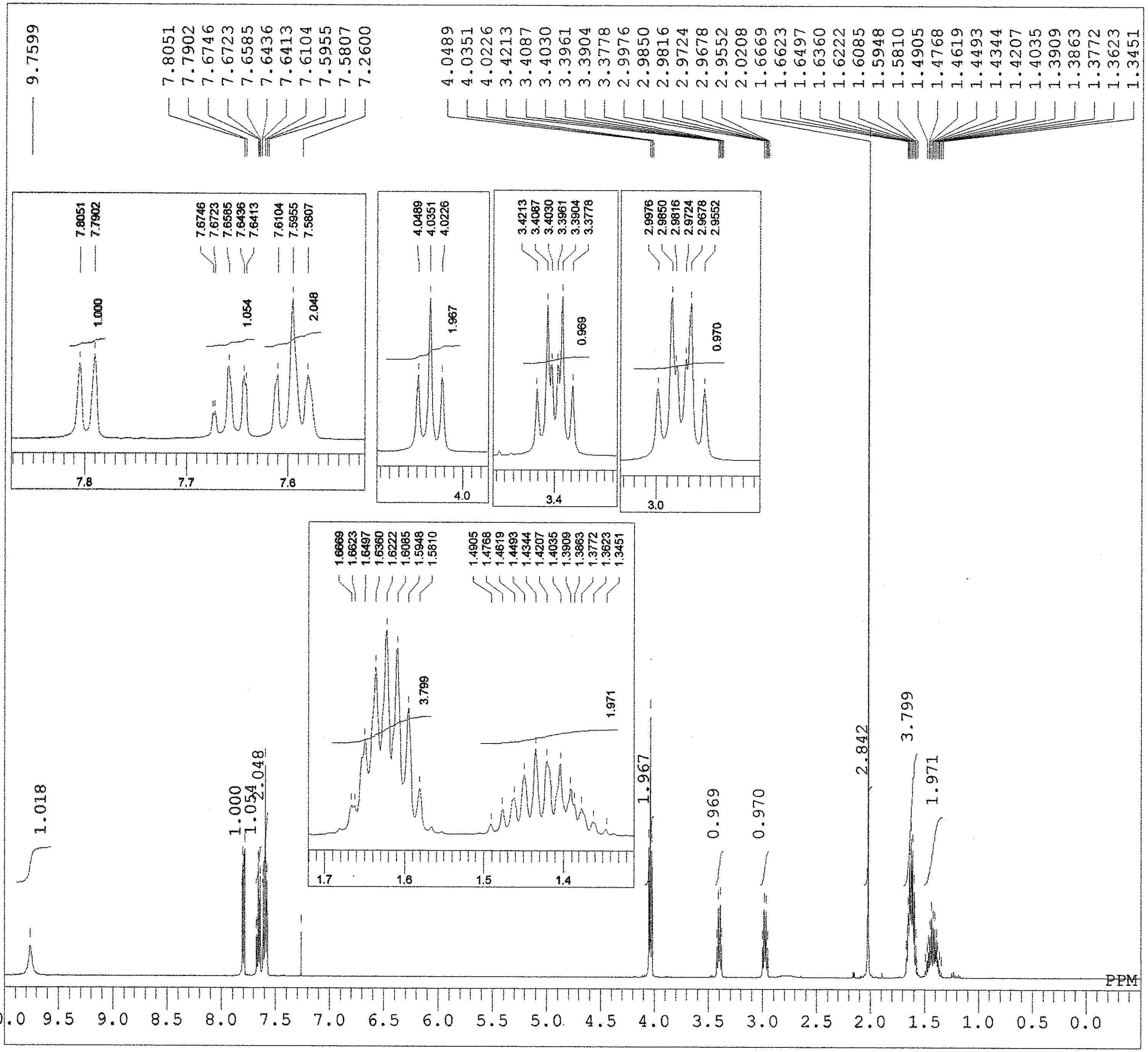

DFILE	ozawa04-024_1H.jdf
COMNT	OAc, OH
DATIM	2014-02-01 21:14:19
OBNUC	1H
EXMOD	proton.jxp
OBFRQ	500.16 MHz
OBSET	2.41 KHz
OBFIN	6.01 Hz
POINT	16384
FREQU	9384.38 Hz
SCANS	4
ACQTM	1.7459 sec
PD	5.0000 sec
PW1	5.55 usec
IRNUC	1H
CTEMP	21.2 c
SLVNT	CDCL3
EXREF	7.26 ppm
BF	0.12 Hz
RGAIN	28

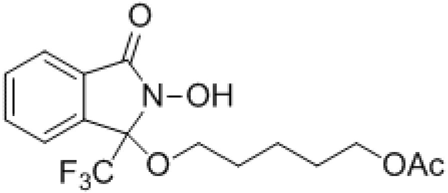

1e

OAc, OH

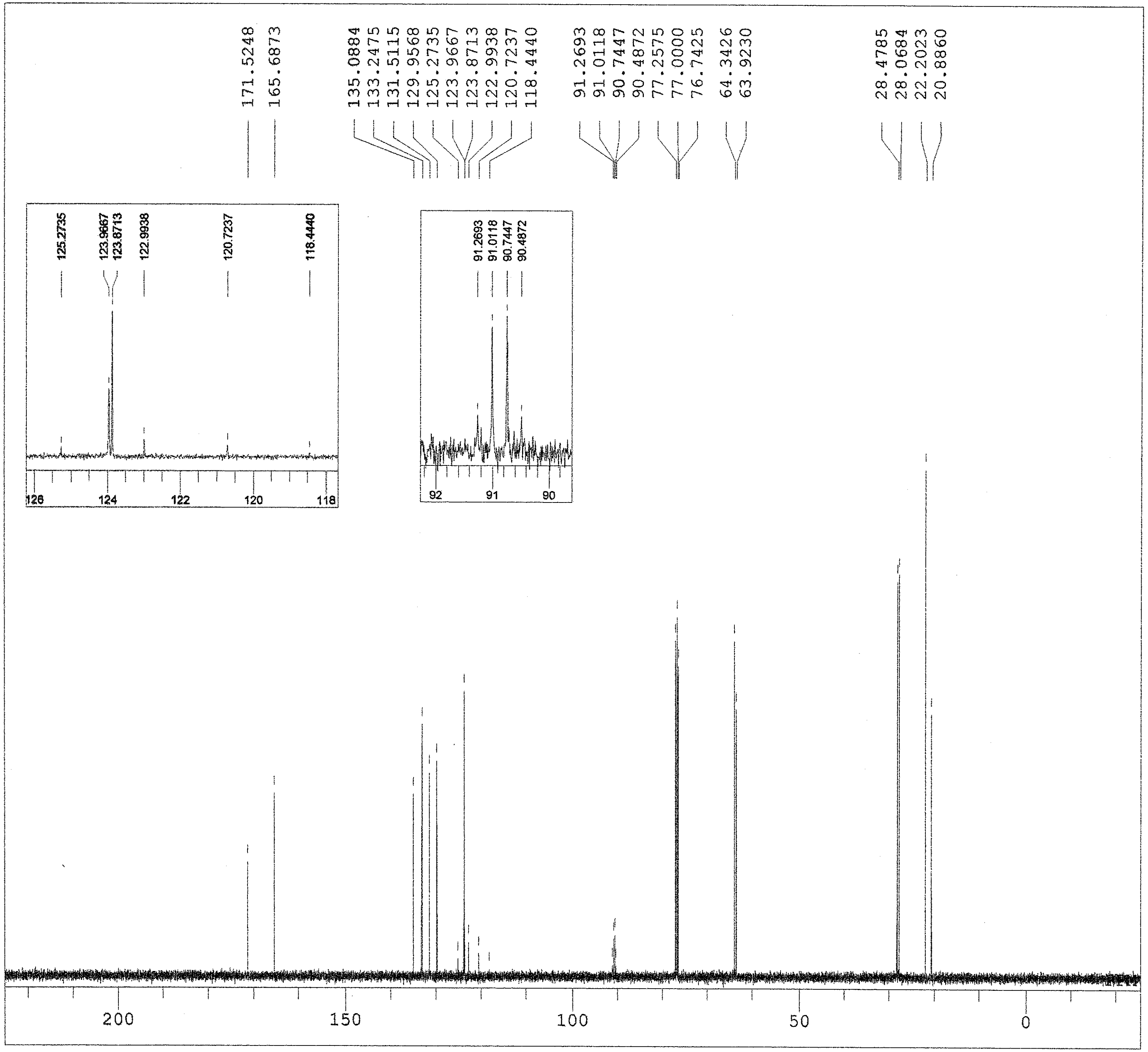

DFILE	ozawa04-024_13C.jdf
COMNT	OAc, OH
DATIM	2014-02-01 21:15:23
OBNUC	13C
EXMOD	carbon.jxp
OBFRQ	125.77 MHz
OBSET	7.87 KHz
OBFIN	4.21 Hz
POINT	32767
FREQU	39308.18 Hz
SCANS	112
ACQTM	0.8336 sec
PD	3.0000 sec
PW1	3.40 usec
IRNUC	1H
CTEMP	21.9 c
SLVNT	CDCL3
EXREF	77.00 ppm
BF	0.12 Hz
RGAIN	60

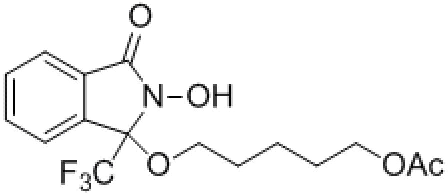

1e

OAc, OH

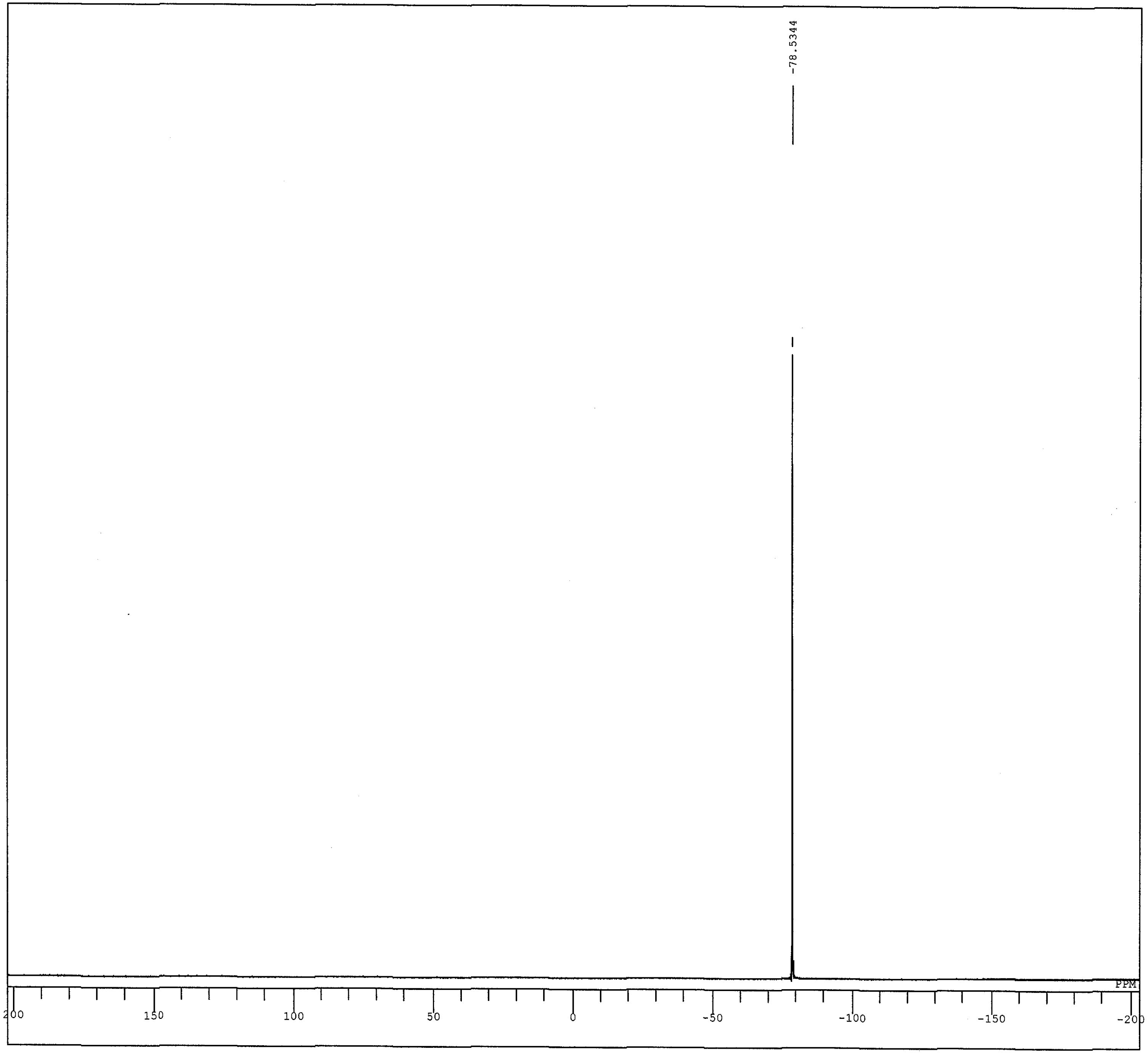


```
DFILE  ozawa04-024_19F.jdf
COMNT  OAc, OH
DATIM  01-02-2014 21:41:09
OBNUC  19F
EXMOD  proton.jxp
OBFRQ          368.64 MHz
OBSET            7.63 KHz
OBFIN            2.85 Hz
POINT           16384
FREQU       186567.17 Hz
SCANS               4
ACQTM          0.0878 sec
PD             5.0000 sec
PW1              3.90 usec
IRNUC  19F
CTEMP            21.2 c
SLVNT  CDCL3
EXREF         -164.90 ppm
BF               0.12 Hz
RGAIN              44
```

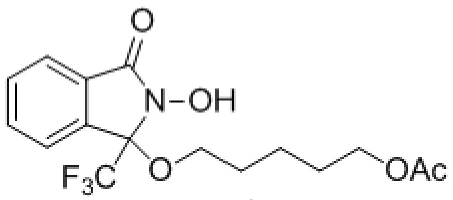

1e

OAc, [0]

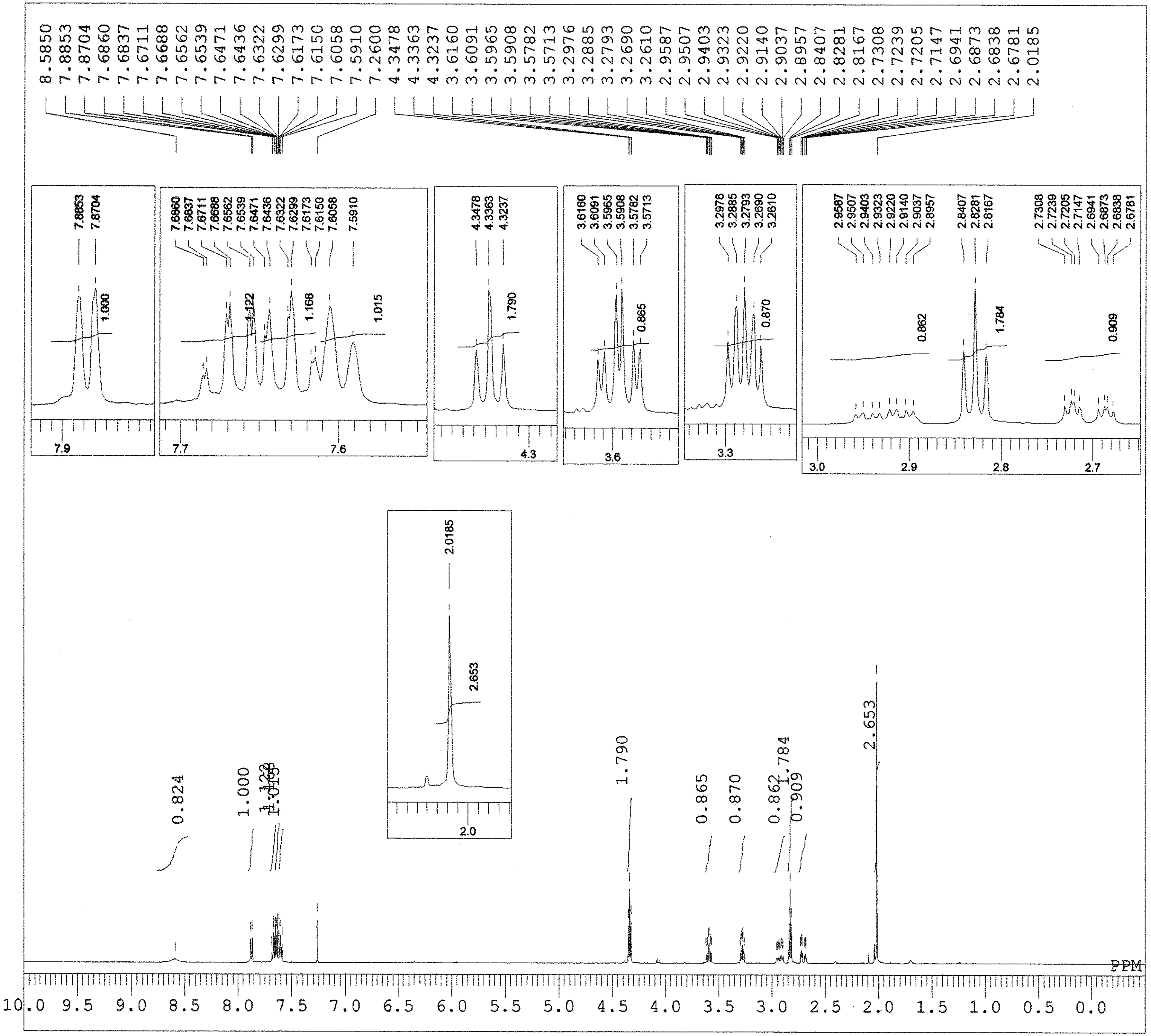

DFILE	ozawa04-121_1H.jdf
COMNT	OAc, [O]
DATIM	2014-02-05 20:43:59
OBNUC	1H
EXMOD	proton.jxp
OBFRQ	500.16 MHz
OBSET	2.41 KHz
OBFIN	6.01 Hz
POINT	16384
FREQU	9384.38 Hz
SCANS	4
ACQTM	1.7459 sec
PD	5.0000 sec
PW1	5.55 usec
IRNUC	1H
CTEMP	22.1 c
SLVNT	CDCL3
EXREF	7.26 ppm
BF	0.12 Hz
RGAIN	36

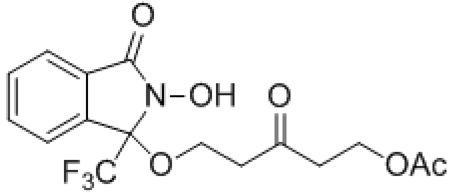

2e

OAc, [O]

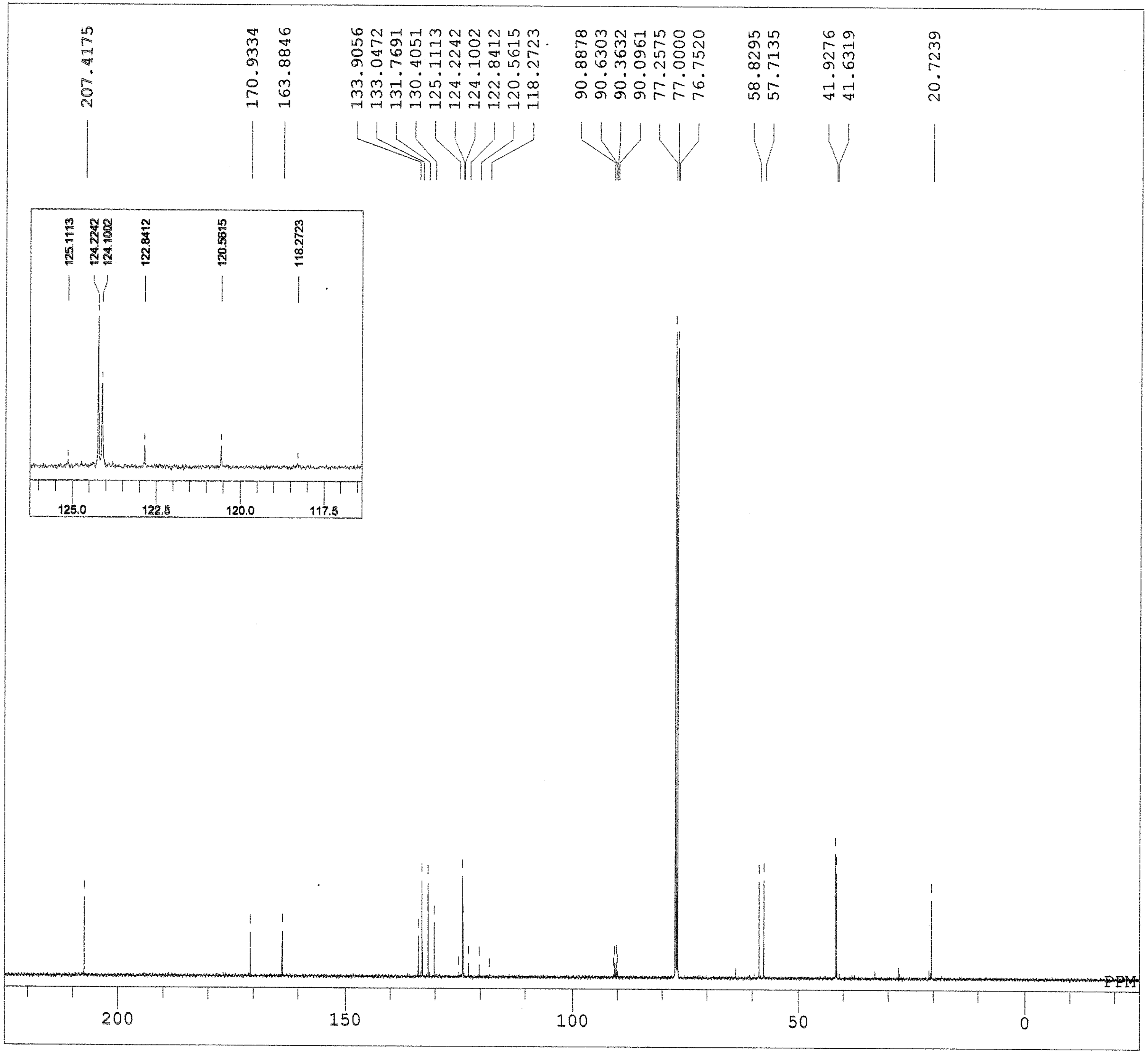

DFILE	2i_ozawa04-121_13C.als
COMNT	OAc, [O]
DATIM	2014-02-09 10:14:17
OBNUC	13C
EXMOD	carbon.jxp
OBFRQ	125.77 MHz
OBSET	7.87 KHz
OBFIN	4.21 Hz
POINT	32767
FREQU	39308.18 Hz
SCANS	2116
ACQTM	0.8336 sec
PD	3.0000 sec
PW1	3.40 usec
IRNUC	1H
CTEMP	21.4 c
SLVNT	CDCL3
EXREF	77.00 ppm
BF	1.00 Hz
RGAIN	60

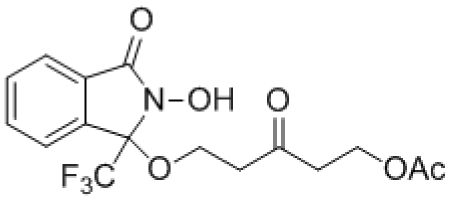

2e

OAc, [O]

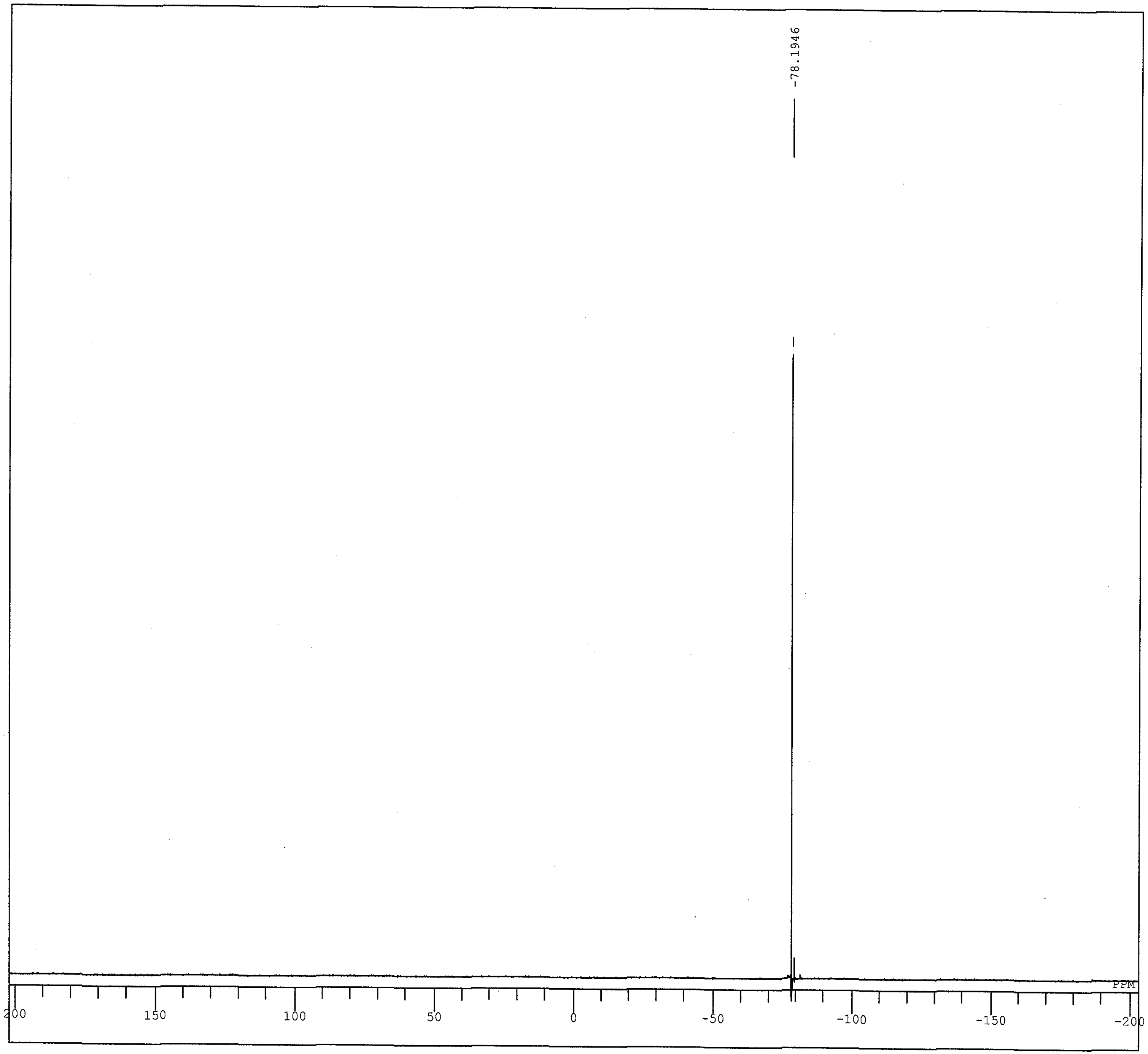

DFILE	ozawa04-121_19F.jdf
COMNT	OAc, [O]
DATIM	09-02-2014 12:54:25
OBNUC	19F
EXMOD	proton.jxp
OBFRQ	368.64 MHz
OBSET	7.63 KHz
OBFIN	2.85 Hz
POINT	16384
FREQU	186567.17 Hz
SCANS	4
ACQTM	0.0878 sec
PD	5.0000 sec
PW1	3.90 usec
IRNUC	19F
CTEMP	20.9 c
SLVNT	CDCL3
EXREF	-164.90 ppm
BF	0.05 Hz
RGAIN	48

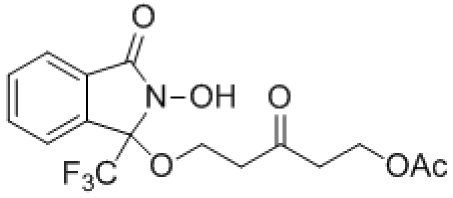

2e

C5-NPhth, Bn

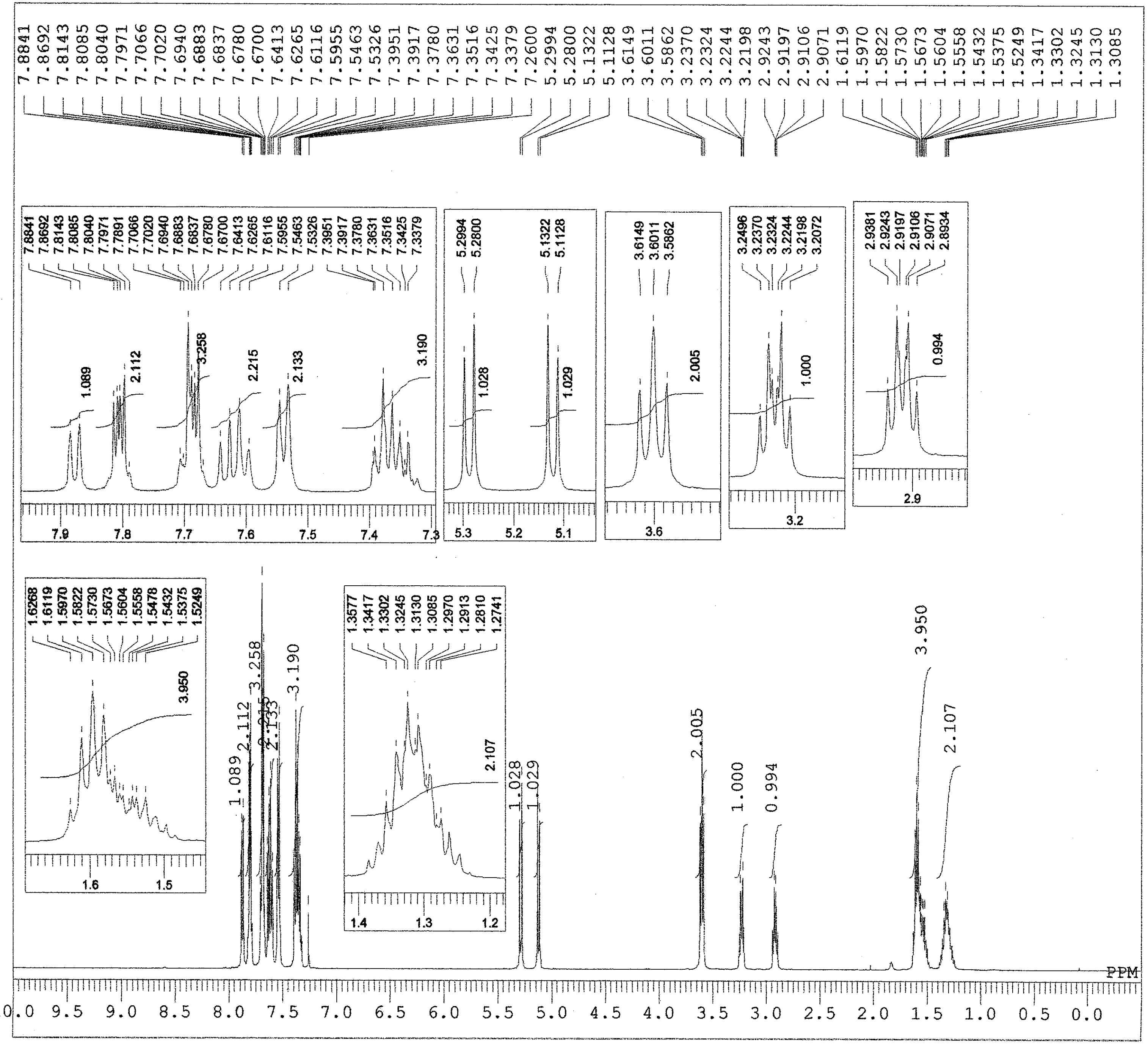

DFILE ozawa04-142_2_1H.jdf
COMNT C5-NPhth, Bn
DATIM 2014-03-06 19:38:37
OBNUC 1H
EXMOD proton.jxp
OBFRQ 500.16 MHz
OBSET 2.41 KHz
OBFIN 6.01 Hz
POINT 16384
FREQU 9384.38 Hz
SCANS 4
ACQTM 1.7459 sec
PD 5.0000 sec
PW1 5.55 usec
IRNUC 1H
CTEMP 21.6 c
SLVNT CDCL3
EXREF 7.26 ppm
BF 0.12 Hz
RGAIN 30

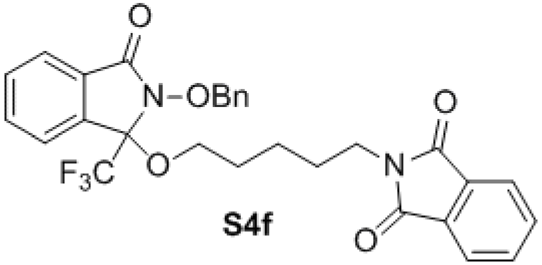

S4f

C5-NPhth, Bn

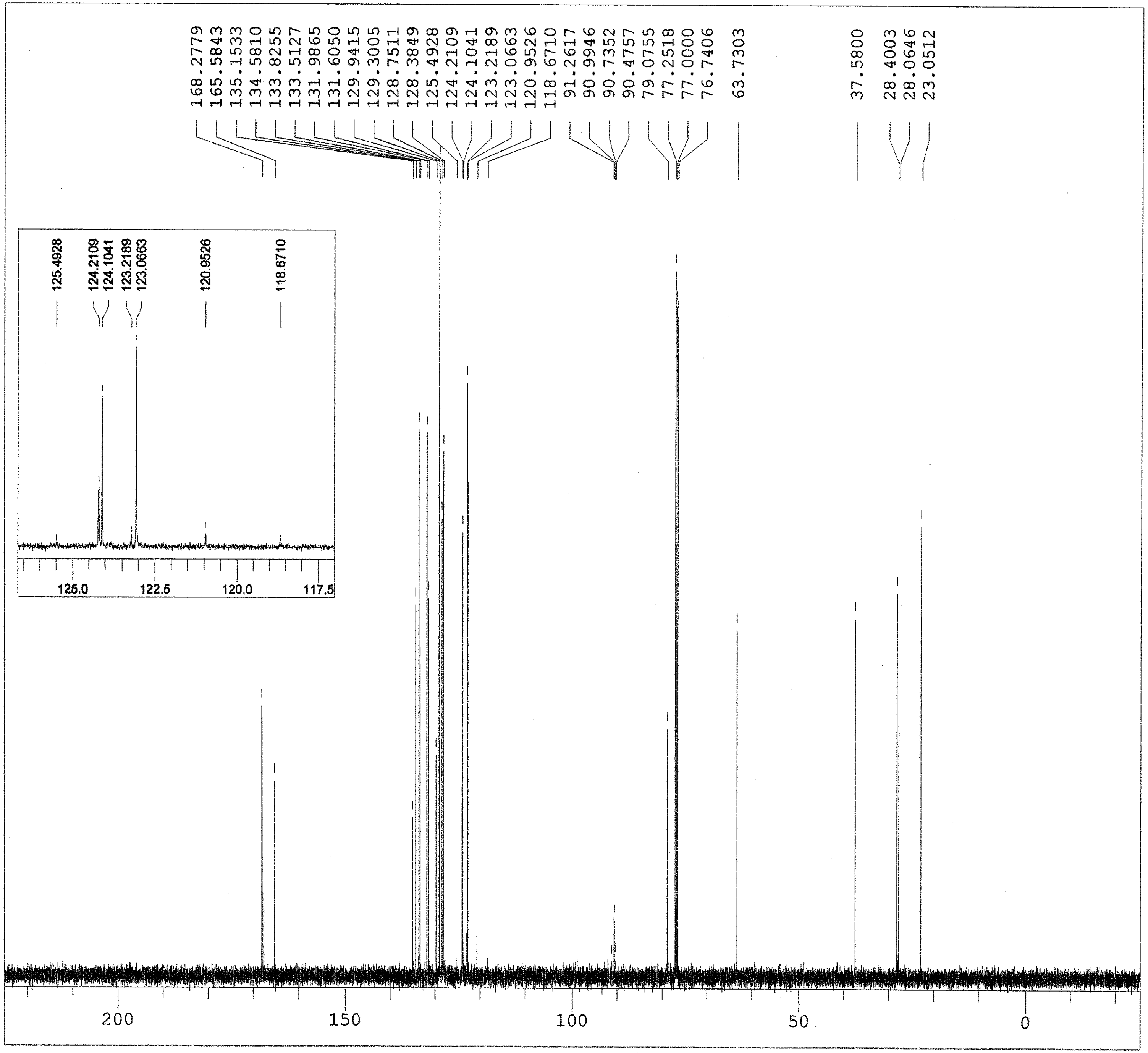

DFILE	ozawa04-142_2_13C.jdf
COMNT	C5-NPhth, Bn
DATIM	2014-03-06 19:39:56
OBNUC	13C
EXMOD	carbon.jxp
OBFRQ	125.77 MHz
OBSET	7.87 KHz
OBFIN	4.21 Hz
POINT	32768
FREQU	31446.54 Hz
SCANS	140
ACQTM	1.0420 sec
PD	3.0000 sec
PW1	3.40 usec
IRNUC	1H
CTEMP	22.3 c
SLVNT	CDCL3
EXREF	77.00 ppm
BF	0.12 Hz
RGAIN	72

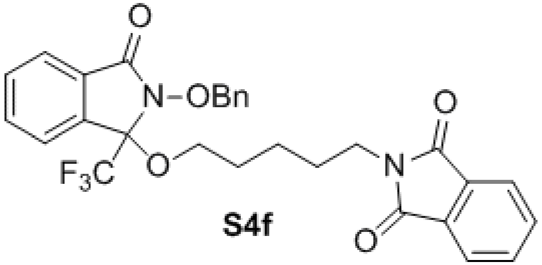

S4f

C5-NPhth, Bn

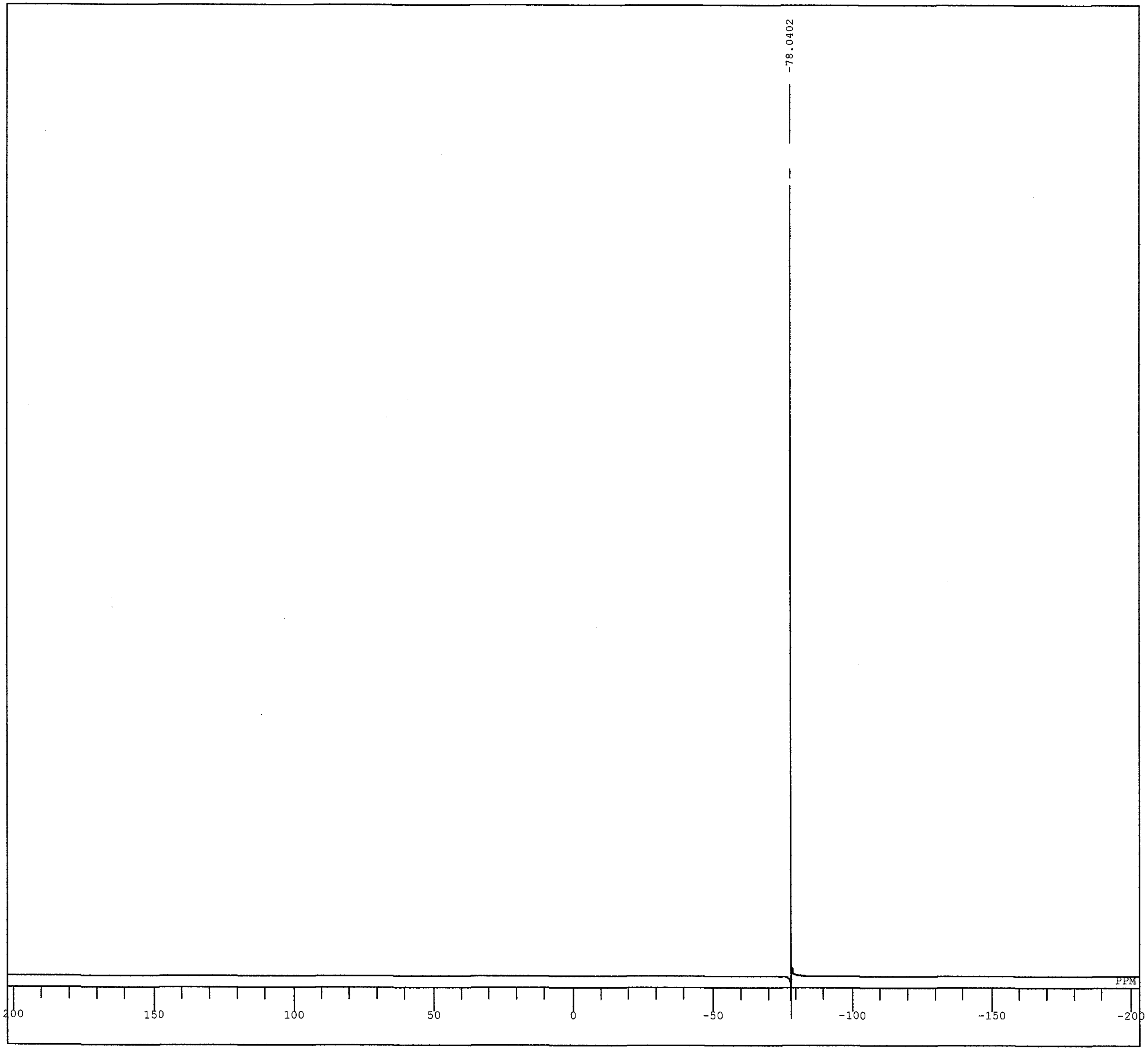

DFILE ozawa04-142_2_19F.jdf
COMNT C5-NPhth, Bn
DATIM 06-03-2014 20:41:53
OBNUC 19F
EXMOD proton.jxp
OBFRQ 368.64 MHz
OBSET 7.63 KHz
OBFIN 2.85 Hz
POINT 16384
FREQU 186567.17 Hz
SCANS 4
ACQTM 0.0878 sec
PD 5.0000 sec
PW1 3.90 usec
IRNUC 19F
CTEMP 21.2 c
SLVNT CDCL3
EXREF -164.90 ppm
BF 0.12 Hz
RGAIN 46

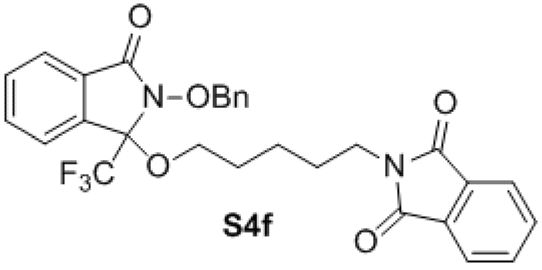

S4f

OH

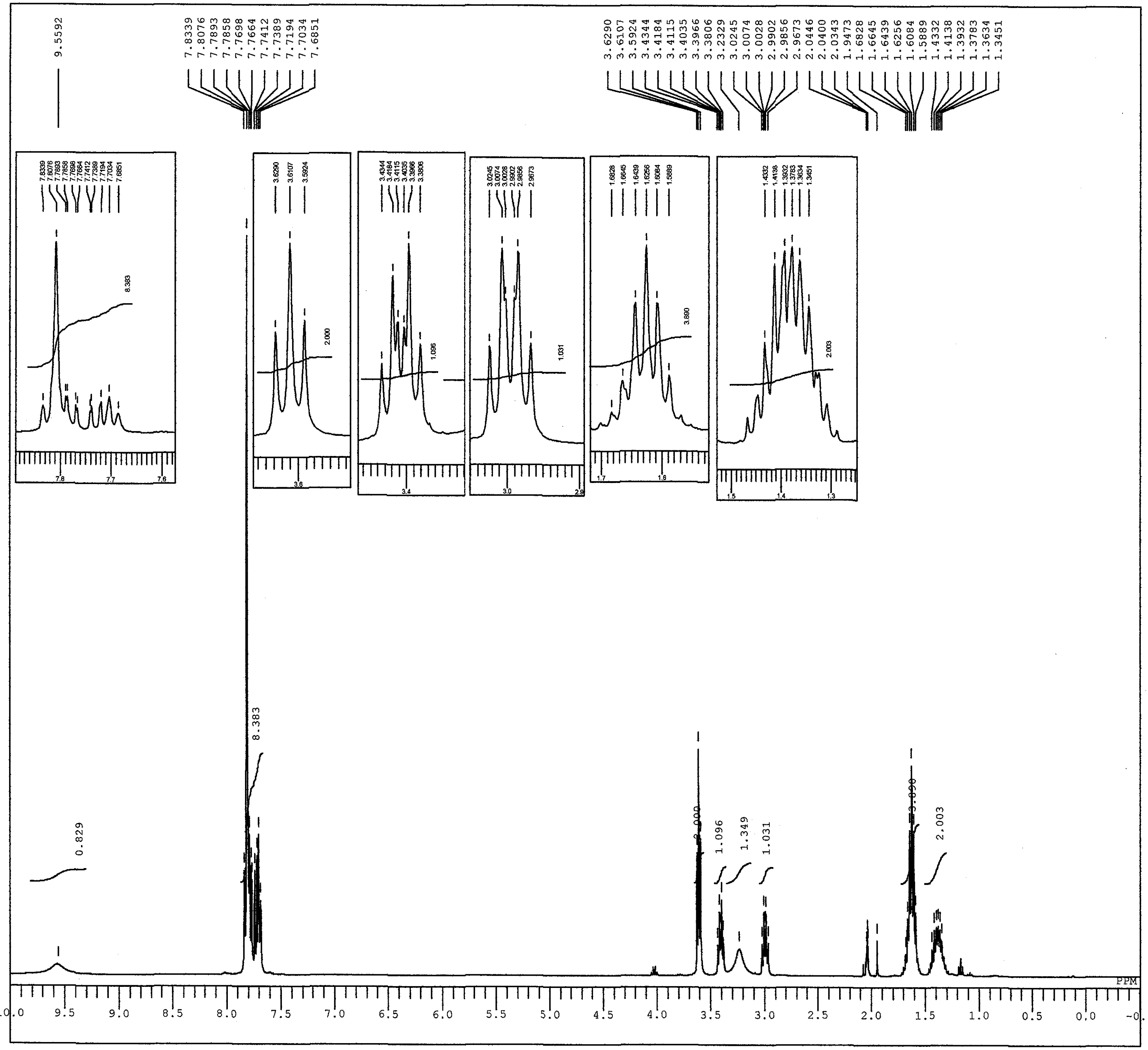

DFILE Ni-C5-NPhth_1H.jdf
COMNT OH
DATIM 03-03-2014 21:37:57
OBNUC 1H
EXMOD proton.jxp
OBFRQ 391.78 MHz
OBSET 8.51 KHz
OBFIN 3.34 Hz
POINT 16384
FREQU 7348.62 Hz
SCANS 4
ACQTM 2.2295 sec
PD 5.0000 sec
PW1 5.25 usec
IRNUC 1H
CTEMP 20.9 c
SLVNT ACETN
EXREF 2.04 ppm
BF 0.12 Hz
RGAIN 22

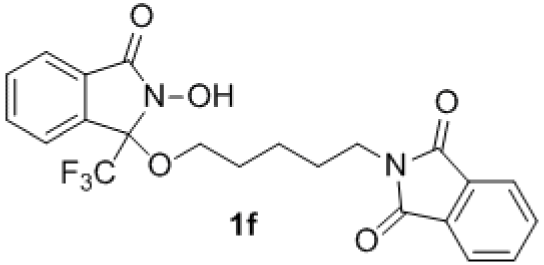

1f

OH

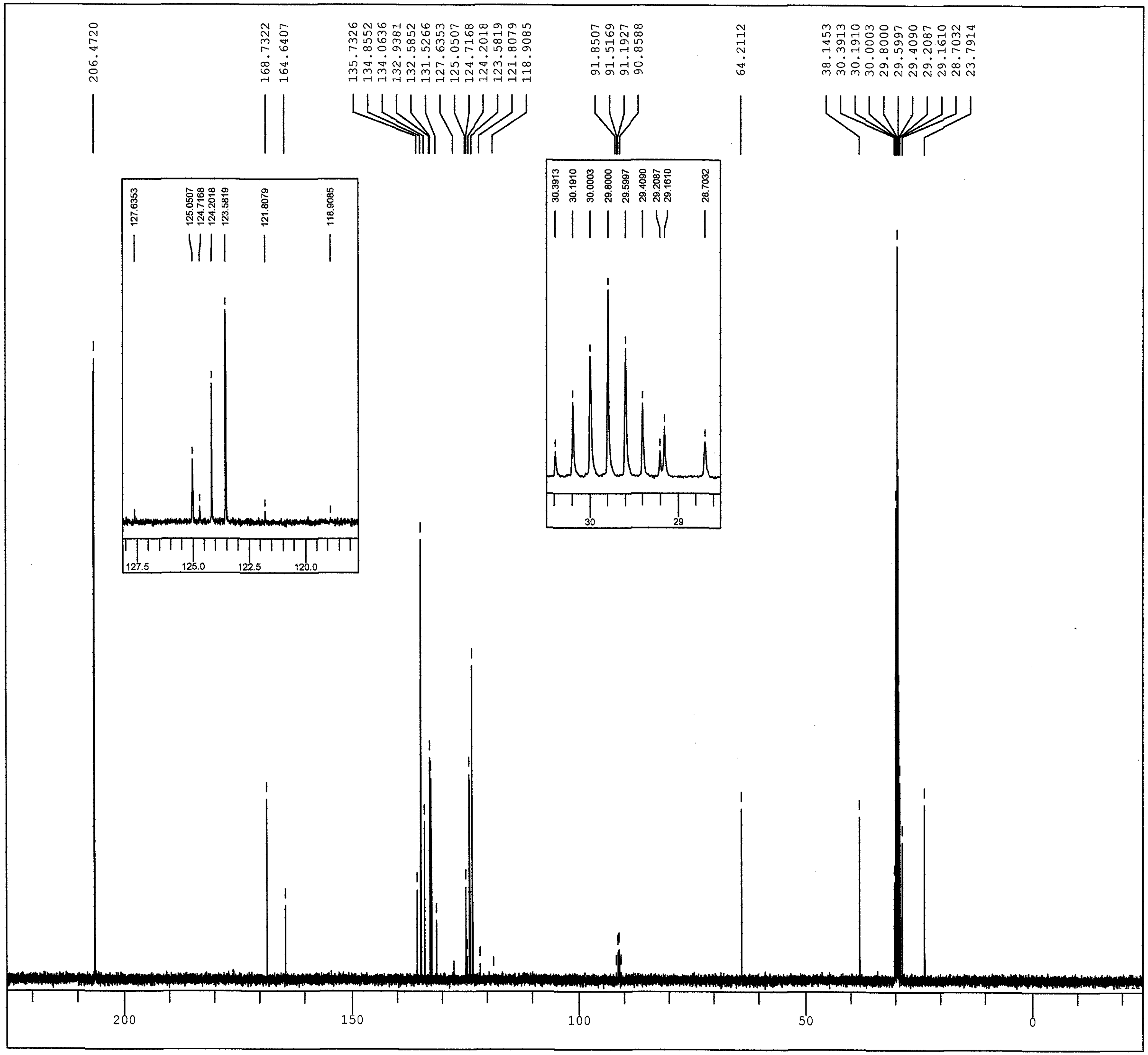

DFILE Ni_C5-NPhth_copy1.jdf
COMNT OH
DATIM 03-03-2014 21:53:04
OBNUC 13C
EXMOD carbon.jxp
OBFRQ 98.52 MHz
OBSET 4.64 KHz
OBFIN 8.74 Hz
POINT 32767
FREQU 30788.18 Hz
SCANS 100
ACQTM 0.0000 sec
PD 3.0000 sec
PW1 3.00 usec
IRNUC 1H
CTEMP 21.6 c
SLVNT ACETN
EXREF 29.80 ppm
BF 0.12 Hz
RGAIN 60

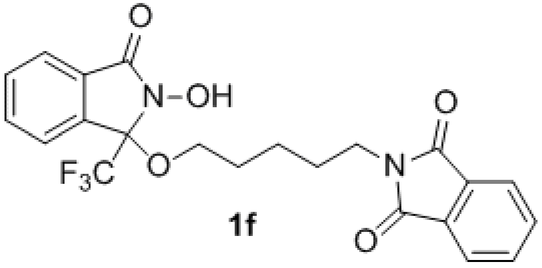

1f

OH

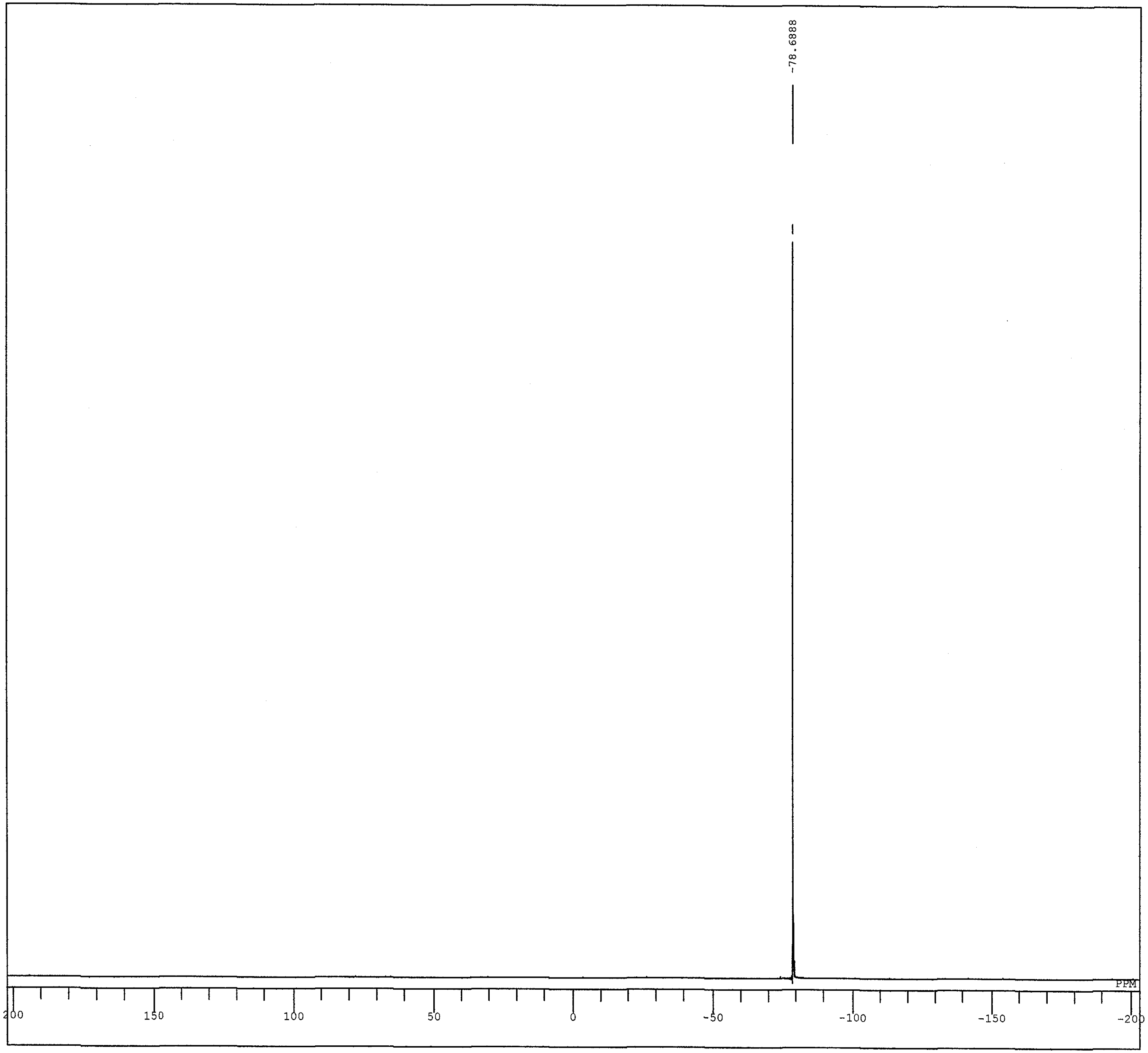

DFILE Ni-C5-NPhth_19F.jdf
COMNT OH
DATIM 03-03-2014 21:35:32
OBNUC 19F
EXMOD proton.jxp
OBFRQ 368.64 MHz
OBSET 7.63 KHz
OBFIN 2.85 Hz
POINT 16384
FREQU 186567.17 Hz
SCANS 4
ACQTM 0.0878 sec
PD 5.0000 sec
PW1 3.90 usec
IRNUC 19F
CTEMP 21.0 c
SLVNT ACETN
EXREF -164.90 ppm
BF 0.12 Hz
RGAIN 44

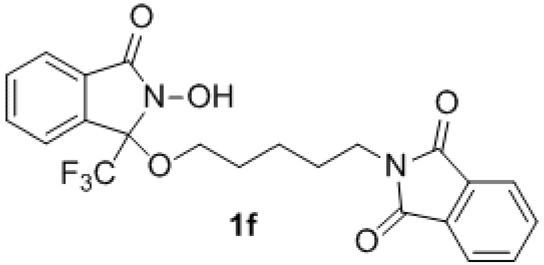

1f

C5-NPhth, [O]

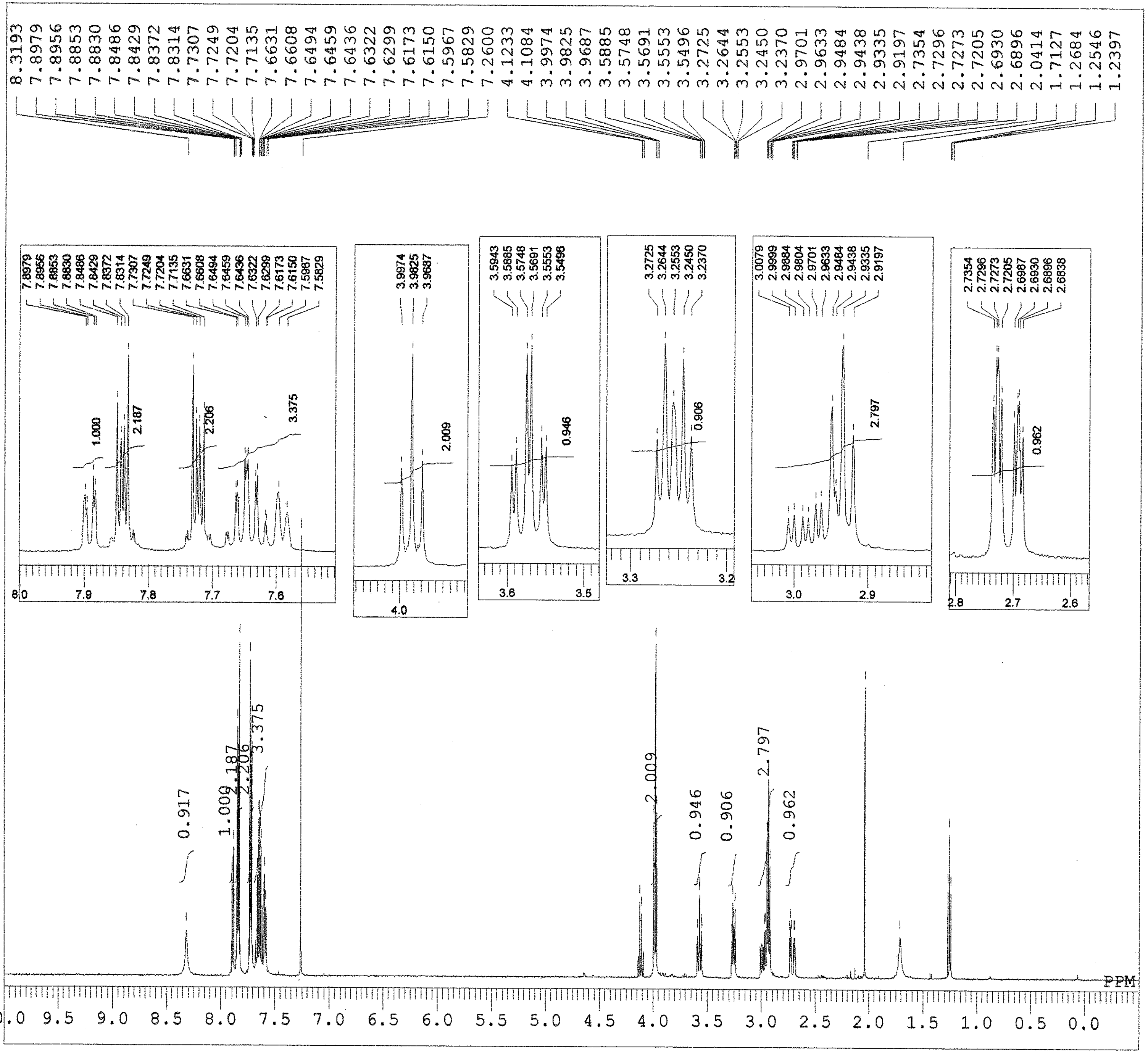

DFILE	ozawa04-141_1_1H.jdf
COMNT	C5-NPhth, [O]
DATIM	2014-03-12 19:02:30
OBNUC	1H
EXMOD	proton.jxp
OBFRQ	500.16 MHz
OBSET	2.41 KHz
OBFIN	6.01 Hz
POINT	16384
FREQU	9384.38 Hz
SCANS	4
ACQTM	1.7459 sec
PD	5.0000 sec
PW1	5.55 usec
IRNUC	1H
CTEMP	20.9 c
SLVNT	CDCL3
EXREF	7.26 ppm
BF	0.12 Hz
RGAIN	40

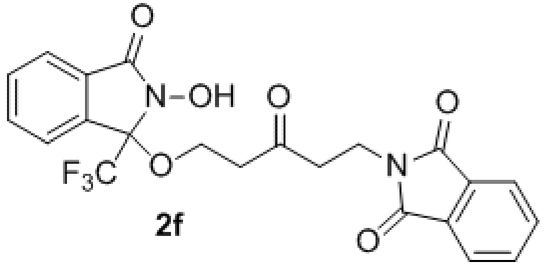

2f

C5-NPhth

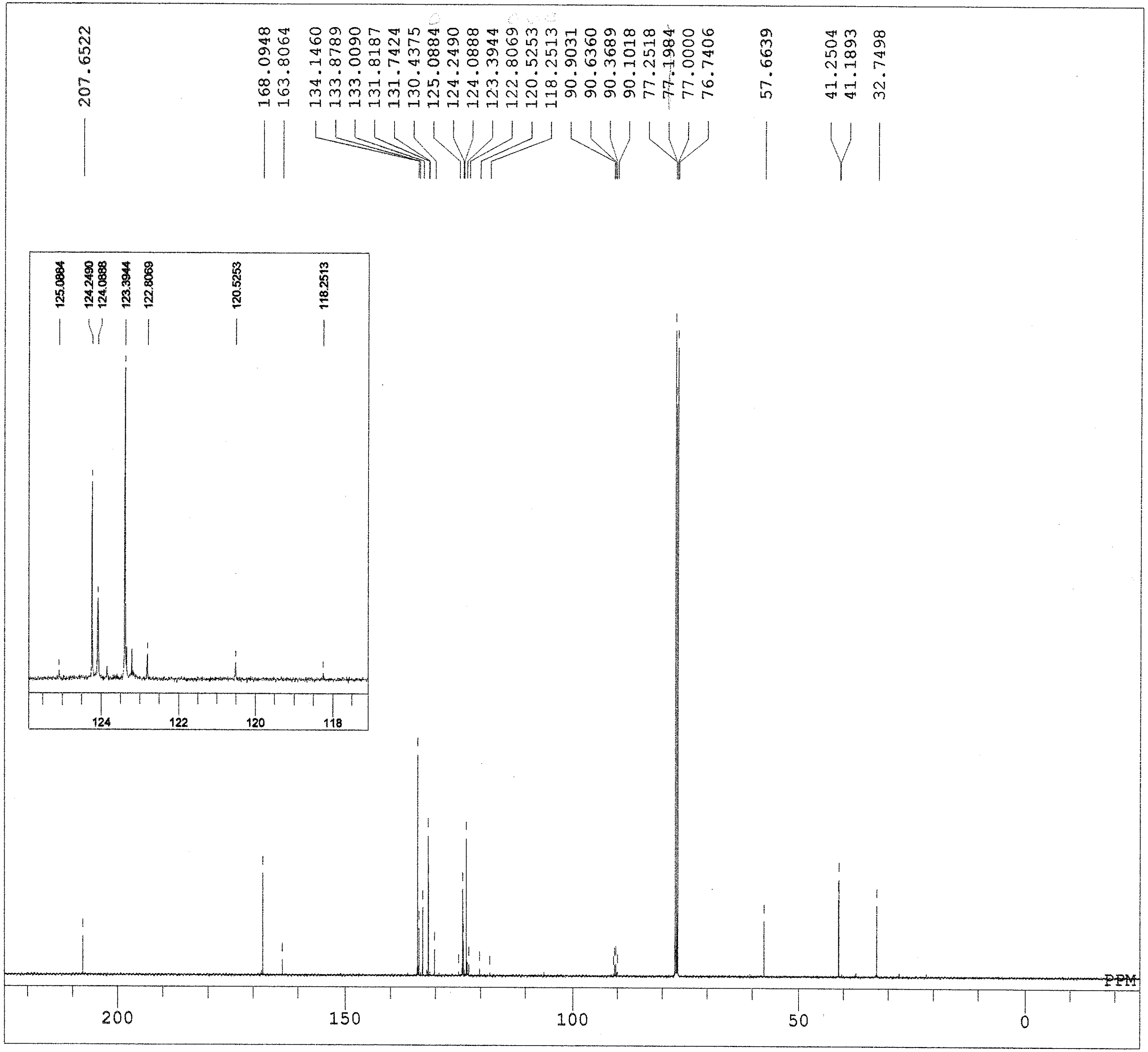

DFILE ozawa04-141_1_13C.jdf
COMNT C5-NPhth
DATIM 2014-03-11 22:54:25
OBNUC 13C
EXMOD carbon.jxp
OBFRQ 125.77 MHz
OBSET 7.87 KHz
OBFIN 4.21 Hz
POINT 32768
FREQU 31446.54 Hz
SCANS 12284
ACQTM 1.0420 sec
PD 3.0000 sec
PW1 3.40 usec
IRNUC 1H
CTEMP 21.7 c
SLVNT CDCL3
EXREF 77.00 ppm
BF 0.12 Hz
RGAIN 72

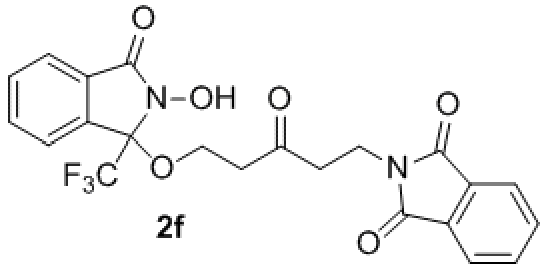

2f

C5-NPhth, [0]

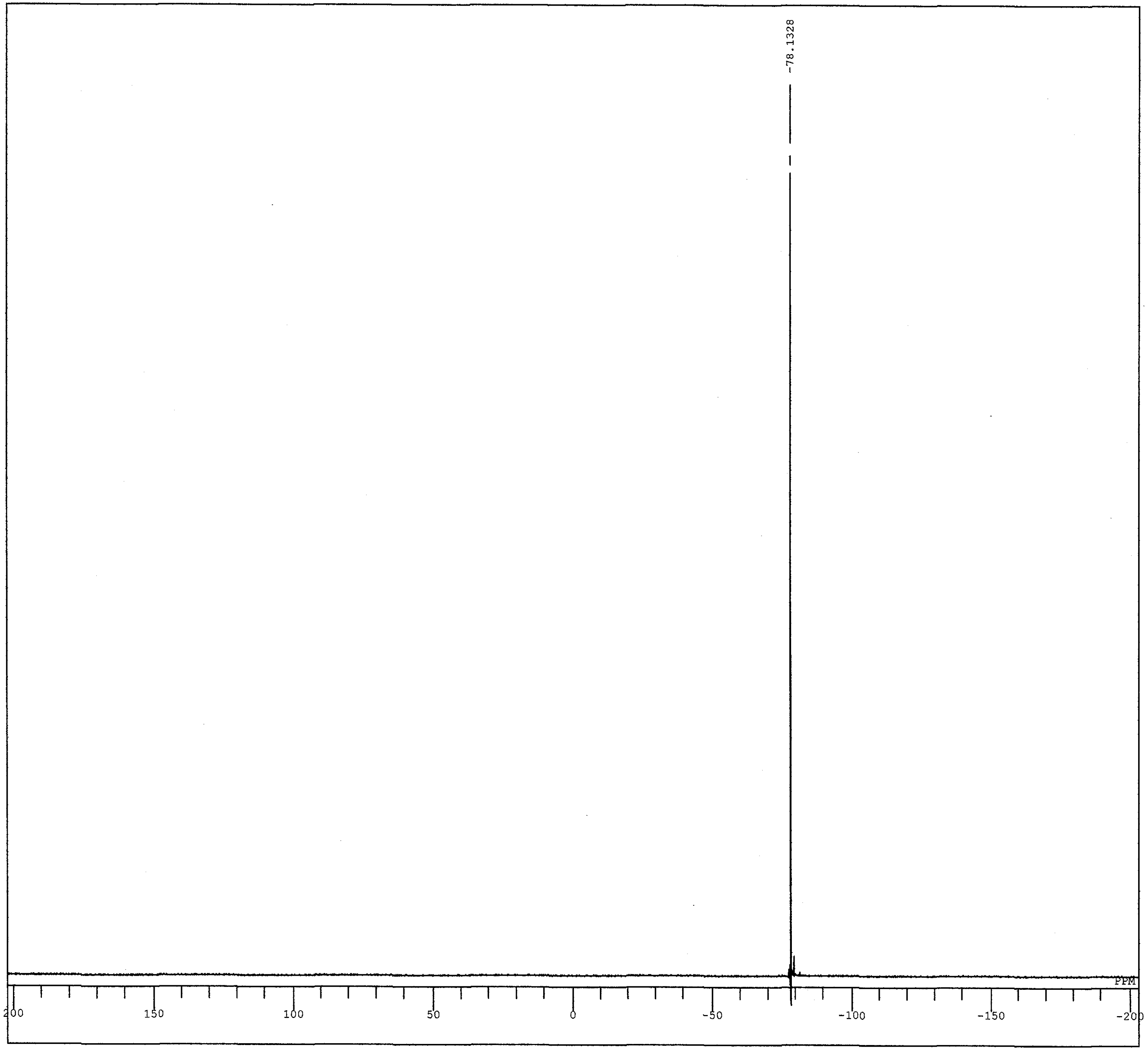

DFILE ozawa04-141_1_19F.jdf
COMNT C5-NPhth, [0]
DATIM 06-03-2014 20:50:21
OBNUC 19F
EXMOD proton.jxp
OBFRQ 368.64 MHz
OBSET 7.63 KHz
OBFIN 2.85 Hz
POINT 16384
FREQU 186567.17 Hz
SCANS 4
ACQTM 0.0878 sec
PD 5.0000 sec
PW1 3.90 usec
IRNUC 19F
CTEMP 21.3 c
SLVNT CDCL3
EXREF -164.90 ppm
BF 0.12 Hz
RGAIN 50

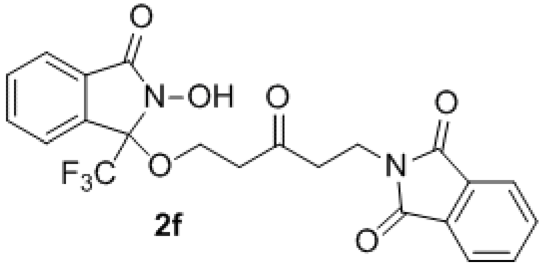

2f

C5-CO2Me, Bn

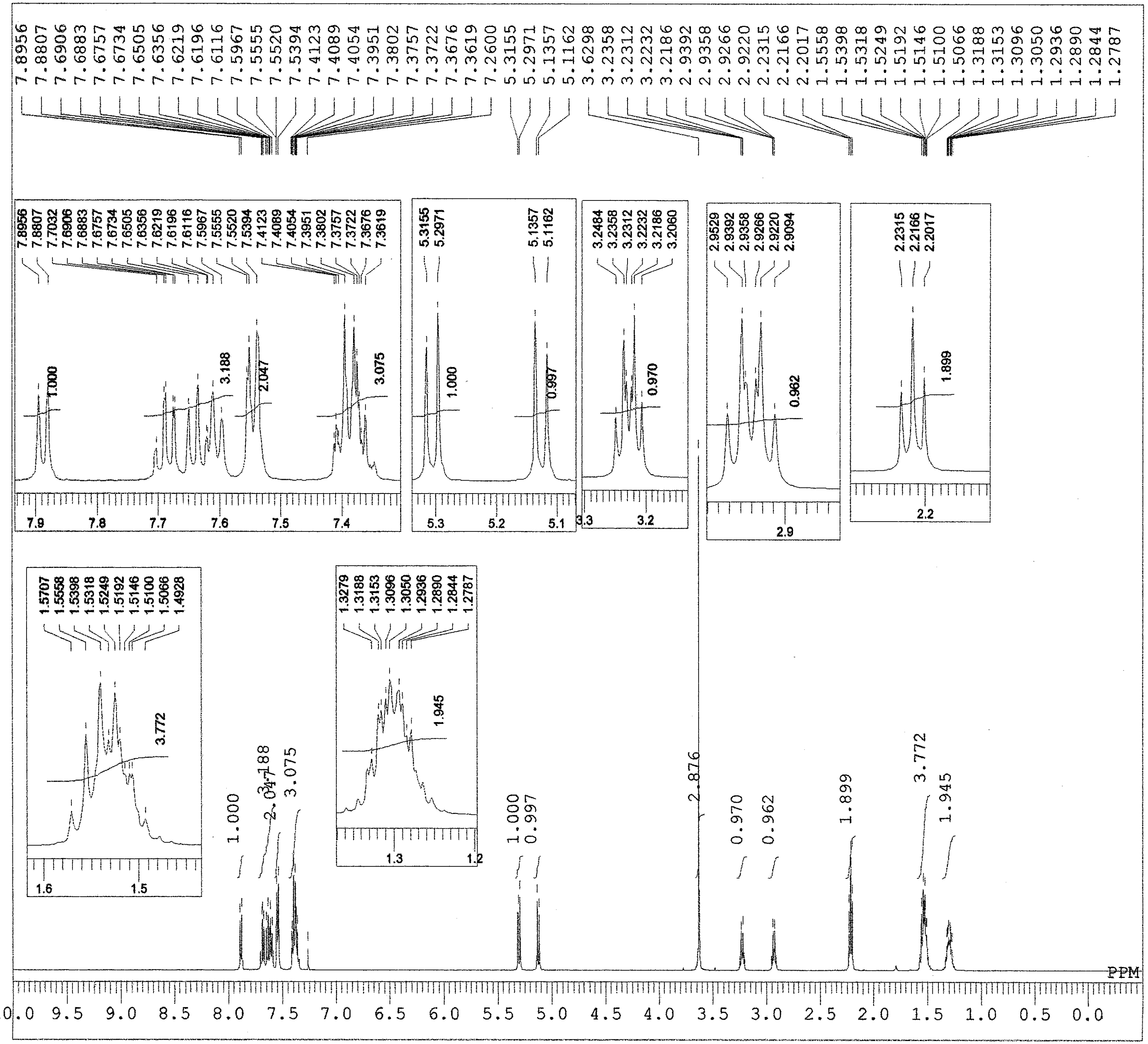

DFILE	ozawa04-142_3_1H.jdf
COMNT	C5-CO2Me, Bn
DATIM	2014-03-06 19:51:58
OBNUC	1H
EXMOD	proton.jxp
OBFRQ	500.16 MHz
OBSET	2.41 KHz
OBFIN	6.01 Hz
POINT	16384
FREQU	9384.38 Hz
SCANS	4
ACQTM	1.7459 sec
PD	5.0000 sec
PW1	5.55 usec
IRNUC	1H
CTEMP	21.9 c
SLVNT	CDCL3
EXREF	7.26 ppm
BF	0.12 Hz
RGAIN	30

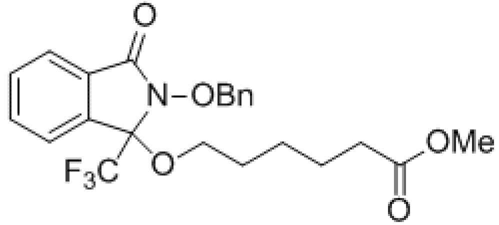

S4g

C5-CO2Me, Bn

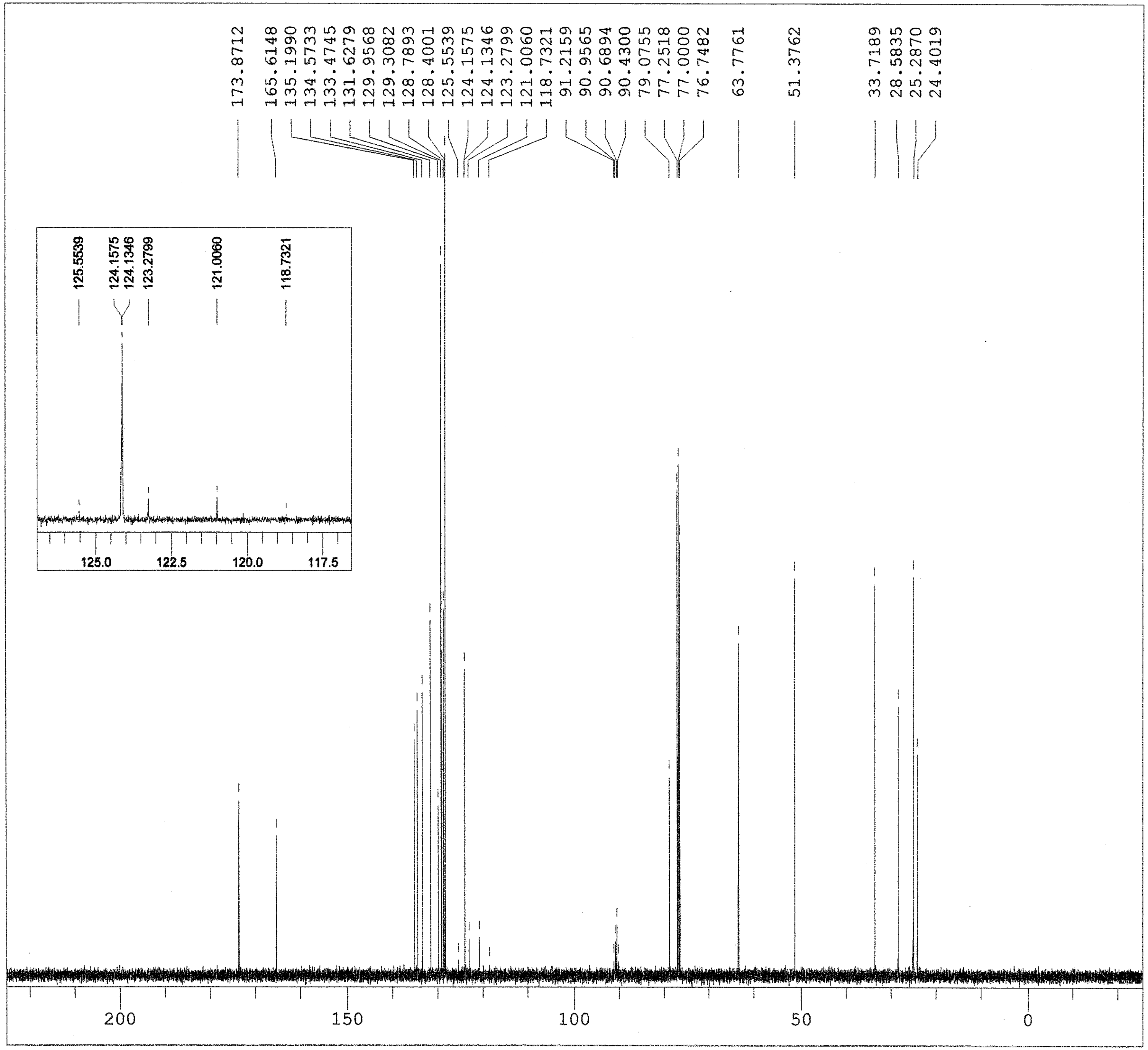

DFILE ozawa04-142_3_13C.jdf
COMNT C5-CO2Me, Bn
DATIM 2014-03-06 19:53:09
OBNUC 13C
EXMOD carbon.jxp
OBFRQ 125.77 MHz
OBSET 7.87 KHz
OBFIN 4.21 Hz
POINT 32768
FREQU 31446.54 Hz
SCANS 144
ACQTM 1.0420 sec
PD 3.0000 sec
PW1 3.40 usec
IRNUC 1H
CTEMP 21.9 c
SLVNT CDCL3
EXREF 77.00 ppm
BF 0.12 Hz
RGAIN 72

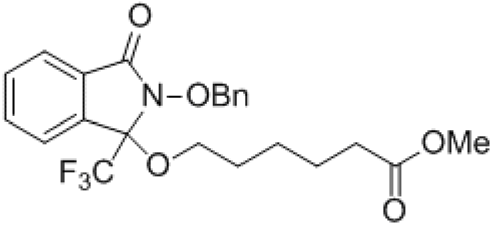

S4g

C5CO2Me, Bn

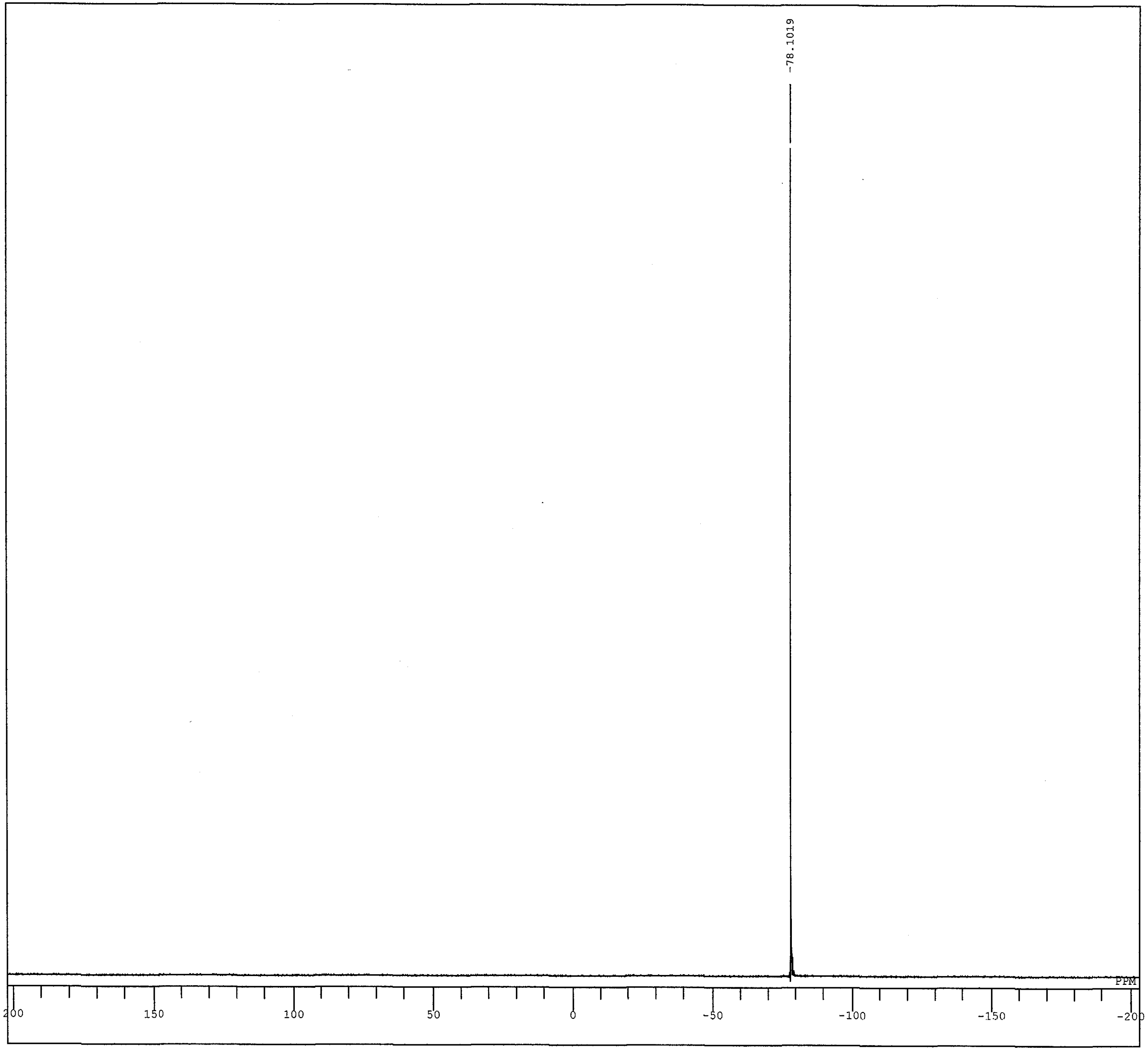


```
DFILE  ozawa04-142_3_19F.jdf
COMNT  C5CO2Me, Bn
DATIM  06-03-2014 20:44:46
OBNUC  19F
EXMOD  proton.jxp
OBFRQ        368.64 MHz
OBSET          7.63 KHz
OBFIN          2.85 Hz
POINT         16384
FREQU     186567.17 Hz
SCANS             4
ACQTM        0.0878 sec
PD           5.0000 sec
PW1            3.90 usec
IRNUC  19F
CTEMP          21.0 c
SLVNT  CDCL3
EXREF       -164.90 ppm
BF             0.12 Hz
RGAIN            44
```

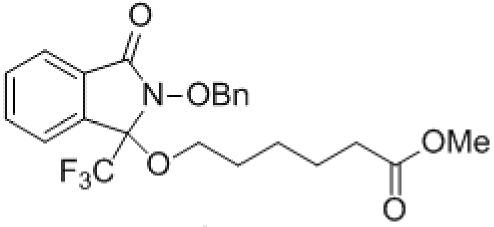

S4g

OH

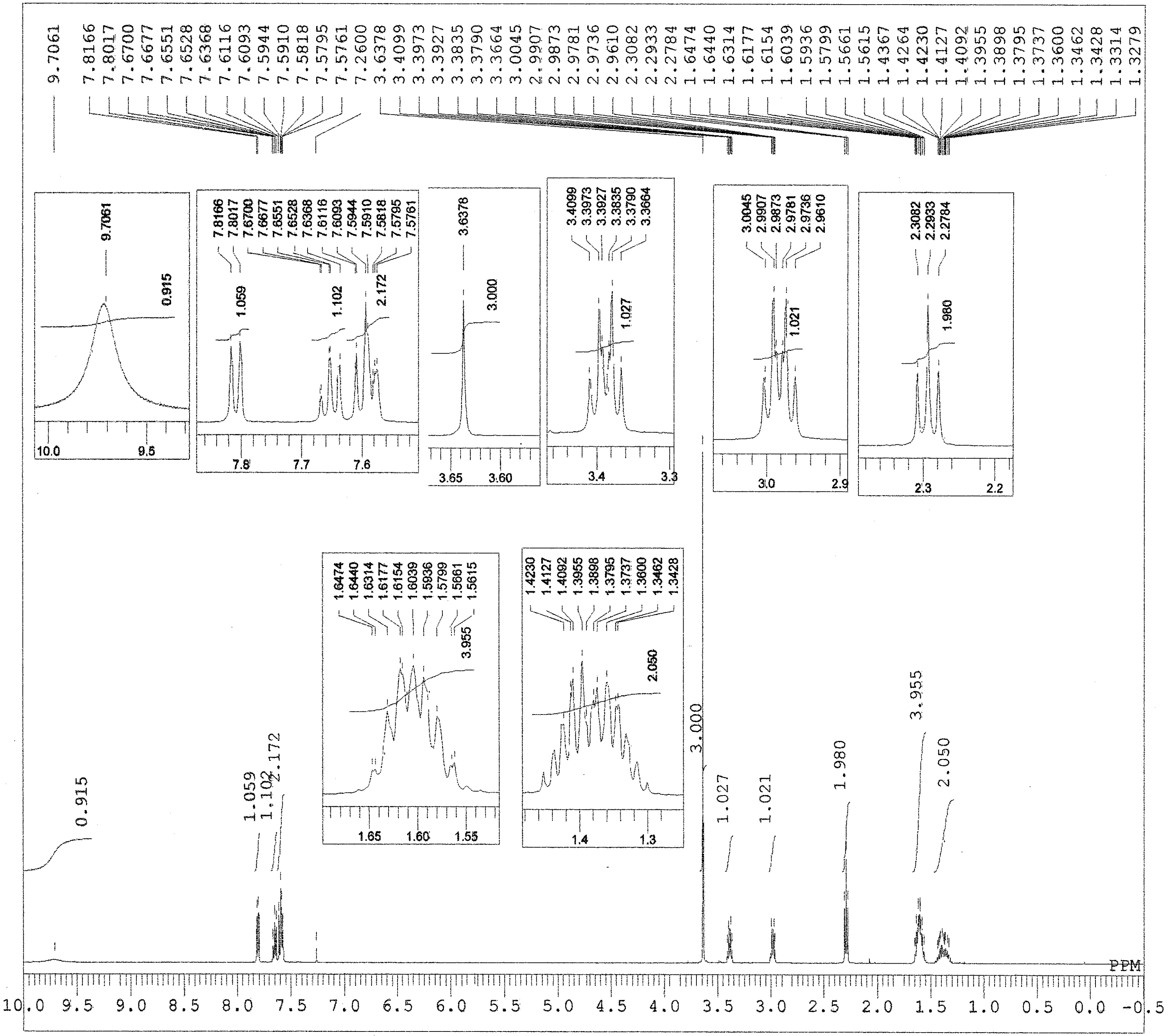

DFILE Ni_C5-CO2Me_1H.jdf
COMNT OH
DATIM 2014-03-03 20:26:24
OBNUC 1H
EXMOD proton.jxp
OBFRQ 500.16 MHz
OBSET 2.41 KHz
OBFIN 6.01 Hz
POINT 16384
FREQU 9384.38 Hz
SCANS 4
ACQTM 1.7459 sec
PD 5.0000 sec
PW1 5.55 usec
IRNUC 1H
CTEMP 21.1 c
SLVNT CDCL3
EXREF 7.26 ppm
BF 0.12 Hz
RGAIN 28

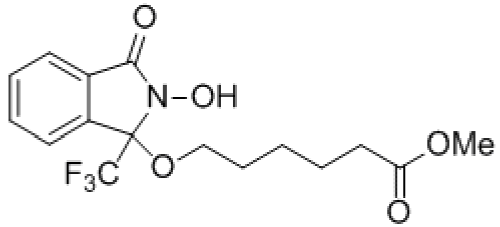

1g

OH

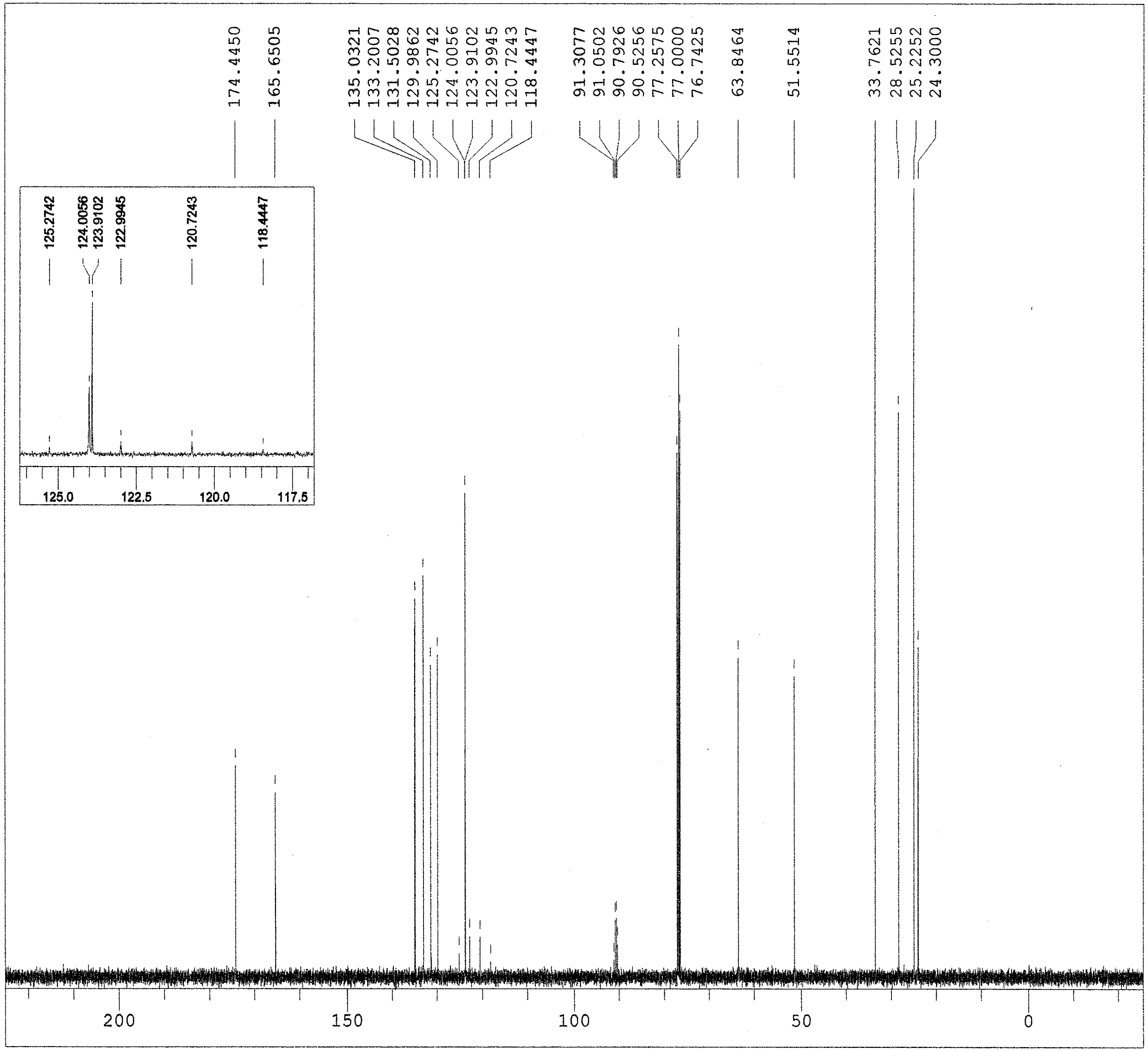

DFILE Ni_C5-CO2Me_13C.als
COMNT OH
DATIM 2014-03-03 20:27:27
OBNUC 13C
EXMOD carbon.jxp
OBFRQ 125.77 MHz
OBSET 7.87 KHz
OBFIN 4.21 Hz
POINT 26214
FREQU 31446.54 Hz
SCANS 124
ACQTM 0.8336 sec
PD 3.0000 sec
PW1 3.40 usec
IRNUC 1H
CTEMP 21.3 c
SLVNT CDCL3
EXREF 77.00 ppm
BF 0.12 Hz
RGAIN 60

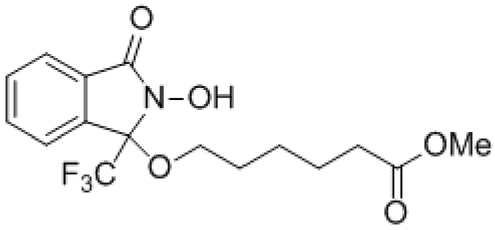

1g

OH

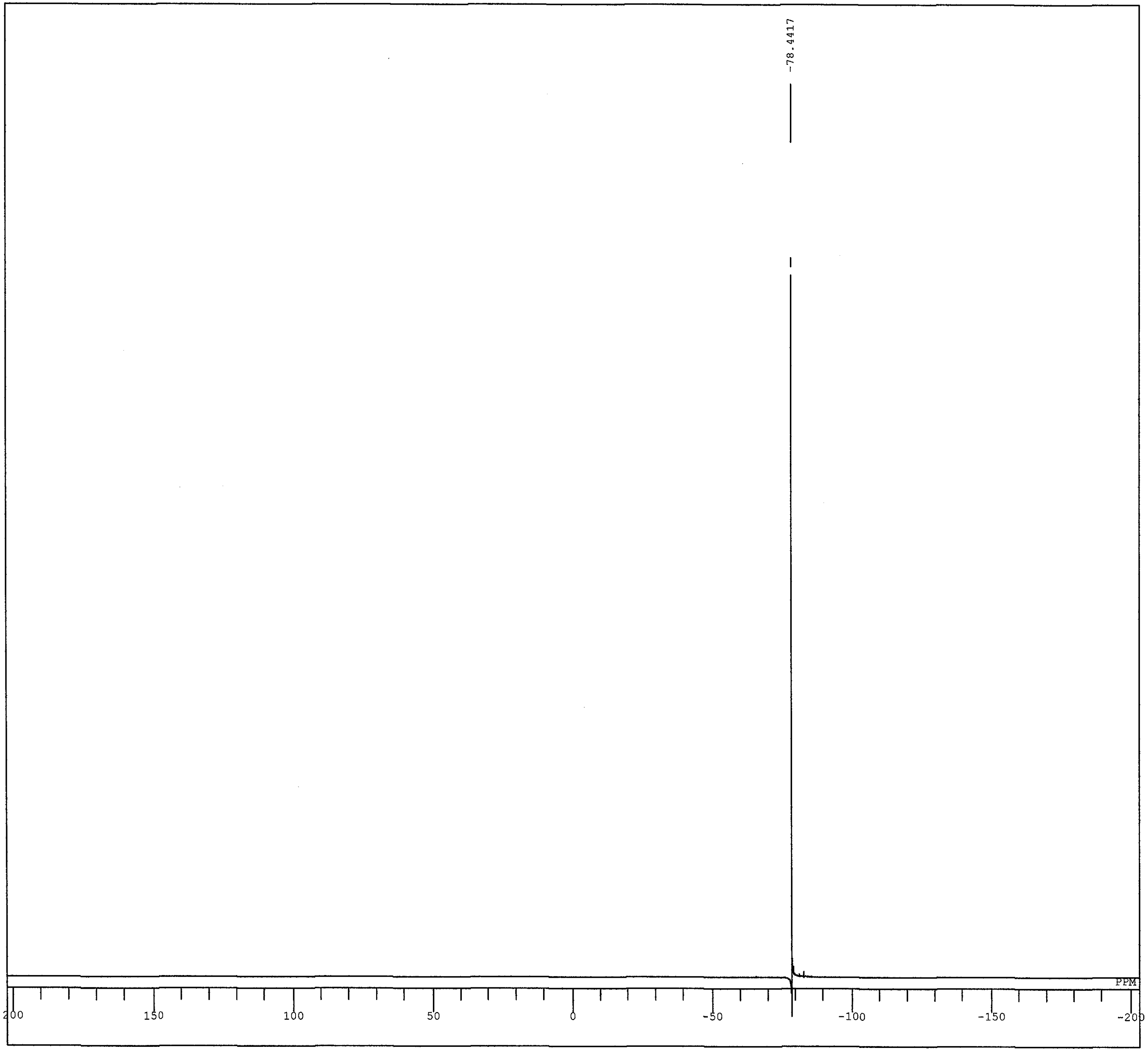

DFILE Ni_C5-CO2Me_19F.jdf
COMNT OH
DATIM 03-03-2014 21:00:27
OBNUC 19F
EXMOD proton.jxp
OBFRQ 368.64 MHz
OBSET 7.63 KHz
OBFIN 2.85 Hz
POINT 16384
FREQU 186567.17 Hz
SCANS 4
ACQTM 0.0878 sec
PD 5.0000 sec
PW1 3.90 usec
IRNUC 19F
CTEMP 20.8 c
SLVNT CDCL3
EXREF -164.90 ppm
BF 0.12 Hz
RGAIN 44

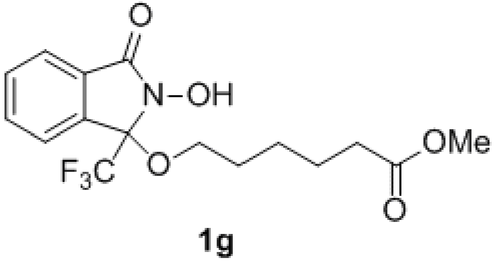

1g

C5-CO2Me, [0]

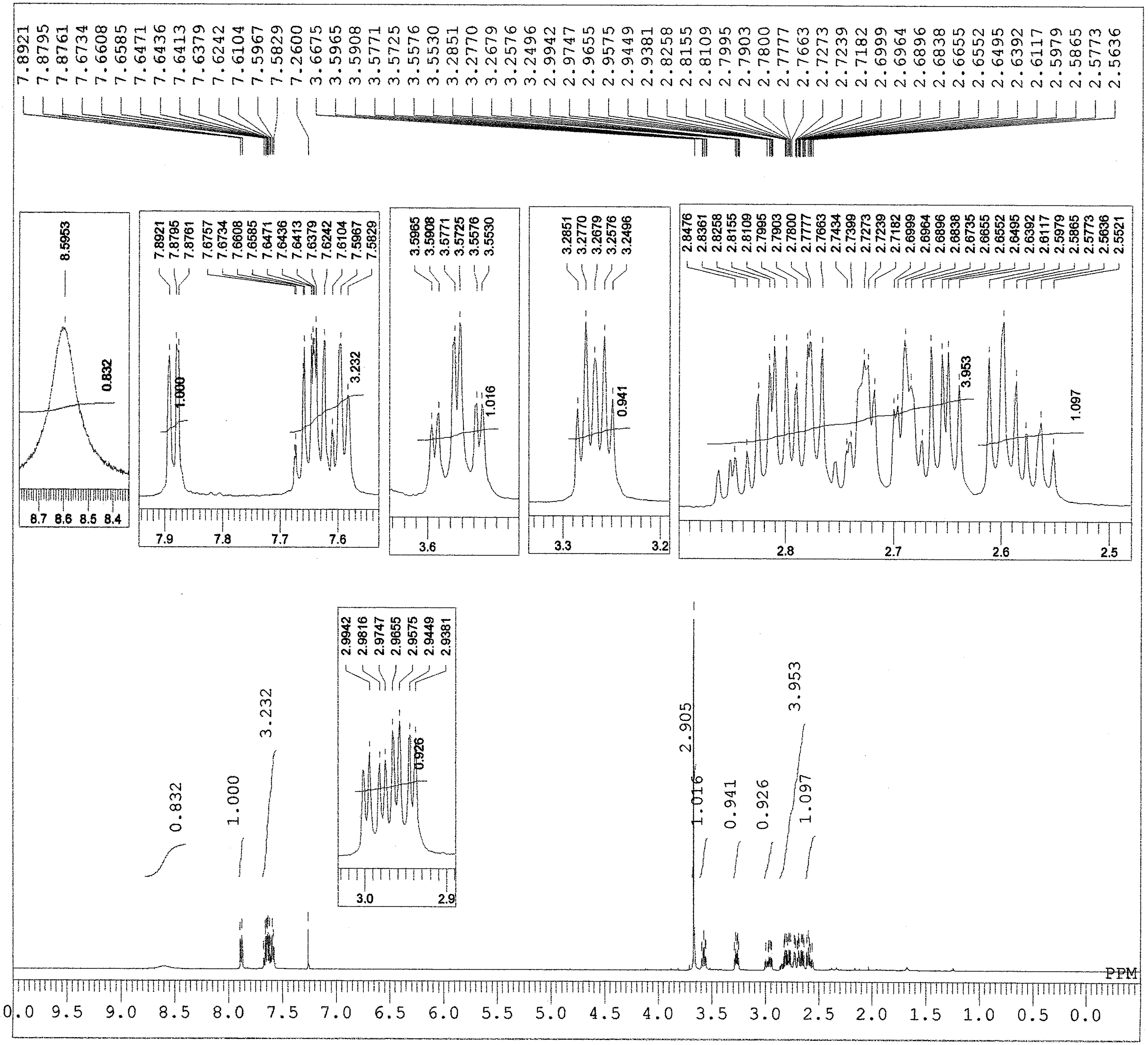

DFILE	ozawa04-141_2_1H.jdf
COMNT	C5-CO2Me, [O]
DATIM	2014-03-06 20:05:37
OBNUC	1H
EXMOD	proton.jxp
OBFRQ	500.16 MHz
OBSET	2.41 KHz
OBFIN	6.01 Hz
POINT	16384
FREQU	9384.38 Hz
SCANS	4
ACQTM	1.7459 sec
PD	5.0000 sec
PW1	5.55 usec
IRNUC	1H
CTEMP	21.3 c
SLVNT	CDCL3
EXREF	7.26 ppm
BF	0.12 Hz
RGAIN	36

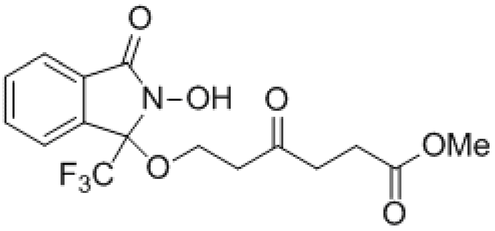

2g

C5-CO2Me, [O]

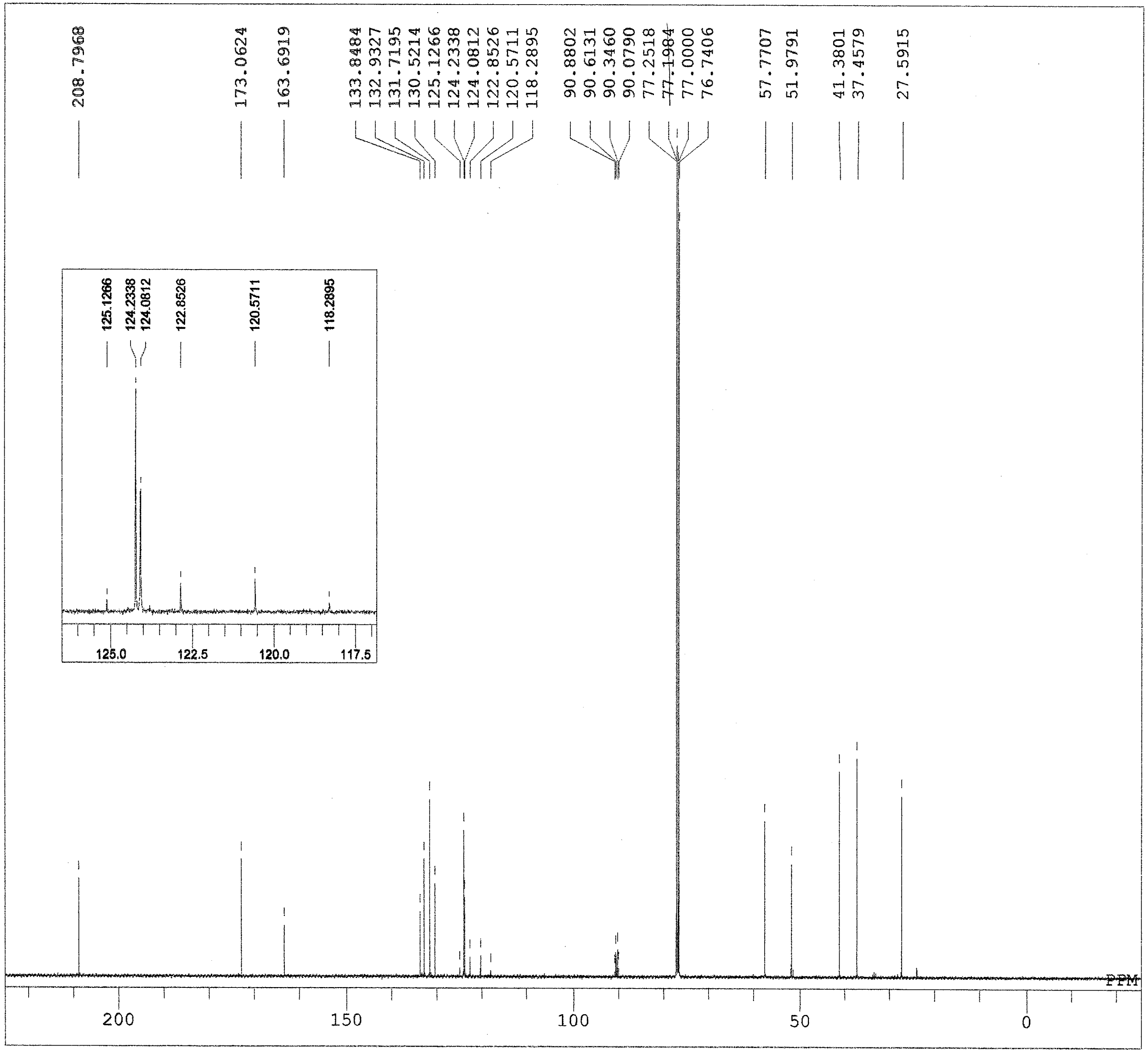

DFILE ozawa04-141_2_13C.jdf
COMNT C5-CO2Me, [O]
DATIM 2014-03-06 22:57:01
OBNUC 13C
EXMOD carbon.jxp
OBFRQ 125.77 MHz
OBSET 7.87 KHz
OBFIN 4.21 Hz
POINT 32768
FREQU 31446.54 Hz
SCANS 9052
ACQTM 1.0420 sec
PD 3.0000 sec
PW1 3.40 usec
IRNUC 1H
CTEMP 21.9 c
SLVNT CDCL3
EXREF 77.00 ppm
BF 0.12 Hz
RGAIN 74

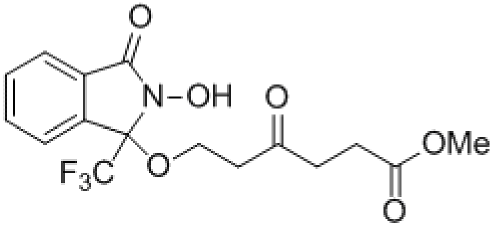

2g

C5-CO2Me, [O]

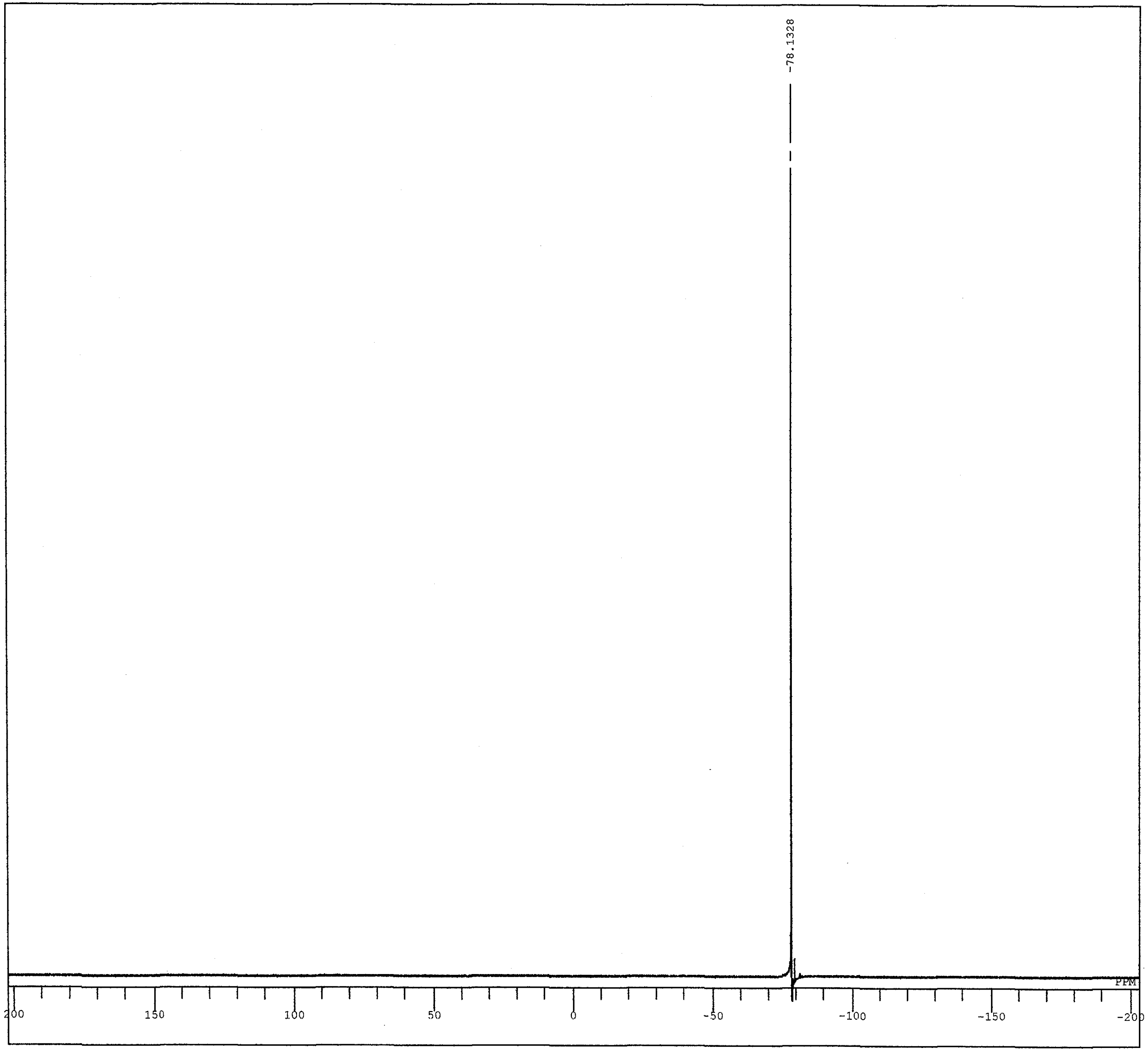

DFILE ozawa04-141_2_19F.jdf
COMNT C5-CO2Me, [O]
DATIM 06-03-2014 20:47:43
OBNUC 19F
EXMOD proton.jxp
OBFRQ 368.64 MHz
OBSET 7.63 KHz
OBFIN 2.85 Hz
POINT 16384
FREQU 186567.17 Hz
SCANS 4
ACQTM 0.0878 sec
PD 5.0000 sec
PW1 3.90 usec
IRNUC 19F
CTEMP 21.2 c
SLVNT CDCL3
EXREF -164.90 ppm
BF 0.12 Hz
RGAIN 48

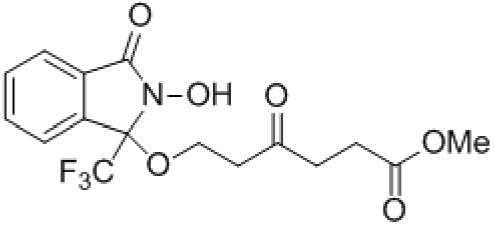

2g

3-MeBuOH

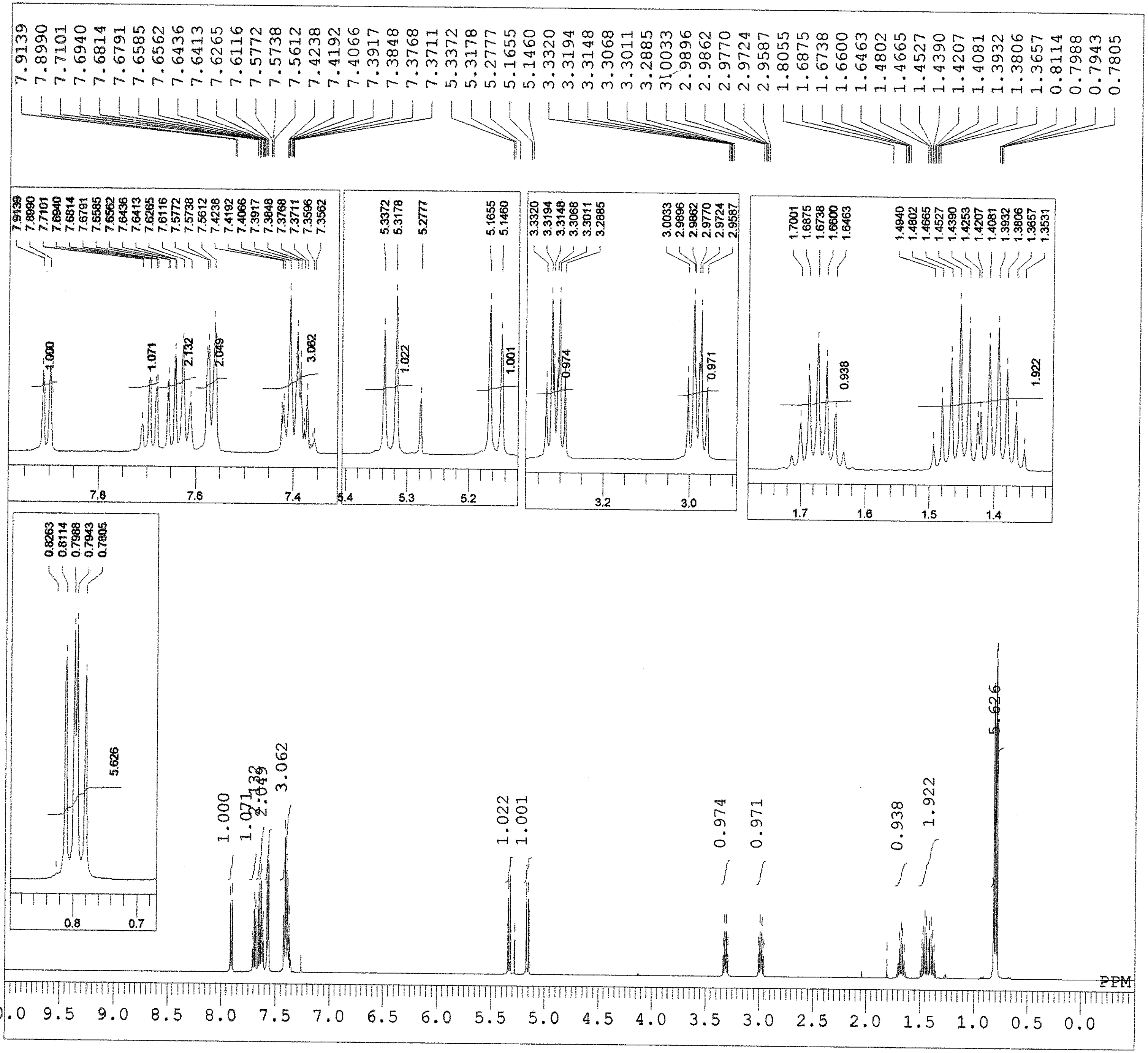

DFILE	ozawa04-053_1_1H.jdf
COMNT	3-MeBuOH
DATIM	2013-11-25 14:22:41
OBNUC	1H
EXMOD	proton.jxp
OBFRQ	500.16 MHz
OBSET	2.41 KHz
OBFIN	6.01 Hz
POINT	16384
FREQU	9384.38 Hz
SCANS	4
ACQTM	1.7459 sec
PD	5.0000 sec
PW1	5.55 usec
IRNUC	1H
CTEMP	22.7 c
SLVNT	CDCL3
EXREF	7.26 ppm
BF	0.12 Hz
RGAIN	26

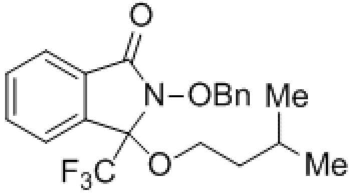

S4h

3-MeBuOH

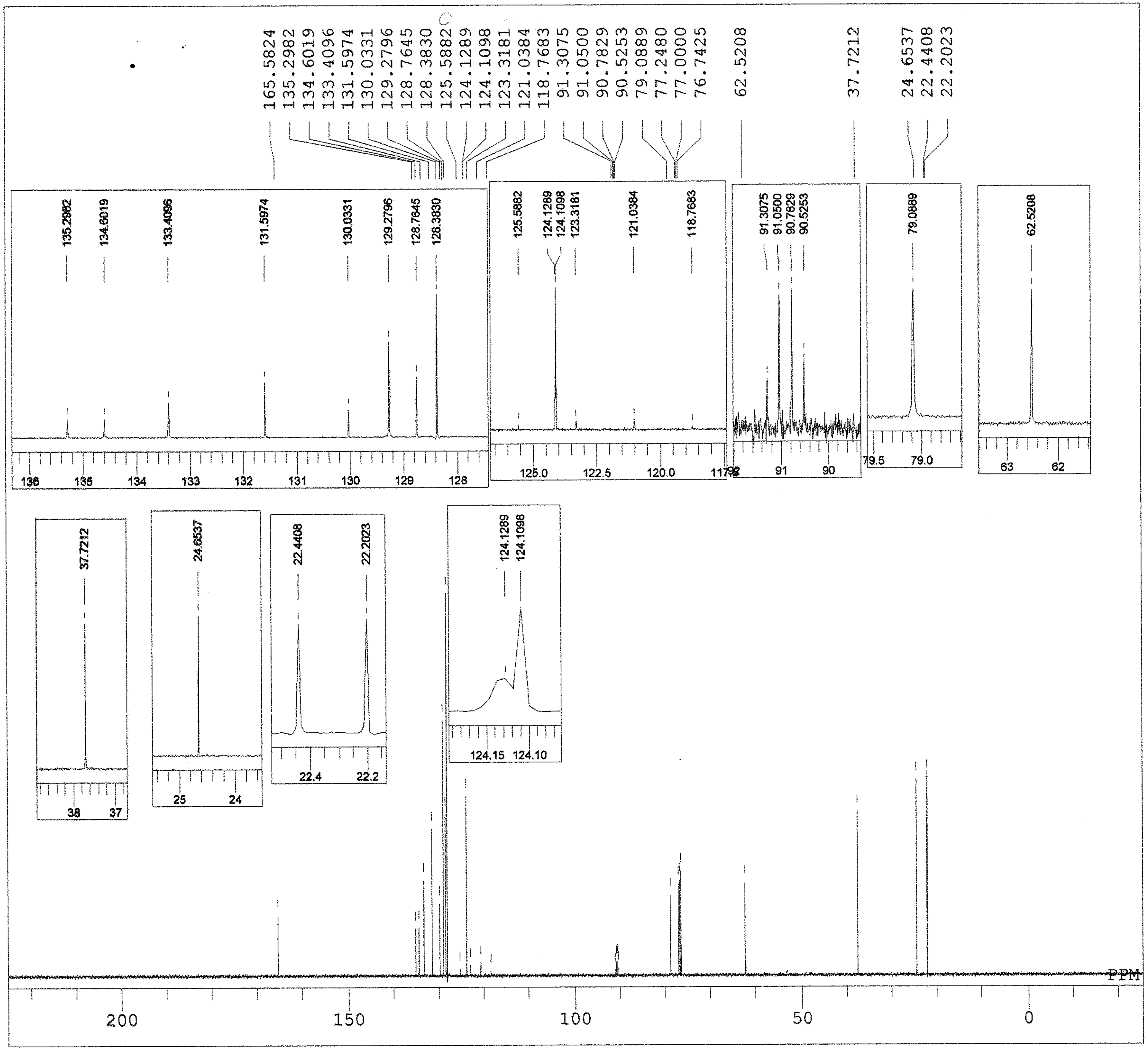

DFILE ozawa04-053_1_13C.jdf
COMNT 3-MeBuOH
DATIM 2013-11-25 17:29:50
OBNUC 13C
EXMOD carbon.jxp
OBFRQ 125.77 MHz
OBSET 7.87 KHz
OBFIN 4.21 Hz
POINT 32767
FREQU 39308.18 Hz
SCANS 140
ACQTM 0.8336 sec
PD 3.0000 sec
PW1 3.40 usec
IRNUC 1H
CTEMP 23.5 c
SLVNT CDCL3
EXREF 77.00 ppm
BF 0.12 Hz
RGAIN 60

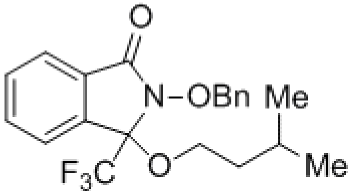

S4h

3-MeBuOH

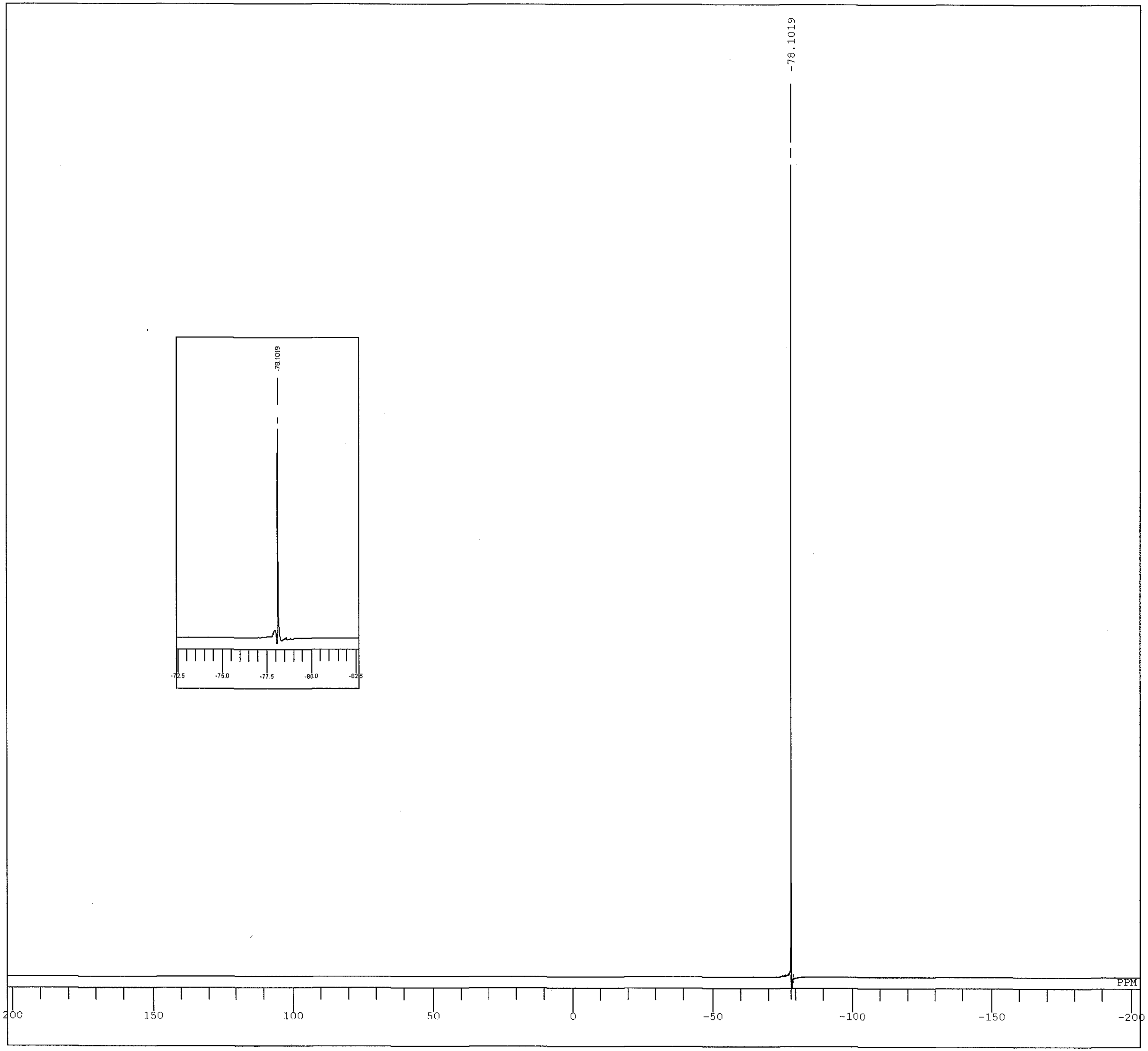

DFILE ozawa04-053_1_19F.jdf
COMNT 3-MeBuOH
DATIM 25-11-2013 13:52:14
OBNUC 19F
EXMOD proton.jxp
OBFRQ 368.64 MHz
OBSET 7.63 KHz
OBFIN 2.85 Hz
POINT 16384
FREQU 186567.17 Hz
SCANS 4
ACQTM 0.0878 sec
PD 5.0000 sec
PW1 3.90 usec
IRNUC 19F
CTEMP 21.5 c
SLVNT CDCL3
EXREF -164.90 ppm
BF 0.12 Hz
RGAIN 44

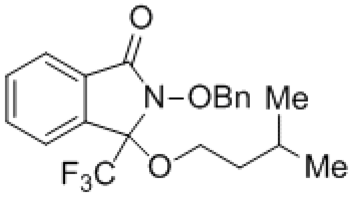

S4h

MeBuOH, OH

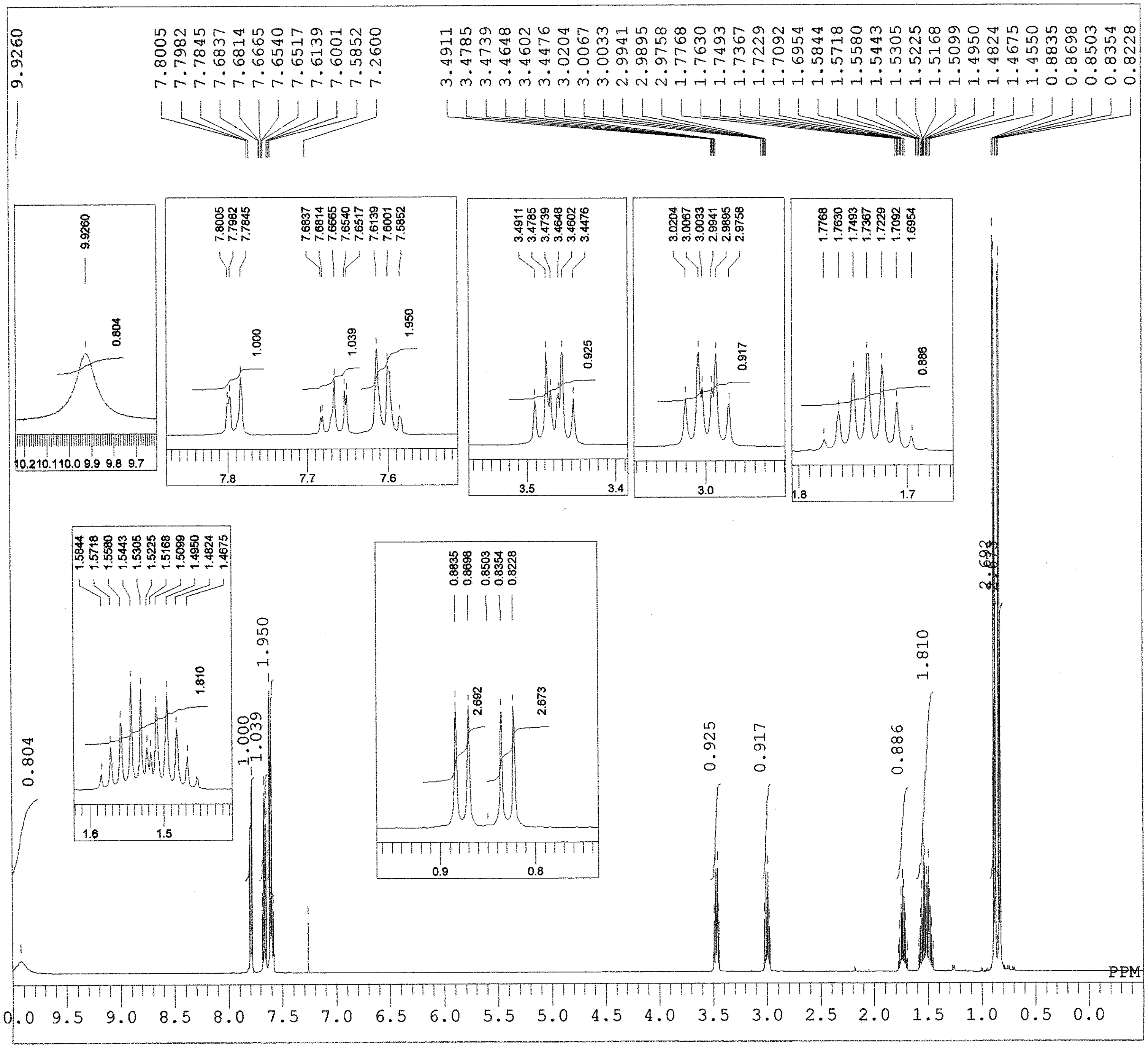

DFILE	ozawa04-063rsm_1H.als
COMNT	MeBuOH, OH
DATIM	2014-01-21 12:34:21
OBNUC	1H
EXMOD	proton.jxp
OBFRQ	500.16 MHz
OBSET	2.41 KHz
OBFIN	6.01 Hz
POINT	13107
FREQU	7507.51 Hz
SCANS	4
ACQTM	1.7459 sec
PD	5.0000 sec
PW1	5.55 usec
IRNUC	1H
CTEMP	21.5 c
SLVNT	CDCL3
EXREF	7.26 ppm
BF	0.12 Hz
RGAIN	30

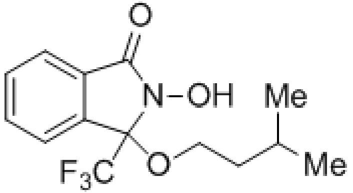

1h

MeBuOH, OH

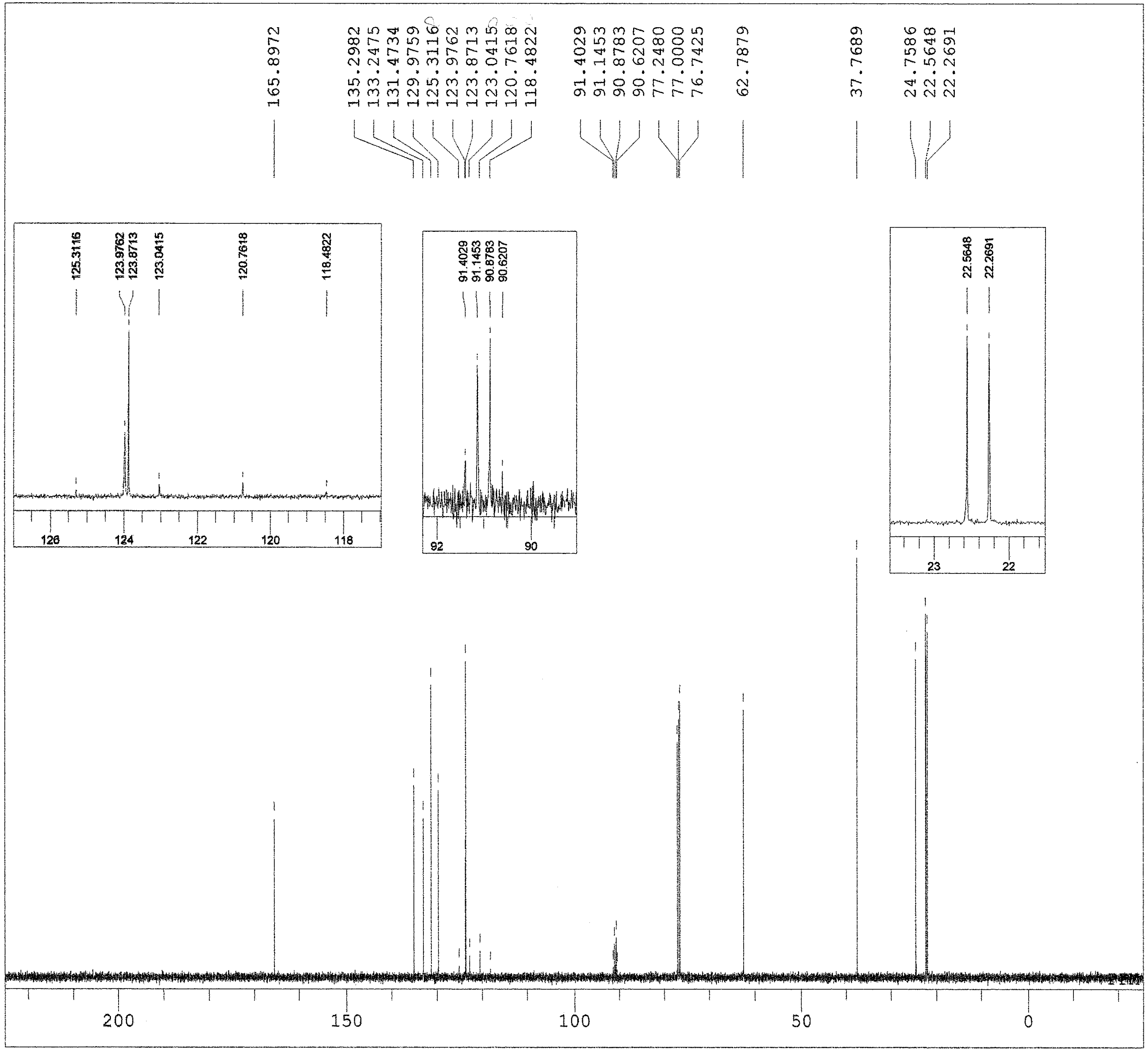

DFILE	ozawa04-063rsm_13C.jdf
COMNT	MeBuOH, OH
DATIM	2014-01-21 12:35:34
OBNUC	13C
EXMOD	carbon.jxp
OBFRQ	125.77 MHz
OBSET	7.87 KHz
OBFIN	4.21 Hz
POINT	32767
FREQU	39308.18 Hz
SCANS	100
ACQTM	0.8336 sec
PD	3.0000 sec
PW1	3.40 usec
IRNUC	1H
CTEMP	21.7 c
SLVNT	CDCL3
EXREF	77.00 ppm
BF	0.12 Hz
RGAIN	60

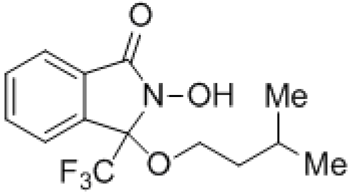

1h

MeBuOH, OH

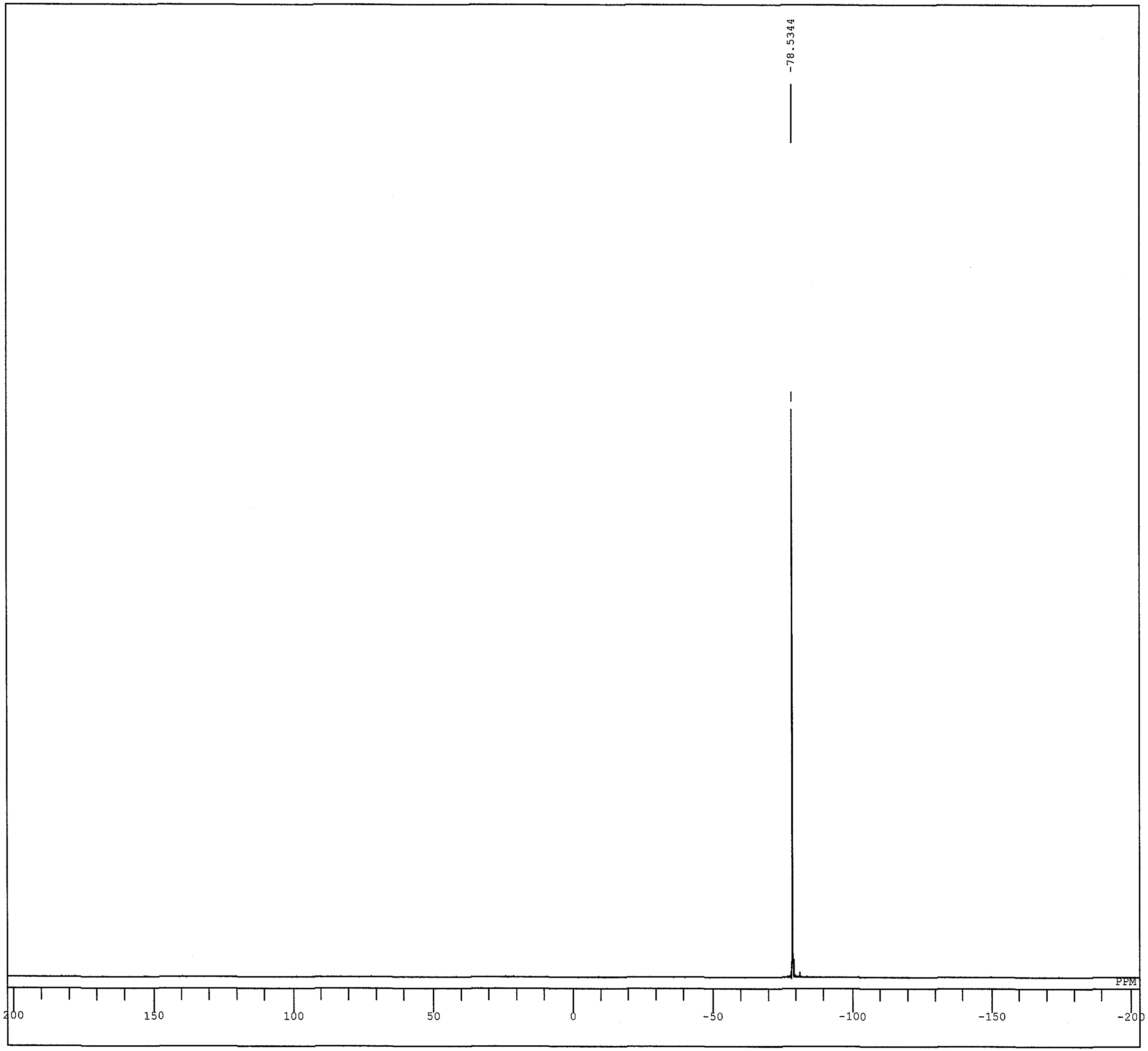

DFILE 1c_ozawa04-63rsm_19F.jdf
COMNT MeBuOH, OH
DATIM 21-01-2014 13:12:14
OBNUC 19F
EXMOD proton.jxp
OBFRQ 368.64 MHz
OBSET 7.63 KHz
OBFIN 2.85 Hz
POINT 16384
FREQU 186567.17 Hz
SCANS 4
ACQTM 0.0878 sec
PD 5.0000 sec
PW1 3.90 usec
IRNUC 19F
CTEMP 20.9 c
SLVNT CDCL3
EXREF -164.90 ppm
BF 0.12 Hz
RGAIN 42

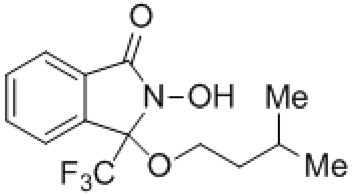

1h

MeBuOH

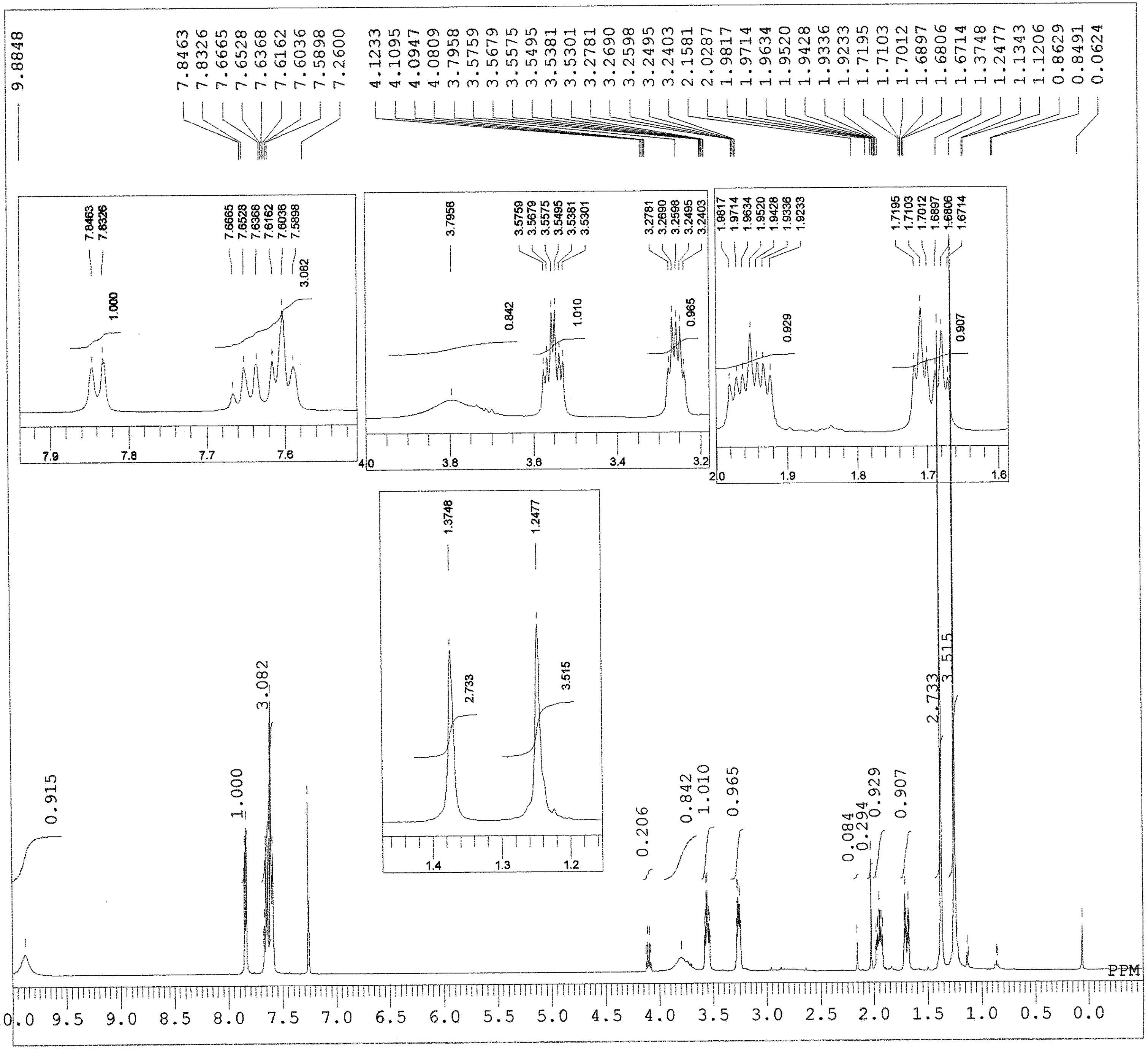

DFILE	ozawa04-046.als
COMNT	MeBuOH
DATIM	2013-11-16 17:26:53
OBNUC	1H
EXMOD	proton.jxp
OBFRQ	500.16 MHz
OBSET	2.41 KHz
OBFIN	6.01 Hz
POINT	13107
FREQU	7507.51 Hz
SCANS	4
ACQTM	1.7459 sec
PD	5.0000 sec
PW1	5.55 usec
IRNUC	1H
CTEMP	22.8 c
SLVNT	CDCL3
EXREF	7.26 ppm
BF	1.00 Hz
RGAIN	30

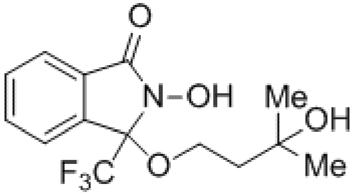

2h

MeBuOH, [0]

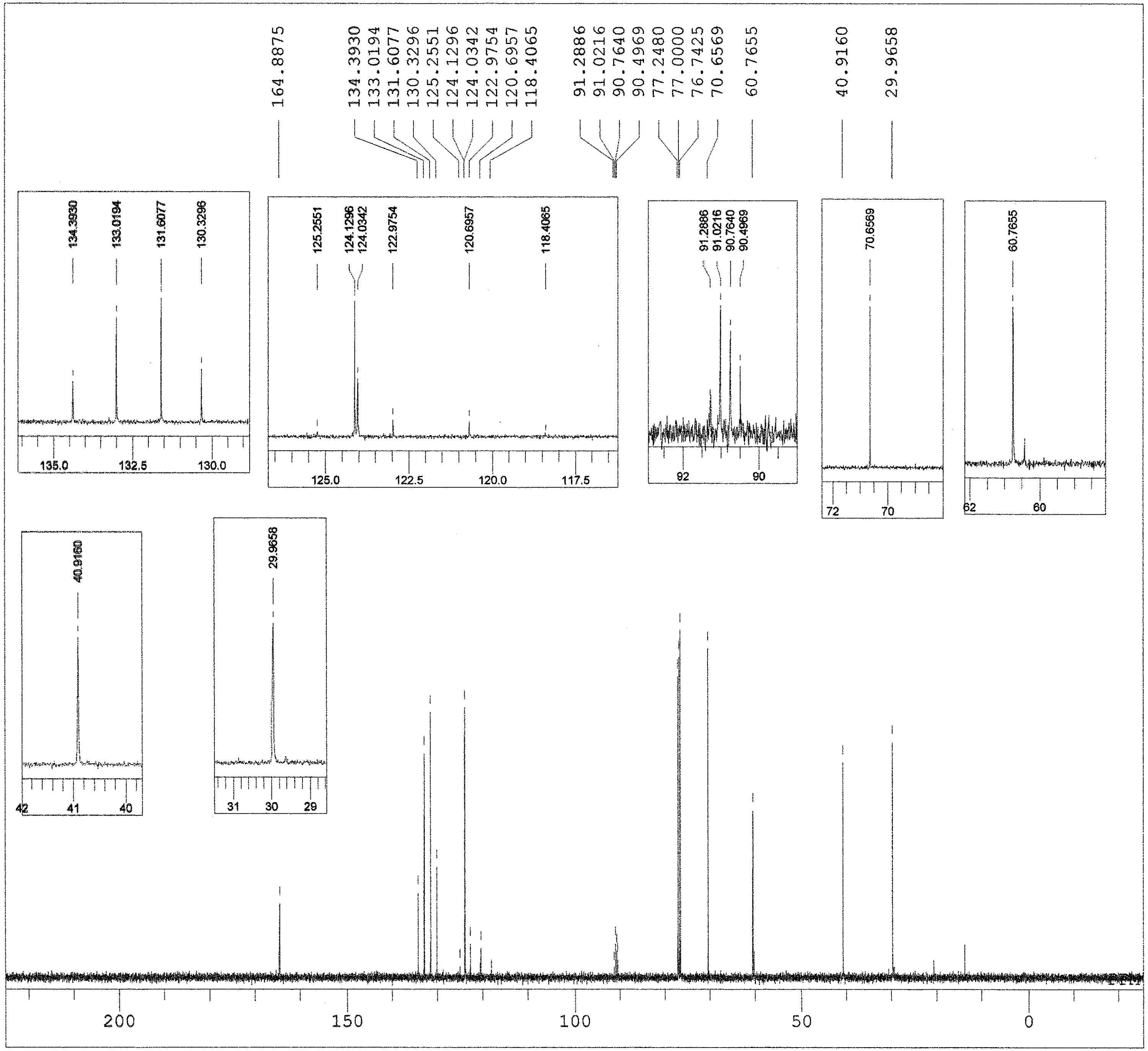

DFILE	ozawa04-046_13C.als
COMNT	MeBuOH, [O]
DATIM	2013-11-16 18:28:22
OBNUC	13C
EXMOD	carbon.jxp
OBFRQ	125.77 MHz
OBSET	7.87 KHz
OBFIN	4.21 Hz
POINT	26214
FREQU	31446.54 Hz
SCANS	160
ACQTM	0.8336 sec
PD	3.0000 sec
PW1	3.40 usec
IRNUC	1H
CTEMP	23.5 c
SLVNT	CDCL3
EXREF	77.00 ppm
BF	0.12 Hz
RGAIN	60

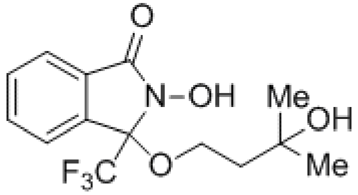

2h

MeBuOH, [O]

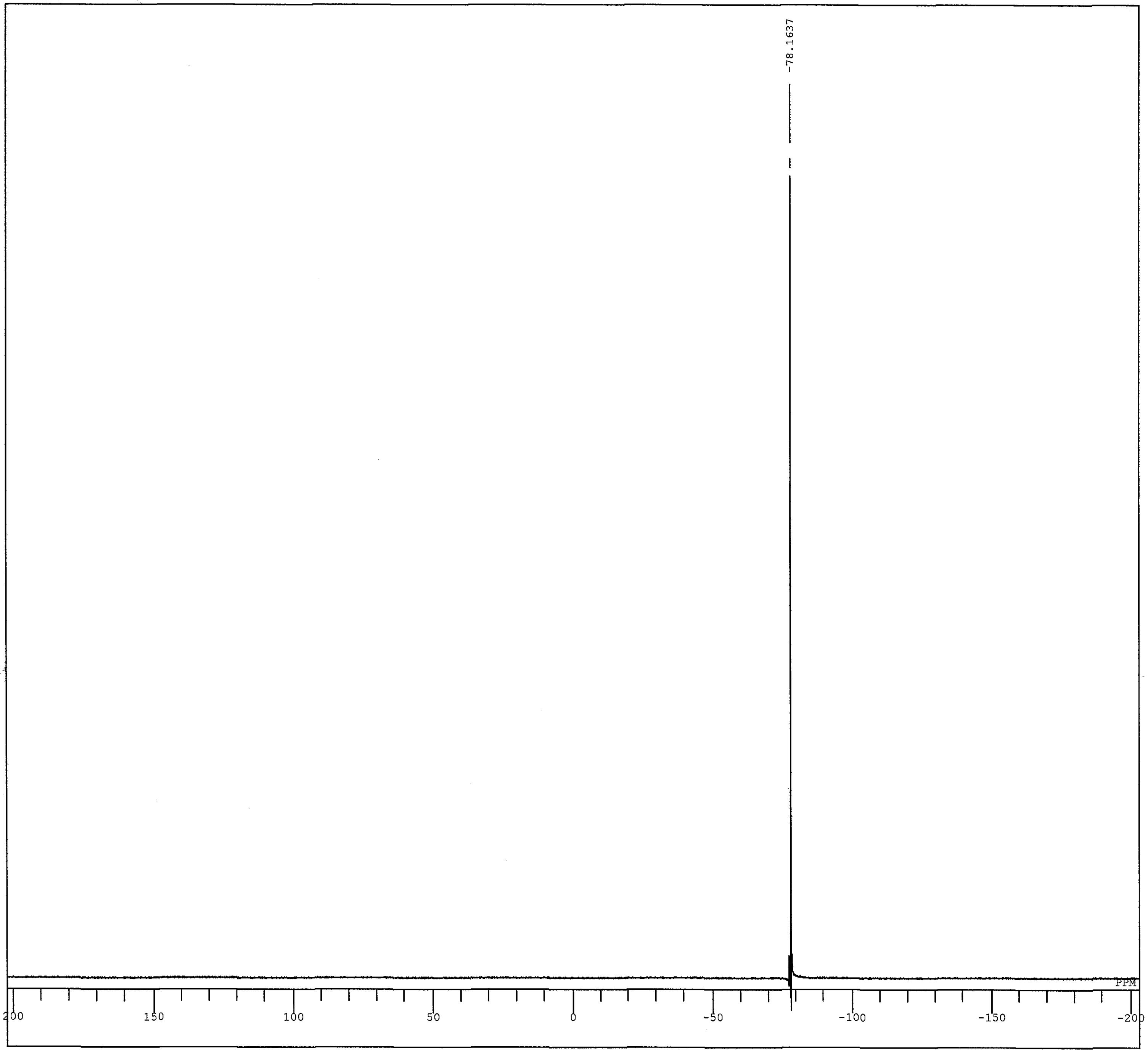

DFILE 2c_ozawa04-095_1_~~1H~~19F.jdf
COMNT MeBuOH, [O]
DATIM 27-01-2014 21:02:12
OBNUC 19F
EXMOD proton.jxp
OBFRQ 368.64 MHz
OBSET 7.63 KHz
OBFIN 2.85 Hz
POINT 16384
FREQU 186567.17 Hz
SCANS 4
ACQTM 0.0878 sec
PD 5.0000 sec
PW1 3.90 usec
IRNUC 19F
CTEMP 21.1 c
SLVNT CDCL3
EXREF -164.90 ppm
BF 0.12 Hz
RGAIN 48

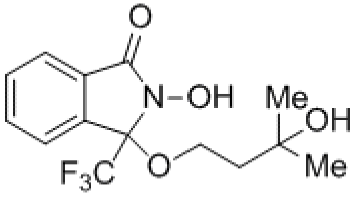

2h

CyEtOH

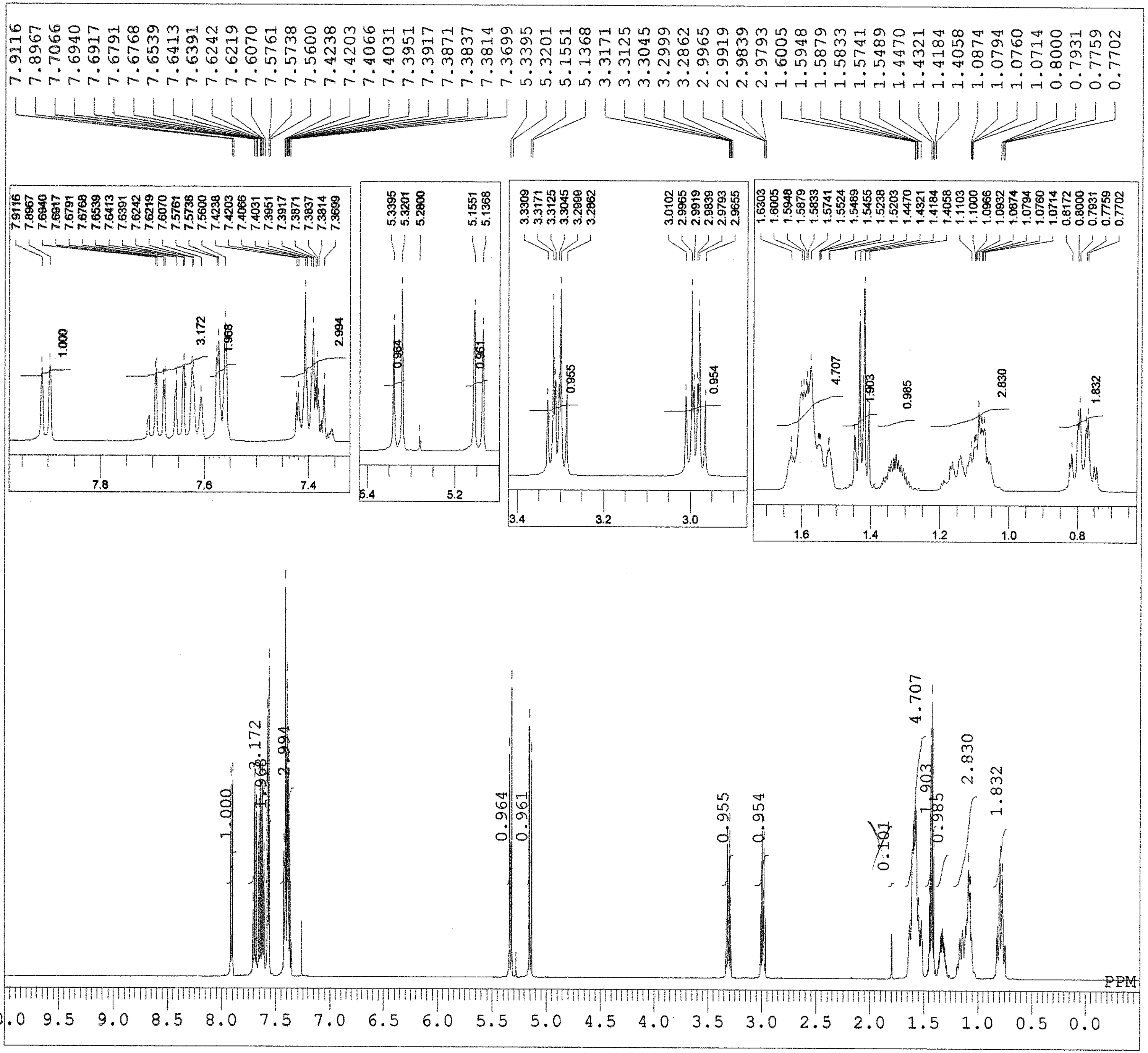

DFILE	ozawa04-050_1H.jdf
COMNT	CyEtOH
DATIM	2013-11-25 14:17:48
OBNUC	1H
EXMOD	proton.jxp
OBFRQ	500.16 MHz
OBSET	2.41 KHz
OBFIN	6.01 Hz
POINT	16384
FREQU	9384.38 Hz
SCANS	4
ACQTM	1.7459 sec
PD	5.0000 sec
PW1	5.55 usec
IRNUC	1H
CTEMP	22.8 c
SLVNT	CDCL3
EXREF	7.26 ppm
BF	0.12 Hz
RGAIN	26

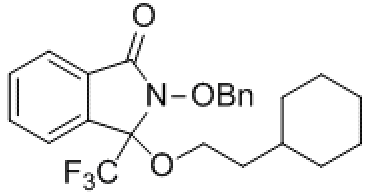

S4i

CyEtOH

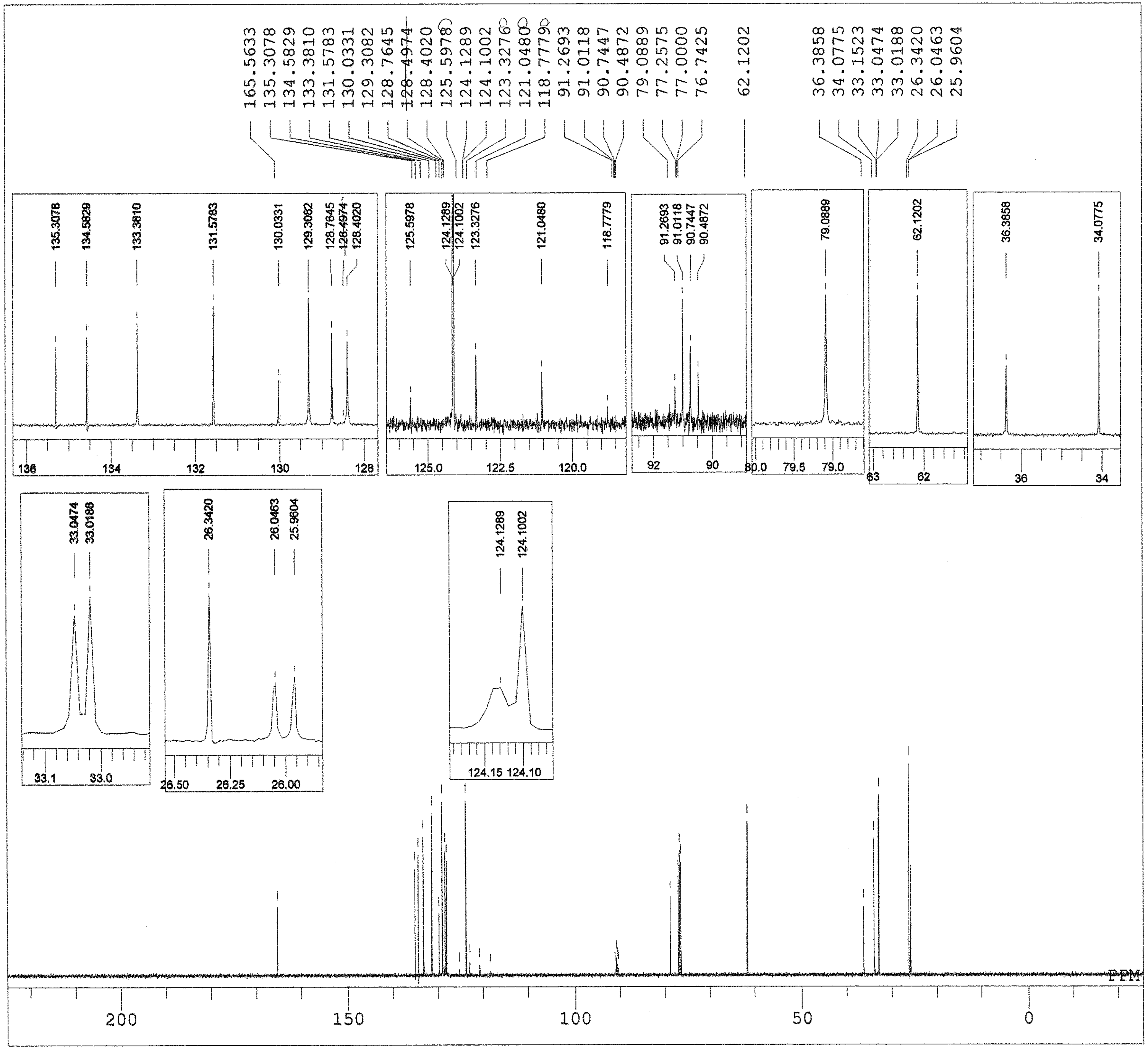

DFILE	ozawa04-050_13C.jdf	
COMNT	CyEtOH	
DATIM	2013-11-25 17:16:21	
OBNUC	13C	
EXMOD	carbon.jxp	
OBFRQ	125.77	MHz
OBSET	7.87	KHz
OBFIN	4.21	Hz
POINT	32767	
FREQU	39308.18	Hz
SCANS	140	
ACQTM	0.8336	sec
PD	3.0000	sec
PW1	3.40	usec
IRNUC	1H	
CTEMP	23.0	c
SLVNT	CDCL3	
EXREF	77.00	ppm
BF	0.12	Hz
RGAIN	60	

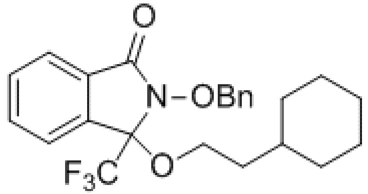

S4i

CyEtOH

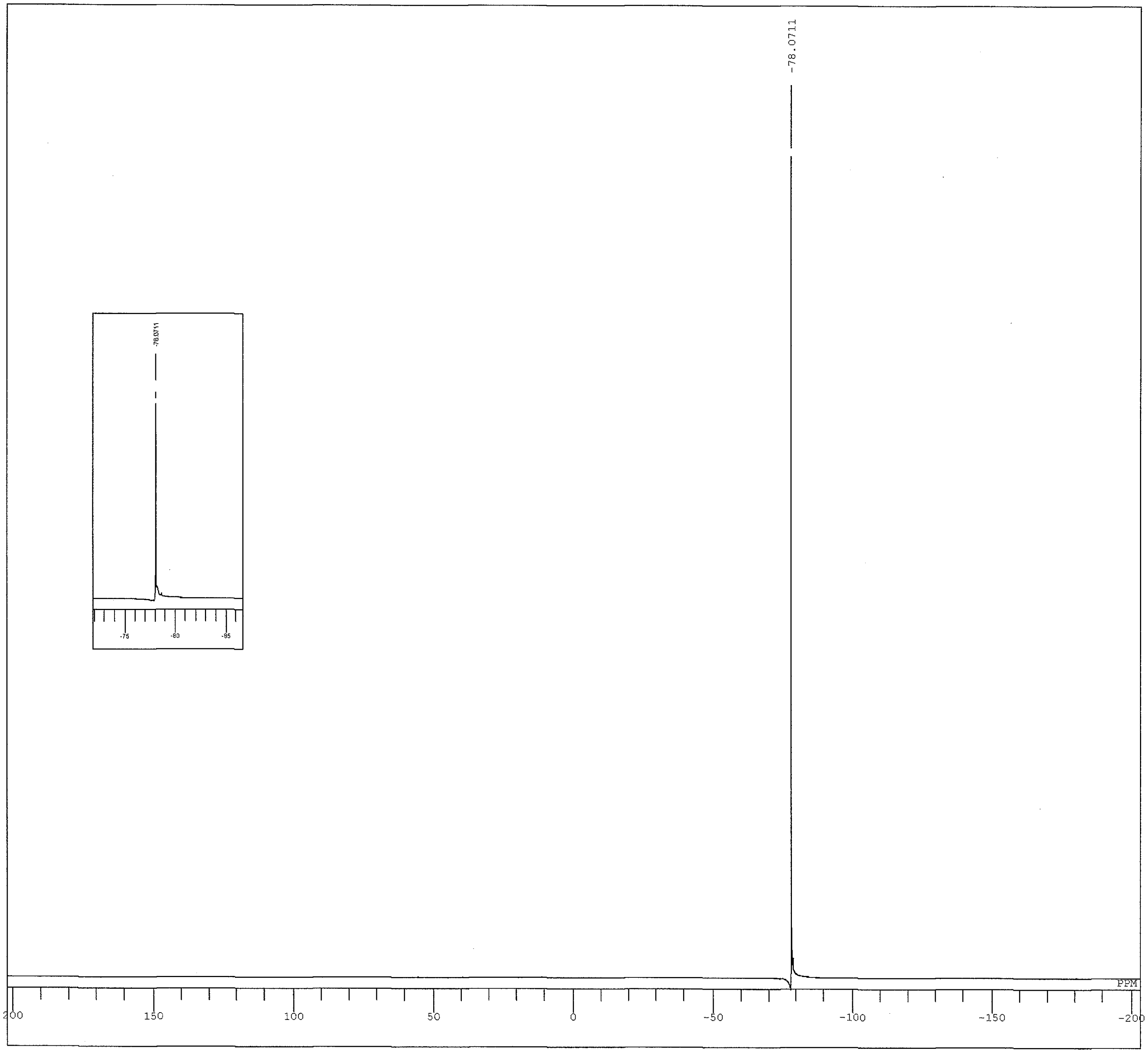

DFILE	ozawa04-050_19F.jdf
COMNT	CyEtOH
DATIM	25-11-2013 13:45:34
OBNUC	19F
EXMOD	proton.jxp
OBFRQ	368.64 MHz
OBSET	7.63 KHz
OBFIN	2.85 Hz
POINT	16384
FREQU	186567.17 Hz
SCANS	4
ACQTM	0.0878 sec
PD	5.0000 sec
PW1	3.90 usec
IRNUC	19F
CTEMP	21.2 c
SLVNT	CDCL3
EXREF	-164.90 ppm
BF	0.12 Hz
RGAIN	46

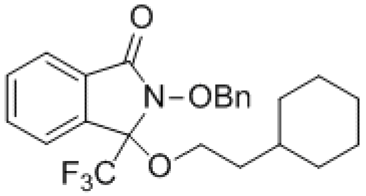

S4i

column, CyEtOH

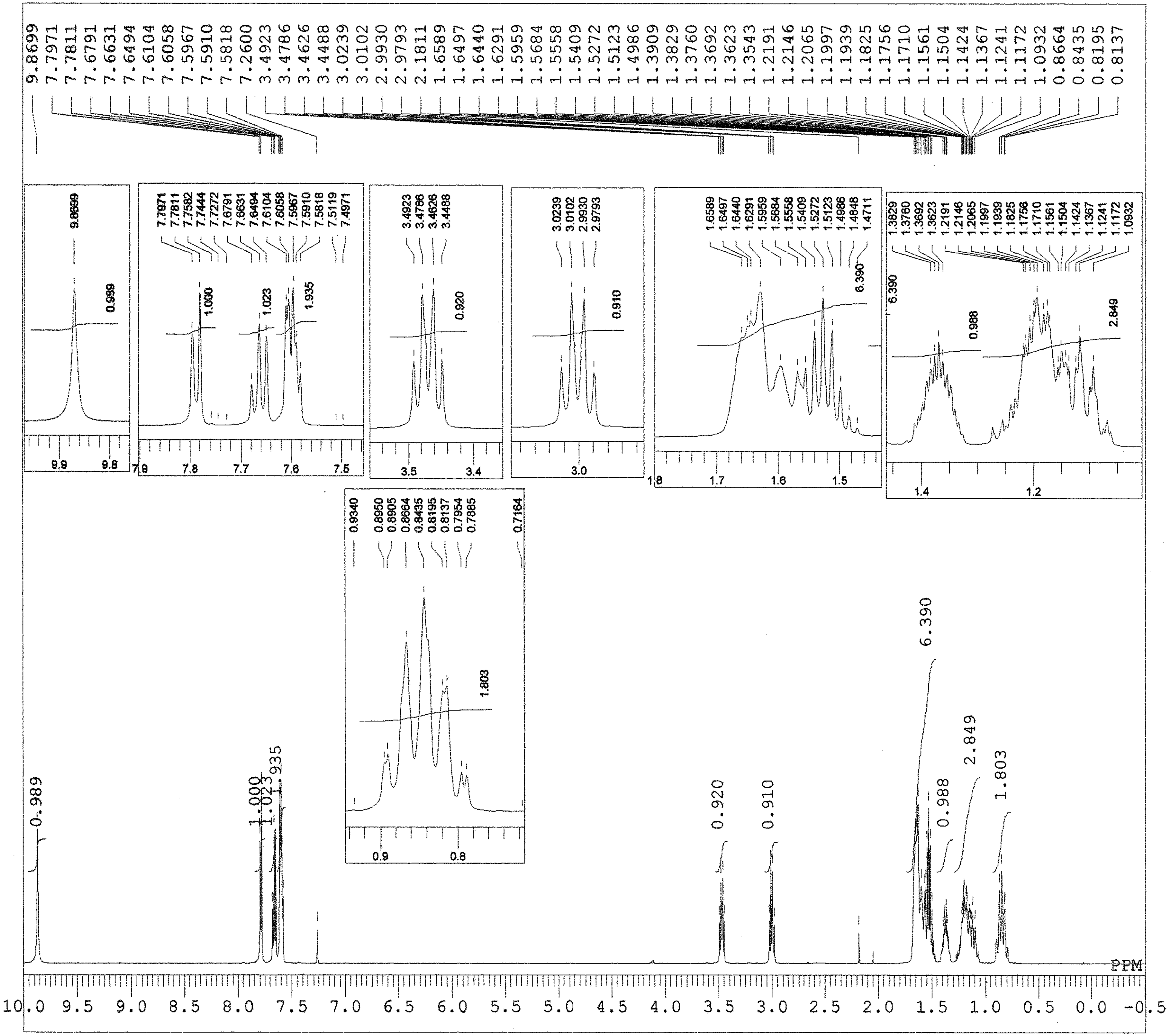

DFILE	ozawa04-056_1_1H.jdf
COMNT	column, CyEtOH
DATIM	2013-11-29 14:27:28
OBNUC	1H
EXMOD	proton.jxp
OBFRQ	500.16 MHz
OBSET	2.41 KHz
OBFIN	6.01 Hz
POINT	16384
FREQU	9384.38 Hz
SCANS	4
ACQTM	1.7459 sec
PD	5.0000 sec
PW1	5.55 usec
IRNUC	1H
CTEMP	22.7 c
SLVNT	CDCL3
EXREF	7.26 ppm
BF	0.12 Hz
RGAIN	30

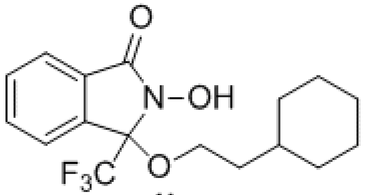

1i

CyEtOH

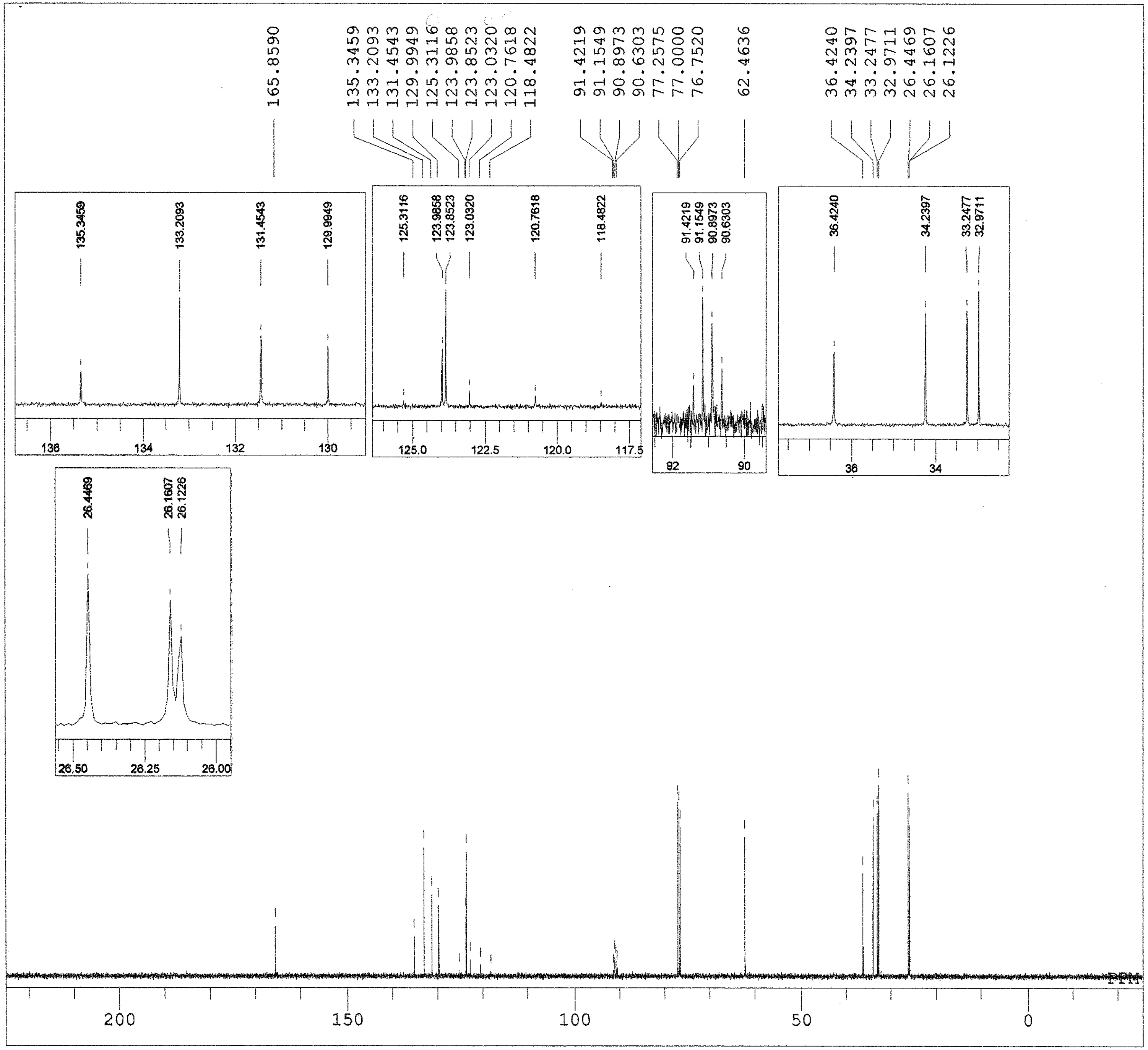

DFILE	ozawa04-056_1_13C.jdf
COMNT	CyEtOH
DATIM	2013-11-29 14:28:32
OBNUC	13C
EXMOD	carbon.jxp
OBFRQ	125.77 MHz
OBSET	7.87 KHz
OBFIN	4.21 Hz
POINT	32767
FREQU	39308.18 Hz
SCANS	124
ACQTM	0.8336 sec
PD	3.0000 sec
PW1	3.40 usec
IRNUC	1H
CTEMP	22.8 c
SLVNT	CDCL3
EXREF	77.00 ppm
BF	0.12 Hz
RGAIN	60

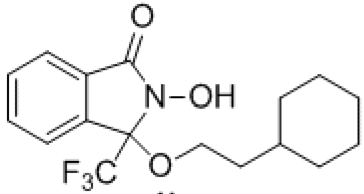

1i

column, CyEtOH

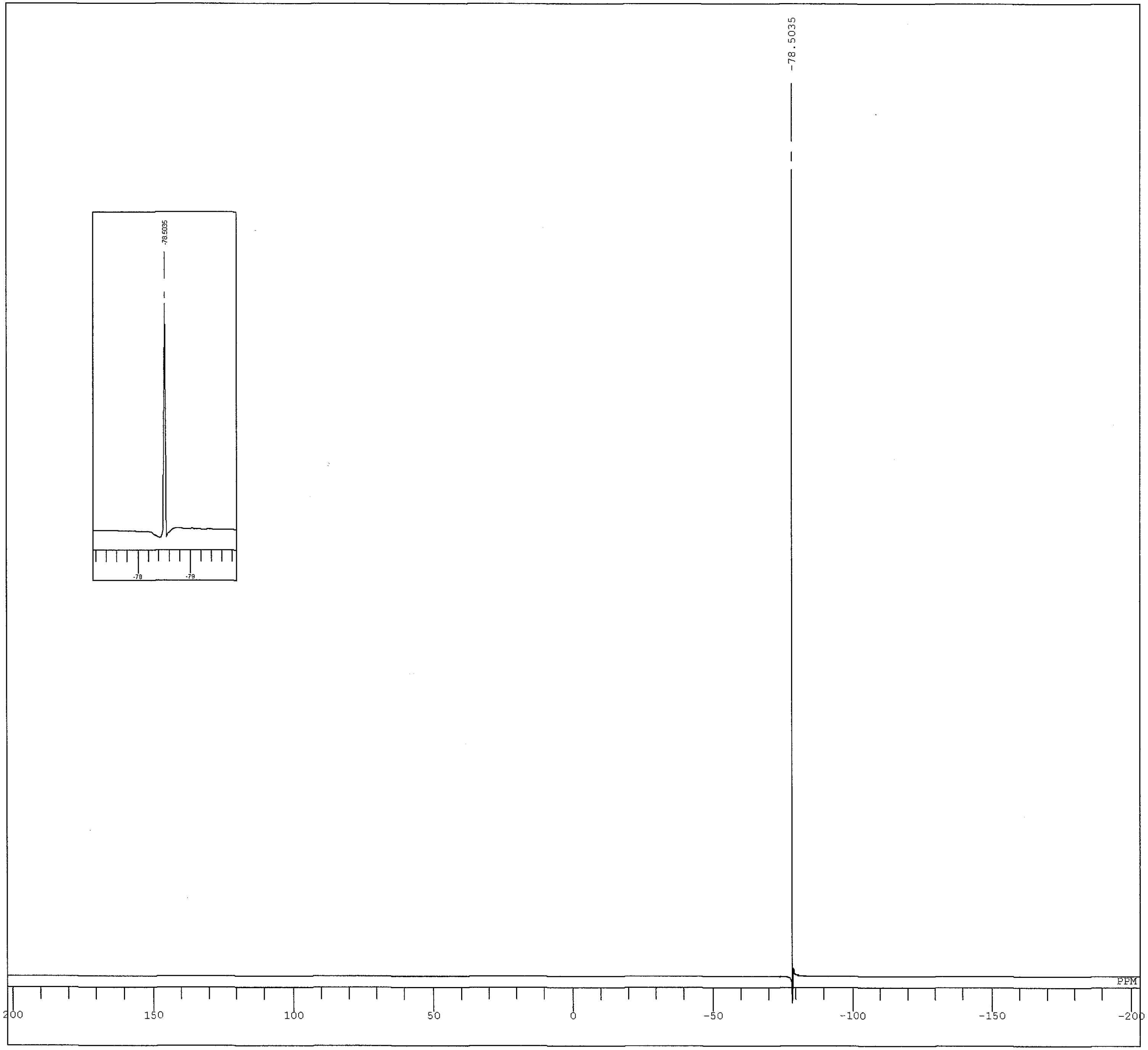

DFILE ozawa04-056_1_19F.jdf
COMNT column, CyEtOH
DATIM 29-11-2013 14:25:48
OBNUC 19F
EXMOD proton.jxp
OBFRQ 368.64 MHz
OBSET 7.63 KHz
OBFIN 2.85 Hz
POINT 16384
FREQU 186567.17 Hz
SCANS 4
ACQTM 0.0878 sec
PD 5.0000 sec
PW1 3.90 usec
IRNUC 19F
CTEMP 21.7 c
SLVNT CDCL3
EXREF -164.90 ppm
BF 0.12 Hz
RGAIN 44

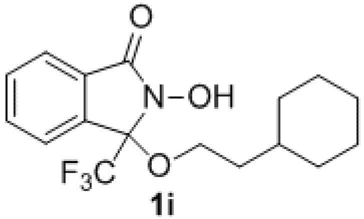

1i

PTLC, CyEtOH, 02

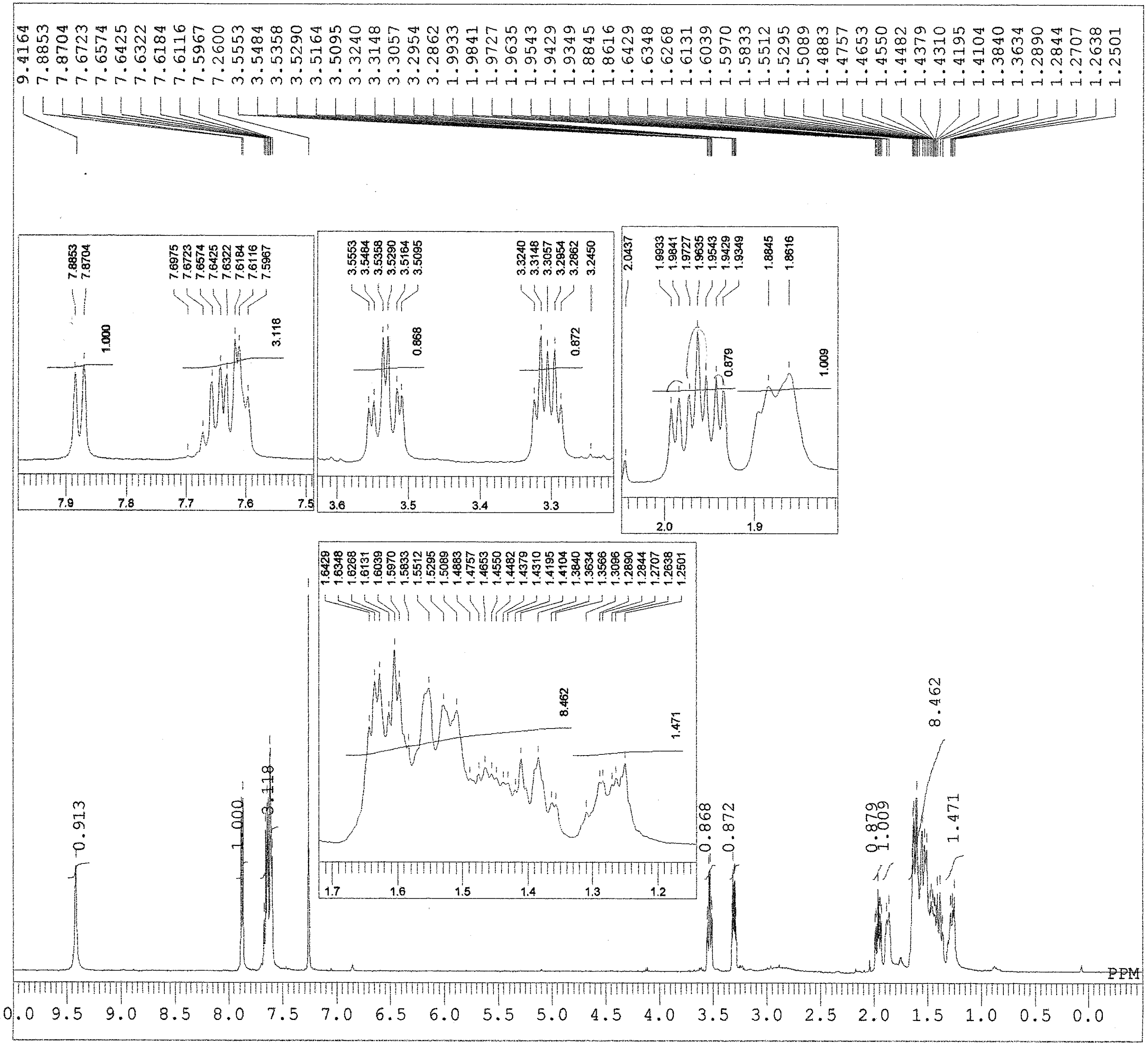

DFILE	ozawa04-095_3_1H.jdf
COMNT	PTLC, CyEtOH, 02
DATIM	2014-01-20 17:45:28
OBNUC	1H
EXMOD	proton.jxp
OBFRQ	500.16 MHz
OBSET	2.41 KHz
OBFIN	6.01 Hz
POINT	16384
FREQU	9384.38 Hz
SCANS	4
ACQTM	1.7459 sec
PD	5.0000 sec
PW1	5.55 usec
IRNUC	1H
CTEMP	21.2 c
SLVNT	CDCL3
EXREF	7.26 ppm
BF	1.00 Hz
RGAIN	38

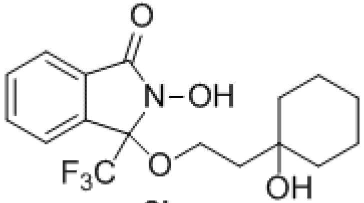

2i

CyEtOH, [O]

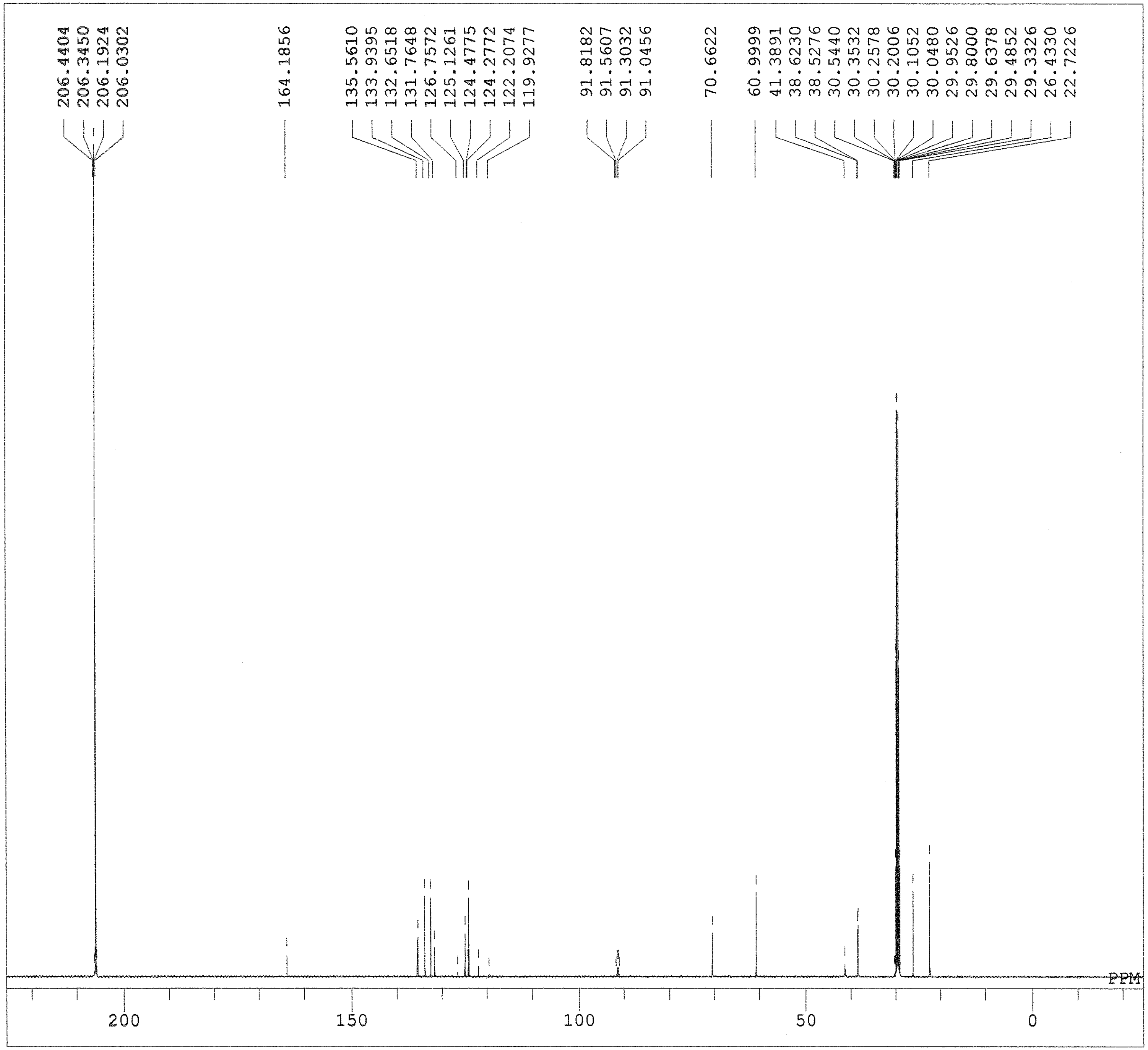

DFILE	ozawa04-095_3_13C.jdf
COMNT	CyEtOH, [O]
DATIM	2014-01-27 17:41:41
OBNUC	13C
EXMOD	carbon.jxp
OBFRQ	125.77 MHz
OBSET	7.87 KHz
OBFIN	4.21 Hz
POINT	32767
FREQU	39308.18 Hz
SCANS	1700
ACQTM	0.8336 sec
PD	3.0000 sec
PW1	3.40 usec
IRNUC	1H
CTEMP	22.5 c
SLVNT	ACETN
EXREF	29.80 ppm
BF	1.00 Hz
RGAIN	60

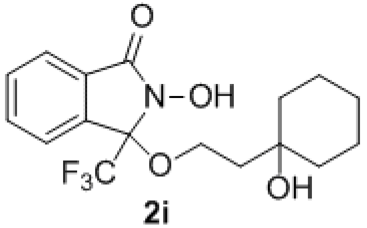

2i

CyEtOH, [O]

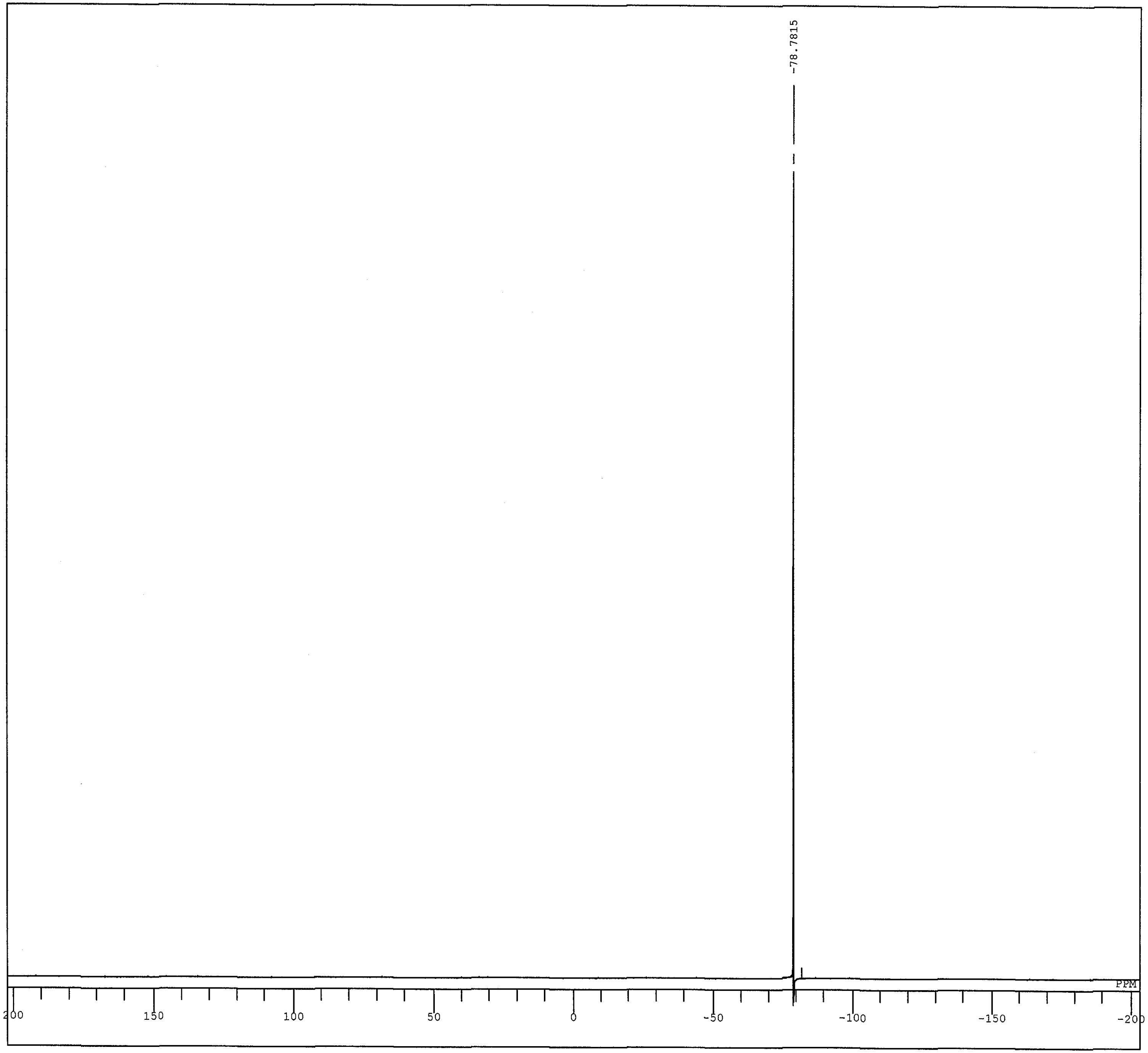

DFILE ozawa04-095_3_19F.jdf
COMNT CyEtOH, [O]
DATIM 27-01-2014 19:53:16
OBNUC 19F
EXMOD proton.jxp
OBFRQ 368.64 MHz
OBSET 7.63 KHz
OBFIN 2.85 Hz
POINT 16384
FREQU 186567.17 Hz
SCANS 4
ACQTM 0.0878 sec
PD 5.0000 sec
PW1 3.90 usec
IRNUC 19F
CTEMP 21.1 c
SLVNT ACETN
EXREF -164.90 ppm
BF 0.10 Hz
RGAIN 48

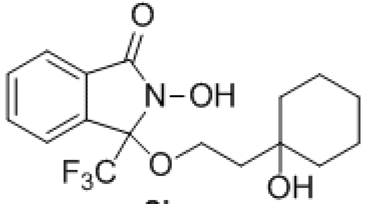

2i

CyMeOH, OH

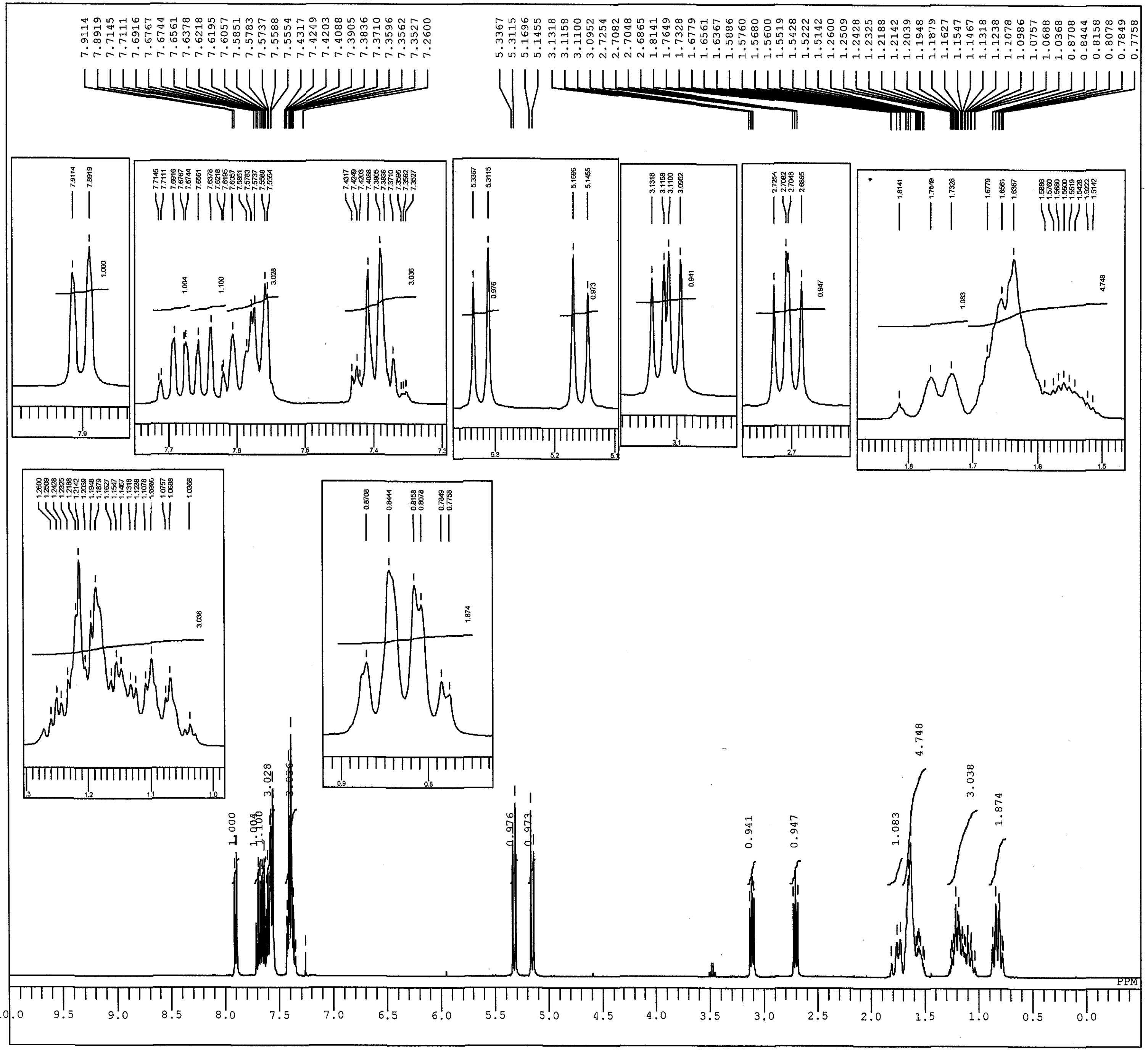

```
DFILE   ozawa04-122_2_1H.jdf
COMNT   CyMeOH, OH
DATIM   02-02-2014 20:32:38
OBNUC   1H
EXMOD   proton.jxp
OBFRQ        391.78 MHz
OBSET          8.51 KHz
OBFIN          3.34 Hz
POINT         16384
FREQU       7348.62 Hz
SCANS             4
ACQTM        2.2295 sec
PD           5.0000 sec
PW1            5.25 usec
IRNUC   1H
CTEMP          21.1 c
SLVNT   CDCL3
EXREF          7.26 ppm
BF             0.12 Hz
RGAIN            22
```

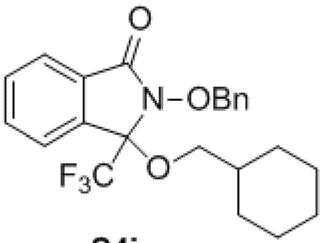

S4j

CyMeOH, OH

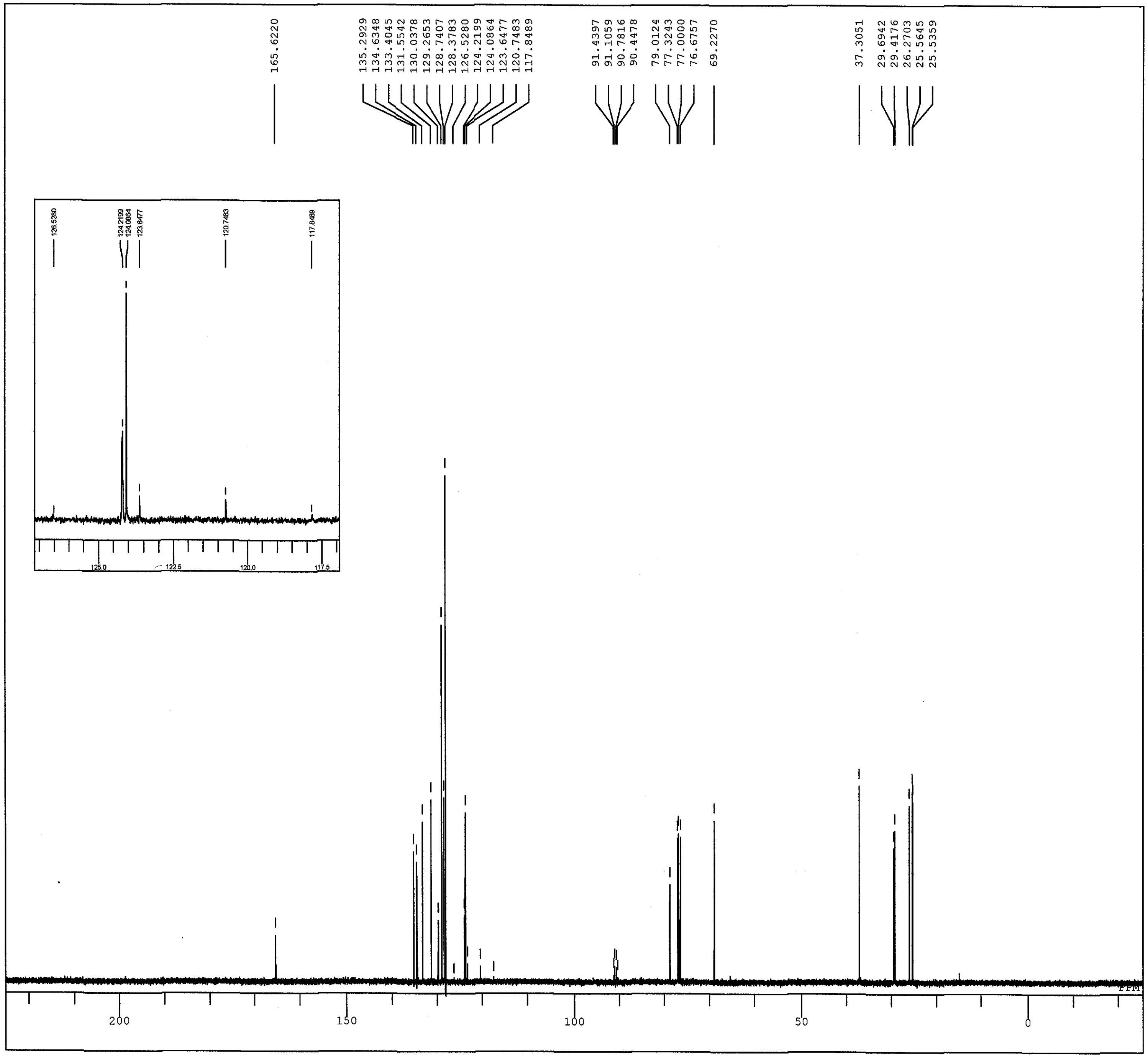

DFILE ozawa04-122_2_13C.jdf
COMNT CyMeOH, OH
DATIM 02-02-2014 20:34:01
OBNUC 13C
EXMOD carbon.jxp
OBFRQ 98.52 MHz
OBSET 4.64 KHz
OBFIN 8.74 Hz
POINT 32767
FREQU 30788.18 Hz
SCANS 112
ACQTM 1.0643 sec
PD 3.0000 sec
PW1 3.00 usec
IRNUC 1H
CTEMP 21.1 c
SLVNT CDCL3
EXREF 77.00 ppm
BF 0.12 Hz
RGAIN 60

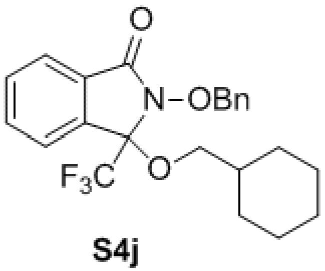

S4j

CyMeOH, Bn

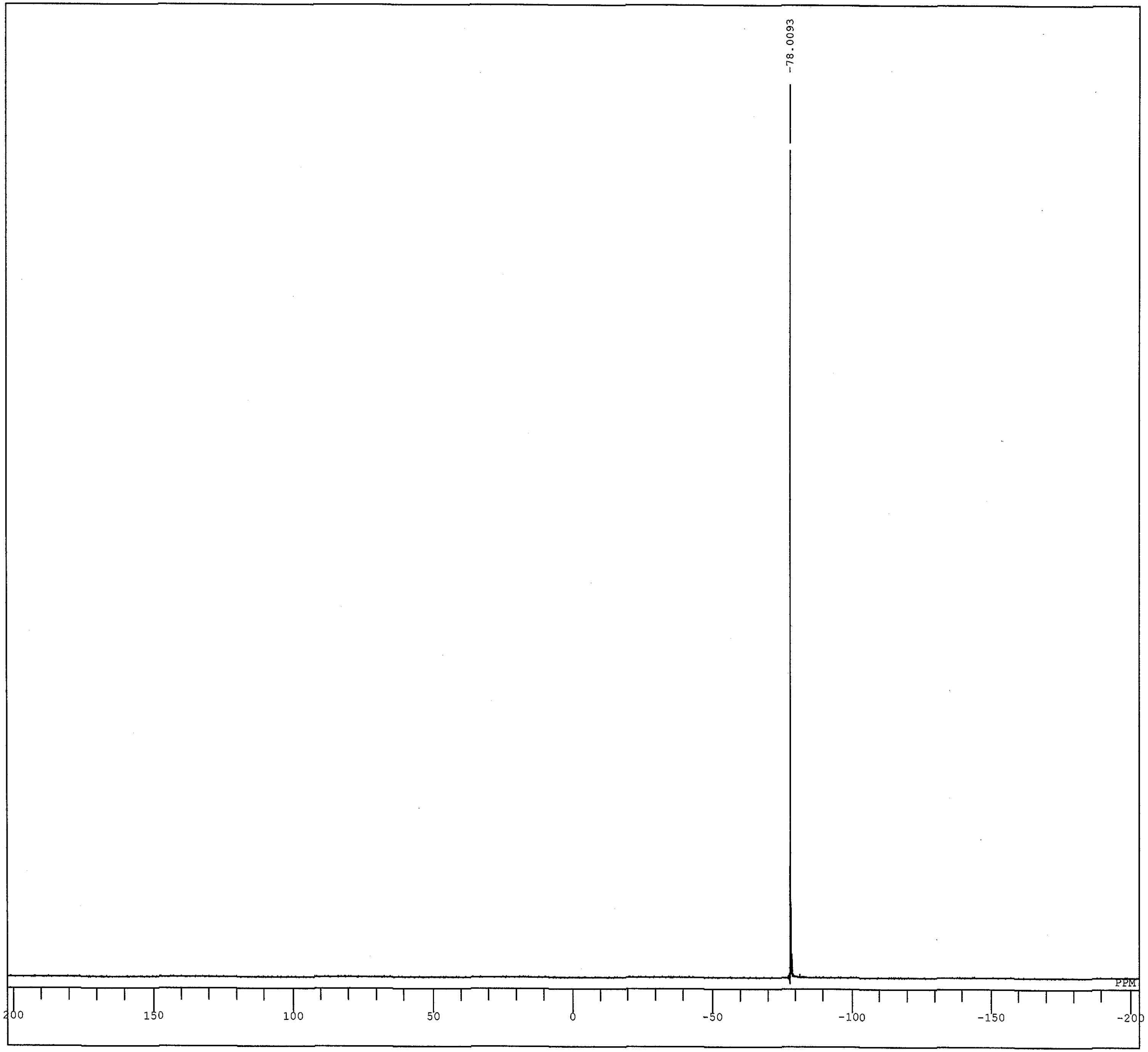

DFILE ozawa04-122_2_C_19F.jdf
COMNT CyMeOH, Bn
DATIM 02-02-2014 14:40:20
OBNUC 19F
EXMOD proton.jxp
OBFRQ 368.64 MHz
OBSET 7.63 KHz
OBFIN 2.85 Hz
POINT 16384
FREQU 186567.17 Hz
SCANS 4
ACQTM 0.0878 sec
PD 5.0000 sec
PW1 3.90 usec
IRNUC 19F
CTEMP 21.1 c
SLVNT CDCL3
EXREF -164.90 ppm
BF 0.12 Hz
RGAIN 46

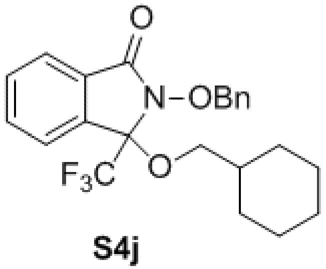

S4j

CyMeOH, OH

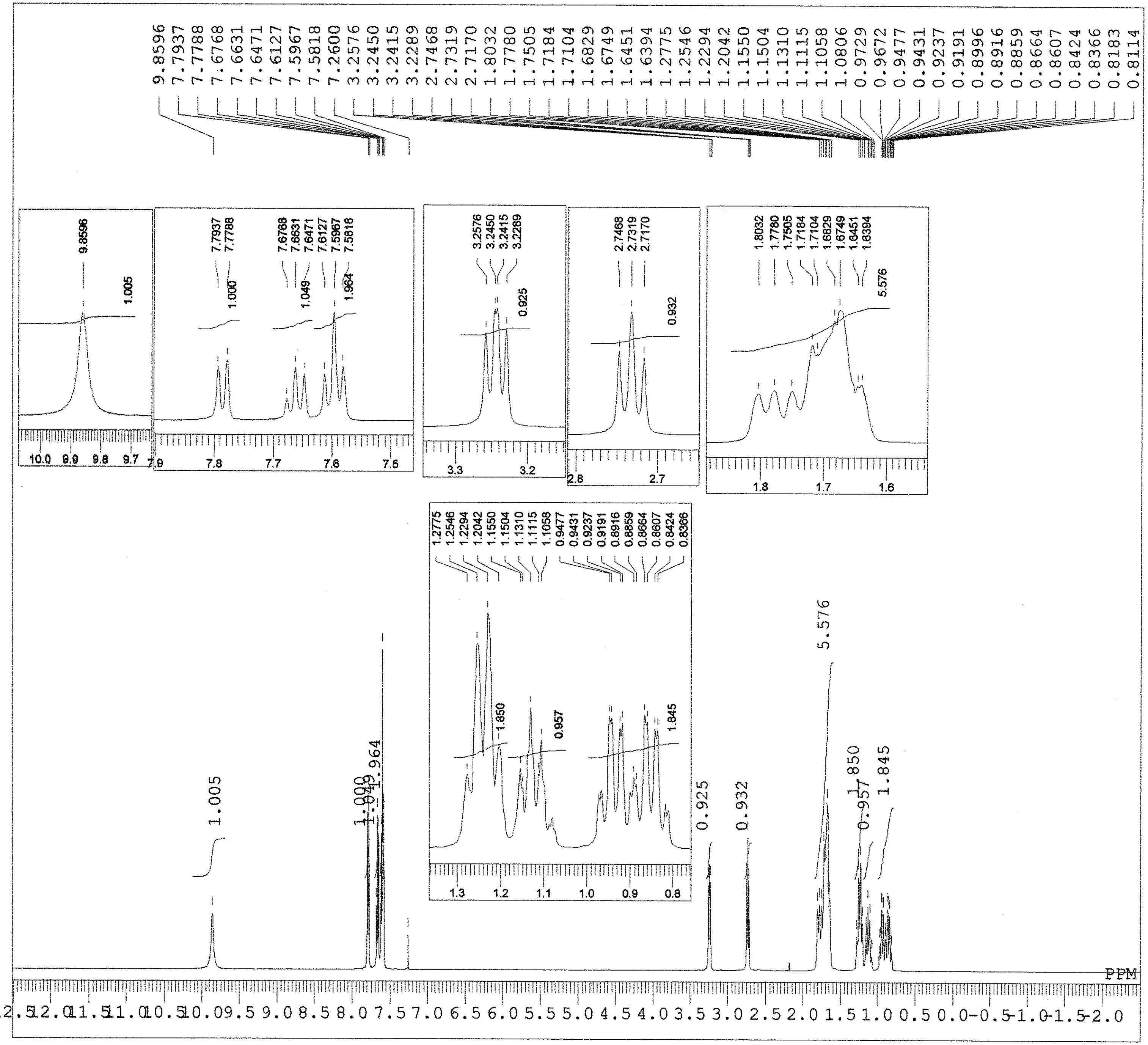

DFILE	ozawa04-016_3_1H.jdf
COMNT	CyMeOH, OH
DATIM	2014-02-01 19:22:50
OBNUC	1H
EXMOD	proton.jxp
OBFRQ	500.16 MHz
OBSET	2.41 KHz
OBFIN	6.01 Hz
POINT	16384
FREQU	9384.38 Hz
SCANS	4
ACQTM	1.7459 sec
PD	5.0000 sec
PW1	5.55 usec
IRNUC	1H
CTEMP	21.5 c
SLVNT	CDCL3
EXREF	7.26 ppm
BF	1.00 Hz
RGAIN	30

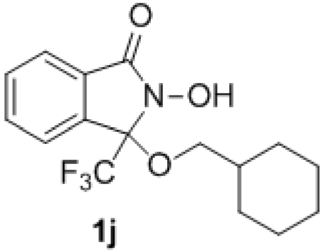

1j

CyMeOH, OH

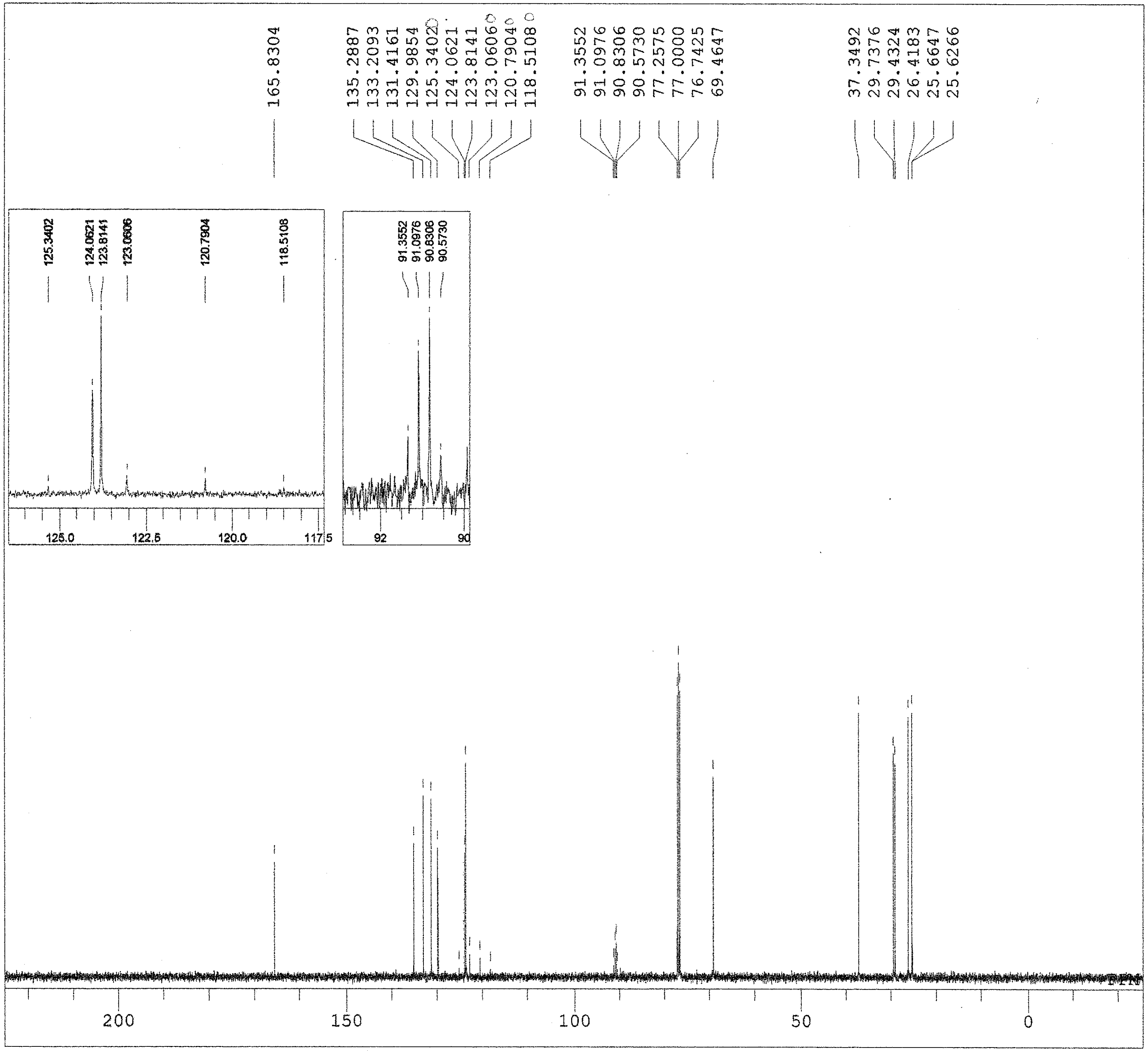

DFILE	ozawa04-016_3_13C.jdf
COMNT	CyMeOH, OH
DATIM	2014-02-01 19:23:53
OBNUC	13C
EXMOD	carbon.jxp
OBFRQ	125.77 MHz
OBSET	7.87 KHz
OBFIN	4.21 Hz
POINT	32767
FREQU	39308.18 Hz
SCANS	60
ACQTM	0.8336 sec
PD	3.0000 sec
PW1	3.40 usec
IRNUC	1H
CTEMP	21.5 c
SLVNT	CDCL3
EXREF	77.00 ppm
BF	1.00 Hz
RGAIN	60

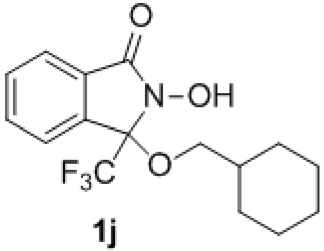

1j

CyMeOH, OH

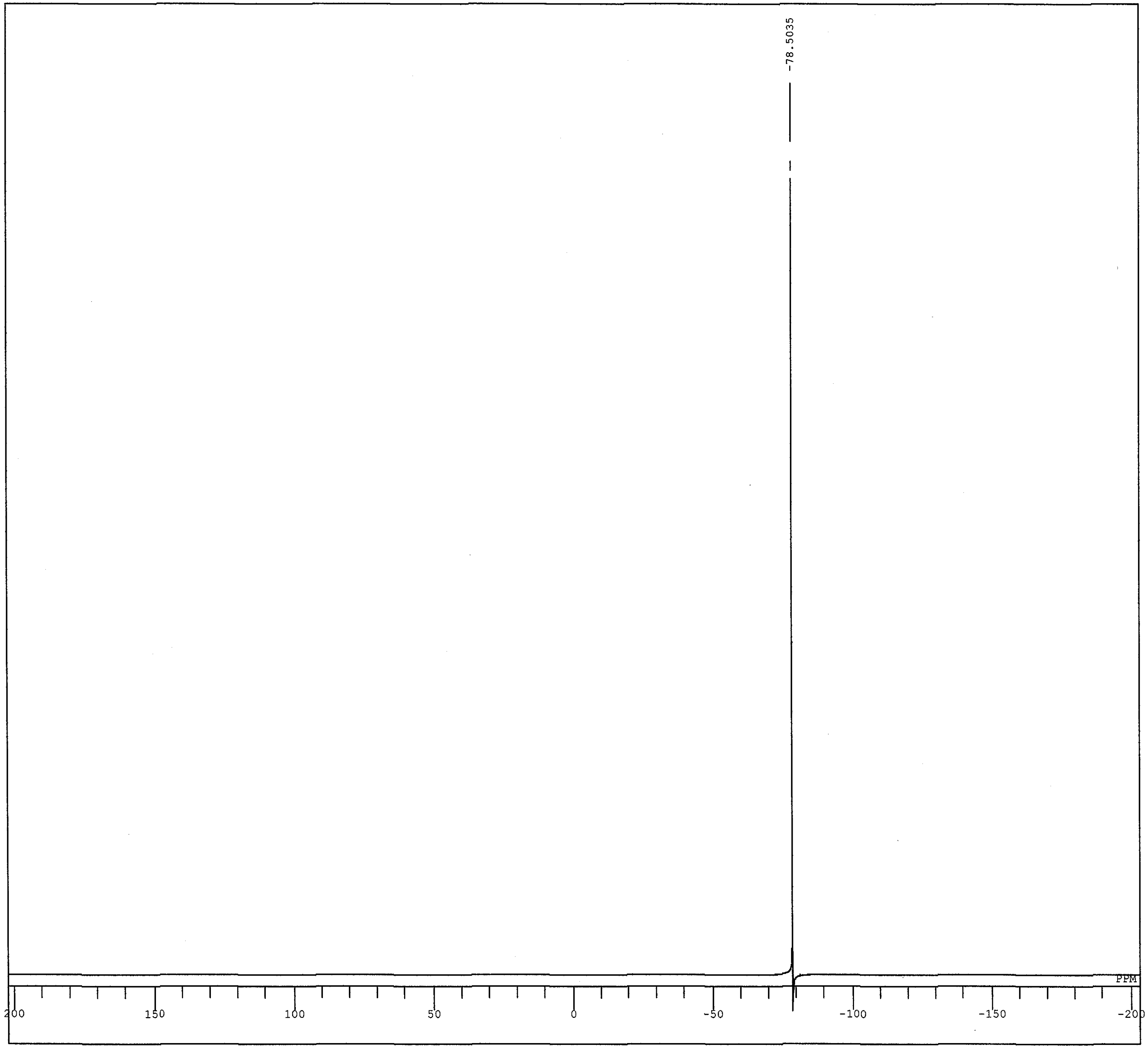

DFILE	ozawa04-016_3_19F.jdf
COMNT	CyMeOH, OH
DATIM	01-02-2014 19:56:07
OBNUC	19F
EXMOD	proton.jxp
OBFRQ	368.64 MHz
OBSET	7.63 KHz
OBFIN	2.85 Hz
POINT	16384
FREQU	186567.17 Hz
SCANS	4
ACQTM	0.0878 sec
PD	5.0000 sec
PW1	3.90 usec
IRNUC	19F
CTEMP	21.1 c
SLVNT	CDCL3
EXREF	-164.90 ppm
BF	0.12 Hz
RGAIN	44

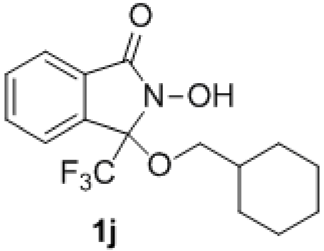

1j

CyMeOH, [O]

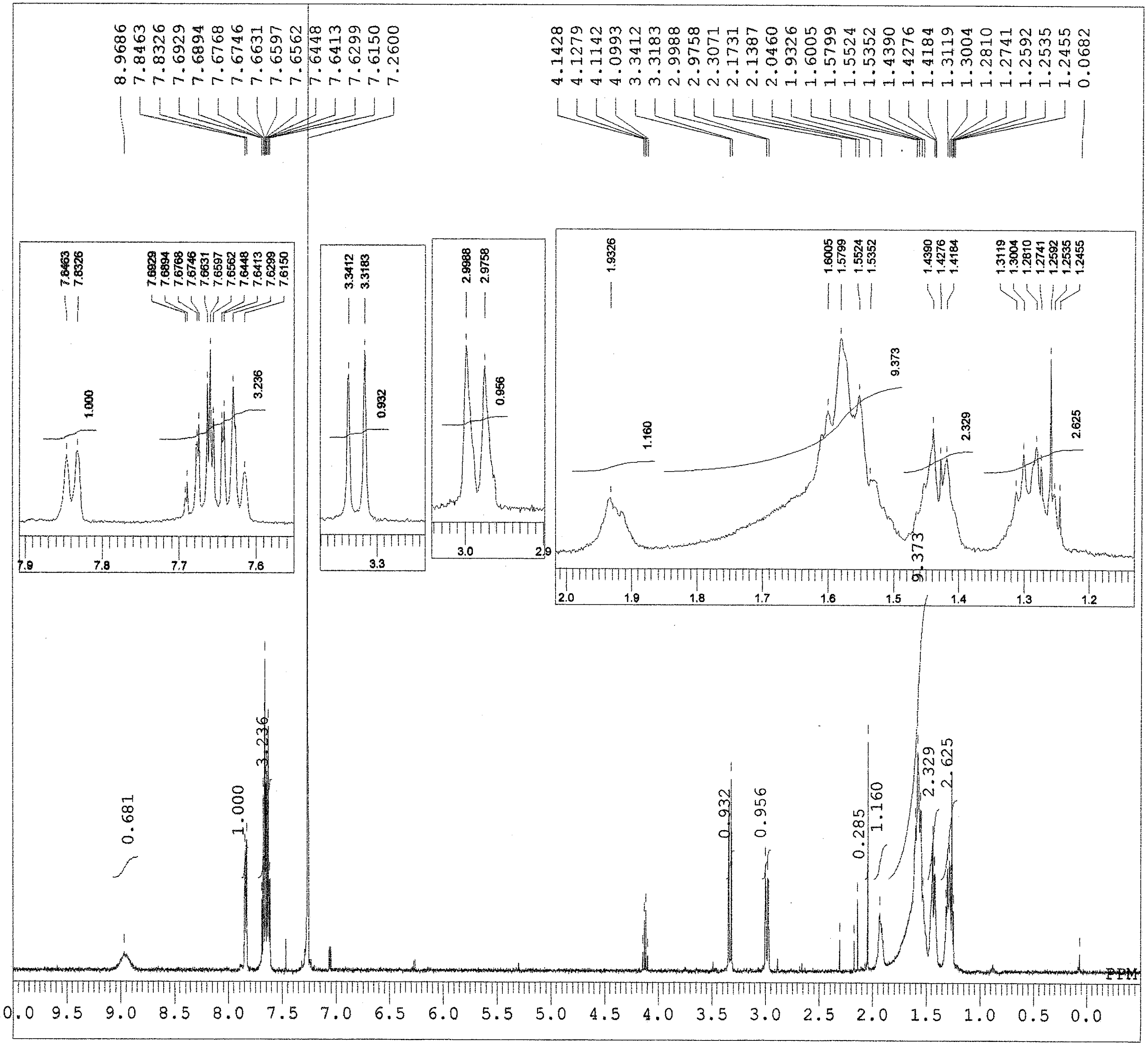

DFILE	ozawa04-126_1H.jdf
COMNT	CyMeOH, [O]
DATIM	2014-03-11 20:54:32
OBNUC	1H
EXMOD	proton.jxp
OBFRQ	500.16 MHz
OBSET	2.41 KHz
OBFIN	6.01 Hz
POINT	16384
FREQU	9384.38 Hz
SCANS	16
ACQTM	1.7459 sec
PD	5.0000 sec
PW1	5.55 usec
IRNUC	1H
CTEMP	20.8 c
SLVNT	CDCL3
EXREF	7.26 ppm
BF	0.12 Hz
RGAIN	40

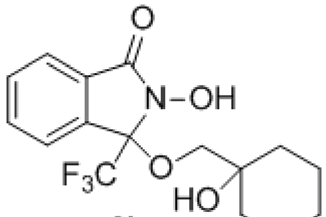

2j

CyMeOH, [O]

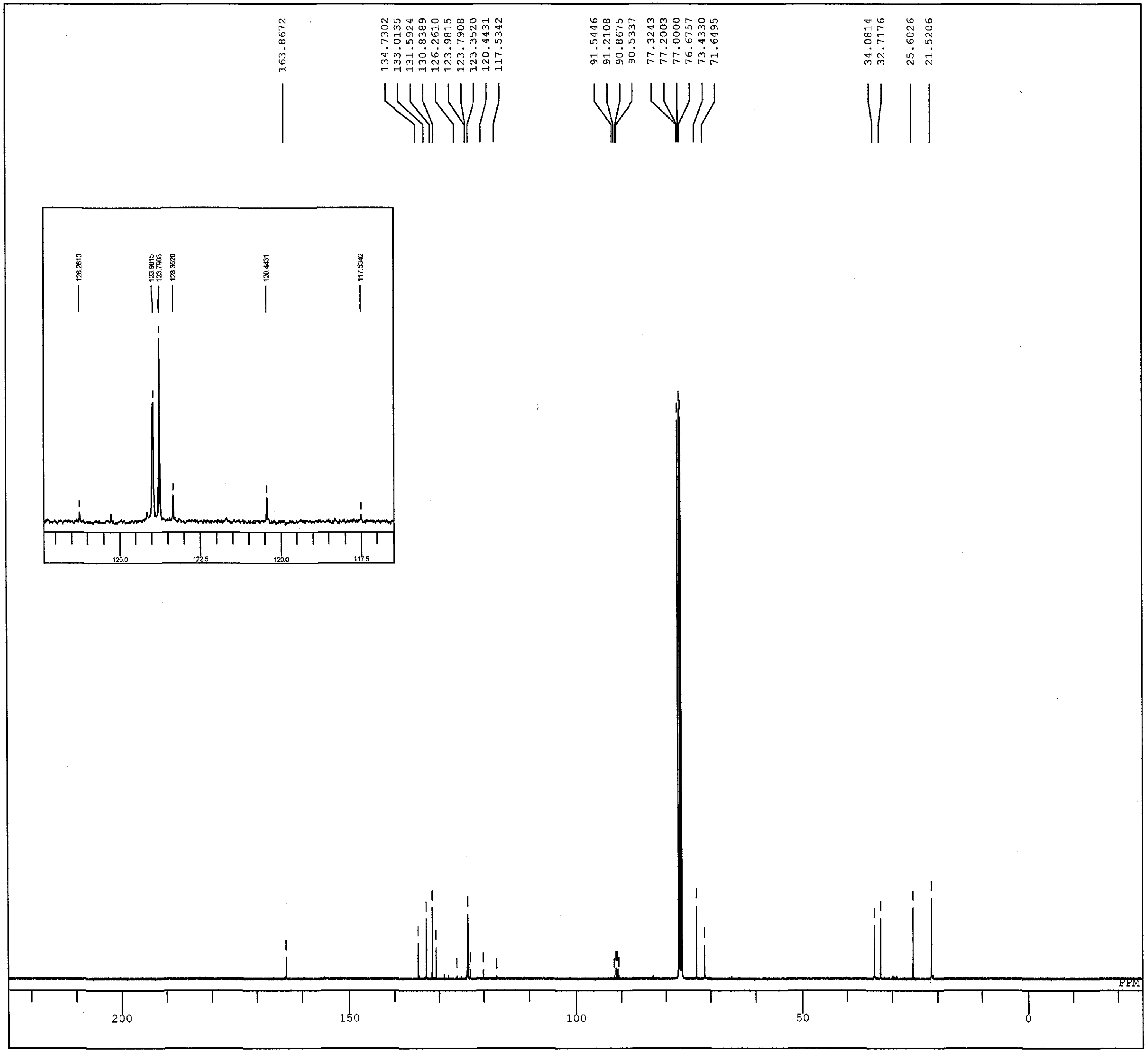

DFILE ozawa04-126_13C.jdf
COMNT CyMeOH, [O]
DATIM 07-03-2014 23:10:53
OBNUC 13C
EXMOD carbon.jxp
OBFRQ 98.52 MHz
OBSET 4.64 KHz
OBFIN 8.74 Hz
POINT 32767
FREQU 30788.18 Hz
SCANS 10334
ACQTM 1.0643 sec
PD 3.0000 sec
PW1 3.00 usec
IRNUC 1H
CTEMP 21.2 c
SLVNT CDCL3
EXREF 77.00 ppm
BF 1.20 Hz
RGAIN 60

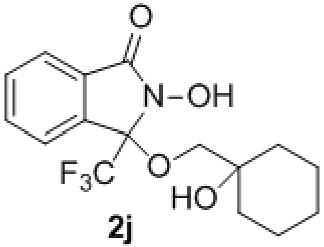

2j

CyMeOH, [O]

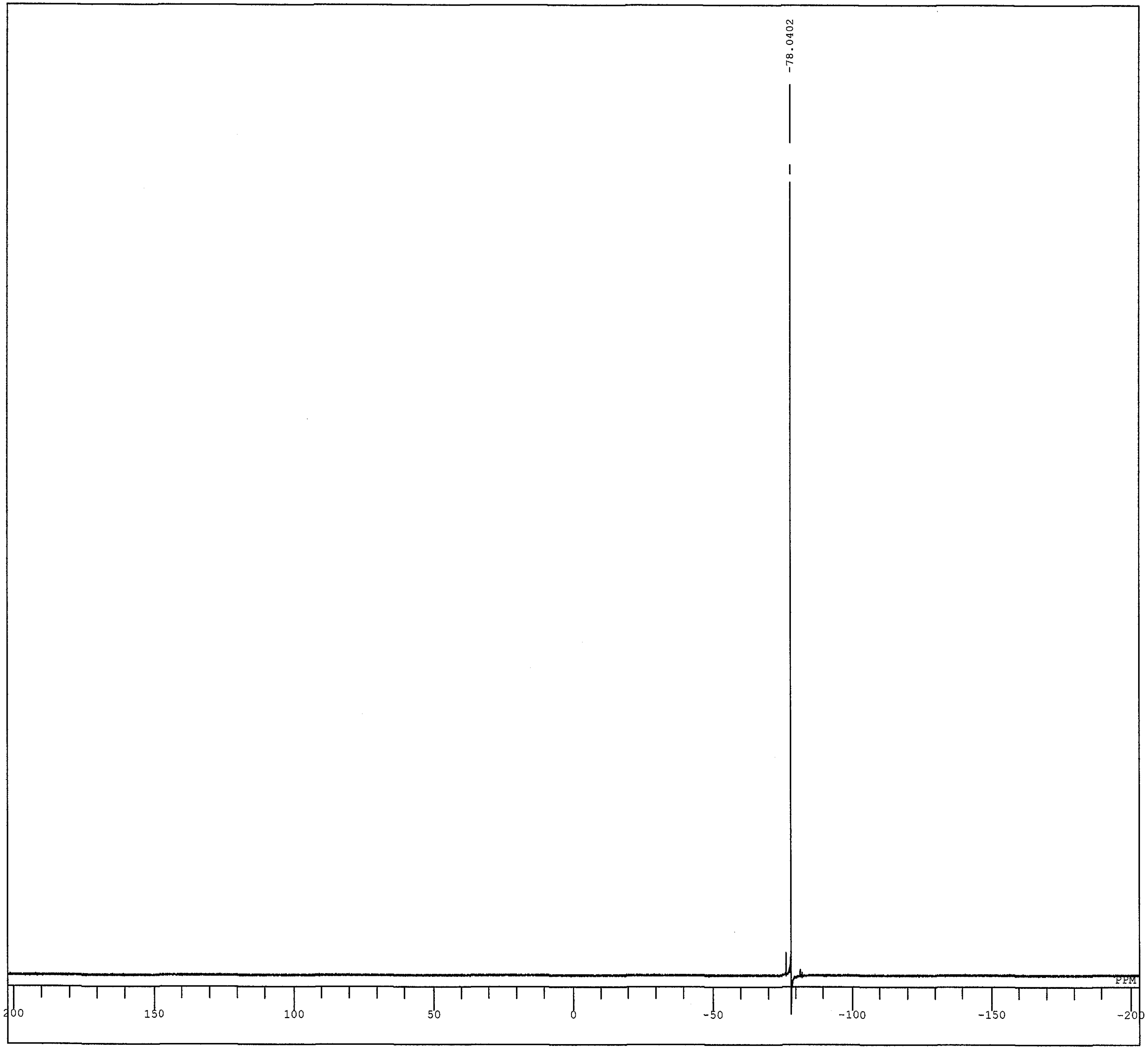

DFILE ozawa04-126_19F.jdf
COMNT CyMeOH, [O]
DATIM 07-03-2014 17:36:34
OBNUC 19F
EXMOD proton.jxp
OBFRQ 368.64 MHz
OBSET 7.63 KHz
OBFIN 2.85 Hz
POINT 16384
FREQU 186567.17 Hz
SCANS 4
ACQTM 0.0878 sec
PD 5.0000 sec
PW1 3.90 usec
IRNUC 19F
CTEMP 21.3 c
SLVNT CDCL3
EXREF -164.90 ppm
BF 0.12 Hz
RGAIN 46

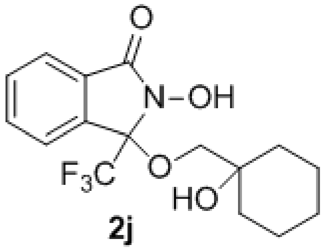

2j

menthol, LP, Bn

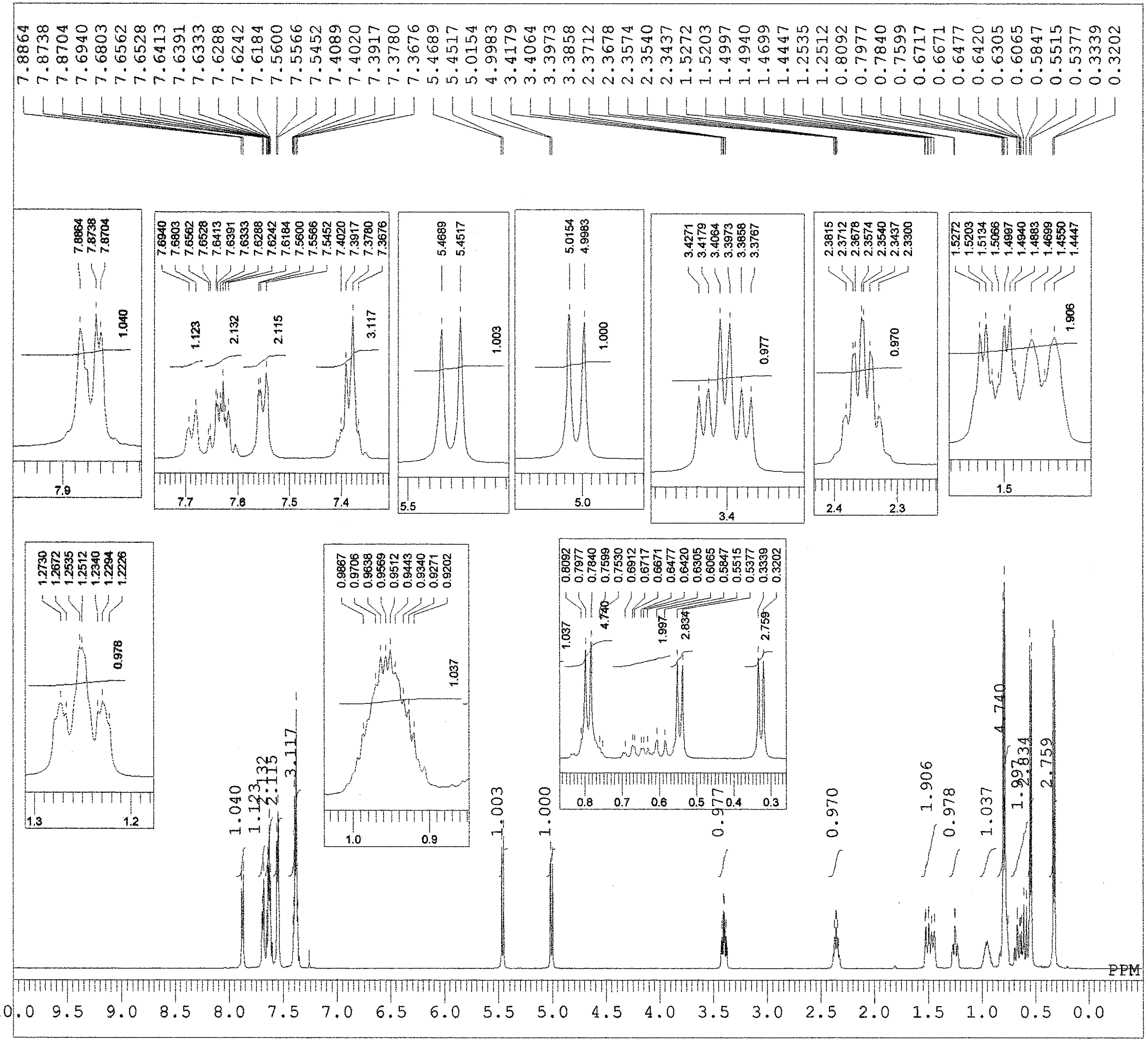

DFILE	ozawa04-036_LP_1H.jdf
COMNT	menthol, LP, Bn
DATIM	2014-01-30 22:31:28
OBNUC	1H
EXMOD	proton.jxp
OBFRQ	500.16 MHz
OBSET	2.41 KHz
OBFIN	6.01 Hz
POINT	16384
FREQU	9384.38 Hz
SCANS	4
ACQTM	1.7459 sec
PD	5.0000 sec
PW1	5.55 usec
IRNUC	1H
CTEMP	21.4 c
SLVNT	CDCL3
EXREF	7.26 ppm
BF	1.00 Hz
RGAIN	24

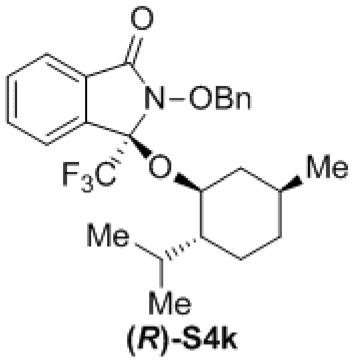

(*R*)-S4k

menthol, LP, Bn

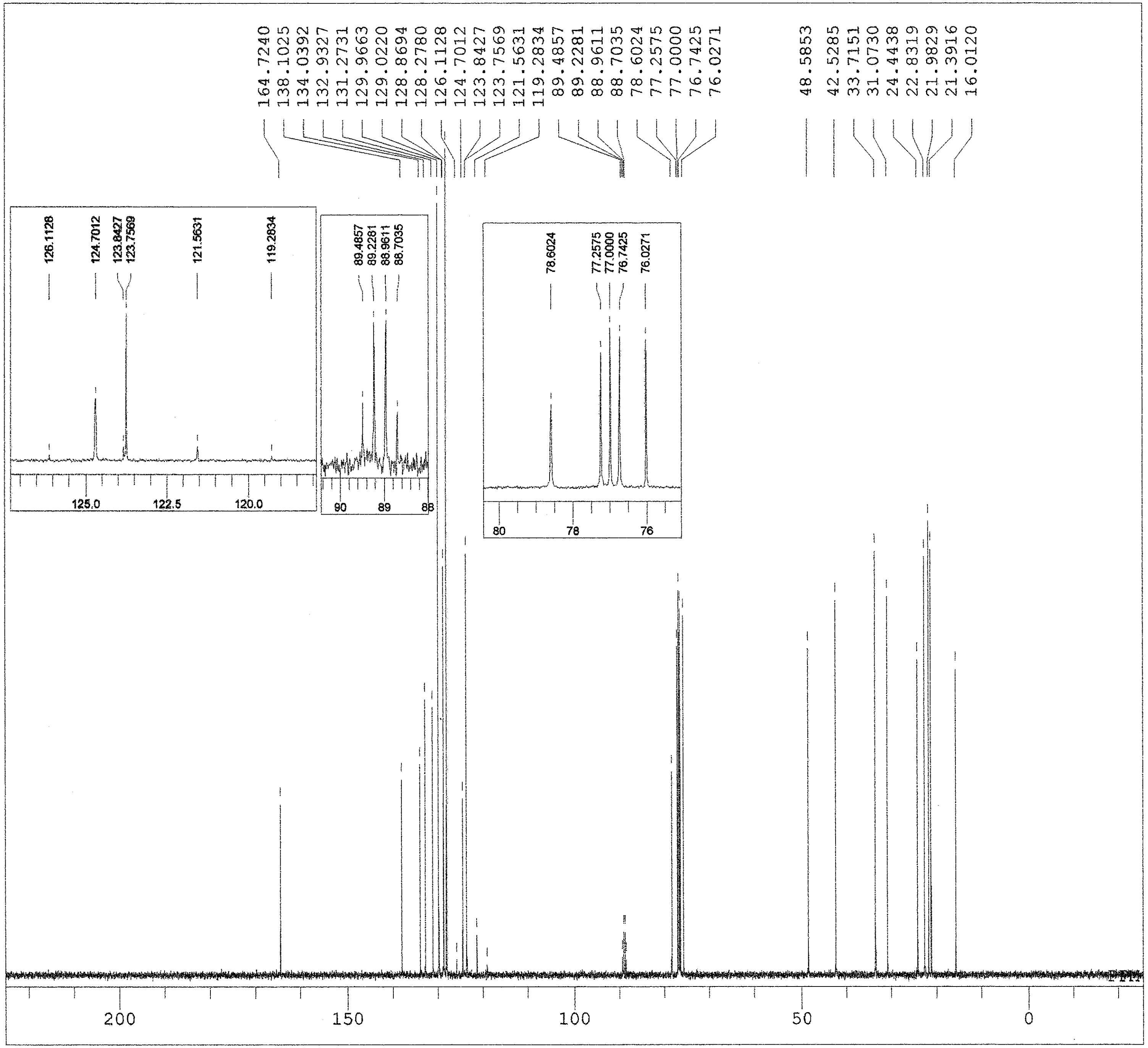

DFILE ozawa04-036_LP_13C.jdf
COMNT menthol, LP, Bn
DATIM 2014-01-30 22:32:32
OBNUC 13C
EXMOD carbon.jxp
OBFRQ 125.77 MHz
OBSET 7.87 KHz
OBFIN 4.21 Hz
POINT 32767
FREQU 39308.18 Hz
SCANS 153
ACQTM 0.8336 sec
PD 3.0000 sec
PW1 3.40 usec
IRNUC 1H
CTEMP 21.7 c
SLVNT CDCL3
EXREF 77.00 ppm
BF 1.00 Hz
RGAIN 60

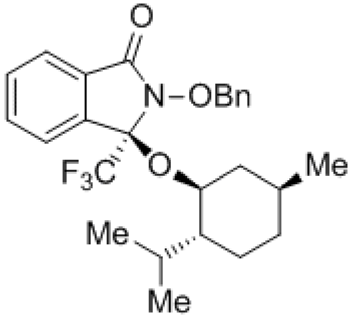

(*R*)-S4k

menthol, LP, Bn

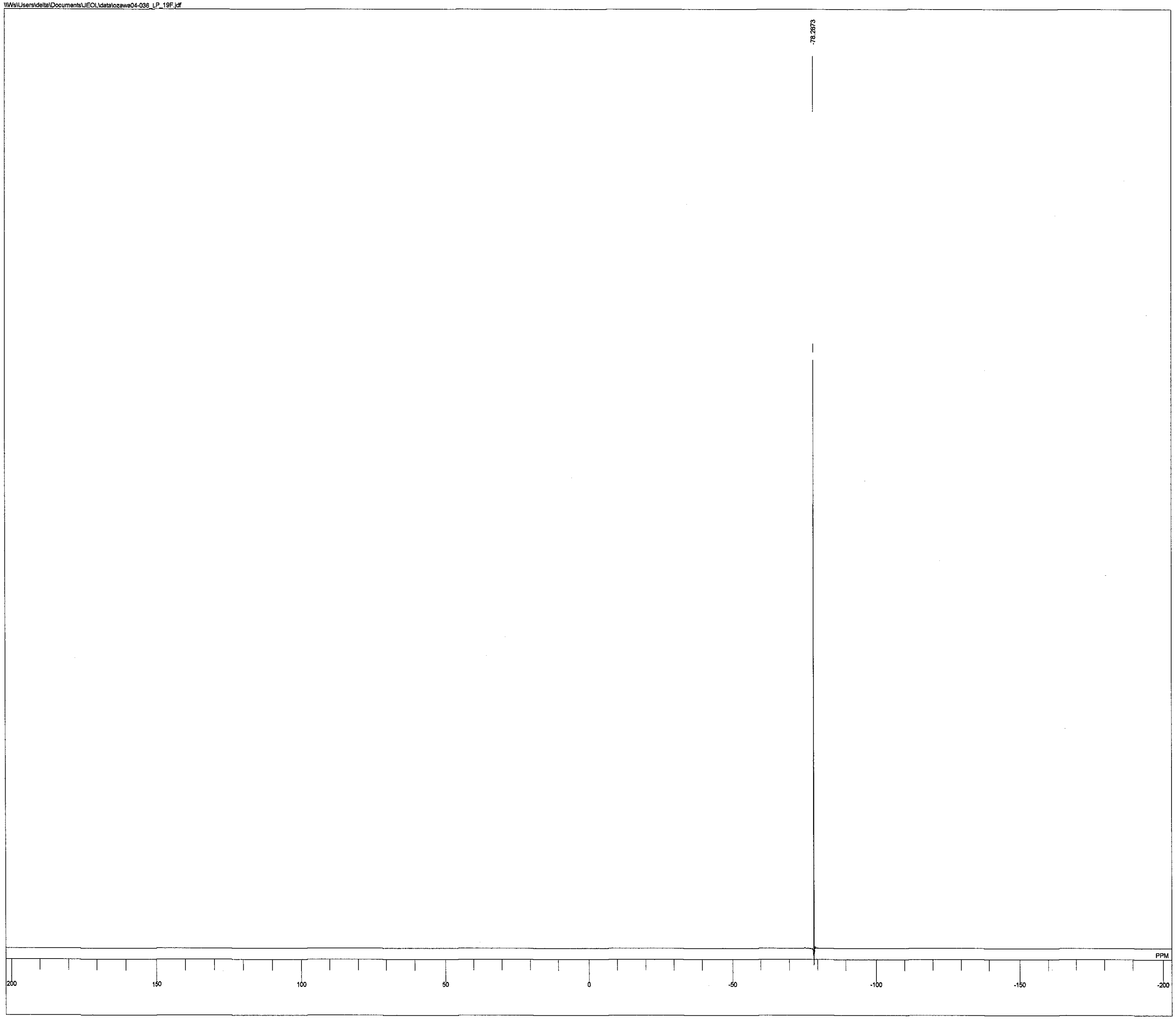

DFILE	ozawa04-036_LP_19F.jdf
COMNT	menthol, LP, Bn
DATIM	30-01-2014 23:00:05
OBNUC	19F
EXMOD	proton.jxp
OBFRQ	368.64 MHz
OBSET	7.63 KHz
OBFIN	2.85 Hz
POINT	16384
FREQU	186567.17 Hz
SCANS	4
ACQTM	0.0878 sec
PD	5.0000 sec
PW1	3.90 usec
IRNUC	19F
CTEMP	21.1 c
SLVNT	CDCL3
EXREF	-164.90 ppm
BF	0.12 Hz
RGAIN	44

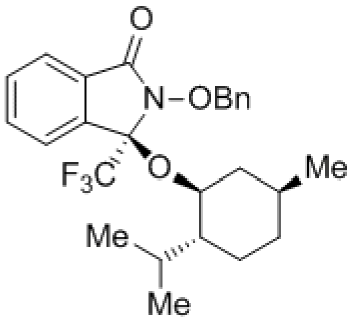

(*R*)-S4k

menthol, OH, LP

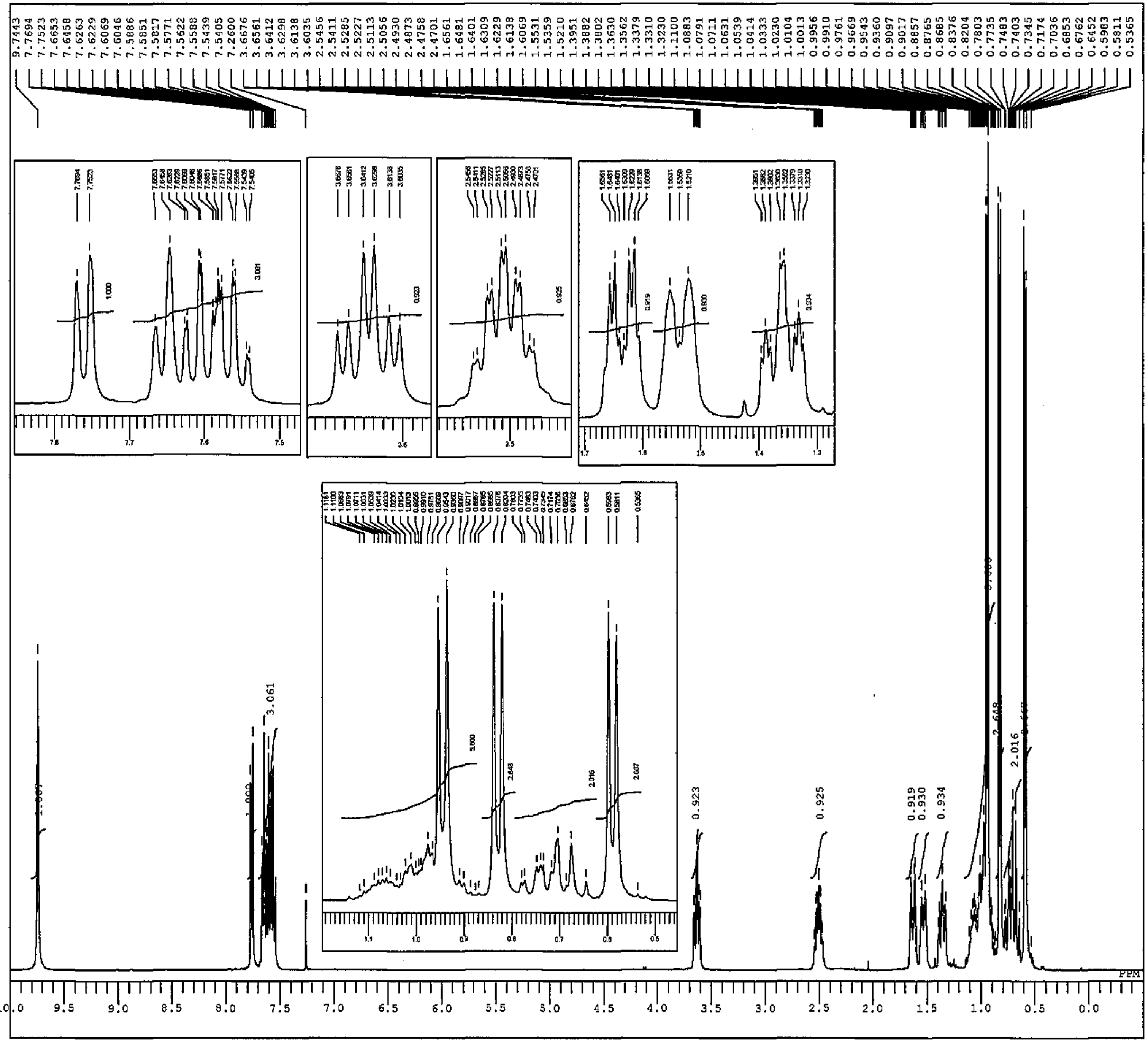

```
DFILE  ozawa04-016_1_1H.jdf
COMNT  menthol, OH, LP
DATIM  06-02-2014 16:11:14
OBNUC  1H
EXMOD  proton.jxp
OBFRQ      391.78 MHz
OBSET        8.51 KHz
OBFIN        3.34 Hz
POINT       16384
FREQU     7348.62 Hz
SCANS           4
ACQTM      2.2295 sec
PD         5.0000 sec
PW1          5.25 usec
IRNUC  1H
CTEMP        21.1 c
SLVNT  CDCL3
EXREF        7.26 ppm
BF           0.12 Hz
RGAIN          24
```

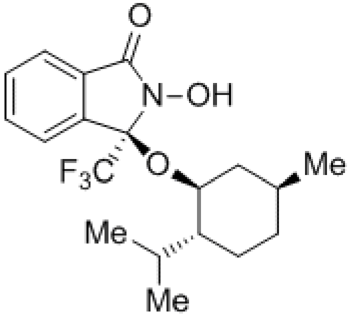

(*R*)-1k

menthol, OH, LP

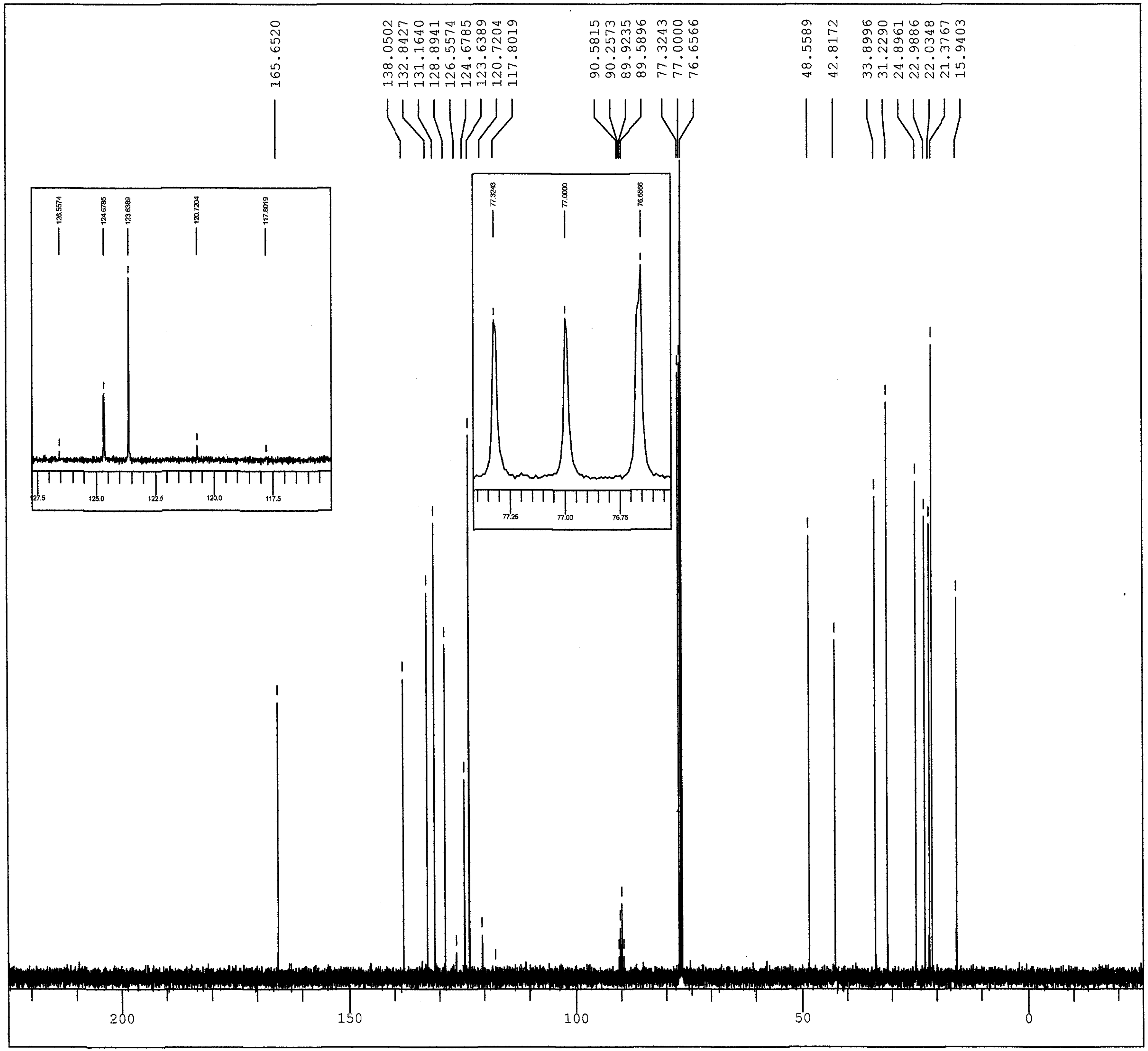

DFILE 1ma_ozawa04-016_1_13C.als
COMNT menthol, OH, LP
DATIM 06-02-2014 16:12:38
OBNUC 13C
EXMOD carbon.jxp
OBFRQ 98.52 MHz
OBSET 4.64 KHz
OBFIN 8.74 Hz
POINT 26214
FREQU 24630.54 Hz
SCANS 160
ACQTM 1.0643 sec
PD 3.0000 sec
PW1 3.00 usec
IRNUC 1H
CTEMP 21.5 c
SLVNT CDCL3
EXREF 77.00 ppm
BF 0.12 Hz
RGAIN 60

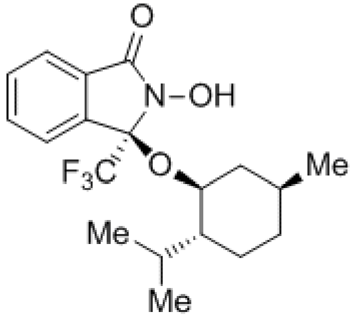

(*R*)-1k

menthol, OH, LP

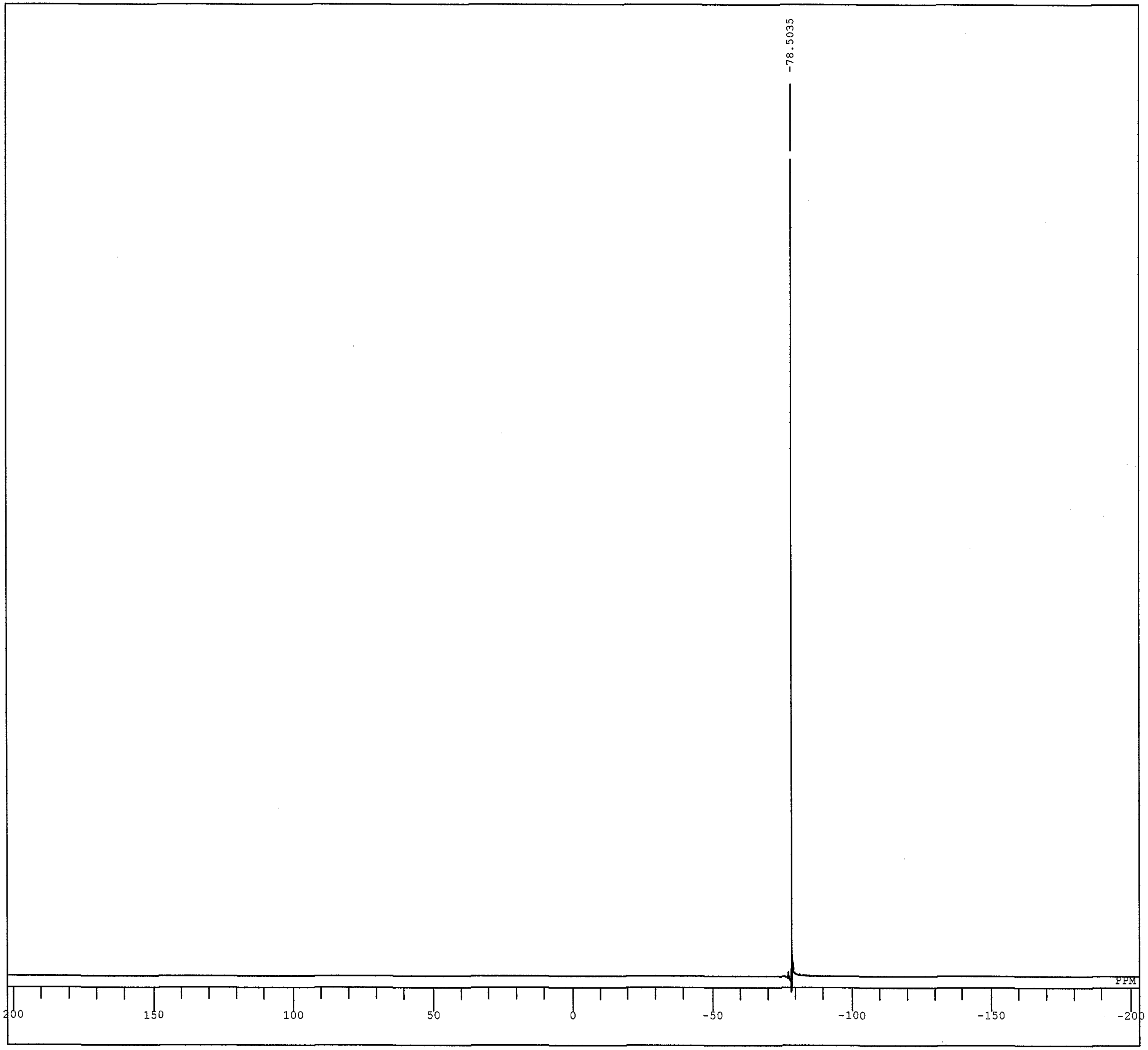

DFILE	ozawa04-016_1_19F.jdf
COMNT	menthol, OH, LP
DATIM	06-02-2014 16:08:51
OBNUC	19F
EXMOD	proton.jxp
OBFRQ	368.64 MHz
OBSET	7.63 KHz
OBFIN	2.85 Hz
POINT	16384
FREQU	186567.17 Hz
SCANS	4
ACQTM	0.0878 sec
PD	5.0000 sec
PW1	3.90 usec
IRNUC	19F
CTEMP	21.0 c
SLVNT	CDCL3
EXREF	-164.90 ppm
BF	0.12 Hz
RGAIN	44

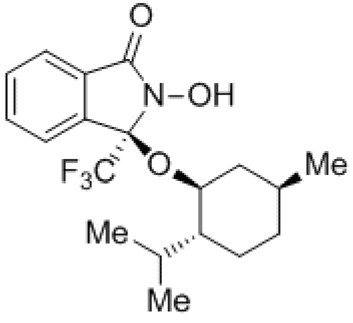

(*R*)-1k

menthol, [O], LP

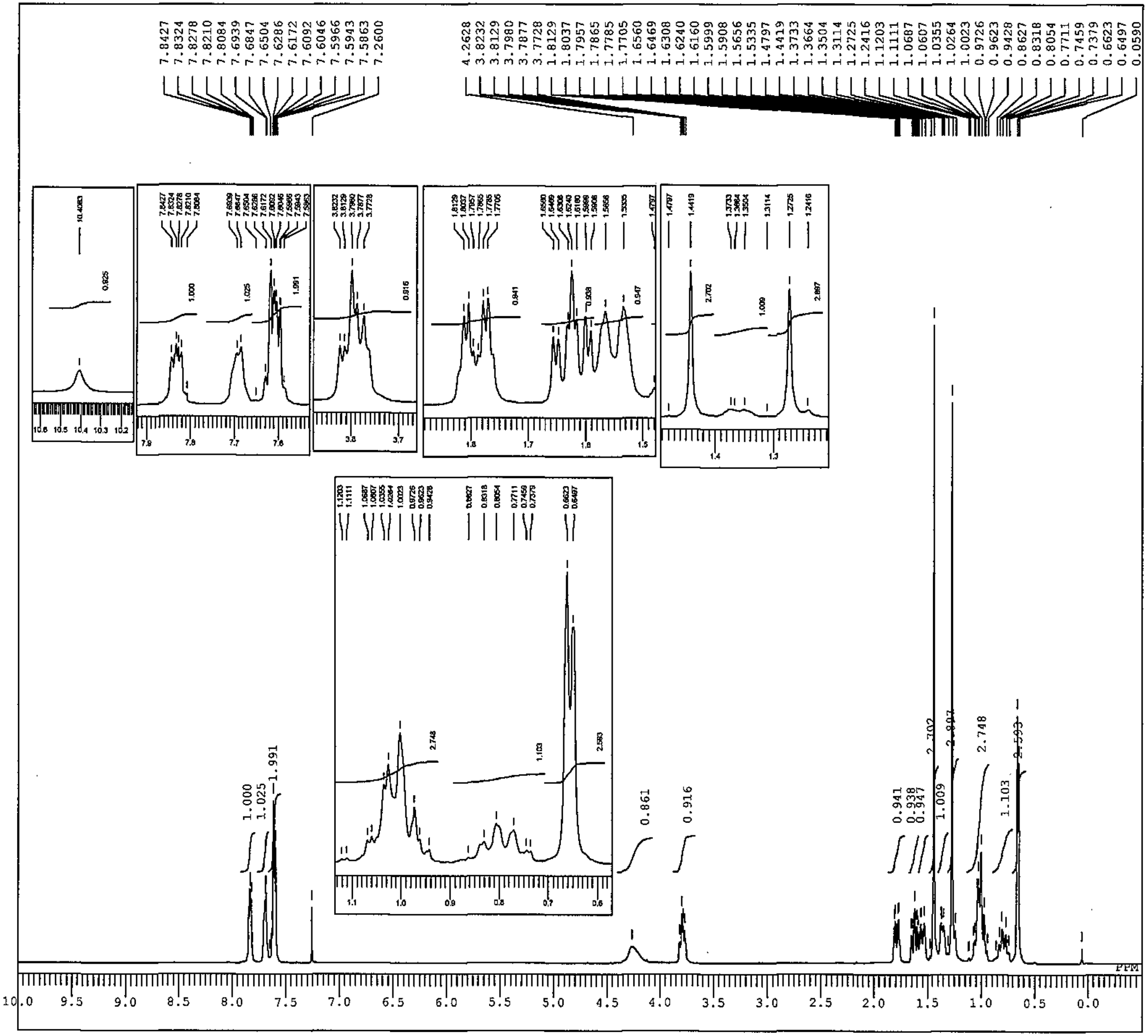

DFILE ozawa04-035_2_1H.als
COMNT menthol, [O], LP
DATIM 07-02-2014 16:39:44
OBNUC 1H
EXMOD proton.jxp
OBFRQ 391.78 MHz
OBSET 8.51 KHz
OBFIN 3.34 Hz
POINT 13107
FREQU 5878.90 Hz
SCANS 4
ACQTM 2.2295 sec
PD 5.0000 sec
PW1 5.25 usec
IRNUC 1H
CTEMP 21.0 c
SLVNT CDCL3
EXREF 7.26 ppm
BF 0.12 Hz
RGAIN 30

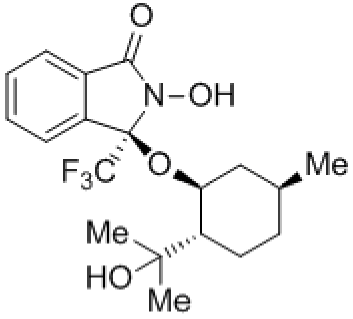

(*R*)-2k

menthol, [O], LP

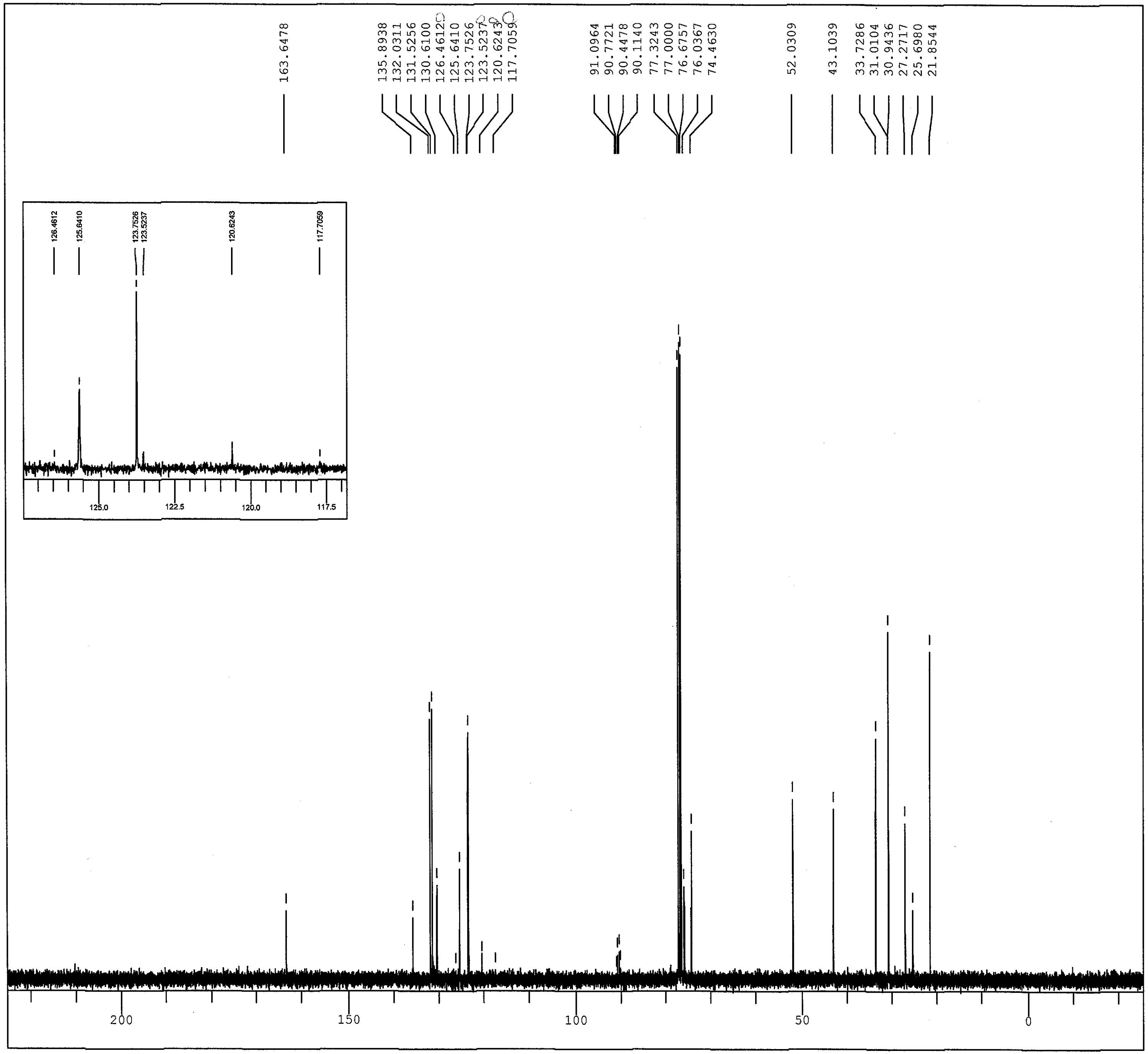


```
DFILE    ozawa04-035_2_13C.jdf
COMNT    menthol, [O], LP
DATIM    07-02-2014 16:41:07
OBNUC    13C
EXMOD    carbon.jxp
OBFRQ         98.52 MHz
OBSET          4.64 KHz
OBFIN          8.74 Hz
POINT         32767
FREQU      30788.18 Hz
SCANS           260
ACQTM        1.0643 sec
PD           3.0000 sec
PW1            3.00 usec
IRNUC    1H
CTEMP          21.6 c
SLVNT    CDCL3
EXREF         77.00 ppm
BF             0.12 Hz
RGAIN            60
```

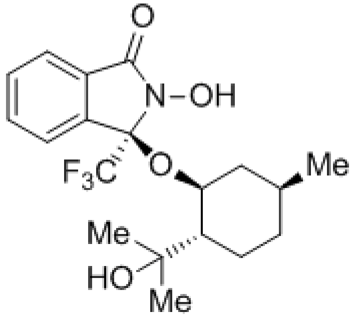

(*R*)-2k

menthol, [O], LP

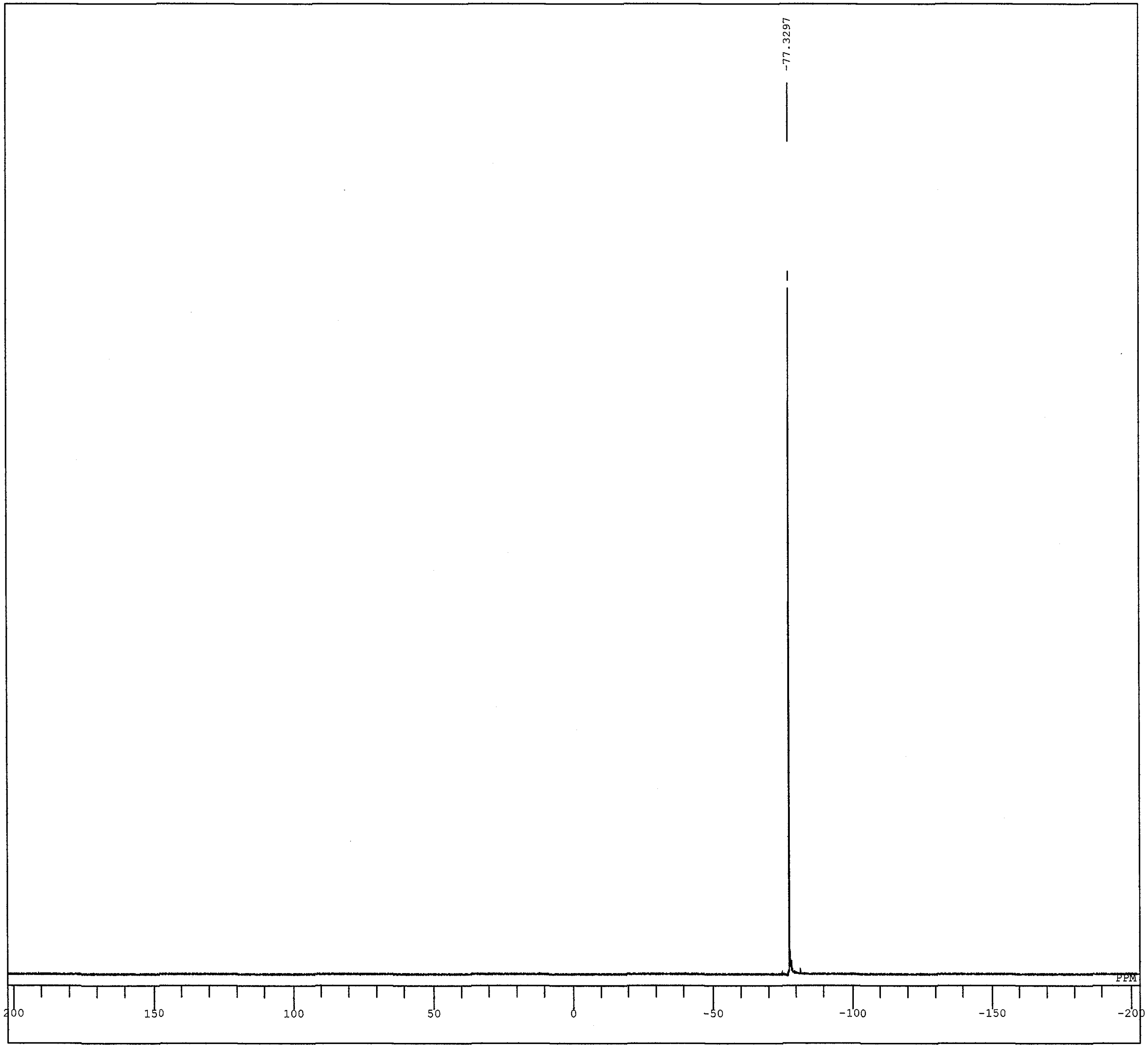

DFILE ozawa04-124_1_19F.jdf
COMNT menthol, [O], LP
DATIM 06-02-2014 19:56:06
OBNUC 19F
EXMOD proton.jxp
OBFRQ 368.64 MHz
OBSET 7.63 KHz
OBFIN 2.85 Hz
POINT 16384
FREQU 186567.17 Hz
SCANS 4
ACQTM 0.0878 sec
PD 5.0000 sec
PW1 3.90 usec
IRNUC 19F
CTEMP 21.3 c
SLVNT CDCL3
EXREF -164.90 ppm
BF 0.12 Hz
RGAIN 48

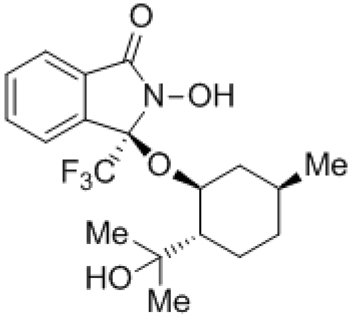

(*R*)-2k

citronellol, Bn

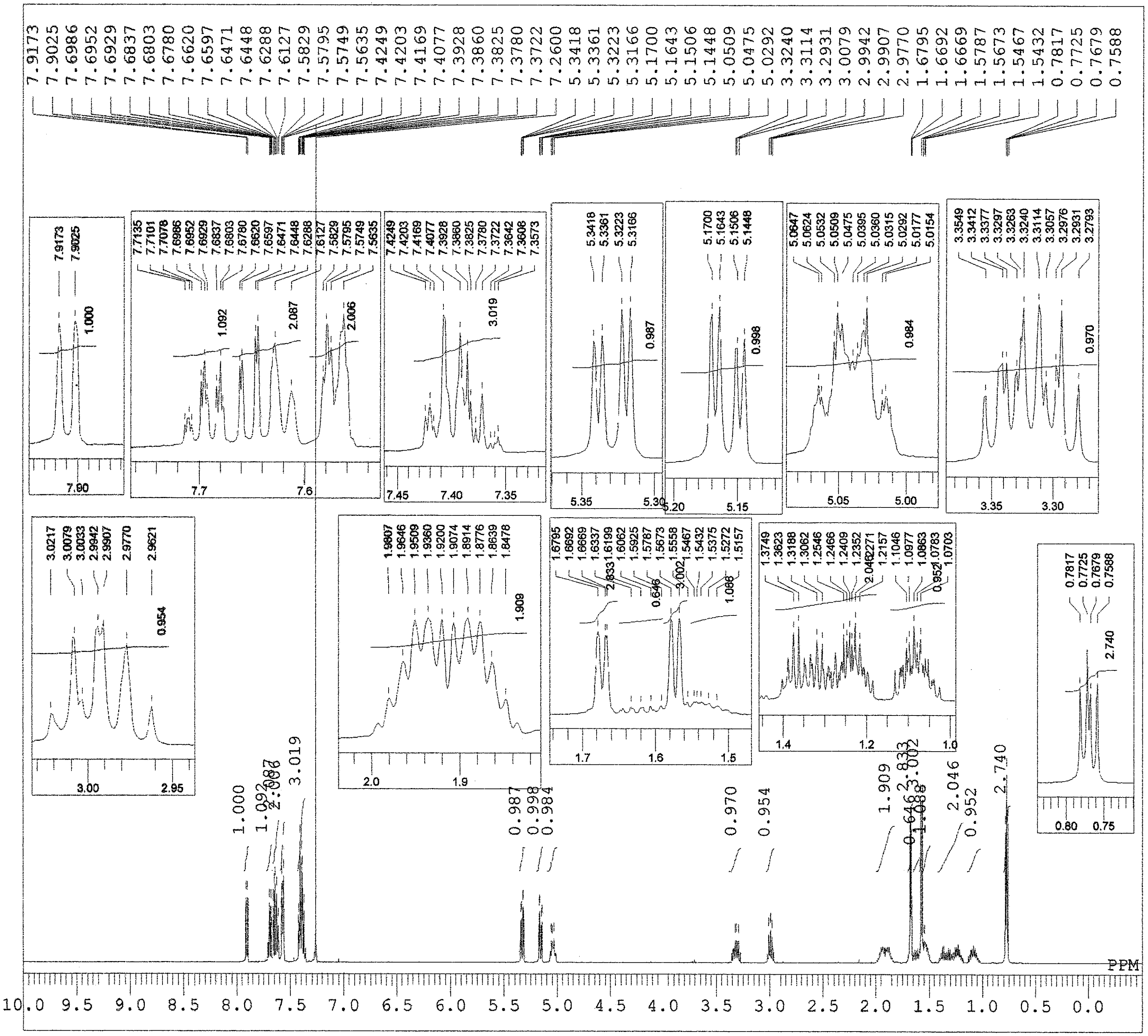

DFILE ozawa04-013_1H.jdf
COMNT citronellol, Bn
DATIM 2014-01-16 21:35:28
OBNUC 1H
EXMOD proton.jxp
OBFRQ 500.16 MHz
OBSET 2.41 KHz
OBFIN 6.01 Hz
POINT 16384
FREQU 9384.38 Hz
SCANS 4
ACQTM 1.7459 sec
PD 5.0000 sec
PW1 5.55 usec
IRNUC 1H
CTEMP 21.2 c
SLVNT CDCL3
EXREF 7.26 ppm
BF 0.12 Hz
RGAIN 24

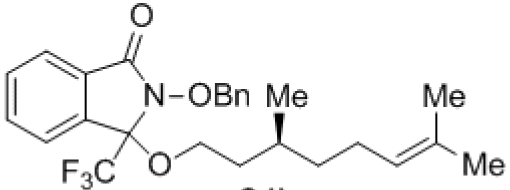

S4I

citronellol, Bn

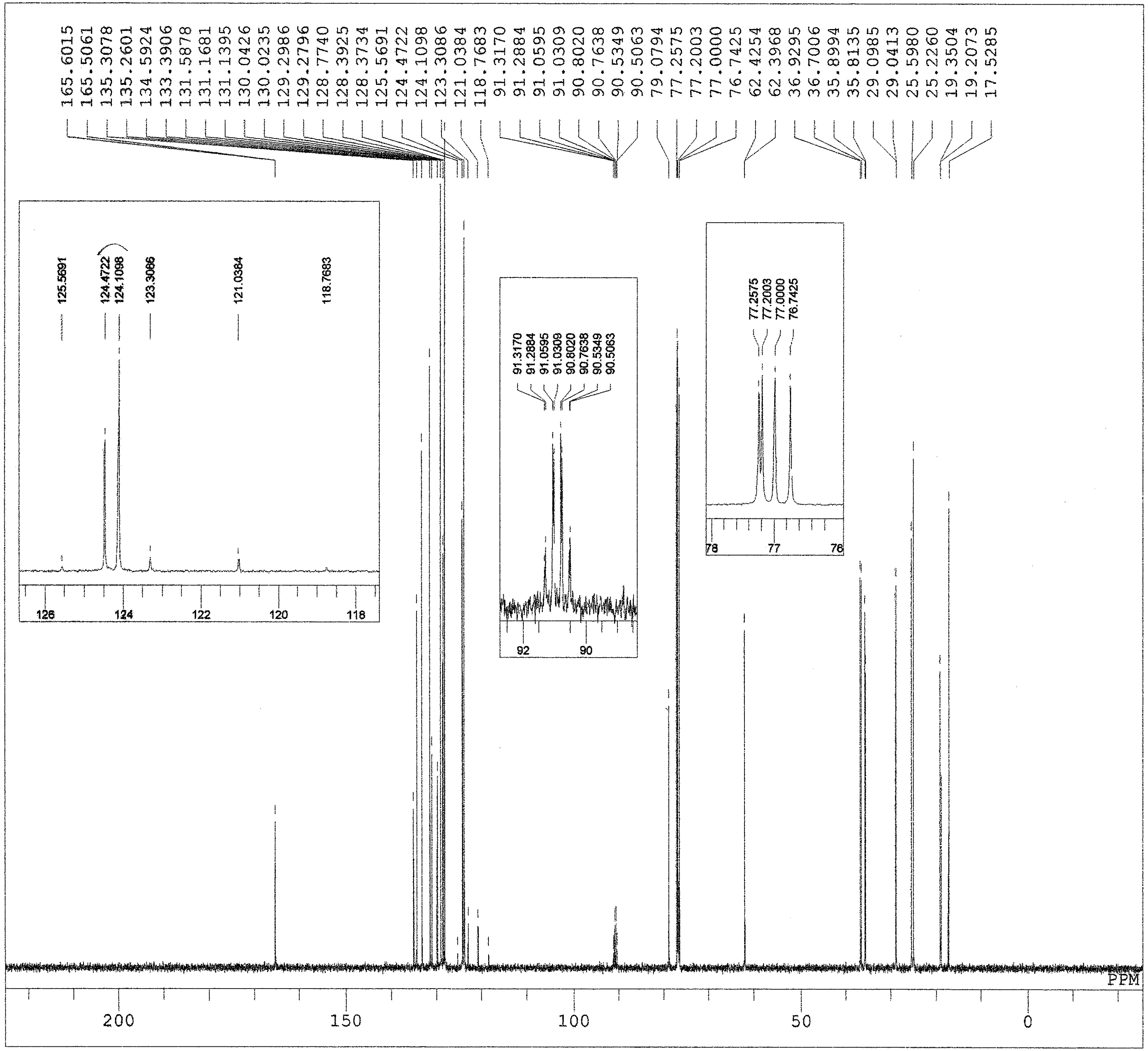

DFILE ozawa04-013_13C.jdf
COMNT citronellol, Bn
DATIM 2014-01-16 21:36:30
OBNUC 13C
EXMOD carbon.jxp
OBFRQ 125.77 MHz
OBSET 7.87 KHz
OBFIN 4.21 Hz
POINT 32767
FREQU 39308.18 Hz
SCANS 352
ACQTM 0.8336 sec
PD 3.0000 sec
PW1 3.40 usec
IRNUC 1H
CTEMP 21.4 c
SLVNT CDCL3
EXREF 77.00 ppm
BF 1.00 Hz
RGAIN 60

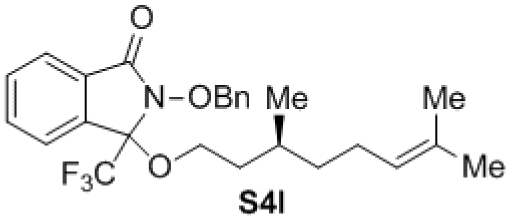

S4l

citronellol, Bn

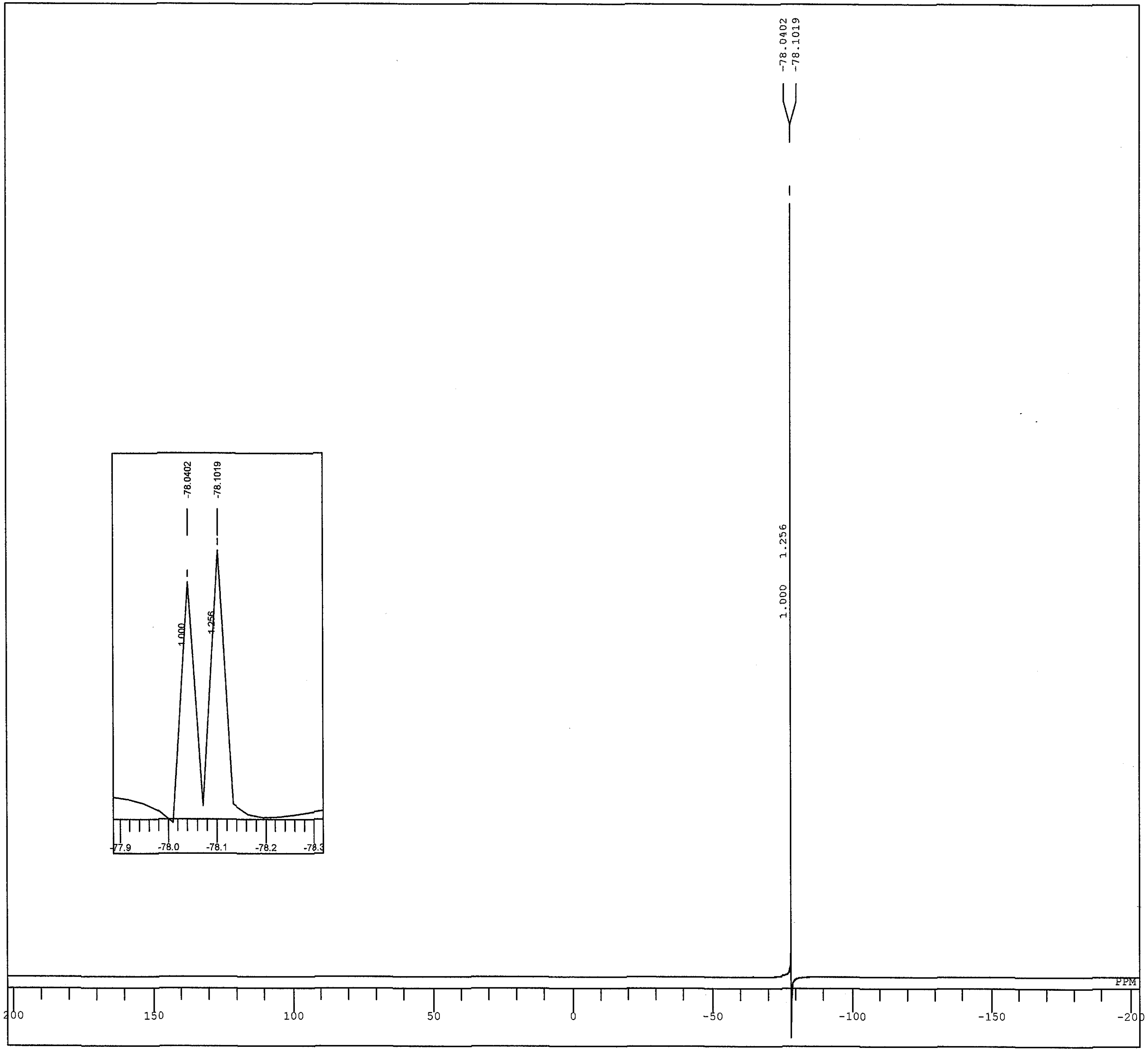

DFILE ozawa04-013_19F.jdf
COMNT citronellol, Bn
DATIM 16-01-2014 22:31:18
OBNUC 19F
EXMOD proton.jxp
OBFRQ 368.64 MHz
OBSET 7.63 KHz
OBFIN 2.85 Hz
POINT 16384
FREQU 186567.17 Hz
SCANS 4
ACQTM 0.0878 sec
PD 5.0000 sec
PW1 3.90 usec
IRNUC 19F
CTEMP 21.2 c
SLVNT CDCL3
EXREF -164.90 ppm
BF 0.12 Hz
RGAIN 44

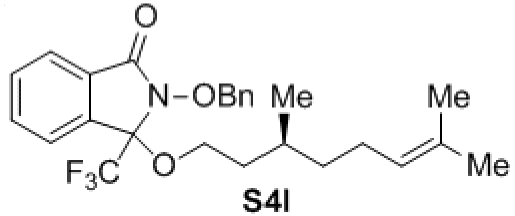

S4l

citr., OH

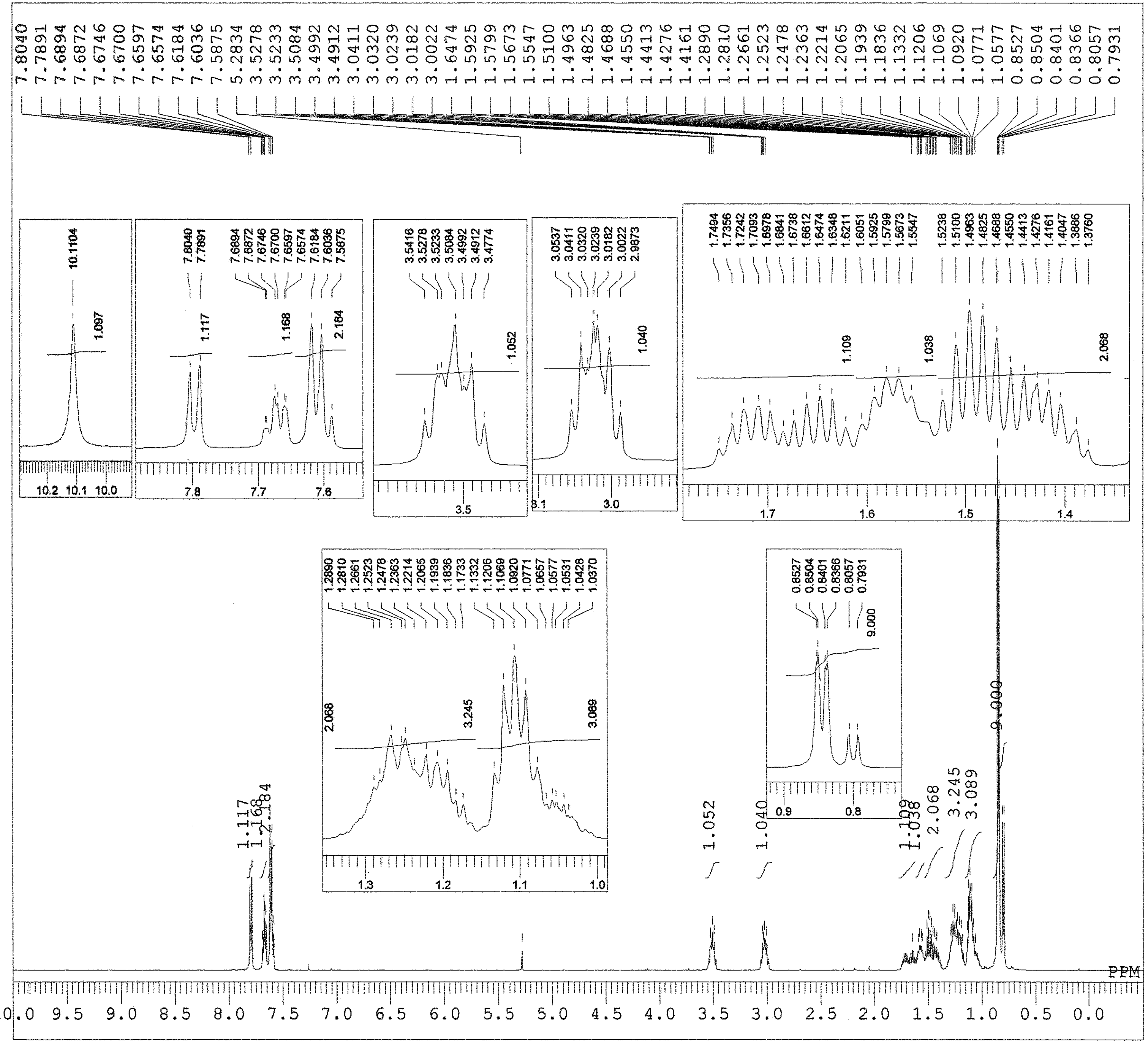

DFILE	ozawa04-017_1H.jdf
COMNT	citr., OH
DATIM	2014-01-25 17:42:01
OBNUC	1H
EXMOD	proton.jxp
OBFRQ	500.16 MHz
OBSET	2.41 KHz
OBFIN	6.01 Hz
POINT	16384
FREQU	9384.38 Hz
SCANS	4
ACQTM	1.7459 sec
PD	5.0000 sec
PW1	5.55 usec
IRNUC	1H
CTEMP	21.5 c
SLVNT	CDCL3
EXREF	7.26 ppm
BF	1.00 Hz
RGAIN	20

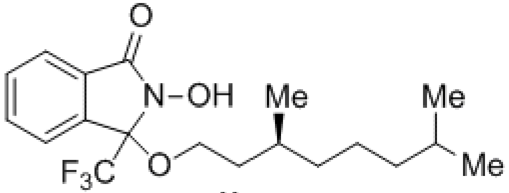

1l

citr., OH

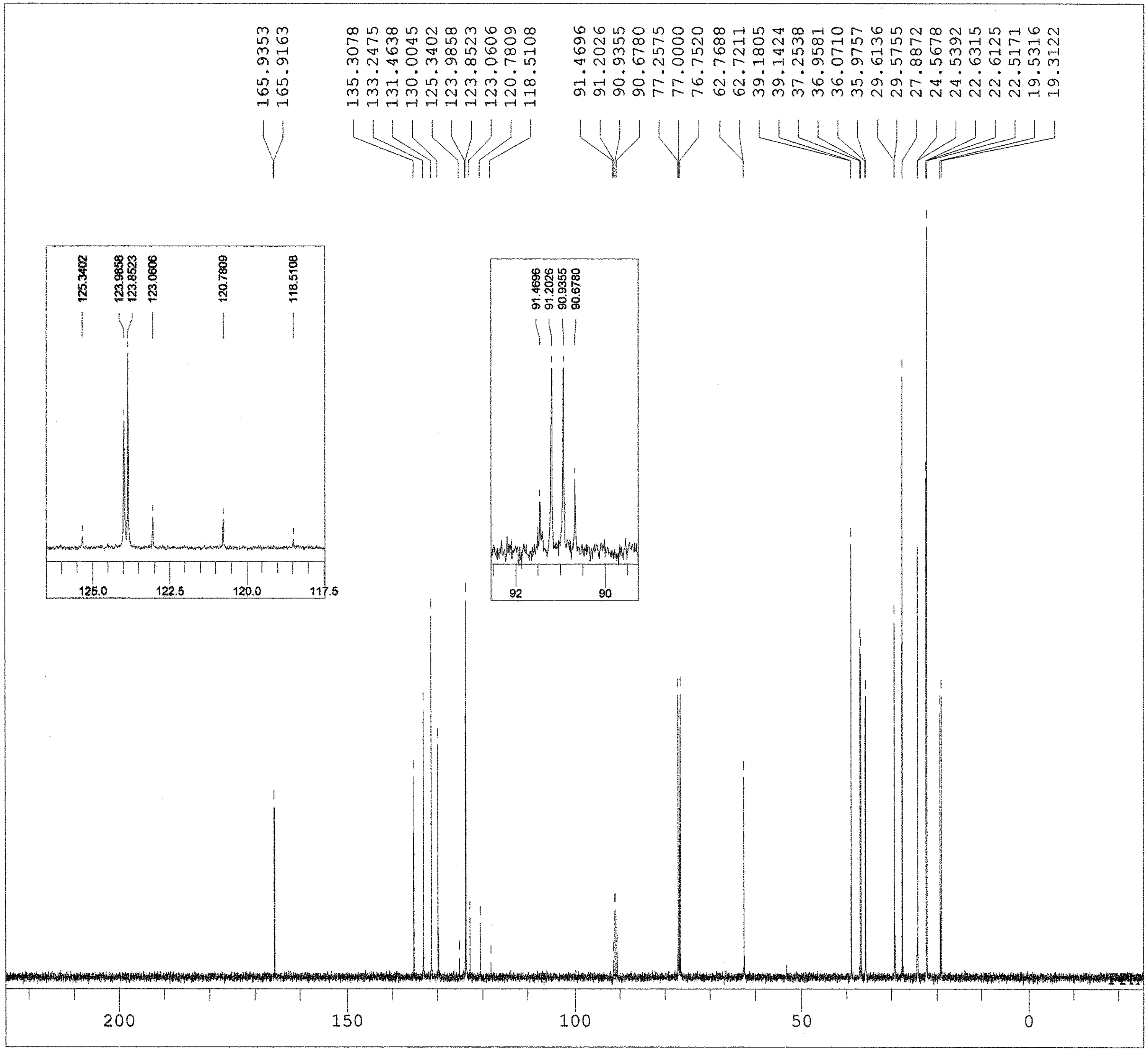

DFILE ozawa04-017_13C.jdf
COMNT citr., OH
DATIM 2014-01-25 17:43:04
OBNUC 13C
EXMOD carbon.jxp
OBFRQ 125.77 MHz
OBSET 7.87 KHz
OBFIN 4.21 Hz
POINT 32767
FREQU 39308.18 Hz
SCANS 64
ACQTM 0.8336 sec
PD 3.0000 sec
PW1 3.40 usec
IRNUC 1H
CTEMP 21.9 c
SLVNT CDCL3
EXREF 77.00 ppm
BF 1.00 Hz
RGAIN 60

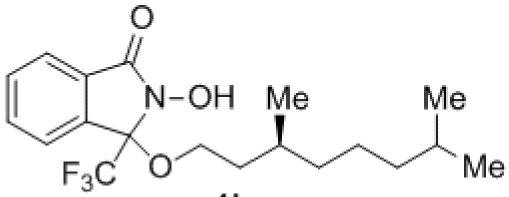

1l

citr., OH

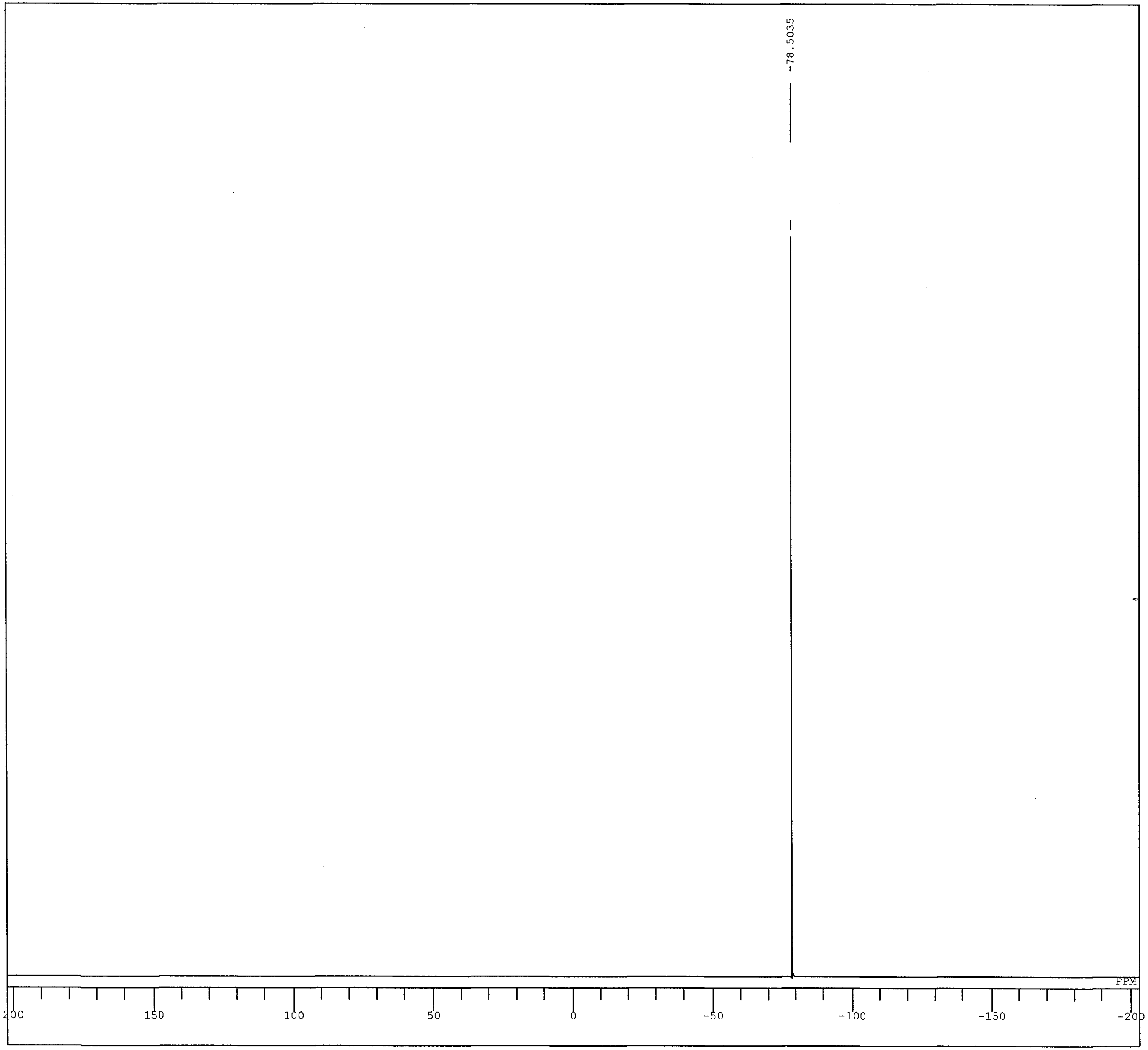

DFILE 1h_ozawa04-017_19F.jdf
COMNT citr., OH
DATIM 25-01-2014 18:16:27
OBNUC 19F
EXMOD proton.jxp
OBFRQ 368.64 MHz
OBSET 7.63 KHz
OBFIN 2.85 Hz
POINT 16384
FREQU 186567.17 Hz
SCANS 4
ACQTM 0.0878 sec
PD 5.0000 sec
PW1 3.90 usec
IRNUC 19F
CTEMP 20.9 c
SLVNT CDCL3
EXREF -164.90 ppm
BF 0.12 Hz
RGAIN 40

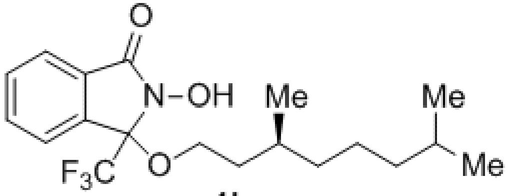

1l

PTLC, citr., 02

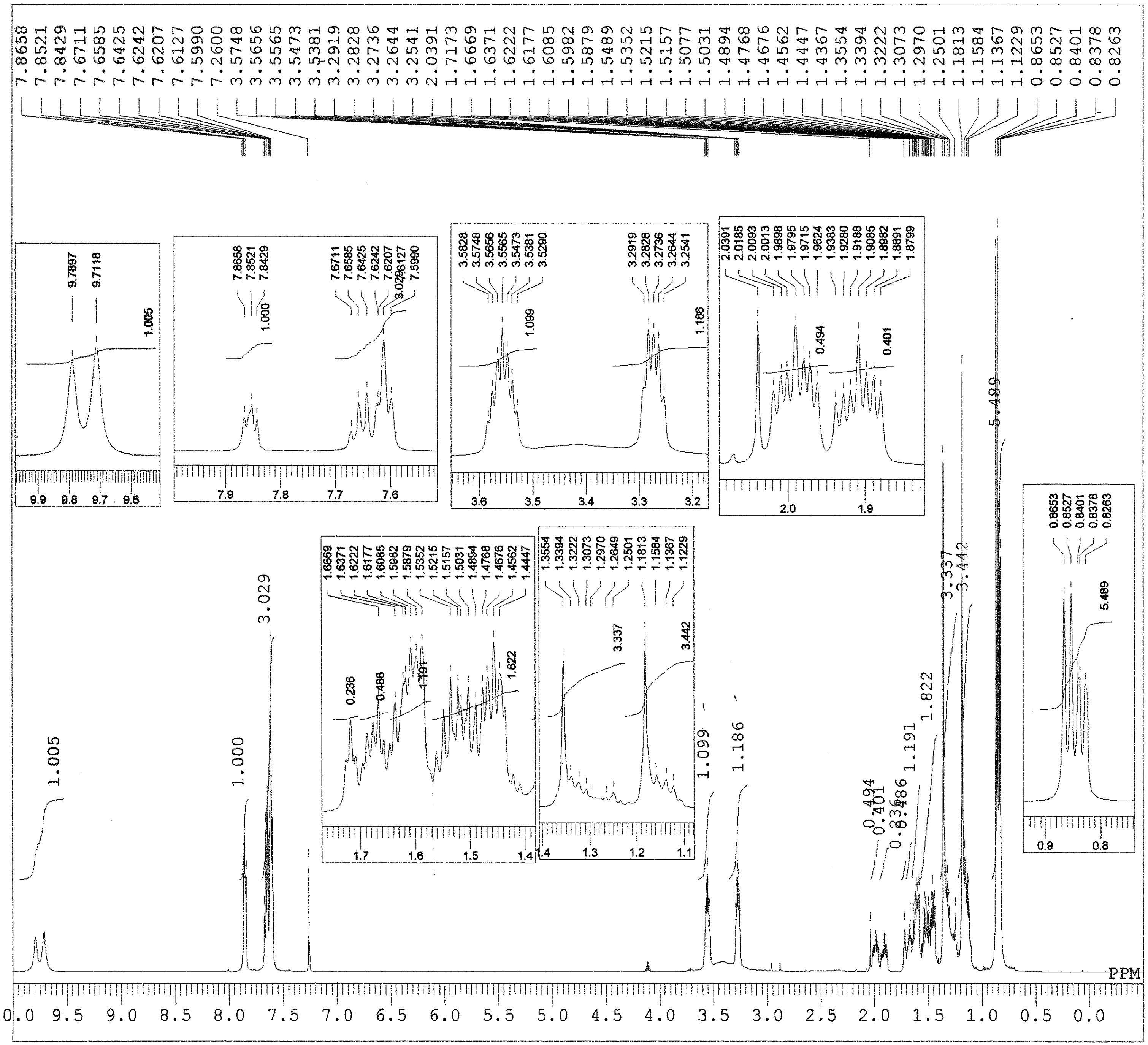

DFILE ozawa04-095_2a_1H.jdf
COMNT PTLC, citr., 02
DATIM 2014-01-20 17:38:20
OBNUC 1H
EXMOD proton.jxp
OBFRQ 500.16 MHz
OBSET 2.41 KHz
OBFIN 6.01 Hz
POINT 16384
FREQU 9384.38 Hz
SCANS 4
ACQTM 1.7459 sec
PD 5.0000 sec
PW1 5.55 usec
IRNUC 1H
CTEMP 21.0 c
SLVNT CDCL3
EXREF 7.26 ppm
BF 1.00 Hz
RGAIN 30

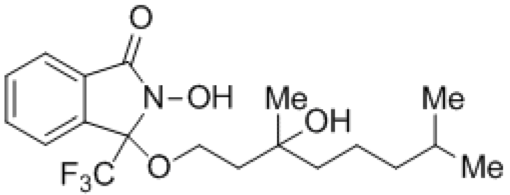

2l

citr., [O]

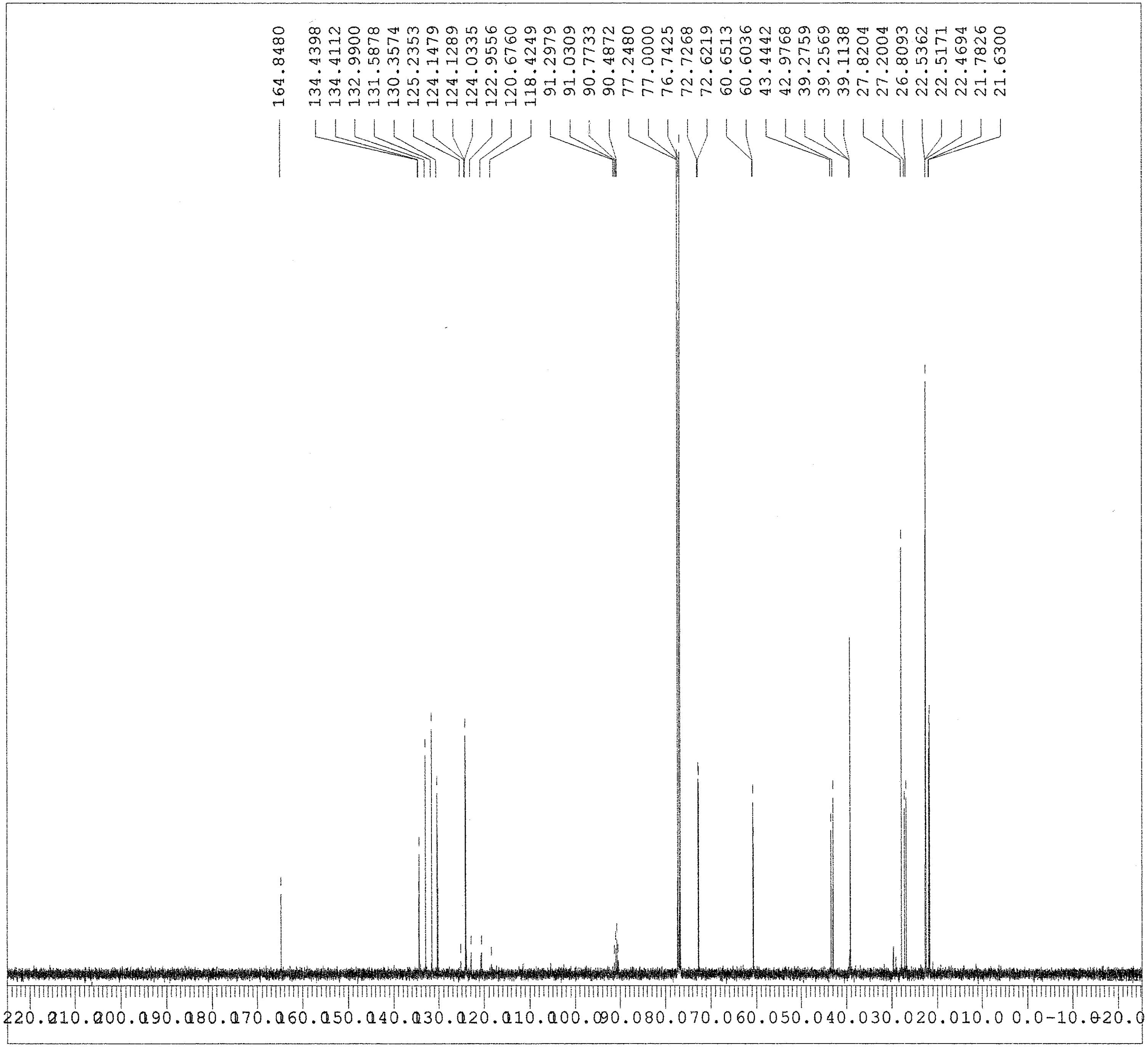

DFILE	ozawa04-095_2_13C.jdf
COMNT	citr., [O]
DATIM	2014-01-26 13:46:45
OBNUC	13C
EXMOD	carbon.jxp
OBFRQ	125.77 MHz
OBSET	7.87 KHz
OBFIN	4.21 Hz
POINT	32767
FREQU	39308.18 Hz
SCANS	464
ACQTM	0.8336 sec
PD	3.0000 sec
PW1	3.40 usec
IRNUC	1H
CTEMP	21.7 c
SLVNT	CDCL3
EXREF	77.00 ppm
BF	0.12 Hz
RGAIN	60

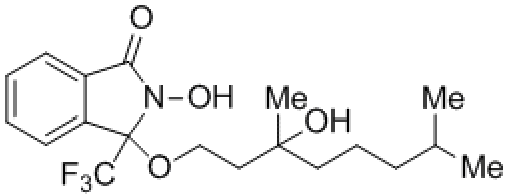

2l

citr-H2

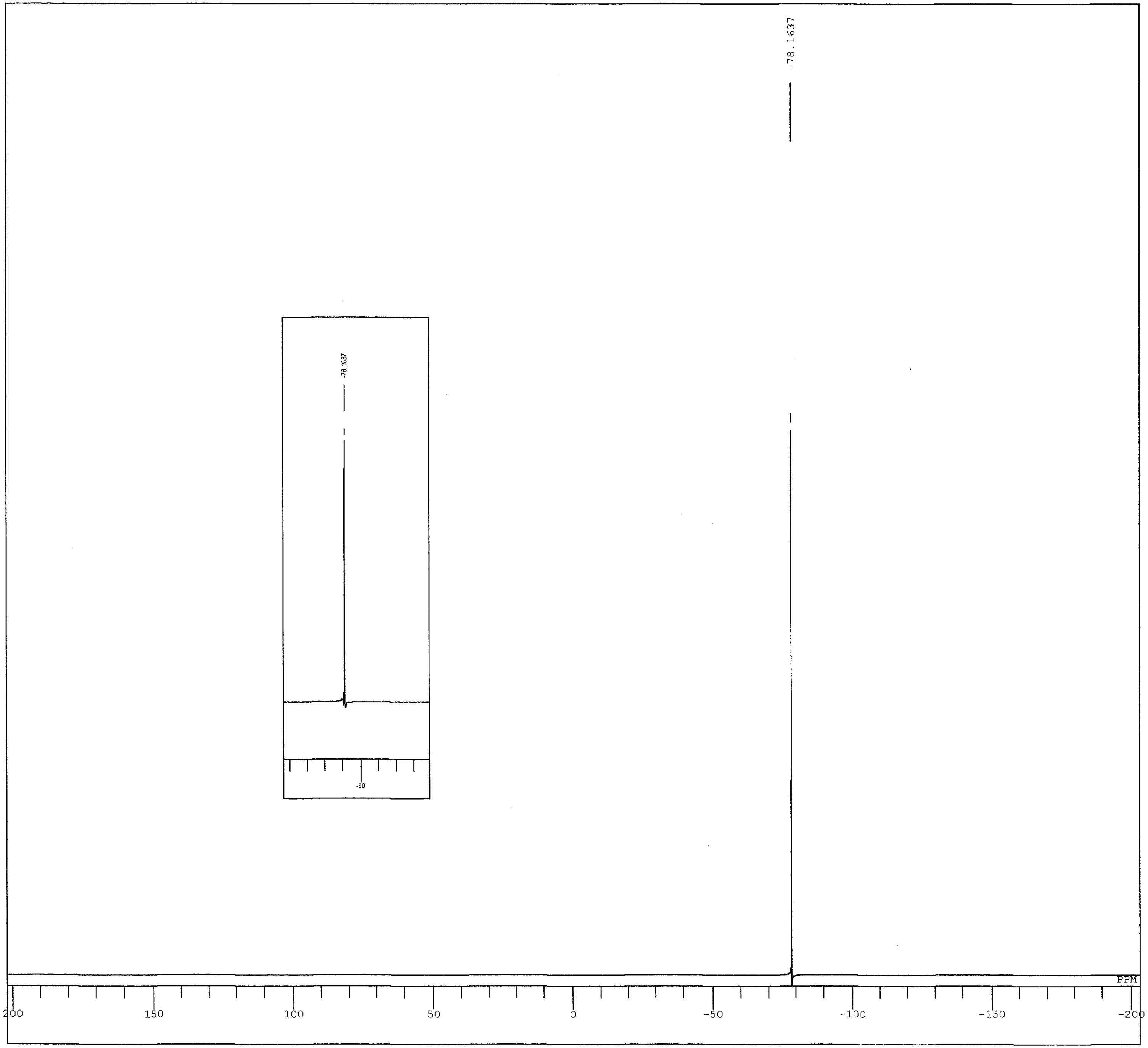

DFILE ozawa04-047_19F.jdf
COMNT citr-H2
DATIM 16-11-2013 13:37:01
OBNUC 19F
EXMOD proton.jxp
OBFRQ 368.64 MHz
OBSET 7.63 KHz
OBFIN 2.85 Hz
POINT 16384
FREQU 186567.17 Hz
SCANS 8
ACQTM 0.0878 sec
PD 5.0000 sec
PW1 3.90 usec
IRNUC 19F
CTEMP 23.0 c
SLVNT CDCL3
EXREF -164.90 ppm
BF 0.12 Hz
RGAIN 48

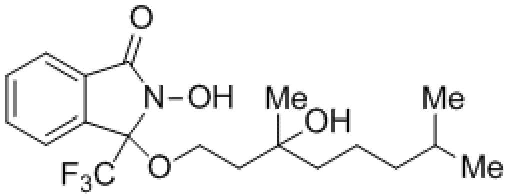

2l

3-Me-1,5-pentdiol

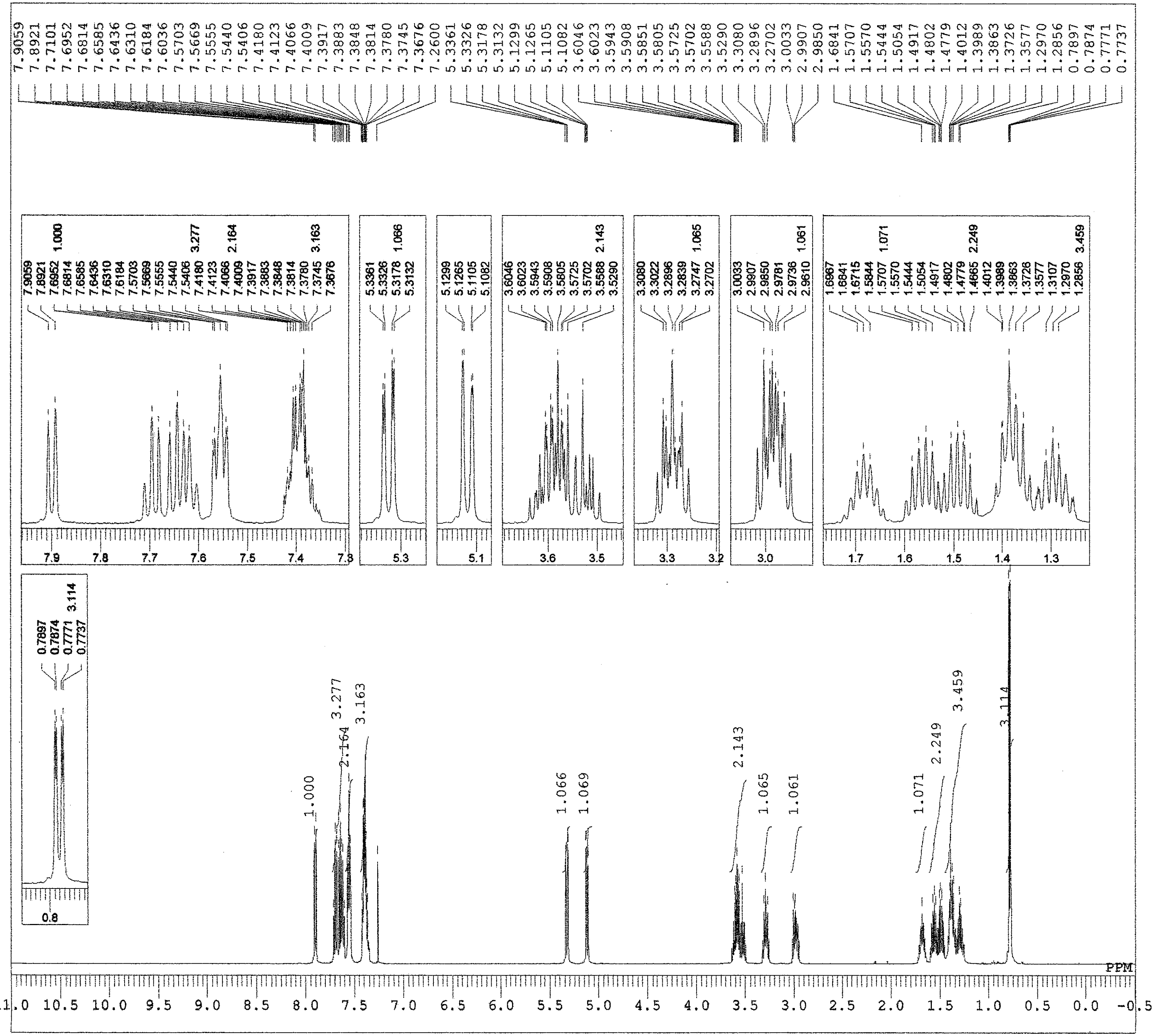

DFILE	ozawa06-062_1H.jdf
COMNT	3-Me-1,5-pentdiol
DATIM	2014-12-05 15:47:26
OBNUC	1H
EXMOD	proton.jxp
OBFRQ	500.16 MHz
OBSET	2.41 KHz
OBFIN	6.01 Hz
POINT	16384
FREQU	9384.38 Hz
SCANS	8
ACQTM	1.7459 sec
PD	5.0000 sec
PW1	5.55 usec
IRNUC	1H
CTEMP	25.3 c
SLVNT	CDCL3
EXREF	7.26 ppm
BF	0.12 Hz
RGAIN	30

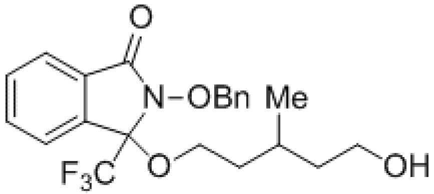

S4m

3-Me-1,5-pentdiol, Bn

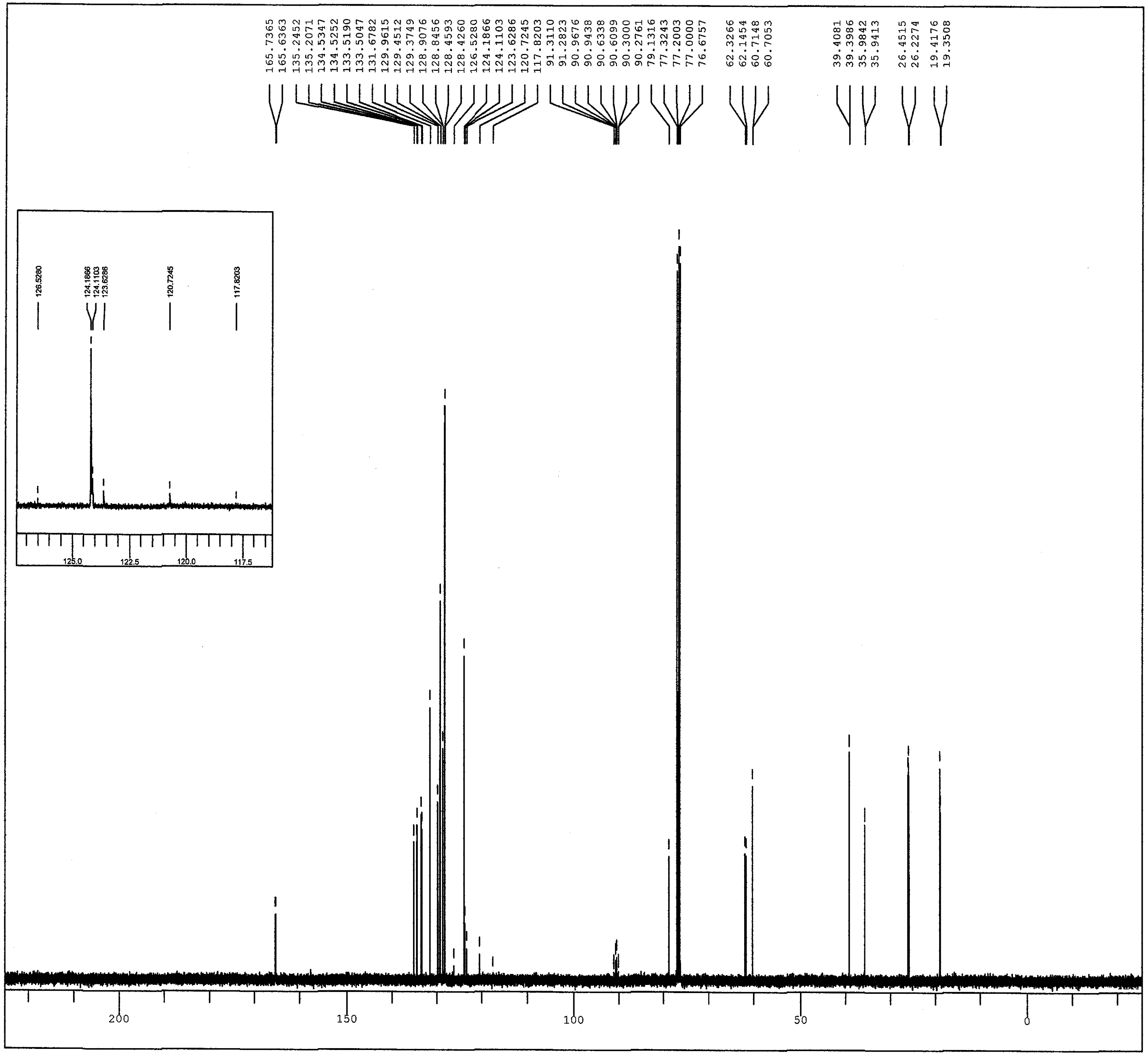

DFILE ozawa06-062_13C.jdf
COMNT 3-Me-1,5-pentdiol, Bn
DATIM 06-12-2014 07:55:19
OBNUC 13C
EXMOD carbon.jxp
OBFRQ 98.52 MHz
OBSET 4.64 KHz
OBFIN 8.74 Hz
POINT 65535
FREQU 30788.18 Hz
SCANS 644
ACQTM 2.1286 sec
PD 2.0000 sec
PW1 3.07 usec
IRNUC 1H
CTEMP 22.3 c
SLVNT CDCL3
EXREF 77.00 ppm
BF 0.12 Hz
RGAIN 60

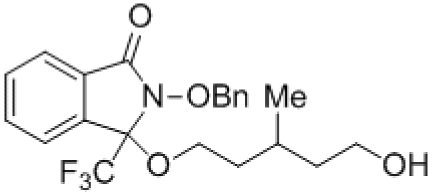

S4m

3-Me-1,5-pentdiol

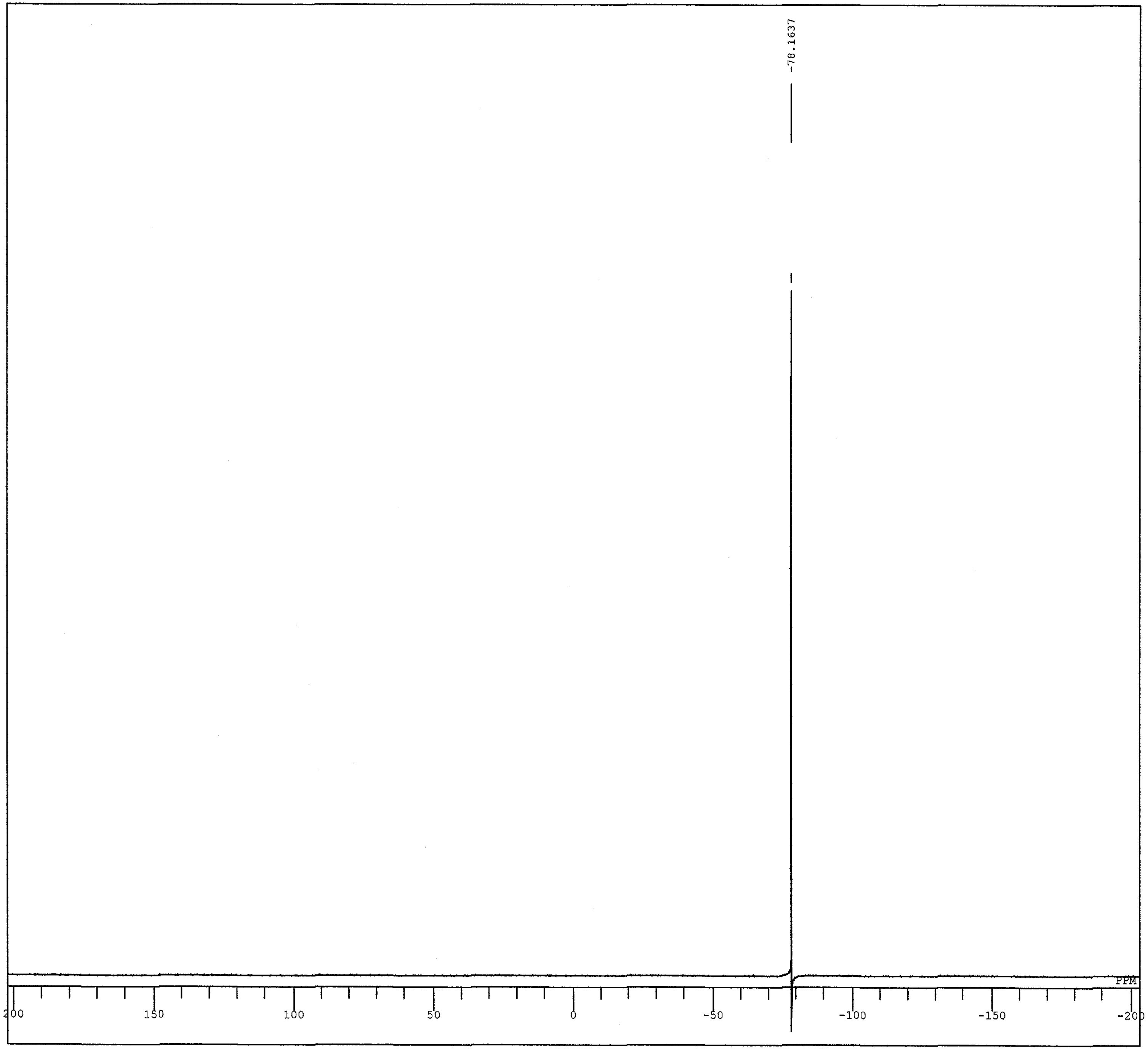

DFILE ozawa06-062_19F.jdf
COMNT 3-Me-1,5-pentdiol
DATIM 06-12-2014 07:52:23
OBNUC 19F
EXMOD proton.jxp
OBFRQ 368.64 MHz
OBSET 7.63 KHz
OBFIN 2.85 Hz
POINT 16384
FREQU 186567.17 Hz
SCANS 4
ACQTM 0.0878 sec
PD 5.0000 sec
PW1 3.90 usec
IRNUC 19F
CTEMP 22.0 c
SLVNT CDCL3
EXREF -164.90 ppm
BF 0.12 Hz
RGAIN 48

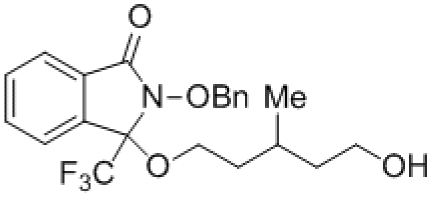

S4m

3-Me-1,5-pentdiol, OH

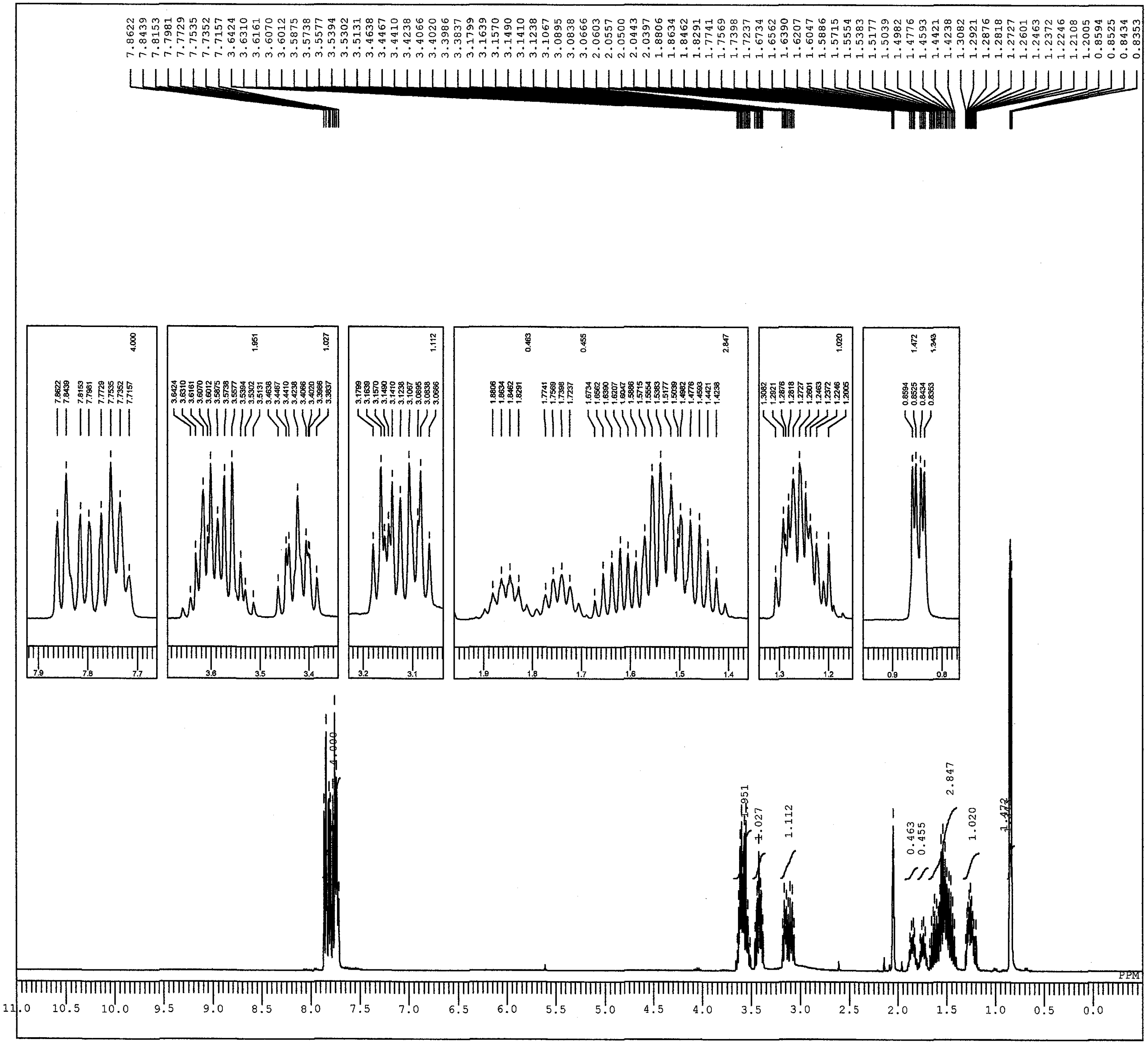

DFILE	ozawa06-064_1H.jdf
COMNT	3-Me-1,5-pentdiol, OH
DATIM	06-12-2014 09:20:16
OBNUC	1H
EXMOD	proton.jxp
OBFRQ	391.78 MHz
OBSET	8.51 KHz
OBFIN	3.34 Hz
POINT	16384
FREQU	7348.62 Hz
SCANS	4
ACQTM	2.2295 sec
PD	5.0000 sec
PW1	5.07 usec
IRNUC	1H
CTEMP	21.9 c
SLVNT	ACETN
EXREF	2.05 ppm
BF	0.12 Hz
RGAIN	30

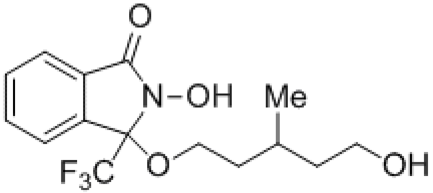

1m

3-Me-1,5-pentdiol, OH

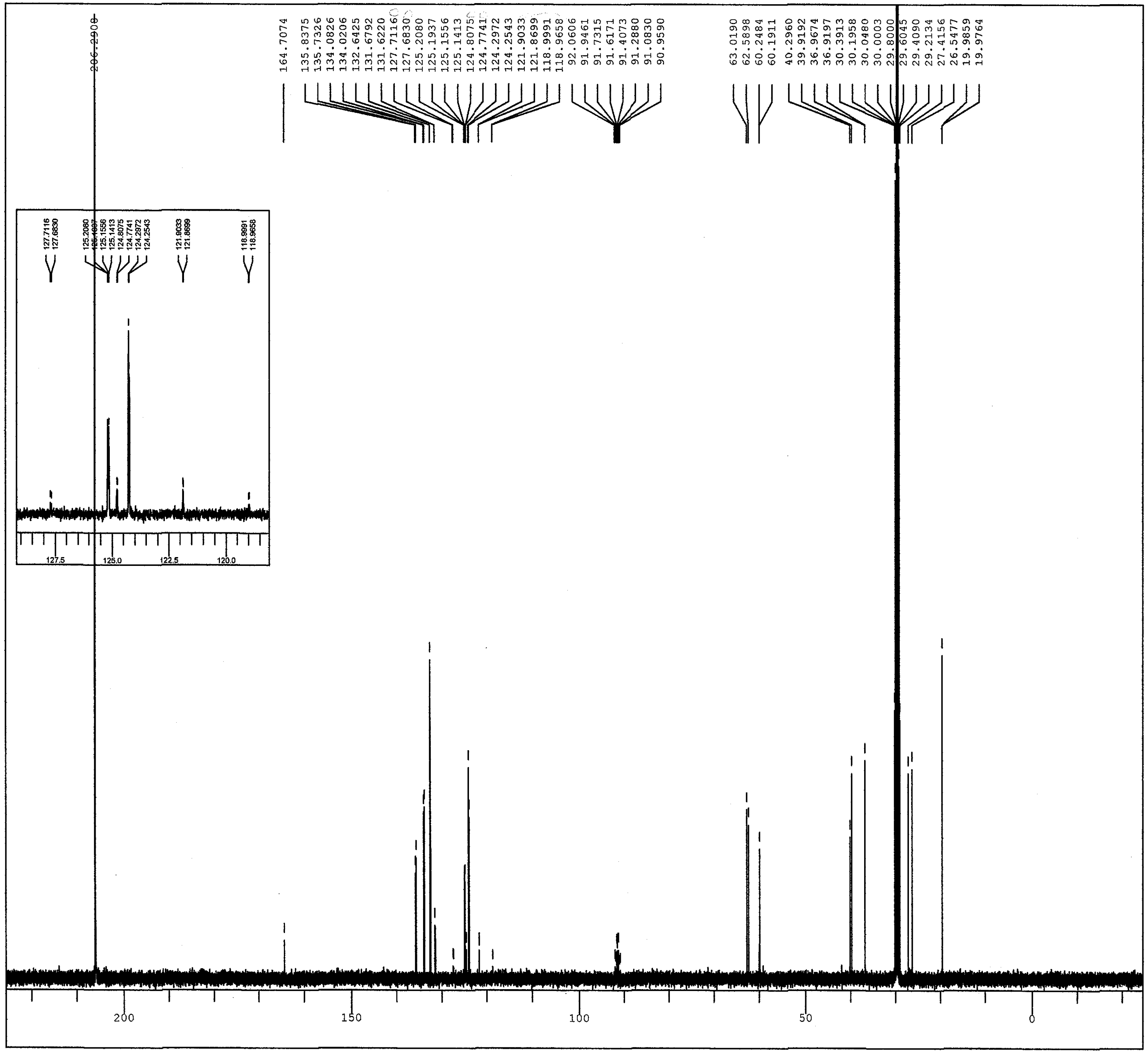

DFILE ozawa06-064_13C.jdf
COMNT 3-Me-1,5-pentdiol, OH
DATIM 06-12-2014 09:21:31
OBNUC 13C
EXMOD carbon.jxp
OBFRQ 98.52 MHz
OBSET 4.64 KHz
OBFIN 8.74 Hz
POINT 65535
FREQU 30788.18 Hz
SCANS 536
ACQTM 2.1286 sec
PD 2.0000 sec
PW1 3.07 usec
IRNUC 1H
CTEMP 22.7 c
SLVNT ACETN
EXREF 29.80 ppm
BF 0.12 Hz
RGAIN 60

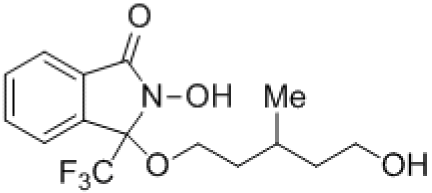

1m

3-Me-1,5-pentdiol, OH

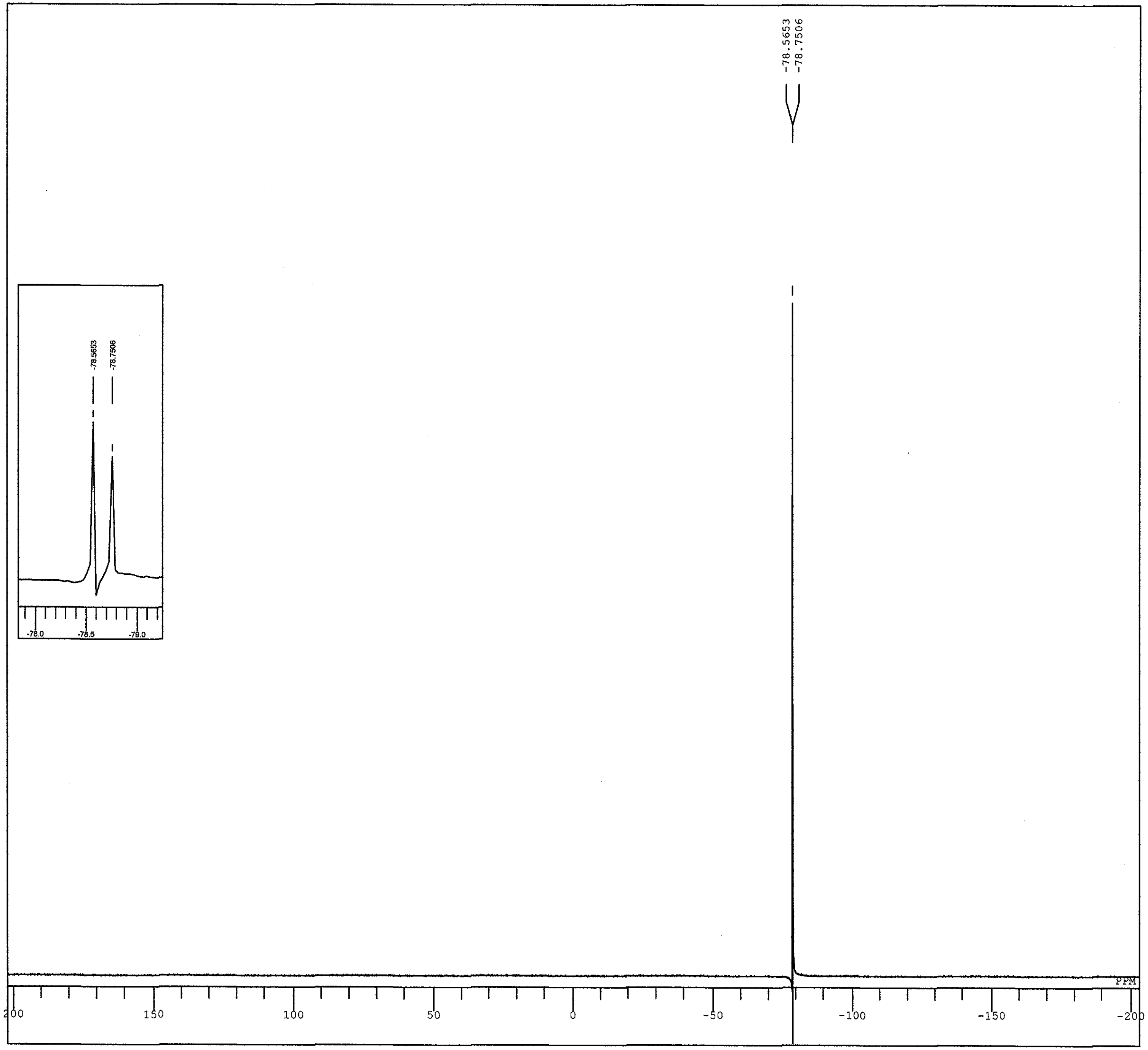


```
DFILE  ozawa06-064_19F.jdf
COMNT  3-Me-1,5-pentdiol, OH
DATIM  06-12-2014 09:18:12
OBNUC  19F
EXMOD  proton.jxp
OBFRQ        368.64 MHz
OBSET          7.63 KHz
OBFIN          2.85 Hz
POINT         16384
FREQU     186567.17 Hz
SCANS             4
ACQTM        0.0878 sec
PD           5.0000 sec
PW1            3.90 usec
IRNUC  19F
CTEMP          21.9 c
SLVNT  ACETN
EXREF       -164.90 ppm
BF             0.12 Hz
RGAIN            46
```

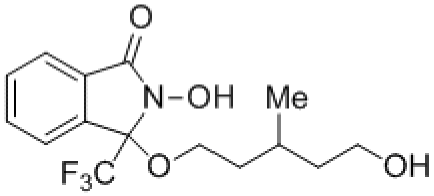

1m

PTLC

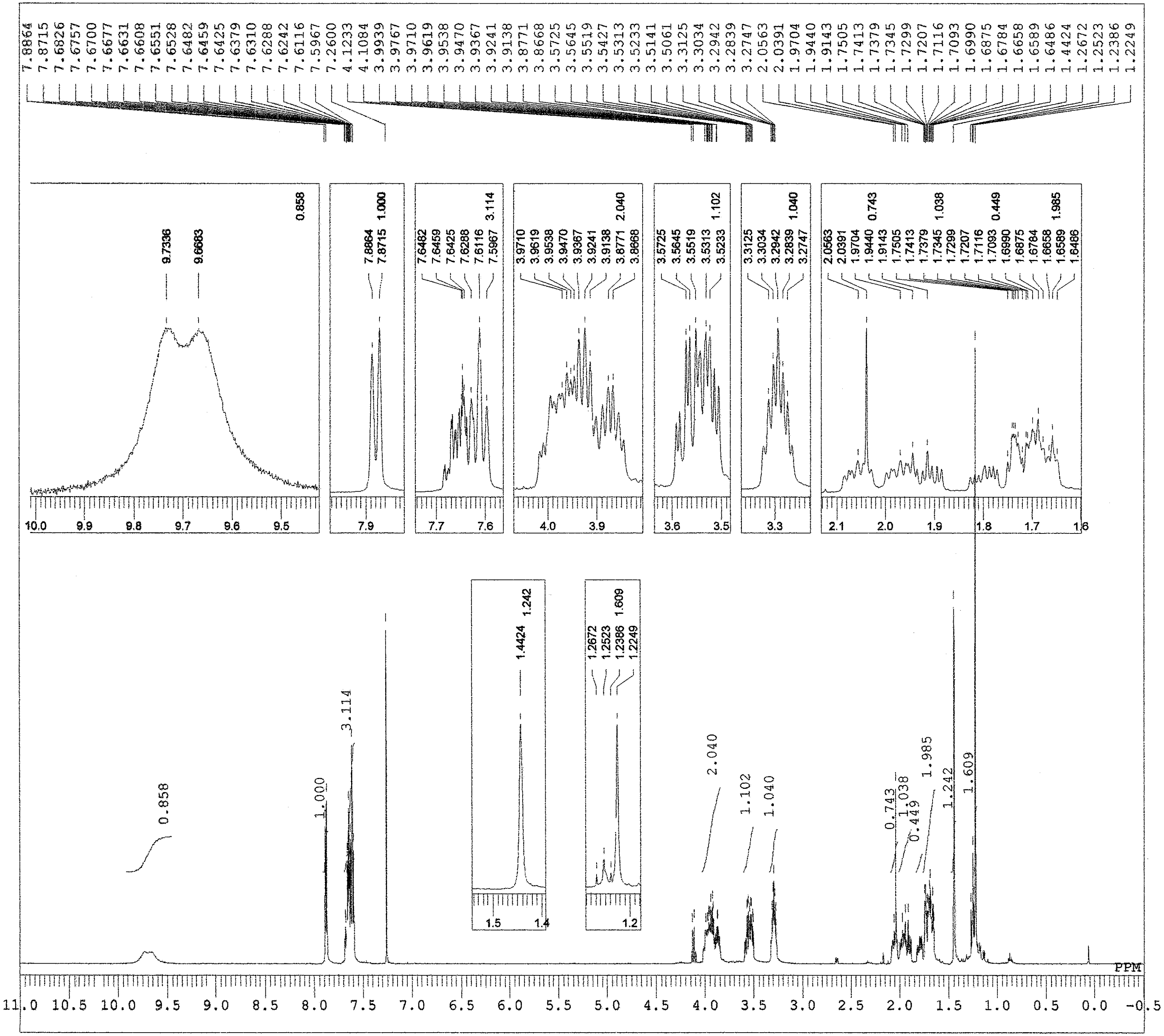

DFILE	ozawa06-086_1H.jdf
COMNT	PTLC
DATIM	2014-12-05 15:43:48
OBNUC	1H
EXMOD	proton.jxp
OBFRQ	500.16 MHz
OBSET	2.41 KHz
OBFIN	6.01 Hz
POINT	16384
FREQU	9384.38 Hz
SCANS	8
ACQTM	1.7459 sec
PD	5.0000 sec
PW1	5.55 usec
IRNUC	1H
CTEMP	25.2 c
SLVNT	CDCL3
EXREF	7.26 ppm
BF	0.12 Hz
RGAIN	30

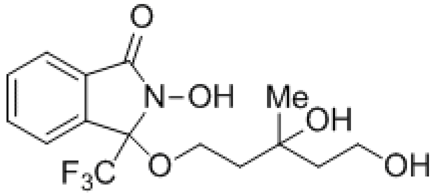

2m

3-Me-1,5-pentdiol, [O]

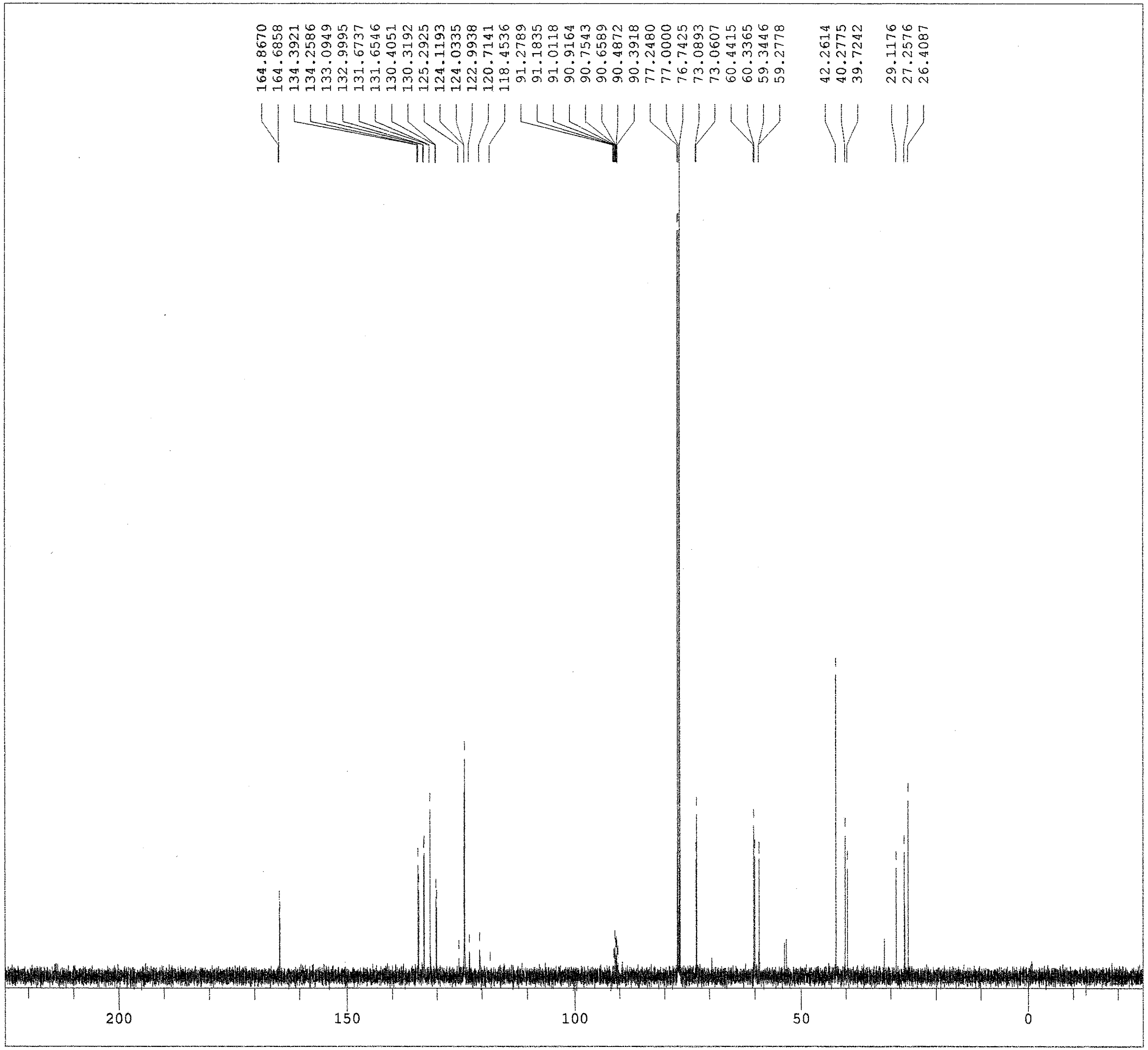

DFILE	ozawa06-086_13C.jdf
COMNT	3-Me-1,5-pentdiol, [O]
DATIM	2014-12-05 12:47:59
OBNUC	13C
EXMOD	carbon.jxp
OBFRQ	125.77 MHz
OBSET	7.87 KHz
OBFIN	4.21 Hz
POINT	32767
FREQU	39308.18 Hz
SCANS	300
ACQTM	0.8336 sec
PD	2.0000 sec
PW1	3.40 usec
IRNUC	1H
CTEMP	25.7 c
SLVNT	CDCL3
EXREF	77.00 ppm
BF	0.12 Hz
RGAIN	60

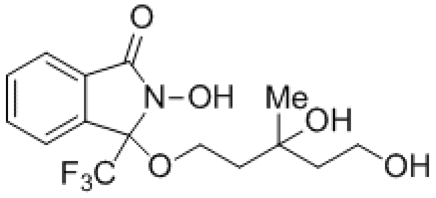

2m

3-Me-1,5-pentdiol, [O]

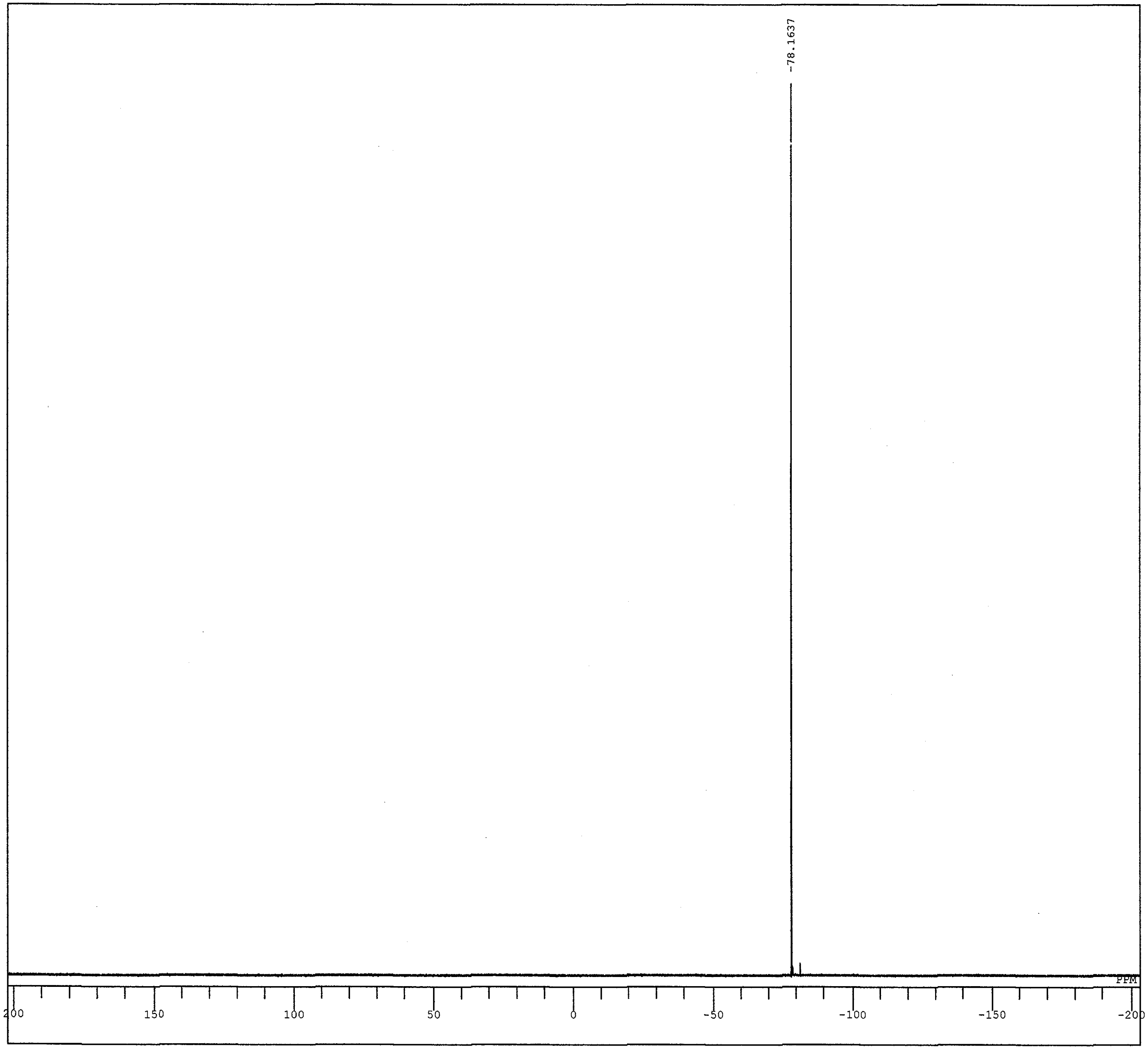


```
DFILE  ozawa06-086_19F.jdf
COMNT  3-Me-1,5-pentdiol, [O]
DATIM  05-12-2014 17:21:06
OBNUC  19F
EXMOD  proton.jxp
OBFRQ        368.64 MHz
OBSET          7.63 KHz
OBFIN          2.85 Hz
POINT         16384
FREQU     186567.17 Hz
SCANS             4
ACQTM        0.0878 sec
PD           5.0000 sec
PW1            3.90 usec
IRNUC  19F
CTEMP          22.6 c
SLVNT  CDCL3
EXREF       -164.90 ppm
BF             0.12 Hz
RGAIN            50
```

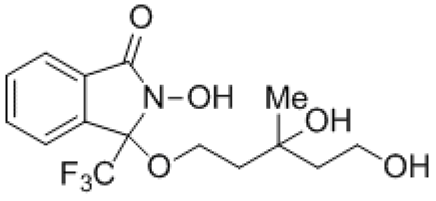

2m

3-Me-5-OMOM, Bn

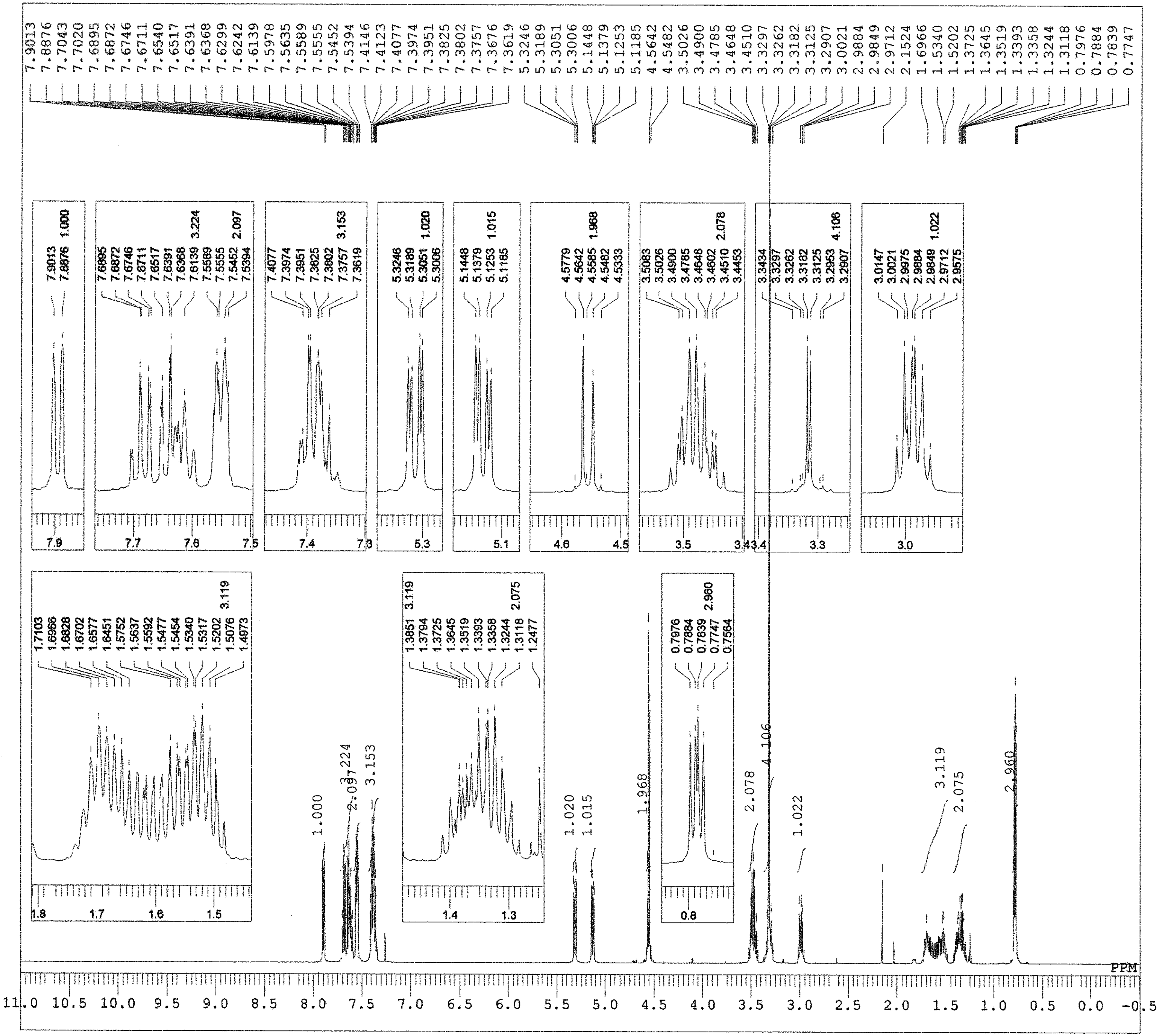

DFILE	ozawa07-022_1H.als
COMNT	3-Me-5-OMOM, Bn
DATIM	2015-03-02 10:00:34
OBNUC	1H
EXMOD	proton.jxp
OBFRQ	500.16 MHz
OBSET	2.41 KHz
OBFIN	6.01 Hz
POINT	13107
FREQU	7507.51 Hz
SCANS	8
ACQTM	1.7459 sec
PD	5.0000 sec
PW1	5.55 usec
IRNUC	1H
CTEMP	23.4 c
SLVNT	CDCL3
EXREF	7.26 ppm
BF	0.12 Hz
RGAIN	24

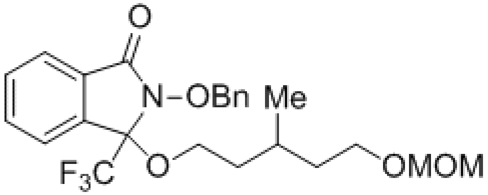

S4n

3-Me-5-OMOM, Bn

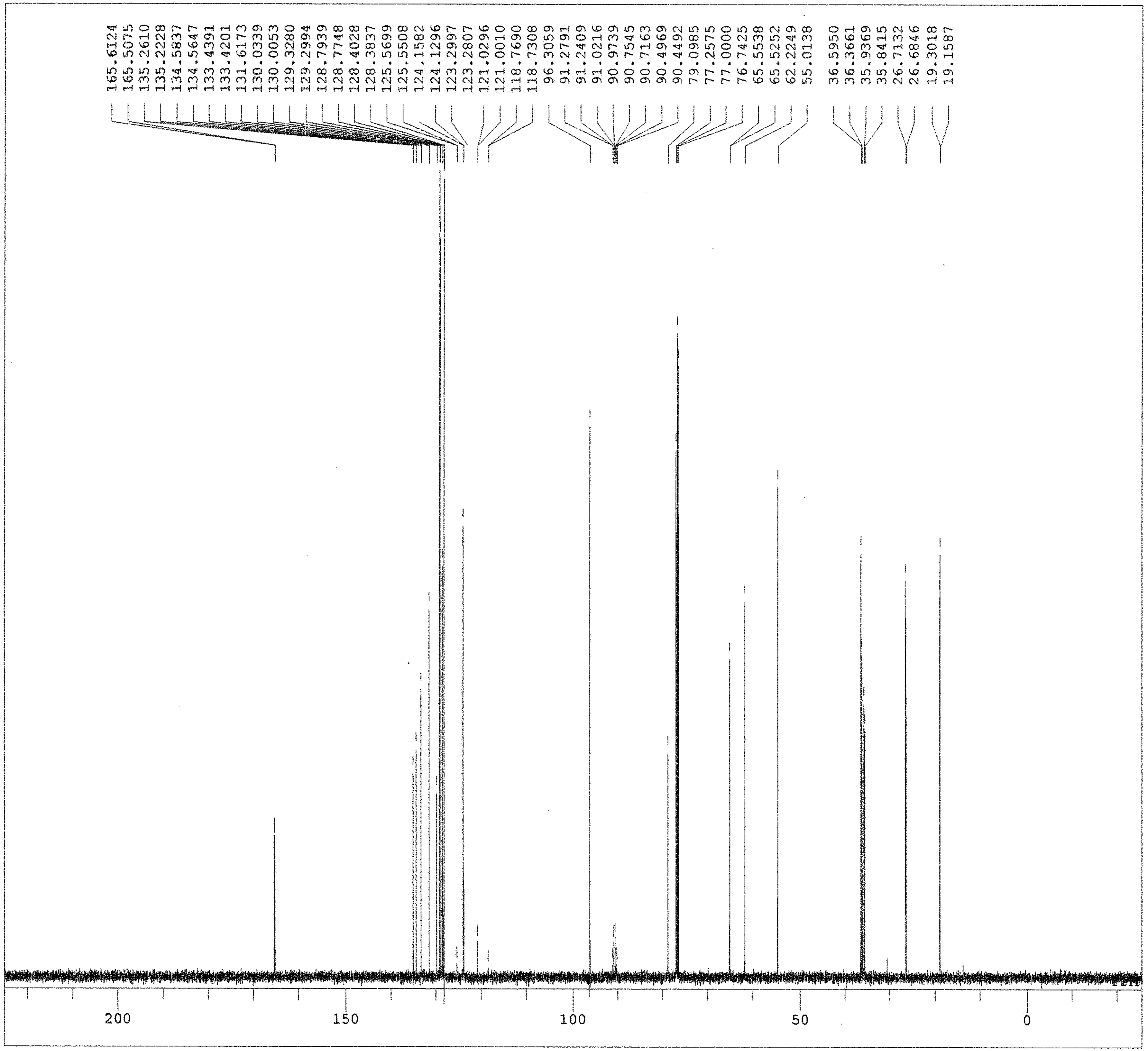

DFILE ozawa07-022_13C.als
COMNT 3-Me-5-OMOM, Bn
DATIM 2015-03-02 10:02:04
OBNUC 13C
EXMOD carbon.jxp
OBFRQ 125.77 MHz
OBSET 7.87 KHz
OBFIN 4.21 Hz
POINT 26214
FREQU 31446.54 Hz
SCANS 476
ACQTM 0.8336 sec
PD 2.0000 sec
PW1 3.40 usec
IRNUC 1H
CTEMP 24.0 c
SLVNT CDCL3
EXREF 77.00 ppm
BF 0.12 Hz
RGAIN 60

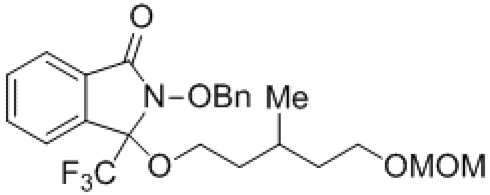

S4n

3-Me-5-OMOM, Bn

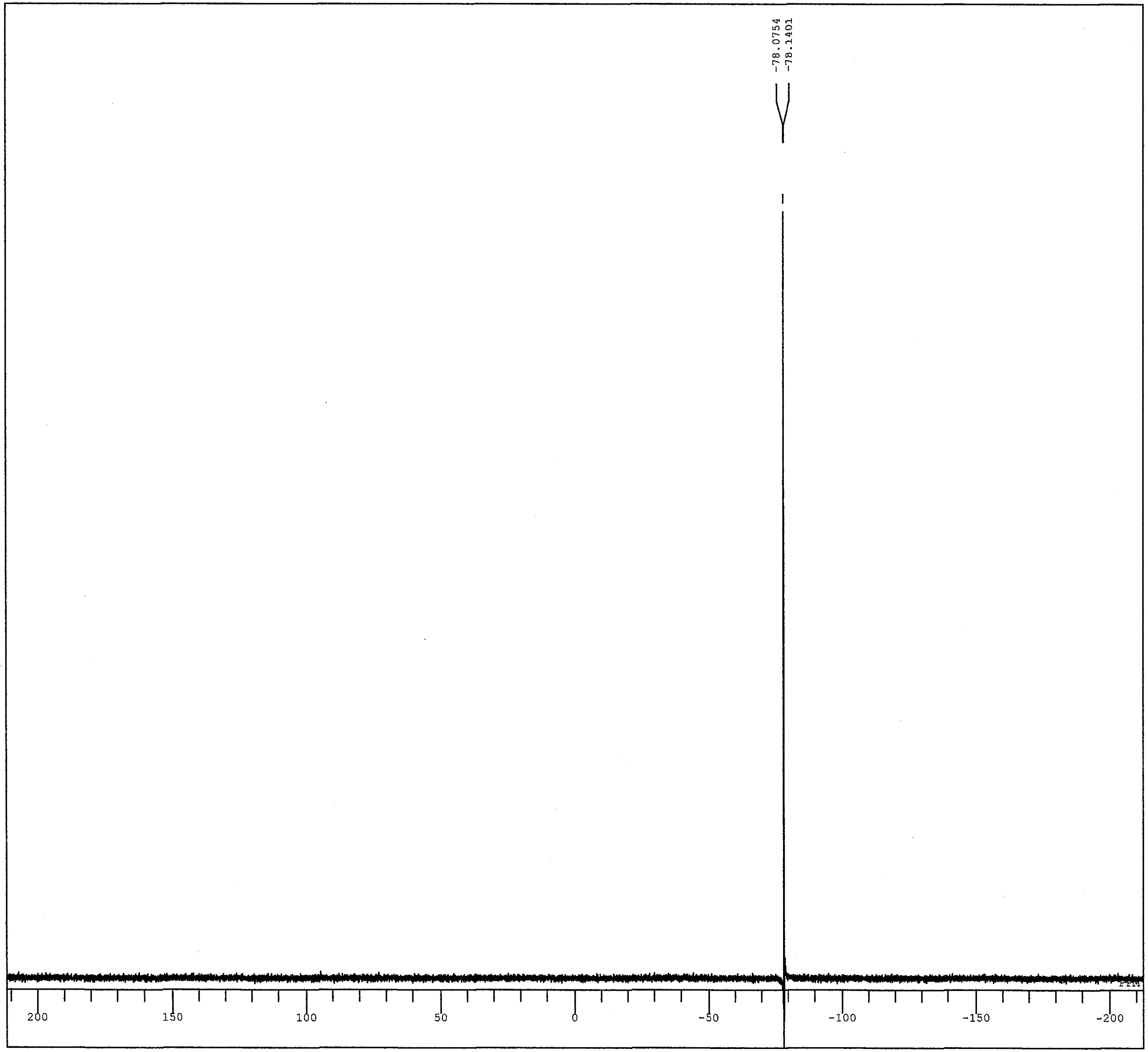

DFILE ozawa07-022_19F.jdf
COMNT 3-Me-5-OMOM, Bn
DATIM 21-03-2015 12:11:35
OBNUC 19F
EXMOD proton.jxp
OBFRQ 368.64 MHz
OBSET 7.63 KHz
OBFIN 2.85 Hz
POINT 16384
FREQU 195312.50 Hz
SCANS 4
ACQTM 0.0839 sec
PD 5.0000 sec
PW1 3.90 usec
IRNUC 19F
CTEMP 25.1 c
SLVNT CDCL3
EXREF -164.90 ppm
BF 0.12 Hz
RGAIN 50

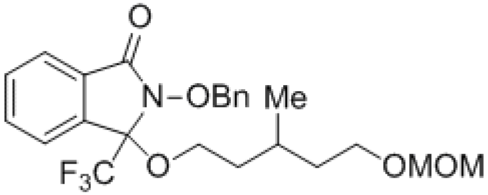

S4n

3-Me-5-OMOM, OH

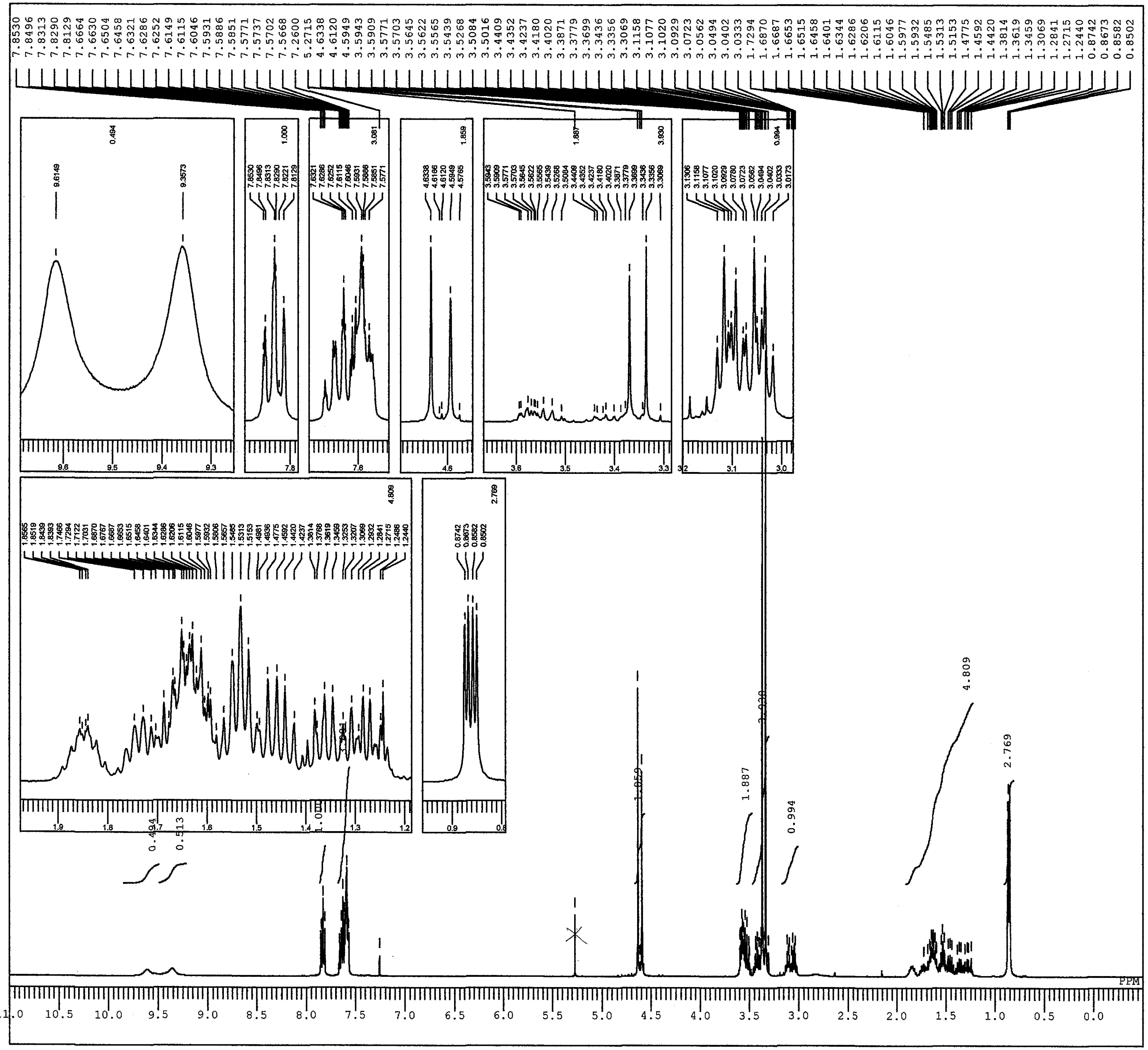

DFILE ozawa07-024_1H.jdf
COMNT 3-Me-5-OMOM, OH
DATIM 21-03-2015 12:26:11
OBNUC 1H
EXMOD proton.jxp
OBFRQ 391.78 MHz
OBSET 8.51 KHz
OBFIN 3.34 Hz
POINT 16384
FREQU 7348.62 Hz
SCANS 4
ACQTM 2.2295 sec
PD 5.0000 sec
PW1 4.99 usec
IRNUC 1H
CTEMP 25.1 c
SLVNT CDCL3
EXREF 7.26 ppm
BF 0.12 Hz
RGAIN 22

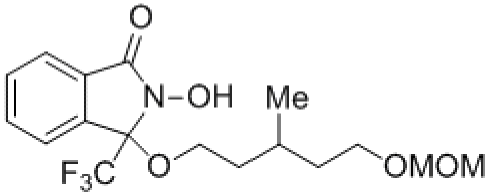

1n

3-Me-5-OMOM, OH

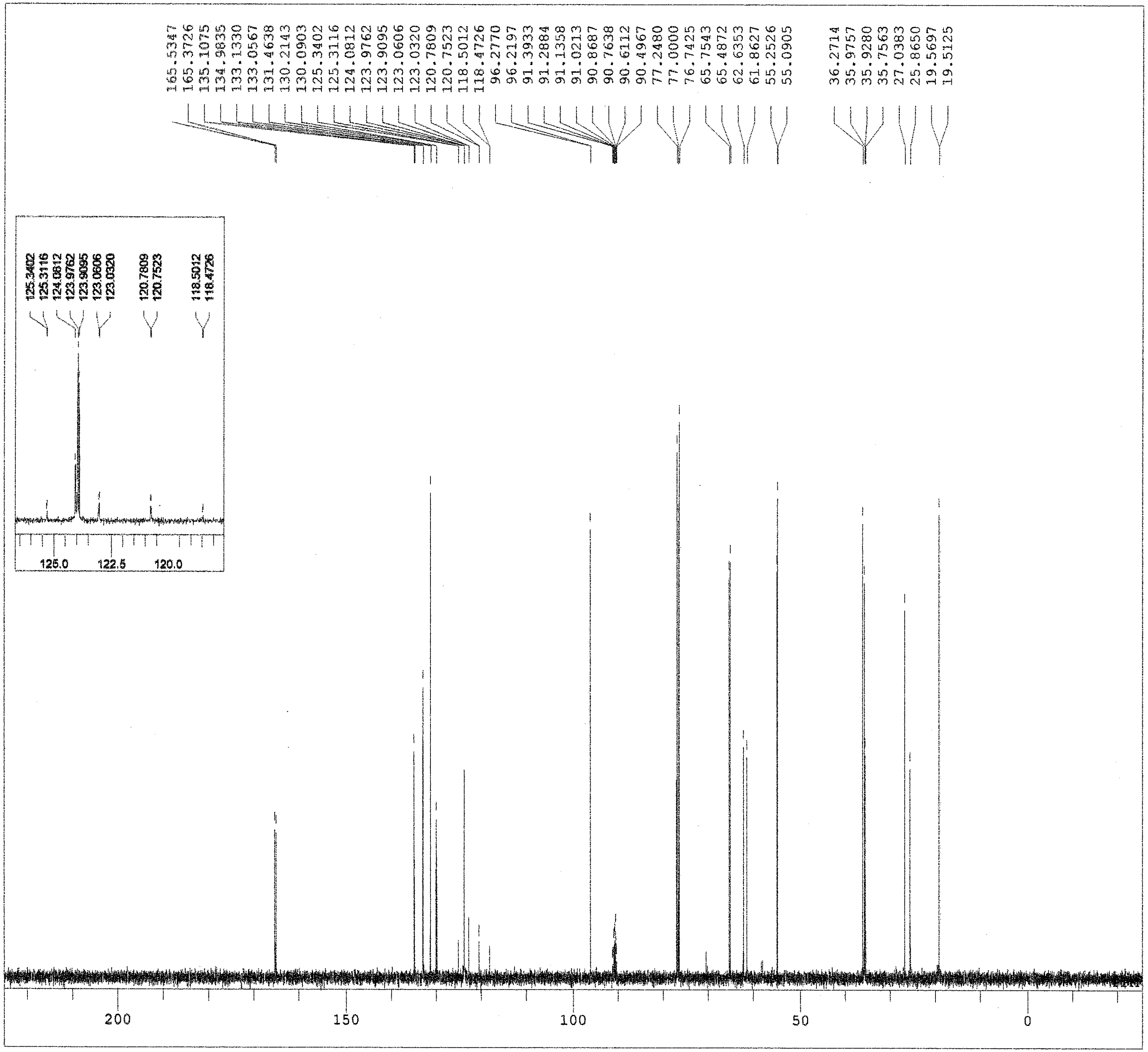

DFILE	ozawa07-024_13C.jdf
COMNT	3-Me-5-OMOM, OH
DATIM	2015-03-21 12:32:50
OBNUC	13C
EXMOD	carbon.jxp
OBFRQ	125.77 MHz
OBSET	7.87 KHz
OBFIN	4.21 Hz
POINT	32767
FREQU	39308.18 Hz
SCANS	220
ACQTM	0.8336 sec
PD	2.0000 sec
PW1	3.40 usec
IRNUC	1H
CTEMP	26.5 c
SLVNT	CDCL3
EXREF	77.00 ppm
BF	0.12 Hz
RGAIN	60

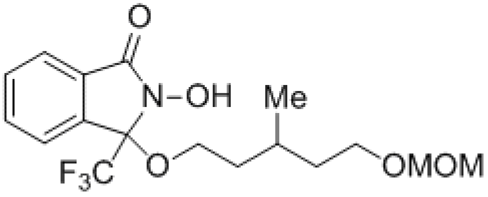

1n

3-Me-5-OMOM. OH

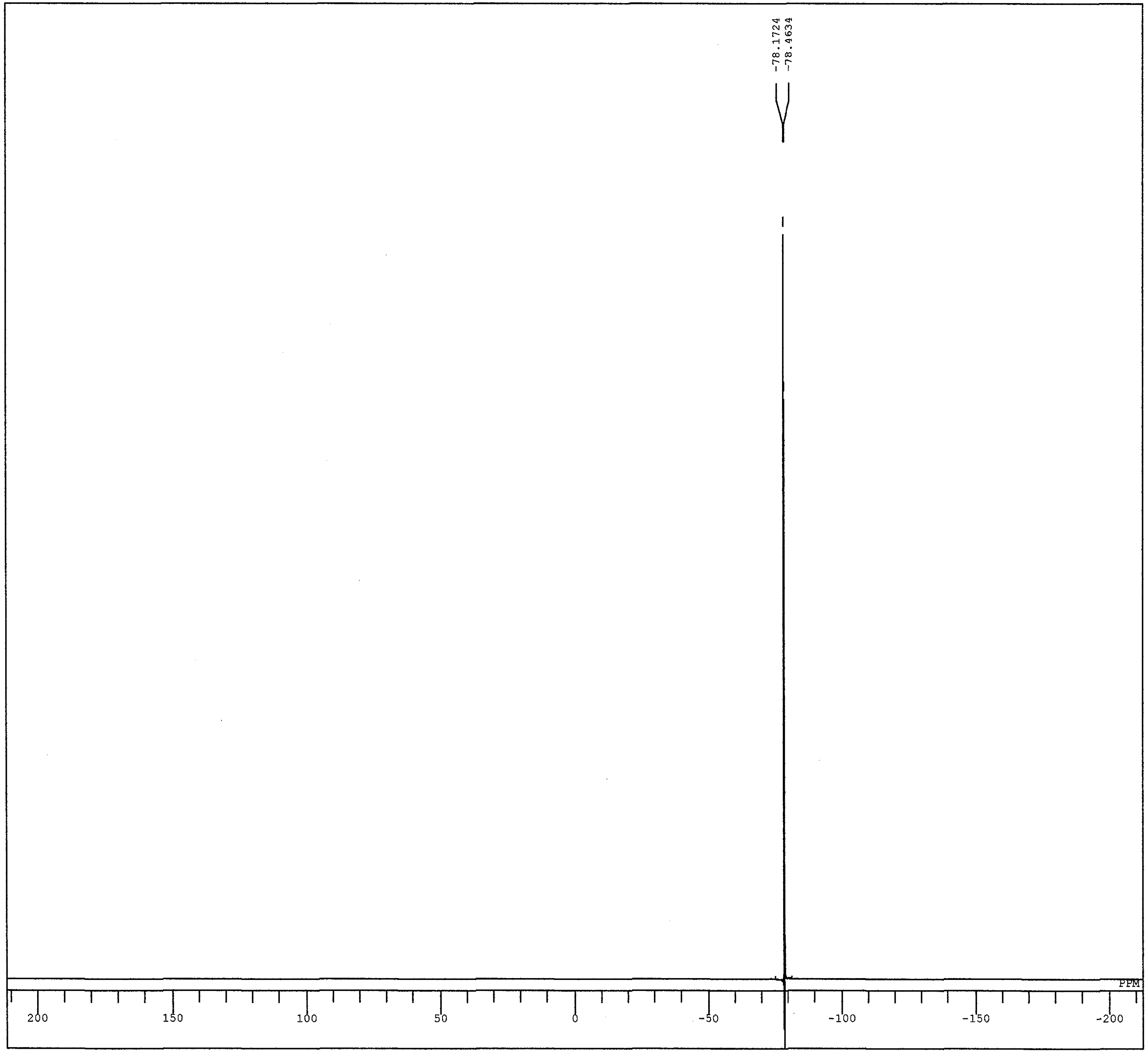

DFILE	ozawa07-024_19F.jdf
COMNT	3-Me-5-OMOM. OH
DATIM	21-03-2015 12:22:07
OBNUC	19F
EXMOD	proton.jxp
OBFRQ	368.64 MHz
OBSET	7.63 KHz
OBFIN	2.85 Hz
POINT	16384
FREQU	195312.50 Hz
SCANS	4
ACQTM	0.0839 sec
PD	5.0000 sec
PW1	3.90 usec
IRNUC	19F
CTEMP	25.1 c
SLVNT	CDCL3
EXREF	-164.90 ppm
BF	0.12 Hz
RGAIN	44

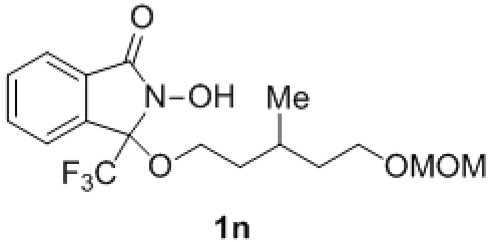

1n

3-Me-5-OMOM, [O]

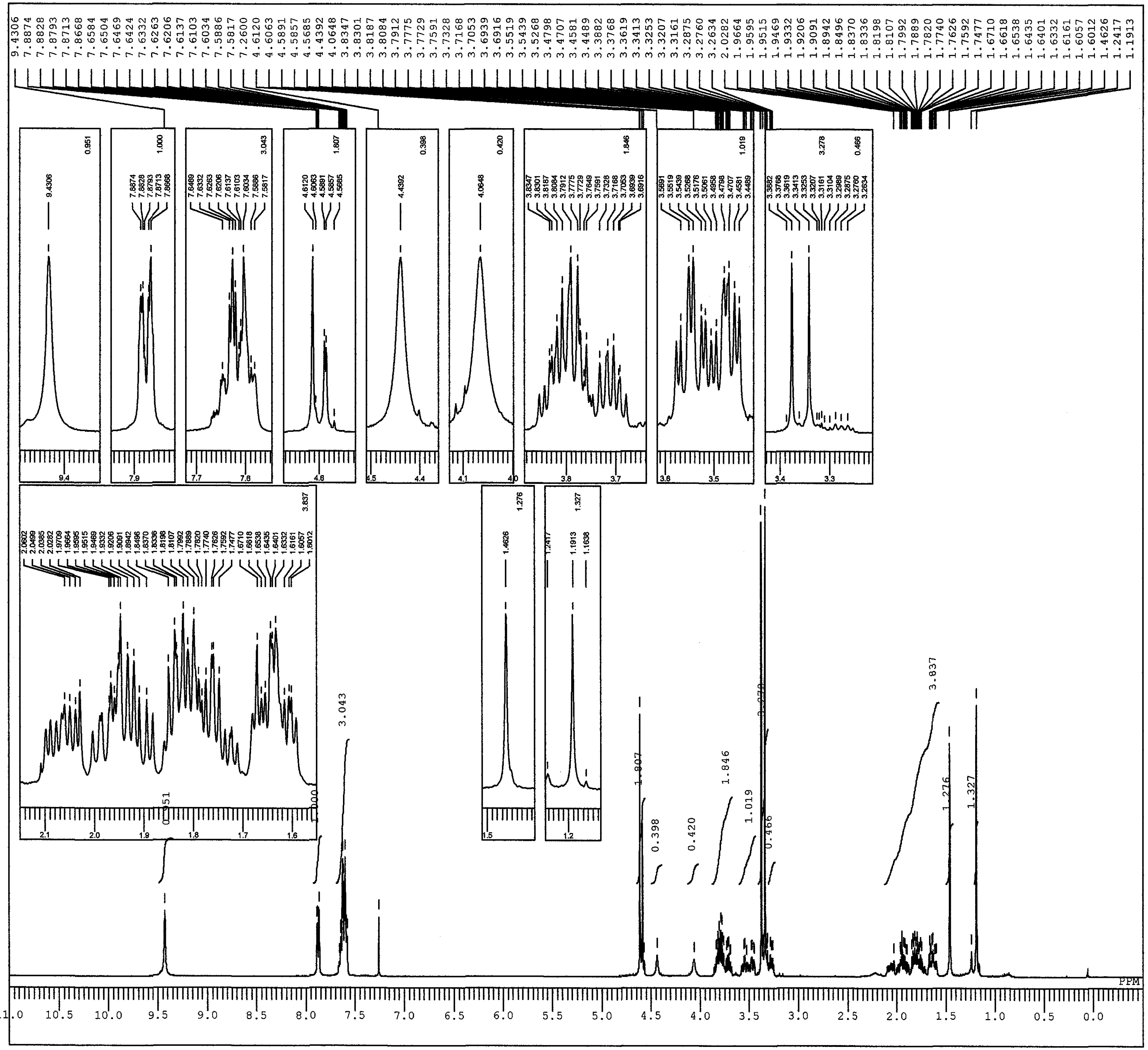

DFILE ozawa07-042_1H.jdf
COMNT 3-Me-5-OMOM, [O]
DATIM 21-03-2015 10:35:50
OBNUC 1H
EXMOD proton.jxp
OBFRQ 391.78 MHz
OBSET 8.51 KHz
OBFIN 3.34 Hz
POINT 16384
FREQU 7348.62 Hz
SCANS 4
ACQTM 2.2295 sec
PD 5.0000 sec
PW1 4.99 usec
IRNUC 1H
CTEMP 25.0 c
SLVNT CDCL3
EXREF 7.26 ppm
BF 0.12 Hz
RGAIN 30

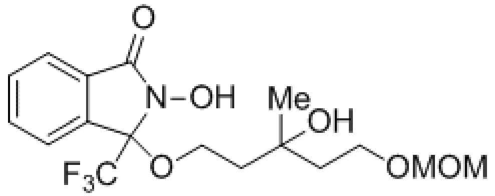

2n

3-Me-5-OMOM, [O]

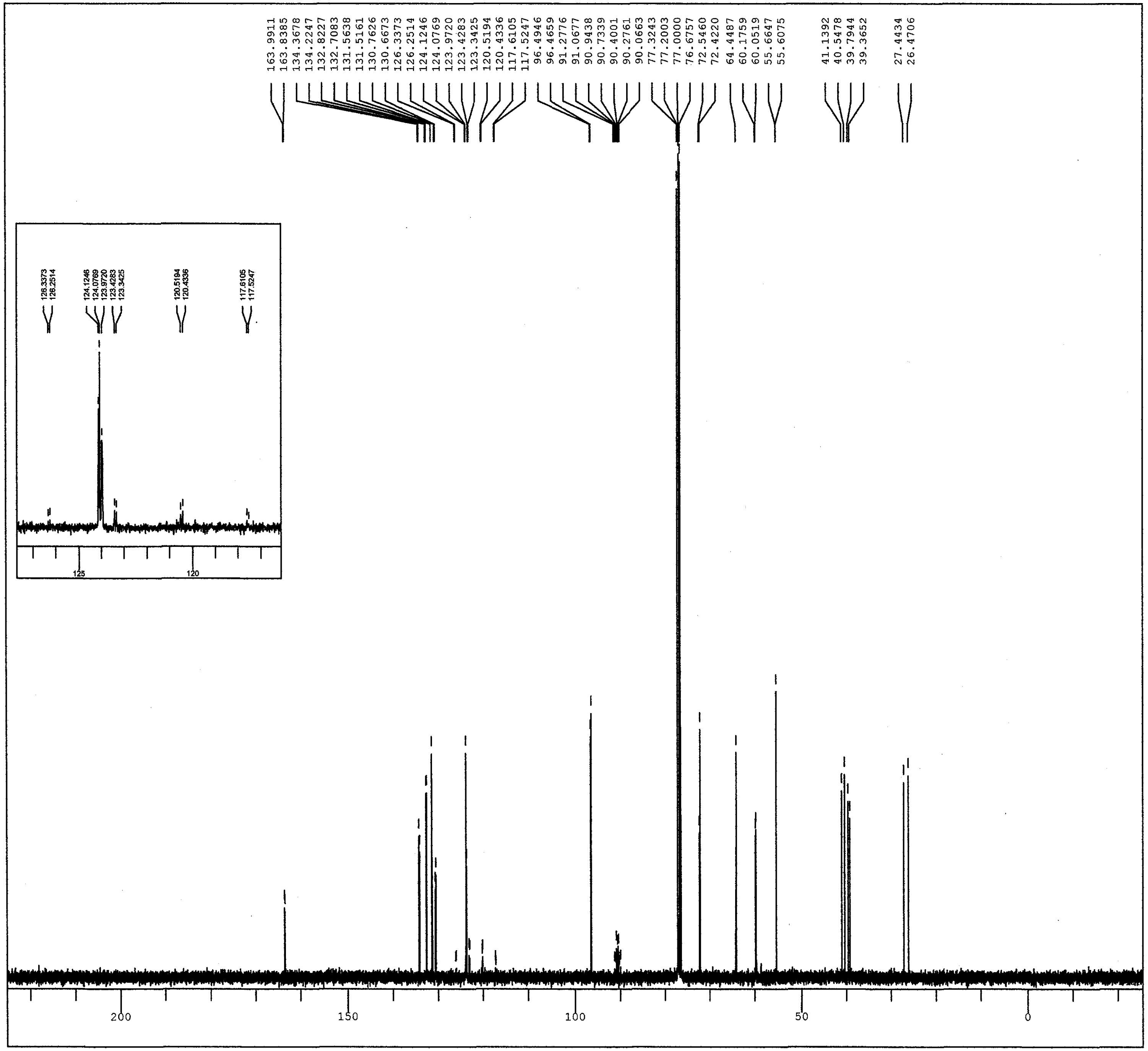

DFILE ozawa07-042_13C.jdf
COMNT 3-Me-5-OMOM, [O]
DATIM 21-03-2015 10:36:53
OBNUC 13C
EXMOD carbon.jxp
OBFRQ 98.52 MHz
OBSET 4.64 KHz
OBFIN 8.74 Hz
POINT 32767
FREQU 30788.18 Hz
SCANS 1100
ACQTM 1.0643 sec
PD 2.0000 sec
PW1 3.16 usec
IRNUC 1H
CTEMP 25.4 c
SLVNT CDCL3
EXREF 77.00 ppm
BF 0.12 Hz
RGAIN 60

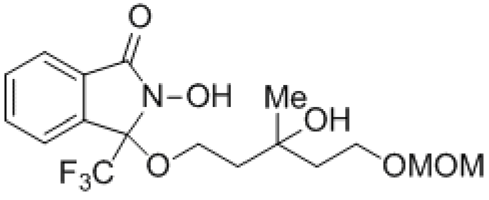

2n

3-Me-5-OMOM, [O]

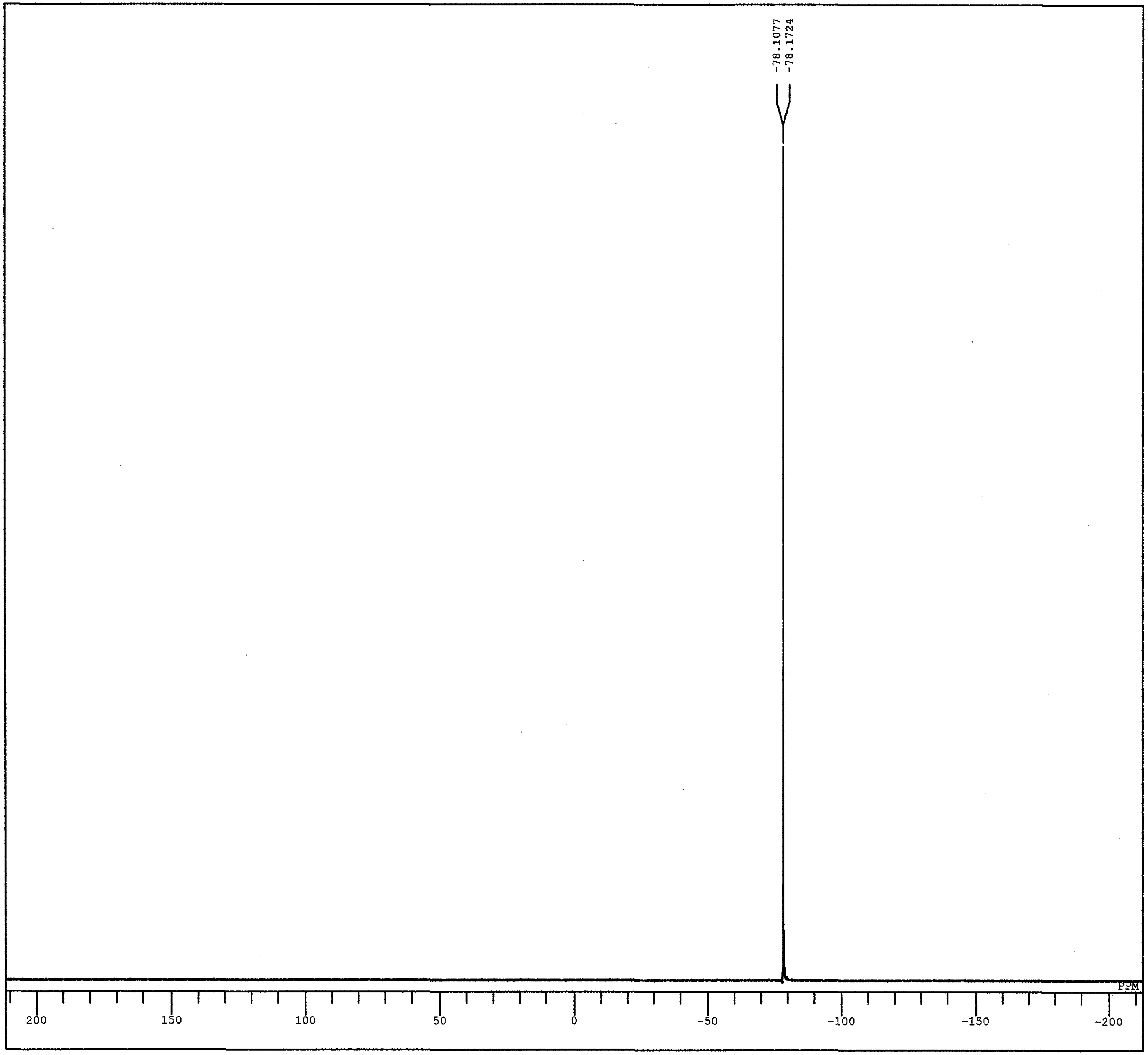


```
DFILE  ozawa07-042_19F.jdf
COMNT  3-Me-5-OMOM, [O]
DATIM  01-04-2015 16:44:15
OBNUC  19F
EXMOD  proton.jxp
OBFRQ        368.64 MHz
OBSET          7.63 KHz
OBFIN          2.85 Hz
POINT         16384
FREQU     195312.50 Hz
SCANS             8
ACQTM        0.0839 sec
PD           5.0000 sec
PW1            3.90 usec
IRNUC  19F
CTEMP          26.7 c
SLVNT  CDCL3
EXREF       -164.90 ppm
BF             0.12 Hz
RGAIN            50
```

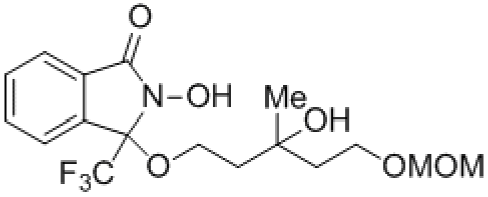

2n

3-Me-5-O(4-pentyne), PMB

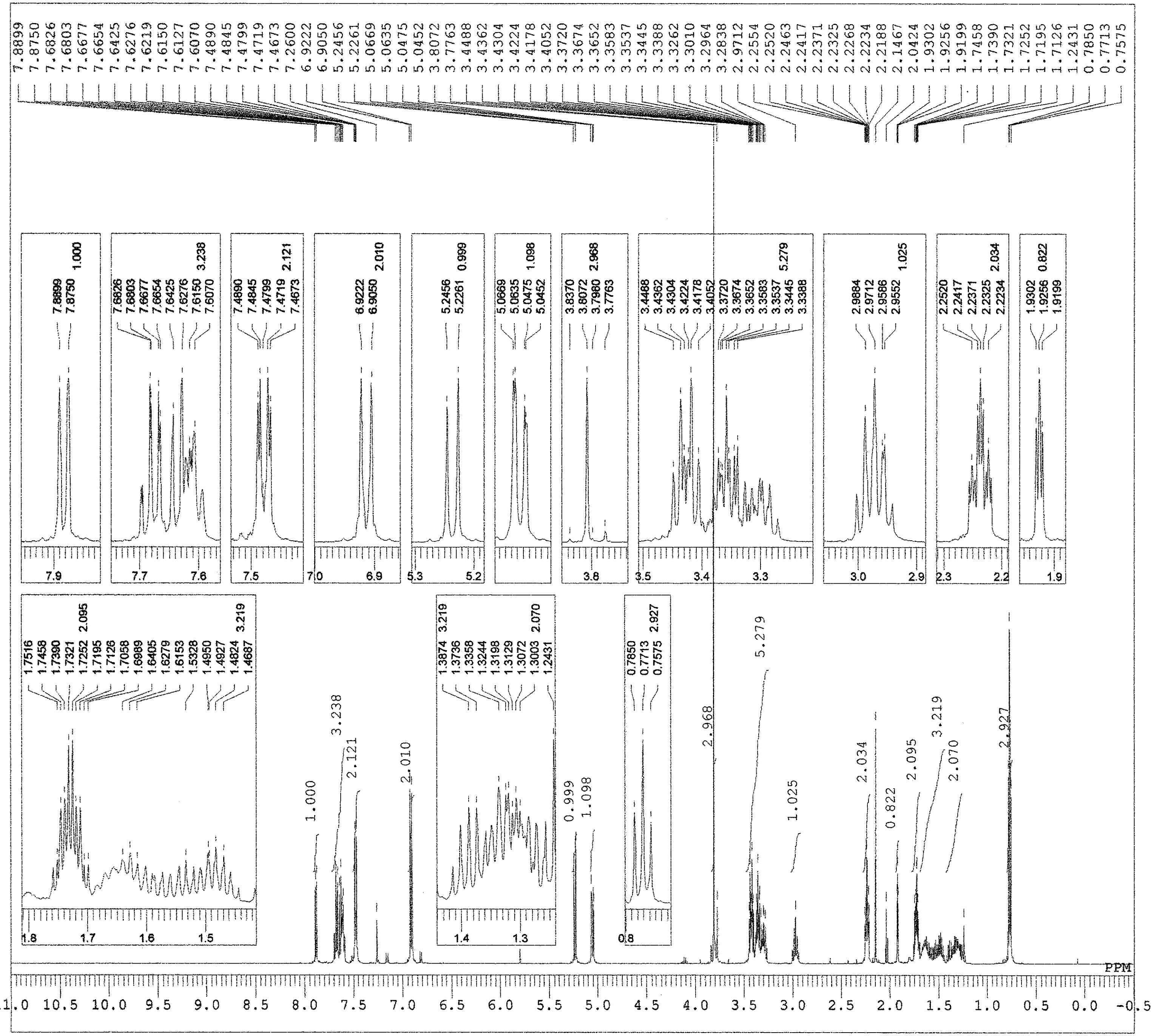

DFILE ozawa07-085_1H.als
COMNT 3-Me-5-O(4-pentyne), PMB
DATIM 2015-04-13 14:35:57
OBNUC 1H
EXMOD proton.jxp
OBFRQ 500.16 MHz
OBSET 2.41 KHz
OBFIN 6.01 Hz
POINT 13107
FREQU 7507.51 Hz
SCANS 4
ACQTM 1.7459 sec
PD 5.0000 sec
PW1 5.55 usec
IRNUC 1H
CTEMP 27.3 c
SLVNT CDCL3
EXREF 7.26 ppm
BF 0.12 Hz
RGAIN 24

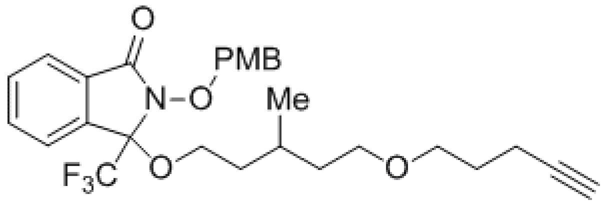

S4'o

3-Me-5-O(4-pentyne), PMB

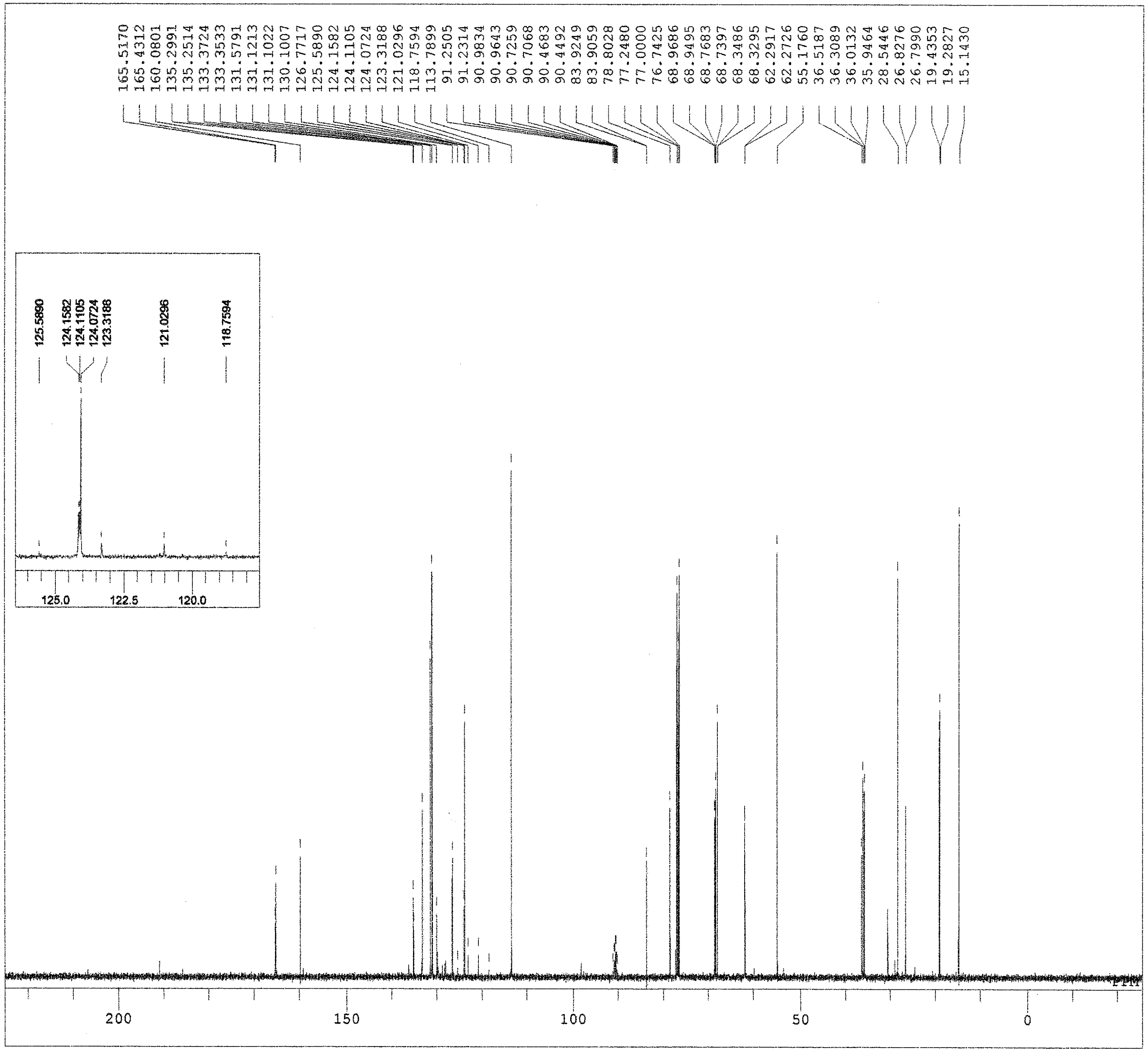

DFILE ozawa07-085_13C.als
COMNT 3-Me-5-O(4-pentyne), PMB
DATIM 2015-04-13 14:36:59
OBNUC 13C
EXMOD carbon.jxp
OBFRQ 125.77 MHz
OBSET 7.87 KHz
OBFIN 4.21 Hz
POINT 26214
FREQU 31446.54 Hz
SCANS 600
ACQTM 0.0000 sec
PD 2.0000 sec
PW1 3.40 usec
IRNUC 1H
CTEMP 27.8 c
SLVNT CDCL3
EXREF 77.00 ppm
BF 0.12 Hz
RGAIN 60

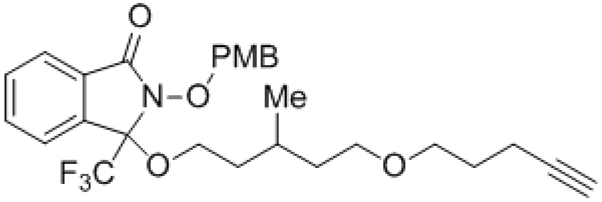

S4'o

tert-yne, PMB

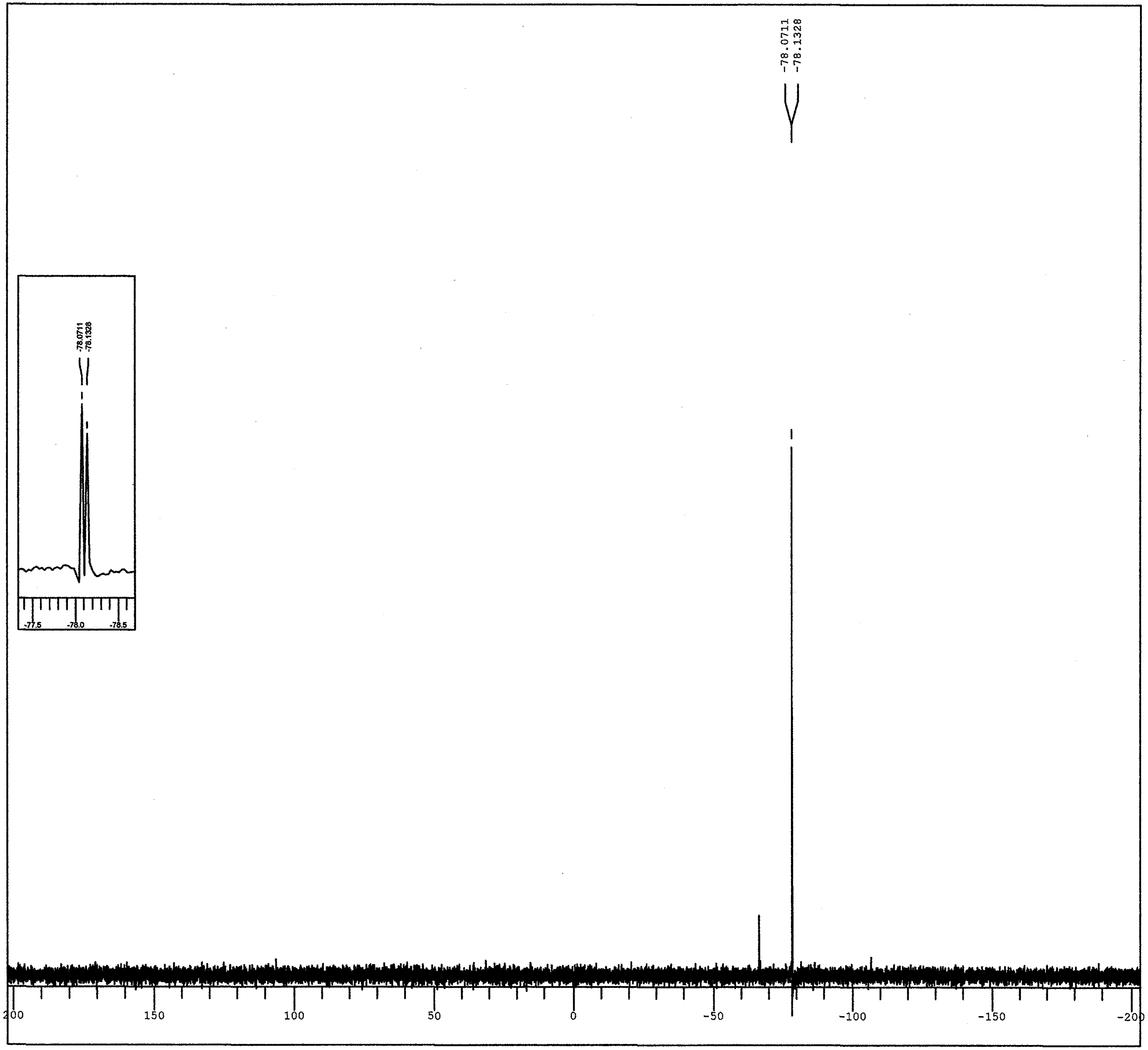

DFILE ozawa07-085_19F.jdf
COMNT tert-yne, PMB
DATIM 11-05-2015 15:30:50
OBNUC 19F
EXMOD proton.jxp
OBFRQ 368.64 MHz
OBSET 7.63 KHz
OBFIN 2.85 Hz
POINT 16384
FREQU 186567.17 Hz
SCANS 4
ACQTM 0.0878 sec
PD 5.0000 sec
PW1 3.90 usec
IRNUC 19F
CTEMP 21.6 c
SLVNT CDCL3
EXREF -164.90 ppm
BF 0.12 Hz
RGAIN 50

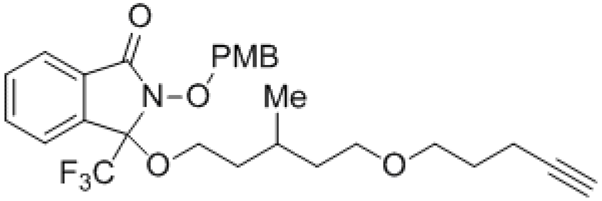

S4'o

3-Me-5-O(4-pentyne), OH

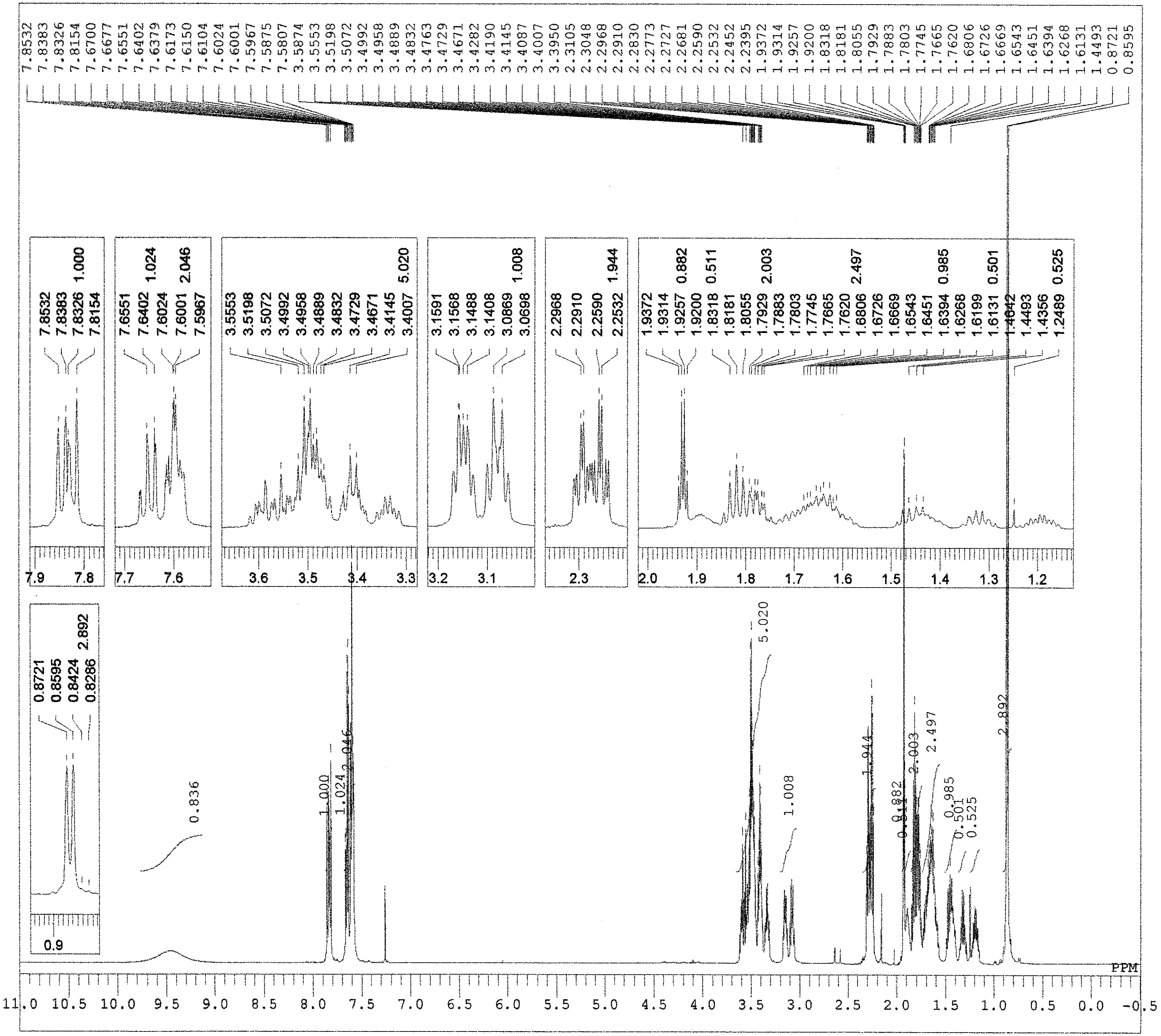

DFILE	ozawa07-099_1H.jdf
COMNT	3-Me-5-O(4-pentyne), OH
DATIM	2015-05-01 10:25:55
OBNUC	1H
EXMOD	proton.jxp
OBFRQ	500.16 MHz
OBSET	2.41 KHz
OBFIN	6.01 Hz
POINT	16384
FREQU	9384.38 Hz
SCANS	4
ACQTM	1.7459 sec
PD	5.0000 sec
PW1	5.55 usec
IRNUC	1H
CTEMP	21.1 c
SLVNT	CDCL3
EXREF	7.26 ppm
BF	0.12 Hz
RGAIN	24

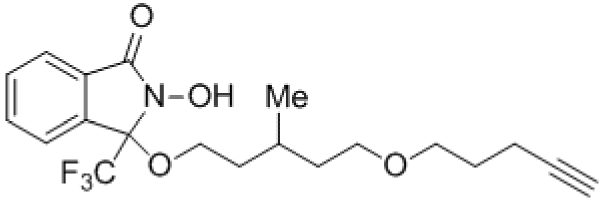

1o

3-Me-5-O(4-pentyne), OH

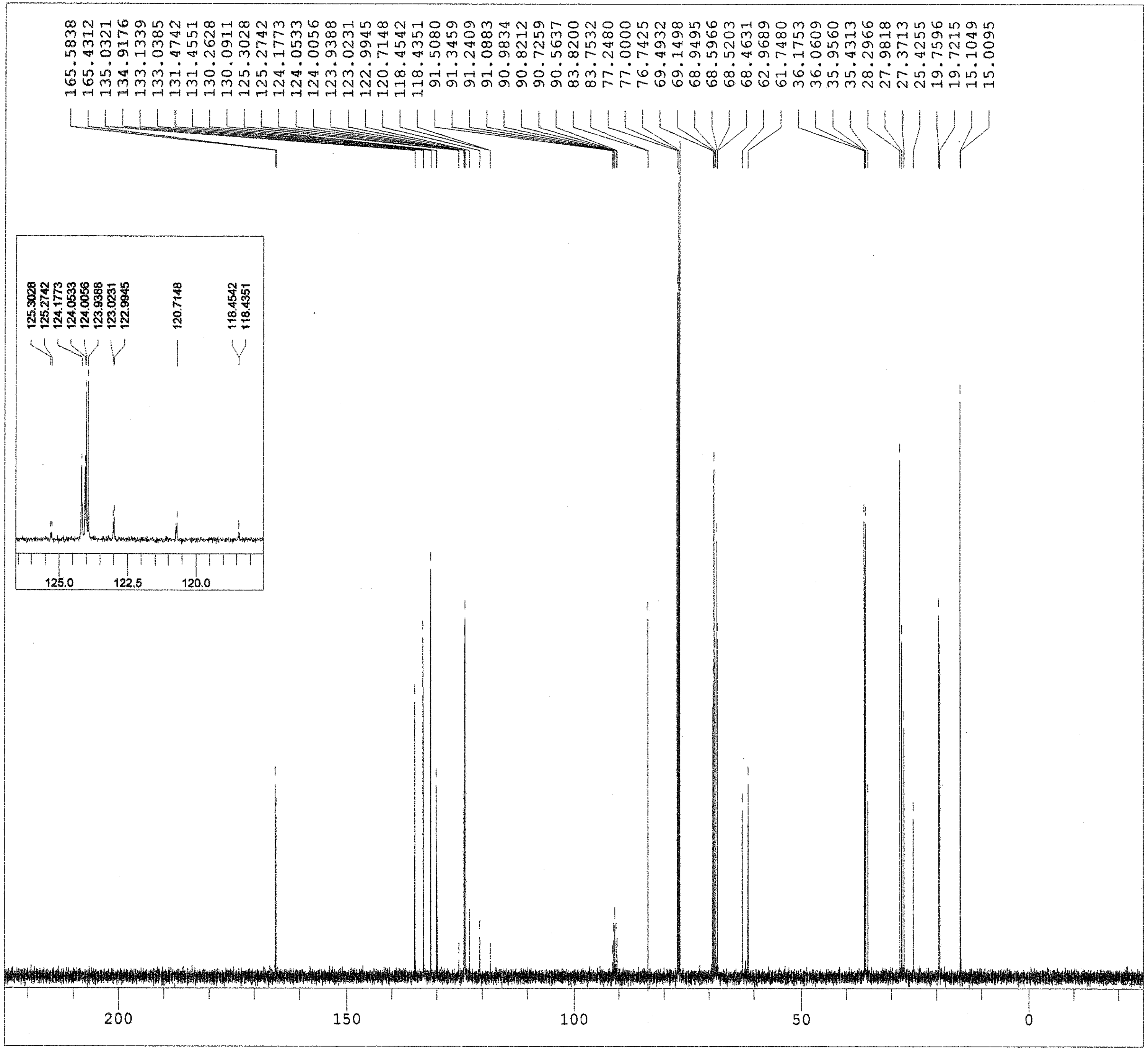

DFILE	ozawa07-099_13C.als
COMNT	3-Me-5-O(4-pentyne), OH
DATIM	2015-05-01 10:26:57
OBNUC	13C
EXMOD	carbon.jxp
OBFRQ	125.77 MHz
OBSET	7.87 KHz
OBFIN	4.21 Hz
POINT	26214
FREQU	31446.54 Hz
SCANS	444
ACQTM	0.8336 sec
PD	2.0000 sec
PW1	3.40 usec
IRNUC	1H
CTEMP	21.8 c
SLVNT	CDCL3
EXREF	77.00 ppm
BF	0.12 Hz
RGAIN	60

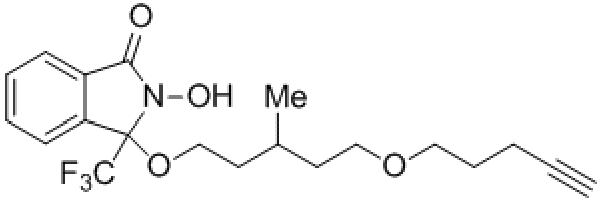

1o

tert-yne, OH

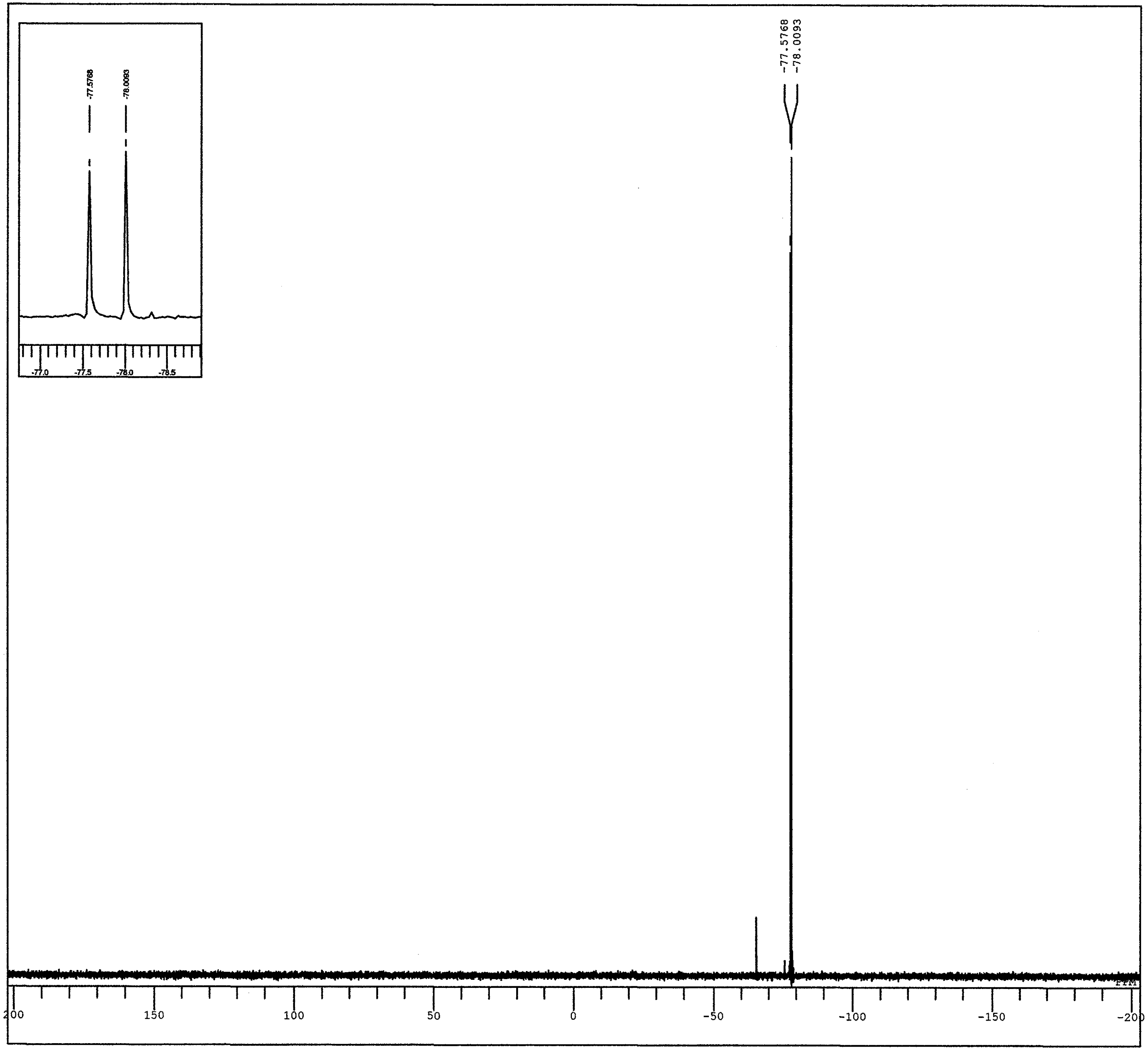

DFILE ozawa07-099_19F.jdf
COMNT tert-yne, OH
DATIM 11-05-2015 15:33:38
OBNUC 19F
EXMOD proton.jxp
OBFRQ 368.64 MHz
OBSET 7.63 KHz
OBFIN 2.85 Hz
POINT 16384
FREQU 186567.17 Hz
SCANS 8
ACQTM 0.0878 sec
PD 5.0000 sec
PW1 3.90 usec
IRNUC 19F
CTEMP 21.6 c
SLVNT CDCL3
EXREF -164.90 ppm
BF 0.12 Hz
RGAIN 50

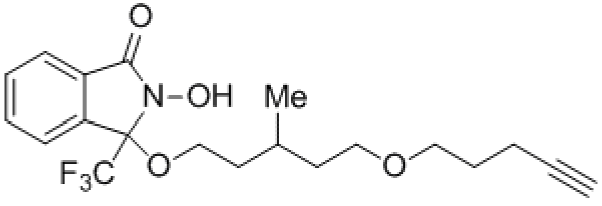

1o

tert-yne, [0]

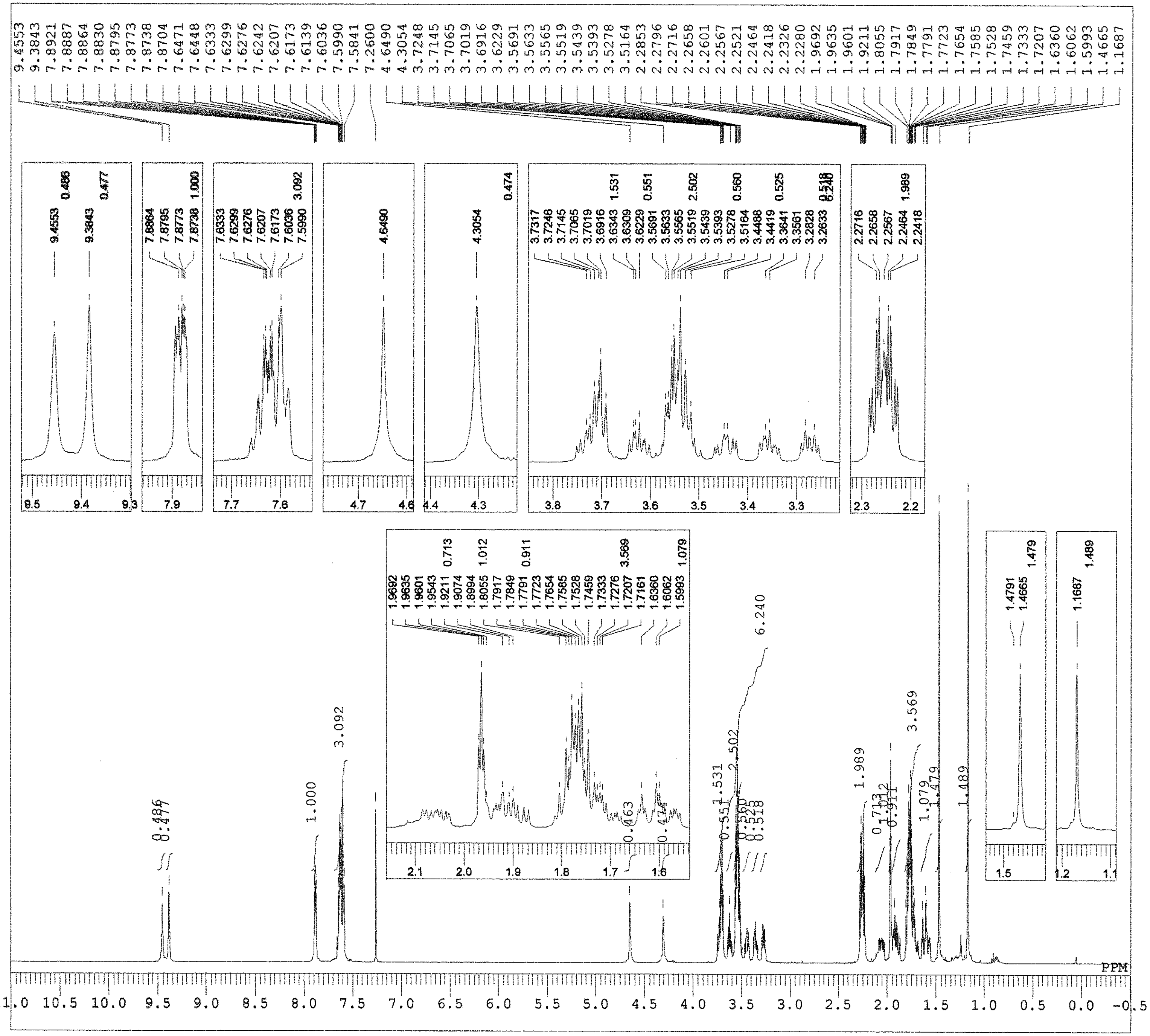

DFILE	ozawa07-102_PTLC_1H.jdf
COMNT	tert-yne, [O]
DATIM	2015-05-11 15:01:17
OBNUC	1H
EXMOD	proton.jxp
OBFRQ	500.16 MHz
OBSET	2.41 KHz
OBFIN	6.01 Hz
POINT	16384
FREQU	9384.38 Hz
SCANS	8
ACQTM	1.7459 sec
PD	5.0000 sec
PW1	5.55 usec
IRNUC	1H
CTEMP	21.3 c
SLVNT	CDCL3
EXREF	7.26 ppm
BF	0.12 Hz
RGAIN	30

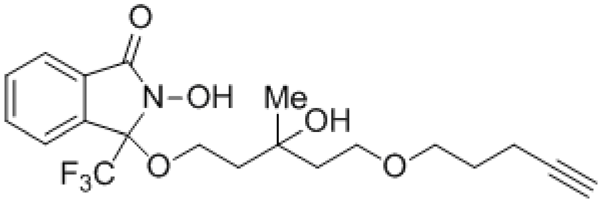

2o

tert-yne, [O]

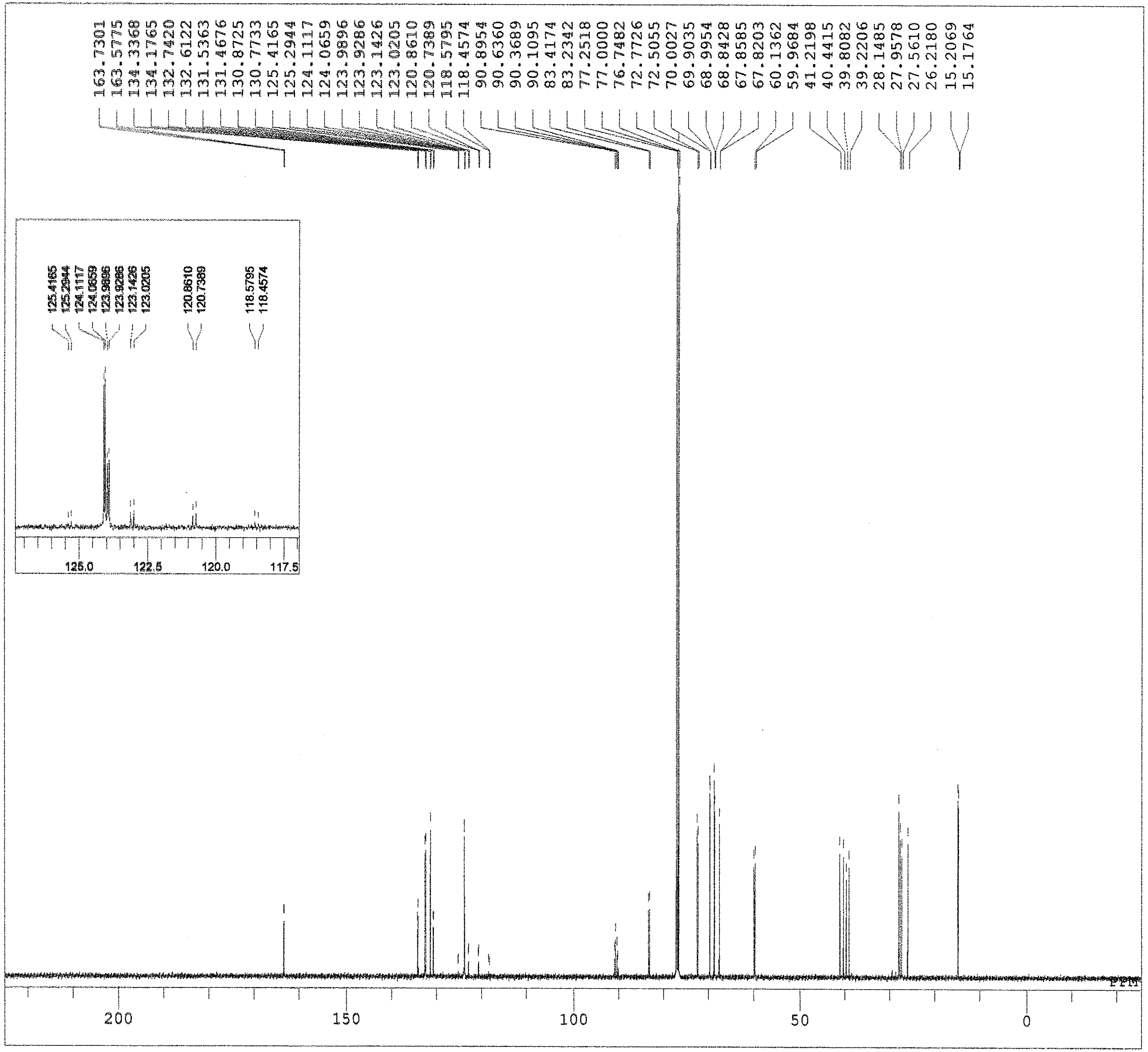

DFILE	ozawa07-102_13C.jdf
COMNT	tert-yne, [O]
DATIM	2015-05-12 17:50:02
OBNUC	13C
EXMOD	carbon.jxp
OBFRQ	125.77 MHz
OBSET	7.87 KHz
OBFIN	4.21 Hz
POINT	32768
FREQU	31446.54 Hz
SCANS	2662
ACQTM	1.0420 sec
PD	2.0000 sec
PW1	3.40 usec
IRNUC	1H
CTEMP	21.8 c
SLVNT	CDCL3
EXREF	77.00 ppm
BF	0.42 Hz
RGAIN	72

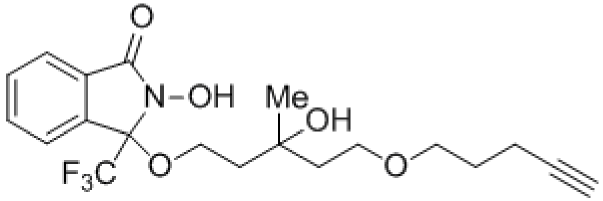

2o

tert-yne, [O]

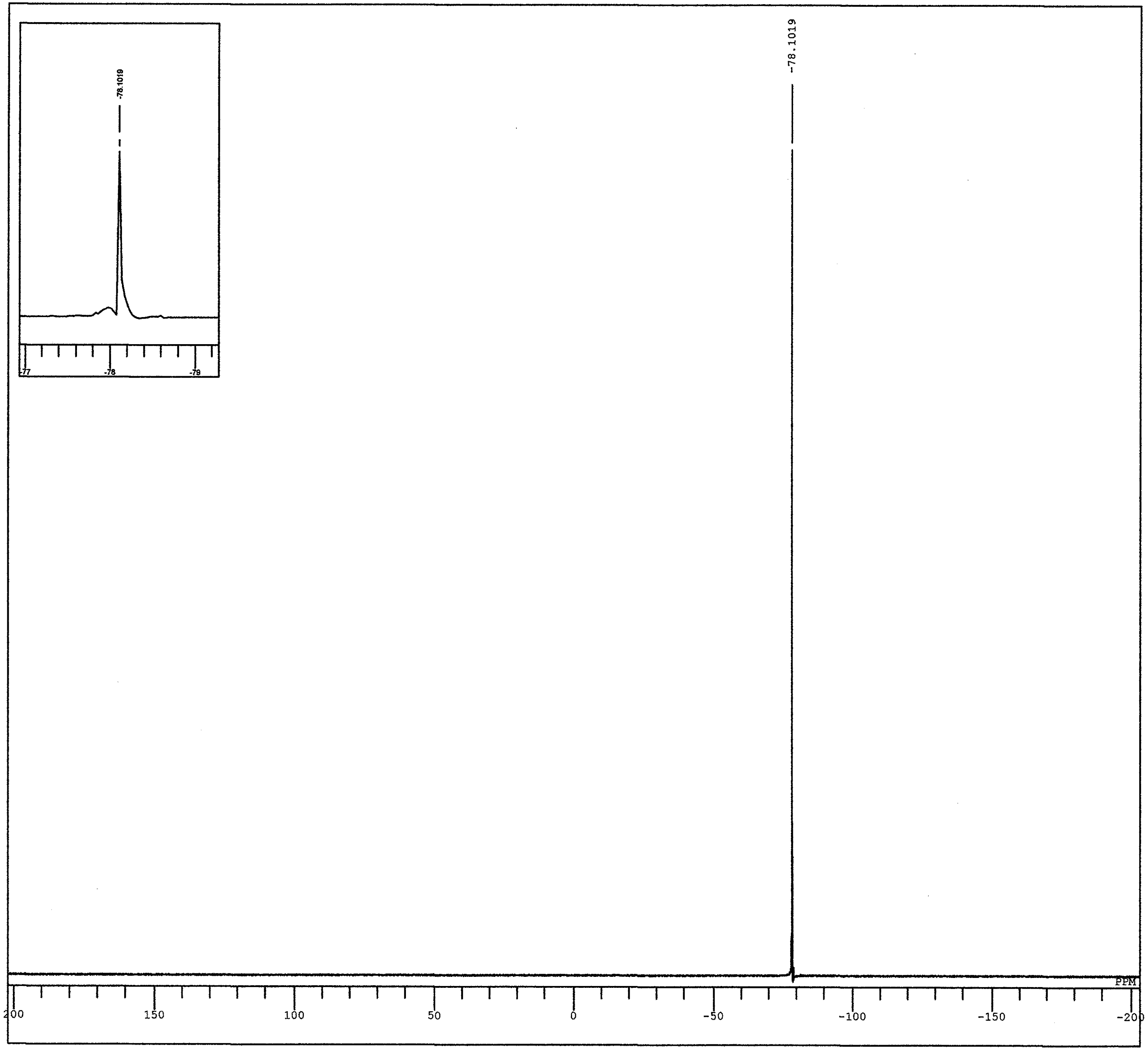


```
DFILE  ozawa07-102_19F.jdf
COMNT  tert-yne, [O]
DATIM  11-05-2015 15:37:59
OBNUC  19F
EXMOD  proton.jxp
OBFRQ        368.64 MHz
OBSET          7.63 KHz
OBFIN          2.85 Hz
POINT         16384
FREQU     186567.17 Hz
SCANS             8
ACQTM        0.0878 sec
PD           5.0000 sec
PW1            3.90 usec
IRNUC  19F
CTEMP          21.6 c
SLVNT  CDCL3
EXREF       -164.90 ppm
BF             0.12 Hz
RGAIN            50
```

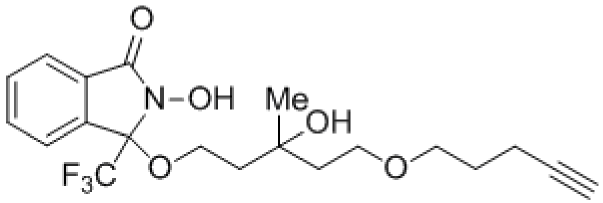

2o

3-Me-7-octenol

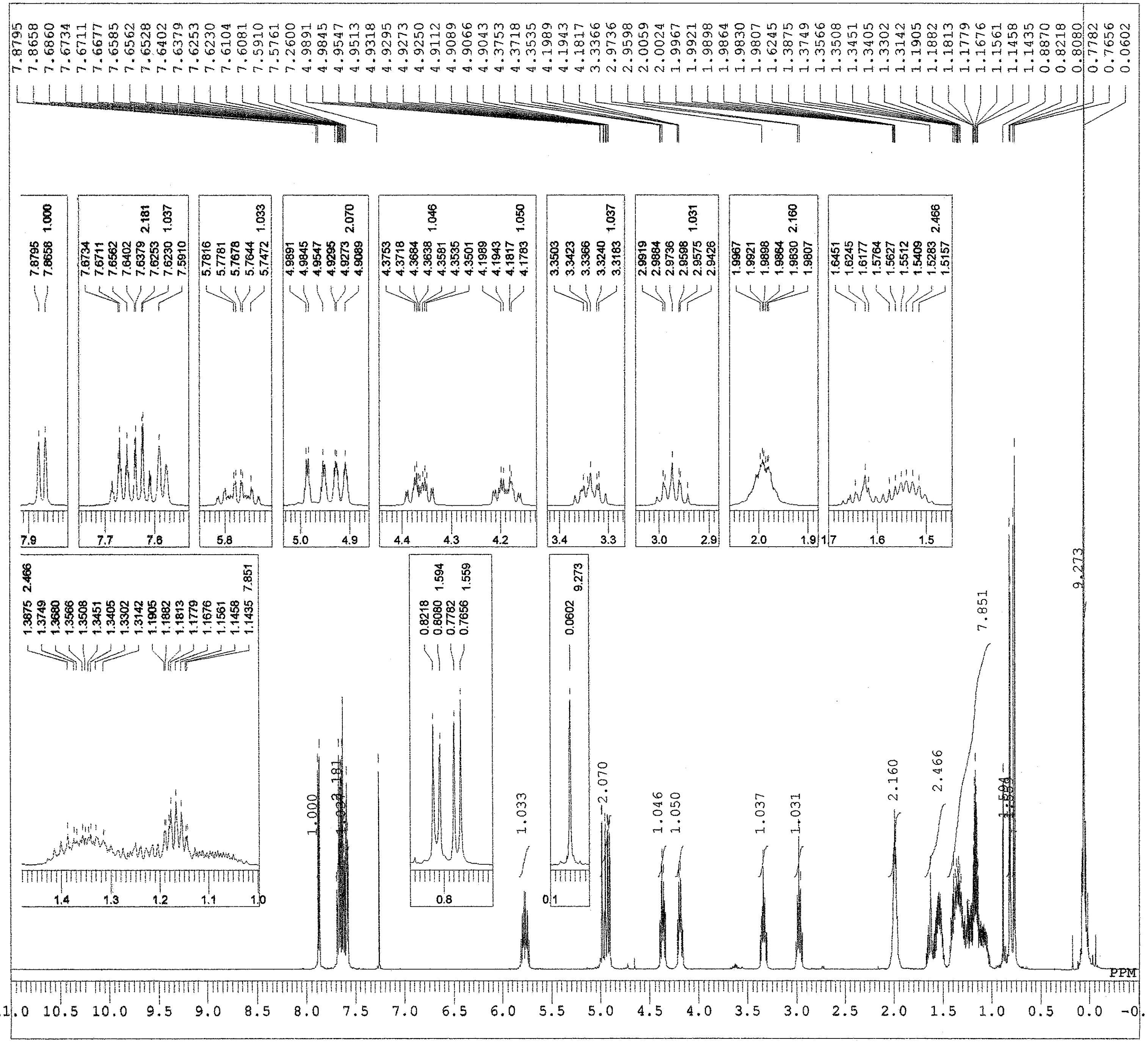

DFILE ozawa06-065_1H.jdf
COMNT 3-Me-7-octenol
DATIM 2014-12-05 15:50:55
OBNUC 1H
EXMOD proton.jxp
OBFRQ 500.16 MHz
OBSET 2.41 KHz
OBFIN 6.01 Hz
POINT 16384
FREQU 9384.38 Hz
SCANS 8
ACQTM 1.7459 sec
PD 5.0000 sec
PW1 5.55 usec
IRNUC 1H
CTEMP 25.5 c
SLVNT CDCL3
EXREF 7.26 ppm
BF 0.12 Hz
RGAIN 28

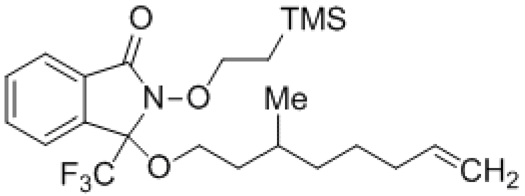

S4"p

3-Me-7-octenol

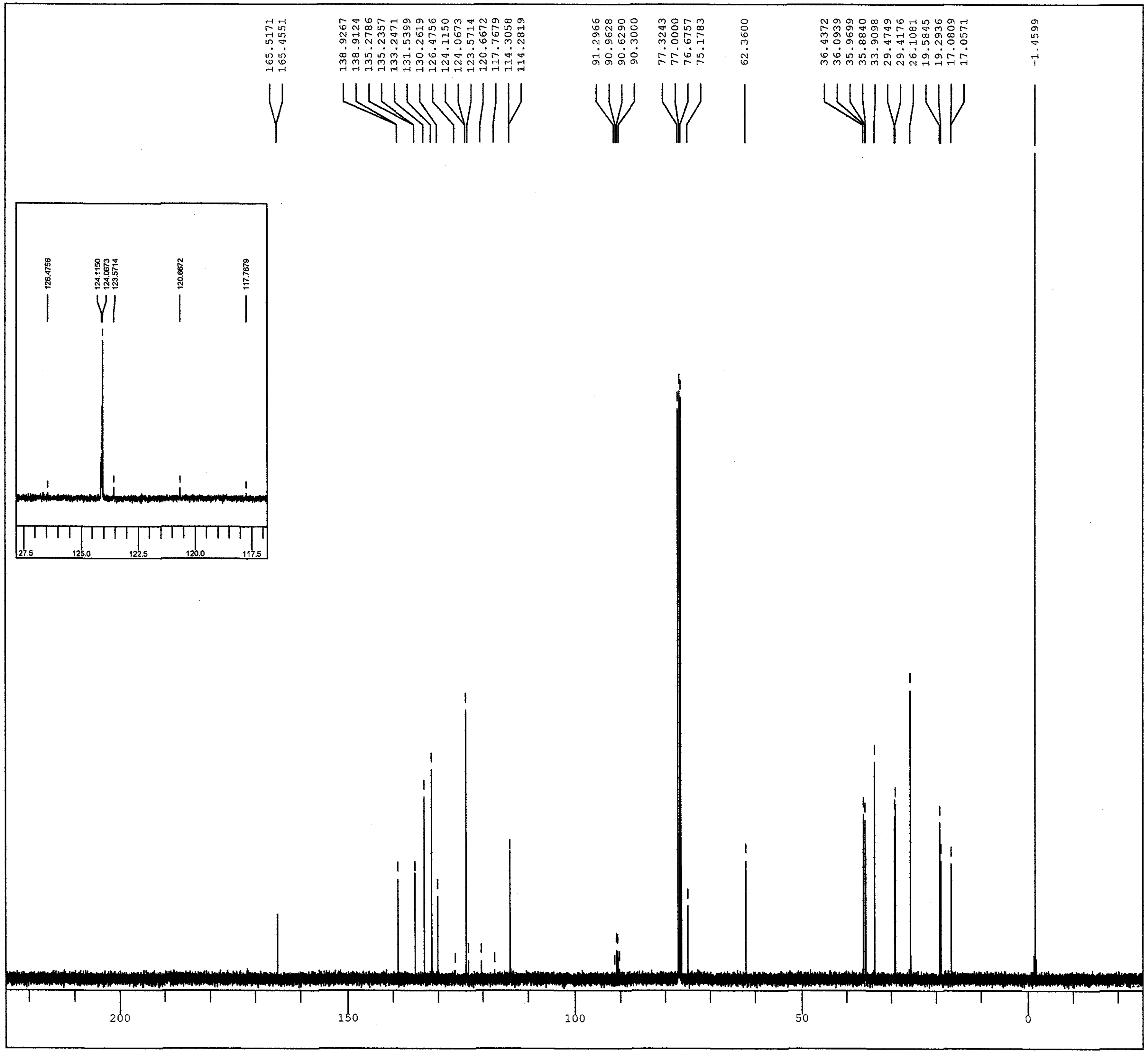

DFILE ozawa06-065_13C.jdf
COMNT 3-Me-7-octenol
DATIM 06-12-2014 07:15:47
OBNUC 13C
EXMOD carbon.jxp
OBFRQ 98.52 MHz
OBSET 4.64 KHz
OBFIN 8.74 Hz
POINT 65535
FREQU 30788.18 Hz
SCANS 452
ACQTM 2.1286 sec
PD 2.0000 sec
PW1 3.07 usec
IRNUC 1H
CTEMP 22.3 c
SLVNT CDCL3
EXREF 77.00 ppm
BF 0.12 Hz
RGAIN 60

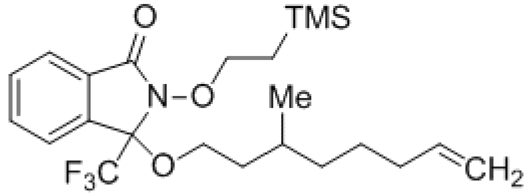

S4"p

3-Me-7-octenol

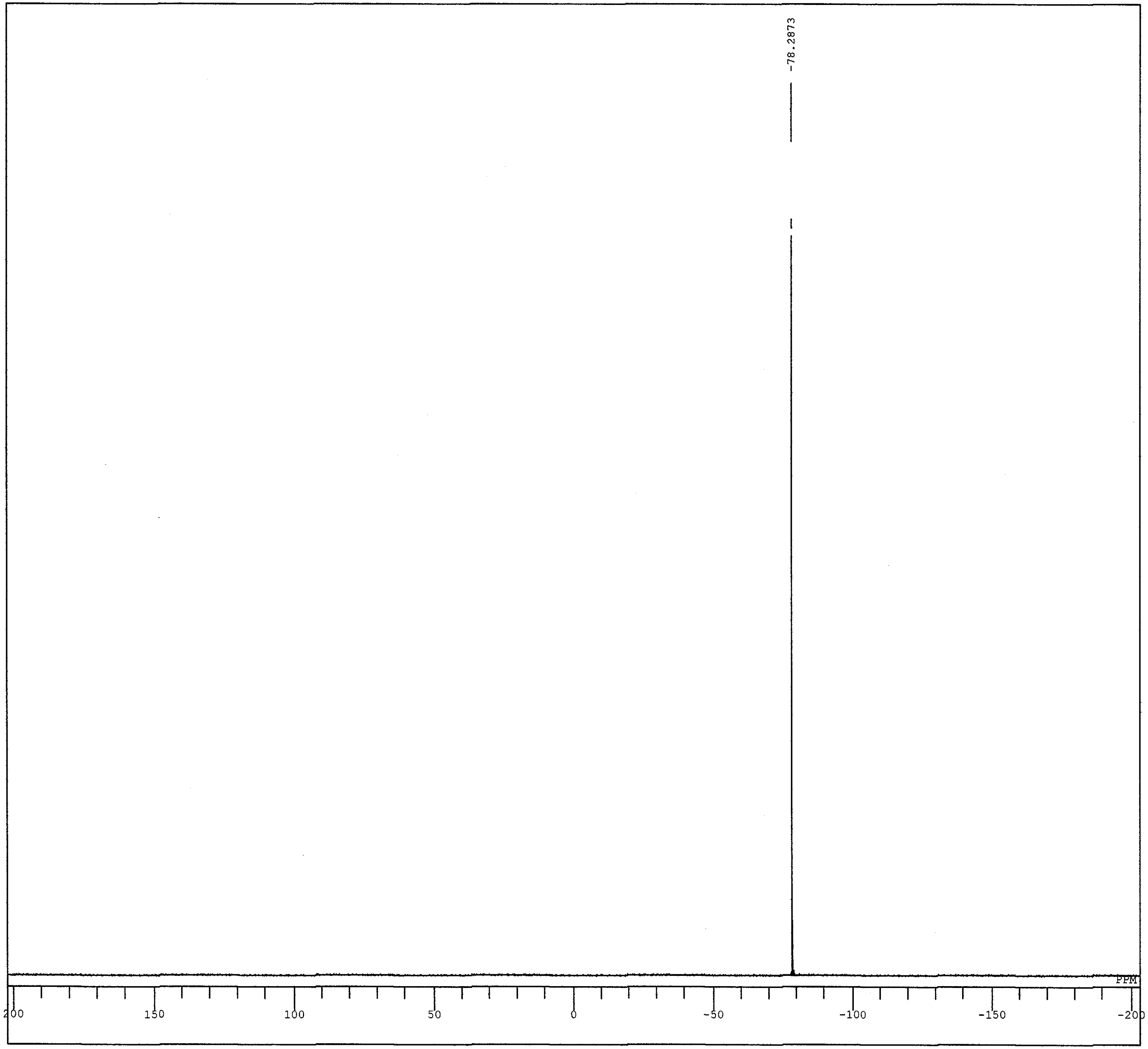

DFILE ozawa06-065_19F.jdf
COMNT 3-Me-7-octenol
DATIM 06-12-2014 07:48:48
OBNUC 19F
EXMOD proton.jxp
OBFRQ 368.64 MHz
OBSET 7.63 KHz
OBFIN 2.85 Hz
POINT 16384
FREQU 186567.17 Hz
SCANS 4
ACQTM 0.0878 sec
PD 5.0000 sec
PW1 3.90 usec
IRNUC 19F
CTEMP 22.1 c
SLVNT CDCL3
EXREF -164.90 ppm
BF 0.12 Hz
RGAIN 48

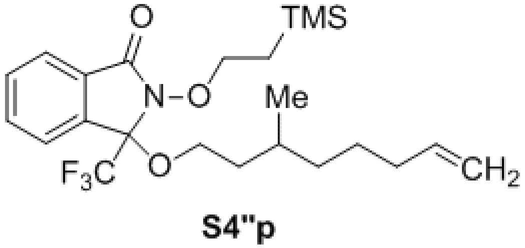

S4"p

3-Me-7-octenol, OH

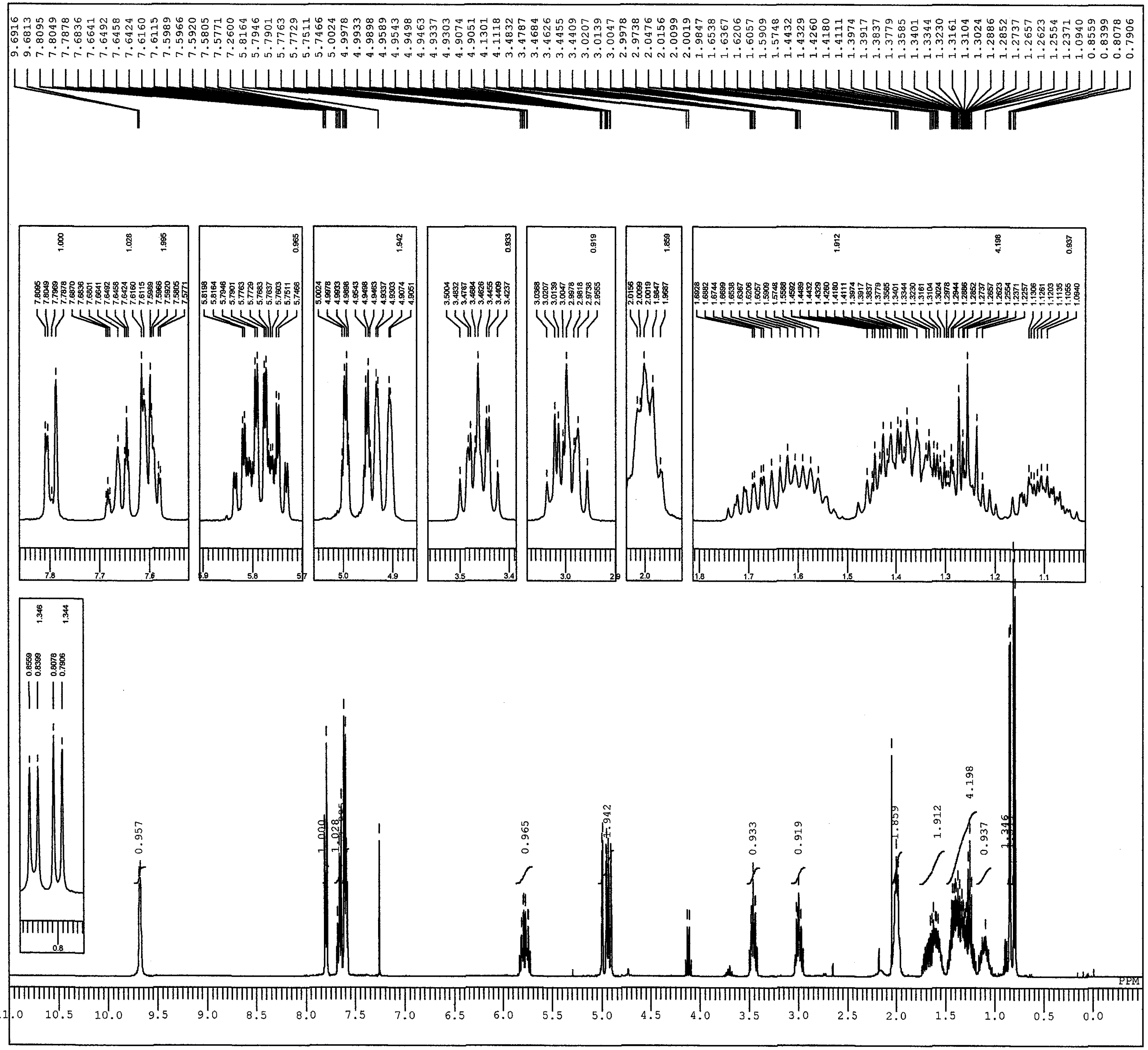

DFILE ozawa06-071_1H.jdf
COMNT 3-Me-7-octenol, OH
DATIM 06-12-2014 08:43:00
OBNUC 1H
EXMOD proton.jxp
OBFRQ 391.78 MHz
OBSET 8.51 KHz
OBFIN 3.34 Hz
POINT 16384
FREQU 7348.62 Hz
SCANS 4
ACQTM 2.2295 sec
PD 5.0000 sec
PW1 5.07 usec
IRNUC 1H
CTEMP 21.9 c
SLVNT CDCL3
EXREF 7.26 ppm
BF 0.12 Hz
RGAIN 30

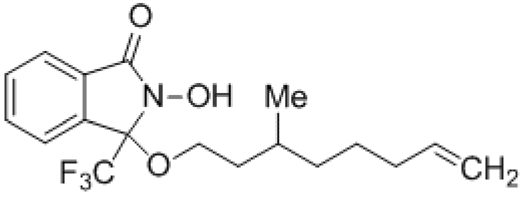

1p

3-Me-7-octenol, OH

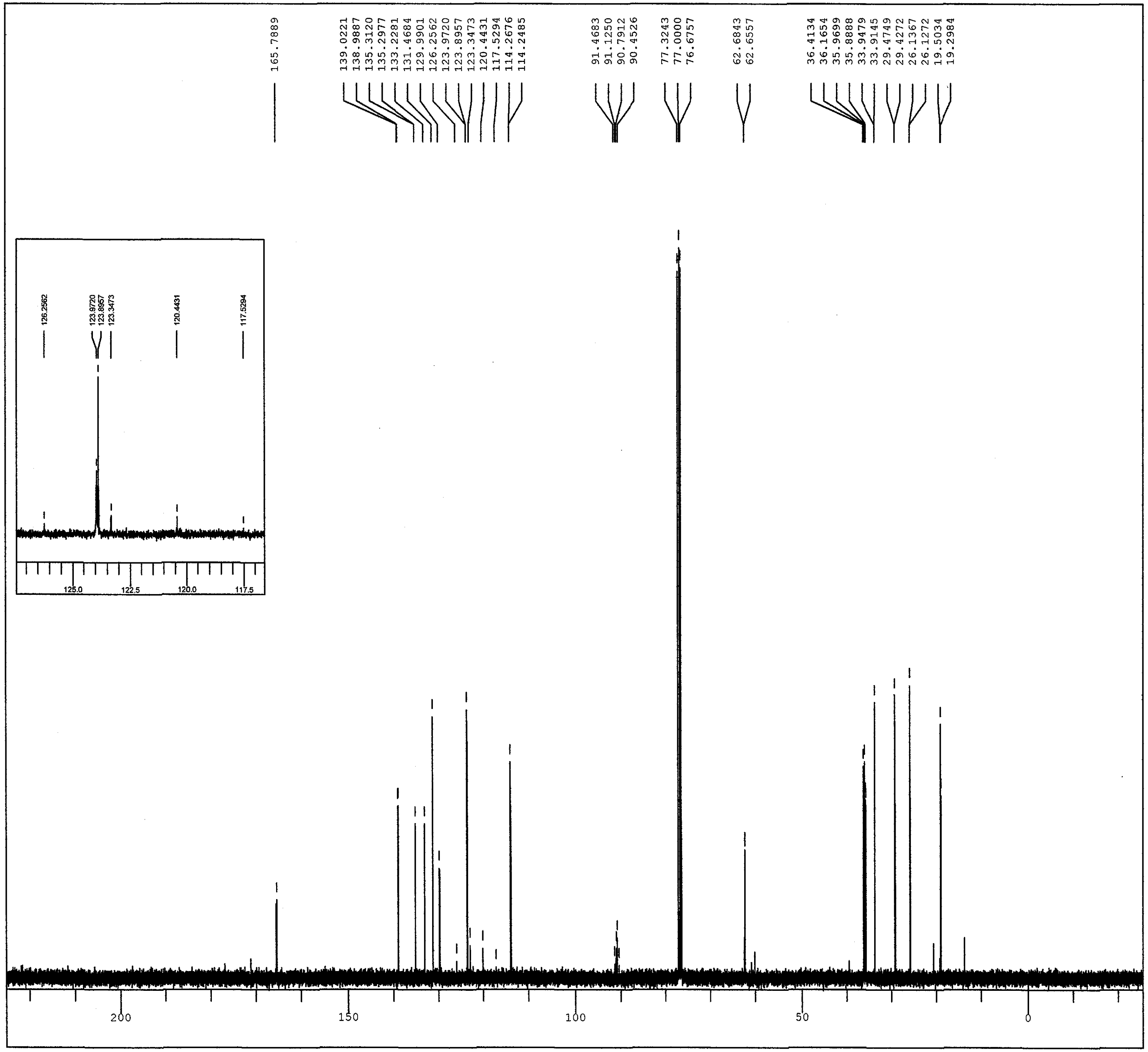

DFILE ozawa06-071_13C.jdf
COMNT 3-Me-7-octenol, OH
DATIM 06-12-2014 08:44:07
OBNUC 13C
EXMOD carbon.jxp
OBFRQ 98.52 MHz
OBSET 4.64 KHz
OBFIN 8.74 Hz
POINT 65535
FREQU 30788.18 Hz
SCANS 404
ACQTM 2.1286 sec
PD 2.0000 sec
PW1 3.07 usec
IRNUC 1H
CTEMP 22.3 c
SLVNT CDCL3
EXREF 77.00 ppm
BF 0.12 Hz
RGAIN 60

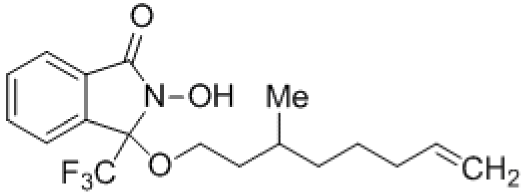

1p

3-Me-7-octenol, OH

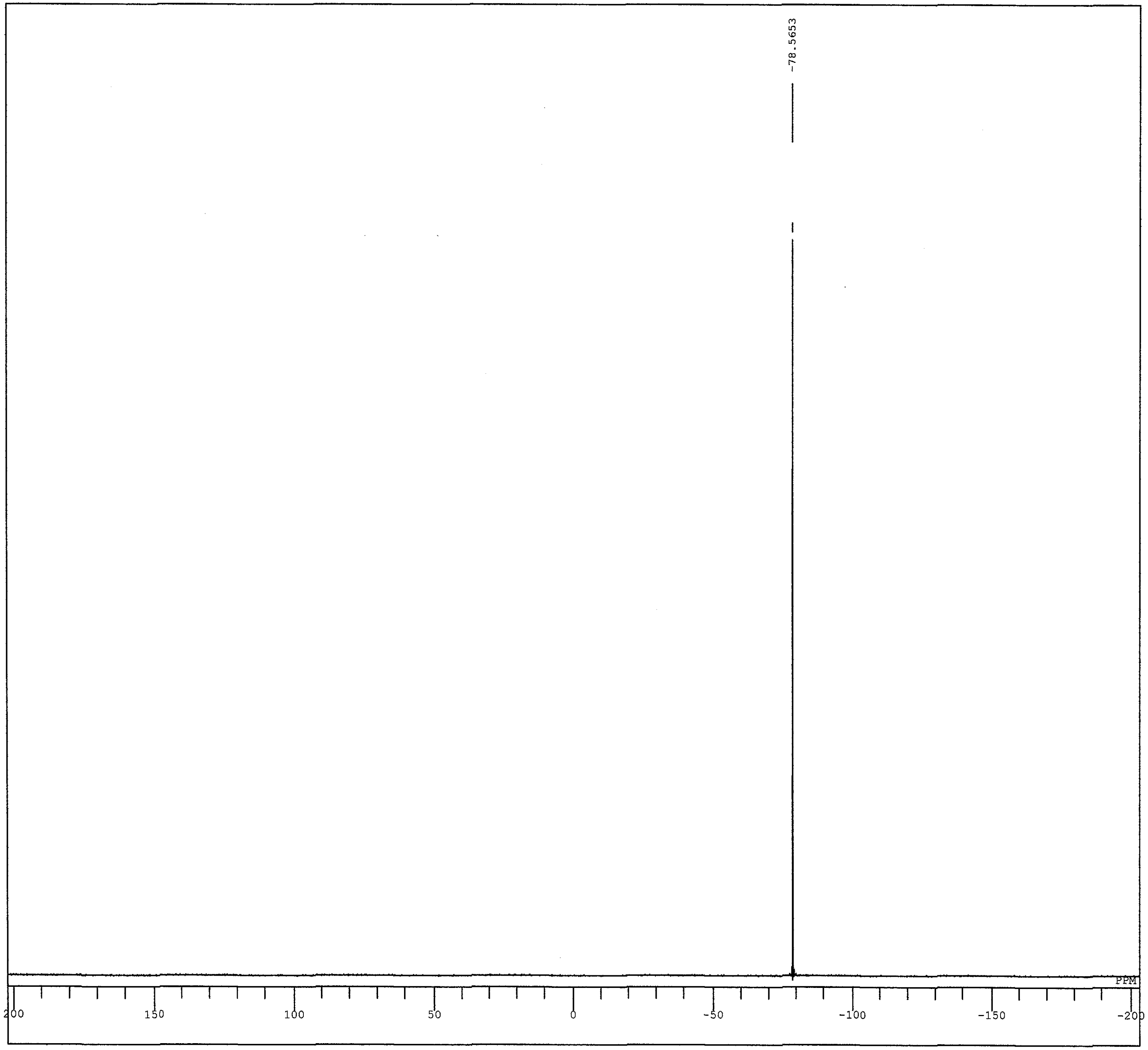


```
DFILE  ozawa06-071_19F.jdf
COMNT  3-Me-7-octenol, OH
DATIM  06-12-2014 09:13:50
OBNUC  19F
EXMOD  proton.jxp
OBFRQ        368.64 MHz
OBSET          7.63 KHz
OBFIN          2.85 Hz
POINT         16384
FREQU     186567.17 Hz
SCANS             4
ACQTM        0.0878 sec
PD           5.0000 sec
PW1            3.90 usec
IRNUC  19F
CTEMP          22.1 c
SLVNT  CDCL3
EXREF       -164.90 ppm
BF             0.12 Hz
RGAIN            46
```

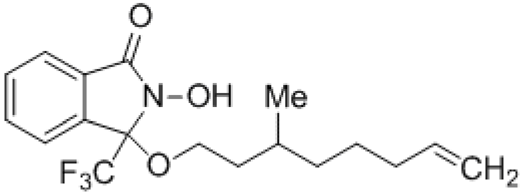

1p

3-Me-7-octenol, [O]

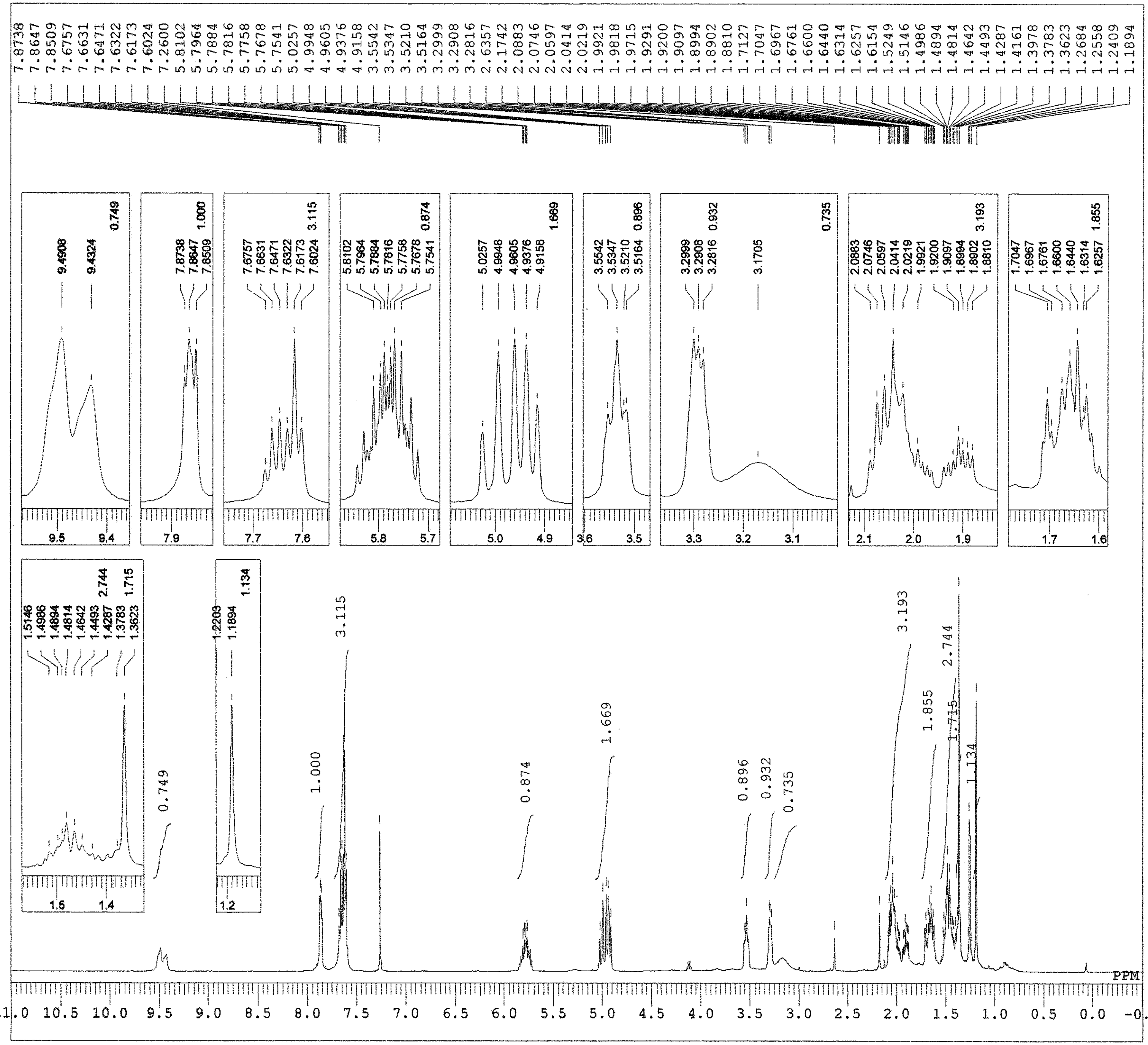

DFILE ozawa06-087_1H.jdf
COMNT 3-Me-7-octenol, [O]
DATIM 2014-12-06 16:17:41
OBNUC 1H
EXMOD proton.jxp
OBFRQ 500.16 MHz
OBSET 2.41 KHz
OBFIN 6.01 Hz
POINT 16384
FREQU 9384.38 Hz
SCANS 8
ACQTM 1.7459 sec
PD 5.0000 sec
PW1 5.55 usec
IRNUC 1H
CTEMP 25.3 c
SLVNT CDCL3
EXREF 7.26 ppm
BF 1.20 Hz
RGAIN 30

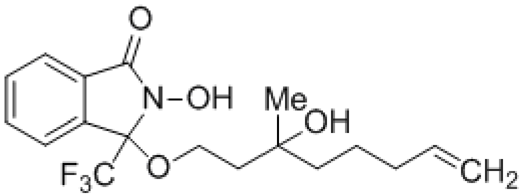

2p

3-Me-7-octenol, [O]

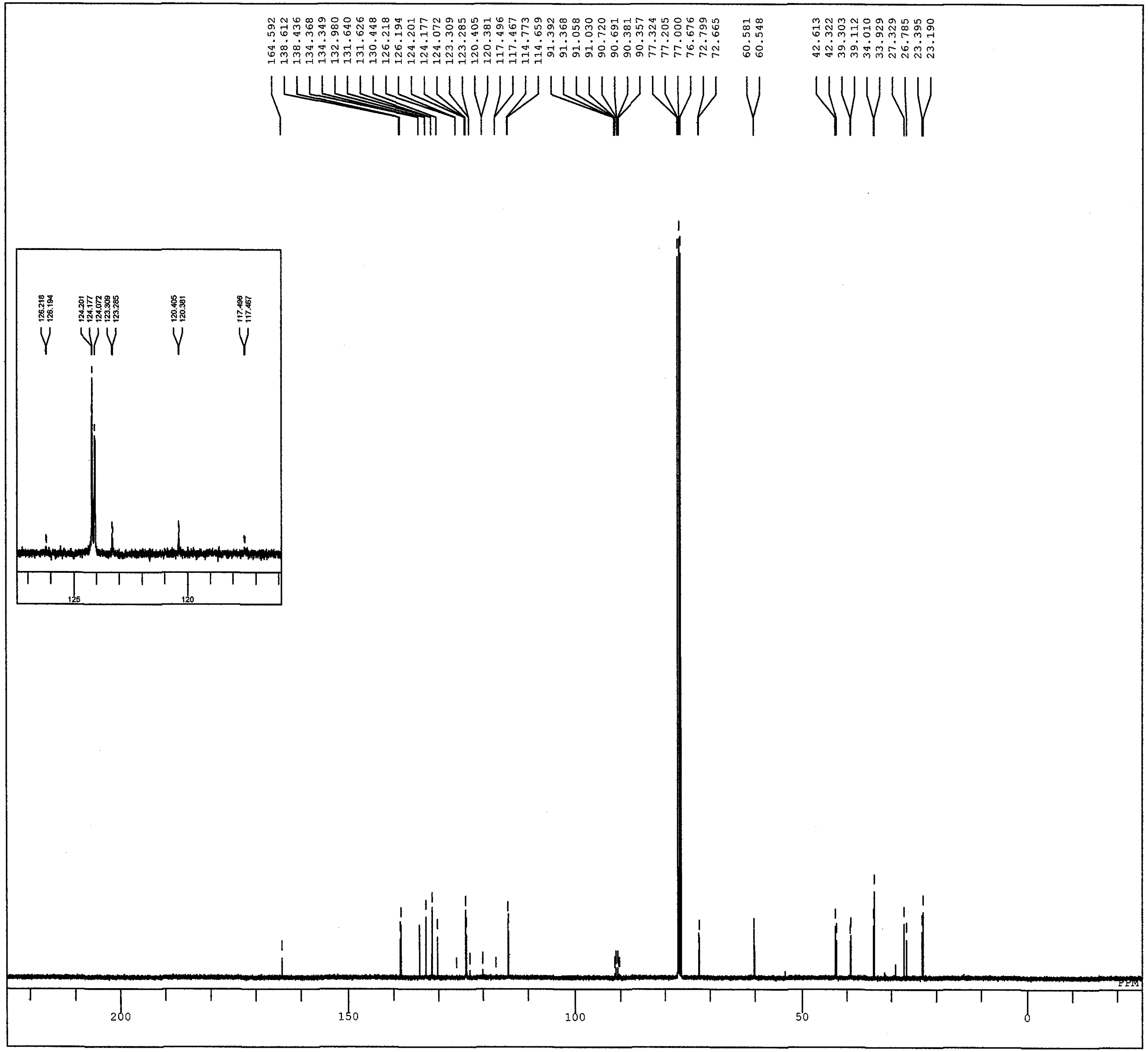

DFILE ozawa060-087_13C.jdf
COMNT 3-Me-7-octenol, [O]
DATIM 12-12-2014 19:14:28
OBNUC 13C
EXMOD carbon.jxp
OBFRQ 98.52 MHz
OBSET 4.64 KHz
OBFIN 8.74 Hz
POINT 65535
FREQU 30788.18 Hz
SCANS 10400
ACQTM 2.1286 sec
PD 2.0000 sec
PW1 3.07 usec
IRNUC 1H
CTEMP 22.7 c
SLVNT CDCL3
EXREF 77.00 ppm
BF 0.12 Hz
RGAIN 60

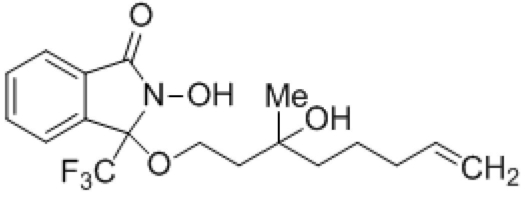

2p

3-Me-7-octenol, [0]

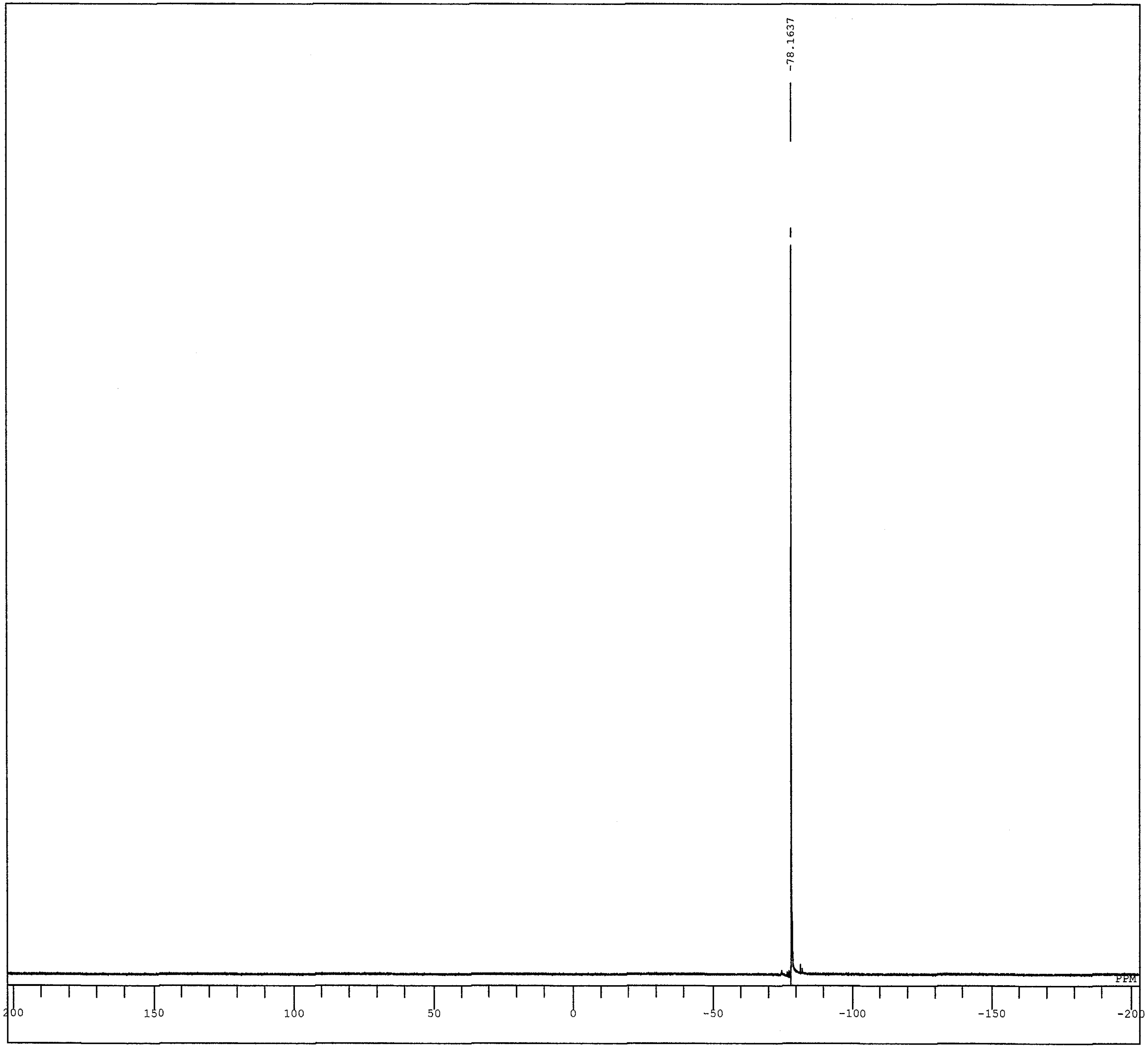

DFILE ozawa06-087_19F.jdf
COMNT 3-Me-7-octenol, [O]
DATIM 09-12-2014 09:04:40
OBNUC 19F
EXMOD proton.jxp
OBFRQ 368.64 MHz
OBSET 7.63 KHz
OBFIN 2.85 Hz
POINT 16384
FREQU 186567.17 Hz
SCANS 12
ACQTM 0.0878 sec
PD 5.0000 sec
PW1 3.90 usec
IRNUC 19F
CTEMP 22.1 c
SLVNT CDCL3
EXREF -164.90 ppm
BF 0.12 Hz
RGAIN 50

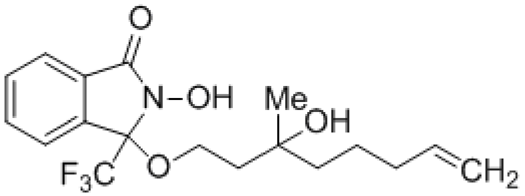

2p

alkyl boronic acid ester, Bn

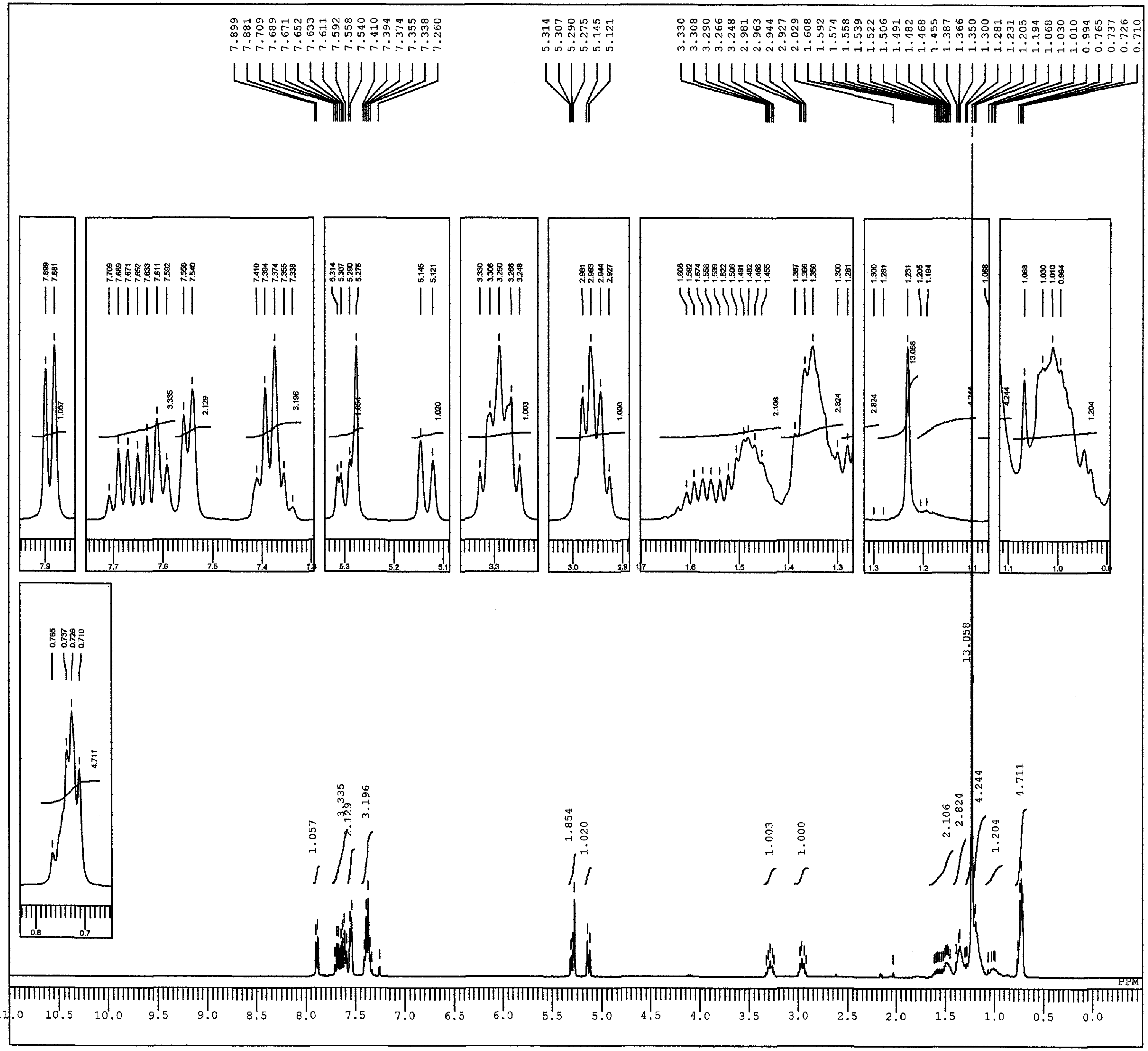

DFILE ozawa06-106_1H.als
COMNT alkyl boronic acid ester, Bn
DATIM 18-12-2014 12:55:37
OBNUC 1H
EXMOD proton.jxp
OBFRQ 391.78 MHz
OBSET 8.51 KHz
OBFIN 3.34 Hz
POINT 13107
FREQU 5878.90 Hz
SCANS 4
ACQTM 2.2295 sec
PD 5.0000 sec
PW1 5.07 usec
IRNUC 1H
CTEMP 21.8 c
SLVNT CDCL3
EXREF 7.26 ppm
BF 1.00 Hz
RGAIN 22

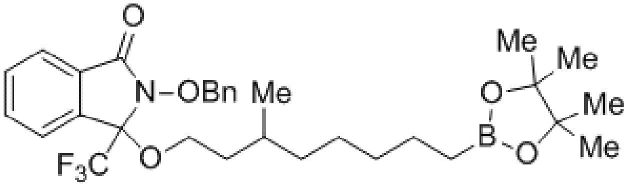

S4q

alkyl boronic acid ester, Bn

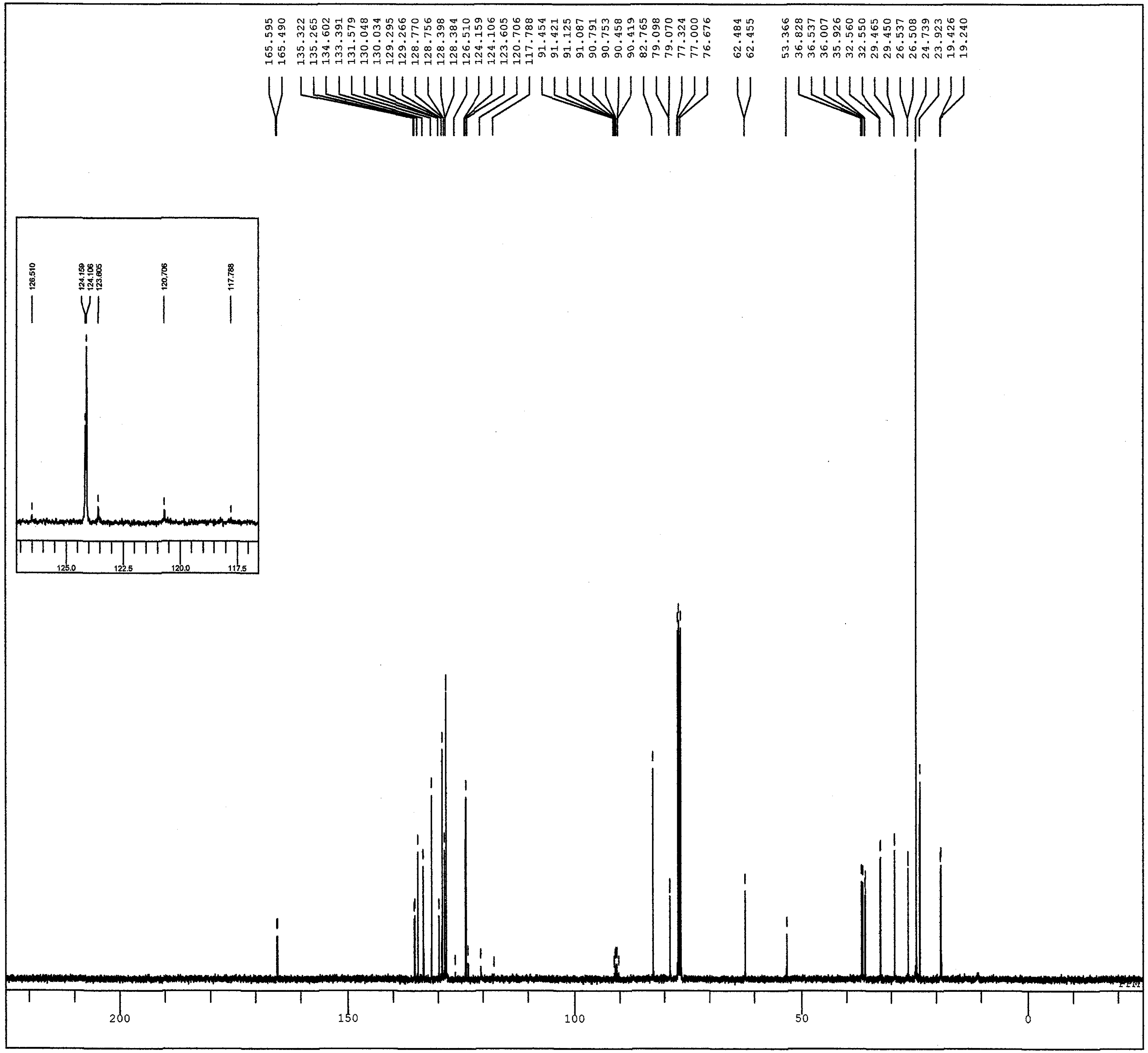

DFILE	ozawa06-106_13C.als
COMNT	alkyl boronic acid ester, Bn
DATIM	18-12-2014 12:56:45
OBNUC	13C
EXMOD	carbon.jxp
OBFRQ	98.52 MHz
OBSET	4.64 KHz
OBFIN	8.74 Hz
POINT	52428
FREQU	24630.54 Hz
SCANS	200
ACQTM	2.1286 sec
PD	2.0000 sec
PW1	3.07 usec
IRNUC	1H
CTEMP	22.1 c
SLVNT	CDCL3
EXREF	77.00 ppm
BF	1.00 Hz
RGAIN	60

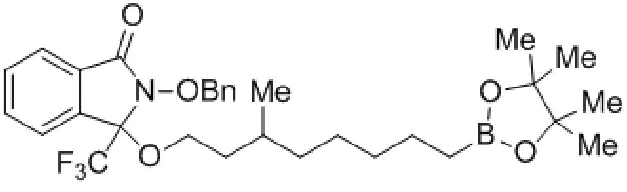

S4q

alkyl-Bpin, Bn

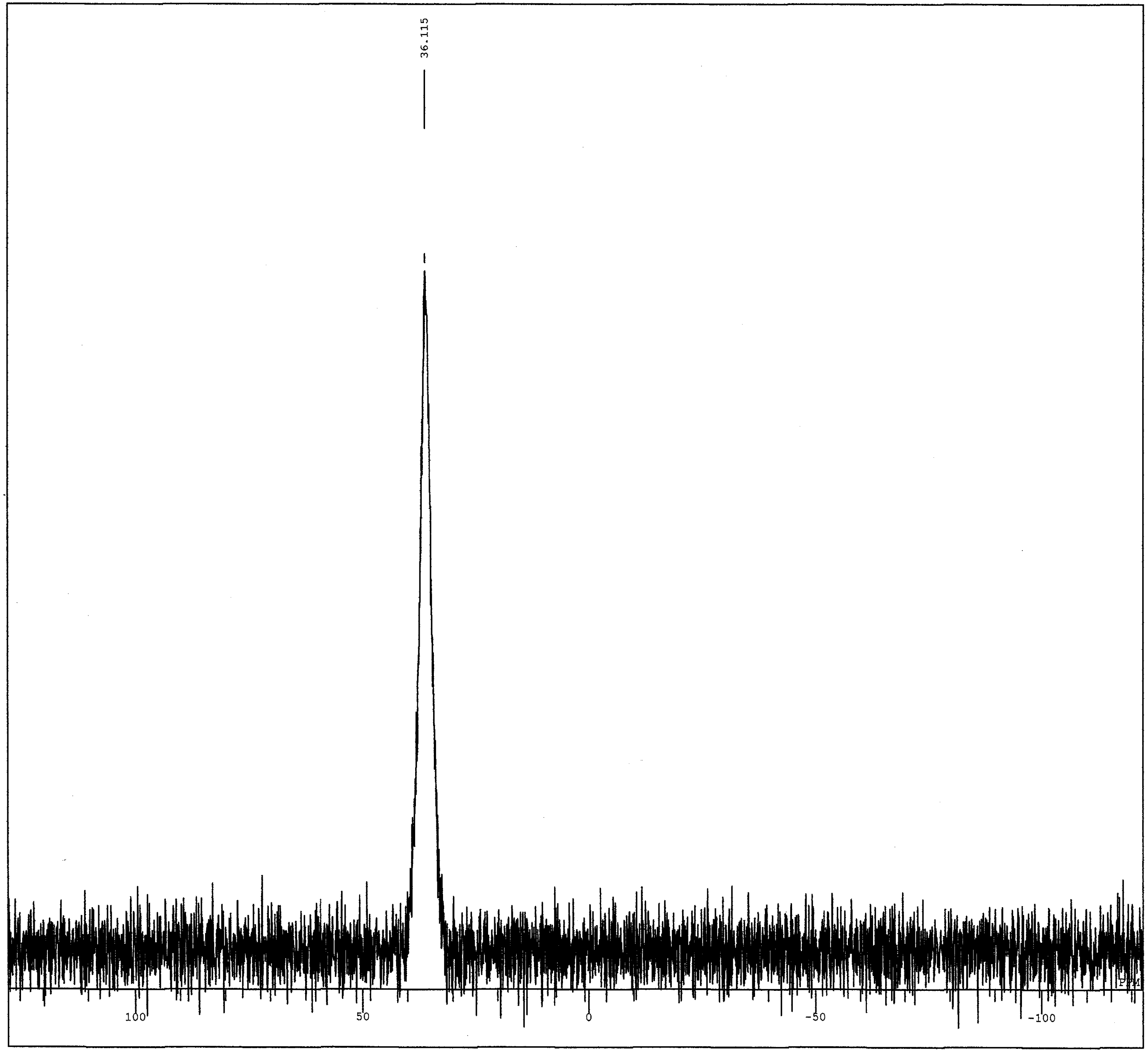

DFILE ozawa06-106_11B.jdf
COMNT alkyl-Bpin, Bn
DATIM 30-12-2014 09:58:14
OBNUC 11B
EXMOD carbon.jxp
OBFRQ 125.70 MHz
OBSET 0.81 KHz
OBFIN 3.25 Hz
POINT 4095
FREQU 39308.18 Hz
SCANS 1000
ACQTM 0.1042 sec
PD 80.0000 sec
PW1 3.98 usec
IRNUC 1H
CTEMP 21.8 c
SLVNT CDCL3
EXREF 31.00 ppm
BF 0.12 Hz
RGAIN 60

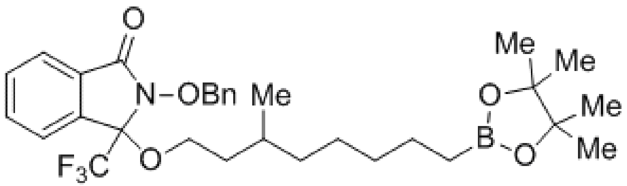

S4q

alkyl boronic acid esterm, Bn

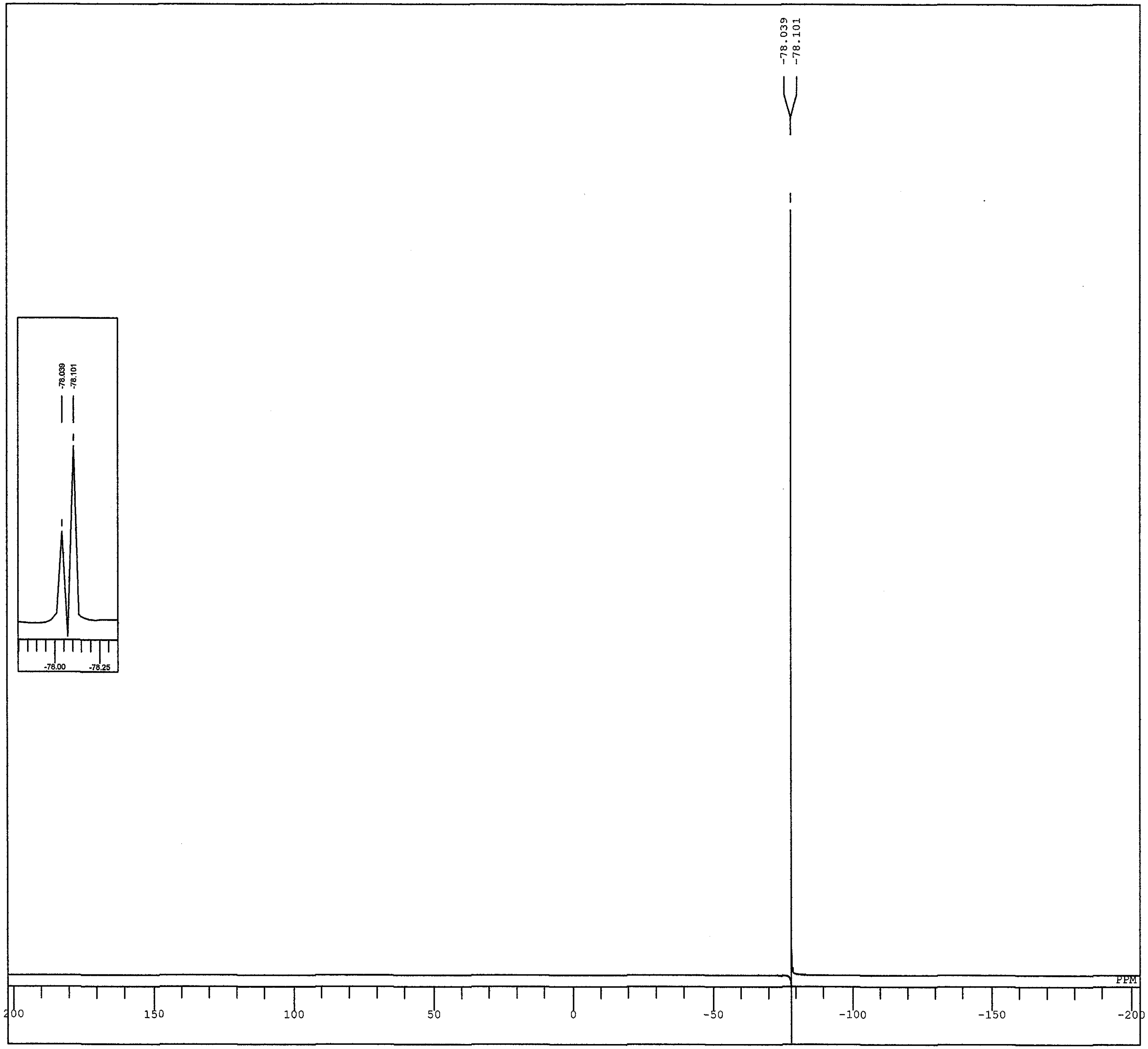

DFILE ozawa06-106_19F.als
COMNT alkyl boronic acid esterm, Bn
DATIM 18-12-2014 12:52:45
OBNUC 19F
EXMOD proton.jxp
OBFRQ 368.64 MHz
OBSET 7.63 KHz
OBFIN 2.85 Hz
POINT 13107
FREQU 149253.73 Hz
SCANS 8
ACQTM 0.0878 sec
PD 5.0000 sec
PW1 3.90 usec
IRNUC 19F
CTEMP 21.8 c
SLVNT CDCL3
EXREF -164.90 ppm
BF 1.00 Hz
RGAIN 48

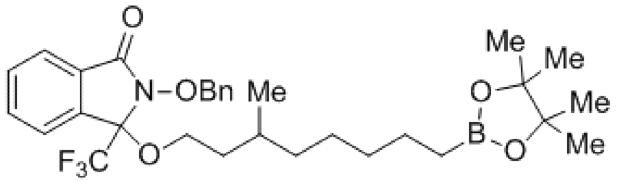

S4q

alkyl boronic acid ester, OH

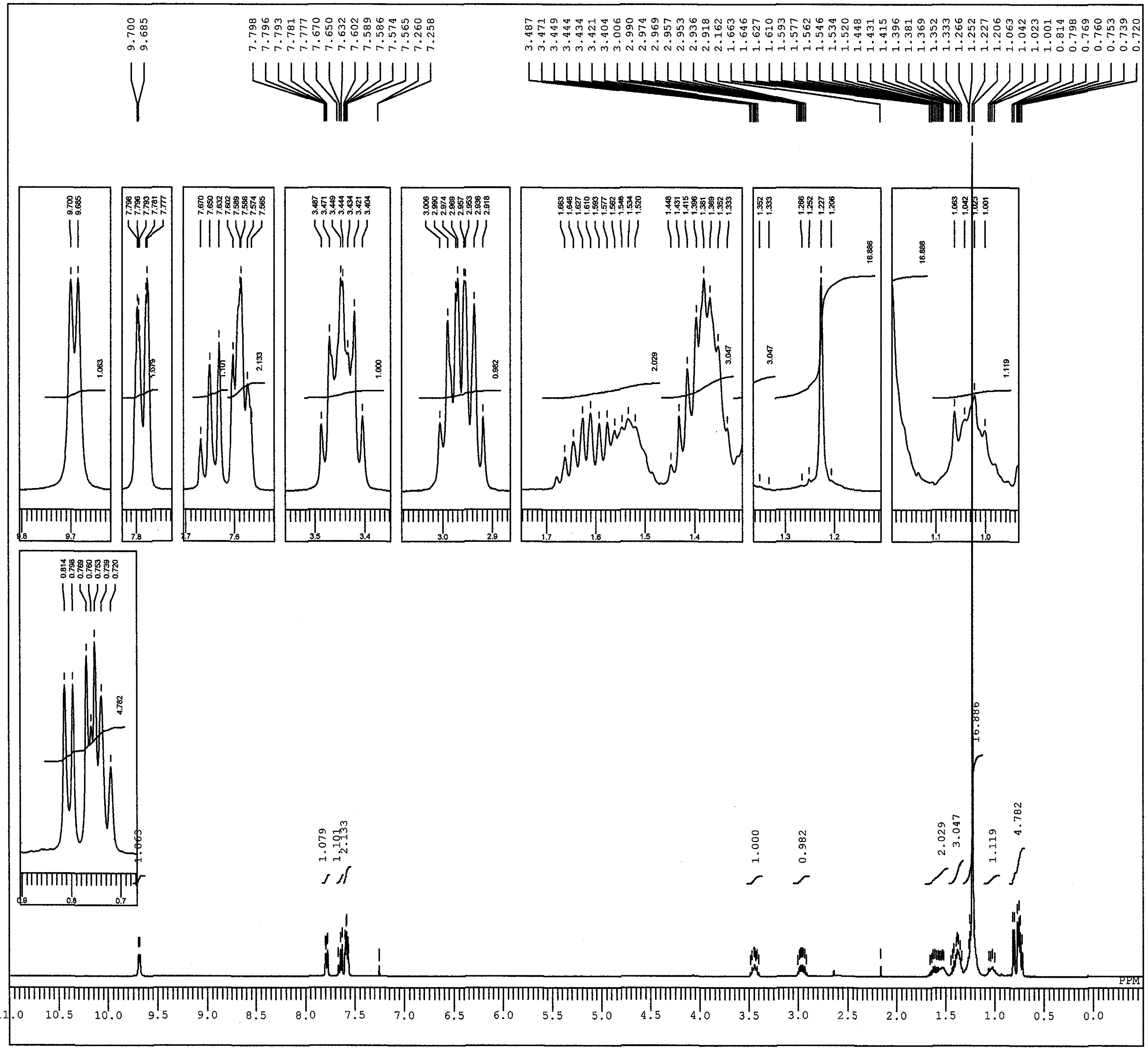

DFILE ozawa06-107_1H.jdf
COMNT alkyl boronic acid ester, OH
DATIM 22-12-2014 17:05:23
OBNUC 1H
EXMOD proton.jxp
OBFRQ 391.78 MHz
OBSET 8.51 KHz
OBFIN 3.34 Hz
POINT 16384
FREQU 7348.62 Hz
SCANS 4
ACQTM 2.2295 sec
PD 5.0000 sec
PW1 5.07 usec
IRNUC 1H
CTEMP 22.1 c
SLVNT CDCL3
EXREF 7.26 ppm
BF 0.12 Hz
RGAIN 22

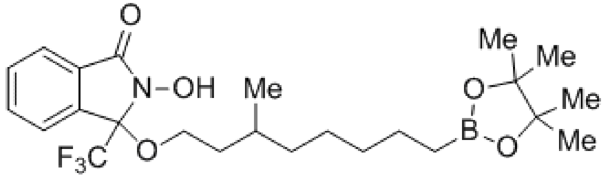

1q

alkyl boronic acid ester, OH

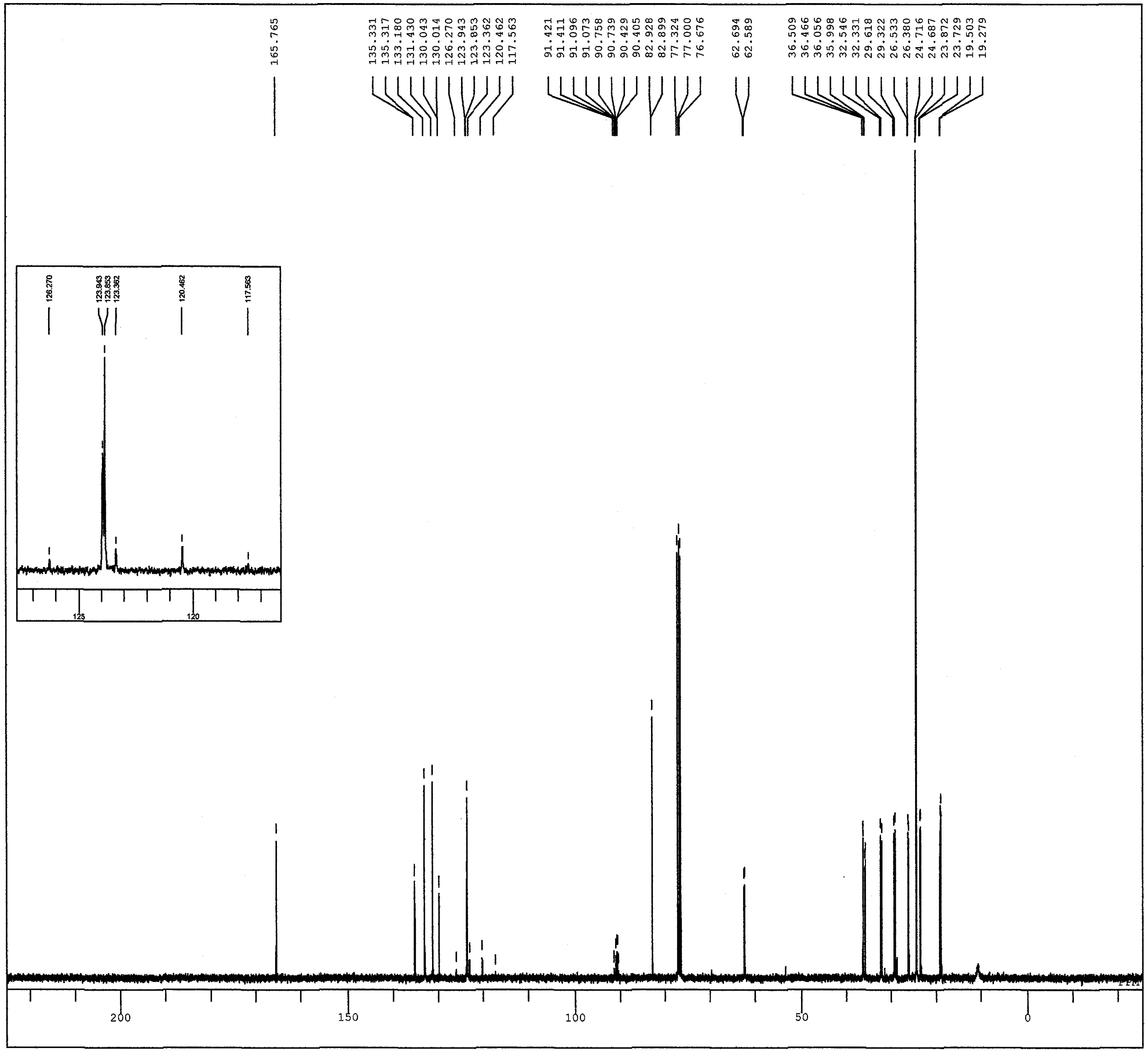

DFILE ozawa06-107_13C.jdf
COMNT alkyl boronic acid ester, OH
DATIM 22-12-2014 17:07:37
OBNUC 13C
EXMOD carbon.jxp
OBFRQ 98.52 MHz
OBSET 4.64 KHz
OBFIN 8.74 Hz
POINT 65535
FREQU 30788.18 Hz
SCANS 264
ACQTM 2.1286 sec
PD 2.0000 sec
PW1 3.07 usec
IRNUC 1H
CTEMP 22.4 c
SLVNT CDCL3
EXREF 77.00 ppm
BF 1.00 Hz
RGAIN 58

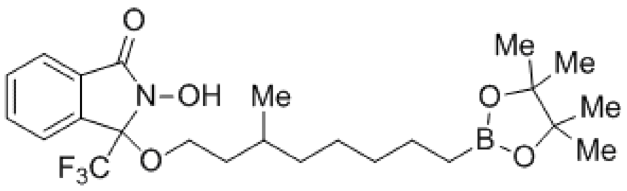

1q

alkyl boronic acid ester, OH

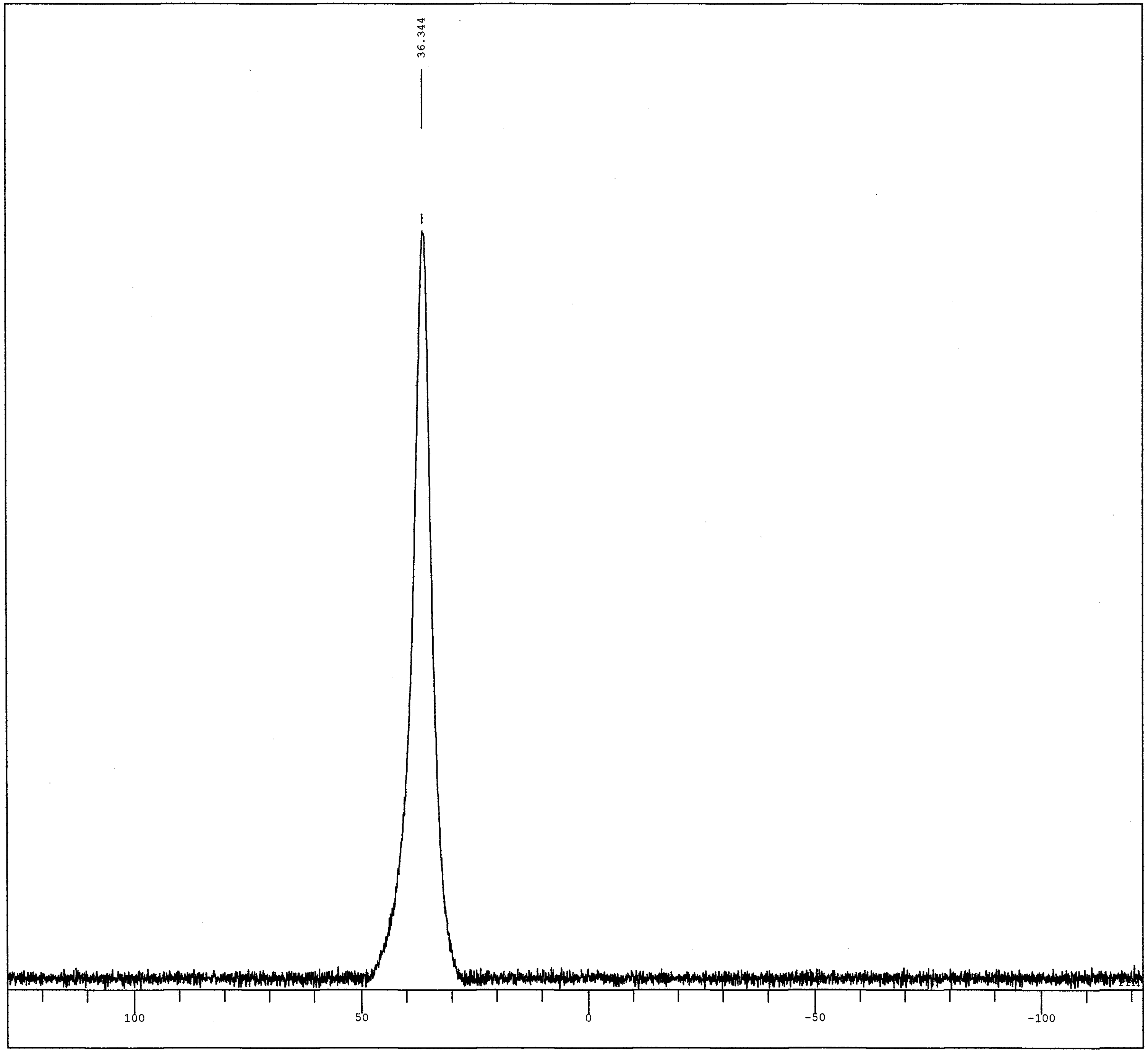

DFILE ozawa06-107_11B.jdf
COMNT alkyl boronic acid ester, OH
DATIM 22-12-2014 17:01:53
OBNUC 11B
EXMOD carbon.jxp
OBFRQ 125.70 MHz
OBSET 0.81 KHz
OBFIN 3.25 Hz
POINT 4095
FREQU 39308.18 Hz
SCANS 500
ACQTM 0.1042 sec
PD 80.0000 sec
PW1 3.98 usec
IRNUC 1H
CTEMP 22.1 c
SLVNT CDCL3
EXREF 31.00 ppm
BF 0.12 Hz
RGAIN 44

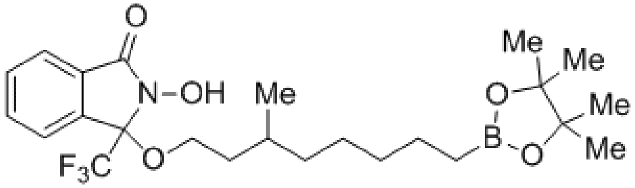

1q

alkyl boronic acid ester, OH

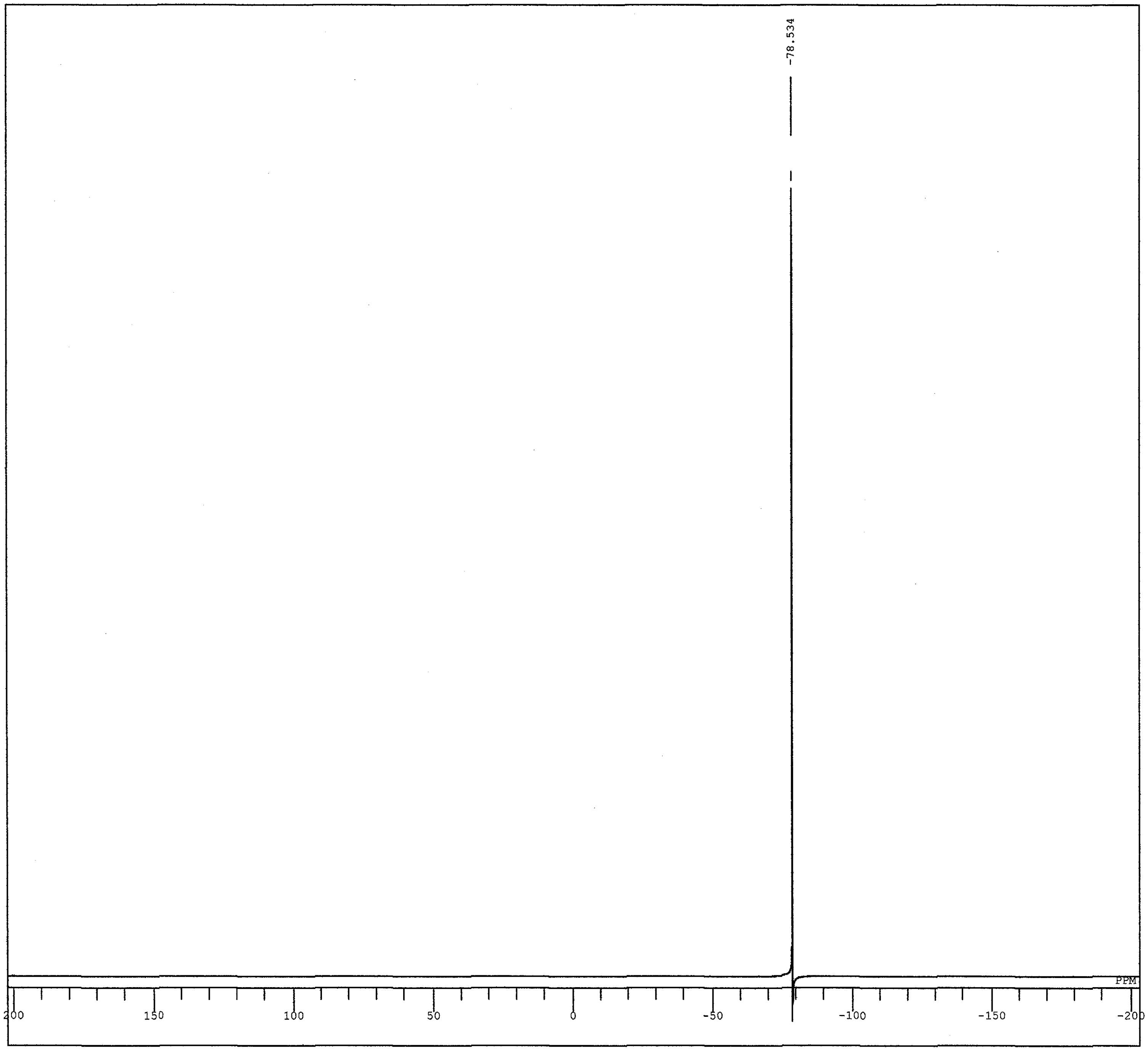

DFILE ozawa06-107_19F.jdf
COMNT alkyl boronic acid ester, OH
DATIM 22-12-2014 16:57:41
OBNUC 19F
EXMOD proton.jxp
OBFRQ 368.64 MHz
OBSET 7.63 KHz
OBFIN 2.85 Hz
POINT 16384
FREQU 186567.17 Hz
SCANS 8
ACQTM 0.0878 sec
PD 5.0000 sec
PW1 3.90 usec
IRNUC 19F
CTEMP 22.1 c
SLVNT CDCL3
EXREF -164.90 ppm
BF 0.12 Hz
RGAIN 46

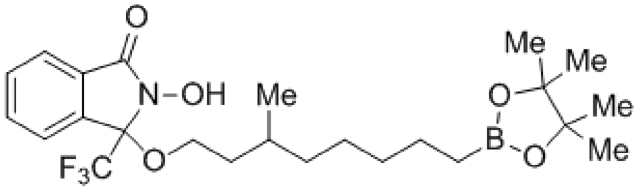

1q

alkyl-Bpin, [0]

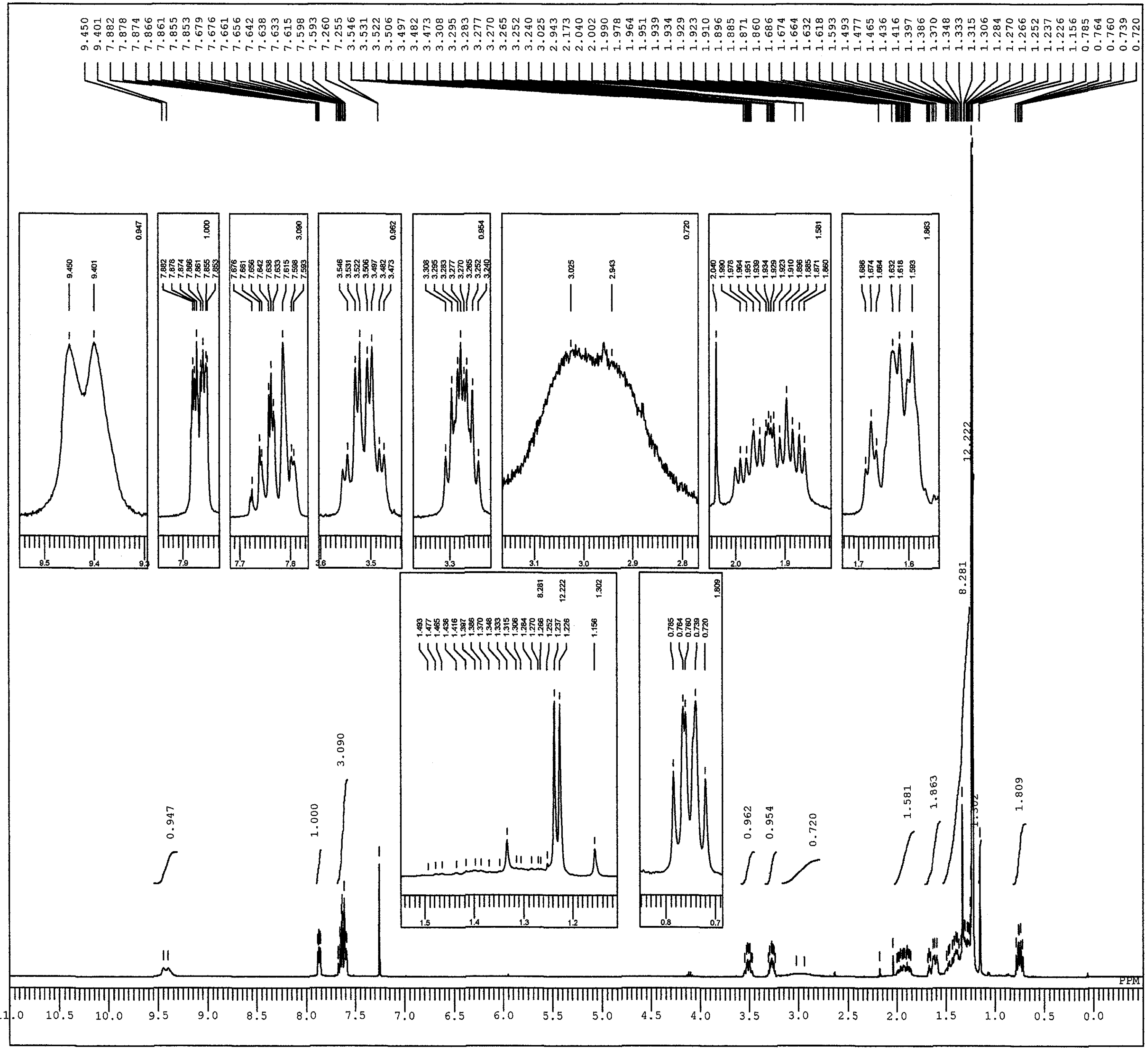

DFILE	ozawa06-115_1H.als
COMNT	alkyl-Bpin, [0]
DATIM	30-12-2014 10:07:05
OBNUC	1H
EXMOD	proton.jxp
OBFRQ	391.78 MHz
OBSET	8.51 KHz
OBFIN	3.34 Hz
POINT	13107
FREQU	5878.90 Hz
SCANS	8
ACQTM	2.2295 sec
PD	5.0000 sec
PW1	5.07 usec
IRNUC	1H
CTEMP	21.6 c
SLVNT	CDCL3
EXREF	7.26 ppm
BF	0.12 Hz
RGAIN	36

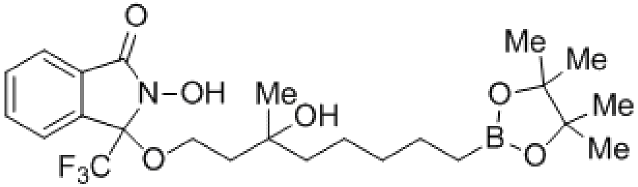

2q

alkyl Bpin, [O]

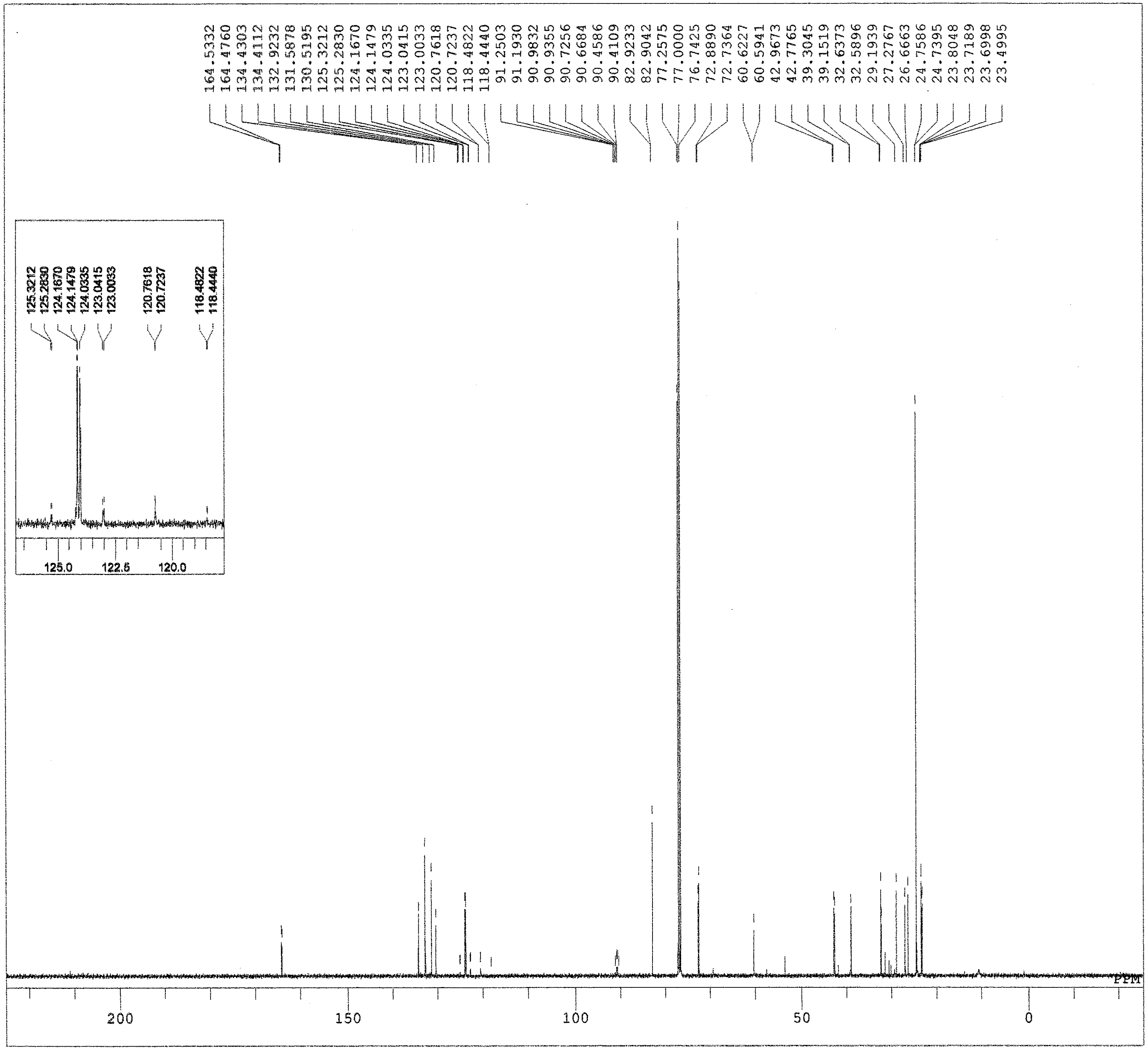

DFILE ozawa06-115_13C.jdf
COMNT alkyl Bpin, [O]
DATIM 2014-12-28 07:52:44
OBNUC 13C
EXMOD carbon.jxp
OBFRQ 125.77 MHz
OBSET 7.87 KHz
OBFIN 4.21 Hz
POINT 32767
FREQU 39308.18 Hz
SCANS 5120
ACQTM 0.8336 sec
PD 2.0000 sec
PW1 3.40 usec
IRNUC 1H
CTEMP 24.5 c
SLVNT CDCL3
EXREF 77.00 ppm
BF 0.12 Hz
RGAIN 60

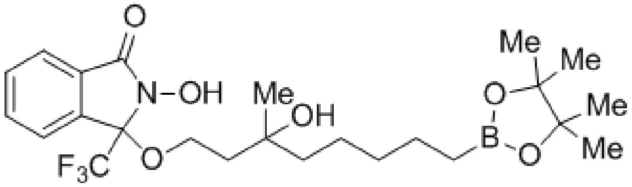

2q

alkyl-Bpin, [O]

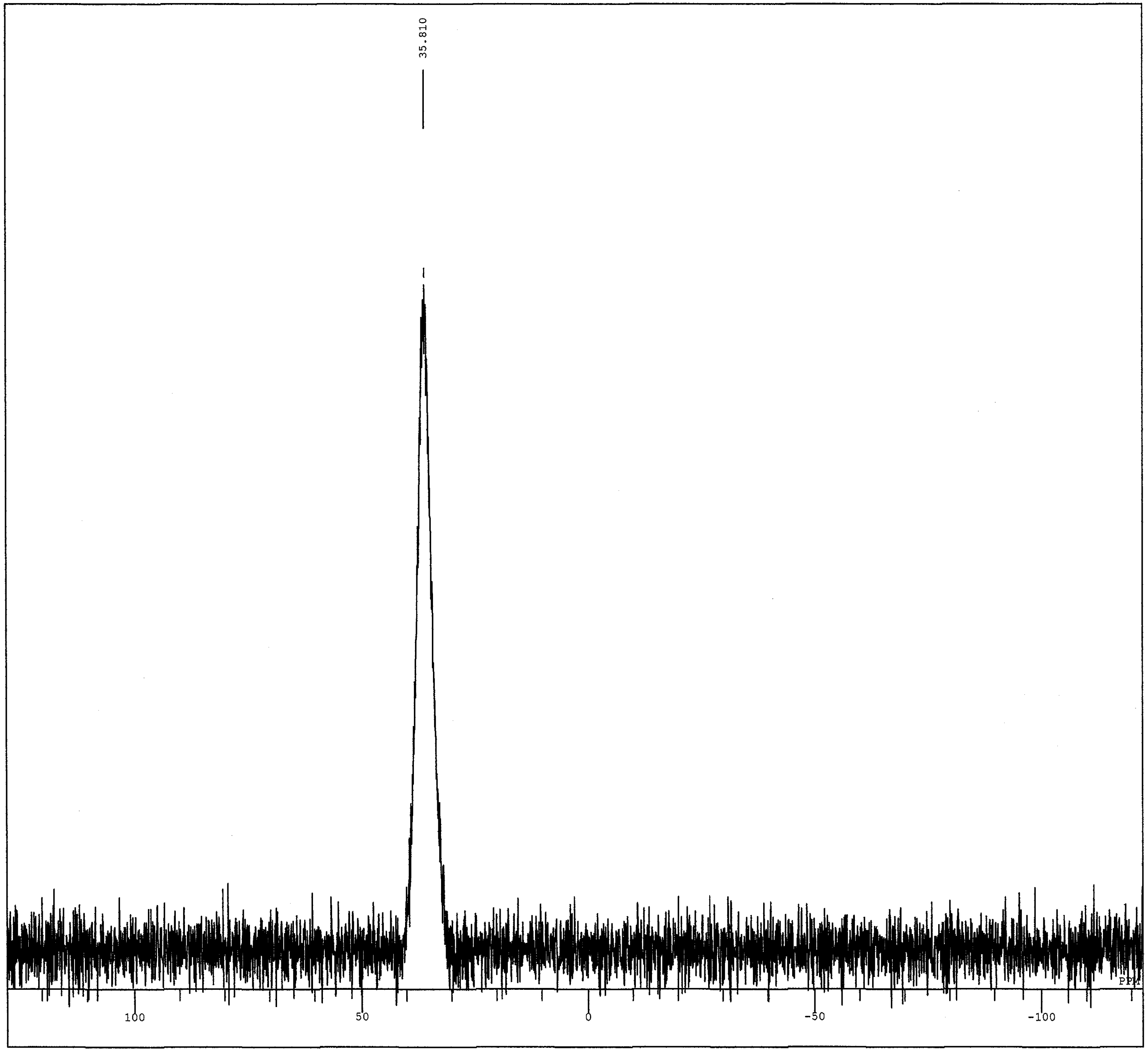

DFILE ozawa06-115_11B.jdf
COMNT alkyl-Bpin, [O]
DATIM 30-12-2014 10:03:48
OBNUC 11B
EXMOD carbon.jxp
OBFRQ 125.70 MHz
OBSET 0.81 KHz
OBFIN 3.25 Hz
POINT 4095
FREQU 39308.18 Hz
SCANS 500
ACQTM 0.1042 sec
PD 80.0000 sec
PW1 3.98 usec
IRNUC 1H
CTEMP 21.7 c
SLVNT CDCL3
EXREF 31.00 ppm
BF 0.12 Hz
RGAIN 60

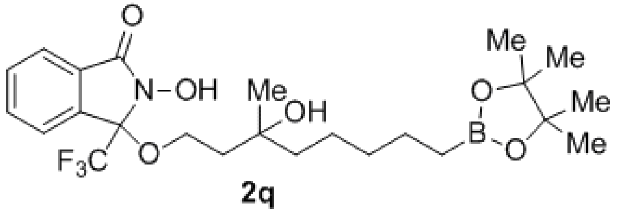

2q

alkyl-Bpin, [O]

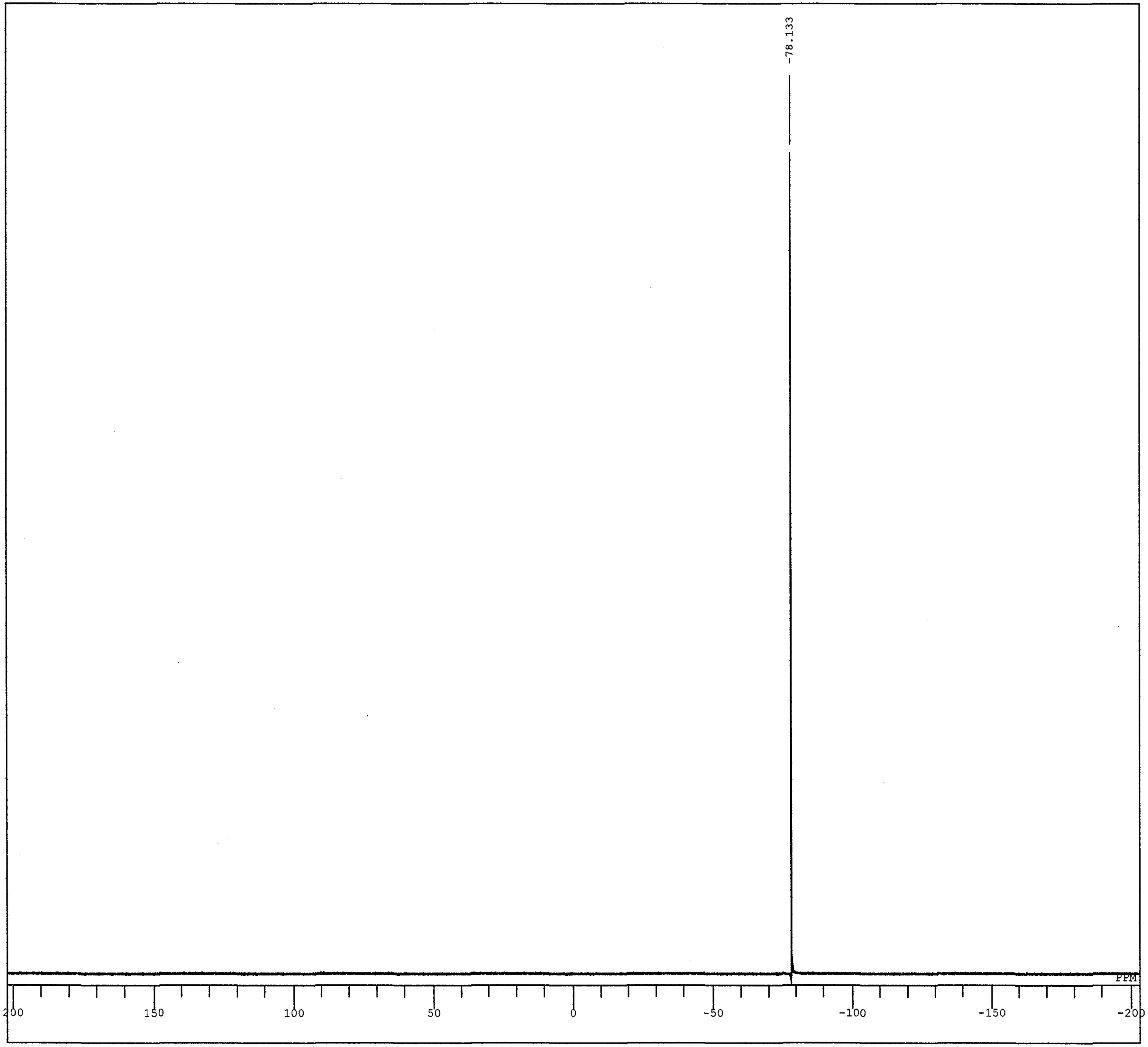

DFILE ozawa06-115_19F.jdf
COMNT alkyl-Bpin, [O]
DATIM 30-12-2014 10:09:12
OBNUC 19F
EXMOD proton.jxp
OBFRQ 368.64 MHz
OBSET 7.63 KHz
OBFIN 2.85 Hz
POINT 16384
FREQU 186567.17 Hz
SCANS 8
ACQTM 0.0878 sec
PD 5.0000 sec
PW1 3.90 usec
IRNUC 19F
CTEMP 21.5 c
SLVNT CDCL3
EXREF -164.90 ppm
BF 0.12 Hz
RGAIN 50

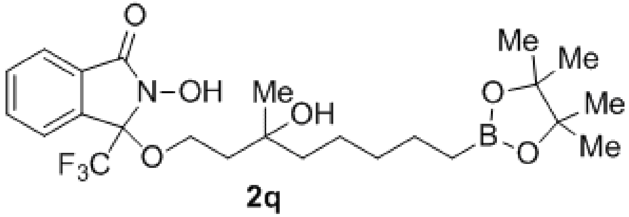

2q

D-valinol-N-phth

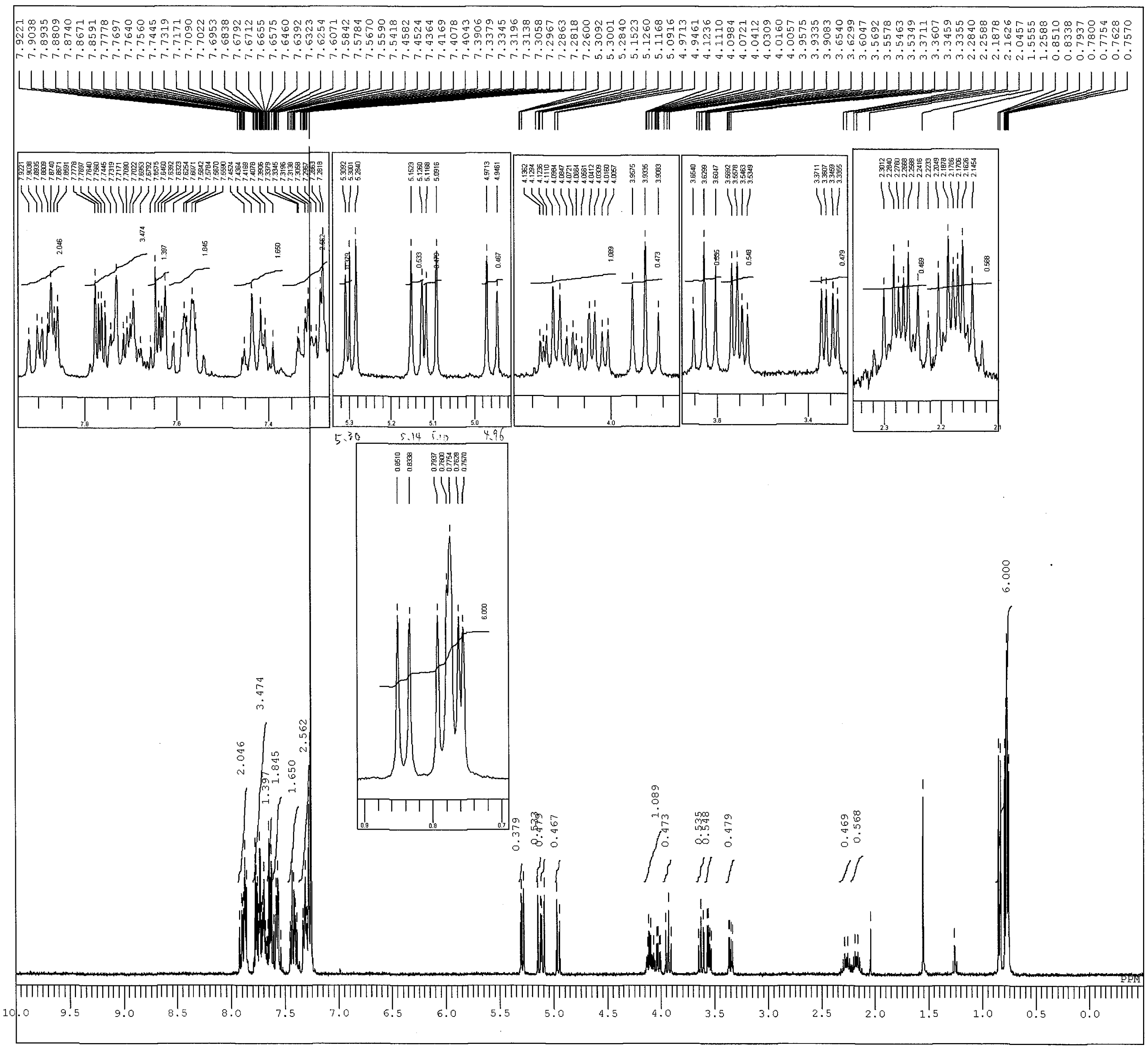

DFILE ozawa04-060.jdf
COMNT D-valinol-N-phth
DATIM 02-12-2013 21:20:35
OBNUC 1H
EXMOD proton.jxp
OBFRQ 391.78 MHz
OBSET 8.51 KHz
OBFIN 3.34 Hz
POINT 16384
FREQU 7352.94 Hz
SCANS 4
ACQTM 2.2282 sec
PD 5.0000 sec
PW1 5.25 usec
IRNUC 1H
CTEMP 21.4 c
SLVNT CDCL3
EXREF 7.26 ppm
BF 0.12 Hz
RGAIN 48

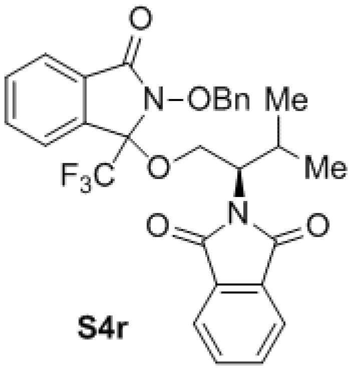

S4r

valinol, Bn

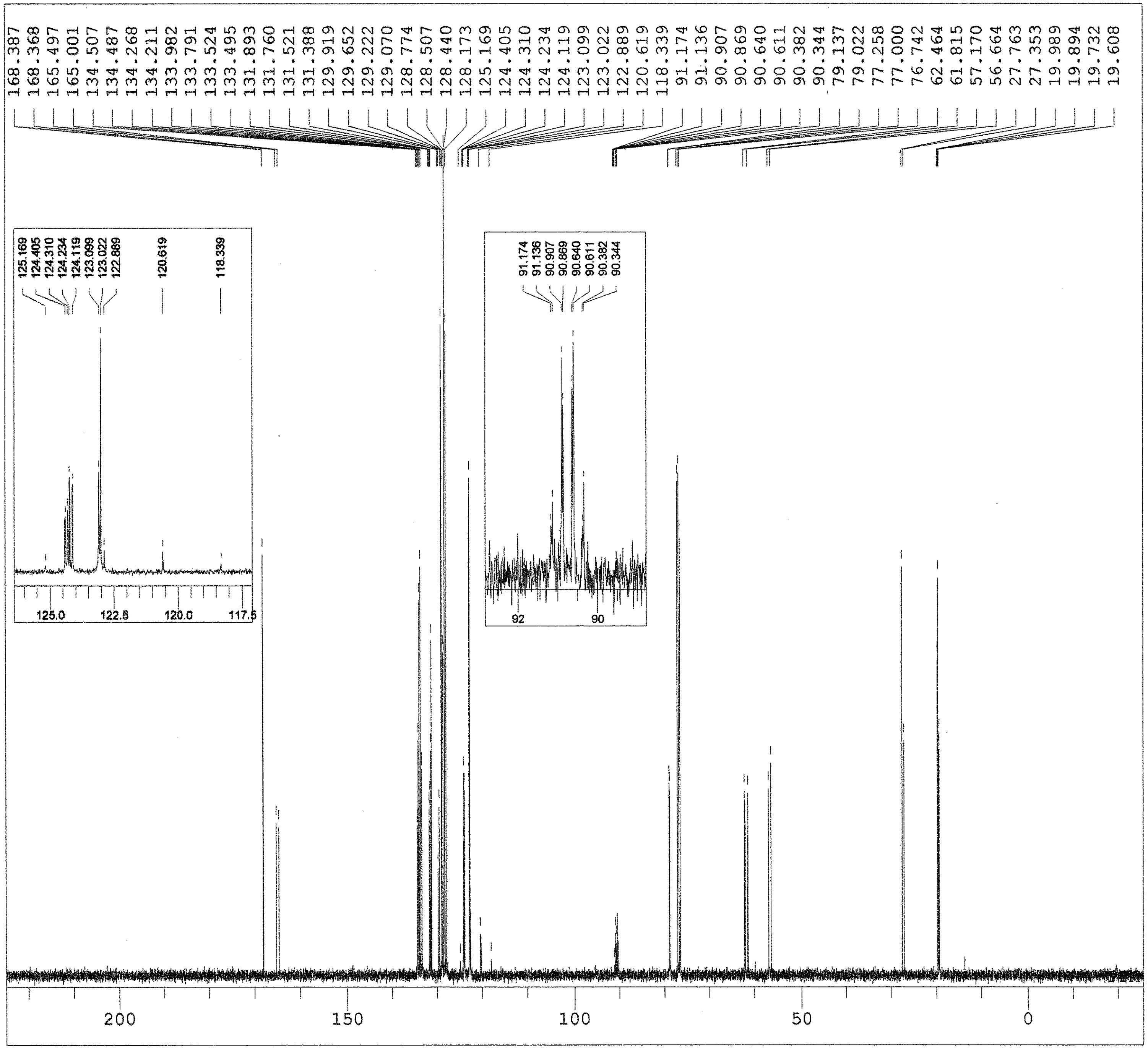

DFILE ozawa04-060_13C.jdf
COMNT valinol, Bn
DATIM 2014-03-17 13:26:26
OBNUC 13C
EXMOD carbon.jxp
OBFRQ 125.77 MHz
OBSET 7.87 KHz
OBFIN 4.21 Hz
POINT 32767
FREQU 39308.18 Hz
SCANS 160
ACQTM 0.8336 sec
PD 3.0000 sec
PW1 3.40 usec
IRNUC 1H
CTEMP 21.3 c
SLVNT CDCL3
EXREF 77.00 ppm
BF 0.42 Hz
RGAIN 60

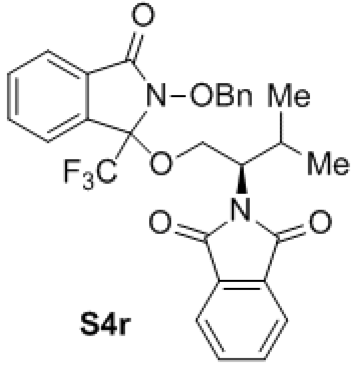

S4r

D-valinol-N-phth , Bn

C:\Documents and Settings\Administrator\ƒƒƒXƒNƒgƒbƒv\Gousei\Ozawa\data\ozawa04-060_19F.als

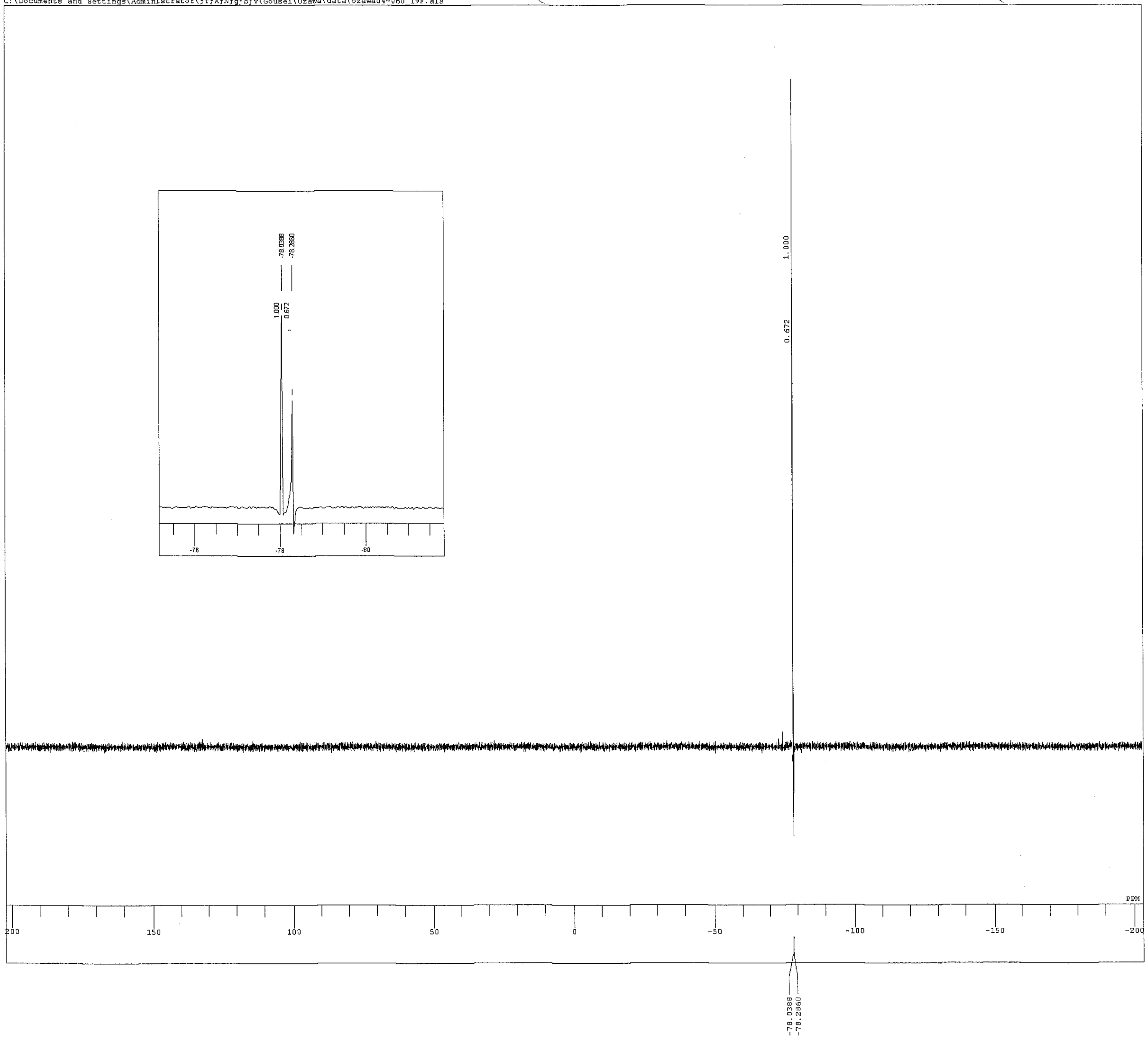

DFILE ozawa04-060_19F.als
COMNT D-valinol-N-phth . Bn
DATIM 02-12-2013 21:18:26
OBNUC 19F
EXMOD proton.jxp
OBFRQ 368.64 MHz
OBSET 7.63 KHz
OBFIN 2.85 Hz
POINT 13107
FREQU 149253.73 Hz
SCANS 4
ACQTM 0.0878 sec
PD 5.0000 sec
PW1 3.90 usec
IRNUC 19F
CTEMP 21.4 c
SLVNT CDCL3
EXREF -164.90 ppm
BF 0.12 Hz
RGAIN 50

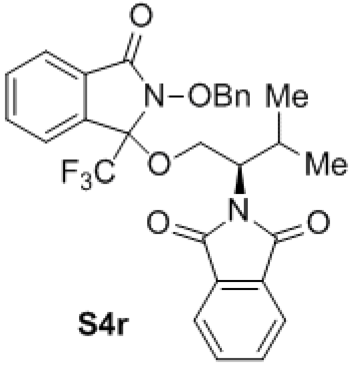

S4r

valinol, OH

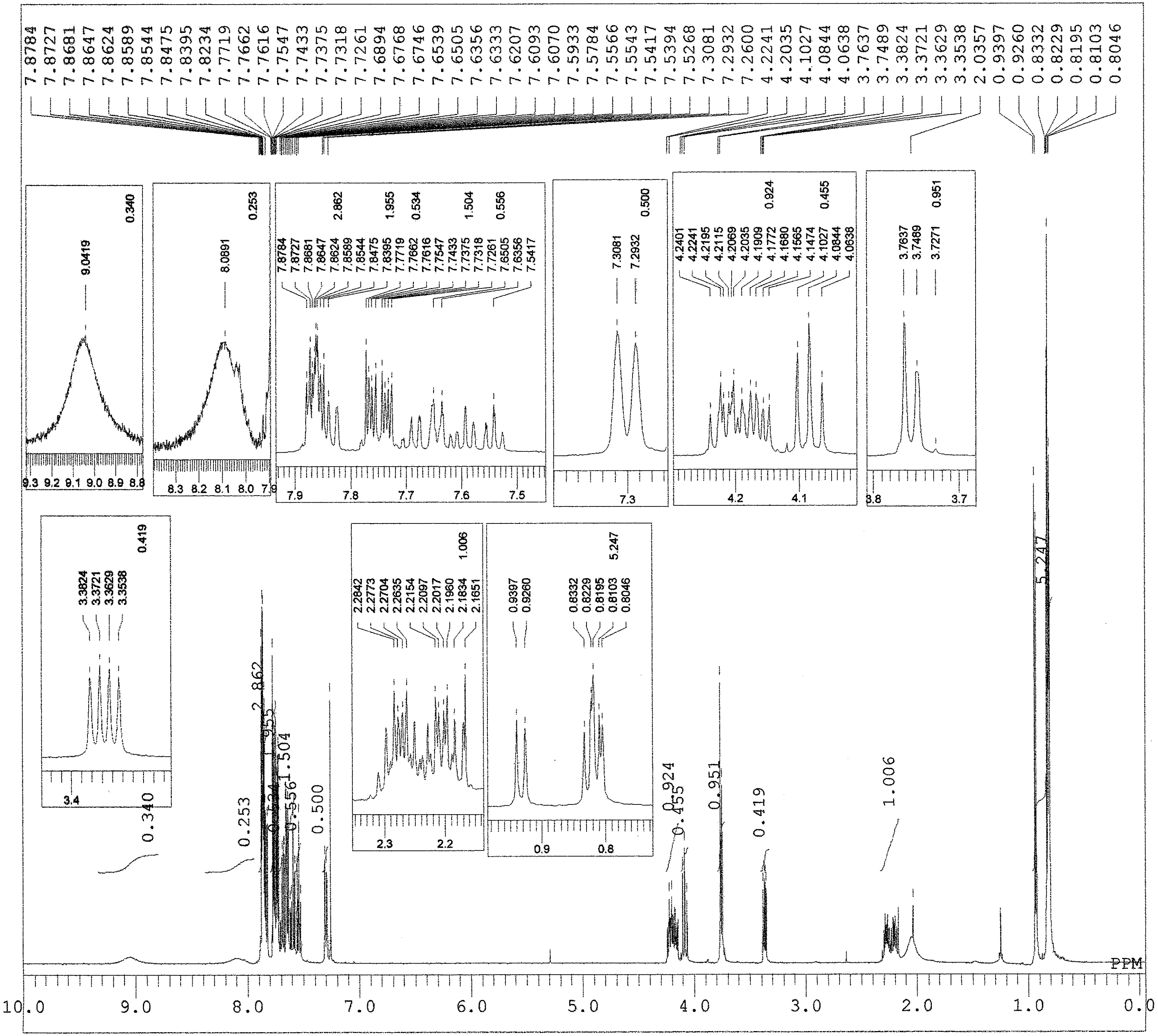

DFILE	ozawa04-064_1H.jdf
COMNT	valinol, OH
DATIM	2014-08-18 19:26:34
OBNUC	1H
EXMOD	proton.jxp
OBFRQ	500.16 MHz
OBSET	2.41 KHz
OBFIN	6.01 Hz
POINT	16384
FREQU	9384.38 Hz
SCANS	4
ACQTM	1.7459 sec
PD	5.0000 sec
PW1	5.55 usec
IRNUC	1H
CTEMP	25.7 c
SLVNT	CDCL3
EXREF	7.26 ppm
BF	0.12 Hz
RGAIN	36

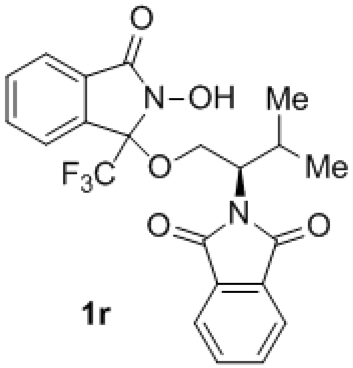

1r

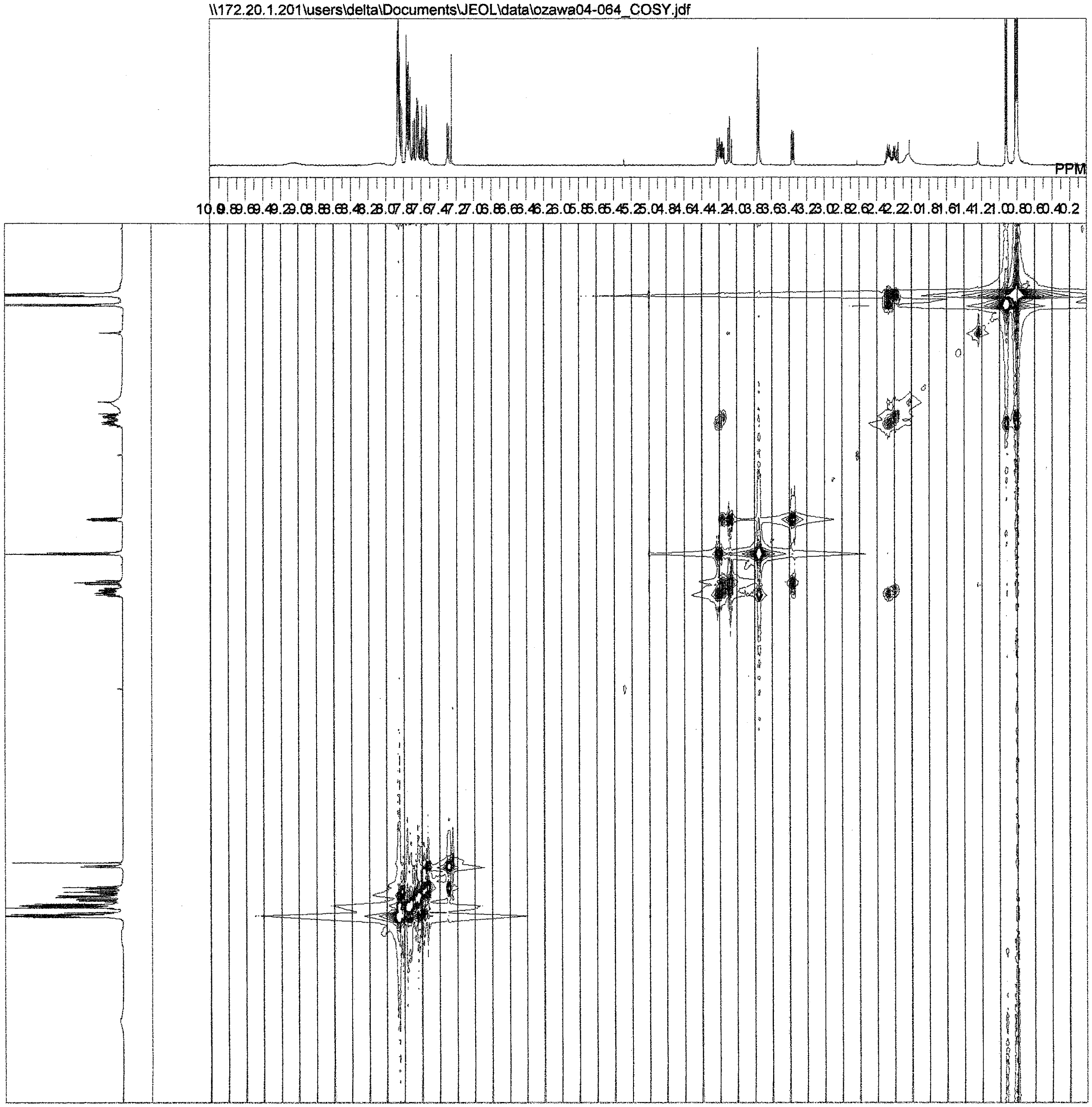

DFILE	ozawa04-064_COSY.jdf
COMNT	valinol, OH
DATIM	18-08-2014 19:34:41
EXMOD	cosy.jxp
OBNUC	1H
OBFRQ	500.16 MHz
OBSET	2.41 KHz
OBFIN	6.01 Hz
POINT	1280
FREQU	6253.13 Hz
CLPNT	256
TODAT	256
CLFRQ	5002.00 Hz
SCANS	2
ACQTM	0.2047 sec
PD	1.5000 sec
PW1	11.10 usec
PW2	0.00 usec
PW3	0.00 usec
PI1	0.0000 msec
PI2	0.0000 msec
PI3	0.0000 msec
IRNUC	1H
CTEMP	25.9 c
SLVNT	CDCL3
EXREF	7.26 ppm
CLEXR	7.26
RGAIN	48

valinol, OH

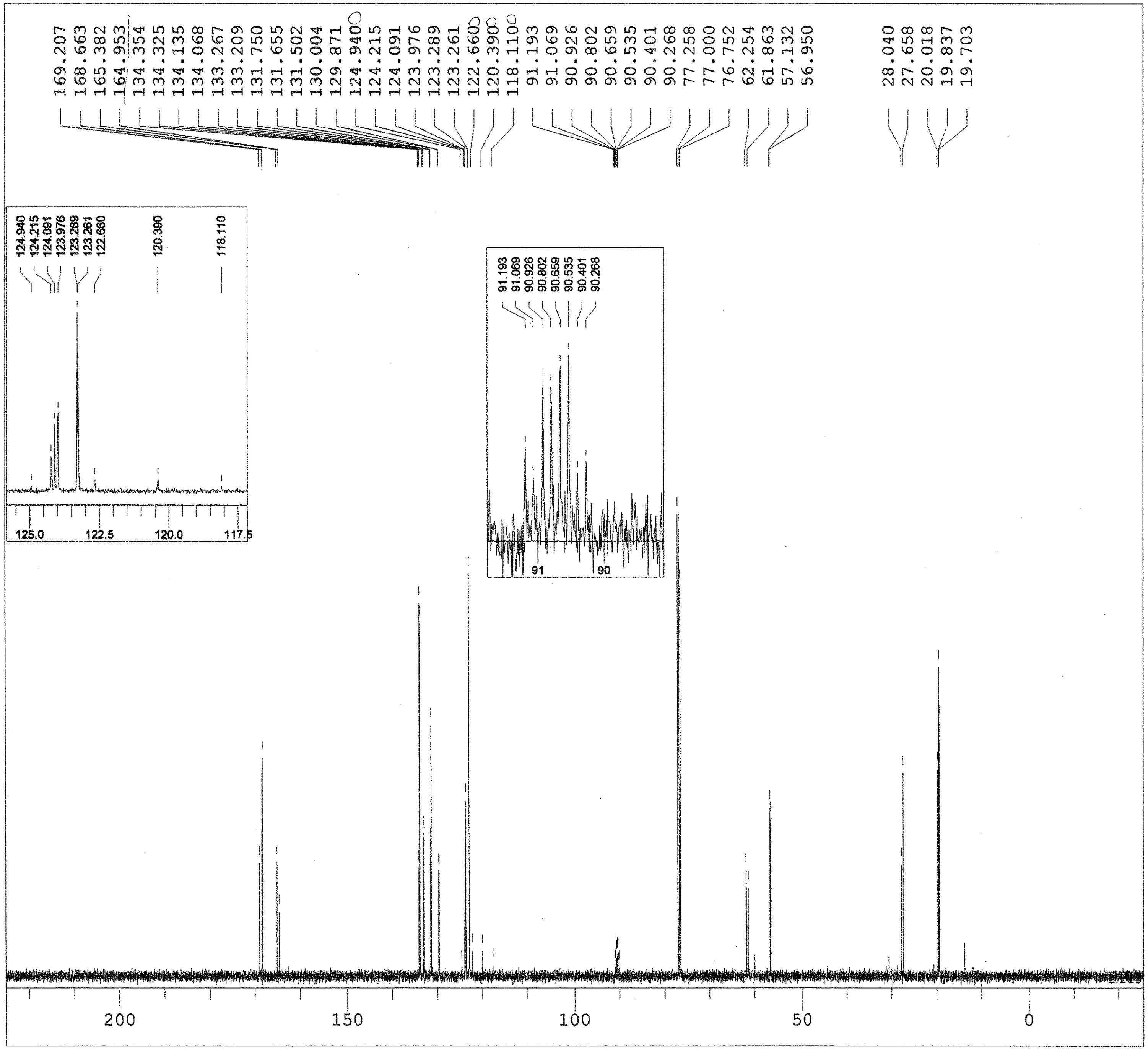

DFILE	ozawa04-064_13C.jdf
COMNT	valinol, OH
DATIM	2014-03-17 13:12:34
OBNUC	13C
EXMOD	carbon.jxp
OBFRQ	125.77 MHz
OBSET	7.87 KHz
OBFIN	4.21 Hz
POINT	32767
FREQU	39308.18 Hz
SCANS	124
ACQTM	0.8336 sec
PD	3.0000 sec
PW1	3.40 usec
IRNUC	1H
CTEMP	21.5 c
SLVNT	CDCL3
EXREF	77.00 ppm
BF	0.42 Hz
RGAIN	60

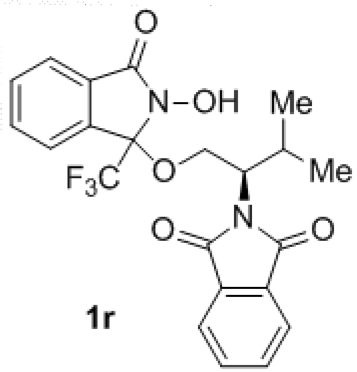

1r

valinol, OH

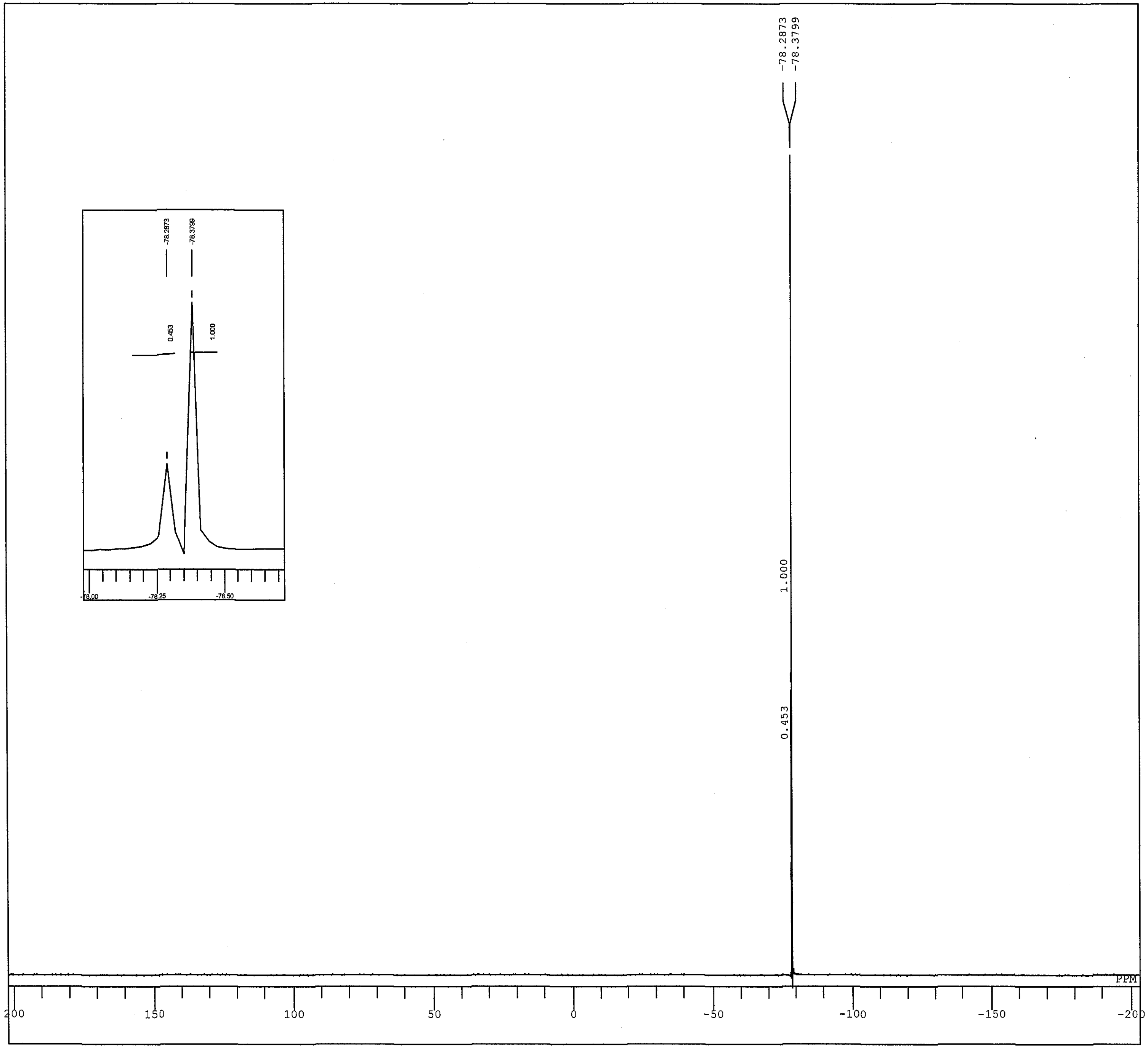


```
DFILE  ozawa04-064_19F.jdf
COMNT  valinol, OH
DATIM  11-02-2014 19:56:06
OBNUC  19F
EXMOD  proton.jxp
OBFRQ        368.64 MHz
OBSET          7.63 KHz
OBFIN          2.85 Hz
POINT         16384
FREQU     186567.17 Hz
SCANS             4
ACQTM        0.0878 sec
PD           5.0000 sec
PW1            3.90 usec
IRNUC  19F
CTEMP          21.2 c
SLVNT  CDCL3
EXREF       -164.90 ppm
BF             1.00 Hz
RGAIN            46
```

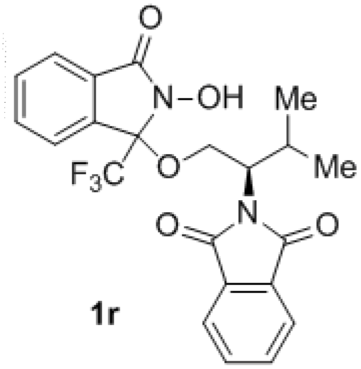

1r

valinol, [O]

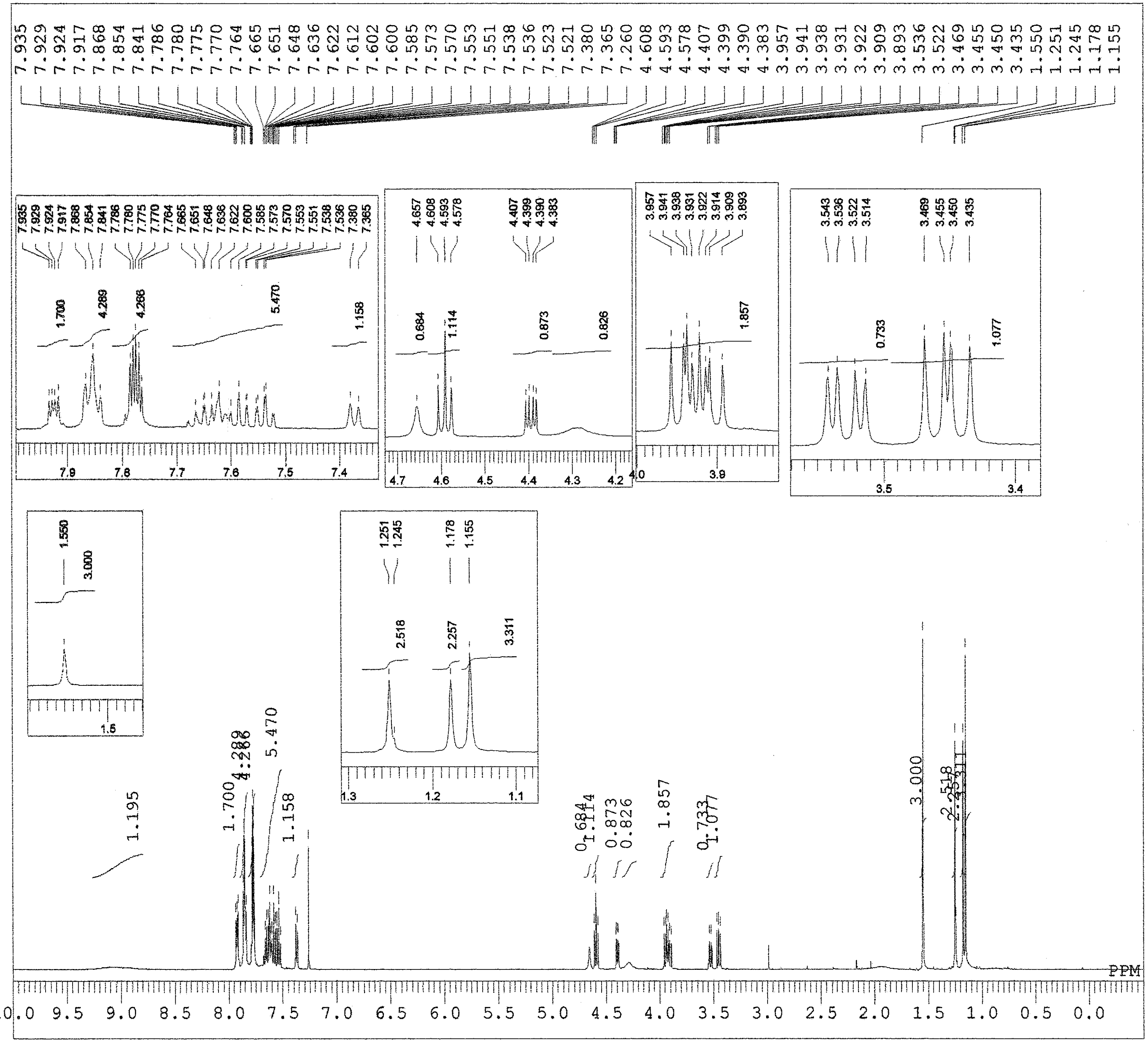

DFILE	ozawa04-116b_1H.jdf
COMNT	valinol, [O]
DATIM	2014-02-08 21:13:58
OBNUC	1H
EXMOD	proton.jxp
OBFRQ	500.16 MHz
OBSET	2.41 KHz
OBFIN	6.01 Hz
POINT	16384
FREQU	9384.38 Hz
SCANS	4
ACQTM	1.7459 sec
PD	5.0000 sec
PW1	5.55 usec
IRNUC	1H
CTEMP	21.9 c
SLVNT	CDCL3
EXREF	7.26 ppm
BF	0.12 Hz
RGAIN	36

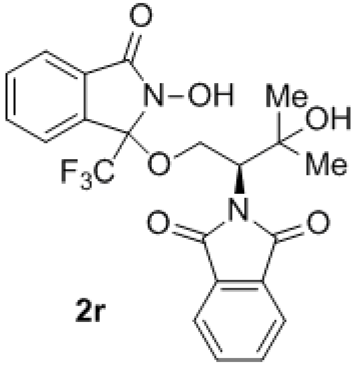

2r

\\172.20.1.201\users\delta\Documents\JEOL\data\ozawa04-116b_COSY.jdf

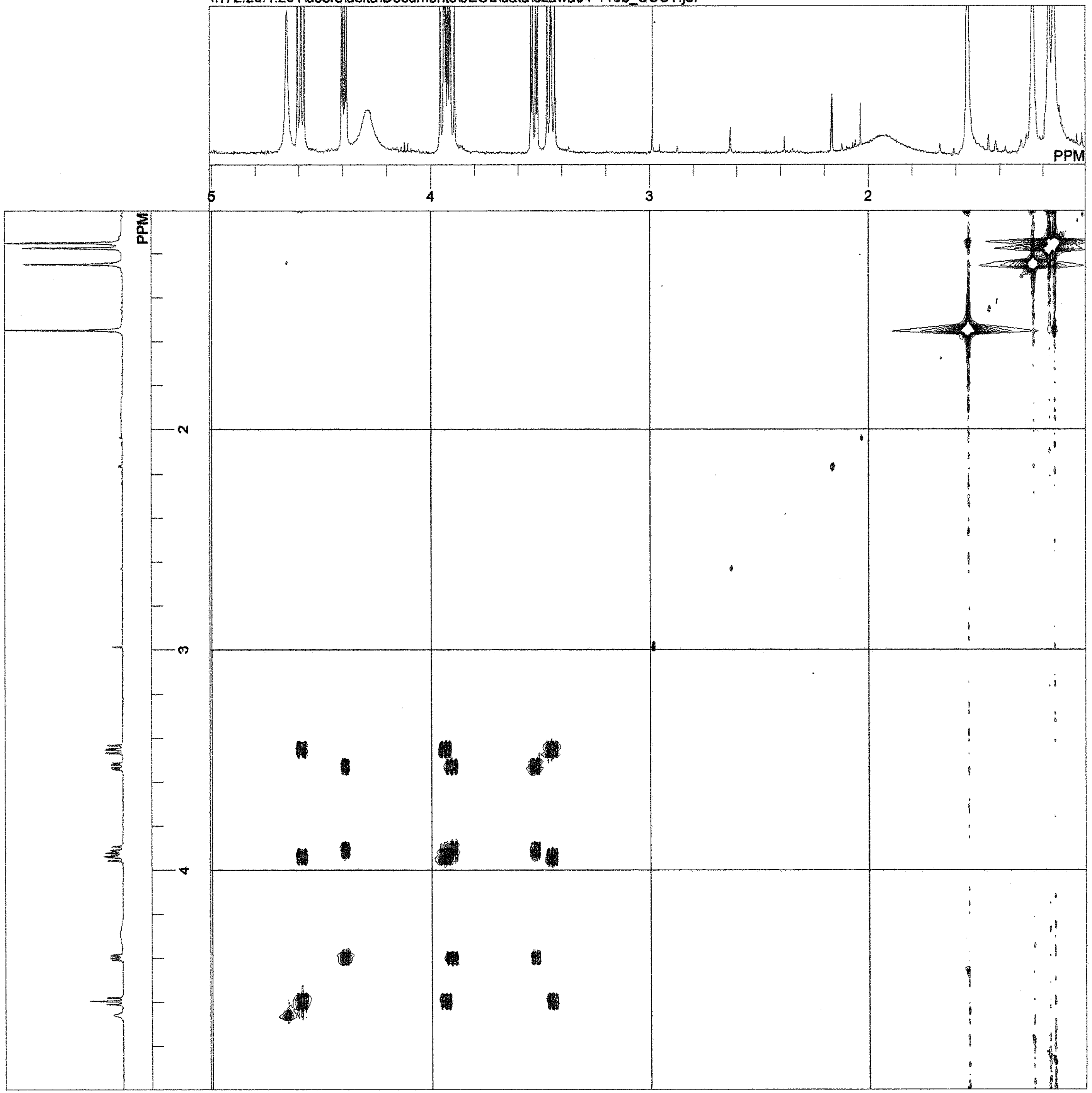

DFILE	ozawa04-116b_COSY.jdf
COMNT	valinol, [O]
DATIM	08-02-2014 21:15:04
EXMOD	cosy.jxp
OBNUC	1H
OBFRQ	500.16 MHz
OBSET	1.41 KHz
OBFIN	5.69 Hz
POINT	1280
FREQU	2501.00 Hz
CLPNT	256
TODAT	256
CLFRQ	2000.80 Hz
SCANS	1
ACQTM	0.5118 sec
PD	2.5000 sec
PW1	11.10 usec
PW2	0.00 usec
PW3	0.00 usec
PI1	0.0000 msec
PI2	0.0000 msec
PI3	0.0000 msec
IRNUC	1H
CTEMP	21.8 c
SLVNT	CDCL3
EXREF	7.26 ppm
CLEXR	7.26
RGAIN	50

\\172.20.1.201\users\delta\Documents\JEOL\data\ozawa04-116b_COSY_aroma.jdf

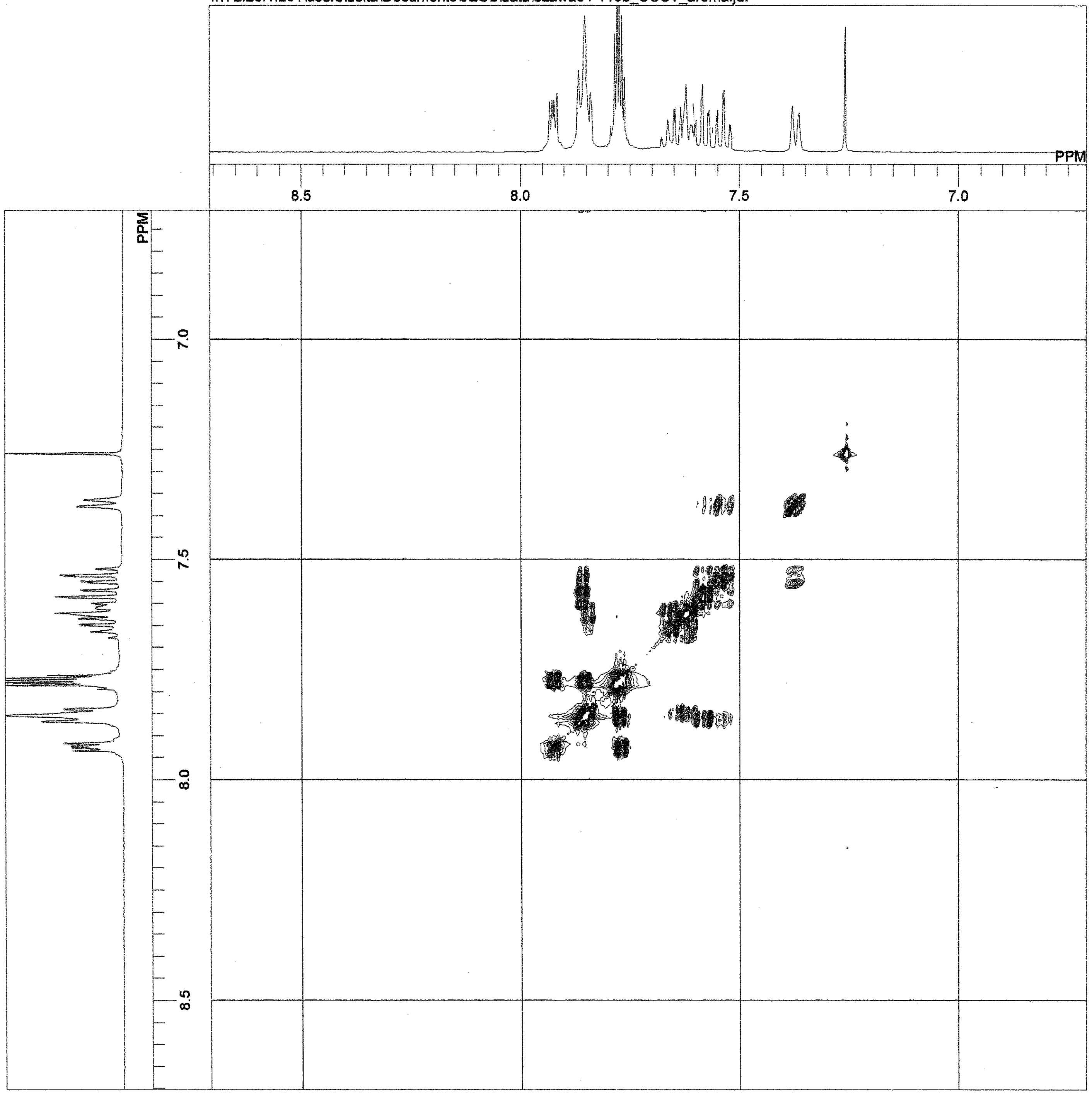

DFILE	ozawa04-116b_COSY_aroma.jdf
COMNT	valinol, [O]
DATIM	11-02-2014 21:00:49
EXMOD	cosy.jxp
OBNUC	1H
OBFRQ	500.16 MHz
OBSET	3.76 KHz
OBFIN	6.44 Hz
POINT	1280
FREQU	1250.50 Hz
CLPNT	256
TODAT	256
CLFRQ	1000.34 Hz
SCANS	1
ACQTM	1.0236 sec
PD	2.5000 sec
PW1	11.10 usec
PW2	0.00 usec
PW3	0.00 usec
PI1	0.0000 msec
PI2	0.0000 msec
PI3	0.0000 msec
IRNUC	1H
CTEMP	21.3 c
SLVNT	CDCL3
EXREF	7.26 ppm
CLEXR	7.26
RGAIN	54

valinol, [O]

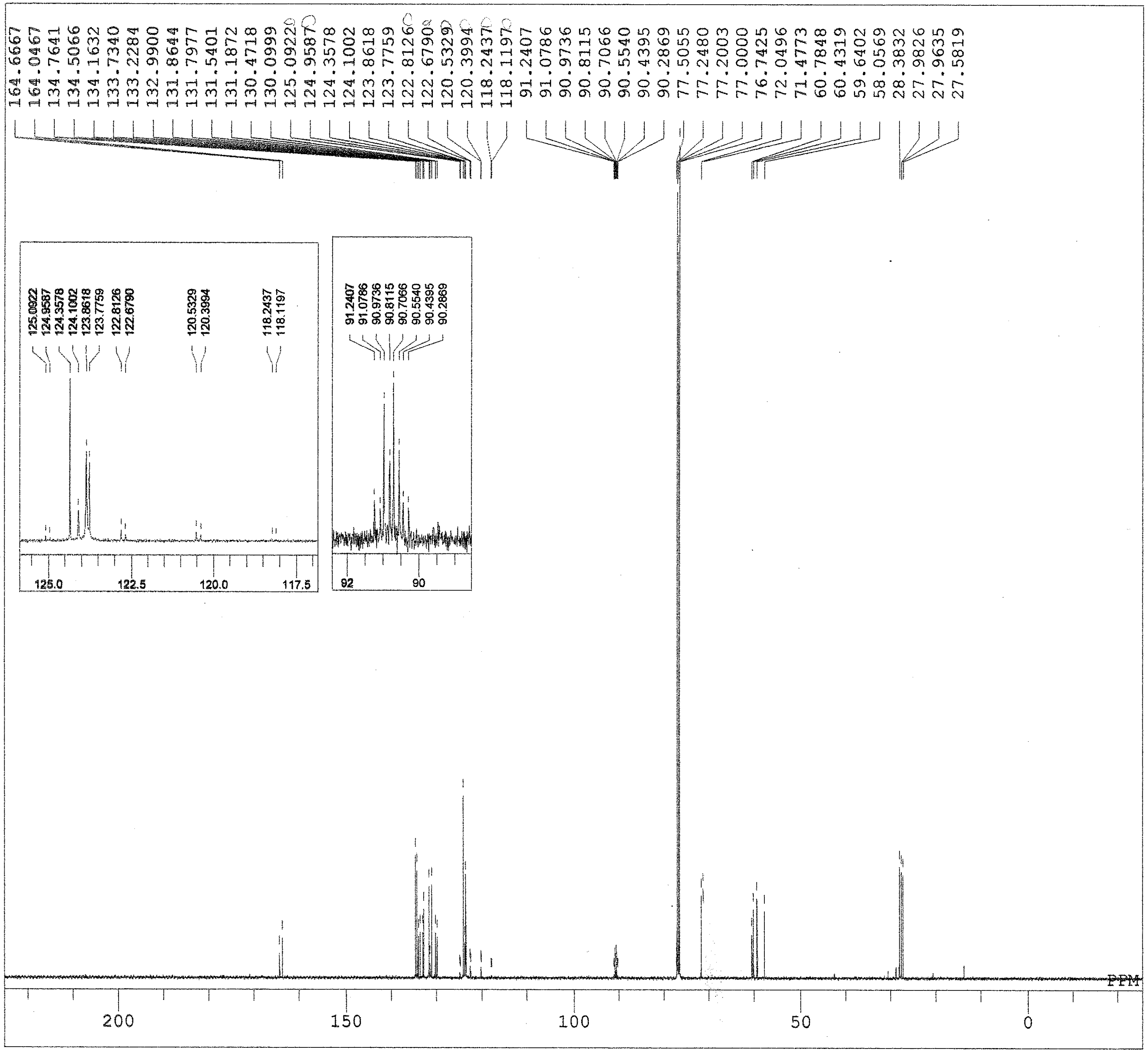

DFILE	ozawa04-116b_13C.jdf
COMNT	valinol, [O]
DATIM	2014-03-22 22:54:06
OBNUC	13C
EXMOD	carbon.jxp
OBFRQ	125.77 MHz
OBSET	7.87 KHz
OBFIN	4.21 Hz
POINT	32767
FREQU	39308.18 Hz
SCANS	12788
ACQTM	0.8336 sec
PD	3.0000 sec
PW1	3.40 usec
IRNUC	1H
CTEMP	21.4 c
SLVNT	CDCL3
EXREF	77.00 ppm
BF	0.12 Hz
RGAIN	60

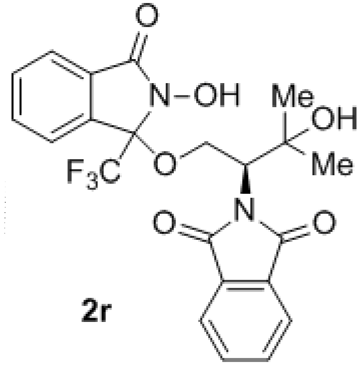

2r

valinol, [0]

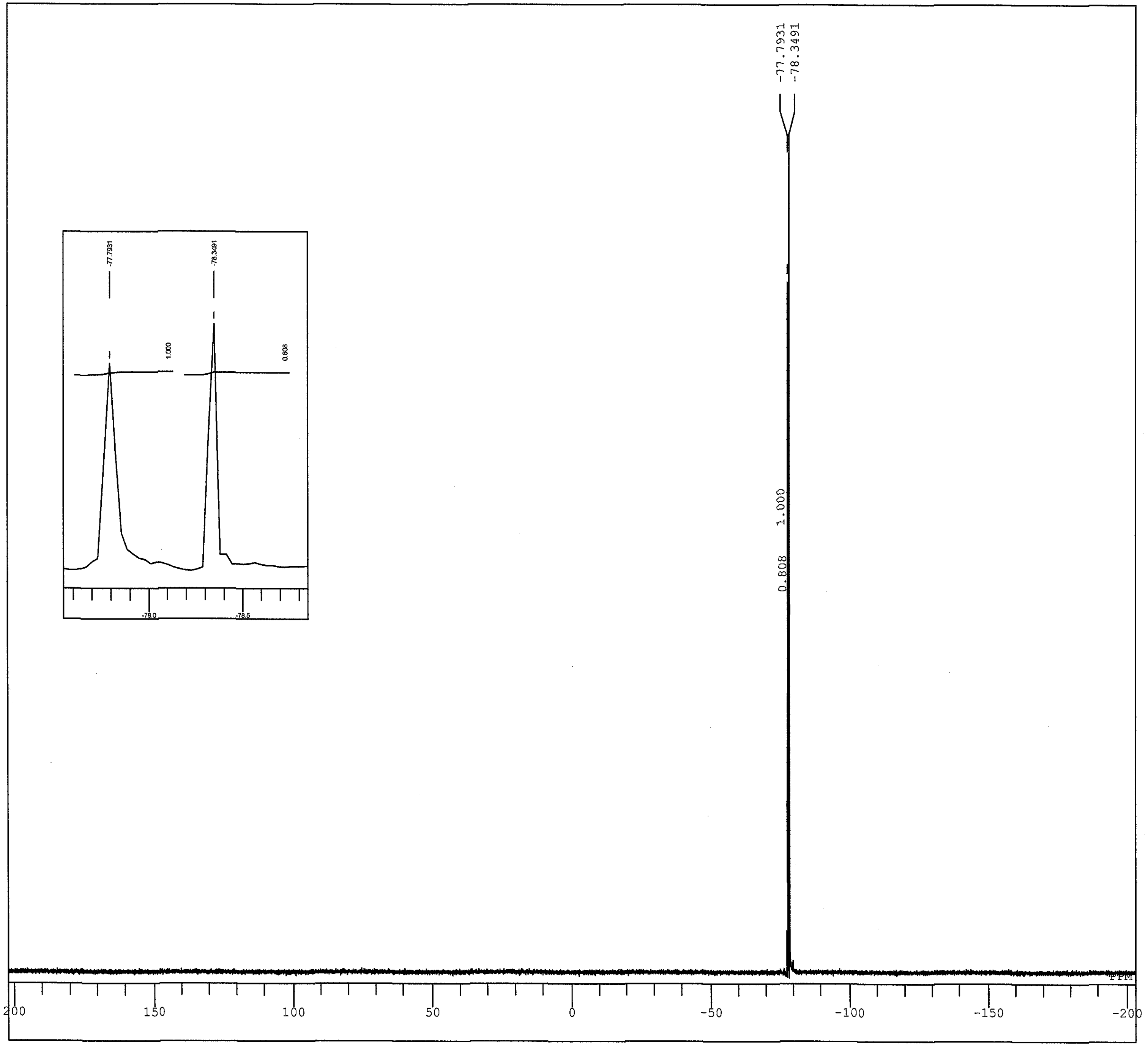

DFILE ozawa04-116b_19F.jdf
COMNT valinol, [O]
DATIM 11-02-2014 19:59:11
OBNUC 19F
EXMOD proton.jxp
OBFRQ 368.64 MHz
OBSET 7.63 KHz
OBFIN 2.85 Hz
POINT 16384
FREQU 186567.17 Hz
SCANS 4
ACQTM 0.0878 sec
PD 5.0000 sec
PW1 3.90 usec
IRNUC 19F
CTEMP 21.1 c
SLVNT CDCL3
EXREF -164.90 ppm
BF 1.00 Hz
RGAIN 48

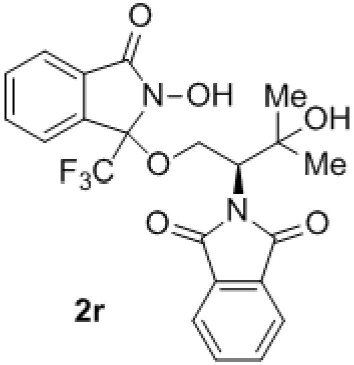

2r

PhPROH, Bn

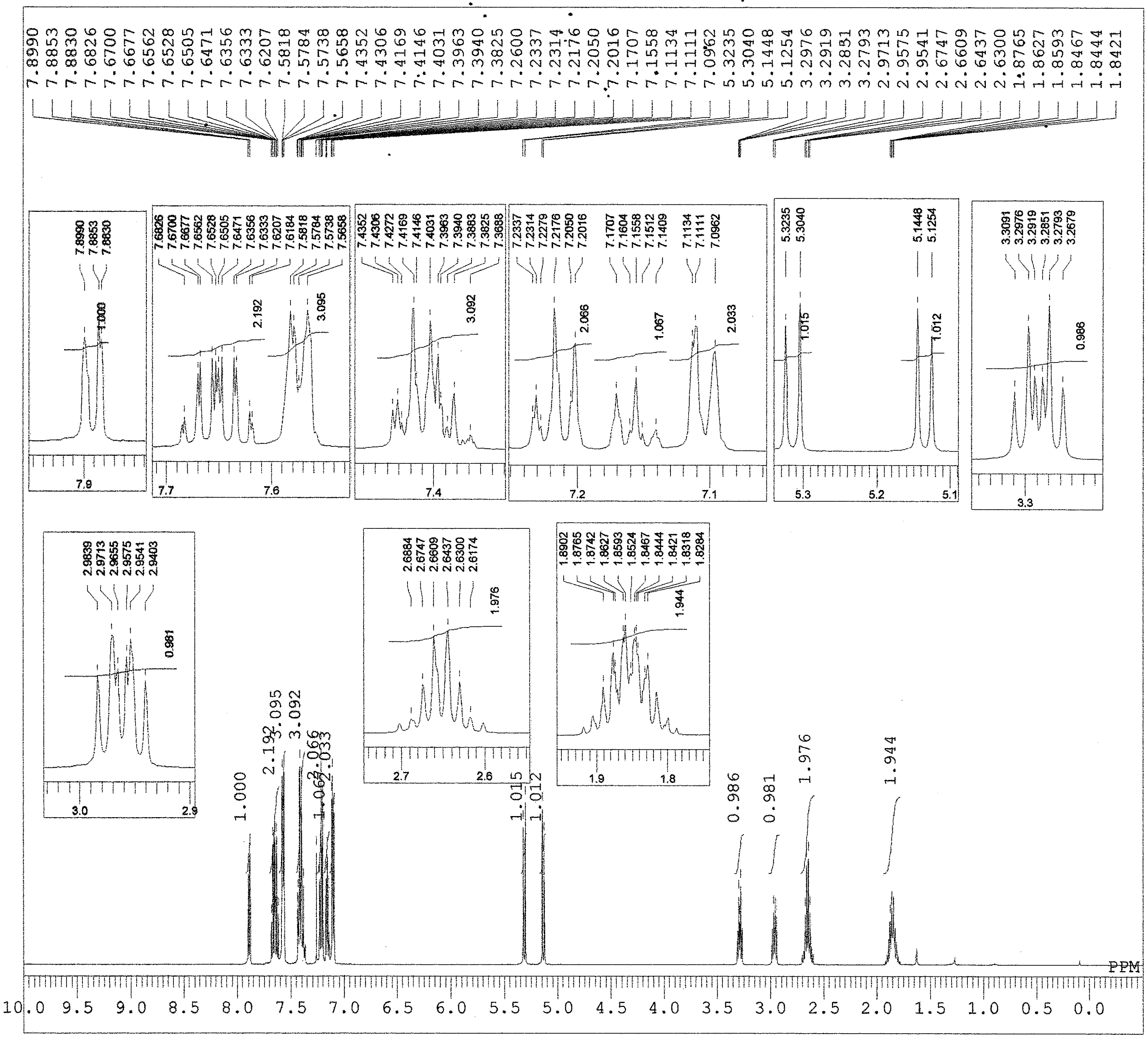

DFILE	ozawa04-115_1H.jdf
COMNT	PhPROH, Bn
DATIM	2014-01-31 20:48:11
OBNUC	1H
EXMOD	proton.jxp
OBFRQ	500.16 MHz
OBSET	2.41 KHz
OBFIN	6.01 Hz
POINT	16384
FREQU	9384.38 Hz
SCANS	4
ACQTM	1.7459 sec
PD	5.0000 sec
PW1	5.55 usec
IRNUC	1H
CTEMP	21.1 c
SLVNT	CDCL3
EXREF	7.26 ppm
BF	0.12 Hz
RGAIN	34

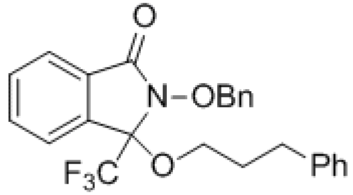

S4s

PhPROH, Bn

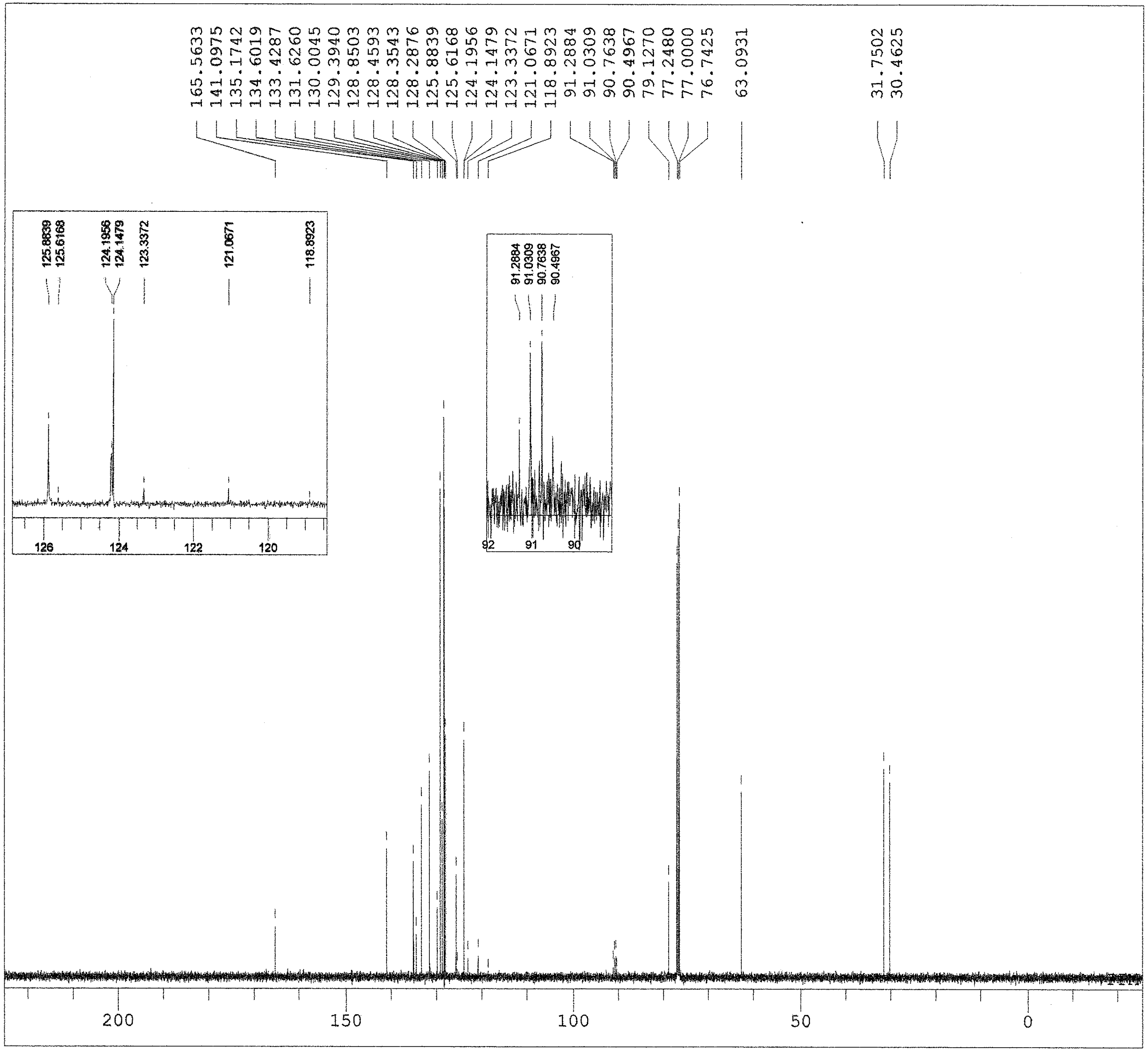

DFILE ozawa04-115_13C.jdf
COMNT PhPROH, Bn
DATIM 2014-01-31 20:49:14
OBNUC 13C
EXMOD carbon.jxp
OBFRQ 125.77 MHz
OBSET 7.87 KHz
OBFIN 4.21 Hz
POINT 32767
FREQU 39308.18 Hz
SCANS 308
ACQTM 0.8336 sec
PD 3.0000 sec
PW1 3.40 usec
IRNUC 1H
CTEMP 21.8 c
SLVNT CDCL3
EXREF 77.00 ppm
BF 0.12 Hz
RGAIN 60

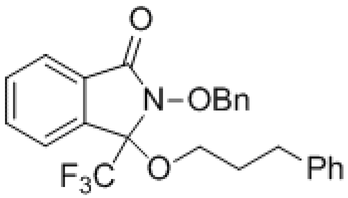

S4s

PhPrOH, Bn

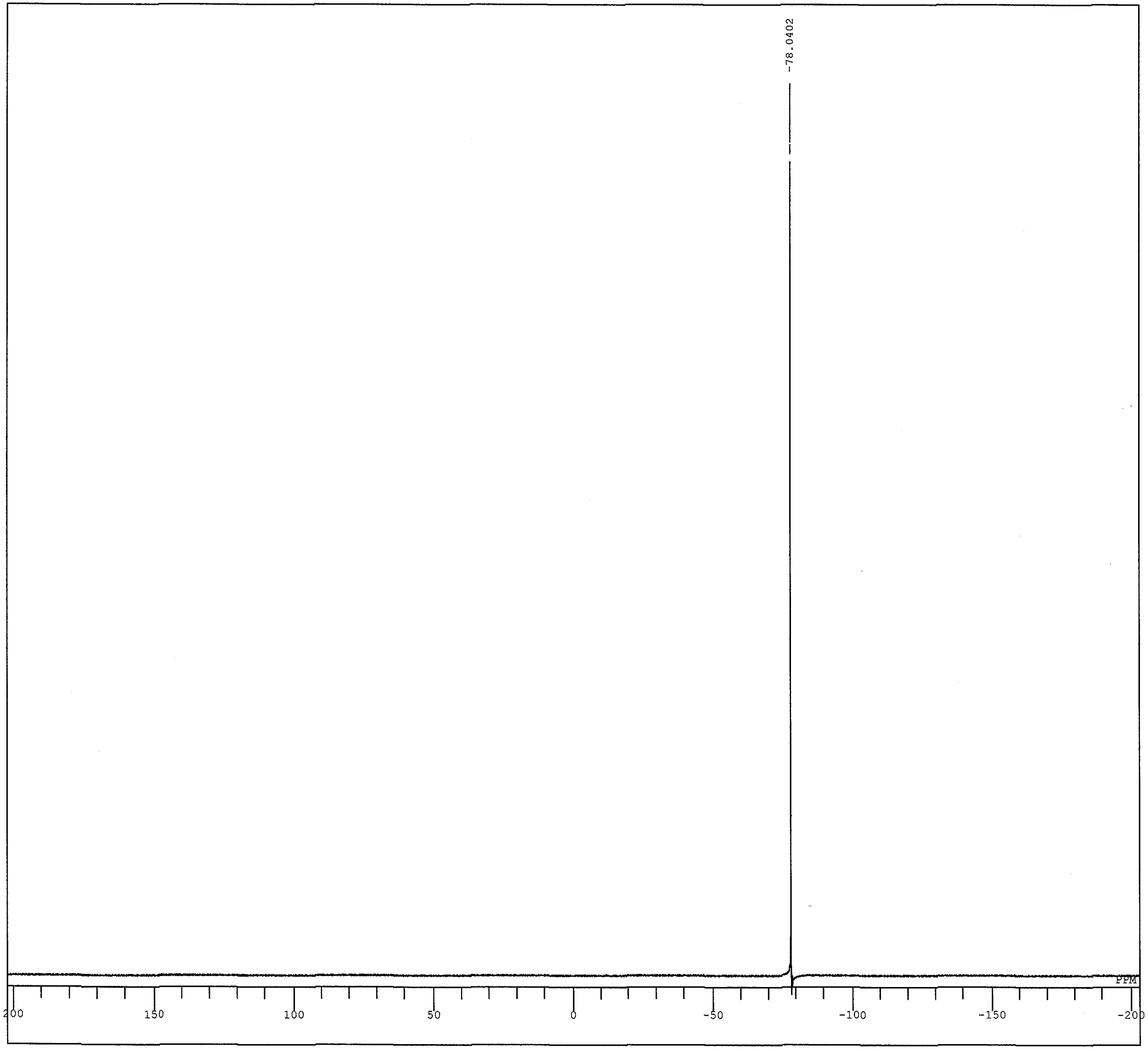

DFILE ozawa04-115_19F.jdf
COMNT PhPrOH, Bn
DATIM 31-01-2014 20:43:35
OBNUC 19F
EXMOD proton.jxp
OBFRQ 368.64 MHz
OBSET 7.63 KHz
OBFIN 2.85 Hz
POINT 16384
FREQU 186567.17 Hz
SCANS 4
ACQTM 0.0878 sec
PD 5.0000 sec
PW1 3.90 usec
IRNUC 19F
CTEMP 21.1 c
SLVNT CDCL3
EXREF -164.90 ppm
BF 0.12 Hz
RGAIN 48

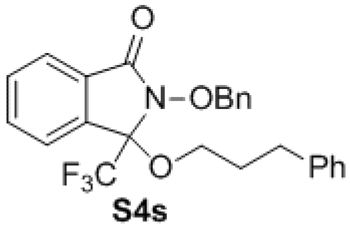

S4s

column, 3-Ph-1-propanol

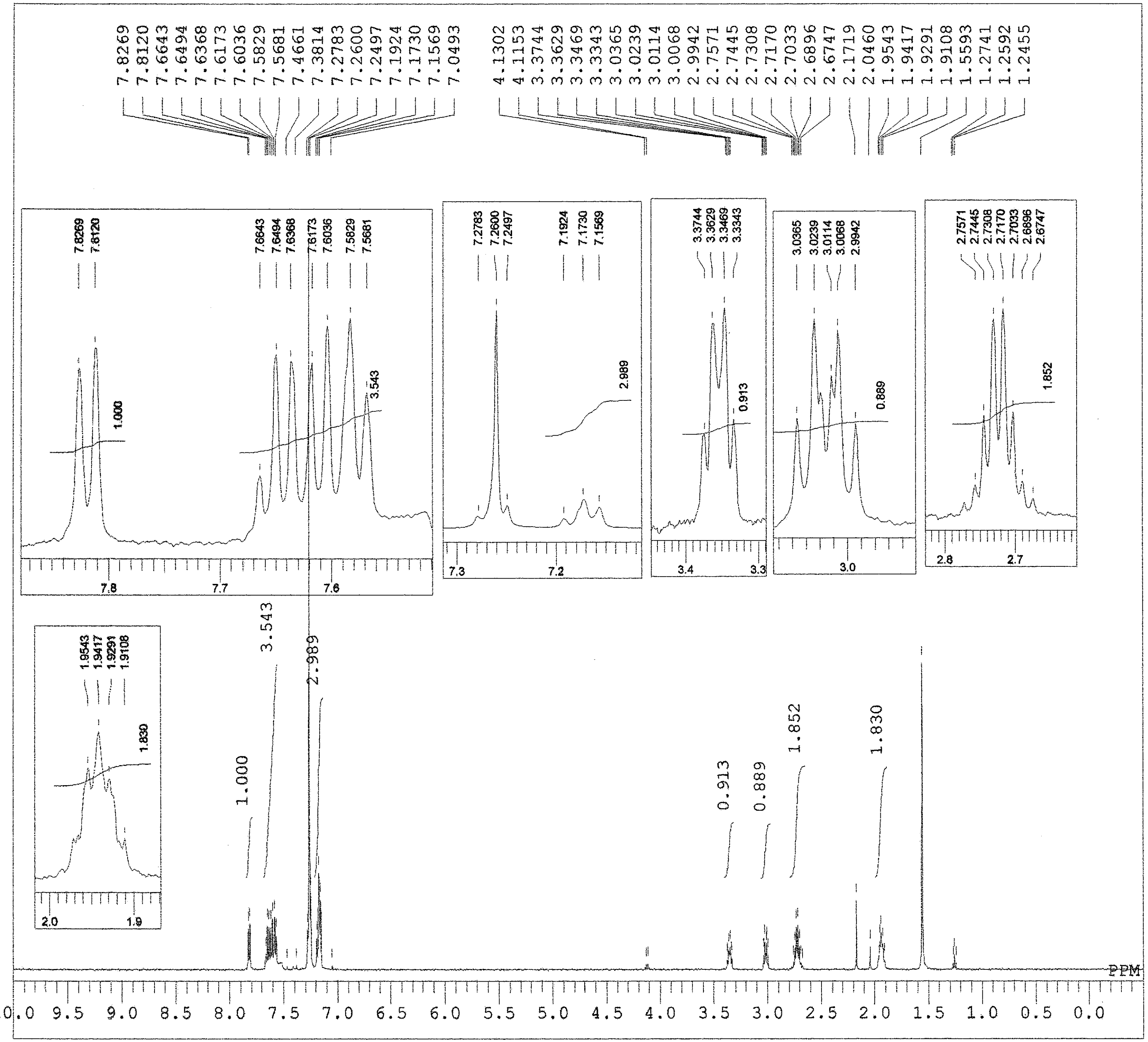

DFILE	ozawa03-198_1.jdf
COMNT	column, 3-Ph-1-propanol
DATIM	2013-10-10 20:11:21
OBNUC	1H
EXMOD	proton.jxp
OBFRQ	500.16 MHz
OBSET	2.41 KHz
OBFIN	6.01 Hz
POINT	16384
FREQU	9384.38 Hz
SCANS	4
ACQTM	1.7459 sec
PD	5.0000 sec
PW1	5.55 usec
IRNUC	1H
CTEMP	25.5 c
SLVNT	CDCL3
EXREF	7.26 ppm
BF	1.00 Hz
RGAIN	40

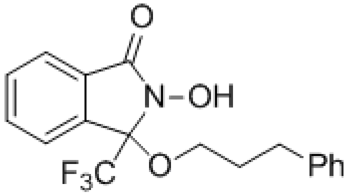

1s

PhPrOH, OH

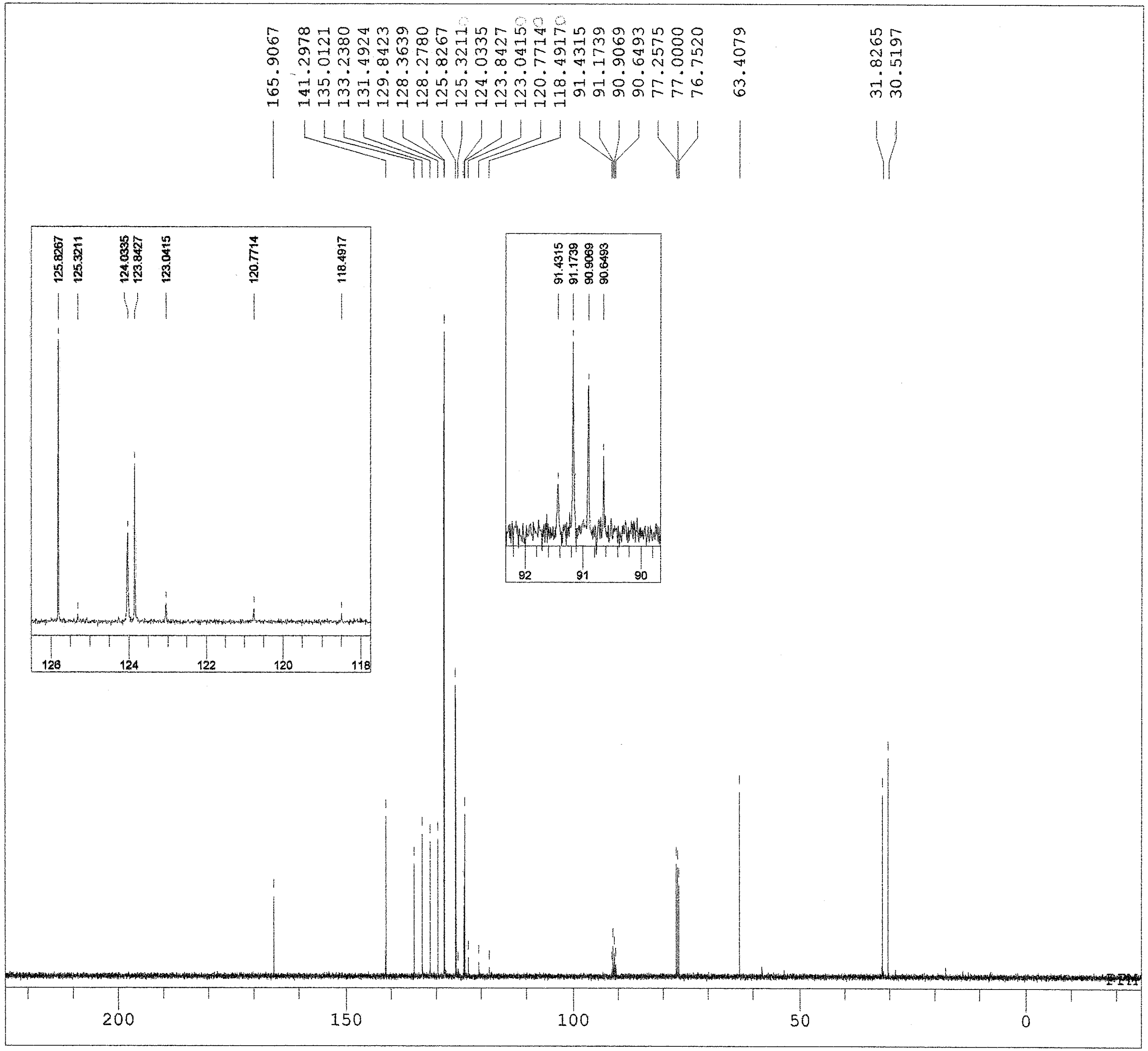

DFILE	ozawa03-198_13C.jdf
COMNT	PhPrOH, OH
DATIM	2014-01-29 12:20:25
OBNUC	13C
EXMOD	carbon.jxp
OBFRQ	125.77 MHz
OBSET	7.87 KHz
OBFIN	4.21 Hz
POINT	32767
FREQU	39308.18 Hz
SCANS	60
ACQTM	0.8336 sec
PD	3.0000 sec
PW1	3.40 usec
IRNUC	1H
CTEMP	21.9 c
SLVNT	CDCL3
EXREF	77.00 ppm
BF	0.12 Hz
RGAIN	60

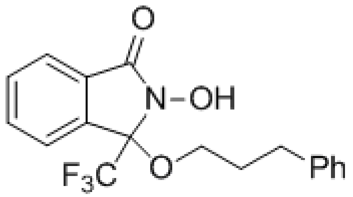

1s

PhPrOH, OH

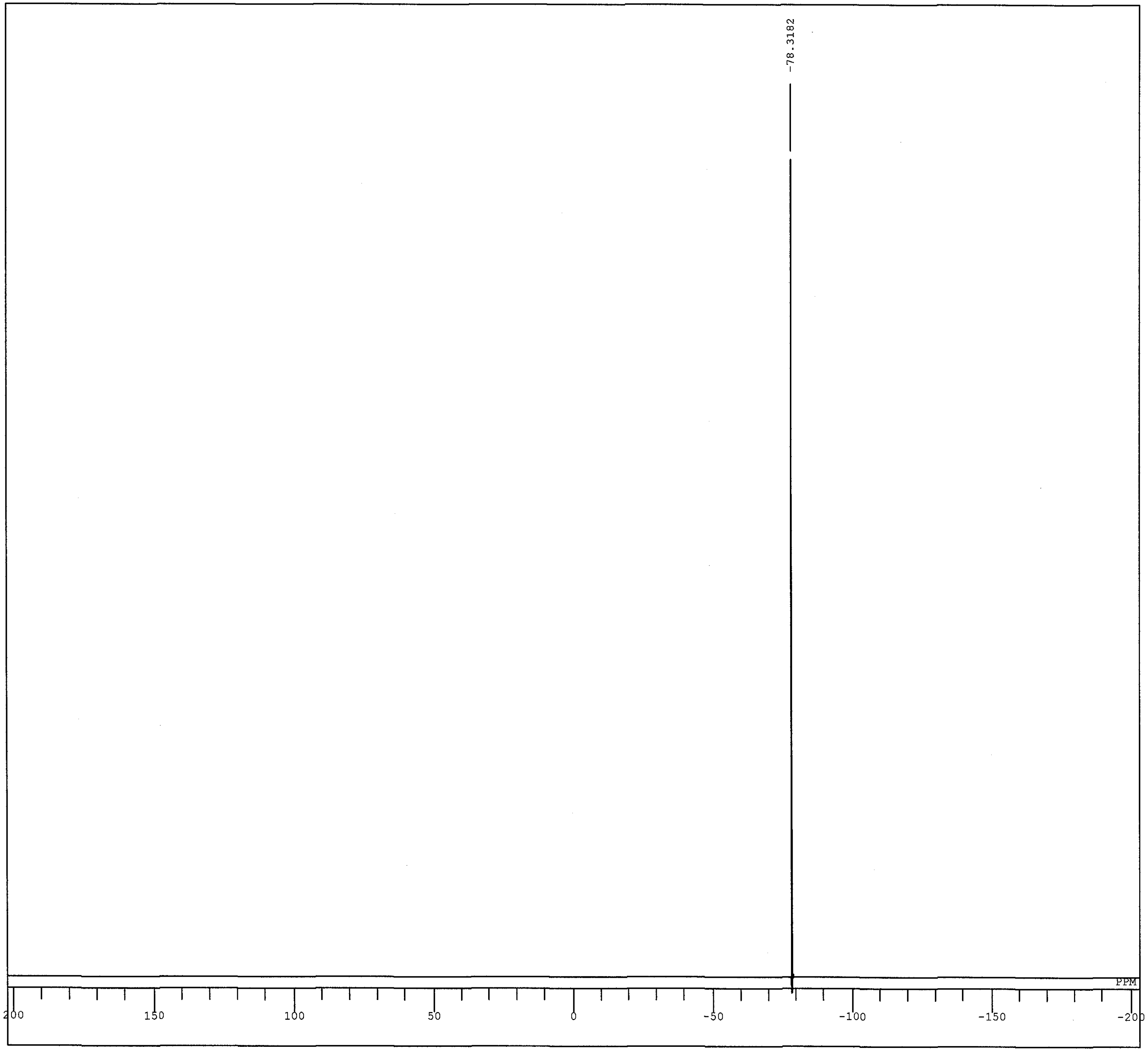

DFILE ozawa03-198_19F.jdf
COMNT PhPrOH, OH
DATIM 29-01-2014 12:48:08
OBNUC 19F
EXMOD proton.jxp
OBFRQ 368.64 MHz
OBSET 7.63 KHz
OBFIN 2.85 Hz
POINT 16384
FREQU 186567.17 Hz
SCANS 4
ACQTM 0.0878 sec
PD 5.0000 sec
PW1 3.90 usec
IRNUC 19F
CTEMP 20.9 c
SLVNT CDCL3
EXREF -164.90 ppm
BF 0.12 Hz
RGAIN 42

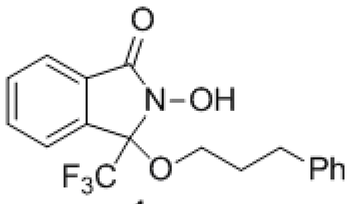

1s

PhPrOH, [O]

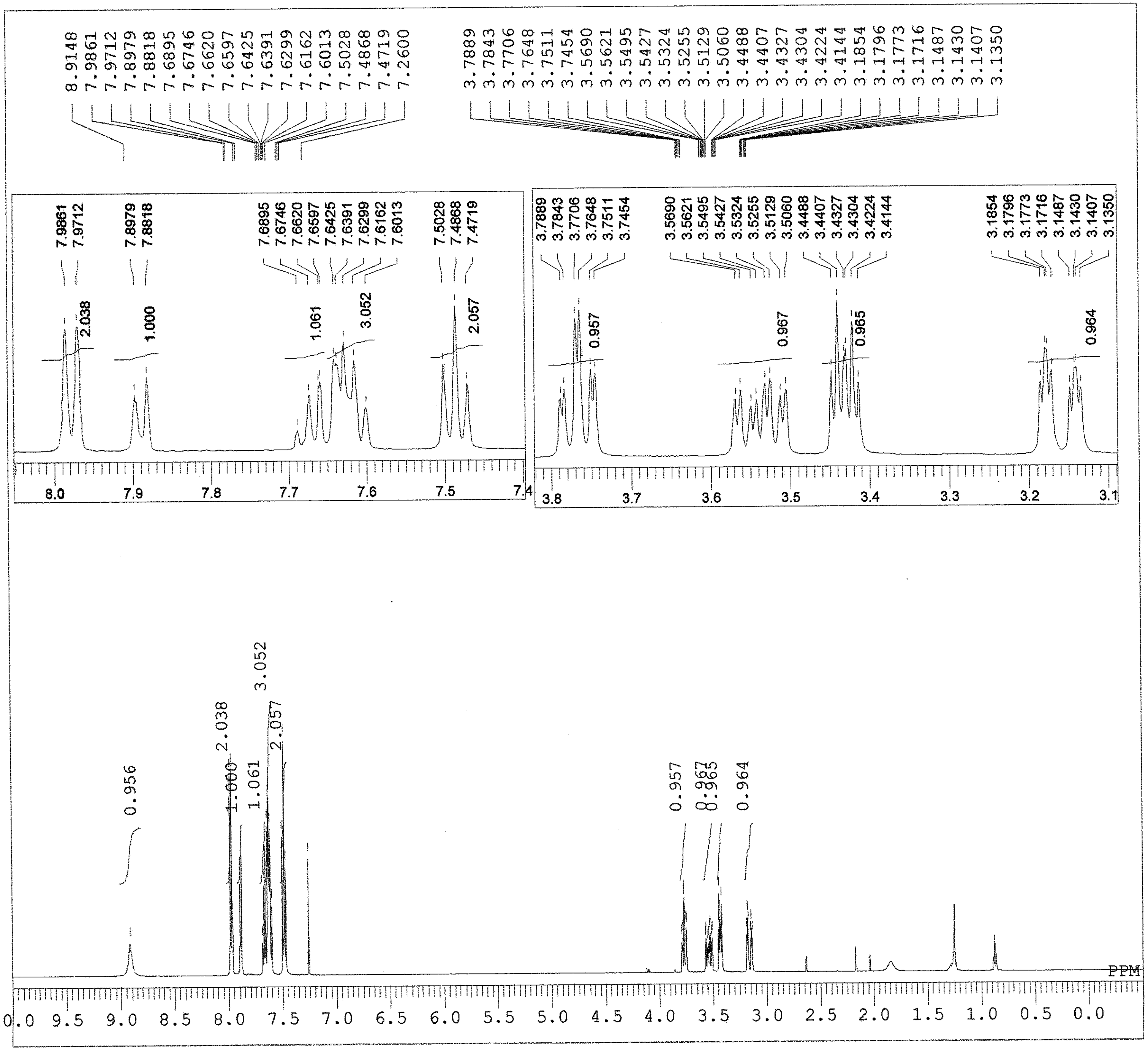

DFILE ozawa04-117_1H.als
COMNT PhPrOH, [O]
DATIM 2014-02-03 22:08:42
OBNUC 1H
EXMOD proton.jxp
OBFRQ 500.16 MHz
OBSET 2.41 KHz
OBFIN 6.01 Hz
POINT 13107
FREQU 7507.51 Hz
SCANS 4
ACQTM 1.7459 sec
PD 5.0000 sec
PW1 5.55 usec
IRNUC 1H
CTEMP 21.3 c
SLVNT CDCL3
EXREF 7.26 ppm
BF 0.20 Hz
RGAIN 36

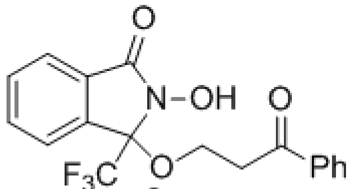

2s

PhPrOH, [O]

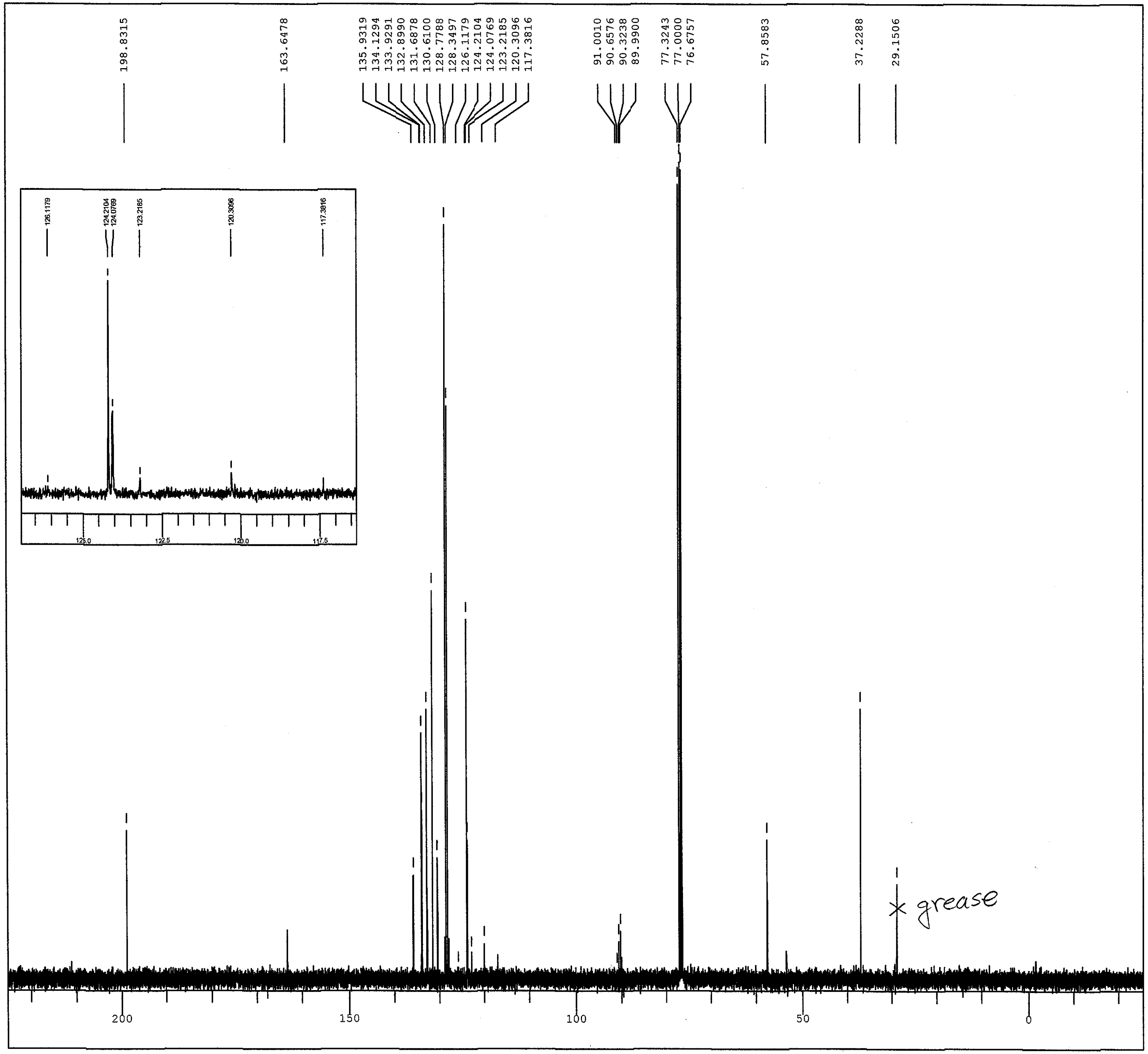

DFILE ozawa04-117_13C.jdf
COMNT PhPrOH, [O]
DATIM 02-02-2014 20:47:05
OBNUC 13C
EXMOD carbon.jxp
OBFRQ 98.52 MHz
OBSET 4.64 KHz
OBFIN 8.74 Hz
POINT 32767
FREQU 30788.18 Hz
SCANS 308
ACQTM 1.0643 sec
PD 3.0000 sec
PW1 3.00 usec
IRNUC 1H
CTEMP 21.2 c
SLVNT CDCL3
EXREF 77.00 ppm
BF 0.12 Hz
RGAIN 60

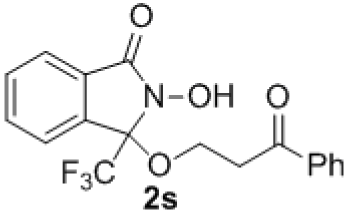

2s

PhPr-OH
PTLC, ~~BuOH,~~ [0]

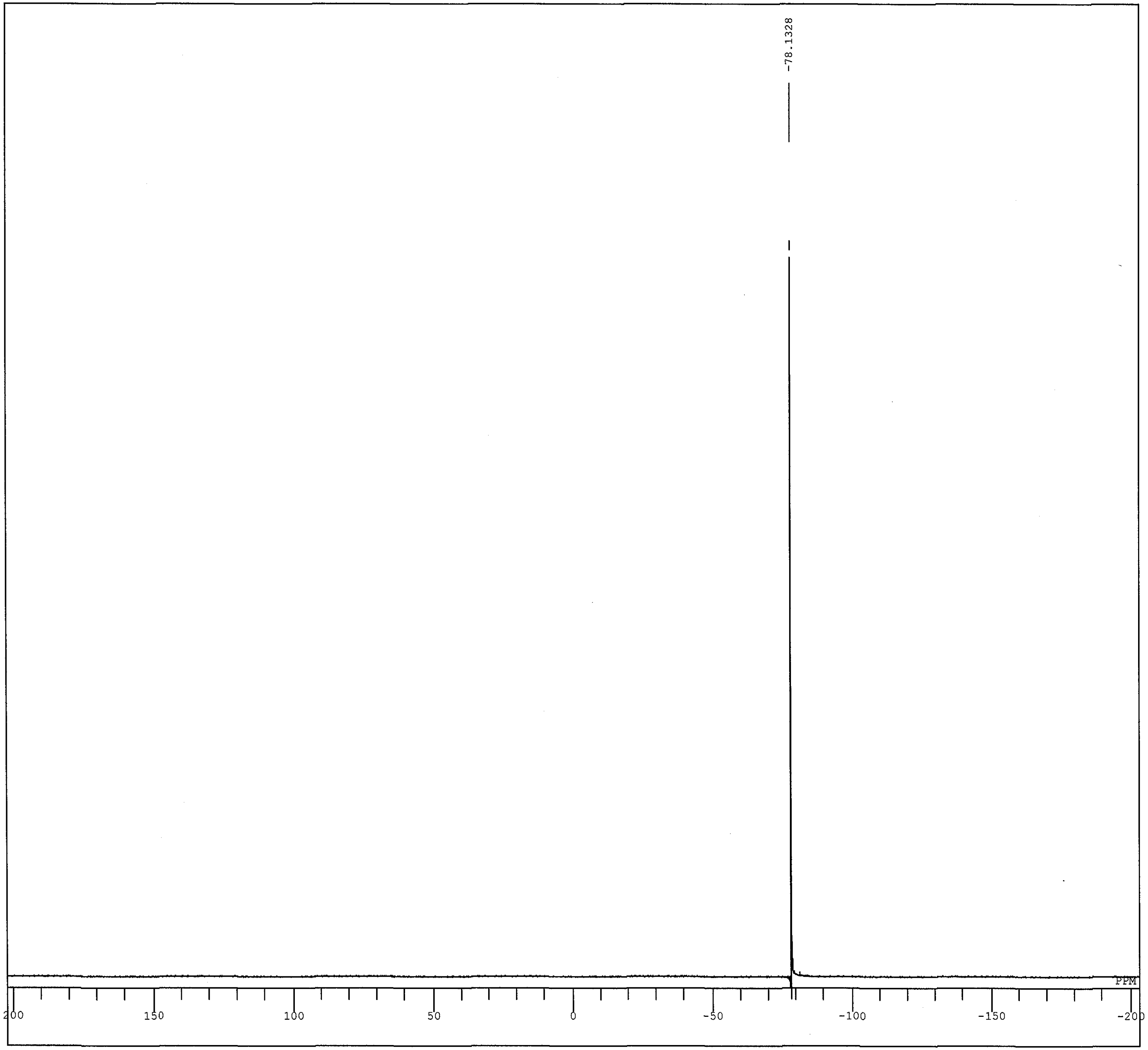


```
DFILE  ozawa04-109_2_19F.jdf
COMNT  PTLC, BuOH, [0]
DATIM  24-01-2014 21:00:10
OBNUC  19F
EXMOD  proton.jxp
OBFRQ        368.64 MHz
OBSET          7.63 KHz
OBFIN          2.85 Hz
POINT         16384
FREQU     186567.17 Hz
SCANS             4
ACQTM        0.0878 sec
PD           5.0000 sec
PW1            3.90 usec
IRNUC  19F
CTEMP          21.0 c
SLVNT  CDCL3
EXREF       -164.90 ppm
BF             0.12 Hz
RGAIN            46
```

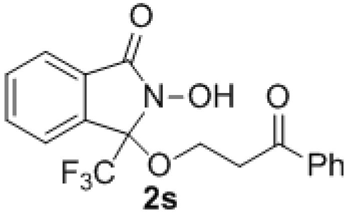

2s

PhOAc, Bn

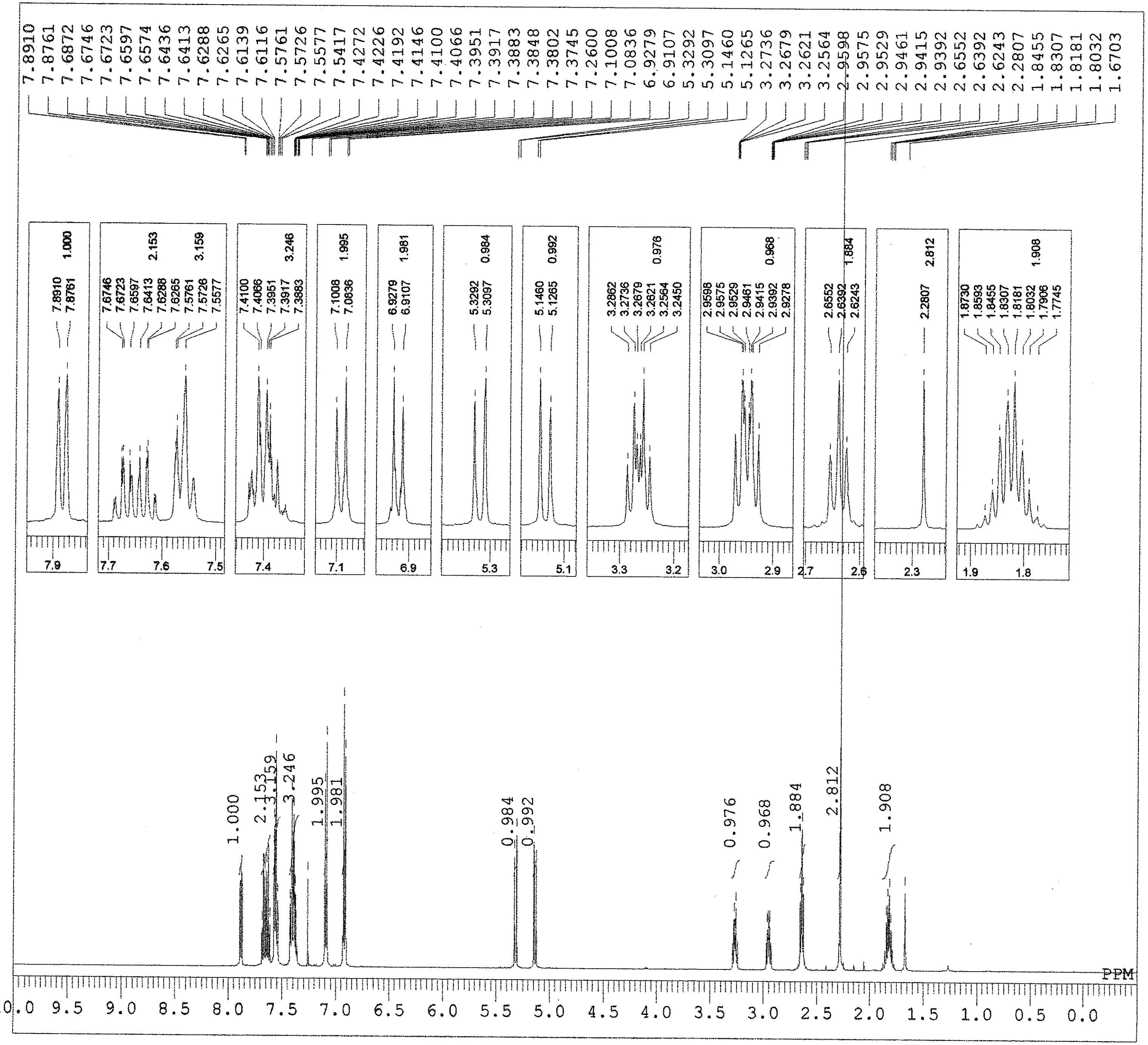

DFILE ozawa05-048_1H.jdf
COMNT PhOAc, Bn
DATIM 2014-08-15 13:12:30
OBNUC 1H
EXMOD proton.jxp
OBFRQ 500.16 MHz
OBSET 2.41 KHz
OBFIN 6.01 Hz
POINT 16384
FREQU 9384.38 Hz
SCANS 4
ACQTM 1.7459 sec
PD 5.0000 sec
PW1 5.55 usec
IRNUC 1H
CTEMP 26.0 c
SLVNT CDCL3
EXREF 7.26 ppm
BF 0.12 Hz
RGAIN 32

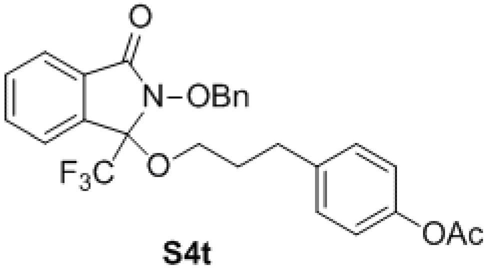

S4t

PhOAc, Bn

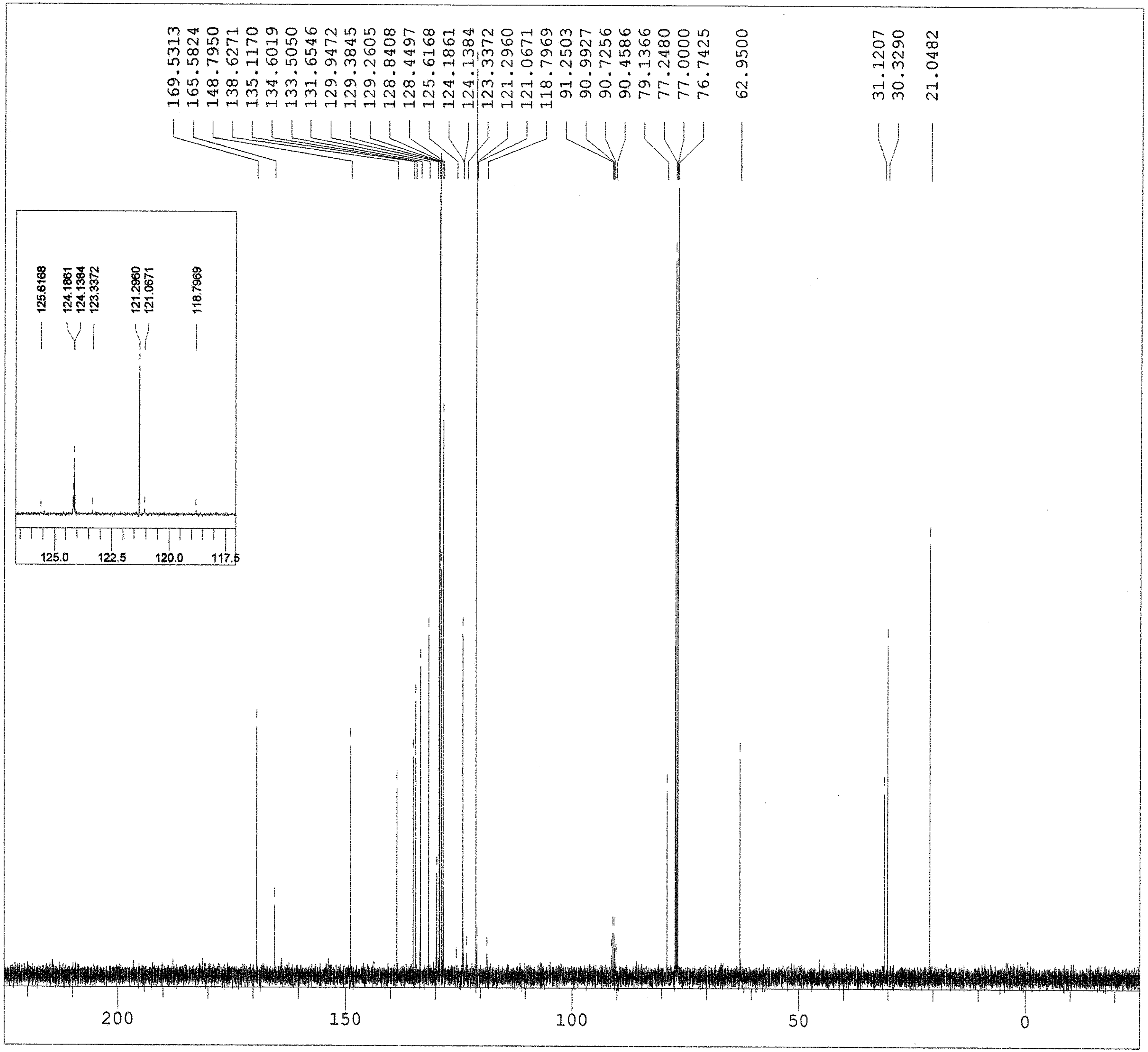

DFILE ozawa05-048_13C.jdf
COMNT PhOAc, Bn
DATIM 2014-08-15 13:13:33
OBNUC 13C
EXMOD carbon.jxp
OBFRQ 125.77 MHz
OBSET 7.87 KHz
OBFIN 4.21 Hz
POINT 32767
FREQU 39308.18 Hz
SCANS 180
ACQTM 0.8336 sec
PD 3.0000 sec
PW1 3.40 usec
IRNUC 1H
CTEMP 26.4 c
SLVNT CDCL3
EXREF 77.00 ppm
BF 0.12 Hz
RGAIN 60

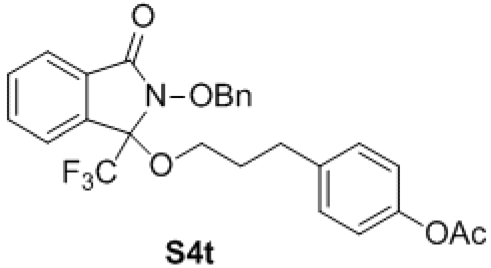

S4t

PhOAc, Bn

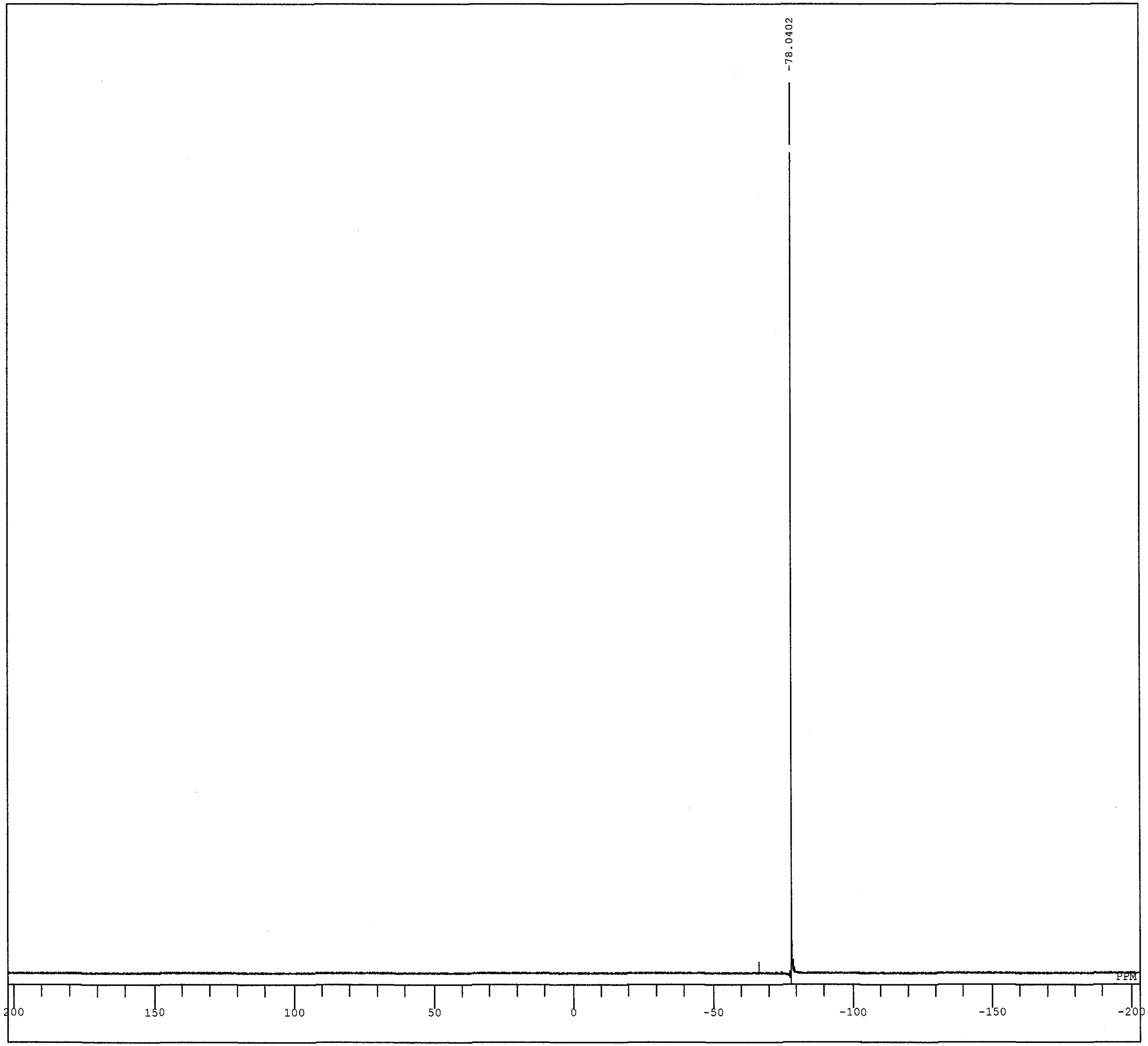

DFILE ozawa05-048_19F.jdf
COMNT PhOAc, Bn
DATIM 15-08-2014 13:57:18
OBNUC 19F
EXMOD proton.jxp
OBFRQ 368.64 MHz
OBSET 7.63 KHz
OBFIN 2.85 Hz
POINT 16384
FREQU 186567.17 Hz
SCANS 4
ACQTM 0.0878 sec
PD 5.0000 sec
PW1 3.90 usec
IRNUC 19F
CTEMP 24.2 c
SLVNT CDCL3
EXREF -164.90 ppm
BF 0.12 Hz
RGAIN 50

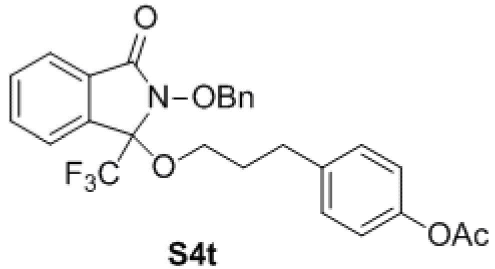

S4t

PhOAc

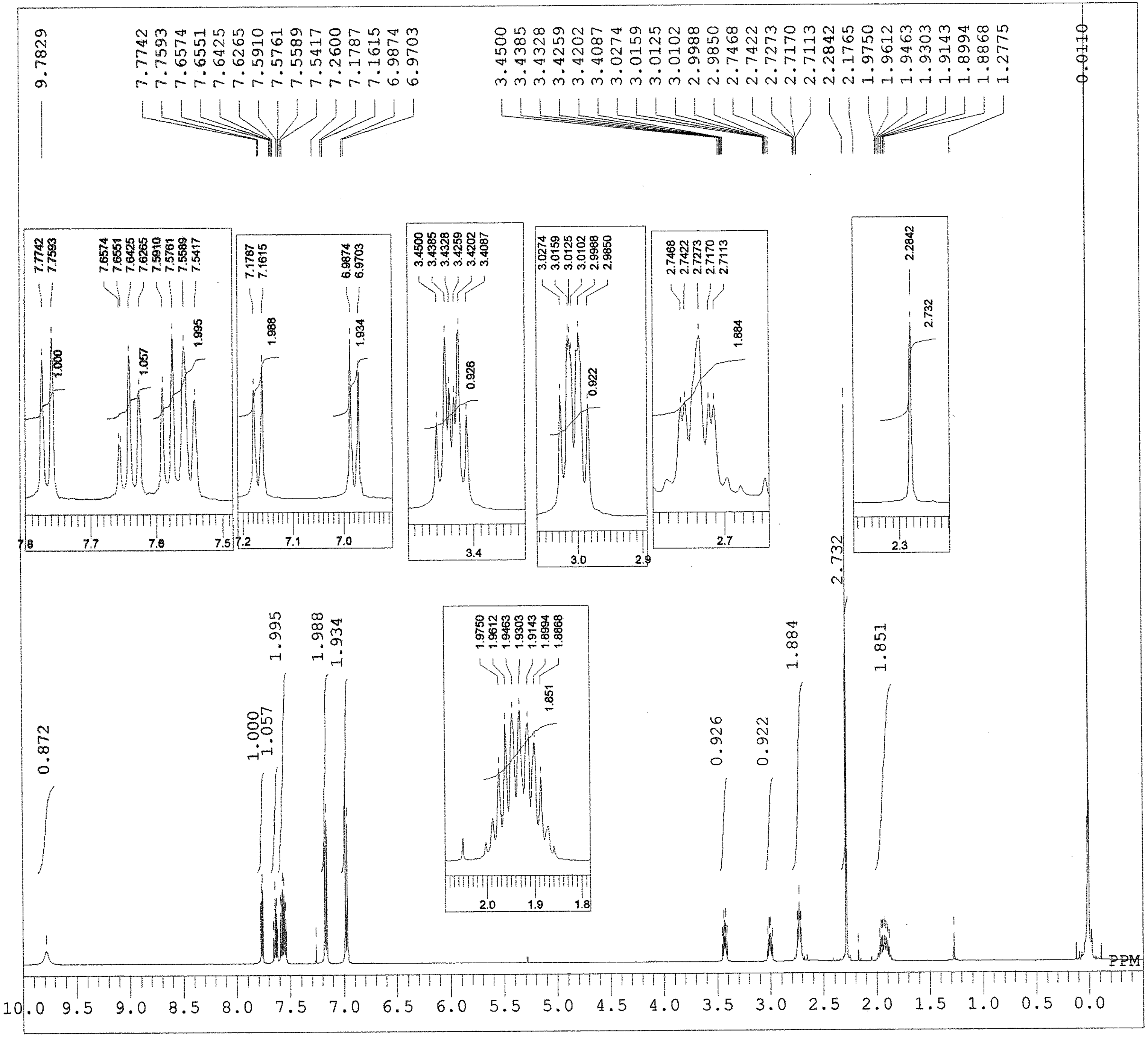

DFILE	ozawa05-054_1H.jdf
COMNT	PhOAc
DATIM	2014-06-14 13:17:60
OBNUC	1H
EXMOD	proton.jxp
OBFRQ	500.16 MHz
OBSET	2.41 KHz
OBFIN	6.01 Hz
POINT	16384
FREQU	9384.38 Hz
SCANS	4
ACQTM	1.7459 sec
PD	5.0000 sec
PW1	5.55 usec
IRNUC	1H
CTEMP	25.4 c
SLVNT	CDCL3
EXREF	7.26 ppm
BF	0.12 Hz
RGAIN	26

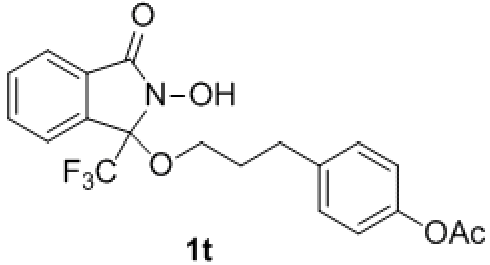

1t

PhOAc

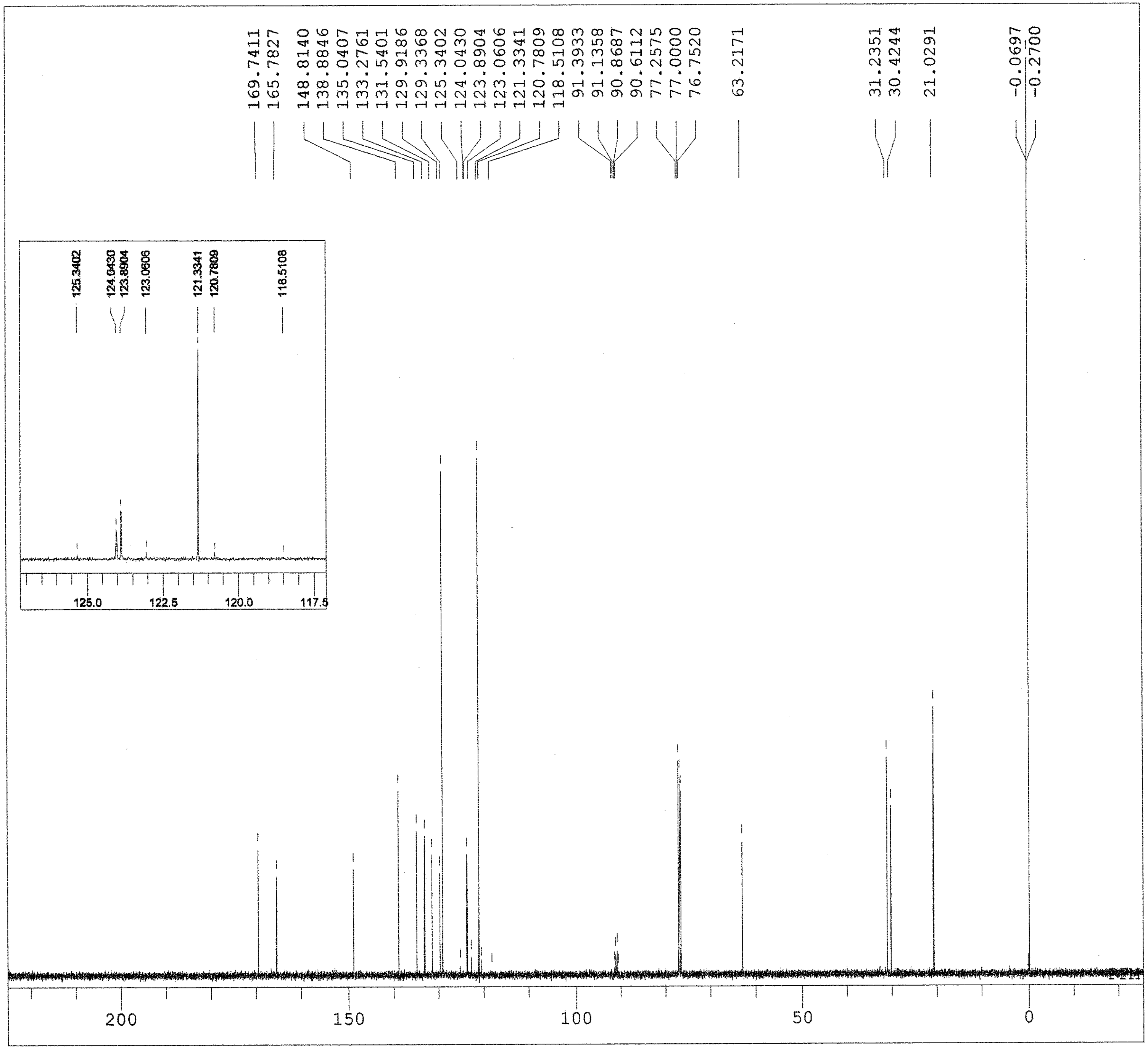

DFILE	ozawa05-054_13C.jdf
COMNT	PhOAc
DATIM	2014-06-14 13:19:03
OBNUC	13C
EXMOD	carbon.jxp
OBFRQ	125.77 MHz
OBSET	7.87 KHz
OBFIN	4.21 Hz
POINT	32767
FREQU	39308.18 Hz
SCANS	108
ACQTM	0.8336 sec
PD	3.0000 sec
PW1	3.40 usec
IRNUC	1H
CTEMP	26.0 c
SLVNT	CDCL3
EXREF	77.00 ppm
BF	0.12 Hz
RGAIN	60

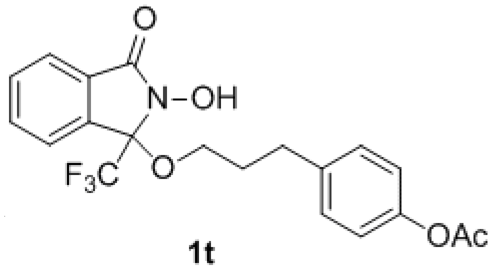

1t

Ph-OAc

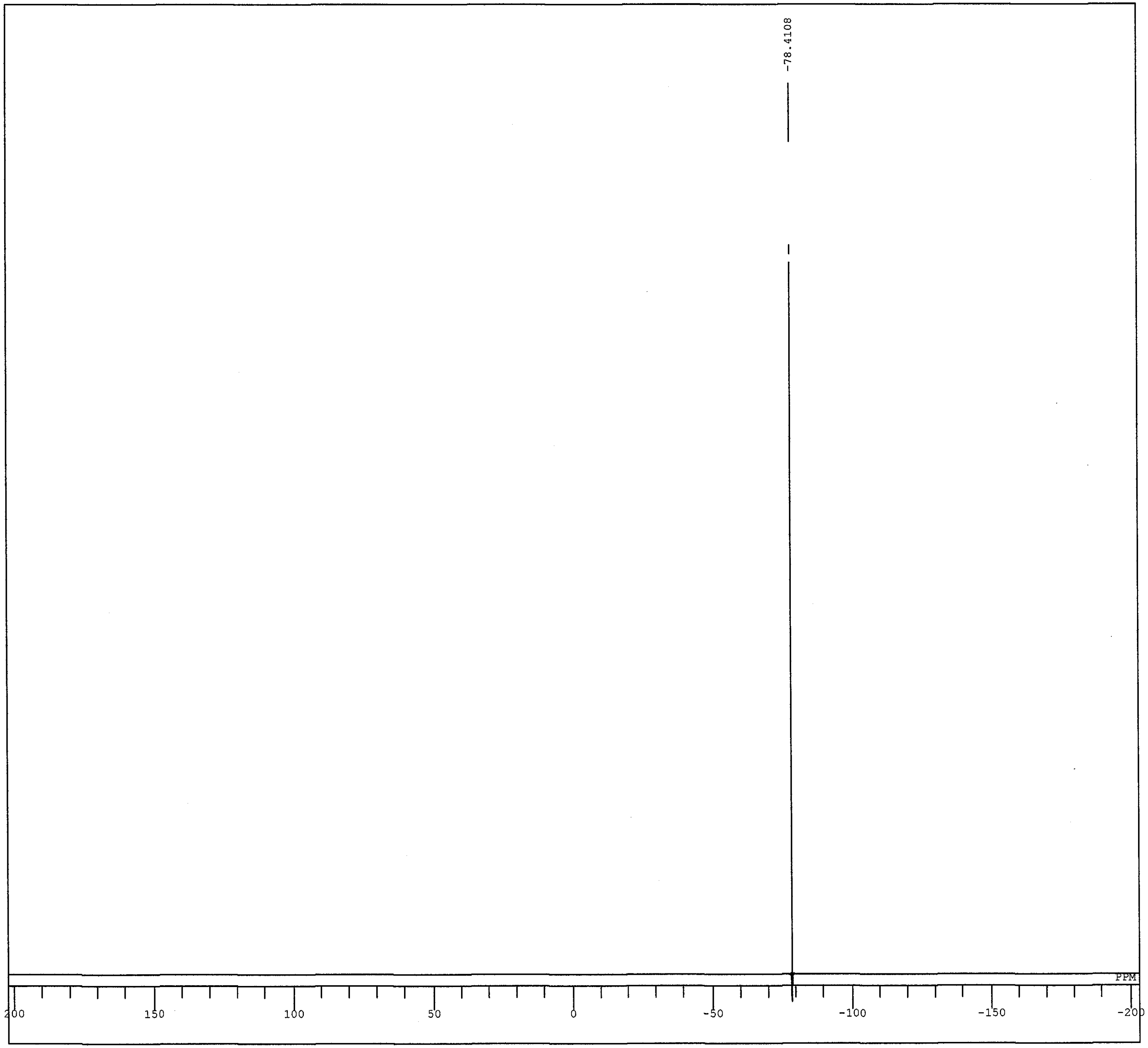

DFILE ozawa05-054_19F.jdf
COMNT Ph-OAc
DATIM 14-06-2014 14:44:50
OBNUC 19F
EXMOD proton.jxp
OBFRQ 368.64 MHz
OBSET 7.63 KHz
OBFIN 2.85 Hz
POINT 16384
FREQU 186567.17 Hz
SCANS 4
ACQTM 0.0878 sec
PD 5.0000 sec
PW1 3.90 usec
IRNUC 19F
CTEMP 23.5 c
SLVNT CDCL3
EXREF -164.90 ppm
BF 0.12 Hz
RGAIN 50

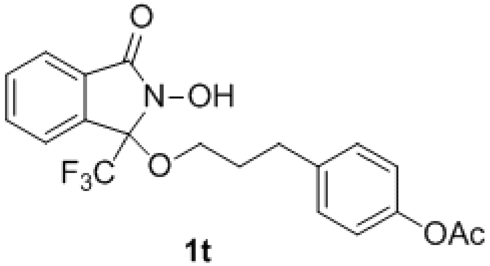

1t

PhOAc, [O]

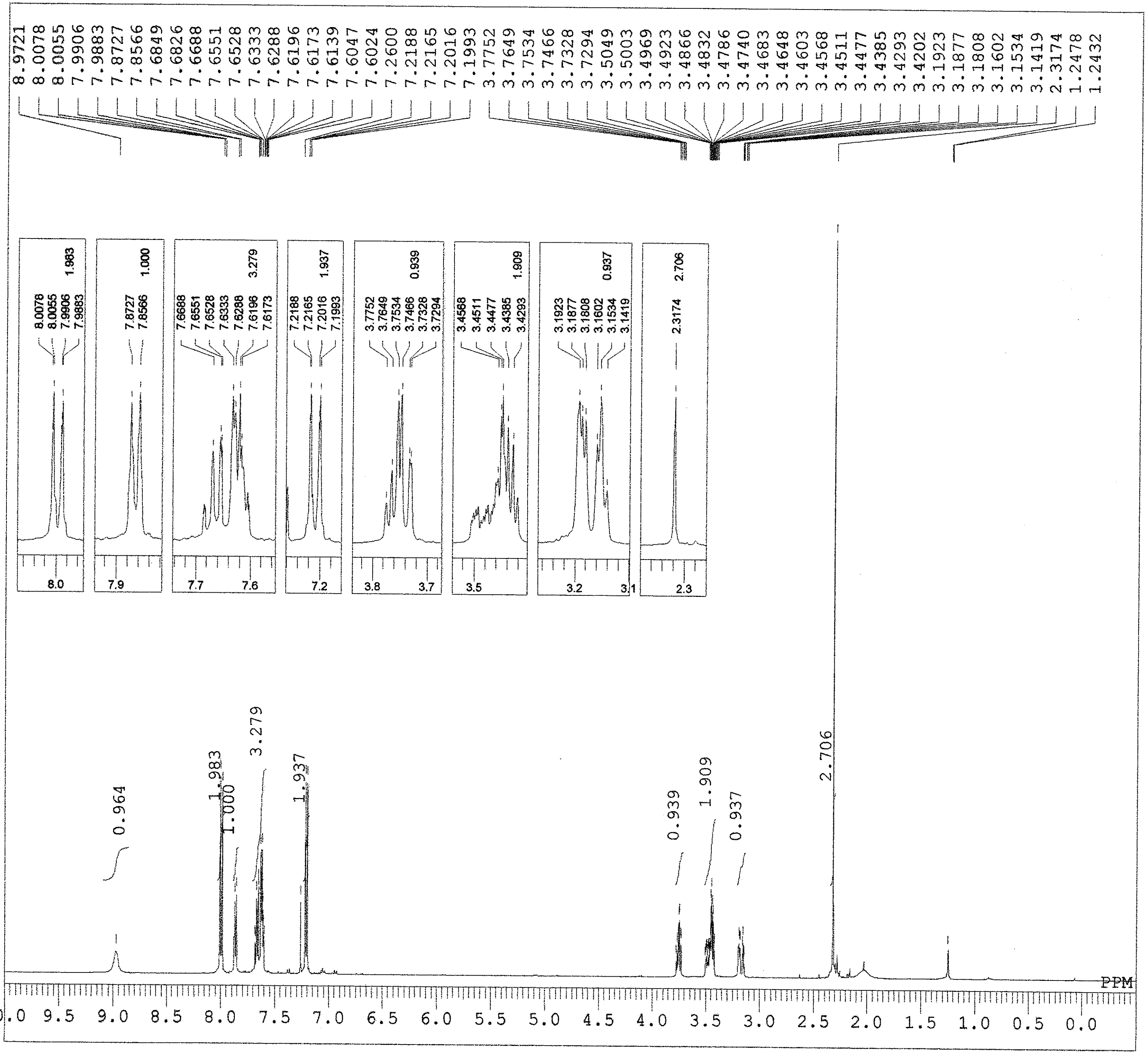

DFILE	ozawa05-095_1_1H.jdf
COMNT	PhOAc, [O]
DATIM	2014-08-16 12:54:38
OBNUC	1H
EXMOD	proton.jxp
OBFRQ	500.16 MHz
OBSET	2.41 KHz
OBFIN	6.01 Hz
POINT	16384
FREQU	9384.38 Hz
SCANS	4
ACQTM	1.7459 sec
PD	5.0000 sec
PW1	5.55 usec
IRNUC	1H
CTEMP	25.9 c
SLVNT	CDCL3
EXREF	7.26 ppm
BF	0.12 Hz
RGAIN	34

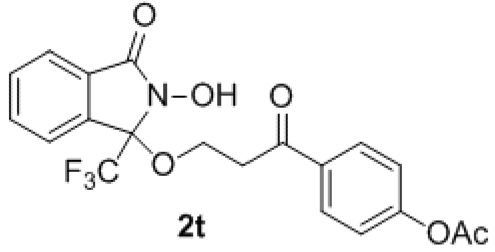

2t

PhOAc, [O]

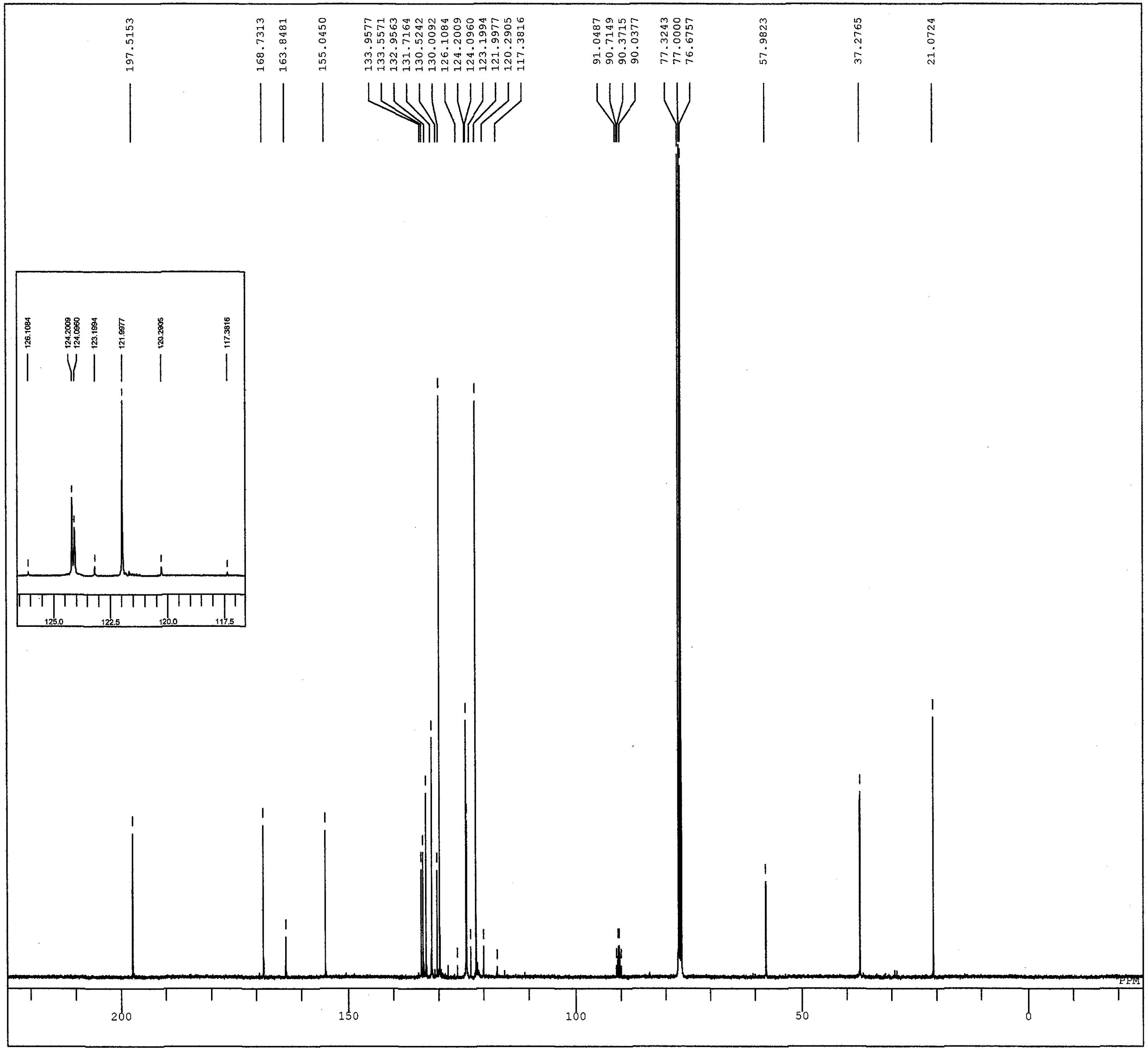

DFILE ozawa05-095_1_13C.jdf
COMNT PhOAc, [O]
DATIM 15-08-2014 22:46:13
OBNUC 13C
EXMOD carbon.jxp
OBFRQ 98.52 MHz
OBSET 4.64 KHz
OBFIN 8.74 Hz
POINT 32767
FREQU 30788.18 Hz
SCANS 10000
ACQTM 1.0643 sec
PD 3.0000 sec
PW1 3.00 usec
IRNUC 1H
CTEMP 24.4 c
SLVNT CDCL3
EXREF 77.00 ppm
BF 1.20 Hz
RGAIN 60

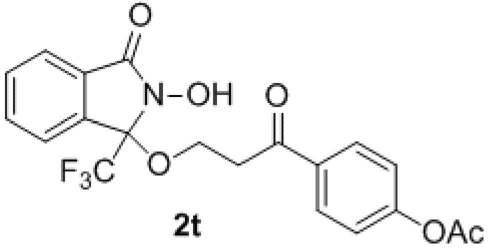

2t

PhOAc, [O]

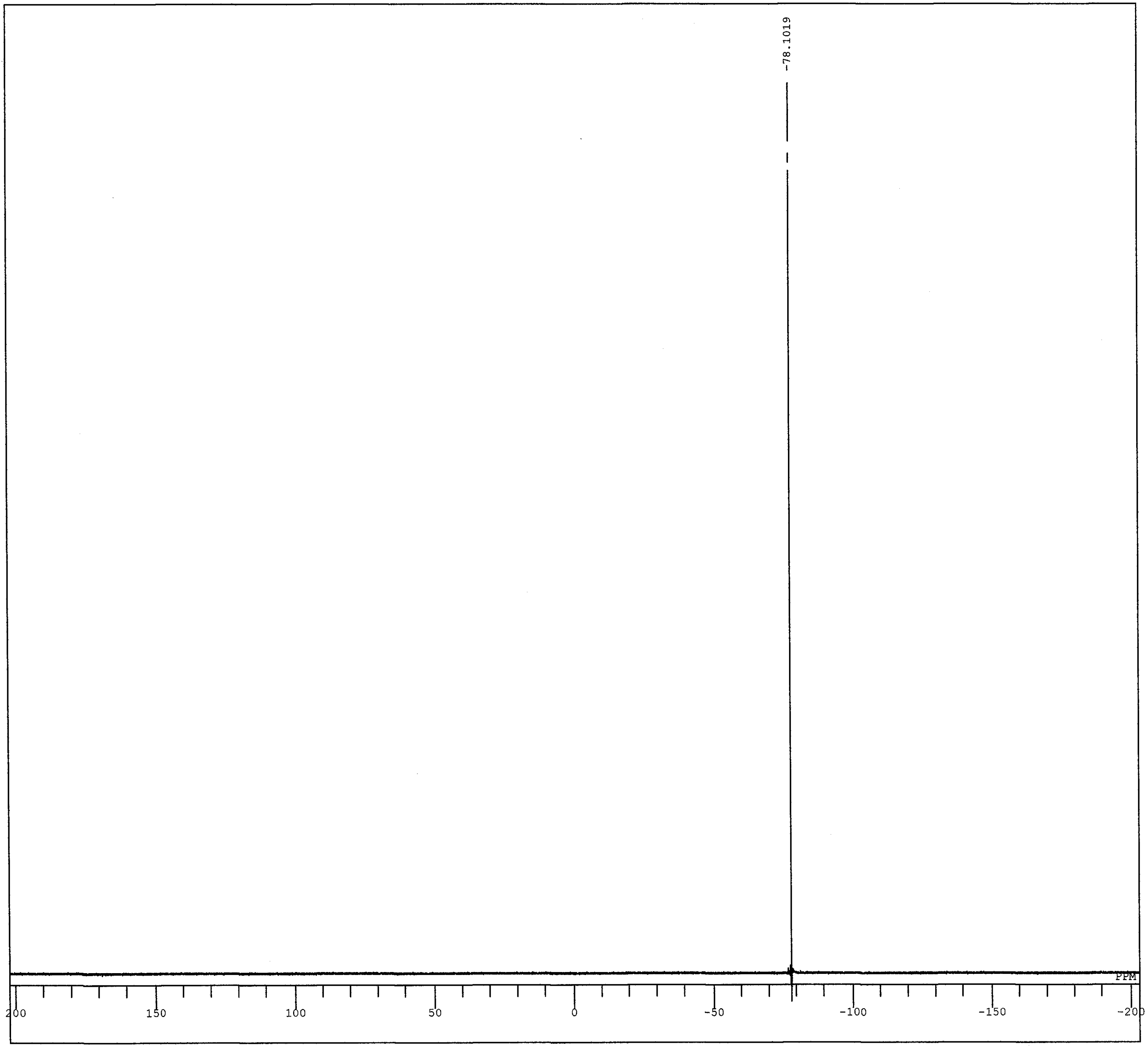

DFILE ozawa05-095_1_19F.jdf
COMNT PhOAc, [O]
DATIM 07-07-2014 18:30:14
OBNUC 19F
EXMOD proton.jxp
OBFRQ 368.64 MHz
OBSET 7.63 KHz
OBFIN 2.85 Hz
POINT 16384
FREQU 186567.17 Hz
SCANS 4
ACQTM 0.0878 sec
PD 5.0000 sec
PW1 3.90 usec
IRNUC 19F
CTEMP 24.3 c
SLVNT CDCL3
EXREF -164.90 ppm
BF 0.12 Hz
RGAIN 50

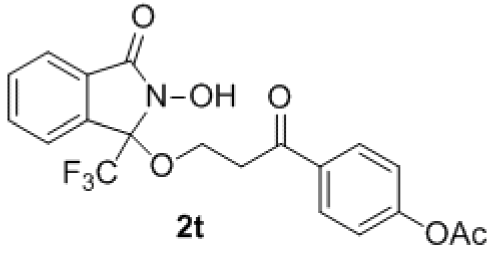

2t

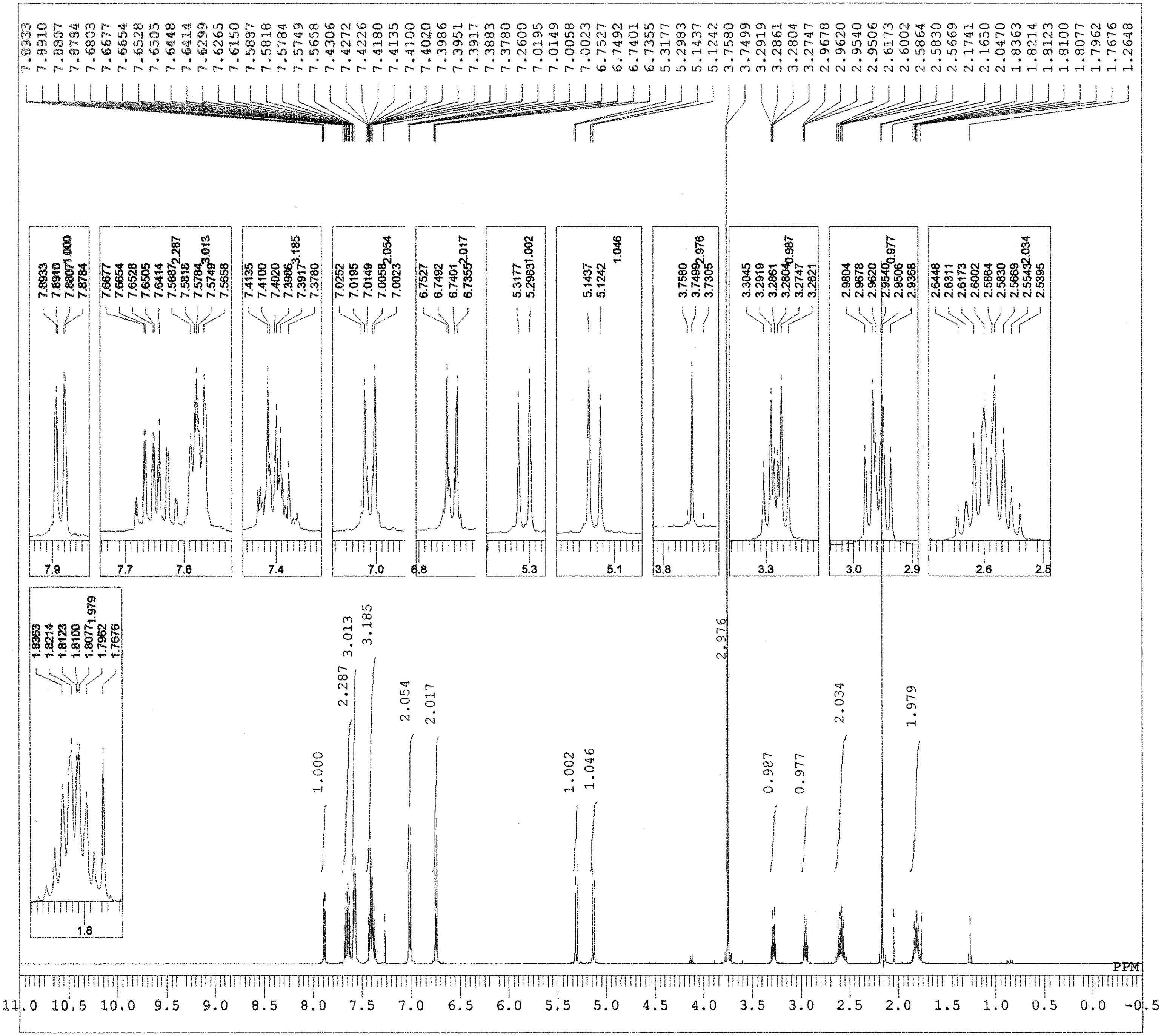

DFILE ozawa07-048_1H.als
COMNT 4-OMe-Ph, Bn
DATIM 2015-03-19 08:31:00
OBNUC 1H
EXMOD proton.jxp
OBFRQ 500.16 MHz
OBSET 2.41 KHz
OBFIN 6.01 Hz
POINT 13107
FREQU 7507.51 Hz
SCANS 4
ACQTM 1.7459 sec
PD 5.0000 sec
PW1 5.55 usec
IRNUC 1H
CTEMP 25.7 c
SLVNT CDCL3
EXREF 7.26 ppm
BF 0.12 Hz
RGAIN 26

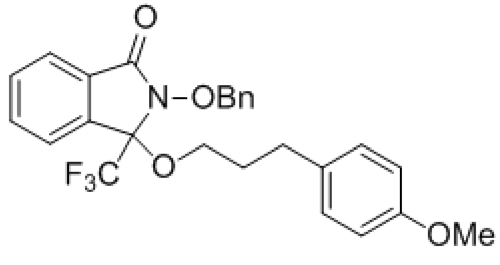

S4u

4-OMe-Ph, Bn

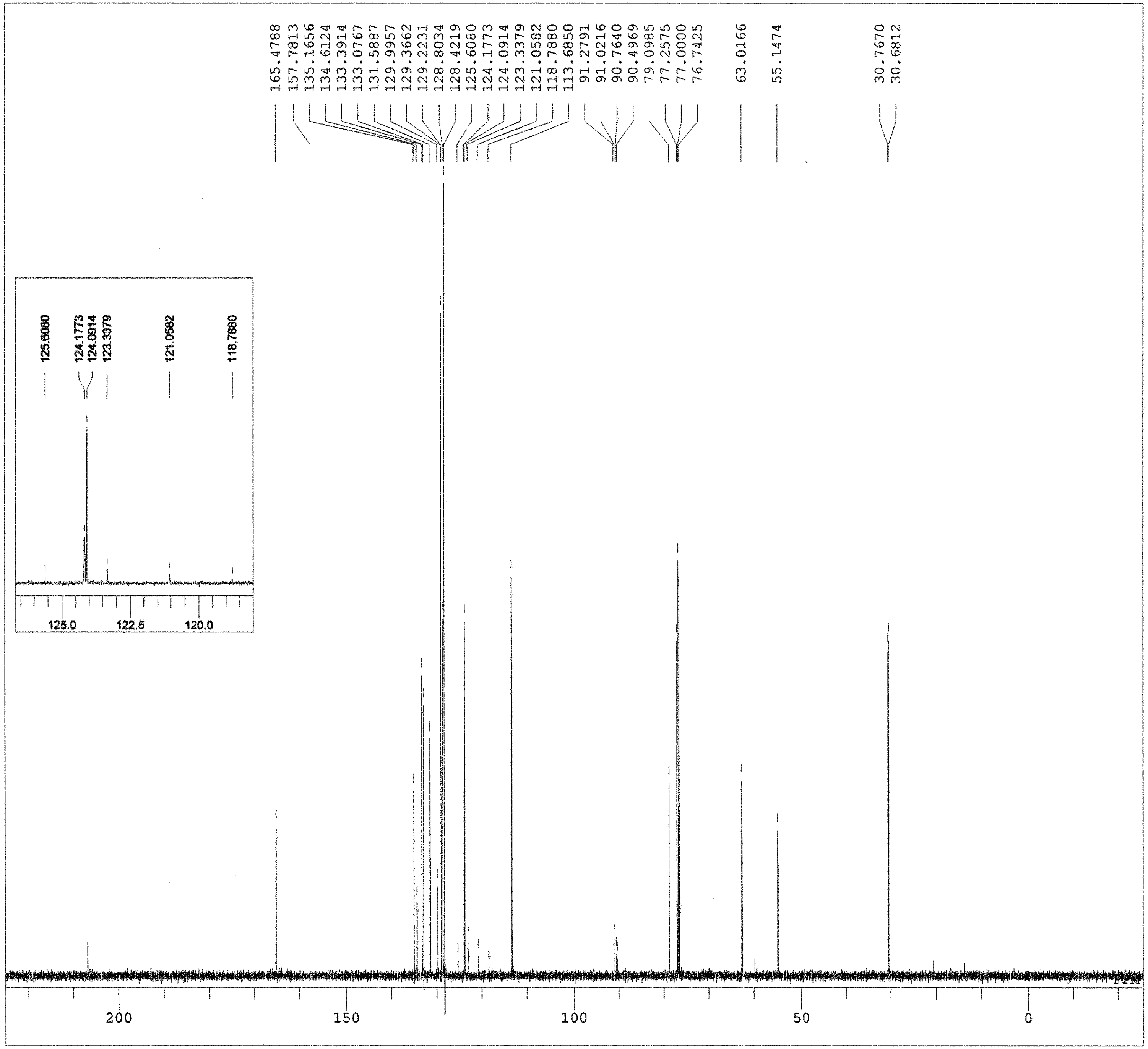

DFILE	ozawa07-048_13C.als
COMNT	4-OMe-Ph, Bn
DATIM	2015-03-19 08:32:02
OBNUC	13C
EXMOD	carbon.jxp
OBFRQ	125.77 MHz
OBSET	7.87 KHz
OBFIN	4.21 Hz
POINT	26214
FREQU	31446.54 Hz
SCANS	244
ACQTM	0.8336 sec
PD	2.0000 sec
PW1	3.40 usec
IRNUC	1H
CTEMP	26.3 c
SLVNT	CDCL3
EXREF	77.00 ppm
BF	0.12 Hz
RGAIN	60

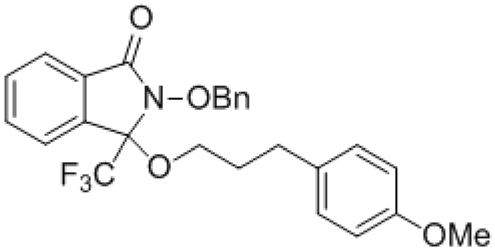

S4u

4-OMe-Ph, Bn

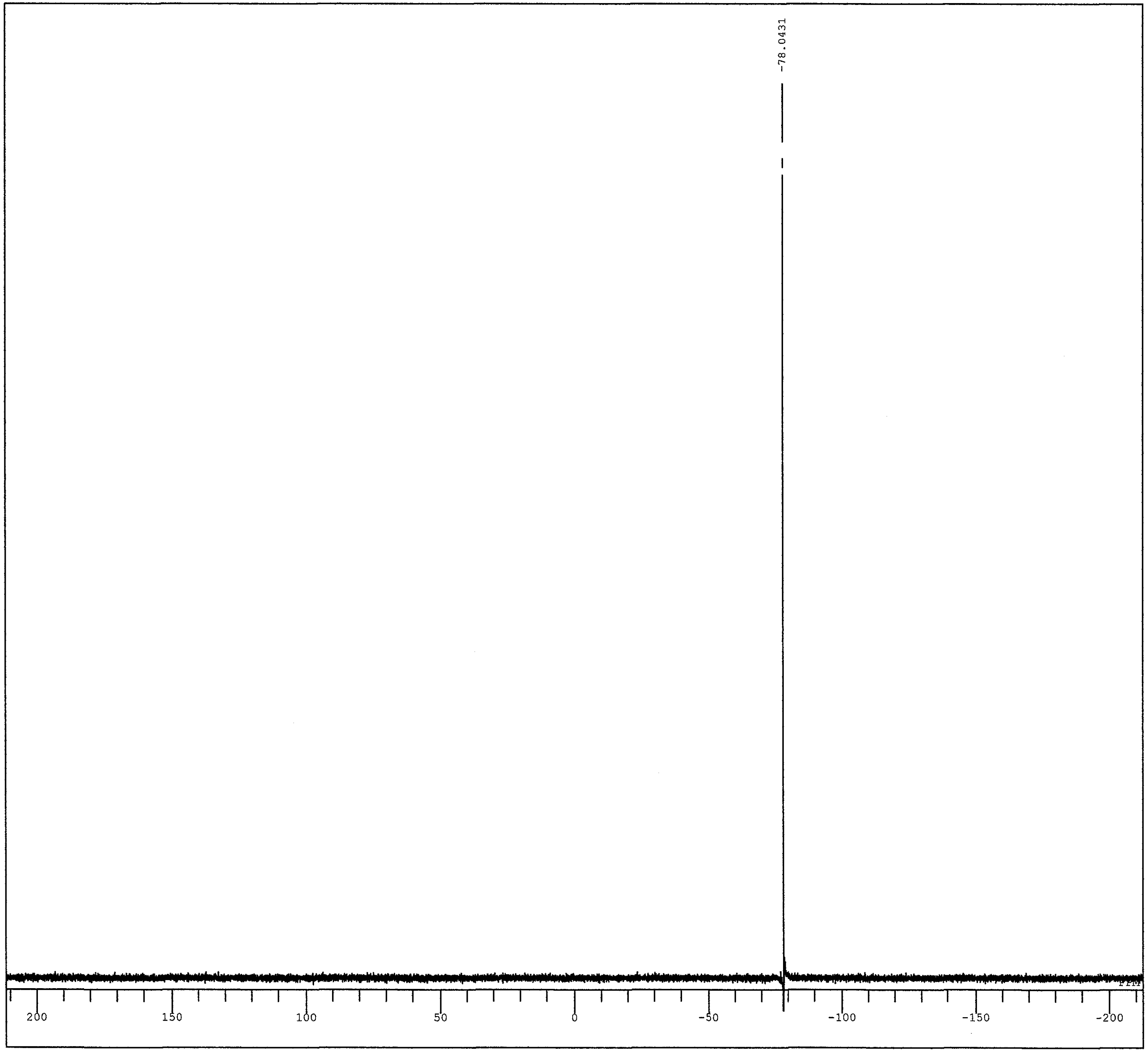

DFILE ozawa07-048_19F.jdf
COMNT 4-OMe-Ph, Bn
DATIM 21-03-2015 12:13:59
OBNUC 19F
EXMOD proton.jxp
OBFRQ 368.64 MHz
OBSET 7.63 KHz
OBFIN 2.85 Hz
POINT 16384
FREQU 195312.50 Hz
SCANS 4
ACQTM 0.0839 sec
PD 5.0000 sec
PW1 3.90 usec
IRNUC 19F
CTEMP 25.1 c
SLVNT CDCL3
EXREF -164.90 ppm
BF 0.12 Hz
RGAIN 50

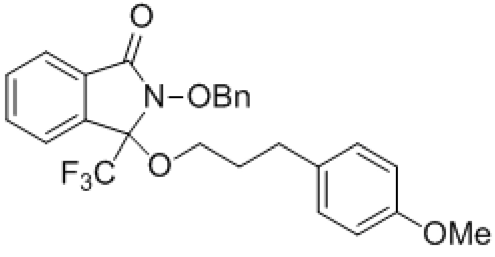

S4u

4-OMe-Ph, OH

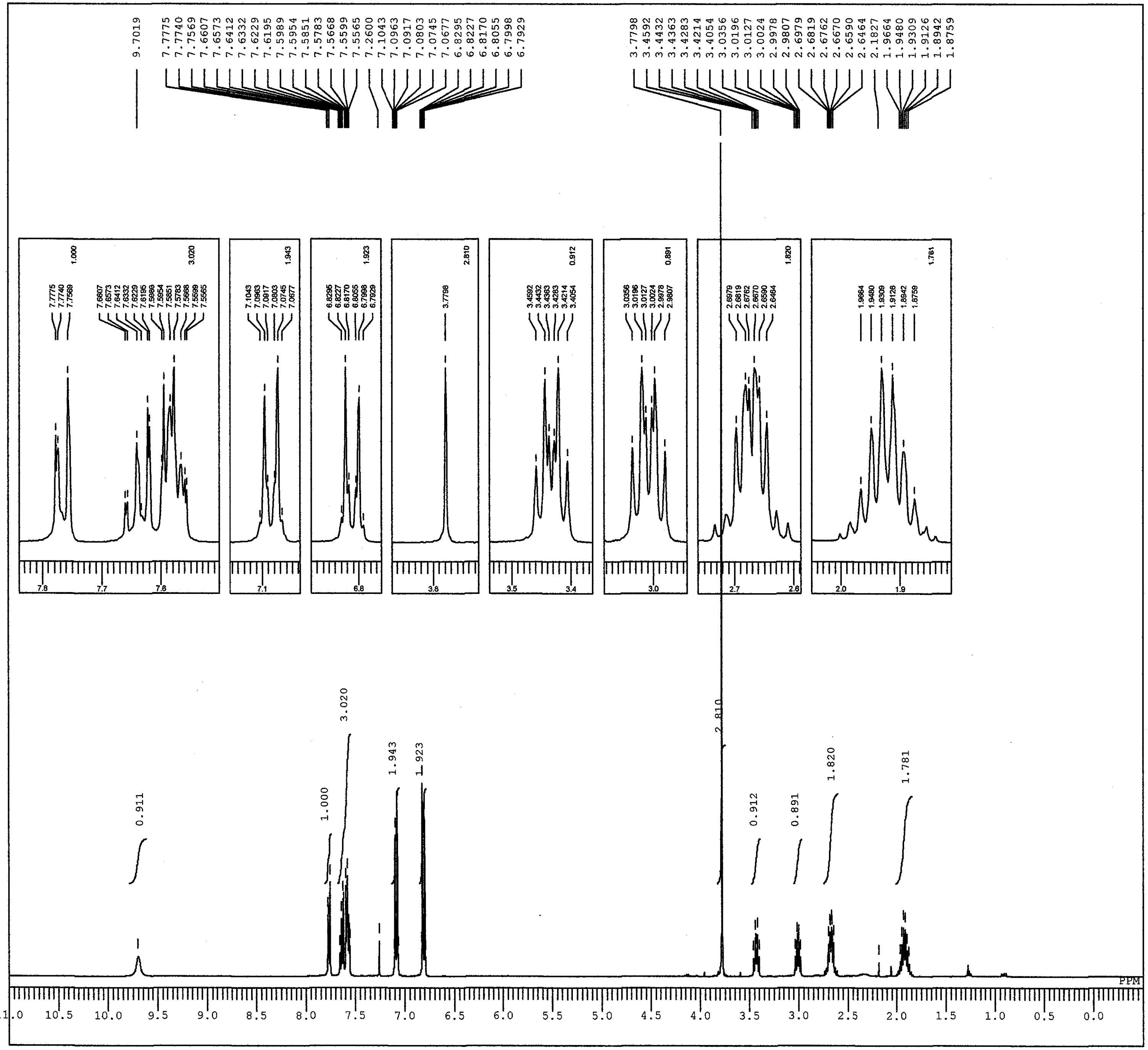

DFILE ozawa07-049_1H.jdf
COMNT 4-OMe-Ph, OH
DATIM 20-03-2015 17:32:44
OBNUC 1H
EXMOD proton.jxp
OBFRQ 391.78 MHz
OBSET 8.51 KHz
OBFIN 3.34 Hz
POINT 16384
FREQU 7348.62 Hz
SCANS 4
ACQTM 2.2295 sec
PD 5.0000 sec
PW1 4.99 usec
IRNUC 1H
CTEMP 25.4 c
SLVNT CDCL3
EXREF 7.26 ppm
BF 0.12 Hz
RGAIN 30

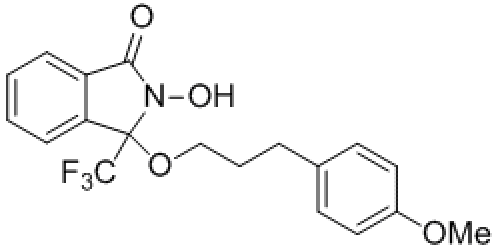

1u

4-OMe-Ph, OH

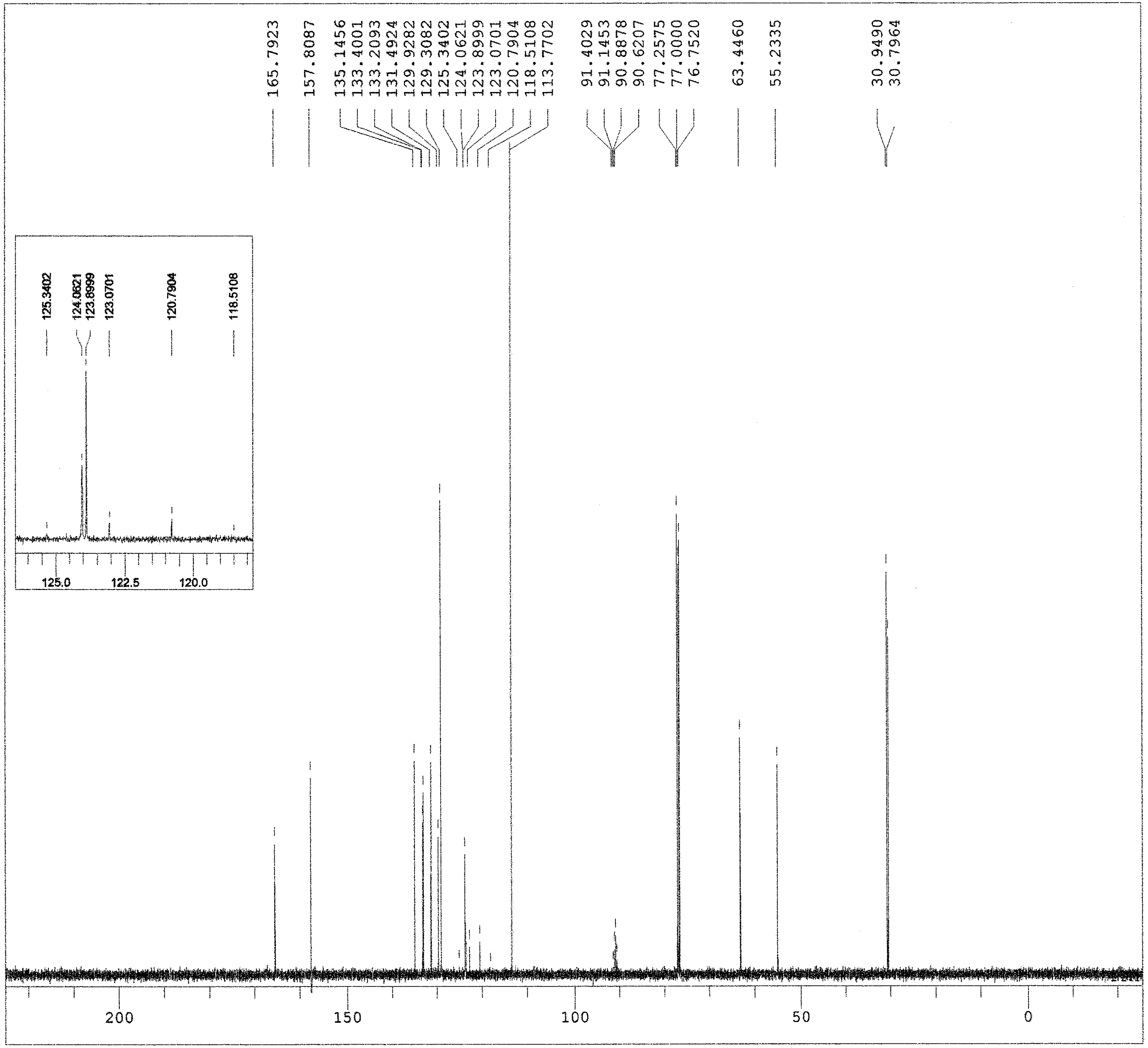

DFILE ozawa07-049_C13.jdf
COMNT 4-OMe-Ph, OH
DATIM 2015-03-20 17:28:55
OBNUC 13C
EXMOD carbon.jxp
OBFRQ 125.77 MHz
OBSET 7.87 KHz
OBFIN 4.21 Hz
POINT 32767
FREQU 39308.18 Hz
SCANS 256
ACQTM 0.8336 sec
PD 2.0000 sec
PW1 3.40 usec
IRNUC 1H
CTEMP 26.7 c
SLVNT CDCL3
EXREF 77.00 ppm
BF 0.12 Hz
RGAIN 60

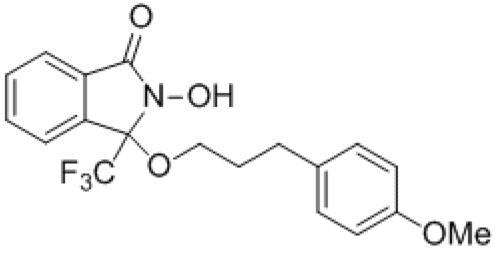

1u

4-OMe-Ph, OH

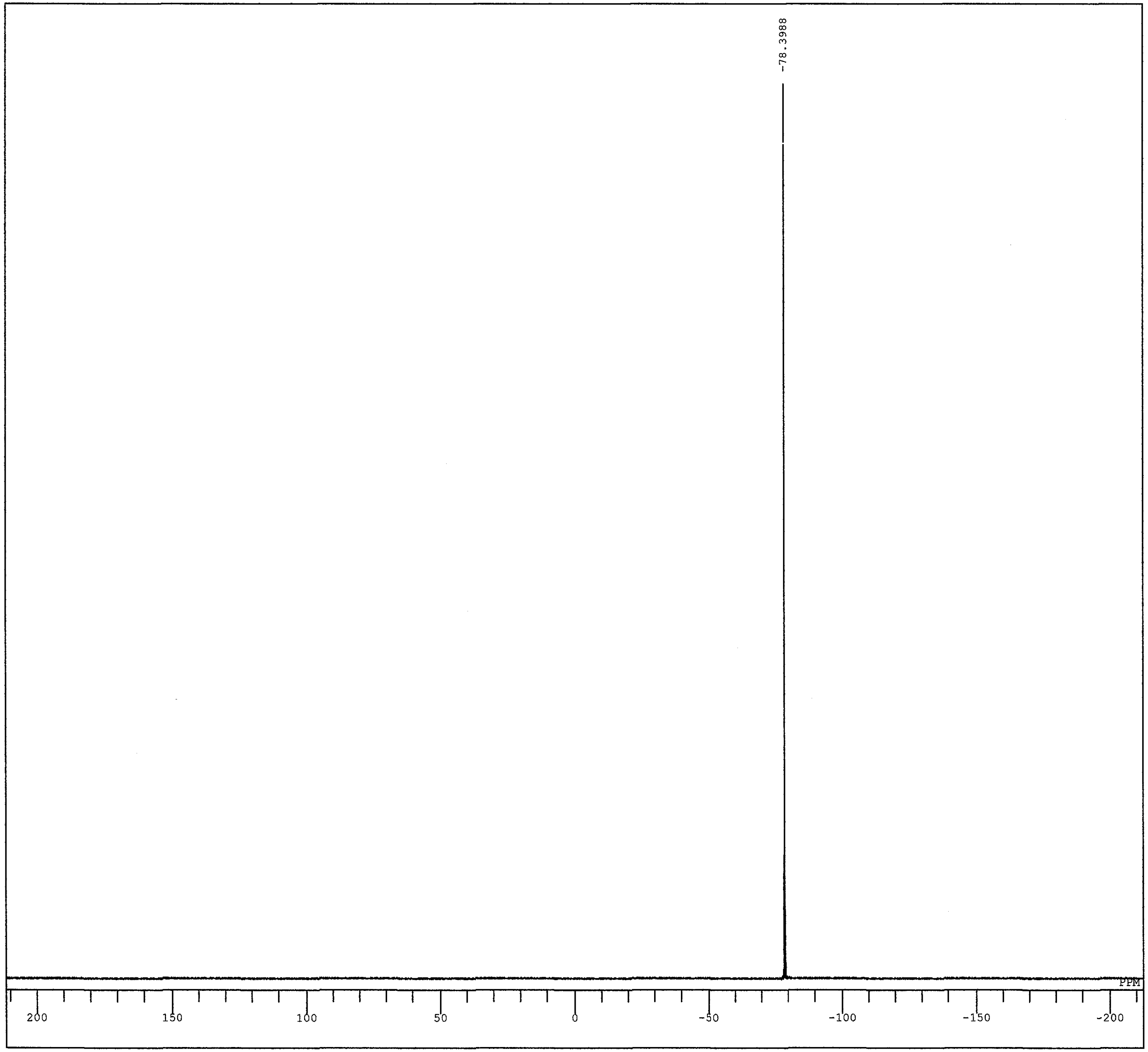

DFILE ozawa07-049_19F.jdf
COMNT 4-OMe-Ph, OH
DATIM 20-03-2015 17:30:46
OBNUC 19F
EXMOD proton.jxp
OBFRQ 368.64 MHz
OBSET 7.63 KHz
OBFIN 2.85 Hz
POINT 16384
FREQU 195312.50 Hz
SCANS 4
ACQTM 0.0839 sec
PD 5.0000 sec
PW1 3.90 usec
IRNUC 19F
CTEMP 25.4 c
SLVNT CDCL3
EXREF -164.90 ppm
BF 0.12 Hz
RGAIN 50

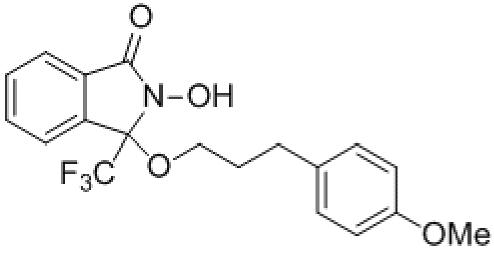

1u

4-OMe-Ph, [O]

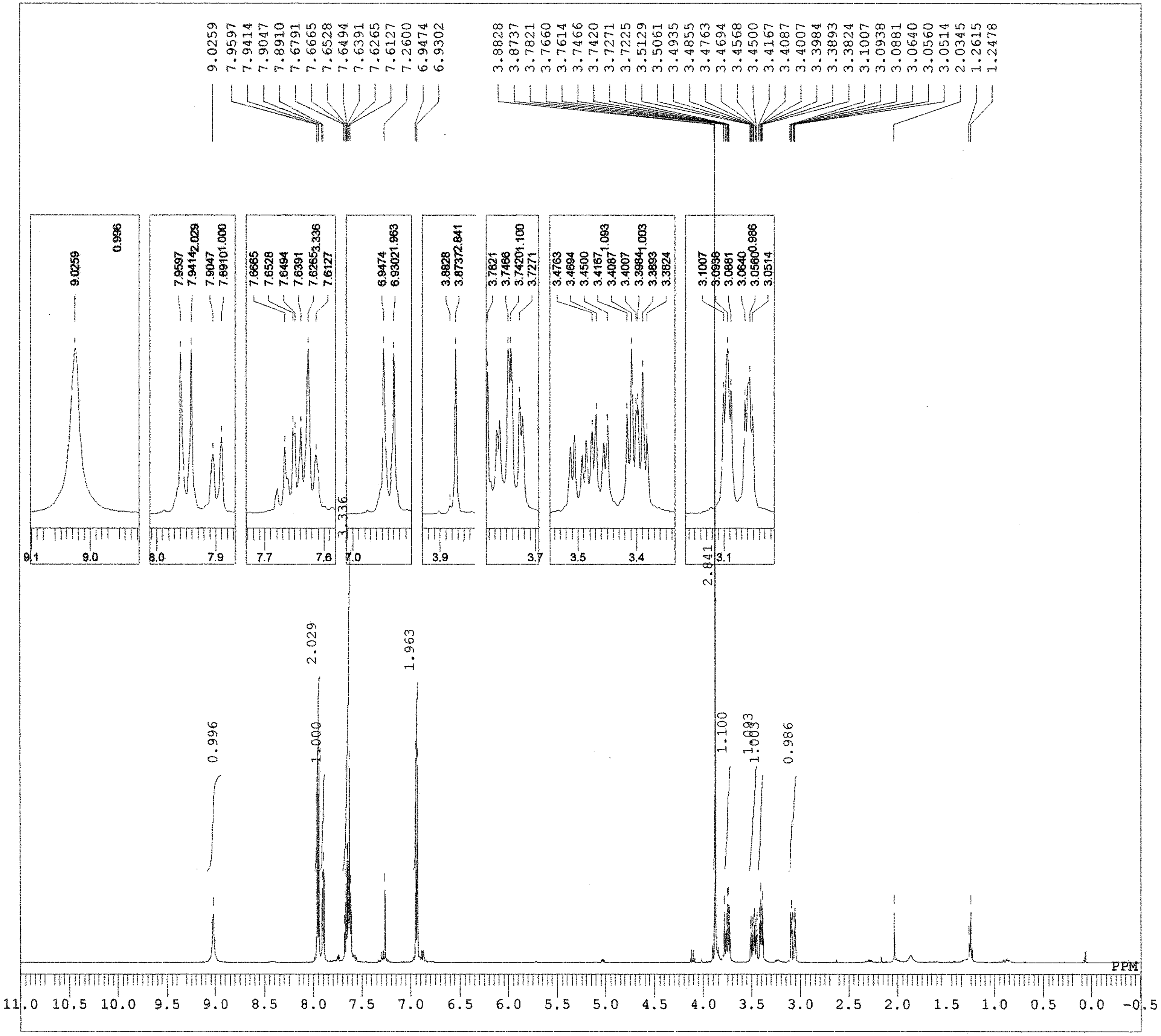

DFILE	ozawa07-053_1H-1-1.jdf
COMNT	4-OMe-Ph, [O]
DATIM	2015-03-23 16:44:10
OBNUC	1H
EXMOD	proton.jxp
OBFRQ	500.16 MHz
OBSET	2.41 KHz
OBFIN	6.01 Hz
POINT	16384
FREQU	9384.38 Hz
SCANS	4
ACQTM	1.7459 sec
PD	5.0000 sec
PW1	5.55 usec
IRNUC	1H
CTEMP	26.2 c
SLVNT	CDCL3
EXREF	7.26 ppm
BF	0.12 Hz
RGAIN	30

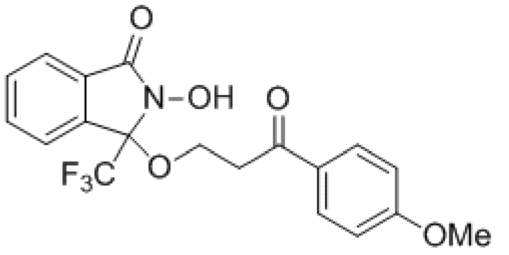

2u

4-OMe-Ph, [O]

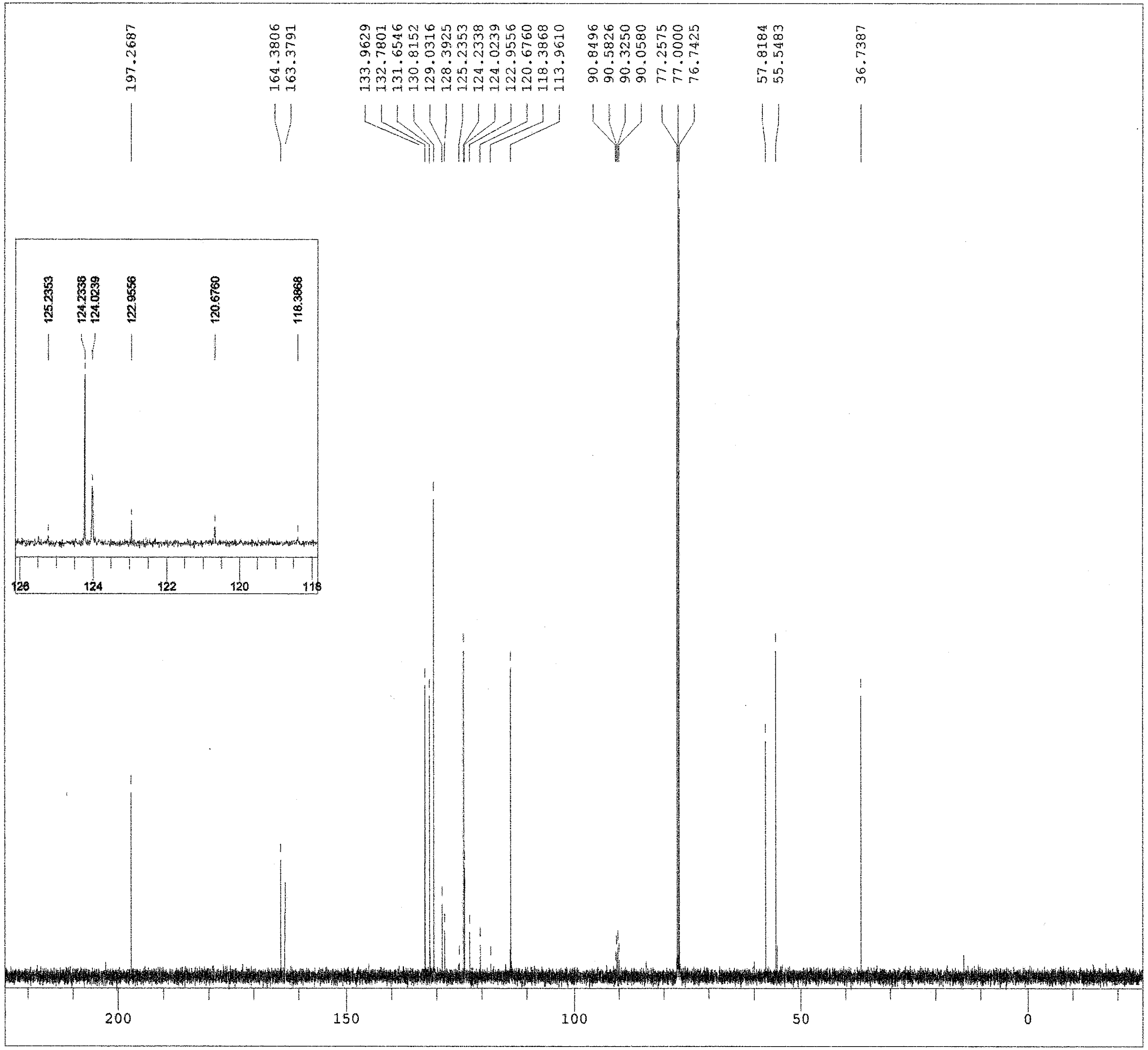

DFILE ozawa07-053_13C.jdf
COMNT 4-OMe-Ph, [O]
DATIM 2015-03-23 16:45:12
OBNUC 13C
EXMOD carbon.jxp
OBFRQ 125.77 MHz
OBSET 7.87 KHz
OBFIN 4.21 Hz
POINT 32767
FREQU 39308.18 Hz
SCANS 500
ACQTM 0.8336 sec
PD 2.0000 sec
PW1 3.40 usec
IRNUC 1H
CTEMP 26.8 c
SLVNT CDCL3
EXREF 77.00 ppm
BF 0.12 Hz
RGAIN 60

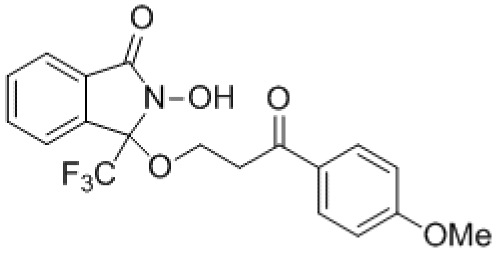

2u

4-OMe-Ph, [O]

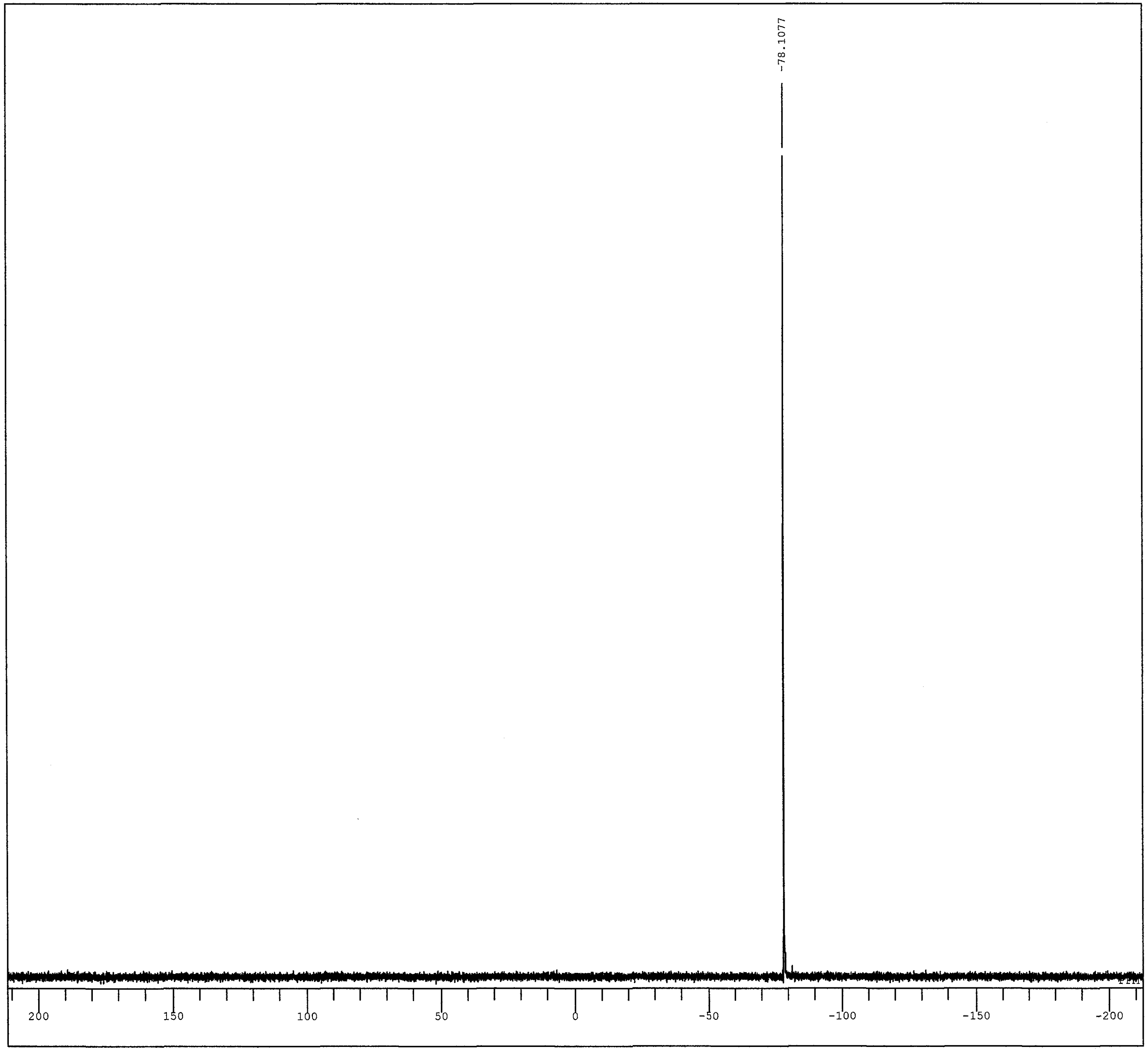

DFILE	ozawa07-050_19F.jdf
COMNT	4-OMe-Ph, [O]
DATIM	20-03-2015 17:27:38
OBNUC	19F
EXMOD	proton.jxp
OBFRQ	368.64 MHz
OBSET	7.63 KHz
OBFIN	2.85 Hz
POINT	16384
FREQU	195312.50 Hz
SCANS	4
ACQTM	0.0839 sec
PD	5.0000 sec
PW1	3.90 usec
IRNUC	19F
CTEMP	25.4 c
SLVNT	CDCL3
EXREF	-164.90 ppm
BF	0.12 Hz
RGAIN	50

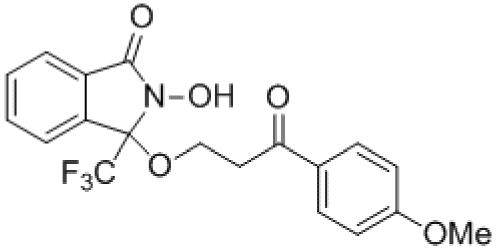

2u

4-BrPh-PrOH, PMB

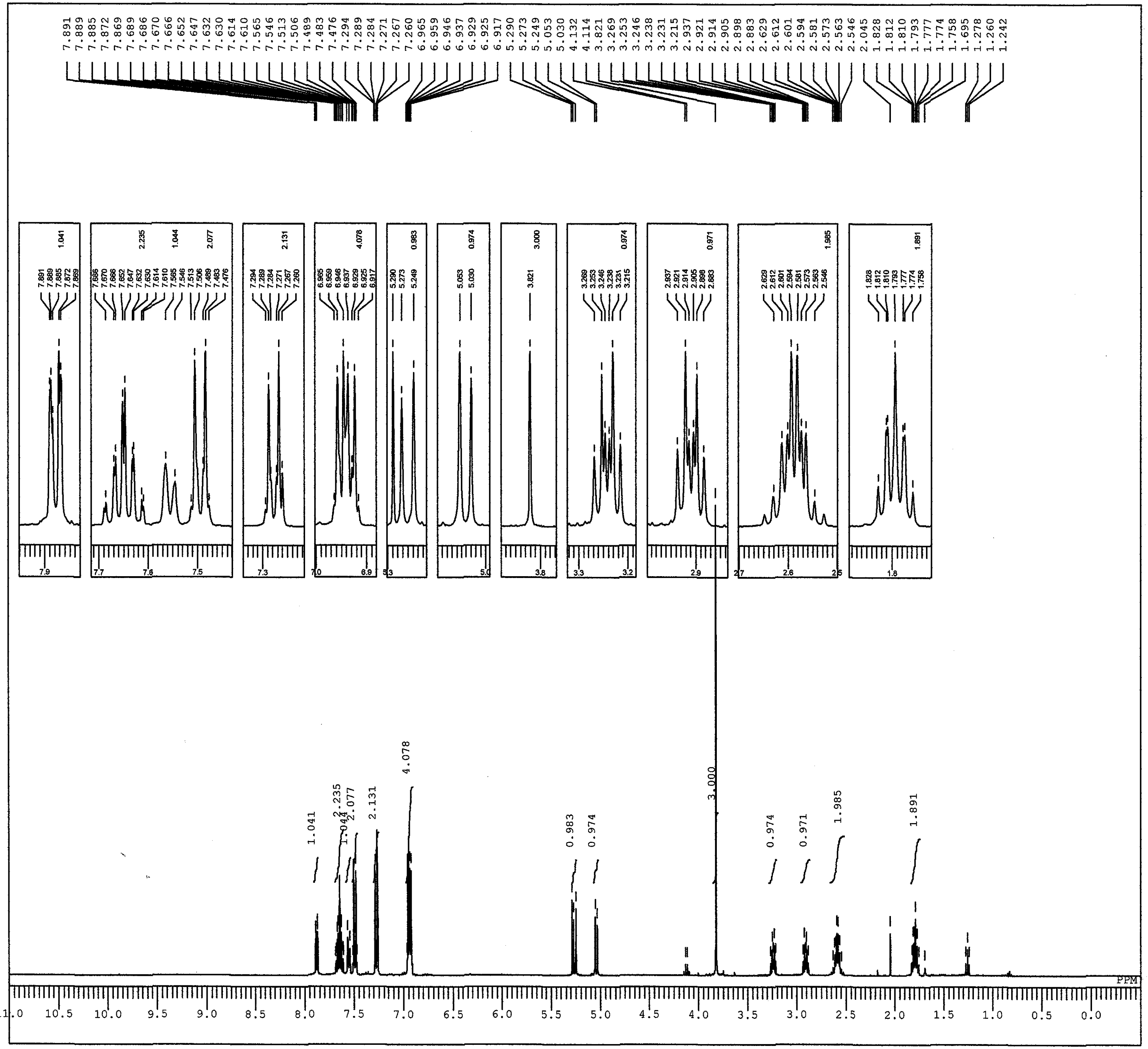

DFILE ozawa06-116_1H.jdf
COMNT 4-BrPh-PrOH, PMB
DATIM 24-12-2014 13:35:46
OBNUC 1H
EXMOD proton.jxp
OBFRQ 391.78 MHz
OBSET 8.51 KHz
OBFIN 3.34 Hz
POINT 16384
FREQU 7348.62 Hz
SCANS 8
ACQTM 2.2295 sec
PD 5.0000 sec
PW1 5.07 usec
IRNUC 1H
CTEMP 22.2 c
SLVNT CDCL3
EXREF 7.26 ppm
BF 0.12 Hz
RGAIN 30

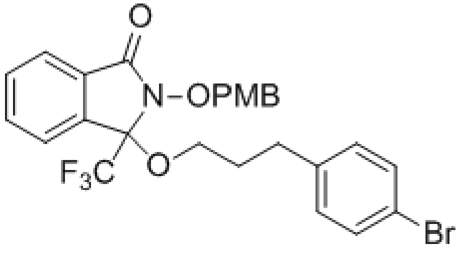

S4'v

4-BrPh-PrOH, PMB

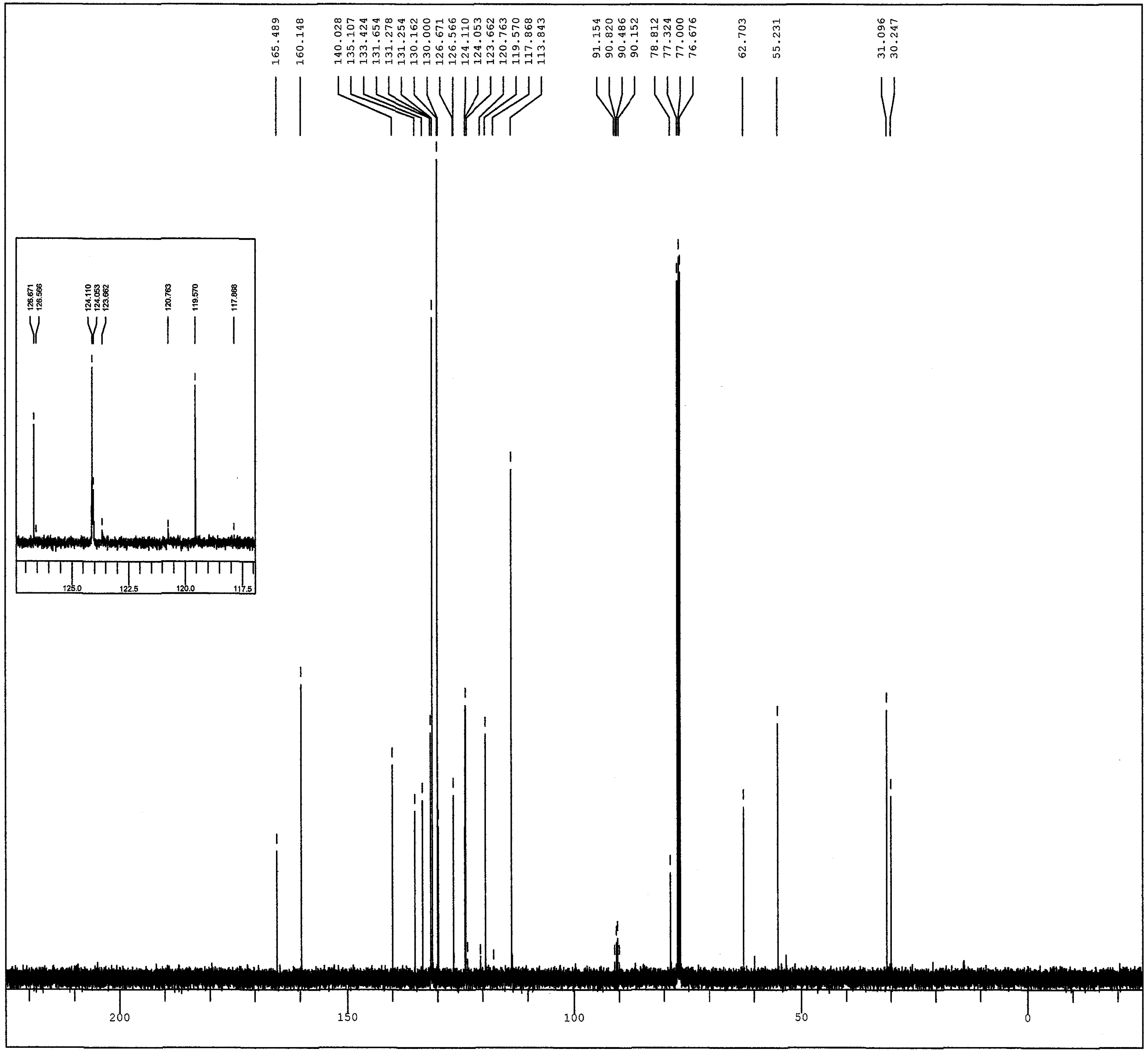

DFILE ozawa06-116_13C.jdf
COMNT 4-BrPh-PrOH, PMB
DATIM 24-12-2014 13:37:22
OBNUC 13C
EXMOD carbon.jxp
OBFRQ 98.52 MHz
OBSET 4.64 KHz
OBFIN 8.74 Hz
POINT 65535
FREQU 30788.18 Hz
SCANS 340
ACQTM 2.1286 sec
PD 2.0000 sec
PW1 3.07 usec
IRNUC 1H
CTEMP 22.3 c
SLVNT CDCL3
EXREF 77.00 ppm
BF 0.12 Hz
RGAIN 60

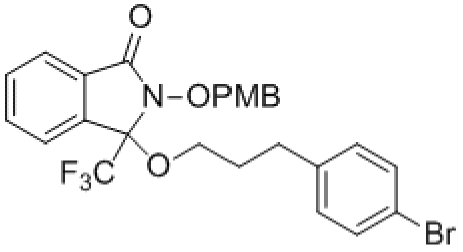

S4'v

4-BrPh-PrOH, PMB

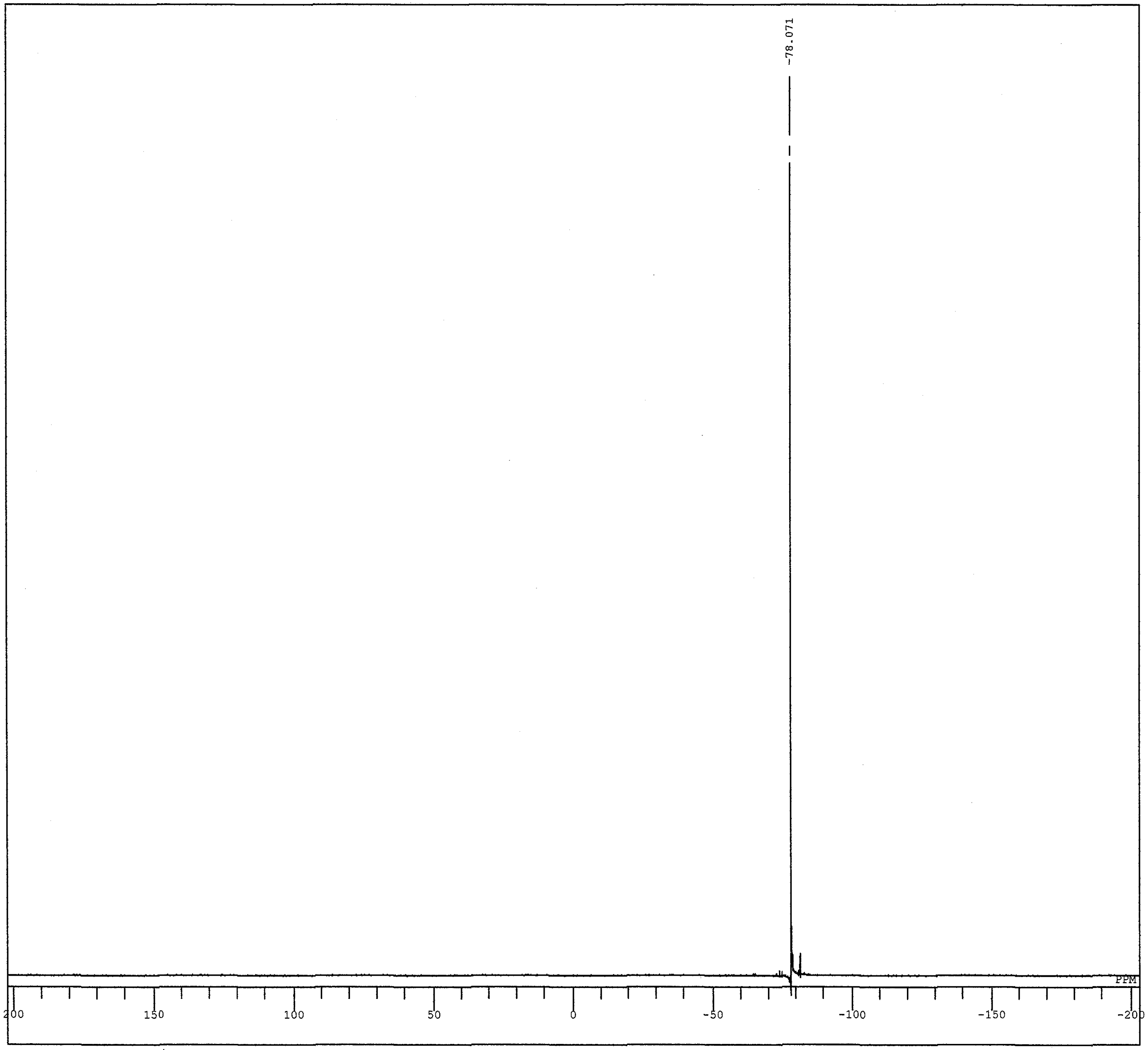

DFILE ozawa06-116_19F.jdf
COMNT 4-BrPh-PrOH, PMB
DATIM 24-12-2014 13:33:22
OBNUC 19F
EXMOD proton.jxp
OBFRQ 368.64 MHz
OBSET 7.63 KHz
OBFIN 2.85 Hz
POINT 16384
FREQU 186567.17 Hz
SCANS 8
ACQTM 0.0878 sec
PD 5.0000 sec
PW1 3.90 usec
IRNUC 19F
CTEMP 22.2 c
SLVNT CDCL3
EXREF -164.90 ppm
BF 0.12 Hz
RGAIN 48

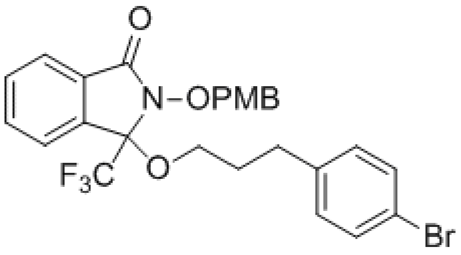

S4'v

4-Br-Ph, OH

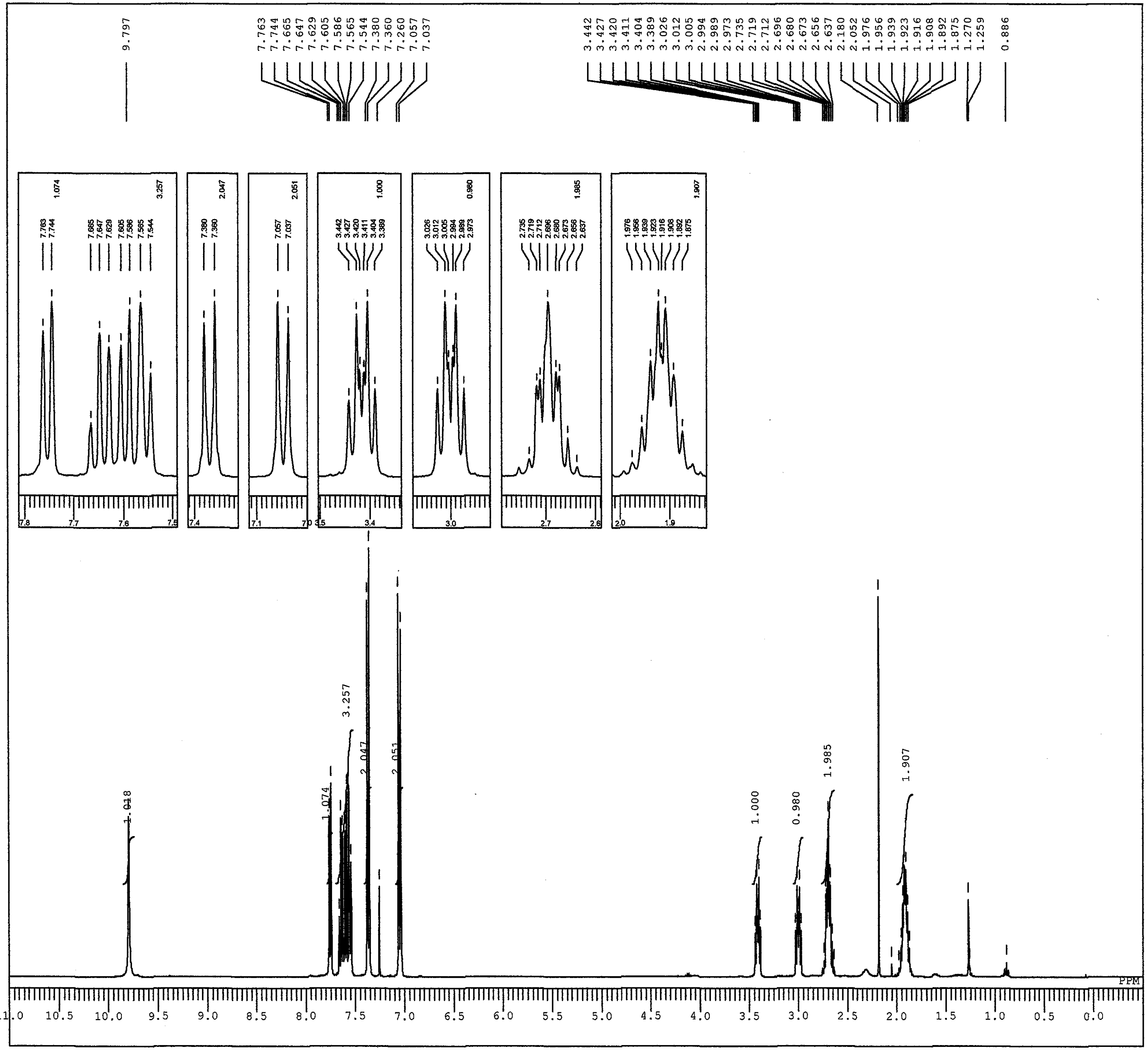

DFILE ozawa06-117_1H.jdf
COMNT 4-Br-Ph, OH
DATIM 30-12-2014 10:15:11
OBNUC 1H
EXMOD proton.jxp
OBFRQ 391.78 MHz
OBSET 8.51 KHz
OBFIN 3.34 Hz
POINT 16384
FREQU 7348.62 Hz
SCANS 8
ACQTM 2.2295 sec
PD 5.0000 sec
PW1 5.07 usec
IRNUC 1H
CTEMP 21.5 c
SLVNT CDCL3
EXREF 7.26 ppm
BF 0.12 Hz
RGAIN 32

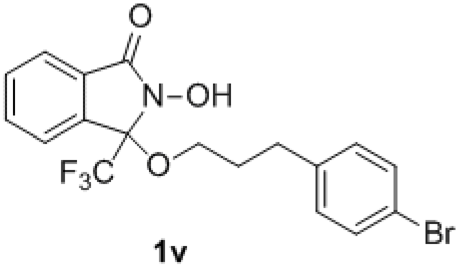

1v

4-Br-Ph, OH

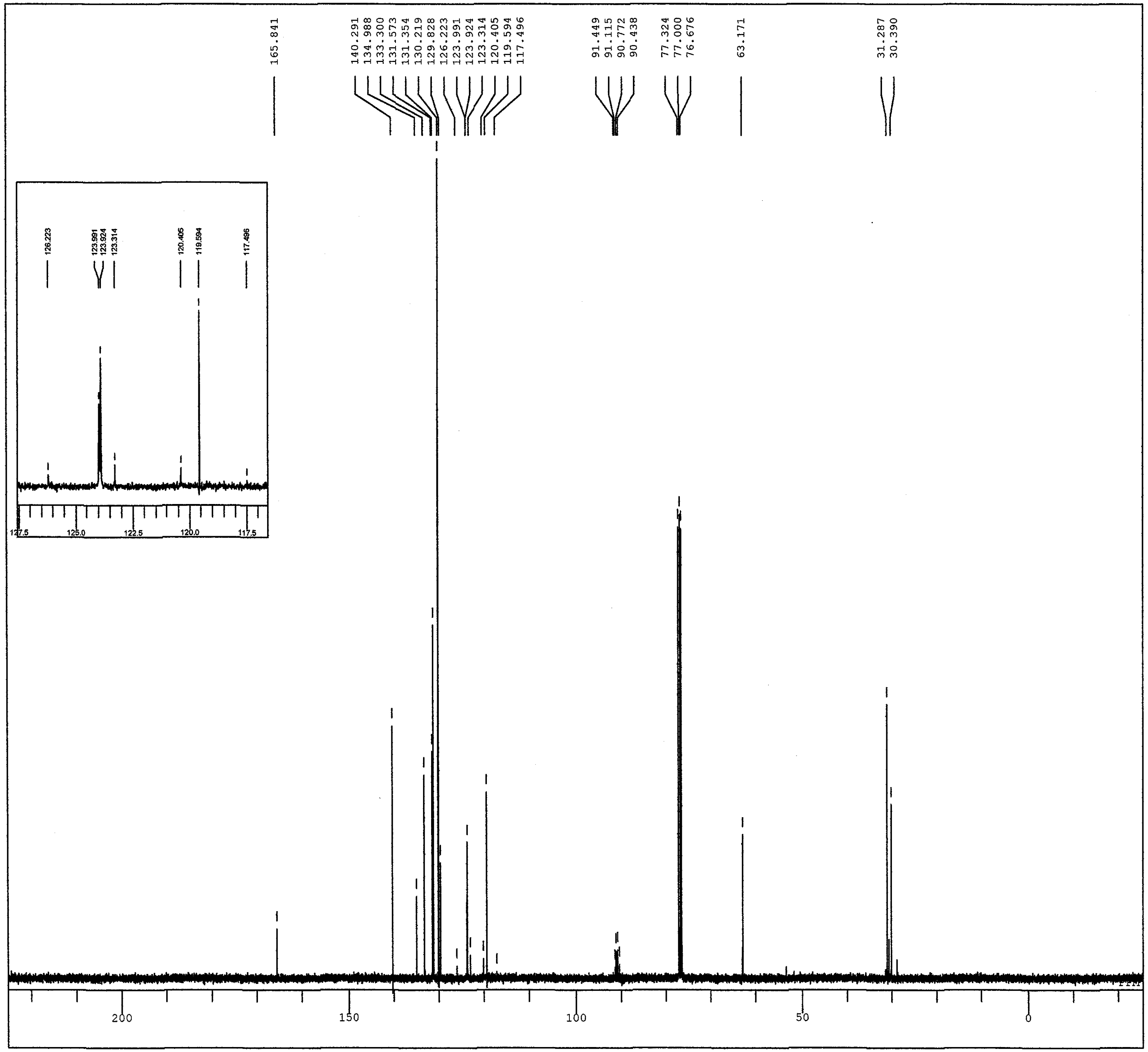

DFILE	ozawa06-117_13C.jdf
COMNT	4-Br-Ph, OH
DATIM	30-12-2014 10:18:35
OBNUC	13C
EXMOD	carbon.jxp
OBFRQ	98.52 MHz
OBSET	4.64 KHz
OBFIN	8.74 Hz
POINT	32767
FREQU	30788.18 Hz
SCANS	460
ACQTM	1.0643 sec
PD	3.0000 sec
PW1	3.07 usec
IRNUC	1H
CTEMP	21.9 c
SLVNT	CDCL3
EXREF	77.00 ppm
BF	0.12 Hz
RGAIN	60

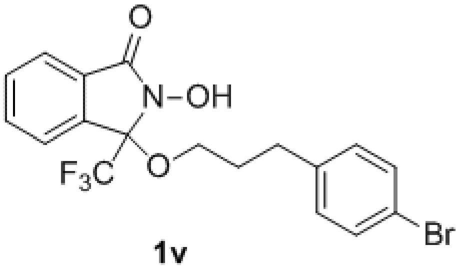

1v

4-Br-Ph, OH

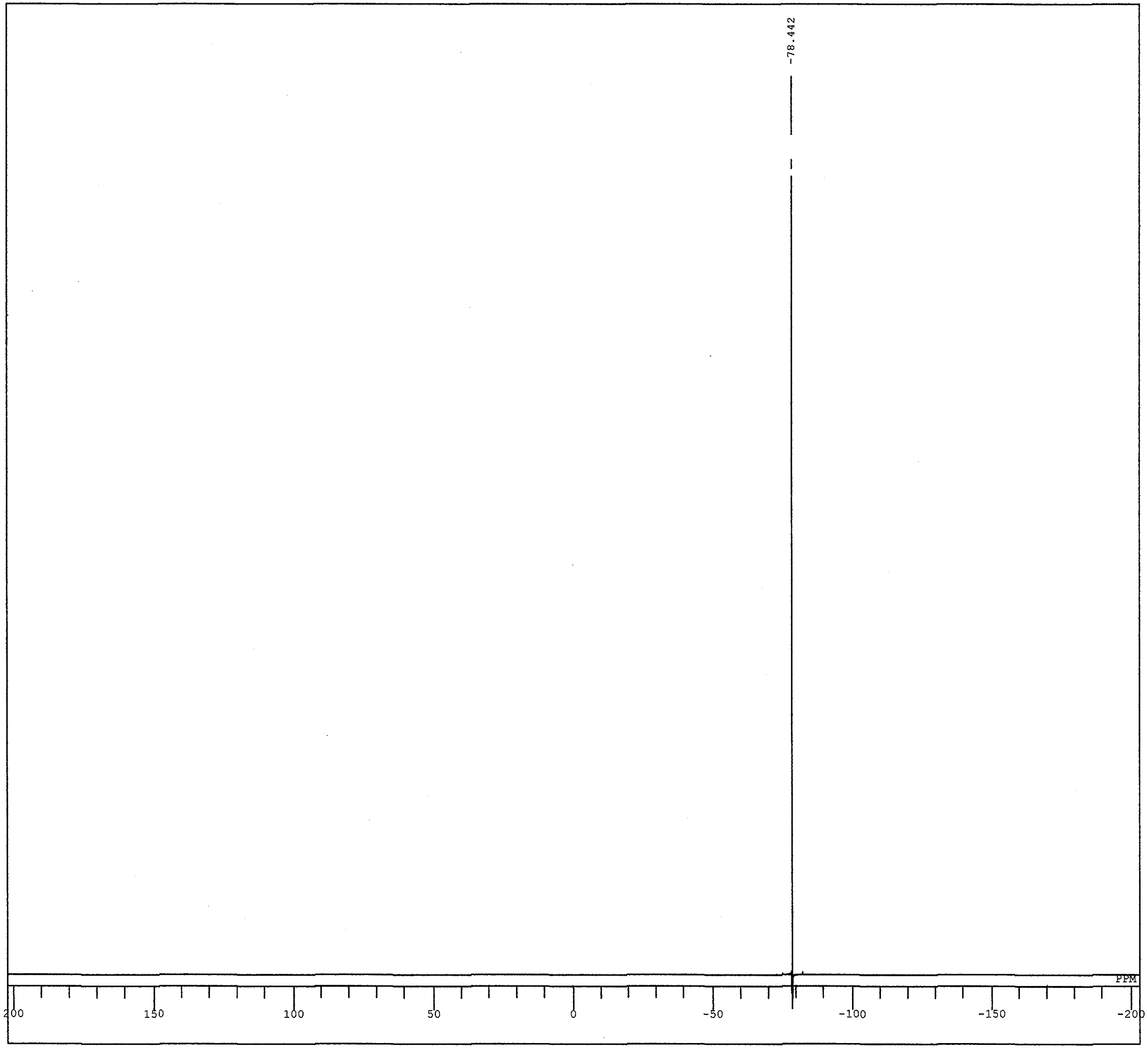

DFILE ozawa06-117_19F.jdf
COMNT 4-Br-Ph, OH
DATIM 30-12-2014 10:12:31
OBNUC 19F
EXMOD proton.jxp
OBFRQ 368.64 MHz
OBSET 7.63 KHz
OBFIN 2.85 Hz
POINT 16384
FREQU 186567.17 Hz
SCANS 8
ACQTM 0.0878 sec
PD 5.0000 sec
PW1 3.90 usec
IRNUC 19F
CTEMP 21.5 c
SLVNT CDCL3
EXREF -164.90 ppm
BF 0.12 Hz
RGAIN 50

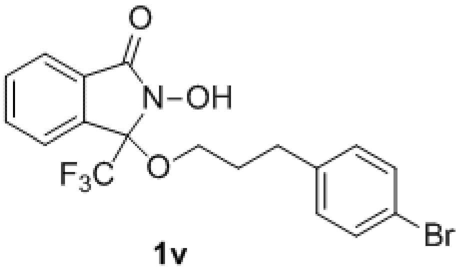

1v

PTLC 4-Br-Ph, [O]

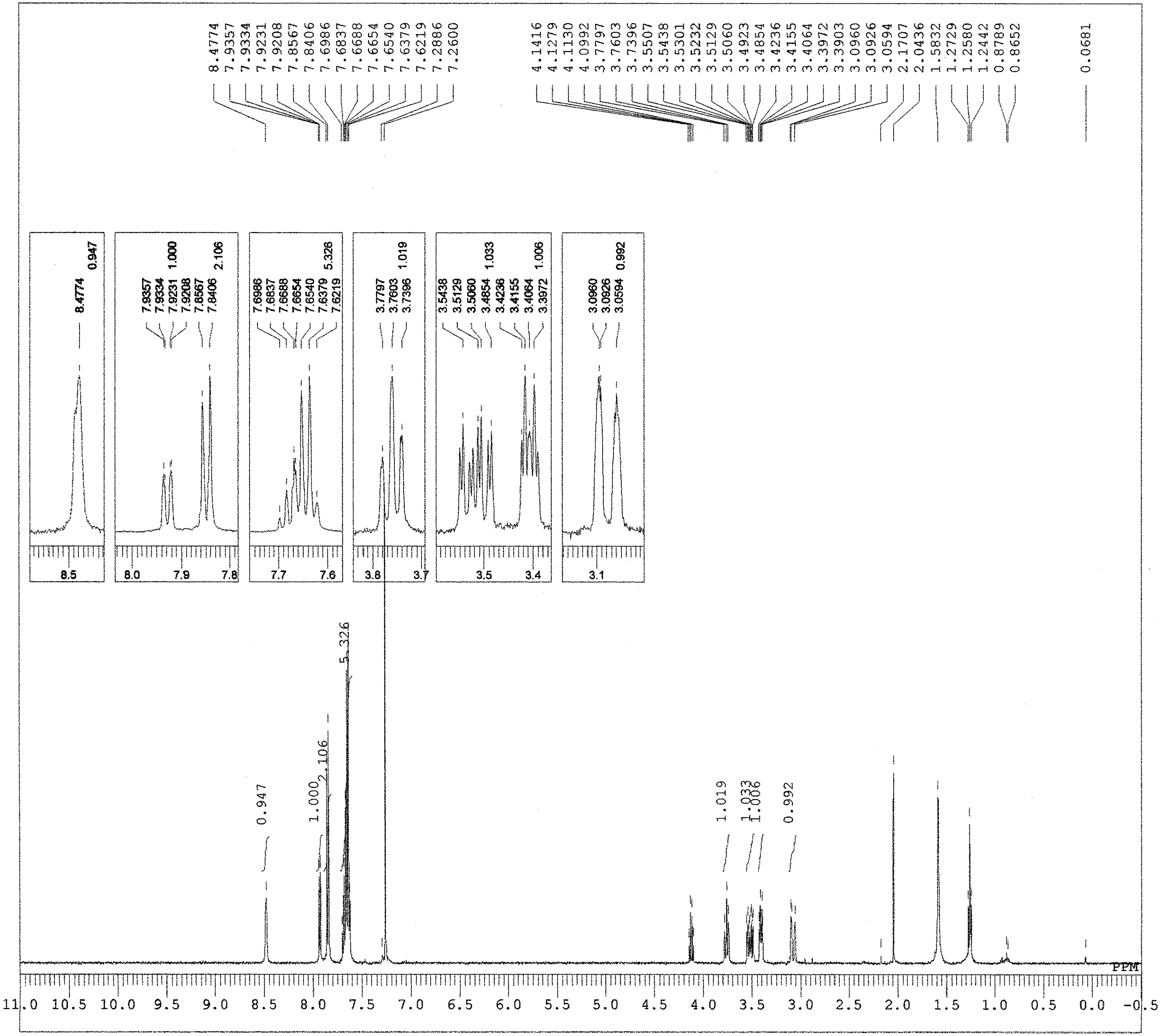

DFILE	ozawa06-118_take2.als
COMNT	PTLC 4-Br-Ph, [O]
DATIM	2014-12-27 08:23:00
OBNUC	1H
EXMOD	proton.jxp
OBFRQ	500.16 MHz
OBSET	2.41 KHz
OBFIN	6.01 Hz
POINT	13107
FREQU	7507.51 Hz
SCANS	4
ACQTM	1.7459 sec
PD	5.0000 sec
PW1	5.55 usec
IRNUC	1H
CTEMP	24.0 c
SLVNT	CDCL3
EXREF	7.26 ppm
BF	0.12 Hz
RGAIN	30

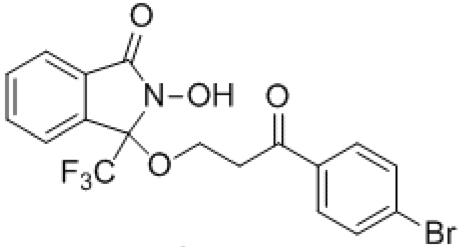

2v

4-Br-Ph, [O]

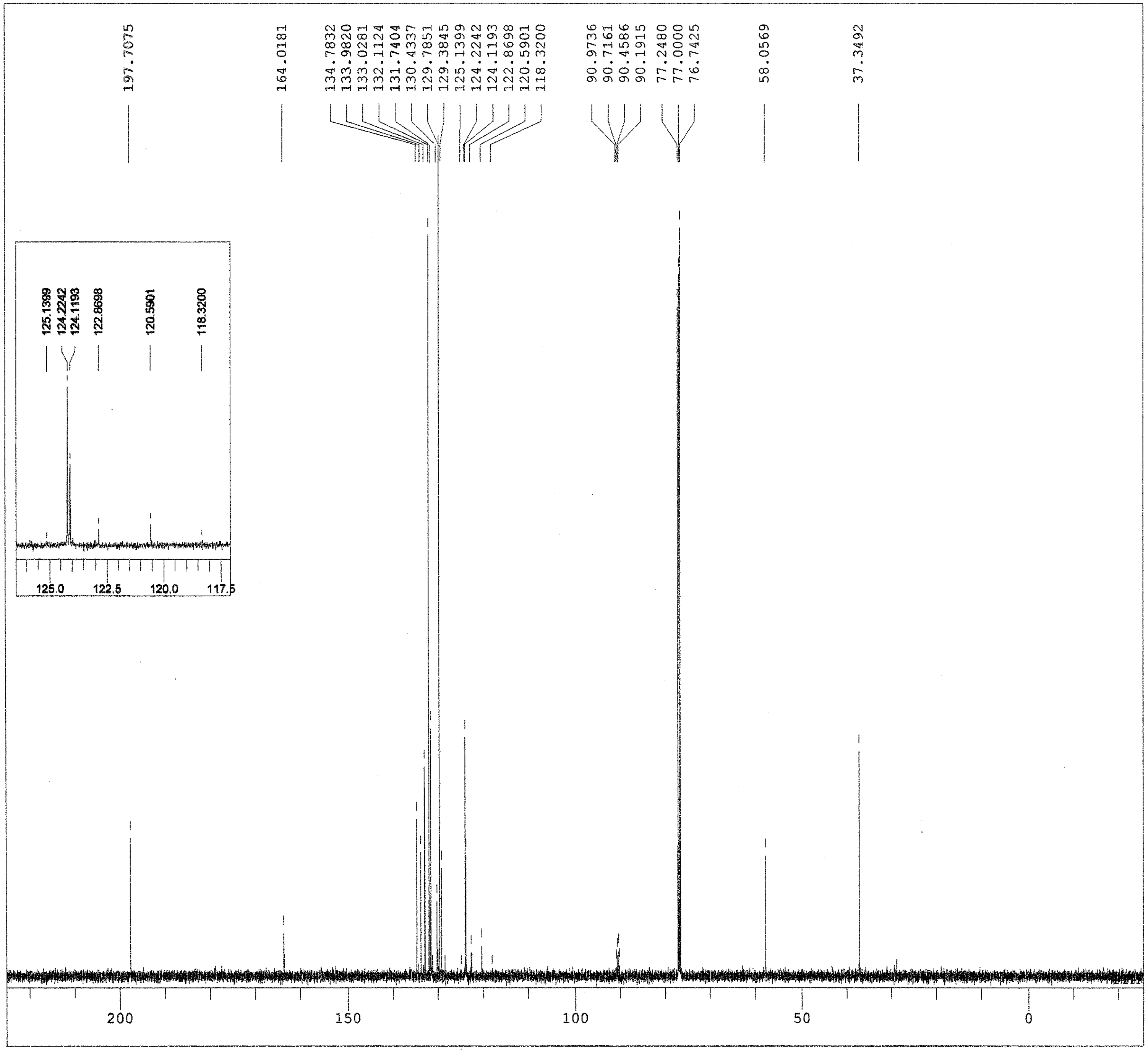

DFILE ozawa06-118_13C.jdf
COMNT 4-Br-Ph, [O]
DATIM 2015-01-05 10:29:28
OBNUC 13C
EXMOD carbon.jxp
OBFRQ 125.77 MHz
OBSET 7.87 KHz
OBFIN 4.21 Hz
POINT 32767
FREQU 39308.18 Hz
SCANS 640
ACQTM 0.8336 sec
PD 2.0000 sec
PW1 3.40 usec
IRNUC 1H
CTEMP 24.2 c
SLVNT CDCL3
EXREF 77.00 ppm
BF 0.12 Hz
RGAIN 60

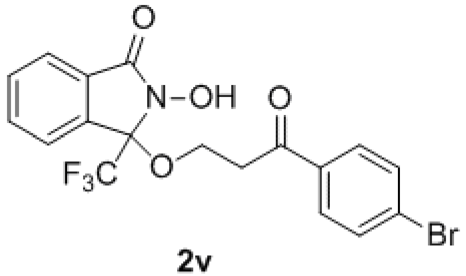

2v

4-Br-Ph, [0]

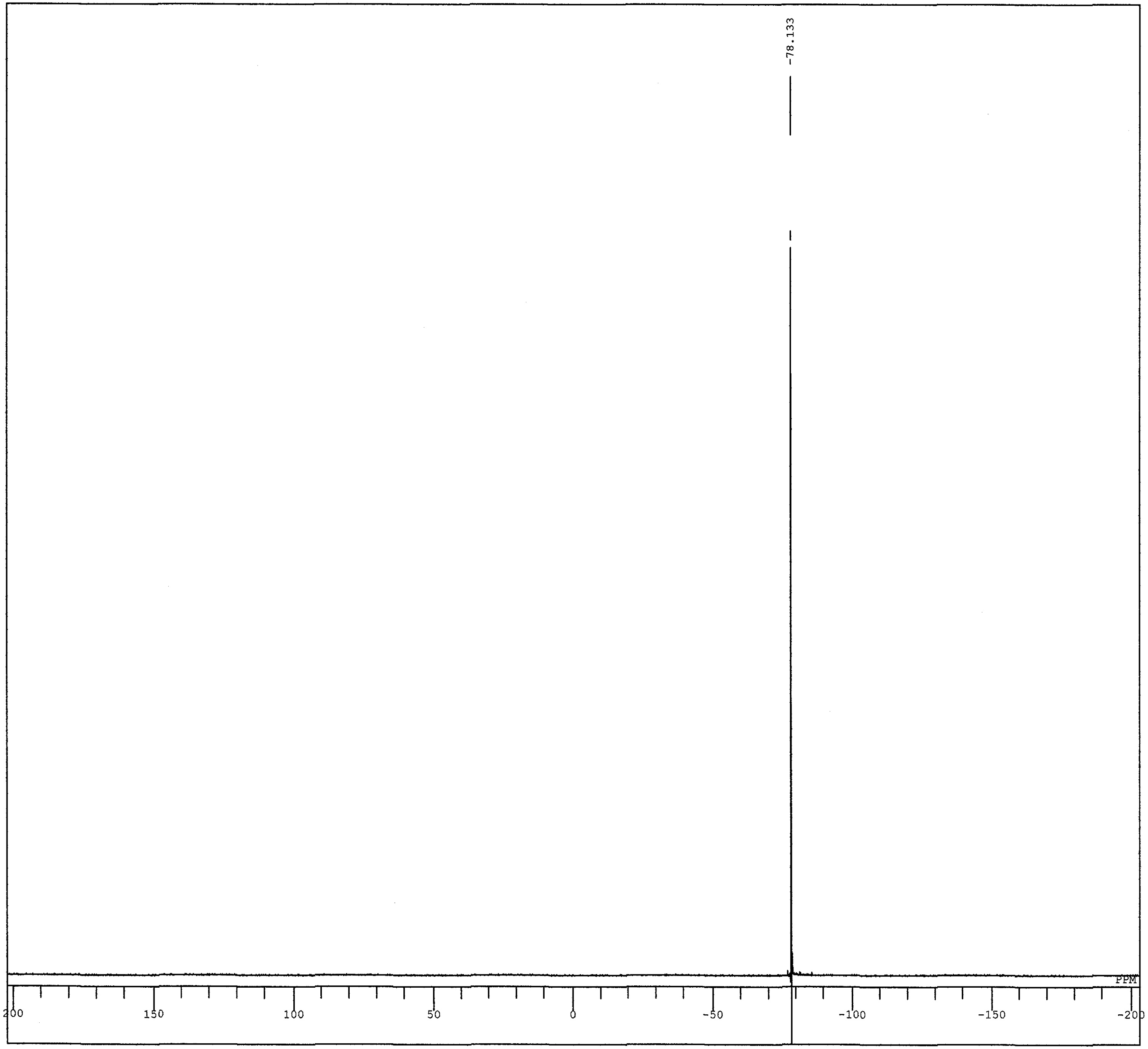

DFILE ozawa06-118_19F.jdf
COMNT 4-Br-Ph, [O]
DATIM 05-01-2015 12:24:41
OBNUC 19F
EXMOD proton.jxp
OBFRQ 368.64 MHz
OBSET 7.63 KHz
OBFIN 2.85 Hz
POINT 16384
FREQU 186567.17 Hz
SCANS 4
ACQTM 0.0878 sec
PD 5.0000 sec
PW1 3.90 usec
IRNUC 19F
CTEMP 21.5 c
SLVNT CDCL3
EXREF -164.90 ppm
BF 0.12 Hz
RGAIN 50

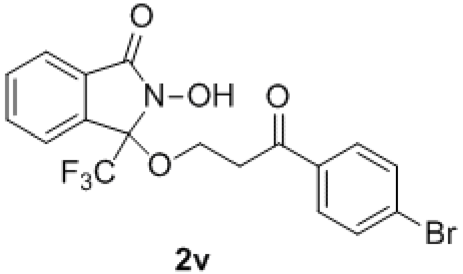

2v

Ph-Bpin, Bn

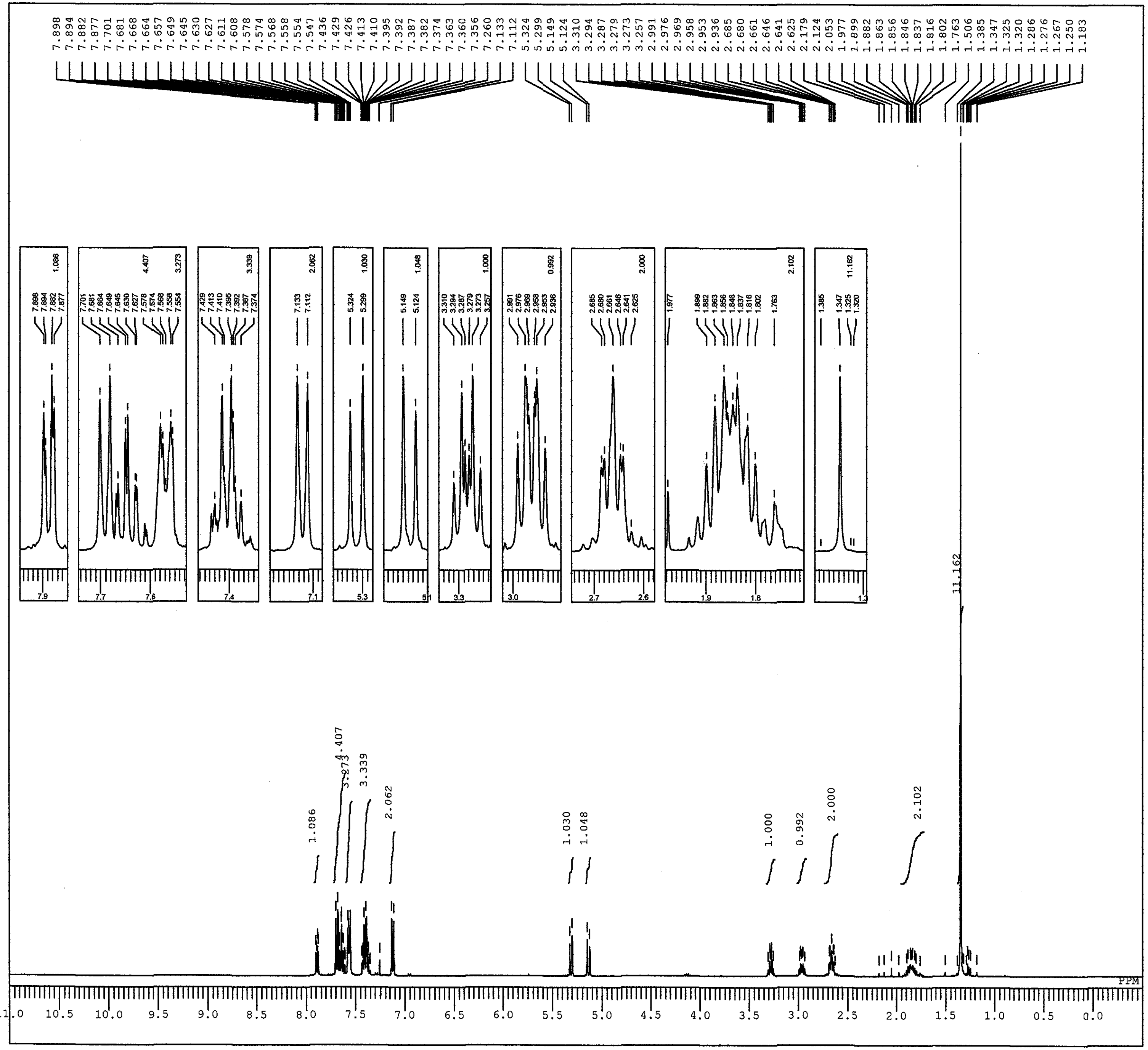

DFILE ozawa06-113_1H.jdf
COMNT Ph-Bpin, Bn
DATIM 25-12-2014 11:29:15
OBNUC 1H
EXMOD proton.jxp
OBFRQ 391.78 MHz
OBSET 8.51 KHz
OBFIN 3.34 Hz
POINT 16384
FREQU 7348.62 Hz
SCANS 8
ACQTM 2.2295 sec
PD 5.0000 sec
PW1 5.07 usec
IRNUC 1H
CTEMP 22.0 c
SLVNT CDCL3
EXREF 7.26 ppm
BF 0.12 Hz
RGAIN 26

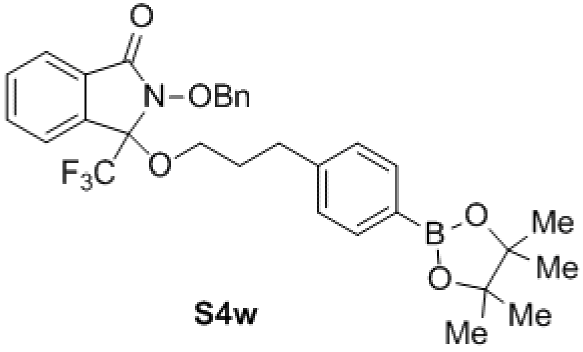

S4w

4-Bpin-Ph, Bn

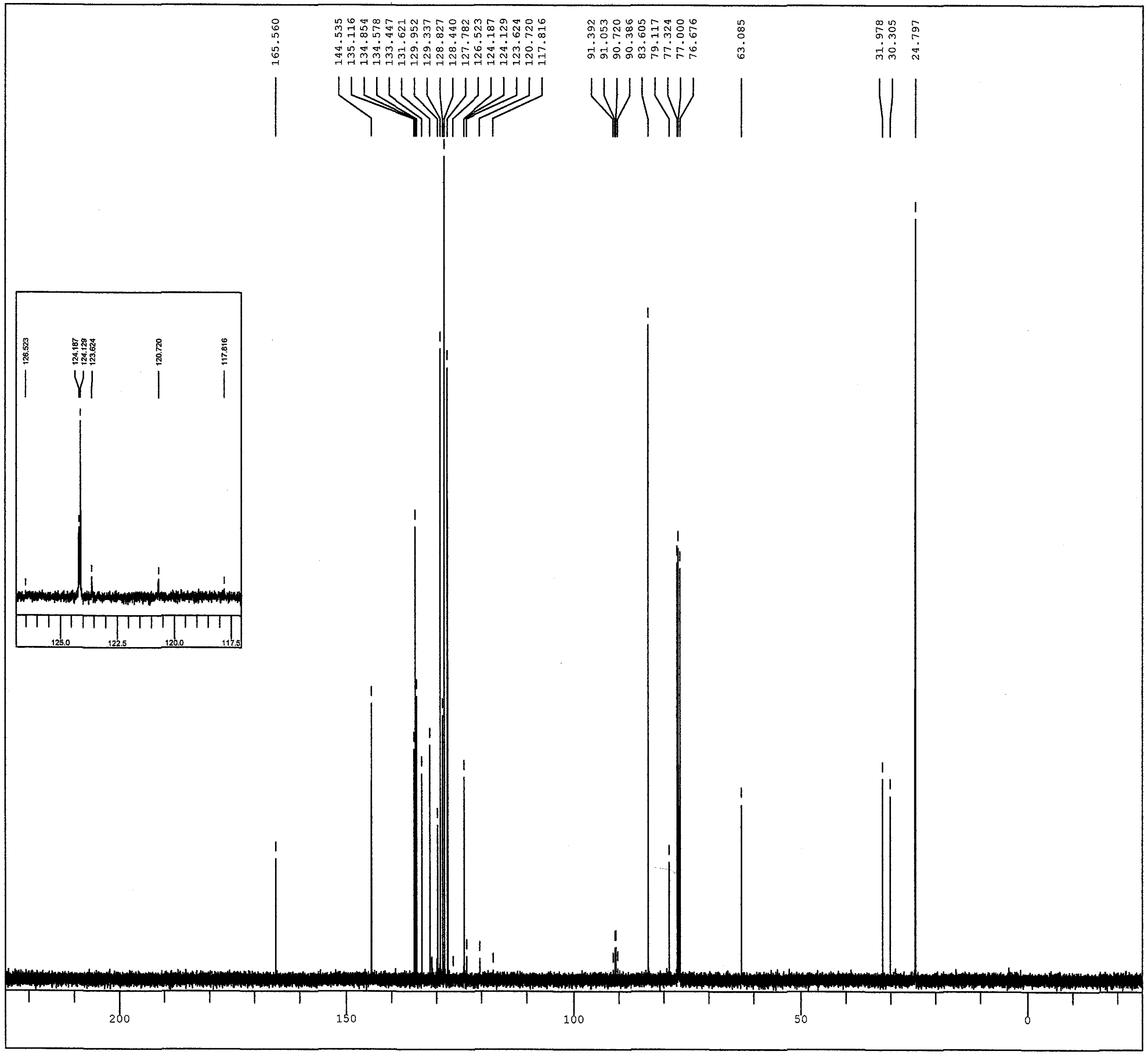

DFILE ozawa06-113_13C.jdf
COMNT 4-Bpin-Ph, Bn
DATIM 25-12-2014 11:32:48
OBNUC 13C
EXMOD carbon.jxp
OBFRQ 98.52 MHz
OBSET 4.64 KHz
OBFIN 8.74 Hz
POINT 65535
FREQU 30788.18 Hz
SCANS 180
ACQTM 2.1286 sec
PD 2.0000 sec
PW1 3.07 usec
IRNUC 1H
CTEMP 22.3 c
SLVNT CDCL3
EXREF 77.00 ppm
BF 0.12 Hz
RGAIN 60

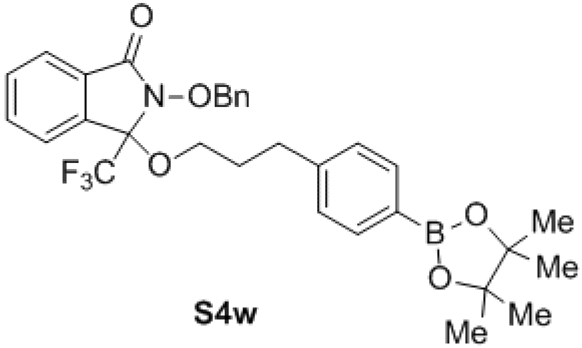

S4w

4-Bpin-Ph, Bn

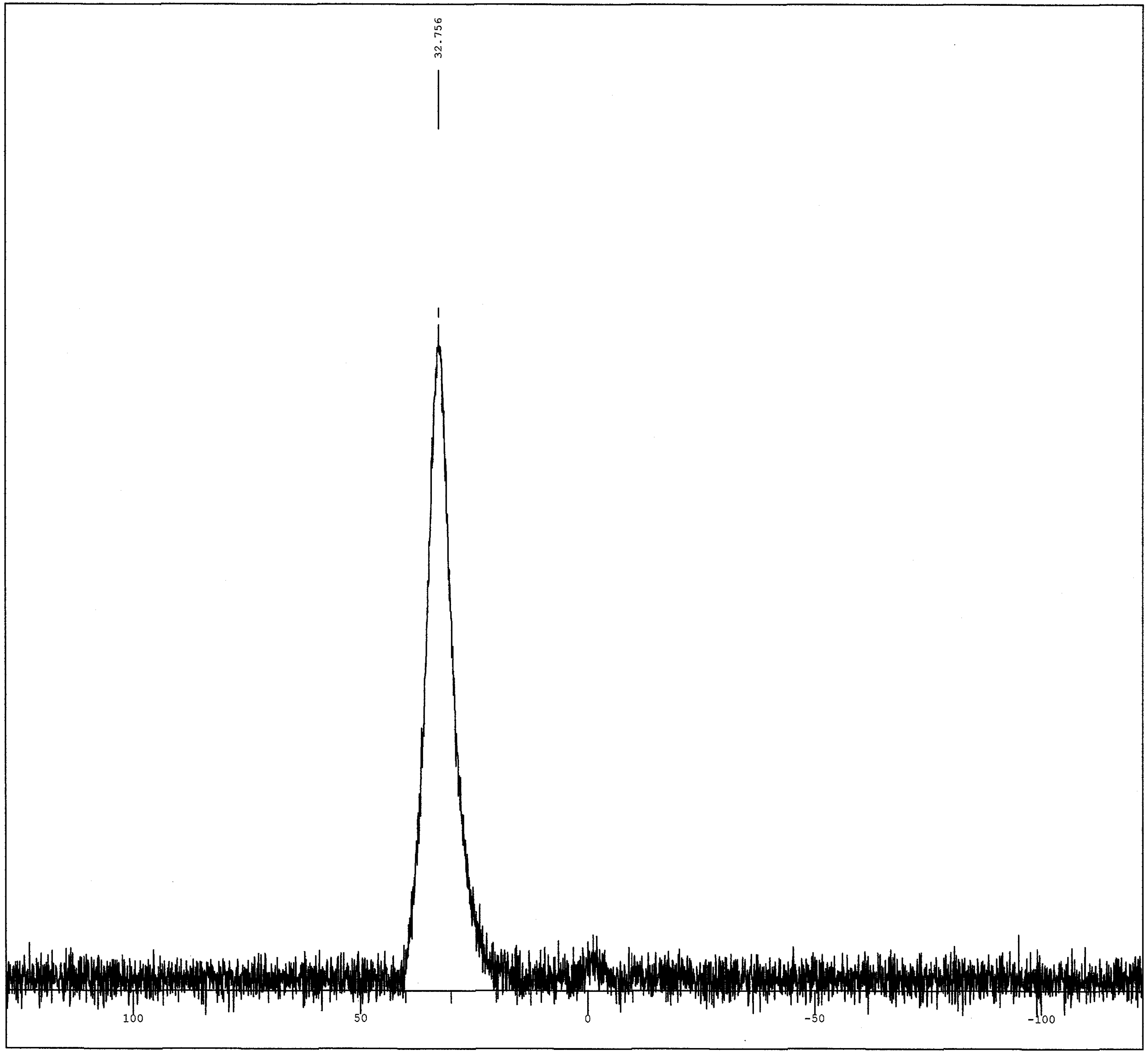

DFILE ozawa06-113_11B.jdf
COMNT 4-Bpin-Ph, Bn
DATIM 25-12-2014 11:24:53
OBNUC 11B
EXMOD carbon.jxp
OBFRQ 125.70 MHz
OBSET 0.81 KHz
OBFIN 3.25 Hz
POINT 4095
FREQU 39308.18 Hz
SCANS 200
ACQTM 0.1042 sec
PD 80.0000 sec
PW1 3.98 usec
IRNUC 1H
CTEMP 22.2 c
SLVNT CDCL3
EXREF 31.00 ppm
BF 0.12 Hz
RGAIN 44

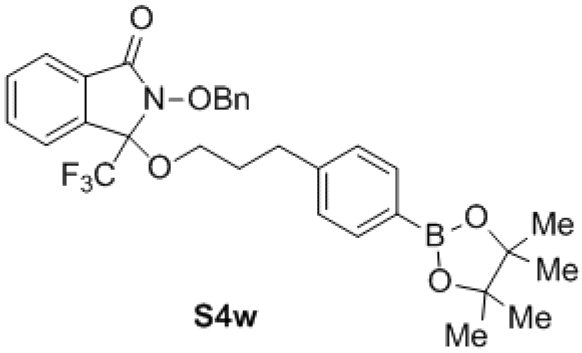

S4w

Ph-Bpin, Bn

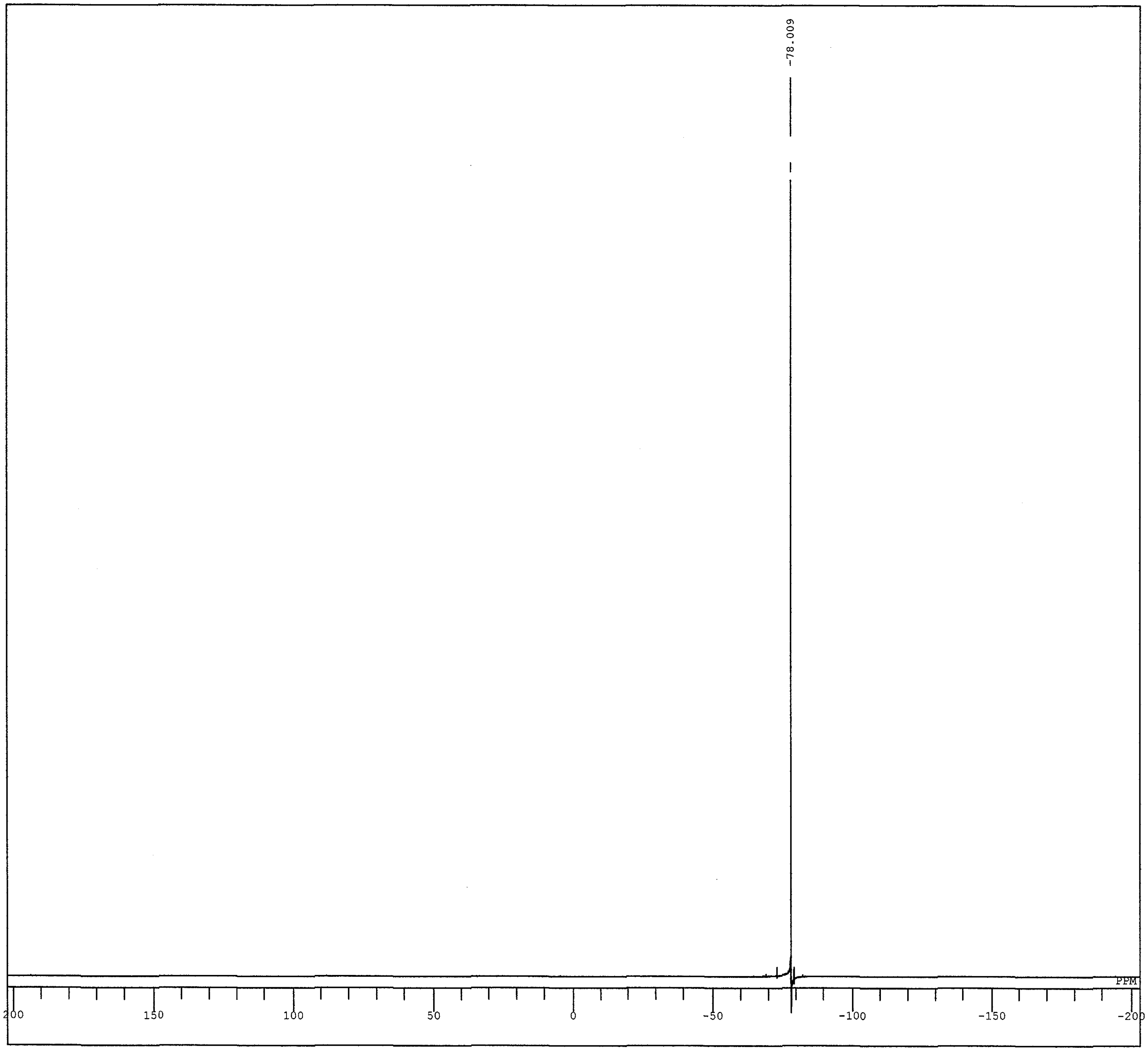

DFILE ozawa06-113_19F.jdf
COMNT Ph-Bpin, Bn
DATIM 25-12-2014 11:26:56
OBNUC 19F
EXMOD proton.jxp
OBFRQ 368.64 MHz
OBSET 7.63 KHz
OBFIN 2.85 Hz
POINT 16384
FREQU 186567.17 Hz
SCANS 4
ACQTM 0.0878 sec
PD 5.0000 sec
PW1 3.90 usec
IRNUC 19F
CTEMP 22.1 c
SLVNT CDCL3
EXREF -164.90 ppm
BF 0.12 Hz
RGAIN 48

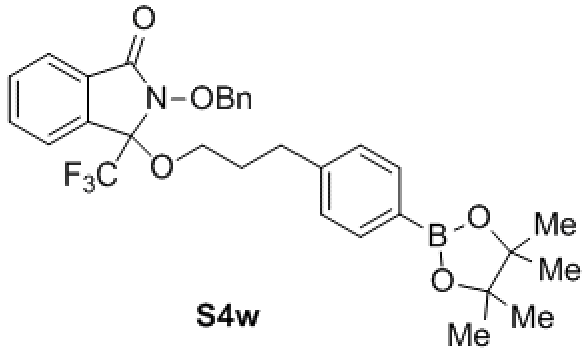

S4w

4-Bpin-Ph, OH

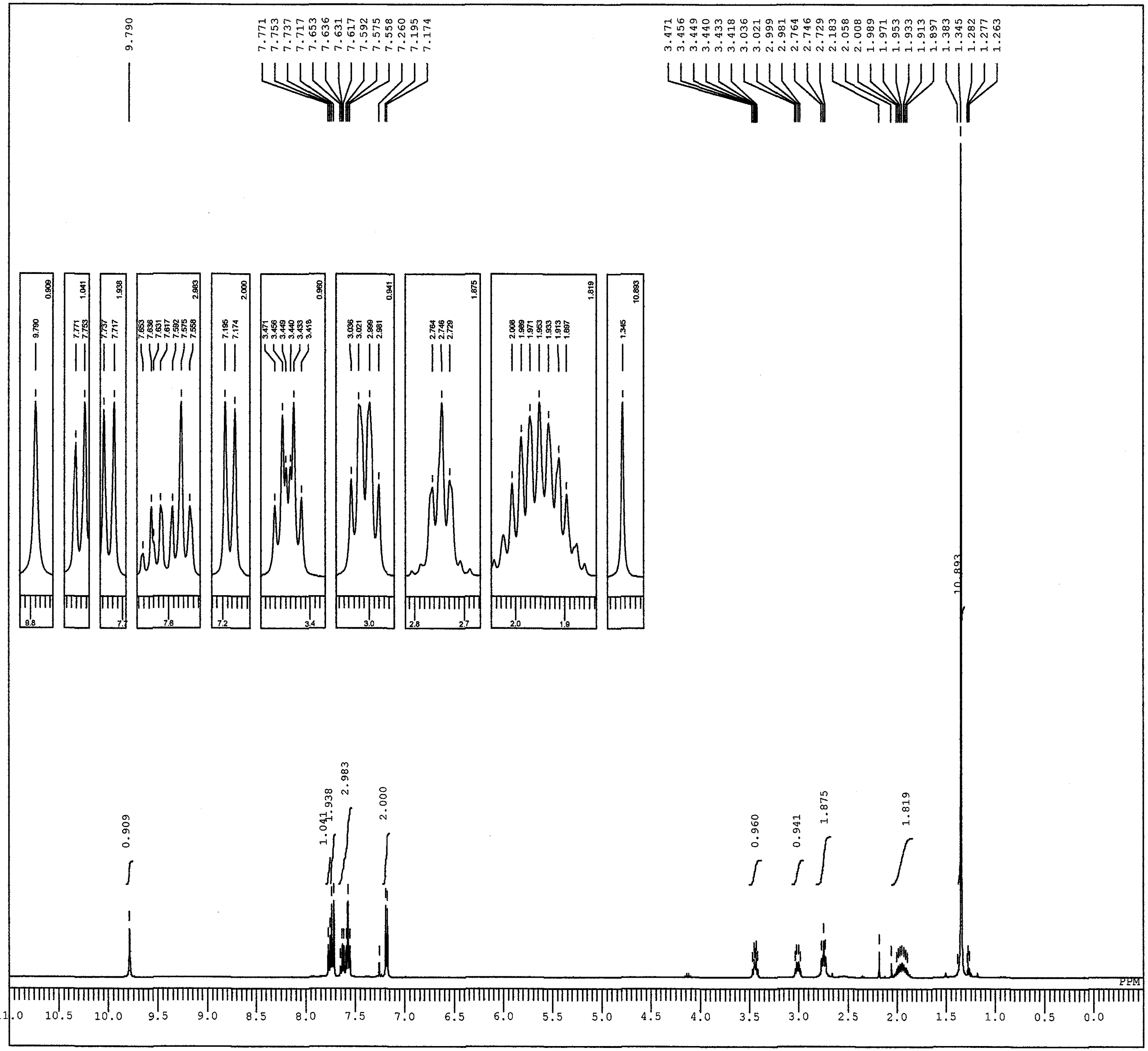

DFILE ozawa06-120_1H.jdf
COMNT 4-Bpin-Ph, OH
DATIM 27-12-2014 11:38:22
OBNUC 1H
EXMOD proton.jxp
OBFRQ 391.78 MHz
OBSET 8.51 KHz
OBFIN 3.34 Hz
POINT 16384
FREQU 7348.62 Hz
SCANS 8
ACQTM 2.2295 sec
PD 5.0000 sec
PW1 5.07 usec
IRNUC 1H
CTEMP 21.9 c
SLVNT CDCL3
EXREF 7.26 ppm
BF 0.12 Hz
RGAIN 26

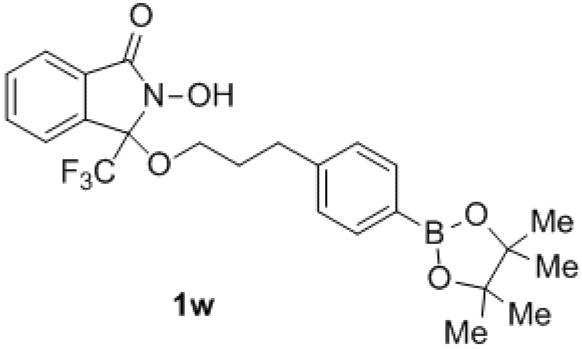

1w

4-Bpin-Ph, OH

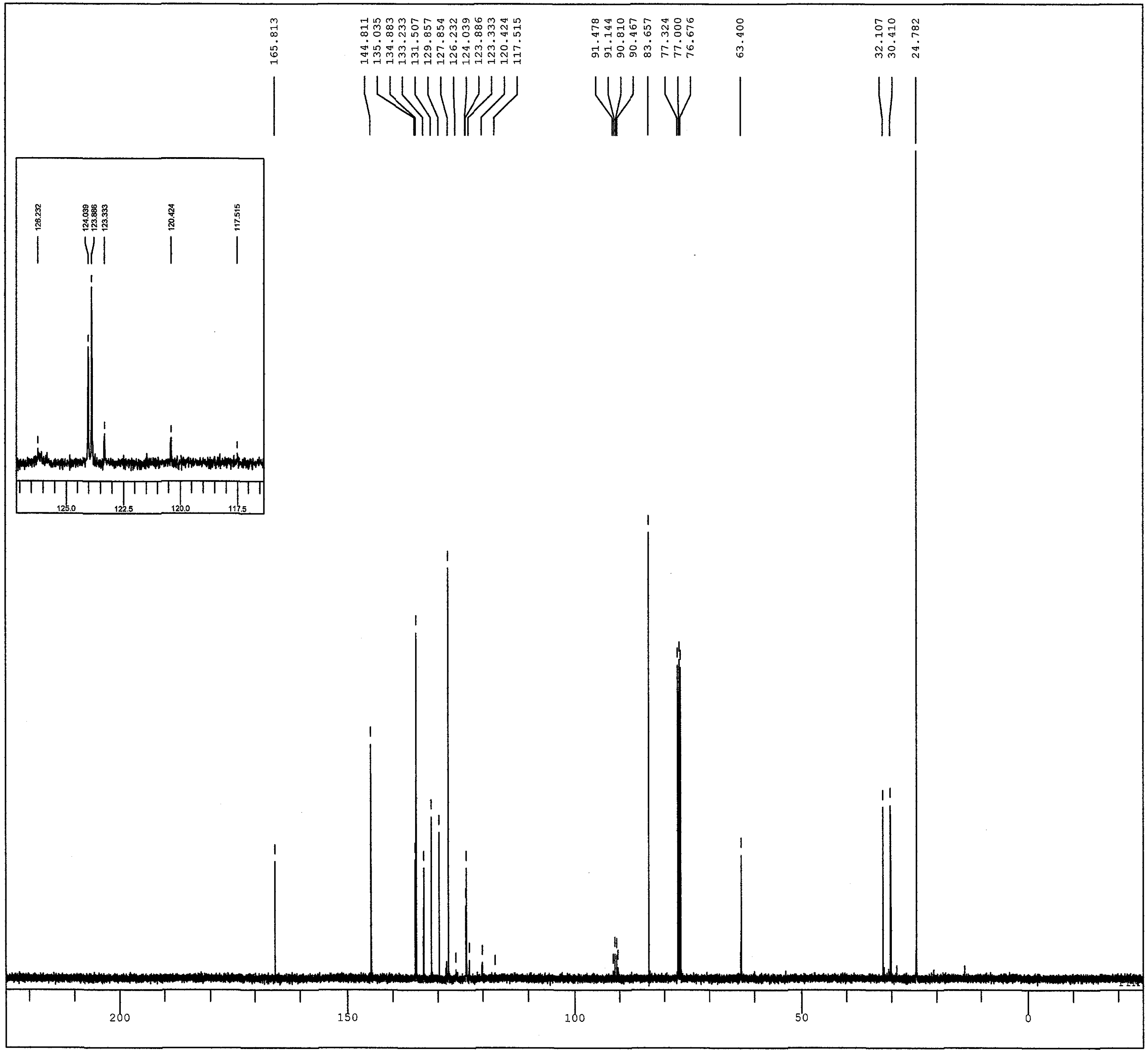


```
DFILE   ozawa06-120_13C.jdf
COMNT   4-Bpin-Ph, OH
DATIM   27-12-2014 11:40:58
OBNUC   13C
EXMOD   carbon.jxp
OBFRQ        98.52 MHz
OBSET         4.64 KHz
OBFIN         8.74 Hz
POINT        32767
FREQU     30788.18 Hz
SCANS          320
ACQTM       1.0643 sec
PD          2.0000 sec
PW1           3.07 usec
IRNUC   1H
CTEMP         22.1 c
SLVNT   CDCL3
EXREF        77.00 ppm
BF            0.12 Hz
RGAIN           60
```

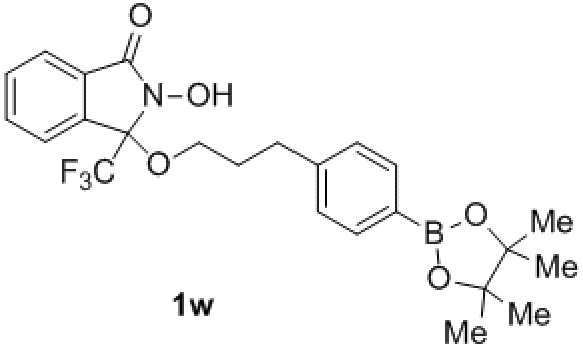

1w

4-Bpin-Ph, OH

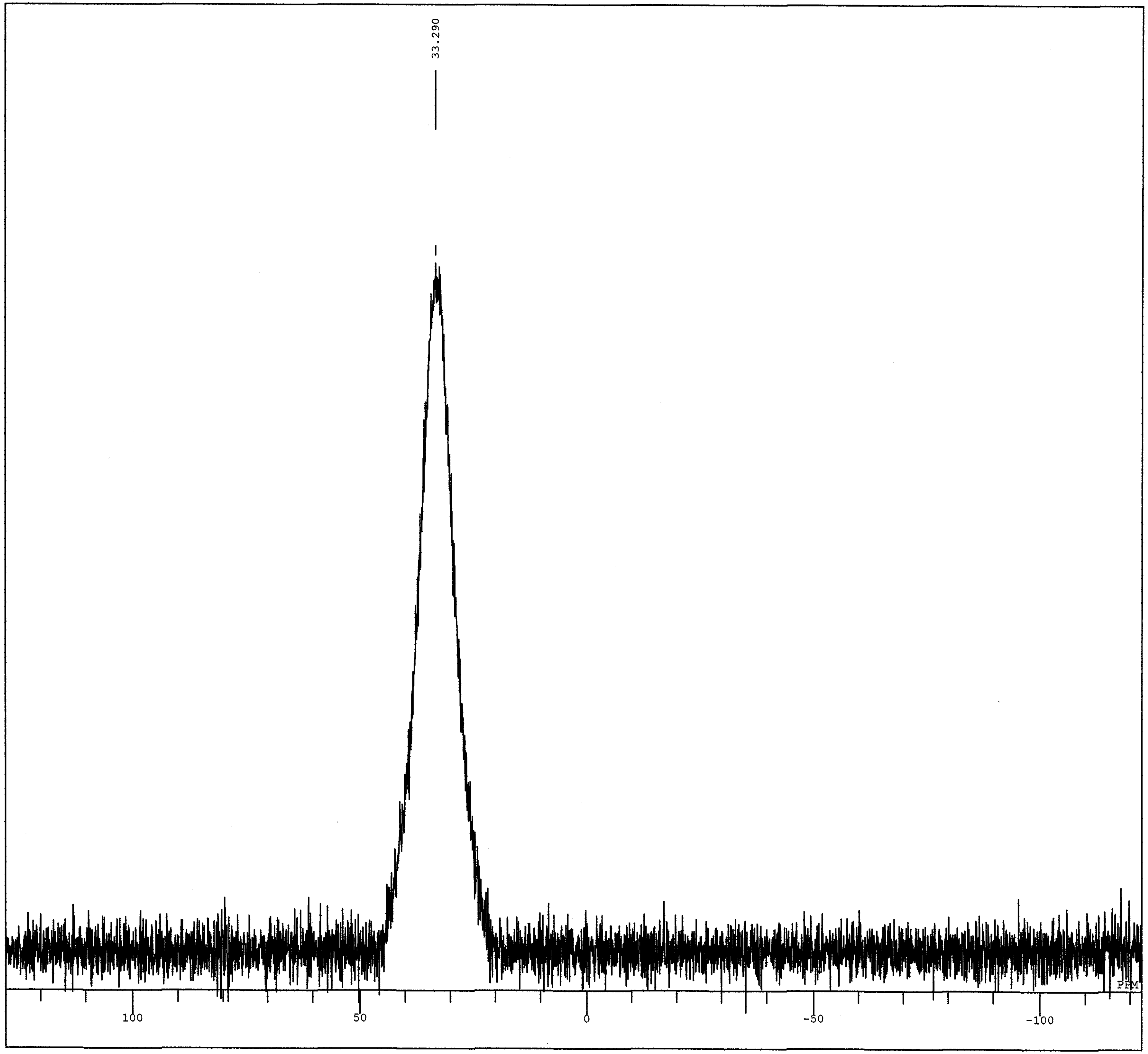

DFILE ozawa06-120_11B.jdf
COMNT 4-Bpin-Ph, OH
DATIM 27-12-2014 11:34:01
OBNUC 11B
EXMOD carbon.jxp
OBFRQ 125.70 MHz
OBSET 0.81 KHz
OBFIN 3.25 Hz
POINT 4095
FREQU 39308.18 Hz
SCANS 200
ACQTM 0.1042 sec
PD 80.0000 sec
PW1 3.98 usec
IRNUC 1H
CTEMP 21.7 c
SLVNT CDCL3
EXREF 31.00 ppm
BF 0.12 Hz
RGAIN 56

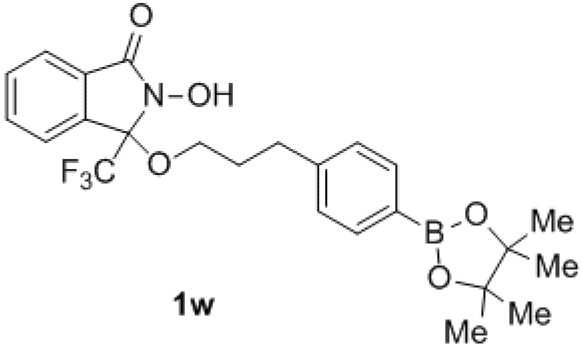

1w

4-Bpin-Ph, OH

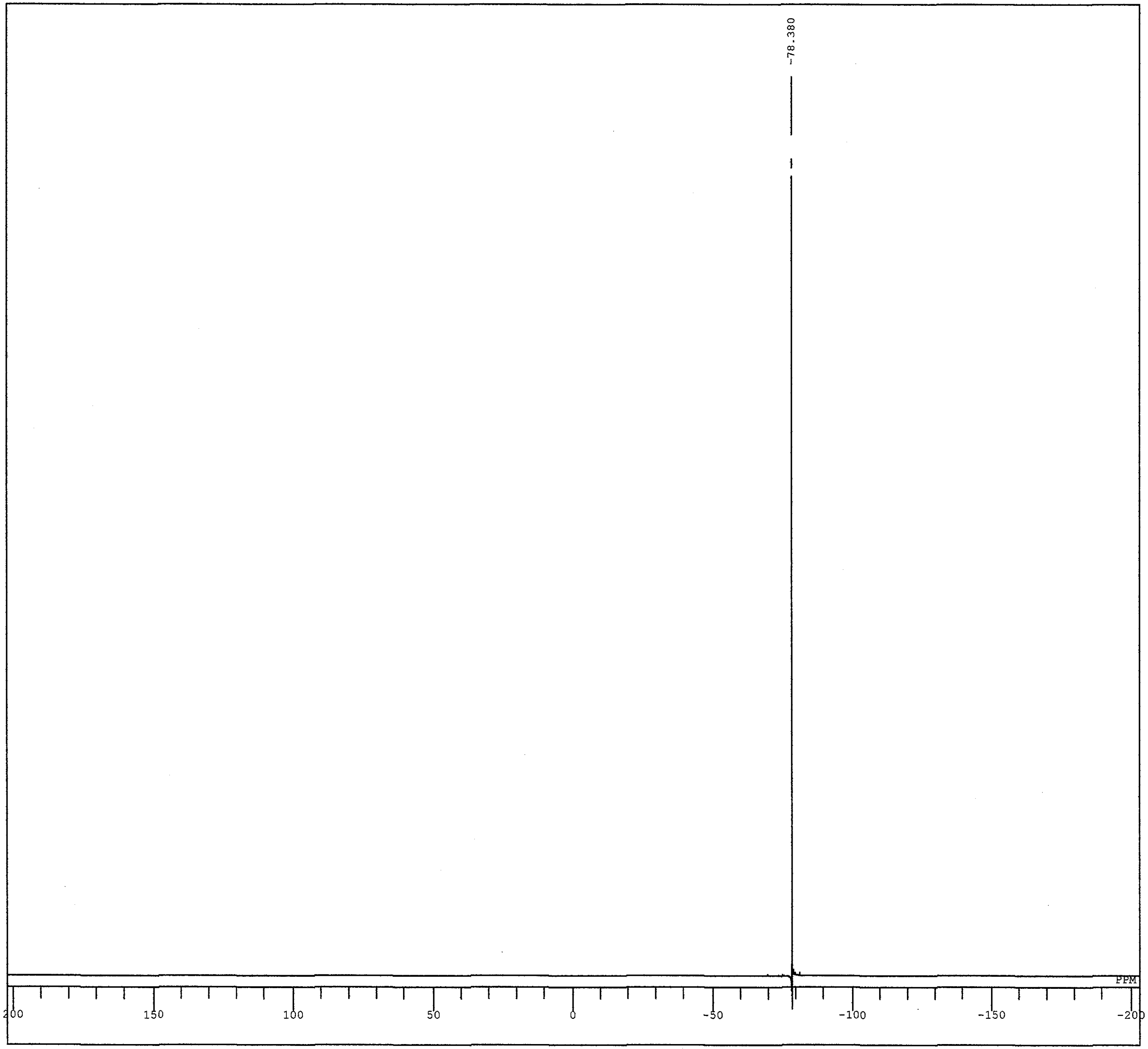


```
DFILE  ozawa06-120_19F.jdf
COMNT  4-Bpin-Ph, OH
DATIM  27-12-2014 11:36:05
OBNUC  19F
EXMOD  proton.jxp
OBFRQ        368.64 MHz
OBSET          7.63 KHz
OBFIN          2.85 Hz
POINT         16384
FREQU     186567.17 Hz
SCANS             4
ACQTM        0.0878 sec
PD           5.0000 sec
PW1            3.90 usec
IRNUC  19F
CTEMP          21.8 c
SLVNT  CDCL3
EXREF       -164.90 ppm
BF             0.12 Hz
RGAIN            46
```

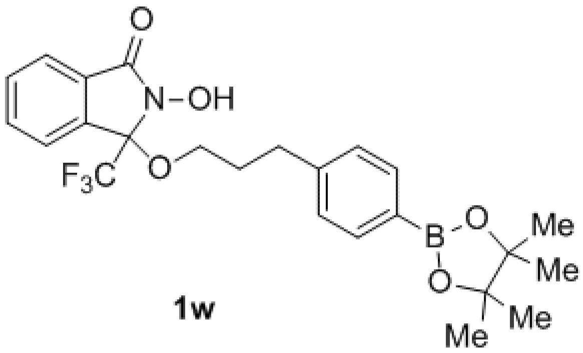

1w

PTLC, 4-Bpin-Ph

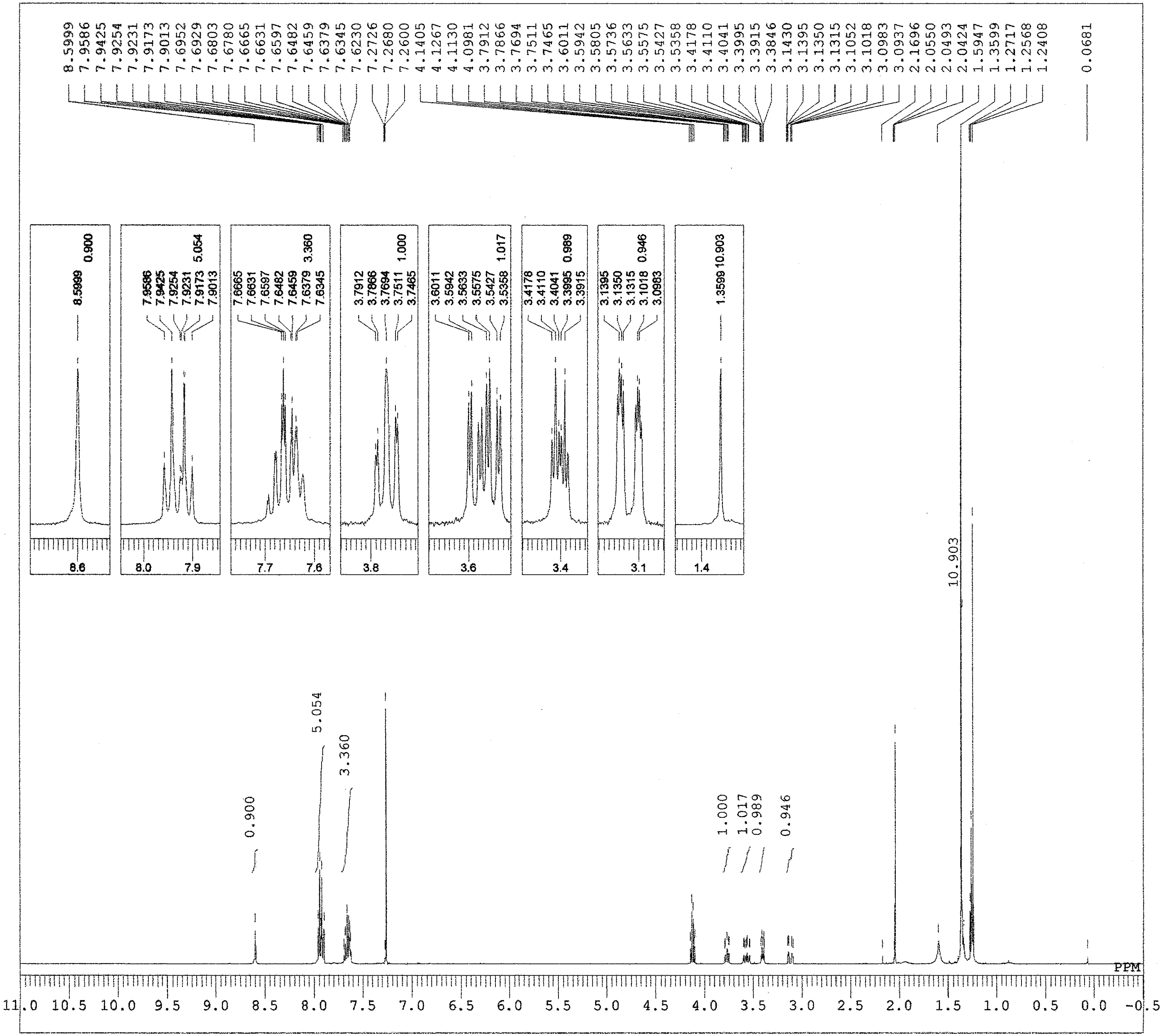

DFILE	ozawa06-121.als
COMNT	PTLC, 4-Bpin-Ph
DATIM	2014-12-27 15:25:04
OBNUC	1H
EXMOD	proton.jxp
OBFRQ	500.16 MHz
OBSET	2.41 KHz
OBFIN	6.01 Hz
POINT	13107
FREQU	7507.51 Hz
SCANS	8
ACQTM	1.7459 sec
PD	5.0000 sec
PW1	5.55 usec
IRNUC	1H
CTEMP	24.6 c
SLVNT	CDCL3
EXREF	7.26 ppm
BF	0.12 Hz
RGAIN	32

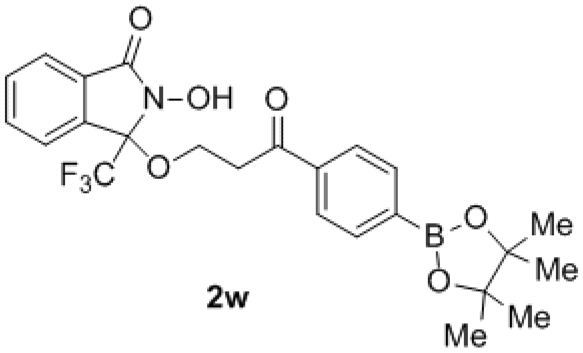

2w

4-Bpin-Ph, [O]

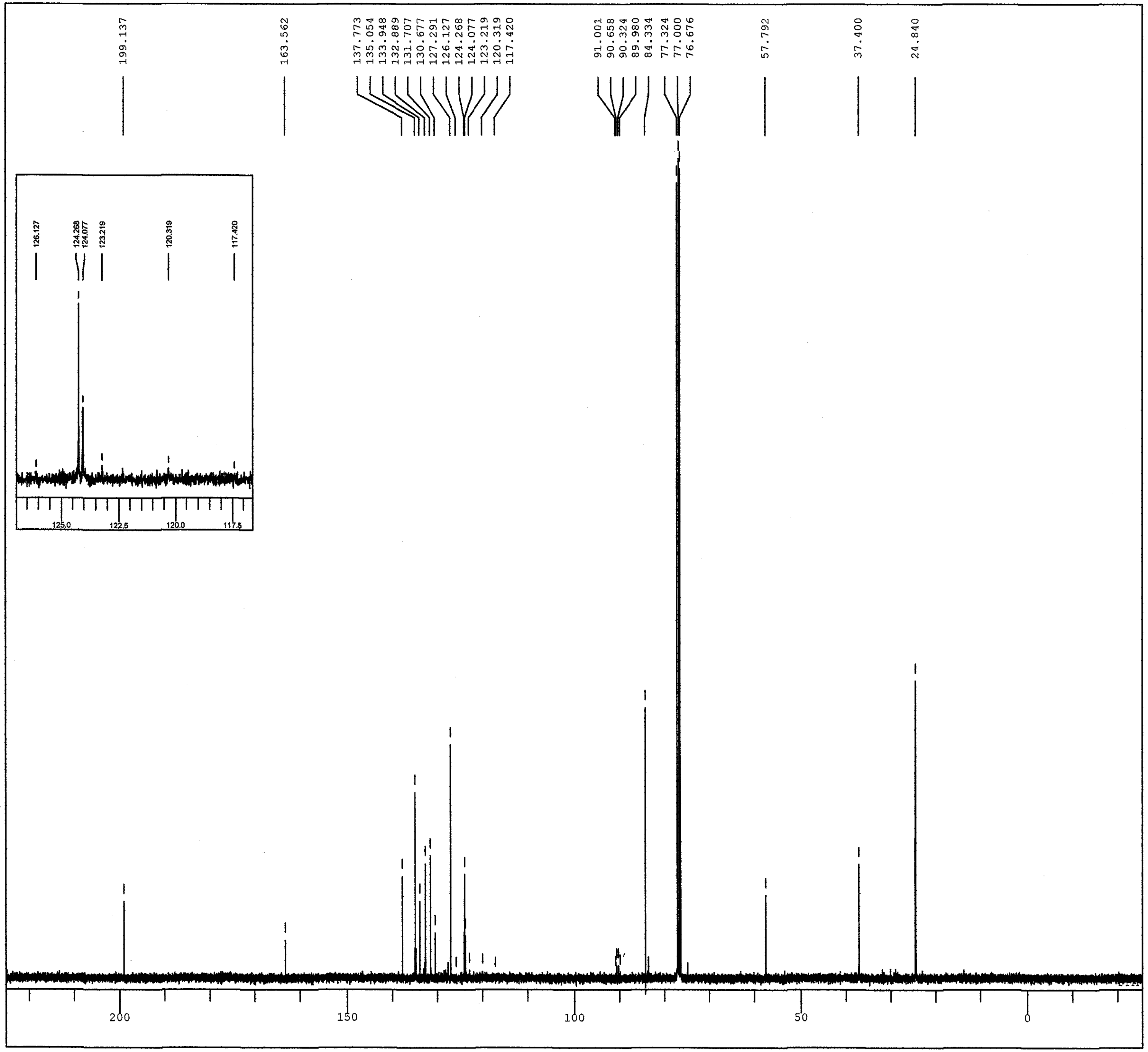

DFILE	ozawa06-121_13C.jdf
COMNT	4-Bpin-Ph, [O]
DATIM	06-01-2015 07:38:31
OBNUC	13C
EXMOD	carbon.jxp
OBFRQ	98.52 MHz
OBSET	4.64 KHz
OBFIN	8.74 Hz
POINT	32767
FREQU	30788.18 Hz
SCANS	2000
ACQTM	1.0643 sec
PD	2.0000 sec
PW1	3.07 usec
IRNUC	1H
CTEMP	21.9 c
SLVNT	CDCL3
EXREF	77.00 ppm
BF	0.10 Hz
RGAIN	60

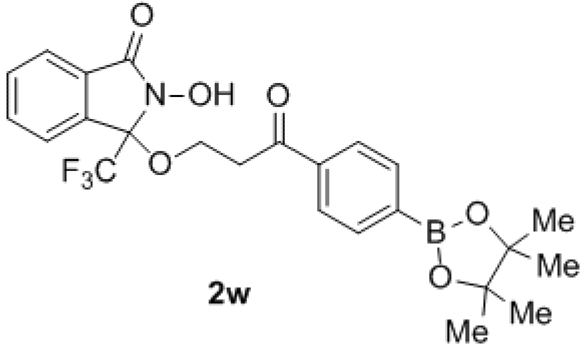

2w

4-Bpin-Ph, [O]

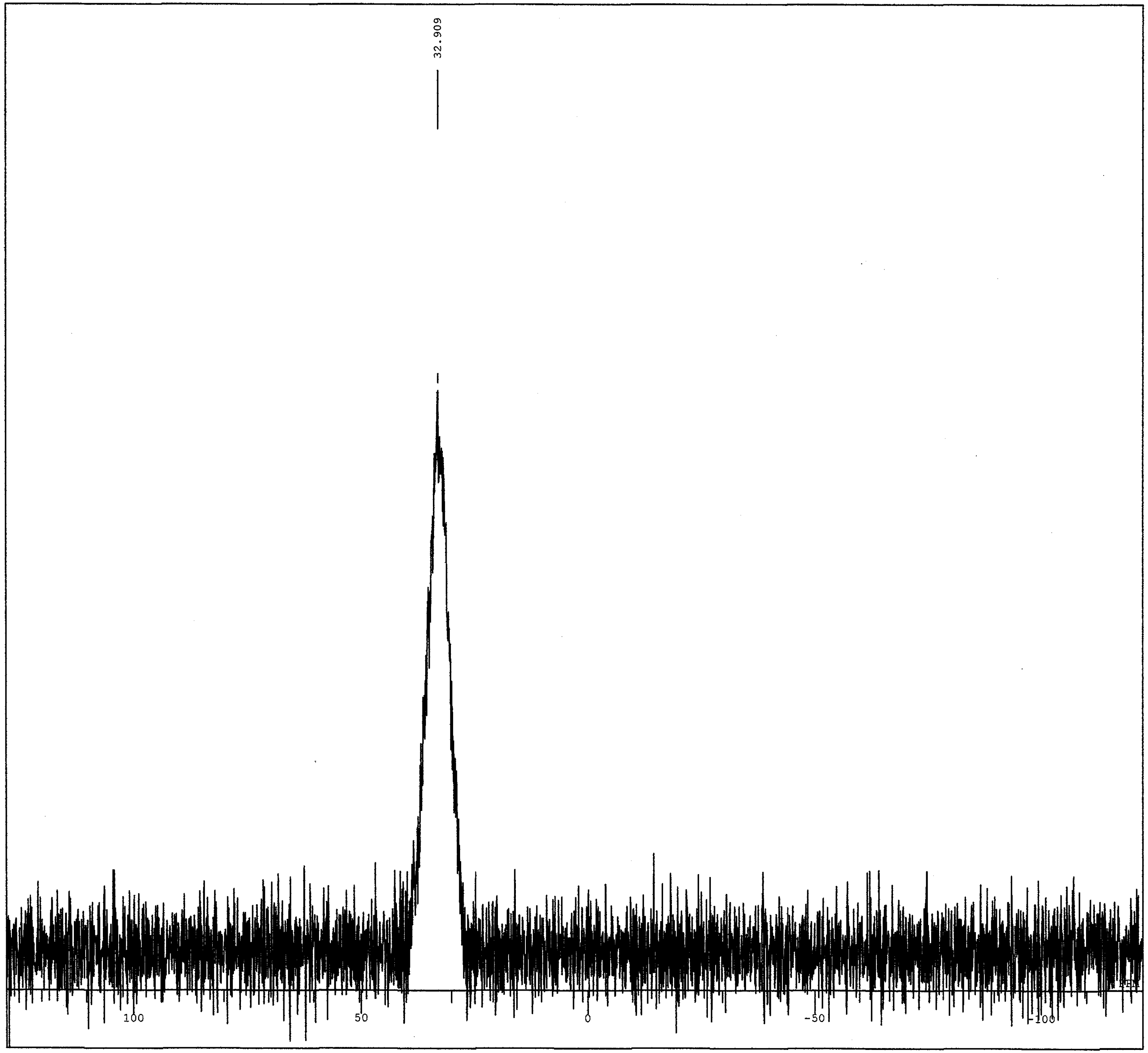

DFILE	ozawa06-121_11B.jdf
COMNT	4-Bpin-Ph, [O]
DATIM	05-01-2015 12:30:28
OBNUC	11B
EXMOD	carbon.jxp
OBFRQ	125.70 MHz
OBSET	0.81 KHz
OBFIN	3.25 Hz
POINT	4095
FREQU	39308.18 Hz
SCANS	500
ACQTM	0.1042 sec
PD	80.0000 sec
PW1	3.98 usec
IRNUC	1H
CTEMP	21.7 c
SLVNT	CDCL3
EXREF	31.00 ppm
BF	0.12 Hz
RGAIN	60

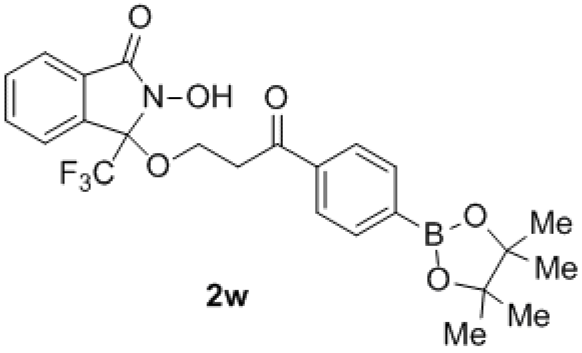

2w

4-Bpin-Ph, [O]

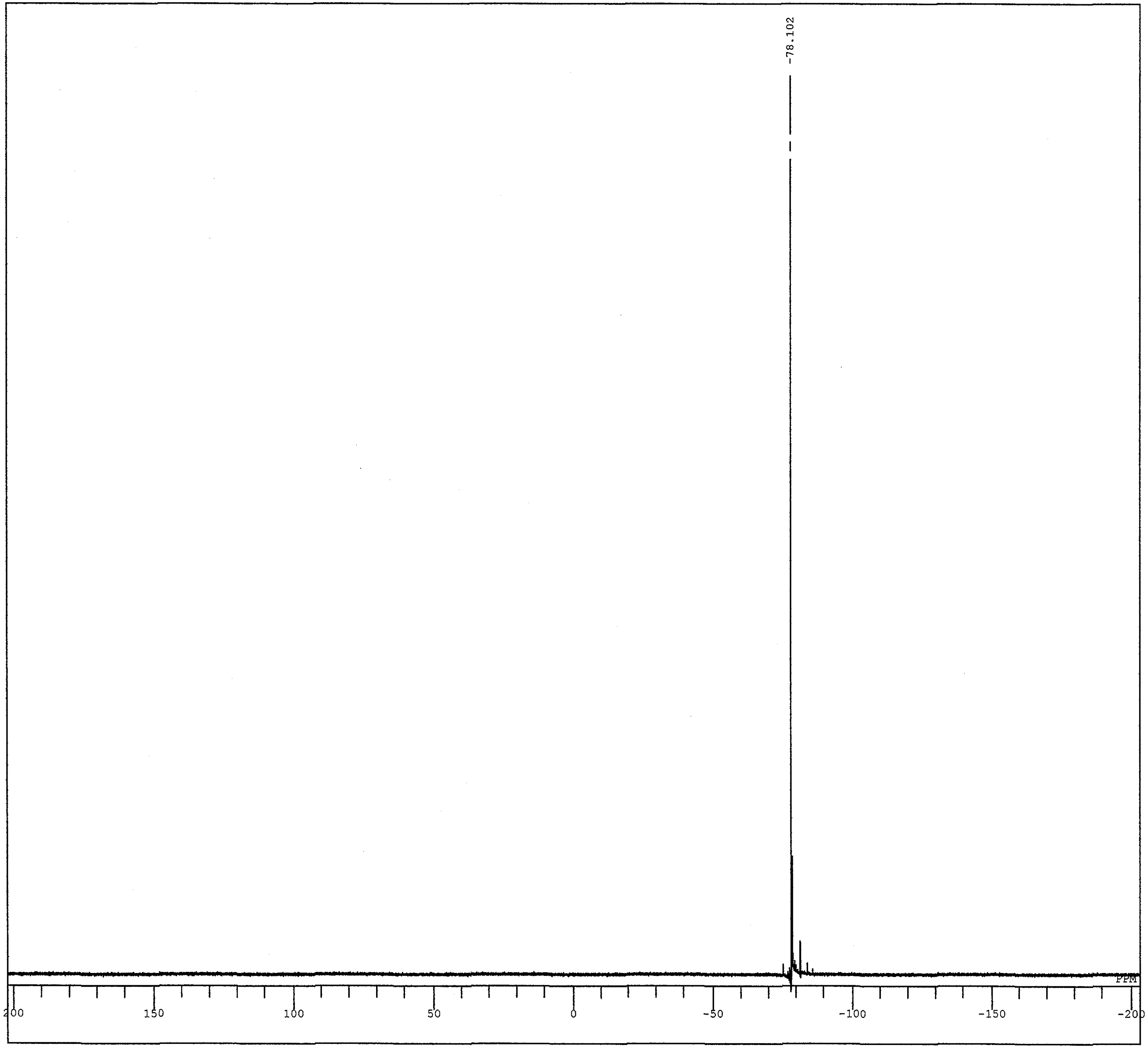

DFILE ozawa06-121_19F.jdf
COMNT 4-Bpin-Ph, [O]
DATIM 05-01-2015 12:27:22
OBNUC 19F
EXMOD proton.jxp
OBFRQ 368.64 MHz
OBSET 7.63 KHz
OBFIN 2.85 Hz
POINT 16384
FREQU 186567.17 Hz
SCANS 4
ACQTM 0.0878 sec
PD 5.0000 sec
PW1 3.90 usec
IRNUC 19F
CTEMP 21.6 c
SLVNT CDCL3
EXREF -164.90 ppm
BF 0.12 Hz
RGAIN 50

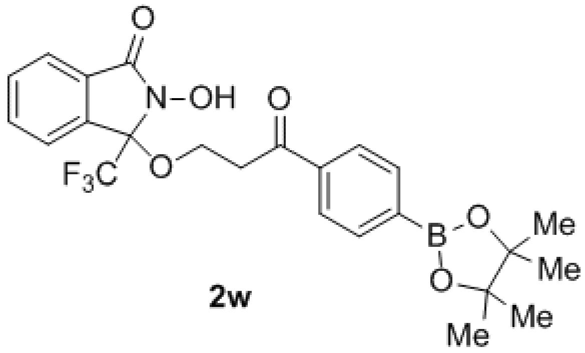

2w

2-I-Ph

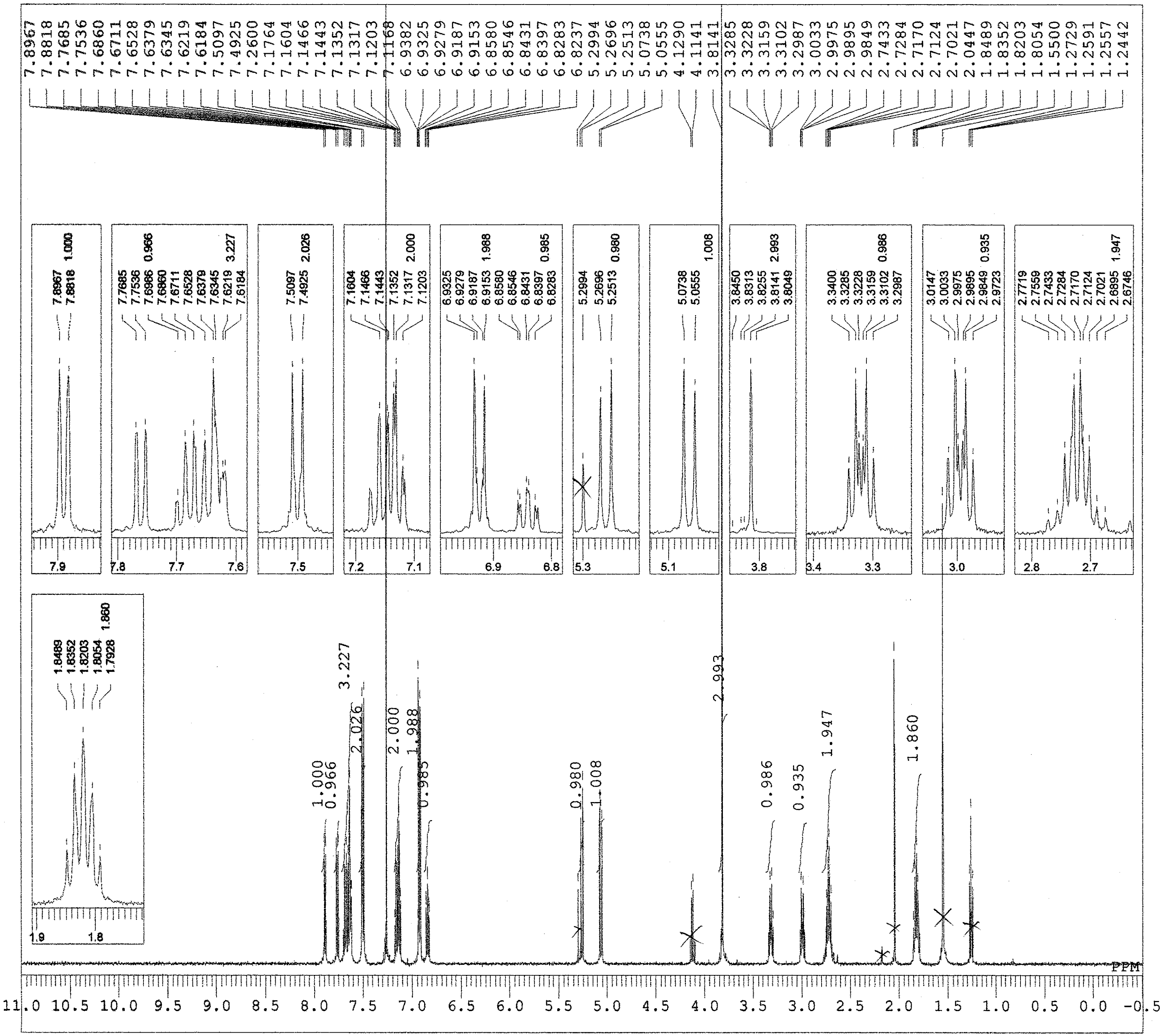

DFILE	ozawa06-144.als
COMNT	2-I-Ph
DATIM	2015-01-22 12:59:51
OBNUC	1H
EXMOD	proton.jxp
OBFRQ	500.16 MHz
OBSET	2.41 KHz
OBFIN	6.01 Hz
POINT	13107
FREQU	7507.51 Hz
SCANS	4
ACQTM	1.7459 sec
PD	5.0000 sec
PW1	5.55 usec
IRNUC	1H
CTEMP	23.2 c
SLVNT	CDCL3
EXREF	7.26 ppm
BF	0.12 Hz
RGAIN	42

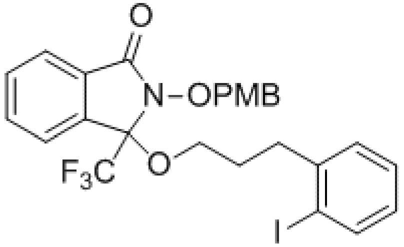

S4'x

PTLC, 2-I-Ph, PMB

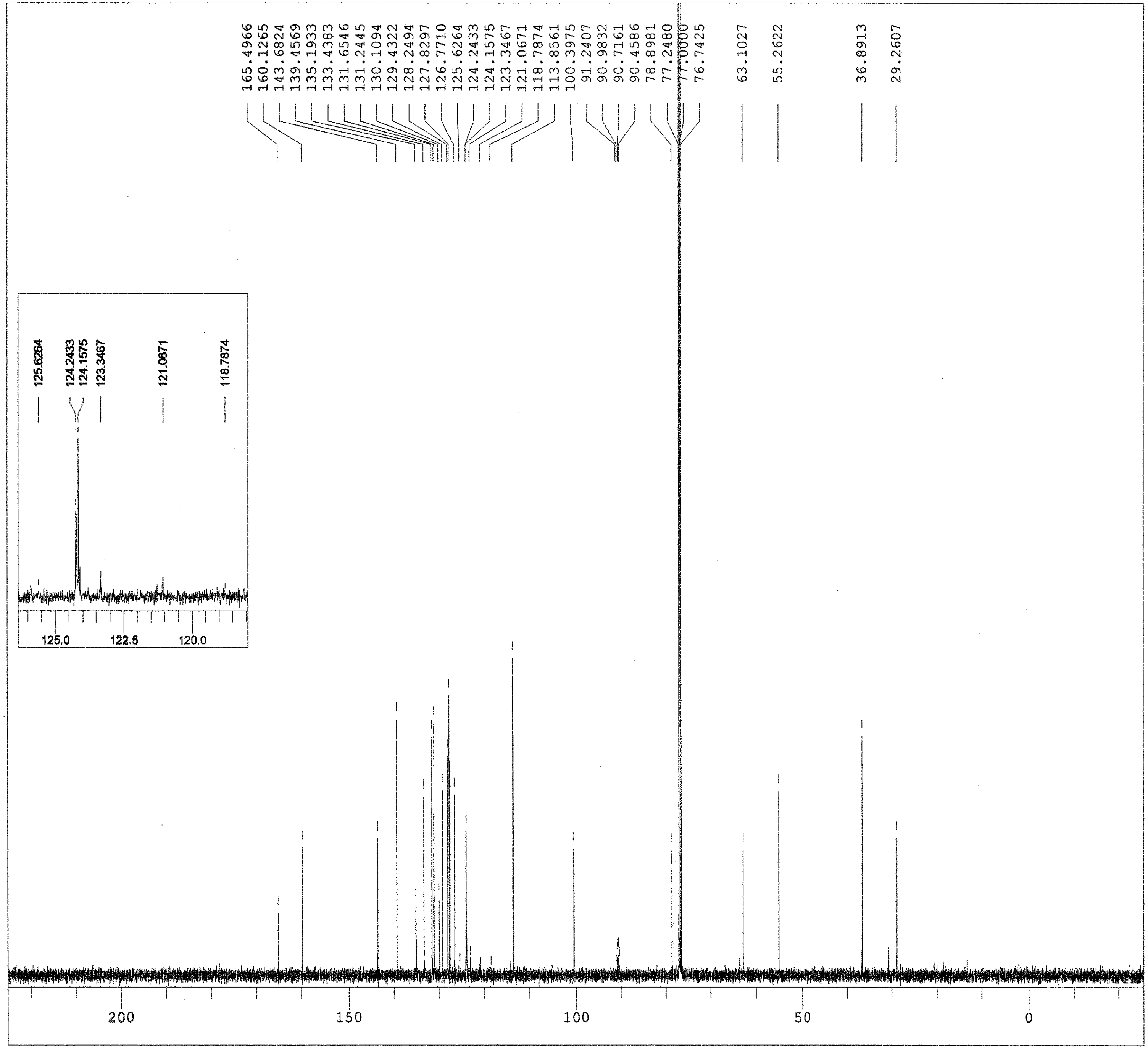

DFILE	ozawa06-144_13C.jdf
COMNT	PTLC, 2-I-Ph, PMB
DATIM	2015-01-26 13:52:31
OBNUC	13C
EXMOD	carbon.jxp
OBFRQ	125.77 MHz
OBSET	7.87 KHz
OBFIN	4.21 Hz
POINT	32767
FREQU	39308.18 Hz
SCANS	1000
ACQTM	0.8336 sec
PD	2.0000 sec
PW1	3.40 usec
IRNUC	1H
CTEMP	23.6 c
SLVNT	CDCL3
EXREF	77.00 ppm
BF	0.12 Hz
RGAIN	60

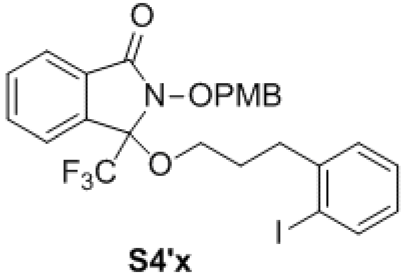

S4'x

2-I-Ph, PMB

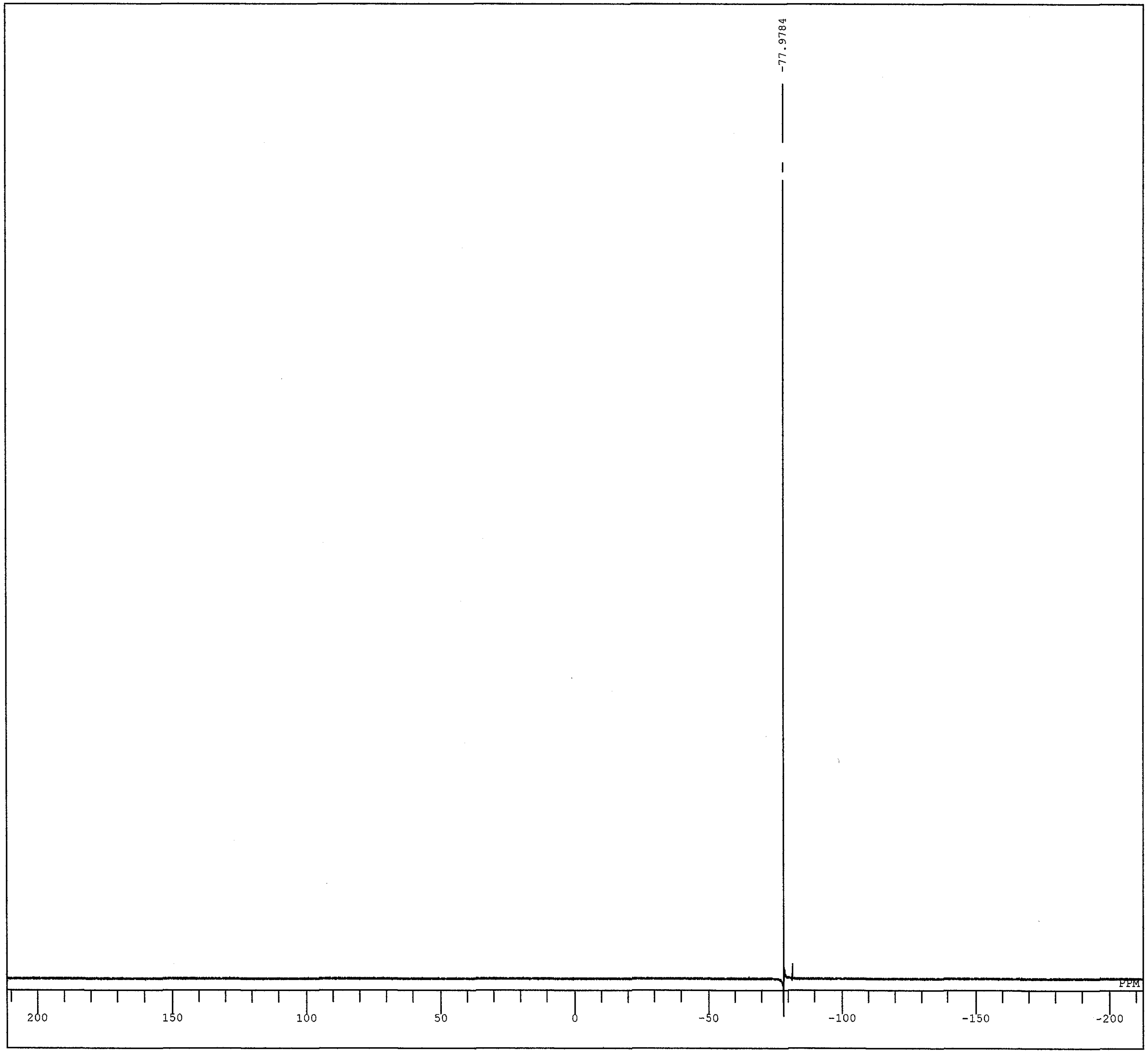

DFILE ozawa06-144_19F.jdf
COMNT 2-I-Ph, PMB
DATIM 21-03-2015 12:18:53
OBNUC 19F
EXMOD proton.jxp
OBFRQ 368.64 MHz
OBSET 7.63 KHz
OBFIN 2.85 Hz
POINT 16384
FREQU 195312.50 Hz
SCANS 4
ACQTM 0.0839 sec
PD 5.0000 sec
PW1 3.90 usec
IRNUC 19F
CTEMP 25.1 c
SLVNT CDCL3
EXREF -164.90 ppm
BF 0.12 Hz
RGAIN 50

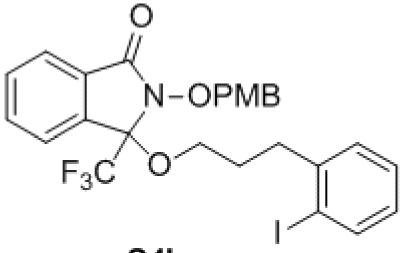

S4'x

2-I-Ph, OH

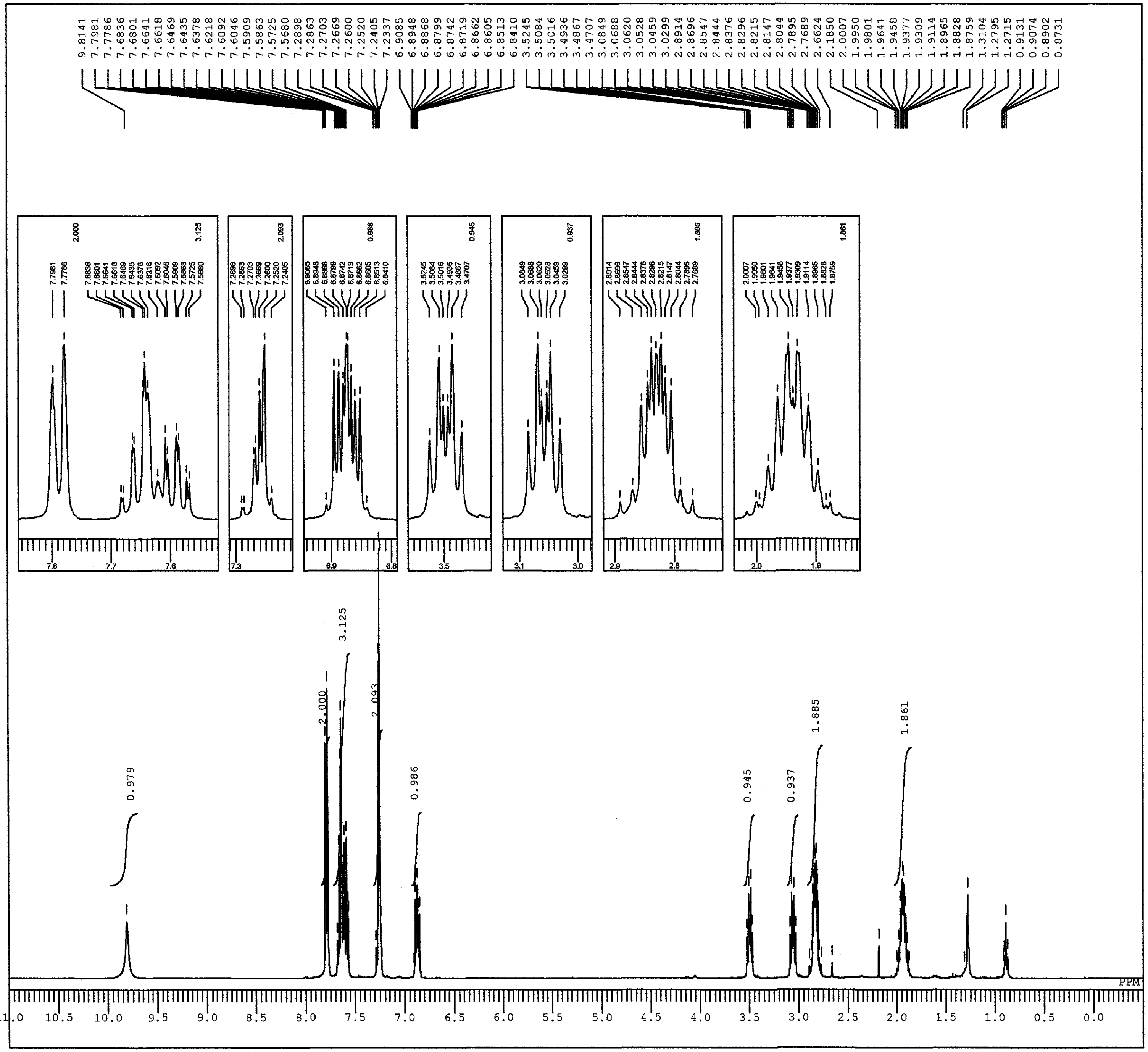

DFILE ozawa06-157_1H.jdf
COMNT 2-I-Ph, OH
DATIM 14-03-2015 08:29:06
OBNUC 1H
EXMOD proton.jxp
OBFRQ 391.78 MHz
OBSET 8.51 KHz
OBFIN 3.34 Hz
POINT 16384
FREQU 7348.62 Hz
SCANS 4
ACQTM 2.2295 sec
PD 5.0000 sec
PW1 4.99 usec
IRNUC 1H
CTEMP 24.4 c
SLVNT CDCL3
EXREF 7.26 ppm
BF 0.12 Hz
RGAIN 30

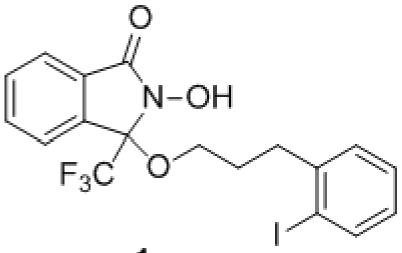

1x

2-I-Ph, OH

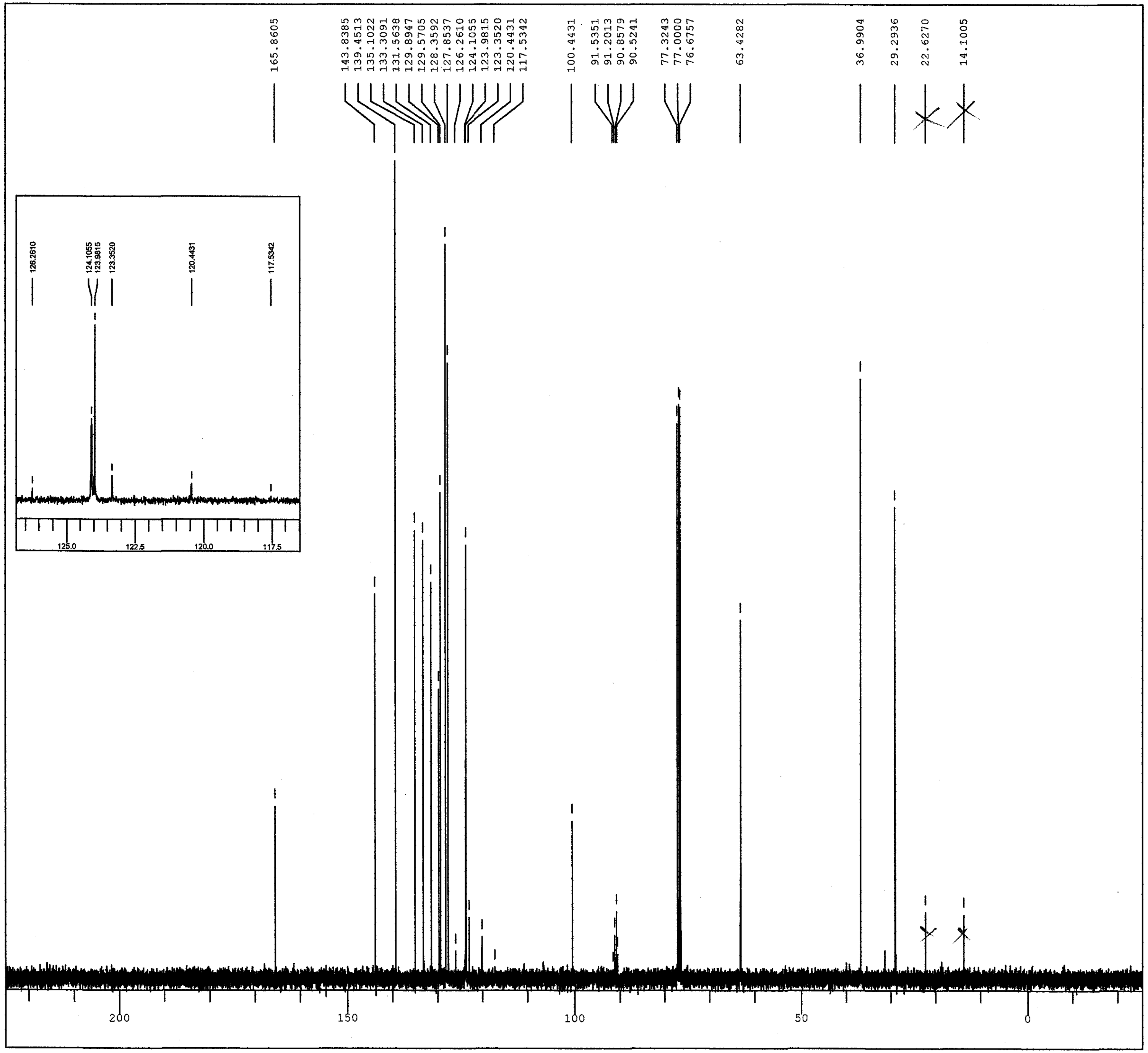

DFILE ozawa06-157_13C.jdf
COMNT 2-I-Ph, OH
DATIM 14-03-2015 08:31:37
OBNUC 13C
EXMOD carbon.jxp
OBFRQ 98.52 MHz
OBSET 4.64 KHz
OBFIN 8.74 Hz
POINT 32767
FREQU 30788.18 Hz
SCANS 348
ACQTM 1.0643 sec
PD 2.0000 sec
PW1 3.16 usec
IRNUC 1H
CTEMP 24.6 c
SLVNT CDCL3
EXREF 77.00 ppm
BF 0.12 Hz
RGAIN 60

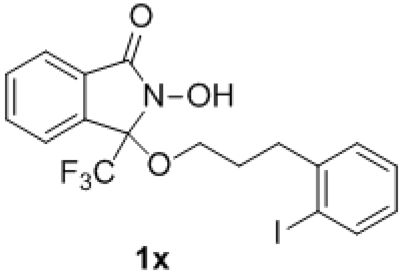

1x

2-I-Ph, OH

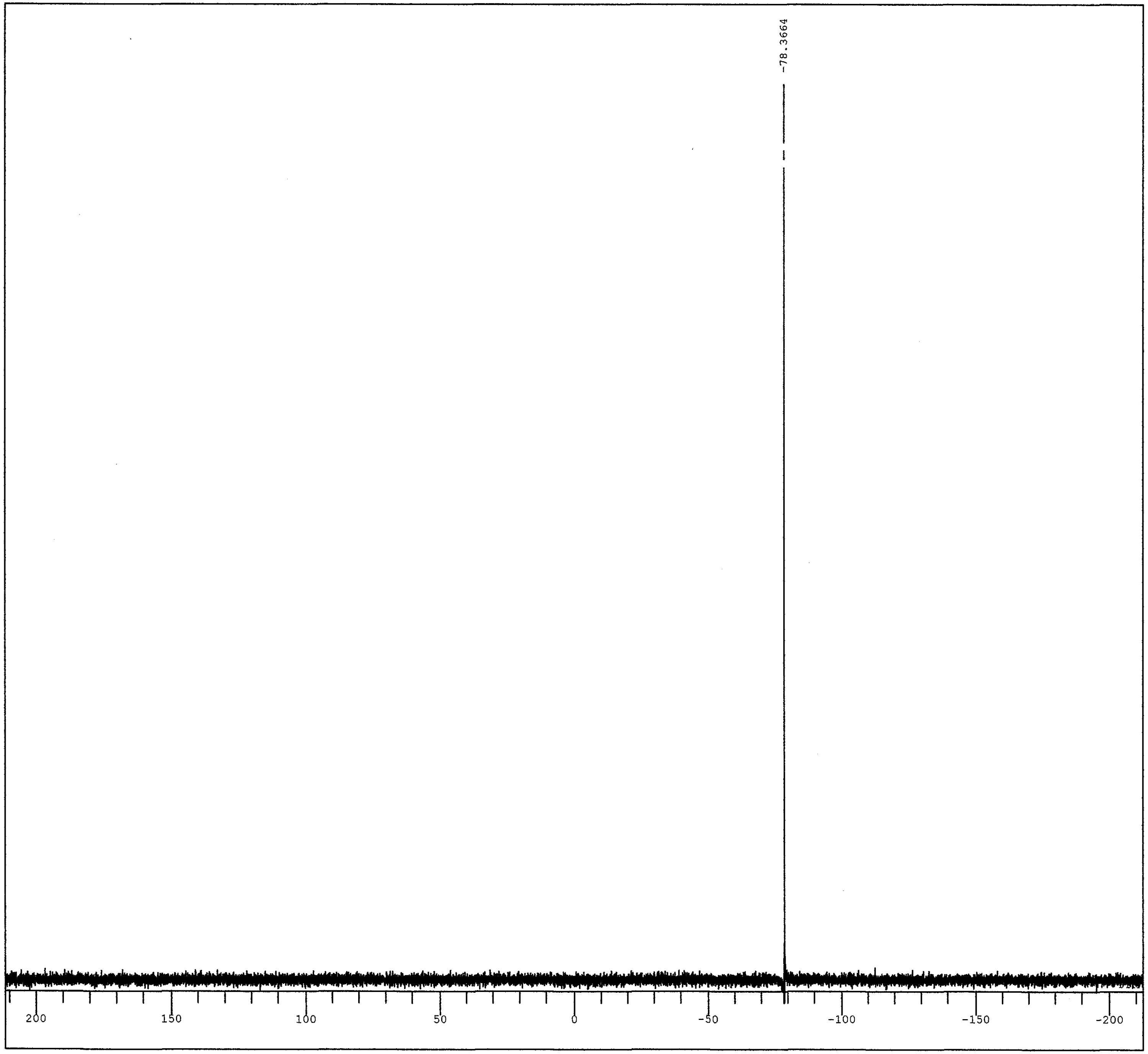

DFILE ozawa06-157_19F.jdf
COMNT 2-I-Ph, OH
DATIM 21-03-2015 11:38:46
OBNUC 19F
EXMOD proton.jxp
OBFRQ 368.64 MHz
OBSET 7.63 KHz
OBFIN 2.85 Hz
POINT 16384
FREQU 195312.50 Hz
SCANS 4
ACQTM 0.0839 sec
PD 5.0000 sec
PW1 3.90 usec
IRNUC 19F
CTEMP 25.1 c
SLVNT CDCL3
EXREF -164.90 ppm
BF 0.12 Hz
RGAIN 50

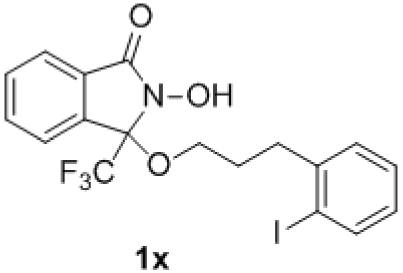

1x

2-I-Ph, [O]

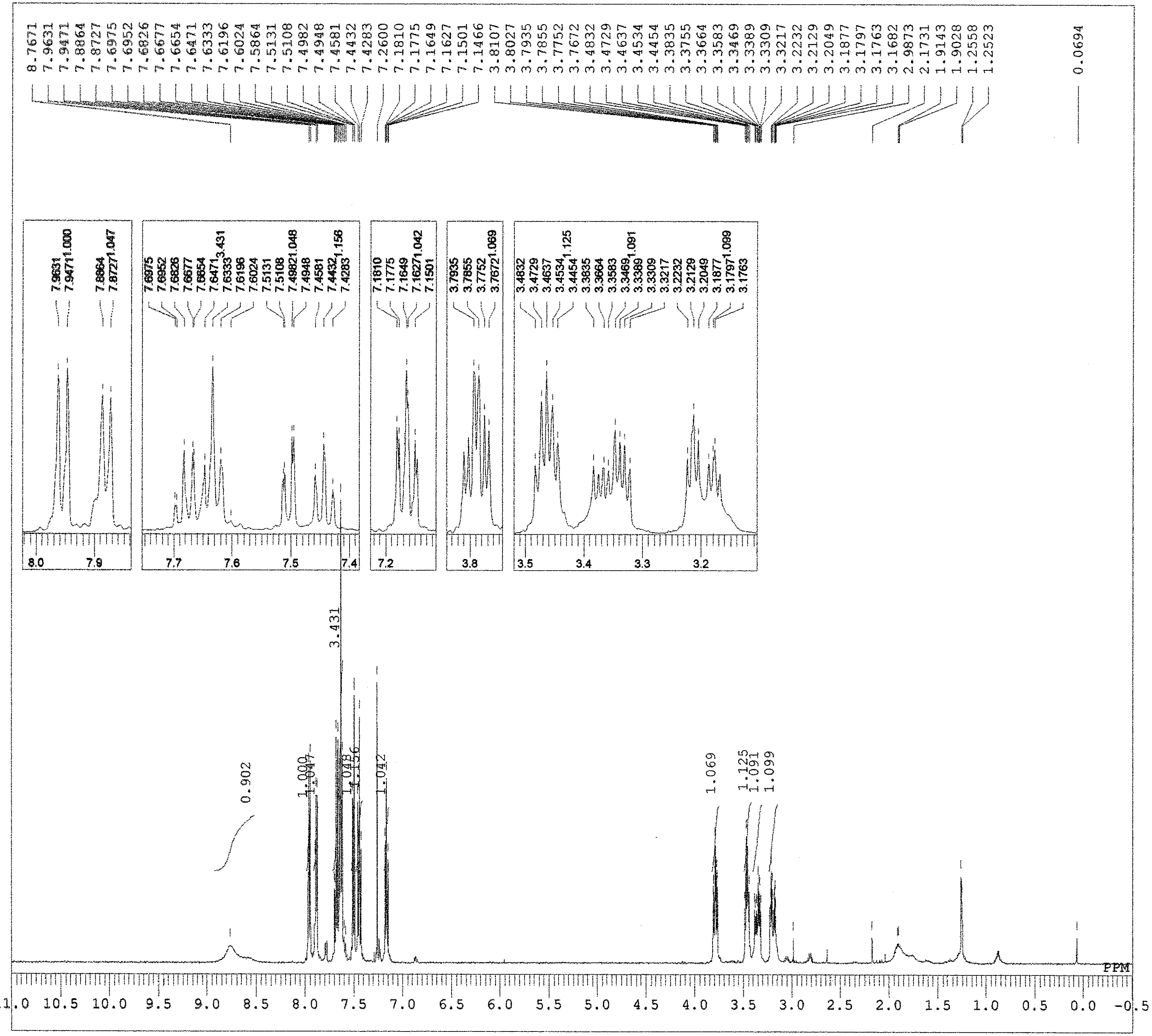

DFILE	ozawa06-161_1H.jdf
COMNT	2-I-Ph, [O]
DATIM	2015-03-14 07:18:52
OBNUC	1H
EXMOD	proton.jxp
OBFRQ	500.16 MHz
OBSET	2.41 KHz
OBFIN	6.01 Hz
POINT	16384
FREQU	9384.38 Hz
SCANS	4
ACQTM	1.7459 sec
PD	5.0000 sec
PW1	5.55 usec
IRNUC	1H
CTEMP	24.8 c
SLVNT	CDCL3
EXREF	7.26 ppm
BF	0.12 Hz
RGAIN	30

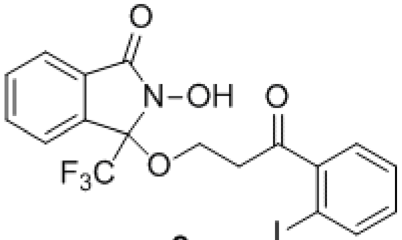

2x

2-I-Ph, [O]

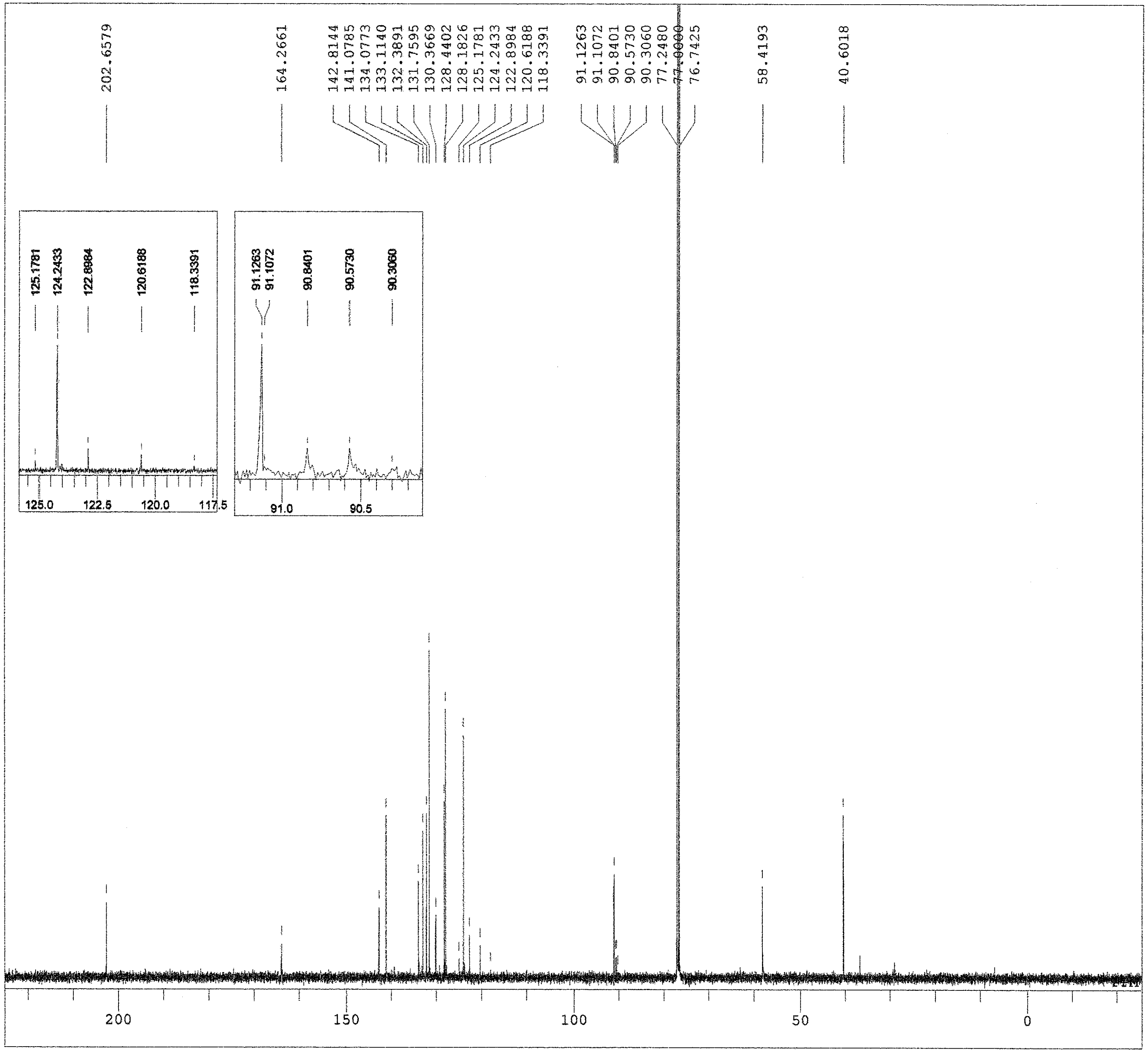

DFILE ozawa06-161_13C.jdf
COMNT 2-I-Ph, [O]
DATIM 2015-03-14 07:19:55
OBNUC 13C
EXMOD carbon.jxp
OBFRQ 125.77 MHz
OBSET 7.87 KHz
OBFIN 4.21 Hz
POINT 32767
FREQU 39308.18 Hz
SCANS 2472
ACQTM 0.8336 sec
PD 2.0000 sec
PW1 3.40 usec
IRNUC 1H
CTEMP 25.7 c
SLVNT CDCL3
EXREF 77.00 ppm
BF 0.12 Hz
RGAIN 60

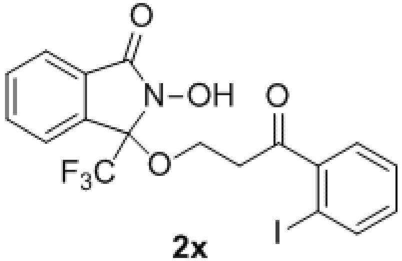

2x

2-I-Ph, [O]

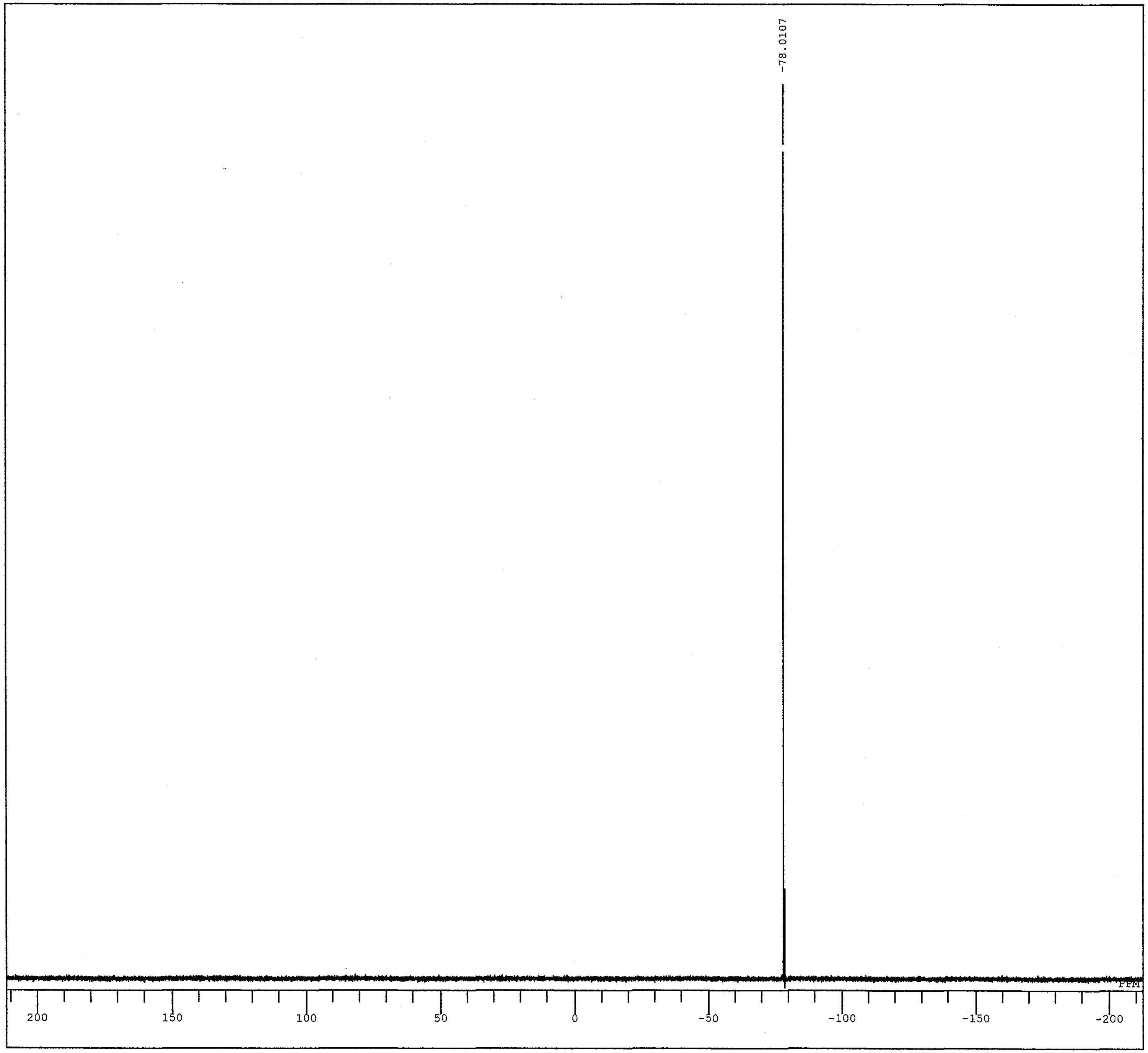


```
DFILE  ozawa06-161_1_19F.jdf
COMNT  2-I-Ph, [O]
DATIM  21-03-2015 11:36:27
OBNUC  19F
EXMOD  proton.jxp
OBFRQ        368.64 MHz
OBSET          7.63 KHz
OBFIN          2.85 Hz
POINT         16384
FREQU     195312.50 Hz
SCANS             4
ACQTM        0.0839 sec
PD           5.0000 sec
PW1            3.90 usec
IRNUC  19F
CTEMP          25.1 c
SLVNT  CDCL3
EXREF       -164.90 ppm
BF             0.12 Hz
RGAIN            50
```

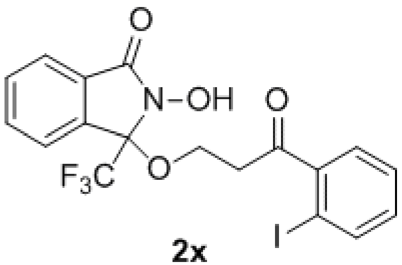

2x

2-TMS-Ph, Bn

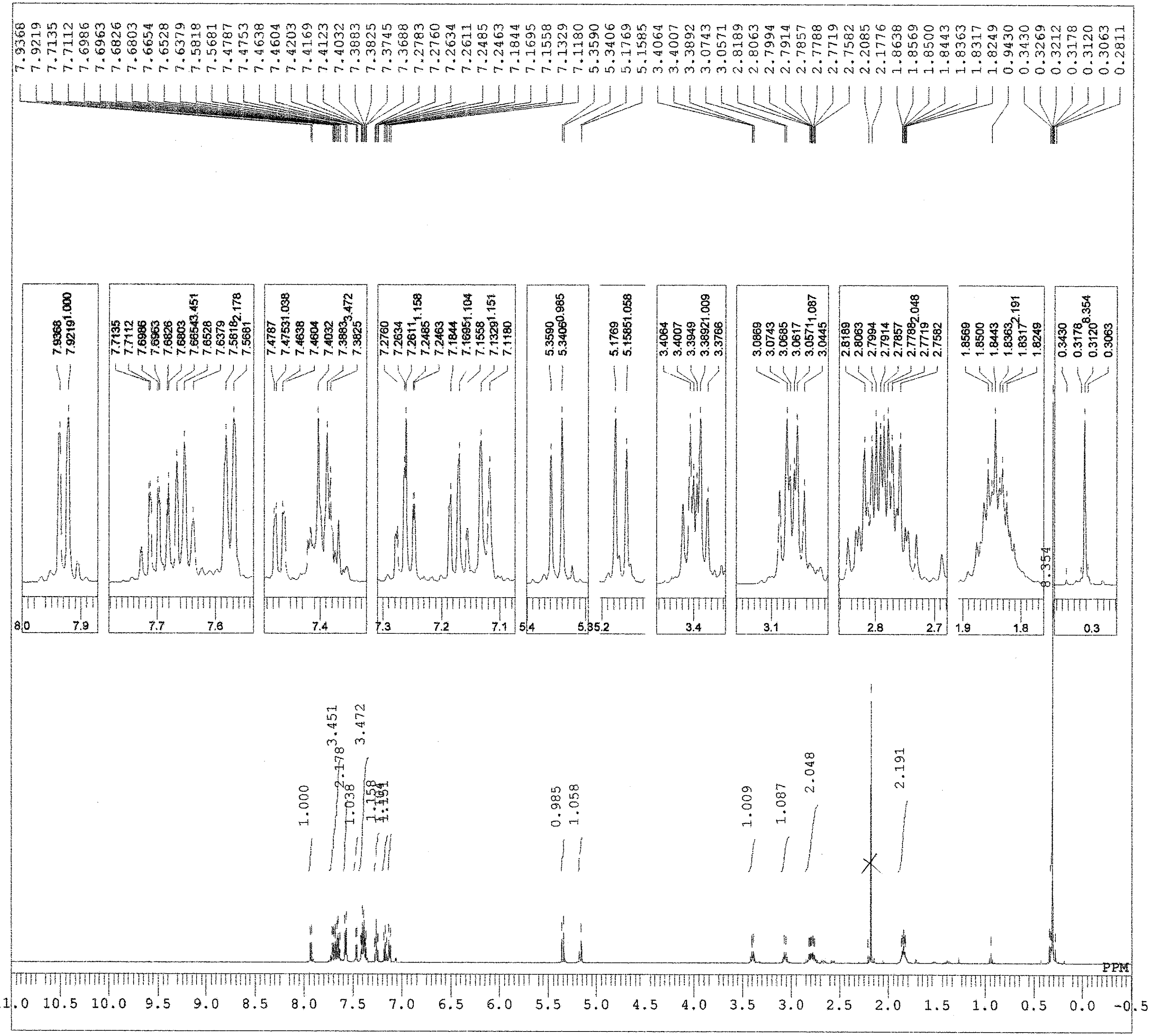

DFILE	ozawa07-034_1H.als
COMNT	2-TMS-Ph, Bn
DATIM	2015-03-09 10:43:25
OBNUC	1H
EXMOD	proton.jxp
OBFRQ	500.16 MHz
OBSET	2.41 KHz
OBFIN	6.01 Hz
POINT	13107
FREQU	7507.51 Hz
SCANS	4
ACQTM	1.7459 sec
PD	5.0000 sec
PW1	5.55 usec
IRNUC	1H
CTEMP	24.6 c
SLVNT	CDCL3
EXREF	7.26 ppm
BF	0.12 Hz
RGAIN	26

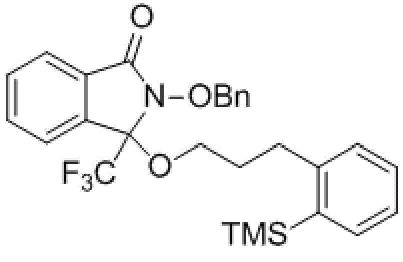

S4y

2-TMS-Ph, Bn

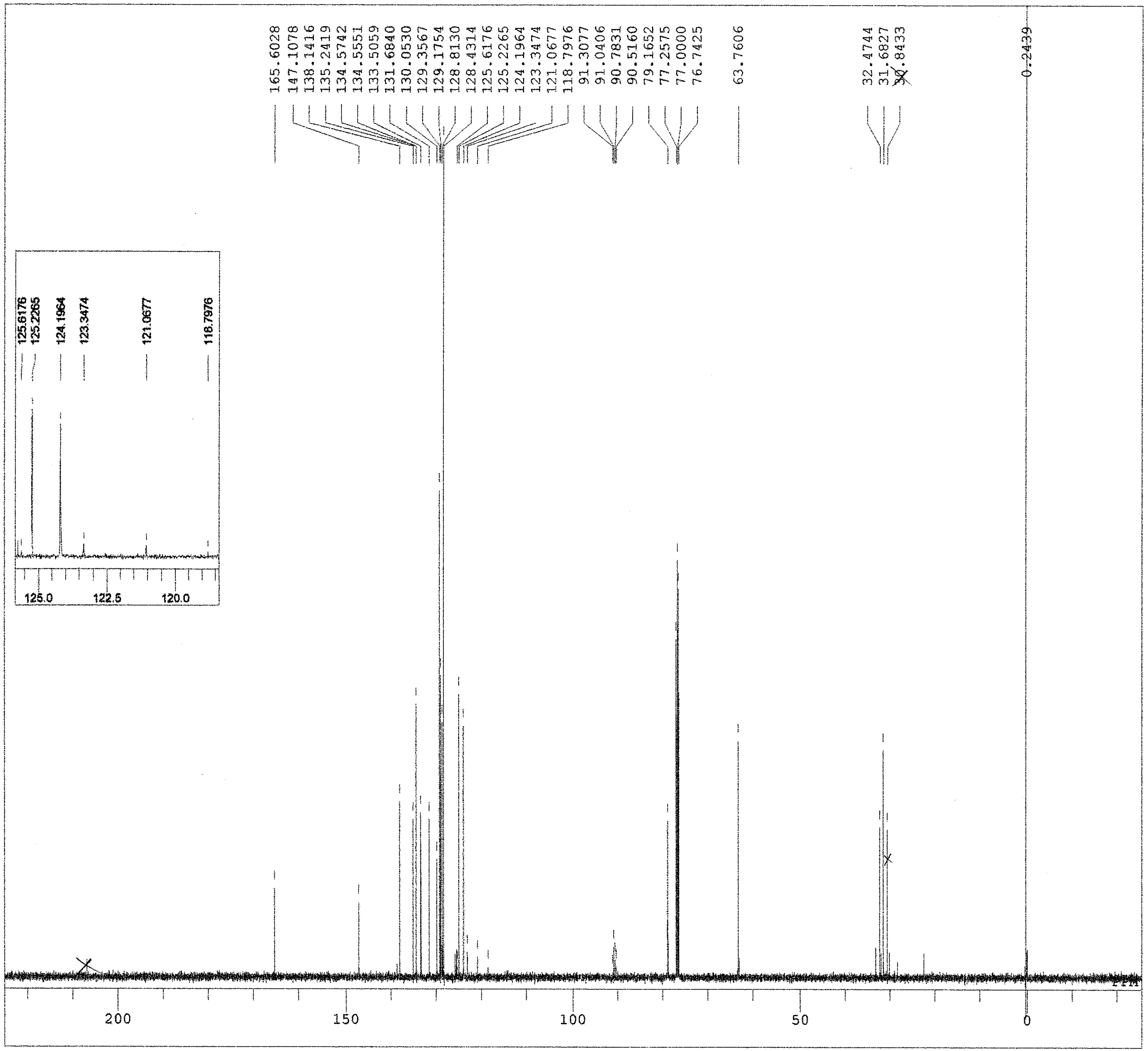

DFILE	ozawa07-034_13C.als
COMNT	2-TMS-Ph, Bn
DATIM	2015-03-09 10:44:27
OBNUC	13C
EXMOD	carbon.jxp
OBFRQ	125.77 MHz
OBSET	7.87 KHz
OBFIN	4.21 Hz
POINT	26214
FREQU	31446.54 Hz
SCANS	368
ACQTM	0.8336 sec
PD	2.0000 sec
PW1	3.40 usec
IRNUC	1H
CTEMP	25.0 c
SLVNT	CDCL3
EXREF	77.00 ppm
BF	0.12 Hz
RGAIN	60

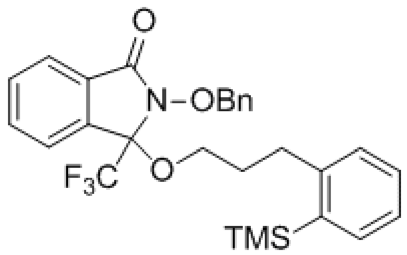

S4y

2-TMS-Ph, Bn

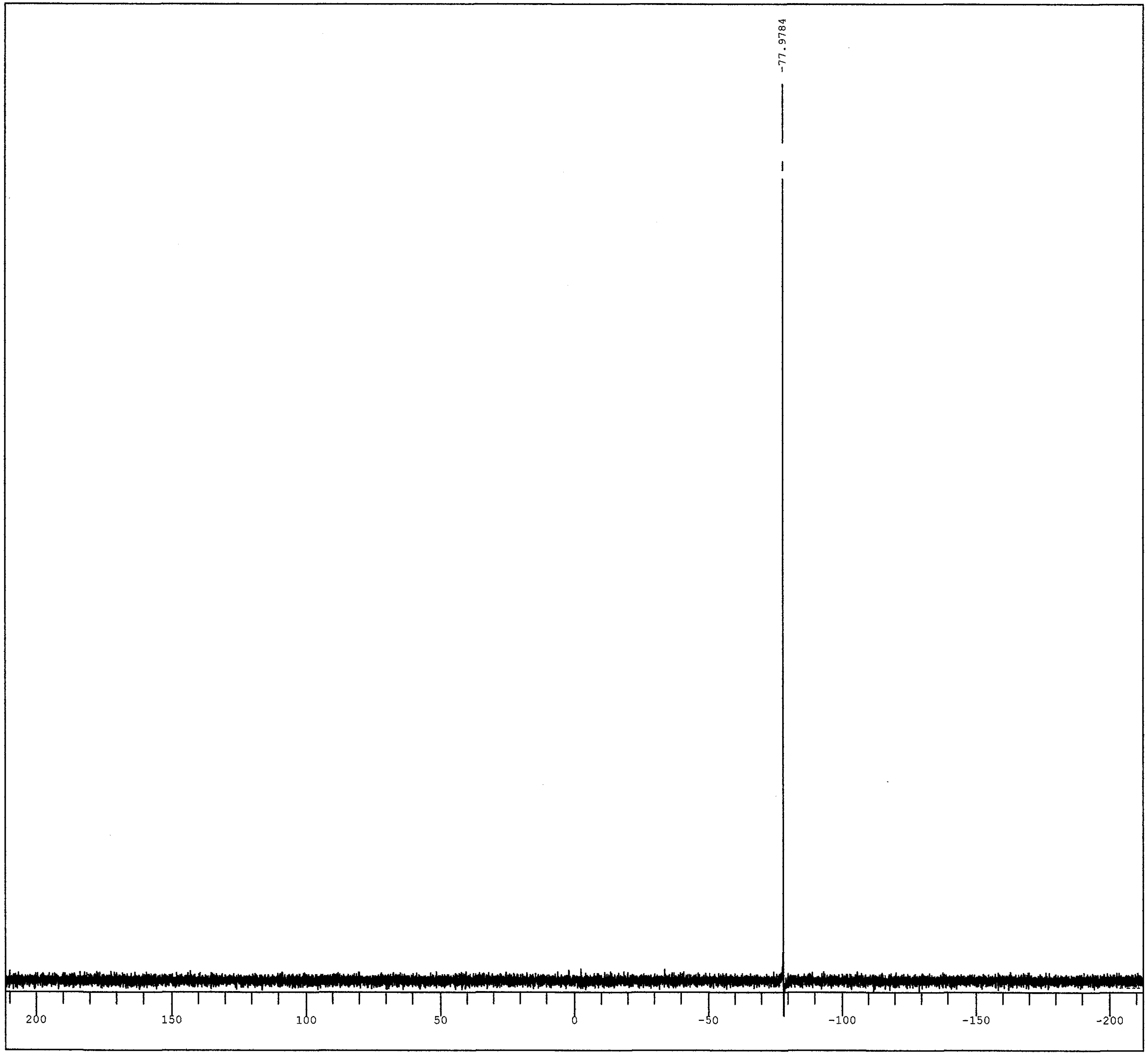


```
DFILE  ozawa07-034_19F.jdf
COMNT  2-TMS-Ph, Bn
DATIM  21-03-2015 12:09:18
OBNUC  19F
EXMOD  proton.jxp
OBFRQ        368.64 MHz
OBSET          7.63 KHz
OBFIN          2.85 Hz
POINT         16384
FREQU     195312.50 Hz
SCANS             4
ACQTM        0.0839 sec
PD           5.0000 sec
PW1            3.90 usec
IRNUC  19F
CTEMP          25.1 c
SLVNT  CDCL3
EXREF       -164.90 ppm
BF             0.12 Hz
RGAIN            50
```

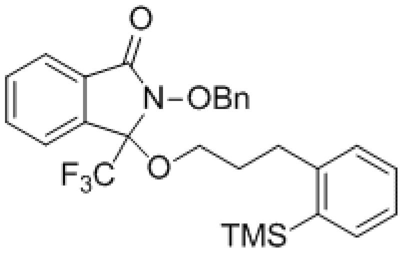

S4y

2-TMS-Ph, OH

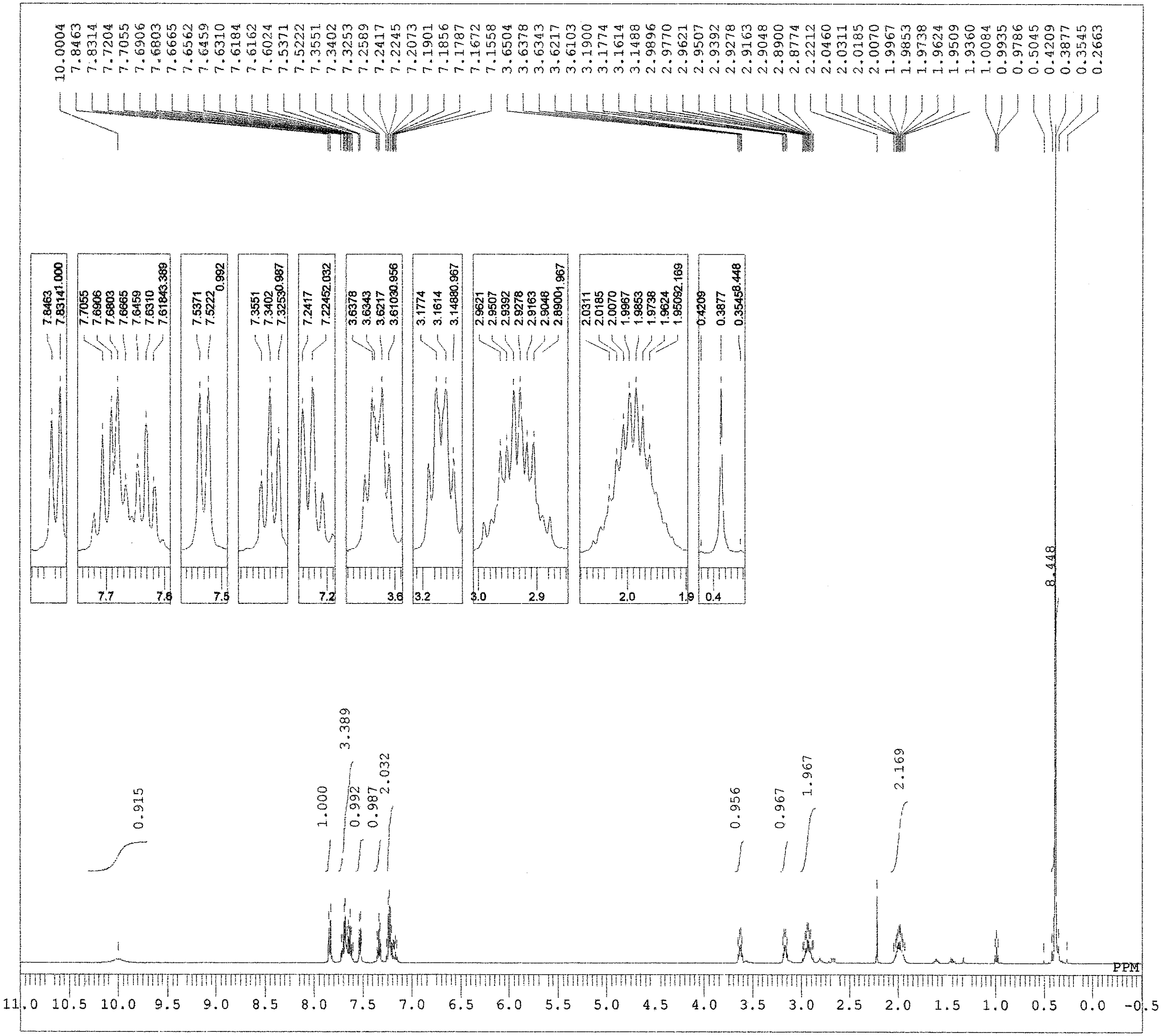

DFILE ozawa07-035_1H.jdf
COMNT 2-TMS-Ph, OH
DATIM 2015-03-24 08:49:33
OBNUC 1H
EXMOD proton.jxp
OBFRQ 500.16 MHz
OBSET 2.41 KHz
OBFIN 6.01 Hz
POINT 16384
FREQU 9384.38 Hz
SCANS 4
ACQTM 1.7459 sec
PD 5.0000 sec
PW1 5.55 usec
IRNUC 1H
CTEMP 27.1 c
SLVNT CDCL3
EXREF 7.26 ppm
BF 0.20 Hz
RGAIN 20

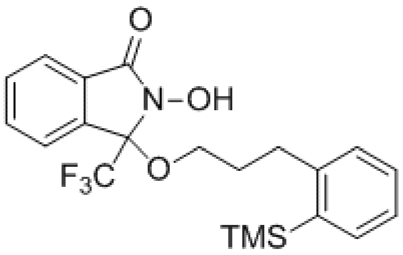

1y

2-TMS-Ph, OH

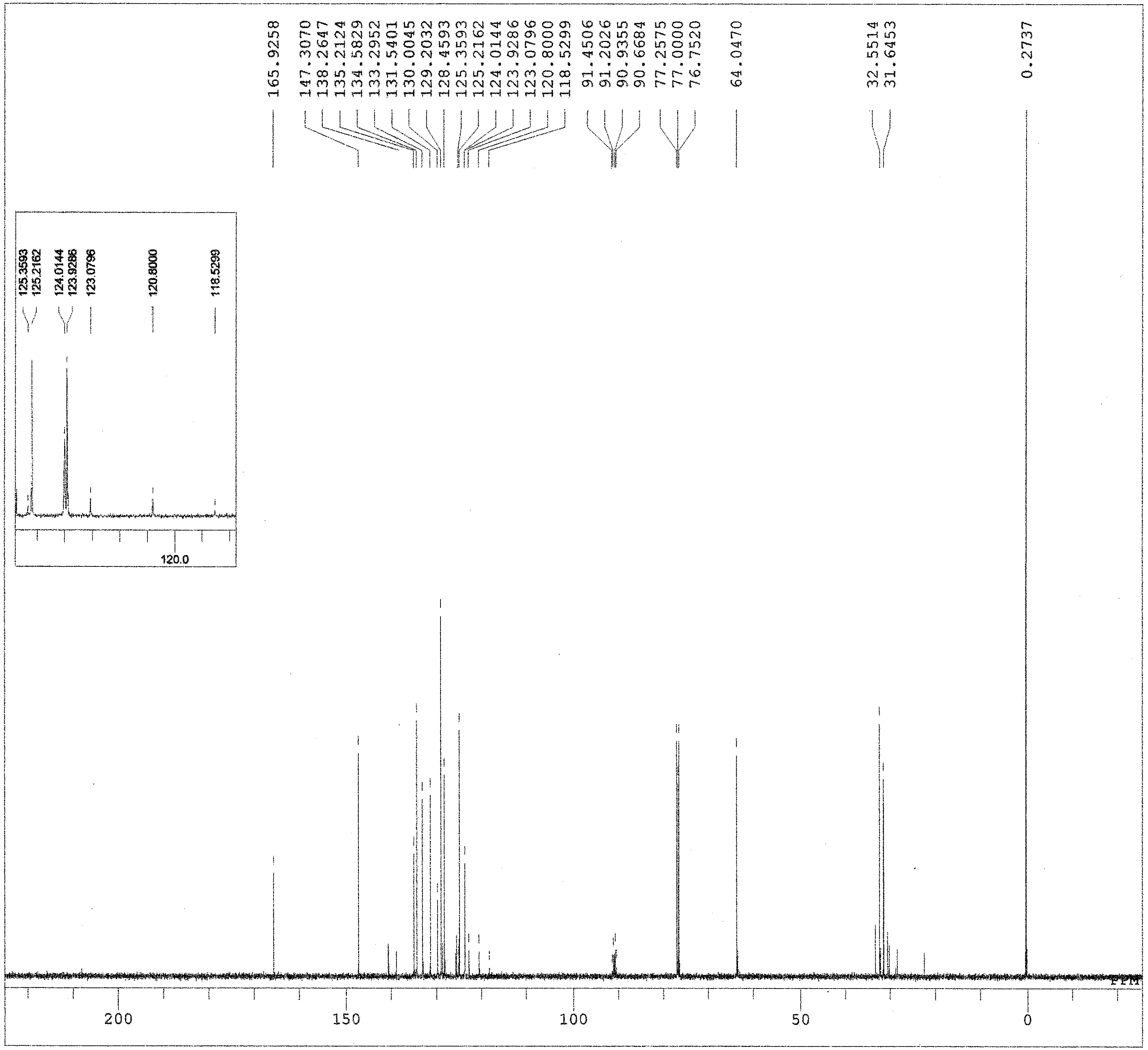


```
DFILE   ozawa07-035_13C.jdf
COMNT   2-TMS-Ph, OH
DATIM   2015-03-24 08:50:36
OBNUC   13C
EXMOD   carbon.jxp
OBFRQ        125.77 MHz
OBSET          7.87 KHz
OBFIN          4.21 Hz
POINT        32767
FREQU      39308.18 Hz
SCANS          240
ACQTM        0.8336 sec
PD           2.0000 sec
PW1            3.40 usec
IRNUC   1H
CTEMP          27.2 c
SLVNT   CDCL3
EXREF         77.00 ppm
BF             0.20 Hz
RGAIN           60
```

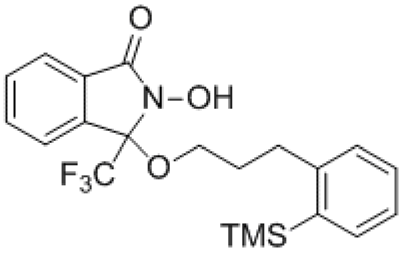

1y

2-TMS-Ph, OH

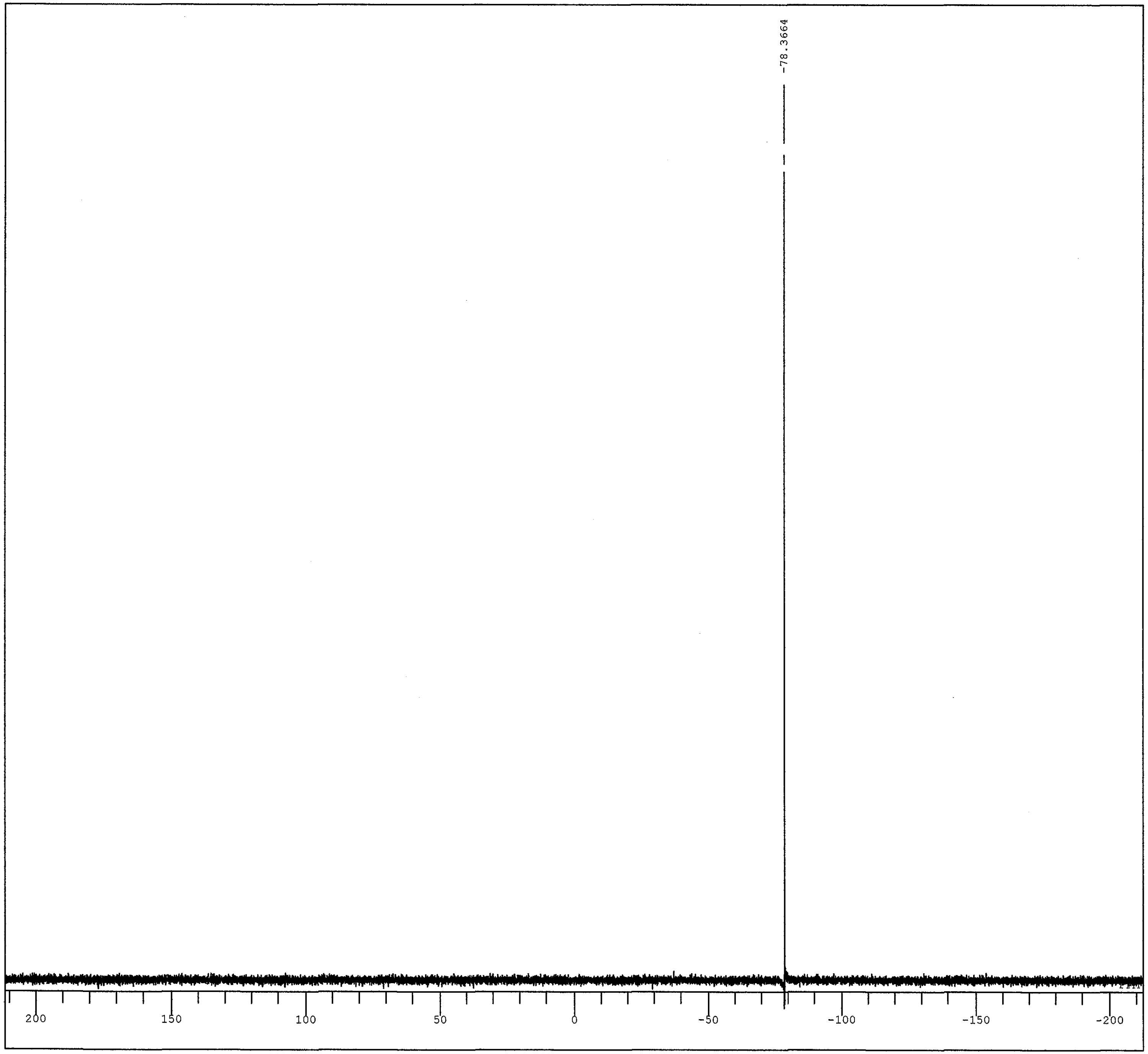

DFILE ozawa07-035_19F.jdf
COMNT 2-TMS-Ph, OH
DATIM 21-03-2015 11:43:48
OBNUC 19F
EXMOD proton.jxp
OBFRQ 368.64 MHz
OBSET 7.63 KHz
OBFIN 2.85 Hz
POINT 16384
FREQU 195312.50 Hz
SCANS 4
ACQTM 0.0839 sec
PD 5.0000 sec
PW1 3.90 usec
IRNUC 19F
CTEMP 25.1 c
SLVNT CDCL3
EXREF -164.90 ppm
BF 0.12 Hz
RGAIN 50

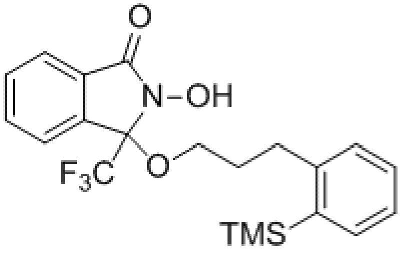

1y

2-TMS-Ph, [0]

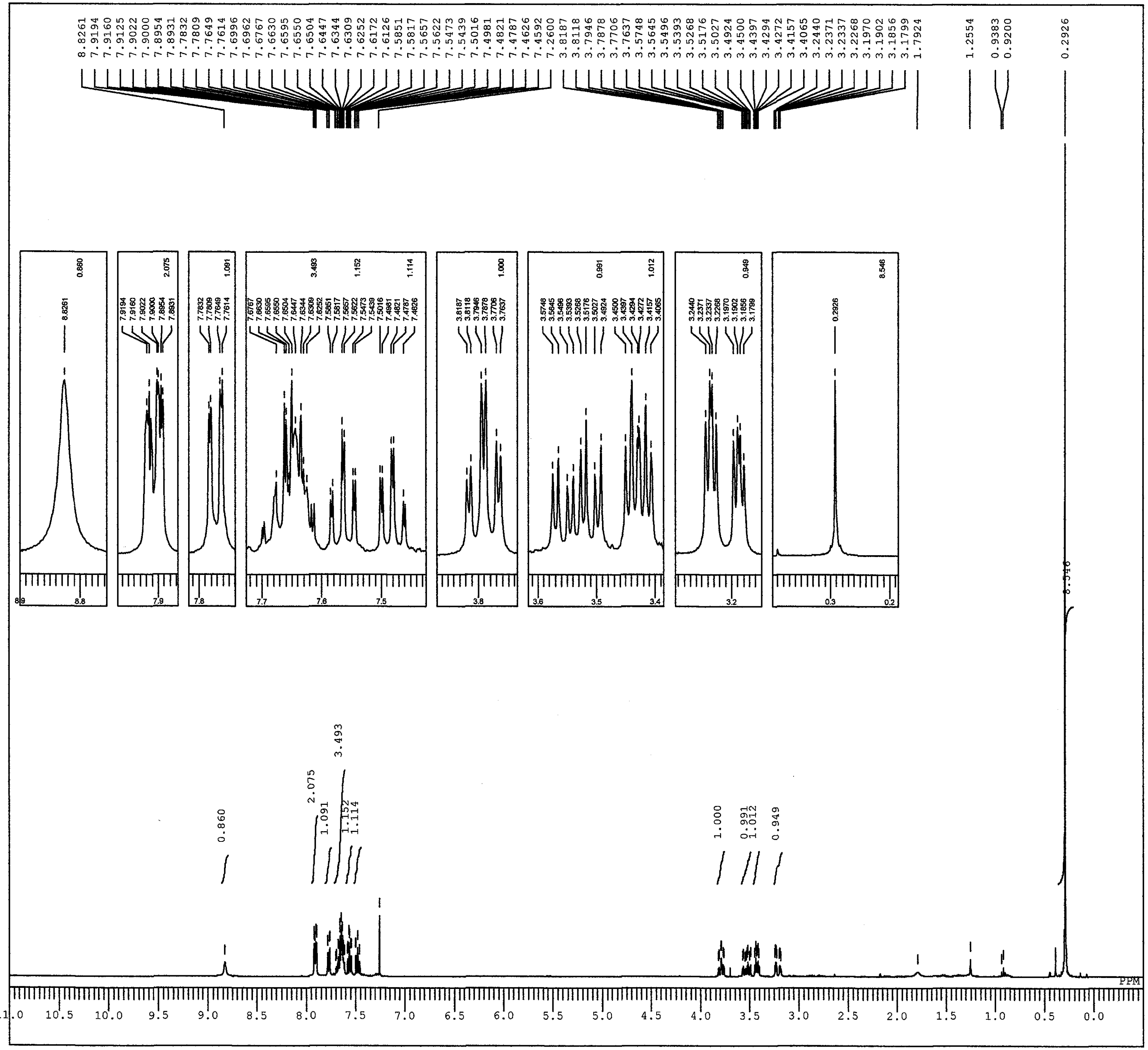

DFILE ozawa07-038_1H.jdf
COMNT 2-TMS-Ph, [0]
DATIM 21-03-2015 08:26:35
OBNUC 1H
EXMOD proton.jxp
OBFRQ 391.78 MHz
OBSET 8.51 KHz
OBFIN 3.34 Hz
POINT 16384
FREQU 7348.62 Hz
SCANS 4
ACQTM 2.2295 sec
PD 5.0000 sec
PW1 4.99 usec
IRNUC 1H
CTEMP 25.1 c
SLVNT CDCL3
EXREF 7.26 ppm
BF 0.12 Hz
RGAIN 38

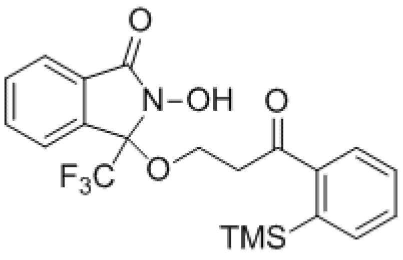

2y

2-TMS-Ph, [O]

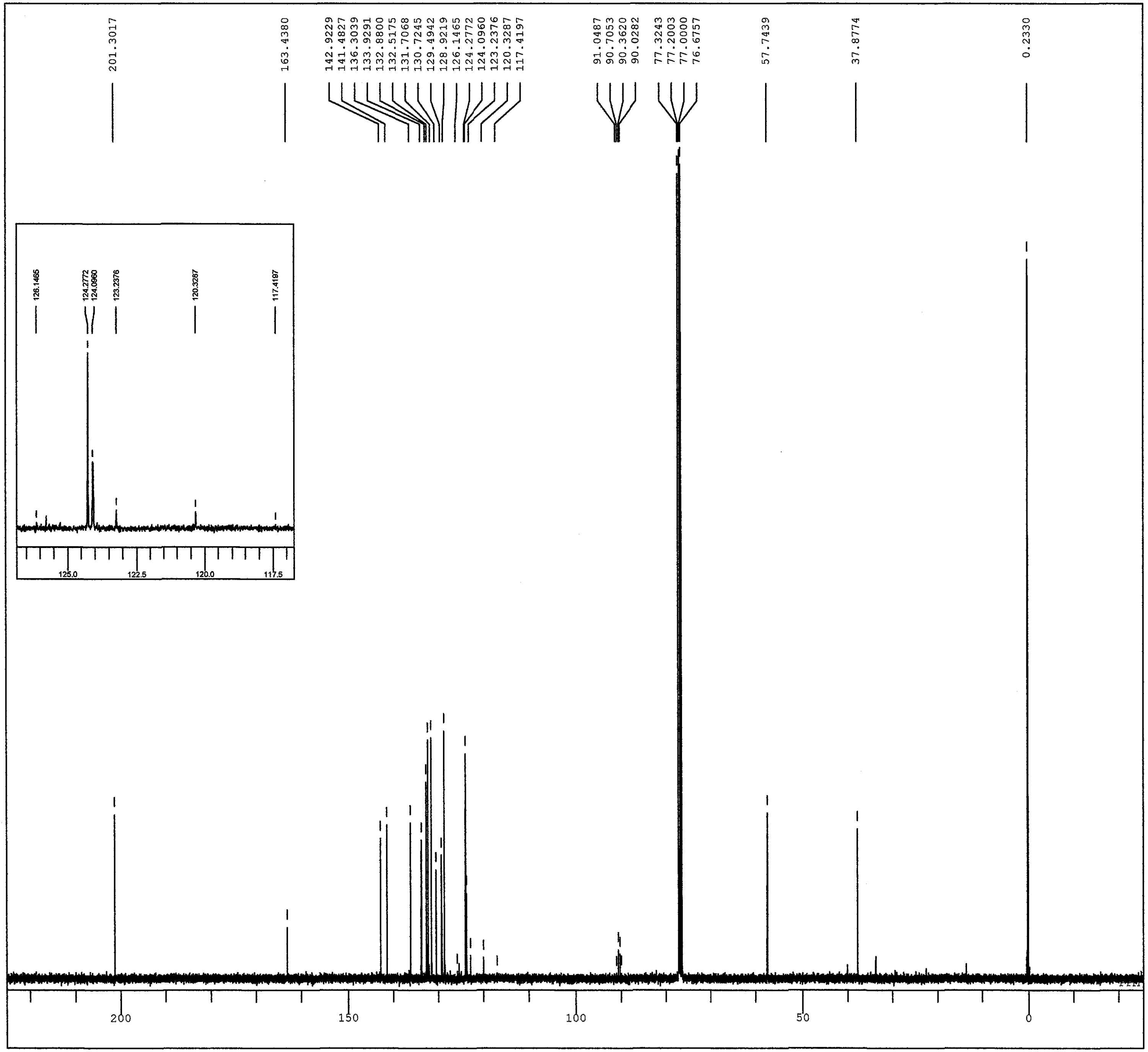

DFILE ozawa07-038_13C.jdf
COMNT 2-TMS-Ph, [O]
DATIM 21-03-2015 08:31:52
OBNUC 13C
EXMOD carbon.jxp
OBFRQ 98.52 MHz
OBSET 4.64 KHz
OBFIN 8.74 Hz
POINT 32767
FREQU 30788.18 Hz
SCANS 2360
ACQTM 1.0643 sec
PD 2.0000 sec
PW1 3.16 usec
IRNUC 1H
CTEMP 25.2 c
SLVNT CDCL3
EXREF 77.00 ppm
BF 0.12 Hz
RGAIN 60

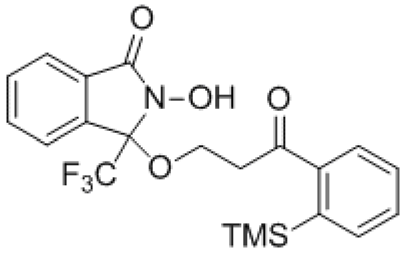

2y

2-TMS-Ph, [O]

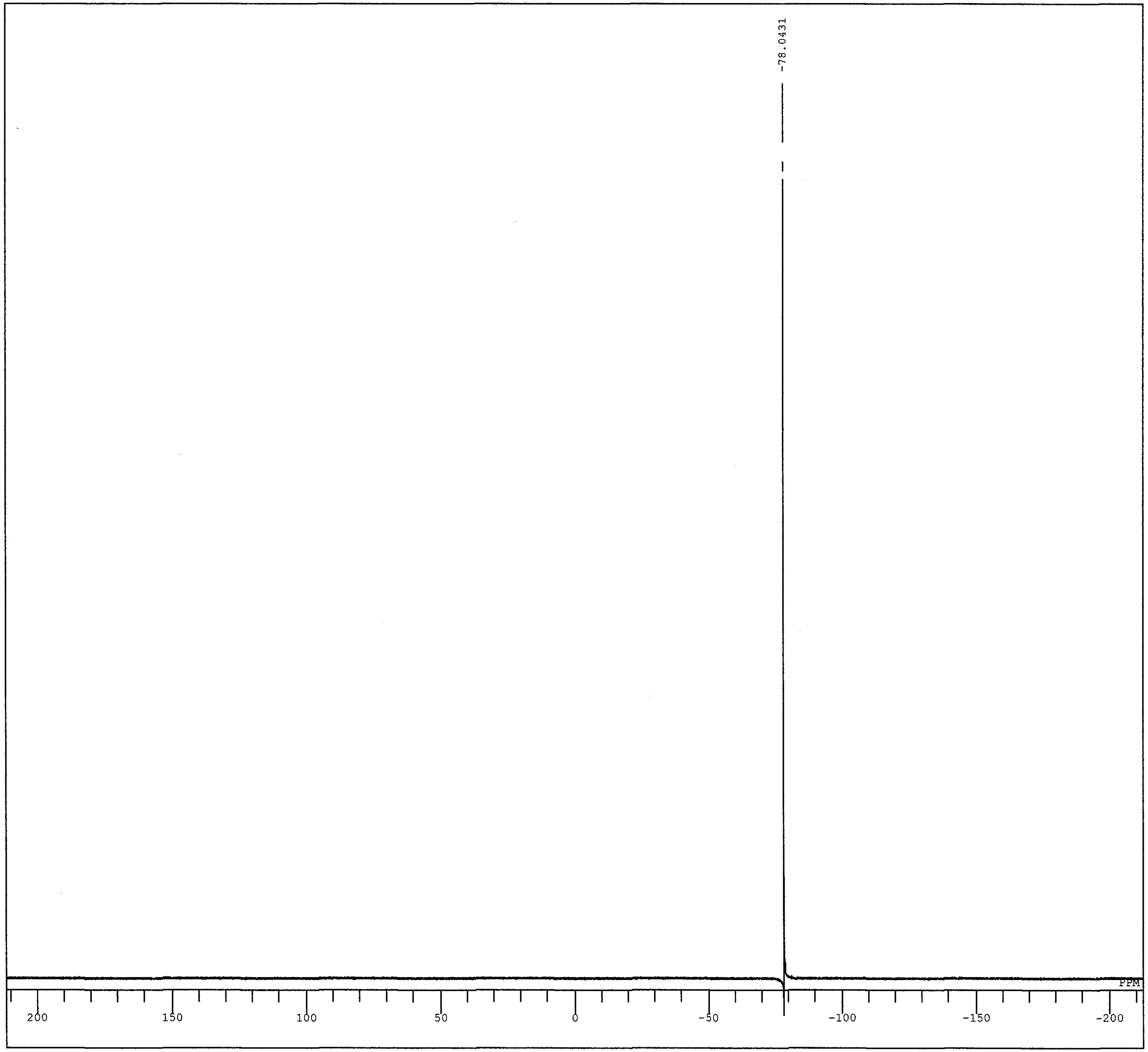


```
DFILE  ozawa07-038_19F.jdf
COMNT  2-TMS-Ph, [O]
DATIM  21-03-2015 08:24:23
OBNUC  19F
EXMOD  proton.jxp
OBFRQ         368.64 MHz
OBSET           7.63 KHz
OBFIN           2.85 Hz
POINT          16384
FREQU      195312.50 Hz
SCANS              4
ACQTM         0.0839 sec
PD            5.0000 sec
PW1             3.90 usec
IRNUC  19F
CTEMP           25.1 c
SLVNT  CDCL3
EXREF        -164.90 ppm
BF              0.12 Hz
RGAIN             50
```

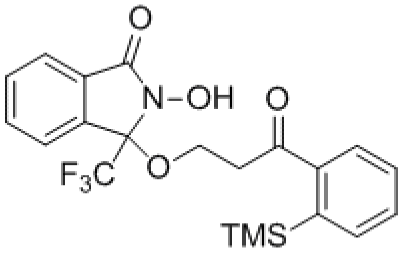

2y

PhEt, Bn

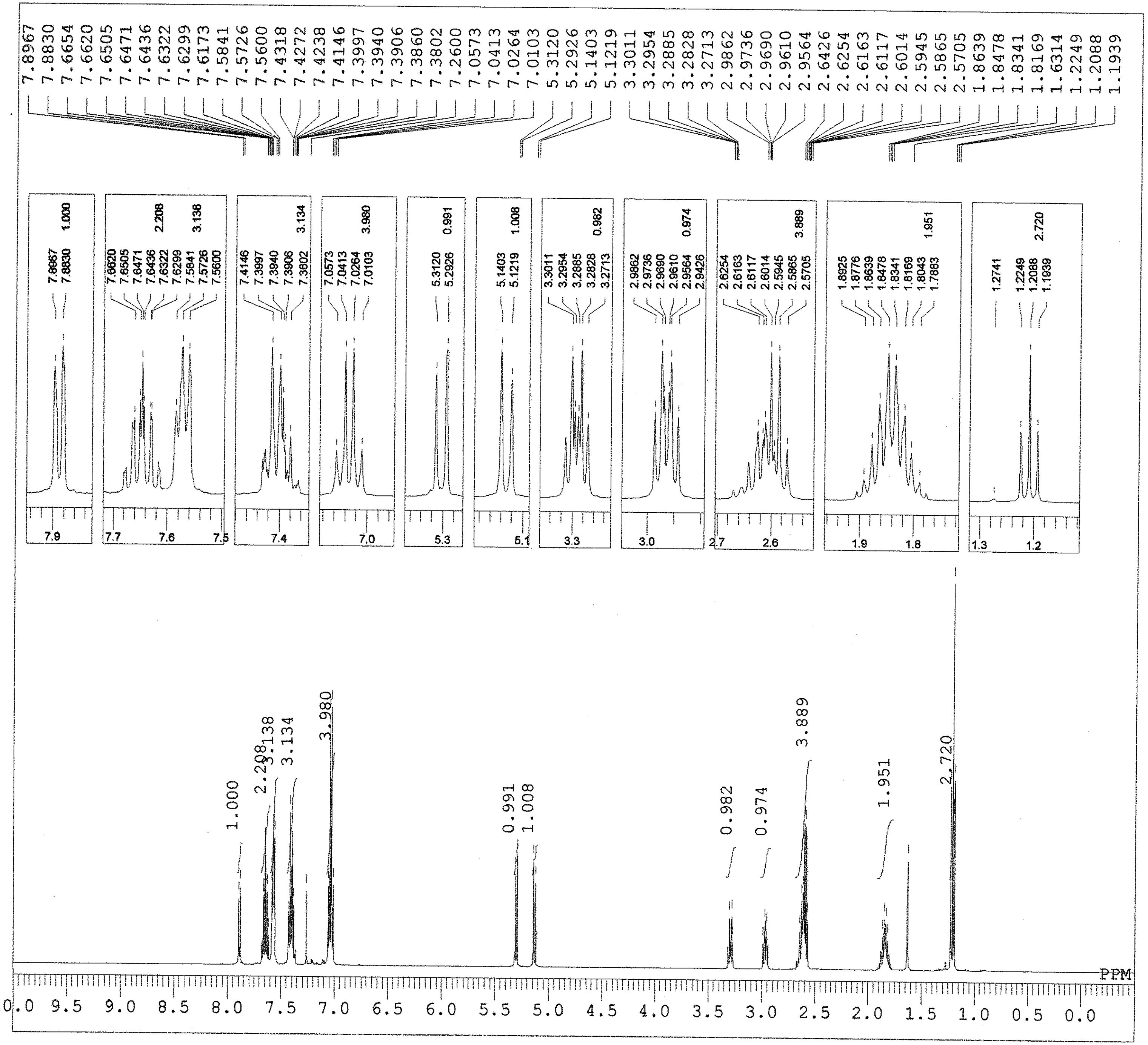

DFILE	ozawa05-050_1H.jdf
COMNT	PhEt, Bn
DATIM	2014-08-15 12:19:02
OBNUC	1H
EXMOD	proton.jxp
OBFRQ	500.16 MHz
OBSET	2.41 KHz
OBFIN	6.01 Hz
POINT	16384
FREQU	9384.38 Hz
SCANS	4
ACQTM	1.7459 sec
PD	5.0000 sec
PW1	5.55 usec
IRNUC	1H
CTEMP	25.7 c
SLVNT	CDCL3
EXREF	7.26 ppm
BF	0.12 Hz
RGAIN	34

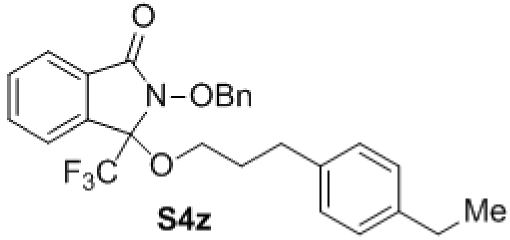

S4z

PhEt, Bn

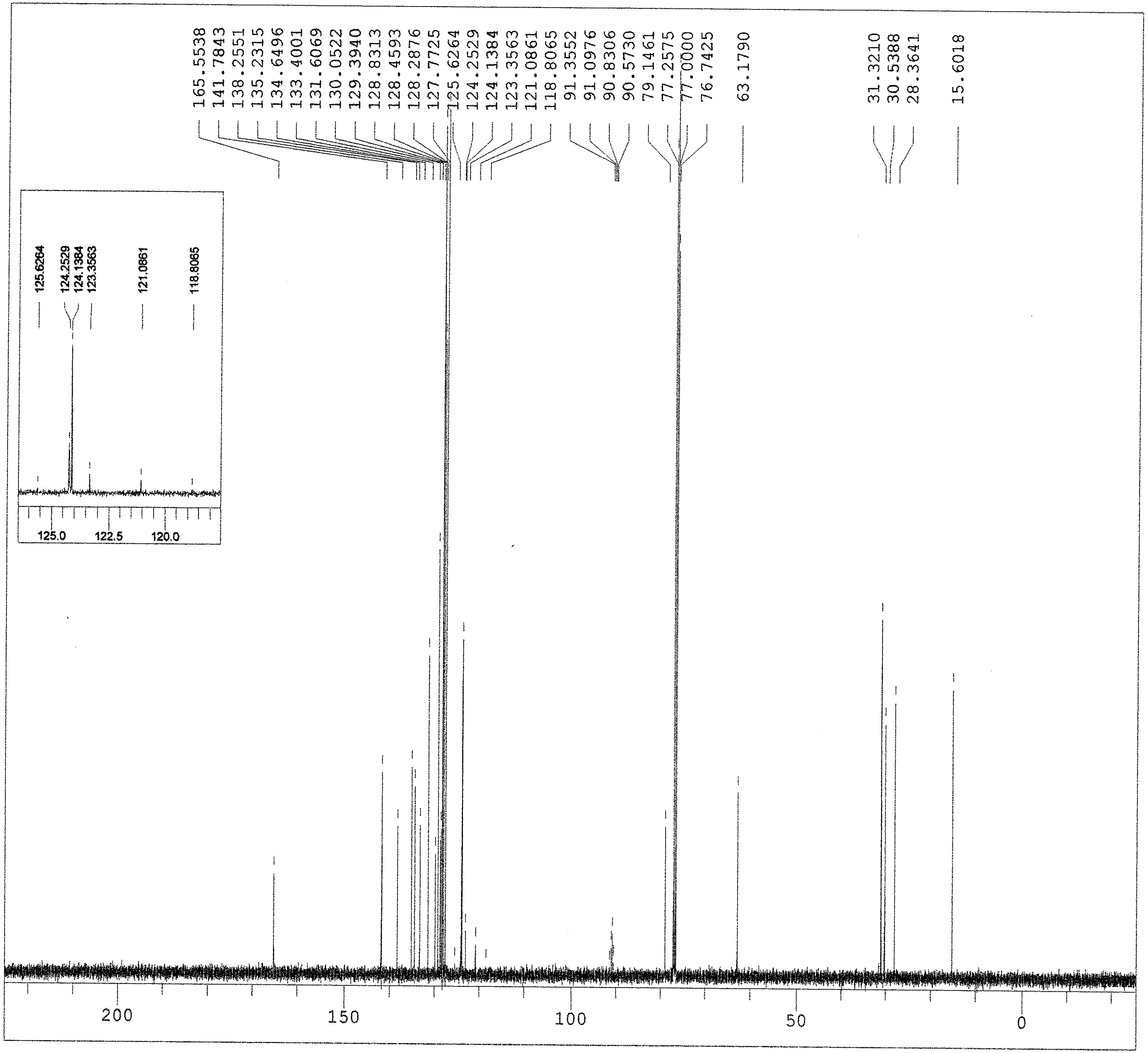

DFILE	ozawa05-050_13C_copy2-1-1.jdf
COMNT	PhEt, Bn
DATIM	2014-08-15 12:20:06
OBNUC	13C
EXMOD	carbon.jxp
OBFRQ	125.77 MHz
OBSET	7.87 KHz
OBFIN	4.21 Hz
POINT	32767
FREQU	39308.18 Hz
SCANS	444
ACQTM	0.0000 sec
PD	3.0000 sec
PW1	3.40 usec
IRNUC	1H
CTEMP	26.2 c
SLVNT	CDCL3
EXREF	77.00 ppm
BF	0.12 Hz
RGAIN	60

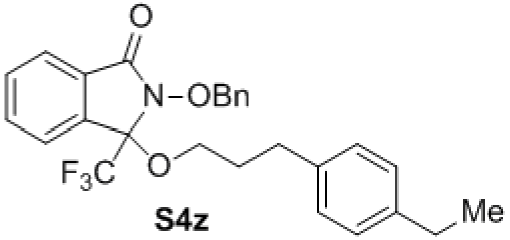

S4z

Ph-Et

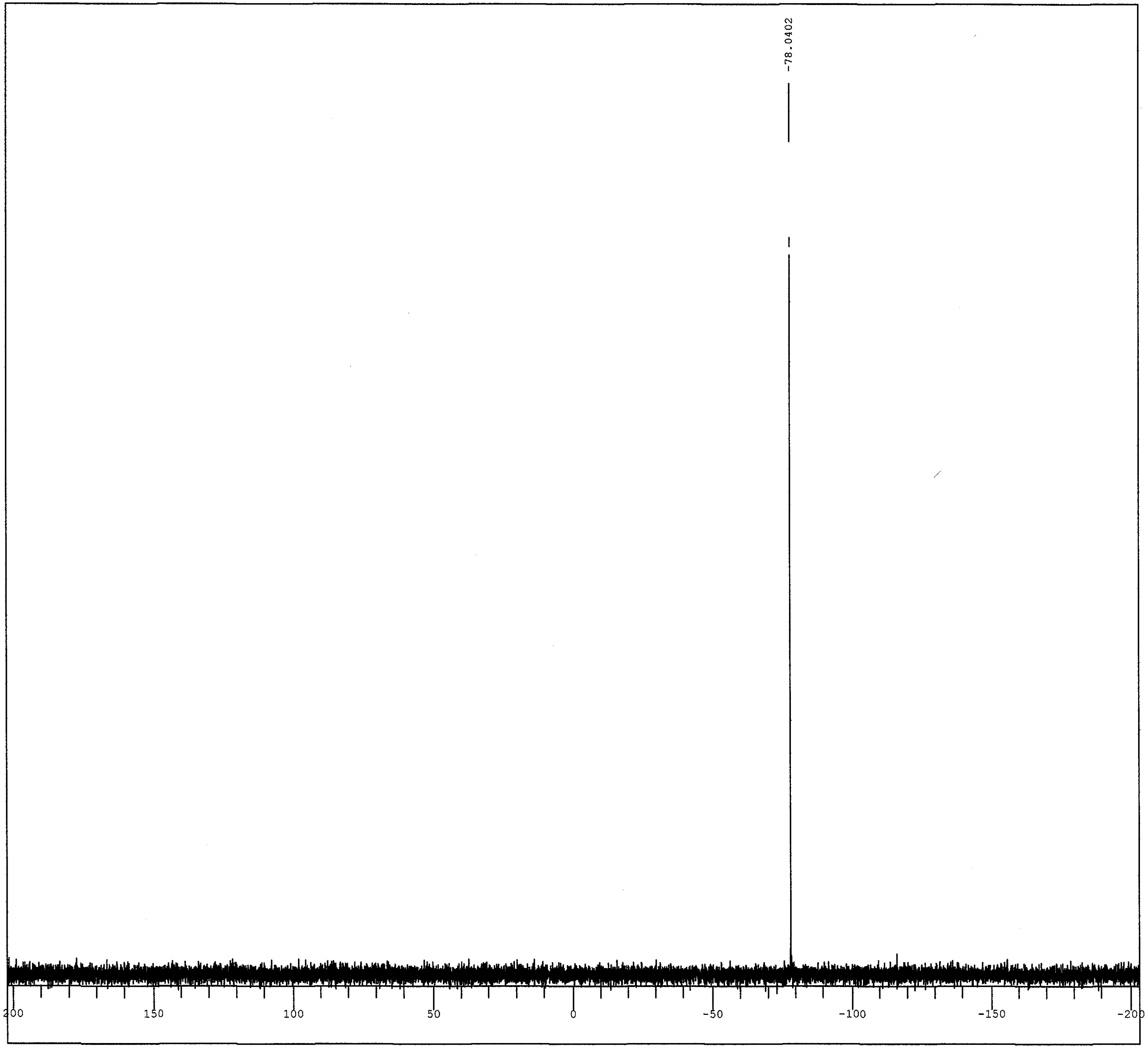

DFILE ozawa05-050_19F.jdf
COMNT Ph-Et
DATIM 14-06-2014 14:47:32
OBNUC 19F
EXMOD proton.jxp
OBFRQ 368.64 MHz
OBSET 7.63 KHz
OBFIN 2.85 Hz
POINT 16384
FREQU 186567.17 Hz
SCANS 4
ACQTM 0.0878 sec
PD 5.0000 sec
PW1 3.90 usec
IRNUC 19F
CTEMP 23.6 c
SLVNT CDCL3
EXREF -164.90 ppm
BF 0.12 Hz
RGAIN 56

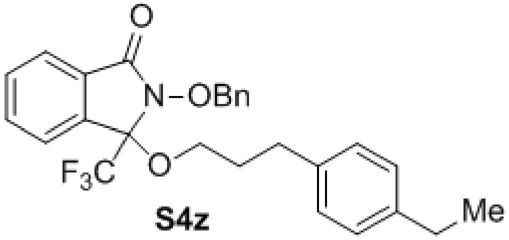

S4z

Ph-Et

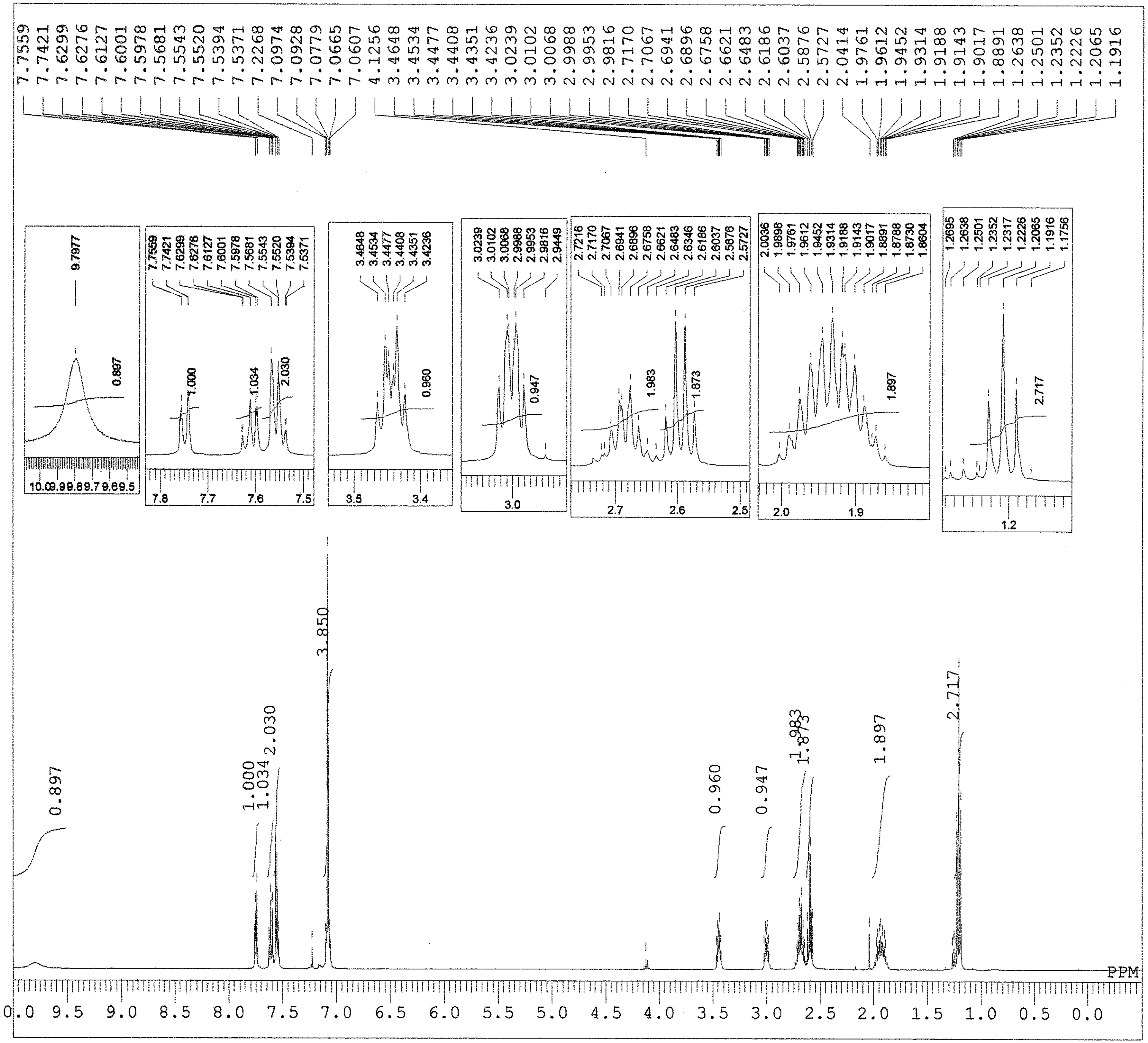

DFILE	ozawa05-055_1H.jdf
COMNT	Ph-Et
DATIM	2014-06-16 19:48:40
OBNUC	1H
EXMOD	proton.jxp
OBFRQ	500.16 MHz
OBSET	2.41 KHz
OBFIN	6.01 Hz
POINT	16384
FREQU	9384.38 Hz
SCANS	4
ACQTM	1.7459 sec
PD	5.0000 sec
PW1	5.55 usec
IRNUC	1H
CTEMP	25.7 c
SLVNT	CDCL3
EXREF	7.26 ppm
BF	0.12 Hz
RGAIN	28

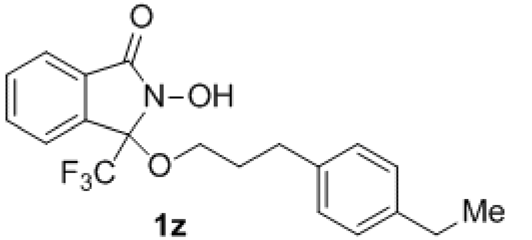

1z

Ph-Et

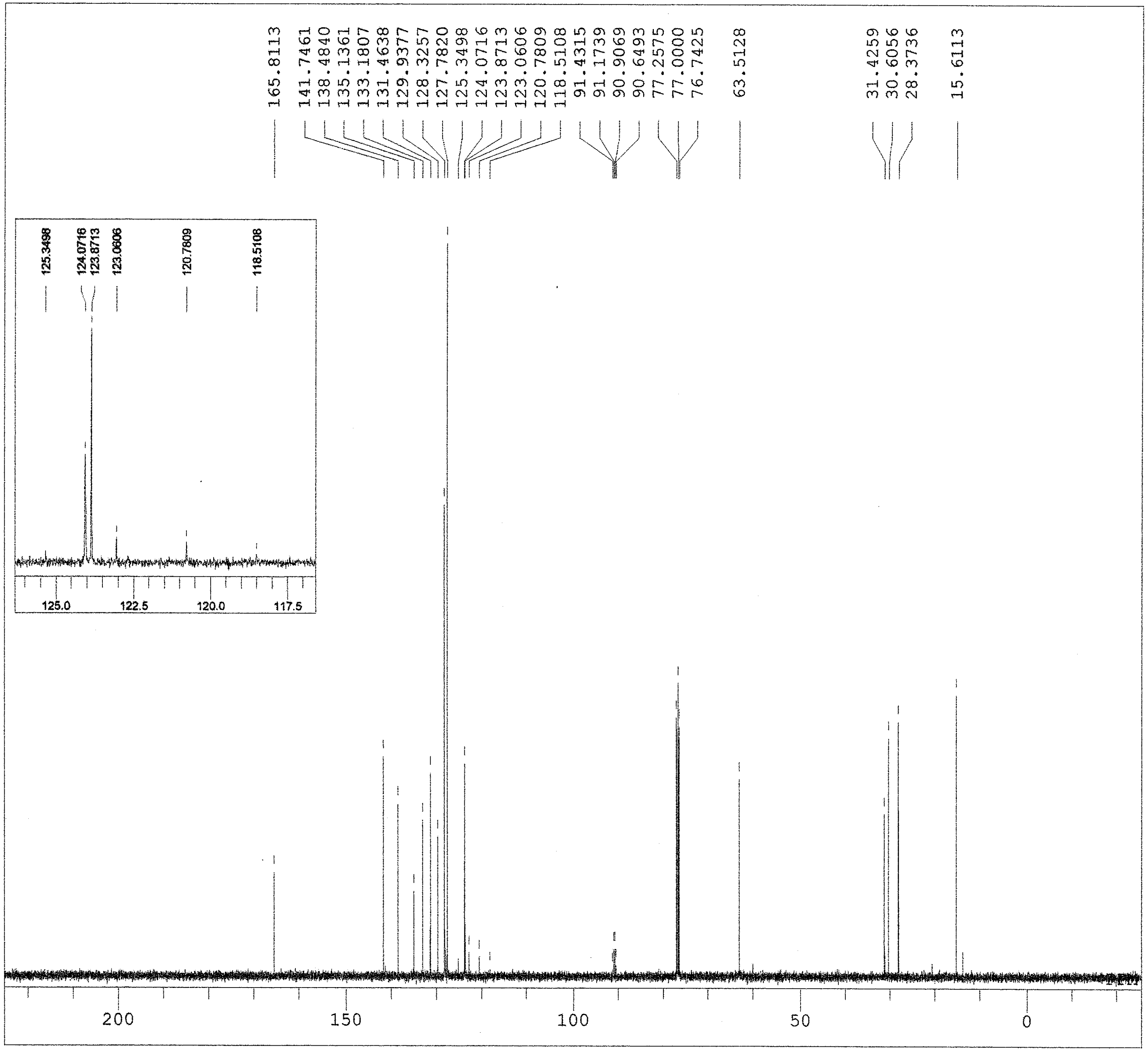

DFILE	ozawa05-055_13C.jdf
COMNT	Ph-Et
DATIM	2014-06-16 19:49:44
OBNUC	13C
EXMOD	carbon.jxp
OBFRQ	125.77 MHz
OBSET	7.87 KHz
OBFIN	4.21 Hz
POINT	32767
FREQU	39308.18 Hz
SCANS	108
ACQTM	0.8336 sec
PD	3.0000 sec
PW1	3.40 usec
IRNUC	1H
CTEMP	26.0 c
SLVNT	CDCL3
EXREF	77.00 ppm
BF	0.12 Hz
RGAIN	60

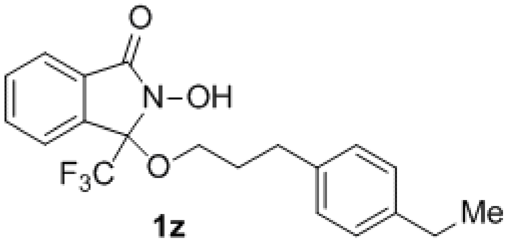

1z

PhEt, OH

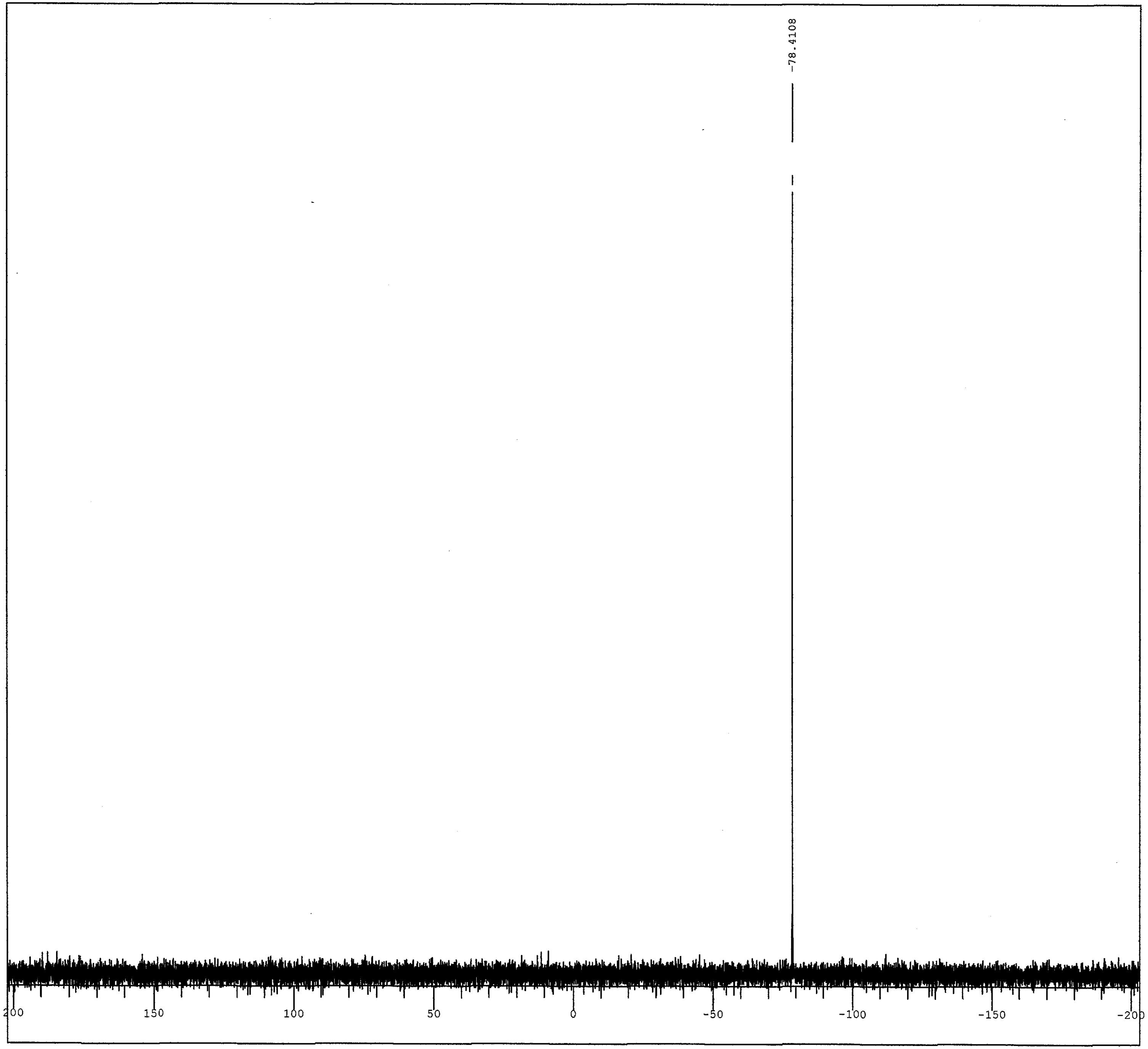


```
DFILE  ozawa05-055_19F.jdf
COMNT  PhEt, OH
DATIM  15-08-2014 20:04:41
OBNUC  19F
EXMOD  proton.jxp
OBFRQ       368.64 MHz
OBSET         7.63 KHz
OBFIN         2.85 Hz
POINT        16384
FREQU    186567.17 Hz
SCANS            4
ACQTM       0.0878 sec
PD          5.0000 sec
PW1           3.90 usec
IRNUC  19F
CTEMP         24.5 c
SLVNT  CDCL3
EXREF      -164.90 ppm
BF            0.12 Hz
RGAIN           48
```

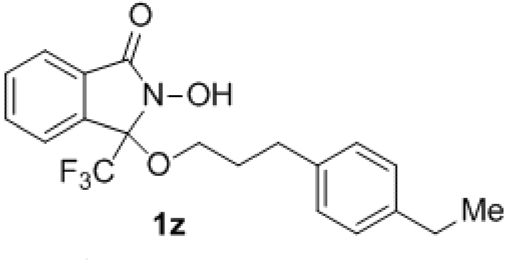

1z

PhEt, [O]

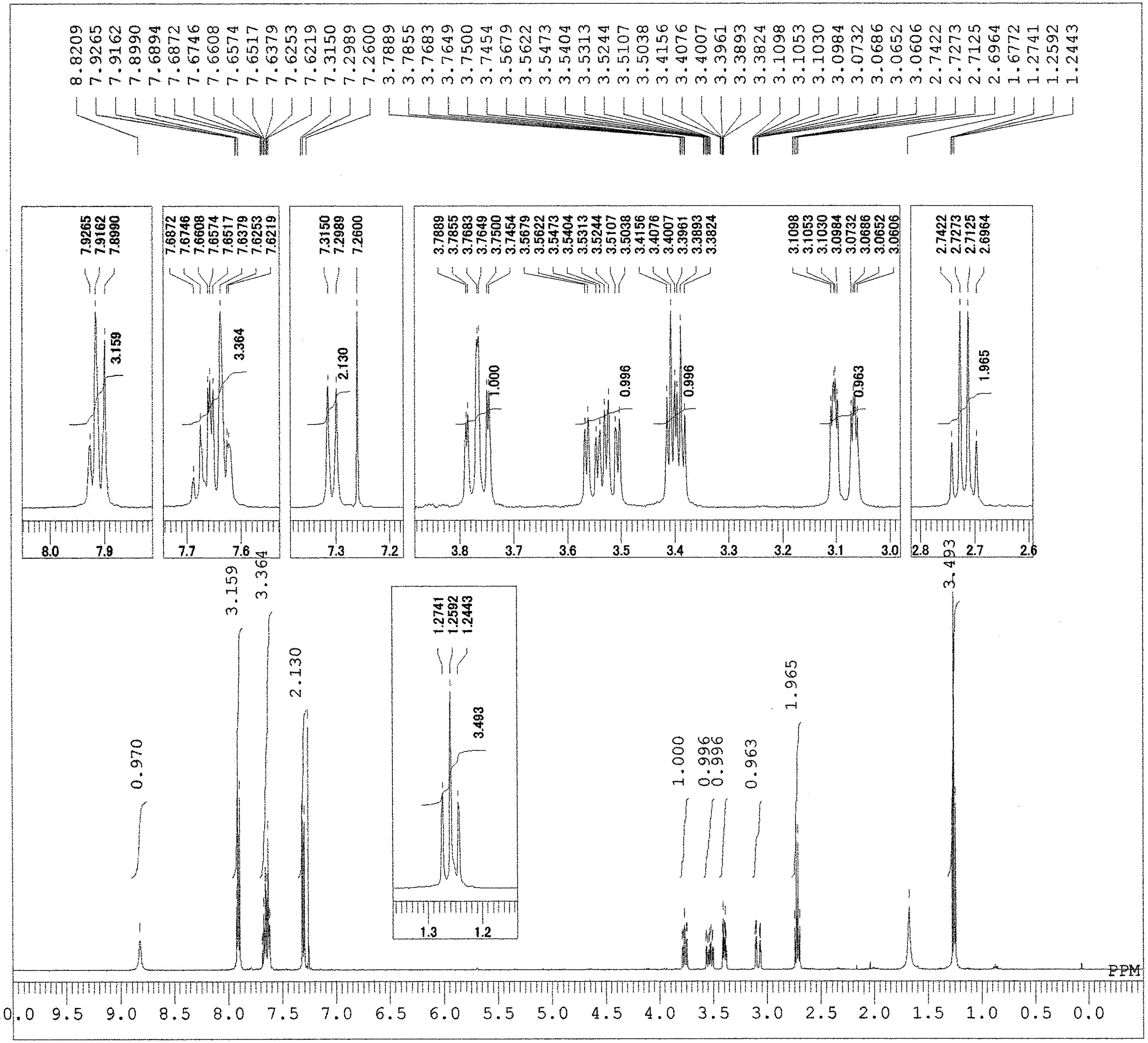

DFILE	ozawa05-097_1H.jdf
COMNT	PhEt, [O]
DATIM	2014-07-09 22:52:48
OBNUC	1H
EXMOD	proton.jxp
OBFRQ	500.16 MHz
OBSET	2.41 KHz
OBFIN	6.01 Hz
POINT	16384
FREQU	9384.38 Hz
SCANS	4
ACQTM	1.7459 sec
PD	5.0000 sec
PW1	5.55 usec
IRNUC	1H
CTEMP	25.8 c
SLVNT	CDCL3
EXREF	7.26 ppm
BF	0.12 Hz
RGAIN	38

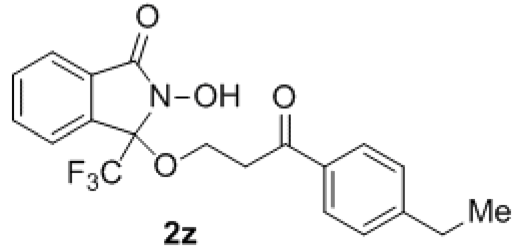

2z

PhEt, [O]

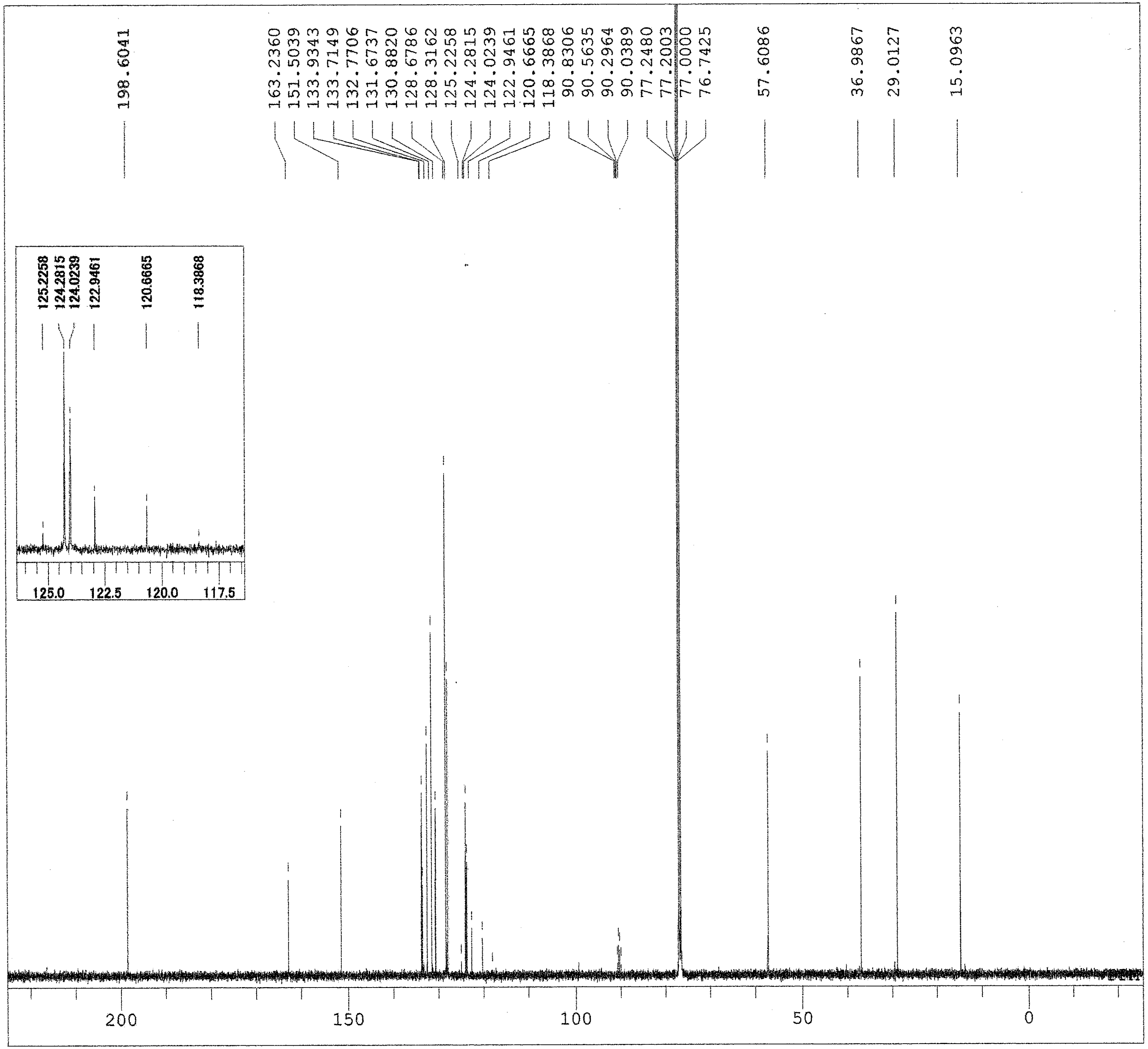

DFILE	ozawa05-097_13C.jdf
COMNT	PhEt, [O]
DATIM	2014-07-09 22:53:52
OBNUC	13C
EXMOD	carbon.jxp
OBFRQ	125.77 MHz
OBSET	7.87 KHz
OBFIN	4.21 Hz
POINT	32767
FREQU	39308.18 Hz
SCANS	9400
ACQTM	0.8336 sec
PD	3.0000 sec
PW1	3.40 usec
IRNUC	1H
CTEMP	26.0 c
SLVNT	CDCL3
EXREF	77.00 ppm
BF	0.12 Hz
RGAIN	60

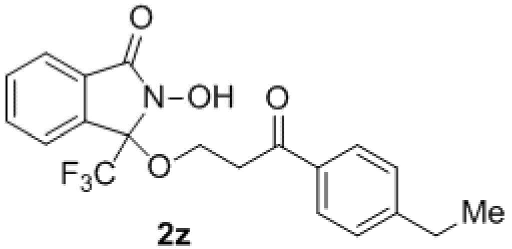

2z

PhEt, [0]

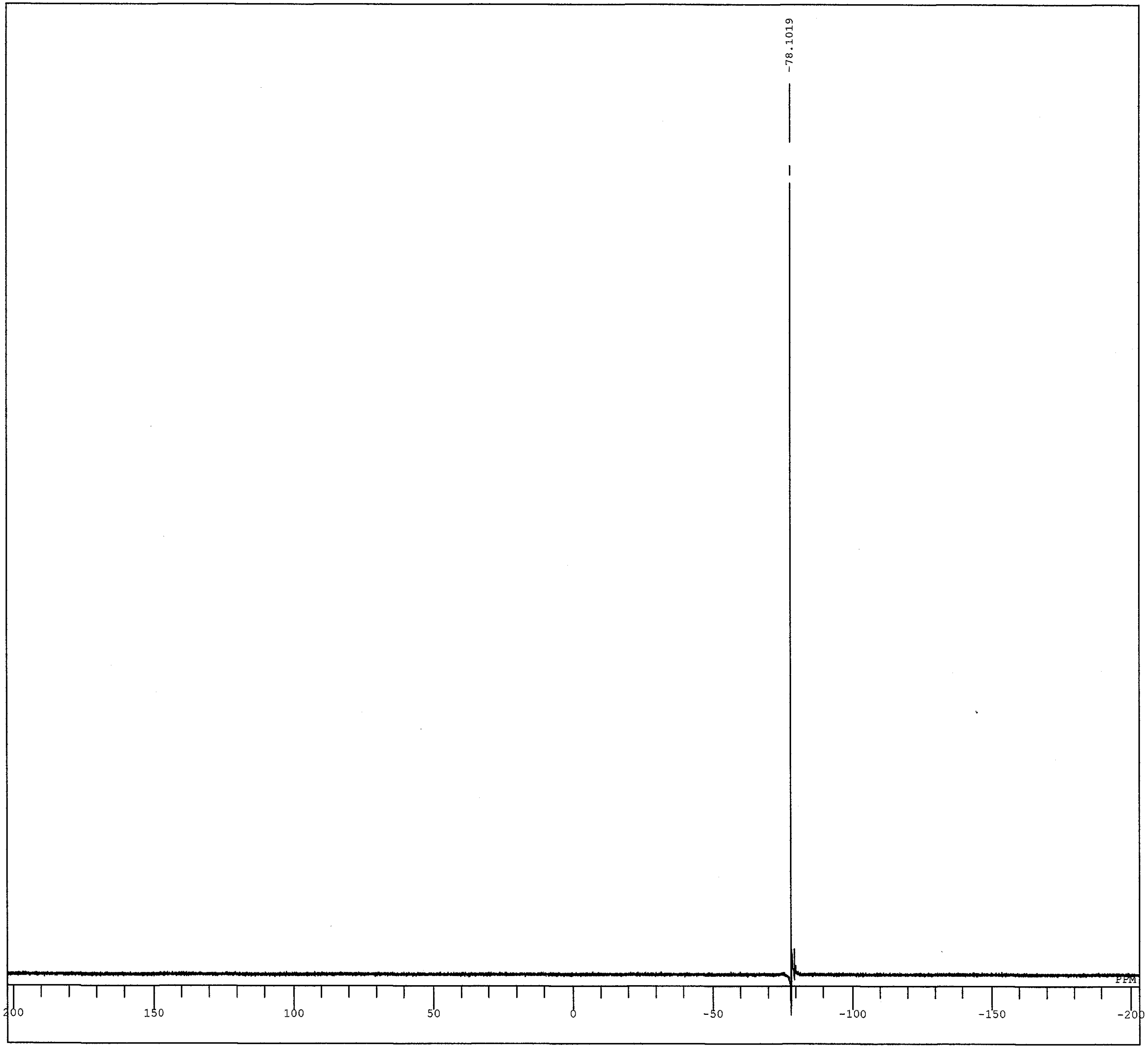

DFILE ozawa05-097_19F.jdf
COMNT PhEt, [O]
DATIM 10-07-2014 14:38:13
OBNUC 19F
EXMOD proton.jxp
OBFRQ 368.64 MHz
OBSET 7.63 KHz
OBFIN 2.85 Hz
POINT 16384
FREQU 186567.17 Hz
SCANS 4
ACQTM 0.0878 sec
PD 5.0000 sec
PW1 3.90 usec
IRNUC 19F
CTEMP 23.7 c
SLVNT CDCL3
EXREF -164.90 ppm
BF 0.12 Hz
RGAIN 40

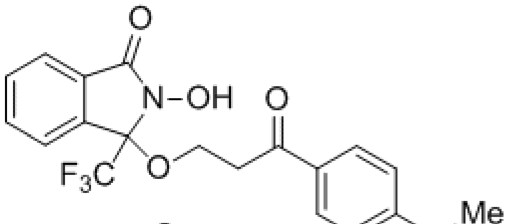

2z

nPr-yne, PMB

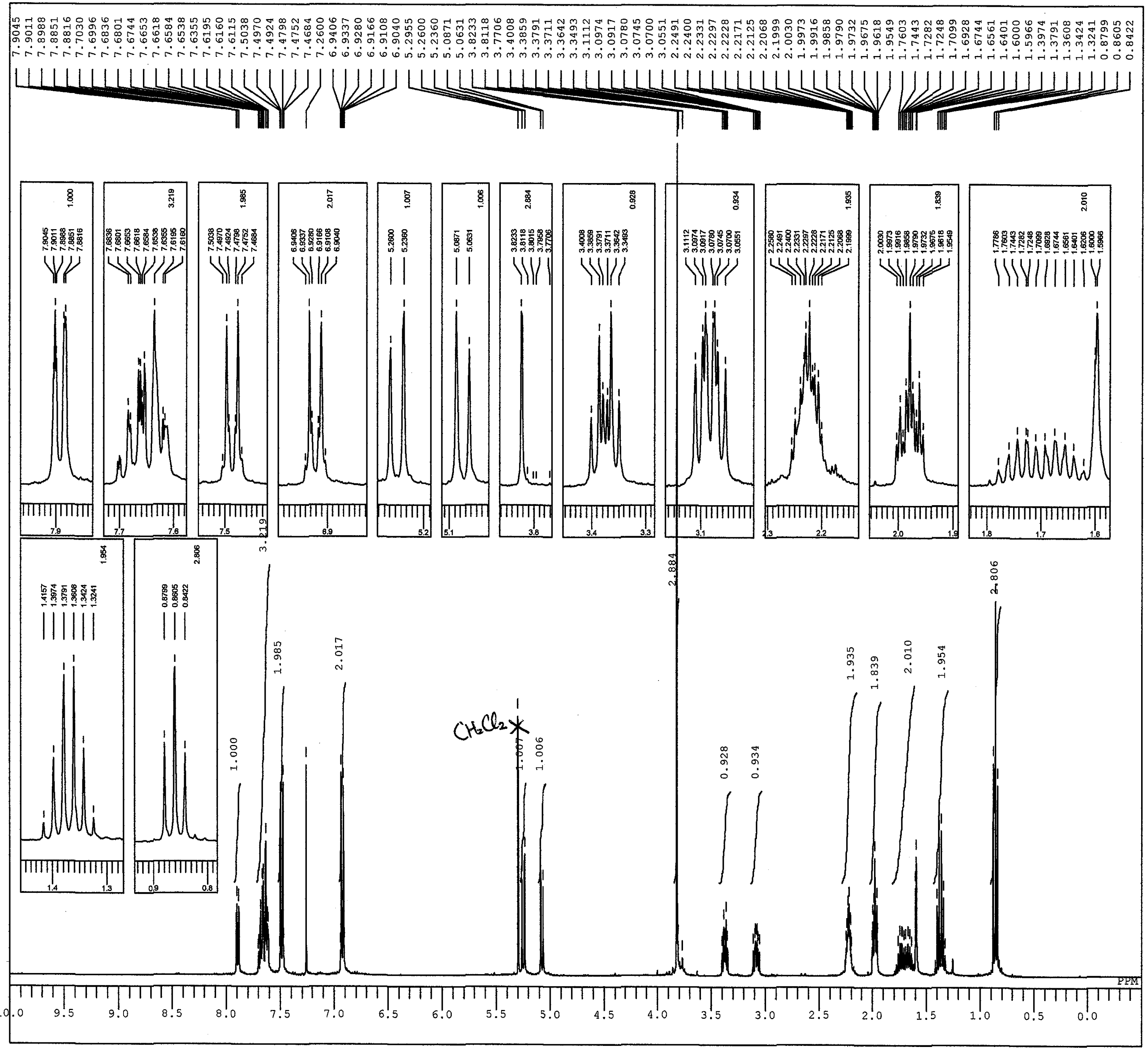

DFILE	ozawa05-127_1_1H.jdf
COMNT	nPr-yne, PMB
DATIM	11-08-2014 20:15:38
OBNUC	1H
EXMOD	proton.jxp
OBFRQ	391.78 MHz
OBSET	8.51 KHz
OBFIN	3.34 Hz
POINT	16384
FREQU	7348.62 Hz
SCANS	8
ACQTM	2.2295 sec
PD	5.0000 sec
PW1	5.25 usec
IRNUC	1H
CTEMP	24.1 c
SLVNT	CDCL3
EXREF	7.26 ppm
BF	0.12 Hz
RGAIN	38

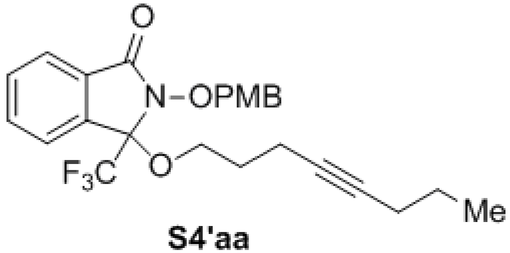

S4'aa

nPr-yne, PMB

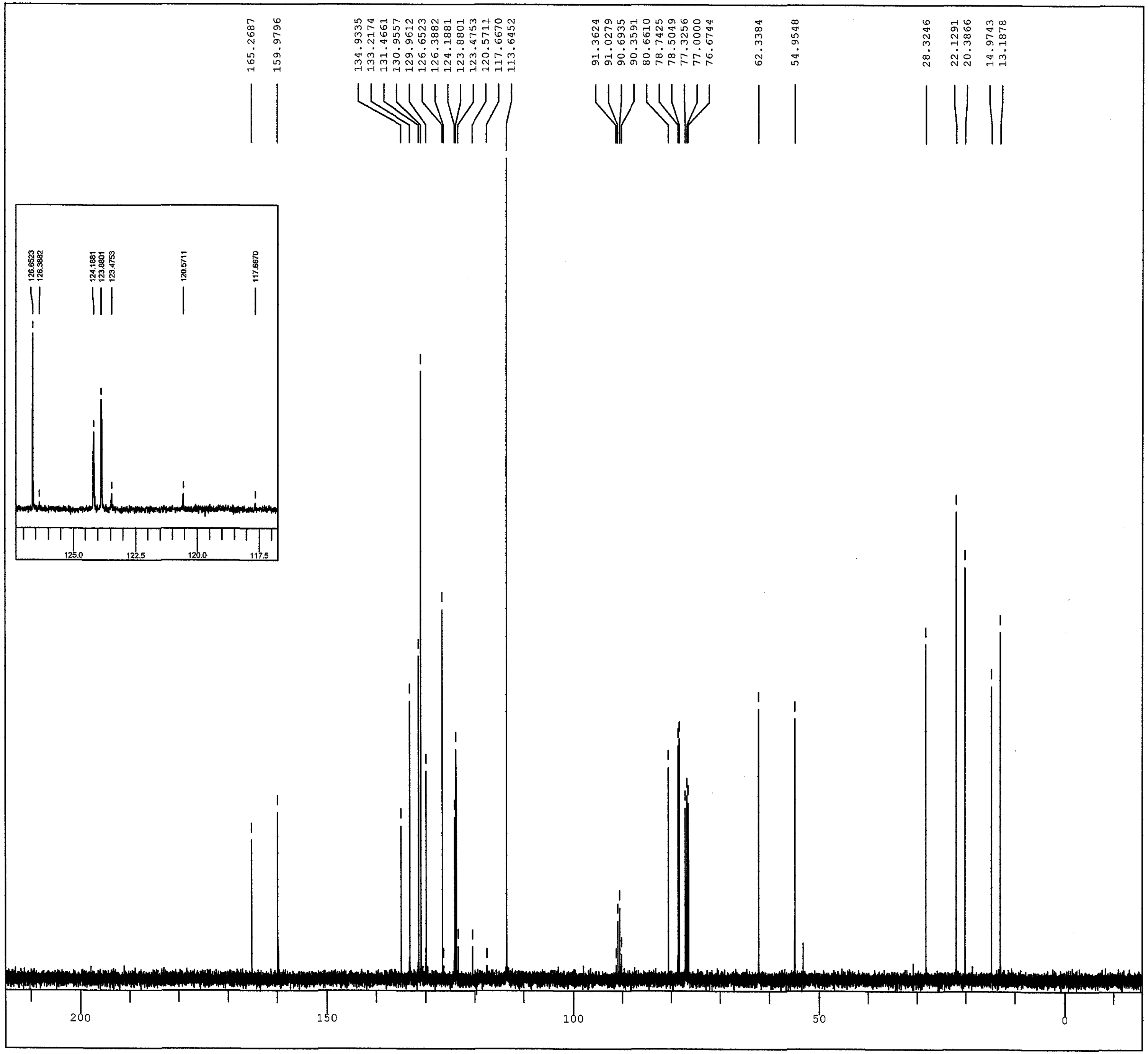

DFILE ozawa05-127_1_13C.jdf
COMNT nPr-yne, PMB
DATIM 11-08-2014 19:30:52
OBNUC 13C
EXMOD carbon.jxp
OBFRQ 98.52 MHz
OBSET 4.64 KHz
OBFIN 8.74 Hz
POINT 32767
FREQU 28409.09 Hz
SCANS 72
ACQTM 0.0000 sec
PD 3.0000 sec
PW1 3.00 usec
IRNUC 1H
CTEMP 24.3 c
SLVNT CDCL3
EXREF 77.00 ppm
BF 0.12 Hz
RGAIN 60

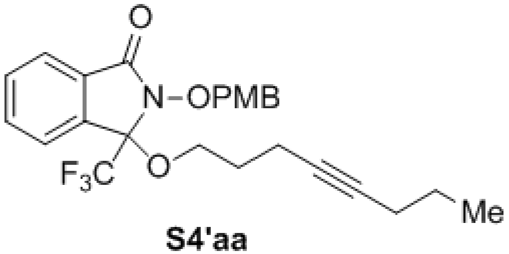

S4'aa

nPr-yne, PMB

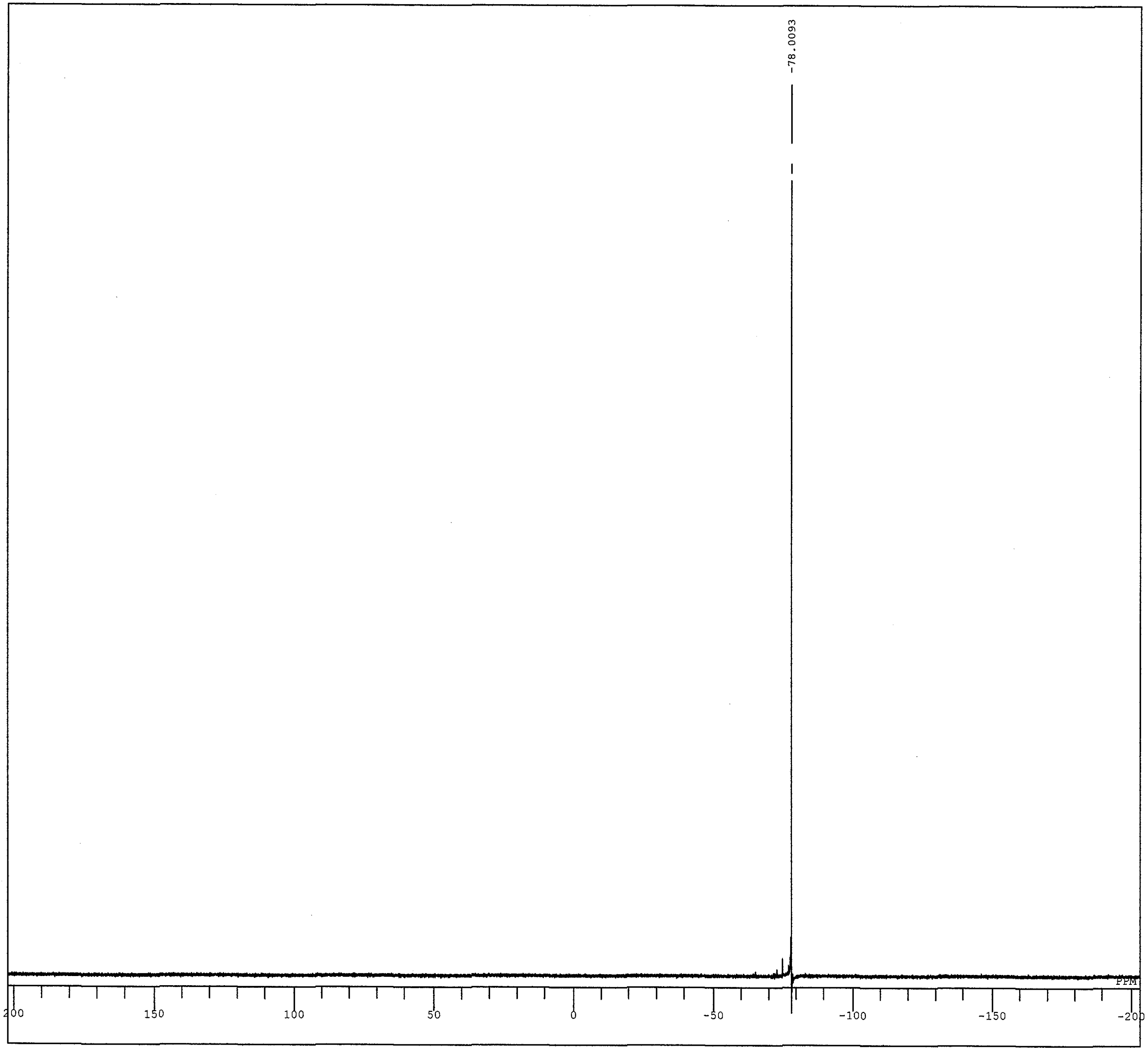


```
DFILE  ozawa05-127_1_19F.jdf
COMNT  nPr-yne, PMB
DATIM  11-08-2014 20:18:02
OBNUC  19F
EXMOD  proton.jxp
OBFRQ        368.64 MHz
OBSET          7.63 KHz
OBFIN          2.85 Hz
POINT         16384
FREQU     186567.17 Hz
SCANS             4
ACQTM        0.0878 sec
PD           5.0000 sec
PW1            3.90 usec
IRNUC  19F
CTEMP          24.1 c
SLVNT  CDCL3
EXREF       -164.90 ppm
BF             0.12 Hz
RGAIN            50
```

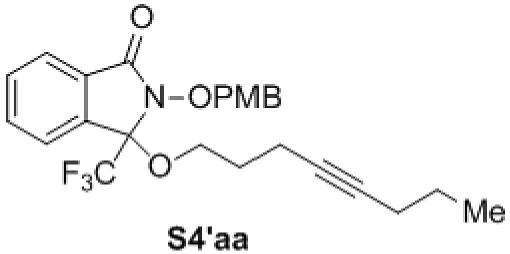

S4'aa

nPr-yne, OH

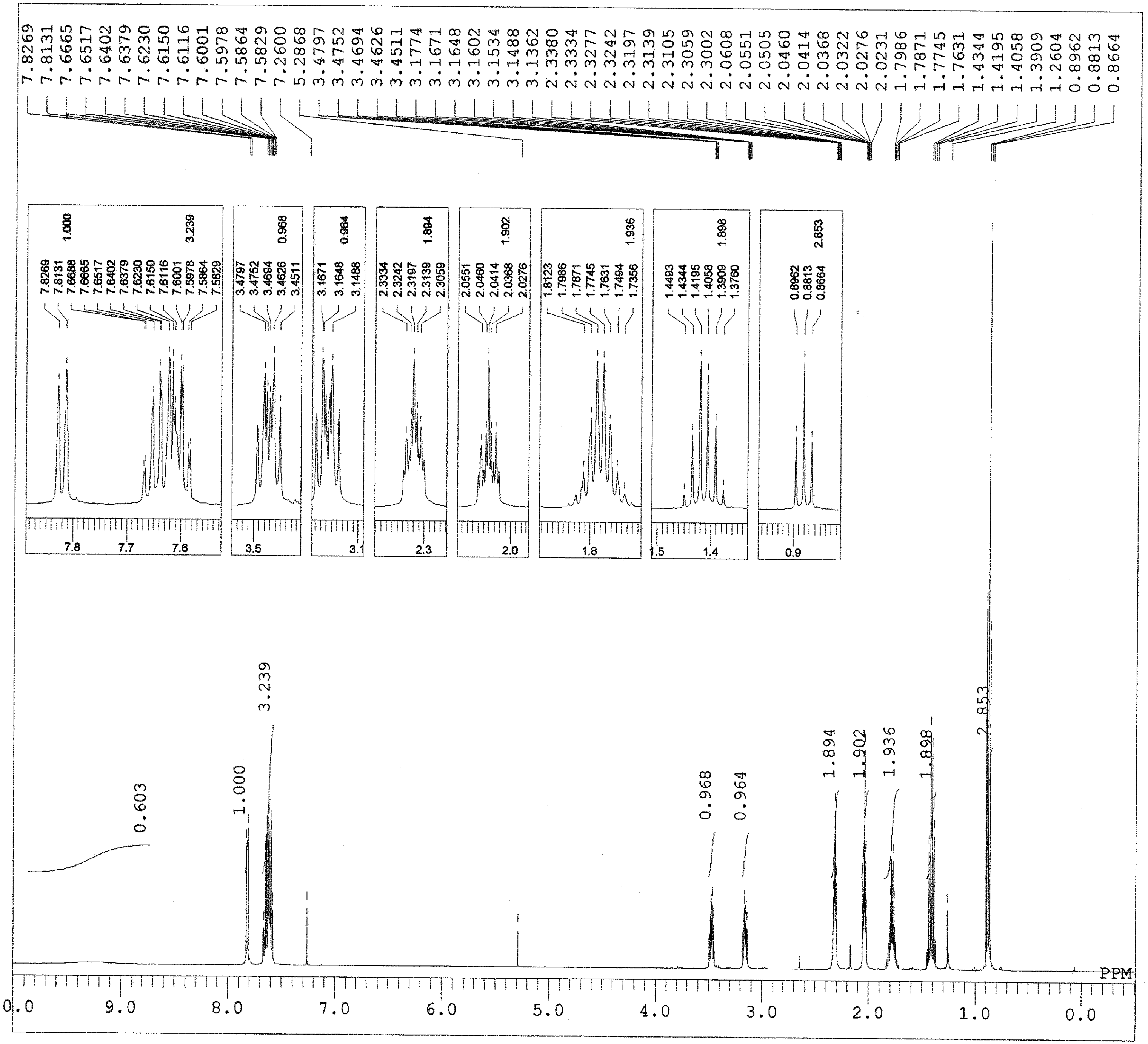

DFILE	ozawa05-129_1_1H.jdf
COMNT	nPr-yne, OH
DATIM	2014-08-15 18:45:04
OBNUC	1H
EXMOD	proton.jxp
OBFRQ	500.16 MHz
OBSET	2.41 KHz
OBFIN	6.01 Hz
POINT	16384
FREQU	9384.38 Hz
SCANS	4
ACQTM	1.7459 sec
PD	5.0000 sec
PW1	5.55 usec
IRNUC	1H
CTEMP	26.3 c
SLVNT	CDCL3
EXREF	7.26 ppm
BF	0.12 Hz
RGAIN	30

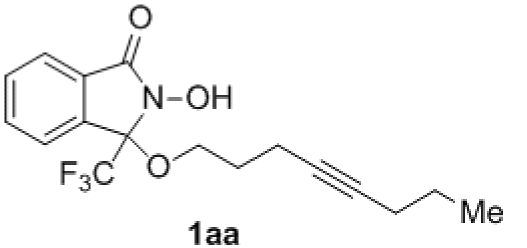

1aa

nPr-yne, OH

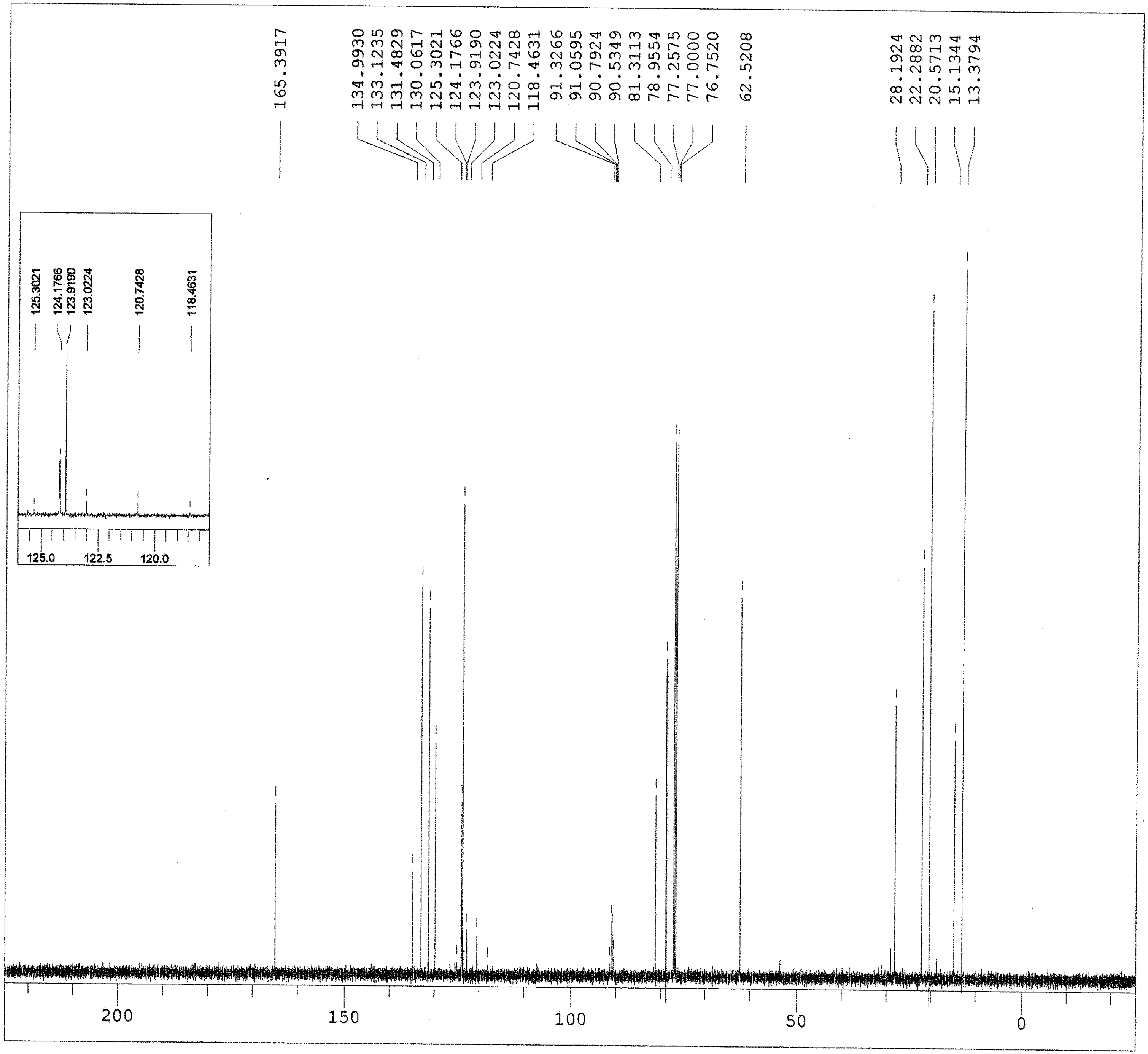

DFILE	ozawa05-129_1_13C.jdf
COMNT	nPr-yne, OH
DATIM	2014-08-15 18:46:07
OBNUC	13C
EXMOD	carbon.jxp
OBFRQ	125.77 MHz
OBSET	7.87 KHz
OBFIN	4.21 Hz
POINT	32767
FREQU	39308.18 Hz
SCANS	200
ACQTM	0.8336 sec
PD	3.0000 sec
PW1	3.40 usec
IRNUC	1H
CTEMP	26.6 c
SLVNT	CDCL3
EXREF	77.00 ppm
BF	0.12 Hz
RGAIN	60

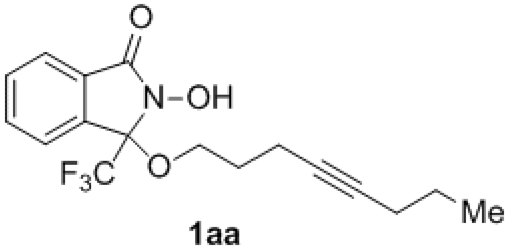

1aa

nPr-yne, OH

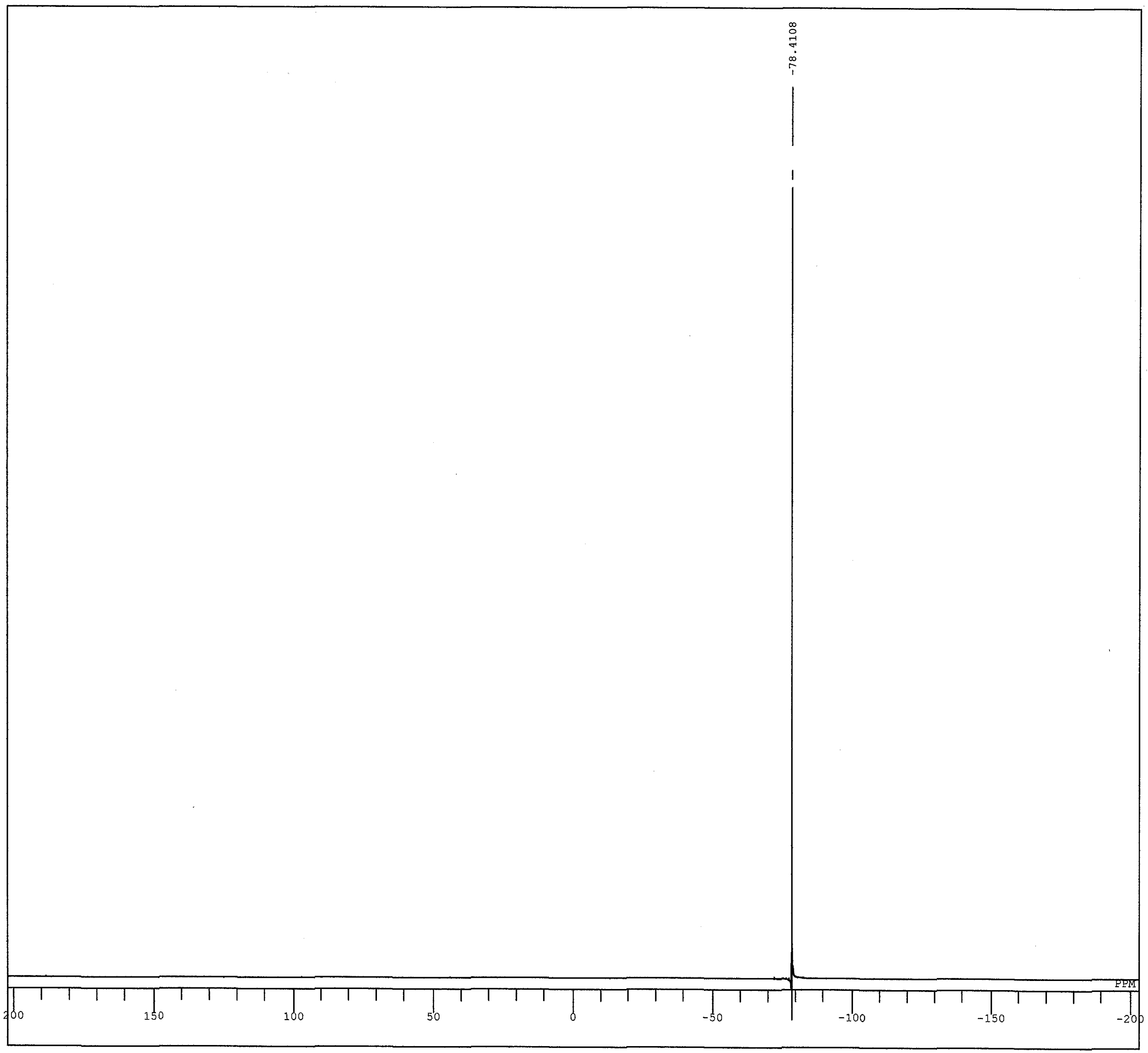


```
DFILE  ozawa05-129_1_19F.jdf
COMNT  nPr-yne, OH
DATIM  15-08-2014 19:57:17
OBNUC  19F
EXMOD  proton.jxp
OBFRQ         368.64 MHz
OBSET           7.63 KHz
OBFIN           2.85 Hz
POINT          16384
FREQU      186567.17 Hz
SCANS              4
ACQTM         0.0878 sec
PD            5.0000 sec
PW1             3.90 usec
IRNUC  19F
CTEMP           24.5 c
SLVNT  CDCL3
EXREF        -164.90 ppm
BF              0.12 Hz
RGAIN             44
```

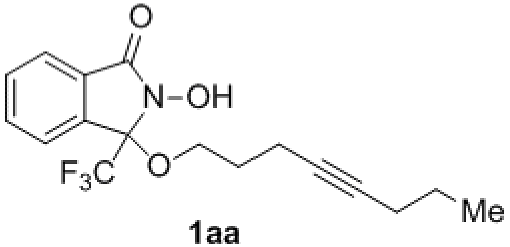

1aa

nPr-yne, [0]

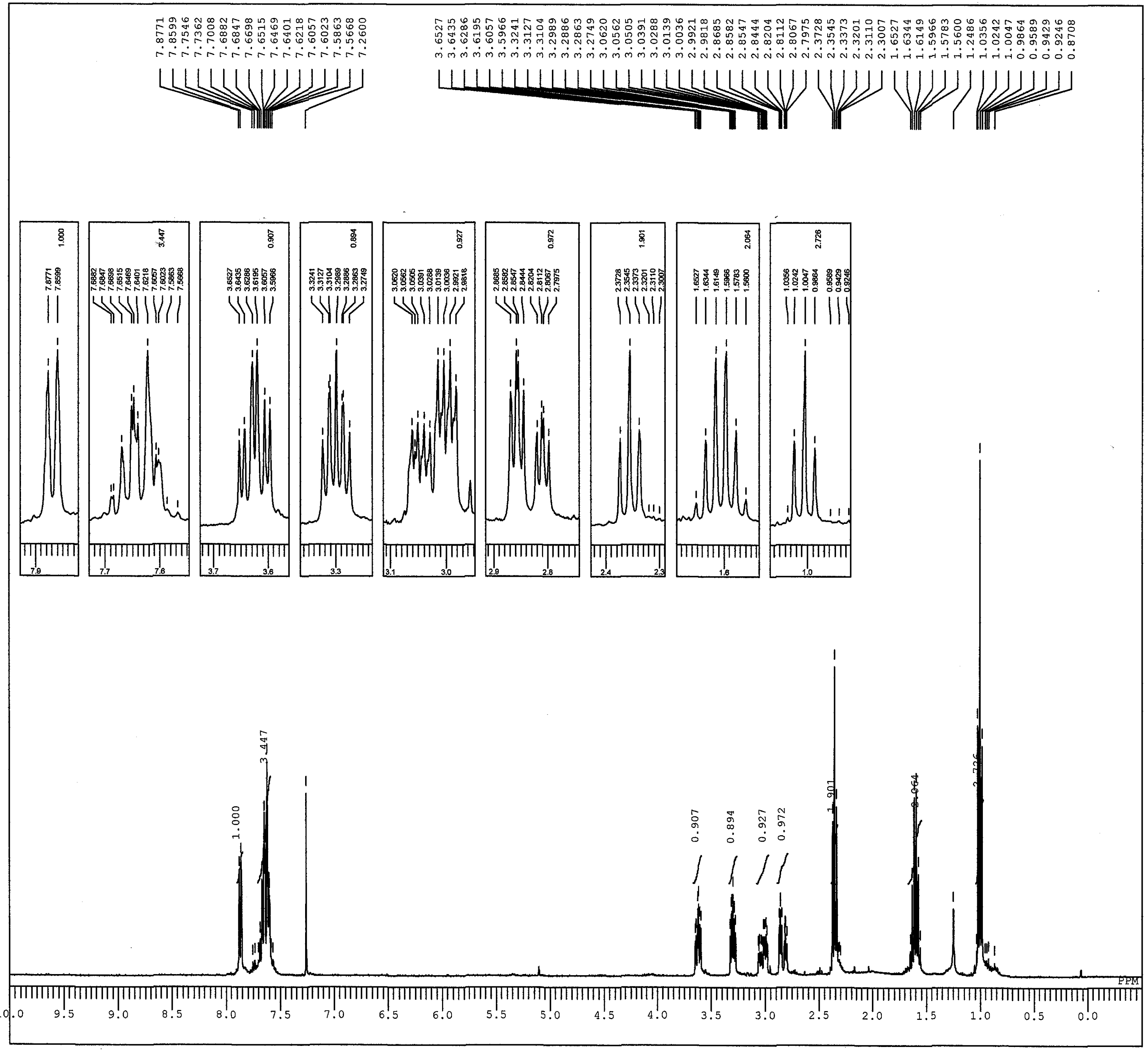

DFILE ozawa05-134_3_1H.jdf
COMNT nPr-yne, [O]
DATIM 14-08-2014 23:08:23
OBNUC 1H
EXMOD proton.jxp
OBFRQ 391.78 MHz
OBSET 8.51 KHz
OBFIN 3.34 Hz
POINT 16384
FREQU 7348.62 Hz
SCANS 4
ACQTM 2.2295 sec
PD 5.0000 sec
PW1 5.25 usec
IRNUC 1H
CTEMP 24.1 c
SLVNT CDCL3
EXREF 7.26 ppm
BF 0.12 Hz
RGAIN 38

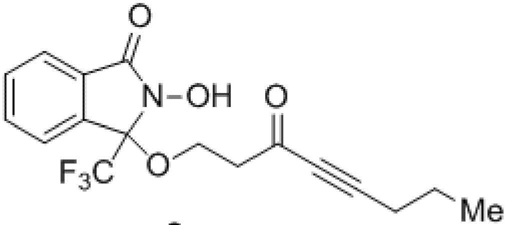

2aa

nPr-yne, [0]

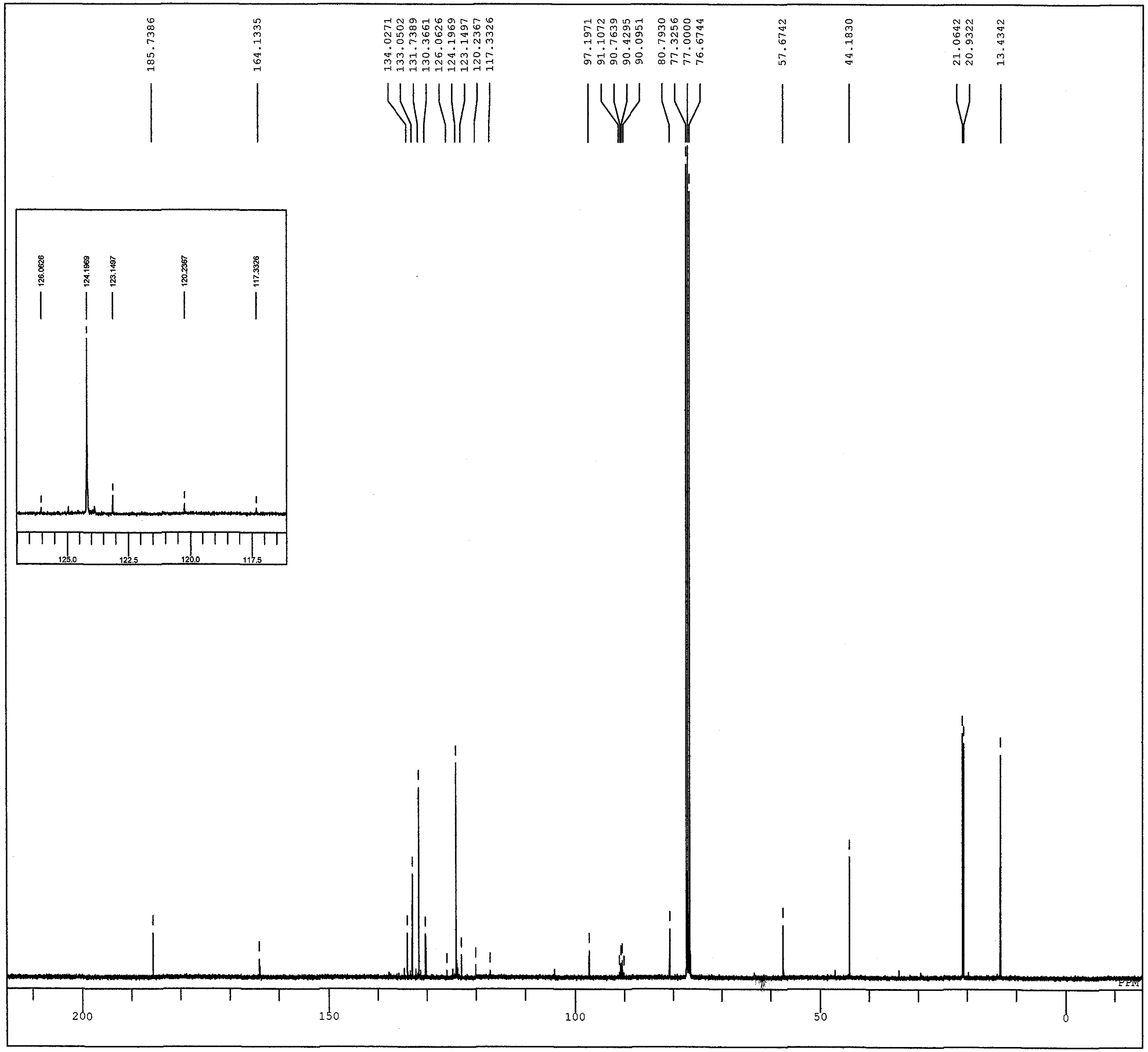

DFILE ozawa05-134_3_13C.jdf
COMNT nPr-yne, [O]
DATIM 14-08-2014 23:09:30
OBNUC 13C
EXMOD carbon.jxp
OBFRQ 98.52 MHz
OBSET 4.64 KHz
OBFIN 8.74 Hz
POINT 32767
FREQU 28409.09 Hz
SCANS 9000
ACQTM 1.1534 sec
PD 3.0000 sec
PW1 3.00 usec
IRNUC 1H
CTEMP 23.9 c
SLVNT CDCL3
EXREF 77.00 ppm
BF 0.12 Hz
RGAIN 60

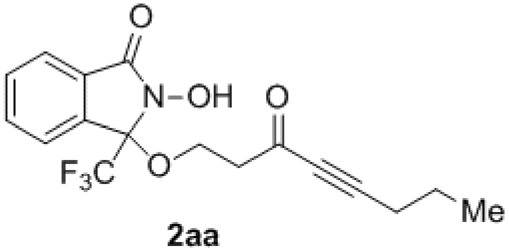

2aa

nPr-yne, [0]

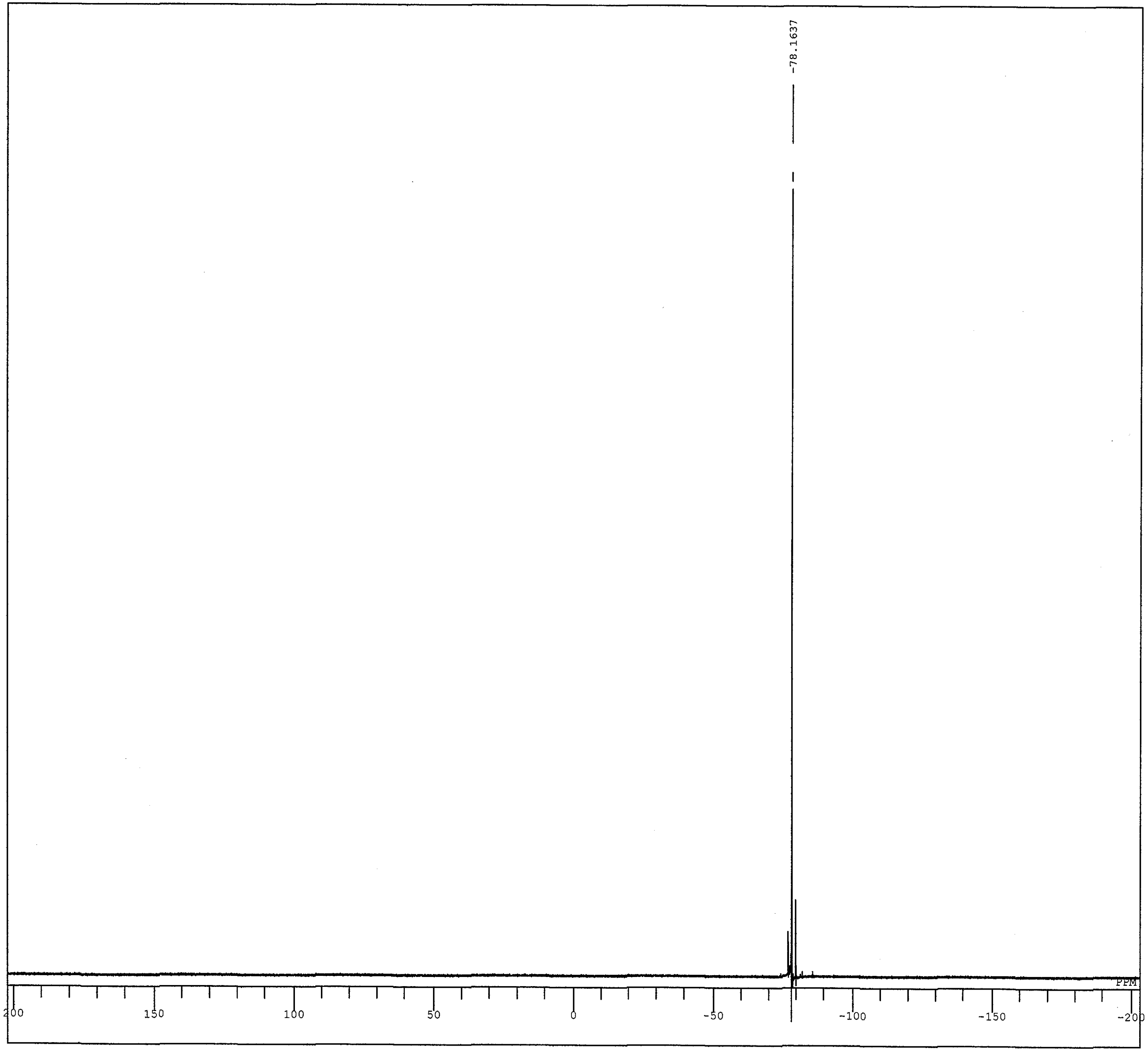

DFILE ozawa05-134_3_19F.jdf
COMNT nPr-yne, [O]
DATIM 15-08-2014 13:52:07
OBNUC 19F
EXMOD proton.jxp
OBFRQ 368.64 MHz
OBSET 7.63 KHz
OBFIN 2.85 Hz
POINT 16384
FREQU 186567.17 Hz
SCANS 4
ACQTM 0.0878 sec
PD 5.0000 sec
PW1 3.90 usec
IRNUC 19F
CTEMP 24.2 c
SLVNT CDCL3
EXREF -164.90 ppm
BF 0.12 Hz
RGAIN 50

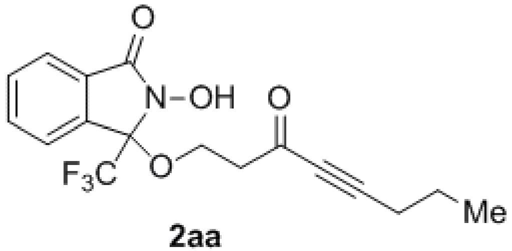

2aa

tBu-yne, PMB

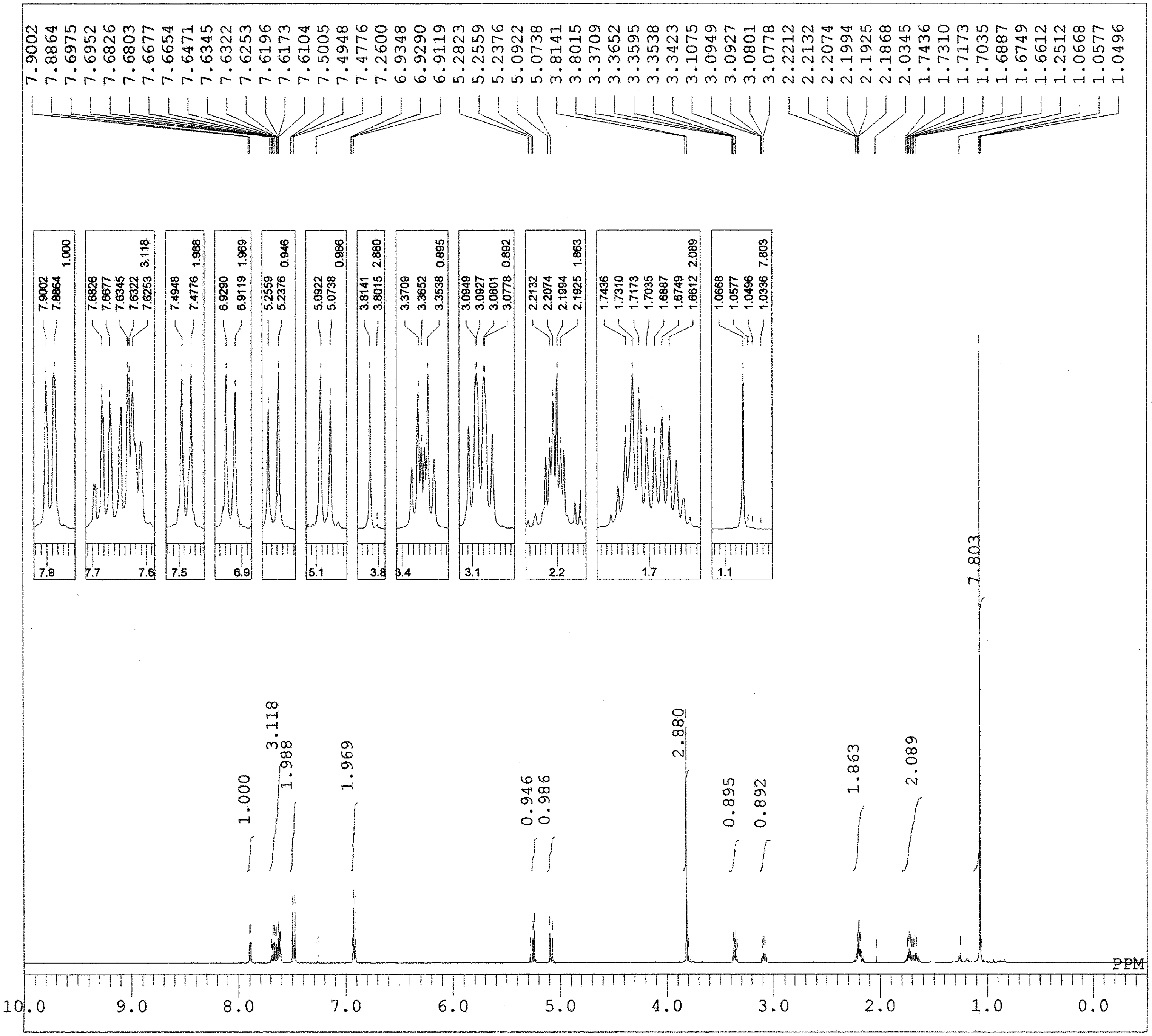

DFILE	ozawa05-125_2_1H.jdf
COMNT	tBu-yne, PMB
DATIM	2014-08-07 15:21:25
OBNUC	1H
EXMOD	proton.jxp
OBFRQ	500.16 MHz
OBSET	2.41 KHz
OBFIN	6.01 Hz
POINT	16384
FREQU	9384.38 Hz
SCANS	4
ACQTM	1.7459 sec
PD	5.0000 sec
PW1	5.55 usec
IRNUC	1H
CTEMP	26.6 c
SLVNT	CDCL3
EXREF	7.26 ppm
BF	0.12 Hz
RGAIN	28

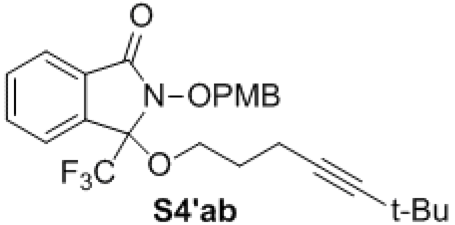

S4'ab

tBu-yne, PMB

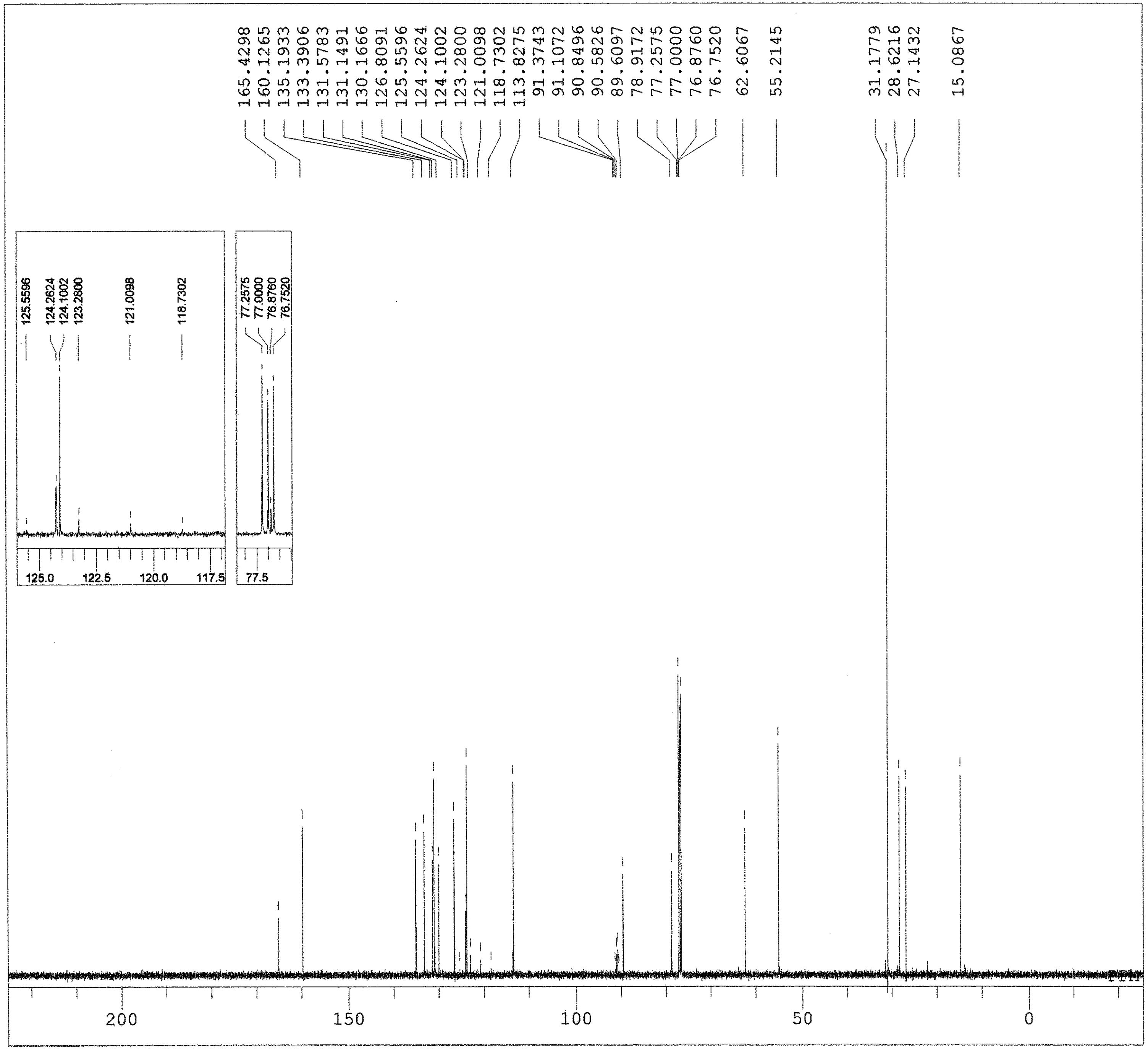

DFILE	ozawa05-125_2_13C.jdf
COMNT	tBu-yne, PMB
DATIM	2014-08-07 15:22:39
OBNUC	13C
EXMOD	carbon.jxp
OBFRQ	125.77 MHz
OBSET	7.87 KHz
OBFIN	4.21 Hz
POINT	32767
FREQU	39308.18 Hz
SCANS	200
ACQTM	0.8336 sec
PD	3.0000 sec
PW1	3.40 usec
IRNUC	1H
CTEMP	27.2 c
SLVNT	CDCL3
EXREF	77.00 ppm
BF	0.12 Hz
RGAIN	60

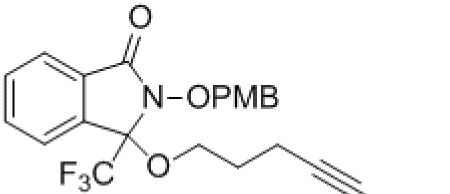

S4'ab

tBu-yne, PMB

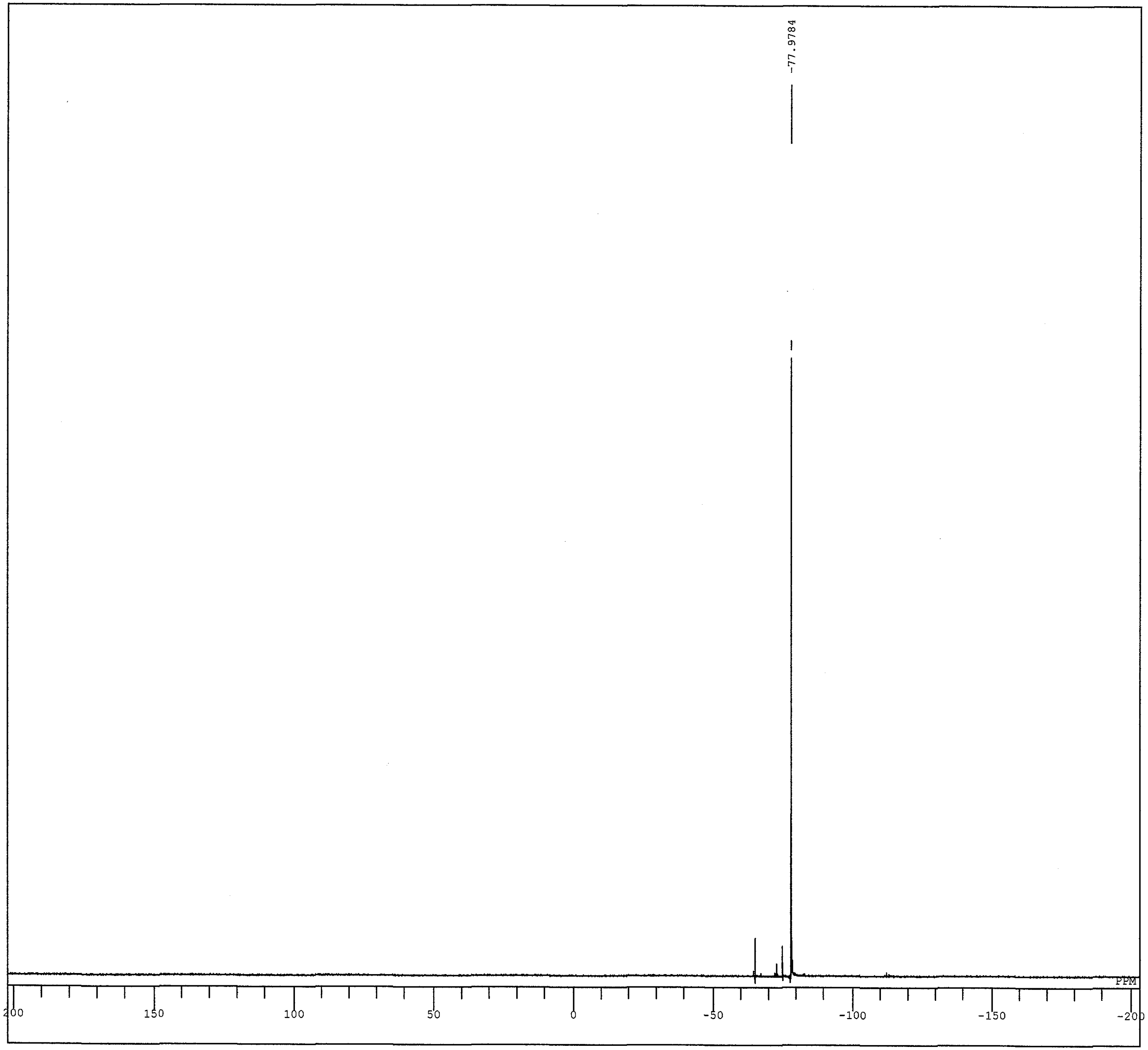


```
DFILE  ozawa05-125_2_19F.jdf
COMNT  tBu-yne, PMB
DATIM  07-08-2014 16:15:31
OBNUC  19F
EXMOD  proton.jxp
OBFRQ        368.64 MHz
OBSET          7.63 KHz
OBFIN          2.85 Hz
POINT         16384
FREQU     186567.17 Hz
SCANS             4
ACQTM        0.0878 sec
PD           5.0000 sec
PW1            3.90 usec
IRNUC  19F
CTEMP          24.9 c
SLVNT  CDCL3
EXREF       -164.90 ppm
BF             0.12 Hz
RGAIN            48
```

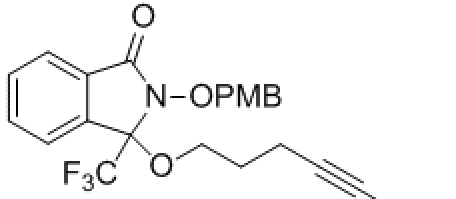

S4'ab

tBu-yne, OH

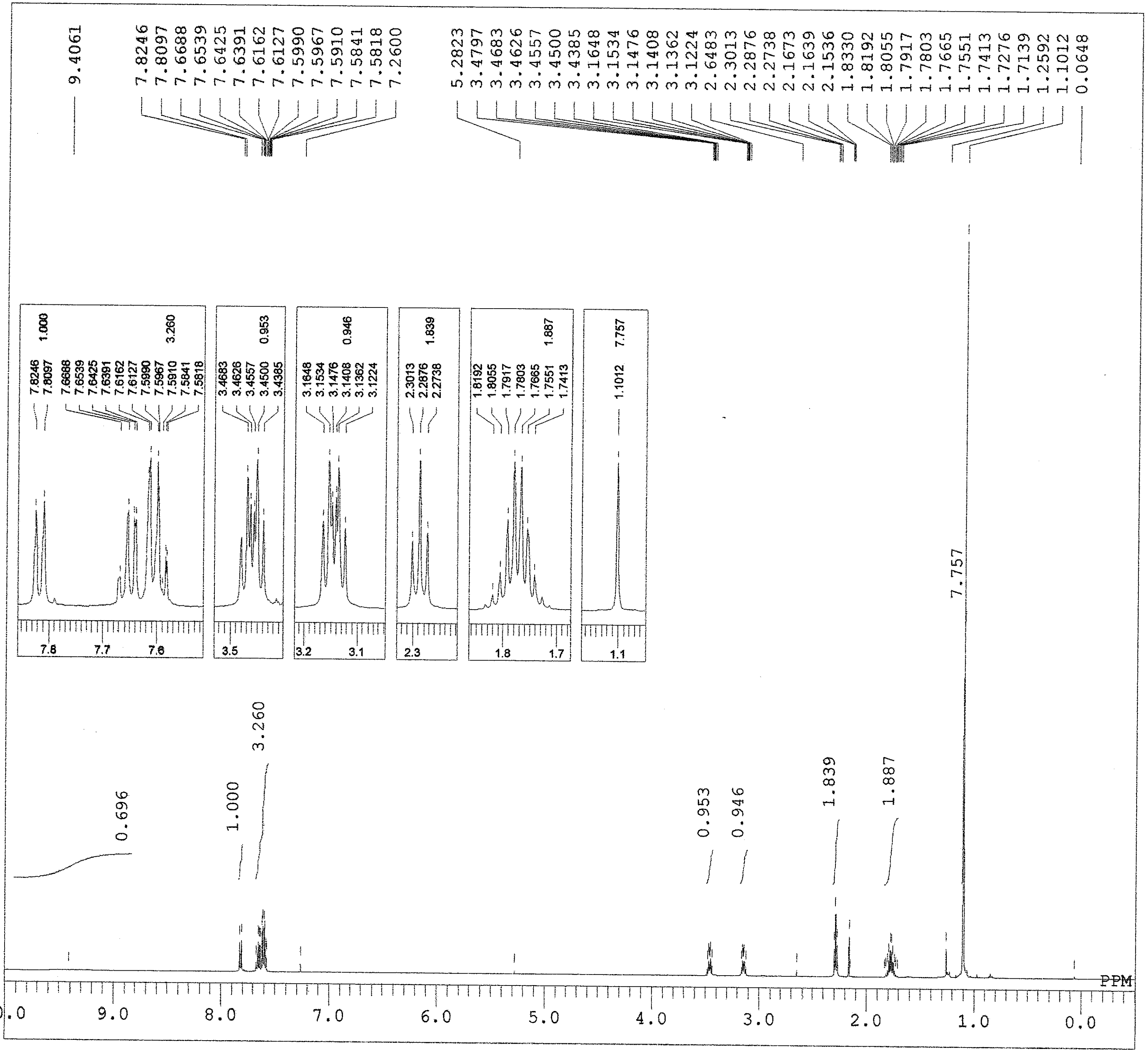

DFILE	ozawa05-128_1_1H.jdf
COMNT	tBu-yne, OH
DATIM	2014-08-15 18:35:12
OBNUC	1H
EXMOD	proton.jxp
OBFRQ	500.16 MHz
OBSET	2.41 KHz
OBFIN	6.01 Hz
POINT	16384
FREQU	9384.38 Hz
SCANS	4
ACQTM	1.7459 sec
PD	5.0000 sec
PW1	5.55 usec
IRNUC	1H
CTEMP	26.1 c
SLVNT	CDCL3
EXREF	7.26 ppm
BF	0.12 Hz
RGAIN	26

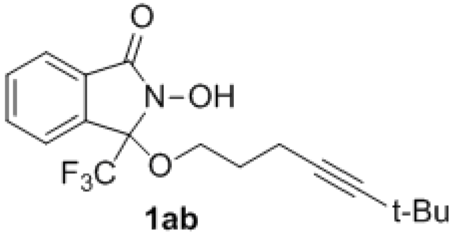

1ab

tBu-yne, OH

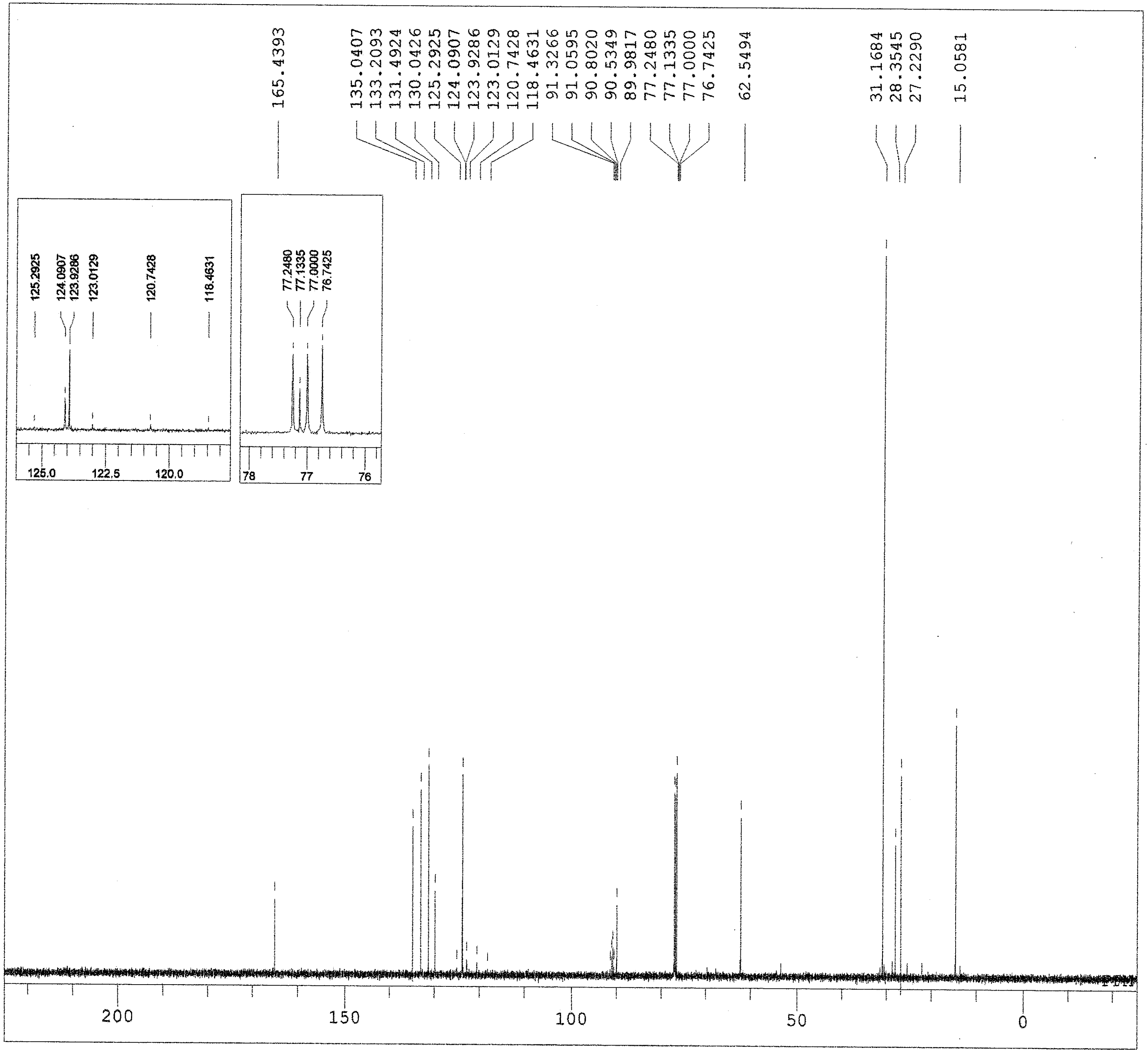

DFILE ozawa05-128_1_13C.jdf
COMNT tBu-yne, OH
DATIM 2014-08-15 18:36:15
OBNUC 13C
EXMOD carbon.jxp
OBFRQ 125.77 MHz
OBSET 7.87 KHz
OBFIN 4.21 Hz
POINT 32767
FREQU 39308.18 Hz
SCANS 81
ACQTM 0.8336 sec
PD 3.0000 sec
PW1 3.40 usec
IRNUC 1H
CTEMP 26.5 c
SLVNT CDCL3
EXREF 77.00 ppm
BF 0.12 Hz
RGAIN 60

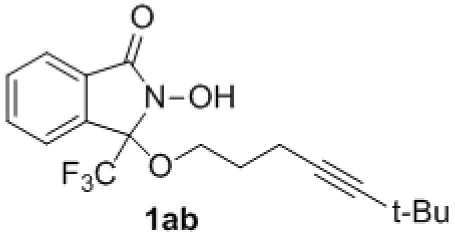

1ab

tBu-yne, OH

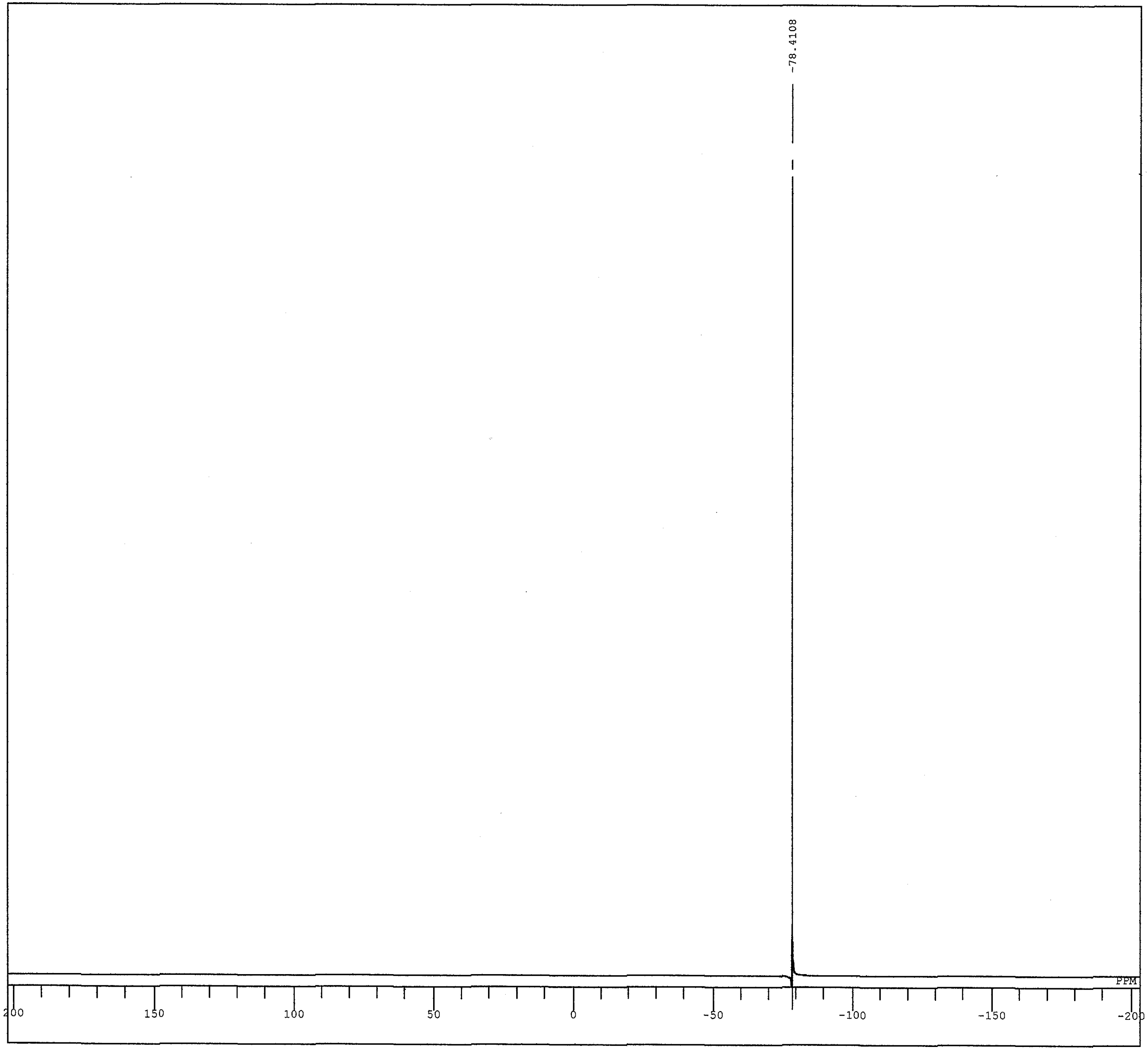

DFILE	ozawa05-128_2_19F.jdf
COMNT	tBu-yne, OH
DATIM	15-08-2014 19:53:50
OBNUC	19F
EXMOD	proton.jxp
OBFRQ	368.64 MHz
OBSET	7.63 KHz
OBFIN	2.85 Hz
POINT	16384
FREQU	186567.17 Hz
SCANS	4
ACQTM	0.0878 sec
PD	5.0000 sec
PW1	3.90 usec
IRNUC	19F
CTEMP	24.5 c
SLVNT	CDCL3
EXREF	-164.90 ppm
BF	0.12 Hz
RGAIN	44

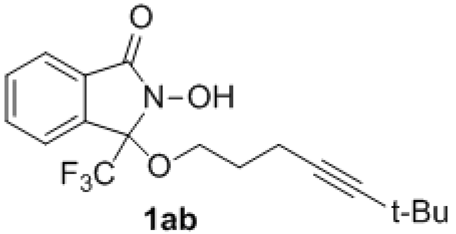

1ab

tBu-yne, [O]

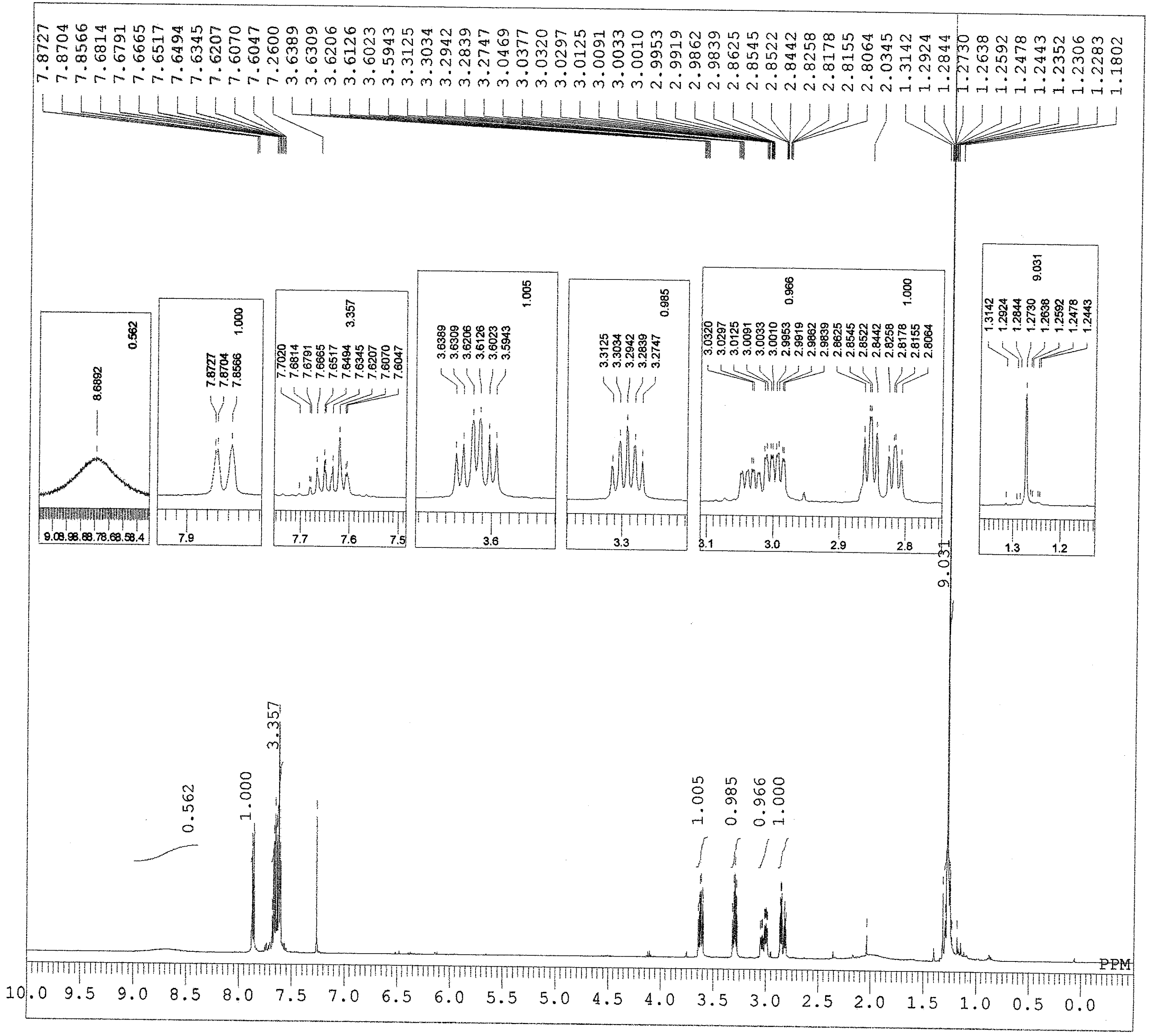

DFILE	ozawa05-134_2_1H.jdf
COMNT	tBu-yne, [O]
DATIM	2014-08-14 22:48:47
OBNUC	1H
EXMOD	proton.jxp
OBFRQ	500.16 MHz
OBSET	2.41 KHz
OBFIN	6.01 Hz
POINT	16384
FREQU	9384.38 Hz
SCANS	4
ACQTM	1.7459 sec
PD	5.0000 sec
PW1	5.55 usec
IRNUC	1H
CTEMP	25.7 c
SLVNT	CDCL3
EXREF	7.26 ppm
BF	0.12 Hz
RGAIN	36

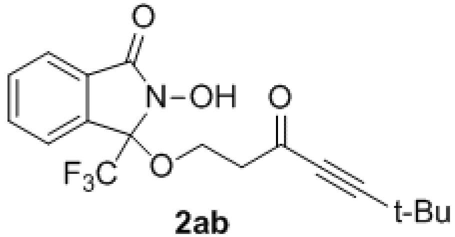

2ab

tBu-yne, [0]

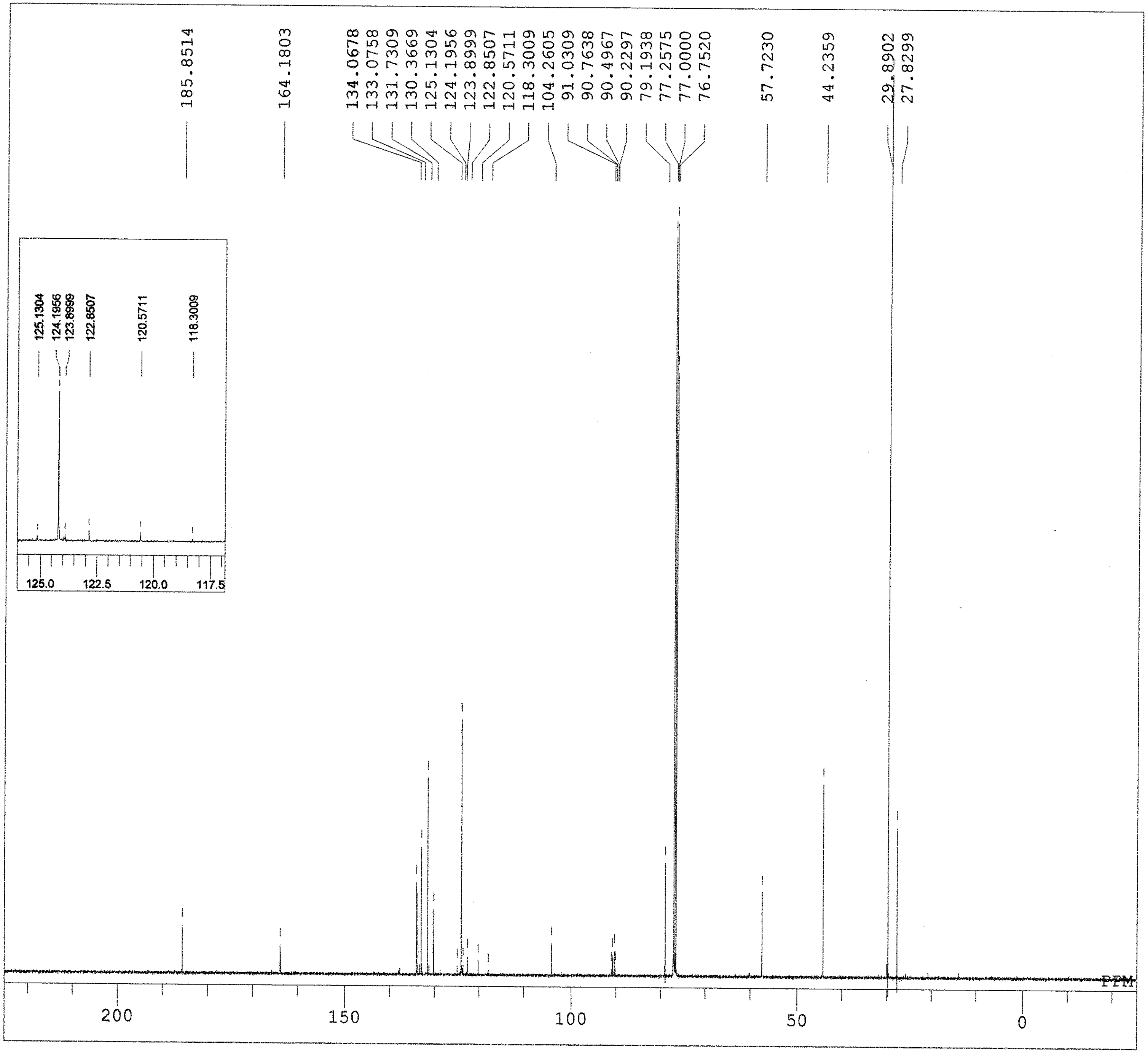

DFILE	ozawa05-134_2_13C.jdf
COMNT	tBu-yne, [0]
DATIM	2014-08-14 22:49:50
OBNUC	13C
EXMOD	carbon.jxp
OBFRQ	125.77 MHz
OBSET	7.87 KHz
OBFIN	4.21 Hz
POINT	32767
FREQU	39308.18 Hz
SCANS	9400
ACQTM	0.8336 sec
PD	3.0000 sec
PW1	3.40 usec
IRNUC	1H
CTEMP	26.0 c
SLVNT	CDCL3
EXREF	77.00 ppm
BF	0.12 Hz
RGAIN	60

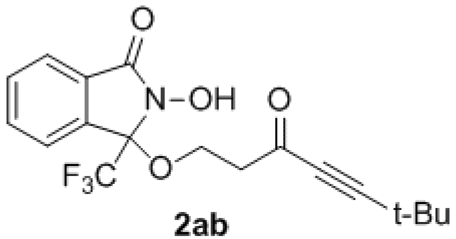

2ab

tBu-yne, [O]

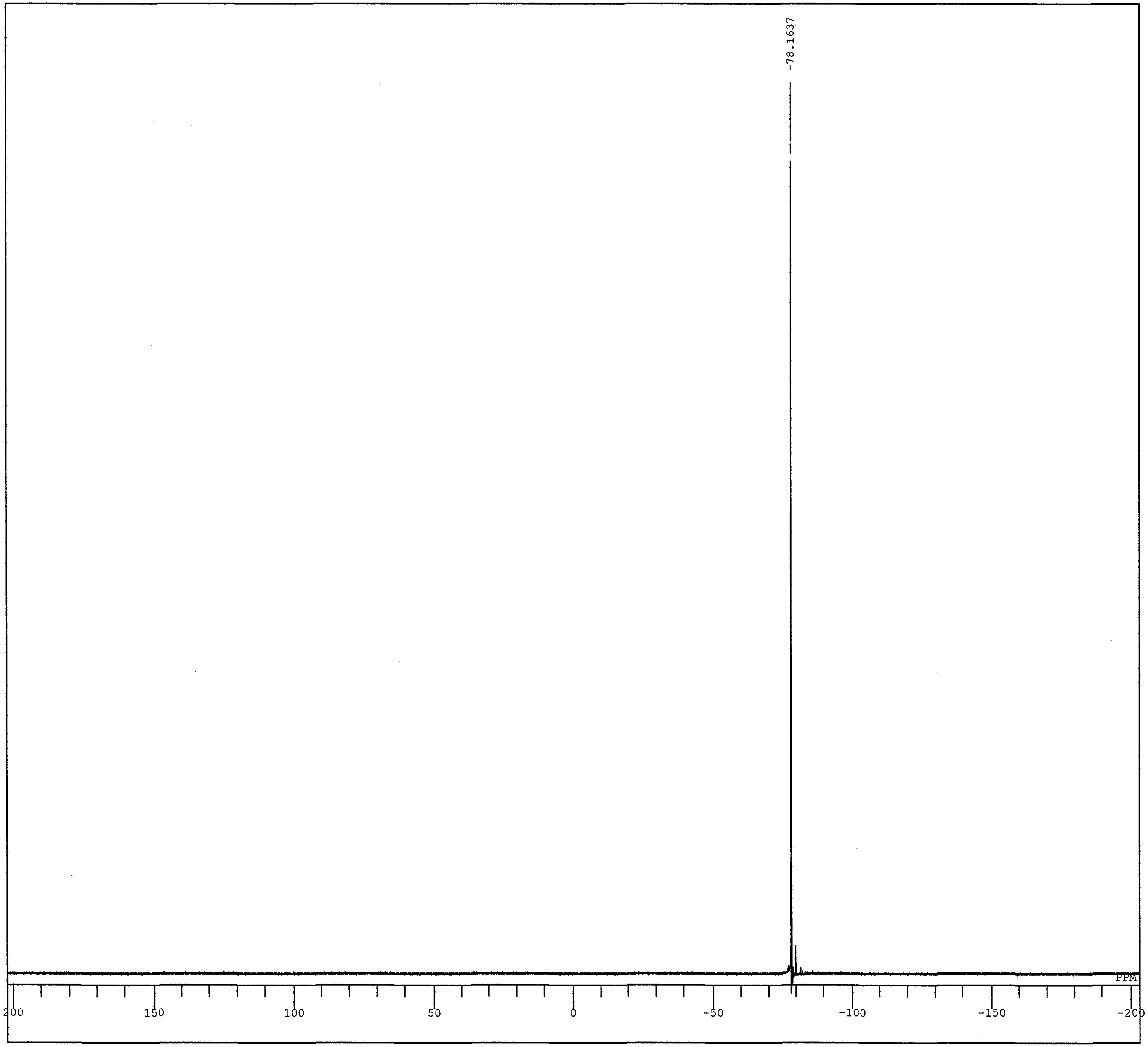

DFILE ozawa05-134_2_19F.jdf
COMNT tBu-yne, [O]
DATIM 15-08-2014 13:49:26
OBNUC 19F
EXMOD proton.jxp
OBFRQ 368.64 MHz
OBSET 7.63 KHz
OBFIN 2.85 Hz
POINT 16384
FREQU 186567.17 Hz
SCANS 4
ACQTM 0.0878 sec
PD 5.0000 sec
PW1 3.90 usec
IRNUC 19F
CTEMP 24.2 c
SLVNT CDCL3
EXREF -164.90 ppm
BF 0.12 Hz
RGAIN 48

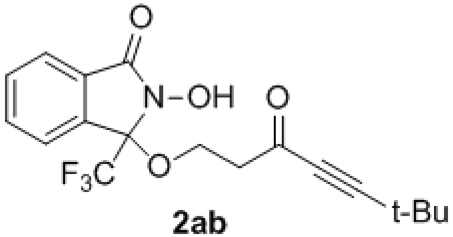

2ab

yne-tert, PMB

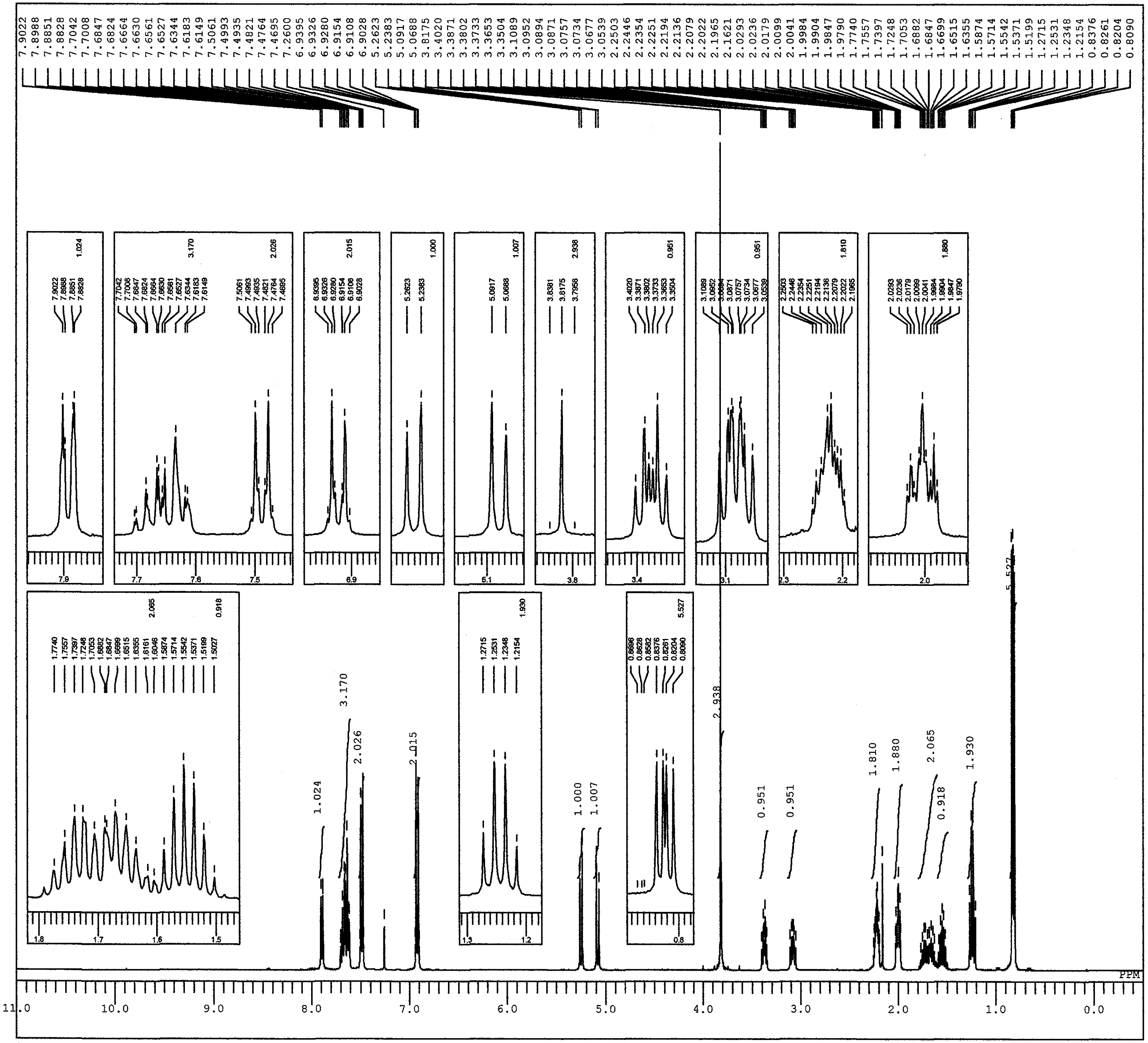

DFILE	ozawa07-028_1H.jdf
COMNT	yne-tert, PMB
DATIM	05-03-2015 13:49:18
OBNUC	1H
EXMOD	proton.jxp
OBFRQ	391.78 MHz
OBSET	8.51 KHz
OBFIN	3.34 Hz
POINT	16384
FREQU	7348.62 Hz
SCANS	4
ACQTM	2.2295 sec
PD	5.0000 sec
PW1	4.99 usec
IRNUC	1H
CTEMP	23.7 c
SLVNT	CDCL3
EXREF	7.26 ppm
BF	0.12 Hz
RGAIN	28

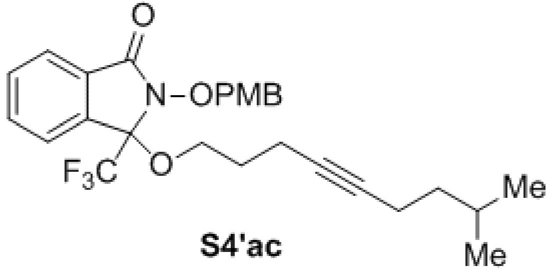

S4'ac

yne-tert, PMB

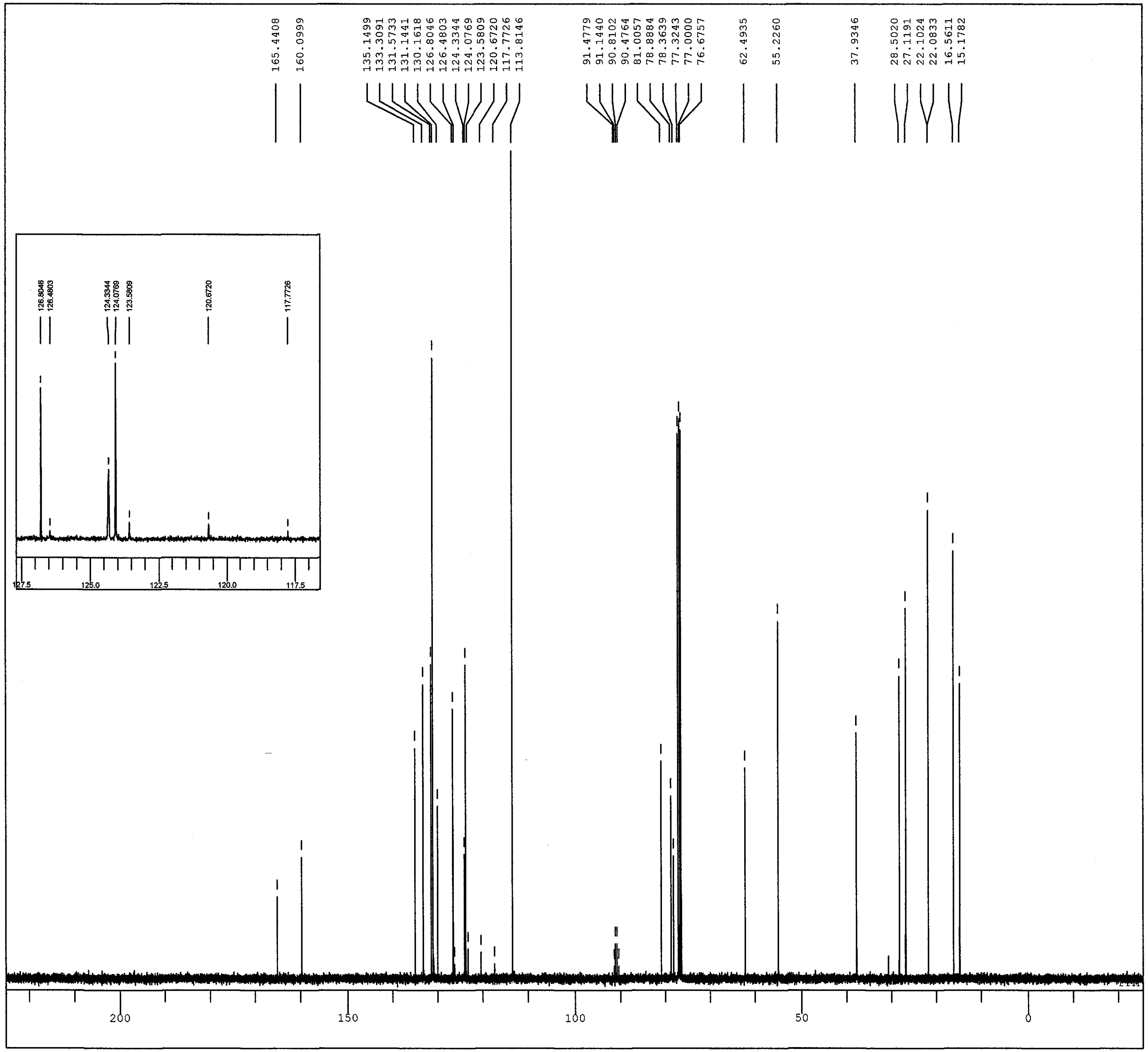

DFILE ozawa07-028_13C.jdf
COMNT yne-tert, PMB
DATIM 05-03-2015 13:50:22
OBNUC 13C
EXMOD carbon.jxp
OBFRQ 98.52 MHz
OBSET 4.64 KHz
OBFIN 8.74 Hz
POINT 32767
FREQU 30788.18 Hz
SCANS 904
ACQTM 1.0643 sec
PD 2.0000 sec
PW1 3.16 usec
IRNUC 1H
CTEMP 23.8 c
SLVNT CDCL3
EXREF 77.00 ppm
BF 0.12 Hz
RGAIN 60

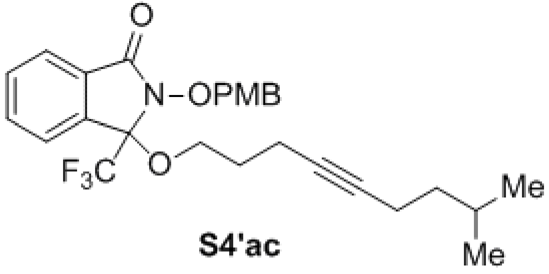

S4'ac

yne-tert, PMB

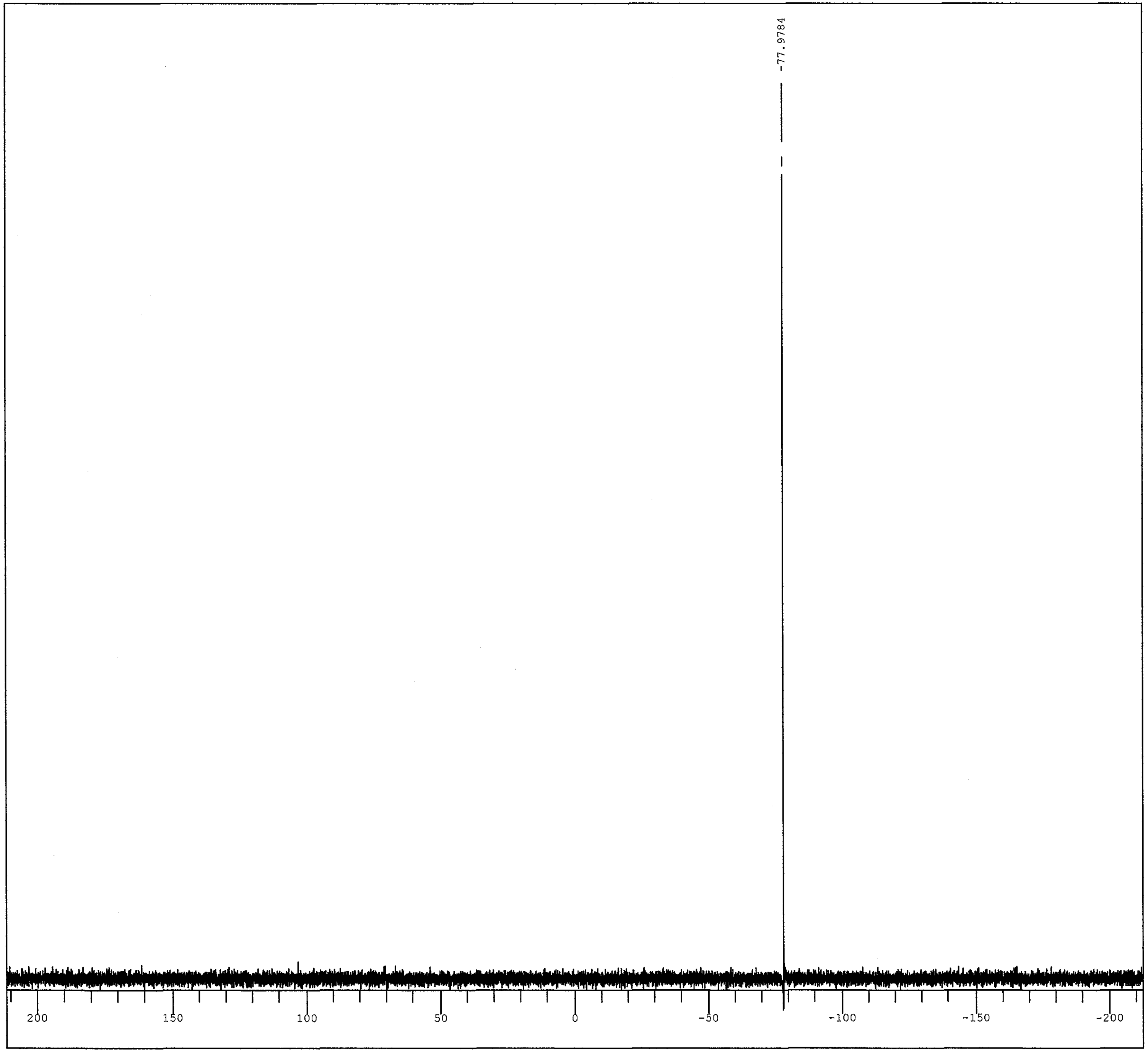


```
DFILE  ozawa07-028_19F.jdf
COMNT  yne-tert, PMB
DATIM  21-03-2015 12:16:18
OBNUC  19F
EXMOD  proton.jxp
OBFRQ        368.64 MHz
OBSET          7.63 KHz
OBFIN          2.85 Hz
POINT         16384
FREQU     195312.50 Hz
SCANS             4
ACQTM        0.0839 sec
PD           5.0000 sec
PW1            3.90 usec
IRNUC  19F
CTEMP          25.0 c
SLVNT  CDCL3
EXREF       -164.90 ppm
BF             0.12 Hz
RGAIN            50
```

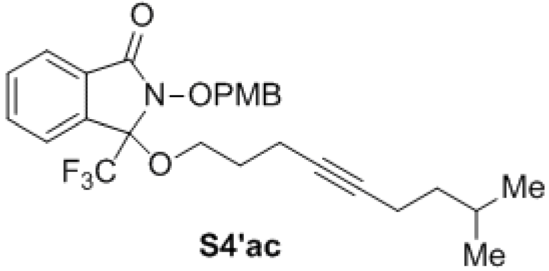

S4'ac

yne-tert, OH

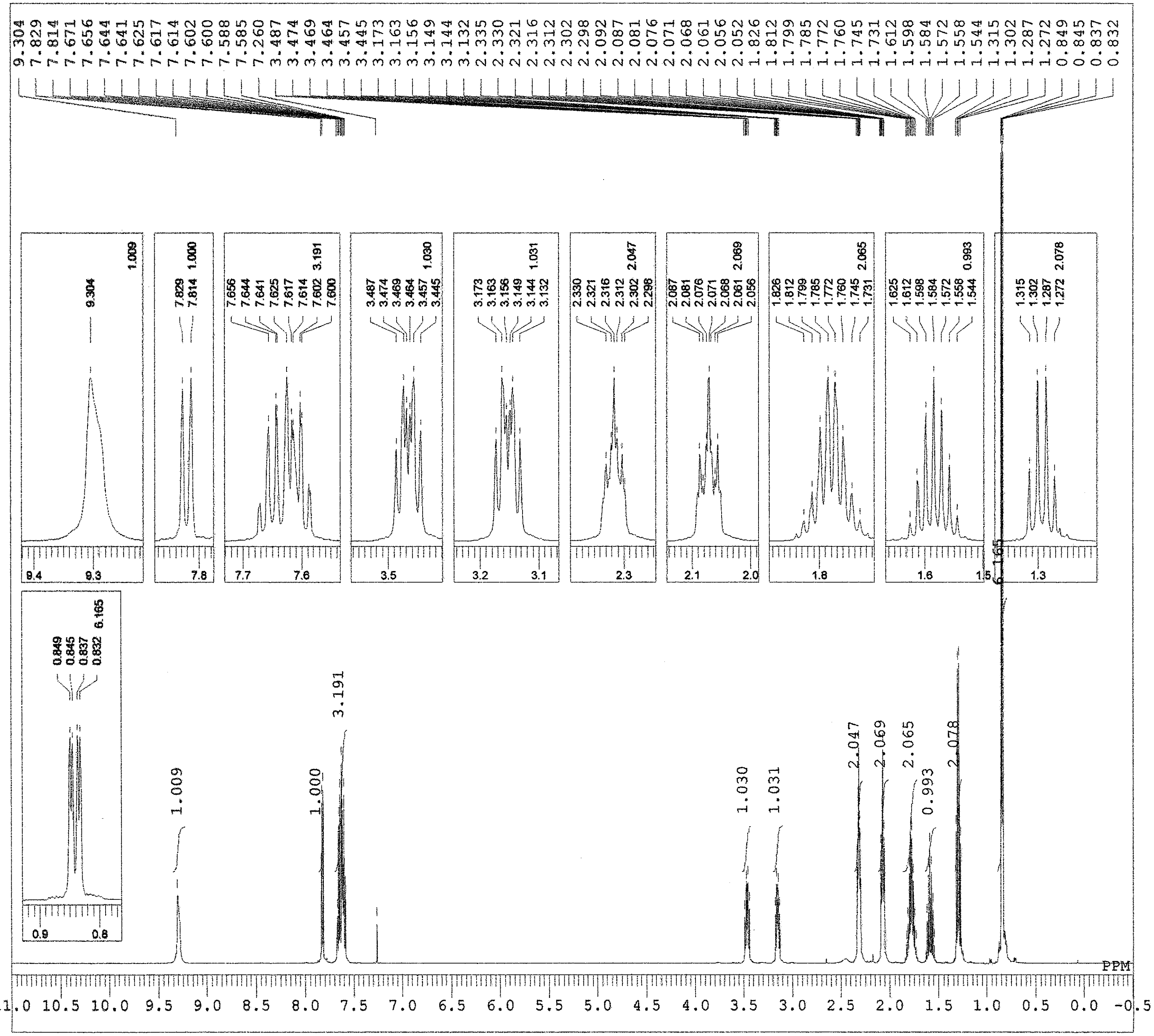

DFILE	ozawa07-025_1H.jdf
COMNT	yne-tert, OH
DATIM	2015-03-07 12:46:00
OBNUC	1H
EXMOD	proton.jxp
OBFRQ	500.16 MHz
OBSET	2.41 KHz
OBFIN	6.01 Hz
POINT	16384
FREQU	9384.38 Hz
SCANS	8
ACQTM	1.7459 sec
PD	5.0000 sec
PW1	5.55 usec
IRNUC	1H
CTEMP	25.2 c
SLVNT	CDCL3
EXREF	7.26 ppm
BF	0.12 Hz
RGAIN	26

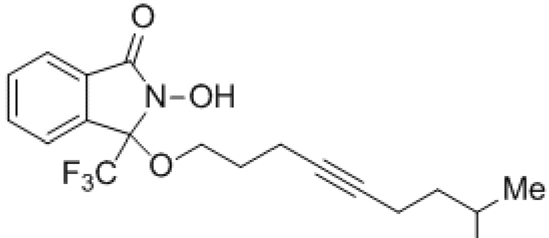

1ac

yne-tert, OH

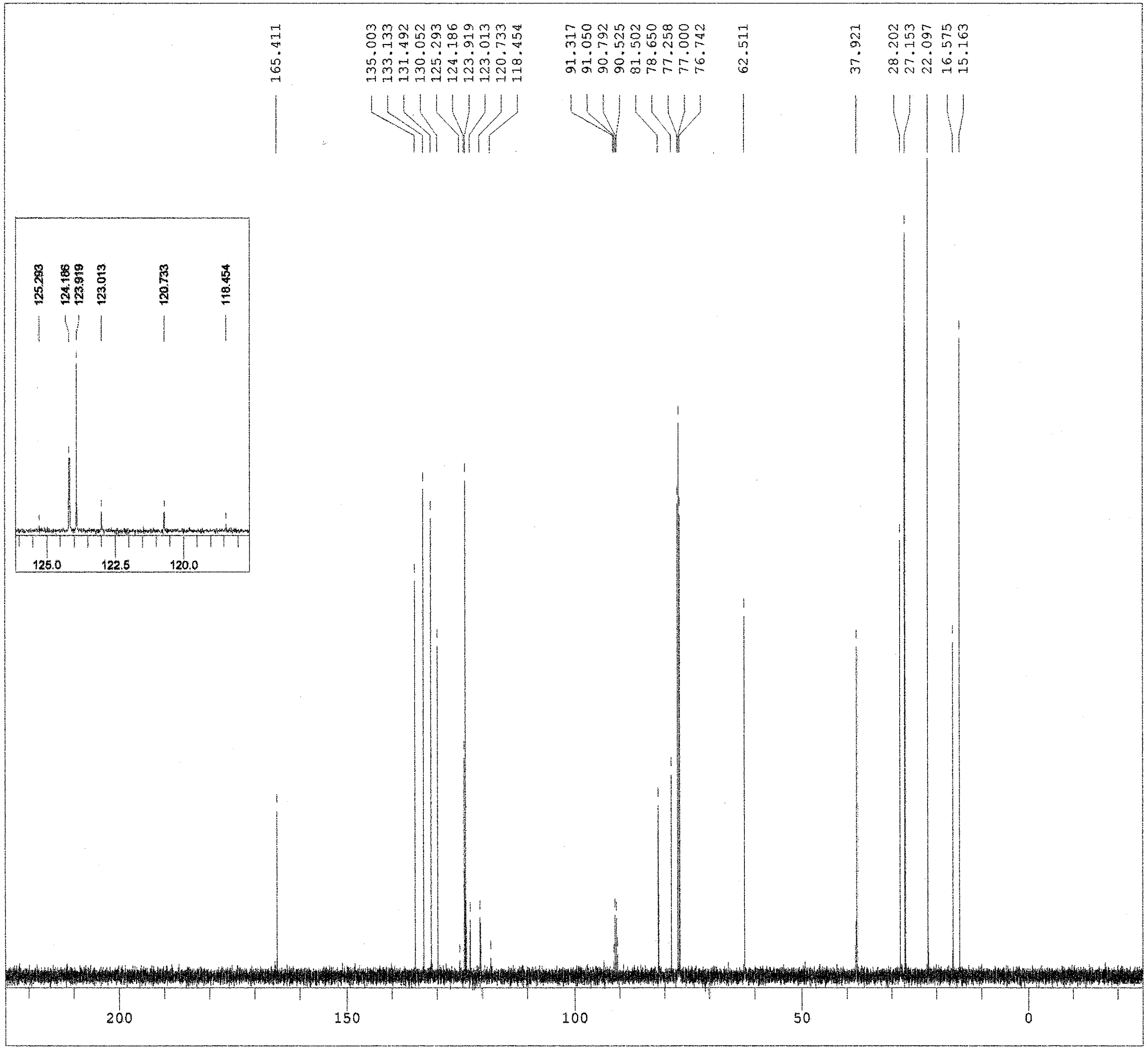

DFILE	ozawa07-025_13C.jdf
COMNT	yne-tert, OH
DATIM	2015-03-07 12:47:30
OBNUC	13C
EXMOD	carbon.jxp
OBFRQ	125.77 MHz
OBSET	7.87 KHz
OBFIN	4.21 Hz
POINT	32767
FREQU	39308.18 Hz
SCANS	180
ACQTM	0.8336 sec
PD	2.0000 sec
PW1	3.40 usec
IRNUC	1H
CTEMP	25.7 c
SLVNT	CDCL3
EXREF	77.00 ppm
BF	0.12 Hz
RGAIN	60

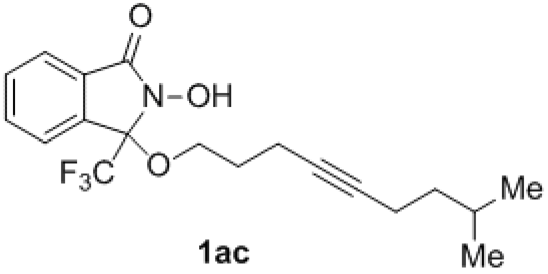

1ac

yne-tert, OH

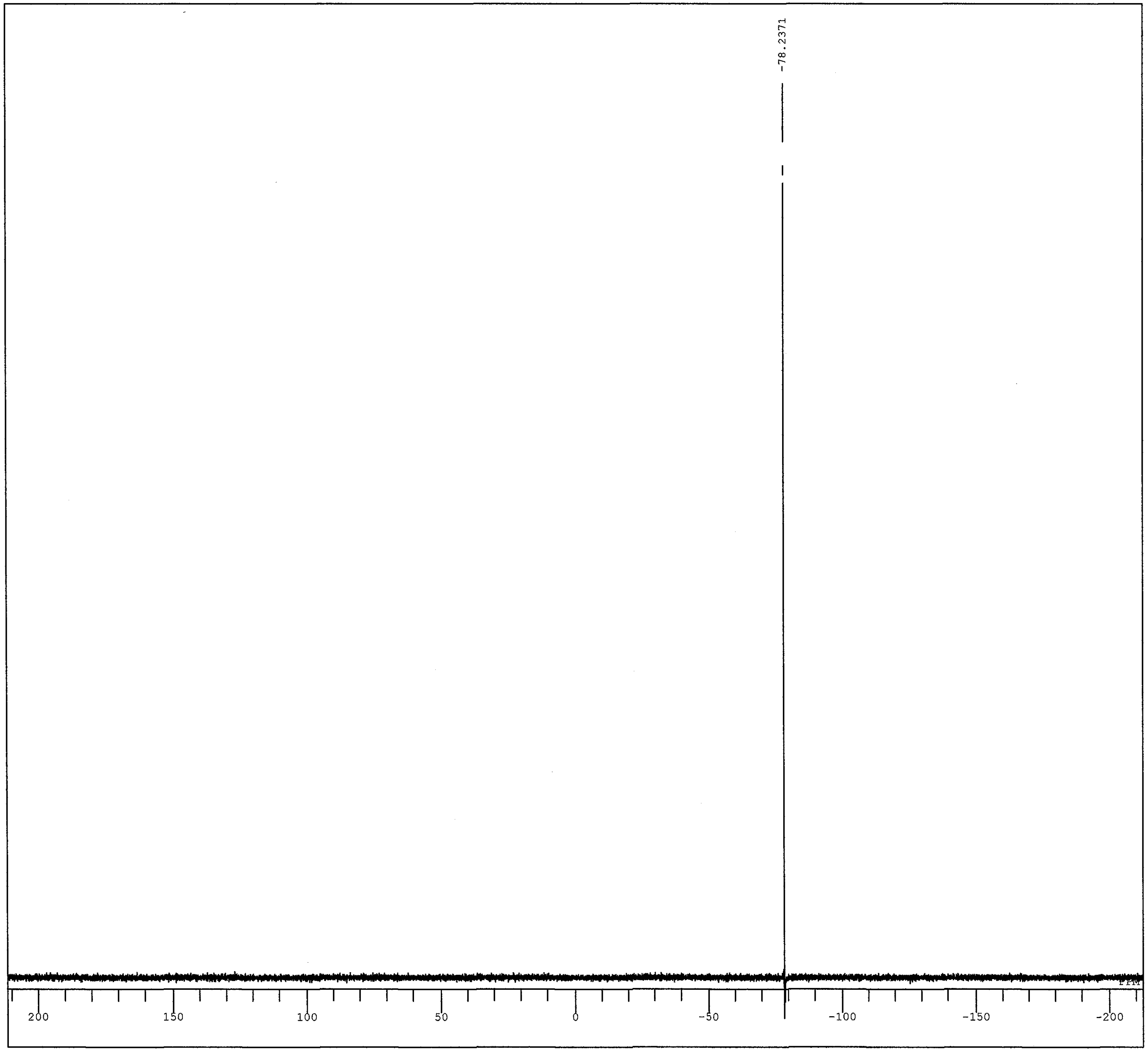


```
DFILE  ozawa07-025_19F.jdf
COMNT  yne-tert, OH
DATIM  21-03-2015 11:41:16
OBNUC  19F
EXMOD  proton.jxp
OBFRQ        368.64 MHz
OBSET          7.63 KHz
OBFIN          2.85 Hz
POINT         16384
FREQU     195312.50 Hz
SCANS             4
ACQTM        0.0839 sec
PD           5.0000 sec
PW1            3.90 usec
IRNUC  19F
CTEMP          25.1 c
SLVNT  CDCL3
EXREF       -164.90 ppm
BF             0.12 Hz
RGAIN            50
```

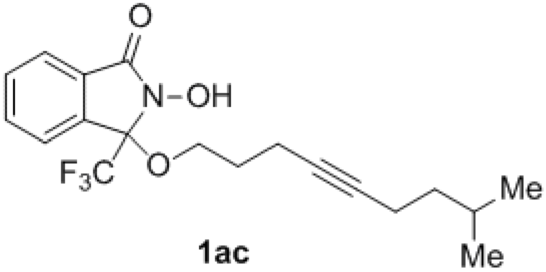

1ac

yne-tert, [0]

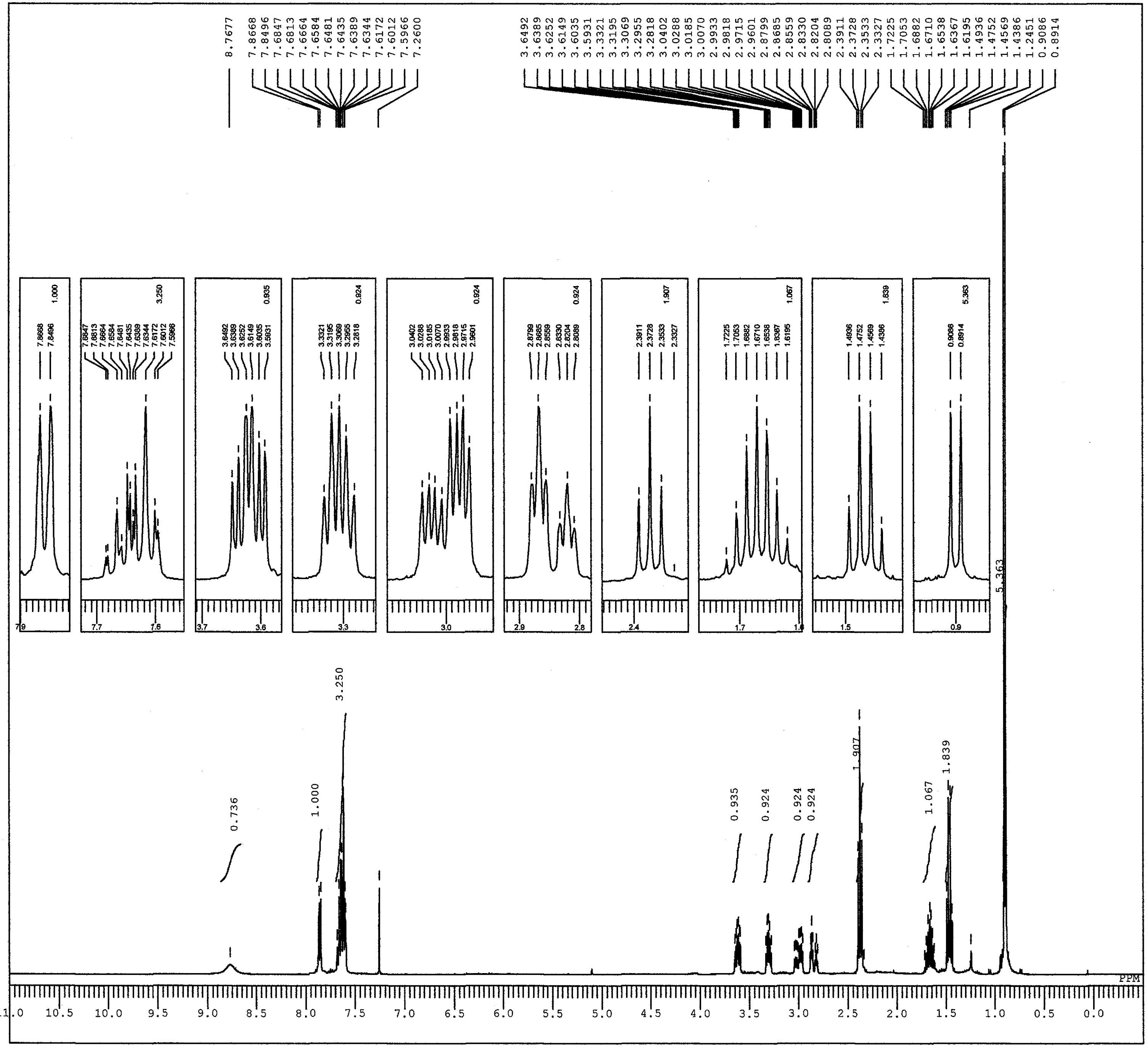

DFILE	ozawa07-004_1H.jdf
COMNT	yne-tert, [0]
DATIM	21-03-2015 07:31:59
OBNUC	1H
EXMOD	proton.jxp
OBFRQ	391.78 MHz
OBSET	8.51 KHz
OBFIN	3.34 Hz
POINT	16384
FREQU	7348.62 Hz
SCANS	4
ACQTM	2.2295 sec
PD	5.0000 sec
PW1	4.99 usec
IRNUC	1H
CTEMP	25.0 c
SLVNT	CDCL3
EXREF	7.26 ppm
BF	0.12 Hz
RGAIN	32

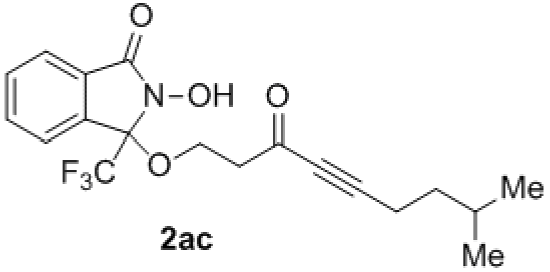

2ac

yne-tert, [O]

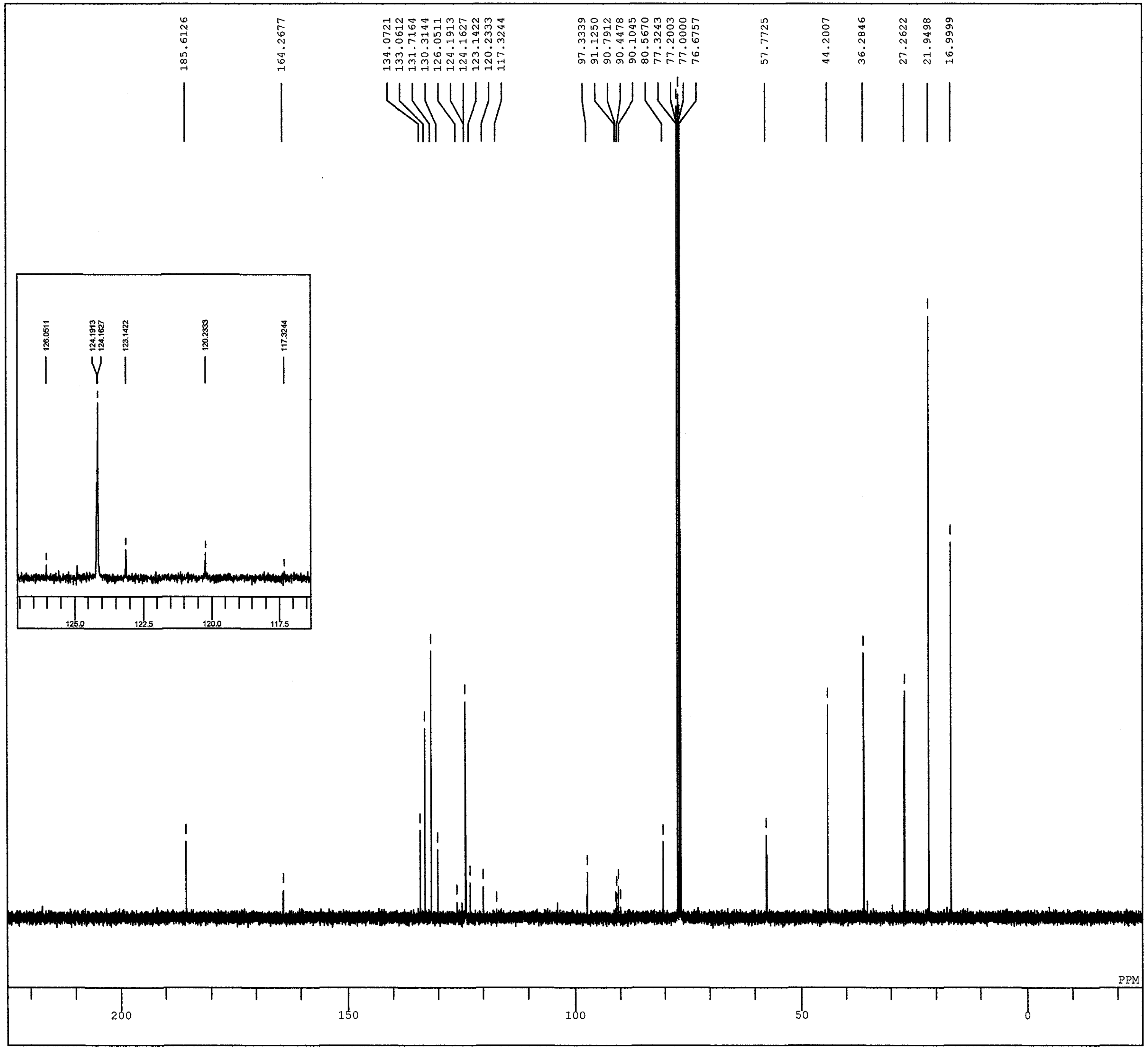

DFILE ozawa07-004_13C.jdf
COMNT yne-tert, [O]
DATIM 21-03-2015 07:33:01
OBNUC 13C
EXMOD carbon.jxp
OBFRQ 98.52 MHz
OBSET 4.64 KHz
OBFIN 8.74 Hz
POINT 32767
FREQU 30788.18 Hz
SCANS 916
ACQTM 1.0643 sec
PD 2.0000 sec
PW1 3.16 usec
IRNUC 1H
CTEMP 25.3 c
SLVNT CDCL3
EXREF 77.00 ppm
BF 0.12 Hz
RGAIN 60

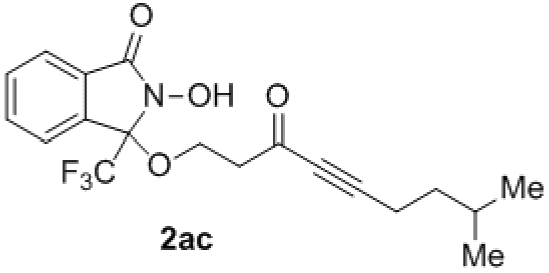

2ac

yne-tert, [0]

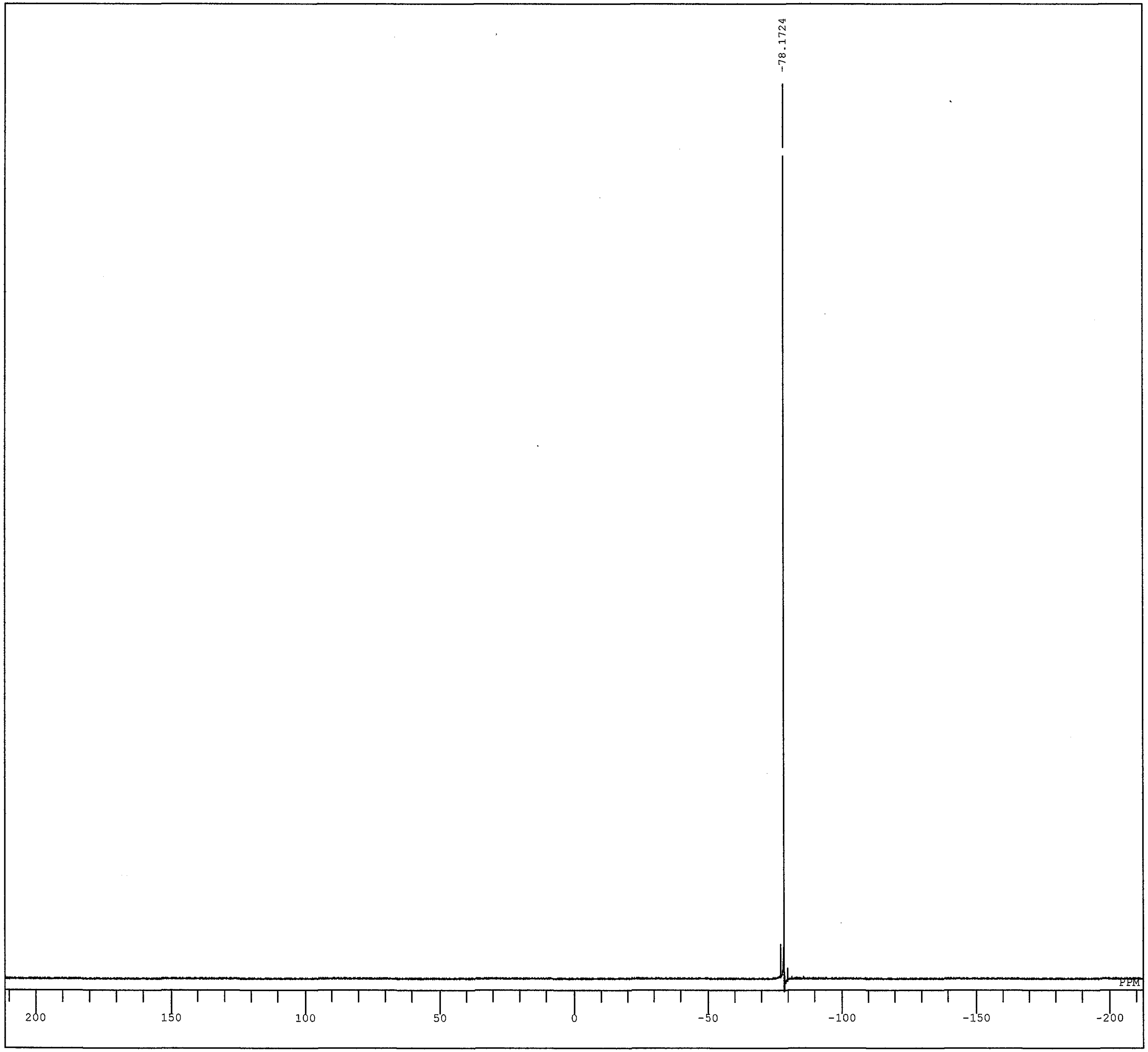


```
DFILE  ozawa07-004_19F.jdf
COMNT  yne-tert, [0]
DATIM  21-03-2015 08:21:33
OBNUC  19F
EXMOD  proton.jxp
OBFRQ        368.64 MHz
OBSET          7.63 KHz
OBFIN          2.85 Hz
POINT         16384
FREQU     195312.50 Hz
SCANS             4
ACQTM        0.0839 sec
PD           5.0000 sec
PW1            3.90 usec
IRNUC  19F
CTEMP          25.2 c
SLVNT  CDCL3
EXREF       -164.90 ppm
BF             0.12 Hz
RGAIN            50
```

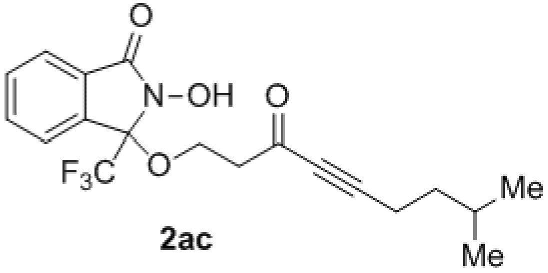

2ac

thiophene, PMB

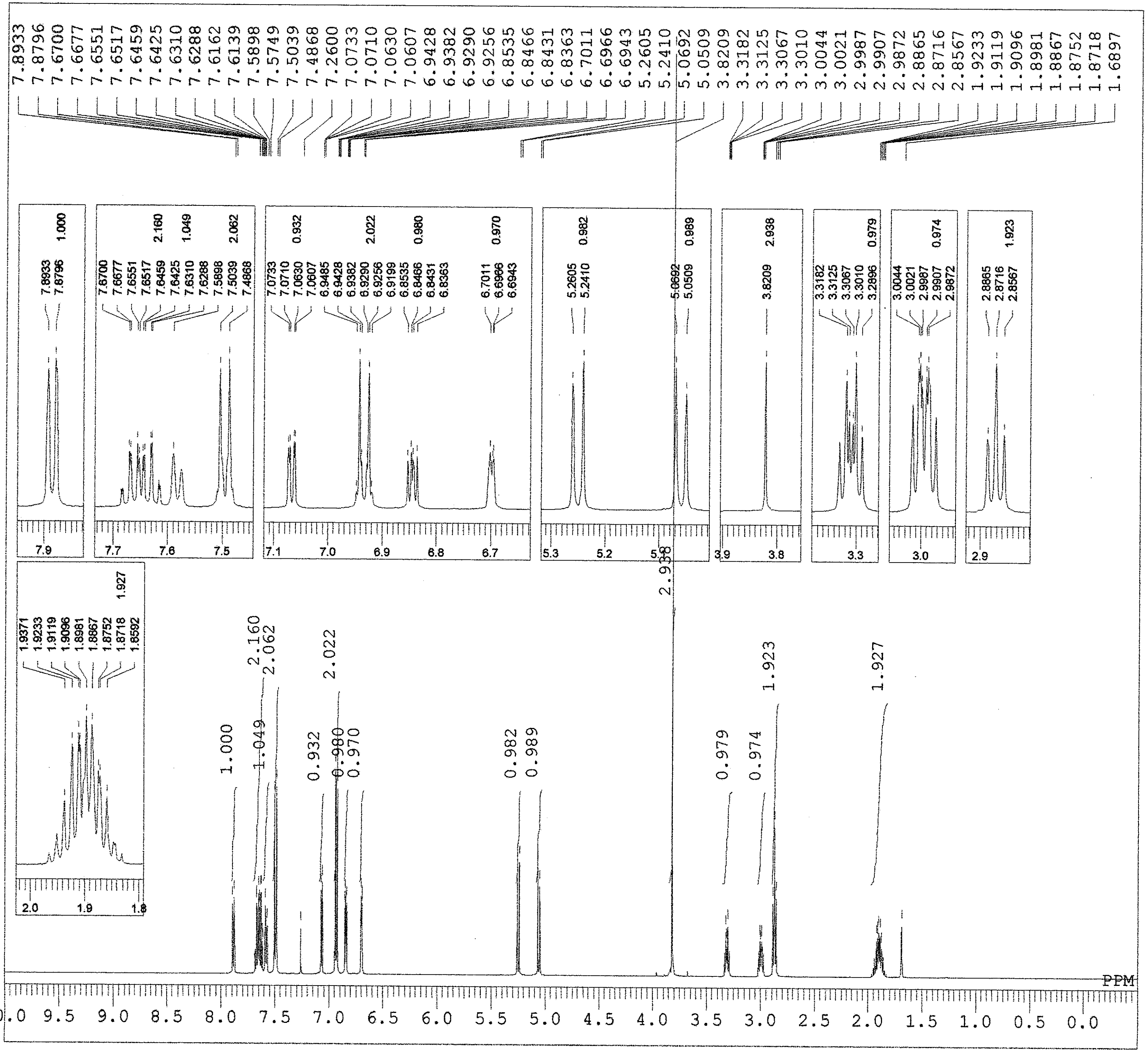

DFILE	ozawa05-046_1H.als
COMNT	thiophene, PMB
DATIM	2014-08-15 12:56:25
OBNUC	1H
EXMOD	proton.jxp
OBFRQ	500.16 MHz
OBSET	2.41 KHz
OBFIN	6.01 Hz
POINT	13107
FREQU	7507.51 Hz
SCANS	4
ACQTM	1.7459 sec
PD	5.0000 sec
PW1	5.55 usec
IRNUC	1H
CTEMP	26.2 c
SLVNT	CDCL3
EXREF	7.26 ppm
BF	0.12 Hz
RGAIN	30

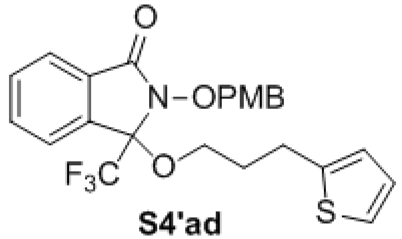

S4'ad

thiophene, PMB

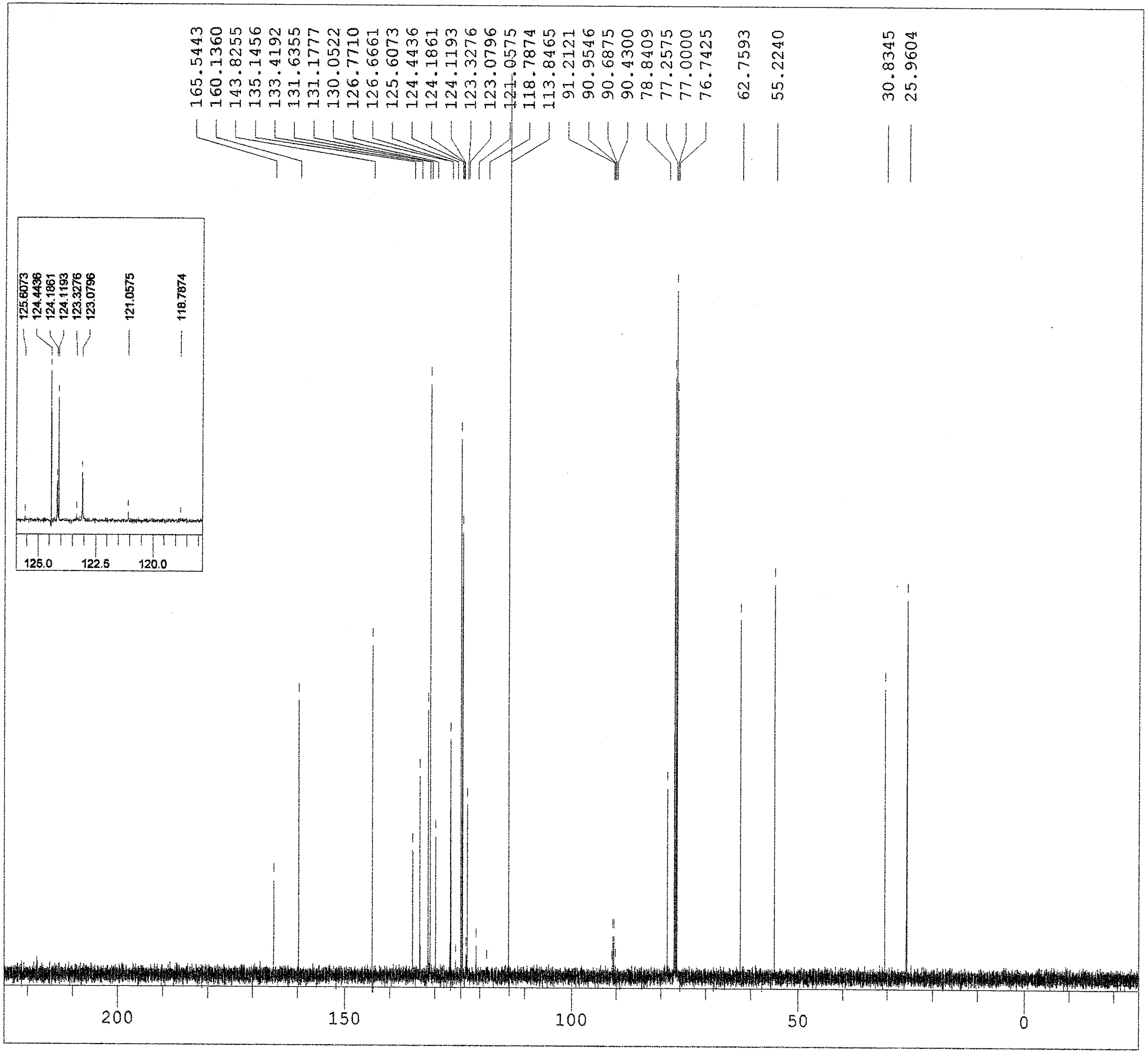

DFILE ozawa05-046_13C.jdf
COMNT thiophene, PMB
DATIM 2014-08-15 12:57:28
OBNUC 13C
EXMOD carbon.jxp
OBFRQ 125.77 MHz
OBSET 7.87 KHz
OBFIN 4.21 Hz
POINT 32767
FREQU 39308.18 Hz
SCANS 180
ACQTM 0.8336 sec
PD 3.0000 sec
PW1 3.40 usec
IRNUC 1H
CTEMP 26.4 c
SLVNT CDCL3
EXREF 77.00 ppm
BF 0.12 Hz
RGAIN 60

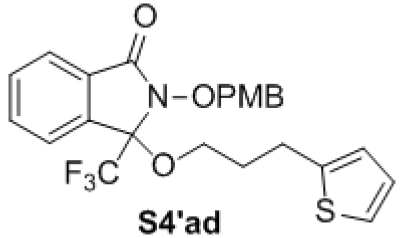

S4'ad

thiophene, PMB

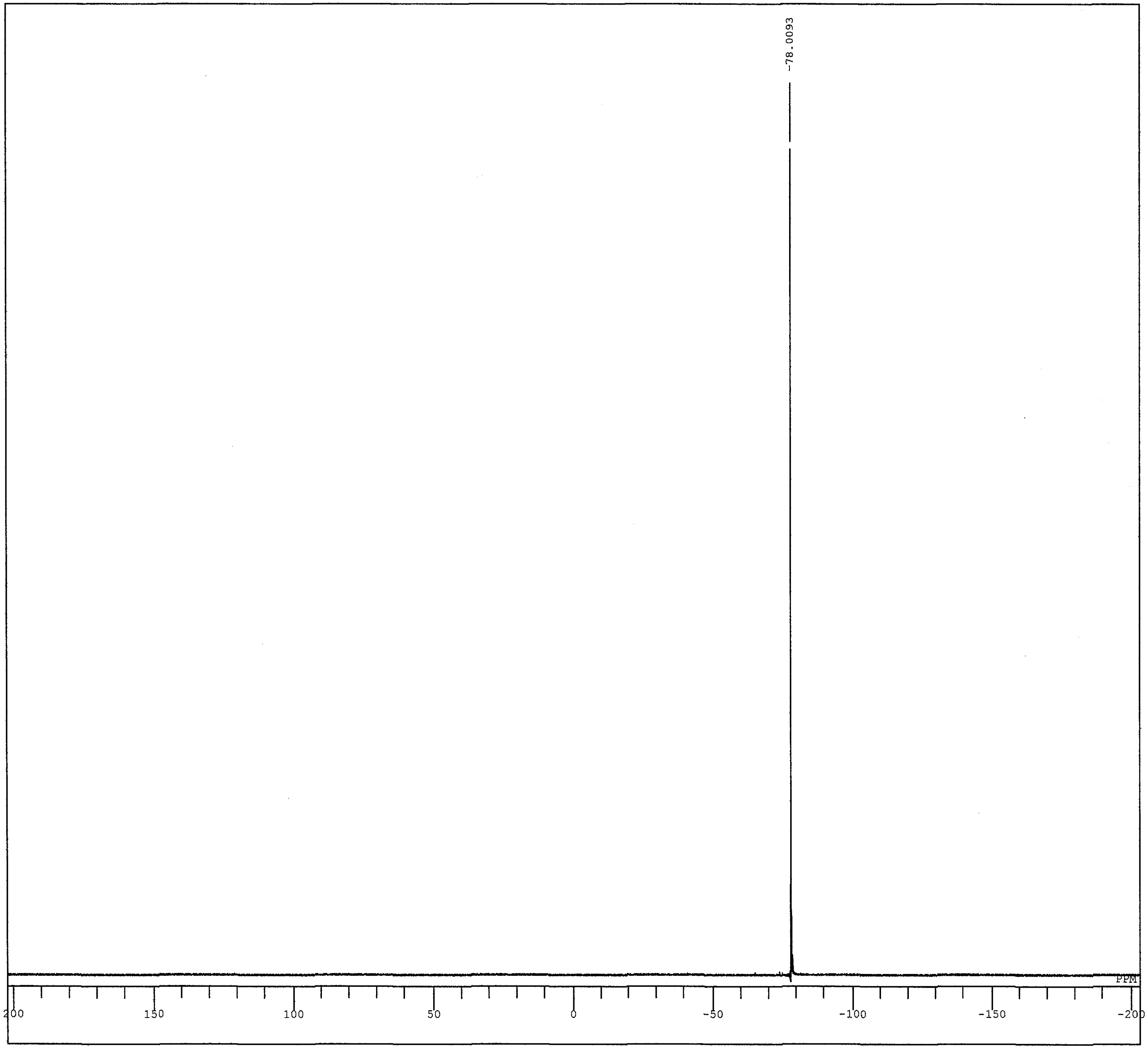

DFILE ozawa05-046_19F.jdf
COMNT thiophene, PMB
DATIM 15-08-2014 13:54:52
OBNUC 19F
EXMOD proton.jxp
OBFRQ 368.64 MHz
OBSET 7.63 KHz
OBFIN 2.85 Hz
POINT 16384
FREQU 186567.17 Hz
SCANS 4
ACQTM 0.0878 sec
PD 5.0000 sec
PW1 3.90 usec
IRNUC 19F
CTEMP 24.2 c
SLVNT CDCL3
EXREF -164.90 ppm
BF 0.12 Hz
RGAIN 50

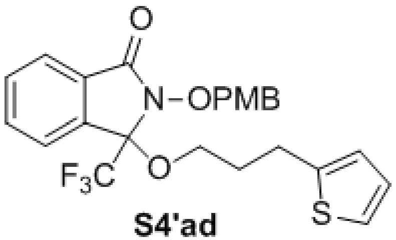

S4'ad

thiophene, OH

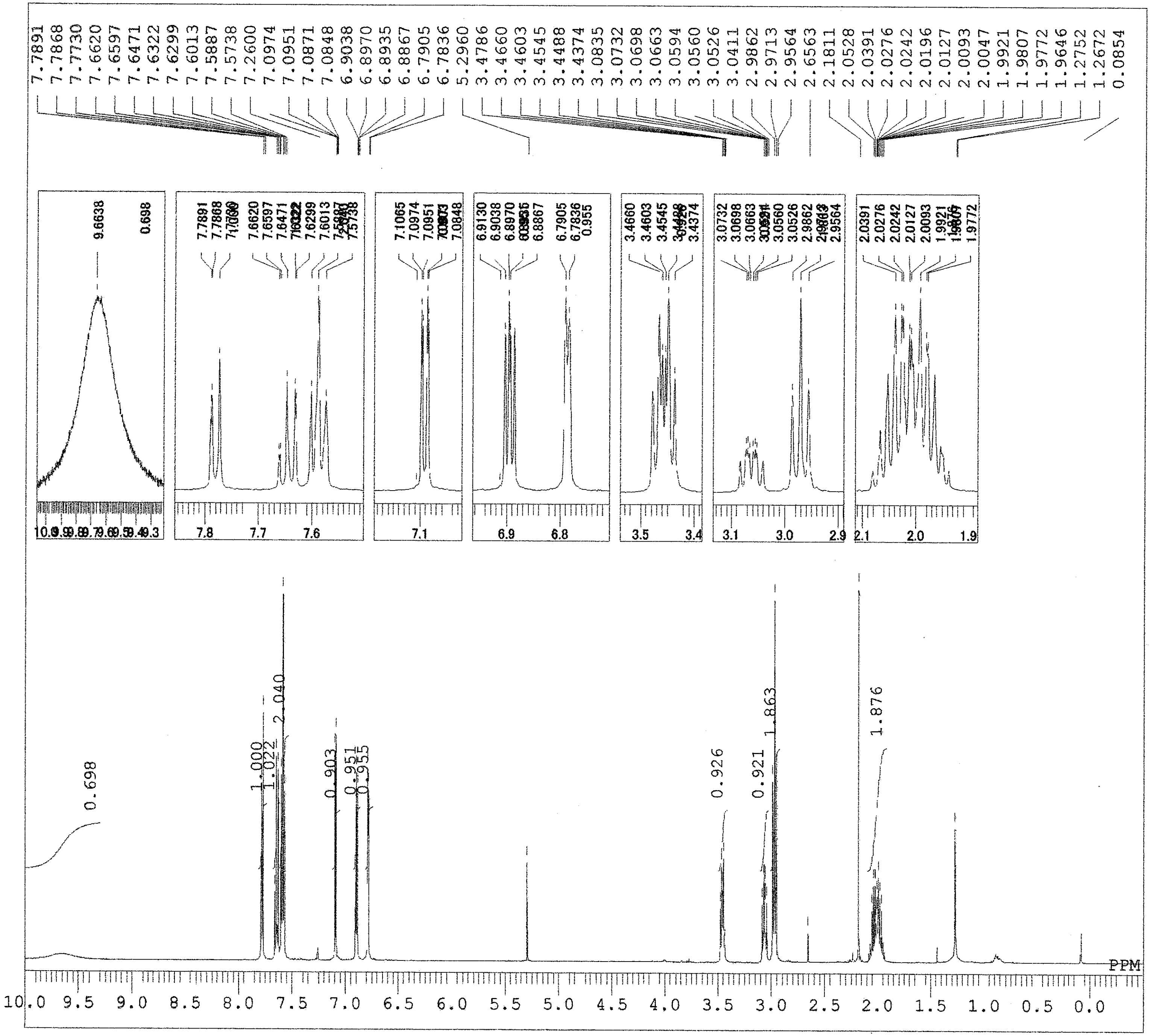

DFILE	ozawa05-076_1H.jdf
COMNT	thiophene, OH
DATIM	2014-07-03 17:14:10
OBNUC	1H
EXMOD	proton.jxp
OBFRQ	500.16 MHz
OBSET	2.41 KHz
OBFIN	6.01 Hz
POINT	16384
FREQU	9384.38 Hz
SCANS	4
ACQTM	1.7459 sec
PD	5.0000 sec
PW1	5.55 usec
IRNUC	1H
CTEMP	25.8 c
SLVNT	CDCL3
EXREF	7.26 ppm
BF	0.12 Hz
RGAIN	34

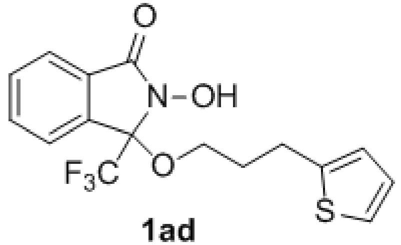

1ad

thiophene, OH

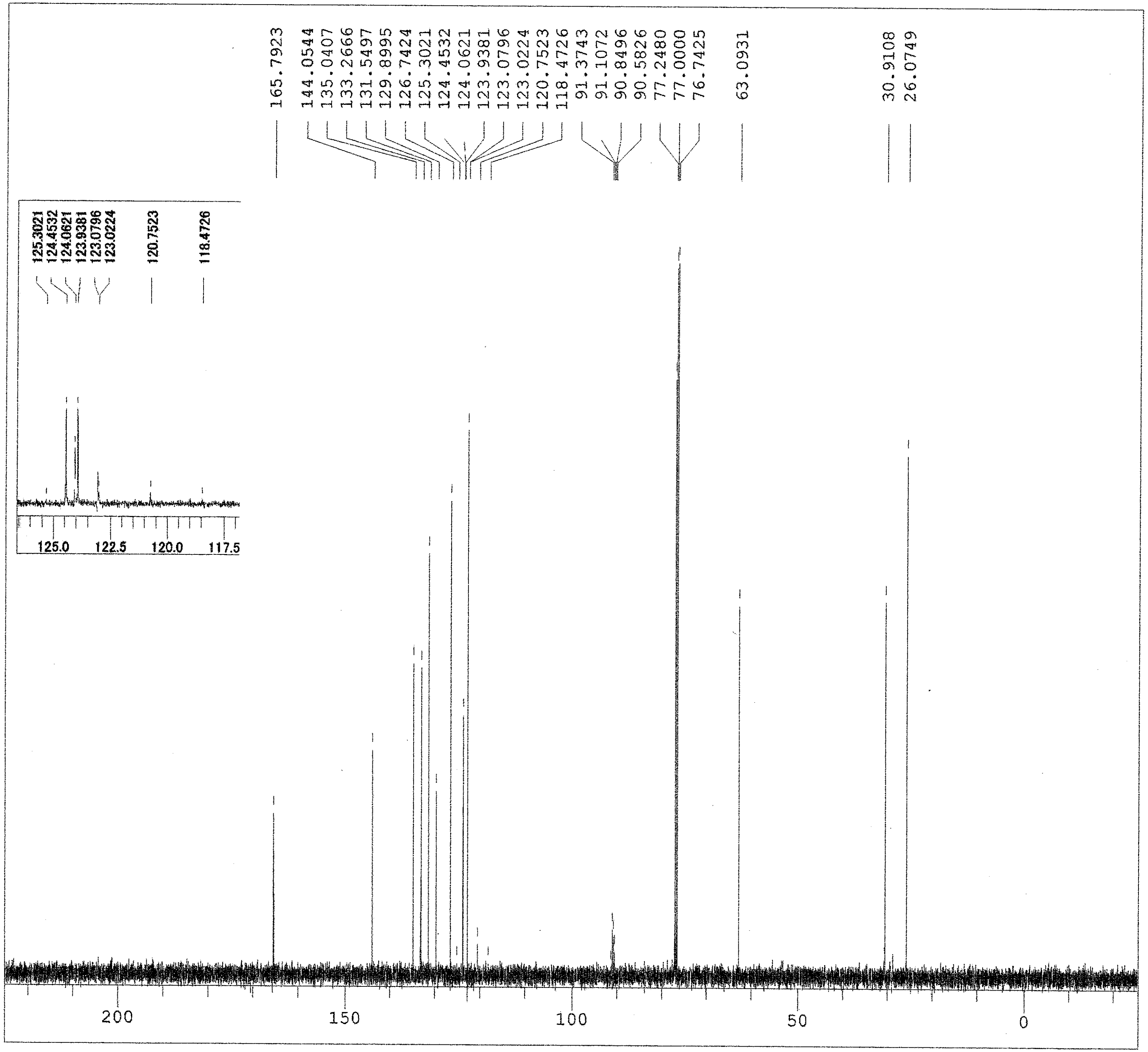

DFILE	ozawa05-076_13C.jdf
COMNT	thiophene, OH
DATIM	2014-07-03 17:15:23
OBNUC	13C
EXMOD	carbon.jxp
OBFRQ	125.77 MHz
OBSET	7.87 KHz
OBFIN	4.21 Hz
POINT	32767
FREQU	39308.18 Hz
SCANS	152
ACQTM	0.8336 sec
PD	3.0000 sec
PW1	3.40 usec
IRNUC	1H
CTEMP	25.9 c
SLVNT	CDCL3
EXREF	77.00 ppm
BF	0.12 Hz
RGAIN	60

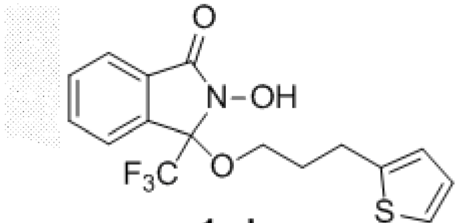

1ad

thiophene, OH

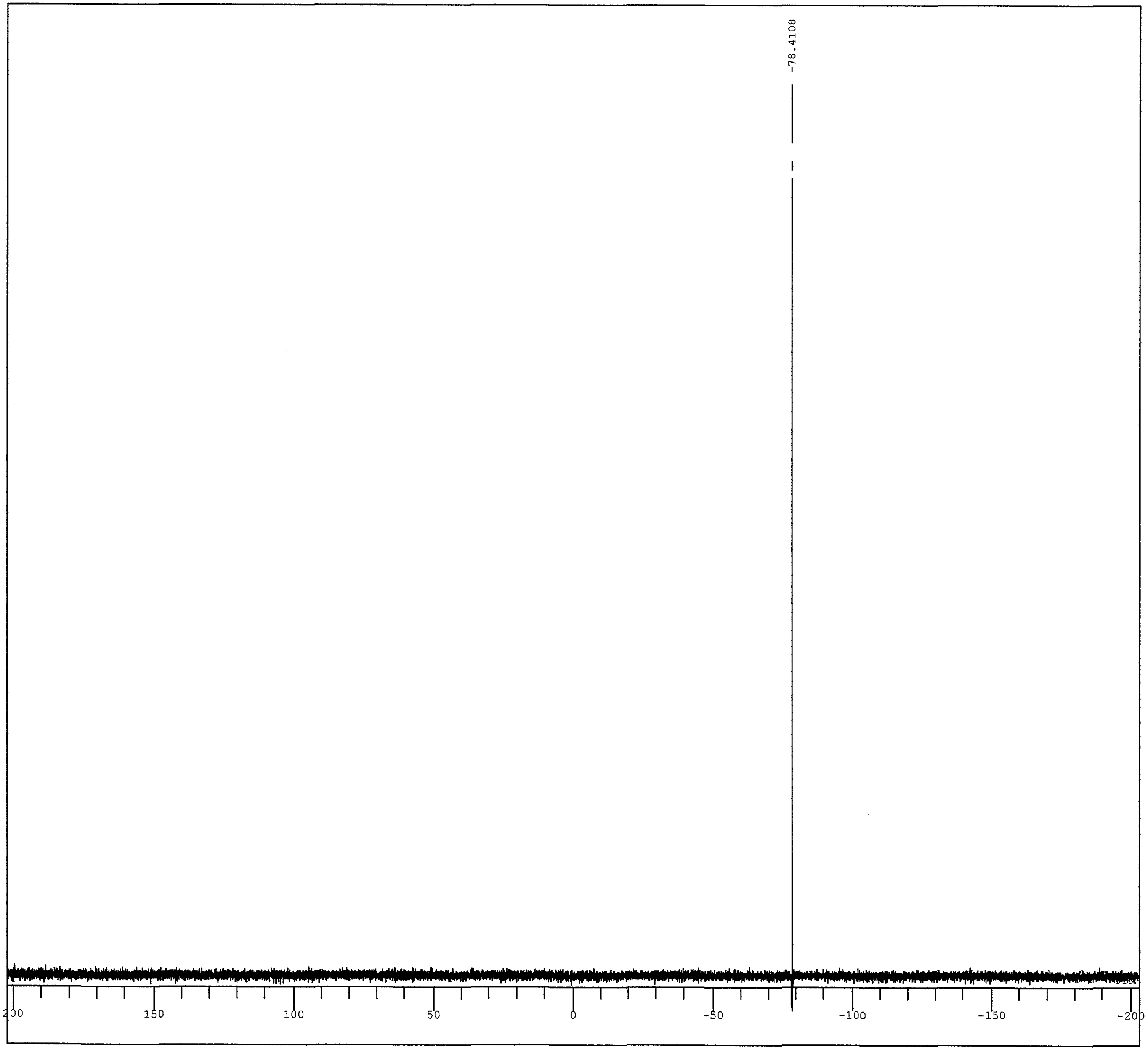

DFILE ozawa05-076_19F.jdf
COMNT thiophene, OH
DATIM 15-08-2014 20:00:49
OBNUC 19F
EXMOD proton.jxp
OBFRQ 368.64 MHz
OBSET 7.63 KHz
OBFIN 2.85 Hz
POINT 16384
FREQU 186567.17 Hz
SCANS 4
ACQTM 0.0878 sec
PD 5.0000 sec
PW1 3.90 usec
IRNUC 19F
CTEMP 24.5 c
SLVNT CDCL3
EXREF -164.90 ppm
BF 0.12 Hz
RGAIN 48

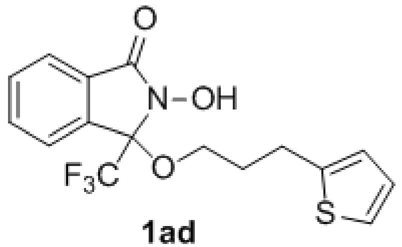

1ad

thiophene, [O]

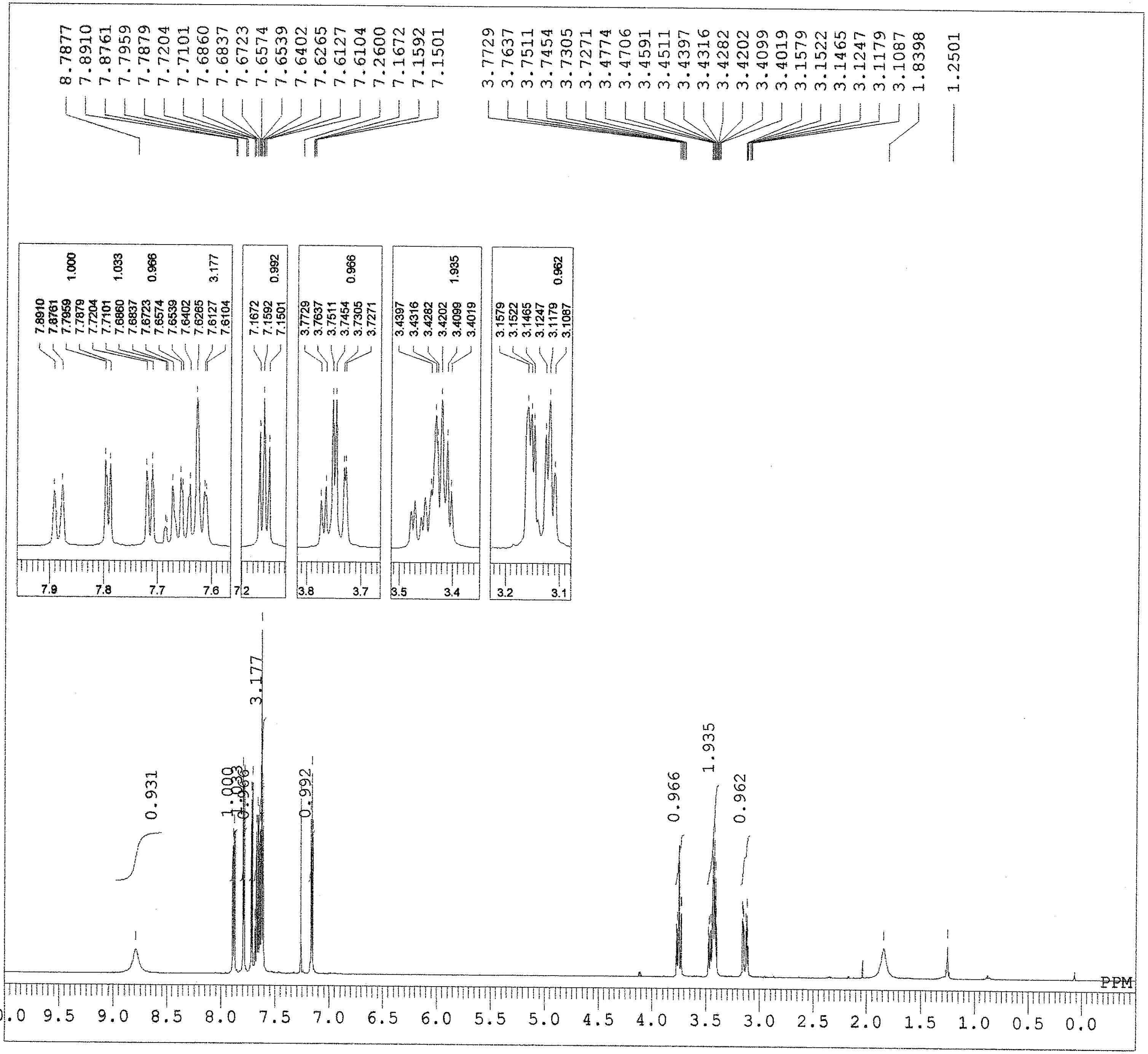

DFILE	ozawa05-095_2_1H.jdf
COMNT	thiophene, [O]
DATIM	2014-08-16 15:25:08
OBNUC	1H
EXMOD	proton.jxp
OBFRQ	500.16 MHz
OBSET	2.41 KHz
OBFIN	6.01 Hz
POINT	16384
FREQU	9384.38 Hz
SCANS	8
ACQTM	1.7459 sec
PD	5.0000 sec
PW1	5.55 usec
IRNUC	1H
CTEMP	26.2 c
SLVNT	CDCL3
EXREF	7.26 ppm
BF	0.12 Hz
RGAIN	38

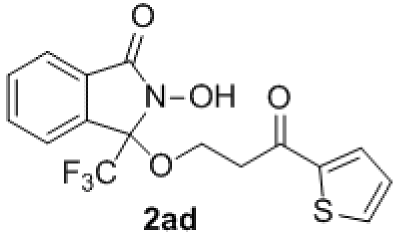

2ad

thiophene, [0]

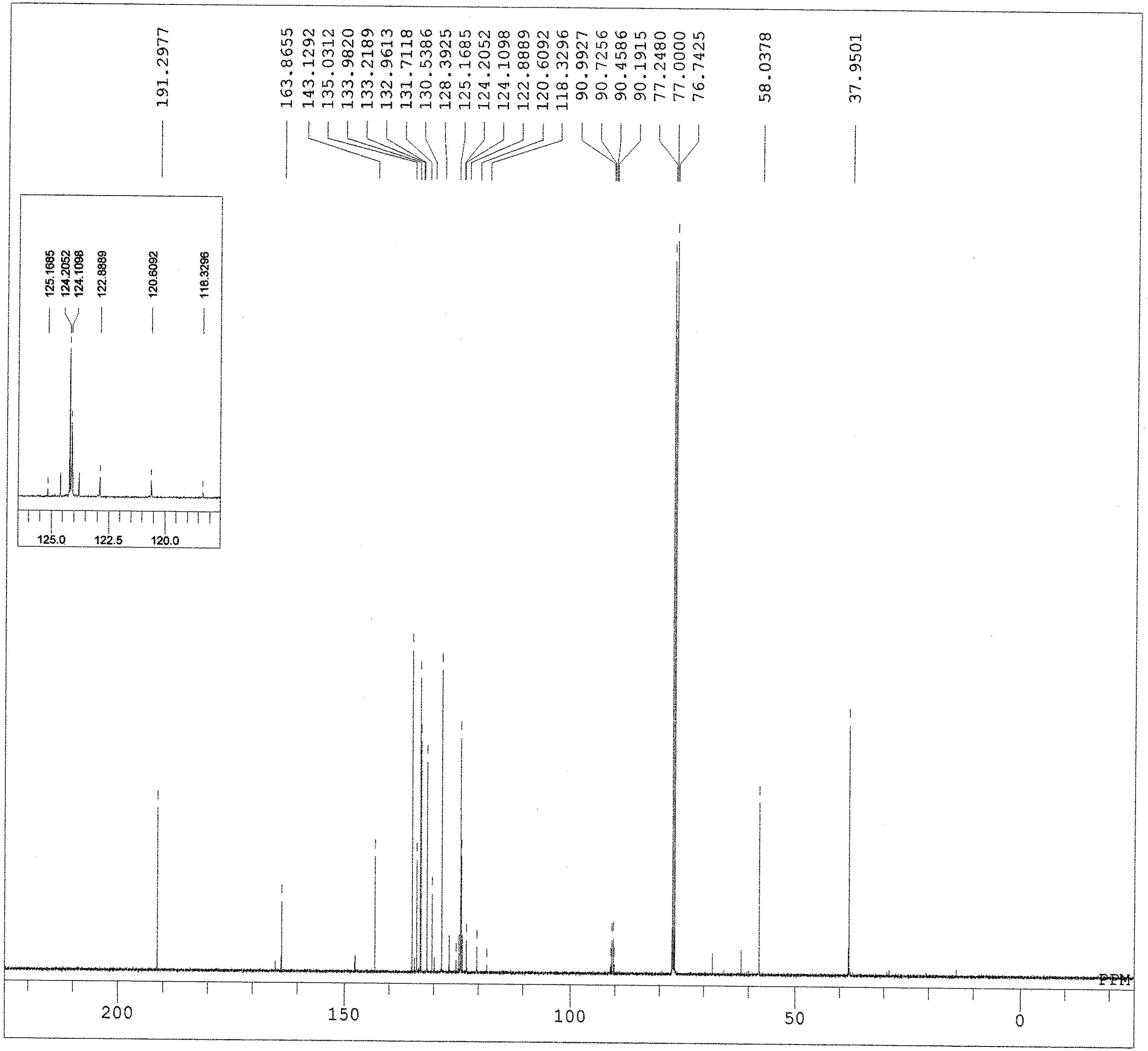

DFILE	ozawa05-095_2_13C.jdf
COMNT	thiophene, [0]
DATIM	2014-08-15 22:28:45
OBNUC	13C
EXMOD	carbon.jxp
OBFRQ	125.77 MHz
OBSET	7.87 KHz
OBFIN	4.21 Hz
POINT	32767
FREQU	39308.18 Hz
SCANS	10000
ACQTM	0.8336 sec
PD	3.0000 sec
PW1	3.40 usec
IRNUC	1H
CTEMP	26.6 c
SLVNT	CDCL3
EXREF	77.00 ppm
BF	0.12 Hz
RGAIN	60

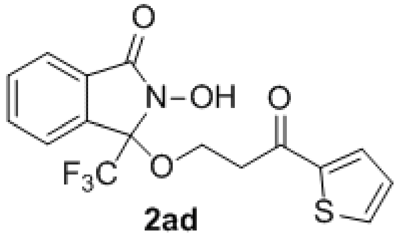

2ad

thiophene, [0]

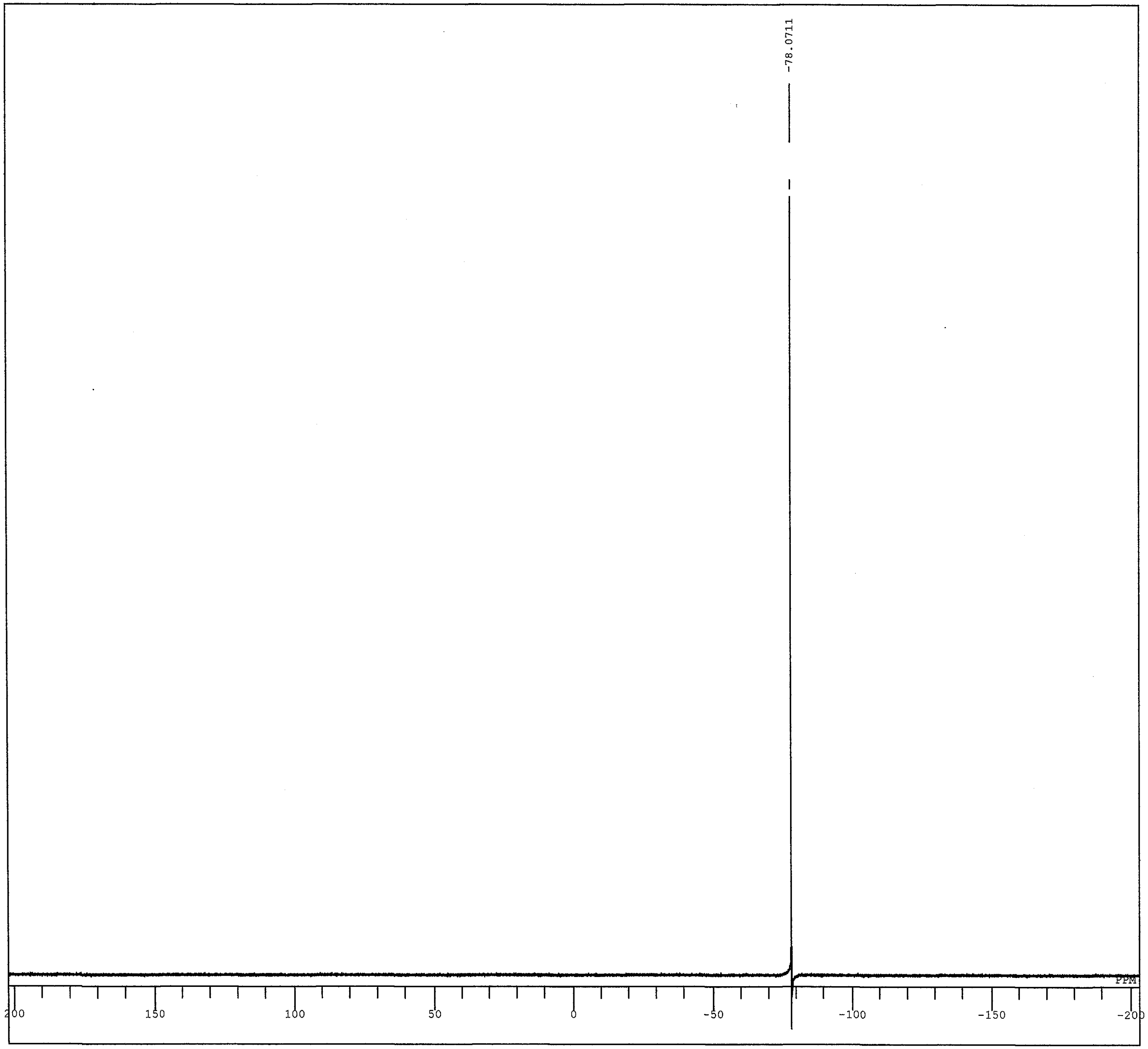

DFILE ozawa05-095_2_19F.jdf
COMNT thiophene, [0]
DATIM 07-07-2014 18:32:55
OBNUC 19F
EXMOD proton.jxp
OBFRQ 368.64 MHz
OBSET 7.63 KHz
OBFIN 2.85 Hz
POINT 16384
FREQU 186567.17 Hz
SCANS 4
ACQTM 0.0878 sec
PD 5.0000 sec
PW1 3.90 usec
IRNUC 19F
CTEMP 24.3 c
SLVNT CDCL3
EXREF -164.90 ppm
BF 0.12 Hz
RGAIN 50

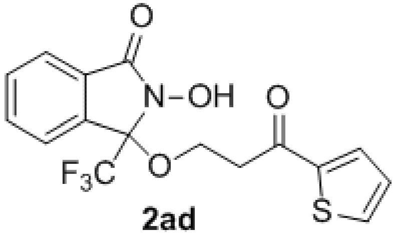

2ad

Ts-indol, Bn

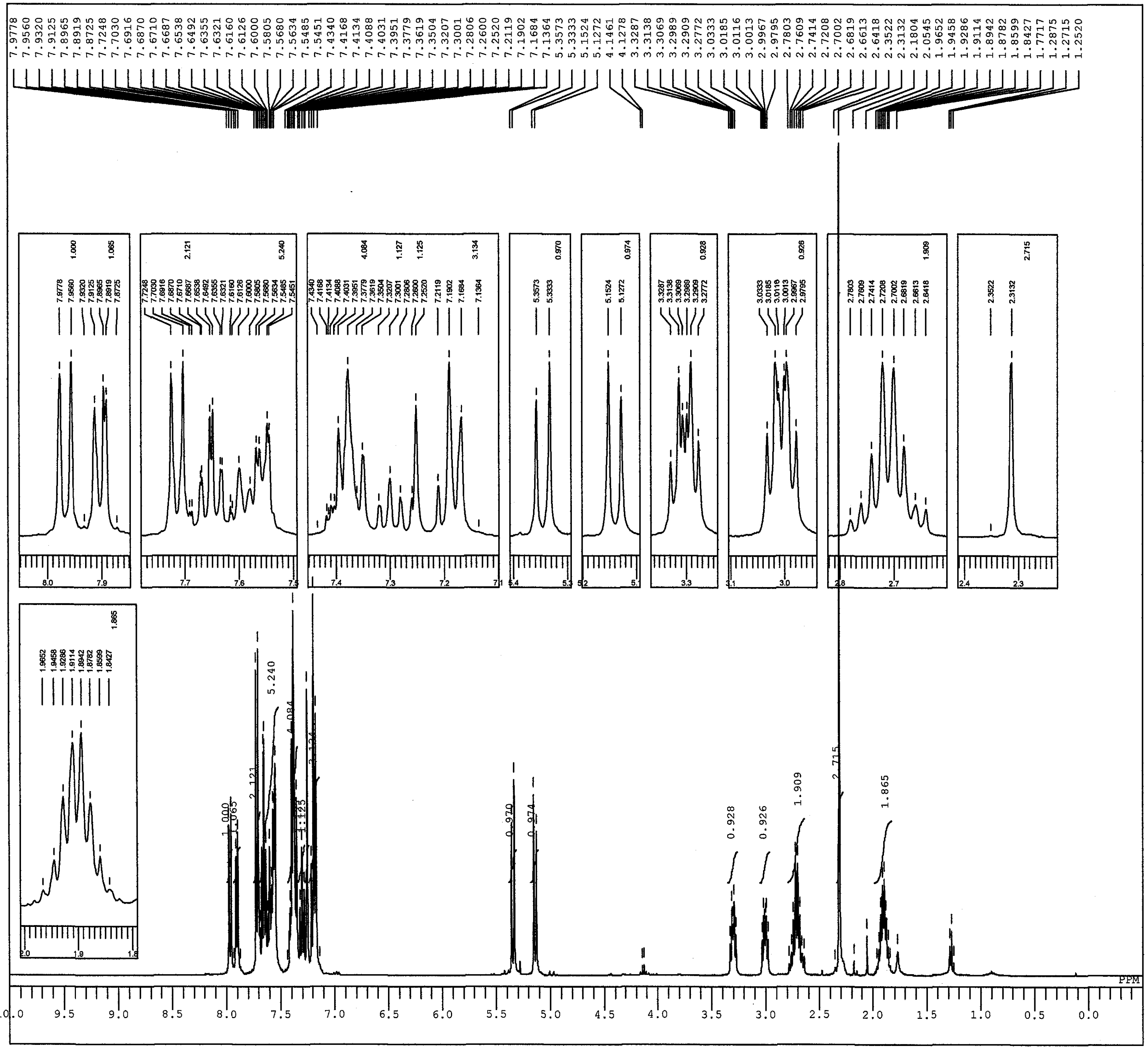

DFILE ozawa05-122_1H.jdf
COMNT Ts-indol, Bn
DATIM 04-08-2014 16:43:13
OBNUC 1H
EXMOD proton.jxp
OBFRQ 391.78 MHz
OBSET 8.51 KHz
OBFIN 3.34 Hz
POINT 16384
FREQU 7348.62 Hz
SCANS 4
ACQTM 2.2295 sec
PD 5.0000 sec
PW1 5.25 usec
IRNUC 1H
CTEMP 24.2 c
SLVNT CDCL3
EXREF 7.26 ppm
BF 0.12 Hz
RGAIN 26

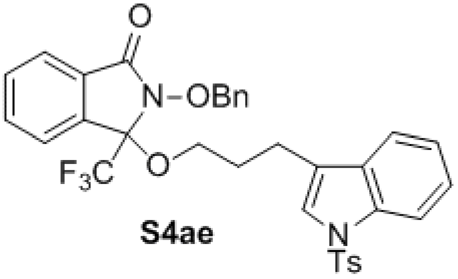

S4ae

Ts-indol, Bn

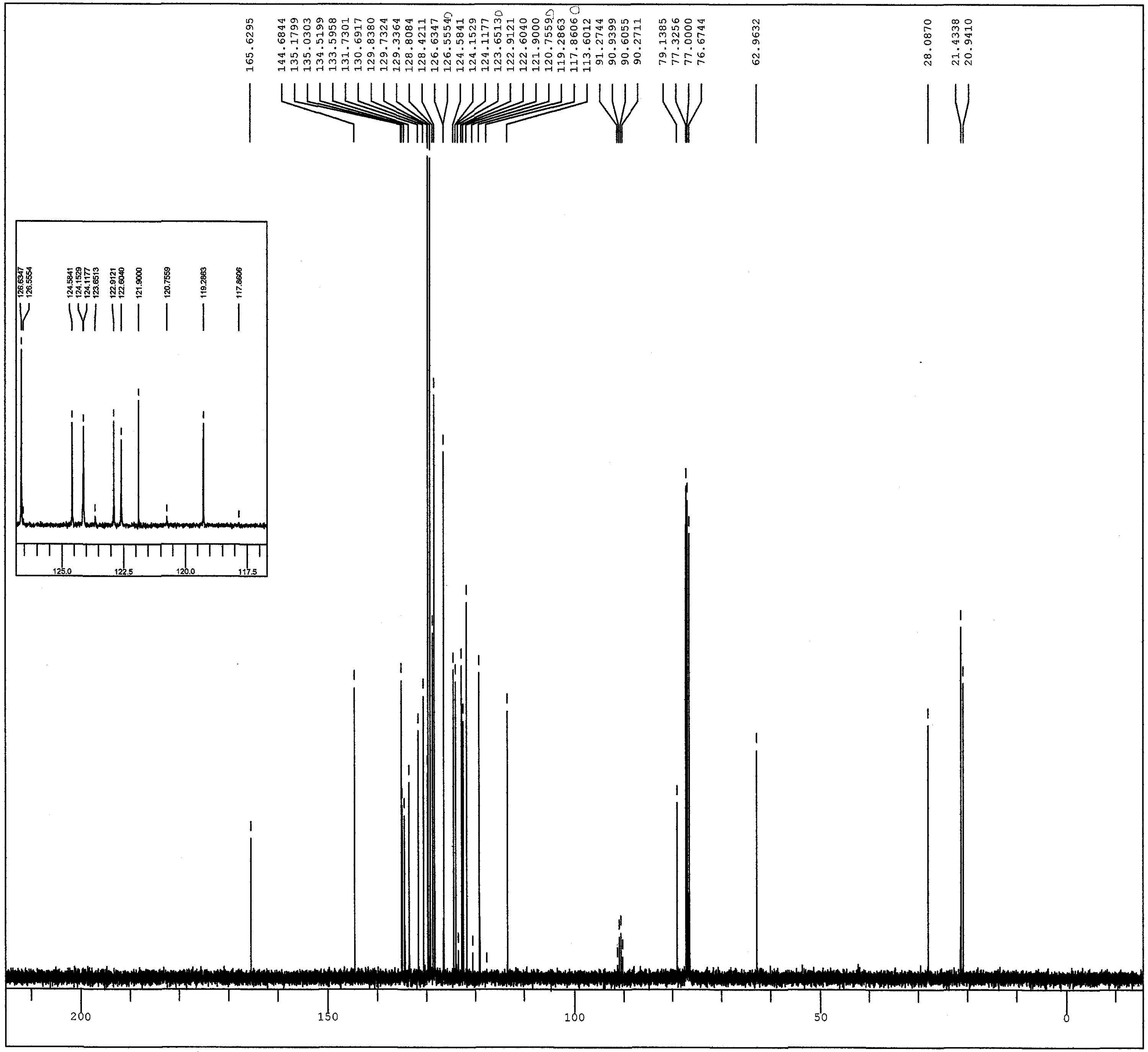

DFILE ozawa05-122_13C.jdf
COMNT Ts-indol, Bn
DATIM 04-08-2014 16:44:20
OBNUC 13C
EXMOD carbon.jxp
OBFRQ 98.52 MHz
OBSET 4.64 KHz
OBFIN 8.74 Hz
POINT 32767
FREQU 28409.09 Hz
SCANS 200
ACQTM 1.1534 sec
PD 3.0000 sec
PW1 3.00 usec
IRNUC 1H
CTEMP 24.5 c
SLVNT CDCL3
EXREF 77.00 ppm
BF 0.12 Hz
RGAIN 60

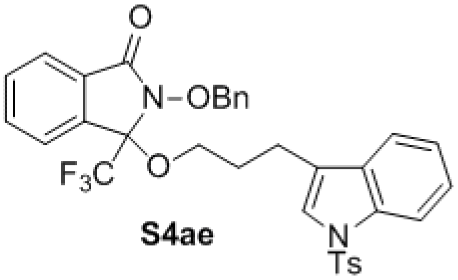

S4ae

Ts-indol, Bn

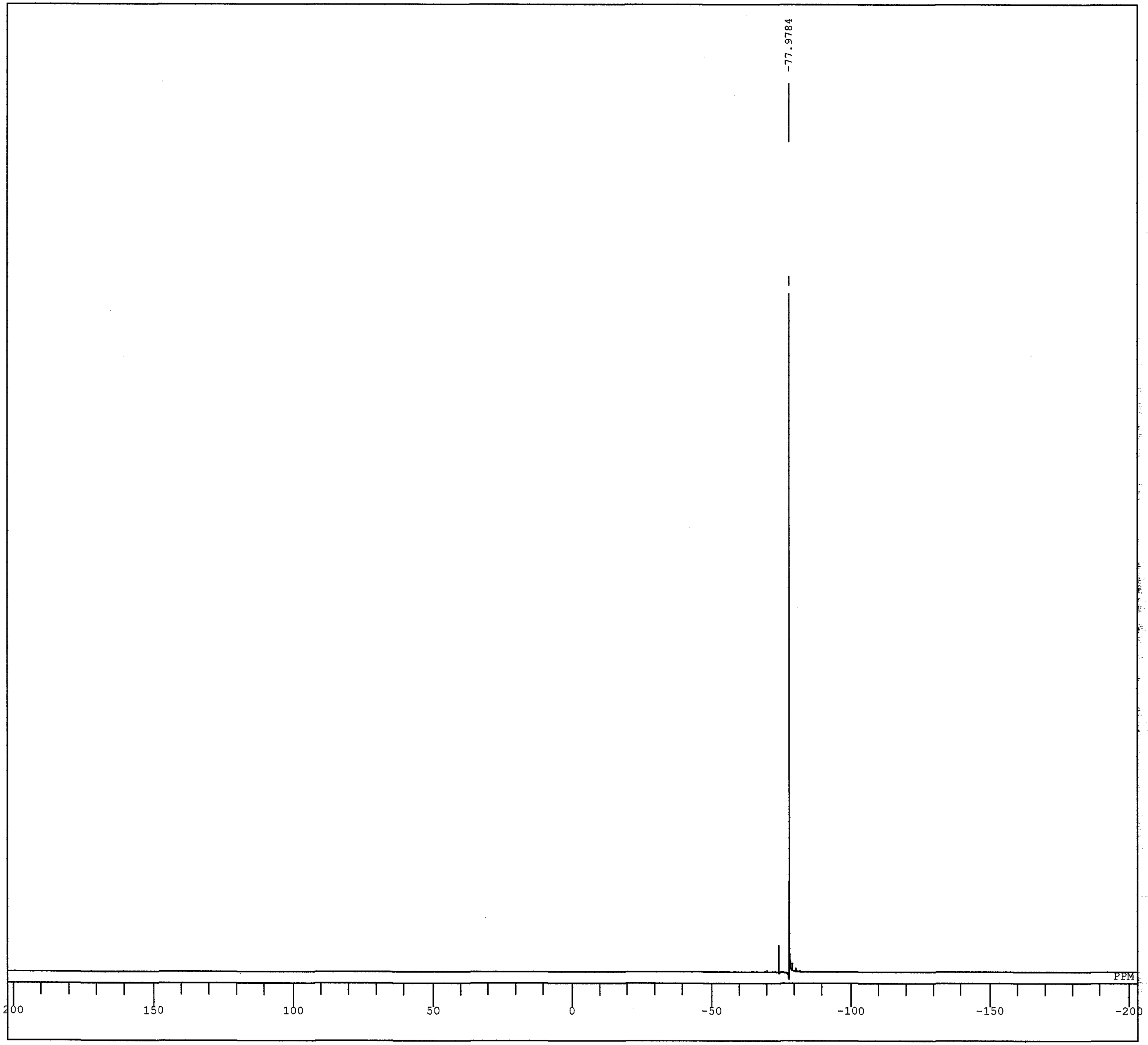

DFILE ozawa05-122_19F.jdf
COMNT Ts-indol, Bn
DATIM 04-08-2014 16:40:51
OBNUC 19F
EXMOD proton.jxp
OBFRQ 368.64 MHz
OBSET 7.63 KHz
OBFIN 2.85 Hz
POINT 16384
FREQU 186567.17 Hz
SCANS 4
ACQTM 0.0878 sec
PD 5.0000 sec
PW1 3.90 usec
IRNUC 19F
CTEMP 24.2 c
SLVNT CDCL3
EXREF -164.90 ppm
BF 0.12 Hz
RGAIN 44

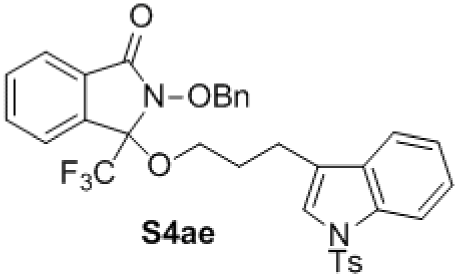

S4ae

Ts-indole, OH

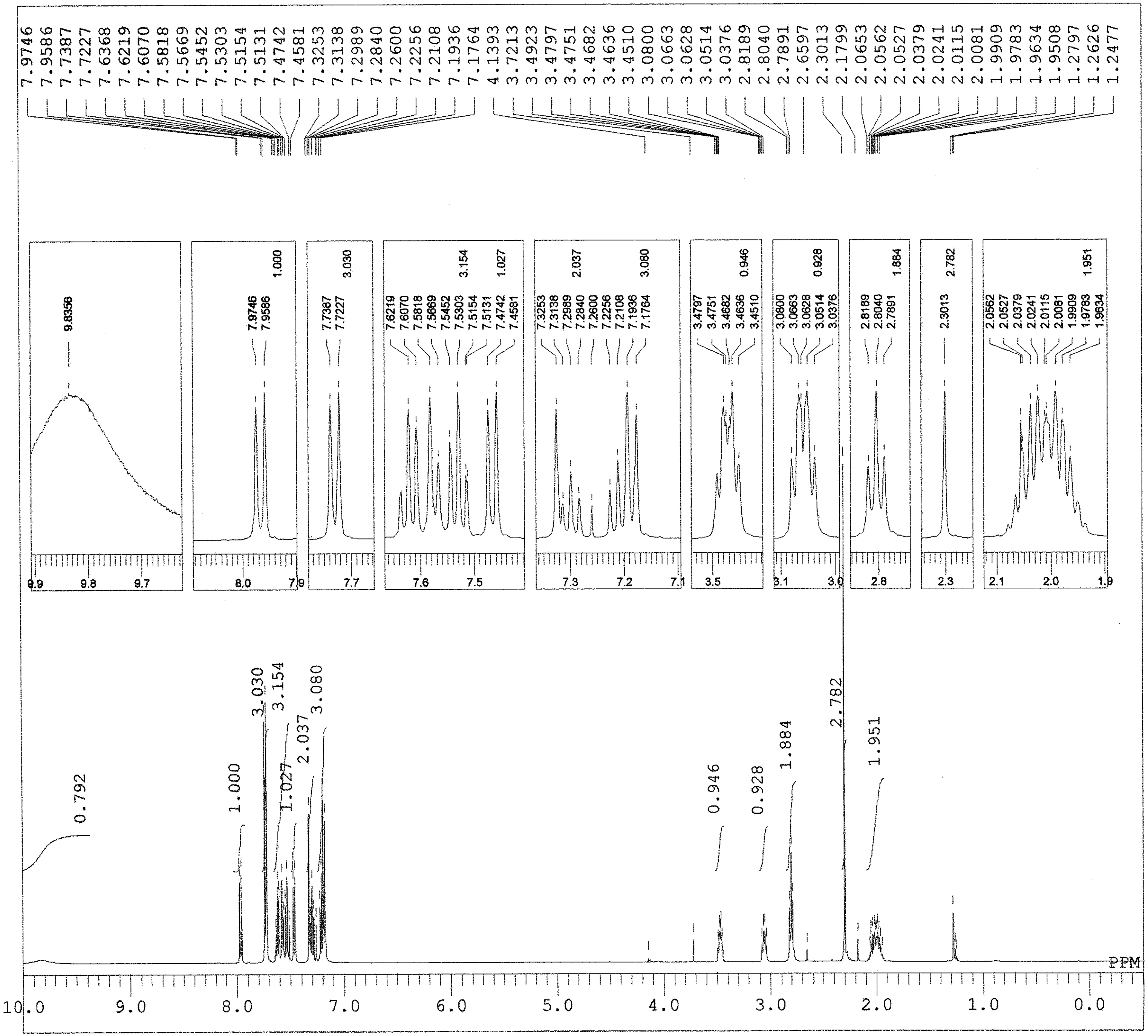

DFILE	ozawa05-123_1H.als
COMNT	Ts-indole, OH
DATIM	2014-08-15 18:19:53
OBNUC	1H
EXMOD	proton.jxp
OBFRQ	500.16 MHz
OBSET	2.41 KHz
OBFIN	6.01 Hz
POINT	13107
FREQU	7507.51 Hz
SCANS	4
ACQTM	1.7459 sec
PD	5.0000 sec
PW1	5.55 usec
IRNUC	1H
CTEMP	25.9 c
SLVNT	CDCL3
EXREF	7.26 ppm
BF	0.12 Hz
RGAIN	30

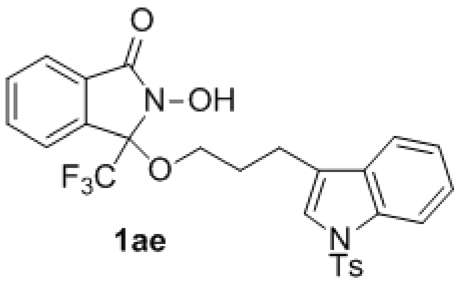

1ae

Ts-indole, OH

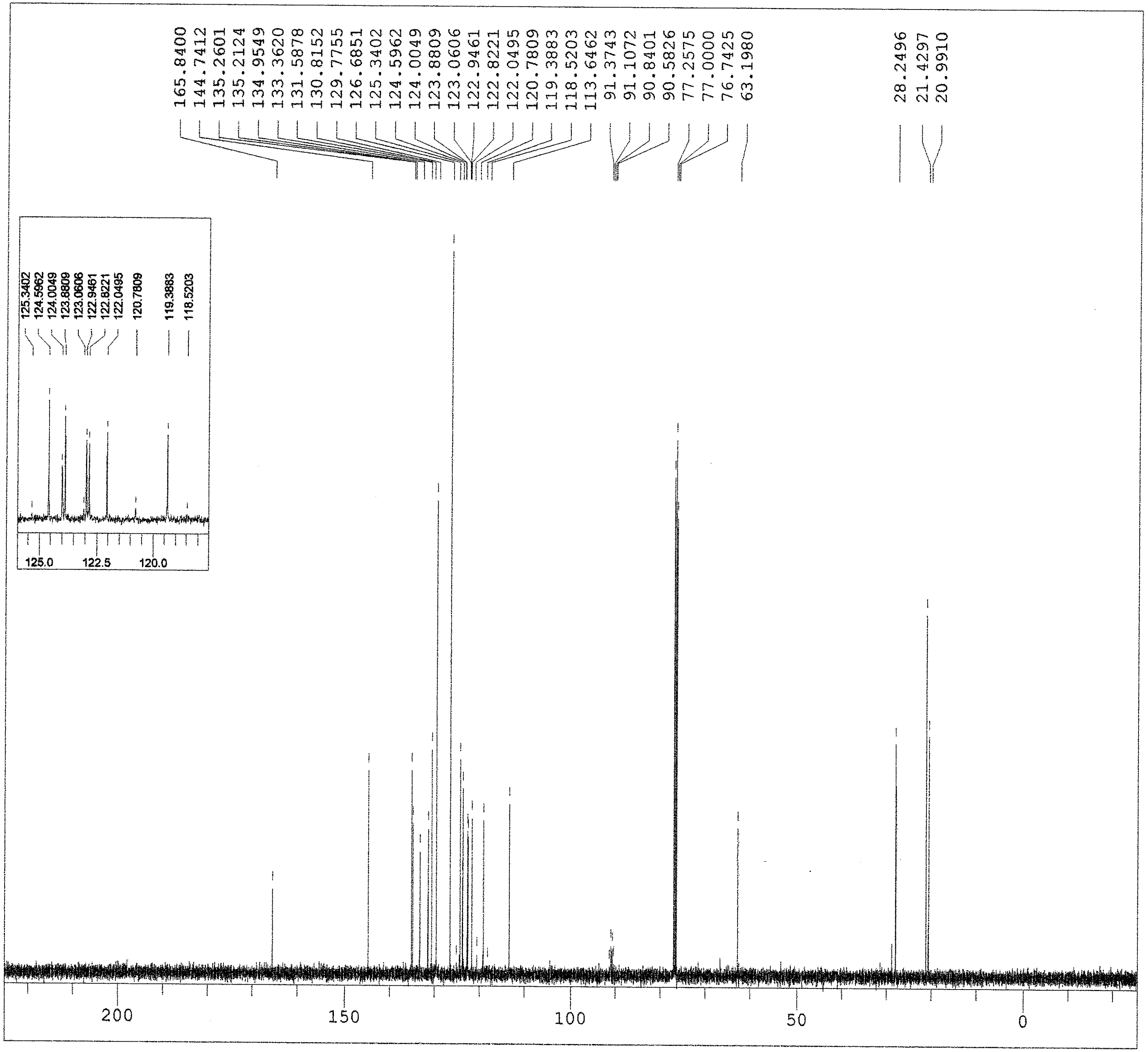

DFILE	ozawa05-123_13C.jdf
COMNT	Ts-indole, OH
DATIM	2014-08-15 18:20:56
OBNUC	13C
EXMOD	carbon.jxp
OBFRQ	125.77 MHz
OBSET	7.87 KHz
OBFIN	4.21 Hz
POINT	32767
FREQU	39308.18 Hz
SCANS	160
ACQTM	0.8336 sec
PD	3.0000 sec
PW1	3.40 usec
IRNUC	1H
CTEMP	26.5 c
SLVNT	CDCL3
EXREF	77.00 ppm
BF	0.12 Hz
RGAIN	60

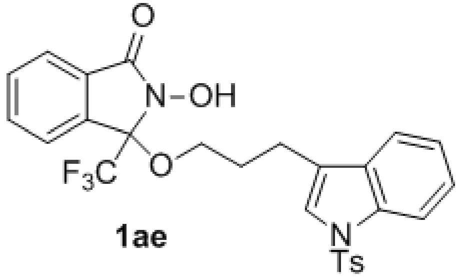

1ae

Ts-indole, OH

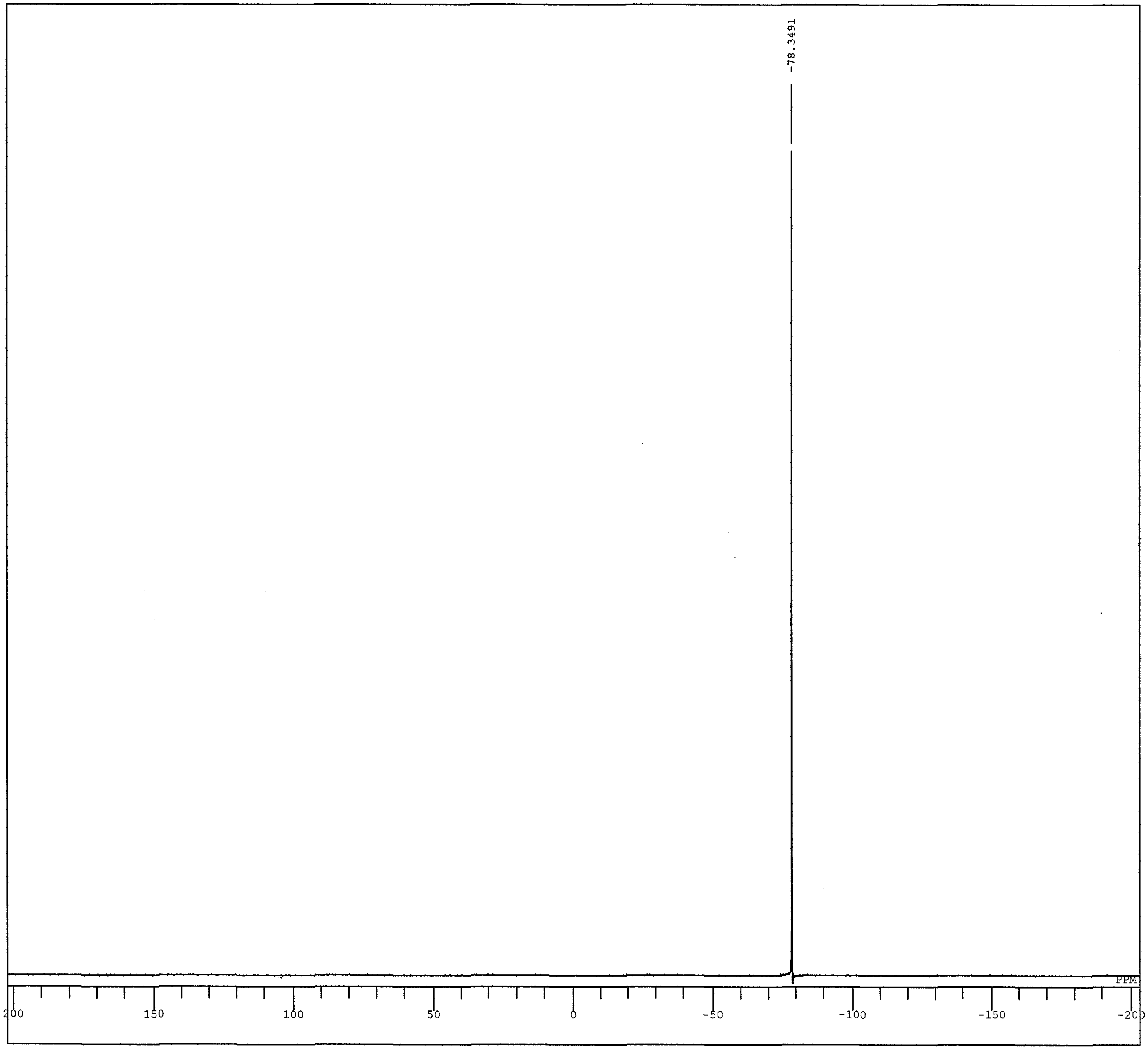

DFILE	ozawa05-123_19F.jdf
COMNT	Ts-indole, OH
DATIM	15-08-2014 19:48:48
OBNUC	19F
EXMOD	proton.jxp
OBFRQ	368.64 MHz
OBSET	7.63 KHz
OBFIN	2.85 Hz
POINT	16384
FREQU	186567.17 Hz
SCANS	4
ACQTM	0.0878 sec
PD	5.0000 sec
PW1	3.90 usec
IRNUC	19F
CTEMP	24.6 c
SLVNT	CDCL3
EXREF	-164.90 ppm
BF	0.12 Hz
RGAIN	46

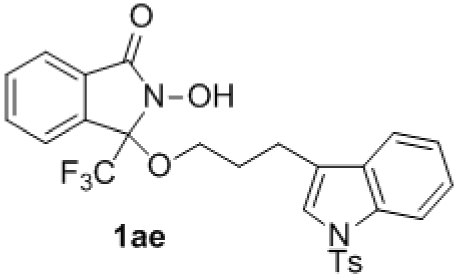

1ae

Ts-indole, [O]

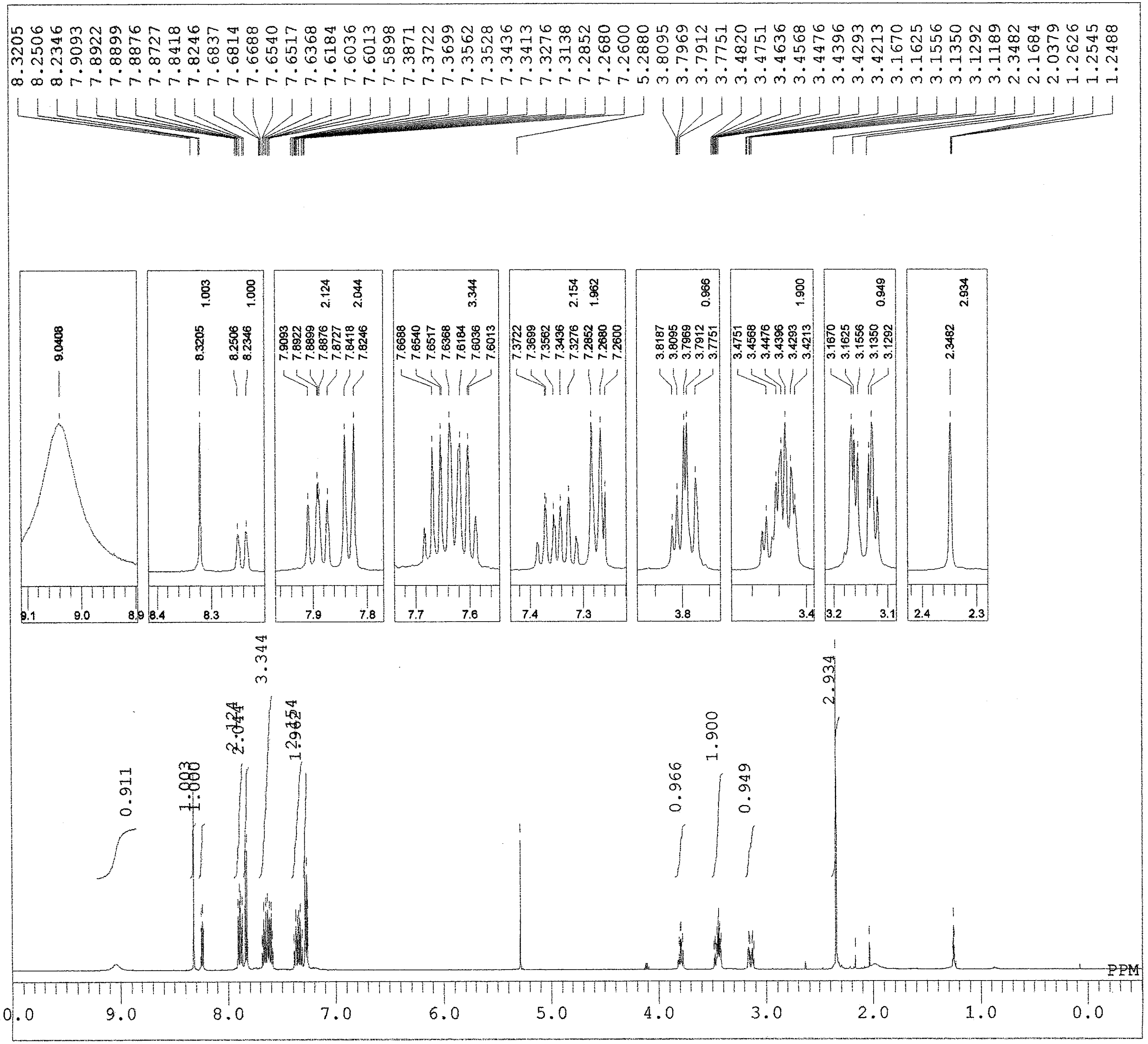

DFILE	ozawa05-132_1H.als
COMNT	Ts-indole, [O]
DATIM	2014-08-14 17:05:43
OBNUC	1H
EXMOD	proton.jxp
OBFRQ	500.16 MHz
OBSET	2.41 KHz
OBFIN	6.01 Hz
POINT	13107
FREQU	7507.51 Hz
SCANS	4
ACQTM	1.7459 sec
PD	5.0000 sec
PW1	5.55 usec
IRNUC	1H
CTEMP	25.5 c
SLVNT	CDCL3
EXREF	7.26 ppm
BF	0.12 Hz
RGAIN	34

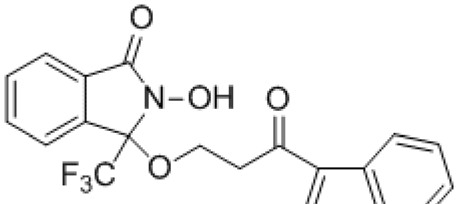

2ae

Ts-indole, [O]

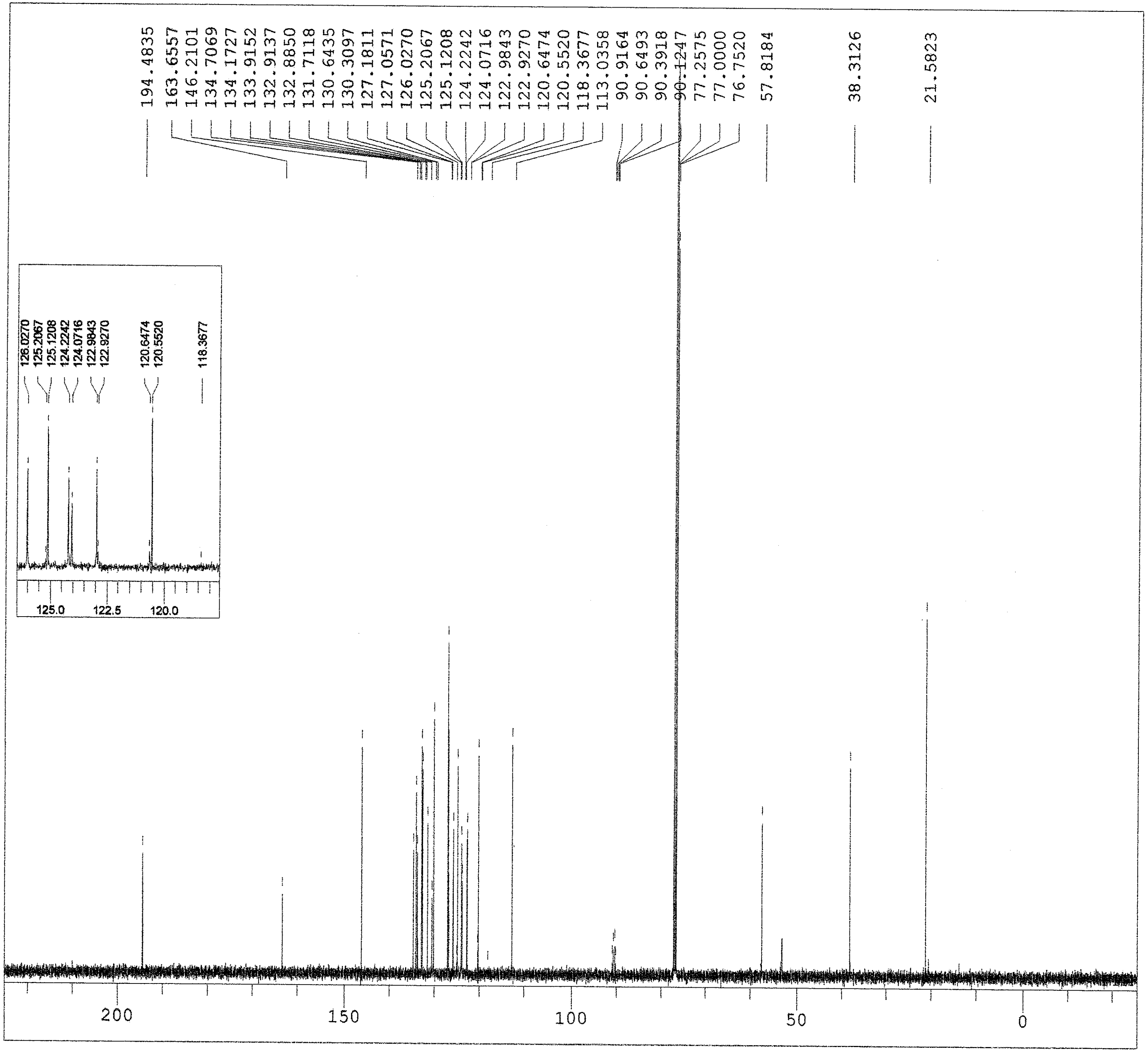

DFILE	ozawa05-132_13C.jdf
COMNT	Ts-indole, [O]
DATIM	2014-08-14 17:06:47
OBNUC	13C
EXMOD	carbon.jxp
OBFRQ	125.77 MHz
OBSET	7.87 KHz
OBFIN	4.21 Hz
POINT	32767
FREQU	39308.18 Hz
SCANS	600
ACQTM	0.8336 sec
PD	3.0000 sec
PW1	3.40 usec
IRNUC	1H
CTEMP	26.3 c
SLVNT	CDCL3
EXREF	77.00 ppm
BF	0.12 Hz
RGAIN	60

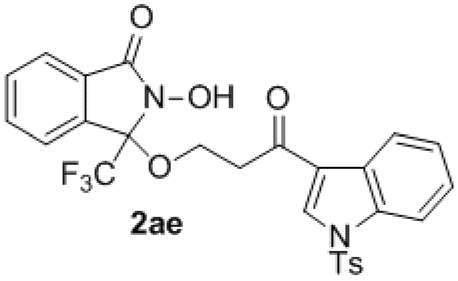

2ae

Ts-indole

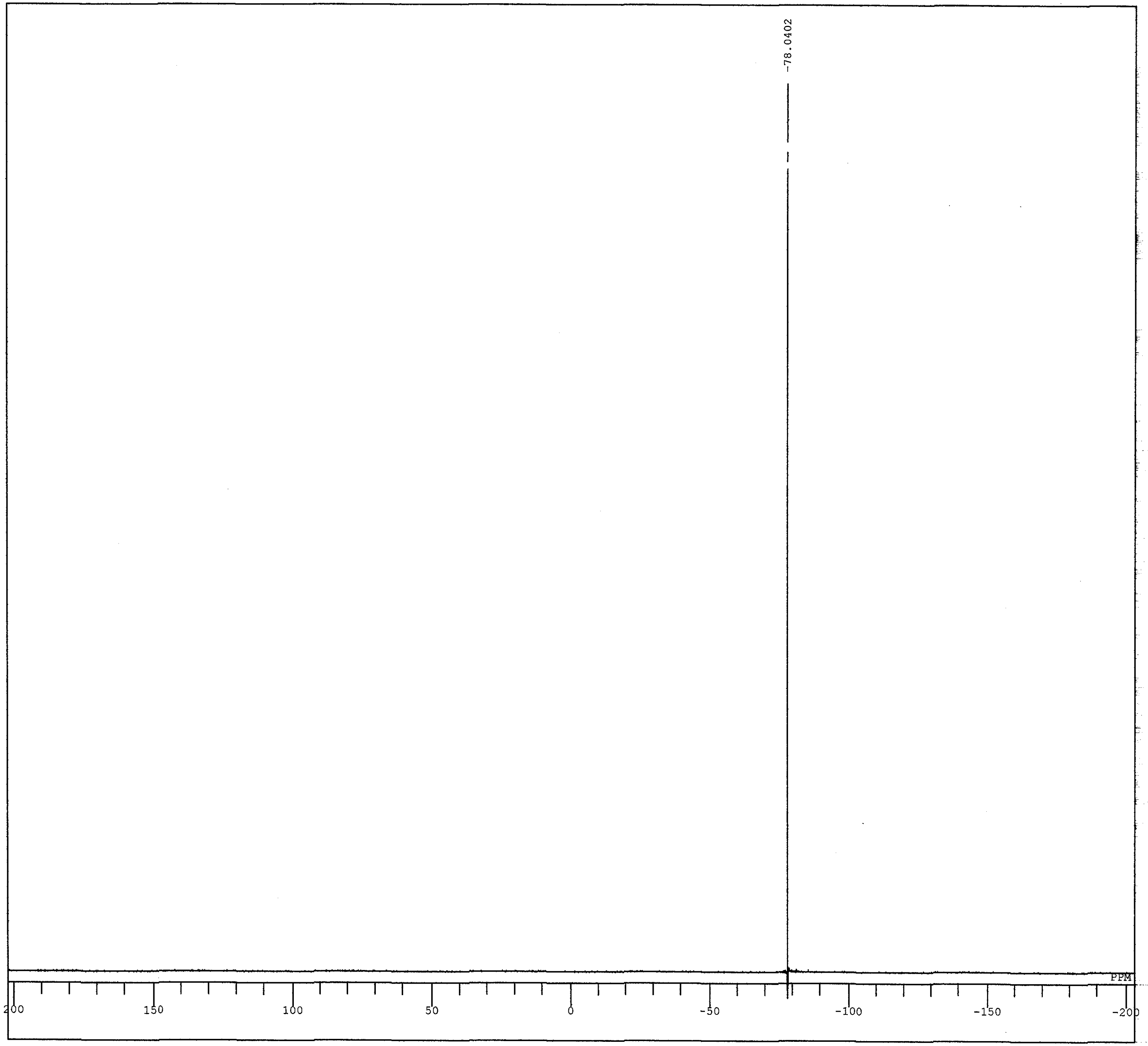

DFILE ozawa05-132_19F.jdf
COMNT Ts-indole
DATIM 14-08-2014 19:23:06
OBNUC 19F
EXMOD proton.jxp
OBFRQ 368.64 MHz
OBSET 7.63 KHz
OBFIN 2.85 Hz
POINT 16384
FREQU 186567.17 Hz
SCANS 4
ACQTM 0.0878 sec
PD 5.0000 sec
PW1 3.90 usec
IRNUC 19F
CTEMP 24.2 c
SLVNT CDCL3
EXREF -164.90 ppm
BF 0.12 Hz
RGAIN 46

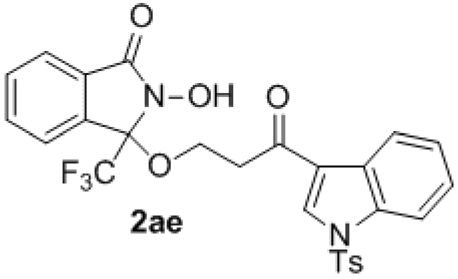

2ae

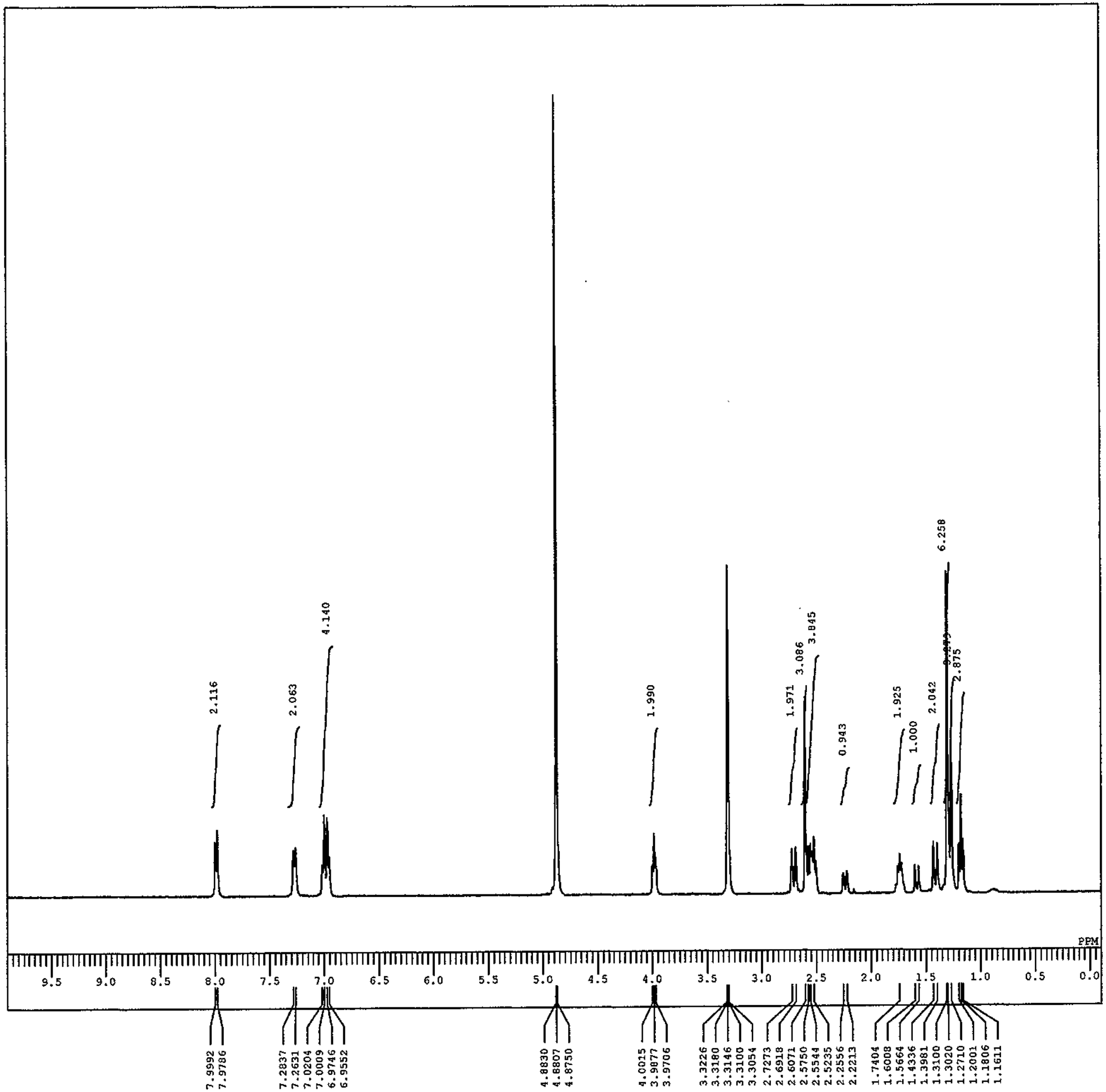

DFILE S7 HNMR-1-1.als
COMNT
DATIM 30-05-2015 11:28:31
OBNUC 1H
EXMOD proton.jxp
OBFRQ 391.78 MHz
OBSET 8.51 KHz
OBFIN 3.34 Hz
POINT 13107
FREQU 5878.90 Hz
SCANS 8
ACQTM 2.2295 sec
PD 5.0000 sec
PW1 4.99 usec
IRNUC 1H
CTEMP 21.6 c
SLVNT CD3OD
EXREF 3.31 ppm
BF 0.12 Hz
RGAIN 44

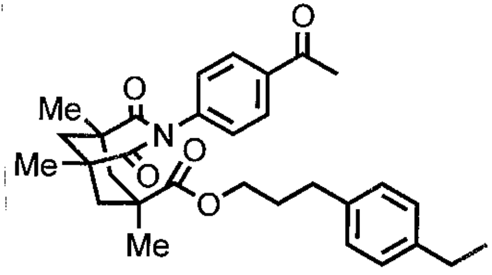

S7

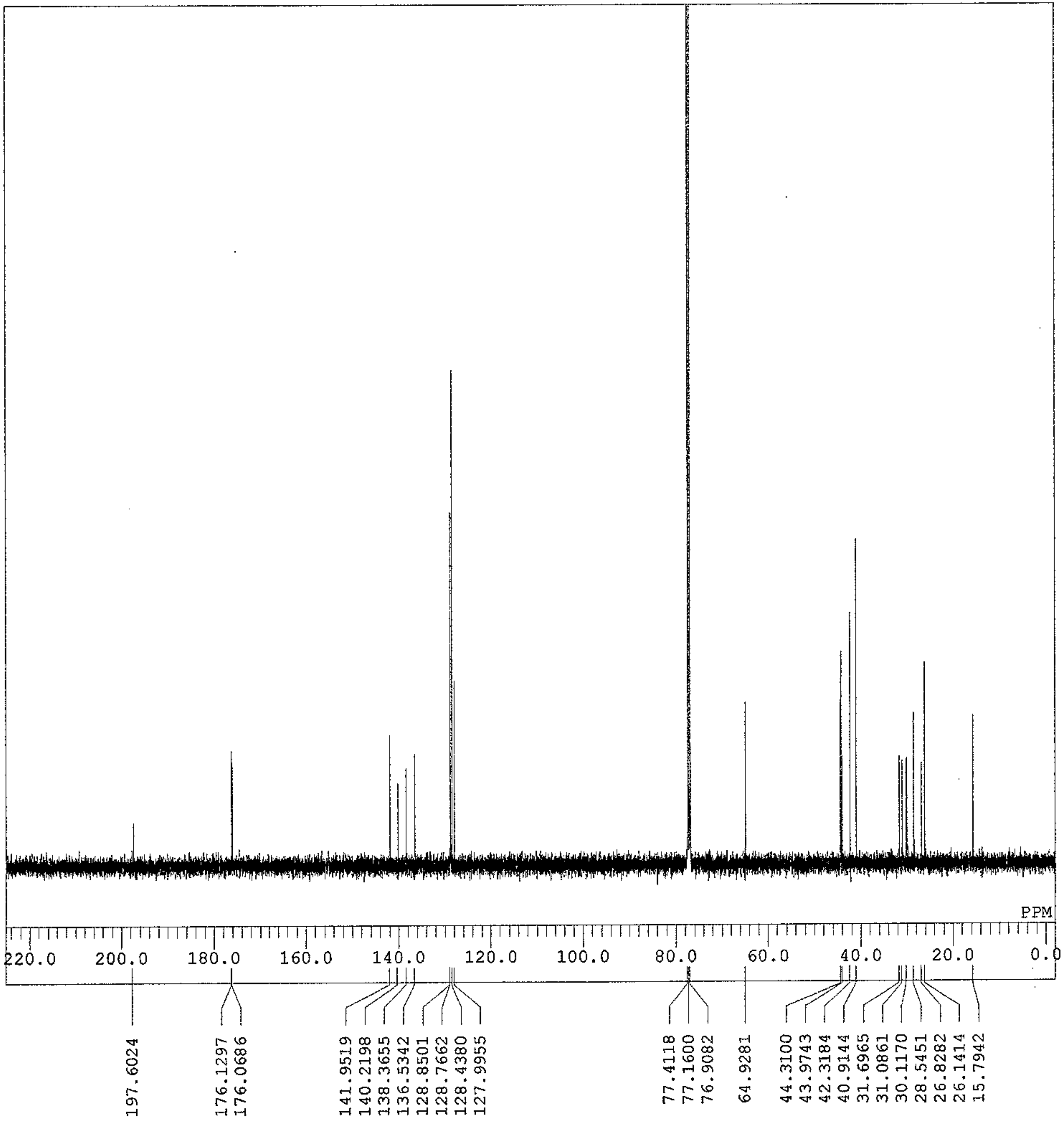

DFILE S7 CNMR.als
COMNT
DATIM 2015-05-06 14:46:47
OBNUC 13C
EXMOD carbon.jxp
OBFRQ 125.77 MHz
OBSET 7.87 KHz
OBFIN 4.21 Hz
POINT 32768
FREQU 31446.54 Hz
SCANS 3017
ACQTM 0.0000 sec
PD 2.0000 sec
PW1 3.40 usec
IRNUC 1H
CTEMP 21.9 c
SLVNT CDCL3
EXREF 77.16 ppm
BF 0.12 Hz
RGAIN 72

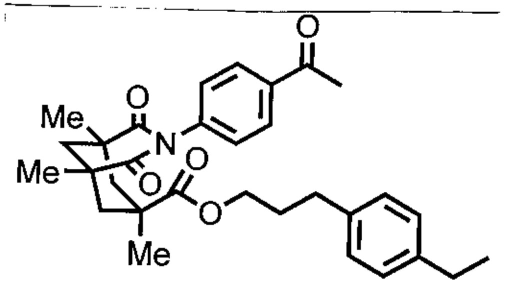

S7

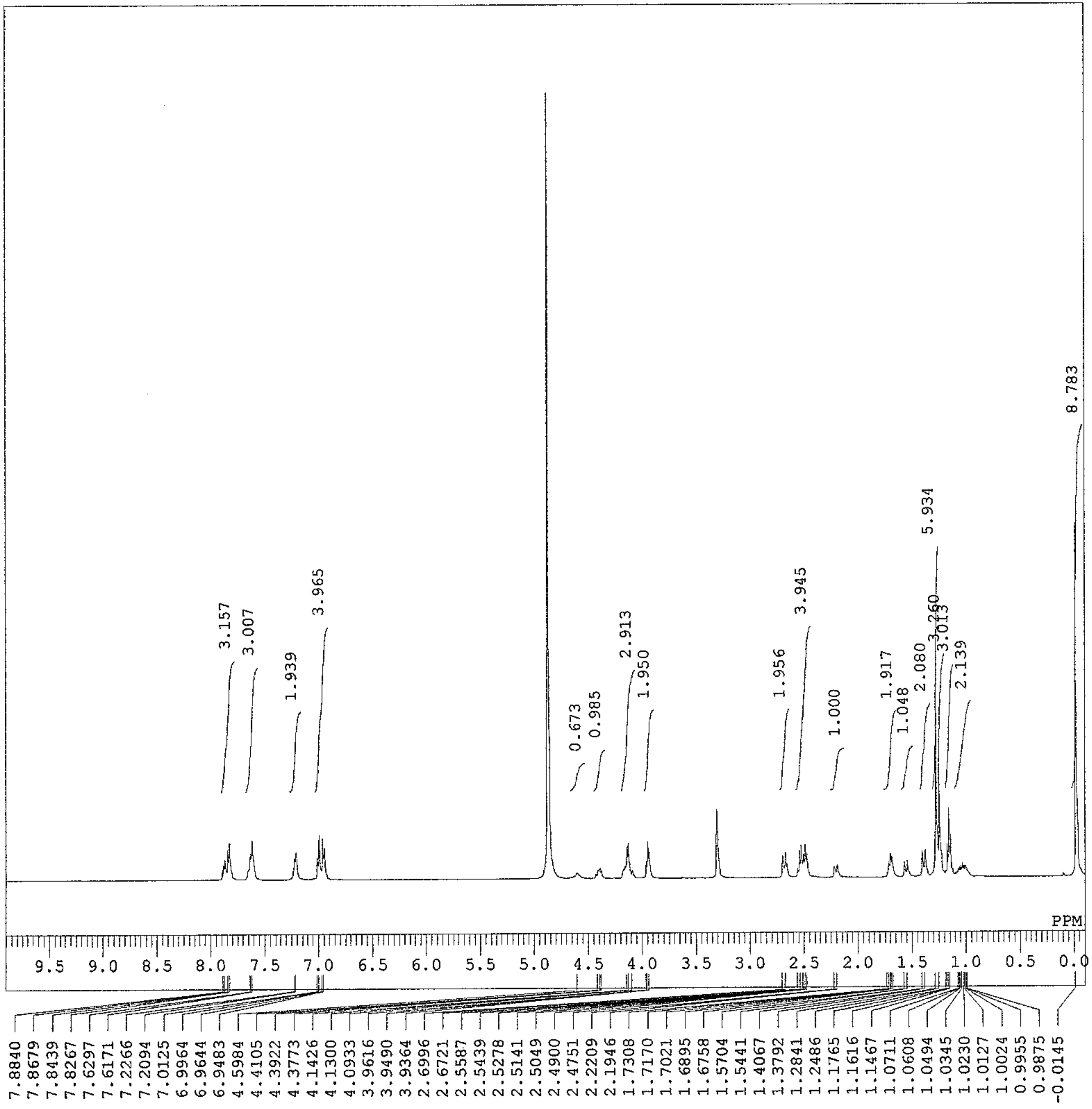

DFILE S8 HNMR.als
COMNT
DATIM 2015-05-06 18:35:39
OBNUC 1H
EXMOD proton.jxp
OBFRQ 500.16 MHz
OBSET 2.41 KHz
OBFIN 6.01 Hz
POINT 13107
FREQU 7507.51 Hz
SCANS 19
ACQTM 0.0000 sec
PD 5.0000 sec
PW1 5.55 usec
IRNUC 1H
CTEMP 21.4 c
SLVNT CD3OD
EXREF 3.31 ppm
BF 0.12 Hz
RGAIN 30

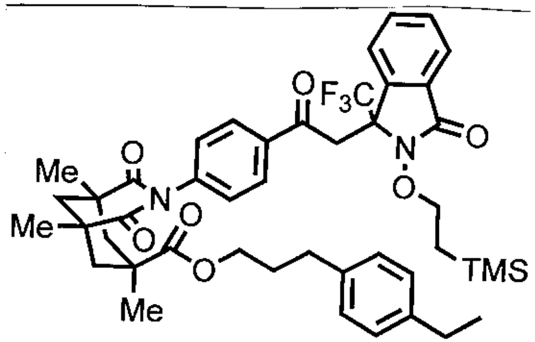

S8

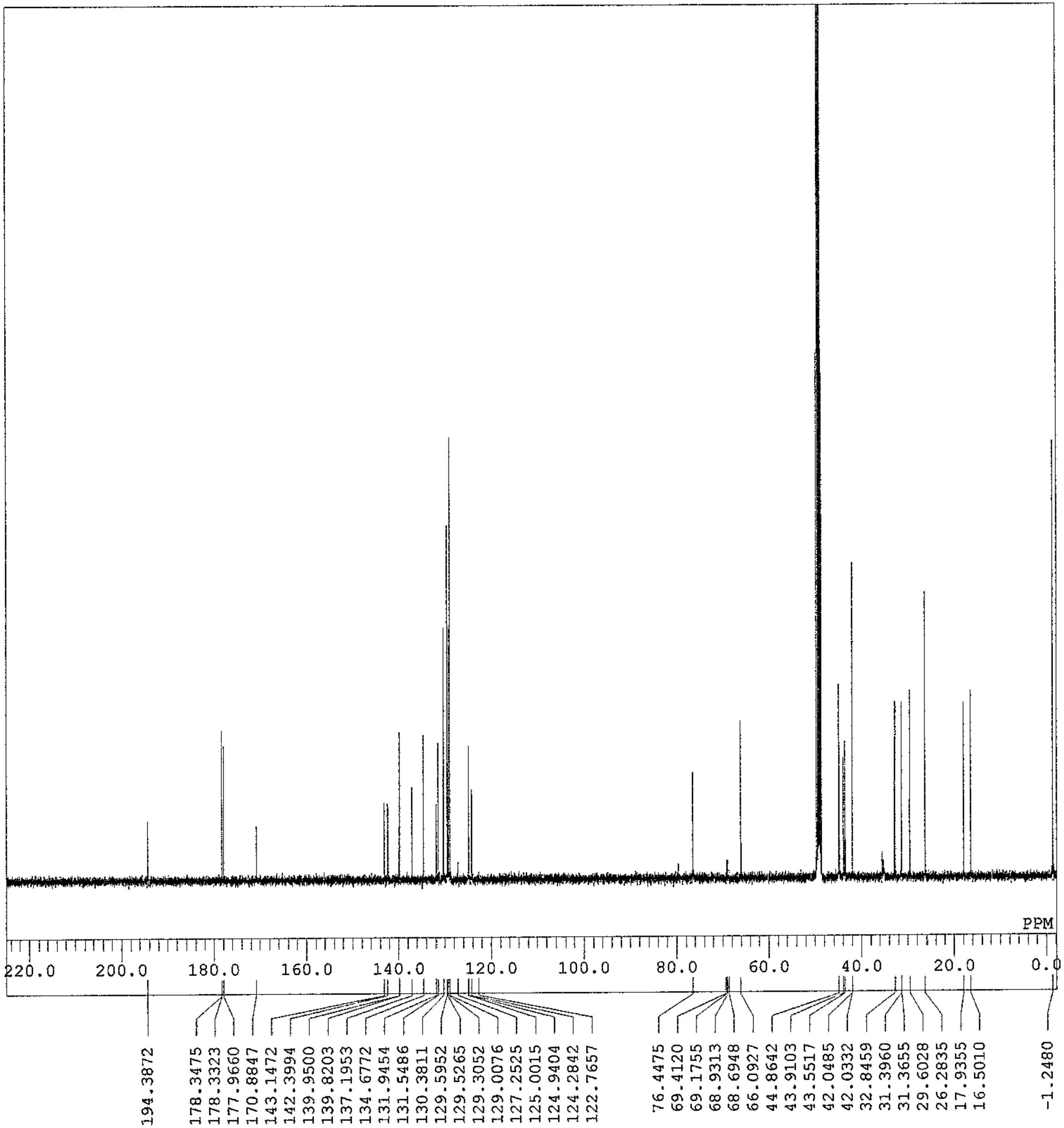

DFILE	S8 CNMR.als
COMNT	
DATIM	2015-05-06 18:43:52
OBNUC	13C
EXMOD	carbon.jxp
OBFRQ	125.77 MHz
OBSET	7.87 KHz
OBFIN	4.21 Hz
POINT	32768
FREQU	31446.54 Hz
SCANS	16000
ACQTM	1.0420 sec
PD	2.0000 sec
PW1	3.40 usec
IRNUC	1H
CTEMP	21.9 c
SLVNT	CD3OD
EXREF	49.00 ppm
BF	0.12 Hz
RGAIN	74

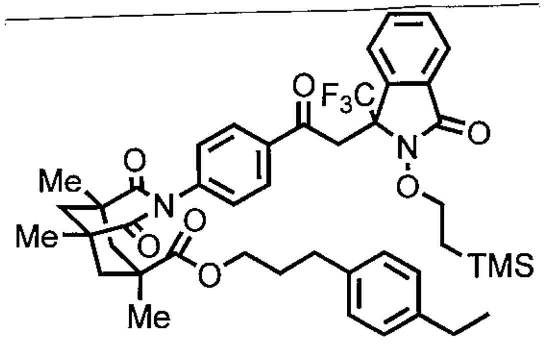

S8

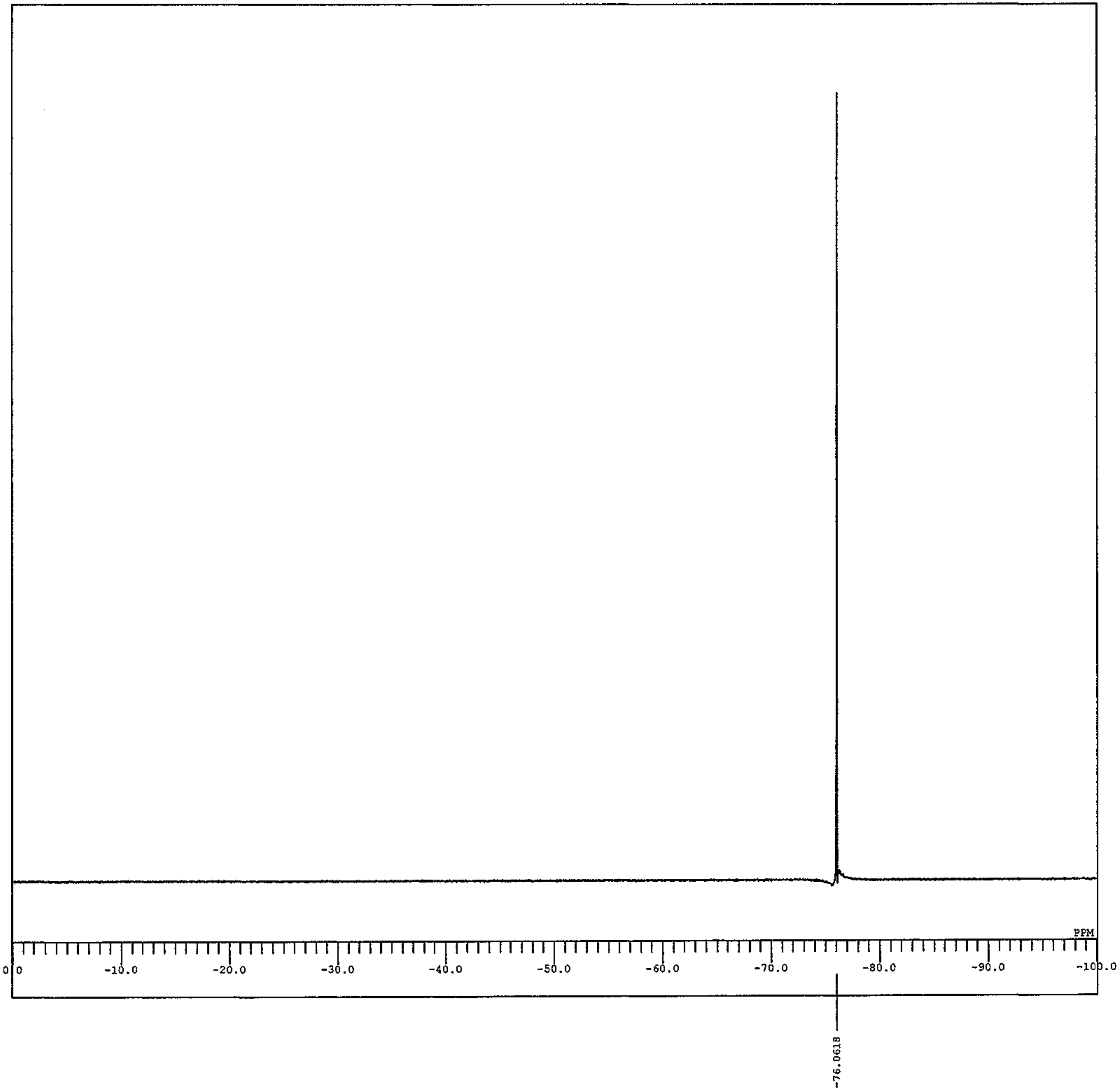


```
DFILE  S8 FNMR-1-1.als
COMNT
DATIM  30-05-2015 11:11:30
OBNUC  19F
EXMOD  proton.jxp
OBFRQ        368.64 MHz
OBSET          7.63 KHz
OBFIN          2.85 Hz
POINT         13107
FREQU     149253.73 Hz
SCANS             8
ACQTM        0.0878 sec
PD           5.0000 sec
PW1            3.90 usec
IRNUC  19F
CTEMP          21.7 c
SLVNT  CDCL3
EXREF       -164.90 ppm
BF             0.12 Hz
RGAIN            50
```

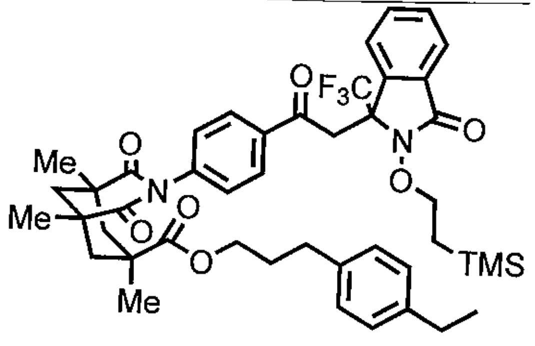

S8

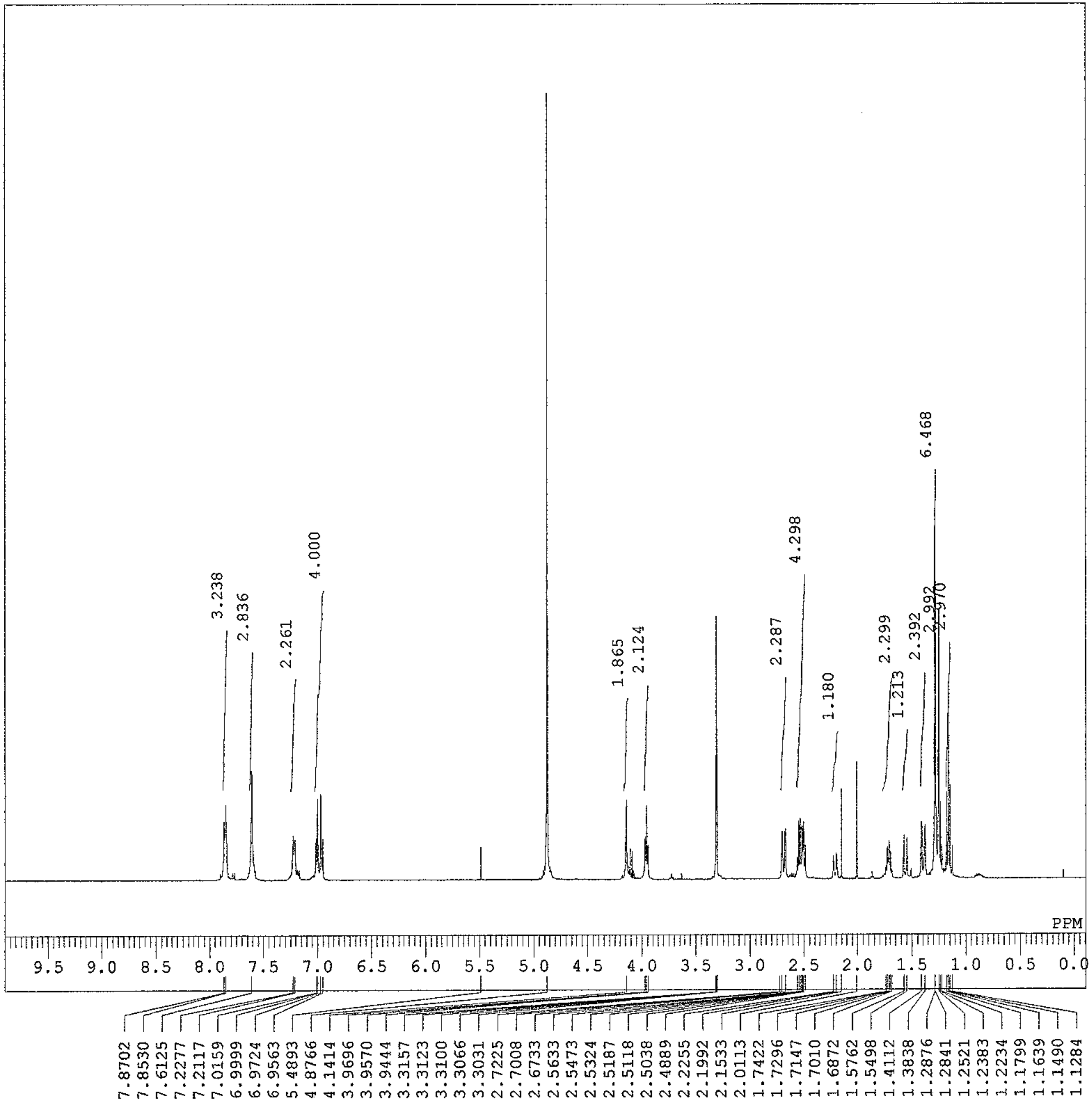

DFILE 3 HNMR.als
COMNT
DATIM 2015-05-28 14:11:51
OBNUC 1H
EXMOD proton.jxp
OBFRQ 500.16 MHz
OBSET 2.41 KHz
OBFIN 6.01 Hz
POINT 13107
FREQU 7507.51 Hz
SCANS 8
ACQTM 1.7459 sec
PD 5.0000 sec
PW1 5.55 usec
IRNUC 1H
CTEMP 21.0 c
SLVNT CD3OD
EXREF 3.31 ppm
BF 0.12 Hz
RGAIN 30

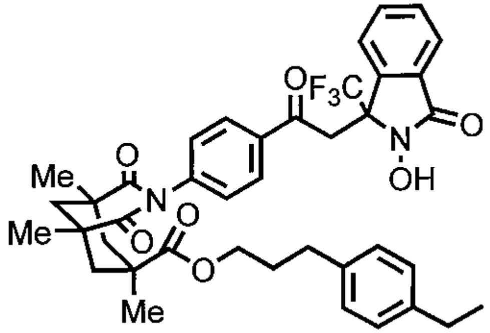

3

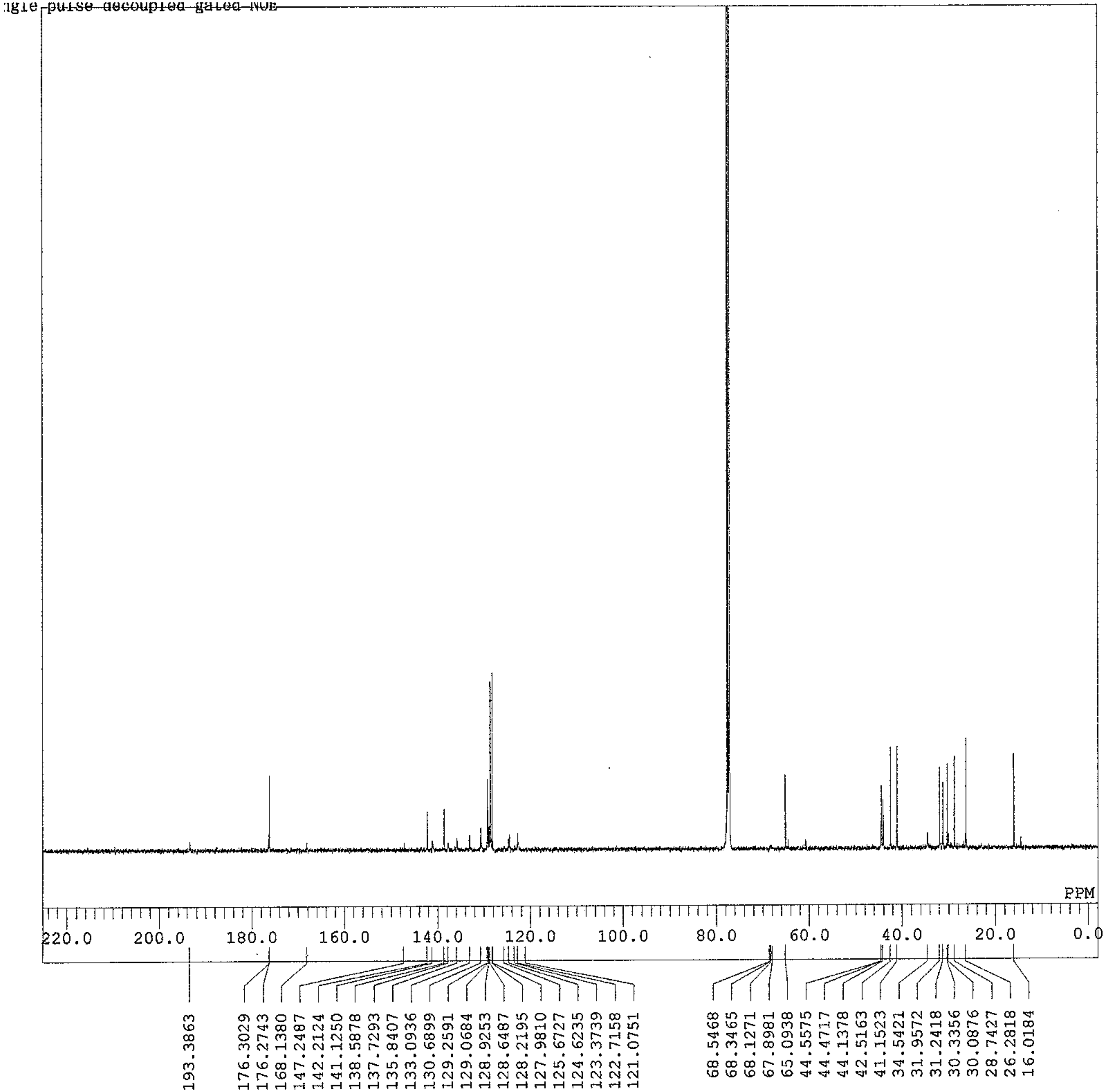

DFILE 3 CNMR.als
COMNT single pulse decoupled gated
DATIM 2015-05-29 23:00:44
OBNUC 13C
EXMOD carbon.jxp
OBFRQ 125.77 MHz
OBSET 7.87 KHz
OBFIN 4.21 Hz
POINT 26214
FREQU 31446.54 Hz
SCANS 13769
ACQTM 0.0000 sec
PD 2.0000 sec
PW1 3.40 usec
IRNUC 1H
CTEMP 21.3 c
SLVNT CDCL3
EXREF 77.16 ppm
BF 1.20 Hz
RGAIN 50

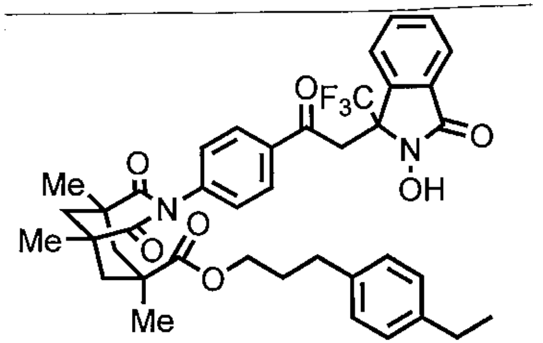

3

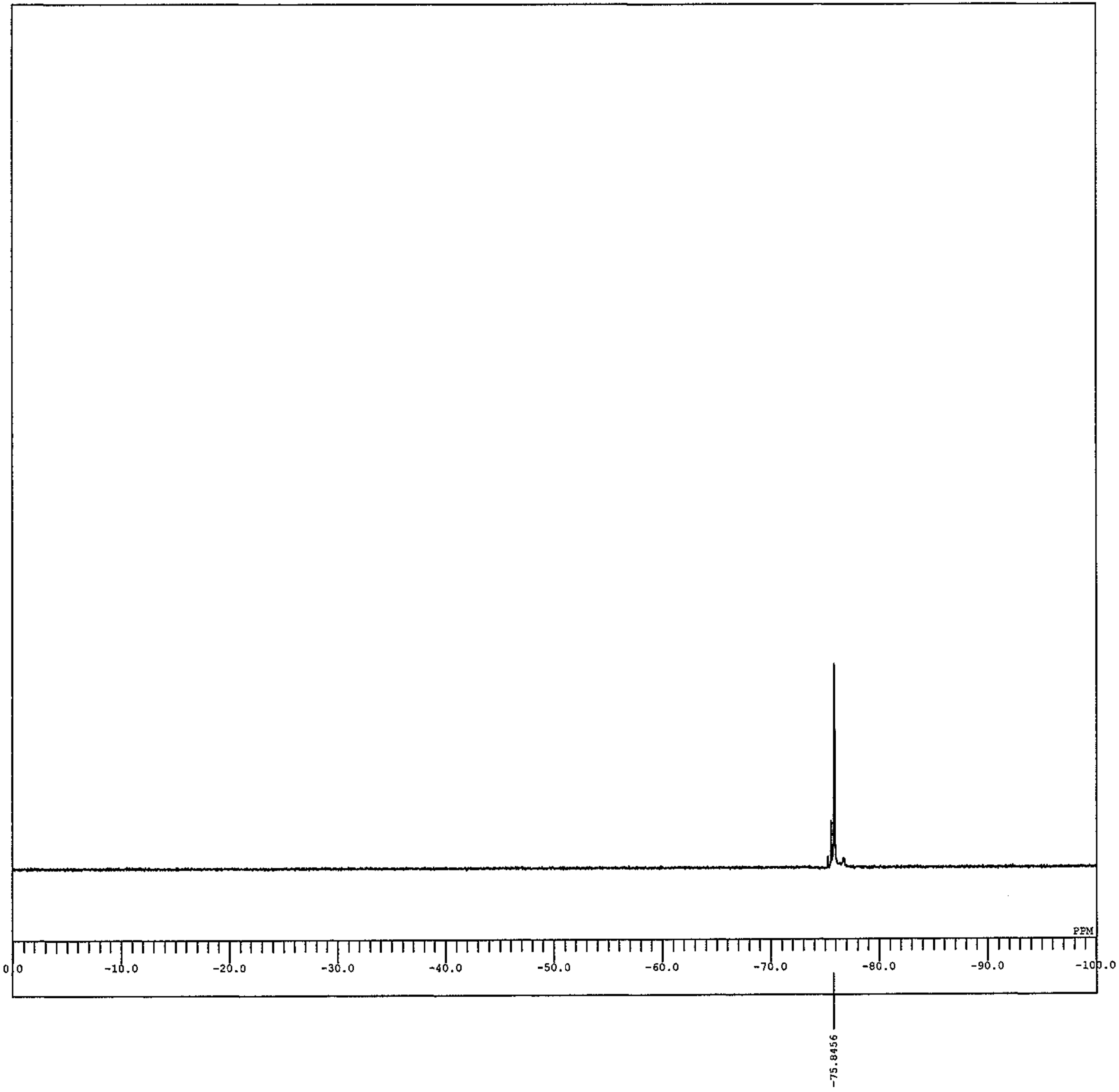


```
DFILE  3 FNMR-1-1.als
COMNT
DATIM  30-05-2015 11:15:10
OBNUC  19F
EXMOD  proton.jxp
OBFRQ        368.64 MHz
OBSET          7.63 KHz
OBFIN          2.85 Hz
POINT         13107
FREQU     149253.73 Hz
SCANS             8
ACQTM        0.0878 sec
PD           5.0000 sec
PW1            3.90 usec
IRNUC  19F
CTEMP          21.7 c
SLVNT  CDCL3
EXREF       -164.90 ppm
BF             0.12 Hz
RGAIN            50
```

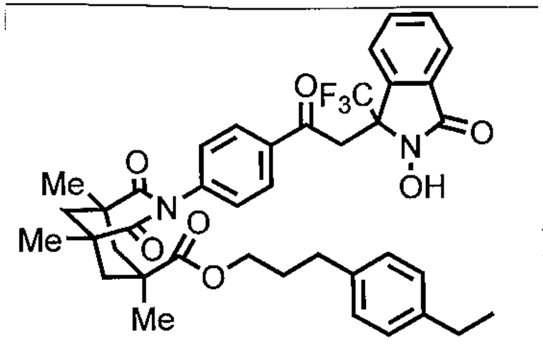

3

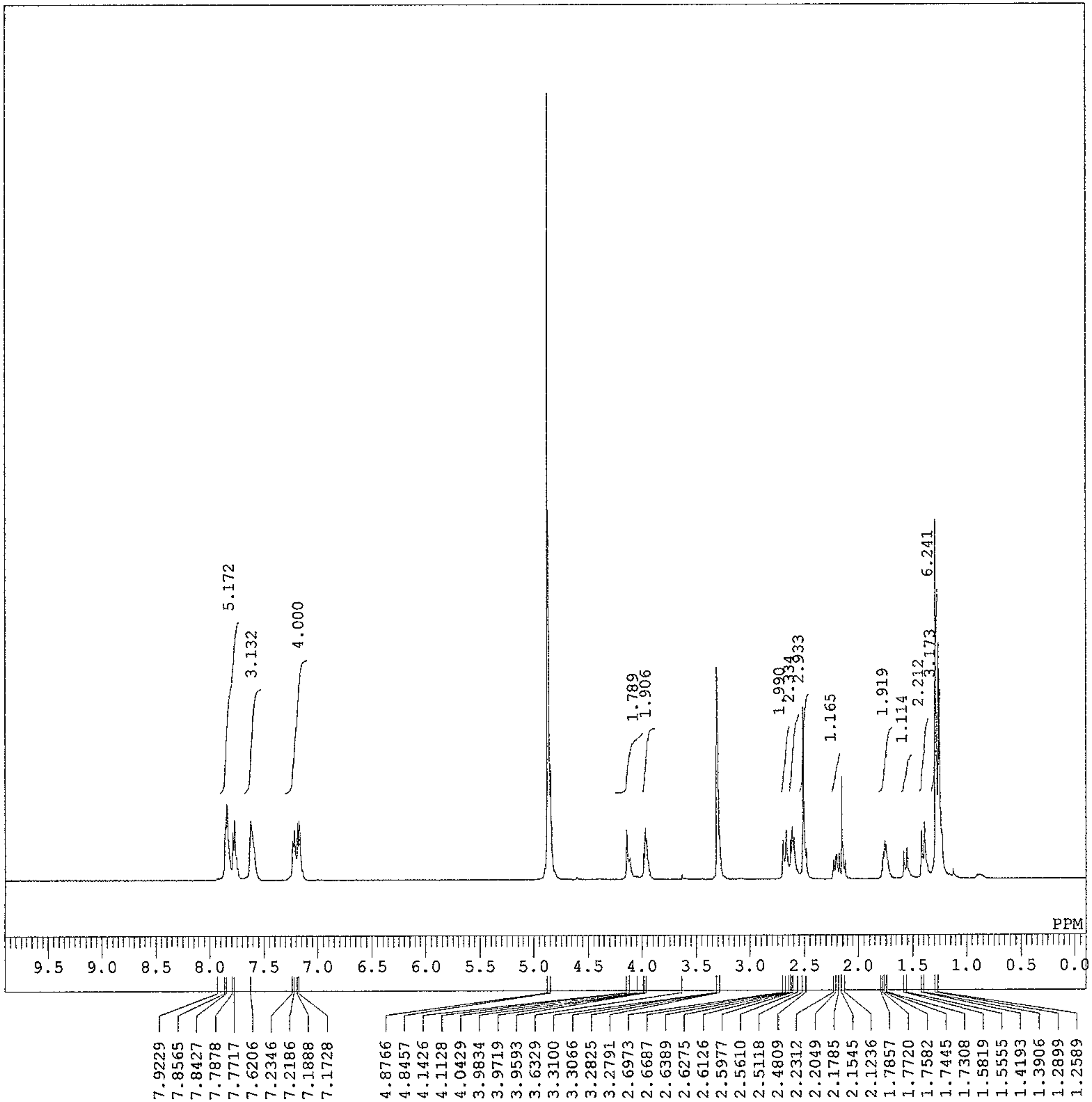

DFILE	4 HNMR.als
COMNT	
DATIM	2015-05-27 22:54:43
OBNUC	1H
EXMOD	proton.jxp
OBFRQ	500.16 MHz
OBSET	2.41 KHz
OBFIN	6.01 Hz
POINT	13107
FREQU	7507.51 Hz
SCANS	8
ACQTM	1.7459 sec
PD	5.0000 sec
PW1	5.55 usec
IRNUC	1H
CTEMP	21.1 c
SLVNT	CD3OD
EXREF	3.31 ppm
BF	0.12 Hz
RGAIN	30

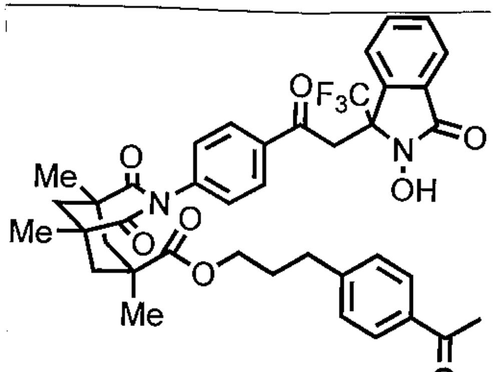

4

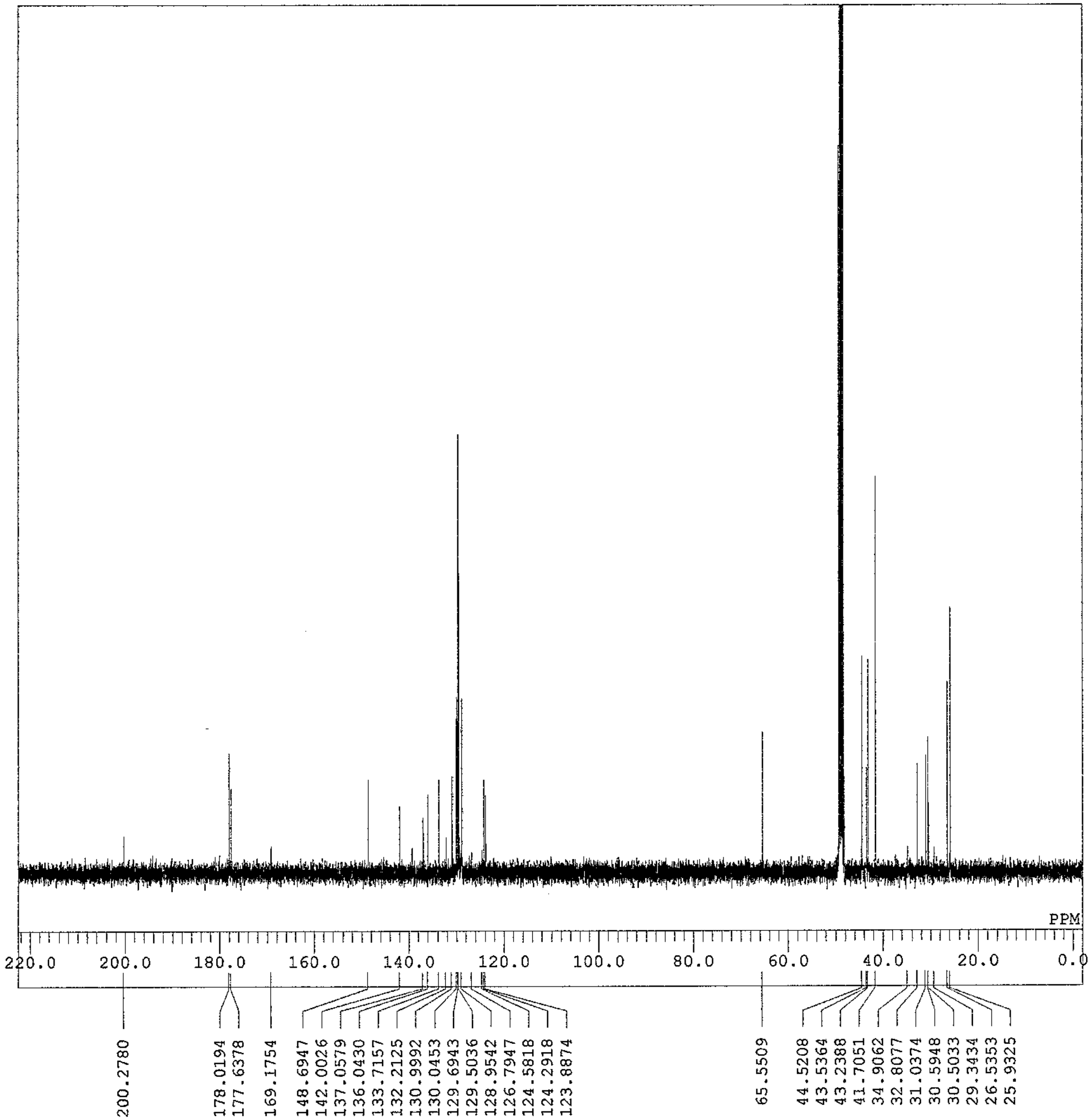

DFILE 4CNMR.als
COMNT
DATIM 2015-05-27 22:57:05
OBNUC 13C
EXMOD carbon.jxp
OBFRQ 125.77 MHz
OBSET 7.87 KHz
OBFIN 4.21 Hz
POINT 32768
FREQU 31446.54 Hz
SCANS 12272
ACQTM 1.0420 sec
PD 2.0000 sec
PW1 3.40 usec
IRNUC 1H
CTEMP 21.6 c
SLVNT CD3OD
EXREF 49.00 ppm
BF 0.12 Hz
RGAIN 74

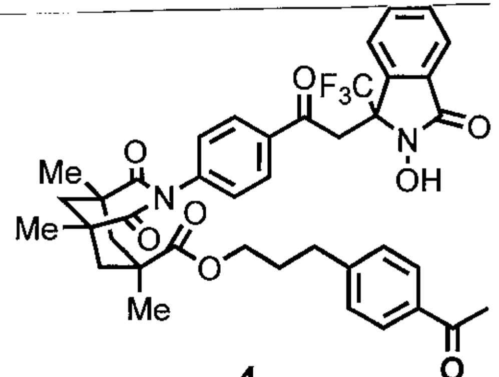

4

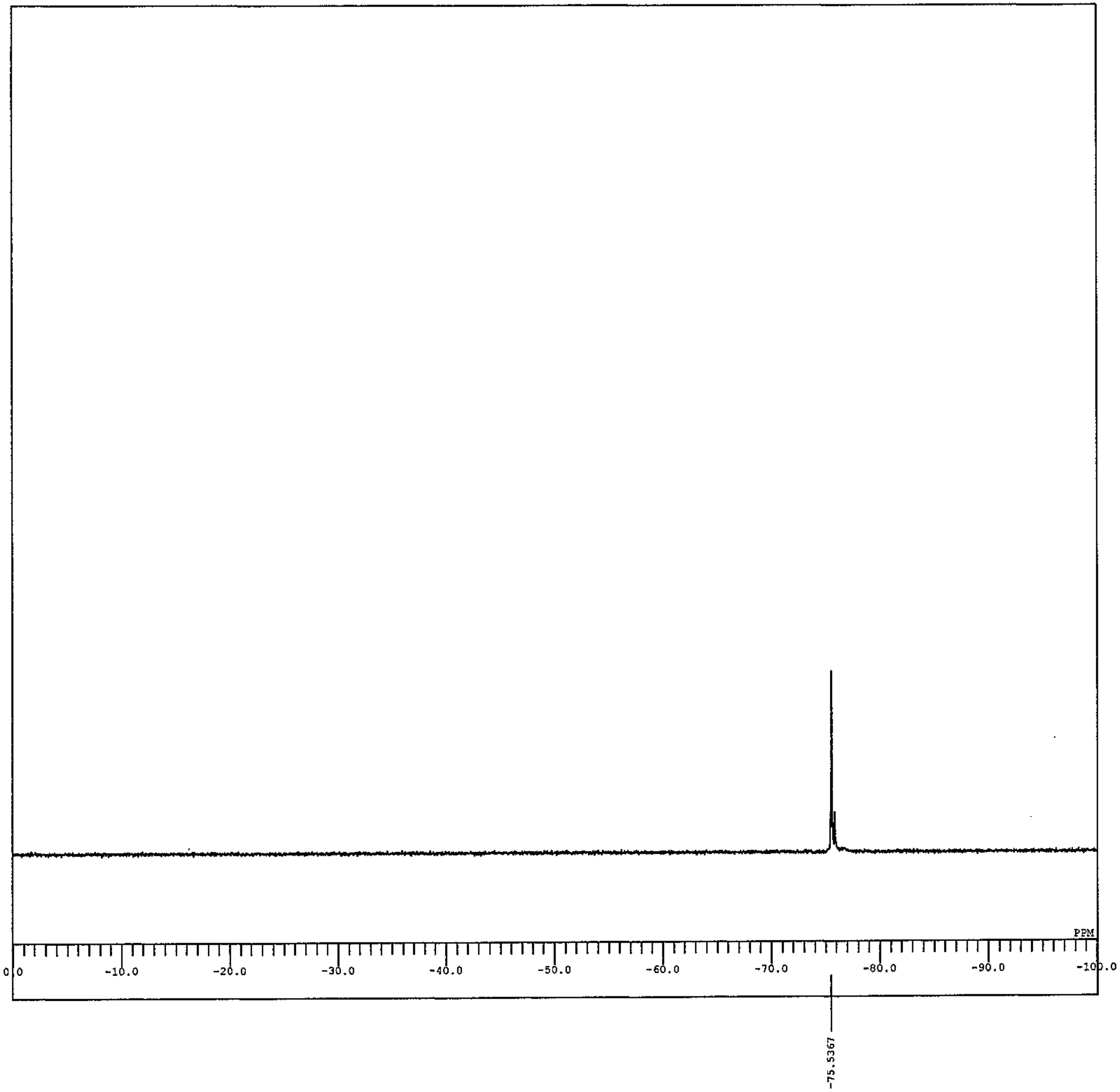


```
DFILE  4 FNMR-1-1.als
COMNT
DATIM  31-05-2015 11:23:39
OBNUC  19F
EXMOD  proton.jxp
OBFRQ        368.64 MHz
OBSET          7.63 KHz
OBFIN          2.85 Hz
POINT         13107
FREQU     149253.73 Hz
SCANS             8
ACQTM        0.0878 sec
PD           5.0000 sec
PW1            3.90 usec
IRNUC  19F
CTEMP          21.6 c
SLVNT  CDCL3
EXREF       -164.90 ppm
BF             0.12 Hz
RGAIN            50
```

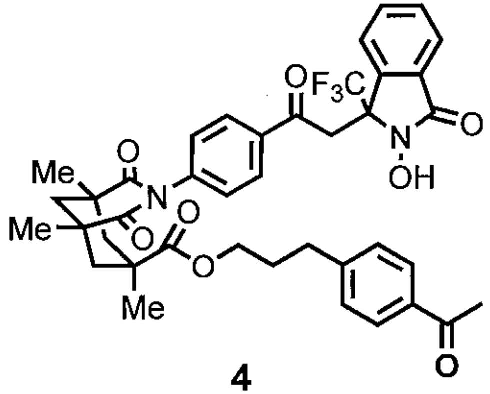

4

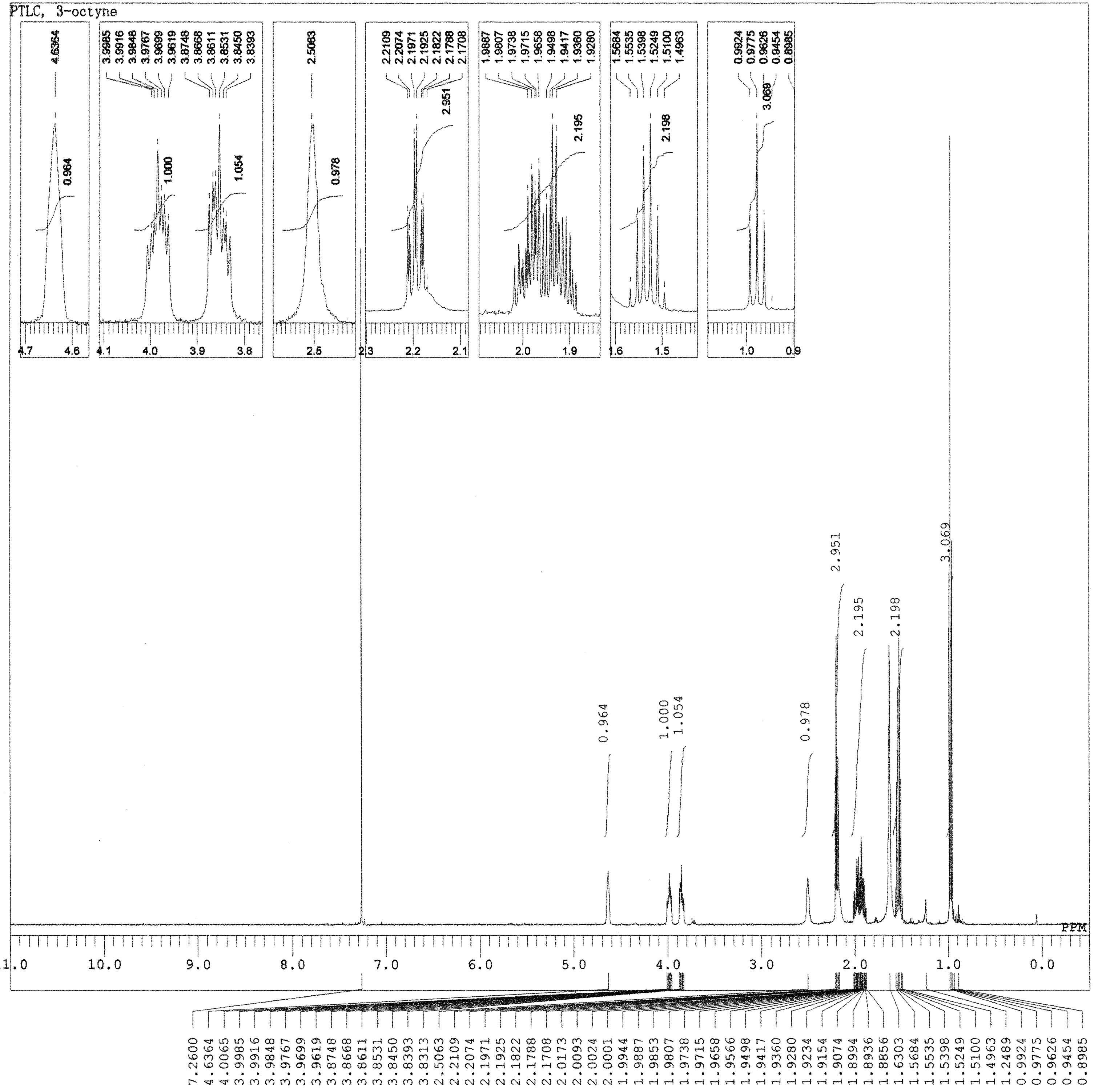

DFILE	ozawa08-103.jdf
COMNT	PTLC, 3-octyne
DATIM	2015-09-02 12:52:20
OBNUC	1H
EXMOD	proton.jxp
OBFRQ	500.16 MHz
OBSET	2.41 KHz
OBFIN	6.01 Hz
POINT	16384
FREQU	9384.38 Hz
SCANS	4
ACQTM	1.7459 sec
PD	5.0000 sec
PW1	5.55 usec
IRNUC	1H
CTEMP	21.2 c
SLVNT	CDCL3
EXREF	7.26 ppm
BF	0.10 Hz
RGAIN	34

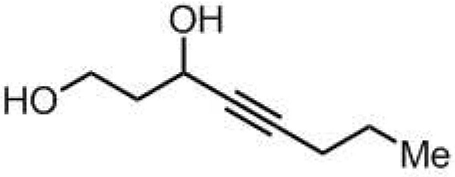

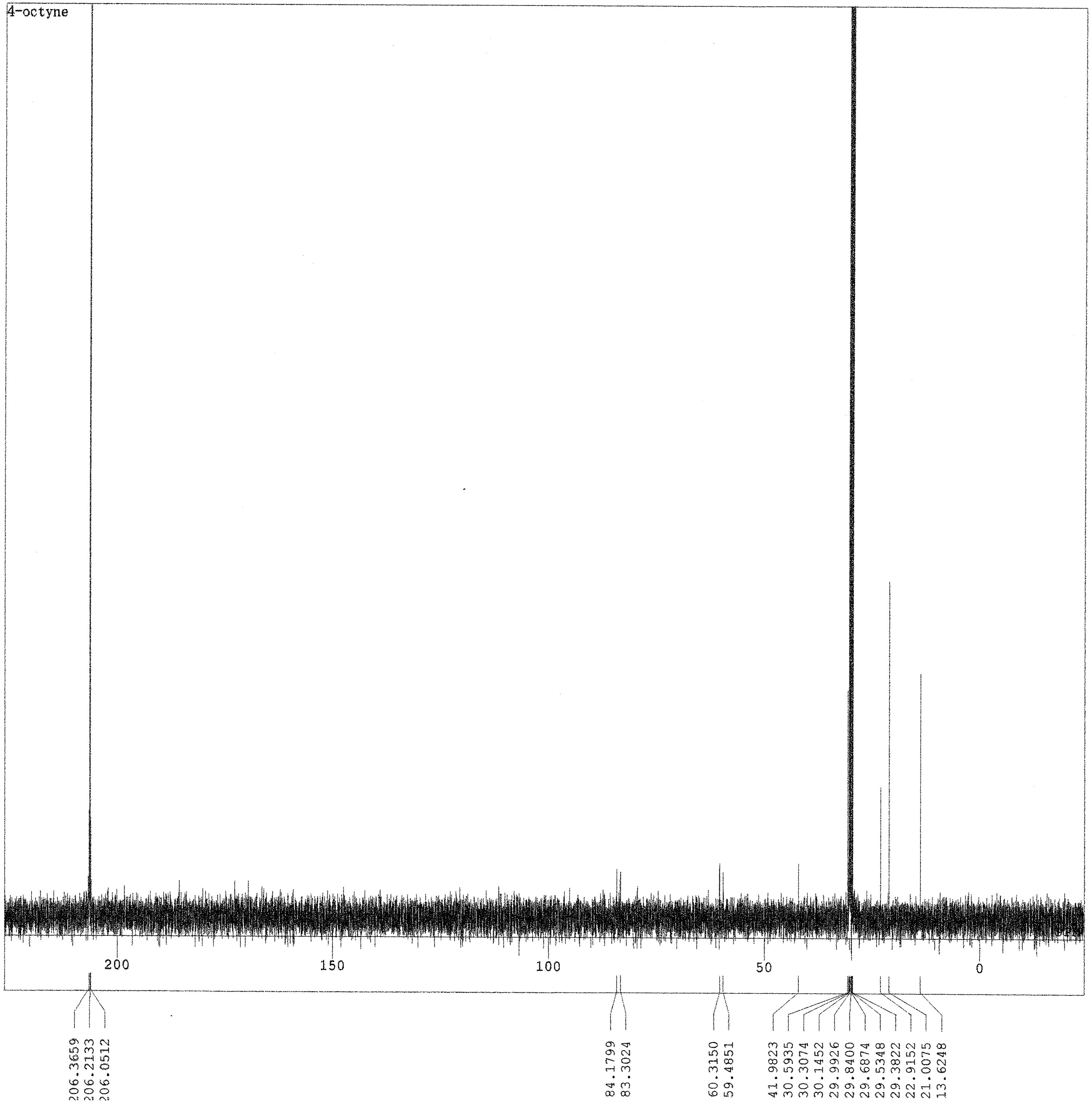

DFILE ozawa08-103_13C_acetone.jdf
COMNT 4-octyne
DATIM 2015-09-16 17:55:18
OBNUC 13C
EXMOD carbon.jxp
OBFRQ 125.77 MHz
OBSET 7.87 KHz
OBFIN 4.21 Hz
POINT 32767
FREQU 39308.18 Hz
SCANS 600
ACQTM 0.8336 sec
PD 2.0000 sec
PW1 3.40 usec
IRNUC 1H
CTEMP 22.1 c
SLVNT ACETN
EXREF 29.84 ppm
BF 0.12 Hz
RGAIN 60

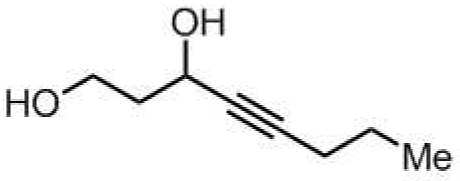

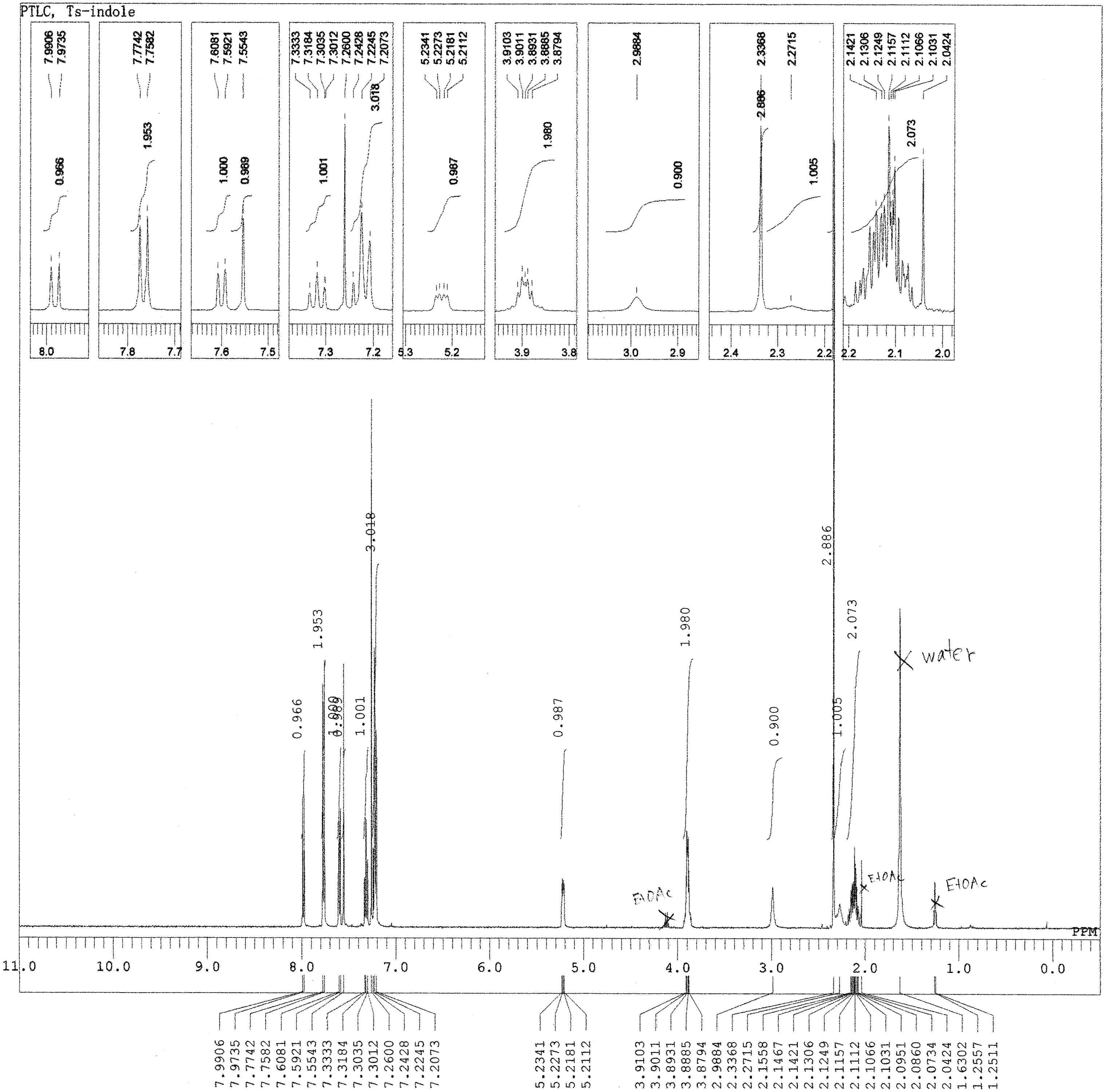

DFILE ozawa08-102.als
COMNT PTLC, Ts-indole
DATIM 2015-09-02 12:57:14
OBNUC 1H
EXMOD proton.jxp
OBFRQ 500.16 MHz
OBSET 2.41 KHz
OBFIN 6.01 Hz
POINT 13107
FREQU 7507.51 Hz
SCANS 4
ACQTM 1.7459 sec
PD 5.0000 sec
PW1 5.55 usec
IRNUC 1H
CTEMP 21.2 c
SLVNT CDCL3
EXREF 7.26 ppm
BF 0.10 Hz
RGAIN 34

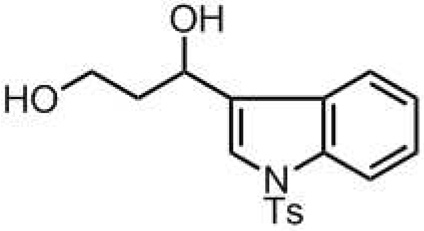

PTLC, Ts-indole

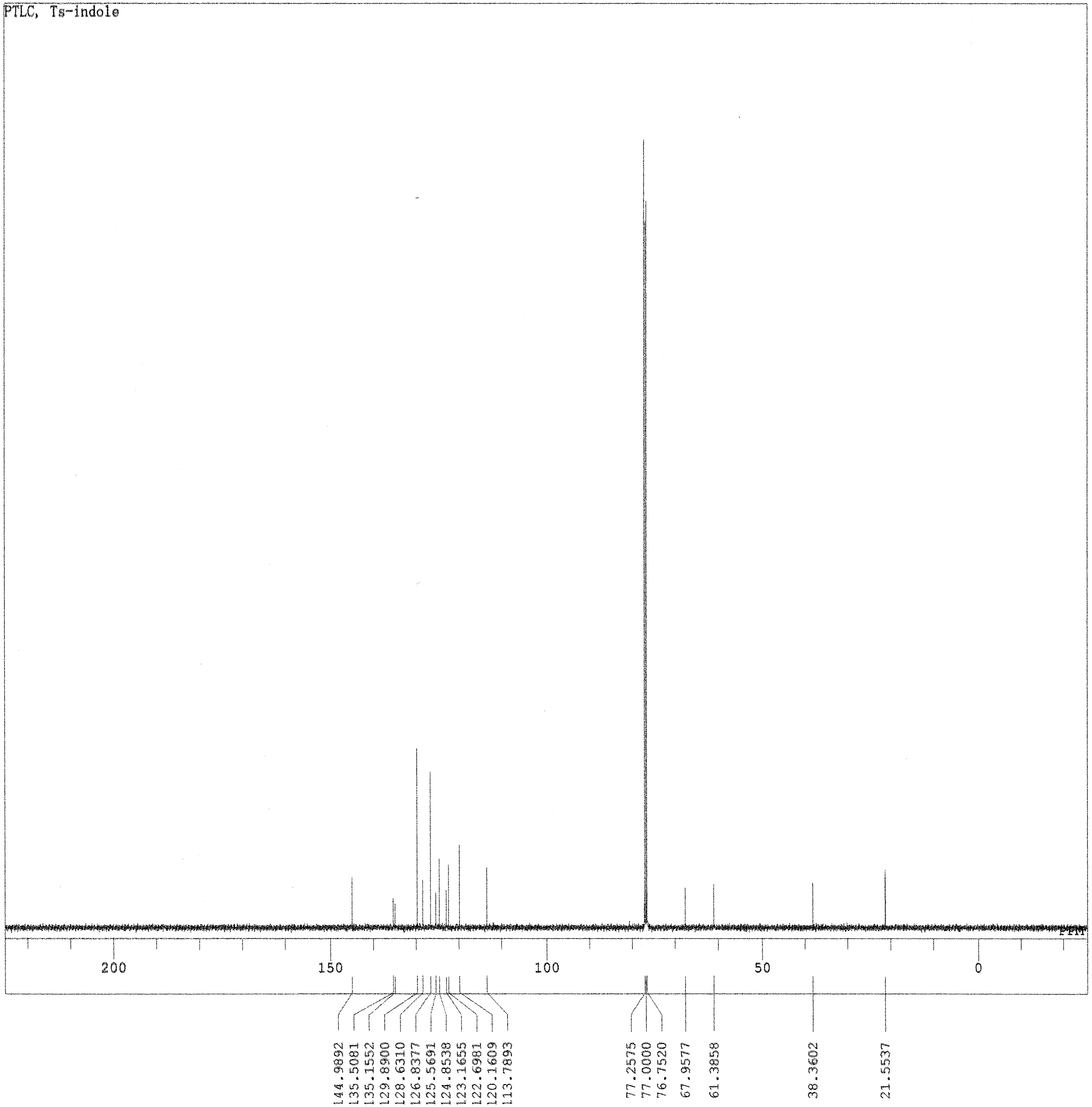

DFILE	ozawa08-102_13C.jdf
COMNT	PTLC, Ts-indole
DATIM	2015-09-17 06:41:60
OBNUC	13C
EXMOD	carbon.jxp
OBFRQ	125.77 MHz
OBSET	7.87 KHz
OBFIN	4.21 Hz
POINT	32767
FREQU	39308.18 Hz
SCANS	2440
ACQTM	0.8336 sec
PD	2.0000 sec
PW1	3.40 usec
IRNUC	1H
CTEMP	21.8 c
SLVNT	CDCL3
EXREF	77.00 ppm
BF	0.12 Hz
RGAIN	60

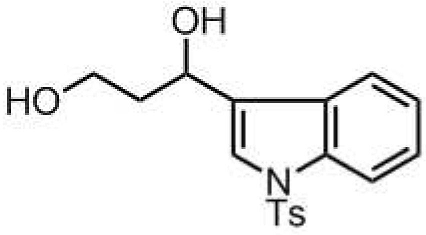

Supplement: Supplementary file 1 [file SC-007-C5SC04476F-s001.pdf]
